# Supplementary material for: Rapid whole brain imaging of neural activity in freely behaving larval zebrafish (Danio rerio)
Source: eLife. 2017 Sep 20;6:e28158. doi: 10.7554/eLife.28158 (PMC5644961; doi:10.7554/eLife.28158)
Supplement: Supplement file 1 [file elife-28158-fig4.docx]

**Supplementary File 1**

**Acquisition parameters for fluorescence imaging**

|  | Zebrafish lines | Age  (dpf) | Volume rate  (Hz) | Flash laser exposure time  (ms) | Average  laser illumination intensity  (mW/mm^2^) |
| --- | --- | --- | --- | --- | --- |
| Figure 1 | huc:h2b-gcamp6f | 5 | 77 | 13 | 2.5 |
| Figure 3 | huc:gcamp6s | 11 | 50 | 1 | 2.8 |
| Video 1 | huc:h2b-gcamp6f | 5 | 77 | 13 | 2.5 |
| Video 2 | huc:h2b-gcamp6f | 5 | 0.6 | 300 | 0.5 |
| Video 3 | huc:gcamp6s | 5 | 0.6 | 100 | 0.37 |
| Video 4 | huc:gcamp6s | 6 | 50 | 20 | 0.37 |
| Video 7 | huc:gcamp6s | 7 | 77 | 0.3 | 1.3 |
| Video 8 | huc:gcamp6s | 11 | 50 | 1 | 2.8 |
| Video 9 | huc:h2b-gcamp6f | 7 | 77 | 0.2 | 0.9 |
