# Supplementary material for: Rapid whole brain imaging of neural activity in freely behaving larval zebrafish (Danio rerio)
Source: eLife. 2017 Sep 20;6:e28158. doi: 10.7554/eLife.28158 (PMC5644961; doi:10.7554/eLife.28158)
Supplement: Source code 3. [file elife-28158-code3.zip › RT analysis code/OpenCV1.1pre.zip!/OpenCV/apps/HaarTraining/doc/haartraining.htm]

Object Detection Using Haar-like Features with Cascade of Boosted
Classifiers


# Rapid Object Detection With A Cascade of Boosted Classifiers Based on Haar-like Features

## Introduction

This document describes how to train and
use a cascade of boosted classifiers for rapid object detection. A large set of
over-complete haar-like features provide the basis for the simple individual
classifiers. Examples of object detection tasks are face, eye and nose
detection, as well as logo detection.

The sample detection task in this document
is logo detection, since logo detection does not require the collection of
large set of registered and carefully marked object samples. Instead we assume
that from one prototype image, a very large set of derived object examples can
be derived (createsamples utility, see below).

A detailed description of the training/evaluation
algorithm can be found in [1] and [2].

## Samples Creation

For training a training samples must be
collected. There are two sample types: negative samples and positive samples.
Negative samples correspond to non-object images. Positive samples correspond
to object images.

### Negative Samples

Negative samples are taken from arbitrary
images. These images must not contain object representations. Negative samples
are passed through background description file. It is a text file in which each
text line contains the filename (relative to the directory of the description
file) of negative sample image. This file must be created manually. Note that
the negative samples and sample images are also called background samples or
background samples images, and are used interchangeably in this document

Example of negative description file:

Directory structure:

/img

  img1.jpg

  img2.jpg

bg.txt

File bg.txt:

img/img1.jpg

img/img2.jpg

### Positive Samples

Positive samples are created by createsamples
utility. They may be created from single object image or from collection of
previously marked up images.

The single object image may for instance
contain a company logo. Then are large set of positive samples are created from
the given object image by randomly rotating, changing the logo color as well as
placing the logo on arbitrary background.

The amount and range of randomness can be
controlled by command line arguments.

Command line arguments:

- vec <vec\_file\_name>

name of the
output file containing the positive samples for training

- img <image\_file\_name>

source object
image (e.g., a company logo)

- bg <background\_file\_name>

background
description file; contains a list of images into which randomly distorted
versions of the object are pasted for positive sample generation

- num <number\_of\_samples>

number of
positive samples to generate

- bgcolor <background\_color>

      background color (currently grayscale images are assumed); the
background color denotes the transparent color. Since there might be
compression artifacts, the amount of color tolerance can be specified by –bgthresh. All pixels between bgcolor-bgthresh and bgcolor+bgthresh are regarded as transparent.

- bgthresh <background\_color\_threshold>

- inv

      if specified, the colors will be inverted

- randinv

      if specified, the colors will be inverted randomly

- maxidev <max\_intensity\_deviation>

  maximal
intensity deviation of foreground samples pixels

- maxxangle <max\_x\_rotation\_angle>,

- maxyangle <max\_y\_rotation\_angle>,

- maxzangle <max\_z\_rotation\_angle>

      maximum rotation angles in radians

-show

      if specified, each sample will be shown. Pressing ‘Esc’ will
continue creation process without samples showing. Useful debugging option.

- w <sample\_width>

  width (in
pixels) of the output samples

- h <sample\_height>

  height (in
pixels) of the output samples

For following procedure is used to create a
sample object instance:

The source image is rotated random around
all three axes. The chosen angle is limited my -max?angle. Next pixels of
intensities in the range of [bg\_color-bg\_color\_threshold;
bg\_color+bg\_color\_threshold] are regarded as
transparent. White noise is added to the intensities of the foreground. If –inv key is
specified then foreground pixel intensities are inverted. If –randinv key is
specified then it is randomly selected whether for this sample inversion will
be applied. Finally, the obtained image is placed onto arbitrary background
from the background description file, resized to the pixel size specified by –w and –h and stored
into the file specified by the –vec command line parameter.

Positive samples also may be obtained from
a collection of previously marked up images. This collection is described by
text file similar to background description file. Each line of this file
corresponds to collection image. The first element of the line is image file
name. It is followed by number of object instances. The following numbers are
the coordinates of bounding rectangles (x, y, width, height).

Example of description file:

Directory structure:

/img

  img1.jpg

  img2.jpg

info.dat

File info.dat:

img/img1.jpg  1  140
100 45 45

img/img2.jpg  2  100
200 50 50   50 30 25 25

Image img1.jpg contains single object
instance with bounding rectangle (140, 100, 45, 45). Image img2.jpg contains
two object instances.

In order to create positive samples from
such collection –info argument should be specified instead of –img:

- info <collection\_file\_name>

description file
of marked up images collection

The scheme of sample creation in this case
is as follows. The object instances are taken from images. Then they are
resized to samples size and stored in output file. No distortion is applied, so
the only affecting arguments are –w, -h, -show and –num.

createsamples utility may be used for examining samples stored in positive samples
file. In order to do this only –vec, –w and –h parameters
should be specified.

Note that for training, it does not matter
how positive samples files are generated. So the createsamples utility is only one way
to collect/create a vector file of positive samples.

## Training

The next step after samples creation is
training of classifier. It is performed by the haartraining utility.

Command line arguments:

- data <dir\_name>

      directory name in which the trained classifier is stored

- vec <vec\_file\_name>

      file name of positive sample file (created by trainingsamples utility or by any other means)

- bg <background\_file\_name>

      background description file

- npos <number\_of\_positive\_samples>,

- nneg <number\_of\_negative\_samples>

      number of positive/negative samples used in training of each
classifier stage. Reasonable values are npos = 7000 and nneg = 3000.

- nstages <number\_of\_stages>

  number of
stages to be trained

- nsplits <number\_of\_splits>

      determines the weak classifier used in stage classifiers. If 1, then a simple stump classifier is used, if 2 and more, then CART classifier with number\_of\_splits internal (split)
nodes is used

- mem <memory\_in\_MB>

      Available memory in MB for precalculation. The more memory you
have the faster the training process

- sym (default),

- nonsym

      specifies whether the object class under training has vertical
symmetry or not. Vertical symmetry speeds up training process. For instance,
frontal faces show off vertical symmetry

- minhitrate <min\_hit\_rate>

      minimal desired hit rate for each stage classifier. Overall hit
rate may be estimated as (min\_hit\_rate^number\_of\_stages)

- maxfalsealarm <max\_false\_alarm\_rate>

      maximal desired false alarm rate for each stage classifier. Overall
false alarm rate may be estimated as (max\_false\_alarm\_rate^number\_of\_stages)

- weighttrimming <weight\_trimming>

  Specifies
wheter and how much weight trimming should be used. A decent choice is 0.90.

- eqw

- mode <BASIC (default) | CORE | ALL>

      selects the type of haar features set used in training. BASIC use
only upright features, while ALL uses the full set of upright and 45 degree
rotated feature set. See [1] for more details.

- w <sample\_width>,

- h <sample\_height>

      Size of training samples (in pixels). Must have exactly the same
values as used during training samples creation (utility trainingsamples)

Note: in order to use multiprocessor
advantage a compiler that supports OpenMP 1.0 standard should be used.

## Application

OpenCV cvHaarDetectObjects() function (in
particular haarFaceDetect demo) is used for detection.

### Test Samples

In order to evaluate the performance of
trained classifier a collection of marked up images is needed. When such
collection is not available test samples may be created from single object
image by createsamples utility. The scheme of test samples creation in this case is
similar to training samples creation since each test sample is a background
image into which a randomly distorted and randomly scaled instance of the
object picture is pasted at a random position.

If both –img and –info arguments are specified then
test samples will be created by createsamples utility. The sample image is arbitrary distorted as it was
described below, then it is placed at random location to background image and
stored. The corresponding description line is added to the file specified by –info argument.

The –w and –h keys determine the minimal size of
placed object picture.

The test image file name format is as
follows:

imageOrderNumber\_x\_y\_width\_height.jpg, where x, y, width and height are the coordinates of placed object bounding rectangle.

Note that you should use a background
images set different from the background image set used during training.

### Performance Evaluation

In order to evaluate the performance of the
classifier performance utility may be used. It takes a collection of marked up images,
applies the classifier and outputs the performance, i.e. number of found
objects, number of missed objects, number of false alarms and other
information.

Command line arguments:

- data <dir\_name>

      directory name in which the trained classifier is stored

- info <collection\_file\_name>

      file with test samples description

- maxSizeDiff <max\_size\_difference>,

- maxPosDiff <max\_position\_difference>

      determine the criterion of reference and detected rectangles
coincidence. Default values are 1.5 and 0.3 respectively.

- sf <scale\_factor>,

      detection parameter. Default value is 1.2.

- w <sample\_width>,

- h <sample\_height>

      Size of training samples (in pixels). Must have exactly the same
values as used during training (utility haartraining)

## References

[1] Rainer Lienhart and Jochen Maydt. An
Extended Set of Haar-like Features for Rapid Object Detection. Submitted to
ICIP2002.

[2] Alexander Kuranov, Rainer Lienhart, and
Vadim Pisarevsky. An Empirical Analysis of Boosting Algorithms for Rapid
Objects With an Extended Set of Haar-like Features. Intel Technical Report
MRL-TR-July02-01, 2002.
