# Supplementary material for: Rapid whole brain imaging of neural activity in freely behaving larval zebrafish (Danio rerio)
Source: eLife. 2017 Sep 20;6:e28158. doi: 10.7554/eLife.28158 (PMC5644961; doi:10.7554/eLife.28158)
Supplement: Source code 3. [file elife-28158-code3.zip › RT analysis code/OpenCV1.1pre.zip!/OpenCV/docs/IPLMAN.pdf]

intel®

Intel® Image Processing Library

Reference Manual

Copyright © 1997-2000, Intel Corporation
All Rights Reserved
Issued in U.S.A.
Document Number 663791-005

How to Use This Online Manual

Click to hide or show subtopics when the bookmarks are shown.

Double-click to jump to a topic when the bookmarks are shown.

Click to display bookmarks.

Click to display thumbnails.

Click to close bookmark or thumbnail view.

Click and use on the page to drag the page in vertical direction.

Click and drag to the page to magnify the view.

Click and drag to the page to reduce the view.

Click and drag the selection cursor to the page.

Click to go to the first page of the manual.

Click to go to the previous page.

Click to go to the next page.

Click to go to the last page.

Click to return back to the previous view. Use this button when you need to go back after using the jump button (see below).

Click to go forward from the previous view.

Click to set 100% of the page view.

Click to display the entire page within the window.

Click to fill the width of the window.

Click to open a dialog to search for a word or multiple words.

Click jump button on manual pages to jump to the related subjects. Use the return back icon above to go back.

Printing an Online File. Select **Print** from the **File** menu to print an online file. The dialog that opens allows you to print full text, range of pages, or selection.
Viewing Multiple Online Manuals. Select **Open** from the **File** menu, and open a .PDF file you need. Select **Cascade** from the **Window** menu to view multiple files.
Resizing the Bookmark Area. Drag the double-headed arrow that appears on the area's border as you pass over it.
Jumping to Topics. Throughout the text of this manual, you can jump to different topics by clicking on keywords printed in green color, underlined style or on page numbers in a box.

To return to the page from which you jumped, use the icon in the tool bar. Try this example:

This software is briefly described in the Overview; see page 1-1.

If you click on the phrase printed in green color, underlined style, or on the page number, the Overview opens.

Intel® Image Processing Library Reference Manual

Document Number: 663791-005

World Wide Web: http://developer.intel.com

Revision	Revision History	Date
-001	First release.	07/97
-002	Documents Image Processing Library release 2.0	06/98
-003	Added the functions MpyRCPack2D, Remap, DecimateExt, Scale, ScaleFP, ColorTwistFP, MinMaxFP, and the compare functions.	01/99
-004	Documents Image Processing Library release 2.2	02/00
-005	Documents Image Processing Library release 2.5	08/00

This documentation as well as the software described in it is furnished under license and may only be used or copied in accordance with the terms of the license. The information in this document is furnished for informational use only, is subject to change without notice, and should not be construed as a commitment by Intel Corporation. Intel Corporation assumes no responsibility or liability for any errors or inaccuracies that may appear in this document or any software that may be provided in association with this document. Except as permitted by such license, no part of this document may be reproduced, stored in a retrieval system, or transmitted in any form or by any means without the express written consent of Intel Corporation.

Information in this document is provided in connection with Intel® products. No license, express or implied, by estoppel or otherwise, to any intellectual property rights is granted by this document. Except as provided in Intel's Terms and Conditions of Sale or License Agreement for such products, Intel assumes no liability whatsoever, and Intel disclaims any express or implied warranty, relating to sale and/or use of Intel products including liability or warranties relating to fitness for a particular purpose, merchantability, or infringement of any patent, copyright or other intellectual property right. Intel products are not intended for use in medical, life saving, or life sustaining applications. Intel may make changes to specifications and product descriptions at any time, without notice.

Designers must not rely on the absence or characteristics of any features or instructions marked "reserved" or "undefined." Intel reserves these for future definition and shall have no responsibility whatsoever for conflicts or incompatibilities arising from future changes to them.

Processors may contain design defects or errors known as errata which may cause the product to deviate from published specifications. Current characterized errata are available on request.

Intel, the Intel logo, and Pentium are registered trademarks, and MMX is a trademark of Intel Corporation.

*Third-party marks and brands are the property of their respective owners.

Copyright 1997-2000, Intel Corporation. All Rights Reserved.

Contents

Chapter 1 Overview

About This Software 1-1
Hardware and Software Requirements 1-1
About This Manual 1-2
Manual Organization 1-2
Function Descriptions 1-4
Audience for This Manual 1-4
Online Version 1-5
Sources of Related Information 1-5
Notational Conventions 1-5
Font Conventions 1-5
Naming Conventions 1-6
Function Name Conventions 1-6
X-Y Argument Order Convention 1-7

Chapter 2 Image Architecture

Data Architecture 2-1
Color Models 2-1
Data Types and Palettes 2-2
The Sequence and Order of Color Channels 2-3
Coordinate Systems 2-4
Image Regions of Interest 2-4
Alpha (Opacity) Channel 2-7
Scanline Alignment 2-7
Image Dimensions 2-7
Execution Architecture 2-8

Handling Overflow and Underflow 2-8
In-Place and Out-of-Place Operations 2-8
Image Tiling 2-8
Tile Size 2-9
Call-backs... 2-9
ROI and Tiling 2-10
In-Place Operations and Tiling 2-10

Chapter 3 Error Handling

Error-handling Functions.. 3-2
Error 3-2
GetErrStatus 3-3
GetErrMode.. 3-4
ErrorStr.. 3-5
RedirectError 3-6
NullDevReport 3-7
StdErrReport 3-7
GuiBoxReport... 3-7
Error Macros 3-9
Status Codes 3-10
Application Notes... 3-12
Error Handling Example 3-13
Adding Your Own Error Handler....................................... 3-15

Chapter 4 Image Creation and Access

Image Header and Attributes 4-4
Tiling Fields in the IplImage Structure........................... 4-8
IplTileInfo Structure 4-8
Creating Images.. 4-9
CreateImageHeader 4-9

AllocateImage 4-13
AllocateImageFP 4-13
DeallocateImage 4-15
CloneImage 4-15
Deallocate 4-16
CheckImageHeader 4-17
CreateImageJaehne 4-18
Setting Regions of Interest 4-20
CreateROI 4-21
DeleteROI 4-21
SetROI 4-22
Image Borders and Image Tiling 4-23
SetBorderMode 4-23
CreateTileInfo 4-25
SetTileInfo 4-26
DeleteTileInfo 4-26
Memory Allocation Functions 4-27
Malloc 4-27
wMalloc 4-28
iMalloc 4-28
sMalloc 4-29
dMalloc 4-30
Free 4-30
Image Data Exchange 4-31
Set 4-31
SetFP 4-31
Copy 4-32
Exchange 4-35
Convert 4-36

PutPixel 4-38
GetPixel 4-38
Scale 4-40
ScaleFP 4-41
NoiseImage 4-42
NoiseUniformInit 4-43
NoiseUniformInitFp 4-43
NoiseGaussianInit 4-44
NoiseGaussianInitFp 4-44
Working in the Windows DIB Environment 4-45
TranslateDIB 4-47
ConvertFromDIB 4-50
ConvertFromDIBSep 4-53
ConvertToDIB 4-54
ConvertToDIBSep 4-55

Chapter 5 Arithmetic and Logical Operations

Monadic Arithmetic Operations 5-3
AddS 5-3
AddSFP 5-3
SubtractS 5-4
SubtractSFP 5-4
MultiplyS 5-4
MultiplySFP 5-4
MultiplySScale 5-5
Square 5-6
Abs 5-6
Dyadic Arithmetic Operations 5-7
Add 5-7
Subtract 5-8

Multiply 5-8
MultiplyScale 5-9
Monadic Logical Operations... 5-10
LShiftS 5-10
RShiftS 5-11
Not 5-12
AndS 5-12
OrS... 5-13
XorS 5-14
Dyadic Logical Operations 5-14
And... 5-15
Or 5-15
Xor 5-16
Image Compositing Based on Opacity 5-16
Using Pre-multiplied Alpha Values 5-17
AlphaComposite, AlphaCompositeC............................. 5-18
PreMultiplyAlpha 5-24

Chapter 6 Image Filtering

Linear Filters 6-2
Blur... 6-2
2D Convolution... 6-3
CreateConvKernel... 6-5
CreateConvKernelChar 6-5
CreateConvKernelFP 6-5
GetConvKernel.. 6-6
GetConvKernelChar 6-6
GetConvKernelFP 6-6
DeleteConvKernel 6-8
DeleteConvKernelFP... 6-8

Convolve2D 6-8
Convolve2DFP 6-8
ConvolveSep2D 6-11
ConvolveSep2DFP 6-11
FixedFilter 6-12
Non-linear Filters 6-14
MedianFilter 6-15
MaxFilter 6-18
MinFilter 6-19

Chapter 7 Linear Image Transforms

Fast Fourier Transform 7-1
Real-Complex Packed (RCPack2D) Format 7-1
RealFft2D 7-4
CcsFft2D 7-7
MpyRCPack2D 7-8
Discrete Cosine Transform 7-8
DCT2D 7-9

Chapter 8 Morphological Operations

Erode 8-2
Dilate 8-5
Open 8-6
Close 8-7

Chapter 9 Color Space Conversion

Reducing the Image Bit Resolution 9-3
ReduceBits 9-3
Conversion from Bitonal to Gray Scale Images 9-7
BitonalToGray 9-7

Conversion of Absolute Colors to and from Palette Colors. 9-7
Conversion from Color to Gray Scale................................ 9-8
ColorToGray.. 9-8
Conversion from Gray Scale to Color (Pseudo-color) 9-9
GrayToColor.. 9-9
Conversion of Color Models.. 9-10
Data ranges in the HLS and HSV Color Models 9-11
RGB2HSV.. 9-12
HSV2RGB.. 9-12
RGB2HLS 9-13
HLS2RGB 9-13
RGB2LUV 9-14
LUV2RGB 9-14
RGB2XYZ 9-15
XYZ2RGB 9-15
RGB2YCrCb.. 9-16
YCrCb2RGB.. 9-16
RGB2YUV.. 9-17
YUV2RGB.. 9-17
YCC2RGB.. 9-18
Using Color-Twist Matrices 9-18
CreateColorTwist... 9-19
SetColorTwist.. 9-20
ApplyColorTwist 9-21
DeleteColorTwist... 9-22
ColorTwistFP... 9-23

Chapter 10 Histogram, Threshold, and Compare Functions

Thresholding... 10-2
Threshold 10-2

Lookup Table (LUT) and Histogram Operations 10-5
The IplLUT Structure 10-5
ContrastStretch 10-7
ComputeHisto 10-9
HistoEqualize 10-10
Comparing Images 10-12
Greater 10-13
Less 10-14
Equal 10-15
EqualFPEps 10-16
GreaterS 10-17
GreaterSFP 10-18
LessS 10-19
LessSFP 10-20
EqualS 10-21
EqualSFP 10-22
EqualSFPEps 10-23

Chapter 11 Geometric Transforms

Changing the Image Size 11-3
Zoom 11-4
Decimate 11-5
DecimateBlur 11-6
Resize 11-7
ZoomFit 11-8
DecimateFit 11-8
ResizeFit 11-8
Changing the Image Orientation 11-9
Rotate 11-9
GetRotateShift 11-11

RotateCenter 11-13
Mirror 11-14
Warping 11-15
Shear 11-16
WarpAffine 11-17
GetAffineBound 11-18
GetAffineQuad 11-18
GetAffineTransform 11-19
WarpBilinear 11-20
GetBilinearBound 11-22
GetBilinearQuad 11-22
GetBilinearTransform 11-23
WarpPerspective 11-24
GetPerspectiveBound 11-26
GetPerspectiveQuad 11-26
GetPerspectiveTransform 11-27
Arbitrary Transforms 11-28
Remap 11-28

Chapter 12 Image Statistics Functions

Image Norms 12-2
Norm 12-2
Image Moments 12-5
Moments 12-6
GetSpatialMoment 12-6
GetCentralMoment 12-7
GetNormalizedSpatialMoment 12-7
GetNormalizedCentralMoment 12-8
SpatialMoment 12-9
CentralMoment 12-9

NormalizedSpatialMoment.. 12-10
NormalizedCentralMoment... 12-11
Cross-Correlation... 12-12
NormCrossCorr... 12-13
Minimum and Maximum.. 12-14
MinMaxFP... 12-14

Chapter 13 User Defined Functions

UserProcess... 13-5
UserProcessFP... 13-7
UserProcessPixel.. 13-8

Chapter 14 Library Version

GetLibVersion.. 14-1

Appendix A Supported Image Attributes and Operation Modes

Appendix B Interpolation Algorithms in Geometric Transforms

Bibliography

Glossary

Index

Tables

Table 2-1 Data Ordering 2-3
Table 3-1 iplError() Status Codes...................................... 3-10
Table 4-1 Image Creation, Data Exchange and Windows DIB Functions....................................... 4-1
Table 4-2 Image Header Attributes 4-4
Table 5-1 Image Arithmetic and Logical Operations........... 5-1
Table 5-2 Types of Image Compositing Operations 5-22
Table 6-1 Image Filtering Functions.................................. 6-1
Table 7-1 Linear Image Transform Functions 7-1
Table 7-2 FFT Output in RCPack2D Format for Even *K* 7-3
Table 7-3 FFT Output in RCPack2D Format for Odd *K*...... 7-3
Table 7-4 RealFFT2D Output Sample for $K = 4$, $L = 4$ 7-3
Table 8-1 Morphological Operation Functions.................... 8-1
Table 9-1 Color Space Conversion Functions.................... 9-1
Table 9-2 Source and Resultant Image Data Types for Reducing the Bit Resolution 9-6
Table 9-3 Source and Resultant Image Data Types for Conversion from Color to Gray Scale.............. 9-8
Table 9-4 Source and Resultant Image Data Types for Conversion from Gray Scale to Color.............. 9-9
Table 10-1 Histogram,Threshold, and Compare Functions 10-1
Table 11-1 Image Geometric Transform Functions............ 11-1
Table 12-1 Image Statistics Functions 12-1
Table A-1 Image Attributes and Modes of Data Exchange Functions.................................. A-1
Table A-2 Windows DIB Conversion Functions.................. A-2
Table A-3 Image Attributes and Modes of Arithmetic and Logical Functions.. A-3

Table A-4 Image Attributes and Modes of Alpha-Blending Functions A-4
Table A-5 Image Attributes and Modes of Filtering Functions... A-4
Table A-6 Image Attributes and Modes of Fourier and DCT Functions................................ A-4
Table A-7 Image Attributes and Modes of Morphological Operations A-5
Table A-8 Image Attributes and Modes of Color Space Conversion Functions..................... A-5
Table A-9 Image Attributes and Modes of Histogram and Thresholding Functions............... A-6
Table A-10 Image Attributes and Modes of Geometric Transform Functions.......................... A-6
Table A-11 Image Attributes and Modes of Image Statisctics Functions................................ A-7
Table A-12 Image Attributes and Modes of Functions for User-Defined Image Processing A-7
Table B-1 Interpolation Modes Supported by Geometric Transform Functions.......................... B-2

Figures

Figure 2-1 Setting an ROI for Multi-Image Operations....... 2-6
Figure 4-1 RGB Image with a Rectangular ROI and a COI 4-6
Figure 4-2 Example of a generated test image 4-19
Figure 8-1 Erosion in a Rectangular ROI 8-3
Figure 9-1 Example of the source and resultant images for the bit reducing function 9-5
Figure B-1 Linear Interpolation... B-4
Figure B-2 Cubic Interpolation.. B-6
Figure B-3 Super-sampling Weights.................................. B-7

Examples

Example 3-1 Error Functions 3-13
Example 3-2 Output for the Error Function Program (IPL_ErrModeParent).. 3-15
Example 3-3 Output for the Error Function Program (IPL_ErrModeParent).. 3-15
Example 3-4 A Simple Error Handler 3-17
Example 4-1 Creating and Deleting an Image Header 4-11
Example 4-2 Allocating and Deallocating the Image Data.. 4-14
Example 4-3 Setting the Border Mode for an Image 4-25
Example 4-4 Allocating an Image and Setting Its Pixel Values 4-32
Example 4-5 Copying Image Pixel Values 4-34
Example 4-6 Converting Images....................................... 4-37
Example 4-7 Using the Function iplGetPixel()................... 4-39
Example 4-8 Translating a DIB Image Into an IplImage..... 4-48
Example 4-9 Converting a DIB Image Into an IplImage 4-51
Example 6-1 Computing the 2-dimensional Convolution.... 6-9
Example 6-2 Applying the Median Filter............................ 6-16
Example 7-1 Computing the FFT of an Image 7-5
Example 7-2 Computing the DCT of an Image.................. 7-10
Example 8-1 Code Used to Produce Erosion in a Rectangular ROI 8-4
Example 10-1 Conversion to a Bitonal Image 10-4
Example 10-2 Using the Function iplContrastStretch() to Enhance an Image 10-7
Example 10-3 Computing and Equalizing the Image Histogram... 10-11
Example 11-1 Using Macro Definition to Resize an Image.. 11-9

Example 11-2 Rotating an Image 11-11
Example 11-3 Using Macro Definition to Rotate
an Image 11-14
Example 11-4 Re-mapping an Image 11-29
Example 12-1 Computing the Norm of Pixel Values........... 12-4
Example 13-1 Image Channel Values Processing
by User-Defined Function 13-6
Example 13-2 Pixel Values Processing
by User-Defined Function 13-9

Overview

1

This manual describes the structure, operation and functions of the Intel® Image Processing Library. This library supports many functions whose performance can be significantly enhanced on processors with the MMX™ technology, as well as on Intel® Pentium® III processors.

The manual describes the library's data and execution architecture and provides detailed descriptions of the library functions.

This chapter introduces the Image Processing Library and explains the organization of this manual.

About This Software

The Image Processing Library focuses on taking advantage of the parallelism of the new SIMD (single-instruction, multiple-data) instructions of the latest generations of Intel processors. These instructions greatly improve the performance of computation-intensive image processing functions. Most functions in the Image Processing Library are specially optimized for the latest generations of processors. However, all functions will successfully execute on older processors as well.

The library does not support the reading and writing of a wide variety of image file formats or the display of images.

Hardware and Software Requirements

The Image Processing Library runs on personal computers that are based on Intel® architecture processors and running Microsoft* Windows*, Windows 95, 98, or Windows NT* operating system. The library integrates into the customer's application or library written in C or C++.

About This Manual

This manual provides a background of the image and execution architecture of the Image Processing Library as well as detailed descriptions of the library functions. The functions are combined in groups by their functionality. Each group of functions is described in a separate chapter (chapters 3 through 14).

Manual Organization

This manual contains fourteen chapters:

Chapter 1	"Overview." Introduces the Image Processing Library, explains the manual organization and notational conventions.
Chapter 2	"Image Architecture." Describes the supported image architecture (color models, data types, data order, and so on) as well as the execution architecture and image tiling.
Chapter 3	"Error Handling." Provides information on the error-handling functions included with the library. User-defined error handler is also described.
Chapter 4	"Image Creation and Access." Describes the functions used to: create, set, and access image attributes; set image border and tiling; and allocate the memory for different data types. The chapter also describes the functions that facilitate operations in the window environment.
Chapter 5	"Image Arithmetic and Logical Operations." Describes image processing operations that modify pixel values using simple arithmetic or logical operations, as well as alpha-blending.

Chapter 6	"Image Filtering." Describes linear and non-linear filtering operations that can be applied to images.
Chapter 7	"Linear Image Transforms." Describes the fast Fourier transform (FFT) and Discrete Cosine Transform (DCT) implemented in the library.
Chapter 8	"Morphological Operations." Describes the functions that perform erosion, dilation, and their combinations.
Chapter 9	"Color Space Conversion." Describes the color space conversions supported in the library; for example, color reduction from high resolution color to low resolution color; conversion from palette to absolute color and vice versa; conversion to different color models.
Chapter 10	"Histogram, Threshold, and Compare Functions." Describes functions that treat an image on a pixel-by-pixel basis: contrast stretching, histogram computation, histogram equalization and thresholding; compare functions.
Chapter 11	"Image Geometric Transforms." Describes the supported geometric transformations: resizing, flipping, rotation, and various kinds of warping.
Chapter 12	"Image Statistics Functions." Describes functions that allow you to compute image norms, moments, minimum and maximum values.
Chapter 13	"User-Defined Functions." Describes library functions that enable you to create and use your own image processing functions.
Chapter 14	"Library Version." Describes the function `iplGetLibVersion()` that returns the library version and other information about the library.

The manual also includes a Glossary, Bibliography, and Index, as well as two appendixes that list supported image attributes and operation modes and describe interpolation algorithms used in the library.

Function Descriptions

In Chapters 3 through 14, each function is introduced by name (without the `ipl` prefix) and a brief description of its purpose. This is followed by the function call sequence, more detailed description of the function's purpose, and definitions of its arguments. The following sections are included in each function description:

Arguments	Describes all the function arguments.
Discussion	Defines the function and describes the operation performed by the function. Often, code examples and the equations the function implements are included.
Return Value	If present, describes a value indicating the result of the function execution.
Application Notes	If present, describe any special information which application programmers or other users of the function need to know.
See Also	If present, lists the names of functions which perform related tasks.

Audience for This Manual

The manual is intended for the developers of image processing applications and image processing libraries. Both parts of the audience are expected to be experienced in using C and to have a working knowledge of the vocabulary and principles of image processing. The developers of image processing software can use the Image Processing Library capabilities to improve performance on the latest generations of processors.

Online Version

This manual is available in an online hypertext format. To obtain a hard copy of the manual, print the online file using the printing capability of Adobe* Acrobat*, the tool used for the online presentation of the document.

Sources of Related Information

For more information about computer graphics concepts and objects, refer to the books and materials listed in the Bibliography. For the latest information about the Image Processing Library, such as new releases, product announcements, updates, and online technical support, check out our Web site at http://developer.intel.com.

Notational Conventions

In this manual, notational conventions include:

- Fonts used for distinction between the text and the code
- Naming conventions
- Function name conventions

Font Conventions

The following font conventions are used:

`UPPERCASE COURIER`	Used in the text for constant identifiers; for example, `IPL_DEPTH_1U`.
`lowercase courier`	Mixed with the uppercase in function names as in `SetExecutionMode`; also used for key words in code examples; for example, in the function call statement `void iplSquare()`.
`lowercase mixed with UpperCase Courier italic`	Variables in arguments and parameters discussion; for example, *`mode`*, *`dstImage`*.

Naming Conventions

The following data type conventions are used by the library:

- Constant identifiers are in uppercase; for example, `IPL_SIDE_LEFT`.
- All constant identifiers have the `IPL` prefix.
- All function names have the `ipl` prefix. In code examples, you can distinguish the library interface functions from the application functions by this prefix.

NOTE. *In this manual, the* `ipl` *prefix in function names is always used in the code examples. In the text, this prefix is sometimes omitted.*

- All image header structures have the `Ipl` prefix; for example, `IplImage`, `IplROI`.
- Each new part of a function name starts with an uppercase character, without underscore; for example, `iplAlphaComposite`.

Function Name Conventions

The function names in the library typically begin with the `ipl` prefix and have the following general format:

```
ipl < action > < target > < mod >()
```

where

`action`	indicates the core functionality; for example, `-Set-`, `-Create-`, or `-Convert-`.
`target`	indicates the area where image processing is being enacted; for example, `-ConvKernel` or `-FromDIB`.

In a number of cases, the target consists of two or more words; for example, `-ConvKernel` in the function `CreateConvKernel`.
Some function names consist of an *`action`* or

	`target` only; for example, the functions `Multiply` or `RealFft2D`, respectively.
`mod`	The *`mod`* field is optional and indicates a modification to the core functionality of a function. For example, in the name `iplAlphaCompositeC()`, `C` indicates that this function is using constant alpha values.

X-Y Argument Order Convention

Where applicable, the Image Processing Library functions use the following order of arguments:

`x, y`	(`x` first, then `y`)
`nCols, nRows`	(columns first, then rows)
`width, height`	(width first, then height).

This page is left blank for double-sided printing

This page is left blank for double-sided printing

Image Architecture

2

This chapter describes the data and execution architecture of the Image Processing Library. It introduces the library's color models, data types, coordinate systems, regions of interest, data alignment, in-place and not-in-place execution, and image tiling.

Data Architecture

Any image in the Image Processing Library has a header that describes the image as a list of attributes and pointers to the data associated with the image. Library functions use the image header to get the format and characteristics of the image(s) passed to the functions. Based on the information obtained from the header, the functions make appropriate calls to set the data structures. Images can have different organization of data. The library supports numerous data formats that use different color models, data types, data order, and coordinate systems.

Color Models

The library image format supports the following color models:

- Monochrome or gray scale image (one color channel)
- Color image (3 or 4 color channels)
- Multi-spectral image (any number of channels).

Color models are defined by the number of channels and the colors they contain. Examples of three-channel models are RGB, HSV, CMY, and YCC. Examples of four-channel color models are CMYK and RGBA.

Image processing operations can be performed on one or all channels in the image. The operations are performed without specific identification of the colors, unless it is a certain color conversion operation where color identification is required.

The multi-spectral image (MSI) model is used for general purpose images. It is used for any kind of multi-spectral data and any kind of image. For example, the Fourier transform operation writes transform coefficients of color or monochrome images to this model—one channel for each channel in the input. The result can be viewed as an MSI image. An MSI image can contain any number of color channels; they may even correspond to invisible parts of the spectrum. The library functions do not need to identify any specific MSI image channels.

Data Types and Palettes

The parameter that determines the image data type is the pixel depth in bits. The data could be signed integer, unsigned integer, or floating-point. The following data types are supported for various color models (s = signed, u = unsigned, f = float):

Gray scale	1, 8s, 8u, 16s, 16u, and 32f bits per pixel
Color (three-channel)	8u and 16u bits per channel
Four-channel and MSI	8s, 8u, 16s, 16u, 32s, and 32f bits per channel.

The library supports only absolute color images in which each pixel is represented by the channel intensities. For example, in an absolute color 24-bit RGB image, three bytes (24 bits) per pixel represent the three channel intensities. LUT (lookup table) images, that is, palette color images are not supported. You must convert palette images to absolute color images for further processing by the library functions. There are special functions for converting DIB palette images to absolute color images.

Color images with 8, 16, or 32 bits per channel simply pack each channel, respectively, into a byte, word, or doubleword. All channels within a given image have the same data type.

Signed data (8s, 16s, or 32s) are used for storing the output of some image processing operations; for example, this is the case for transforms such as FFT. Unless specified otherwise, signed data cannot be used as input to image processing operations.

The Sequence and Order of Color Channels

Channel sequence corresponds to the order of the color channels in absolute color images. For example, in an RGB image the channels could be stored in the sequence RGB or in the sequence BGR.

NOTE. *For functions that perform color space conversions or image format conversions, the channel sequence information is required and therefore must be provided. All other functions ignore channel sequence.*

For images with pixel-oriented data, the channel sequence corresponds to the color data order for each pixel. Data ordering corresponds to the way the color data is arranged: by planes or by pixels. Table 2-1 lists the orderings that are supported for planes and for pixels.

Table 2-1 Data Ordering

Data Ordering	Description	RGB Example<br>(channel ordering = RGB)
Pixel-oriented	All channels for each pixel are clustered.	RGBRGBRGB (line 1)<br>RGBRGBRGB (line 2)<br>RGBRGBRGB (line 3)
Plane-oriented	All image data for each channel is contiguous followed by the next channel.	RRRRRRRRR (line 1)<br>RRRRRRRRR (line 2) R plane<br>RRRRRRRRR (line 3)<br><br>GGGGGGGGG (line 1)<br>GGGGGGGGG (line 2) G plane<br>GGGGGGGGG (line 3)<br>...

Coordinate Systems

Two coordinate systems are supported by the library's image format.

- The origin of the image is in the top left corner, the x values increase from left to right, and y values increase from top to bottom.
- The origin of the image is in the bottom left corner, the x values increase from left to right, and y values increase from the bottom to the top.

Image Regions of Interest

A very important concept in the Image Processing Library architecture is an image's region of interest (ROI). All image processing functions can operate not only on entire images but also on image regions.

Depending on the processing needs, the following image regions can be specified:

- **Channel of interest** (COI). A COI can be one or all channels of the image. By default, unless the COI is changed by the `SetROI()` function, processing will be carried out on all channels in the image.
- **Rectangular region of interest** (rectangular ROI). A rectangular ROI is a portion of the image or, possibly, the entire image. By default, unless changed by the `SetROI()` function, the entire image is the rectangular region of interest.
- **Mask region of interest** (mask ROI). It is specified by another (bitonal) image pointed to by the *`maskROI`* pointer of the `IplImage` structure.
  A mask ROI allows an application to determine on a pixel-by-pixel basis whether to perform an operation. Pixels corresponding to zeros in the mask are not read (if in a source image) or written (if in the destination image). Pixels corresponding to 1's in the mask are processed normally.
  The origin of the mask ROI is aligned to the origin of the rectangular ROI if there is one, or the origin of the image.

An image can simultaneously have any combination of a rectangular ROI, a mask ROI, and a COI. Operations are performed on the intersection of all

applicable ROIs. For example, if an image has both types of ROI and a COI, operations are performed only on the values of this COI, and only for those pixels that belong to the intersection of mask ROI and rectangular ROI.

Both the source and destination image can have a region of interest. In such cases, operations will be performed on the intersection of the ROIs. Thus, an image region of interest specifies some part of an image or the entire image. Once set, the region information of the image remains the same until changed by the function `SetROI()`.

NOTE. *Not all functions support mask ROI. For example, FFT functions use only rectangular ROI and COI even if you specify a mask ROI.*

Setting an ROI for Multi-Image Operations

Figure 2-1 illustrates image processing operations that take one or more input images and store the results onto an output image. (Mask ROIs are not set for the images in this figure.) Before performing any operations, each function checks that the ROI sizes and offsets are positive. However, not all functions check that the ROI is within the actual image borders.

All images (input and output) in Figure 2-1 have rectangular ROIs that specify either the entire image or specific regions set by the `SetROI()` function. The first step is to align the rectangular ROIs of all the images so that their top left corners coincide. The operation is, then, performed in the rectangular region where all the images overlap. This scheme gives much flexibility, effectively enabling translation of image data (even for equal-size images) from one region of an input image to another region of an output image.

To successfully perform an image processing operation, one of the following conditions must be met for the channel of interest (COI):

- Each image (input and output) has one COI,
- Each image (input and output) has all channels included in the ROI (COI = 0) and all images (input and output) have the same number of channels (one or more).

If one image (input or output) has one channel in its COI and another image (input or output) has more than one channel included in its COI, an error will occur.

Figure 2-1 Setting an ROI for Multi-Image Operations

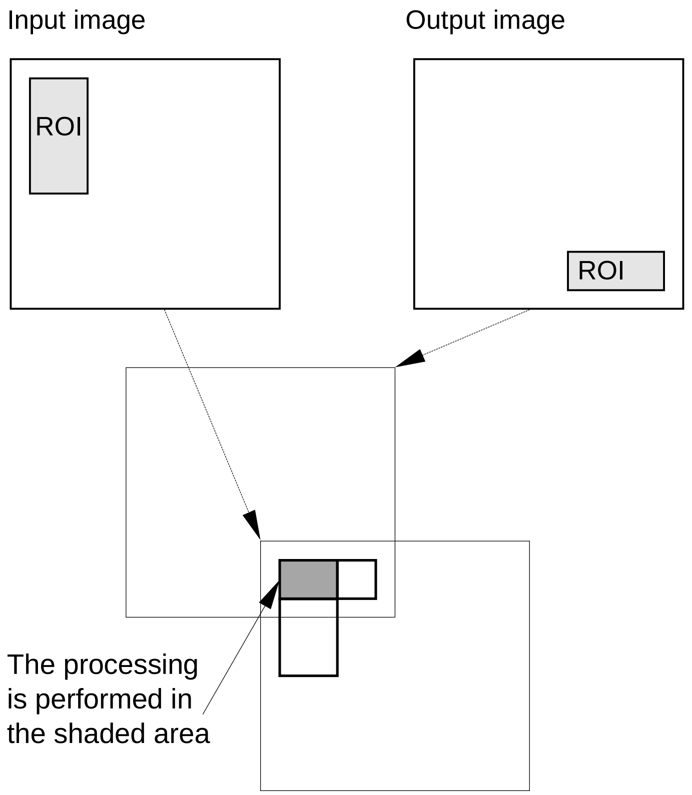

Alpha (Opacity) Channel

In addition to the color channels, an image can have one alpha channel, also known as an opacity channel, which is mainly used for image compositing operations (see "Image Compositing Based on Opacity" in Chapter 5). The alpha channel must be the last channel in the image.

The interpretation of operations on the alpha channel is usually different from that for color channels. For example, adding a constant to the RGB channels in an RGBA image would brighten the image, while adding a constant to the A (alpha) channel would make the image more opaque.

For this reason, by default most functions ignore the alpha channel if one is specified. The exceptions are the compositing functions, which use this channel as the image's opacity value, and geometric transform functions, which treat it as any other channel.

To apply any other function to the alpha channel, in the `IplImage` structure temporarily set the *`alphaChannel`* field to 0 before calling the function.

Scanline Alignment

Image row data (scanline) can be aligned on doubleword (32-bit) or quadword (64-bit) boundaries. Each row is padded with zeros if required. For maximum performance with MMX technology, it is important to have the image data aligned on quadword boundaries.

Image Dimensions

There is no practical limit of the image size. A long integer is used for the height and width of the image. This allows you to create images of such sizes that are much beyond the hardware and OS constraints of today's PCs or workstations. For large image support, see also "Image Tiling."

Execution Architecture

Handling Overflow and Underflow

Overflow and underflow are handled in each image processing function. The Image Processing Library uses saturation to prevent the pixel values from potential overflow or underflow. Thus, when an overflow of a pixel value is about to happen, this value is clamped to the maximum permissible value (for example, 255 for an unsigned byte). Similarly, when underflow of a value is about to happen, it is clamped to the minimum permissible value, which is always zero for the case of unsigned bytes.

In-Place and Out-of-Place Operations

Image processing operations in the library can be in-place or out-of-place operations. With an in-place operation, the output image is one of the input images modified (that is, the pointer to the output image is the same as the pointer to the input one). With an out-of-place operation, the output image is a new image, not the same as any of the input images. Not all functions can perform in-place operations. See Appendix A to check if a partucular function supports in-place operation.

Image Tiling

Tiling is a method of image representation in which the image is broken up into smaller images, or tiles, to allow for complicated memory management schemes. Conceptually, the whole image would be reconstructed by arranging the individual tiles in a grid. But the intent of the tiling mechanism is to allow only a few of these tiles within an image to reside in memory at one time. The application provides an actual memory location for a tile only when requested to do so.

Most functions can use tiled images in the same way as non-tiled, and procuce the same results. However, there are some differences, particularly in the call-back requirement (see “Call-backs“ for more information).

This section gives a short overview of image tiling in the Image Processing Library. In Chapter 4 you will find more information about tiling, namely, the descriptions of the `TileInfo` structure, the `imageID` parameter, and the functions `CreateTileInfo`, `SetTileInfo`, and `DeleteTileInfo`.

Tile Size

In the Image Processing Library, all tiles must be of the same size, including those on the edge of an image. The tiles on the edge of an image must contain valid data up to the border of the image; beyond that, the pixels are ignored, and the border mode is used instead.

The size of the image tiles is contained within the `IplTileInfo` structure. It is restricted to being an even multiple of 8 in each dimension. Typical tile sizes are 32x32 and 64x64.

For functions that take more than one source image, either all source images must be tiled with equally-sized tiles or they must all be non-tiled. The source and destination images tiling and tile sizes need not be the same.

Call-backs

For tiled images, the `IplImage` structure does not contain a pointer to image data; therefore, functions operating on tiled images must acquire data tile-by-tile. To do this, the library uses a system of call-backs, in which the functions request pointers to individual tiles based on need.

The call-back system is implemented (by the library user) as a single function, the prototype and behavior of which are specified below. When called **by the library**, this function must provide or release one tile's worth of data. The function is specified to the library in the `callBack` field of the `IplTileInfo` structure. The prototype is as follows:

```
void (*IplCallBack)  (const IplImage* img, int xIndex,
                      int yIndex, int mode);
```

where `img` is the header of the parent image;
`xIndex` and `yIndex` are the indices of the requested tile; they refer to the

tile number, not pixel number, and count from the origin at (0,0); `mode` is one of the following:

`IPL_GET_TILE_TO_READ`	get a tile for reading;<br>the tile data must be returned in<br>`img->tileInfo->tileData`<br>and must not be changed;
`IPL_GET_TILE_TO_WRITE`	get a tile for writing;<br>the tile data must be returned in<br>`img->tileInfo->tileData`<br>and may be changed;<br>changes will be reflected in the image;
`IPL_RELEASE_TILE`	release tile; commit writes.

Memory pointers provided by a get function will not be used after the corresponding release function has been called.

ROI and Tiling

The meaning and behavior of ROI for a tiled image are identical to those for a non-tiled image. As with all coordinates in tiled images, the origin of the ROI is offset from the origin of the image, not of any one tile.

In-Place Operations and Tiling

Many functions can perform in-place operations even with tiling; see Appendix A to check whether this feature is supported for a particular function. If the source and destination image pointers are not equal, no support for source and destination overlap is provided.

Note that the presence of the `IplROI` structure does not affect this restriction.

Error Handling

3

This chapter describes the error handling facility of the Image Processing Library. The library functions report a variety of errors including bad arguments and out-of-memory conditions.

Most functions in the library do not return any status code. When a function detects an error, it sets the error status code by calling `iplSetErrStatus()`. This allows the error handling mechanism to work separately from the normal flow of the image processing code. Thus, the code is cleaner and more compact as shown in this example:

```
ColorTwist = iplSetColorTwist(data, scalingValue);
if(iplGetErrStatus()<0)  // check for errors
```

The error handling system is hidden within the function `iplSetColorTwist()`. As a result, this statement is uncluttered by error handling code and closely resembles a mathematical formula.

Your application should assume that every library function call may result in some error condition. The Image Processing Library performs extensive error checks (for example, `NULL` pointers, out-of-range parameters, corrupted states) for every library function.

Error macros are provided to simplify the coding for error checking and reporting. You can modify the way your application handles errors by calling `iplRedirectError()` with a pointer to your own error handling function. For more information, see "Adding Your Own Error Handler" later in this chapter. For even more flexibility, you can replace the whole error handling facility with your own code. The source code of the default error handling facility is provided.

The Image Processing Library does not process numerical exceptions (for example, overflow, underflow, and division by zero). The underlying floating point library or processor has the responsibility for catching and

reporting these exceptions. A floating-point library is needed if a processor that handles floating-point is not present. You can attach an exception handler using an underlying floating-point library for your application, if your system supports such a library.

Error-handling Functions

The following sections describe the error functions in the Image Processing Library.

Error

Performs basic error handling.

```
void iplError(IPLStatus status, const char *func,
   const char *context);
```

status	Code that indicates the type of error (see Table 3-1, "iplError() Status Codes".)
func	Name of the function where the error occurred.
context	Additional information about the context in which the error occurred. If the value of *context* is `NULL` or empty, this string will not appear in the error message.

Discussion

The `iplError()` function must be called whenever any of the library functions encounters an error. The actual error reporting is handled differently, depending on whether the program is running in Windows mode or in console mode. Within each invocation mode, you can set the error mode flag to alter the behavior of the `iplError()` function. For more information on the defined error modes, see "SetErrMode" section.

To simplify the coding for error checking and reporting, the error handling system of the Image Processing Library supports a set of error macros. See "Error Macros" for a detailed description of the error handling macros.

The `iplError()` function calls the default error reporting function. You can change the default error reporting function by calling `iplRedirectError()`. For more information, see the description of `iplRedirectError`.

GetErrStatus SetErrStatus

Gets and sets the error codes that describe the type of error being reported.

```
typedef int IPLStatus;
IPLStatus iplGetErrStatus();
void iplSetErrStatus(IPLStatus status);
```

status	Code that indicates the type of error (see Table 3-1, "iplError() Status Codes").

Discussion

The `iplGetErrStatus()` and `iplSetErrStatus()` functions get and set the error status codes that describe the type of error being reported. See "Status Codes" for descriptions of each of the error status codes.

GetErrMode
SetErrMode

Gets and sets the error modes that describe how an error is processed.

```
#define IPL_ErrModeLeaf    0
#define IPL_ErrModeParent  1
#define IPL_ErrModeSilent  2
int iplGetErrMode();
void iplSetErrMode(int errMode);
```

errMode	Indicates how errors will be processed. The possible values for *errMode* are `IPL_ErrModeLeaf`, `IPL_ErrModeParent`, or `IPL_ErrModeSilent`.

Discussion

NOTE. *This section describes how the default error handler handles errors for applications which run in console mode. If your application has a custom error handler, errors will be processed differently than described below*

The `iplSetErrMode()` function sets the error modes that describe how errors are processed. The defined error modes are `IPL_ErrModeLeaf`, `IPL_ErrModeParent`, and `IPL_ErrModeSilent`.

If you specify `IPL_ErrModeLeaf`, errors are processed in the "leaves" of the function call tree. The `iplError()` function (in console mode) prints an error message describing *status*, *func*, and *context*. It then terminates the program.

If you specify `IPL_ErrModeParent`, errors are processed in the “parents” of the function call tree. When `iplError()` is called as the result of detecting an error, an error message will print, but the program will not terminate. Each time a function calls another function, it must check to see if an error has occurred. When an error occurs, the function should call `iplError()` specifying `IPL_StsBackTrace`, and then return. The macro `IPL_ERRCHK()` may be used to perform both the error check and back-trace call. This passes the error “up” the function call tree until eventually some parent function (possibly `main()`) detects the error and terminates the program.

`IPL_ErrModeSilent` is similar to `IPL_ErrModeParent`, except that error messages are not printed.

`IPL_ErrModeLeaf` is the default, and is the simplest method of processing errors. `IPL_ErrModeParent` requires more programming effort, but provides more detailed information about where and why an error occurred. All of the functions in the library support both options (that is, they use `IPL_ERRCHK()` after function calls). If an application uses the `IPL_ErrModeParent` option, it is essential that it checks for errors after all library functions that it calls.

The status code of the last detected error is stored into the variable `IplLastStatus` and can be returned by calling `iplGetErrStatus()`. The value of this variable may be used by the application during the back-trace process to determine what type of error initiated the back trace.

ErrorStr

Translates an error or status code into a textual description.

```
const char* iplErrorStr(IPLStatus status);
```

status	Code that indicates the type of error (see Table 3-1, “iplError() Status Codes”).

Discussion

The function `iplErrorStr()` returns a short string describing *`status`*. Use this function to produce error messages for users. The returned pointer is a pointer to an internal static buffer that may be overwritten on the next call to `iplErrorStr()`.

RedirectError

Assigns a new error handler to call when an error occurs.

```
IPLErrorCallBack iplRedirectError(IPLErrorCallBack func);
```

`func`	Pointer to the function that will be called when an error occurs.

Discussion

The `iplRedirectError()` function assigns a new function to be called when an error occurs in the Image Processing Library. If *`func`* is `NULL`, `iplRedirectError()` installs the library's default error handler.

The return value of `iplRedirectError()` is a pointer to the previously assigned error handling function.

For the definition of the function typedef `IPLErrorCallBack`, and for more information on the `iplRedirectError()` function, see "Adding Your Own Error Handler" below.

NullDevReport
StdErrReport
GuiBoxReport

Predefined error-handling functions that send error messages to different output destinations.

```
IPLStatus iplNulDevReport ( IPLStatus status,
    const char *funcname, const char *context,
    const char *file, int line);
```

```
IPLStatus iplStdErrReport ( IPLStatus status,
     const char *funcname, const char *context,
     const char *file, int line);
```

```
IPLStatus iplGuiBoxReport ( IPLStatus status,
     const char *funcname, const char *context,
     const char *file, int line);
```

`status`	Code that indicates the type of error (see Table 3-1, "iplError() Status Codes".)
`funcname`	Name of the function where the error occurred.
`context`	Additional information about the context in which the error occurred. If the value of `context` is `NULL` or empty, this string will not appear in the error message.
`file`	Name of the source file in which the error occured.
`line`	Line number in the source file where the error occurred.

Discussion

You can use these predefined functions as error handlers to redirect error reporting in your application to a different output destination.

The `iplNulDevReport()` function directs error reporting to the NULL device, that is, outputs no error messages.
The `iplStdErrReport()` function is used in programs running in the console mode, it outputs error messages to the console.
For applications running in Windows mode use `iplGuiBoxReport()` function that outputs error messages to the message box.
The default for dynamic libraries is `iplGuiBoxReport()`.

To change the error output stream call `iplRedirectError()` using the pointer to one of the predefined error handling functions as the argument. If you need to define your own error handler, see Adding Your Own Error Handler below.

Error Macros

The error macros associated with the `iplError()` function are described below.

```
#define IPL_ERROR(status, func, context) \
     iplError((status),(func),(context));

#define IPL_ERRCHK(func, context)\
     ( (iplGetErrStatus()>=0) ? IPL_StsOk \
            : IPL_ERROR(IPL_StsBackTrace,(func),(context)) )

#define IPL_ASSERT(expr, func, context)\
     ( ( expr) ? IPL_StsOk\
            : IPL_ERROR(IPL_StsInternal,(func),(context)) )

#define IPL_RSTERR()       (iplSetErrStatus(IPL_StsOk))
```

context	Provides additional information about the context in which the error has occurred. If the value of *context* is NULL or empty, this string does not appear in the error message.
expr	An expression that checks for an error condition and returns FALSE if an error has occurred.
func	Name of the function where the error occurred.
status	Code that indicates the type of error (see Table 3-1, "iplError() Status Codes.")

Discussion

The `IPL_ASSERT()` macro checks for the error condition *expr* and sets the error status `IPL_StsInternal` if the error occurred.

The `IPL_ERRCHK()` macro checks to see if an error has occurred by checking the error status. If an error has occurred, `IPL_ERRCHK()` creates an error back trace message and returns a non-zero value. This macro should normally be used after any call to a function that might have signaled an error.

The `IPL_ERROR()` macro simply calls the `iplError()` function by default. This macro is used by other error macros. By changing `IPL_ERROR()` you can modify the error reporting behavior without changing a single line of source code.

The `IPL_RSTERR()` macro resets the error status to `IPL_StsOk`, thus clearing any error condition. This macro should be used by an application when it decides to ignore an error condition.

Status Codes

Some of the status codes used by the library are listed in Table 3-1. Status codes are integers, not an enumerated type. This allows an application to extend the set of status codes beyond those used by the library itself. Negative codes indicate errors, while non-negative codes indicate success. To obtain a short string describing the status code use `iplErrorStr()` function.

Table 3-1 iplError() Status Codes

Status Code	Value	Description
`IPL_StsOk`	0	No error. The `iplError()` function does nothing if called with this status code.
`IPL_StsBackTrace`	-1	Implements a back-trace of the function calls that lead to an error. If `IPL_ERRCHK()` detects that a function call resulted in an error, it calls `IPL_ERROR()` with this status code to provide further context information for the user.
`IPL_StsError`	-2	An error of unknown origin, or of an origin not correctly described by the other error codes.
`IPL_StsInternal`	-3	An internal “consistency” error, often the result of a corrupted state structure. These errors are typically the result of a failed assertion.

continued ☞

Table 3-1 iplError() Status Codes (continued)

Status Code	Value	Description
IPL_StsNoMem	-4	A function attempted to allocate memory using malloc() or a related function and was unsuccessful. The message *context* indicates the intended use of the memory.
IPL_StsBadArg	-5	One of the arguments passed to the function is invalid. The message *context* indicates which argument and why.
IPL_StsBadFunc	-6	The function is not supported by the implementation, or the particular operation implied by the given arguments is not supported.
IPL_HeaderIsNull	-9	Null pointer to the image header.
IPL_BadImageSize	-10	Incorrect image size.
IPL_BadOffset	-11	Incorrect offset of the image's ROI.
IPL_BadDataPtr	-12	Image must be tiled or must have non-zero data pointer.
IPL_BadStep	-13	Incorrect *widthStep* of the image.
IPL_BadModelOrChSeq	-14	Incorrect color model or channel sequence of the image.
IPL_BadNumChannels	-15	Incorrect number of channels in the image.
IPL_BadNumChannel1U	-16	Number of channels for 1U depth image must be one.
IPL_BadDepth	-17	Incorrect depth value in the image header.
IPL_BadAlphaChannel	-18	Incorrect alpha channel number in the image header.
IPL_BadOrder	-19	Incorrect data order value in the image header.

continued ☞

Table 3-1 iplError() Status Codes (continued)

Status Code	Value	Description
IPL_BadOrigin	-20	Incorrect data origin value in the image header.
IPL_BadAlign	-21	Incorrect data alignment value in the image header.
IPL_BadCallBack	-22	Null pointer to callback function.
IPL_BadTileSize	-23	Incorrect size of the tile.
IPL_BadCOI	-24	Incorrect COI of the image.
IPL_BadROISize	-25	Incorrect size of ROI in the image header.

Application Notes

The variable `IplLastStatus` records the status of the last error reported. Its value is initially `IPL_StsOk`. The value of `IplLastStatus` is not explicitly set by the library function detecting an error. Instead, it is set by `iplSetErrStatus()`.

If the application decides to ignore an error, it should reset `IplLastStatus` back to `IPL_StsOk` (see `IPL_RSTERR()` under "Error Macros"). An application-supplied error-handling function must update `IplLastStatus` correctly; otherwise the Image Processing Library might fail. This is because the macro `IPL_ERRCHK()`, which is used internally to the library, refers to the value of this variable.

Error Handling Example

The following example describes the default error handling for a console application. In the example program, test.c, assume that the function libFuncB() represents a library function such as ipl?AddS(), and the function libFuncD() represents a function that is called internally to the library. In this scenario, main() and appFuncA() represent application code.

The value of the error mode is set to IPL_ErrModeParent. The IPL_ErrModeParent option produces a more detailed account of the error conditions.

Example 3-1 Error Functions

```
/* application main function */
main() {
  iplSetErrMode(IPL_ErrModeParent);
  appFuncA(5, 45, 1.0);
  if (IPL_ERRCHK("main","compute something")) exit(1);
  return 0;
}
/* application subroutine */
void appFuncA(int order1, int order2, double a) {
  libFuncB(a, order1);
  if (IPL_ERRCHK("appFuncA","compute using order1")) return;
  libFuncB(a, order2);
  if (IPL_ERRCHK("appFuncA","compute using order2")) return;
}
  /* do some more work  */
```

continued ☞

Example 3-1 Error Functions (continued)

```
/* library function */
void libFuncB(double a, int order) {
  float *vec;
  if (order > 31) {
    IPL_ERROR(IPL_StsBadArg, "libFuncB",
    "order must be less than or  equal to 31");
    return;
  }
  if ((vec = libFuncD(a, order)) == NULL) {
    IPL_ERRCHK("libFuncB", "compute using a");
    return;
  }
/* code to do some real work goes here */
  free(vec);
}             // next: library function called internally
double *libFuncD(double a, int order) {
  double *vec;
  if ((vec=(double*)malloc(order*sizeof(double))) == NULL) {
    IPL_ERROR(IPL_StsNoMem, "libFuncD",
    "allocating a vector of doubles");
    return NULL;
  }
  /* do something with vec */
return vec;
}
```

When the program is run, it produces the output illustrated in Example 3-2.

Example 3-2 Output for the Error Function Program (IPL_ErrModeParent)

```
IPL Library Error: Invalid argument in function libFuncB: order must be
less than or equal to 31
       called from function appFuncA: compute using order2
       called from function main: compute something
```

If the program runs with the `IPL_ErrModeLeaf` option instead of `IPL_ErrModeParent`, only the first line of the above output is produced before the program terminated.

If the program in Example 3-1 runs out of heap memory while using the `IPL_ErrModeParent` option, then the output illustrated in Example 3-3 is produced.

Example 3-3 Output for the Error Function Program (IPL_ErrModeParent)

```
IPL Library Error: Out of memory in function libFuncD: allocating a
vector of doubles
       called from function libFuncB: compute using a
       called from function appFuncA: compute using order1
       called from function main[]: compute something
```

Again, if the program is run with the `IPL_ErrModeLeaf` option instead of `IPL_ErrModeParent`, only the first line of the output is produced.

Adding Your Own Error Handler

The Image Processing Library allows you to define your own error handler. User-defined error handlers are useful if you want your application to send error messages to a destination other than the standard error output stream. For example, you can choose to send error messages to a dialog box if your

application is running under a Windows system or you can choose to send error messages to a special log file.

There are two methods of adding your own error handler. In the first method, you can replace the `iplError()` function or the complete error handling library with your own code. Note that this method can only be used at link time.

In the second method, you can use the `iplRedirectError()` function to replace the error handler at run time. The steps below describe how to create your own error handler and how to use the `iplRedirectError()` function to redirect error reporting.

1. Define a function with the function prototype as follows:

```
typedef int (_STDCALL *IPLErrorCallBack)

(IPLStatus status, const char *funcname,
const char *context, const char *file, int line);
```

2. Your application should then call the `iplRedirectError()` function to redirect error reporting for your own function. All subsequent calls to `iplError()` will call your own error handler.

3. To redirect the error handling back to the default handler, simply call `iplRedirectError()` with a `NULL` pointer.

Example 3-4 illustrates a user-defined error handler function, `ownError()`, which simply prints an error message constructed from its arguments and exits.

Example 3-4 A Simple Error Handler

```
IPLStatus ownError(IPLStatus status, const char *func,
 const char *context, const char *file, int line);
{
  fprintf(stderr, "IPL Library error: %s, ", iplErrorStr(status));
  fprintf(stderr, "function %s, ", func ? func : "<unknown>");
  if (line > 0) fprintf(stderr, "line %d, ", line);
  if (file != NULL) fprintf(stderr, "file %s, ", file);
  if (context) fprintf(stderr, "context %s\n", context);
  IplSetErrStatus(status);
  exit(1);
}
main () {
  extern IPLErrorCallBack ownError;
/* Redirect errors to your own error handler */
  iplRedirectError( ownError);
/* Redirect errors back to the default error handler */
  iplRedirectError(NULL);
}
```

This page is left blank for double-sided printing

This page is left blank for double-sided printing

Image Creation and Access

4

This chapter describes the functions that provide the following functionalities:

- Creating and accessing attributes of images (both tiled and non-tiled)
- Allocating memory for data of required type (see also the functions CreateConvKernel in Chapter 6 and CreateColorTwist in Chapter 9)
- Setting, copying, exchanging, and scaling image data.
- Generating and adding noise to image data.
- Working in the Windows DIB (device-independent bitmap) environment.

Table 4-1 Image Creation, Data Exchange and Windows DIB Functions

Group	Function Name	Description
Creating Images	`iplCreateImageHeader`	Creates an image header according to the specified attributes.
	`iplCloneImage`	Creates a copy of an image.
	`iplAllocateImage`	Allocates memory for image data of all supported types except 32-bit FP data.
	`iplAllocateImageFP`	Allocates memory for image data of 32-bit floating-point type.
	`iplDeallocateImage`	Frees memory for image data pointed to in the image header.
	`iplCreateROI`	Creates a region of interest (ROI) header with specified attributes.
	`iplDeallocate`	Deallocates header attributes or image data or ROI or all of the above.

continued ☞

Table 4-1 Image Creation, Data Exchange and Windows DIB Environment Functions (continued)

Group	Function Name	Description
Creating Images (cont-d)	iplSetROI	Sets a region of interest for an image.
	iplSetBorderMode	Sets the mode for handling the border pixels.
	iplCreateTileInfo	Creates the IplTileInfo structure.
	iplSetTileInfo	Sets the tiling information.
	iplDeleteTileInfo	Deletes the IplTileInfo structure.
	iplCreateImageJaehne	Creates a one-channel test image.
	iplCheckImageHeader	Validates the field values of the image header.
Memory Allocation	iplMalloc	Allocates memory aligned to 8 bytes boundary.
	iplwMalloc	Allocates memory aligned to 8 bytes boundary for 16-bit words.
	ipliMalloc	Allocates memory aligned to 8 bytes boundary 32-bit double words.
	iplsMalloc	Allocates memory aligned to 8 bytes boundary for single float elements.
	ipldMalloc	Allocates memory aligned to 8 bytes boundary for double float elements.
	iplFree	Frees memory allocated by the ipl?Malloc functions.
Data Exchange	iplSet iplSetFP	Sets a constant value for all pixels in the image.
	iplPutPixel iplGetPixel	Sets/retrieves the value of the pixel with coordinates (x, y).
	iplCopy	Copies image data from one image to another.

continued ☞

Table 4-1 Image Creation, Data Exchange and Windows DIB Environment Functions (continued)

Group	Function Name	Description
	iplExchange	Exchanges image data between two images.
	iplConvert	Converts images based on the input and output image requirements.
Data Scaling	iplScale	Scales image data from one data type to another, mapping the whole data range of the input data type to the whole range of output data type. (Floating-point data is not supported.)
	iplScaleFP	Converts 32-bit floating-point image data to and from any other data type supported by the library.
Noise Generation	iplNoiseImage	Adds noise signal to image pixel values.
	iplNoiseUniformInit, iplNoiseUniformInitFP	Initializes the structure for generating a noise signal with uniform distribution.
	iplNoiseGaussianInit, iplNoiseGaussianInitFP	Initializes the structure for generating a noise signal with Gaussian distribution.
Windows DIB	iplTranslateDIB	Translates a DIB image into an IplImage structure.
	iplConvertFromDIB	Converts a DIB image to an IplImage with specified attributes.
	iplConvertFromDIBSep	Same as above, but uses separate parameters for DIB header and data.
	iplConvertToDIB	Converts an IplImage to a DIB image with specified attributes.
	iplConvertToDIBSep	Same as above, but uses separate parameters for DIB header and data.

Image Header and Attributes

The Image Processing Library functions operate on a single format for images in memory. This format consists of a header of type `IPLImage` containing the information for all image attributes. The header also contains a pointer to the image data. (See the attributes description in Chapter 2, section "Data Architecture.") The values that these attributes can assume are listed in Table 4-2.

Table 4-2 Image Header Attributes

Description	Value	Corresponding DIB Attribute
Size of the `IplImage` header (for internal use)	`nSize` in bytes	
Image Header Revision ID (internal use)	ID number	
Number of Channels	1 to `N` (including alpha channel, if any)	1 (Gray)<br>3 (RGB)<br>4 (RGBA)
Alpha channel number	0 (if not present)<br>`N`	4 (RGBA)
Bits per channel		
Gray only<br>All images: color, gray, and multi-spectral	`IPL_DEPTH_1U` (1-bit)<br>`IPL_DEPTH_8U` (8-bit unsigned)	Supported<br>Supported (RGB, RGBA)
(The signed data is used only as output for some image output operations.)	`IPL_DEPTH_8S` (8-bit signed)<br>`IPL_DEPTH_16U` (16-bit unsign.)<br>`IPL_DEPTH_16S` (16-bit signed)<br>`IPL_DEPTH_32S` (32-bit signed)<br>`IPL_DEPTH_32F` (32-bit float)	Not supported<br>Not supported<br>Not supported<br>Not supported<br>Not supported
Color model	4 character string: "Gray", "RGB," "RGBA", "CMYK," etc.	Not supported. Implicitly, RGB color model.

continued ☞

Table 4-2 Image Header Attributes (continued**)**

**Description**	**Value**	**Corresponding DIB Attribute**
Channel sequence	4-character string. Can be “G”, “GRAY”, “BGR”, “BGRA”, “RGB”, “RGBA”, “HSV”, “HLS”, “XYZ”, “YUV”, “YCr”, “YCC”, or “LUV”.	Not supported (implicitly BGR for RGB images.)
Data Ordering	`IPL_DATA_ORDER_PIXEL`<br>`IPL_DATA_ORDER_PLANE`	Supported<br>Not supported
Origin	`IPL_ORIGIN_TL` (top left corner)<br>`IPL_ORIGIN_BL` (bottom left)	Supported<br>Supported
Scanline alignment	`IPL_ALIGN_DWORD`<br>`IPL_ALIGN_QWORD`	Supported<br>Not Supported
Image size: height<br>width	Integer<br>Integer	m<br>n
Region of interest (ROI)	Pointer to structure	Not supported
Mask	Pointer to another `IplImage`	Not supported
Image size (bytes)	Integer	
Image data pointer	Pointer to data	
Aligned width	Width (row length in bytes) of image padded for alignment	
Border mode of the top, bottom, left, and right sides of the image.	BorderMode [4]	
Border constant on the top, bottom, left, and right side of the image.	BorderConst [4]	
Original Image	Pointer to original image data	
Image ID	For application use only; ignored by the library.	
Tiling information	Pointer to `IplTileInfo` structure	

Figure 4-1 presents a graphical depiction of an RGB image with a rectangular ROI and a COI.

Figure 4-1 RGB Image with a Rectangular ROI and a COI

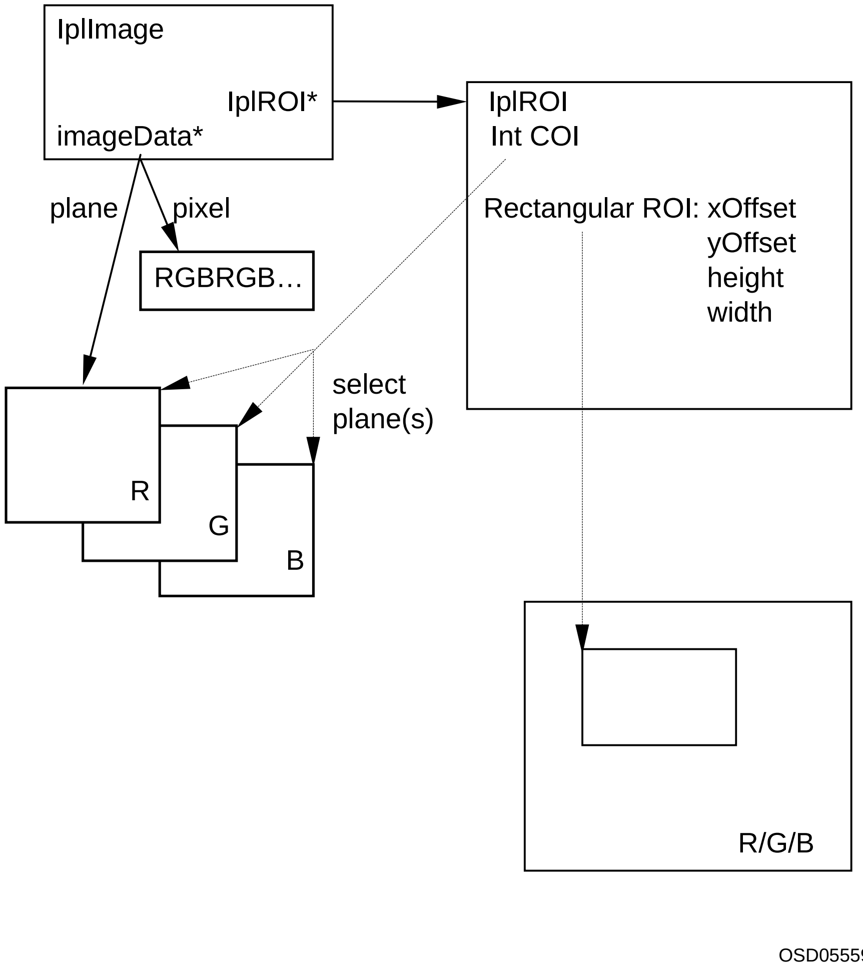

The C language definition for the `IPLImage` structure is given below.

IplImage Structure Definition

```
typedef struct _IplImage {
                                        IPL.H
  int    nSize            /* size of iplImage struct */
  int    ID                 /* image header version    */
  int    nChannels;
  int    alphaChannel;
  int    depth;           /* pixel depth in bits */
  char   colorModel[4];
  char   channelSeq[4];
  int    dataOrder;
  int    origin;
  int    align;           /* 4- or 8-byte align  */
  int    width;
  int    height;
  struct _IplROI *roi;        /* pointer to ROI if any   */
  struct _IplImage *maskROI;  /*pointer to mask ROI if any */
  void   *imageId;            /* use of the application */
  struct _IplTileInfo *tileInfo;  /* contains information
                                     on tiling */
  int   imageSize;        /* useful size in bytes */
  char  *imageData;       /* pointer to aligned image   */
  int   widthStep;        /* size of aligned line in bytes */
  int   BorderMode[4];    /* the top, bottom, left,
                            and right border mode */
  int   BorderConst[4];   /* constants for the top, bottom,
                             left, and right border     */
  char  *imageDataOrigin; /* ptr to full, nonaligned image */
} IplImage;
```

Tiling Fields in the IplImage Structure

Image tiling in the Image Processing Library was described in Chapter 2. The following fields from the `IplImage` structure are used in tiled images:

```
struct IplImage {
   ...
   void* imageId;
   IplTileInfo *tileInfo;
   ...
}
```

The `imageId` field can be used by the application, and is ignored by the library. The `tileInfo` field contains information on tiling. It is described in the next section.

The library expects either the `tileInfo` pointer or the `imageData` pointer to be `NULL`. If the former is `NULL`, the image is not tiled; if the latter is `NULL`, the image is tiled. It is an error condition if both or neither of the two are `NULL`.

IplTileInfo Structure

This structure provides information for image tiling:

```
typedef struct _IplTileInfo
 {
   IplCallBack callBack;
   void *id;
   char* tileData
   int  width, height;
 } IplTileInfo;
```

Here `callBack` is the call-back function (see “Call-backs” in Chapter 2); `id` is an additional identification field; `width` and `height` are the tile sizes for the image; and `tileData` is the field which the call-back function should point to the requested tile.

Creating Images

There are several ways of creating a new image:

- Construct an `IplImage` header by setting the attributes to appropriate values, then call the function `iplAllocateImage()` to allocate memory for the image or set the image data pointer to image data (in a compatible format) that already exists.
- Call `iplCreateImageHeader()` to create an `IplImage` header, then call the function `iplAllocateImage()` to allocate memory for the image or set the image data pointer to existing image data.
- Convert a DIB image to an `IplImage` using the functions `iplTranslateDIB()` or `iplConvertFromDIB()`. See the section "Working in the Windows DIB Environment."
- Create a copy of existing image by calling `iplCloneImage()`.

CreateImageHeader

Creates an `IplImage` *header according to the specified attributes.*

```
IplImage* iplCreateImageHeader(int nChannels,
   int alphaChannel, int depth, char* colorModel,
   char* channelSeq, int dataOrder, int origin, int align,
   int width, int height, IplROI* roi, IplImage* maskROI,
   void* imageID, IplTileInfo* tileInfo);
```

nChannels	Number of channels in the image.
alphaChannel	Alpha channel number (0 if there is no alpha channel in the image).
depth	Bit depth of pixels. Can be one of `IPL_DEPTH_1U`, `IPL_DEPTH_8U`, `IPL_DEPTH_8S`, `IPL_DEPTH_16U`, `IPL_DEPTH_16S`, `IPL_DEPTH_32S`, or `IPL_DEPTH_32F`. See Table 4-2.

`colorModel`	A four-character string describing the color model: “RGB”, “GRAY”, “HLS” etc.
`channelSeq`	The sequence of color channels; can be one of the following: “G”, “GRAY”, “BGR”, “BGRA”, “RGB”, “RGBA”, “HSV”, “HLS”, “XYZ”, “YUV”, “YCr”, “YCC”, “LUV”. The library uses this information only for image type conversions of known image channel formats.
`dataOrder`	`IPL_DATA_ORDER_PIXEL` or `IPL_DATA_ORDER_PLANE`.
`origin`	The origin of the image. Can be `IPL_ORIGIN_TL` or `IPL_ORIGIN_BL`.
`align`	Alignment of image data. Can be `IPL_ALIGN_DWORD` or `IPL_ALIGN_QWORD`.
`width`	Width of the image in pixels.
`height`	Height of the image in pixels.
`roi`	Pointer to an ROI (region of interest) structure. This argument can be `NULL`, which implies that a region of interest comprises all channels and the entire image area.
`maskROI`	Pointer to the header of another image that specifies the mask ROI. This argument can be `NULL`, which indicates that no mask ROI is used. A pixel is processed if the corresponding mask pixel is 1, and is not processed if the mask pixel is 0. The `maskROI` field of the mask image’s header is ignored.
`imageID`	The image ID (field reserved for the use of the application to identify the image).
`tileInfo`	The pointer to the `IplTileInfo` structure containing information used for image tiling.

Discussion

The function `iplCreateImageHeader()` creates an `IplImage` header according to the specified attributes; see Example 4.1. The image data pointer is set to `NULL`; no memory for image data is allocated.

Example 4-1 Creating and Deleting an Image Header

```
int example41( void ) {
   IplImage *imgh = iplCreateImageHeader(
      3,                        // number of channels
      0,                        // no alpha channel
      IPL_DEPTH_8U,             // data of byte type
      "RGB",                    // color model
      "BGR",                    // color order
      IPL_DATA_ORDER_PIXEL,     // channel arrangement
      IPL_ORIGIN_TL,            // top left orientation
      IPL_ALIGN_QWORD,          // 8 bytes align
      150,                      // image width
      100,                      // image height
      NULL,                     // no ROI
      NULL,                     // no mask ROI
      NULL,                     // no image ID
      NULL);                    // not tiled
   if( NULL == imgh ) return 0;
   iplDeallocate( imgh, IPL_IMAGE_HEADER );
   return IPL_StsOk == iplGetErrStatus();
}
```

The function `iplCreateImageHeader()` sets the image size attribute in the header to zero. To allocate memory for image data, call the function `iplAllocateImage()`.

The mask region of interest specified by the *maskROI* pointer is discussed in the section Image Regions of Interest (Chapter 2). The *intersection* of aligned rectangular ROI(s) and maskROI(s) for *all* source images and the destination image forms the actual region to be processed.

For geometric transformation functions, such as `Zoom()` or `Mirror()`, the shape and orientation of rectangular ROIs and mask ROIs of the source image changes according to the function. In these cases, the functions write the results of image processing to the intersection of the destination ROI and the *transformed* source ROI.

For more information about geometric transformation, see Chapter 11.

Return Value

The newly constructed `IplImage` header.

AllocateImage, AllocateImageFP

Allocates memory for image data according to the specified header.

```
void iplAllocateImage(IplImage* image, int doFill,
    int fillValue);
void iplAllocateImageFP(IplImage* image, int doFill,
    float fillValue);
```

image	An image header with a NULL image data pointer. The pointer will be set to newly allocated image data memory after calling this function.
doFill	A flag: if zero, indicates that the pixel data should not be initialized by *fillValue*.
fillValue	The initial value for pixel data.

Discussion

These functions are used to allocate image data on the basis of a specified image header. The header must be properly constructed before calling this function. Note that IPL_DEPTH_32F is the only admissible depth for IplImage passed into iplAllocateImageFP(); this depth must not be used for iplAllocateImage().

Memory is allocated for the image data according to the attributes specified in the image header; see Example 4-2. The image data pointer will then point to the allocated memory. It is highly preferable, for efficiency considerations, that the scanline alignment attribute (argument *align*) in the image header be set to IPL_ALIGN_QWORD. This will force the image data to be aligned on a quadword (64-bit) memory boundary.

The functions set the image size attribute in the header to the number of bytes allocated for the image.

Example 4-2 Allocating and Deallocating the Image Data

```
int example42( void ) {

   IplImage img;
   char colorModel[4] = "RGB";
   char channelSeq[4] = "BGR";

   img.nSize = sizeof( IplImage );
   img.nChannels = 3;            // number of channels
   img.alphaChannel = 0;         // no alpha channel
   img.depth = IPL_DEPTH_16U;    // data of ushort type
   img.dataOrder = IPL_DATA_ORDER_PIXEL;
   img.origin = IPL_ORIGIN_TL;            // top left
   img.align = IPL_ALIGN_QWORD;           // align
   img.width = 100;
   img.height = 100;
   img.roi = NULL;                        // no ROI
   img.maskROI = NULL;                    // no mask ROI
   img.tileInfo = NULL;                   // not tiled

   // The following fields will be set by the function

   img.widthStep = 0;
   img.imageSize = 0;
   img.imageData = NULL;
   img.imageDataOrigin = NULL;

   *((int*)img.colorModel) =* *((int*)colorModel);
   *((int*)img.channelSeq) =* *((int*)channelSeq);

   iplAllocateImage( &img, 0, 0 ); // allocate image data
   if( NULL == img.imageData ) return 0;  // check result

   iplDeallocate( &img, IPL_IMAGE_DATA );
                         // deallocate image data only
   return Ipl_StsOk == iplGetErrStatus();
}
```

DeallocateImage

Deallocates (frees) memory for image data pointed to in the image header.

```
void iplDeallocateImage(IplImage* image)
```

image	An image header with a pointer to the allocated image data memory. The image data pointer will be set to `NULL` after this function executes.

Discussion

The function `iplDeallocateImage()` is used to free image data memory pointed to by the *imageData* member of the image header. The respective pointer to image data or ROI data is set to `NULL` after the memory is freed up.

CloneImage

Creates a copy of an image.

```
IplImage* iplCloneImage (const IplImage* image);
```

image	Header of the image to be cloned.

Discussion

The function creates a copy of *image*, including its data and ROI. The *imageID*, *maskROI*, and *tileInfo* fields of the copy are set to `NULL`.

Return Value

A pointer to the created copy of *image*. If the source image is tiled, the function creates a non-tiled image and does not copy the image data.

Deallocate

Deallocates or frees memory for image header or data or mask ROI or rectangular ROI or all four.

```
void iplDeallocate (IplImage* image, int flag)
```

image	An image header with a pointer to allocated image data memory. The image data pointer will be set to `NULL` after this function executes.
flag	Flag indicating what memory area to free:
`IPL_IMAGE_HEADER`	Free header structure.
`IPL_IMAGE_IMAGE`	Free image data, set pointer to `NULL`.
`IPL_IMAGE_ROI`	Free image ROI, set pointer to `NULL`.
`IPL_IMAGE_MASK`	Free mask image data, set pointer to `NULL`.
`IPL_IMAGE_ALL`	Free header, image data, mask ROI and rectangular ROI.
`IPL_IMAGE_ALL_WITHOUT_MASK`	Free header, image data, and rectangular ROI.

Discussion

The function `iplDeallocate()` is used to free memory allocated for header structure, image data, ROI data, mask image data, or all four. The respective pointer is set to `NULL` after the memory is freed up.

CheckImageHeader

Validates field values of an existing image header structure.

```
IPLStatus iplCheckImageHeader ( const IplImage* hdr )
```

hdr	Pointer to an image header structure

Discussion

The function `iplCheckImageHeader()` checks whether the `IplImage` header structure of an image has valid field values, and returns the corresponding status code. This function works on the assumption that the referenced image contains non-empty data. Many image processing functions in Image Processing Library call `iplCheckImageHeader()` to verify that the image information is correct. You can also use this function in your application to check that some imported image data, not created by Image Processing Library functions but referenced in the `IplImage` header, has the valid header structure.
The following main status codes can be returned by the `iplCheckImageHeader()` function (see Image Header and Attributes for the explanation of image header fields):

`IPL_StsOK`	Indicates no errors in image header structure.
`IPL_HeaderIsNull`	Indicates an error condition if the *hdr* pointer to the image header is NULL.
`IPL_BadDataPtr`	Indicates an error condition if a non-tiled image has NULL `imageData` pointer.
`IPL_BadImageSize`	Indicates an error condition if a non-tiled image has negative or zero `imageSize`.
`IPL_BadStep`	Indicates an error condition if a non-tiled image has negative or zero `widthStep`.

`IPL_BadCallBack`	Indicates an error condition if the image is tiled but the call-back function is not set in the `_IplTileInfo` structure.
`IPL_BadTileSize`	Indicates an error condition if a tiled image has tile sizes not multiple of 8.
`IPL_BadCOI`	Indicates an error condition if an image with ROI has incorrect `coi` field value in the `_IplROI` structure (that is, `coi` is negative or greater than `nChannels`).
`IPL_BadROISize`	Indicates an error condition if an image with ROI has negative or zero ROI size value.
`IPL_BadOffset`	Indicates an error condition if an image with ROI has negative ROI offset value.

CreateImageJaehne

Creates a one-channel test image.

```
IplImage* iplCreateImageJaehne ( int depth, int width,
   int height )
```

depth	Bit depth of the image to be created.
width, height	Size of the image to be created.

Discussion

The function `iplCreateImageJaehne()` creates a specific one-channel test image that has the user-defined bit depth and size. This function returns the pointer to the corresponding `IplImage` structure. The *depth* parameter can specify any data type that is used in the Image Processing library.
For the 32f floating point data type the pixel values in the created image can vary in the range between 0 (inclusive) and 1 (exclusive).

Figure 4-2 illustrates an example of the test image generated by the `iplCreateImageJaehne()` function. These test images can be effectively used when you need to visualize and interpret the results of applying filtering functions, similarly to what is proposed in [Jaehne].

Figure 4-2 Example of a Generated Test Image

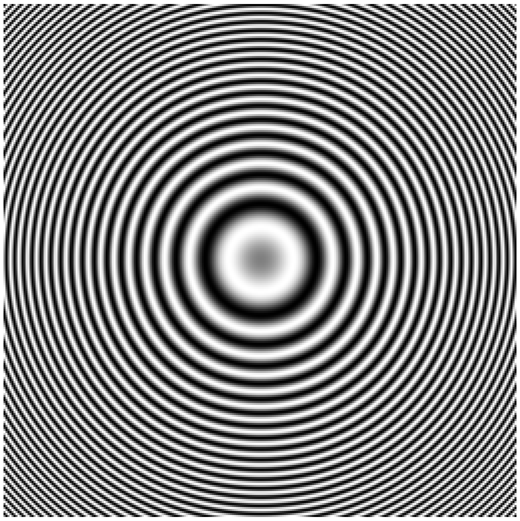

Setting Regions of Interest

To set a region of interest, the function `iplSetROI()` uses a ROI structure `IplROI` presented below. The `IplROI` member of the image header must point to this `IplROI` structure to be effective. This can be done by a simple assignment. The application may choose to construct the ROI structure explicitly without the use of the function.

IplROI Structure Definition

```
typedef struct _IplROI {
  unsigned int coi;

  int xOffset;
  int yOffset;
  int width;
  int height;
 } IplROI;
```

The members in the `IplROI` structure define:

coi	The channel of interest number. This parameter indicates which channel in the original image will be affected by processing taking place in the region of interest; *coi* equal to 0 indicates that all channels will be affected.
xOffset and *yOffset*	The offset from the origin of the rectangular ROI. (See section "Image Regions" in Chapter 2 for the description of image regions.)
width and *height*	The size of the rectangular ROI.

CreateROI

Allocates and sets the region of interest (ROI) structure.

```
IplROI* iplCreateROI(int coi, int xOffset, int yOffset,
    int width, int height);
```

`coi`	The channel of interest. It can be set to 0 (for all channels) or to a specific channel number.
`xOffset, yOffset`	The offsets from the origin of the rectangular region.
`width, height`	The size of the rectangular region.

Discussion

The function `iplCreateROI()` allocates a new ROI structure with the specified attributes and returns a pointer to this structure. You can delete this structure by calling `iplDeleteROI()`.

Return Value

A pointer to the newly constructed ROI structure or `NULL`.

DeleteROI

Allocates and sets the region of interest (ROI) structure.

```
void iplDeleteROI(IplROI* roi);
```

`roi`	The ROI structure to be deleted.

Discussion

The function `iplDeleteROI()` deallocates a ROI structure previously created by `iplCreateROI()`.

SetROI

Sets the region of interest (ROI) structure.

```
void iplSetROI(IplROI* roi, int coi, int xOffset,
    int yOffset, int width, int height);
```

roi	The pointer to the ROI structure to modify in the original image.
coi	The channel of interest in the original image. It can be set to 0 (for all channels) or to a specific channel number.
xOffset, yOffset	The offset from the origin of the rectangular region.
width, height	The size of the rectangular region.

Discussion

The function `iplSetROI()` sets the channel of interest and the rectangular region of interest in the structure *roi*.

The argument *coi* defines the number of the channel of interest. The arguments *xOffset* and *yOffset* define the offset from the origin of the rectangular ROI. The members *height* and *width* define the size of the rectangular ROI.

Image Borders and Image Tiling

Many neighborhood operators need intensity values for pixels that lie outside the image, that is, outside the borders of the image. For example, a 3 by 3 filter, when operating on the first row of an image, needs to assume pixel values of the preceding (non-existent) row. A larger filter will require more rows from the border. These border issues therefore exist at the top and bottom, left and right sides, and the four corners of the image. The library provides a function `iplSetBorderMode` that the application can use to set the border mode within the image. This function specifies the behavior for handling border pixels.

For tiled images, the border mode is handled in the same way as for non-tiled images. (Outer tiles might contain extra data if the image size is not an integer multiple of the tile size, but these values are ignored and the border mode is used instead.)

SetBorderMode

Sets the mode for handling the border pixels.

```
void iplSetBorderMode(IplImage *src, int mode,
    int border, int constVal)
```

src	The image for which the border mode is to be set.
mode	The following modes are supported:

IPL_BORDER_CONSTANT	The value `constVal` is used for all pixels.
IPL_BORDER_REPLICATE	The last row or column is replicated for the border.
IPL_BORDER_REFLECT	The last rows or columns are reflected in reverse order, as necessary to create the border.

`IPL_BORDER_WRAP`	The required border rows or columns are taken from the opposite side of the image.

`border` The side that this function is called for. Can be an OR of one or more of the following four sides of an image:

`IPL_SIDE_TOP`	Top side.
`IPL_SIDE_BOTTTOM`	Bottom side.
`IPL_SIDE_LEFT`	Left side.
`IPL_SIDE_RIGHT`	Right side.
`IPL_SIDE_ALL`	All sides.

The top side is also used to define all border pixels in the top left and right corners. Similarly, the bottom side is used to define the border pixels in the bottom left and right corners.

`constVal` The value to use for the border when the mode is set to `IPL_BORDER_CONSTANT`.

Discussion

The function `iplSetBorderMode()` is used to set the border handling mode of one or more of the four sides of an image (see Example 4-3). Intensity values for the border pixels are assumed or created based on the mode.

Example 4-3 Setting the Border Mode for an Image

```
int example43( void ) {
 IplImage *imgh = iplCreateImageHeader( 3,0,IPL_DEPTH_8U,
   "RGB", "BGR", IPL_DATA_ORDER_PIXEL, IPL_ORIGIN_TL,
   IPL_ALIGN_QWORD, 100, 150, NULL, NULL, NULL, NULL);
 if( NULL == imgh ) return 0;
 iplSetBorderMode( imgh, IPL_BORDER_REPLICATE, IPL_SIDE_TOP|
   IPL_SIDE_BOTTOM | IPL_SIDE_LEFT | IPL_SIDE_RIGHT, 0 );
 iplDeallocate( imgh, IPL_IMAGE_HEADER );
 return Ipl_StsOk == iplGetErrStatus();
}
```

CreateTileInfo

Creates the IplTileInfo structure.

```
IplTileInfo* iplCreateTileInfo(IplCallBack callBack,
    void* id, int width, int height);
```

callBack	The call-back function.
id	The image ID (for application use).
width, height	The tile sizes.

Discussion

The function `iplCreateTileInfo()` allocates a new `IplTileInfo` structure with the specified attributes and returns a pointer to this structure. To delete this structure, call `iplDeleteTileInfo()`.

Return Value

The pointer to the created `IplTileInfo` structure or `NULL`.

SetTileInfo

Sets the IplTileInfo structure fields.

```
void iplSetTileInfo(IplTileInfo* tileInfo,
    IplCallBack callBack, void* id, int width, int height);
```

tileInfo	The pointer to the `IplTileInfo` structure.
callBack	The call-back function.
id	The image ID (for application use).
width, height	The tile sizes.

Discussion

This function sets attributes for an existing `IplTileInfo` structure.

DeleteTileInfo

Deletes the IplTileInfo structure.

```
void iplDeleteTileInfo(IplTileInfo* tileInfo);
```

tileInfo	The pointer to the `IplTileInfo` structure.

Discussion

This function deletes the `IplTileInfo` structure previously created by the CreateTileInfo function.

Memory Allocation Functions

Functions of the `ipl?Malloc()` group allocate aligned memory blocks for the image data. The size of allocated memory is specified by the *`size`* parameter. The “`?`” in `ipl?Malloc()` stands for `w`, `i`, `s`, or `d`; these letters indicate the data type in the function names as follows:

`iplMalloc()`	byte
`iplwMalloc()`	16-bit word
`ipliMalloc()`	32-bit double word
`iplsMalloc()`	4-byte single floating-point element
`ipldMalloc()`	8-byte double floating-point element

NOTE. *The only function to free the memory allocated by any of these functions is* `iplFree()`.

Malloc

Allocates memory aligned to an 8-byte boundary.

```
void* iplMalloc(int size);
```

`size`	Size (in bytes) of memory block to allocate.

Discussion

The `iplMalloc()` function allocates memory block aligned to an 8-byte boundary. To free this memory, use `iplFree()`.

Return Value

The function returns a pointer to an aligned memory block. If no memory is available in the system, then the `NULL` value is returned.

wMalloc

Allocates memory aligned to an 8-byte boundary for 16-bit words.

```
short* iplwMalloc(int size);
```

size	Size in words (16 bits) of memory block to allocate.

Discussion

The `iplwMalloc()` function allocates memory block aligned to an 8-byte boundary for 16-bit words. To free this memory, use `iplFree()`.

Return Value

The function returns a pointer to an aligned memory block. If no memory is available in the system, then the `NULL` value is returned.

iMalloc

Allocates memory aligned to an 8-byte boundary for 32-bit double words.

```
int* ipliMalloc(int size);
```

size	Size in double words (32 bits) of memory block to allocate.

Discussion

The `ipliMalloc()` function allocates memory block aligned to an 8-byte boundary for 32-bit double words. To free this memory, use `iplFree()`.

Return Value

The function returns a pointer to an aligned memory block. If no memory is available in the system, then the `NULL` value is returned.

sMalloc

Allocates memory aligned to an 8-byte boundary for floating-point elements.

```
float * iplsMalloc(int size);
```

size	Size in float elements (4 bytes) of memory block to allocate.

Discussion

The `iplsMalloc()` function allocates memory block aligned to an 8-byte boundary for floating-point elements. To free this memory, use `iplFree()`.

Return Value

The function returns a pointer to an aligned memory block. If no memory is available in the system, then the `NULL` value is returned.

dMalloc

Allocates memory aligned to an 8-byte boundary for double floating-point elements.

```
double* ipldMalloc(int size);
```

`size`	Size in double elements (8 bytes) of memory block to allocate.

Discussion

The `ipldMalloc()` function allocates memory block aligned to an 8-byte boundary for double floating-point elements. To free this memory, use `iplFree()`.

Return Value

The function returns a pointer to an aligned memory block. If no memory is available in the system, then the `NULL` value is returned.

iplFree

Frees memory allocated by one of the `ipl?Malloc` *functions.*

```
void  iplFree(void * ptr);
```

`ptr`	Pointer to memory block to free.

Discussion

The iplFree() function frees the aligned memory block allocated by one of the functions iplMalloc(), iplwMalloc(), ipliMalloc(), iplsMalloc(), or ipldMalloc().

NOTE. *The function* iplFree() *cannot be used to free memory allocated by standard functions like* malloc() or calloc().

Image Data Exchange

The functions described in this section provide image manipulation capabilities, such as setting the image pixel data, copying data from one image to another, exchanging the data between the images, and converting one image to another according to the attributes defined in the source and resultant IplImage headers.

Set, SetFP

Sets a value for an image's pixel data.

```
void iplSet(IplImage* image, int fillValue);
void iplSetFP(IplImage* image, float fillValue);
```

image	An image header with allocated image data.
fillValue	The value to set the pixel data.

Discussion

The functions iplSet() and iplSetFP() set the image pixel data. Before calling the functions, you must properly construct the image header and allocate memory for image data; see Example 4-4. For images with the bit

depth lower than the `fillVallue`, the `fillValue` is saturated when assigned to pixel. If an ROI is specified, only that ROI is filled.

Example 4-4 Allocating an Image and Setting Its Pixel Values

```
int example44( void ) { IplImage *img;
  __try {
     img = iplCreateImageHeader( 1,0,IPL_DEPTH_8U,"GRAY",
       "GRAY", IPL_DATA_ORDER_PIXEL, IPL_ORIGIN_TL,
       IPL_ALIGN_QWORD, 100,150, NULL, NULL, NULL, NULL);
     if( NULL == img ) return 0;
     iplAllocateImage( img, 0, 0 );
     if( NULL == img->imageData ) return 0;
     iplSet( img, 255 );
  }
  __finally {
    iplDeallocate(img, IPL_IMAGE_HEADER|IPL_IMAGE_DATA);
  }
  return IPL_StsOk == iplGetErrStatus();
}
```

Copy

Copies image data from one image to another.

```
void iplCopy(IplImage* srcImage, IplImage* dstImage);
```

srcImage	The source image.
dstImage	The resultant image.

Discussion

The function `iplCopy()` copies image data from a source image to a resultant image. Before calling this function, the source and resultant

headers must be properly constructed and image data for both images must be allocated; see Example 4-5. The following constraints apply to the copying:

- The bit depth per channel of the source image should be equal to that of the resultant image.
- The number of channels of interest in the source image should be equal to the number of channels of interest in the resultant image; that is, either the source `coi` = the resultant `coi` = 0 or both cois are nonzero.
- The data ordering (by pixel or by plane) of the source image should be the same as that of the resultant image.

The `align`, `height`, and `width` field values (see Table 4-2) may differ in source and resultant images. Copying applies to the areas that intersect between the source ROI and the destination ROI.

Example 4-5 Copying Image Pixel Values

```
int example45( void ) {
   IplImage *imga, *imgb;
   __try {
      imga = iplCreateImageHeader( 1, 0, IPL_DEPTH_8U,
         "GRAY", "GRAY", IPL_DATA_ORDER_PIXEL,
         IPL_ORIGIN_TL, IPL_ALIGN_QWORD, 100, 150,
         NULL, NULL, NULL, NULL);
      if( NULL == imga ) return 0;
      imgb = iplCreateImageHeader(
         1, 0, IPL_DEPTH_8U, "GRAY", "GRAY",
         IPL_DATA_ORDER_PIXEL, IPL_ORIGIN_TL,
         IPL_ALIGN_QWORD, 100, 150, NULL, NULL,
         NULL, NULL);
      if( NULL == imgb ) return 0;

      iplAllocateImage( imga, 1, 255 );
      if( NULL == imga->imageData ) return 0;

      iplAllocateImage( imgb, 0, 0 );
      if( NULL == imgb->imageData ) return 0;
      // Copy pixel values of imga to imgb
      iplCopy( imga, imgb );
      // Check if an error occurred
      if( iplGetErrStatus() != IPL_StsOk ) return 0;
   }
   __finally {
     iplDeallocate(imga,IPL_IMAGE_HEADER|IPL_IMAGE_DATA);
     iplDeallocate(imgb,IPL_IMAGE_HEADER|IPL_IMAGE_DATA);
   }
   return IPL_StsOk == iplGetErrStatus();
}
```

Exchange

Exchanges image data between two images.

```
void iplExchange(IplImage* ImageA, IplImage* ImageB);
```

`ImageA`	The first image.
`ImageB`	The second image.

Discussion

The function `iplExchange()` exchanges image data between two images, the first and the second. The image headers must be properly constructed before calling this function, and image data for both images must be allocated. The following constraints apply to the data exchanging:

- The bit depths per channel of both images should be equal.
- The numbers of channels of interest in both images should be equal.
- The data ordering of both images should be the same (either pixel- or plane-oriented) .

The `align`, `width`, and `height` field values (see Table 4-2) may differ in the first and the second image. The data are exchanged at the areas of intersection between the ROI of the first image and the ROI of the second image.

Convert

Converts source image data to resultant image according to the image headers.

```
void iplConvert(IplImage* srcImage, IplImage* dstImage);
```

`srcImage`	The source image.
`dstImage`	The resultant image.

Discussion

The function `iplConvert()` converts image data from the source image to the resultant image according to the attributes defined in the source and resultant `IplImage` headers; see Example 4-6.

The main conversion rule is *saturation*. The images that can be converted may have the following different characteristics:

- Bit depth per channel
- Data ordering
- Origins

(For more information about these characteristics, see Table 4-2.)

The following constraints apply to the conversion:

- If the source image has a bit depth per channel equal to 1, the resultant image should also have the bit depth equal to 1.
- The number of channels in the source image should be equal to the number of channels in the resultant image.
- The height and width of the source image should be equal to those of the resultant image.

All ROIs are ignored.

Example 4-6 Converting Images

```
int example46( void ) {
  IplImage *imga, *imgb;
  __try {
     imga = iplCreateImageHeader(
        1, 0, IPL_DEPTH_8U, "GRAY", "GRAY",
        IPL_DATA_ORDER_PIXEL, IPL_ORIGIN_TL,
        IPL_ALIGN_QWORD, 100, 150, NULL, NULL,
        NULL, NULL);
     if( NULL == imga ) return 0;

     imgb = iplCreateImageHeader(
        1, 0, IPL_DEPTH_16S, "GRAY", "GRAY",
        IPL_DATA_ORDER_PIXEL, IPL_ORIGIN_TL,
        IPL_ALIGN_QWORD, 100, 150, NULL, NULL,
        NULL, NULL);
     if( NULL == imgb ) return 0;

     iplAllocateImage( imga, 1, 128 );
     if( NULL == imga->imageData ) return 0;
     iplAllocateImage( imgb, 0, 0 );
     if( NULL == imgb->imageData ) return 0;
     // Convert unsigned char to short
     iplConvert( imga, imgb );
     // Check if an error occurred
     if( iplGetErrStatus() != IPL_StsOk ) return 0;
  }
  __finally {
    iplDeallocate(imga,IPL_IMAGE_HEADER|IPL_IMAGE_DATA);
    iplDeallocate(imgb,IPL_IMAGE_HEADER|IPL_IMAGE_DATA);
  }
  return IPL_StsOk == iplGetErrStatus();
}
```

PutPixel, GetPixel

Sets/retrieves a value of an image's pixel.

```
void iplPutPixel(IplImage* image, int x, int y,
    void* pixel);
void iplGetPixel(IplImage* image, int x, int y,
    void* pixel);
```

`image`	An image header with allocated image data.
`x, y`	The pixel coordinates.
`pixel`	The pointer to a buffer storing the consecutive channel values for the pixel.

Discussion

The function `iplPutPixel()` sets the channels in `image`'s pixel (`x`,`y`) to the values specified in the buffer `pixel`.

The function `iplGetPixel()` retrieves the values of all channels in `image`'s pixel (`x`,`y`) to the buffer `pixel`.

All channels are processed, including the alpha channel (if applicable). The channel values in the buffer are stored consecutively.

The functions work for all pixel depths supported in the library. The ROI and mask are ignored.

Example 4-7 on the next page illustrates the usage of the function `iplGetPixel()`.

Example 4-7 Using the Function iplGetPixel()

```
int example_1001( void ) {
   char pixel[4];    /// buffer to get pixel data

   /// roi to set different data in different channels
   IplROI roi = { 0, 0,0, 4,4 };
   IplImage *img = iplCreateImageHeader(
      4, 4, IPL_DEPTH_8U, "RGBA", "BGRA",
      IPL_DATA_ORDER_PIXEL, IPL_ORIGIN_TL,
      IPL_ALIGN_DWORD, 4, 4, &roi, NULL,
      NULL, NULL);

   /// alpha-channel will be 4
   iplAllocateImage( img, 1, 4 );
   roi.coi = 1;
   iplSet( img, 1 );
   roi.coi = 2;
   iplSet( img, 2 );
   roi.coi = 3;
   iplSet( img, 3 );

   iplGetPixel( img, 0,0, pixel );

   iplDeallocate( img, IPL_IMAGE_ALL & ~IPL_IMAGE_ROI );
   return IPL_StsOk == iplGetErrStatus();
}
```

Scale

Scales the image data.

```
IPLStatus iplScale (const IplImage* src, IplImage* dst);
```

src	The source image.
dst	The resultant image with data of a different type.

Discussion

The function `iplScale()` converts the data of the input image *src* to the data type of the output image *dst*.

Unlike `iplConvert()`, which *saturates* the converted data as necessary, `iplScale()` *scales* the data, using a linear mapping of the whole range of the input data type onto the range of the output data type:

$$\text{output value} = A + B * \text{input value}.$$

Here *A* and *B* are such that the minimum and maximum presentable values of the input data type (*src_type_min* and *src_type_max*) are mapped, respectively, to the minimum and maximum presentable values of the output data type (*dst_type_min* and *dst_type_max*):

$B = (dst\_type\_max - dst\_type\_min)/(src\_type\_max - src\_type\_min)$
$A = dst\_type\_min - B * src\_type\_min.$

The input and output images must have the same data ordering and coordinate origins. The data types in *src* and *dst* must be different. The supported data types for input and output images are 8-bit per channel (signed or unsigned), 16 bit per channel (signed or unsigned), or 32-bit signed. (For converting image data to and from 32-bit floating-point data type, use the function `iplScaleFP`.)

Return Value

If the execution is successful, the function returns `IPL_StsOK`; otherwise, it returns an error status code.

ScaleFP

Converts the image data to and from floating-point type by scaling.

```
IPLStatus iplScaleFP (const IplImage* src, IplImage* dst,
   float minVal, float maxVal);
```

srcImage	The source image.
dstImage	The resultant image.
minVal, maxVal	The floating-point data range (*minVal*<*maxVal*).

Discussion

The function `iplScaleFP()` converts the data of the input image *src* to the data type of the output image *dst* by scaling. One of the images must contain data of 32-bit floating-point type; the other image's bit depth can be 8-bit per channel (signed or unsigned), 16 bit per channel (signed or unsigned), or 32-bit signed.

If the *input* image data is 32-bit floating-point, the function linearly maps the user-defined floating-point data range [*minVal*..*maxVal*] onto the whole range of the output data type, [*dst_type_min*..*dst_type_max*]. If some of the input floating-point values are outside the specified input data range [*minVal*..*maxVal*], the corresponding output values will saturate. (To determine the actual floating-point data range in your image, use the function `iplMinMaxFP`.)

If the *output* image data is 32-bit floating-point, the function linearly maps the whole range of the intput data type [*src_type_min*..*src_type_max*] onto the user-defined floating-point data range [*minVal*..*maxVal*].

Return Value

If the execution is successful, the function returns `IPL_StsOK`; otherwise, it returns an error status code.

NoiseImage

Generates noise signal and adds it to an image data.

```
IPLStatus iplNoiseImage ( IplImage* image,
    const IplNoiseParam* noiseParam);
```

image	Pointer to the image header structure.
noiseParam	Pointer to the structure that contains parameters for the noise generator.

Discussion

The function `iplNoiseImage()` generates a random noise signal and adds it to a source image *image* that is passed to this function as an argument. The resulting pixel values that exceed the output data range are saturated to the respective data-range limits. The noise signal can have either uniform or Gaussian distribution. Before calling `iplNoiseImage()` you must first initialize the *noiseParam* structure using one of the initialization functions described below.
To obtain an output image which contains pure noise, call `iplNoiseImage()` using a source image with zero data as input.

Return Value

If the execution is successful, the function returns `IPL_StsOK`; otherwise, it returns an error status code.

NoiseUniformInit, NoiseUniformInitFp

Initializes parameters for generating noise signal with uniform distribution.

```
void iplNoiseUniformInit ( IplINoiseParam* noiseParam,
   unsigned int seed, int low, int high);
```

```
void iplNoiseUniformInitFp ( IplINoiseParam* noiseParam,
   unsigned int seed, float low, float high);
```

noiseParam	Pointer to the structure that contains parameters for the noise generator.
seed	The initial seed value for the pseudo-random number generator.
low, high	The lower and upper bounds for the range of uniformly distributed values.

Discussion

Use functions `iplNoiseUniformInit()`, `iplNoiseUniformInitFp()` to initialize the *noiseParam* structure if you want to generate the noise signal with uniform distribution over the range [*low*, *high*]. After that you can call the `iplNoiseImage()` function, which actually generates and adds the noise signal.

NoiseGaussianInit, NoiseGaussianInitFp

Initializes parameters for generating noise signal with Gaussian distribution.

```
void iplNoiseGaussianInit ( IplINoiseParam* noiseParam,
   unsigned int seed, int mean, int stDev);
void iplNoiseGaussianInitFp ( IplINoiseParam* noiseParam,
   unsigned int seed, float mean, float stDev);
```

noiseParam	Pointer to the structure that contains parameters for the noise generator.
seed	The initial seed value for the pseudo-random number generator.
mean	The mean of the Gaussian distribution.
stDev	The standard deviation of the Gaussian distribution.

Discussion

Use functions `iplNoiseGaussianInit(), iplNoiseGaussianInitFp()` to initialize the *noiseParam* structure if you want to generate the noise signal with Gaussian distribution that has the mean value *mean* and standard deviation *stDev*. After that you can call the `iplNoiseImage()` function, which actually generates and adds the noise signal.

Working in the Windows DIB Environment

The Image Processing Library provides functions to convert images to and from the Windows* device-independent bitmap (DIB). Table 4-2 shows that the `IplImage` format can represent more features than the DIB image format. However, the DIB palette images and 8-bit- and 16-bit-per-pixel absolute color DIB images have no equivalent in the Image Processing Library.

The DIB palette images must be first converted to the Image Processing Library's absolute color images; 8-bit- and 16-bit-per-pixel DIB images have to be unpacked into the library's 8-bit-, 16-bit- or 32-bit-per-channel images.

Any 24-bit absolute color DIB image can be directly converted to the Image Processing Library format. You just need to create an `IplImage` header corresponding to the DIB attributes. The DIB image data can be pointed to by the header or it can be duplicated.

There are the following restrictions for the DIB conversion functions:

- You can use `IplImage` structures with unsigned data only.
- The DIB and IPL images should be the same size. The following functions can perform conversion to and from the DIB format, with additional useful capabilities:

`iplTranslateDIB()` Performs a simple translation of a DIB image to an `IplImage` as described above. Also converts a DIB palette image to the Image Processing Library's absolute color image.

While this is the most efficient way of converting a DIB image, it is not the most efficient format for the library functions to manipulate because the DIB image data is doubleword-aligned, not quadword-aligned.

`iplConvertFromDIB()`, `iplConvertFromDIBSep()`	Provides more control of the conversion and can convert a DIB image to an image with a prepared `IplImage` header. The header must be set to the desired attributes. The bit depth of the channels in the `IplImage` header must be equal to or greater than that in the DIB header.
`iplConvertToDIB()`, `iplConvertToDIBSep()`	Converts an `IplImage` to a DIB image. This function performs dithering if the bit depth of the DIB is less than that of the `IplImage`. It can also be used to create a DIB palette image from an absolute color `IplImage`. The function can optionally create a new palette.

TranslateDIB

Translates a DIB image into the corresponding `IplImage`.

```
iplImage* iplTranslateDIB(BITMAPINFOHEADER* dib,
    BOOL* cloneData);
```

`dib`	The DIB image.
`cloneData`	An output flag (Boolean): if false, indicates that the image data pointer in the `IplImage` will point to the DIB image data; if true, indicates that the data was copied.

Discussion

The function `iplTranslateDIB()` translates a DIB image to the `IplImage` format; see Example 4-8. The `IplImage` attributes corresponding to the DIB image are automatically chosen (see Table 4-2), so no explicit control of the conversion is provided. A DIB palette image will be converted to an absolute color `IplImage` with a bit depth of 8 bits per channel, and the image data will be copied, returning `cloneData` = true.

A 24-bit-per-pixel RGB DIB image will be converted to an 8-bit-per-channel RGB `IplImage`.

A 32-bit-per-pixel DIB RGBA image will be converted to an 8-bit-per-channel RGBA `IplImage` with an alpha channel.

An 8-bit-per-pixel or 16-bit-per-pixel DIB absolute color RGB image will be converted (by unpacking) into an 8-bit-per-channel RGB `IplImage`. The image data will be copied, returning `cloneData` = true.

A 1-bit-per-pixel or 8-bit-per-pixel DIB gray scale image with a standard gray palette will be converted to a 1-bit-per-channel or 8-bit-per-channel gray-scale `IplImage`, respectively.

Example 4-8 Translating a DIB Image Into an IplImage

```
int example47( void ) {
#define WIDTH  8
#define HEIGHT 8
   BITMAPINFO *dib;         // pointer to bitmap
   RGBQUAD *rgb;            // pointer to bitmap colors
   unsigned char *data;     // pointer to bitmap data
   BITMAPINFOHEADER *dibh;  // header beginning
   IplImage *img = NULL;
   BOOL cloneData;          // variable to get result
   int i;
  __try {
    int size = HEIGHT * ((WIDTH+3) & ~3);
    // allocate memory for bitmap
    dib = malloc(sizeof(BITMAPINFOHEADER)
         + sizeof(RGBQUAD)*256 + size );
    if( NULL == dib ) return 0;

    // define the pointers
    dibh = (BITMAPINFOHEADER*)dib;
    rgb=(RGBQUAD*)((char*)dib + sizeof(BITMAPINFOHEADER));
    data=(unsigned char*)((char*)rgb+sizeof(RGBQUAD)*256);

    // define bitmap
    dibh->biSize = sizeof(BITMAPINFOHEADER);
    dibh->biWidth = WIDTH;
    dibh->biHeight = HEIGHT;
    dibh->biPlanes = 1;
    dibh->biBitCount = 8;
    dibh->biCompression = BI_RGB;
    dibh->biSizeImage = size;
    dibh->biClrUsed = 256;
    dibh->biClrImportant = 0;
```

continued ☞

Example 4-8 Translating a DIB Image Into an IplImage (continued)

```
        // fill in colors of the bitmap
        for( i=0; i<256; i++)
          rgb[i].rgbBlue = rgb[i].rgbGreen = rgb[i].rgbRed =
          (unsigned char)i;
        // set the bitmap data
        for( i=0; i<WIDTH*HEIGHT; i++)
          data[i] = (unsigned char)(100 + i);
        // create ipl image using the bitmap
        if( NULL==(img = iplTranslateDIB( dibh,&cloneData )))
          return 0;
      }
      __finally {
        int flags = IPL_IMAGE_HEADER;
        if( cloneData ) flags |= IPL_IMAGE_DATA;
        if( dib ) free( dib );
        iplDeallocate( img, flags );
      }
      return IPL_StsOk == iplGetErrStatus();
   }
```

A 4-bit-per-pixel gray-scale DIB image with a standard gray palette will be converted into an 8-bit-per-pixel gray-scale `IplImage` and the image data will be copied, returning *`cloneData`* = true.

If *`cloneData`* is false, the data in the output image will be 4-byte-aligned; if *`cloneData`* is true, the output image will have 32-byte-aligned data.

Note that if image data is not copied, the library functions inefficiently access the data. This is because DIB image data is aligned on doubleword (4-byte) boundaries. Alternatively, when *`cloneData`* is true, the DIB image data is replicated into newly allocated image data memory and automatically aligned to 32-byte boundaries, which results in a better memory access.

Return Value

The constructed `IplImage`. If no memory is available in the system to allocate the `IplImage` header or image data, `NULL` value is returned.

ConvertFromDIB

Converts a DIB image to an `IplImage` with specified attributes.

```
void iplConvertFromDIB(BITMAPINFOHEADER* dib,
   IplImage* image)
```

dib	The input DIB image.
image	The `IplImage` header with specified attributes. If the data pointer is `NULL`, image data memory will be allocated and the pointer set to it.

Discussion

The function `iplConvertFromDIB()` converts DIB images to Image Processing Library images according to the attributes set in the `IplImage` header; see Example 4-9. If the image data pointer is `NULL` and there is no memory to allocate the converted image data, the conversion will be interrupted and the function will return a `NULL` pointer.

The following constraints apply to the conversion:

- The bit depth per channel of the `IplImage` should be greater than or equal to that of the DIB image.
- The number of channels (not including the alpha channel) in the `IplImage` should be greater than or equal to the number of channels in the DIB image (not including the alpha channel if present).

- The dimensions of the converted `IplImage` should be greater than or equal to that of the DIB image. When the converted image is larger than the DIB image, the origins of `IplImage` and the DIB image are made coincident for the purposes of copying.
- When converting a DIB RGBA image, the destination `IplImage` should also contain an alpha channel.

Example 4-9 Converting a DIB Image Into an IplImage

```
int example48( void ) {
  BITMAPINFO *dib;              // pointer to bitmap
  RGBQUAD *rgb;                 // pointer to bitmap colors
  unsigned char *data;          // pointer to bitmap data
  BITMAPINFOHEADER *dibh;       // header beginning
  IplImage *img = NULL;
  int i;
  __try {
    int size = HEIGHT * ((WIDTH+3) & ~3);
    // allocate memory for bitmap
    dib = malloc(sizeof(BITMAPINFOHEADER)
     + sizeof(RGBQUAD)*256 + size );
    if( NULL == dib ) return 0;
    // define corresponedt pointers
    dibh = (BITMAPINFOHEADER*)dib;
    rgb=(RGBQUAD*)((char*)dib + sizeof(BITMAPINFOHEADER));
    data = (unsigned char*)((char*)rgb +
           sizeof(RGBQUAD)*256);
    // define bitmap
    dibh->biSize = sizeof(BITMAPINFOHEADER);
    dibh->biWidth = WIDTH;
    dibh->biHeight = HEIGHT;
    dibh->biPlanes = 1;
    dibh->biBitCount = 8;
```

continued ☞

Example 4-9 Converting a DIB Image Into an IplImage (continued)

```
            dibh->biCompression = BI_RGB;
            dibh->biSizeImage = size;
            dibh->biClrUsed = 256;
            dibh->biClrImportant = 0;
            // fill in colors of the bitmap
            for( i=0; i<256; i++)
               rgb[i].rgbBlue = rgb[i].rgbGreen = rgb[i].rgbRed=
                  (unsigned char)i;
            // set the bitmap data
            for( i=0; i<WIDTH*HEIGHT; i++)
               data[i] = (unsigned char)(100 + i);
            // create header of the desired image
            img = iplCreateImageHeader( 1,0, IPL_DEPTH_16U,
               "GRAY", "GRAY", IPL_DATA_ORDER_PIXEL,
               IPL_ORIGIN_BL,  // bottom left as in DIB
               IPL_ALIGN_QWORD, WIDTH, HEIGHT, NULL, NULL, NULL,
               NULL);
            if( NULL == img ) return 0;

            // create ipl image converting 8u to 16u
            iplConvertFromDIB ( dibh, img );
            if( !img->imageData ) return 0;
          }
          __finally {
            if( dib ) free( dib );
            iplDeallocate(img,IPL_IMAGE_HEADER|IPL_IMAGE_DATA);
          }
          return IPL_StsOk == iplGetErrStatus();
        }
```

As necessary, the conversion result is saturated.

ConvertFromDIBSep

Converts a DIB image to an `IplImage`*, using two arguments for the DIB header and data.*

```
IPLStatus iplConvertFromDIBSep (BITMAPINFOHEADER*
    dibHeader, const char* dibData, IplImage* image);
```

dibHeader	The input DIB image header.
dibData	The input DIB image data.
image	The `IplImage` header with specified attributes. If the data pointer is `NULL`, image data memory will be allocated and the pointer set to it.

Discussion

Similar to `iplConvertFromDIB`, the function `iplConvertFromDIBSep` converts DIB images to Image Processing Library images according to the attributes set in the `IplImage` header. The input and output images must satisfy the same conditions as for `iplConvertFromDIB`.

The function `iplConvertFromDIBSep` uses an additional argument for the DIB data. This allows you to supply the DIB header and data stored separately.

Return Value

The function returns an `IPLStatus` status code.

ConvertToDIB

Converts an `IplImage` to a DIB image with specified attributes.

```
void iplConvertToDIB(iplImage* image, BITMAPINFOHEADER*
    dib, int dither, int paletteConversion)
```

image	The input `IplImage`.
dib	The output DIB image.
dither	The dithering algorithm to use if applicable. Dithering will be done if the bit depth in the DIB is less than that of the `IplImage`. The following algorithms are supported corresponding to these *dither* identifiers:

`IPL_DITHER_FS`	The Floid-Steinberg error diffusion dithering algorithm is used.
`IPL_DITHER_JJH`	The Jarvice-Judice-Ninke error diffusion dithering algorithm is used.
`IPL_DITHER_STUCKEY`	The Stucki dithering algorithm is used.
`IPL_DITHER_BAYER`	The Bayer threshold dithering algorithm is used.
`IPL_DITHER_NONE`	No dithering is done. The most significant bits in the input image pixel data are retained.

paletteConversion	Applicable when the DIB is a palette image. Specifies the palette algorithm to use when converting an absolute color `IplImage`. The following options are supported:

`IPL_PALCONV_NONE`	The existing palette in the DIB is used.

`IPL_PALCONV_POPULATE`	The popularity palette conversion algorithm is used.
`IPL_PALCONV_MEDCUT`	The median cut algorithm for palette conversion is used.

Discussion

The function `iplConvertToDIB()` converts an `IplImage` to a DIB image. The conversion takes place according to the source and destination image attributes. While `IplImage` format always uses absolute color, DIB images can be in absolute or palette color. When the DIB is a palette image, the absolute color `IplImage` is converted to a palette image according to the palette conversion option specified. When the bit depth of an absolute color DIB image is less than that of the `IplImage`, then dithering according to the specified option is performed.

The following constraints apply when using this function:

- The number of channels in the `IplImage` should be equal to the number of channels in the DIB image.
- The alpha channel in an `IplImage` will be passed on only when the DIB is an RGBA image.

ConvertToDIBSep

Converts an `IplImage` to a DIB image, with DIB header and data stored separately.

```
IPLStatus iplConvertToDIBSep(iplImage* image,
   BITMAPINFOHEADER* dib, char* dibData, int dither,
   int paletteConversion)
```

image	The input `IplImage`.
dib	The output DIB image header.

dibData	The output DIB image data.
dither	The dithering algorithm to use if applicable. Dithering will be done if the bit depth in the DIB is less than that of the `IplImage`. The following algorithms are supported corresponding to these *dither* identifiers:
`IPL_DITHER_FS`	The Floid-Steinberg error diffusion dithering algorithm is used.
`IPL_DITHER_JJH`	The Jarvice-Judice-Ninke error diffusion dithering algorithm is used.
`IPL_DITHER_STUCKEY`	The Stucki dithering algorithm is used.
`IPL_DITHER_BAYER`	The Bayer threshold dithering algorithm is used.
`IPL_DITHER_NONE`	No dithering is done. The most significant bits in the input image pixel data are retained.
paletteConversion	Applicable when the DIB is a palette image. Specifies the palette algorithm to use when converting an absolute color `IplImage`. The following options are supported:
`IPL_PALCONV_NONE`	The existing palette in the DIB is used.
`IPL_PALCONV_POPULATE`	The popularity palette conversion algorithm is used.
`IPL_PALCONV_MEDCUT`	The median cut algorithm for palette conversion is used.

Discussion

The function `iplConvertToDIBSep()` converts an `IplImage` to a DIB image with header and data stored separately, in *dib* and *dibData*. See `iplConvertToDIB` for more information about the conversion.

Image Arithmetic and Logical Operations 5

This chapter describes image processing functions that modify pixel values using simple arithmetic or logical operations. It also includes the library functions that perform image compositing based on opacity (alpha-blending). All these operations can be broken into two categories: monadic operations, which use single input images, and dyadic operations, which use two input images. Table 5-1 lists the functions that perform arithmetic and logical operations.

Table 5-1 Image Arithmetic and Logical Operations

Group	Function Name	Description
Arithmetic operations	`iplAddS` `iplAddSFP`	Adds a constant to the image pixel values.
	`iplSubtractS` `iplSubtractSFP`	Subtracts a constant from the pixel values or the values from a constant.
	`iplMultiplyS` `iplMultiplySFP`	Multiplies pixel values by a constant.
	`iplMultiplySScale`	Multiplies pixel values by a constant and scales the product.
	`iplAbs`	Computes absolute pixel values.
	`iplAdd`	Adds pixel values of two images.
	`iplSubtract`	Subtracts pixel values of one image from those of another image.
	`iplSquare`	Squares the pixel values of an image.

Continued ☞

Table 5-1 Image Arithmetic and Logical Operations (continued)

Group	Function Name	Description
Arithmetic operations (continued)	`iplMultiply`	Multiplies pixel values of two images.
	`iplMultiplyScale`	Multiplies pixel values of two images and scales the product.
Logical operations	`iplAndS`	Performs a bitwise AND operation on each pixel with a constant.
	`iplOrS`	Performs a bitwise OR operation on each pixel with a constant.
	`iplXorS`	Performs a bitwise XOR operation on each pixel with a constant.
	`iplNot`	Performs a bitwise NOT operation on each pixel
	`iplLShiftS`	Shifts bits in pixel values to the left.
	`iplRShiftS`	Divides pixel values by a constant power of 2 by shifting bits to the right.
	`iplAnd`	Combines corresponding pixels of two images by a bitwise AND operation.
	`iplOr`	Combines corresponding pixels of two images by a bitwise OR operation.
	`iplXor`	Combines corresponding pixels of two images by a bitwise XOR operation.
Alpha-blending	`iplPreMultiplyAlpha`	Pre-multiplies pixel values of an image by alpha values.
	`iplAlphaComposite`	Composites two images using alpha (opacity) values.
	`iplAlphaCompositeC`	Composites two images using constant alpha (opacity) values.

The functions `iplSquare()`, `iplNot()`, `iplPreMultiplyAlpha()`, and `iplAbs()` as well as all functions with names containing an additional `S` use single input images (perform monadic operations). All other functions in the above table use two input images (perform dyadic operations).

Monadic Arithmetic Operations

The sections that follow describe the library functions that perform monadic arithmetic operations (note that the `iplPreMultiplyAlpha` function is described in the "Image Compositing Based on Opacity" section of this chapter). All these functions use a single input image to create an output image.

AddS, AddSFP

Adds a constant to pixel values of the source image.

```
void iplAddS(IplImage* srcImage, IplImage* dstImage, int
value);
```

```
void iplAddSFP(IplImage* srcImage, IplImage* dstImage,
float value);  /* images with IPL_DEPTH_32F only */
```

`srcImage`	The source image.
`dstImage`	The resultant image.
`value`	The value to be added to the pixel values.

Discussion

The functions change the image intensity by adding the `value` to pixel values. A positive `value` brightens the image (increases the intensity); a negative `value` darkens the image (decreases the intensity).

SubtractS, SubtractSFP

Subtracts a constant from pixel values, or pixel values from a constant.

```
void iplSubtractS(IplImage* srcImage, IplImage* dstImage,
int value, BOOL flip);
```

```
void iplSubtractSFP(IplImage* srcImage,IplImage* dstImage,
float value, BOOL flip); /*  IPL_DEPTH_32F only */
```

srcImage	The source image.
dstImage	The resultant image.
value	The value to be subtracted from the pixel values.
flip	A Boolean used to change the order of subtraction.

Discussion

The functions change the image intensity as follows:

If *flip* is false, the *value* is subtracted from the image pixel values.
If *flip* is true, the image pixel values are subtracted from the *value*.

MultiplyS, MultiplySFP

Multiplies pixel values by a constant.

```
void iplMultiplyS (IplImage* srcImage, IplImage* dstImage,
int value);
```

```
void iplMultiplySFP(IplImage* srcImage,IplImage* dstImage,
float value);   /* images with IPL_DEPTH_32F only */
```

srcImage	The source image.

`dstImage` The resultant image.

`value` An integer value by which to multiply the pixel values.

Discussion

The functions change the image intensity by multiplying each pixel by a constant `value`.

MultiplySScale

Multiplies pixel values by a constant and scales the products.

```
void iplMultiplySScale(IplImage* srcImage, IplImage*
dstImage, int value);
```

`srcImage` The source image.

`dstImage` The resultant image.

`value` A positive value by which to multiply the pixel values.

Discussion

The function `iplMultiplySScale()` multiplies the input image pixel values by `value` and scales the products using the following formula:

`dst_pixel` = `src_pixel` * `value` / `max_val`

where `src_pixel` is a pixel value of the source images, `dst_pixel` is the resultant pixel value, and `max_val` is the maximum presentable pixel value. This function can be used to multiply the image by a number between 0 and 1.

The source and resultant images must have the same pixel depth. The function is implemented only for 8-bit and 16-bit unsigned data types.

Square

Squares the pixel values of the image.

```
void iplSquare(IplImage* srcImage, IplImage* dstImage);
```

srcImage	The source image.
dstImage	The resultant image.

Discussion

The function `iplSquare()` increases the intensity of an image by squaring each pixel value.

Abs

Computes absolute pixel values of the image.

```
void iplAbs(IplImage* srcImage, IplImage* dstImage);
```

srcImage	The source image.
dstImage	The resultant image.

Discussion

The function `iplAbs()` takes the absolute value of each channel in each pixel of the image.

Dyadic Arithmetic Operations

The sections that follow describe the functions that perform dyadic arithmetic operations. These functions use two input images to create an output image.

Add

Combines corresponding pixels of two images by addition.

```
void iplAdd(IplImage* srcImageA, IplImage* srcImageB,
IplImage* dstImage);
```

srcImageA	The first source image.
srcImageB	The second source image.
dstImage	The resultant image obtained as *dst_pixel* = *srcA_pixel* + *srcB_pixel*.

Discussion

The function `iplAdd()` adds corresponding pixels of two input images to produce the output image.

Subtract

Combines corresponding pixels of two images by subtraction.

```
void iplSubtract(IplImage* srcImageA, IplImage* srcImageB,
IplImage* dstImage);
```

`srcImageA` The first source image.

`srcImageB` The second source image.

`dstImage` The resultant image obtained as:
dst_pixel = *srcA_pixel* - *srcB_pixel*.

Discussion

The function `iplSubtract()` subtracts corresponding pixels of two input images to produce the output image.

Multiply

Combines corresponding pixels of two images by multiplication.

```
void iplMultiply(IplImage* srcImageA, IplImage* srcImageB,
IplImage* dstImage);
```

`srcImageA` The first source image.

`srcImageB` The second source image.

`dstImage` The resultant image.

Discussion

The function `iplMultiply()` multiplies corresponding pixels of two input images to produce the output image.

MultiplyScale

Multiplies pixel values of two images and scales the products.

```
void iplMultiplyScale(IplImage* srcImageA, IplImage*
srcImageB, IplImage* dstImage);
```

srcImageA	The first source image.
srcImageB	The second source image.
dstImage	The resultant image.

Discussion

The function `iplMultiplyScale()` multiplies corresponding pixels of two input images and scales the products using the following formula:

dst_pixel = *srcA_pixel* * *srcB_pixel* / *max_val*

where *srcA_pixel* and *srcB_pixel* are pixel values of the source images, *dst_pixel* is the resultant pixel value, and *max_val* is the maximum presentable pixel value. Both source images and the resultant image must have the same pixel depth. The function is implemented only for 8-bit and 16-bit unsigned data types.

Monadic Logical Operations

The sections that follow describe the functions that perform monadic logical operations. All these functions use a single input image to create an output image.

LShiftS

Shifts pixel values' bits to the left.

```
void iplLShiftS(IplImage* srcImage, IplImage* dstImage,
unsigned int nShift);
```

srcImage	The source image.
dstImage	The resultant image.
nShift	The number of bits by which to shift each pixel value to the left.

Discussion

The function `iplLShiftS()` changes the intensity of the source image by shifting the bits in each pixel value by *nShift* bits to the left. The positions vacated after shifting the bits are filled with zeros.

RShiftS

Divides pixel values by a constant power of 2 by shifting bits to the right.

```
void iplRShiftS(IplImage* srcImage, IplImage* dstImage,
unsigned int nShift);
```

srcImage	The source image.
dstImage	The resultant image.
nShift	The number of bits by which to shift each pixel value to the right.

Discussion

The function `iplRShiftS()` decreases the intensity of the source image by shifting the bits in each pixel value by *nShift* bits. The positions vacated after shifting the bits are filled with zeros.

Not

Performs a bitwise NOT operation on each pixel.

```
void iplNot(IplImage* srcImage, IplImage* dstImage);
```

`srcImage`	The source image.
`dstImage`	The resultant image.

Discussion

The function `iplNot()` performs a bitwise NOT operation on each pixel value.

AndS

Performs a bitwise AND operation of each pixel with a constant.

```
void iplAndS(IplImage* srcImage, IplImage* dstImage,
unsigned int value);
```

`srcImage`	The source image.
`dstImage`	The resultant image.
`value`	The bit sequence used to perform the bitwise AND operation on each pixel.

Discussion

The function `iplAndS()` performs a bitwise AND operation between each pixel value and `value`. The least significant bit(s) of the `value` are used.

OrS

Performs a bitwise OR operation of each pixel with a constant.

```
void iplOrS(IplImage* srcImage, IplImage* dstImage,
unsigned int value);
```

srcImage	The source image.
dstImage	The resultant image.
value	The bit sequence used to perform the bitwise OR operation on each pixel.

Discussion

The function `iplOrS()` performs a bitwise OR between each pixel value and *value*. The least significant bit(s) of the *value* are used.

XorS

Performs a bitwise XOR operation of each pixel with a constant.

```
void iplXorS(IplImage* srcImage, IplImage* dstImage,
unsigned int value);
```

`srcImage`	The source image.
`dstImage`	The resultant image.
`value`	The bit sequence used to perform the bitwise XOR operation on each pixel.

Discussion

The function `iplXorS()` performs a bitwise XOR between each pixel value and `value`. The least significant bit(s) of the `value` are used.

Dyadic Logical Operations

This section describes the library functions that perform dyadic logical operations. These functions use two input images to create an output image.

And

Combines corresponding pixels of two images by a bitwise AND operation.

```
void iplAnd(IplImage* srcImageA, IplImage* srcImageB,
IplImage* dstImage);
```

`srcImageA` The first source image.

`srcImageB` The second source image.

`dstImage` The image resulting from the bitwise operation between input images `srcImageA` and `srcImageB`.

Discussion

The function `iplAnd()` performs a bitwise AND operation between the values of corresponding pixels of two input images.

Or

Combines corresponding pixels of two images by a bitwise OR operation.

```
void iplOr(IplImage* srcImageA, IplImage* srcImageB,
IplImage* dstImage);
```

`srcImageA` The first source image.

`srcImageB` The second source image.

`dstImage` The image resulting from the bitwise operation between input images `srcImageA` and `srcImageB`.

Discussion

The function `iplOR()` performs a bitwise OR operation between the values of corresponding pixels of two input images.

Xor

Combines corresponding pixels of two images by a bitwise XOR operation.

```
void iplXor(IplImage* srcImageA, IplImage* srcImageB,
IplImage* dstImage);
```

srcImageA	The first source image.
srcImageB	The second source image.
dstImage	The image resulting from the bitwise operation between input images *srcImageA* and *srcImageB*.

Discussion

The function `iplXor()` performs a bitwise XOR operation between the values of corresponding pixels of two input images.

Image Compositing Based on Opacity

The Image Processing Library provides functions to composite two images using either the opacity (alpha) channel in the images or a provided alpha value. Alpha values range from 0 (100% translucent, 0% coverage) to full range (0% translucent, 100% coverage). Coverage is the percentage of the pixel's own intensity that is visible.

Using the opacity channel for image compositing provides the capability of overlaying the arbitrarily shaped and transparent images in arbitrary positions. It also reduces aliasing effects along the edges of the combined regions by allowing some of the bottom image's color to show through.

Let us consider the example of RGBA images. Here each pixel is a quadruple (r, g, b, α) where r, g, b, and α are the red, green, blue and alpha channels, respectively. In the formulas that follow, the Greek letter α with subscripts always denotes the normalized (scaled) alpha value in the range 0 to 1. It is related to the integer alpha value `aphaValue` as follows:

$$\alpha = aphaValue / max\_val$$

where `max_val` is 255 for 8-bit or 65535 for 16-bit unsigned pixel data.

There are many ways of combining images using alpha values. In all compositing operations a resultant pixel (r_C, g_C, b_C, α_C) in image C is created by overlaying a pixel (r_A, g_A, b_A, α_A) from the foreground image A over a pixel (r_B, g_B, b_B, α_B) from the background image B. The resulting pixel values for an OVER operation (A OVER B) are computed as shown below.

$$r_C = \alpha_A * r_A + (1 - \alpha_A) * \alpha_B * r_B$$

$$g_C = \alpha_A * g_A + (1 - \alpha_A) * \alpha_B * g_B$$

$$b_C = \alpha_A * b_A + (1 - \alpha_A) * \alpha_B * b_B$$

The above three expressions can be condensed into one as follows:

$$C = \alpha_A * A + (1 - \alpha_A) * \alpha_B * B$$

In this example, the color of the background image B influences the color of the resultant image through the second term $(1 - \alpha_A) * \alpha_B * B$. The resulting alpha value is computed as

$$\alpha_C = \alpha_A + (1 - \alpha_A) * \alpha_B$$

Using Pre-multiplied Alpha Values

In many cases it is computationally more efficient to store the color channels pre-multiplied by the alpha values. In the RGBA example, the pixel (r, g, b, α) would actually be stored as (r*α, g*α, b*α, α). This storage format reduces the number of multiplications required in the compositing operations. In interactive environments, when an image is composited many times, this capability is especially efficient.

One known disadvantage of the pre-multiplication is that once a pixel is marked as transparent, its color value is gone because the pixel's color channels are multiplied by 0.

The function `iplPreMultiplyAlpha()` implements various alpha compositing operations between two images. One of them is converting the pixel values to pre-multiplied form.

The color channels in images with the alpha channel can be optionally pre-multiplied with the alpha value. This saves a significant amount of computation for some of the alpha compositing operations. For example, in an RGBA color model image, if (r, g, b, α) are the channel values for a pixel, then upon pre-multiplication they are stored as (r*α, g*α, b*α, α).

AlphaComposite
AlphaCompositeC

Composite two images using alpha (opacity) values.

```
void iplAlphaComposite(IplImage* srcImageA, IplImage*
srcImageB, IplImage* dstImage, int compositeType,
IplImage* alphaImageA, IplImage* alphaImageB, IplImage*
alphaImageDst, BOOL premulAlpha, BOOL divideMode);
```

```
void iplAlphaCompositeC(IplImage* srcImageA, IplImage*
srcImageB, IplImage* dstImage, int compositeType, int aA,
int aB, BOOL premulAlpha, BOOL divideMode);
```

`srcImageA`	The foreground input image.
`srcImageB`	The background input image.
`dstImage`	The resultant output image.
`compositeType`	The composition type to perform. See Table 5-2 for the type value and description.
`aA`	The constant alpha value to use for the source image `srcImageA`. Should be a positive number.
`aB`	The constant alpha value to use for the source image `srcImageB`. Should be a positive number.
`alphaImageA`	The image to use as the alpha channel for `srcImageA`. If the image `alphaImageA` contains an alpha channel, that channel is used. Otherwise channel 1 in `alphaImageA` is used as the alpha channel. If this is not suitable for the application, then the alpha channel number in the `IplImage` header for the image should be set appropriately before calling this function. If the argument `alphaImageA` is `NULL`, then the internal alpha channel of `srcImageA` is used. If `srcImageA` does not contain an alpha channel, an error message is issued.
`alphaImageB`	The image to use as the alpha channel for `srcImageB`. If the image `alphaImageB` already contains an alpha channel, that channel is used. Otherwise channel 1 in `alphaImageB` is used as the alpha channel. If this is not suitable for the application, then the alpha channel number in the image header for the image should be set appropriately before calling this function. If the argument `alphaImageB` is `NULL`, then the internal alpha channel of `srcImageB` is used.

If `srcImageB` does not contain an alpha channel, then the value ($1 - \alpha_A$) is used for the alpha, where α_A is a scaled alpha value of `srcImageA` in the range 0 to 1.

`alphaImageDst` The image to use as the alpha channel for `dstImage`. If the image already contains an alpha channel, that channel is used. Otherwise channel 1 in the image is used as the alpha channel. If this is not suitable for the application, then the alpha channel number in the image header for the image should be set appropriately before calling this function. This argument can be `NULL`, in which case the resultant alpha values are not saved.

`premulAlpha` A Boolean flag indicating whether or not the input images contain pre-multiplied alpha values. If true, they contain these values.

`divideMode` A Boolean flag related to `premulAlpha`. When true, the resultant pixel color (see Table 5-2) is further divided by the resultant alpha value to get the final resultant pixel color.

Discussion

The function `iplAlphaComposite()` performs an image compositing operation by overlaying the foreground image `srcImageA` with the background image `srcImageB` to produce the resultant image `dstImage`.

The function `iplAlphaComposite()` executes under one of the following conditions for the alpha channels:

- If `alphaImageA` and `alphaImageB` are both `NULL`, then the internal alpha channels of the two input images specified by their respective `IplImage` headers are used. The application has to ensure that these are set to the proper channel number prior to calling this function. If `srcImageB` does not have an alpha channel, then its alpha value is set to $(1 - \alpha_A)$ where α_A is the scaled alpha value of image `srcImageA` in the range 0 to 1.
- If both alpha images `alphaImageA` and `alphaImageB` are not `NULL`, then they are used as the alpha values for the two input images. If `alphaImageB` is `NULL`, then its alpha value is set to $(1 - \alpha_A)$ where α_A is the scaled alpha value of image `alphaImageA` in the range 0 to 1.

It is an error if none of the above conditions is satisfied.

If `alphaImageDst` is not `NULL`, then the resultant alpha values are written to it. If it is `NULL` and the output image `imageDst` contains an alpha channel (specified by the `IplImage` header), then it is set to the resulting alpha values.

The function `iplAlphaCompositeC()` is used to specify constant alpha values α_A and α_B to be used for the two input images (usually α_B is set to the value $1 - \alpha_A$). The resultant alpha values (also constant) are not saved.

The type of compositing is specified by the argument `compositeType` which can assume the values shown in Table 5-2.

The functions `iplAlphaCompositeC()` and `iplAlphaCompositeC()` can be used for unsigned pixel data only. They support ROI, mask ROI and tiling.

Table 5-2 Types of Image Compositing Operations

Type	Output Pixel (see Note)	Output Pixel (pre-mult. α)	Resultant Alpha	Description
OVER	α_A*A+ (1- α_A)*α_B*B	A+(1-α_A)*B	α_A+ (1- α_A)* α_B	A occludes B
IN	α_A*A* α_B	A*α_B	α_A* α_B	A within B. A acts as a matte for B. A shows only where B is visible.
OUT	α_A*A*(1- α_B)	A*(1- α_B)	α_A *(1- α_B)	A outside B. NOT-B acts as a matte for A. A shows only where B is not visible.
ATOP	α_A*A* α_B+ (1- α_A)*α_B*B	A* α_B+ (1- α_A)*B	α_A* α_B+ (1- α_A)* α_B	Combination of (A IN B) and (B OUT A). B is both background and matte for A.
XOR	α_A*A*(1-α_B)+ (1- α_A)* α_B*B	A*(1- α_B)+ (1- α_A)*B	α_A*(1- α_B)+ (1- α_A)* α_B	Combination of (A OUT B) and (B OUT A). A and B mutually exclude each other.
PLUS	α_A *A + α_B*B	A + B	α_A + α_B	Blend without precedence

NOTE. *In Table 5-2, the resultant pixel value is divided by the resultant alpha when* `divideMode` *is set to true (see the argument descriptions for the* `iplAlphaComposite()` *function). The Greek letter α here and below denotes normalized (scaled) alpha values in the range 0 to 1.*

For example, for the OVER operation, the output C for each pixel in the inputs A and B is determined as

$$C = \alpha_A * A + (1 - \alpha_A) * \alpha_B * B$$

The above operation is done for each color channel in A, B, and C. When the images A and B contain pre-multiplied alpha values, C is determined as

$$C = A + (1 - \alpha_A) * B$$

The resultant alpha value `aC` (alpha in the resultant image C) is computed as (both pre-multiplied and not pre-multiplied alpha cases) from `aA` (alpha in the source image A) and `aB` (alpha in the source image B):

$$\alpha_C = \alpha_A + (1 - \alpha_A) * \alpha_B$$

Thus, to perform an OVER operation, use the `IPL_COMPOSITE_OVER` identifier for the argument *`compositeType`*. For all other types, use `IPL_COMPOSITE_IN`, `IPL_COMPOSITE_OUT`, `IPL_COMPOSITE_ATOP`, `IPL_COMPOSITE_XOR`, and `IPL_COMPOSITE_PLUS`, respectively.

The argument *`divideMode`* is typically set to false to give adequate results as shown in the above example for an OVER operation and in Table 5-2. When *`divideMode`* is set to true, the resultant pixel color is divided by the resultant alpha value. This gives an accurate result pixel value, but the division operation is expensive. In terms of the OVER example without pre-multiplication, the final value of the pixel C is computed as

$$C = (\alpha_A * A + (1 - \alpha_A) * \alpha_B * B)/\alpha_C$$

There is no change in the value of α_C, and it is computed as shown above. When both A and B are 100% transparent (that is, α_A is zero and α_B is zero), α_C is also zero and the result cannot be determined. In many cases, the value of α_C is 1, so the division has no effect.

PreMultiplyAlpha

Pre-multiplies alpha values of an image.

```
void iplPreMultiplyAlpha (IplImage* image,
int alphaValue);
```

`image`	The image for which the alpha pre-multiplication is performed.
`alphaValue`	The global alpha value to use in the range 0 to 256. If this value is negative (for example, -1), the internal alpha channel of the image is used. It is an error condition if an alpha channel does not exist.

Discussion

The function `iplPreMultiplyAlpha()` converts an image to the pre-multiplied alpha form. If (R, G, B, A) are the red, green, blue, and alpha values of a pixel, then the pixel is stored as (R*α, G*α, B*α, A) after execution of this function. Here α is the pixel's normalized alpha value in the range 0 to 1.

Optionally, a global alpha value `alphaValue` can be used for the entire image. Then the pixels are stored as (R*α, G*α, B*α, `alphaValue`) if the image has an alpha channel or (R*α, G*α, B*α) if the image does not have an alpha channel. Here α is the normalized `alphaValue` in the range 0 to 1.

The function `iplPreMultiplyAlpha()` can be used for unsigned pixel data only. It supports ROI, mask ROI and tiling.

Image Filtering

6

This chapter describes linear and non-linear filtering operations supported by the Image Processing Library. Most linear filtering is performed through convolution, either with user-defined convolution kernels or with the provided fixed filter kernels. Table 6-1 lists the filtering functions.

Table 6-1 Image Filtering Functions

Group	Function Name	Description
Linear Filters	iplBlur	Applies a simple neighborhood averaging filter.
2-dimensional Convolution Linear Filters	iplCreateConvKernel<br>iplCreateConvKernelChar<br>iplCreateConvKernelFP	Creates a convolution kernel.
	iplGetConvKernel<br>iplGetConvKernelChar<br>iplGetConvKernelFP	Reads the attributes of a convolution kernel.
	iplDeleteConvKernel<br>iplDeleteConvKernelFP	Deallocates a convolution kernel.
	iplConvolve2D<br>iplConvolve2DFP	Convolves an image with one or more convolution kernels.
	iplConvolveSep2D<br>iplConvolveSep2DFP	Convolves an image with a separable convolution kernel.
	iplFixedFilter	Convolves an image with a predefined kernel.
Non-linear Filters	iplMedianFilter	Applies a median filter.
	iplColorMedianFilter	Applies a color median filter
	iplMaxFilter	Applies a maximum filter.
	iplMinFilter	Applies a minimum filter.

Linear Filters

Linear filtering includes a simple neighborhood averaging filter, 2D convolution operations, and a number of filters with fixed effects.

Blur

Applies simple neighborhood averaging filter to blur the image.

```
void iplBlur(IplImage* srcImage, IplImage* dstImage,
int nCols, int nRows, int anchorX, int anchorY);
```

`srcImage`	The source image.
`dstImage`	The resultant image.
`nCols`	Number of columns in the neighborhood to use.
`nRows`	Number of rows in the neighborhood to use.
`anchorX, anchorY`	The [*x*, *y*] coordinates of the anchor cell in the neighborhood. In this coordinate system, the top left corner would be [0, 0] and the bottom right corner would be [`nCols-1`, `nRows-1`]. For a 3 by 3 neighborhood, the coordinates of the geometric center would be [1, 1]. This specification allows the neighborhood to be skewed with respect to its geometric center.

Discussion

The function `iplBlur()` sets each pixel in the output image as the average of all the input image pixels in the neighborhood of size `nRows` by `nCols` with the anchor cell at that pixel. This has the effect of smoothing or blurring the input image. The linear averaging filter of an image is also called a box filter.

2D Convolution

The 2D convolution is a versatile image processing primitive which can be used in a variety of image processing operations; for example, edge detection, blurring, noise removal, and feature detection. It is also known as mask convolution or spatial convolution.

NOTE. *In some literature sources, the 2D convolution is referred to as box filtering, which is an incorrect use of the term. A box filter is a linear averaging filter (see function* `iplBlur` *above). Technically, a box filter can be effectively (although less efficiently) implemented by 2D convolution using a kernel with unit or constant values.*

For 2D convolution, a rectangular kernel is used. The kernel is a matrix of signed integers or single-precision real values. The kernel could be a single row (a row filter) or a single column (a column filter) or composed of many rows and columns. There is a cell in the kernel called the “anchor,” which is usually a geometric center of the kernel, but can be skewed with respect to the geometric center.

For each input pixel, the kernel is placed on the image such that the anchor coincides with the input pixel. The output pixel value is computed as

$$y_{m,n} = \sum_i \sum_k h_{i,k} \, x_{m-i,n-k}$$

where $x_{m-i,n-k}$ is the input pixel value and $h_{i,k}$ denotes the kernel. Optionally, the output pixel value may be scaled.

The convolution function can be used in two ways. The first way uses a single kernel for convolution. The second way uses multiple kernels and allows the specification of a method to combine the results of convolution with each kernel. This enables efficient implementation of multiple kernels which eliminates the need of storing the intermediate results when using each kernel. The functions `iplConvolve2D()` and `iplConvolve2DFP()` can implement both ways.

In addition, `iplConvolveSep2D()`, a convolution function that uses separable kernels, is also provided. It works with convolution kernels that are separable into the *x* and *y* components.

Before performing a convolution, you should create the convolution kernel and be able to access the kernel attributes. You can do this using the functions `iplCreateConvKernel()`, `iplGetConvKernel()`, `iplCreateConvKernelFP()` and `iplGetConvKernelFP()`.

In release 2.0, the function `iplFixedFilter()` function has been added to the library. It allows you to convolve images with a number of commonly used kernels that correspond to Gaussian, Laplacian, highpass, and gradient filtering.

Also, for compatibility with previous releases, the functions `iplCreateConvKernelChar()` and `iplGetConvKernelChar()` have been added. They use 1-byte `char` kernel values, as opposed to integer kernel values in `iplCreateConvKernel()` and `iplGetConvKernel()` .

CreateConvKernel, CreateConvKernelChar, CreateConvKernelFP

Creates a convolution kernel.

```
IplConvKernel* iplCreateConvKernel(int nCols, int nRows,
int anchorX, int anchorY, int* values, int nShiftR);
```

```
IplConvKernel* iplCreateConvKernelChar(int nCols, int
nRows, int anchorX, int anchorY, char* values, int
nShiftR);
```

```
IplConvKernelFP* iplCreateConvKernelFP(int nCols, int
nRows, int anchorX, int anchorY, float *values);
```

`nCols`	The number of columns in the convolution kernel.
`nRows`	The number of rows in the convolution kernel.
`anchorX, anchorY`	The [x, y] coordinates of the anchor cell in the kernel. In this coordinate system, the top left corner would be [0, 0] and the bottom right corner would be [`nCols-1`, `nRows-1`]. For a 3 by 3 kernel, the coordinates of the geometric center would be [1, 1]. This specification allows the kernel to be skewed with respect to its geometric center.
`values`	A pointer to an array of values to be used for the kernel matrix. The values are read in row-major form starting with the top left corner. There should be exactly `nRows*nCols` entries in this array. For example, the array [1, 2, 3, 4, 5, 6, 7, 8, 9] would represent the following kernel matrix:<br>1 2 3<br>4 5 6<br>7 8 9

nShiftR — Scale the resulting output pixel by shifting it to the right *nShiftR* times.

Discussion

Functions `iplCreateConvKernel()` and `iplCreateConvKernelFP()` are used to create convolution kernels of arbitrary size with arbitrary anchor point. The function `iplCreateConvKernelChar()` serves primarily for compatibility with previous releases of the library. It uses `char` rather than integer input values to creates the same kernel as `iplCreateConvKernel()`.

Return Value

A pointer to the convolution kernel structure `IplConvKernel`.

GetConvKernel, GetConvKernelChar GetConvKernelFP

Reads the attributes of a convolution kernel.

```
void iplGetConvKernel(IplConvKernel* kernel, int* nCols,
int* nRows, int* anchorX, int* anchorY, int** values,
int* nShiftR);
```

```
void iplGetConvKernelChar(IplConvKernel* kernel, int*
nCols, int* nRows, int* anchorX, int* anchorY, char**
values, int* nShiftR);
```

```
void iplGetConvKernelFP(IplConvKernelFP* kernel, int*
nCols, int* nRows, int* anchorX, int* anchorY, float**
values);
```

kernel — The kernel to get the attributes for. The attributes are returned in the remaining arguments.

`nCols, nRows`	Numbers of columns and rows in the convolution kernel. Set by the function.
`anchorX, anchorY`	Pointers to the [x, y] coordinates of the anchor cell in the kernel. (See iplCreateConvKernel above.) Set by the function.
`values`	A pointer to an array of values to be used for the kernel matrix. The values are read in row-major form starting with the top left corner. There will be exactly `nRows`*`nCols` entries in this array. For example, the array [1, 2, 3, 4, 5, 6, 7, 8, 9] would represent the kernel matrix<br>1 2 3<br>4 5 6<br>7 8 9
`nShiftR`	A pointer to the number of bits to shift (to the right) the resulting output pixel of each convolution. Set by the function.

Discussion

Functions `iplGetConvKernel()` and `iplGetConvKernelFP()` are used to read the convolution kernel attributes. The `iplGetConvKernelChar()` function serves primarily for compatibility with previous releases. It gives you 1-byte `char` rather than integer values of the convolution kernel; you'll probably need this function only if you create kernels using `iplCreateConvKernelChar()`.

DeleteConvKernel DeleteConvKernelFP

Deletes a convolution kernel.

```
void iplDeleteConvKernel(IplConvKernel* kernel);
void iplDeleteConvKernelFP(IplConvKernelFP* kernel);
```

`kernel` The kernel to delete.

Discussion

Functions `iplDeleteConvKernel()` and `iplDeleteConvKernelFP()` must be used to delete convolution kernels created, respectively, by `iplCreateConvKernel()` and `iplCreateConvKernelFP()`.

Convolve2D Convolve2DFP

Convolves an image with one or more convolution kernels.

```
void iplConvolve2D(IplImage* srcImage, IplImage* dstImage,
IplConvKernel** kernel, int nKernels, int combineMethod);
```

```
void iplConvolve2DFP(IplImage* srcImage, IplImage* dstImage,
IplConvKernelFP** kernel, int nKernels, int combineMethod);
```

`srcImage` The source image.

`dstImage` The resultant image.

`kernel` A pointer to an array of pointers to convolution kernels. The length of the array is `nKernels`.

nKernels — The number of kernels in the array *kernel*. The value of *nKernels* can be 1 or more.

combineMethod — The way in which the results of applying each kernel should be combined. This argument is ignored when a single kernel is used. The following combinations are supported:

IPL_SUM	Sums the results.
IPL_SUMSQ	Sums the squares of the results.
IPL_SUMSQROOT	Sums the squares of the results and then takes the square root.
IPL_MAX	Takes the maximum of the results.
IPL_MIN	Takes the minimum of the results.

Discussion

Functions `iplConvolve2D()` and `iplConvolve2D()` are used to convolve an image with a set of convolution kernels. The results of using each kernel are then combined using the *combineMethod* argument; see Example 6-1.

Example 6-1 Computing the 2-dimensional Convolution

```
int example61( void ) {
   IplImage *imga, *imgb;
   int one[9] = {1,0,1, 0,0,0, 1,0,1};  // a kernel to check
   IplConvKernel* kernel;               // REFLECT border mode
   __try {
      int i;
      imga= iplCreateImageHeader( 1, 0, IPL_DEPTH_8U, "GRAY",
         "GRAY", IPL_DATA_ORDER_PIXEL, IPL_ORIGIN_TL,
         IPL_ALIGN_DWORD, 4, 4, NULL, NULL, NULL, NULL);
```

continued ☞

Example 6-1 Computing 2-dimensional Convolution (continued)

```
      if( NULL == imga ) return 0;
      iplSetBorderMode( imga, IPL_BORDER_REFLECT, IPL_SIDE_TOP|
         IPL_SIDE_BOTTOM|IPL_SIDE_LEFT|IPL_SIDE_RIGHT, 0);
      imgb = iplCreateImageHeader(
         1, 0, IPL_DEPTH_8U, "GRAY", "GRAY",
         IPL_DATA_ORDER_PIXEL, IPL_ORIGIN_TL,
         IPL_ALIGN_DWORD, 4, 4, NULL, NULL,
         NULL, NULL);
      if( NULL == imgb ) return 0;
      iplAllocateImage( imga, 0, 0 );
      if( NULL == imga->imageData ) return 0;
      // fill image by meaningless
      for( i=0; i<16; i++)
         ((char*)imga->imageData)[i] = (char)(i+1);
      iplAllocateImage( imgb, 0, 0 );
      if( NULL == imgb->imageData ) return 0;
      // create kernel 3x3 with (1,1) cross point
      kernel = iplCreateConvKernel( 3, 3, 1, 1, one, 0 );
      // convolve imga by kernel and place the result in imgb
      iplConvolve2D( imga, imgb, &kernel, 1, IPL_SUM );
      // Check if an error occurred
      if( iplGetErrStatus() != IPL_StsOk ) return 0;
   }
   __finally {
      iplDeleteConvKernel( kernel );
      iplDeallocate( imga, IPL_IMAGE_HEADER | IPL_IMAGE_DATA );
      iplDeallocate( imgb, IPL_IMAGE_HEADER | IPL_IMAGE_DATA );
   }
   return IPL_StsOk == iplGetErrStatus();
}
```

ConvolveSep2D, ConvolveSep2DFP

Convolves an image with a separable convolution kernel.

```
void iplConvolveSep2D (IplImage* srcImage,
   IplImage* dstImage, IplConvKernel* xKernel,
   IplConvKernel* yKernel);
```

```
void iplConvolveSep2DFP (IplImage* srcImage,
   IplImage* dstImage, IplConvKernelFP* xKernel,
   IplConvKernelFP* yKernel);
```

srcImage	The source image.
dstImage	The resultant image.
xKernel	The *x* or row kernel. Must contain only one row.
yKernel	The *y* or column kernel. Must contain only one column.

Discussion

The functions `iplConvolveSep2D()` and `iplConvolveSep2DFP()` are used to convolve the input image *srcImage* with the separable kernel specified by the row kernel *xKernel* and column kernel *yKernel*. The functions write the convolution results to the output image *dstImage*.

Use `iplConvolveSep2DFP()` only for images with 32-bit floating-point data. For all other image data types, use `iplConvolveSep2D()`.

One of the kernel arguments *xKernel* or *yKernel* (but not both) can be `NULL`, for example:

```
iplConvolveSep2DFP (src, dst, xKernel, NULL);
iplConvolveSep2DFP (src, dst, NULL, yKernel);
```

FixedFilter

Convolves an image with a predefined kernel.

```
int iplFixedFilter(IplImage* srcImage,
    IplImage* dstImage, IplFilter filter);
```

srcImage	The source image.
dstImage	The resultant image.
filter	One of predefined filter kernels (see *Discussion* for supported filters).

Discussion

The function `iplFixedFilter()` is used to convolve the input image *srcImage* with a predefined filter kernel specified by *filter*. The resulting output image is *dstImage*.

The *filter* kernel can be one of the following:

`IPL_PREWITT_3x3_V` A gradient filter (vertical Prewitt operator). This filter uses the kernel

```
-1  0  1
-1  0  1
-1  0  1
```

`IPL_PREWITT_3x3_H` A gradient filter (horizontal Prewitt operator). This filter uses the kernel

```
 1  1  1
 0  0  0
-1 -1 -1
```

`IPL_SOBEL_3x3_V` A gradient filter (vertical Sobel operator). This filter uses the kernel

```
-1  0  1
-2  0  2
-1  0  1
```

`IPL_SOBEL_3x3_H` A gradient filter (horizontal Sobel operator). This filter uses the kernel

```
   1  2  1
   0  0  0
  -1 -2 -1
```

`IPL_LAPLACIAN_3x3` A 3x3 Laplacian highpass filter. This filter uses the kernel

```
  -1 -1 -1
  -1  8 -1
  -1 -1 -1
```

`IPL_LAPLACIAN_5x5` A 5x5 Laplacian highpass filter. This filter uses the kernel

```
  -1 -3 -4 -3 -1
  -3  0  6  0 -3
  -4  6 20  6 -4
  -3  0  6  0 -3
  -1 -3 -4 -3 -1
```

`IPL_GAUSSIAN_3x3` A 3x3 Gaussian lowpass filter. This filter uses the kernel `A/16`, where

```
        1  2  1
   A =  2  4  2
        1  2  1
```

These filter coefficients correspond to a 2-dimensional Gaussian distribution with standard deviation 0.85.

`IPL_GAUSSIAN_5x5` A 5x5 Gaussian lowpass filter. This filter uses the kernel `A/571`, where

```
         2  7  12  7  2
         7 31  52 31  7
  A =   12 52 127 52 12
         7 31  52 31  7
         2  7  12  7  2
```

These filter coefficients correspond to a 2-dimensional Gaussian distribution with standard deviation 1.0.

`IPL_HIPASS_3x3` A 3x3 highpass filter.
This filter uses the kernel

```
-1 -1 -1
-1  8 -1
-1 -1 -1
```

`IPL_HIPASS_5x5` A 5x5 highpass filter.
This filter uses the kernel

```
-1 -1 -1 -1 -1
-1 -1 -1 -1 -1
-1 -1 24 -1 -1
-1 -1 -1 -1 -1
-1 -1 -1 -1 -1
```

`IPL_SHARPEN_3x3` A 3x3 sharpening filter.
This filter uses the kernel

```
        -1 -1 -1
(1/8) * -1 16 -1
        -1 -1 -1
```

Return Value

The function returns zero if the execution is completed successfully, and a non-zero integer if an error occurred.

Non-linear Filters

Non-linear filtering involves performing non-linear operations on some neighborhood of the image. Most common are the minimum, maximum and median filters.

MedianFilter

Apply a median filter to the image.

```
void iplMedianFilter(IplImage* srcImage, IplImage*
dstImage, int nCols, int nRows, int anchorX,
int anchorY);
```

`srcImage`	The source image.
`dstImage`	The resultant image.
`nCols`	Number of columns in the neighborhood to use.
`nRows`	Number of rows in the neighborhood to use.
`anchorX, anchorY`	The [*x*, *y*] coordinates of the anchor cell in the neighborhood. In this coordinate system, the top left corner would be [0, 0] and the bottom right corner would be [`nCols-1`, `nRows-1`]. For a 3 by 3 neighborhood, the coordinates of the geometric center would be [1, 1]. This specification allows the neighborhood to be skewed with respect to its geometric center.

Discussion

The function `iplMedianFilter()` sets each pixel in the output image as the median value of all the input image pixel values in the neighborhood of size `nRows` by `nCols` with the anchor cell at that pixel. This has the effect of removing the noise in the image.

Example 6-2 Applying the Median Filter

```
int example62( void ) {
   IplImage *imga, *imgb;
   __try {
      imga = iplCreateImageHeader(
         1, 0, IPL_DEPTH_8U, "GRAY", "GRAY",
         IPL_DATA_ORDER_PIXEL, IPL_ORIGIN_TL,
         IPL_ALIGN_DWORD, 4, 4, NULL, NULL,
         NULL, NULL);
      if( NULL == imga ) return 0;
      iplSetBorderMode( imga, IPL_BORDER_REFLECT, IPL_SIDE_TOP|
         IPL_SIDE_BOTTOM|IPL_SIDE_LEFT|IPL_SIDE_RIGHT, 0);
      imgb = iplCreateImageHeader(
         1, 0, IPL_DEPTH_8U, "GRAY", "GRAY",
         IPL_DATA_ORDER_PIXEL, IPL_ORIGIN_TL,
         IPL_ALIGN_DWORD, 4, 4, NULL, NULL,
         NULL, NULL);
      if( NULL == imgb ) return 0;
      iplAllocateImage( imga, 1, 10 );
      if( NULL == imga->imageData ) return 0;
      // make a spike
      ((char*)imga->imageData)[2*4+2] = (char)15;
      iplAllocateImage( imgb, 0, 0 );
      if( NULL == imgb->imageData ) return 0;
      // Filter imga and place the result in imgb
      iplMedianFilter( imga, imgb, 3,3, 1,1 );
      if( iplGetErrStatus() != IPL_StsOk ) return 0;
   }
   __finally {
      iplDeallocate( imga, IPL_IMAGE_HEADER | IPL_IMAGE_DATA );
      iplDeallocate( imgb, IPL_IMAGE_HEADER | IPL_IMAGE_DATA );
   }
   return IPL_StsOk == iplGetErrStatus();
}
```

ColorMedianFilter

Apply a color median filter to the image.

```
void iplColorMedianFilter(IplImage* srcImage, IplImage*
dstImage, int nCols, int nRows, int anchorX, int anchorY);
```

`srcImage`	The source image.
`dstImage`	The resultant image.
`nCols`	Number of columns in the neighborhood to use.
`nRows`	Number of rows in the neighborhood to use.
`anchorX, anchorY`	The [*x*, *y*] coordinates of the anchor cell in the neighborhood.

Discussion

The previously described function `iplMedianFilter()` processes R, G, and B color planes of an image separately, and as a result any correlation between color components is lost. If you want to preserve this information, use the `iplColorMedianFilter()` function instead. For each input pixel, this function computes differences between red, green, and blue components of pixels in the neighborhood area of size *`nRows`* by *`nCols`* and the input pixel. The 'distance' between the input pixel *i* and the neighborhood pixel *j* is formed as sum of absolute values

abs (R(i)-R(j)) + abs (G(i)-G(j)) + abs (B(i)-B(j)) .

After scanning all neighborhood area, the function sets the output value for pixel *i* as the value of the neighborhood pixel with the smallest distance to *i*.

The function `iplColorMedianFilter()` supports color images with or without alpha channel.

MaxFilter

Apply a max filter to the image.

```
void iplMaxFilter(IplImage* srcImage, IplImage* dstImage,
int nCols, int nRows, int anchorX, int anchorY);
```

`srcImage`	The source image.
`dstImage`	The resultant image.
`nCols`	Number of columns in the neighborhood to use.
`nRows`	Number of rows in the neighborhood to use.
`anchorX, anchorY`	The [*x*, *y*] coordinates of the anchor cell in the neighborhood. In this coordinate system, the top left corner would be [0, 0] and the bottom right corner would be [`nCols`-1, `nRows`-1]. For a 3 by 3 neighborhood, the coordinates of the geometric center would be [1, 1]. This specification allows the neighborhood to be skewed with respect to its geometric center.

Discussion

The function `iplMaxFilter()` sets each pixel in the output image as the maximum value of all the input image pixel values in the neighborhood of size `nRows` by `nCols` with the anchor cell at that pixel. This has the effect of increasing the contrast in the image.

MinFilter

Apply a min filter to the image.

```
void iplMinFilter(IplImage* srcImage, IplImage* dstImage,
int nCols, int nRows, int anchorX, int anchorY);
```

`srcImage`	The source image.
`dstImage`	The resultant image.
`nCols`	Number of columns in the neighborhood to use.
`nRows`	Number of rows in the neighborhood to use.
`anchorX, anchorY`	The [*x*, *y*] coordinates of the anchor cell in the neighborhood. (In this coordinate system, the top left corner would be [0, 0] and the bottom right corner would be [`nCols-1`, `nRows-1`]. For a 3 by 3 neighborhood the coordinates of the geometric center would be [1, 1]). This specification allows the neighborhood to be skewed with respect to its geometric center.

Discussion

The function `iplMinFilter()` sets each pixel in the output image as the minimum value of all the input image pixel values in the neighborhood of size `nRows` by `nCols` with the anchor cell at that pixel. This has the effect of decreasing the contrast in the image.

Linear Image Transforms

7

This chapter describes the linear image transforms implemented in the library: Fast Fourier Transform (FFT) and Discrete Cosine Transform (DCT). Table 7-1 lists the functions performing linear image transform operations.

Table 7-1 Linear Image Transform Functions

Group	Function Name	Description
Fast Fourier Transform (FFT)	`iplRealFft2D`	Computes the forward or inverse 2D FFT of an image.
	`iplCcsFft2D`	Computes the forward or inverse 2D FFT of an image in a complex-conjugate format.
	`iplMpyRCPack2D`	Multiplies data in the RCPack format.
Discrete Cosine Transform (DCT)	`iplDCT2D`	Computes the forward or inverse 2D DCT of an image.

Fast Fourier Transform

This section describes the functions that implement the forward and inverse Fast Fourier Transform (FFT) on the 2-dimensional (2D) image data.

Real-Complex Packed (RCPack2D) Format

The FFT of any real 2D signal, in particular, the FFT of an image is conjugate-symmetric. Therefore, it can be fully specified by storing only half the output data. A special format called `RCPack2D` is provided for this purpose.

The function `iplRealFft2D()` transforms a 2D image and produces the Fourier coefficients in the `RCPack2D` format. To complement this, function `iplCcsFft2D()` is provided that uses its input in `RCPack2D` format, performs the Fourier transform, and produces its output as a real 2D image. The functions `iplRealFft2D()` and `iplCcsFft2D()` together can be used to perform frequency domain filtering of images.

`RCPack2D` format is defined based on the following Fourier transform equations:

$$A_{s,j} = \sum_{l=0}^{L-1} \sum_{k=0}^{K-1} f_{k,l} \exp\left(-\frac{2\pi ijl}{L}\right) \exp\left(-\frac{2\pi iks}{K}\right)$$

$$f_{k,l} = \frac{1}{LK} \sum_{j=0}^{L-1} \sum_{s=0}^{K-1} A_{s,j} \exp\left(\frac{2\pi ijl}{L}\right) \exp\left(\frac{2\pi iks}{K}\right)$$

where $i = \sqrt{-1}$, $f_{k,l}$ is the pixel value in the *k*-th row and *l*-th column.

Note that the Fourier coefficients have the following relationship:

$A_{s,j} = \text{conj}(A_{K-s,\ L-j})$ $s = 1, \ldots, K-1;\ j = 1, \ldots, L-1;$

$A_{0,j} = \text{conj}(A_{0,\ L-j})$ $j = 1, \ldots, L-1;$

$A_{s,0} = \text{conj}(A_{K-s,\ 0})$ $s = 1, \ldots, K-1.$

Hence, to reconstruct the $L*K$ complex coefficients $A_{s,j}$, it is enough to store only $L*K$ real values. The Fourier transform functions actually use $s = 0, \ldots, K-1;\ j = 0, \ldots, L/2.$

Other Fourier coefficients can be found using complex-conjugate relations. Fourier coefficients $A_{s,j}$ can be stored in the `RCPack2D` format, which is a convenient compact representation of a complex conjugate-symmetric sequence. In the `RCPack2D` format, the output samples of the FFT are arranged as shown in Tables 7-2 and 7-3, where Re corresponds to Real and Im corresponds to Imaginary. Table 7-4 is an example of output samples storage for $K = 4$ and $L = 4$.

Table 7-2 FFT Output in RCPack2D Format for Even *K*

Re $A_{0,0}$	Re $A_{0,1}$	Im $A_{0,1}$	. . .	Re $A_{0,(L-1)/2}$	Im $A_{0,(L-1)/2}$	Re $A_{0,L/2}$
Re $A_{1,0}$	Re $A_{1,1}$	Im $A_{1,1}$	. . .	Re $A_{1,(L-1)/2}$	Im $A_{1,(L-1)/2}$	Re $A_{1,L/2}$
Im $A_{1,0}$	Re $A_{2,1}$	Im $A_{2,1}$	. . .	Re $A_{2,(L-1)/2}$	Im $A_{2,(L-1)/2}$	Im $A_{1,L/2}$
. . .	. . .	. . .	. . .	. . .	. . .	. . .
Re $A_{K/2-1,0}$	Re $A_{K-3,1}$	Im $A_{K-3,1}$	. . .	Re $A_{K-3,(L-1)/2}$	Im $A_{K-3,(L-1)/2}$	Re $A_{K/2-1,L/2}$
Im $A_{K/2-1,0}$	Re $A_{K-2,1}$	Im $A_{K-2,1}$	. . .	Re $A_{K-2,(L-1)/2}$	Im $A_{K-2,(L-1)/2}$	Im $A_{K/2-1,L/2}$
Re $A_{K/2,0}$	Re $A_{K-1,1}$	Im $A_{K-1,1}$	. . .	Re $A_{K-1,(L-1)/2}$	Im $A_{K-1,(L-1)/2}$	Re $A_{K/2,L/2}$

(the last column is used for even *L* only)

Table 7-3 FFT Output in RCPack2D Format for Odd *K*

Re $A_{0,0}$	Re $A_{0,1}$	Im $A_{0,1}$	. . .	Re $A_{0,(L-1)/2}$	Im $A_{0,(L-1)/2}$	Re $A_{0,L/2}$
Re $A_{1,0}$	Re $A_{1,1}$	Im $A_{1,1}$	. . .	Re $A_{1,(L-1)/2}$	Im $A_{1,(L-1)/2}$	Re $A_{1,L/2}$
Im $A_{1,0}$	Re $A_{2,1}$	Im $A_{2,1}$	. . .	Re $A_{2,(L-1)/2}$	Im $A_{2,(L-1)/2}$	Im $A_{1,L/2}$
. . .	. . .	. . .	. . .	. . .	. . .	. . .
Re $A_{K/2,0}$	Re $A_{K-2,1}$	Im $A_{K-2,1}$	. . .	Re $A_{K-2,(L-1)/2}$	Im $A_{K-2,(L-1)/2}$	Re $A_{K/2,L/2}$
Im $A_{K/2,0}$	Re $A_{K-1,1}$	Im $A_{K-1,1}$	. . .	Re $A_{K-1,(L-1)/2}$	Im $A_{K-1,(L-1)/2}$	Im $A_{K/2,L/2}$

(the last column is used for even *L* only)

Table 7-4 RealFFT2D Output Sample for *K* = 4, *L* = 4

Re $A_{0,0}$	Re $A_{0,1}$	Im $A_{0,1}$	Re $A_{0,2}$
Re $A_{1,0}$	Re $A_{1,1}$	Im $A_{1,1}$	Re $A_{1,2}$
Im $A_{1,0}$	Re $A_{2,1}$	Im $A_{2,1}$	Im $A_{1,2}$
Re $A_{2,0}$	Re $A_{3,1}$	Im $A_{3,1}$	Re $A_{2,2}$

RealFft2D

Computes the forward or inverse 2D FFT of an image.

```
void iplRealFft2D(IplImage* srcImage, IplImage* dstImage,
                  int flags);
```

`srcImage`	The source image.
`dstImage`	The resultant image in `RCPack2D` format containing the Fourier coefficients. This image must be a multi-channel image containing the same number of channels as *`srcImage`*. The data type for the image must be 8, 16 or 32 bits.<br><br>This image cannot be the same as the input image *`srcImage`* (that is, an in-place operation is not allowed).
`flags`	Specifies how to perform the FFT. This is an integer whose bits can be assigned the following values using bitwise logical `OR`:

`IPL_FFT_Forw`	Do forward transform
`IPL_FFT_Inv`	Do inverse transform
`IPL_FFT_NoScale`	Do inverse transform without scaling
`IPL_FFT_UseInt`	Use only integer core
`IPL_FFT_UseFloat`	Use only float core
`IPL_FFT_Free`	Only free all working arrays and exit.

Discussion

The function `iplRealFft2D()` performs an FFT on each channel in the specified rectangular ROI of the input image *`srcImage`* and writes the Fourier coefficients in `RCPack2D` format into the corresponding channel of the output image *`dstImage`*. The output data will be clamped (saturated) to the limits `Min` and `Max`, which are determined by the data type of the output image. For best results, use 32-bit data or, at least, 16-bit data.

Example 7-1 Computing the FFT of an Image

```
/*-----------------------------------------------
; Matlab example
» rand('seed',12345); x=round(rand(4,4)*10), fft2(x)
  89        10 - 7i   -9        10 + 7i
  -1 + 6i    8 -21i   13 + 2i  -8 - 3i
  -3        10 + 1i    3        10 - 1i
  -1 - 6i   -8 + 3i   13 - 2i   8 +21i
// Result of iplRealFft2D function:
  89      10      -7      -9
  -1       8     -21      13
   6      10       1       2
  -3      -8       3       3
-----------------------------------------------*/
int example71( void ) {
   IplImage *imga, *imgb; int i;
   const int src[16] = {
     9,     7,     4,     1,     7,     5,     1,     7,
     6,     6,     1,     9,     3,    10,     9,     4};
   __try {
      imga = iplCreateImageHeader(
         1, 0, IPL_DEPTH_8U, "GRAY", "GRAY",
         IPL_DATA_ORDER_PIXEL, IPL_ORIGIN_TL,
         IPL_ALIGN_DWORD, 4, 4, NULL, NULL,
         NULL, NULL);
```

continued ☞

Example 7-1 Computing the FFT of an Image (continued)

```
        if( NULL == imga ) return 0;
        imgb = iplCreateImageHeader(
           1, 0, IPL_DEPTH_16S, "GRAY", "GRAY",
           IPL_DATA_ORDER_PIXEL, IPL_ORIGIN_TL,
           IPL_ALIGN_DWORD, 4, 4, NULL, NULL,
           NULL, NULL);
        if( NULL == imgb ) return 0;

        // Create without filling
        iplAllocateImage( imga, 0,0 );
        if( NULL == imga->imageData ) return 0;
        // Fill by sample data
        for( i=0; i<16; i++)
           ((char*)imga->imageData)[i] = (char)src[i];

        iplAllocateImage( imgb, 0, 0 );
        if( NULL == imgb->imageData ) return 0;

        iplRealFft2D( imga, imgb, IPL_FFT_Forw );
        // Compare Matlab and ipl result here
        iplCcsFft2D( imgb, imga, IPL_FFT_Inv );
        // Compare source data and obtained data

        // Check if an error was occured
        if( iplGetErrStatus() != IPL_StsOk ) return 0;
     }
     __finally {
       iplRealFft2D( NULL, NULL, IPL_FFT_Free );
       iplDeallocate(imga,IPL_IMAGE_HEADER|IPL_IMAGE_DATA);
       iplDeallocate(imgb,IPL_IMAGE_HEADER|IPL_IMAGE_DATA);
     }
     return IPL_StsOk == iplGetErrStatus();
  }
```

CcsFft2D

Computes the forward or inverse 2D FFT of an image in complex-conjugate format.

```
void iplCcsFft2D(IplImage* srcImage, IplImage* dstImage,
                 int flags);
```

srcImage	The source image in RCPack2D format.
dstImage	The resultant image. This image must be a multi-channel image containing the same number of channels as *srcImage*. This image cannot be the same as the input image *srcImage* (that is, an in-place operation is not allowed).
flags	Specifies how to perform the FFT. This is an integer whose bits can be assigned the following values using bitwise logical OR:

IPL_FFT_Forw	Do forward transform.
IPL_FFT_Inv	Do inverse transform.
IPL_FFT_NoScale	Do inverse transform without scaling.
IPL_FFT_UseInt	Use only integer core.
IPL_FFT_UseFloat	Use only float core.
IPL_FFT_Free	Only free all working arrays and exit.

Discussion

The function iplCcsFft2D() performs an FFT on each channel in the specified rectangle ROI of the input image *srcImage* and writes the output in RCPack2D format to the image *dstImage*. The output data will be clamped (saturated) to the limits Min and Max that are determined by the data type of the output image.

MpyRCPack2D

Multiplies data of two images in the RCPack format.

```
void iplMpyRCPack2D (IplImage* srcA, IplImage* srcB,
IplImage* dst);
```

srcA, srcB	The source images in `RCPack2D` format.
dst	The resultant image. This image must be a multi-channel image containing the same number of channels as *srcA* and *srcB*.<br>This image cannot be the same as the input images (that is, an in-place operation is not allowed) .

Discussion

The function `iplMpyRCPack2D()` multiplies the data of the image *srcA* by that of *srcB* and writes the result to *dst*. All images are assumed to be in the RCPack format, the format for storing the results of forward FFTs. Thus, this function multiplies the data in "frequency domain". (This corresponds to cyclic convolution in the original data domain.)

Discrete Cosine Transform

This section describes the functions that implement the forward and inverse Discrete Cosine Transform (DCT) on the 2D image data. The output of the DCT for real input data is real. Therefore, unlike FFT, no special format for the transform output is needed.

DCT2D

Computes the forward or inverse 2D DCT of an image.

```
void iplDCT2D(IplImage* srcImage, IplImage* dstImage,
int flags);
```

`srcImage` The source image.

`dstImage` The resultant image containing the DCT coefficients. This image must be a multi-channel image containing the same number of channels as `srcImage`. The data type for the image must be 8, 16 or 32 bits.

This image cannot be the same as the input image `srcImage` (that is, an in-place operation is not allowed).

`flags` Specifies how to perform the DCT. This is an integer whose bits can be assigned the following values using bitwise logical `OR`:

`IPL_DCT_Forward` Do forward transform.

`IPL_DCT_Inverse` Do inverse transform.

`IPL_DCT_Free` Only free all working arrays and exit.

`IPL_DCT_UseInpBuf`
Use the input image array for the intermediate calculations. The performance of DCT increases, but the input image is destroyed. You may use this value only if both the source and destination image data types are 16-bit signed.

Discussion

The function `iplDCT2D()` performs a DCT on each channel in the specified rectangular ROI of the input image *`srcImage`* and writes the DCT coefficients into the corresponding channel of the output image *`dstImage`*. The output data will be clamped (saturated) to the limits `Min` and `Max`, where `Min` and `Max` are determined by the data type of the output image. For best results, use 32-bit data or, at least, 16-bit data.

Example 7-2 Computing the DCT of an Image

```
int example72( void ) {
   IplImage *imga, *imgb;
   const int width = 8, height = 8;
   int i, x, y;

   __try {
      imga = iplCreateImageHeader(
         1, 0, IPL_DEPTH_8U, "GRAY", "GRAY",
         IPL_DATA_ORDER_PIXEL, IPL_ORIGIN_TL,
         IPL_ALIGN_DWORD, width, height, NULL, NULL,
         NULL, NULL);
      if( NULL == imga ) return 0;

      imgb = iplCreateImageHeader(
         1, 0, IPL_DEPTH_16S, "GRAY", "GRAY",
         IPL_DATA_ORDER_PIXEL, IPL_ORIGIN_TL,
         IPL_ALIGN_DWORD, width, height, NULL, NULL,
         NULL, NULL);

      if( NULL == imgb ) return 0;
```

continued ☞

Example 7-2 Computing the DCT of an Image (continued)

```
        // Create without filling
        iplAllocateImage( imga, 0,0 );
        if( NULL == imga->imageData ) return 0;

        // Fill by sample data
        for( i=0; i<width*height; i++)
           ((char*)imga->imageData)[i] = (char)(i+1);
        iplAllocateImage( imgb, 0, 0 );
        if( NULL == imgb->imageData ) return 0;

        iplDCT2D( imga, imgb, IPL_DCT_Forward );

        // Now there are (width+height-1) DCT coefficients
        for( y=1; y<height; y++)
          for( x=1; x<width; x++)
            ((short*)imgb->imageData)[y*width+x]= (short)0;

        // Restore source image from some DCT coefficients
        iplDCT2D( imgb, imga, IPL_DCT_Inverse );

        // Check if an error occurred
        if( iplGetErrStatus() != IPL_StsOk ) return 0;
     }
     __finally {
       iplDCT2D( NULL, NULL, IPL_DCT_Free );
       iplDeallocate(imga,IPL_IMAGE_HEADER|IPL_IMAGE_DATA);
       iplDeallocate(imgb,IPL_IMAGE_HEADER|IPL_IMAGE_DATA);
     }
     return IPL_StsOk == iplGetErrStatus();
  }
```

This page is left blank for double-sided printing

This page is left blank for double-sided printing

Morphological Operations 8

The morphological operations of the Image Processing Library are simple erosion and dilation of an image. A specified number of erosions and dilations are performed as part of image opening or closing operations in order to (respectively) eliminate or fill small and thin holes in objects, break objects at thin points or connect nearby objects, and generally smooth the boundaries of objects without significantly changing their area.

Table 8-1 lists the functions that perform these operations.

Table 8-1 Morphological Operation Functions

Group	Function Name	Description
Erode, Dilate	`iplErode`	Erodes the image an indicated number of times.
	`iplDilate`	Dilates the image an indicated number of times.
Open, Close	`iplOpen`	Opens the image while smoothing the boundaries of large objects.
	`iplClose`	Closes the image while smoothing the boundaries of large objects.

Erode

Erodes the image.

```
void iplErode(IplImage* srcImage, IplImage* dstImage,
              int nIterations);
```

srcImage	The source image.
dstImage	The resultant image.
nIterations	The number of times to erode the image.

Discussion

The function `iplErode()` performs an erosion of the image *nIterations* times. The way the image is eroded depends on whether it is a binary image, a gray-scale image, or a color image.

- For a binary input image, the output pixel is set to zero if the corresponding input pixel or any of its 8 neighboring pixels is a zero.
- For a gray scale or color image, the output pixel is set to the minimum of the corresponding input pixel and its 8 neighboring pixels.
- For a color image, each color channel in the output pixel is set to the minimum of this channel's values at the corresponding input pixel and its 8 neighboring pixels.

The effect of erosion is to remove spurious pixels (such as noise) and to thin boundaries of objects on a dark background (that is, objects whose pixel values are greater than those of the background).

Figure 8-1 shows an example of 8-bit gray scale image before erosion (left) and the same image after erosion of a rectangular ROI (right).

Figure 8-1 Erosion in a Rectangular ROI: the Source (left) and Result (right)

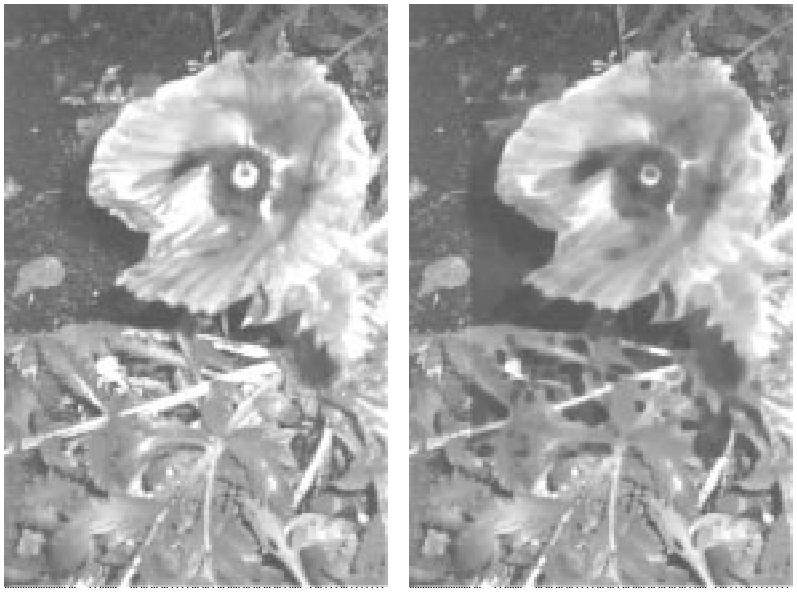

The following code (Example 8-1) performs erosion of the image inside the selected rectangular ROI.

Example 8-1 Code Used to Produce Erosion in a Rectangular ROI

```
int example81( void ) {  IplImage *imga, *imgb;
   __try {
      imga = iplCreateImageHeader(
         1, 0, IPL_DEPTH_8U, "GRAY", "GRAY",
         IPL_DATA_ORDER_PIXEL, IPL_ORIGIN_TL,
         IPL_ALIGN_DWORD, 4, 4, NULL, NULL,
         NULL, NULL);
      if( NULL == imga ) return 0;
      imgb = iplCreateImageHeader(
         1, 0, IPL_DEPTH_8U, "GRAY", "GRAY",
         IPL_DATA_ORDER_PIXEL, IPL_ORIGIN_TL,
         IPL_ALIGN_DWORD, 4, 4, NULL, NULL,
         NULL, NULL);
      if( NULL == imgb ) return 0;
      iplAllocateImage( imga, 1, 7 );
      if( NULL == imga->imageData ) return 0;
      // Create a hole
      ((char*)imga->imageData)[2*4+2] = 0;
      // Border is taken from the opposite side
      iplSetBorderMode( imga, IPL_BORDER_WRAP,
                        IPL_SIDE_ALL, 0 );
      iplAllocateImage( imgb, 0, 0 );
      if( NULL == imgb->imageData ) return 0;
      // Erosion will increase the hole
      iplErode( imga, imgb, 1 );
      // Check if an error occurred
      if( iplGetErrStatus() != IPL_StsOk ) return 0;
   }
   __finally {
      iplDeallocate(imga,IPL_IMAGE_HEADER|IPL_IMAGE_DATA);
      iplDeallocate(imgb,IPL_IMAGE_HEADER|IPL_IMAGE_DATA);
   }
   return IPL_StsOk == iplGetErrStatus();
}
```

NOTE. *All source image attributes are defined in the image header pointed to by* `srcImage`*.*

Dilate

Dilates the image.

```
void iplDilate(IplImage* srcImage, IplImage* dstImage, int
nIterations);
```

`srcImage`	The source image.
`dstImage`	The resultant image.
`nIterations`	The number of times to dilate the image.

Discussion

The function `iplDilate()` performs a dilation of the image `nIterations` times. The way the image is dilated depends on whether the image is binary, gray-scale, or a color image.

- For a binary input image, the output pixel is set to 1 if the corresponding input pixel is 1 or any of 8 neighboring input pixels is 1.
- For a gray-scale image, the output pixel is set to the maximum of the corresponding input pixel and its 8 neighboring pixels.
- For a color image, each color channel in the output pixel is set to the maximum of this channel's values at the corresponding input pixel and its 8 neighboring pixels.

The effect of dilation is to fill up holes and to thicken boundaries of objects on a dark background (that is, objects whose pixel values are greater than those of the background).

Open

Opens the image by performing erosions followed by dilations.

```
void iplOpen(IplImage* srcImage, IplImage* dstImage,
                int nIterations);
```

`srcImage`	The source image.
`dstImage`	The resultant image.
`nIterations`	The number of times to erode and dilate the image.

Discussion

The function `iplOpen()` performs `nIterations` of erosion followed by `nIterations` of dilation performed by `iplErode()` and `iplDilate()`, respectively.

The process of opening has the effect of eliminating small and thin objects, breaking objects at thin points, and generally smoothing the boundaries of larger objects without significantly changing their area.

See Also

Erode

Dilate

Close

Closes the image by performing dilations followed by erosions.

```
void iplClose(IplImage* srcImage, IplImage* dstImage,
int nIterations);
```

`srcImage`	The source image.
`dstImage`	The resultant image.
`nIterations`	The number of times to dilate and erode the image.

Discussion

The function `iplClose()` performs *`nIterations`* of dilation followed by *`nIterations`* of erosion performed by `iplDilate()` and `iplErode()`, respectively.

The process of closing has the effect of filling small and thin holes in objects, connecting nearby objects, and generally smoothing the boundaries of objects without significantly changing their area.

See Also

Erode

Dilate

This page is left blank for double-sided printing

This page is left blank for double-sided printing

Color Space Conversion

9

This chapter describes the Image Processing Library functions that perform color space conversion. The library supports the following color space conversions:

- Reduction from high bit resolution color to low bit resolution color
- Conversion of absolute color images to and from palette color images
- Color model conversion
- Conversion from color to gray scale and vice versa

Table 9-1 lists color space conversion functions. For information on the absolute-to-palette and palette-to-absolute color conversion, see “Working in the Windows DIB Environment” in Chapter 4.

Table 9-1 Color Space Conversion Functions

Conversion Type	Function Name	Description
Reducing Bit Resolution	`iplReduceBits`	Reduces the number of bits per channel in an image.
Bitonal to gray scale	`iplBitonalToGray`	Converts bitonal images to 8- and 16-bit gray scale images.
Color to gray scale and vice versa	`iplColorToGray`<br>`iplGrayToColor`	Convert color images to and from gray scale images.
Color Models Conversion	`iplRGB2HSV`, `iplHSV2RGB`	Convert RGB images to and from HSV color model.
	`iplRGB2HLS`, `iplHLS2RGB`	Convert RGB images to and from HLS color model.

continued ☞

Table 9-1 Color Space Conversion Functions (continued)

Conversion Type	Function Name	Description
Color Models Conversion (continued)	iplRGB2LUV, iplLUV2RGB	Convert RGB images to and from LUV color model.
	iplRGB2XYZ, iplXYZ2RGB	Convert RGB images to and from XYZ color model.
	iplRGB2YCrCb, iplYCrCb2RGB	Convert RGB images to and from YC_rC_b color model.
	iplRGB2YUV, iplYUV2RGB	Convert RGB images to and from YUV color model.
	iplYCC2RGB	Convert PhotoYCC* images to RGB color model.
Color Twist	iplApplyColorTwist	Applies a color-twist matrix to an image.
	iplCreateColorTwist	Allocates memory for color-twist matrix data structure.
	iplDeleteColorTwist	Deletes the color-twist matrix data structure.
	iplSetColorTwist	Sets a color-twist matrix data structure.
	iplColorTwistFP	Applies a color-twist matrix to an image with floating-point pixel values.

Reducing the Image Bit Resolution

This section describes functions that reduce the bit resolution of absolute color and gray scale images.

ReduceBits

Reduces the number of intensity levels in an image.

```
void iplReduceBits(IplImage* srcImage, IplImage* dstImage,
   int noise, int ditherType, int levels);
```

srcImage The source image .

dstImage The resultant image.

noise The number specifying the noise added. This parameter is set as a percentage of range [0..100].

ditherType The type of dithering to be used. The following types are supported:

IPL_DITHER_NONE	No dithering is done
IPL_DITHER_FS	The Floid-Steinberg error diffusion dithering algorithm
IPL_DITHER_JJH	The Jarvice-Judice-Ninke error diffusion dithering algorithm
IPL_DITHER_STUCKEY	The Stucki error diffusion dithering algorithm

	`IPL_DITHER_BAYER` The Bayer threshold dithering algorithm.
levels	The number of output levels for halftoning (dithering); can be varied in the range `[2..(1<<`*depth*`)]`, where *depth* is the bit depth of the destination image.

Discussion

The function `iplReduceBits()` reduces the number of intensity levels in each channel of the source image *srcImage* and places the results in respective channels of the destination image *dstImage*.
The *levels* parameter sets the resultant number of intensity levels in each channel of the destination image.
If the *noise* value is greater than 0, some random noise is added to the threshold level used in computations; see [Schumacher]. The amplitude of the noise signal is specified by the *noise* parameter set as a percentage of the destination image luminance range. For the 4x4 ordered dithering mode (see [Bayer]) the threshold value is determined by the dither matrix used, whereas for the error diffusion dithering mode the input threshold is set as half of the *range* value, where

range = ((1<<depth) - 1)/(levels – 1)

and *depth* is the bit depth of the source image.

The figure below illustrates the results of applying the `iplReduceBits()` function with Stucki dithering to a source image that has 256 intensity levels. The output images both have 2 intensity levels, the difference is in the value of noise added for the error diffusion dithering algorithm.

Figure 9-1 Example of the source and resultant images for the bit reducing function

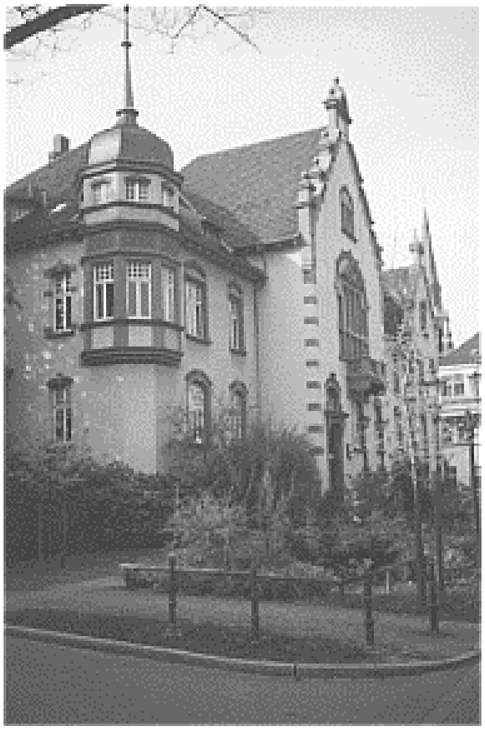

Source image with 256
intensity levels

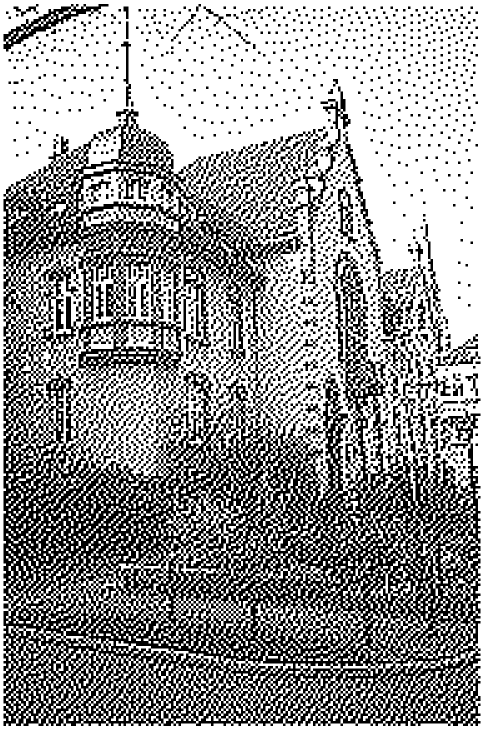

Output image
(`levels`=2, `noise`=0)

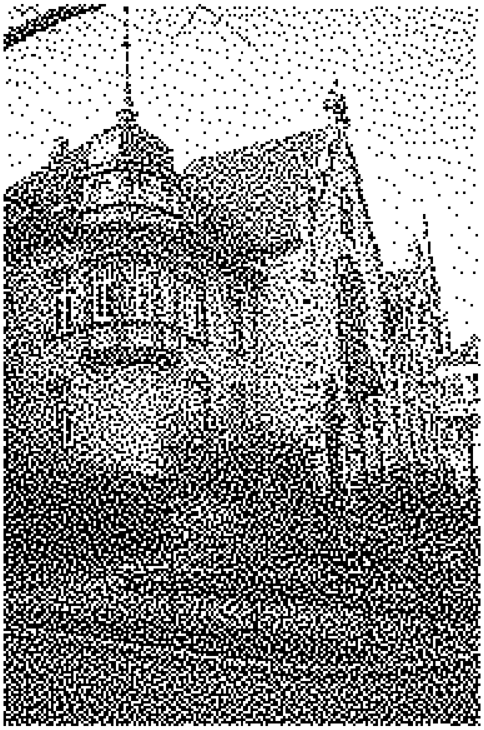

Output image
(`levels`=2, `noise`=20)

Table 9-2 lists the valid combinations of the source and resultant image bit data types for reducing the bit resolution.

Table 9-2 Source and Resultant Image Data Types for Reducing the Bit Resolution

Source Image	Resultant Image
32 bits per channel	32s, 16u, 8u, 1u (1u for Gray only) bits per channel
16 bits per channel	16u, 8u, 1u (1u for Gray only) bits per channel
8 bits per channel	8u, 1u (1u for Gray only) bits per channel

Conversion from Bitonal to Gray Scale Images

This section describes the function that performs the conversion of bitonal images to gray scale.

BitonalToGray

Converts a bitonal image to gray scale.

```
void iplBitonalToGray(IplImage* srcImage, IplImage*
dstImage, int ZeroScale, int OneScale);
```

srcImage	The bitonal source image.
dstImage	The resultant gray scale image. (See the discussion below.)
ZeroScale	The value that zero pixels of the source image should have in the resultant image.
OneScale	The value given to a resultant pixel if the corresponding input pixel is 1.

Discussion

The function `iplBitonalToGray()` converts the input 1-bit bitonal image *srcImage* to an 8s, 8u, 16s or16u gray scale image *dstImage*.

If an input pixel is 0, the corresponding output pixel is set to *ZeroScale*. If an input pixel is 1, the corresponding output pixel is set to *OneScale*.

Conversion of Absolute Colors to and from Palette Colors

Since the `IplImage` format supports only absolute color images, this functionality is provided only within the context of converting an absolute color image `IplImage` to and from a palette color DIB image. See the section "Working in the Windows DIB Environment" in Chapter 4.

Conversion from Color to Gray Scale

This section describes the function that performs the conversion of absolute color images to gray scale.

ColorToGray

Converts a color image to gray scale.

```
void iplColorToGray(IplImage* srcImage, IplImage*
dstImage);
```

`srcImage` The source image. See Table 9-3 for a list of valid source and resultant image combinations.

`dstImage` The resultant image. See Table 9-3 for a list of valid source and resultant image combinations.

Discussion

The function `iplColorToGray()` converts a color source image `srcImage` to a gray scale resultant image `dstImage`.
Table 9-3 lists the valid combinations of source and resultant image bit data types for conversion from color to gray scale.

Table 9-3 Source and Resultant Image Data Types for Conversion from Color to Gray Scale

Source Image (data type)	Resultant image (data type)
32 bit per channel	Gray scale; 1, 8, or 16 bits per pixel
16 bit per channel	Gray scale; 1, 8, or 16 bits per pixel
8 bit per channel	Gray scale; 1, 8, or 16 bits per pixel

The weights to compute true luminance from linear red, green and blue are these:

Y = 0.212671 * R + 0.715160 * G + 0.072169 * B

Conversion from Gray Scale to Color (Pseudo-color)

This section describes the conversion of gray scale image to pseudo color.

GrayToColor

Converts a gray scale to color image.

```
void iplGrayToColor (IplImage* srcImage, IplImage*
dstImage, float FractR, float FractG, float FractB);
```

srcImage	The source image. See Table 9-4 for a list of valid source and resultant image combinations.
dstImage	The resultant image. See Table 9-4 for a list of valid source and resultant image combinations.
FractR,FractG,FractB	The red, green and blue intensities for image reconstruction. See *Discussion* for a list of valid *FractR*, *FractG*, and *FractB* values.

Discussion

The function `iplGrayToColor()` converts a gray scale source image *srcImage* to a resultant pseudo-color image *dstImage*. Table 9-4 lists the valid combinations of source and resultant image bit data types for conversion from gray scale to color.

Table 9-4 Source and Resultant Image Data Types for Conversion from Gray Scale to Color

Source Image (data type)	Resultant image (data type)
Gray scale 1 bit	8 bit per channel
Gray scale 8 bit	8 bit per channel
Gray scale 16 bit	16 bit per channel
Gray scale 32 bit	32 bit per channel

The equations for chrominance in RGB from luminance `Y` are:

```
R = FractR * Y;        0 <= FractR <= 1
G = FractG * Y;        0 <= FractG <= 1
B = FractB * Y;        0 <= FractB <= 1.
```

If all three values *FractR*, *FractG*, *FractB* are zero, then the default values are used in above equations so that:

```
R = 0.212671 * Y,  G = 0.715160 * Y,  B = 0.072169 * Y.
```

Conversion of Color Models

This section describes the conversion of red-green-blue (RGB) images to and from other common color models: hue-saturation-value model (HSV), hue-lightness-saturation (HLS) model, and a number of others.

As an alternative way of color models conversion (that works only for *some* color models) you can just multiply pixel values by a color twist matrix; see “Color Twist Matrices” section in this chapter.

Note also that conversion of RGB images to and from the cyan-magenta-yellow (CMY) model can be performed by a simple subtraction. You can use the function `iplSubtractS` to accomplish this conversion. For example, with maximum pixel value of 255 for 8-bit unsigned images, the `iplSubtractS()` function is used as follows:

```
iplSubtractS(rgbImage, cmyImage, 255, TRUE)
```

This call converts the RGB image `rgbImage` to the CMY image `cmyImage` by setting each channel in the CMY image as follows:

```
C = 255 - R
M = 255 - G
Y = 255 - B
```

The conversion from CMY to RGB is similar: just switch the RGB and CMY images.

Data ranges in the HLS and HSV Color Models

The ranges of color components in the hue-lightness-saturation (HLS) and hue-saturation-value (HSV) color models are defined as follows:

hue H is in the range 0 to 360
lightness L is in the range 0 to 1
saturation S is in the range 0 to 1
value V is in the range 0 to 1.

In the Image Processing Library, these color components are represented by the following integer values of hue H', lightness L', saturation S', and value V':

$H' = H/2$ for 8-bit unsigned color channels, $H' = H$ otherwise,
$L' = L$*`MAX_VAL`
$S' = S$*`MAX_VAL`
$V' = V$*`MAX_VAL`.

Here

`MAX_VAL` = 255	for 8-bit unsigned color channels,
`MAX_VAL` = 65,535	for 16-bit unsigned color channels,
`MAX_VAL` = 2,147,483,647	for 32-bit signed color channels.

RGB2HSV

Converts RGB images to the HSV color model.

```
void iplRGB2HSV(IplImage* rgbImage, IplImage* hsvImage);
```

`rgbImage`	The source RGB image.
`hsvImage`	The resultant HSV image.

Discussion

The function converts the RGB image `rgbImage` to the HSV image `hsvImage`. The function checks that the input image is an RGB image. The channel sequence and color model of the output image are set to HSV.

HSV2RGB

Converts HSV images to the RGB color model.

```
void iplHSV2RGB(IplImage* hsvImage, IplImage* rgbImage);
```

`hsvImage`	The source HSV image.
`rgbImage`	The resultant RGB image.

Discussion

The function converts the HSV image `hsvImage` to the RGB image `rgbImage`. The function checks that the input image is an HSV image and that the output image is RGB.

RGB2HLS

Converts RGB images to the HLS color model.

```
void iplRGB2HLS(IplImage* rgbImage, IplImage* hlsImage);
```

`rgbImage`	The source RGB image.
`hlsImage`	The resultant HLS image.

Discussion

The function converts the RGB image `rgbImage` to the HLS image `hlsImage`. The function checks that the input image is an RGB image. The function sets the channel sequence and color model of the output image to HLS.

HLS2RGB

Converts HLS images to the RGB color model.

```
void iplHLS2RGB(IplImage* hlsImage, IplImage* rgbImage);
```

`hlsImage`	The source HLS image.
`rgbImage`	The resultant RGB image.

Discussion

The function converts the HLS image `hlsImage` to the RGB image `rgbImage`; see [Rogers85]. The function checks that the input image is an HLS image and that the output image is RGB.

RGB2LUV

Converts RGB images to the LUV color model.

```
void iplRGB2LUV(IplImage* rgbImage, IplImage* luvImage);
```

`rgbImage`	The source RGB image.
`luvImage`	The resultant LUV image.

Discussion

The function converts the RGB image `rgbImage` to the LUV image `luvImage`. The function checks that the input image is an RGB image; it sets the channel sequence and color model of the output image to LUV. The function processes 32f images only.

LUV2RGB

Converts LUV images to the RGB color model.

```
void iplLUV2RGB(IplImage* luvImage, IplImage* rgbImage);
```

`luvImage`	The source LUV image.
`rgbImage`	The resultant RGB image.

Discussion

The function converts the LUV image `luvImage` to the RGB image `rgbImage`. The function checks that the input image is an LUV image and that the output image is RGB.
The function processes 32f images only.

RGB2XYZ

Converts RGB images to the XYZ color model.

```
void iplRGB2XYZ(IplImage* rgbImage, IplImage* xyzImage);
```

`rgbImage`	The source RGB image.
`xyzImage`	The resultant XYZ image.

Discussion

The function converts the RGB image `rgbImage` to the XYZ image `xyzImage` according to the following formulas:

$$X = 0.4124 \cdot R + 0.3576 \cdot G + 0.1805 \cdot B$$
$$Y = 0.2126 \cdot R + 0.7151 \cdot G + 0.0721 \cdot B$$
$$Z = 0.0193 \cdot R + 0.1192 \cdot G + 0.9505 \cdot B.$$

The function checks that the input image is an RGB image; it sets the channel sequence and color model of the output image to XYZ. Since $0.0193 + 0.1192 + 0.9505 > 1$, the Z value might saturate.

XYZ2RGB

Converts XYZ images to the RGB color model.

```
void iplXYZ2RGB(IplImage* xyzImage, IplImage* rgbImage);
```

`xyzImage`	The source XYZ image.
`rgbImage`	The resultant RGB image.

Discussion

The function converts the XYZ image `xyzImage` to the RGB image `rgbImage`. The function checks that the input image is an XYZ image and that the output image is RGB.

RGB2YCrCb

Converts RGB images to the YCrCb color model.

```
void iplRGB2YCrCb(IplImage* rgbImage, IplImage*
    YCrCbImage);
```

`rgbImage` The source RGB image.

`YCrCbImage` The resultant YCrCb image.

Discussion

The function converts the RGB image `rgbImage` to the YCrCb image `YCrCbImage` (via the YUV model) according to the following formulas:

$Y = 0.3 \cdot R + 0.6 \cdot G + 0.1 \cdot B$

$U = B - Y \qquad Cb = 0.5 \cdot (U + 1)$

$V = R - Y \qquad Cr = V/1.6 + 0.5.$

The function checks that the input image is an RGB image; it sets the channel sequence and color model of the output image to “YCr”.

YCrCb2RGB

Converts YCrCb images to the RGB color model.

```
void iplYCrCb2RGB(IplImage* YCrCbImage, IplImage*
    rgbImage);
```

`YCrCbImage` The source YCrCb image.

`rgbImage` The resultant RGB image.

Discussion

The function converts the YCrCb image `YCrCbImage` to the RGB image `rgbImage`. The function checks that the input image is a YCrCb image and that the output image is RGB.

RGB2YUV

Converts RGB images to the YUV color model.

```
void iplRGB2YUV(IplImage* rgbImage, IplImage* yuvImage);
```

`rgbImage`	The source RGB image.
`yuvImage`	The resultant YUV image.

Discussion

The function converts the RGB image `rgbImage` to the YUV image `yuvImage` according to the following formulas:

$$Y = 0.3 \cdot R + 0.6 \cdot G + 0.1 \cdot B$$
$$U = B - Y$$
$$V = R - Y.$$

The function checks that the input image is an RGB image; it sets the channel sequence and color model of the output image to YUV.

YUV2RGB

Converts YUV images to the RGB color model.

```
void iplYUV2RGB(IplImage* yuvImage, IplImage* rgbImage);
```

`yuvImage`	The source YUV image.
`rgbImage`	The resultant RGB image.

Discussion

The function converts the YUV image `yuvImage` to the RGB image `yuvImage`. The function checks that the input image is an YUV image and that the output image is RGB.

YCC2RGB

Converts HLS images to the RGB color model.

```
void iplYCC2RGB(IplImage* YCCImage, IplImage* rgbImage);
```

YCCImage	The source YCC image.
rgbImage	The resultant RGB image.

Discussion

The function converts the YCC image *YCCImage* to the RGB image *rgbImage*; see [Rogers85]. The function checks that the input image is an YCC image and that the output image is RGB. Both images must be 8-bit unsigned.

Using Color-Twist Matrices

One of the methods of color model conversion is using a color-twist matrix. The color-twist matrix is a generalized 4 by 4 matrix $[t_{i,j}]$ that converts the three channels (a, b, c) into (d, e, f) according to the following matrix multiplication by a color-twist matrix (the superscript `T` is used to indicate the transpose of the matrix).

$$[d, e, f, 1]^T = \begin{bmatrix} t11 & t12 & t13 & t14 \\ t21 & t22 & t23 & t24 \\ t31 & t32 & t33 & t34 \\ 0 & 0 & 0 & t44 \end{bmatrix} * [a, b, c, 1]^T$$

To apply a color-twist matrix to an image, use the function `iplApplyColorTwist()`. But first call the `iplCreateColorTwist()` and `iplSetColorTwist()`functions to create the data structure `IplColorTwist`. This data structure contains the color-twist matrix and allows you to store the data internally in a form that is efficient for computation.

CreateColorTwist

Creates a color-twist matrix data structure.

```
IplColorTwist* iplCreateColorTwist(int data[16],
int scalingValue);
```

`data` — An array containing the sixteen values that constitute the color-twist matrix. The values are in row-wise order. Color-twist values that are in the range -1 to 1 should be scaled up to be in the range -2^{31} to $2^{31}-1$. (Simply multiply the floating point number in the -1 to 1 range by 2^{31}.)

`scalingValue` — The scaling value: an exponent of a power of 2 that was used to convert to integer values; for example, if 2^{31} was used to multiply the values, the `scalingValue` is 31. This value is used for normalization.

Discussion

The function `iplCreateColorTwist()` allocates memory for the data structure `IplColorTwist` and creates the color-twist matrix that can subsequently be used by the function `iplApplyColorTwist()`.

Return Value

A pointer to the `IplColorTwist` data structure containing the color-twist matrix in the form suitable for efficient computation by the function `iplApplyColorTwist()`.

SetColorTwist

Sets a color-twist matrix data structure.

```
void iplSetColorTwist(IplColorTwist* cTwist, int data[16],
int scalingValue);
```

data	An array containing the sixteen values that constitute the color-twist matrix. The values are in row-wise order. Color-twist values that are in the range -1 to 1 should be scaled up to be in the range -2^{31} to 2^{31}. (Simply multiply the floating point number in the -1 to 1 range by 2^{31}.)
scalingValue	The scaling value: an exponent of a power of 2 that was used to convert to integer values; for example, if 2^{31} was used to multiply the values, the *scalingValue* is 31. This value is used for normalization.

Discussion

The function `iplSetColorTwist()` is used to set the vaules of the color-twist matrix in the data structure `IplColorTwist` that can subsequently be used by the function `iplApplyColorTwist()`.

Return Value

A pointer to the `IplColorTwist` data structure containing the color-twist matrix in the form suitable for efficient computation by the function `iplApplyColorTwist()`.

ApplyColorTwist

Applies a color-twist matrix to an image.

```
void iplApplyColorTwist(IplImage* srcImage,
IplImage* dstImage, IplColorTwist* cTwist, int offset);
```

srcImage	The source image.
dstImage	The resultant image.
cTwist	The color-twist matrix data structure that was prepared by a call to the function `iplSetColorTwist()`.
offset	An offset value that will be added to each pixel channel after multiplication by the color-twist matrix.

Discussion

The function `iplApplyColorTwist()` applies the color-twist matrix to each of the first three color channels in the input image to obtain the resulting data for the three channels.

For example, the matrix below can be used to convert normalized `PhotoYCC` to normalized `PhotoRGB` (both with an opacity channel) when the channels are in the order YCC and RGB, respectively:

$$\begin{matrix} 2^{31} & 0 & 2^{31} & 0 \\ 2^{31} & X & Y & 0 \\ 2^{31} & 2^{31} & 0 & 0 \\ 0 & 0 & 0 & 2^{31} \end{matrix}$$

where $X = -416611827$ (that is, $-0.194 \cdot 2^{31}$) and
$Y = -1093069176$ (that is, $-0.509 \cdot 2^{31}$).

Color-twist matrices may also be used to perform many other color conversions as well as the following operations:

- Lightening an image
- Color saturation
- Color balance
- R, G, and B color adjustments
- Contrast adjustment.

DeleteColorTwist

Frees memory used for a color-twist matrix.

```
void iplDeleteColorTwist(IplColorTwist* cTwist);
```

`cTwist`	The color-twist matrix data structure that was prepared by a call to the function `iplCreateColorTwist()`.

Discussion

The function `iplDeleteColorTwist()` frees memory used for the color-twist matrix structure referred to by `cTwist`.

ColorTwistFP

Applies a color-twist matrix to an image with floating-point pixel values.

```
IPLStatus iplColorTwistFP (const IplImage* src, IplImage*
dst, float* cTwist)
```

src	The source image.
dst	The resultant image.
cTwist	The array containing color-twist matrix elements.

Discussion

The function `iplColorTwistFP()` applies the color-twist matrix stored in the array *cTwist* to each of the first three color channels.

Mathematically, the function performs the following operation:

$$R' = t_{00}\cdot R + t_{01}\cdot G + t_{02}\cdot B + t_{03}$$
$$G' = t_{10}\cdot R + t_{11}\cdot G + t_{12}\cdot B + t_{13}$$
$$B' = t_{20}\cdot R + t_{21}\cdot G + t_{22}\cdot B + t_{23}$$

Here (R', G', B') are the output values of the first three channels, and (R, G, B) are the input values of these channels. The array *cTwist* should contain the color-twise matrix elements in this order:

$$t_{00}\ t_{01}\ t_{02}\ t_{03}\ t_{10}\ t_{11}\ t_{12}\ t_{13}\ t_{20}\ t_{21}\ t_{22}\ t_{23}$$

Both *src* and *dst* images must contain 32-bit floating-point pixel data. Tiling and rectangular ROIs are supported; masking and COIs are not.

The function returns `IPL_StsOk` on success, or an error status code on failure (if the application passes invalid arguments or if there is insufficient memory to perform the operation).

This page is left blank for double-sided printing

This page is left blank for double-sided printing

Histogram, Threshold, and Compare Functions 10

This chapter describes functions that operate on an image on a pixel-by-pixel basis: compare, threshold, and histogram functions. Table 10-1 lists all functions in these groups.

Table 10-1 Histogram, Threshold, and Compare Functions

Group	Function Name	Description
Thresholding	`iplThreshold`	Performs a simple thresholding of an image.
Lookup Table and Histogram	`iplContrastStretch`	Stretches the contrast of an image using intensity transformation.
	`iplComputeHisto`	Computes the intensity histogram of an image.
	`iplHistoEqualize`	Enhances an image by flattening its intensity histogram.
Comparing Images	`iplGreater`<br>`iplLess`<br>`iplEqual`	Compares the pixels of two input images and writes the results (0 or 1) to the corresponding pixels of the 1-bit output image.
	`iplGreaterS`<br>`iplGreaterSFP`<br>`iplLessS`<br>`iplLessSFP`<br>`iplEqualS`<br>`iplEqualSFP`	Compares the input image's pixels with a constant and writes the results (0 or 1) to the corresponding pixels in the 1-bit output image.

continued ☞

Table 10-1 Compare, Threshold, and Histogram Functions (continued)

Group	Function Name	Description
Comparing Images (continued)	`iplEqualFPEps`	Performs an equality test with tolerance ε for two input images containing 32-bit floating-point pixel data and writes the results (0 or 1) to each pixel of the output image.
	`iplEqualSFPEps`	Performs an equality test with tolerance ε for the input image and a constant, and writes the results (0 or 1) to the corresponding pixels of the output image.

Thresholding

The threshold operation changes pixel values depending on whether they are less or greater than the specified *`threshold`*. If an input pixel value is less than the *`threshold`*, the corresponding output pixel is set to the minimum presentable value. Otherwise, it is set to the maximum presentable value.

Threshold

Performs a simple thresholding of an image.

```
void iplThreshold(IplImage* srcImage, IplImage* dstImage,
                  int threshold);
```

`srcImage`	The source image.
`dstImage`	The resultant image.

threshold	The threshold value to use for each pixel. The pixel value in the output is set to the maximum presentable value if it is greater than or equal to the threshold value (for each channel). Otherwise the pixel value in the output is set to the minimum presentable value.

Discussion

The function `iplThreshold()` thresholds the source image *srcImage* using the value *threshold* to create the resultant image *dstImage*. The pixel value in the output is set to the maximum presentable value (for example, 255 for an 8-bit-per-channel image) if it is greater than or equal to the threshold value. Otherwise it is set to the minimum presentable value (for example, 0 for an 8-bit-per-channel image). This is done for each channel in the input image.

To convert an image to bitonal, you can use `iplThreshold()` function as shown in Example 10-1.

Example 10-1 Conversion to a Bitonal Image

```
int example101( void ) {
   IplImage *imga, *imgb;
   const int width = 4, height = 4;

   __try {
      imga = iplCreateImageHeader(
         1, 0, IPL_DEPTH_8U, "GRAY", "GRAY",
         IPL_DATA_ORDER_PIXEL, IPL_ORIGIN_TL,
         IPL_ALIGN_DWORD, width, height, NULL, NULL,
         NULL, NULL);
      if( NULL == imga ) return 0;

      imgb = iplCreateImageHeader(
         1, 0, IPL_DEPTH_1U, "GRAY", "GRAY",
         IPL_DATA_ORDER_PIXEL, IPL_ORIGIN_TL,
         IPL_ALIGN_DWORD, width, height, NULL, NULL,
         NULL, NULL);
      if( NULL == imgb ) return 0;

      // Create with filling
      iplAllocateImage( imga, 1, 3 );
      if( NULL == imga->imageData ) return 0;
      // Make a spike
      ((char*)imga->imageData)[7] = (char)7;
      iplAllocateImage( imgb, 0, 0 );
      if( NULL == imgb->imageData ) return 0;

      // This is important. 4 bits occupy 4 bytes
      // in the imgb image because of IPL_ALIGN_DWORD
      iplThreshold( imga, imgb, 7 );

      // Check if an error occurred
      if( iplGetErrStatus() != IPL_StsOk ) return 0;
   }
   __finally {
      iplDeallocate(imga, IPL_IMAGE_HEADER | IPL_IMAGE_DATA );
      iplDeallocate(imgb, IPL_IMAGE_HEADER | IPL_IMAGE_DATA );
   }
   return IPL_StsOk == iplGetErrStatus();
}
```

Lookup Table (LUT) and Histogram Operations

A LUT can be used to specify an intensity transformation. Given an input intensity, LUT can be used to look up an output intensity. Usually a LUT is provided for each channel in the image, although sometimes the same LUT can be shared by many channels.

The IplLUT Structure

You can set a lookup table using the `IplLUT` structure. The C language definition of the `IplLUT` structure is as follows:

IplLUT Structure Definition

```
typedef struct _IplLUT {
    int         num;    /* number of keys or values */
    int*        key;
    int*        value;
    int*        factor;
    int         interpolateType;
 } IplLUT;
```

The *`key`* array has the length *`num`*; the *`value`* and *`factor`* are arrays of the same length *`num`*`-1`. The *`interpolateType`* can be either `IPL_LUT_LOOKUP` or `IPL_LUT_INTER`.
Consider the following example of *`num`* = 4:

key	*value*	*factor*
k0	v0	f0
k1	v1	f1
k2	v2	f2
k3		

If *`interpolateType`* is `LOOKUP`, then any input intensity `D` in the range `k0 ≤ D < k1` will result in the value `v0`, in the range `k1 ≤ D < k2` will result in the value `v1` and so on. If *`interpolateType`* is `INTER`, then an intensity `D` in the range `k0 ≤ D < k1` will result in the linearly interpolated value

```
v0 + [(v1 – v0)/(k1 – k0)] * (D – k0)
```

The value `(v1-v0)/(k1-k0)` is pre-computed and stored as `f0` in the array *`factor`* in the `IplLUT` data structure, the value `(v2-v1)/(k2-k1)` is stored as `f1` and so on. Thus, the actual formula used by library functions to compute the interpolated value of `D` for example in the range `k2 ≤ D < k3` is as follows:

```
D′ = v2 + f2 * (D – k2)
```

Note that to calculate the interpolated value of `D` in this last interval, library functions do not need the value `v3`, which is used only by the application to pre-compute the factor `f2` .

The data structure described above can be used to specify a piece-wise linear transformation that is ideal for the purpose of contrast stretching.

The histogram is a data structure that shows how the intensities in the image are distributed. The same data structure `IplLUT` is used for a histogram except that *`interpolateType`* is always `IPL_LUT_LOOKUP` and *`factor`* is a `NULL` pointer for a histogram. However, unlike the LUT, the *`value`* array represents counts of pixels falling in the specified ranges in the *`key`* array.

The sections that follow describe the functions that use the above data structure.

ConstrastStretch

Stretches the contrast of an image using an intensity transformation.

```
void iplContrastStretch(IplImage* srcImage,
IplImage* dstImage, IplLUT** lut);
```

`srcImage` The source image.

`dstImage` The resultant image.

`lut` An array of pointers to LUTs, one pointer for each channel. Each lookup table should have the `key`, `value` and `factor` arrays fully initialized (see "The `IplLUT` Structure"). One or more channels may share the same LUT. Specifies an intensity transformation.

Discussion

The function `iplContrastStretch()` stretches the contrast in a color source image `srcImage` by applying intensity transformations specified by LUTs in `lut` to produce an output image `dstImage`. Fully specified LUTs should be provided to this function.

Example 10-2 Using the Function iplContrastStretch() to Enhance an Image

```
void fullRange() {
    const int width = 32, height = 32, range = 256;
    IplLUT lut = { range+1, NULL,NULL,NULL, IPL_LUT_INTER };
    IplLUT* plut = &lut;
    int i, mn, mx;
    /// make a full range image
    IplImage* img = iplCreateImageJaehne( IPL_DEPTH_8U, width,
    height );
```

Continued ☞

Example 10-2 Using the Function iplContrastStretch() to Enhance an Image (continued)

```
    /// allocate LUT's arrays
    lut.key = malloc( sizeof(int)*(range+1) );
    lut.value = malloc( sizeof(int)*range );
    lut.factor = malloc( sizeof(int)*range );

    /// make the image with a narrow and shifted range
    iplRShiftS( img, img, 4 );
    iplAddS( img, img, 4 );

    /// compute histogram and find min and max values
    for( i=0; i<=range; i++) lut.key[i] = i;
    iplComputeHisto( img, &plut );
    mn = 0; while( !lut.value[mn] ) mn++;
    mx = 255; while( !lut.value[mx] ) mx--;

    /// prepare LUT for stretching
    lut.interpolateType = IPL_LUT_INTER; /// interpolation
    mode, not lookup
    lut.num = 2; /// num of key values
    lut.key[0] = 0; /// lower value
    lut.key[1] = 255; /// upper value
    lut.factor[0] = 255 / (mx - mn); /// factor to extend
    range
    lut.value[0] = -lut.factor[0] * mn; /// value to shift

    /// The operation is: x(i) = x(i) * factor + value
    iplContrastStretch( img, img, &plut );

    /// compute histogram and find min and max values again
    lut.num = 257;
    lut.key[1] = 1;
    iplComputeHisto( img, &plut );
    mn = 0; while( !lut.value[mn] ) mn++;
    mx = 255; while( !lut.value[mx] ) mx--;

    free( lut.factor);
    free( lut.value );
    free( lut.key );
    iplDeallocate( img, IPL_IMAGE_ALL );
}
```

ComputeHisto

Computes the intensity histogram of an image.

```
void iplComputeHisto(IplImage* srcImage, IplLUT** lut);
```

srcImage	The source image for which the histogram will be computed.
lut	An array of pointers to LUTs, one pointer for each channel. Each lookup table should have the *key* array fully initialized. The *value* array will be filled by this function. (For the *key* and *value* arrays, see "The IplLUT Structure" above.) The same LUT can be shared by one or more channels.

Discussion

The function iplComputeHisto() computes the intensity histogram of an image. The histograms (one per channel in the image) are stored in the array *lut* containing all the LUTs. The *key* array in each LUT should be initialized before calling this function. The *value* array containing the histogram information will be filled in by this function. (For the *key* and *value* arrays, see "The IplLUT Structure" above.)

HistoEqualize

Enhances an image by flattening its intensity histogram.

```
void iplHistoEqualize(IplImage* srcImage,
IPLImage* dstImage, IplLUT** lut);
```

`srcImage`	The source image for which the histogram will be computed.
`dstImage`	The resultant image after equalizing.
`lut`	The histogram of the image is represented as an array of pointers to LUTs, one pointer for each channel. Each lookup table should have the `key` and `value` arrays fully initialized. (For the `key` and `value` arrays, see "The `IplLUT` Structure" above.) These LUTs will contain flattened histograms after this function is executed. In other words, the call of `iplHistoEqualize()` is destructive with respect to the LUTs.

Discussion

The function `iplHistoEqualize()` enhances the source image `srcImage` by flattening its histogram represented by `lut` and places the enhanced image in the output image `dstImage`. After execution, `lut` points to the flattened histogram of the output image; see Example 10-2.

Example 10-3 Computing and Equalizing the Image Histogram

```
int example102( void ) {
   IplImage *imga;
   const int width = 4, height = 4, range = 256;
   IplLUT lut = { range+1, NULL,NULL,NULL, IPL_LUT_LOOKUP };
   IplLUT* plut = &lut;

   __try {
      int i;
      lut.key = malloc( sizeof(int)*(range+1) );
      lut.value = malloc( sizeof(int)*range );
      imga = iplCreateImageHeader(
         1, 0, IPL_DEPTH_8U, "GRAY", "GRAY",
         IPL_DATA_ORDER_PIXEL, IPL_ORIGIN_TL,
         IPL_ALIGN_DWORD, width, height, NULL, NULL,
         NULL, NULL);
      if( NULL == imga ) return 0;

      // Create with filling
      iplAllocateImage( imga, 1, 3 );
      if( NULL == imga->imageData ) return 0;
      // Make the two level data
      for( i=0; i<8; i++) ((char*)imga->imageData)[i] = (char)7;
      // Initialize the histogram levels
      for( i=0; i<=range; i++) lut.key[i] = i;

      // Compute histogram
      iplComputeHisto( imga, &plut );
      // Equalize histogram = rescale range of image data
      iplHistoEqualize( imga, imga, &plut );

      // Check if an error occurred
      if( iplGetErrStatus() != IPL_StsOk ) return 0;
   }
   __finally {
      iplDeallocate( imga, IPL_IMAGE_HEADER | IPL_IMAGE_DATA );
      if( lut.key ) free( lut.key );
      if( lut.value ) free( lut.value );
   }
   return IPL_StsOk == iplGetErrStatus();
}
```

Comparing Images

This section describes the functions that allow you to compare images. Each compare function writes its results to a 1-bit output image. The output pixel is set to 1 if the corresponding input pixel(s) satisfied the compare condition; otherwise, the output pixel is set to 0. Often, you might wish to use the compare functions to generate a 1-bit mask image for future use in other image-processing operations.

Functions whose names have a capital `S` (for example, `iplGreaterS`) compare the pixels of *a single input image* and a scalar variable. Functions whose names don't have an `S` (such as `iplGreater`) compare the corresponding pixels in *two input images*. The two input images must have the same bit depth, origin, and channel of interest (COI) setting.

When the input pixels have more than one channel and the COI is not set, the result will be 1 only for those pixels in which *each channel* satisfies the compare condition.

For example, in case of `iplGreater` (two input images) one RGB pixel is "greater" than another only if all three channel values of the first pixel are greater than those of the second. Thus, if at least one of the channel values in an input pixel is less than or equal to that channel's value in the other image, then `iplGreater` will set the corresponding output pixel to 0.

Functions that use a single input image work similarly. If you don't set the COI, the function compares all channel values to the input scalar value. Again, the result will be 1 only for those pixels in which each channel satisfies the required condition. For example, an RGB pixel is considered to be "equal" to the input scalar value only if all three RGB channels are equal to that value. If at least one of the channel values is greater or less than the scalar value, the function `iplEqualS` will set the corresponding output pixel to 0.

Greater

Tests if the pixel values of the first image are greater than those of the second image.

```
IPLStatus iplGreater (IplImage* img1, IplImage* img2,
   IplImage* dst);
```

img1, img2	The source images.
dst	The resultant 1-bit image.

Discussion

The function `iplGreater()` compares the corresponding pixels of two input images for “greater than” and writes the results to a 1-bit image *dst*. If a pixel’s value in *img1* is greater than that pixel’s value in *img2*, then the corresponding pixel in *dst* is set to 1; otherwise the pixel in *dst* is set to 0.

The images *img1* and *img2* must have the same bit depth, origin, and COI settings. If the COI is not set, an *img1* pixel is considered to be “greater” than an *img2* pixel only if each channel in the *img1* pixel is greater than that channel in the *img2* pixel. If the COI is set, the function compares only the COI values.

The function returns `IPL_StsOK` if the compare operation is successful. If you pass incompatible *img1* and *img2* or a null pointer, the function does not perform the compare operation and returns an error status code.

Less

Tests if the pixel values of the first image are less than those of the second image.

```
IPLStatus iplLess (IplImage* img1, IplImage* img2,
   IplImage* dst);
```

`img1, img2`	The source images.
`dst`	The resultant 1-bit image.

Discussion

The function `iplLess()` compares the corresponding pixels of two input images for “less than” and writes the results to a 1-bit image `dst`. If a pixel’s value in `img1` is less than that pixel’s value in `img2`, then the corresponding pixel in `dst` is set to 1; otherwise the pixel in `dst` is set to 0.

The images `img1` and `img2` must have the same bit depth, origin, and COI settings. If the COI is not set, an `img1` pixel is considered to be “less” than an `img2` pixel only if each channel in the `img1` pixel is less than that channel in the `img2` pixel. If the COI is set, the function compares only the COI values.

The function returns `IPL_StsOK` if the compare operation is successful. If you pass incompatible `img1` and `img2` or a null pointer, the function does not perform the compare operation and returns an error status code.

Equal

Tests if the pixel values of the first image are equal to those of the second image.

```
IPLStatus iplEqual (IplImage* img1, IplImage* img2,
   IplImage* dst);
```

img1, img2	The source images.
dst	The resultant 1-bit image.

Discussion

The function `iplEqual()` compares the corresponding pixels of two input images for equality and writes the results to a 1-bit image *dst*. If a pixel's value in *img1* is equal to that pixel's value in *img2*, then the corresponding pixel in *dst* is set to 1; otherwise the pixel in *dst* is set to 0.

The images *img1* and *img2* must have the same bit depth, origin, and COI settings. If the COI is not set, an *img1* pixel is considered to be equal to an *img2* pixel only if each channel in the *img1* pixel is equal to that channel in the *img2* pixel. If the COI is set, the function compares only the COI values.

The function returns `IPL_StsOK` if the compare operation is successful. If you pass incompatible *img1* and *img2* or a null pointer, the function does not perform the compare operation and returns an error status code.

EqualFPEps

Tests if the floating-point pixel values in two images are equal within a tolerance ε.

```
IPLStatus iplEqualFPEps (IplImage* img1, IplImage* img2,
   IplImage* dst, float eps);
```

`img1, img2`	The source images.
`dst`	The resultant 1-bit image.
`eps`	The tolerance value.

Discussion

The function `iplEqualFPEps()` tests if the corresponding pixels of two input images are equal within the tolerance *eps*, and writes the results to a 1-bit image *dst*. If the absolute value of difference of the pixel values in *img1* and *img2* is less than *eps*, then the corresponding pixel in *dst* is set to 1; otherwise the pixel in *dst* is set to 0.

Both *img1* and *img2* must contain 32-bit floating-point pixel data. They must have the same origin and COI settings. If the COI is not set, pixels in *img1* and *img2* are considered to be “equal” only if each channel in the *img1* pixel is equal, within the tolerance *eps*, to that channel in the *img2* pixel. If the COI is set, the function compares only the COI values.

The function returns `IPL_StsOK` if the compare operation is successful. If you pass incompatible *img1* and *img2* or a null pointer, the function does not perform the compare operation and returns an error status code.

GreaterS

Tests if the image's pixel values are greater than an integer scalar value.

```
IPLStatus iplGreaterS (IplImage* src, int s,
   IplImage* dst);
```

src	The source image.
s	The integer scalar value to be compared with pixel values.
dst	The resultant 1-bit image.

Discussion

The function `iplGreaterS()` compares the pixels of the input image *src* and a scalar value *s* for "greater than" and writes the results to a 1-bit image *dst*. If a pixel's value is greater than *s*, then the corresponding pixel in *dst* is set to 1; otherwise the pixel in *dst* is set to 0.

The function supports all pixel data types except 32-bit floating-point data. (For images with floating-point data, use the function `iplGreaterSFP()` described on the next page.) If the source image COI is not set, a pixel is considered to be "greater" than *s* only if each channel in the pixel is greater than *s*. If the COI is set, the function compares *s* and the pixel values in the COI.

The function returns `IPL_StsOK` if the compare operation is successful. If you pass an image with data of an unsupported type or a null pointer, the function does not perform the compare operation and returns an error status code.

GreaterSFP

Tests if the image's pixel values are greater than a floating-point scalar value.

```
IPLStatus iplGreaterSFP (IplImage* src, float s,
   IplImage* dst);
```

`src`	The source image.
`s`	The 32-bit floating-point scalar value to be compared with pixel values.
`dst`	The resultant 1-bit image.

Discussion

The function `iplGreaterSFP()` compares the pixels of the input image `src` and a scalar value `s` for "greater than" and writes the results to a 1-bit image `dst`. If an input pixel's value is greater than `s`, then the corresponding pixel in `dst` is set to 1; otherwise the pixel in `dst` is set to 0.

The function supports only images with 32-bit floating-point pixel data. (For images with data of other types, use the function `iplGreaterS()` described on the previous page.) If the source image COI is not set, a pixel is considered to be "greater" than `s` only if each channel in the pixel is greater than `s`. If the COI is set, the function compares `s` and the pixel values in the COI.

The function returns `IPL_StsOK` if the compare operation is successful. If you pass an image with data of an unsupported type or a null pointer, the function does not perform the compare operation and returns an error status code.

LessS

Tests if the image's pixel values are less than an integer scalar value.

```
IPLStatus iplLessS (IplImage* src, int s,
   IplImage* dst);
```

`src`	The source image.
`s`	The integer scalar value to be compared with pixel values.
`dst`	The resultant 1-bit image.

Discussion

The function `iplLessS()` compares the pixels of the input image `src` and a scalar value `s` for "less than" and writes the results to a 1-bit image `dst`. If a pixel's value is less than `s`, then the corresponding pixel in `dst` is set to 1; otherwise the pixel in `dst` is set to 0.

The function supports all pixel data types except 32-bit floating-point data. (For images with floating-point data, use the function `iplLessSFP()` described on the next page.) If the source image COI is not set, a pixel is considered to be "less" than `s` only if each channel in the pixel is less than `s`. If the COI is set, the function compares `s` and the pixel values in the COI.

The function returns `IPL_StsOK` if the compare operation is successful. If you pass an image with data of an unsupported type or a null pointer, the function does not perform the compare operation and returns an error status code.

LessSFP

Tests if the image's pixel values are less than a floating-point scalar value.

```
IPLStatus iplLessSFP (IplImage* src, float s,
   IplImage* dst);
```

`src`	The source image.
`s`	The 32-bit floating-point scalar value to be compared with pixel values.
`dst`	The resultant 1-bit image.

Discussion

The function `iplLessSFP()` compares the pixels of the input image `src` and a scalar value `s` for "less than" and writes the results to a 1-bit image `dst`. If an input pixel's value is less `s`, then the corresponding pixel in `dst` is set to 1; otherwise the pixel in `dst` is set to 0.

The function supports only images with 32-bit floating-point pixel data. (For images with data of other types, use the function `iplLessS()` described on the previous page.) If the source image COI is not set, a pixel is considered to be "less" than `s` only if each channel in the pixel is less than `s`. If the COI is set, the function compares `s` and the pixel values in the COI.

The function returns `IPL_StsOK` if the compare operation is successful. If you pass an image with data of an unsupported type or a null pointer, the function does not perform the compare operation and returns an error status code.

EqualS

Tests if the image's pixel values are equal to an integer scalar value.

```
IPLStatus iplEqualS (IplImage* src, int s,
   IplImage* dst);
```

`src`	The source image.
`s`	The integer scalar value to be compared with pixel values.
`dst`	The resultant 1-bit image.

Discussion

The function `iplEqualS()` compares the pixels of the input image `src` and an integer scalar value `s` for equality and writes the results to a 1-bit image `dst`. If a pixel's value is equal to `s`, then the corresponding pixel in `dst` is set to 1; otherwise the pixel in `dst` is set to 0.

The function supports all pixel data types except 32-bit floating-point data. (For images with floating-point data, use the function `iplEqualSFP()` described on the next page.) If the source image COI is not set, a pixel is considered to be equal to `s` only if each channel in the pixel is equal to `s`. If the COI is set, the function compares `s` and the pixel values in the COI.

The function returns `IPL_StsOK` if the compare operation is successful. If you pass an image with data of an unsupported type or a null pointer, the function does not perform the compare operation and returns an error status code.

EqualSFP

Tests if the image's pixel values are equal to a floating-point scalar value.

```
IPLStatus iplEqualSFP (IplImage* src, float s,
   IplImage* dst);
```

src	The source image.
s	The 32-bit floating-point scalar value to be compared with pixel values.
dst	The resultant 1-bit image.

Discussion

The function `iplEqualSFP()` compares the pixels of the input image *src* and a scalar value *s* for equality and writes the results to a 1-bit image *dst*. If an input pixel's value is equal to *s*, then the corresponding pixel in *dst* is set to 1; otherwise the pixel in *dst* is set to 0.

The function supports only images with 32-bit floating-point pixel data. (For images with data of other types, use the function `iplEqualS()` described on the previous page.) If the source image COI is not set, a pixel is considered to be "equal" to *s* only if each channel in the pixel is equal to *s*. If the COI is set, the function compares *s* and the pixel values in the COI.

The function returns `IPL_StsOK` if the compare operation is successful. If you pass an image with data of an unsupported type or a null pointer, the function does not perform the compare operation and returns an error status code.

EqualSFPEps

Tests if the pixel values are equal to a floating-point scalar value within a tolerance ε.

```
IPLStatus iplEqualSFPEps (IplImage* src, float s,
   IplImage* dst, float eps);
```

src	The source image.
s	The 32-bit floating-point scalar value to be compared with pixel values.
dst	The resultant 1-bit image.
eps	The tolerance ε.

Discussion

The function `iplEqualSFPEps()` tests if pixels of the input image *src* are equal to a scalar value *s* within the tolerance *eps*, and writes the results to a 1-bit image *dst*. If the absolute value of difference of the input pixel value and *s* is less than *eps*, then the corresponding pixel in *dst* is set to 1; otherwise the pixel in *dst* is set to 0.

The function supports only images with 32-bit floating-point pixel data. If the source image COI is not set, a pixel is considered to be "equal" to *s* only if each channel in the pixel is equal to *s* within the given tolerance. If the COI is set, the function compares *s* and the pixel values in the COI.

The function returns `IPL_StsOK` if the compare operation is successful. If you pass an image with data of an unsupported type or a null pointer, the function does not perform the compare operation and returns an error status code.

This page is left blank for double-sided printing

This page is left blank for double-sided printing

Geometric Transforms

11

This chapter describes the functions that perform geometric transforms to resize the image, change the image orientation, or warp the image. There is also a special function, `iplRemap()`, for performing geometric transforms with a user-defined coordinate mapping.

Table 11-1 lists image geometric transform functions and macro definitions.

Table 11-1 Image Geometric Transform Functions and Macros

Group	Function Name	Description
Resizing	iplZoom	Zooms or expands an image.
	iplDecimate	Decimates (shrinks) an image.
	iplDecimateBlur	Blurs an image, then decimates the blurred image.
	iplResize	Resizes an image.
	iplZoomFit<br>iplDecimateFit<br>iplResizeFit	Change image size using image's dimensions to set scaling factors (macro definitions).
Changing Orientation	iplMirror	Mirrors an image.
	iplRotate	Rotates an image.
	iplGetRotateShift	Computes the shift for iplRotate() , given the rotation center and angle.
	iplRotateCenter	Rotates an image around an arbitrary center (macro definition).
Warping	iplShear	Shears an image.
	iplWarpAffine	Performs affine transforms with the specified coefficients.

Continued ☞

Table 11-1 Image Geometric Transform Functions (continued)

Group	Function Name	Description
Warping (cont.)	iplWarpBilinear	Performs a bilinear transform with the specified coefficients.
	iplWarpBilinearQ	Performs a bilinear transform with the specified reference quadrangle.
	iplWarpPerspective	Performs a perspective transform with the specified coefficients.
	iplWarpPerspectiveQ	Performs a perspective transform with the specified reference quadrangle.
Warping support	iplGetAffineBound<br>iplGetBilinearBound<br>iplGetPerspectiveBound	Compute the bounding rectangle for the rectangular ROI transformed by the warping functions.
	iplGetAffineQuad<br>iplGetBilinearQuad<br>iplGetPerspectiveQuad	Compute coordinates of the quadrangle to which the ROI is mapped by the warping functions.
	iplGetAffineTransform<br>iplGetBilinearTransform<br>iplGetPerspectiveTransform	Compute the coefficients of transforms performed by the warping functions.
Arbitrary mapping	iplRemap	Re-maps the image using a doordinate look-up table.

Internally, all geometric transformation functions handle regions of interest (ROIs) with the following sequence of operations:

- transform the rectangular ROI of the source image to a quadrangle in the destination image
- find the intersection of this quadrangle and the rectangular ROI of the destination image
- update the destination image in the intersection area, taking into account mask images (if any).

The source and destination images must be different; that is, in-place operations are not supported. The coordinates in the source and destination images must have the same origin.

Most of the geometric transformation functions have to *interpolate* the pixel values of the source image in order to compute the pixel values of the destination image. The Image Processing Library supports several interpolation algorithms. For more information on the algorithms supported in the library, see Appendix B.

Changing the Image Size

This section describes the functions that scale the input image in the *x*- or *y*-directions, without changing the image orientation.

These functions perform image resampling by using various kinds of interpolation algorithms: nearest neighbor, linear interpolation, cubic interpolation, and super-sampling.

Zoom

Zooms or expands an image.

```
void iplZoom(IplImage* srcImage, IplImage* dstImage, int
   xDst, int xSrc, int yDst, int ySrc, int interpolate);
```

`srcImage`	The source image.
`dstImage`	The resultant image.
`xDst,xSrc,yDst,ySrc`	Positive integers specifying the fractions `xDst/xSrc` ≥ 1 and `yDst/ySrc` ≥ 1 - the factors by which the *x* and *y* dimensions of the image's ROI are changed. For example, setting `xDst` = 2, `xSrc` = 1, `yDst` = 2, `ySrc` = 1 doubles the image size in each dimension to increase the image area by a factor of four.
`interpolate`	The type of interpolation to perform for resampling. Can be one of the following:<br>`IPL_INTER_NN` Nearest neighbor.<br>`IPL_INTER_LINEAR` Linear interpolation.<br>`IPL_INTER_CUBIC` Cubic interpolation.

Discussion

The function `iplZoom()` zooms or expands the source image `srcImage` by `xDst/xSrc` in the *x* direction and `yDst/ySrc` in the *y* direction. The interpolation specified by `interpolate` is used for resampling the input image.

Decimate

Decimates or shrinks an image.

```
void iplDecimate(IplImage* srcImage, IplImage* dstImage,
int xDst, int xSrc, int yDst, int ySrc, int interpolate);
```

`srcImage`	The source image.
`dstImage`	The resultant image.
`xDst,xSrc,yDst,ySrc`	Positive integers specifying the fractions `xDst/xSrc` ≤ 1 and `yDst/ySrc` ≤ 1 - the factors by which the *x* and *y* dimensions of the image's ROI are changed. For example, setting `xDst` = 1, `xSrc` = 2, `yDst` = 1, `ySrc` = 2 decreases the image size in each dimension by half.
`interpolate`	The type of interpolation to perform for resampling. Can be one of the following:<br>`IPL_INTER_NN` Nearest neighbor.<br>`IPL_INTER_LINEAR` Linear interpolation.<br>`IPL_INTER_CUBIC` Cubic interpolation.<br>`IPL_INTER_SUPER` Super-sampling.

Discussion

The function `iplDecimate()` decimates or shrinks the source image `srcImage` by `xDst/xSrc` in the *x* direction and `yDst/ySrc` in the *y* direction. The interpolation specified by `interpolate` is used for resampling the input image.

DecimateBlur

Blurs and decimates an image.

```
void iplDecimateBlur (IplImage* srcImage,
   IplImage* dstImage, int xDst, int xSrc, int yDst, int
   ySrc, int interpolate, int xMaskSize, int yMaskSize);
```

`srcImage`	The source image.
`dstImage`	The resultant image.
`xDst, xSrc, yDst, ySrc`	Positive integers specifying the fractions `xDst/xSrc` ≤ 1 and `yDst/ySrc` ≤ 1 - the factors by which the *x* and *y* dimensions of the image's ROI are changed (similar to `iplDecimate`).
`interpolate`	The type of interpolation to perform for resampling. Can be one of the following:<br>`IPL_INTER_NN` Nearest neighbor.<br>`IPL_INTER_LINEAR` Linear interpolation.<br>`IPL_INTER_CUBIC` Cubic interpolation.
`xMaskSize, yMaskSize`	The *x* and *y* size of the blur mask.

Discussion

The function `iplDecimateBlur()` blurs the input image using an `xMaskSize` by `yMaskSize` mask, then decimates the blurred image by a factor of `xDst/xSrc` in the *x* direction and `yDst/ySrc` in the *y* direction.

If mask rows and columns contain odd numbers of pixels, the mask anchor is exactly at the center of the mask. Otherwise, the function *rounds up* the center coordinates. Thus, in a 3x3 mask with top left corner at (0,0), the anchor is at (1,2). In a 3x4 mask, the anchor would be at (1,2).

The interpolation specified by `interpolate` is used for resampling the input image.

Resize

Resizes an image.

```
void iplResize(IplImage* srcImage, IplImage* dstImage, int
   xDst, int xSrc, int yDst, int ySrc, int interpolate);
```

`srcImage` The source image.

`dstImage` The resultant image.

`xDst,xSrc,yDst,ySrc` Positive integers specifying the fractions `xDst/xSrc` and `yDst/ySrc` - the factors by which the *x* and *y* dimensions of the image's ROI are changed. For example, setting `xDst` = 1, `xSrc` = 2, `yDst` = 2, `ySrc` = 1 halves the *x* and doubles the *y* dimension.

`interpolate` The type of interpolation to perform for resampling. Can be one of the following:

`IPL_INTER_NN` Nearest neighbor.

`IPL_INTER_LINEAR` Linear interpolation.

`IPL_INTER_CUBIC` Cubic interpolation.

`IPL_INTER_SUPER` Super-sampling (can be used only for `xDst` ≤ `xSrc`, `yDst` ≤ `ySrc`).

Discussion

The function `iplResize()` resizes the source image `srcImage` by `xDst/xSrc` in the *x* direction and `yDst/ySrc` in the *y* direction. The function differs from `iplZoom` and `iplDecimate` in that it can increase one dimension of an image while decreasing the other dimension.

The interpolation specified by `interpolate` is used for resampling the input image.

iplZoomFit
iplDecimateFit
iplResizeFit

Macro definitions that change the image size using the images' dimensions as scaling factors.

```
iplZoomFit( SRC, DST, INTER );
iplDecimateFit( SRC, DST, INTER );
iplResizeFit( SRC, DST, INTER );
```

SRC	The source image.
DST	The destination image.
INTER	The type of interpolation to perform for resampling the source image.

Discussion

Use macro definitions `iplZoomFit()`, `iplDecimateFit()`, `iplResizeFit()` to resize a source image ROI so that its dimensions fit into the destination ROI (or the whole image) size. These macros use dimensions of source and destination images' ROIs (or the sizes of whole images) to determine the respective scaling factors in *x*- and *y*- directions. Note that *SRC* and *DST* pointers to `IplImage` structures are used but not checked in the macros. Thus, it is essential that your application checks that these pointers specify valid source and destination images.

Example 11-1 Using Macro Definition to Resize an Image

```
int ResizeFit( void ) {
   IplImage *imga = iplCreateImageJaehne(
     IPL_DEPTH_8U, 5, 5 );
   IplImage *imgb = iplCreateImageJaehne(
      IPL_DEPTH_8U, 7, 7 );
   IPLStatus st;

   iplResizeFit( imga, imgb, IPL_INTER_NN );
   st = iplGetErrStatus();

   iplDeallocate( imga, IPL_IMAGE_ALL );
   iplDeallocate( imgb, IPL_IMAGE_ALL );

   return IPL_StsOk == st;

}
```

Changing the Image Orientation

The functions described in this section change the image orientation by rotating or mirroring the source image. Rotation involves image resampling by using various kinds of interpolation: nearest neighbor, linear, or cubic interpolation (see Appendix B). Mirroring is performed by flipping the image axis in horizontal or vertical direction.

Rotate

Rotates an image around the (0,0) origin.

```
void iplRotate(IplImage* srcImage, IplImage* dstImage,
   double angle, double xShift, double yShift,
   int interpolate);
```

`srcImage` The source image.

`dstImage`	The resultant image.
`angle`	The angle (in degrees) to rotate the image. The image is rotated around the corner with coordinates (0,0).
`xShift, yShift`	The shifts along the *x*- and *y*-axes to be performed after the rotation.
`interpolate`	The type of interpolation to perform for resampling the source image. The following modes are supported:

`IPL_INTER_NN`	Nearest neighbor.
`IPL_INTER_LINEAR`	Linear interpolation.
`IPL_INTER_CUBIC`	Cubic interpolation.
`+IPL_SMOOTH_EDGE`	Smooth edges of an image. Can be added to interpolation by using bitwise logical OR (see Appendix B for details).

Discussion

The function `iplRotate()` rotates the source image `srcImage` by `angle` degrees around the origin (0,0) and shifts it by `xShift` and `yShift` along the *x*- and *y*-axis, respectively. The interpolation specified by `interpolate` is used for resampling the input image.

If you need to rotate the image around an arbitrary center (`xCenter`, `yCenter`) rather than the origin (0,0), you can compute `xShift` and `yShift` using the function `iplGetRotateShift` and then call `iplRotate()`. Alternatively, you can use the `iplRotateCenter` macro definition.

GetRotateShift

Computes shifts for iplRotate, given the rotation center and angle.

```
void iplGetRotateShift(double xCenter, double yCenter,
   double angle, double* xShift, double* yShift);
```

`xCenter, yCenter`	Coordinates of the rotation center for which you wish to compute the shifts.
`angle`	The angle (in degrees) to rotate the image around the point with coordinates (`xCenter`, `yCenter`).
`xShift, yShift`	Output parameters: the shifts along the *x*- and *y*-axes to be passed to `iplRotate()` in order to achieve rotation around the specified center (`xCenter`, `yCenter`) by the specified `angle`.

Discussion

Use the function `iplGetRotateShift()` if you wish to rotate an image around an arbitrary center (`xCenter`, `yCenter`) rather than the origin (0,0). Just pass the rotation center (`xCenter`, `yCenter`) and the angle of rotation to `iplGetRotateShift()`, and the function will recompute the shifts `xShift`, `yShift`.

Calling `iplRotate()` with these `xShift` and `yShift` is equivalent to rotating the image around the center (`xCenter`, `yCenter`).

Example 11-2 Rotating an Image

```
int example111( void ) {
   IplImage *imga, *imgb;
   const int width = 5, height = 5;
   __try {
      int i;
```

continued ☞

Example 11-2 Rotating an Image (continued)

```
            double xshift=0, yshift=0;
            imga = iplCreateImageHeader(
               1, 0, IPL_DEPTH_8U, "GRAY", "GRAY",
               IPL_DATA_ORDER_PIXEL, IPL_ORIGIN_TL,
               IPL_ALIGN_DWORD, width, height, NULL, NULL,
               NULL, NULL);
            if( NULL == imga ) return 0;
            imgb = iplCreateImageHeader(
               1, 0, IPL_DEPTH_8U, "GRAY", "GRAY",
               IPL_DATA_ORDER_PIXEL, IPL_ORIGIN_TL,
               IPL_ALIGN_DWORD, width, height, NULL, NULL,
               NULL, NULL);
            if( NULL == imgb ) return 0;
            // Create with filling
            iplAllocateImage( imga, 1, 0 );
            if( NULL == imga->imageData ) return 0;
            // Make horizontal line
            for( i=0; i<width; i++)
               (imga->imageData + 2*imga->widthStep)[i] =
               (uchar)7;
            iplAllocateImage( imgb, 0, 0 );
            if( NULL == imgb->imageData ) return 0;
            // Rotate by 45 degrees around point(2,2)
            iplGetRotateShift(2.0,2.0,45.0, &xshift, &yshift);
            iplRotate( imga, imgb, 45.0, xshift, yshift,
                      IPL_INTER_LINEAR );
            // Check if an error occurred
            if( iplGetErrStatus() != IPL_StsOk ) return 0;
         }
        __finally {
          iplDeallocate(imga, IPL_IMAGE_HEADER|IPL_IMAGE_DATA);
          iplDeallocate(imgb, IPL_IMAGE_HEADER|IPL_IMAGE_DATA);
        }
        return IPL_StsOk == iplGetErrStatus();
      }
```

iplRotateCenter

This function-like macro allows to rotate an image around the given center.

```
iplRotateCenter(srcImage, dstImage, angle, xCenter,
   yCenter, interpolate);
```

`srcImage`	The source image.
`dstImage`	The destination image.
`angle`	The angle (in degrees) to rotate the image around the point with coordinates (`xCenter`, `yCenter`).
`xCenter, yCenter`	Coordinates of the center of rotation.
`interpolate`	The type of interpolation to perform for resampling the input image. The following modes are supported:

`IPL_INTER_NN`	Nearest neighbor.
`IPL_INTER_LINEAR`	Linear interpolation.
`IPL_INTER_CUBIC`	Cubic interpolation.
`+IPL_SMOOTH_EDGE`	Smooth edges of an image. Can be added to interpolation by using bitwise logical OR.

Discussion

Use the macro `iplRotateCenter` to rotate an image around an arbitrary center. The rotation center coordinates (`xCenter`, `yCenter`) are passed as arguments, and the call to the auxiliary function that recomputes the shifts is hidden.

Example 11-3 Using Macro Definition to Rotate an Image

```
int RotateCenter( void ) {

   IplImage *imga = iplCreateImageJaehne(IPL_DEPTH_8U, 5, 5);
   IplImage *imgb = iplCloneImage( imga );
   IPLStatus st;

   // Rotate by 45 about point(2,2)
   iplRotateCenter( imga, imgb, 45, 2, 2, IPL_INTER_NN );
   st = iplGetErrStatus();

   iplDeallocate( imga, IPL_IMAGE_ALL );
   iplDeallocate( imgb, IPL_IMAGE_ALL );

   return IPL_StsOk == st;
}
```

Mirror

Mirrors an image about a horizontal or vertical axis.

```
void iplMirror(IplImage* srcImage, IplImage* dstImage,
   int flipAxis);
```

srcImage	The source image.
dstImage	The resultant image.
flipAxis	Specifies the axis to mirror the image.<br>Use the following values to specify the axes:<br>0 for the horizontal axis, 1 for the vertical axis,<br>-1 for both horizontal and vertical axes.

Discussion

The function `iplMirror()` mirrors or flips the source image *srcImage* about a horizontal or vertical axis or both.

Warping

This section describes shearing and warping functions of the Image Processing Library. These functions have been added in release 2.0. They perform the following operations:

- affine warping (the functions `iplWarpAffine` and `iplShear`)
- bilinear warping (`iplWarpBilinear`, `iplWarpBilinearQ`)
- perspective warping (`iplWarpPerspective`, `iplWarpPerspectiveQ`).

Affine warping operations are more complex and more general than resizing or rotation. A single call to `iplWarpAffine()` can perform a rotation, resizing, and mirroring. (This can require some matrix math on the part of the user to calculate the transform coefficients.)

Bilinear and *perspective* warping operations can be viewed as further generalizations of affine warping. They give you even more degrees of freedom in transforming the image. For example, an affine transformation always maps parallel lines to parallel lines, while bilinear and perspective transformations might not preserve parallelism; a bilinear transformation might even map straight lines to curves.

Unlike rotation or zooming, the warping functions do not necessarily map the rectangular ROI of the source image to a rectangle in the destination image. Affine warping functions map the rectangular ROI to a parallelogram; bilinear and perspective warping functions map the ROI to a general quadrangle.

To help you cope with the complex behavior of warping transformations, the library includes a number of auxiliary functions that compute the following warping parameters:

- coordinates of the four points to which the ROI’s vertices are mapped
- the bounding rectangle for the transformed ROI
- the transformation coefficients.

These auxiliary functions are described immediately after the function that performs the respective warping operation.

Shear

Performs a shear of the source image.

```
void iplShear(IplImage* srcImage, IplImage* dstImage, double xShear,
   double yShear, double xShift, double yShift, int interpolate);
```

`srcImage`	The source image.
`dstImage`	The resultant image.
`xShear, yShear`	The shear coefficients.
`xShift, yShift`	Additional shift values for the *x* and *y* directions.
`interpolate`	The type of interpolation to perform for resampling. Can be one of the following:

`IPL_INTER_NN`	Nearest neighbor.
`IPL_INTER_LINEAR`	Linear interpolation.
`IPL_INTER_CUBIC`	Cubic interpolation.
`+IPL_SMOOTH_EDGE`	Smooth edges of an image. Can be added to interpolation by using bitwise logical OR (see Appendix B for details).

Discussion

The function `iplShear()` performs a shear of the source image according to the following formulas:

$$x' = x + \texttt{xShear}\cdot y + \texttt{xShift}$$
$$y' = y + \texttt{yShear}\cdot x + \texttt{yShift}$$

where x and y denote the original pixel coordinates; x' and y' denote the pixel coordinates in the sheared image. This shear transform is a special case of affine transform performed by `iplWarpAffine` (see below).

The interpolation specified by *`interpolate`* is used for resampling the input image.

WarpAffine

Warps an image by an affine transform.

```
void iplWarpAffine(IplImage* srcImage, IplImage* dstImage,
   const double coeffs[2][3], int interpolate);
```

`srcImage`	The source image.
`dstImage`	The resultant image.
`coeffs`	The affine transform coefficients.
`interpolate`	The type of interpolation to perform for resampling. Can be one of the following:

`IPL_INTER_NN`	Nearest neighbor.
`IPL_INTER_LINEAR`	Linear interpolation.
`IPL_INTER_CUBIC`	Cubic interpolation.
`+IPL_SMOOTH_EDGE`	Smooth edges of an image. Can be added to interpolation by using bitwise logical OR (see Appendix B for details).

Discussion

The function `iplWarpAffine()` warps the source image by an affine transformation according to the following formulas:

$$x' = coeffs[0][0] \cdot x + coeffs[0][1] \cdot y + coeffs[0][2]$$
$$y' = coeffs[1][0] \cdot x + coeffs[1][1] \cdot y + coeffs[1][2]$$

where x and y denote the original pixel coordinates; x' and y' denote the pixel coordinates in the transformed image.

The interpolation specified by *`interpolate`* is used for resampling the input image. To compute the affine transform parameters, use the functions `iplGetAffineBound()`, `iplGetAffineQuad()` and `iplGetAffineTransform()`. These functions are described in the sections that follow.

GetAffineBound

Computes the bounding rectangle for ROI transformed by iplWarpAffine.

```
void iplGetAffineBound(IplImage* image, const double
   coeffs[2][3], double rect[2][2]);
```

image	The image to be passed to `iplWarpAffine()`.
coeffs	The `iplWarpAffine()` transform coefficients.
rect	Output array: the coordinates of vertices of the rectangle bounding the figure to which `iplWarpAffine()` maps *image*'s ROI.

Discussion

The function `iplGetAffineBound()` computes the coordinates of vertices of the smallest possible rectangle with horizontal and vertical sides that bounds the figure to which `iplWarpAffine()` maps *image*'s ROI.

GetAffineQuad

Computes the quadrangle to which the image ROI would be mapped by iplWarpAffine.

```
void iplGetAffineQuad(IplImage* image, const double
   coeffs[2][3], double quad[4][2]);
```

image	The image to be passed to `iplWarpAffine()`.
coeffs	The affine transform coefficients.

`quad`	Output array: coordinates of the quadrangle to which the *`image`*'s ROI would be mapped by `iplWarpAffine()`.

Discussion

The function `iplGetAffineQuad()` computes coordinates of the quadrangle to which the *`image`*'s ROI would be mapped by `iplWarpAffine()` with the transform coefficients *`coeffs`*.

GetAffineTransform

Computes the iplWarpAffine coefficients, given the ROI-quadrangle pair.

```
void iplGetAffineTransform(IplImage* image, double
   coeffs[2][3], const double quad[4][2]);
```

`image`	The image to be passed to `iplWarpAffine()`.
`coeffs`	Output array: the affine transform coefficients.
`quad`	Coordinates of the 4 points to which the *`image`*'s ROI vertices would be mapped by `iplWarpAffine()`.

Discussion

The function `iplGetAffineTransform()` computes the coefficients of `iplWarpAffine()` transform, given the vertices of the quadrangle to which the *`image`*'s ROI would be mapped by `iplWarpAffine()` with these coefficients.

WarpBilinear
WarpBilinearQ

Warps an image by a bilinear transform.

```
void iplWarpBilinear(IplImage* srcImage, IplImage* dstImage,
   const double coeffs[2][4], int warpFlag, int interpolate);

void iplWarpBilinearQ(IplImage* srcImage, IplImage* dstImage,
   const double quad[4][2], int warpFlag, int interpolate);
```

srcImage	The source image.
dstImage	The resultant image.
coeffs	Array with bilinear transform coefficients.
warpFlag	A flag: either `IPL_R_TO_Q` (ROI to quadrangle) or `IPL_Q_TO_R` (quadrangle to ROI). See *Discussion*.
interpolate	The type of interpolation to perform for resampling. Can be one of the following:

`IPL_INTER_NN`	Nearest neighbor.
`IPL_INTER_LINEAR`	Linear interpolation.
`IPL_INTER_CUBIC`	Cubic interpolation.
`+IPL_SMOOTH_EDGE`	Smooth edges of an image. Can be added to interpolation by using bitwise logical OR (see Appendix B for details).

quad	Array of coordinates of the reference quadrangle vertices. If *warpFlag* is `IPL_R_TO_Q`, the rectangular ROI of the source image is mapped to the reference quadrangle. If *warpFlag* is `IPL_Q_TO_R`, the source quadrangle is mapped to the rectangular ROI of the destination image.

Discussion

The functions `iplWarpBilinear()` and `iplWarpBilinearQ()` warp the source image by a bilinear transformation according to the following formulas:

$$x' = c_{00} \cdot xy + c_{01} \cdot x + c_{02} \cdot y + c_{03}$$
$$y' = c_{10} \cdot xy + c_{11} \cdot x + c_{12} \cdot y + c_{13}$$

where x and y denote the original pixel coordinates; x' and y' denote the pixel coordinates in the transformed image.

The two functions differ in their third argument: `iplWarpBilinear()` uses a 2-by-4 input array of transform coefficients c_{mn} = `coeff[m][n]`, whereas `iplWarpBilinearQ()` computes the coefficients internally from the input array *`quad`* containing coordinates of the reference quadrangle.

If *`warpFlag`* is `IPL_R_TO_Q`, the functions transform the rectangular ROI of the source image into the reference quadrangle of the resultant image. If *`warpFlag`* is `IPL_Q_TO_R`, the functions transform the source quadrangle into the rectangular ROI of the resultant image.

The interpolation specified by *`interpolate`* is used for resampling the input image.

To compute the bilinear transform parameters, use the auxiliary functions: `iplGetBilinearBound()`, `iplGetBilinearQuad()` and `iplGetBilinearTransform()`. These functions are described in the sections that follow.

GetBilinearBound

Computes the bounding rectangle for ROI transformed by iplWarpBilinear.

```
void iplGetBilinearBound(IplImage* image, const double
    coeffs[2][4], double rect[2][2]);
```

`image`	The image to be passed to `iplWarpBilinear()`.
`coeffs`	The bilinear transform coefficients.
`rect`	Output array: the coordinates of vertices of the rectangle bounding the figure to which `iplWarpBilinear()` maps `image`'s ROI.

Discussion

The function `iplGetBilinearBound()` computes the coordinates of vertices of the smallest possible rectangle with horizontal and vertical sides that bounds the figure to which `iplWarpBilinear()` maps `image`'s ROI.

GetBilinearQuad

Computes the quadrangle to which the image ROI would be mapped by iplWarpBilinear.

```
void iplGetBilinearQuad(IplImage* image, const double
    coeffs[2][4], double quad[4][2]);
```

`image`	The image to be passed to `iplWarpBilinear()`.
`coeffs`	The bilinear transform coefficients.

quad	Output array: coordinates of the quadrangle to which the *image*'s ROI would be mapped by iplWarpBilinear().

Discussion

The function iplGetBilinearQuad() computes coordinates of the quadrangle to which the *image*'s ROI would be mapped by iplWarpBilinear() with the transform coefficients *coeffs*.

GetBilinearTransform

Computes the iplWarpBilinear coefficients, given the ROI-quadrangle pair.

```
void iplGetBilinearTransform(IplImage* image, double
   coeffs[2][4], const double quad[4][2]);
```

image	The image to be passed to iplWarpBilinear().
coeffs	Output array: the bilinear transform coefficients.
quad	Coordinates of the 4 points to which the *image*'s ROI vertices would be mapped by iplWarpBilinear().

Discussion

The function iplGetBilinearTransform() computes the iplWarpBilinear() transform coefficients, given the vertices of the quadrangle to which the *image*'s ROI would be mapped by iplWarpBilinear() with these coefficients.

WarpPerspective
WarpPerspectiveQ

Warps an image by a perspective transform.

```
void iplWarpPerspective(IplImage* srcImage, IplImage* dstImage,
   const double coeffs[3][3], int warpFlag, int interpolate);
```

```
void iplWarpPerspectiveQ(IplImage* srcImage, IplImage* dstImage,
   const double quad[4][2], int warpFlag, int interpolate);
```

srcImage	The source image.
dstImage	The resultant image.
coeffs	Array with perspective transform coefficients.
warpFlag	A flag: either IPL_R_TO_Q (ROI to quadrangle) or IPL_Q_TO_R (quadrangle to ROI). See *Discussion*.
interpolate	The type of interpolation to perform for resampling. Can be one of the following:
	IPL_INTER_NN — Nearest neighbor.
	IPL_INTER_LINEAR — Linear interpolation.
	IPL_INTER_CUBIC — Cubic interpolation.
	+IPL_SMOOTH_EDGE — Smooth edges of an image. Can be added to interpolation by using bitwise logical OR (see Appendix B for details).
quad	Array of coordinates of the reference quadrangle vertices. If *warpFlag* is IPL_R_TO_Q, the rectangular ROI of the source image is mapped to the reference quadrangle. If *warpFlag* is IPL_Q_TO_R, the source quadrangle is mapped to the rectangular ROI of the destination image.

Discussion

The functions iplWarpPerspective() and iplWarpPerspectiveQ() warp the source image by a perspective transformation according to the following formulas:

$$x' = (c_{00}\cdot x + c_{01}\cdot y + c_{02})/(c_{20}\cdot x + c_{21}\cdot y + c_{22})$$
$$y' = (c_{10}\cdot x + c_{11}\cdot y + c_{12})/(c_{20}\cdot x + c_{21}\cdot y + c_{22})$$

where x and y denote the original pixel coordinates; x' and y' denote the pixel coordinates in the transformed image.

The two functions differ in their third argument: iplWarpPerspective() uses a 3-by-3 input array of transform coefficients c_{mn} = *coeff*[*m*][*n*], whereas iplWarpPerspectiveQ() computes the coefficients internally from the input array *quad* containing coordinates of the reference quadrangle.

If *warpFlag* is IPL_R_TO_Q, the functions transform the rectangular ROI of the source image into the reference quadrangle of the resultant image. If *warpFlag* is IPL_Q_TO_R, the functions transform the source quadrangle into the rectangular ROI of the resultant image.

The interpolation specified by *interpolate* is used for resampling the input image.

To compute the perspective transform parameters, use these auxiliary functions: iplGetPerspectiveBound(), iplGetPerspectiveQuad() and iplGetPerspectiveTransform(). They are described in the sections that follow.

GetPerspectiveBound

Computes the bounding rectangle for ROI transformed by iplWarpPerspective.

```
void iplGetPerspectiveBound(IplImage* image, const double
    coeffs[3][3], double rect[2][2]);
```

image	The image to be passed to `iplWarpPerspective()`.
coeffs	The perspective transform coefficients.
rect	Output array: the coordinates of vertices of the rectangle bounding the figure to which `iplWarpPerspective()` maps *image*'s ROI.

Discussion

The function `iplGetPerspectiveBound()` computes the coordinates of vertices of the smallest possible rectangle with horizontal and vertical sides that bounds the figure to which `iplWarpPerspective()` maps *image*'s ROI.

GetPerspectiveQuad

Computes the quadrangle to which the ROI is mapped by iplWarpPerspective.

```
void iplGetPerspectiveQuad(IplImage* image, const double
    coeffs[3][3], double quad[4][2]);
```

image	The image to be passed to `iplWarpPerspective()`.
coeffs	The perspective transform coefficients.

quad	Output array: coordinates of the quadrangle to which the *image*'s ROI would be mapped by `iplWarpPerspective()`.

Discussion

The function `iplGetPerspectiveQuad()` computes coordinates of the quadrangle to which the *image*'s ROI would be mapped by `iplWarpPerspective()` with the transform coefficients *coeffs*.

GetPerspectiveTransform

Computes the coefficients of iplWarpPerspective, given the ROI-quadrangle pair.

```
void iplGetPerspectiveTransform(IplImage* image, double
   coeffs[3][3], const double quad[4][2]);
```

image	The image to be passed to `iplWarpPerspective()`.
coeffs	Output array: perspective transform coefficients.
quad	Coordinates of the 4 points to which the *image*'s ROI vertices would be mapped by `iplWarpPerspective()`.

Discussion

The function `iplGetPerspectiveTransform()` computes the `iplWarpPerspective()` transform coefficients, given the vertices of the quadrangle to which the *image*'s ROI would be mapped by `iplWarpBilinear()` with these coefficients.

Arbitrary Transforms

To perform special geometric transforms not covered in the above sections, the Image Processing Library includes the `iplRemap()` function. Unlike other geometric transform functions, `iplRemap()` uses coordinate tables supplied by the application. For each pixel in the destination image, you have to provide coordinates of the source image's point which you would like to be mapped to that destination pixel.

Remap

Re-maps the image using a coordinate look-up table.

```
void iplRemap(IplImage* srcImage, IplImage* xMap,
   IplImage * yMap, IplImage* dstImage,
   int interpolate );
```

srcImage	The source image.
dstImage	The resultant image.
xMap	One-channel 32-bit floating-point image storing the table of *x*-coordinates.
yMap	One-channel 32-bit floating-point image storing the table of *y*-coordinates.
interpolate	The type of interpolation to perform for resampling. Can be one of the following:

IPL_INTER_NN	Nearest neighbor.
IPL_INTER_LINEAR	Linear interpolation.
IPL_INTER_CUBIC	Cubic interpolation.

Discussion

The function `iplRemap()` maps the image *`srcImage`* to *`dstImage`* using a coordinate table supplied by the application in the images *`xMap`* and *`yMap`*. To each pixel in the destination image *`dstImage`*, the function assigns the value taken from the point (*x*,*y*) in the source image; the coordinates *x* and *y* are retrieved from the locations in *`xMap`* and *`yMap`* corresponding to the destination pixel.

Your application has to compute the floating-point coordinates and store them in *`xMap`* and *`yMap`* prior to calling `iplRemap()`; see Example 11-2.

Data order and bit depth of *`srcImage`* and *`dstImage`* must be the same. The function supports source and destination images with 1-bit, 8-bit unsigned, and 16-bit unsigned pixel channels. ROIs and tiling of *`srcImage`* and *`dstImage`* are supported. Mask is not directly supported. For masking some of the image pixels, you can just specify the corresponding *x* and *y* values that are outside the source image's ROI.

Example 11-4 Re-mapping an Image

```
int example_remap( void ) {
   const int width = 8, height = 8;
   int x, y; float norm;
   /// source and destination images: 8u
   IplImage *src = iplCreateImageJaehne(IPL_DEPTH_8U,
                   width,height);
   IplImage *dst = iplCreateImageHeader (
      1, 0, IPL_DEPTH_8U, "GRAY", "GRAY",
      IPL_DATA_ORDER_PIXEL, IPL_ORIGIN_TL,
      IPL_ALIGN_DWORD, width, height, NULL,
      NULL, NULL, NULL );
   /// create images for x and y coordinates
   IplImage *xmap = iplCreateImageJaehne(IPL_DEPTH_32F,
                    width, height);
   IplImage *ymap = iplCloneImage( xmap );
```

continued ☞

Example 11-4 Re-mapping an Image (continued)

```
/// allocate memory destination with zero data
   iplAllocateImage( dst, 1, 0 );
   /// provide the x and y coordinates
   /// these coords map the image to an identical one
   for( y=0; y<height; ++y ) {
      float yy = (float)y;
      for( x=0; x<width; ++x ) {
         float xx = (float)x;
         iplPutPixel( xmap, x, y, &xx );
         iplPutPixel( ymap, x, y, &yy );
      }
   }
   /// now remap to get the same image
   iplRemap( src, xmap, ymap, dst, IPL_INTER_LINEAR );
   /// find max abs difference, should be 0
   norm = (float)iplNorm( src, dst, IPL_C );
   /// deallocate images
   iplDeallocate( xmap, IPL_IMAGE_ALL );
   iplDeallocate( ymap, IPL_IMAGE_ALL );
   iplDeallocate( src, IPL_IMAGE_ALL );
   iplDeallocate( dst, IPL_IMAGE_ALL );
   return IPL_StsOk == iplGetErrStatus() && norm == 0;
}
```

Image Statistics Functions

12

This chapter describes the Image Processing Library functions that allow you to compute the following statistical parameters of an image:

- the C, L_1, and L_2 norms of the image pixel values
- spatial moments of order 0 to 3
- central moments of order 0 to 3
- minimum and maximum pixel values (for floating-point data only)

Table 12-1 lists the image statistics functions.

Table 12-1 Image Statistics Functions

Group	Function Name	Description
Norms	`iplNorm`	Computes the C, L_1, or L_2 norm of pixel values.
Moments	`iplMoments`	Computes all image moments of order 0 to 3.
	`iplGetCentralMoment`<br>`iplGetSpatialMoment`	Return image moments computed by `iplMoments()`.
	`iplGetNormalizedCentralMoment`<br>`iplGetNormalizedSpatialMoment`	Return normalized image moments computed by `iplMoments()`.
	`iplCentralMoment`<br>`iplSpatialMoment`	Compute an image moment of the specified order.
	`iplNormalizedCentralMoment`<br>`iplNormalizedSpatialMoment`	Compute a normalized image moment of the specified order.
Cross-correlation	`iplNormCrossCorr`	Computes the normalized cross-correlation of an image and a template.
Minimum and maximum	`iplMinMaxFP`	Retrieves the actual minimum and maximum pixel values in an image with 32-bit floating-point data.

Image Norms

The `iplNorm()` function described in this section allows you to compute the following norms of the image pixel values:

- L_1 norm (the sum of absolute pixel values)
- L_2 norm (the square root of the sum of squared pixel values)
- *C* norm (the largest absolute pixel value).

This function also helps you compute the norm of differences in pixel values of two input images as well as the relative error for two input images.

Norm

Computes the norm of pixel values or of differences in pixel values of two images.

```
double iplNorm(IplImage* srcImageA, IplImage* srcImageB,
int normType);
```

srcImageA	The first source image.
srcImageB	The second source image.
normType	Specifies the norm type. Can be `IPL_C`, `IPL_L1`, or `IPL_L2`; if the *srcImageB* pointer is not `NULL`, the *normType* argument can also be `IPL_RELATIVEC`, `IPL_RELATIVEL1`, or `IPL_RELATIVEL2`.

Discussion

You can use the `iplNorm()` function to compute the following norms of pixel values:

(1) the norm of `srcImageA` pixel values, $||a||$

(2) the norm of differences of the source images' pixel values, $||a - b||$

(3) the relative error $||a - b|| / ||b||$ (see formulas below).

Let $a = \{a_k\}$ and $b = \{b_k\}$ be vectors containing pixel values of `srcImageA` and `srcImageB`, respectively (all channels are used except alpha channel).

(1) If the `srcImageB` pointer is `NULL`, the function returns the norm of `srcImageA` pixel values:

$||a||_{L_1} = \Sigma_k |a_k|$ for `normType` = `IPL_L1`

$||a||_{L_2} = (\Sigma_k |a_k|^2)^{1/2}$ for `normType` = `IPL_L2`

$||a||_C = \max_k |a_k|$ for `normType` = `IPL_C`.

(2) If the `srcImageB` pointer is not `NULL`, the function returns the norm of differences of `srcImageA` and `srcImageB` pixel values:

$||a - b||_{L_1} = \Sigma_k |a_k - b_k|$ for `normType` = `IPL_L1`

$||a - b||_{L_2} = (\Sigma_k |a_k - b_k|^2)^{1/2}$ for `normType` = `IPL_L2`

$||a - b||_C = \max_k |a_k - b_k|$ for `normType` = `IPL_C`.

(3) If `normType` is `IPL_RELATIVEC`, `IPL_RELATIVEL1`, or `IPL_RELATIVEL2`, the `srcImageB` pointer must not be `NULL`.
The function first computes the norm of differences, as defined in (2). Then this norm is divided by the norm of b, and the function returns the relative error $||a - b|| / ||b||$.

Return Value

The computed norm or relative error in double floating-point format.

Example 12-1 Computing the Norm of Pixel Values

```
int example51( void ) {
  IplImage *imga, *imgb;
  const int width = 4;
  const int height = 4;
  double norm;
  __try {
    imga = iplCreateImageHeader(
       1, 0, IPL_DEPTH_8U, "GRAY", "GRAY",
       IPL_DATA_ORDER_PIXEL, IPL_ORIGIN_TL,
       IPL_ALIGN_QWORD, height, width, NULL, NULL,
       NULL, NULL);
    if( NULL == imga ) return 0;
    imgb = iplCreateImageHeader(
       1, 0, IPL_DEPTH_8U, "GRAY", "GRAY",
       IPL_DATA_ORDER_PIXEL, IPL_ORIGIN_TL,
       IPL_ALIGN_QWORD, height, width, NULL, NULL,
       NULL, NULL);
    if( NULL == imgb ) return 0;
    iplAllocateImage( imga, 1, 127 );
    if( NULL == imga->imageData ) return 0;
    iplAllocateImage( imgb, 1, 1 );
    if( NULL == imgb->imageData ) return 0;

    norm = iplNorm( imga, imgb, IPL_RELATIVEC );
    // Check if an error occurred
    if( iplGetErrStatus() != IPL_StsOk ) return 0;
  }
  __finally {
    iplDeallocate(imga,IPL_IMAGE_HEADER|IPL_IMAGE_DATA);
    iplDeallocate(imgb,IPL_IMAGE_HEADER|IPL_IMAGE_DATA);
  }
  return IPL_StsOk == iplGetErrStatus();
}
```

Image Moments

Spatial and central moments are important statistical characteristics of an image. The spatial moment $M_U(m,n)$ and central moment $U_U(m,n)$ are defined as follows:

$$M_U(m,n) = \sum_{j=0}^{nRows-1} \sum_{k=0}^{nCols-1} x_k^m y_j^n P_{j,k}$$

$$U_U(m,n) = \sum_{j=0}^{nRows-1} \sum_{k=0}^{nCols-1} (x_k - x_0)^m (y_j - y_0)^n P_{j,k}$$

where the summation is performed for all rows and columns in the image; $P_{j,k}$ are pixel values; x_k and y_j are pixel coordinates; m and n are integer power exponents; x_0 and y_0 are the gravity center's coordinates:

$x_0 = M_U(1,0)/M_U(0,0)$

$y_0 = M_U(0,1)/M_U(0,0)$.

The sum of exponents $m + n$ is called the moment order. The library functions support moments of order 0 to 3 (that is, $0 \le m + n \le 3$).

In the Image Processing Library image moments are stored in structures of the `IplMomentState` type. The type declaration is given below.

IplMomentState Structure Definition

```
typedef struct {
   double scale   /* scaling factor for the moment */
   double value   /* the moment */
} ownMoment;
typedef ownMoment IplMomentState[4][4];
```

Moments

Computes all image moments of order 0 to 3.

```
void iplMoments(IplImage* image, IplMomentState mState);
```

image	The image for which the moments will be computed.
mState	The structure for storing the image moments.

Discussion

The function `iplMoments()` computes all spatial and central moments of order 0 to 3 for the *image*. The moments and the corresponding scaling factors are stored in the *mState* structure. To retrieve a particular moment value, use the functions described in the sections that follow.

GetSpatialMoment

Returns a spatial moment computed by iplMoments.

```
double iplGetSpatialMoment(IplMomentState mState, int
mOrd, int nOrd);
```

mState	The structure storing the image moments.
mOrd, nOrd	The integer exponents *m* and *n* (see the moment definition in the beginning of this section). These arguments must satisfy the condition $0 \le mOrd + nOrd \le 3$.

Discussion

The function `iplGetSpatialMoment()` returns the spatial moment $M_U(m,n)$ previously computed by the `iplMoments()` function.

GetCentralMoment

Returns a central moment computed by iplMoments.

```
double iplGetCentralMoment(IplMomentState mState, int
mOrd, int nOrd);
```

mState	The structure storing the image moments.
mOrd, nOrd	The integer exponents *m* and *n* (see the moment definition in the beginning of this section). These arguments must satisfy the condition $0 \le$ *mOrd* + *nOrd* ≤ 3.

Discussion

The function `iplGetCentralMoment()` returns the central moment $U_U(m,n)$ previously computed by the `iplMoments()` function.

GetNormalizedSpatialMoment

Returns the normalized spatial moment computed by iplMoments.

```
double iplGetNormalizedSpatialMoment(IplMomentState
mState, int mOrd, int nOrd);
```

`mState`	The structure storing the image moments.
`mOrd, nOrd`	The integer exponents *m* and *n* (see the moment definition in the beginning of this section). These arguments must satisfy the condition 0 ≤ `mOrd` + `nOrd` ≤ 3.

Discussion

The function `iplGetNormalizedSpatialMoment()` returns the normalized spatial moment $M_U(m,n)/(nCols^m \cdot nRows^n)$, where $M_U(m,n)$ is the spatial moment previously computed by the `iplMoments()` function, `nCols` and `nRows` are the numbers of columns and rows, respectively.

GetNormalizedCentralMoment

Returns the normalized central moment computed by iplMoments.

```
double iplGetNormalizedCentralMoment(IplMomentState
mState, int mOrd, int nOrd);
```

`mState`	The structure storing the image moments.
`mOrd, nOrd`	The integer exponents *m* and *n* (see the moment definition in the beginning of this section). These arguments must satisfy the condition 0 ≤ `mOrd` + `nOrd` ≤ 3.

Discussion

The function `iplGetNormalizedCentralMoment()` returns the normalized central moment $U_U(m,n)/(nCols^m \cdot nRows^n)$, where $U_U(m,n)$ is the central moment previously computed by the `iplMoments()` function, `nCols` and `nRows` are the numbers of columns and rows, respectively.

SpatialMoment

Computes a spatial moment.

```
double iplSpatialMoment(IplImage* image, int mOrd, int
nOrd);
```

image	The image for which the moment will be computed.
mOrd, nOrd	The integer exponents m and n (see the moment definition in the beginning of this section). These arguments must satisfy the condition $0 \le$ *mOrd* + *nOrd* ≤ 3.

Discussion

The function `iplSpatialMoment()` computes the spatial moment $M_U(m,n)$ for the *image*.

CentralMoment

Computes a central moment.

```
double iplCentralMoment(IplImage* image, int mOrd, int
nOrd);
```

image	The image for which the moment will be computed.
mOrd, nOrd	The integer exponents m and n (see the moment definition in the beginning of this section). These arguments must satisfy the condition $0 \le$ *mOrd* + *nOrd* ≤ 3.

Discussion

The function `iplCentralMoment()` computes the central moment $U_U(m,n)$ for the *`image`*.

NormalizedSpatialMoment

Computes a normalized spatial moment.

```
double iplNormalizedSpatialMoment(IplImage* image, int
mOrd, int nOrd);
```

`image`	The image for which the moment will be computed.
`mOrd, nOrd`	The integer exponents *m* and *n* (see the moment definition in the beginning of this section). These arguments must satisfy the condition $0 \le$ *`mOrd`* + *`nOrd`* ≤ 3.

Discussion

The function `iplNormalizedSpatialMoment()` computes the normalized spatial moment $M_U(m,n)/(nCols^m \cdot nRows^n)$ for the *`image`*. Here $M_U(m,n)$ is the spatial moment, *`nCols`* and *`nRows`* are the numbers of pixel columns and rows, respectively.

NormalizedCentralMoment

Computes a normalized central moment.

```
double iplNormalizedCentralMoment(IplImage* image, int
mOrd, int nOrd);
```

`image`	The image for which the moment will be computed.
`mOrd, nOrd`	The integer exponents *m* and *n* (see the moment definition in the beginning of this section). These arguments must satisfy the condition $0 \leq$ `mOrd` $+$ `nOrd` ≤ 3.

Discussion

The function `iplNormalizedCentralMoment()` computes the normalized central moment $U_U(m,n)/($`nCols`$^m \cdot$`nRows`$^n)$ for the `image`. Here $U_U(m,n)$ is the central moment, `nCols` and `nRows` are the numbers of pixel columns and rows, respectively.

Cross-Correlation

This section describes the `iplNormCrossCorr()` function that allows you to compute the cross-correlation of an image and a template (another image). The cross-correlation values are image similarity measures: the higher cross-correlation at a particular pixel, the more similarity between the template and the image in the neighborhood of the pixel.

The mathematical definition of the cross-correlation $R_{tx}(r,c)$ between a template and an image at the pixel in row r and column c is given by this equation:

$$R_{tx}(r,c) = \sum_{j=0}^{tplRows-1} \sum_{i=0}^{tplCols-1} t(j,i)\; x(r+j-tplRows/2, c+i-tplCols/2)$$

where $x(r,c)$ is the image's pixel value in row r and column c, and $t(r,c)$ is the template's pixel value; the template size is *tplCols* x *tplRows*.

The `iplNormCrossCorr()` function of the Image Processing Library computes *normalized* cross-correlation values, $\rho_{tx}(r,c)$, defined as follows:

$$\rho_{tx}(r,c) = A \frac{R_{tx}(r,c)}{\sqrt{R_{xx}(r,c) R_{tt}(tplRows/2, tplCols/2)}}\,.$$

Here A is a factor for scaling the computed values to the full range of pixel values in the destination image; R_{xx} and R_{tt} denote the auto-correlation of the image and the template, respectively:

$$R_{xx}(r,c) = \sum_{j=r-(tplRows-1)/2}^{r+(tplRows-1)/2} \sum_{i=c-(tplCols-1)/2}^{c+(tplCols-1)/2} x_{j,i} x_{j,i}$$

$$R_{tt}(tplRows/2, tplCols/2) = \sum_{j=0}^{tplRows-1} \sum_{i=0}^{tplCols-1} t_{j,i} t_{j,i}\,.$$

NormCrossCorr

Computes normalized cross-correlation between an image and a template.

```
IPLStatus iplNormCrossCorr (IplImage* srcImage,
   IplImage* tplImage, IplImage* dstImage);
```

`srcImage, tplImage`	The source and template images.
`dstImage`	The destination image.

Discussion

For each pixel in `srcImage`, the function `iplNormCrossCorr()` computes the normalized cross-correlation value $\rho_{tx}(r,c)$ with the template `tplImage`, and stores the computed value in the corresponding pixel of the output image `dstImage`. The template anchor for matching the image pixel is always at the geometric center of the template. (See the formula for ρ_{tx} on the previous page.)

All three images passed to `iplNormCrossCorr()` must have the same data order (pixels or planes), origin (top-left or bottom-left), number of channels, alpha channel number, and COI number. The function supports images with 8-bit and 16-bit pixel data (both signed and unsigned) as well as 32-bit signed and 32-bit floating-point data.

Both `srcImage` and `dstImage` can have any combination of ROIs (rectangular ROIs, mask ROIs, and COIs). If you set any of these ROIs, the function will update pixels of `dstImage` only in the intersection of all applicable ROIs.

The `tplImage`'s mask, even if present, has no effect on the results.

The source and destination images can be either tiled or non-tiled. The template image must be non-tiled only.

The function returns `IPL_StsOK` on success, and an error status code on failure.

Minimum and Maximum

The `iplMinMaxFP()` function described in this section allows you to compute the minimum and maximum pixel values for an image with 32-bit floating-point data.

MinMaxFP

Retrieves the minimum and maximum floating-point pixel value.

```
IPLStatus iplMinMaxFP (const IplImage* image, float* min,
float* max);
```

image	The image with 32-bit floating-point pixel data for which the minimum and maximum values will be retrieved.
min, max	The output values: minimum and maximum.

Discussion

The function `iplMinMaxFP()` stores in *min* and *max* the actual minimum and maximum pixel values of the *image*. The function returns `IPL_StsOK` on success, and an error status code on failure.

User Defined Functions

13

This chapter describes library functions that enable users to create their own image processing functions and make calls to them from application programs. You can define functions that perform point operations either on each channel value of processed pixels of an image separately, or on all channel values simultaneously. Both integer and floating-point image data can be processed.

To introduce your own image processing function, you must first define it as one of the following types:

`IplUserFunc`	For functions that operate on images with integer data and process each channel value of a pixel separately.
`IplUserFuncFP`	For functions that operate on images with all data types and process each channel value of a pixel separately.
`IplUserFuncPixel`	For functions that operate on images with all data types and process all channel values of a pixel simultaneously.

Afterwards you can call your own functions by using the respective library functions `IplUserProcess()`, `IplUserProcessFP()`, or `IplUserProcessPixel()`, described later in this chapter.

UserFunc

The type of user-defined functions that perform point operations on a separate channel value of a pixel (for images with integer data).

The prototype specified by the callback function of type `IplUserFunc` must be as follows:

```
typedef int (__STDCALL *IplUserFunc)(int src);
```

src	The source pixel channel value converted to `int` type.

Discussion

The user function defined with the above prototype must take the channel value *src* of type `int` as input and return the computed destination pixel channel value also as `int` type. To use the function for image processing, its name must be passed to the calling function `iplUserProcess()` as the last parameter in the arguments list.

The saturation of the returned result to the destination data range is done by the calling function.

The user function of type `IplUserFunc` may call `IPL_ERROR` to set the IPL error status.

See `iplUserProcess()` for more information.

UserFuncFP

The type of user-defined functions that perform point operations on a separate channel value of a pixel (for images with all data types).

The prototype specified by the callback function of type `IplUserFuncFP` must be as follows:

```
typedef float (__STDCALL *IplUserFuncFP)(float src);
```

src	The source pixel channel value converted to `float` type.

Discussion

The user function defined with the above prototype must take the `float` channel value *src* as input and return the computed destination pixel channel value also as `float`. To use the function for image processing, its name must be passed to the calling function `iplUserProcessFP()` as the last parameter in the arguments list.
The saturation of the returned result to the destination data range is done by the calling function in case when the source and destination images contain integer data.

The user function of type `IplUserFuncFP` may call `IPL_ERROR` to set the IPL error status.

See `iplUserProcessFP()` for more information.

UserFuncPixel

The type of user-defined functions that perform point operations simultaneously on all channel values of a pixel in an image.

The prototype specified by the callback function of type `IplUserFuncPixel` must be as follows:

```
typedef void (__STDCALL *IplUserFuncPixel)(IplImage*
   srcImage, void* srcPixel, IplImage* dstImage, void*
   dstPixel);
```

srcImage	The source image header (used by the function to determine the source image depth and number of channels).
dstImage	The destination image header (used by the function to determine the destination image depth and number of channels).
srcPixel	Pointer to the array of channel values of the source pixel.
dstPixel	Pointer to the array of channel values of the destination pixel.

Discussion

Function of the type `IplUserFuncPixel` performs user-defined point operations on a source image pixel by processing all channel values of a given pixel simultaneously. The *srcPixel* and *dstPixel* pointers must be converted by the user function to arrays of source and destination channel values that have respective bit depths.

To use the function for image processing, its name must be passed to the calling function `iplUserProcessPixel()` as the last parameter in the arguments list.

If saturation of the computed result is necessary, it must be provided within the user function.

The user function of type `IplUserFuncPixel` may call `IPL_ERROR` to set the IPL error status.

See `iplUserProcessPixel()` for more information.

UserProcess

Calls user-defined function to separately process each channel value of pixels in an image with integer data.

```
void iplUserProcess( IplImage* srcImage, IplImage*
   dstImage, IplUserFunc cbFunc );
```

srcImage	The source image.
dstImage	The destination image.
cbFunc	The pointer to the user-defined function (of `IplUserFunc` type).

Discussion

The function `iplUserProcess()` scans pixels of a source image *srcImage*, retrieves respective channel values, and passes them to the user-defined function *cbFunc* for processing.
The source image must contain integer data of 8-, 16-, or 32-bit depth. Before passing channel values to *cbFunc* , the function `iplUserProcess()` converts them to `int` type.
After processing by *cbFunc*, the returned values are saturated to the destination data range, and written to the respective channel of the destination image *dstImage*. The saturation is done only for 8- or 16-bit

data. To perform saturation of 32-bit integer data, use `iplUserProcessFP()` function instead.

The function `iplUserProcess()` supports tiled images and images with rectangle ROI and mask ROI. The operations can be performed in-place. The source and destination images must contain data of the same bit depth and have the same number of processed channels.

Example 13-1 Image Channel Values Processing by User Defined Function

```
static int __STDCALL bw( int src ) {
   if( src < 127 ) return 0;
   return 255;
}

void UserFunc( void ) {

   IplImage *imga = iplCreateImageJaehne( IPL_DEPTH_8U,
     16, 5 );
   IplImage *imgb = iplCloneImage( imga );

   iplUserProcess( imga, imgb, bw );

   iplDeallocate( imga, IPL_IMAGE_ALL );
   iplDeallocate( imgb, IPL_IMAGE_ALL );

}
```

UserProcessFP

Calls user-defined function to separately process each channel value of pixels in images with all data types.

```
void iplUserProcessFP( IplImage* srcImage, IplImage*
   dstImage, IplUserFuncFP cbFunc );
```

srcImage	The source image.
dstImage	The destination image.
cbFunc	The pointer to the user-defined function (of `IplUserFuncFP` type).

Discussion

The function `iplUserProcessFP()` scans pixels of a source image *srcImage*, retrieves respective channel values, and passes them to the user-defined function *cbFunc* for processing. The source image can contain either integer data of 8-, 16-, 32-bit depth, or floating-point 32f data. Before passing channel values to *cbFunc* , the function `iplUserProcessFP()` converts them to `float` type. After processing by *cbFunc*, the returned values are saturated to the destination data range (except the case of 32f image data), and written to the respective channel of the destination image *dstImage*.

The function `iplUserProcessFP()` supports tiled images and images with rectangle ROI and mask ROI. The operations can be performed in-place. The source and destination images must contain data of the same bit depth and have the same number of processed channels.

UserProcessPixel

Calls user-defined function to simultaneously process channel values of pixels in an image.

```
void iplUserProcessPixel( IplImage* srcImage, IplImage*
   dstImage, IplUserFuncPixel cbFunc );
```

srcImage	The source image.
dstImage	The destination image.
cbFunc	The pointer to the user-defined function (of `IplUserFuncPixel` type).

Discussion

Use the function `iplUserProcessPixel()` if you want to call your own image processing function *cbFunc* of type `IplUserFuncPixel` that performs point operations using all channel values of a pixel.
For each pixel to be processed, the function `iplUserProcessPixel()` creates arrays of source and destination pixel channel values, and calls the function *cbFunc*, passing the pointers to these arrays as arguments. Thus, all channel values of a source image pixel are processed simultaneously.
After processing by *cbFunc*, the results are placed into the respective pixel channel values of the destination image *dstImage* without saturation.
When necessary, saturation should be provided by *cbFunc*.
On return from *cbFunc*, the function `iplUserProcessPixel()` checks `IplError()` status to see if an error has occurred.
The source image can contain either integer data of 8-, 16-, 32-bit depth, or floating-point 32f data. The bit depths and the number of channels in the source and destination images may be different. The function `iplUserProcessPixel()` supports tiled images and images with rectangle ROI and mask ROI. The channel ROI is not supported, it must be provided by the user function when necessary.

Example 13-2 Pixel Values Processing by User Defined Function

```
static void __STDCALL rgb2gray( IplImage* srcImage,
   void* srcPixel, IplImage* dstImage, void* dstPixel )
{
   uchar* src = (uchar*)srcPixel;
   uchar* dst = (uchar*)dstPixel;
   if( 1 != dstImage->nChannels ) {
     IPL_ERROR( IPL_BadNumChannels, "rgb2gray",
      "Output image must be one-channel image");
     return;
   }
   dst[0] = (uchar)( 0.212671 * src[0] +
      0.71516 * src[1] + 0.072169 * src[2] + 0.5 );
}
void exmRgb2Gray( void ) {
   const int side = 5;
   IplROI roi = { 1, 0, 0, side, side };
   IplImage *jmg, *dst, *src = iplCreateImageHeader(
      3, 0, IPL_DEPTH_8U, "RGBA", "BGRA",
      IPL_DATA_ORDER_PIXEL, IPL_ORIGIN_TL,
      IPL_ALIGN_DWORD, side, side, &roi, NULL,
      NULL, NULL);
   iplAllocateImage( src, 0, 0 );
   dst = iplCreateImageHeader(
      1, 0, IPL_DEPTH_8U, "GRAY", "GRAY",
      IPL_DATA_ORDER_PIXEL, IPL_ORIGIN_TL,
      IPL_ALIGN_DWORD, side, side, NULL, NULL,
      NULL, NULL);
   iplAllocateImage( dst, 1, 0 );
   jmg = iplCreateImageJaehne( IPL_DEPTH_8U, side, side );
   iplCopy( jmg, src );
   src->roi = 0;
   iplUserProcessPixel( src, dst, rgb2gray );
   iplDeallocate( jmg, IPL_IMAGE_ALL );
   iplDeallocate( dst, IPL_IMAGE_ALL );
   iplDeallocate( src, IPL_IMAGE_ALL );
}
```

This page is left blank for double-sided printing

This page is left blank for double-sided printing

Library Version

14

This chapter describes the function `iplGetLibVersion()` that returns the version number and other information about the Image Processing Library.

GetLibVersion

Returns information about the library version.

```
const IPLLibVersion* iplGetLibVersion(void);
```

Discussion

The function `iplGetLibVersion()` retrieves the following information about the Image Processing Library:

- major version number
- minor version number
- build number
- DLL or static library file name
- version number string
- internal version string
- build date string
- calling convention string

Return Value

The function returns the library information in the structure `IPLLibVersion`.

The `IPLLibVersion` structure is defined as follows:

```
typedef struct _IPLLibVersion {
  int    major;           /* e.g. 2     */
  int    minor;           /* e.g. 0     */
  int    build;           /* e.g. 1     */
  const char * Name;      /* "ipl6l.lib","iplm5.dll" */
  const char * Version;   /* e.g. "v2.00"  */
  const char * InternalVersion;  /* e.g.
                           "[2.00.01.023,01/01/99]"  */
  const char * BuildDate; /* e.g. "Jan 1 99" */
  const char * CallConv;
} IPLLibVersion;
```

Supported Image Attributes and Operation Modes

A

This appendix contains tables that list the supported image attributes and operation modes for functions that have input and/or output images. The `ipl` prefixes in the function names are omitted.

Table A-1 Image Attributes and Modes of Data Exchange Functions

Function	Depths	Input and output images must have the same				Rect. ROI	In-place	Tiling
		depth	order	origin	COI	supported (x)		
`Set`	u or s†	operates on a single image				x	x	x
`SetFP`	32f†	operates on a single image				x	x	x
`PutPixel`	all	operates on a single image					x	
`GetPixel`	all	operates on a single image					x	
`Copy`	all	x	x	x	x	x	x	x
`CloneImage`	all	x	x	x	x	x	x	x
`Exchange`	all	x	x	x	x	x	x	x
`Scale`	u or s		x	x	x	x		x
`ScaleFP`	32f‡		x	x	x	x		x
`NoiseImage`	all	x	x	x	x	x	x	x
`Convert`	u or s							x

† u or s = 1u, 8s, 8u, 16s, 16u, 32s bits per channel; u = unsigned; s = signed; f = float.
‡ only one of the images is 32f, the other must be 8s, 8u, 16s, 16u, 32s bits per channel

Table A-2 Windows DIB Conversion Functions

Function	Depths		Input and output images have the same		
	input	output	order	origin	number of channels
ConvertFromDIB	all‡	1u,8u,16u			
ConvertFromDIBSep	all‡	1u,8u,16u			
ConvertToDIB	1u,8u,16u	all‡			x
ConvertToDIBSep	1u,8u,16u	all‡			x
TranslateDIB	1bpp	1u	clone	x	x
	≥4bpp‡	8u	clone	x	x

‡ all = 1, 4, 8, 16, 24, 32 bpp DIB images;
≥4bpp stands for 4, 8, 16, 24, 32 bpp DIB images.

For `iplConvertFromDIB` and `iplConvertFromDIBSep`, the number of channels, bit depth per channel and the dimensions of the `IplImage` should be greater than or equal to those of the DIB image. When converting a DIB RGBA image, the `IplImage` should also contain an alpha channel.

Table A-3 Image Attributes and Modes of Arithmetic and Logical Functions

Function	Depths	Input and output images must have the same: depth	order	origin	COI	Rect. ROI (supported (x))	In-place	Tiling	Mask
Abs	u or s†	x	x	x	x	x	x	x	x
AddS	u or s	x	x	x	x	x	x	x	x
SubtractS	u or s	x	x	x	x	x	x	x	x
MultiplyS	u or s	x	x	x	x	x	x	x	x
AddSFP	32f	x	x	x	x	x	x	x	x
SubtractSFP	32f	x	x	x	x	x	x	x	x
MultiplySFP	32f	x	x	x	x	x	x	x	x
MultiplySScale	8u,16u	x	x	x	x	x	x	x	x
Square	all†	x	x	x	x	x	x	x	x
Add	all	x	x	x	x	x	x	x	x
Subtract	all	x	x	x	x	x	x	x	x
Multiply	all	x	x	x	x	x	x	x	x
MultiplyScale	8u,16u	x	x	x	x	x	x	x	x
LShiftS	u or s	x	x	x	x	x	x	x	x
RShiftS	u or s	x	x	x	x	x	x	x	x
Not	u or s	x	x	x	x	x	x	x	x
AndS	u or s	x	x	x	x	x	x	x	x
OrS	u or s	x	x	x	x	x	x	x	x
XorS	u or s	x	x	x	x	x	x	x	x
And	u or s	x	x	x	x	x	x	x	x
Or	u or s	x	x	x	x	x	x	x	x
Xor	u or s	x	x	x	x	x	x	x	x

† u or s = 1u, 8s, 8u, 16s, 16u, 32s bits per channel (that is, all except 32f)
all = 1u, 8s, 8u, 16s, 16u, 32s, or 32f bits per channel

Table A-4 Image Attributes and Modes of Alpha-Blending Functions

Function	Depths	Input and output images must have the same: depth	order	origin	COI	Rect. ROI	In-place	Tiling	Mask
						supported (x)			
PreMultiplyAlpha	8u,16u	x	x	x	x	x	x	x	x
AlphaComposite	8u,16u	x	x	x	x	x	x	x	x
AlphaCompositeC	8u,16u	x	x	x	x	x	x	x	x

Table A-5 Image Attributes and Modes of Filtering Functions

Function	Depths	Input and output images must have the same: depth	order	origin	COI	Rect. ROI	Border Mode	In-place	Tiling	Mask
						supported (x)				
Blur	u or s	x	x	x	x	x	x	x	x	x
Convolve2D	u or s	x	x	x	x	x	x	x	x	x
Convolve2DFP	32f	x	x	x	x	x	x		x	x
ConvolveSep2D	u or s	x	x	x	x	x	x		x	x
ConvolveSep2DFP	32f	x	x	x	x	x	x		x	x
MaxFilter	u or s	x	x	x	x	x	x		x	x
MinFilter	u or s	x	x	x	x	x	x		x	x
MedianFilter	u or s	x	x	x	x	x	x		x	x
ColorMedianFilter	8u/s, 16u/s,32f	x	x	x	x	x	x		x	x
FixedFilter	all	x	x	x	x	x	x		x	x

Table A-6 Image Attributes and Modes of Fourier and DCT Functions

Function	Depths: input	Depths: output	Input & output images have the same: order	origin	COI	Rect. ROI	In-place	Tiling	Mask
						supported (x)			
DCT2D	≥8u/s‡, 32f	≥8u/s, 32f	x	x		x			
RealFft2D	≥8u/s, 32f	≥8u/s, 32f	x	x	x	x			
CcsFft2D	≥8u/s, 32f	≥8u/s, 32f	x	x	x	x			
MpyRCPack2D	≥8s, 32f	≥8s, 32f			x	x			

‡ ≥8u/s stands for 8u, 8s, 16u, 16s, 32s; ≥8s stands for 8s, 16s, 32s bits per channel

Table A-7 Image Attributes and Modes of Morphological Operations

Function	Depths	Input and output images must have the same				Rect. ROI	Border Mode	In-place	Tiling
		depth	order	origin	COI	supported (x)			
Erode	1u,8u,16u	x	x	x	x	x	x	x	x
Dilate	1u,8u,16u	x	x	x	x	x	x	x	x
Open	1u,8u,16u	x	x	x	x	x	x	x	x
Close	1u,8u,16u	x	x	x	x	x	x	x	x

Table A-8 Image Attributes and Modes of Color Space Conversion Functions

Function	Depths		Input & output images have the same				Rect. ROI	In-place	Tiling
	input	output	depth	order	origin	COI	supported (x)		
ReduceBits	32s	1u, 8u,16u, 32s		x	x	x			x
	16u	1u, 8u,16u		x	x	x			x
	8u	1u, 8u		x	x	x			x
GrayToColor	32s, gray†	color†		x	x	x			x
ColorToGray	color†	gray†		x	x	x			x
BitonalToGray	1u	≥8u/s‡							x
RGB to/from other color model	8u,16u,32s; for LUV, also 32f		x	x	x	x			x
ApplyColorTwist	8u,16u		x	x	x	x	x	x	x
ColorTwistFP	32f		x	x	x	x	x	x	x

† gray = 1u, 8u, 16u bits per pixel
color = 8u, 16u, 32s bits per channel
‡ ≥8u/s = 8u, 8s, 16u, 16s, 32s bits per channel

Table A-9 Image Attributes and Modes of Histogram and Thresholding Functions

Function	Depths	Input and output images must have the same				Rect. ROI	In-place	Tiling
		depth	order	origin	COI	supported (x)		
Threshold	8u,8s,16u, 16s, 32s†		x	x	x	x	x	x
ComputeHisto	1u,8u,16u	no output image				x		x
HistoEqualize	8u,16u	x	x	x	x	x	x	x
ContrastStretch	8u,16u	x	x	x	x	x	x	x
Compare functions	all‡	x	x	x	x	x		x

†output image can also be 1u bit per channel

‡ Functions with FP postfix compare 32f data; in-place mode for 1u images is not supported.

Table A-10 Image Attributes and Modes of Geometric Transform Functions

Function	Depths	Input and output images must have the same				Rect. ROI	In-place	Tiling	Mask
		depth	order	origin	COI	supported (x)			
Mirror	1u,8u,16u,32f	x	x	x	x	x	x	x	x
Rotate	1u,8u,16u,32f	x	x	x	x	x		x	
Zoom	1u,8u,16u,32f	x	x	x	x	x		x	x
Decimate	1u,8u,16u,32f	x	x	x	x	x		x	
DecimateBlur	1u,8u,16u,32f	x	x	x	x	x		x	
Resize	1u,8u,16u,32f	x	x	x	x	x		x	
WarpAffine	1u,8u,16u,32f	x	x	x	x	x		x	
WarpBilinear	1u,8u,16u,32f	x	x	x	x	x		x	
WarpBilinearQ	1u,8u,16u,32f	x	x	x	x	x		x	
WarpPerspective	1u,8u,16u,32f	x	x	x	x	x		x	
Warp PerspectiveQ	1u,8u,16u,32f	x	x	x	x	x		x	
Shear	1u,8u,16u,32f	x	x	x	x	x		x	
Remap†	1u,8u,16u,32f	x	x	x	x	x		x	

† In `iplRemap`, the mapping coordinates are stored in one-channel 32-bit floating-point images.

Table A-11 Image Attributes and Modes of Image Statisctics Functions

Function	Depths	All images must have the same				Rect. ROI	Tiling	Mask
		depth	order	origin	COI	supported (x)		
Norm	all †	x	x	x	x	x	x	x
Moments	all	operates on a single image				x	x	x
[Normalized] SpatialMoment	all	operates on a single image				x	x	x
[Normalized] CentralMoment	all	operates on a single image				x	x	x
NormCrossCorr	≥8	x	x	x	x	x	x	x
MinMaxFP	32f	operates on a single image				x	x	

† **Bit depth shorthand:**

u or s = 1u, 8s, 8u, 16s, 16u, 32s bits per channel (that is, all except 32f)

all = 1u, 8s, 8u, 16s, 16u, 32s, or 32f bits per channel

≥8 stands for 8s, 8u, 16s, 16u, 32s, or 32f bits per channel

Table A-12 Image Attributes and Modes of Functions for User-Defined Image Processing

Function	Depths	All images must have the same				Rect. ROI	Tiling	Mask
		depth	order	origin	COI	supported (x)		
UserProcess	≥8u/s †	x	x	x	x	x	x	x
UserProcessFP	≥8	x	x	x	x	x	x	x
UserProcessPixel	≥8		x	x	n/s ‡	x	x	x

† **Bit depth shorthand:**

≥8u/s = 8u, 8s, 16u, 16s, 32s bits per channel

≥8 stands for 8u, 8s, 16u, 16s, 32s, or 32f bits per channel

‡ n/s - not supported

This page is left blank for double-sided printing

This page is left blank for double-sided printing

Interpolation in Geometric Transform Functions B

This appendix describes the interpolation algorithms used in the geometric transformation functions of the Image Processing Library. For more information about each of the geometric transform functions, see Chapter 11, *Geometric Transforms*.

Overview of Interpolation Modes

In geometric transformations, the grid of input image pixels is not necessarily mapped onto the grid of pixels in the output image. Therefore, to compute the pixel intensities in the output image, the geometric transform functions need to *interpolate* the intensity values of several input pixels that are mapped to a certain neighborhood of the output pixel.

Geometric transformations can use various interpolation algorithms. When calling the geometric transform functions of the Image Processing Library, the application code specifies the interpolation mode (that is, the type of interpolation algorithm) by using the parameter *`interpolate`*. The library supports the following interpolation modes:

- nearest neighbor interpolation (*`interpolate`* `= IPL_INTER_NN`)
- linear interpolation (*`interpolate`* `= IPL_INTER_LINEAR`)
- cubic interpolation (*`interpolate`* `= IPL_INTER_CUBIC`)
- super-sampling (*`interpolate`* `= IPL_INTER_SUPER`)

Table B-1 lists the supported interpolation modes for all geometric transform functions. For certain functions, you can combine the above interpolation algorithms with additional smoothing (antialiasing) of edges to which the original image's borders are transformed. To use this edge smoothing, set the parameter `interpolate` to the bitwise OR of `IPL_SMOOTH_EDGE` and the desired interpolation mode. For example, in order to rotate an image with cubic interpolation and smooth the rotated image's edges, you pass to `iplRotate()` the following value: `interpolate = IPL_INTER_CUBIC | IPL_SMOOTH_EDGE`.

Table B-1 Interpolation Modes Supported by Geometric Transform Functions

Function	Nearest neighbor	Linear	Cubic	Super-sampling	Edge smoothing
Mirror	This function does not need interpolation				
Rotate	x	x	x		x
Zoom	x	x	x		
Decimate	x	x	x	x	
DecimateBlur	x	x	x		
Resize	x	x	x	x	
WarpAffine	x	x	x		x
WarpBilinear	x	x	x		x
WarpBilinearQ	x	x	x		x
Warp Perspective	x	x	x		x
Warp PerspectiveQ	x	x	x		x
Shear	x	x	x		x

The sections that follow provide more details on each interpolation mode.

Mathematical Notation

In this appendix we'll use the following notation:

(x_D, y_D)	pixel coordinates in the destination image (integer values)
(x_S, y_S)	the computed coordinates of a point in the source image that is mapped exactly to (x_D, y_D)
$S(x, y)$	pixel value (intensity) in the source image
$D(x, y)$	pixel value (intensity) in the destination image.

Nearest Neighbor Interpolation

This is the fastest and least accurate interpolation mode. The pixel value in the destination image is set to the value of the source image's pixel closest to the point (x_S, y_S): $D(x_D, y_D) = S(\text{round}(x_S), \text{round}(y_S))$.

To use the nearest neighbor interpolation, set the parameter `interpolate` to `IPL_INTER_NN`.

Linear Interpolation

The linear interpolation is slower but more accurate than the nearest neighbor interpolation. On the other hand, it is faster but less accurate than cubic interpolation. The linear interpolation algorithm uses source image intensities at the four pixels (x_{S0}, y_{S0}), (x_{S1}, y_{S0}), (x_{S0}, y_{S1}), (x_{S1}, y_{S1}) which are closest to (x_S, y_S) in the source image:

$$x_{S0} = \text{int}(x_S),\ \ x_{S1} = x_{S0} + 1,\ \ y_{S0} = \text{int}(y_S),\ \ y_{S1} = y_{S0} + 1.$$

First, the intensity values are interpolated along the x-axis to produce two intermediate results I_0 and I_1 (see Figure B-1):

$$I_0 = S(x_S, y_{S0}) = S(x_{S0}, y_{S0})*(x_{S1} - x_S) + S(x_{S1}, y_{S0})*(x_S - x_{S0})$$

$$I_1 = S(x_S, y_{S1}) = S(x_{S0}, y_{S1})*(x_{S1} - x_S) + S(x_{S1}, y_{S1})*(x_S - x_{S0}).$$

Then, the sought-for intensity $D(x_D,y_D)$ is computed by interpolating the intermediate values I_0 and I_1 along the y-axis:

$$D(x_D,y_D) = I_0 * (y_{S1} - y_S) + I_1 * (y_S - y_{S0}).$$

To use the linear interpolation, set the parameter `interpolate` to `IPL_INTER_LINEAR`.

For images with 1-bit and 8-bit unsigned color channels, the functions `iplWarpAffine`, `iplRotate`, and `iplShear` compute the coordinates (x_S,y_S) with the accuracy $2^{-16} = 1/65536$. For images with 16-bit unsigned color channels, these functions compute the coordinates with floating-point precision.

Figure B-1 Linear Interpolation

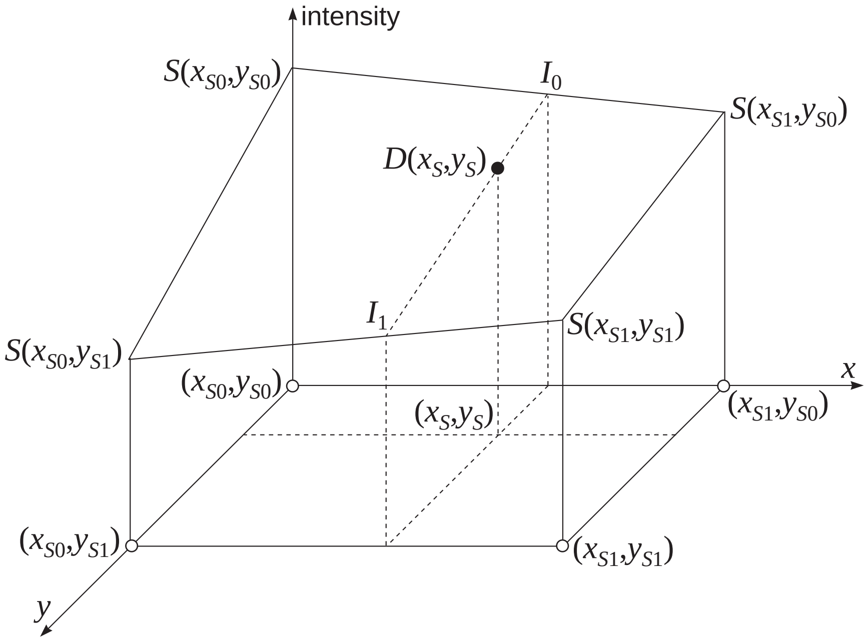

Cubic Interpolation

The cubic interpolation algorithm (see Figure B-2) uses source image intensities at sixteen pixels in the neighborhood of the point (x_S, y_S) in the source image:

$x_{S0} = \text{int}(x_S) - 1$ $\quad x_{S1} = x_{S0} + 1$ $\quad x_{S2} = x_{S0} + 2$ $\quad x_{S3} = x_{S0} + 3$

$y_{S0} = \text{int}(y_S) - 1$ $\quad y_{S1} = y_{S0} + 1$ $\quad y_{S2} = y_{S0} + 2$ $\quad y_{S3} = y_{S0} + 3.$

First, for each y_{Sk} the algorithm determines four cubic polynomials $F_0(x)$, $F_1(x)$, $F_2(x)$, and $F_3(x)$:

$$F_k(x) = a_k x^3 + b_k x^2 + c_k x + d_k \qquad 0 \le k \le 3,$$

such that

$$F_k(x_{S0}) = S(x_{S0}, y_{Sk}),\ F_k(x_{S1}) = S(x_{S1}, y_{Sk}),\ F_k(x_{S2}) = S(x_{S2}, y_{Sk}),\ F_k(x_{S3}) = S(x_{S3}, y_{Sk}).$$

In Figure B-2, these polynomials are shown by solid curves.

Then, the algorithm determines a cubic polynomial $F_y(y)$ such that

$$F_y(y_{S0}) = F_0(x_S),\ F_y(y_{S1}) = F_1(x_S),\ F_y(y_{S2}) = F_2(x_S),\ F_y(y_{S3}) = F_3(x_S).$$

The polynomial $F_y(y)$ is represented by the dashed curve in Figure B-2.

Finally, the sought-for intensity $D(x_D, y_D)$ is set to the value $F_y(y_S)$.

To use the cubic interpolation, set the parameter *`interpolate`* to `IPL_INTER_CUBIC`.

For images with 1-bit and 8-bit unsigned color channels, the functions `iplWarpAffine`, `iplRotate`, and `iplShear` compute the coordinates (x_S, y_S) with the accuracy $2^{-16} = 1/65536$. For images with 16-bit unsigned color channels, these functions compute the coordinates with floating-point precision.

Figure B-2 Cubic Interpolation

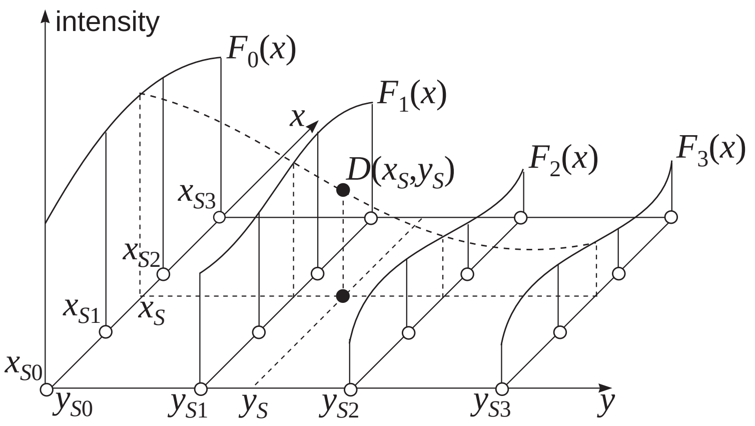

Super-Sampling

If the destination image is much smaller than the source image, the above interpolation algorithms may skip some pixels in the source image (that is, these algorithms not necessarily use all source pixels when computing the destination pixels' intensity). In order to use all pixel values of the source image, the `iplDecimate` and `iplResize` functions support the *super-sampling* algorithm, which is free of the above drawback.

The super-sampling algorithm is as follows:

(1) Divide the source image's rectangular ROI (or the whole image, if there is no ROI) into equal rectangles, each rectangle corresponding to some pixel in the destination image. Note that each source pixel is represented by a 1x1 square.

(2) Compute a weighted sum of source pixel values for all pixels that are contained in the rectangle or have a non-zero intersection with the rectangle. If a source pixel is fully contained in the rectangle, that pixel's value is taken with weight 1. If the rectangle and the source pixel's square have an intersection of area $a < 1$, that pixel's value is taken with weight a.

For each source pixel intersecting with the rectangle, Figure B-3 shows the corresponding weight value.

(3) To compute the pixel value in the destination image, divide this weighted sum by the rectangle area *`(xSrc*ySrc)/(xDst*yDst)`*.

Here *`xSrc`*, *`xDst`*, *`ySrc`*, and *`yDst`* are parameters passed to the functions `iplDecimate` and `iplResize` to set the decimation ratios *`xDst`*/*`xSrc`* and *`yDst`*/*`ySrc`*.

Figure B-3 Super-sampling Weights

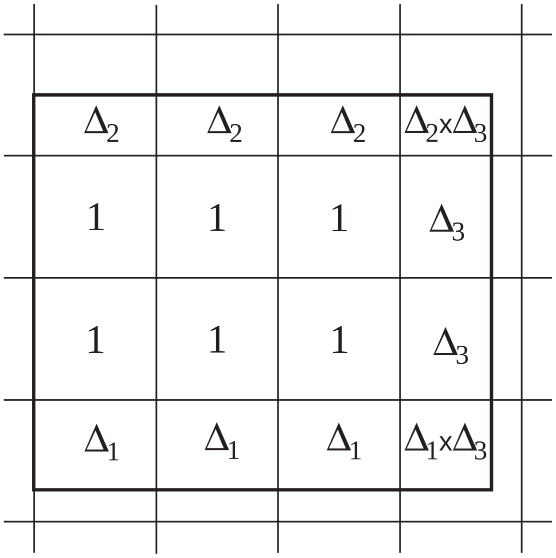

To use super-sampling, set the value `IPL_INTER_SUPER` for the parameter *`interpolate`*.

This page is left blank for double-sided printing

This page is left blank for double-sided printing

Bibliography

This bibliography provides a list of publications that might be useful to the Image Processing Library users. This list is not complete; it serves only as a starting point. The books [Rogers85], [Rogers90], and [Foley90] are good resources of information on image processing and computer graphics, with mathematical formulas and code examples.

The Image Processing Library is part of Intel® Performance Library Suite. The manuals [RPL] and [SPL] describe Intel Recognition Primitives Library and Intel Signal Processing Library, which are other parts of the Performance Library Suite.

[Bragg] Dennis Bragg. *A simple color reduction filter*, Graphic Gems III: 20–22.

[Foley90] James D. Foley, Andries van Dam, Steven K. Feiner, and John F. Hughes. *Computer Graphics — Principles and Practice,* Second Edition. Addison Wesley, 1990.

[J95] Jaehne, Bernd. *Digital Image Processing*, 3rd Edition, Springer-Verlag, Berlin 1995.

[J97] Jaehne, Bernd. *Practical Handbook on Image Processing for Scientific Applications*, CRC Press, New York, 1997.

[Rec709] ITU-R Recommendation BT.709, *Basic Parameter Values for the HDTV Standard for the Studio and International Programme Exchange* [formerly CCIR Rec.709] ITU, Geneva, Switzerland, 1990.

[Rogers85] David Rogers. *Procedural Elements for Computer Graphics,* McGraw-Hill, 1985.

[Rogers90] David Rogers and J.Alan Adams. *Mathematical Elements for Computer Graphics*, McGraw-Hill, 1990.

[RPL] *Intel® Recognition Primitives Library Reference Manual.* Intel Corp. Order number 637785.

[SPL] *Intel® Signal Processing Library Reference Manual.* Intel Corp. Order number 630508.

[Schumacher] Dale A. Schumacher. *A comparison of digital halftoning techniques,* Graphic Gems III: 57–71.

[Thomas] Spencer W. Thomas and Rod G. Bogart. *Color dithering*, Graphic Gems II: 72–77.

You may also find useful the following publications, which are not referenced in this manual but contain valuable information on particular functions:

Geometrical transforms

G.Wolberg. *Digital Image Warping*, IEEE Computer Society Press, 1996.

Wavelet transforms

A.Akansu, M.Smith (editors). *Subband and Wavelet transform. Design and Applications*, Kluwer Academic Publishers, 1996.

Median filter

H.Myler, A.Weeks. *Computer Imaging Recipes in C*, Prentice Hall, 1993.

Randy Crane. *A Simplified Approach to Image Processing,* Prentice Hall PTR, 1997

Moments functions

G.Ritter, J.Wilson. *Computer Vision. Algorithms in Image Algebra*. CRC Press, New York, 1996.

Glossary

absolute colors	Colors specified by each pixel's coordinates in a color space. Intel Image Processing Library functions use images with absolute colors. *See* palette colors.
alpha channel	A color channel, also known as the opacity channel, that can be used in color models; for example, the RGBA model.
arithmetic operation	An operation that adds, subtracts, multiplies, shifts, or squares the image pixel values.
channel of interest	The color channel on which an operation acts (or processing occurs). Channel of interest (COI) can be considered as a separate case of region of interest (ROI).
CMY	Cyan-magenta-yellow. A three-channel color model that uses cyan, magenta, and yellow color channels.
CMYK	Cyan-magenta-yellow-black. A four-channel color model that uses cyan, magenta, yellow, and black color channels.
COI	*See* channel of interest.
color-twist matrix	A matrix used to multiply the pixel coordinates in one color space for determining the coordinates in another color space.
conjugate	The conjugate of a complex number $a+bi$ is $a-bi$.
DCT	Acronym for the discrete cosine transform. *See* "Discrete Cosine Transform" in Chapter 7.
decimation	A geometric transform operation that shrinks the source image.

DIB	Device-independent bitmap, an image format used by the library in Windows environment.
dilation	A morphological operation that sets each output pixel to the minimum of the corresponding input pixel and its 8 neighbors.
dyadic operation	An operation that has two input images. It can have other input parameters as well.
erosion	A morphological operation that sets each output pixel to the maximum of the corresponding input pixel and its 8 neighbors.
FFT	Acronym for the fast Fourier transform. *See* "Fast Fourier Transform" in Chapter 7.
four-channel model	A color model that uses four color channels; for example, the RGBA color model.
geometric transform functions	Functions that perform geometric transformations of images: resizing, rotation, mirror, shear, and warping functions.
gray scale image	An image characterized by a single intensity channel so that each intensity value corresponds to a certain shade of gray.
HLS	Hue-lightness-saturation. A three-channel color model that uses hue, lightness, and saturation channels. The HLS and HSV models differ in the way of scaling the image luminance. *See* HSV.
HSV	Hue-saturation-value. A three-channel color model that uses hue, saturation, and value channels. HSV is often used as a synonym for the HSB (hue-saturation-brightness) and HSI (hue-saturation-intensity) models. *See* HLS.
hue	A color channel in several color models that measures the "angular" distance (in degrees) from red to the particular color: 60 corresponds to yellow, 120 to green, 180 to cyan, 240 to blue, and 300 to magenta. Hue is undefined for shades of gray.

in-place operation	An operation whose output image is one of the input images. *See* out-of-place operation.
linear filtering	In this library, either neighborhood averaging (blur) or 2D convolution operations.
linear image transforms	In this library, the fast Fourier transform (FFT) or the discrete cosine transform (DCT).
luminance	A measure of image intensity, as perceived by a "standard observer". Since human eyes are more sensitive to green and less to red or blue, different colors of equal physical intensity make different contribution to luminance. *See* `ColorToGray` in Chapter 9.
LUT	Acronym for lookup table (palette).
LUV	A three-channel color model designed to acieve perceptual uniformity, that is, to make the perceived distance between two colors proportional to the numerical distance.
MMX™ technology	A major enhancement to the Intel Architecture aimed at better performance in multimedia and communications applications. The technology uses four new data types, eight 64-bit MMX registers, and 57 new instructions implementing the SIMD (single instruction, multiple data) technique.
monadic operation	An operation that has a single input image. It can have other input parameters as well.
morphological operation	An erosion, dilation, or their combinations.
MSI	Acronym for multi-spectral image. An MSI can use any number of channels and colors.
non-linear filtering	In the Image Processing Library, minimum, maximum, or median filtering operation.
opacity channel	*See* alpha channel.
out-of-place operation	An operation whose output is an image other than the input image(s). *See* in-place operation.

palette colors	Colors specified by a palette, or lookup table. The Image Processing Library uses palette colors only in operations of image conversion to and from absolute colors. *See* absolute colors.
PhotoYCC*	A Kodak* proprietary color encoding and image compression scheme. *See* YCC.
pixel depth	The number of bits determining a single pixel in the image.
pixel-oriented ordering	Storing the image information in such an order that the values of all color channels for each pixel are clustered; for example, RGBRGB... . *See* "Channel Sequence" in Chapter 2.
plane-oriented ordering	Storing the image information so that all data of one color channel follow all data of another channel, thus forming a separate "plane" for each channel; for example, RRRRRGGGGG...
region of interest	An image region on which an operation acts (or processing occurs).
RGB	Red-green-blue. A three-channel color model that uses red, green, and blue color channels.
RGBA	Red-green-blue-alpha. A four-channel color model that uses red, green, blue, and alpha (or opacity) channels.
ROI	*See* region of interest.
saturation	A quantity used for measuring the purity of colors. The maximum saturation corresponds to the highest degree of color purity; the minimum (zero) saturation corresponds to shades of gray.
scanline	All image data for one row of pixels.
standard gray palette	A complete palette of a DIB image whose red, green, and blue values are equal for each entry and monotonically increasing from entry to entry.
three-channel model	A color model that uses three color channels; for example, the CMY color model.

XYZ	A three-channel color model designed to represent a wider range of colors than the RGB model: some XYZ-representable colors would have a negative value of R. For conversion formulas, see RGB2XYZ.
YCC	A three-channel color model that uses one luminance channel (Y) and two chroma channels (usually denoted by C_R and C_B). The term is sometimes used as a synonym for the entire PhotoYCC encoding scheme. *See* PhotoYCC.
YUV	A three-channel color model frequently used in television. For conversion formulas, see RGB2YUV.
zoom	A geometric transform function that magnifies the source image.

This page is left blank for double-sided printing

This page is left blank for double-sided printing

Index

A

a function that helps you

- add a constant to pixel values, 5-3
- add pixel values of two images, 5-7
- allocate a quadword-aligned memory block, 4-27
- allocate image data, 4-13
- allocate memory for 16-bit words, 4-28
- allocate memory for 32-bit double words, 4-28
- allocate memory for double floating-point elements, 4-30
- allocate memory for floating-point elements, 4-29
- apply a color-twist matrix, 9-21, 9-23
- assign a new error-handling function, 3-6
- average neighboring pixels, 6-2
- change the image orientation, 11-9
- change the image size, 11-3
- compare pixel values and a constant for equality, 10-21, 10-22, 10-23
- compare pixel values and a constant for *greater than*, 10-17, 10-18
- compare pixel values and a constant for *less than*, 10-19, 10-20
- compare pixels in two images for equality, 10-15
  - within tolerance ε, 10-16
- compare pixels in two images for *greater than*, 10-13
- compare pixels in two images for *less than*, 10-14
- compute absolute pixel values, 5-6
- compute bitwise AND of pixel values and a constant, 5-12
- compute bitwise AND of pixel values of two images, 5-15
- compute bitwise NOT of pixel values, 5-12
- compute bitwise OR of pixel values and a constant, 5-13
- compute bitwise OR of pixel values of two images, 5-15
- compute bitwise XOR of pixel values and a constant, 5-14
- compute bitwise XOR of pixel values of two images, 5-16
- compute CCS fast Fourier transform, 7-7
- compute discrete cosine transform, 7-9
- compute image moments, 12-5
- compute moments of order 0 to 3, 12-6
- compute real fast Fourier transform, 7-4
- compute the image histogram, 10-9
- compute the norm of pixel values, 12-2
- convert a bitonal image to gray scale, 9-7

convert a color image to gray scale, 9-8
convert a gray scale image to color, 9-9
convert images from DIB (changing attributes), 4-50, 4-53
convert images from DIB (preserving attributes), 4-47
convert images to DIB, 4-54, 4-55
convert RGB images to and from other color models, 9-10
convolve an image with 2D kernel, 6-8
convolve an image with a predefined kernel, 6-12
convolve an image with a separable kernel, 6-11
copy entire images, 4-15
copy image data, 4-32
create 2D convolution kernel, 6-5
create a color twist matrix, 9-19
create a region of interest (ROI), 4-21
create image header, 4-9
create the IplTileInfo structure, 4-25
decimate the image, 11-5, 11-6
delete 2D convolution kernel, 6-8
delete a color twist matrix, 9-22
delete a region of interest (ROI) structure, 4-21
delete the IplTileInfo structure, 4-26
dilate an image, 8-5
divide pixel values by 2^N, 5-11
equalize the image histogram, 10-10
erode an image, 8-2
exchange data of two images, 4-35
filter the image, 6-1
free memory allocated by Malloc functions, 4-30
free the image data memory, 4-15
free the image header memory, 4-16
get error-handling mode, 3-4
get the error status code, 3-3
get the value of pixel (x,y), 4-38
handle an error, 3-2, 3-7
initialize the image data, 4-31
magnify the image, 11-4
mirror the image, 11-14
multiply data in RCPack format, 7-8
multiply pixel values by a color-twist matrix, 9-21, 9-23
multiply pixel values by a constant, 5-4
multiply pixel values by a constant and scale the products, 5-5
multiply pixel values of two images, 5-8
multiply pixel values of two images and scale the products, 5-9
perform several erosions and dilations, 8-6, 8-7
pre-multiply pixel values by alpha values, 5-24
produce error messages for users, 3-5
read convolution kernel's attributes, 6-6
reduce the image bit resolution, 9-3
re-map images by using coordinate tables, 11-28
report an error, 3-2, 3-7
resize the image, 11-7
rotate the image, 11-9
scale the image data, 4-40, 4-41
set a color twist matrix, 9-20

set a region of interest (ROI), 4-22
set error-handling mode, 3-4
set one pixel to a new value, 4-38
set pixels to the maximum value of the neighbors, 6-17
set pixels to the median value of the neighbors, 6-15
set pixels to the minimum value of the neighbors, 6-18
set the error status code, 3-3
set the image border mode, 4-23
set the IplTileInfo structure fields, 4-26
shear images, 11-16
shift pixel bits to the left, 5-10
shift pixel bits to the right, 5-11
shrink the image, 11-5, 11-6
smooth the image, 8-6, 8-7
square pixel values, 5-6
stretch the image contrast, 10-7
subtract pixel values from a constant, 5-4
subtract pixel values of two images, 5-8
threshold the source image, 10-3
warp images by affine transforms, 11-17
warp images by bilinear transforms, 11-20
warp images by perspective transforms, 11-24
warp images by using coordinate tables, 11-28
zoom the image, 11-4
about this manual, 1-2
about this software, 1-1
Abs function, 5-6
absolute color images, 2-2
absolute pixel values, 5-6
Add function, 5-7
adding a constant to pixel values, 5-3
adding pixels of two images, 5-7
AddS function, 5-3
AddSFP function, 5-3
alignment
image data, 2-7
rectangular ROIs, 2-5
scanline, 2-7
AllocateImage function, 4-13
AllocateImageFP function, 4-13
allocating memory
for 16-bit words, 4-28
for 32-bit double words, 4-28
for double floating-point elements, 4-30
for floating-point elements, 4-29
quadword-aligned blocks, 4-27
alpha channel, 2-7
alpha pre-multiplication, 5-24
alpha-blending
alpha pre-multiplication, 5-24
AlphaComposite function, 5-18
AlphaCompositeC function, 5-18
ATOP operation, 5-22
IN operation, 5-22
OUT operation, 5-22
OVER operation, 5-22
PLUS operation, 5-22
PreMultiplyAlpha function, 5-24
XOR operation, 5-22
AlphaComposite function, 5-18
AlphaCompositeC function, 5-18
And function, 5-15

AndS function, 5-12
ApplyColorTwist function, 9-21
argument order conventions, 1-7
arithmetic operations, 5-1
  Abs, 5-6
  Add, 5-7
  AddS, 5-3
  AddSFP, 5-3
  AlphaComposite, 5-18
  AlphaCompositeC, 5-18
  Multiply, 5-8
  MultiplyS, 5-4
  MultiplyScale, 5-9
  MultiplySFP, 5-4
  MultiplySScale, 5-5
  PreMultiplyAlpha, 5-24
  Square, 5-6
  Subtract, 5-8
  SubtractS, 5-4
  SubtractSFP, 5-4
ATOP compositing operation, 5-22
attributes of an image, 4-4
audience for this manual, 1-4
averaging the neighboring pixels, 6-2

B

bit depths supported, A-1
BitonalToGray function, 9-7
bitwise AND
  with a constant, 5-12
  with another image, 5-15
bitwise NOT, 5-12
bitwise OR
  with a constant, 5-13
  with another image, 5-15
bitwise XOR
  with a constant, 5-14
  with another image, 5-16
Blur function, 6-2
brightening the image, 5-3

C

call-backs, 2-9
CcsFft2D function, 7-7
CentralMoment function, 12-9
changing the image orientation, 11-9
changing the image size, 11-3
channel of interest, 2-4
channel sequence, 2-3
CheckImageHeader function, 4-17
CloneImage function, 4-15
Close function, 8-7
COI. *See* channel of interest
color data order, 2-3
color models, 2-1
  gray scale, 2-1
  multi-spectral image, 2-2
  three or four channels, 2-1
color space conversion functions
  ApplyColorTwist, 9-21
  BitonalToGray, 9-7
  ColorToGray, 9-8
  ColorTwistFP, 9-23
  CreateColorTwist, 9-19

DeleteColorTwist, 9-22
GrayToColor, 9-9
HLS2RGB, 9-13
HSV2RGB, 9-12
LUV2RGB, 9-14
ReduceBits, 9-3
RGB2HLS, 9-13
RGB2HSV, 9-12
RGB2LUV, 9-14
RGB2XYZ, 9-15
RGB2YCrCb, 9-16
RGB2YUV, 9-17
SetColorTwist, 9-20
XYZ2RGB, 9-15
YCC2RGB, 9-18
YCrCb2RGB, 9-16
YUV2RGB, 9-17
ColorToGray function, 9-8
color-twist matrices, 9-18
ColorTwistFP function, 9-23
compare operations, 10-12
Equal, 10-15
EqualFPEps, 10-16
EqualS, 10-21
EqualSFP, 10-22
EqualSFPEps, 10-23
Greater, 10-13
GreaterS, 10-17
GreaterSFP, 10-18
Less, 10-14
LessS, 10-19
LessSFP, 10-20
ComputeHisto function, 10-9
computing the norm of pixel values, 12-2
ContrastStretch function, 10-7
conventions
font, 1-5
names of constants and variables, 1-6
names of functions, 1-6
order of arguments, 1-7
Convert function, 4-36
ConvertFromDIB function, 4-50
ConvertFromDIBSep function, 4-53
converting bitonal images to gray scale, 9-7
converting color images to gray scale, 9-8
converting gray-scale images to color, 9-9
converting HLS images to RGB, 9-13
converting HSV images to RGB, 9-12
converting images from DIB (changing attributes), 4-50, 4-53
converting images from DIB (preserving attributes), 4-47
converting images to DIB, 4-54, 4-55
converting LUV images to RGB, 9-14
converting RGB images to HLS, 9-13
converting RGB images to HSV, 9-12
converting RGB images to LUV, 9-14
converting RGB images to XYZ, 9-15
converting RGB images to YCrCb, 9-16
converting RGB images to YUV, 9-17
converting XYZ images to RGB, 9-15
converting YCC images to RGB, 9-18
converting YCrCb images to RGB, 9-16
converting YUV images to RGB, 9-17
ConvertToDIB function, 4-54
ConvertToDIBSep function, 4-55

convolution, 6-3
Convolve2D function, 6-8
Convolve2DFP function, 6-8
ConvolveSep2D function, 6-11
ConvolveSep2DFP function, 6-11
coordinate systems, 2-4
Copy function, 4-32
copying entire images, 4-15
copying the image data, 4-32
CreateColorTwist function, 9-19
CreateConvKernel function, 6-5
CreateConvKernelChar function, 6-5
CreateConvKernelFP function, 6-5
CreateImageHeader function, 4-9
CreateImageJaehne function, 4-18
CreateROI function, 4-21
CreateTileInfo function, 4-25
creating images, 4-1, 4-9
cross-correlation, 12-12

D

darkening the image, 5-3
data architecture, 2-1
data exchange, 4-2
data exchange functions, 4-31
  Convert, 4-36
  Copy, 4-32
  Exchange, 4-35
  GetPixel, 4-38
  NoiseGaussianInit, 4-44
  NoiseGaussianInitFp, 4-44
  NoiseImage, 4-42
  NoiseUniformInit, 4-43
  NoiseUniformInitFp, 4-43
  PutPixel, 4-38
  Scale, 4-40
  ScaleFP, 4-41
  Set, 4-31
  SetFP, 4-31
data ordering, 2-3
data ranges in HLS and HSV models, 9-11
data types, 2-2
DCT. ***See*** discrete cosine transform
DCT2D function, 7-9
Deallocate function, 4-16
DeallocateImage function, 4-15
Decimate function, 11-5
DecimateBlur function, 11-6
DecimateFit macro, 11-8
decimating the image, 11-7
DeleteColorTwist function, 9-22
DeleteConvKernel function, 6-8
DeleteConvKernelFP function, 6-8
DeleteROI function, 4-21
DeleteTileInfo function, 4-26
device-independent bitmap, 4-3
DIB. *See* device-independent bitmap
DIB palette images, 2-2
Dilate function, 8-5
dilation of an image, 8-5
discrete cosine transform, 7-8
dividing pixel values by 2^N, 5-11
dMalloc function, 4-30
dyadic operations, 5-1

E

Equal function, 10-15
EqualFPEps function, 10-16
equalizing the image histogram, 10-10
EqualS function, 10-21
EqualSFP function, 10-22
EqualSFPEps function, 10-23
Erode function, 8-2
erosion of an image, 8-2
ErrModeLeaf error mode, 3-4
ErrModeParent error mode, 3-5
ErrModeSilent error mode, 3-5
error checks, 3-1
Error function, 3-2
error handling, 3-1
  example, 3-13
  status codes, 3-10
  user-defined error handler, 3-15
error handling macros, 3-9
error processing modes
  IPL_ErrModeLeaf, 3-4
  IPL_ErrModeParent, 3-5
  IPL_ErrModeSilent, 3-5
error-handling functions, 3-2
  Error, 3-2
  ErrorStr, 3-5
  GetErrMode, 3-4
  GetErrStatus, 3-3
  GuiBoxReport, 3-7
  NullDevReport, 3-7
  RedirectError, 3-6
  SetErrMode, 3-4
  SetErrStatus, 3-3
  StdErrReport, 3-7
ErrorStr function, 3-5
Exchange function, 4-35
execution architecture, 2-8
  in-place and out-of-place operations, 2-8
  overflow and underflow, 2-8
  saturation, 2-8

F

fast Fourier and discrete cosine transforms
  CcsFft2D, 7-7
  DCT2D, 7-9
  MpyRCPack2D, 7-8
  RealFft2D, 7-4
fast Fourier transform, 7-1
FFT. *See* fast Fourier transform
filling image’s pixels with a value, 4-38
filtering functions, 6-1
  Blur, 6-2
  Convolve2D, 6-8
  Convolve2DFP, 6-8
  ConvolveSep2D, 6-11
  ConvolveSep2DFP, 6-11
  CreateConvKernel, 6-5
  CreateConvKernelChar, 6-5
  CreateConvKernelFP, 6-5
  DeleteConvKernel, 6-8
  DeleteConvKernelFP, 6-8
  FixedFilter, 6-12
  GetConvKernel, 6-6
  GetConvKernelChar, 6-6

GetConvKernelFP, 6-6
MaxFilter, 6-17
MedianFilter, 6-15
MinFilter, 6-18
FixedFilter function, 6-12
font conventions, 1-5
Free function, 4-30
free memory allocated by Malloc functions, 4-30
function descriptions, 1-4
function name conventions, 1-6

G

geometric transform functions
Decimate, 11-5
DecimateBlur, 11-6
GetAffineBound, 11-18
GetAffineQuad, 11-18
GetAffineTransform, 11-19
GetBilinearBound, 11-22
GetBilinearQuad, 11-22
GetBilinearTransform, 11-23
GetPerspectiveBound, 11-26
GetPerspectiveQuad, 11-26
GetPerspectiveTransform, 11-27
GetRotateShift, 11-11
Mirror, 11-14
Remap, 11-28
Resize, 11-7
Rotate, 11-9
Shear, 11-16
WarpAffine, 11-17
WarpBilinear, 11-20
WarpBilinearQ, 11-20
WarpPerspective, 11-24
WarpPerspectiveQ, 11-24
Zoom, 11-4
geometric transform macros
DecimateFit, 11-8
ResizeFit, 11-8
RotateCenter, 11-13
ZoomFit, 11-8
GetAffineBound function, 11-18
GetAffineQuad function, 11-18
GetAffineTransform function, 11-19
GetBilinearBound function, 11-22
GetBilinearQuad function, 11-22
GetBilinearTransform function, 11-23
GetCentralMoment function, 12-7
GetConvKernel function, 6-6
GetConvKernelChar function, 6-6
GetConvKernelFP function, 6-6
GetErrMode function, 3-4
GetErrStatus function, 3-3
GetLibVersion function, 14-1
GetNormalizedCentralMoment function, 12-8
GetNormalizedSpatialMoment function, 12-7
GetPerspectiveBound function, 11-26
GetPerspectiveQuad function, 11-26
GetPerspectiveTransform function, 11-27
GetPixel function, 4-38
GetRotateShift function, 11-11
GetSpatialMoment function, 12-6
gray-scale images, 2-1
GrayToColor function, 9-9

Greater function, 10-13
GreaterS function, 10-17
GreaterSFP function, 10-18
GuiBoxReport function, 3-7

H

handling overflow and underflow, 2-8
hardware and software requirements, 1-1
HistoEqualize function, 10-10
histogram and thresholding functions, 10-1
    ComputeHisto, 10-9
    ContrastStretch, 10-7
    HistoEqualize, 10-10
    Threshold, 10-2
histogram of an image, 10-9
histogram operations, 10-5
HLS2RGB function, 9-13
HSV2RGB function, 9-12

I

image attributes, 4-4, A-1
image compositing
    alpha pre-multiplication, 5-24
    AlphaComposite function, 5-18
    AlphaCompositeC function, 5-18
    ATOP operation, 5-22
    IN operation, 5-22
    OUT operation, 5-22
    OVER operation, 5-17, 5-22
    PLUS operation, 5-22
    PreMultiplyAlpha function, 5-24
    XOR operation, 5-22
image creation functions, 4-1
    AllocateImage, 4-13
    AllocateImageFP, 4-13
    CheckImageHeader, 4-17
    CloneImage, 4-15
    CreateImageHeader, 4-9
    CreateImageJaehne, 4-18
    CreateROI, 4-21
    CreateTileInfo, 4-25
    Deallocate, 4-16
    DeallocateImage, 4-15
    DeleteROI, 4-21
    DeleteTileInfo, 4-26
    SetBorderMode, 4-23
    SetROI, 4-22
    SetTileInfo, 4-26
image dimensions, 2-7
image filtering functions, 6-1
image format, 4-4
image header, 4-4
image histogram, 10-9
image moments, 12-5
image norms, 12-2
Image Processing Library functionality
    2D convolution, 6-3
    alpha-blending, 5-1
    arithmetic operations, 5-1
    color space conversion, 9-1
    compare functions, 10-1
    data exchange, 4-1
    DIB environment functions, 4-45
    discrete cosine transform, 7-8

error handling, 3-1
fast Fourier transform, 7-1
filtering functions, 6-1
geometric transform functions, 11-1
histogram and thresholding functions, 10-1
image creation, 4-1
image statistics, 12-1
image tiling, 2-8, 4-8
interpolation algorithms, B-1
logical operations, 5-1
memory allocation, 4-27
moments and norms, 12-1
morphological operations, 8-1
supported image attributes and modes, A-1
user-defined functions, 13-1
version of the library, 14-1
image row data, 2-7
image size, 2-7
image structure
borders, 4-23
channel sequence, 2-3
color models, 2-1
coordinate systems, 2-4
data architecture, 2-1
data ordering, 2-3
data types, 2-2
header attributes, 4-4
image size, 2-7
regions of interest, 2-4
tile size, 2-9
tiling, 2-8, 4-8
image tiling, 2-8, 4-8
call-backs, 2-9
IplTileInfo structure, 4-8
iMalloc function, 4-28
IN compositing operation, 5-22
in-place operations, 2-8
interpolation algorithms, B-1
IPL_ErrModeLeaf, 3-4
IPL_ErrModeParent, 3-5
IPL_ErrModeSilent, 3-5
iplAbs, 5-6
iplAdd, 5-7
iplAddS, 5-3
iplAddSFP, 5-3
iplAllocateImage, 4-13
iplAllocateImageFP, 4-13
iplAlphaComposite, 5-18
iplAlphaCompositeC, 5-18
iplAnd, 5-15
iplAndS, 5-12
iplApplyColorTwist, 9-21
iplBitonalToGray, 9-7
iplBlur, 6-2
iplCcsFft2D, 7-7
iplCentralMoment, 12-9
iplCheckImageHeader, 4-17
iplCloneImage, 4-15
iplClose, 8-7
iplColorToGray, 9-8
iplColorTwistFP, 9-23
iplComputeHisto, 10-9
iplContrastStretch, 10-7
iplConvert, 4-36
iplConvertFromDIB, 4-50
iplConvertFromDIBSep, 4-53

iplConvertToDIB, 4-54
iplConvertToDIBSep, 4-55
iplConvolve2D, 6-8
iplConvolve2DFP, 6-8
iplConvolveSep2D, 6-11
iplConvolveSep2DFP, 6-11
iplCopy, 4-32
iplCreateColorTwist, 9-19
iplCreateConvKernel, 6-5
iplCreateConvKernelChar, 6-5
iplCreateConvKernelFP, 6-5
iplCreateImageHeader, 4-9
iplCreateImageJaehne, 4-18
iplCreateROI, 4-21
iplCreateTileInfo, 4-25
iplDCT2D, 7-9
iplDeallocate, 4-16
iplDeallocateImage, 4-15
iplDecimate, 11-5
iplDecimateBlur, 11-6
iplDecimateFit, 11-8
iplDeleteColorTwist, 9-22
iplDeleteConvKernel, 6-8
iplDeleteConvKernelFP, 6-8
iplDeleteROI, 4-21
iplDeleteTileInfo, 4-26
iplDilate, 8-5
ipldMalloc, 4-30
iplEqual, 10-15
iplEqualFPEps, 10-16
iplEqualS, 10-21
iplEqualSFP, 10-22
iplEqualSFPEps, 10-23
iplErode, 8-2
iplError, 3-2
iplErrorStr, 3-5
iplExchange, 4-35
iplFixedFilter, 6-12
iplFree, 4-30
iplGetAffineBound, 11-18
iplGetAffineQuad, 11-18
iplGetAffineTransform, 11-19
iplGetBilinearBound, 11-22
iplGetBilinearQuad, 11-22
iplGetBilinearTransform, 11-23
iplGetCentralMoment, 12-7
iplGetConvKernel, 6-6
iplGetConvKernelChar, 6-6
iplGetConvKernelFP, 6-6
iplGetErrMode, 3-4
iplGetErrStatus, 3-3
iplGetLibVersion, 14-1
iplGetNormalizedCentralMoment, 12-8
iplGetNormalizedSpatialMoment, 12-7
iplGetPerspectiveBound, 11-26
iplGetPerspectiveQuad, 11-26
iplGetPerspectiveTransform, 11-27
iplGetPixel, 4-38
iplGetRotateShift, 11-11
iplGetSpatialMoment, 12-6
iplGrayToColor, 9-9
iplGreater, 10-13
iplGreaterS, 10-17
iplGreaterSFP, 10-18
iplGuiBoxReport, 3-7
iplHistoEqualize, 10-10

iplHLS2RGB, 9-13
iplHSV2RGB, 9-12
IplImage structure, 4-7
ipliMalloc, 4-28
IplLastStatus variable, 3-5
iplLess, 10-14
iplLessS, 10-19
iplLessSFP, 10-20
iplLShiftS, 5-10
iplLUV2RGB, 9-14
iplMalloc, 4-27
iplMaxFilter, 6-17
iplMedianFilter, 6-15
iplMinFilter, 6-18
iplMinMaxFP, 12-14
iplMirror, 11-14
iplMoments, 12-6
IplMomentState structure, 12-5
iplMpyRCPack2D, 7-8
iplMultiply, 5-8
iplMultiplyS, 5-4
iplMultiplyScale, 5-9
iplMultiplySFP, 5-4
iplMultiplySScale, 5-5
iplNoiseGaussianInit, 4-44
iplNoiseGaussianInitFp, 4-44
iplNoiseImage, 4-42
iplNoiseUniformInit, 4-43
iplNoiseUniformInitFp, 4-43
iplNorm, 12-2
iplNormalizedCentralMoment, 12-11
iplNormalizedSpatialMoment, 12-10
iplNormCrossCorr, 12-13
iplNot, 5-12
iplNullDevReport, 3-7
iplOpen, 8-6
iplOr, 5-15
iplOrS, 5-13
iplPreMultiplyAlpha, 5-24
iplPutPixel, 4-38
iplRealFft2D, 7-4
iplRedirectError, 3-6
iplReduceBits, 9-3
iplRemap, 11-28
iplResize, 11-7
iplResizeFit, 11-8
iplRGB2HLS, 9-13
iplRGB2HSV, 9-12
iplRGB2LUV, 9-14
iplRGB2XYZ, 9-15
iplRGB2YCrCb, 9-16
iplRGB2YUV, 9-17
iplRotate, 11-9
iplRotateCenter, 11-13
iplRShiftS, 5-11
iplScale, 4-40
iplScaleFP, 4-41
iplSet, 4-31
iplSetBorderMode, 4-23
iplSetColorTwist, 9-20
iplSetErrMode, 3-4
iplSetErrStatus, 3-3
iplSetFP, 4-31
iplSetROI, 4-22
iplSetTileInfo, 4-26
iplShear, 11-16

iplsMalloc, 4-29
iplSpatialMoment, 12-9
iplSquare, 5-6
iplStdErrReport, 3-7
iplSubtract, 5-8
iplSubtractS, 5-4
iplSubtractSFP, 5-4
iplThreshold, 10-2
IplTileInfo structure, 4-8
iplTranslateDIB, 4-47
iplUserFunc, 13-2
iplUserFuncFP, 13-3
iplUserFuncPixel, 13-4
iplUserProcess, 13-5
iplUserProcessFP, 13-7
iplUserProcessPixel, 13-8
iplWarpAffine, 11-17
iplWarpBilinear, 11-20
iplWarpBilinearQ, 11-20
iplWarpPerspective, 11-24
iplWarpPerspectiveQ, 11-24
iplwMalloc, 4-28
iplXor, 5-16
iplXorS, 5-14
iplXYZ2RGB, 9-15
iplYCC2RGB, 9-18
iplYCrCb2RGB, 9-16
iplYUV2RGB, 9-17
iplZoom, 11-4
iplZoomFit, 11-8

L

Less function, 10-14
LessS function, 10-19
LessSFP function, 10-20
linear filters, 6-2
logical operations, 5-1
    And, 5-15
    AndS, 5-12
    LShiftS, 5-10
    Not, 5-12
    Or, 5-15
    OrS, 5-13
    RShiftS, 5-11
    Xor, 5-16
    XorS, 5-14
lookup table. *See* palette color images
lookup table operations, 10-5
LShiftS function, 5-10
LUV2RGB function, 9-14

M

magnifying the image, 11-4, 11-7
Malloc function, 4-27
manual organization, 1-2
mask, 2-4
MaxFilter function, 6-17
maximum permissible value, 2-8
maximum pixel value, 12-14
MedianFilter function, 6-15
memory allocation functions, 4-2, 4-27
    dMalloc, 4-30
    Free, 4-30

iMalloc, 4-28
Malloc, 4-27
sMalloc, 4-29
wMalloc, 4-28
MinFilter function, 6-18
minimum permissible value, 2-8
minimum pixel value, 12-14
MinMaxFP function, 12-14
Mirror function, 11-14
mirroring the image, 11-14
moments, 12-5
moments and norms
CentralMoment, 12-9
GetCentralMoment, 12-7
GetNormalizedCentralMoment, 12-8
GetNormalizedSpatialMoment, 12-7
GetSpatialMoment, 12-6
MinMaxFP, 12-14
Moments, 12-6
Norm, 12-2
NormalizedCentralMoment, 12-11
NormalizedSpatialMoment, 12-10
SpatialMoment, 12-9
Moments function, 12-6
monadic operations, 5-1
morphological operations
Close, 8-7
Dilate, 8-5
Erode, 8-2
Open, 8-6
MpyRCPack2D function, 7-8
MSI. *See* multi-spectral image
multi-image operations, 2-5
Multiply function, 5-8
multiplying and scaling pixel values
by a constant, 5-5
in two input images, 5-9
multiplying pixel values
by a color-twist matrix, 9-21, 9-23
by a constant, 5-4
by a negative power of 2, 5-11
in two input images, 5-8
squares of pixel values, 5-6
MultiplyS function, 5-4
MultiplyScale function, 5-9
MultiplySFP function, 5-4
MultiplySScale function, 5-5
multi-spectral image, 2-2

N

naming conventions, 1-6
NoiseGaussianInit function, 4-44
NoiseGaussianInitFp function, 4-44
NoiseImage function, 4-42
NoiseUniformInit function, 4-43
NoiseUniformInitFp function, 4-43
Norm function, 12-2
normalized cross-correlation, 12-12
NormalizedCentralMoment function, 12-11
NormalizedSpatialMoment function, 12-10
NormCrossCorr function, 12-13
Not function, 5-12
notational conventions, 1-5
NullDevReport function, 3-7
numerical exceptions, 3-1

O

online version of this manual, 1-5
opacity channel. *See* alpha channel
Open function, 8-6
opening and smoothing the image, 8-6
operation modes of library functions, A-1
Or function, 5-15
OrS function, 5-13
OUT compositing operation, 5-22
out-of-place operations, 2-8
OVER compositing operation, 5-17, 5-22

P

palette color images, 2-2
parallelism, 1-1
pixel depth, 2-2
pixel values, setting and retrieving, 4-38
PLUS compositing operation, 5-22
PreMultiplyAlpha function, 5-24
producing error messages for users, 3-6
PutPixel function, 4-38

R

RCPack2D format, 7-1
real-complex packed format, 7-1
RealFft2D function, 7-4
rectangular region of interest, 2-4
RedirectError function, 3-6
ReduceBits function, 9-3
reducing the image bit resolution, 9-3
region of interest, 2-4, 4-20
    channel, 2-4
    mask image, 2-4
    rectangular, 2-4
Remap function, 11-28
reporting an error, 3-2, 3-8
Resize function, 11-7
ResizeFit macro, 11-8
return values, 1-4
RGB2HLS function, 9-13
RGB2HSV function, 9-12
RGB2LUV function, 9-14
RGB2XYZ function, 9-15
RGB2YCrCb function, 9-16
RGB2YUV function, 9-17
ROI. *See* region of interest
Rotate function, 11-9
RotateCenter macro, 11-13
rotating the image
    around an arbitrary center, 11-11
    around the origin, 11-9
RShiftS function, 5-11

S

saturation, 2-8
Scale function, 4-40
ScaleFP function, 4-41
scanline. *See* image row data
scanline alignment, 2-7
Set function, 4-31
SetBorderMode function, 4-23
SetColorTwist function, 9-20
SetErrMode function, 3-4

SetErrStatus function, 3-3
SetFP function, 4-31
SetROI function, 4-22
SetTileInfo function, 4-26
Shear function, 11-16
shearing the image, 11-16
shifting pixel bits
  to the left, 5-10
  to the right, 5-11
shrinking the image, 11-5, 11-6, 11-7
signed data, 2-2
SIMD instructions, 1-1
sMalloc function, 4-29
smoothing the image, 8-7
SpatialMoment function, 12-9
Square function, 5-6
squares of pixel values, 5-6
status codes, 3-10
StdErrReport function, 3-7
stretching the image contrast, 10-7
Subtract function, 5-8
subtracting pixel values
  from a constant, 5-4
  two input images, 5-8
SubtractS function, 5-4
SubtractSFP function, 5-4
supported image attributes and modes, A-1

T

Threshold function, 10-2
thresholding the source image, 10-3
tiling, 2-8, 4-8
  call-backs, 2-9
  CreateTileInfo function, 4-25
  DeleteTileInfo function, 4-26
  IplTileInfo structure, 4-8
  SetTileInfo function, 4-26
TranslateDIB function, 4-47
two-dimensional convolution, 6-3

U

user-defined coordinate transformations, 11-28
user-defined error handler, 3-15
user-defined functions
  UserFunc type, 13-2
  UserFuncFP type, 13-3
  UserFuncPixel type, 13-4
  UserProcess, 13-5
  UserProcessFP, 13-7
  UserProcessPixel, 13-8

V

version of the library, 14-1

W

WarpAffine function, 11-17
WarpBilinear function, 11-20
WarpBilinearQ function, 11-20
warping the image, 11-15, 11-28
WarpPerspective function, 11-24
WarpPerspectiveQ function, 11-24
Windows DIB functions, 4-3, 4-45
  ConvertFromDIB, 4-50

ConvertFromDIBSep, 4-53
ConvertToDIB, 4-54
ConvertToDIBSep, 4-55
TranslateDIB, 4-47
wMalloc function, 4-28

X

XOR compositing operation, 5-22
Xor function, 5-16
XorS function, 5-14
XYZ2RGB function, 9-15

Y

YCC2RGB function, 9-18
YCrCb2RGB function, 9-16
YUV2RGB function, 9-17

Z

Zoom function, 11-4
ZoomFit macro, 11-8
zooming the image, 11-4, 11-7
