# Supplementary material for: Rapid whole brain imaging of neural activity in freely behaving larval zebrafish (Danio rerio)
Source: eLife. 2017 Sep 20;6:e28158. doi: 10.7554/eLife.28158 (PMC5644961; doi:10.7554/eLife.28158)
Supplement: Source code 3. [file elife-28158-code3.zip › RT analysis code/OpenCV1.1pre.zip!/OpenCV/docs/faq.htm]

OpenCV Documentation (draft)

*Frequently Asked Questions / Troubleshootings / HOWTOs*

# General Questions

---

### How to install OpenCV properly?

Read installation guide


---

### How can I get acquainted with OpenCV fast?

1. Look at samples.- Within Visual Studio you may load OpenCV workspace:
     - opencv.dsw for Microsoft Visual Studio 6.0- opencv.sln for Microsoft Visual Studio .NET 2003- cbuilderx/opencv.bpgr for Borland C++ BuilderX
     select `cvsample` project, build it and run. Look into the code
     and modify it as you wish.- Also, scan through reference manuals - they contain some example code as well.- Search OpenCV archives at http://groups.yahoo.com/group/OpenCV
         for the topic you are interesting in.- Create a new project from scratch
           or modify existing `cvsample`.
           There are application wizards for Microsoft Visual Studio that create OpenCV-aware projects;
           look for them at http://groups.yahoo.com/group/OpenCV
           (Files section - you have to be registered OpenCV@yahoogroups.com user)

---

### Where do I submit Bug reports for the computer vision library?

Send email to OpenCV@yahoogroups.com 
Subject: BUG <....your title...>

---

### How do I send bug reports on the Intel� Integrated Performance Primitives

Send email to developer\_support@intel.com

---

### How do I join the OpenCV forum?

Send email to OpenCV-subscribe@yahoogroups.com,
after you are a member and select your login, you can read the web group at
http://groups.yahoo.com/group/OpenCV

---

### How do I modify the web group so that I don't receive tons of email's?

To get the messages real time, or once a day as a daily digest,
you can go to http://groups.yahoo.com/mygroups
and choose your setting from the pull down list to the right of OpenCV.;

---

### Ok, I found the group completely useless for me. How can I unsubscribe?

Mail to OpenCV-unsubscribe@yahoogroups.com
with subject **[OpenCV]** and arbitrary message contents.

---

### When using OpenCV and IPL simultaneously, I get compiler errors. How to resolve this problem?

To be completely independent from IPL, OpenCV duplicates declarations of IplImage
and few other structures and constants if it is not told explicitly that IPL is present.
Defining HAVE\_IPL before including OpenCV headers or putting "#include <ipl.h>"
before OpenCV headers resolves the conflict.

---

### Does OpenCV work on other processors?

Yes, OpenCV itself is open source and it is quite portable, especially across
32-bit platforms. On the other hand, OpenCV can run much faster on Intel processors
because of IPP.

---

# Windows® OS related Qs:

---

### When I try to build one of the apps, I get an error, streams.h not found.

You need install and setup DirectShow SDK that is now a part of Platform SDK
(Windows Server 2003 SP1 SDK or later).
Here is the procedure:

1. Download the Platform SDK from
   http://www.microsoft.com/msdownload/platformsdk/sdkupdate/  
   and DirectX SDK from 
   http://msdn.microsoft.com/directx/
   (They are huge, but you can download and install them by parts).  
   If it doesn't work for you, consider HighGUI that can capture video via VFW or MIL- Install Platform SDK *together* with DirectShow SDK.  
     Install DirectX SDK (with or without sample code).- Build baseclasses.
       See <PlatformSDKInstallFolder>\samples\multimedia\directshow\readme.txt.- Copy the built libraries (called strmbase.lib and strmbasd.lib
         in Release and Debug versions, respectively) to
         <PlatformSDKInstallFolder>\lib.- In Developer Studio add the following paths:  
           <DirectXSDKInstallFolder>\include  
           <PlatformSDKInstallFolder>\include  
           <PlatformSDKInstallFolder>\samples\multimedia\directshow\baseclasses  
           to the includes' search path
           (at Tools->Options->Directories->Include files in case of Visual Studio 6.0,  
           at Tools->Options->Projects and Solutions->VC++ Directories->Include files in case
           of Visual Studio 2005)  
           Add  
           <DirectXSDKInstallFolder>\lib  
           <PlatformSDKInstallFolder>\lib  
           to the libraries' search path (in the same dialog, ...->"Library files" page)

           NOTE: PUT THE ADDED LINES ON THE VERY TOP OF THE LISTS, OTHERWISE YOU MAY STILL GET
           COMPILER OR LINKER ERRORS. This is necessary, because Visual Studio
           may include older versions of the same headers and libraries.

           - Now you can build OpenCV DirectShow filters.

---

### After installing DirectX SDK I'm still getting linker error about undefined or redefined "TransInPlace" filter class constructors etc.

Put the paths to the SDKs' include and lib folders on the top of the search lists.

---

### When I use try to use cvcam, it just crashes

Make sure, you registered ProxyTrans.ax and SyncFilter.ax


---

### How to register \*.ax (DirectShow filter)?

Open the file (within explorer) using regsvr32.exe (under Win2000 it is done by
Open with->Choose Program...->Browse...->c:\windows\system32\regsvr32.exe (path may be different).
You may remember association to save clicks later.

---

### Filter couldn't be registered (regsvr32 reports an error)

The most probable reason is that the filter requires some DLLs that are not in the path.
In case of OpenCV make sure <OpenInstallFolder>\bin is in the path


---

### cxcore096d.dll or cxcored.lib seem to be missing

cxcore096d.dll means debug version of cxcore DLL and cxcored.lib is the import library for cxcore096d.dll.
Open OpenCV workspace, select "cxcore" as active project and
select "Win32 Debug" configuration. Build the library and you will get bin\cxcore096d.dll and
lib\cxcored.lib files. The same should be repeat for *all* the other OpenCV components
- name of binary, ending with *d* means Debug version that are not shipped.

---

### When compiling HighGUI I get the error message "mil.h is not found"

mil.h is a part of Matrox Imaging Library (MIL) that is usually supplied with
Matrox (or compatible) framegrabbers, such as Meteor, Meteor II etc.

- If you have such
  a framegrabber and MIL installed, add mil\include and mil\lib to the search paths
  within Developer Studio (submenu Tools->Options->Directories).- If you do not have MIL, just ignore the error. The file mil.h is only required
    to build MIL-aware version of Highgui "Win32 MIL Debug" or "Win32 MIL Release".
    Select "Win32 Debug" or "Win32 Release" configuration of highgui
    (submenu Build->Set Active Configuration...) instead - these versions of highgui
    can still be used to grab video via VFW interface, work with AVIs and still images.

---

### How can I debug DirectShow filter?

- Open workspace with the filter (e.g. opencv.dsw),- select the filter as active project and build it in debug configuration,- switch to explorer for a minute to register debug version of the filter
      (e.g. regsvr32 camshiftd.ax) (it needs to be done only when debug/release version are switched - not
      every time when filter is recompiled, because registry stores only the filter name),- get back to Developer Studio and start debugging session (F5).
        It will ask, what application do you want to run to debug the module.
        You may choose camshiftdemo to debug camshift.ax and
        DirectX SDK tool graphedit to debug arbitrary DirectShow filter.- Within graphedit build filter graph (e.g. camera->camshift->renderer)- Save the graph (you may just load it next time)- Set the breakpoint inside ::Transform method of the filter or in other location.- Run the filter and ... have fun

---

### How can I create Developer Studio project to start playing with OpenCV

(note: this is a lengthy answer)

To create your own OpenCV-based project in Developer Studio
from scratch do the following:

1. Within Developer Studio create new application:
   1. select from menu "File"->"New..."->"Projects" tab.
      Choose "Win32 Application" or "Win32 console application" - the latter is
      the easier variant and the both sample projects have this type.- type the project name and choose location- you may create own workspace for the project ("Create new workspace")
          or include the new project into the currently loaded workspace
          ("Add to current workspace").- click "next" button- choose "An empty project", click "Finish", "OK".
   After the above steps done Developer Studio will create the project
   folder (by default it has the same name as the project),
   <project name>.dsp file and, optionally, <project name>.dsw,.ncb ... files
   if you create own workspace.- Add a file to the project:
     - select from menu "File"->"New..."->"Files" tab.- choose "C++ Source File", type file name and press "OK"- add OpenCV-related #include directives:

           ```
                   #include "cv.h"
                   /* #inlcude "cvaux.h" // experimental stuff (if need) */
                   #include "highgui.h"
           ```

           Or, you may copy some existing file (say, opencv\samples\c\morphology.c) to the project folder,
           open it and add to the project
           (right click in editor view -> "Insert File into Project" -> <your project name> ).
     - Customize project settings:
       - Activate project setting dialog by choosing menu item
         "Project"->"Settings...".- Select your project in the right pane.- Tune settings, common to both Release and Debug configurations:
             - Select "Settings For:"->"All Configurations"- Choose "C/C++" tab -> "Preprocessor" category -> "Additional Include Directories:".
                 Add comma-separated relative (to the .dsp file) or absolute paths
                 to opencv\cxcore\include, opencv\cv\include, opencv\otherlibs\highgui and, optionally,
                 opencv\cvaux\include.- Choose "Link" tab -> "Input" category -> "Additional library path:".
                   Add the paths to all necessary import libraries (cxcore[d].lib cv[d].lib hihghui[d].lib cvaux[d].lib)
             - Tune settings for "Debug" configuration
               - Select "Settings For:"->"Win32 Debug".- Choose "Link" tab -> "General" category -> "Object/library modules".
                   Add space-separated cvd.lib, highguid.lib, cvauxd.lib (optionally)- You may also want to change location and name of output file. For example,
                     if you want the output .exe file to be put into the project folder, rather
                     than Debug/ subfolder, you may type ./<exe-name>d.exe in
                     "Link" tab -> "General" category -> "Output file name:".
               - Tune settings for "Release" configuration
                 - Select "Settings For:"->"Win32 Release".- Choose "Link" tab -> "General" category -> "Object/library modules".
                     Add space-separated cv.lib, highgui.lib, cvaux.lib (optionally)- Optionally, you may change name of the .exe file:
                       type ./<exe-name>.exe in "Link" tab -> "General" category -> "Output file name:".
       - Add dependency projects into workspace:
         - Choose from menu: "Project" -> "Insert project into workspace".- Select opencv\cv\make\cv.dsp.- Do the same for opencv\cvaux\make\cvaux.dsp, opencv\otherlibs\highgui\highgui.dsp.- Set dependencies:
                 - Choose from menu: "Project" -> "Dependencies..."- For "cv" choose "cxcore",- For "cvaux" choose "cv", "cxcore",- for "highgui" choose "cxcore",- for your project choose all: "cxcore", "cv", "cvaux", "highgui".
                 The dependencies customization allows to automatically build debug versions
                 of opencv libraries and rebuild the binaries if the sources are changed somehow.
         - That's it. Now compile and run everything.

---

# Linux Related Qs:

TODO

---

# Technical Questions on Library use:

---

### How to access image pixels

(The coordinates are 0-based and counted from image origin, either top-left
(img->origin=IPL\_ORIGIN\_TL) or bottom-left (img->origin=IPL\_ORIGIN\_BL)

- Suppose, we have 8-bit 1-channel image I (IplImage\* img):

  ```
  I(x,y) ~ ((uchar*)(img->imageData + img->widthStep*y))[x]
  ```

  - Suppose, we have 8-bit 3-channel image I (IplImage\* img):

    ```
    I(x,y)blue ~ ((uchar*)(img->imageData + img->widthStep*y))[x*3]
    I(x,y)green ~ ((uchar*)(img->imageData + img->widthStep*y))[x*3+1]
    I(x,y)red ~ ((uchar*)(img->imageData + img->widthStep*y))[x*3+2]
    ```

    e.g. increasing brightness of point (100,100) by 30 can be done this way:

    ```
    CvPoint pt = {100,100};
    ((uchar*)(img->imageData + img->widthStep*pt.y))[pt.x*3] += 30;
    ((uchar*)(img->imageData + img->widthStep*pt.y))[pt.x*3+1] += 30;
    ((uchar*)(img->imageData + img->widthStep*pt.y))[pt.x*3+2] += 30;
    ```

    or more efficiently

    ```
    CvPoint pt = {100,100};
    uchar* temp_ptr = &((uchar*)(img->imageData + img->widthStep*pt.y))[x*3];
    temp_ptr[0] += 30;
    temp_ptr[1] += 30;
    temp_ptr[2] += 30;
    ```

    - Suppose, we have 32-bit floating point, 1-channel image I (IplImage\* img):

      ```
      I(x,y) ~ ((float*)(img->imageData + img->widthStep*y))[x]
      ```

      - Now, the general case: suppose, we have N-channel image of type T:

        ```
        I(x,y)c ~ ((T*)(img->imageData + img->widthStep*y))[x*N + c]
        or you may use macro CV_IMAGE_ELEM( image_header, elemtype, y, x_Nc )
        I(x,y)c ~ CV_IMAGE_ELEM( img, T, y, x*N + c )
        ```

There are functions that work with arbitrary (up to 4-channel) images and matrices
(cvGet2D, cvSet2D), but they are pretty slow.

---

### How to access matrix elements?

The technique is very similar.
(In the samples below i - 0-based row index, j - 0-based column index)

- Suppose, we have 32-bit floating point real matrix M (CvMat\* mat):

  ```
  M(i,j) ~ ((float*)(mat->data.ptr + mat->step*i))[j]
  ```

  - Suppose, we have 64-bit floating point complex matrix M (CvMat\* mat):

    ```
    Re M(i,j) ~ ((double*)(mat->data.ptr + mat->step*i))[j*2]
    Im M(i,j) ~ ((double*)(mat->data.ptr + mat->step*i))[j*2+1]
    ```

    - For single-channel matrices there is a macro CV\_MAT\_ELEM( matrix, elemtype, row, col ),
      i.e. for 32-bit floating point real matrix

      ```
      M(i,j) ~ CV_MAT_ELEM( mat, float, i, j ),
      ```

      e.g. here is
      initialization of 3x3 identity matrix:

      ```
      CV_MAT_ELEM( mat, float, 0, 0 ) = 1.f;
      CV_MAT_ELEM( mat, float, 0, 1 ) = 0.f;
      CV_MAT_ELEM( mat, float, 0, 2 ) = 0.f;
      CV_MAT_ELEM( mat, float, 1, 0 ) = 0.f;
      CV_MAT_ELEM( mat, float, 1, 1 ) = 1.f;
      CV_MAT_ELEM( mat, float, 1, 2 ) = 0.f;
      CV_MAT_ELEM( mat, float, 2, 0 ) = 0.f;
      CV_MAT_ELEM( mat, float, 2, 1 ) = 0.f;
      CV_MAT_ELEM( mat, float, 2, 2 ) = 1.f;
      ```

---

### How to process my data with OpenCV

Suppose, you have 300x200 32-bit floating point image/array, that
resides in 60000-element array.

```
int cols = 300, rows = 200;
float* myarr = new float[rows*cols];

// step 1) initializing CvMat header
CvMat mat = cvMat( rows, cols,
                   CV_32FC1, // 32-bit floating-point, single channel type
                   myarr // user data pointer (no data is copied)
                   );
// step 2) using cv functions, e.g. calculating l2 (Frobenius) norm
double norm = cvNorm( &mat, 0, CV_L2 );

...
delete myarr;
```

Other scenaria are described in the reference manual.
See cvCreateMatHeader, cvInitMatHeader, cvCreateImageHeader, cvSetData etc.

---

### How to load and display image

```
/* usage: prog <image_name> */
#include "cv.h"
#include "highgui.h"

int main( int argc, char** argv )
{
    IplImage* img;
    if( argc == 2 && (img = cvLoadImage( argv[1], 1)) != 0 )
    {
        cvNamedWindow( "Image view", 1 );
        cvShowImage( "Image view", img );
        cvWaitKey(0); // very important, contains event processing loop inside
        cvDestroyWindow( "Image view" );
        cvReleaseImage( &img );
        return 0;
    }
    return -1;
}
```

---

### How to find and process contours

Look at squares demo

---

### How to calibrate camera using OpenCV

TODO
