# Supplementary material for: Rapid whole brain imaging of neural activity in freely behaving larval zebrafish (Danio rerio)
Source: eLife. 2017 Sep 20;6:e28158. doi: 10.7554/eLife.28158 (PMC5644961; doi:10.7554/eLife.28158)
Supplement: Source code 3. [file elife-28158-code3.zip › RT analysis code/OpenCV1.1pre.zip!/OpenCV/docs/index.htm]

OpenCV Documentation

|  |  |
| --- | --- |
|  | *Open Source Computer Vision Library* |


# What is OpenCV

OpenCV means Open Source Computer Vision Library.
It is a collection of C functions and a few C++ classes that implement
many popular Image Processing and Computer Vision algorithms.

---

# OpenCV Book

The official book for OpenCV is published by O'Reilly Press
Learning OpenCV Computer Vision with the OpenCV Library
by Gary Bradski and Adrian Kaehler
http://www.amazon.com/Learning-OpenCV-Computer-Vision-Library/dp/0596516134

This book is:

1. A tutorial on computer vision in general- A user's guide to OpenCV- A source of useful working sample code and detailed function descriptions.

---

# The key features

OpenCV provides cross-platform middle-to-high level API that includes
about 300 C functions and a few C++ classes. Also there are
constantly improving Python bindings to OpenCV, see
interfaces/swig/python and samples/python.
OpenCV has no strict dependencies on external libraries,
though it can use some (such as libjpeg, ffmpeg, GTK+ etc.)
when it is possible.

OpenCV is free for both non-commercial and commercial use
(see the license for details).

OpenCV provides transparent interface to Intel® Integrated Performance
Primitives (IPP).
That is, it loads automatically IPP libraries optimized
for specific processor at runtime, if they are available.
More information about IPP can be retrieved at
http://www.intel.com/software/products/ipp/index.htm

---

# Who created it

The list of authors and major contributors can be found in the file THANKS.

---

# What's New

See the ChangeLog.

---

# Where to get OpenCV

Go http://www.sourceforge.net/projects/opencvlibrary.
If it does not work, type "OpenCV" in
Google (http://www.google.com).

---

# If you have a problem with installing/running/using OpenCV

1. Visit OpenCV Wiki-pages at
   http://opencvlibrary.sourceforge.net and
   look for the information there (and/or add it when you have found a solution)- Read FAQs- Search through OpenCV archives at www.yahoogroups.com
       (http://groups.yahoo.com/group/OpenCV/)- Join OpenCV mailing list at yahoo groups (see FAQs on how to do it) and mail your questions
         (the mailing list will probably migrate to
         OpenCV's SourceForge site)- Look at the OpenCV sample code, read the reference manual :)

---

# OpenCV Reference Manuals

- CXCORE Reference Manual- CV Reference Manual- Machine Learning Reference Manual- HighGUI Reference Manual

You may also look at the PDF manual, but do not trust it much -
it is pretty out of date, especially, the reference part.

---
