# Supplementary material for: Rapid whole brain imaging of neural activity in freely behaving larval zebrafish (Danio rerio)
Source: eLife. 2017 Sep 20;6:e28158. doi: 10.7554/eLife.28158 (PMC5644961; doi:10.7554/eLife.28158)
Supplement: Source code 3. [file elife-28158-code3.zip › RT analysis code/OpenCV1.1pre.zip!/OpenCV/docs/opencvman_old.pdf]

Open Source Computer Vision Library

Reference Manual

Copyright © 2001 Intel Corporation
All Rights Reserved
Issued in U.S.A.
Order Number: A77028-004

World Wide Web: http://developer.intel.com

Version	Version History	Date
-001	Original Issue.	12/2000
-002	Document OpenCV Reference Manual Beta 1 version. Changed Manual structure.	04/2001
-003	Document OpenCV Reference Manual Beta 2 version. Added `ContourBoundingRect` function.	08/2001
-004	Document OpenCV Reference Manual Beta 2 version. Updated 22 and added 35 functions to Basic Structures and Operations Reference.	12/2001

This OpenCV Reference Manual as well as the software described in it is furnished under license and may only be used or copied in accordance with the terms of the license. The information in this manual is furnished for informational use only, is subject to change without notice, and should not be construed as a commitment by Intel Corporation. Intel Corporation assumes no responsibility or liability for any errors or inaccuracies that may appear in this document or any software that may be provided in association with this document.

Except as permitted by such license, no part of this document may be reproduced, stored in a retrieval system, or transmitted in any form or by any means without the express written consent of Intel Corporation.

INFORMATION IN THIS DOCUMENT IS PROVIDED IN CONNECTION WITH INTEL PRODUCTS. NO LICENSE, EXPRESS OR IMPLIED, BY ESTOPPEL OR OTHERWISE, TO ANY INTELLECTUAL PROPERTY RIGHTS IS GRANTED BY THIS DOCUMENT. EXCEPT AS PROVIDED IN INTEL'S TERMS AND CONDITIONS OF SALE FOR SUCH PRODUCTS, INTEL ASSUMES NO LIABILITY WHATSOEVER, AND INTEL DISCLAIMS ANY EXPRESS OR IMPLIED WARRANTY, RELATING TO SALE AND/OR USE OF INTEL PRODUCTS INCLUDING LIABILITY OR WARRANTIES, RELATING TO FITNESS FOR A PARTICULAR PURPOSE , MERCHANTABILITY, OR INFRINGEMENT OF ANY PATENT, COPYRIGHT OR OTHER INTELLECTUAL PROPERTY RIGHT. INTEL PRODUCTS ARE NOT INTENDED FOR USE IN MEDICAL, LIFE SAVING, OR LIFE SUSTAINING APPLICATIONS. INTEL MAY MAKE CHANGES TO SPECIFICATIONS AND PRODUCT DESCRIPTIONS AT ANY TIME, WITHOUT NOTICE.

Designers must not rely on the absence or characteristics of any features or instructions marked "reserved" or "undefined." Intel reserves these for future definition and shall have no responsibility whatsoever for conflicts or incompatibilities arising from future changes to them.

The OpenCV may contain design defects or errors known as errata which may cause the product to deviate from published specifications. Current characterized errata are available on request.

Intel, the Intel logo and Pentium are trademarks or registered trademarks of Intel Corporation or its subsidiaries in the United States and other countries.

*Other names and brands may be claimed as the property of others.

Copyright © Intel Corporation 2001.

Contents

Chapter Contents

Chapter 1
Overview

About This Software 1-1
- Why We Need OpenCV Library 1-2
- Relation Between OpenCV and Other Libraries 1-2
- Data Types Supported 1-3
- Error Handling 1-3
- Hardware and Software Requirements 1-3
- Platforms Supported 1-4

About This Manual 1-4
- Manual Organization 1-4
- Function Descriptions 1-8
- Audience for This Manual 1-8
- On-line Version 1-8
- Related Publications 1-8

Notational Conventions 1-8
- Font Conventions 1-9
- Naming Conventions 1-9
- Function Name Conventions 1-9

Chapter 2
Motion Analysis and Object Tracking

Background Subtraction 2-1

Motion Templates 2-2

Motion Representation and Normal Optical Flow Method 2-2
Motion Representation 2-2
A) Updating MHI Images 2-3
B) Making Motion Gradient Image 2-3
C) Finding Regional Orientation or Normal Optical Flow 2-6
Motion Segmentation 2-7
CamShift 2-9
Mass Center Calculation for 2D Probability Distribution 2-11
CamShift Algorithm 2-12
Calculation of 2D Orientation 2-14
Active Contours 2-15
Optical Flow 2-18
Lucas & Kanade Technique 2-19
Horn & Schunck Technique 2-19
Block Matching 2-20
Estimators 2-20
Models 2-20
Estimators 2-21
Kalman Filtering 2-22
ConDensation Algorithm 2-23

Chapter 3
Image Analysis

Contour Retrieving 3-1
Basic Definitions 3-1
Contour Representation 3-3
Contour Retrieving Algorithm 3-4
Features 3-5
Fixed Filters 3-5
Sobel Derivatives 3-6
Optimal Filter Kernels with Floating Point Coefficients 3-9
First Derivatives 3-9
Second Derivatives 3-10

Laplacian Approximation 3-10
Feature Detection 3-10
Corner Detection 3-11
Canny Edge Detector 3-11
Hough Transform 3-14
Image Statistics 3-15
Pyramids 3-15
Morphology 3-19
Flat Structuring Elements for Gray Scale 3-21
Distance Transform 3-23
Thresholding 3-24
Flood Filling 3-25
Histogram 3-25
Histograms and Signatures 3-26
Example Ground Distances 3-29
Lower Boundary for EMD 3-30

Chapter 4
Structural Analysis

Contour Processing 4-1
Polygonal Approximation 4-1
Douglas-Peucker Approximation 4-4
Contours Moments 4-5
Hierarchical Representation of Contours 4-8
Geometry 4-14
Ellipse Fitting 4-14
Line Fitting 4-15
Convexity Defects 4-16

Chapter 5
Object Recognition

Eigen Objects 5-1
Embedded Hidden Markov Models 5-2

Chapter 6
3D Reconstruction

Camera Calibration........ 6-1
Camera Parameters........ 6-1
Pattern........ 6-3
View Morphing........ 6-3
Algorithm........ 6-4
Using Functions for View Morphing Algorithm........ 6-7
POSIT........ 6-8
Geometric Image Formation........ 6-8
Pose Approximation Method........ 6-10
Algorithm........ 6-12
Gesture Recognition........ 6-14

Chapter 7
Basic Structures and Operations

Image Functions........ 7-1
Dynamic Data Structures........ 7-4
Memory Storage........ 7-4
Sequences........ 7-5
Writing and Reading Sequences........ 7-6
Sets........ 7-8
Graphs........ 7-11
Matrix Operations........ 7-15
Interchangability between IplImage and CvMat........ 7-18
Drawing Primitives........ 7-18
Utility........ 7-19

Chapter 8
Library Technical Organization and System Functions

Error Handling........ 8-1

Memory Management ... 8-1
Interaction With Low-Level Optimized Functions ... 8-1
User DLL Creation ... 8-1

Chapter 9
Motion Analysis and Object Tracking Reference

Background Subtraction Functions ... 9-3
- Acc ... 9-3
- SquareAcc ... 9-4
- MultiplyAcc ... 9-4
- RunningAvg ... 9-5

Motion Templates Functions ... 9-6
- UpdateMotionHistory ... 9-6
- CalcMotionGradient ... 9-6
- CalcGlobalOrientation ... 9-7
- SegmentMotion ... 9-8

CamShift Functions ... 9-9
- CamShift ... 9-9
- MeanShift ... 9-10

Active Contours Function ... 9-11
- SnakeImage ... 9-11

Optical Flow Functions ... 9-12
- CalcOpticalFlowHS ... 9-12
- CalcOpticalFlowLK ... 9-13
- CalcOpticalFlowBM ... 9-13
- CalcOpticalFlowPyrLK ... 9-14

Estimators Functions ... 9-16
- CreateKalman ... 9-16
- ReleaseKalman ... 9-16
- KalmanUpdateByTime ... 9-17
- KalmanUpdateByMeasurement ... 9-17
- CreateConDensation ... 9-17

ReleaseConDensation 9-18
ConDensInitSampleSet 9-18
ConDensUpdateByTime 9-19
Estimators Data Types 9-19

Chapter 10
Image Analysis Reference

Contour Retrieving Functions 10-6
FindContours 10-6
StartFindContours 10-7
FindNextContour 10-8
SubstituteContour 10-9
EndFindContours 10-9
Features Functions 10-10
Fixed Filters Functions 10-10
Laplace 10-10
Sobel 10-10
Feature Detection Functions 10-11
Canny 10-11
PreCornerDetect 10-12
CornerEigenValsAndVecs 10-12
CornerMinEigenVal 10-13
FindCornerSubPix 10-14
GoodFeaturesToTrack 10-16
Hough Transform Functions 10-17
HoughLines 10-17
HoughLinesSDiv 10-18
HoughLinesP 10-19
Image Statistics Functions 10-20
CountNonZero 10-20
SumPixels 10-20
Mean 10-21
Mean_StdDev 10-21

MinMaxLoc 10-22
Norm 10-22
Moments 10-24
GetSpatialMoment 10-25
GetCentralMoment 10-25
GetNormalizedCentralMoment 10-26
GetHuMoments 10-27
Pyramid Functions 10-28
PyrDown 10-28
PyrUp 10-28
PyrSegmentation 10-29
Morphology Functions 10-30
CreateStructuringElementEx 10-30
ReleaseStructuringElement 10-31
Erode 10-31
Dilate 10-32
MorphologyEx 10-33
Distance Transform Function 10-34
DistTransform 10-34
Threshold Functions 10-36
AdaptiveThreshold 10-36
Threshold 10-38
Flood Filling Function 10-40
FloodFill 10-40
Histogram Functions 10-41
CreateHist 10-41
ReleaseHist 10-42
MakeHistHeaderForArray 10-42
QueryHistValue_1D 10-43
QueryHistValue_2D 10-43
QueryHistValue_3D 10-44
QueryHistValue_nD 10-44

GetHistValue_1D 10-45
GetHistValue_2D 10-45
GetHistValue_3D 10-46
GetHistValue_nD 10-46
GetMinMaxHistValue 10-47
NormalizeHist 10-47
ThreshHist 10-48
CompareHist 10-48
CopyHist 10-49
SetHistBinRanges 10-50
CalcHist 10-50
CalcBackProject 10-51
CalcBackProjectPatch 10-52
CalcEMD 10-54
CalcContrastHist 10-55
Pyramid Data Types 10-56
Histogram Data Types 10-57

Chapter 11
Structural Analysis Reference

Contour Processing Functions 11-3
ApproxChains 11-3
StartReadChainPoints 11-4
ReadChainPoint 11-5
ApproxPoly 11-5
DrawContours 11-6
ContourBoundingRect 11-7
ContoursMoments 11-8
ContourArea 11-8
MatchContours 11-9
CreateContourTree 11-10
ContourFromContourTree 11-11
MatchContourTrees 11-12

Geometry Functions 11-12
FitEllipse 11-12
FitLine2D 11-13
FitLine3D 11-15
Project3D 11-16
ConvexHull 11-17
ContourConvexHull 11-18
ConvexHullApprox 11-18
ContourConvexHullApprox 11-20
CheckContourConvexity 11-21
ConvexityDefects 11-21
MinAreaRect 11-22
CalcPGH 11-23
MinEnclosingCircle 11-24
Contour Processing Data Types 11-24
Geometry Data Types 11-25

Chapter 12
Object Recognition Reference

Eigen Objects Functions 12-3
CalcCovarMatrixEx 12-3
CalcEigenObjects 12-4
CalcDecompCoeff 12-5
EigenDecomposite 12-6
EigenProjection 12-7
Use of Eigen Object Functions 12-7
Embedded Hidden Markov Models Functions 12-12
Create2DHMM 12-12
Release2DHMM 12-13
CreateObsInfo 12-13
ReleaseObsInfo 12-14
ImgToObs_DCT 12-14
UniformImgSegm 12-15

InitMixSegm 12-16
EstimateHMMStateParams 12-17
EstimateTransProb 12-17
EstimateObsProb 12-18
EViterbi 12-18
MixSegmL2 12-19
HMM Structures 12-19

Chapter 13
3D Reconstruction Reference

Camera Calibration Functions 13-4
CalibrateCamera 13-4
CalibrateCamera_64d 13-5
FindExtrinsicCameraParams 13-6
FindExtrinsicCameraParams_64d 13-7
Rodrigues 13-7
Rodrigues_64d 13-8
UnDistortOnce 13-9
UnDistortInit 13-9
UnDistort 13-10
FindChessBoardCornerGuesses 13-11
View Morphing Functions 13-12
FindFundamentalMatrix 13-12
MakeScanlines 13-13
PreWarpImage 13-13
FindRuns 13-14
DynamicCorrespondMulti 13-15
MakeAlphaScanlines 13-16
MorphEpilinesMulti 13-16
PostWarpImage 13-17
DeleteMoire 13-18
POSIT Functions 13-19
CreatePOSITObject 13-19

POSIT 13-19
ReleasePOSITObject... 13-20
Gesture Recognition Functions 13-21
FindHandRegion 13-21
FindHandRegionA 13-22
CreateHandMask 13-23
CalcImageHomography 13-23
CalcProbDensity 13-24
MaxRect... 13-25

Chapter 14
Basic Structures and Operations Reference

Image Functions Reference 14-9
CreateImageHeader 14-9
CreateImage 14-9
ReleaseImageHeader 14-10
ReleaseImage.. 14-10
CreateImageData.. 14-11
ReleaseImageData 14-12
SetImageData 14-12
SetImageCOI 14-13
SetImageROI 14-13
GetImageRawData 14-14
InitImageHeader 14-14
CopyImage 14-15
Pixel Access Macros 14-15
CV_INIT_PIXEL_POS 14-17
CV_MOVE_TO 14-17
CV_MOVE 14-18
CV_MOVE_WRAP 14-18
CV_MOVE_PARAM.. 14-19
CV_MOVE_PARAM_WRAP 14-19
Dynamic Data Structures Reference 14-21

Memory Storage Reference 14-21
CreateMemStorage.. 14-22
CreateChildMemStorage 14-22
ReleaseMemStorage 14-23
ClearMemStorage... 14-23
SaveMemStoragePos 14-24
RestoreMemStoragePos... 14-24
Sequence Reference 14-26
CreateSeq... 14-29
SetSeqBlockSize 14-30
SeqPush 14-30
SeqPop 14-31
SeqPushFront.. 14-31
SeqPopFront.. 14-32
SeqPushMulti... 14-32
SeqPopMulti 14-33
SeqInsert 14-33
SeqRemove 14-34
ClearSeq... 14-34
GetSeqElem 14-35
SeqElemIdx 14-35
CvtSeqToArray.. 14-36
MakeSeqHeaderForArray... 14-36
Writing and Reading Sequences Reference 14-37
StartAppendToSeq 14-37
StartWriteSeq.. 14-38
EndWriteSeq... 14-39
FlushSeqWriter 14-39
StartReadSeq.. 14-40
GetSeqReaderPos... 14-41
SetSeqReaderPos 14-41
Sets Reference 14-42

CreateSet 14-42
SetAdd 14-42
SetRemove 14-43
GetSetElem 14-43
ClearSet 14-44
Graphs Reference 14-46
CreateGraph 14-46
GraphAddVtx 14-46
GraphRemoveVtx 14-47
GraphRemoveVtxByPtr 14-47
GraphAddEdge 14-48
GraphAddEdgeByPtr 14-49
GraphRemoveEdge 14-50
GraphRemoveEdgeByPtr 14-50
FindGraphEdge 14-51
FindGraphEdgeByPtr 14-52
GraphVtxDegree 14-52
GraphVtxDegreeByPtr 14-53
ClearGraph 14-54
GetGraphVtx 14-54
GraphVtxIdx 14-54
GraphEdgeIdx 14-55
Graphs Data Structures 14-55
Matrix Operations Reference 14-57
CreateMat 14-58
CreateMatHeader 14-58
ReleaseMat 14-59
ReleaseMatHeader 14-60
InitMatHeader 14-60
CloneMat 14-61
SetData 14-62
GetMat 14-62

GetAt 14-63
SetAt 14-64
GetAtPtr 14-65
GetSubArr 14-65
GetRow 14-66
GetCol 14-66
GetDiag 14-67
GetRawData 14-67
GetSize 14-68
CreateData 14-69
AllocArray 14-69
ReleaseData 14-69
FreeArray 14-70
Copy 14-70
Set 14-71
Add 14-71
AddS 14-72
Sub 14-73
SubS 14-73
SubRS 14-74
Mul 14-75
And 14-75
AndS 14-76
Or 14-77
OrS 14-78
Xor 14-79
XorS 14-80
DotProduct 14-81
CrossProduct 14-82
ScaleAdd 14-82
MatMulAdd 14-83
MatMulAddS 14-84

MulTransposed 14-85
Invert 14-85
Trace 14-86
Det 14-86
Mahalonobis 14-86
Transpose 14-87
Flip 14-87
Reshape 14-88
SetZero 14-89
SetIdentity 14-90
SVD 14-90
PseudoInv 14-91
EigenVV 14-92
PerspectiveTransform 14-93
Drawing Primitives Reference 14-94
Line 14-94
LineAA 14-94
Rectangle 14-95
Circle 14-96
Ellipse 14-96
EllipseAA 14-98
FillPoly 14-98
FillConvexPoly 14-99
PolyLine 14-100
PolyLineAA 14-100
InitFont 14-101
PutText 14-102
GetTextSize 14-102
Utility Reference 14-103
AbsDiff 14-103
AbsDiffS 14-104
MatchTemplate 14-104

CvtPixToPlane 14-107
CvtPlaneToPix 14-107
ConvertScale 14-108
LUT 14-109
InitLineIterator 14-110
SampleLine 14-111
GetRectSubPix 14-111
bFastArctan 14-112
Sqrt 14-112
bSqrt 14-113
InvSqrt 14-113
bInvSqrt 14-114
bReciprocal 14-114
bCartToPolar 14-115
bFastExp 14-115
bFastLog 14-116
RandInit 14-116
bRand 14-117
Rand 14-117
FillImage 14-118
RandSetRange 14-118
KMeans 14-119

Chapter 15
System Functions
LoadPrimitives 15-1
GetLibraryInfo 15-2

Bibliography

Appendix A

Supported Image Attributes and Operation Modes

Glossary

Index

Overview 1

This manual describes the structure, operation, and functions of the Open Source Computer Vision Library (OpenCV) for Intel® architecture. The OpenCV Library is mainly aimed at real time computer vision. Some example areas would be Human-Computer Interaction (HCI); Object Identification, Segmentation, and Recognition; Face Recognition; Gesture Recognition; Motion Tracking, Ego Motion, and Motion Understanding; Structure From Motion (SFM); and Mobile Robotics.

The OpenCV Library is a collection of low-overhead, high-performance operations performed on images.

This manual explains the OpenCV Library concepts as well as specific data type definitions and operation models used in the image processing domain. The manual also provides detailed descriptions of the functions included in the OpenCV Library software.

This chapter introduces the OpenCV Library software and explains the organization of this manual.

About This Software

The OpenCV implements a wide variety of tools for image interpretation. It is compatible with Intel® Image Processing Library (IPL) that implements low-level operations on digital images. In spite of primitives such as binarization, filtering, image statistics, pyramids, OpenCV is mostly a high-level library implementing algorithms for calibration techniques (Camera Calibration), feature detection (Feature) and tracking (Optical Flow), shape analysis (Geometry, Contour Processing), motion

analysis (Motion Templates, Estimators), 3D reconstruction (View Morphing), object segmentation and recognition (Histogram, Embedded Hidden Markov Models, Eigen Objects).

The essential feature of the library along with functionality and quality is performance. The algorithms are based on highly flexible data structures (Dynamic Data Structures) coupled with IPL data structures; more than a half of the functions have been assembler-optimized taking advantage of Intel® Architecture (Pentium® MMX™, Pentium® Pro, Pentium® III, Pentium® 4).

Why We Need OpenCV Library

The OpenCV Library is a way of establishing an open source vision community that will make better use of up-to-date opportunities to apply computer vision in the growing PC environment. The software provides a set of image processing functions, as well as image and pattern analysis functions. The functions are optimized for Intel® architecture processors, and are particularly effective at taking advantage of MMX™ technology.

The OpenCV Library has platform-independent interface and supplied with whole C sources. OpenCV is open.

Relation Between OpenCV and Other Libraries

OpenCV is designed to be used together with Intel® Image Processing Library (IPL) and extends the latter functionality toward image and pattern analysis. Therefore, OpenCV shares the same image format (`IplImage`) with IPL.

Also, OpenCV uses Intel® Integrated Performance Primitives (IPP) on lower-level, if it can locate the IPP binaries on startup.

IPP provides cross-platform interface to highly-optimized low-level functions that perform domain-specific operations, particularly, image processing and computer vision primitive operations. IPP exists on multiple platforms including IA32, IA64, and StrongARM. OpenCV can automatically benefit from using IPP on all these platforms.

Data Types Supported

There are a few fundamental types OpenCV operates on, and several helper data types that are introduced to make OpenCV API more simple and uniform.

The fundamental data types include array-like types: `IplImage` (IPL image), `CvMat` (matrix), growable collections: `CvSeq` (deque), `CvSet`, `CvGraph` and mixed types: `CvHistogram` (multi-dimensional histogram). See Basic Structures and Operations chapter for more details.

Helper data types include: `CvPoint` (2d point), `CvSize` (width and height), `CvTermCriteria` (termination criteria for iterative processes), `IplConvKernel` (convolution kernel), `CvMoments` (spatial moments), etc.

Error Handling

Error handling mechanism in OpenCV is similar to IPL.

There are no return error codes. Instead, there is a global error status that can be set or retrieved via `cvError` and `cvGetErrStatus` functions, respectively. The error handling mechanism is adjustable, e.g., it can be specified, whether `cvError` prints out error message and terminates the program execution afterwards, or just sets an error code and the execution continues.

See Library Technical Organization and System Functions chapter for list of possible error codes and details of error handling mechanism.

Hardware and Software Requirements

The OpenCV software runs on personal computers that are based on Intel® architecture processors and running Microsoft* Windows* 95, Windows 98, Windows 2000, or Windows NT*. The OpenCV integrates into the customer's application or library written in C or C++.

Platforms Supported

The OpenCV software run on Windows platforms. The code and syntax used for function and variable declarations in this manual are written in the ANSI C style. However, versions of the OpenCV for different processors or operating systems may, of necessity, vary slightly.

About This Manual

This manual provides a background for the computer image processing concepts used in the OpenCV software. The manual includes two major parts, one is the Programmer Guide and the other is Reference. The fundamental concepts of each of the library components are extensively covered in the Programmer Guide. The Reference provides the user with specifications of each OpenCV function. The functions are combined into groups by their functionality (chapters 10 through 16). Each group of functions is described along with appropriate data types and macros, when applicable. The manual includes example codes of the library usage.

Manual Organization

This manual includes two principal parts: Programmer Guide and Reference.

The Programmer Guide contains

Overview (Chapter 1) that provides information on the OpenCV software, application area, overall functionality, the library relation to IPL, data types and error handling, along with manual organization and notational conventions.

and the following functionality chapters:

Chapter 2 Motion Analysis and Object Tracking comprising sections:

- Background Subtraction. Describes basic functions that enable building statistical model of background for its further subtraction.

- Motion Templates. Describes motion templates functions designed to generate motion template images that can be used to rapidly determine where a motion occurred, how it occurred, and in which direction it occurred.
- Cam Shift. Describes the functions implemented for realization of “Continuously Adaptive Mean-SHIFT” algorithm (CamShift) algorithm.
- Active Contours. Describes a function for working with active contours (snakes).
- Optical Flow. Describes functions used for calculation of optical flow implementing Lucas & Kanade, Horn & Schunck, and Block Matching techniques.
- Estimators. Describes a group of functions for estimating stochastic models state.

Chapter 3 Image Analysis comprising sections:

- Contour Retrieving. Describes contour retrieving functions.
- Features. Describes various fixed filters, primarily derivative operators (1st & 2nd Image Derivatives); feature detection functions; Hough Transform method of extracting geometric primitives from raster images.
- Image Statistics. Describes a set of functions that compute different information about images, considering their pixels as independent observations of a stochastic variable.
- Pyramids. Describes functions that support generation and reconstruction of Gaussian and Laplacian Pyramids.
- Morphology. Describes an expanded set of morphological operators that can be used for noise filtering, merging or splitting image regions, as well as for region boundary detection.
- Distance Transform. Describes the distance transform functions used for calculating the distance to an object.

- Thresholding. Describes threshold functions used mainly for masking out some pixels that do not belong to a certain range, for example, to extract blobs of certain brightness or color from the image, and for converting grayscale image to bi-level or black-and-white image.
- Flood Filling. Describes the function that performs flood filling of a connected domain.
- Histogram. Describes functions that operate on multi-dimensional histograms.

Chapter 4 Structural Analysis comprising sections:

- Contour Processing. Describes contour processing functions.
- Geometry. Describes functions from computational geometry field: line and ellipse fitting, convex hull, contour analysis.

Chapter 5 Image Recognition comprising sections:

- Eigen Objects. Describes functions that operate on eigen objects.
- Embedded HMM. Describes functions for using Embedded Hidden Markov Models (HMM) in face recognition task.

Chapter 6 3D Reconstruction comprising sections:

- Camera Calibration. Describes undistortion functions and camera calibration functions used for calculating intrinsic and extrinsic camera parameters.
- View Morphing. Describes functions for morphing views from two cameras.
- POSIT. Describes functions that together perform POSIT algorithm used to determine the six degree-of-freedom pose of a known tracked 3D rigid object.
- Gesture Recognition. Describes specific functions for the static gesture recognition technology.

Chapter 7 Basic Structures and Operations comprising sections:

- Image Functions. Describes basic functions for manipulating raster images: creation, allocation, destruction of images. Fast pixel access macros are also described.
- Dynamic Data Structures. Describes several resizable data structures and basic functions that are designed to operate on these structures.
- Matrix Operations. Describes functions for matrix operations: basic matrix arithmetics, eigen problem solution, SVD, 3D geometry and recognition-specific functions.
- Drawing Primitives. Describes simple drawing functions intended mainly to mark out recognized or tracked features in
- Utility. Describes unclassified OpenCV functions.

Chapter 8 Library Technical Organization and System Fuctions comprising sections:

- Error Handling.
- Memory Management.
- Interaction With Low-Level Optimized Functions.
- User DLL Creation.

Reference contains the following chapters describing respective functions, data types and applicable macros:

Chapter 9 Motion Analysis and Object Tracking Reference.

Chapter 10 Image Analysis Reference.

Chapter 11 Structural Analysis Reference.

Chapter 12 Image Recognition Reference.

Chapter 13 3D Reconstruction Reference.

Chapter 14 Basic Structures and Operations Reference.

Chapter 15 System Functions Reference.

The manual also includes Appendix A that describes supported image attributes and operation modes, a Glossary of terms, a Bibliography, and an Index.

Function Descriptions

In Chapters 10 through 16, each function is introduced by name and a brief description of its purpose. This is followed by the function call sequence, definitions of its arguments, and more detailed explanation of the function purpose. The following sections are included in function description:

Arguments	Describes all the function arguments.
Discussion	Defines the function and describes the operation performed by the function. This section also includes descriptive equations.

Audience for This Manual

The manual is intended for all users of OpenCV: researchers, commercial software developers, government and camera vendors.

On-line Version

This manual is available in an electronic format (Portable Document Format, or PDF). To obtain a hard copy of the manual, print the file using the printing capability of Adobe* Acrobat*, the tool used for the on-line presentation of the document.

Related Publications

For more information about signal processing concepts and algorithms, refer to the books and materials listed in the Bibliography.

Notational Conventions

In this manual, notational conventions include:

- Fonts used for distinction between the text and the code
- Naming conventions
- Function name conventions

Font Conventions

The following font conventions are used:

`THIS TYPE STYLE`	Used in the text for OpenCV constant identifiers; for example, `CV_SEQ_KIND_GRAPH`.
`This type style`	Mixed with the uppercase in structure names as in `CvContourTree`; also used in function names, code examples and call statements; for example, `int cvFindContours()`.
`This type style`	Variables in arguments discussion; for example, *`value`*, *`src`*.

Naming Conventions

The OpenCV software uses the following naming conventions for different items:

- Constant identifiers are in uppercase; for example, `CV_SEQ_KIND_GRAPH`.
- All names of the functions used for image processing have the `cv` prefix. In code examples, you can distinguish the OpenCV interface functions from the application functions by this prefix.
- All OpenCV external functions' names start with `cv` prefix, all structures' names start with `Cv` prefix.

NOTE. *In this manual, the* `cv` *prefix in function names is always used in the code examples. In the text, this prefix is usually omitted when referring to the function group.*

Each new part of a function name starts with an uppercase character, without underscore; for example, `cvContourTree`.

Function Name Conventions

The function names in the OpenCV library typically begin with `cv` prefix and have the following general format:

cv `<action> <target> <mod> ()`

where

action — indicates the core functionality, for example, `-Set-`, `-Create-`, `-Convert-`.

target — indicates the area where the image processing is being enacted, for example, `-FindContours` or `-ApproxPoly`.

In a number of cases the target consists of two or more words, for example, `-MatchContourTree`. Some function names consist of an *action* or *target* only; for example, the functions `cvUnDistort` or `cvAcc` respectively.

mod — an optional field; indicates a modification to the core functionality of a function. For example, in the function name `cvFindExtrinsicCameraParams_64d`, `_64d` indicates that this particular function works with double precision numbers.

Motion Analysis and Object Tracking 2

Background Subtraction

This section describes basic functions that enable building statistical model of background for its further subtraction.

In this chapter the term "background" stands for a set of motionless image pixels, that is, pixels that do not belong to any object, moving in front of the camera. This definition can vary if considered in other techniques of object extraction. For example, if a depth map of the scene is obtained, background can be determined as parts of scene that are located far enough from the camera.

The simplest background model assumes that every background pixel brightness varies independently, according to normal distribution.The background characteristics can be calculated by accumulating several dozens of frames, as well as their squares. That means finding a sum of pixel values in the location $S_{(x,y)}$ and a sum of squares of the values $Sq_{(x,y)}$ for every pixel location.

Then mean is calculated as $m_{(x,y)} = \frac{S_{(x,y)}}{N}$, where N is the number of the frames collected, and

standard deviation as $\sigma_{(x,y)} = sqrt\left(\frac{Sq_{(x,y)}}{N} - \left(\frac{S_{(x,y)}}{N}\right)^2\right)$.

After that the pixel in a certain pixel location in certain frame is regarded as belonging to a moving object if condition $abs(m_{(x,y)} - p_{(x,y)}) > C\sigma_{(x,y)}$ is met, where C is a certain constant. If C is equal to 3, it is the well-known "three sigmas" rule. To obtain that background model, any objects should be put away from the camera for a few seconds, so that a whole image from the camera represents subsequent background observation.

The above technique can be improved. First, it is reasonable to provide adaptation of background differencing model to changes of lighting conditions and background scenes, e.g., when the camera moves or some object is passing behind the front object.

The simple accumulation in order to calculate mean brightness can be replaced with running average. Also, several techniques can be used to identify moving parts of the scene and exclude them in the course of background information accumulation. The techniques include change detection, e.g., via `cvAbsDiff` with `cvThreshold`, optical flow and, probably, others.

The functions from the section (See Motion Analysis and Object Tracking Reference) are simply the basic functions for background information accumulation and they can not make up a complete background differencing module alone.

Motion Templates

The functions described in Motion Templates Functions section are designed to generate motion template images that can be used to rapidly determine where a motion occurred, how it occurred, and in which direction it occurred. The algorithms are based on papers by Davis and Bobick [Davis97] and Bradski and Davis [Bradsky00]. These functions operate on images that are the output of background subtraction or other image segmentation operations; thus the input and output image types are all grayscale, that is, have a single color channel.

Motion Representation and Normal Optical Flow Method

Motion Representation

Figure 2-1 (left) shows capturing a foreground silhouette of the moving object or person. Obtaining a clear silhouette is achieved through application of some of background subtraction techniques briefly described in the section on Background Subtraction. As the person or object moves, copying the most recent foreground silhouette as the highest values in the motion history image creates a layered history of the resulting motion; typically this highest value is just a floating point timestamp of time elapsing since the application was launched in milliseconds. Figure 2-1 (right)

shows the result that is called the *Motion History Image (MHI)*. A pixel level or a time delta threshold, as appropriate, is set such that pixel values in the *MHI* image that fall below that threshold are set to zero.

Figure 2-1 Motion History Image From Moving Silhouette

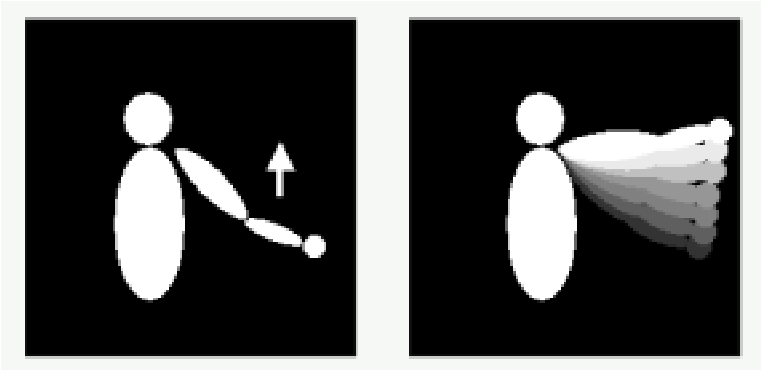

The most recent motion has the highest value, earlier motions have decreasing values subject to a threshold below which the value is set to zero. Different stages of creating and processing motion templates are described below.

A) Updating MHI Images

Generally, floating point images are used because system time differences, that is, time elapsing since the application was launched, are read in milliseconds to be further converted into a floating point number which is the value of the most recent silhouette. Then follows writing this current silhouette over the past silhouettes with subsequent thresholding away pixels that are too old (beyond a maximum `mhiDuration`) to create the MHI.

B) Making Motion Gradient Image

1. Start with the MHI image as shown in Figure 2-2(left).
2. Apply `3x3` Sobel operators `X` and `Y` to the image.

3. If the resulting response at a pixel location (X,Y) is $S_x(x, y)$ to the Sobel operator X and $S_y(x, y)$ to the operator Y, then the orientation of the gradient is calculated as:

   $A(x, y) = arc\tan S_y((x, y)/S_x(x, y))$,

   and the magnitude of the gradient is:

   $M(x, y) = \sqrt{S_x^2(x, y) + S_y^2(x, y)}$.

4. The equations are applied to the image yielding direction or angle of a flow image superimposed over the *MHI* image as shown in Figure 2-2.

Figure 2-2 Direction of Flow Image

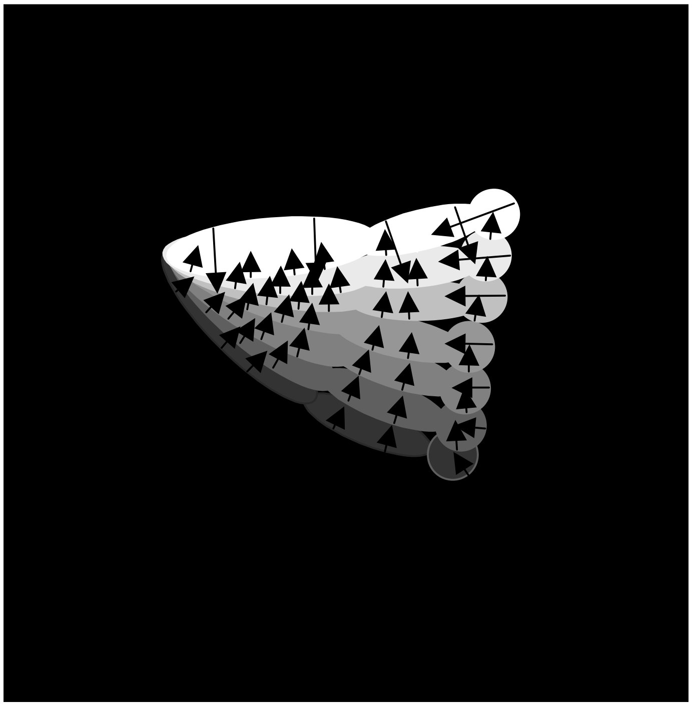

5. The boundary pixels of the *MH* region may give incorrect motion angles and magnitudes, as Figure 2-2 shows. Thresholding away magnitudes that are either too large or too small can be a remedy in this case. Figure 2-3 shows the ultimate results.

Figure 2-3 Resulting Normal Motion Directions

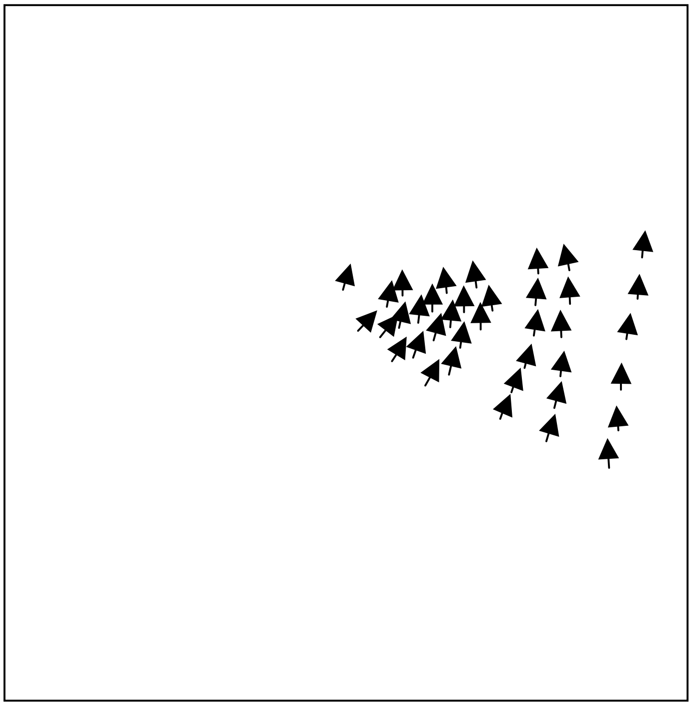

C) Finding Regional Orientation or Normal Optical Flow

Figure 2-4 shows the output of the motion gradient function described in the section above together with the marked direction of motion flow.

Figure 2-4 MHI Image of Kneeling Person

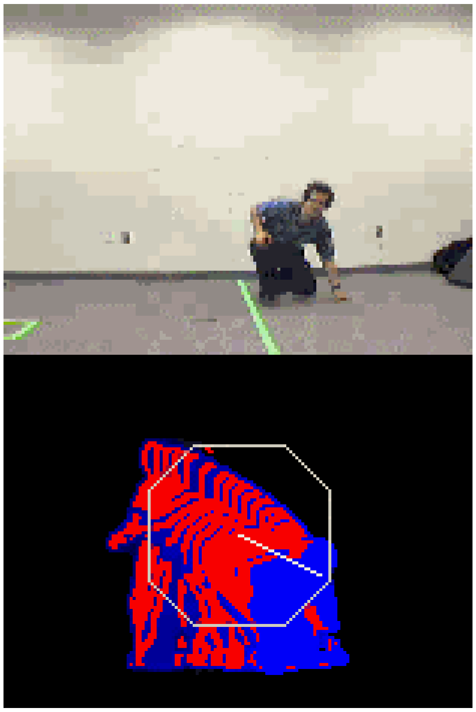

The current silhouette is in bright blue with past motions in dimmer and dimmer blue. Red lines show where valid normal flow gradients were found. The white line shows computed direction of global motion weighted towards the most recent direction of motion.

To determine the most recent, salient global motion:

1. Calculate a histogram of the motions resulting from processing (see Figure 2-3).
2. Find the average orientation of a circular function: angle in degrees.

   a. Find the maximal peak in the orientation histogram.

   b. Find the average of minimum differences from this base angle. The more recent movements are taken with lager weights.

Motion Segmentation

Representing an image as a single moving object often gives a very rough motion picture. So, the goal is to group MHI pixels into several groups, or connected regions, that correspond to parts of the scene that move in different directions. Using then a downward stepping floodfill to label motion regions connected to the current silhouette helps identify areas of motion directly attached to parts of the object of interest.

Once MHI image is constructed, the most recent silhouette acquires the maximal values equal to the most recent timestamp in that image. The image is scanned until any of these values is found, then the silhouette’s contour is traced to find attached areas of motion, and searching for the maximal values continues. The algorithm for creating masks to segment motion region is as follows:

1. Scan the MHI until a pixel of the most recent silhouette is found, use floodfill to mark the region the pixel belongs to (see Figure 2-5 (a)).
2. Walk around the boundary of the current silhouette region looking outside for unmarked motion history steps that are recent enough, that is, within the threshold. When a suitable step is found, mark it with a downward floodfill. If the size of the fill is not big enough, zero out the area (see Figure 2-5 (b)).
3. [Optional]:

— Record locations of minimums within each downfill (see Figure 2-5 (c));

— Perform separate floodfills up from each detected location (see Figure 2-5 (d));

— Use logical AND to combine each upfill with downfill it belonged to.

4. Store the detected segmented motion regions into the mask.
5. Continue the boundary “walk” until the silhouette has been circumnavigated.

6. [Optional] Go to 1 until all current silhouette regions are found.

Figure 2-5 Creating Masks to Segment Motion Region

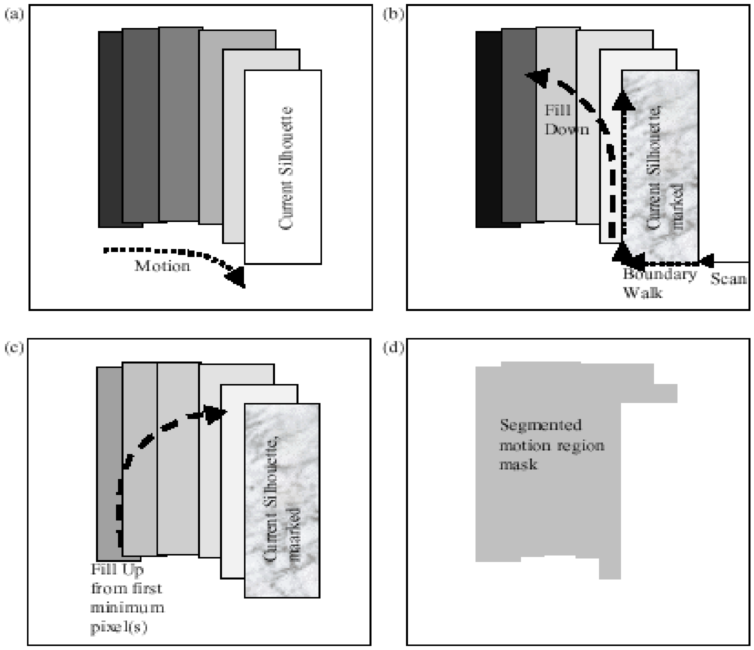

CamShift

This section describes CamShift algorithm realization functions.

CamShift stands for the “Continuously Adaptive Mean-SHIFT” algorithm. Figure 2-6 summarizes this algorithm. For each video frame, the raw image is converted to a color probability distribution image via a color histogram model of the color being tracked, e.g., flesh color in the case of face tracking. The center and size of the color object are found via the CamShift algorithm operating on the color probability image. The current size and location of the tracked object are reported and used to set the size and location of the search window in the next video image. The process is then repeated for continuous tracking. The algorithm is a generalization of the Mean Shift algorithm, highlighted in gray in Figure 2-6.

Figure 2-6 Block Diagram of CamShift Algorithm

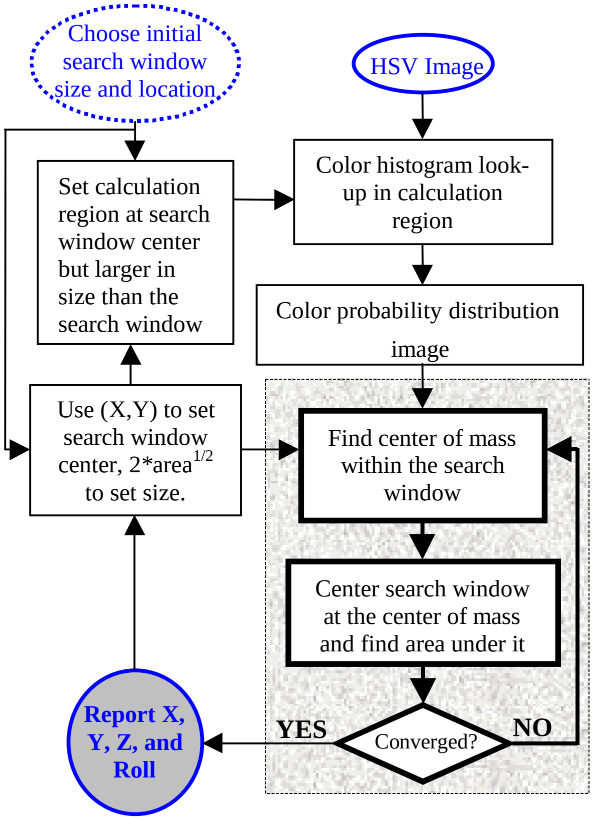

CamShift operates on a *2D* color probability distribution image produced from histogram back-projection (see the section on Histogram in Image Analysis). The core part of the CamShift algorithm is the Mean Shift algorithm.

The Mean Shift part of the algorithm (gray area in Figure 2-6) is as follows:

1. Choose the search window size.
2. Choose the initial location of the search window.

3. Compute the mean location in the search window.
4. Center the search window at the mean location computed in Step 3.
5. Repeat Steps 3 and 4 until the search window center converges, i.e., until it has moved for a distance less than the preset threshold.

Mass Center Calculation for *2D* Probability Distribution

For discrete *2D* image probability distributions, the mean location (the centroid) within the search window, that is computed at step 3 above, is found as follows:

Find the zero[th] moment

$$M_{00} = \sum_x \sum_y I(x, y).$$

Find the first moment for x and y

$$M_{10} = \sum_x \sum_y xI(x, y); \; M_{01} = \sum_x \sum_y yI(x, y).$$

Mean search window location (the centroid) then is found as

$$x_c = \frac{M_{10}}{M_{00}}; \; y_c = \frac{M_{01}}{M_{00}},$$

where $I(x,y)$ is the pixel (probability) value in the position (x,y) in the image, and x and y range over the search window.

Unlike the Mean Shift algorithm, which is designed for static distributions, CamShift is designed for dynamically changing distributions. These occur when objects in video sequences are being tracked and the object moves so that the size and location of the probability distribution changes in time. The CamShift algorithm adjusts the search window size in the course of its operation. Initial window size can be set at any reasonable value. For discrete distributions (digital data), the minimum window length or width is three. Instead of a set, or externally adapted window size, CamShift relies on the zero[th] moment information, extracted as part of the internal workings of the algorithm, to continuously adapt its window size within or over each video frame.

CamShift Algorithm

1. Set the calculation region of the probability distribution to the whole image.
2. Choose the initial location of the *2D* mean shift search window.
3. Calculate the color probability distribution in the *2D* region centered at the search window location in an ROI slightly larger than the mean shift window size.
4. Run Mean Shift algorithm to find the search window center. Store the zeroth moment (area or size) and center location.
5. For the next video frame, center the search window at the mean location stored in Step 4 and set the window size to a function of the zeroth moment found there. Go to Step 3.

Figure 2-7 shows CamShift finding the face center on a *1D* slice through a face and hand flesh hue distribution. Figure 2-8 shows the next frame when the face and hand flesh hue distribution has moved, and convergence is reached in two iterations.

Figure 2-7 Cross Section of Flesh Hue Distribution

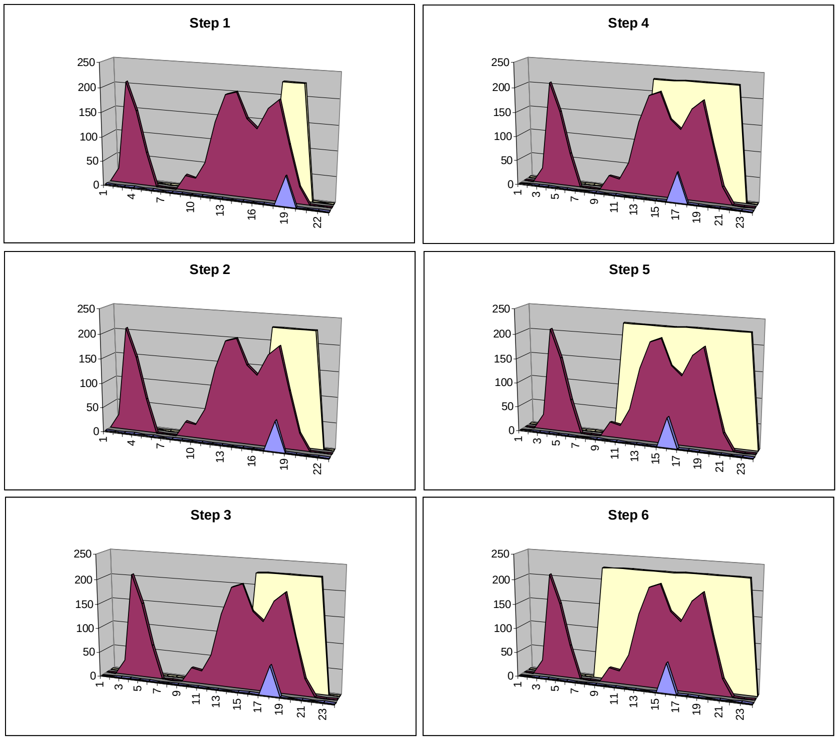

Rectangular CamShift window is shown behind the hue distribution, while triangle in front marks the window center. CamShift is shown iterating to convergence down the left then right columns.

Figure 2-8 Flesh Hue Distribution (Next Frame)

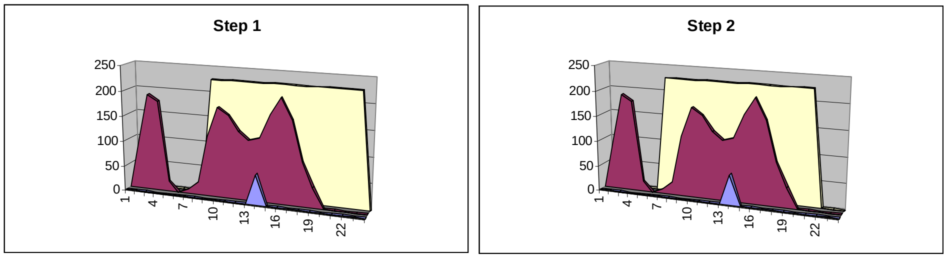

Starting from the converged search location in Figure 2-7 bottom right, CamShift converges on new center of distribution in two iterations.

Calculation of *2D* Orientation

The *2D* orientation of the probability distribution is also easy to obtain by using the second moments in the course of CamShift operation, where the point (x,y) ranges over the search window, and $I(x,y)$ is the pixel (probability) value at the point (x,y).

Second moments are

$$M_{20} = \sum_x \sum_y x^2 I(x,y),\ M_{02} = \sum_x \sum_y x^2 I(x,y).$$

Then the object orientation, or direction of the major axis, is

$$\theta = \frac{arc\tan\left(\frac{2\left(\frac{M_{11}}{M_{00}} - x_c y_c\right)}{\left(\frac{M_{20}}{M_{00}} - x_c^2\right) - \left(\frac{M_{02}}{M_{00}} - y_c^2\right)}\right)}{2}.$$

The first two eigenvalues, that is, length and width, of the probability distribution of the blob found by CamShift may be calculated in closed form as follows:

Let

$a = \frac{M_{20}}{M_{00}} - x_c^2$, $b = 2\left(\frac{M_{11}}{M_{00}} - x_c y_c\right)$, and $c = \frac{M_{02}}{M_{00}} - y_c^2$.

Then length l and width w from the distribution centroid are

$$l = \sqrt{\frac{(a + c) + \sqrt{b^2 + (a - c)^2}}{2}},$$

$$w = \sqrt{\frac{(a + c) - \sqrt{b^2 + (a - c)^2}}{2}}.$$

When used in face tracking, the above equations give head roll, length, and width as marked in the source video image in Figure 2-9.

Figure 2-9 Orientation of Flesh Probability Distribution

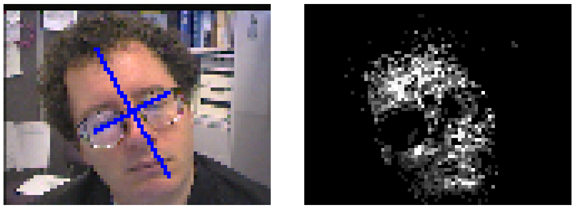

Active Contours

This section describes a function for working with active contours, also called snakes.

The snake was presented in [Kass88] as an energy-minimizing parametric closed curve guided by external forces. Energy function associated with the snake is

$E = E_{int} + E_{ext}$,

where E_{int} is the internal energy formed by the snake configuration, E_{ext} is the external energy formed by external forces affecting the snake. The aim of the snake is to find a location that minimizes energy.

Let $p_1, \ldots, p_n$ be a discrete representation of a snake, that is, a sequence of points on an image plane.

In OpenCV the internal energy function is the sum of the contour continuity energy and the contour curvature energy, as follows:

$E_{int} = E_{cont} + E_{curv}$, where

E_{cont} is the contour continuity energy. This energy is $E_{cont} = |\bar{d} - \|p_i - p_{i-1}\||$, where $\bar{d}$ is the average distance between all pairs $(p_i - p_{i-1})$. Minimizing E_{cont} over all the snake points $p_1, \ldots, p_n$, causes the snake points become more equidistant.

E_{curv} is the contour curvature energy. The smoother the contour is, the less is the curvature energy. $E_{curv} = \|p_{i-1} - 2p_i + p_{i+1}\|^2$.

In [Kass88] external energy was represented as $E_{ext} = E_{img} + E_{con}$, where

E_{img} – image energy and E_{con} - energy of additional constraints.

Two variants of image energy are proposed:

1. $E_{img} = -I$, where I is the image intensity. In this case the snake is attracted to the bright lines of the image.
2. $E_{img} = -\|grad(I)\|$. The snake is attracted to the image edges.

A variant of external constraint is described in [Kass88]. Imagine the snake points connected by springs with certain image points. Then the spring force $k(x - x_0)$ produces the energy $\frac{kx^2}{2}$. This force pulls the snake points to fixed positions, which can be useful when

snake points need to be fixed. OpenCV does not support this option now.

Summary energy at every point can be written as

$$E_i = \alpha_i E_{cont,i} + \beta_i E_{curv,i} + \gamma_i E_{img,i}, \quad (2.1)$$

where α, β, γ are the weights of every kind of energy. The full snake energy is the sum of E_i over all the points.

The meanings of α, β, γ are as follows:

α is responsible for contour continuity, that is, a big α makes snake points more evenly spaced.

β is responsible for snake corners, that is, a big β for a certain point makes the angle between snake edges more obtuse.

γ is responsible for making the snake point more sensitive to the image energy, rather than to continuity or curvature.

Only relative values of α, β, γ in the snake point are relevant.

The following way of working with snakes is proposed:

- create a snake with initial configuration;
- define weights α, β, γ at every point;
- allow the snake to minimize its energy;
- evaluate the snake position. If required, adjust α, β, γ, and, possibly, image data, and repeat the previous step.

There are three well-known algorithms for minimizing snake energy. In [Kass88] the minimization is based on variational calculus. In [Yuille89] dynamic programming is used. The greedy algorithm is proposed in [Williams92].

The latter algorithm is the most efficient and yields quite good results. The scheme of this algorithm for each snake point is as follows:

1. Use Equation (3.1) to compute E for every location from point neighborhood. Before computing E, each energy term $E_{cont}, E_{curv}, E_{img}$ must be normalized using formula $E_{normalized} = (E_{img} - min)/(max - min)$, where max and min are maximal and minimal energy in scanned neighborhood.
2. Choose location with minimum energy.
3. Move snakes point to this location.
4. Repeat all the steps until convergence is reached.

Criteria of convergence are as follows:

- maximum number of iterations is achieved;
- number of points, moved at last iteration, is less than given threshold.

In [Williams92] the authors proposed a way, called high-level feedback, to adjust b coefficient for corner estimation during minimization process. Although this feature is not available in the implementation, the user may build it, if needed.

Optical Flow

This section describes several functions for calculating optical flow between two images.

Most papers devoted to motion estimation use the term optical flow. Optical flow is defined as an apparent motion of image brightness. Let $I(x,y,t)$ be the image brightness that changes in time to provide an image sequence. Two main assumptions can be made:

1. Brightness $I(x,y,t)$ smoothly depends on coordinates x, y in greater part of the image.
2. Brightness of every point of a moving or static object does not change in time.

Let some object in the image, or some point of an object, move and after time dt the object displacement is `(dx, dy)`. Using Taylor series for brightness $I(x,y,t)$ gives the following:

$$I(x+dx, y+dy, t+dt) = I(x, y, t) + \frac{\partial I}{\partial x}dx + \frac{\partial I}{\partial y}dy + \frac{\partial I}{\partial t}dt + \ldots, \qquad (2.2)$$

where "…" are higher order terms.

Then, according to Assumption 2:

$$I(x+dx, y+dy, t+dt) = I(x, y, t), \qquad (2.3)$$

and

$$\frac{\partial I}{\partial x}dx + \frac{\partial I}{\partial y}dy + \frac{\partial I}{\partial t}dt + \ldots = 0. \qquad (2.4)$$

Dividing (18.3) by dt and defining

$$\frac{dx}{dt} = u, \frac{dy}{dt} = v \qquad (2.5)$$

gives an equation

$$-\frac{\partial I}{\partial t} = \frac{\partial I}{\partial x}u + \frac{\partial I}{\partial y}v, \qquad (2.6)$$

usually called *optical flow constraint equation*, where u and v are components of optical flow field in x and y coordinates respectively. Since Equation (2.6) has more than one solution, more constraints are required.

Some variants of further steps may be chosen. Below follows a brief overview of the options available.

Lucas & Kanade Technique

Using the optical flow equation for a group of adjacent pixels and assuming that all of them have the same velocity, the optical flow computation task is reduced to solving a linear system.

In a non-singular system for two pixels there exists a single solution of the system. However, combining equations for more than two pixels is more effective. In this case the approximate solution is found using the least square method. The equations are usually weighted. Here the following 2x2 linear system is used:

$$\sum_{x,y} W(x,y) I_x I_y u + \sum_{x,y} W(x,y) I_y^2 v = -\sum_{x,y} W(x,y) I_y I_t ,$$

$$\sum_{x,y} W(x,y) I_x^2 u + \sum_{x,y} W(x,y) I_x I_y v = -\sum_{x,y} W(x,y) I_x I_t ,$$

where $W(x,y)$ is the Gaussian window. The Gaussian window may be represented as a composition of two separable kernels with binomial coefficients. Iterating through the system can yield even better results. It means that the retrieved offset is used to determine a new window in the second image from which the window in the first image is subtracted, while I_t is calculated.

Horn & Schunck Technique

Horn and Schunck propose a technique that assumes the smoothness of the estimated optical flow field [Horn81]. This constraint can be formulated as

$$S = \iint_{image} \left[\left(\frac{\partial u}{\partial x}\right)^2 + \left(\frac{\partial u}{\partial y}\right)^2 + \left(\frac{\partial v}{\partial x}\right)^2 + \left(\frac{\partial v}{\partial y}\right)^2 \right] (dx)dy \quad . \tag{2.7}$$

This optical flow solution can deviate from the optical flow constraint. To express this deviation the following integral can be used:

$$C = \iint_{image} \left(\frac{\partial I}{\partial x} u + \frac{\partial I}{\partial y} v + \frac{\partial I}{\partial t} \right)^2 dxdy \, . \tag{2.8}$$

The value $S + \lambda C$, where λ is a parameter, called Lagrangian multiplier, is to be minimized. Typically, a smaller λ must be taken for a noisy image and a larger one for a quite accurate image.

To minimize $S + \lambda C$, a system of two second-order differential equations for the whole image must be solved:

$$\frac{\partial^2 u}{\partial x^2} + \frac{\partial^2 u}{\partial y^2} = \lambda\left(\frac{\partial I}{\partial x}u + \frac{\partial I}{\partial y}v + \frac{\partial I}{\partial t}\right)\frac{\partial I}{\partial x},$$
$$\frac{\partial^2 v}{\partial x^2} + \frac{\partial^2 v}{\partial y^2} = \lambda\left(\frac{\partial I}{\partial x}u + \frac{\partial I}{\partial y}v + \frac{\partial I}{\partial t}\right)\frac{\partial I}{\partial x}. \qquad (2.9)$$

Iterative method could be applied for the purpose when a number of iterations are made for each pixel. This technique for two consecutive images seems to be computationally expensive because of iterations, but for a long sequence of images only an iteration for two images must be done, if the result of the previous iteration is chosen as initial approximation.

Block Matching

This technique does not use an optical flow equation directly. Consider an image divided into small blocks that can overlap. Then for every block in the first image the algorithm tries to find a block of the same size in the second image that is most similar to the block in the first image. The function searches in the neighborhood of some given point in the second image. So all the points in the block are assumed to move by the same offset that is found, just like in Lucas & Kanade method. Different metrics can be used to measure similarity or difference between blocks - cross correlation, squared difference, etc.

Estimators

This section describes group of functions for estimating stochastic models state.

State estimation programs implement a model and an estimator. A model is analogous to a data structure representing relevant information about the visual scene. An estimator is analogous to the software engine that manipulates this data structure to compute beliefs about the world. The OpenCV routines provide two estimators: standard Kalman and condensation.

Models

Many computer vision applications involve repeated estimating, that is, tracking, of the system quantities that change over time. These dynamic quantities are called the system *state*. The system in question can be anything that happens to be of interest to a particular vision task.

To estimate the state of a system, reasonably accurate knowledge of the system *model* and *parameters* may be assumed. Parameters are the quantities that describe the model configuration but change at a rate much slower than the state. Parameters are often assumed known and static.

In OpenCV a state is represented with a vector. In addition to this output of the state estimation routines, another vector introduced is a vector of *measurements* that are input to the routines from the sensor data.

To represent the model, two things are to be specified:

- Estimated dynamics of the state change from one moment of time to the next
- Method of obtaining a measurement vector z_t from the state.

Estimators

Most estimators have the same general form with repeated propagation and update phases that modify the state's uncertainty as illustrated in Figure 2-10.

Figure 2-10 Ongoing Discrete Kalman Filter Cycle

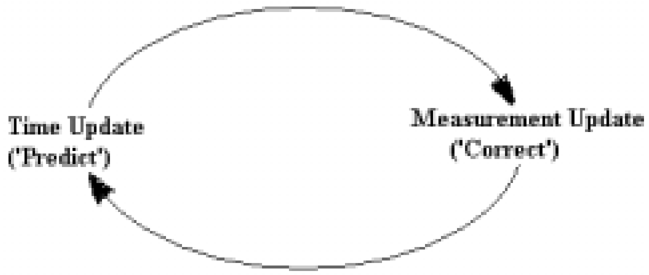

The time update projects the current state estimate ahead in time. The measurement update adjusts the projected estimate using an actual measurement at that time.

An estimator should be preferably unbiased when the probability density of estimate errors has an expected value of 0. There exists an optimal propagation and update formulation that is the best, linear, unbiased estimator (BLUE) for any given model of the form. This formulation is known as the discrete Kalman estimator, whose standard form is implemented in OpenCV.

Kalman Filtering

The following explanation was taken from University of North Carolina at Chapel Hill technical report TR 95-041 by Greg Welch and Gary Bishop [Welsh95].

The Kalman filter addresses the general problem of trying to estimate the state x of a discrete-time process that is governed by the linear stochastic difference equation

$$x_{k+1} = Ax_k + w_k \tag{2.10}$$

with a measurement z, that is

$$z_k = Hx_k + v_k \tag{2.11}$$

The random variables w_k and v_k respectively represent the process and measurement noise. They are assumed to be independent of each other, white, and with normal probability distributions

$$p(w) = N(0, Q), \tag{2.12}$$

$$p(w) = N(0, R). \tag{2.13}$$

The $N \times N$ matrix A in the difference equation (2.10) relates the state at time step k to the state at step $k+1$, in the absence of process noise. The $M \times N$ matrix H in the measurement equation (2.11) relates the state to the measurement z_k.

If $x_{\bar{k}}$ denotes a priori state estimate at step k provided the process prior to step k is known, and x_k denotes a posteriori state estimate at step k provided measurement z_k is known, then a priori and a posteriori estimate errors can be defined as $\begin{array}{l} e_{\bar{k}} = x_k - x_{\bar{k}} \\ e_k = x_k - x_k \end{array}$. The a priori estimate error covariance is then $P_{\bar{k}} = E[e_{\bar{k}} e_k^{-T}]$ and the a posteriori estimate error covariance is $P_k = E[e_k e_k^T]$.

The Kalman filter estimates the process by using a form of feedback control: the filter estimates the process state at some time and then obtains feedback in the form of noisy measurements. As such, the equations for the Kalman filter fall into two groups: time

update equations and measurement update equations. The time update equations are responsible for projecting forward in time the current state and error covariance estimates to obtain the a priori estimates for the next time step. The measurement update equations are responsible for the feedback, that is, for incorporating a new measurement into the a priori estimate to obtain an improved a posteriori estimate. The time update equations can also be viewed as predictor equations, while the measurement update equations can be thought of as corrector equations. Indeed, the final estimation algorithm resembles that of a predictor-corrector algorithm for solving numerical problems as shown in Figure 2-10. The specific equations for the time and measurement updates are presented below.

Time Update Equations

$$x_{\bar{k}+1} = A_k x_k \text{ ,}$$

$$P_{\bar{k}+1} = A_k P_k A_k^T + Q_k \text{ .}$$

Measurement Update Equations:

$$K_k = P_{\bar{k}} H_k^T (H_k P_{\bar{k}} H_k^T + R_k)^{-1} \text{ ,}$$

$$x_k = x_{\bar{k}} + K_k (z_k - H_k x_{\bar{k}}) \text{ ,}$$

$$P_k = (I - K_k H_k) P_{\bar{k}} \text{ ,}$$

where K is the so-called Kalman gain matrix and I is the identity operator. See `CvKalman` in Motion Analysis and Object Tracking Reference.

ConDensation Algorithm

This section describes the ConDensation (conditional density propagation) algorithm, based on factored sampling. The main idea of the algorithm is using the set of randomly generated samples for probability density approximation. For simplicity, general principles of ConDensation algorithm are described below for linear stochastic dynamical system:

$$x_{k+1} = Ax_k + w_k \tag{2.14}$$

with a measurement Z.

To start the algorithm, a set of samples X^n must be generated. The samples are randomly generated vectors of states. The function ConDensInitSampleSet does it in OpenCV implementation.

During the first phase of the condensation algorithm every sample in the set is updated according to Equation (3.14).

Further, when the vector of measurement Z is obtained, the algorithm estimates conditional probability densities of every sample $P(X^n|Z)$. The OpenCV implementation of the ConDensation algorithm enables the user to define various probability density functions. There is no such special function in the library. After the probabilities are calculated, the user may evaluate, for example, moments of tracked process at the current time step.

If dynamics or measurement of the stochastic system is non-linear, the user may update the dynamics (A) or measurement (H) matrices, using their Taylor series at each time step. See CvConDensation in Motion Analysis and Object Tracking Reference.

Image Analysis

3

Contour Retrieving

This section describes contour retrieving functions.

Below follow descriptions of:

- several basic functions that retrieve contours from the binary image and store them in the chain format;
- functions for polygonal approximation of the chains.

Basic Definitions

Most of the existing *vectoring* algorithms, that is, algorithms that find contours on the raster images, deal with binary images. A binary image contains only *0-pixels*, that is, pixels with the value 0, and *1-pixels*, that is, pixels with the value 1. The set of *connected* 0- or 1-pixels makes the *0-(1-) component*. There are two common sorts of connectivity, the *4-connectivity* and *8-connectivity.* Two pixels with coordinates (*x*', *y*') and (*x*", *y*") are called *4-connected* if, and only if, $|x' - x''| + |y' - y''| = 1$ and *8-connected* if, and only if, $max(|x' - x''|, |y' - y''|) = 1$. Figure 1-1 shows these relations.:

Figure 3-1 Pixels Connectivity Patterns

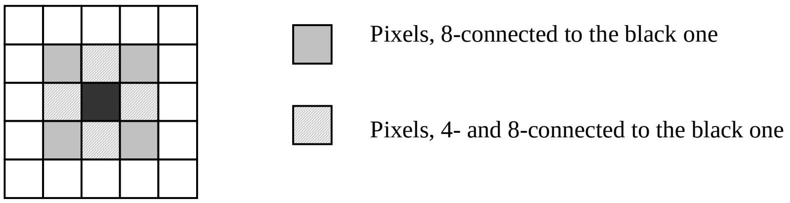

Using this relationship, the image is broken into several non-overlapped 1-(0-) 4-connected (8-connected) components. Each set consists of pixels with equal values, that is, all pixels are either equal to 1 or 0, and any pair of pixels from the set can be linked by a sequence of 4- or 8-connected pixels. In other words, a 4-(8-) path exists between any two points of the set. The components shown in Figure 1-2 may have interrelations.

Figure 3-2 Hierarchical Connected Components

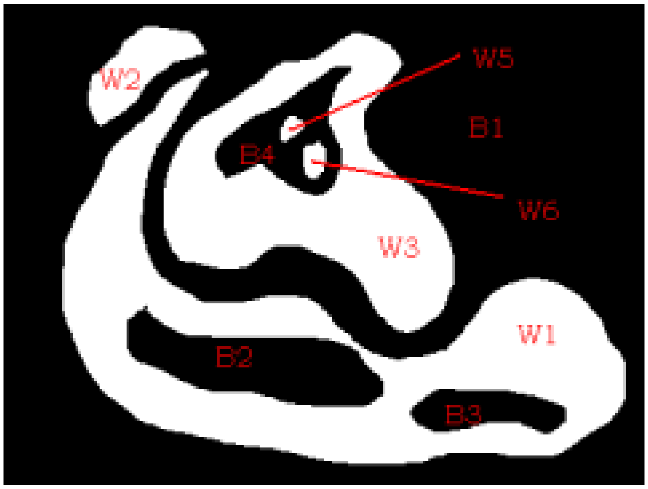

1-components *W1*, *W2*, and *W3* are inside the *frame* (0-component B1), that is, *directly* surrounded by *B1*.

0-components *B2* and *B3* are inside *W1*.

1-components *W5* and *W6* are inside *B4*, that is inside *W3*, so these 1-components are inside *W3 indirectly*. However, neither *W5* nor *W6* enclose one another, which means they are on the same level.

In order to avoid a topological contradiction, 0-pixels must be regarded as 8-(4-) connected pixels in case 1-pixels are dealt with as 4-(8-) connected. Throughout this document 8-connectivity is assumed to be used with 1-pixels and 4-connectivity with 0-pixels.

Since 0-components are complementary to 1-components, and separate 1-components are either nested to each other or their internals do not intersect, the library considers 1-components only and only their topological structure is studied, 0-pixels making up the background. A 0-component directly surrounded by a 1-component is called the *hole* of the 1-component. The *border point* of a 1-component could be any pixel that belongs to the component and has a 4-connected 0-pixel. A connected set of border points is called the *border.*

Each 1-component has a single *outer border* that separates it from the surrounding 0-component and zero or more *hole borders* that separate the 1-component from the 0-components it surrounds. It is obvious that the outer border and hole borders give a full description of the component. Therefore all the borders, also referred to as *contours*, of all components stored with information about the hierarchy make up a compressed representation of the source binary image. See Reference for description of the functions `FindContours`, `StartFindContours`, and `FindNextContour` that build such a contour representation of binary images.

Contour Representation

The library uses two methods to represent contours. The first method is called the Freeman method or the chain code (Figure 1-3). For any pixel all its neighbors with numbers from 0 to 7 can be enumerated:

Figure 3-3 Contour Representation in Freeman Method

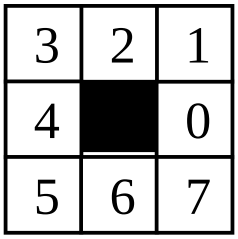

The 0-neighbor denotes the pixel on the right side, etc. As a sequence of 8-connected points, the border can be stored as the coordinates of the initial point, followed by codes (from 0 to 7) that specify the location of the next point relative to the current one (see Figure 1-4).

Figure 3-4 Freeman Coding of Connected Components

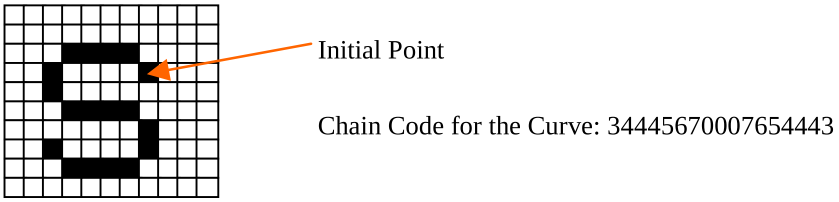

The chain code is a compact representation of digital curves and an output format of the contour retrieving algorithms described below.

Polygonal representation is a different option in which the curve is coded as a sequence of points, vertices of a polyline. This alternative is often a better choice for manipulating and analyzing contours over the chain codes; however, this representation is rather hard to get directly without much redundancy. Instead, algorithms that approximate the chain codes with polylines could be used.

Contour Retrieving Algorithm

Four variations of algorithms described in [Suzuki85] are used in the library to retrieve borders.

1. The first algorithm finds only the extreme outer contours in the image and returns them linked to the list. Figure 1-2 shows these external boundaries of *W1*, *W2*, and *W3* domains.
2. The second algorithm returns all contours linked to the list. Figure 1-2 shows the total of 8 such contours.

3. The third algorithm finds all connected components by building a two-level hierarchical structure: on the top are the external boundaries of 1-domains and every external boundary contains a link to the list of holes of the corresponding component. The third algorithm returns all the connected components as a two-level hierarchical structure: on the top are the external boundaries of 1-domains and every external boundary contour header contains a link to the list of holes in the corresponding component. The list can be accessed via `v_next` field of the external contour header. Figure 1-2 shows that *W2*, *W5*, and *W6* domains have no holes; consequently, their boundary contour headers refer to empty lists of hole contours. *W1* domain has two holes - the external boundary contour of *W1* refers to a list of two hole contours. Finally, *W3* external boundary contour refers to a list of the single hole contour.
4. The fourth algorithm returns the complete hierarchical tree where all the contours contain a list of contours surrounded by the contour directly, that is, the hole contour of *W3* domain has two children: external boundary contours of *W5* and *W6* domains.

All algorithms make a single pass through the image; there are, however, rare instances when some contours need to be scanned more than once. The algorithms do line-by-line scanning.

Whenever an algorithm finds a point that belongs to a new border the border following procedure is applied to retrieve and store the border in the chain format. During the border following procedure the algorithms mark the visited pixels with special positive or negative values. If the right neighbor of the considered border point is a 0-pixel and, at the same time, the 0-pixel is located in the right hand part of the border, the border point is marked with a negative value. Otherwise, the point is marked with the same magnitude but of positive value, if the point has not been visited yet. This can be easily determined since the border can cross itself or tangent other borders. The first and second algorithms mark all the contours with the same value and the third and fourth algorithms try to use a unique ID for each contour, which can be used to detect the parent of any newly met border.

Features

Fixed Filters

This section describes various fixed filters, primarily derivative operators.

Sobel Derivatives

Figure 1-5 shows first x derivative Sobel operator. The grayed bottom left number indicates the origin in a “p-q” coordinate system. The operator can be expressed as a polynomial and decomposed into convolution primitives.

Figure 3-5 First x Derivative Sobel Operator

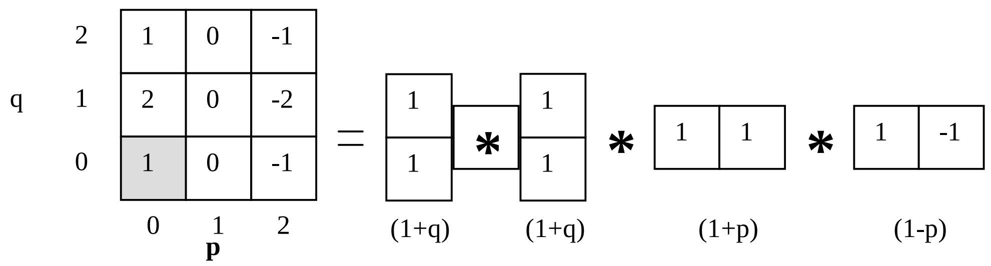

For example, first x derivative Sobel operator may be expressed as a polynomial $1 + 2q + q^2 - p^2 - 2p^2q - p^2q^2 = (1+q)^2(1-p^2) = (1+q)(1+q)(1+p)(1-p)$ and decomposed into convolution primitives as shown in Figure 1-5.

This may be used to express a hierarchy of first x and y derivative Sobel operators as follows:

$$\frac{\partial}{\partial x} \Rightarrow (1+p)^{n-1}(1+q)^n(1-p) \qquad (3.1)$$

$$\frac{\partial}{\partial x} \Rightarrow (1+p)^{n}(1+q)^{n-1}(1-q) \qquad (3.2)$$

for $n > 0$.

Figure 1-6 shows the Sobel first derivative filters of equations (3.1) and (3.2) for n = `2`, `4`. The Sobel filter may be decomposed into simple “add-subtract” convolution primitives.

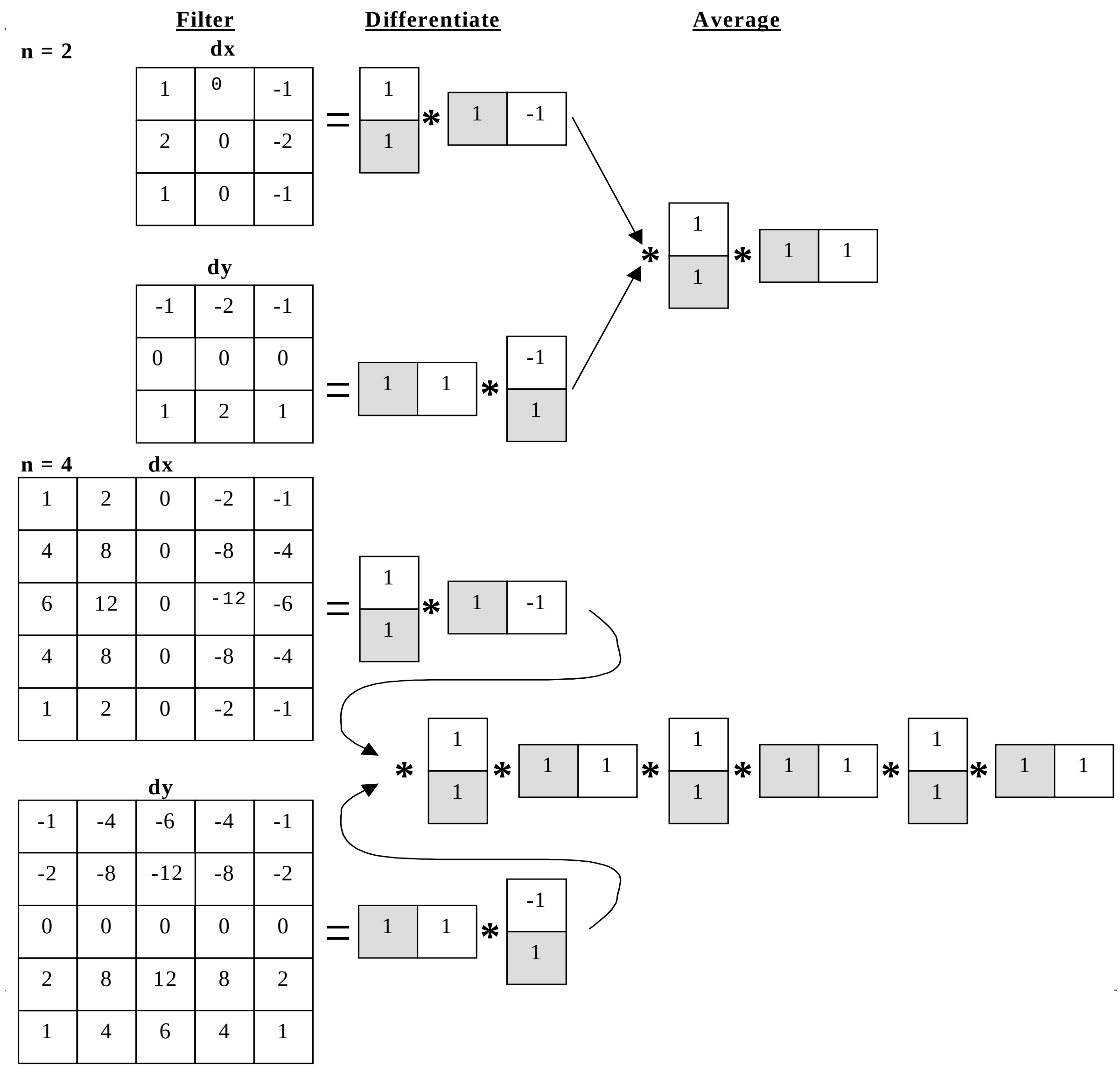

Second derivative Sobel operators can be expressed in polynomial decomposition similar to equations (3.1) and (3.2). The second derivative equations are:

$$\frac{\partial^2}{\partial x^2} \Rightarrow (1+p)^{n-2}(1+q)^n(1-p)^2, \tag{3.3}$$

$$\frac{\partial^2}{\partial y^2} \Rightarrow (1+p)^{n-1}(1+q)^{n-2}(1-q)^2, \tag{3.4}$$

$$\frac{\partial^2}{\partial x \partial y} \Rightarrow (1+p)^{n-1}(1+q)^{n-1}(1-p)(1-q) \tag{3.5}$$

for $n = 2, 3, \ldots$.

Figure 1-7 shows the filters that result for $n = 2$ and 4. Just as shown in Figure 1-6, these filters can be decomposed into simple “add-subtract” separable convolution operators as indicated by their polynomial form in the equations.

Figure 3-7 Sobel Operator Second Order Derivators for n = 2 and n = 4

The polynomial decomposition is shown above each operator.

$\delta^2/\delta x^2 = (1+q)^2(1-p)^2$

1	-2	1
2	-4	2
1	-2	1

$\delta^2/\delta y^2 = (1+p)^2(1-q)^2$

1	2	1
-2	-4	-2
1	2	1

$\delta^2/\delta x\delta y = (1+q)(1+p)(1-q)(1-p)$

-1	0	1
0	0	0
1	0	-1

$\delta^2/\delta x^2 = (1+p)^2(1+q)^4(1-p)^2$

1	0	-2	0	1
4	0	-4	0	4
6	0	-12	0	6
4	0	-8	0	4
1	0	-2	0	1

$\delta^2/\delta y^2 = (1+q)^2(1+p)^4(1-q)^2$

1	4	6	4	1
0	0	0	0	0
-2	-8	-12	-8	-2
0	0	0	0	0
1	4	6	4	1

$\delta^2/\delta x\delta y = (1+p)^3(1+q)^3(1-p)(1-q)$

-1	-2	0	2	1
-2	-4	0	4	2
0	0	0	0	0
2	4	0	-4	-2
1	2	0	-2	-1

Third derivative Sobel operators can also be expressed in the polynomial decomposition form:

$$\frac{\partial^3}{\partial x^3} \Rightarrow (1+p)^{n-3}(1+q)^n(1-p)^3, \qquad (3.6)$$

$$\frac{\partial^3}{\partial y^3} \Rightarrow (1+p)^n(1+q)^{n-3}(1-q)^3, \qquad (3.7)$$

$$\frac{\partial^3}{\partial x^2 \partial y} \Rightarrow (1-p)^2(1+p)^{n-2}(1+q)^{n-1}(1-q), \qquad (3.8)$$

$$\frac{\partial^3}{\partial x \partial y^2} \Rightarrow (1-p)(1+p)^{n-1}(1+q)^{n-2}(1-q)^2 \qquad (3.9)$$

for $n = 3, 4, \ldots$. The third derivative filter needs to be applied only for the cases $n = 4$ and general.

Optimal Filter Kernels with Floating Point Coefficients

First Derivatives

Table 1-1 gives coefficients for five increasingly accurate *x* derivative filters, the *y* filter derivative coefficients are just column vector versions of the *x* derivative filters.

Table 3-1 Coefficients for Accurate First Derivative Filters

Anchor		DX Mask Coefficients				
0	0.74038	-0.12019				
0	0.833812	-0.229945	0.0420264			
0	0.88464	-0.298974	0.0949175	-0.0178608		
0	0.914685	-0.346228	0.138704	-0.0453905	0.0086445	
0	0.934465	-0.378736	0.173894	-0.0727275	0.0239629	-0.00459622

Five increasingly accurate separable x derivative filter coefficients. The table gives half coefficients only. The full table can be obtained by mirroring across the central anchor coefficient. The greater the number of coefficients used, the less distortion from the ideal derivative filter.

Second Derivatives

Table 1-2 gives coefficients for five increasingly accurate *x* second derivative filters. The *y* second derivative filter coefficients are just column vector versions of the *x* second derivative filters.

Table 3-2 Coefficients for Accurate Second Derivative Filters

Anchor	DX Mask Coefficients				
-2.20914	1.10457				
-2.71081	1.48229	-0.126882			
-2.92373	1.65895	-0.224751	0.0276655		
-3.03578	1.75838	-0.291985	0.0597665	-0.00827	
-3.10308	1.81996	-0.338852	0.088077	-0.0206659	0.00301915

The table gives half coefficients only. The full table can be obtained by mirroring across the central anchor coefficient. The greater the number of coefficients used, the less distortion from the ideal derivative filter.

Laplacian Approximation

The Laplacian operator is defined as the sum of the second derivatives *x* and *y*:

$$L = \frac{\partial^2}{\partial x^2} + \frac{\partial^2}{\partial y^2} . \qquad (3.10)$$

Thus, any of the equations defined in the sections for second derivatives may be used to calculate the Laplacian for an image.

Feature Detection

A set of Sobel derivative filters may be used to find edges, ridges, and blobs, especially in a scale-space, or image pyramid, situation. Below follows a description of methods in which the filter set could be applied.

- D_x is the first derivative in the direction *x* just as D_y.
- D_{xx} is the second derivative in the direction *x* just as D_{yy}.
- D_{xy} is the partial derivative with respect to *x* and *y*.
- D_{xxx} is the third derivative in the direction *x* just as D_{yyy}.

- D_{xxy} and D_{xyy} are the third partials in the directions x, y.

Corner Detection

Method 1

Corners may be defined as areas where level curves multiplied by the gradient magnitude raised to the power of 3 assume a local maximum

$$D_x^2 D_{yy} + D_y^2 D_{xx} - 2D_x D_y D_{xy} . \qquad (3.11)$$

Method 2

Sobel first derivative operators are used to take the derivatives x and y of an image, after which a small region of interest is defined to detect corners in. A 2x2 matrix of the sums of the derivatives x and y is subsequently created as follows:

$$C = \begin{bmatrix} \sum D_x^2 & \sum D_x D_y \\ \sum D_x D_y & \sum D_y^2 \end{bmatrix} \qquad (3.12)$$

The eigenvalues are found by solving $det(C - \lambda I) = 0$, where λ is a column vector of the eigenvalues and I is the identity matrix. For the 2x2 matrix of the equation above, the solutions may be written in a closed form:

$$\lambda = \frac{\sum D_x^2 + \sum D_y^2 \pm \sqrt{\left(\sum D_x^2 + \sum D_y^2\right)^2 - 4\left(\sum D_x^2 \sum D_y^2 - \left(\sum D_x D_y\right)^2\right)}}{2} . \qquad (3.13)$$

If $\lambda_1, \lambda_2 > t$, where t is some threshold, then a corner is found at that location. This can be very useful for object or shape recognition.

Canny Edge Detector

Edges are the boundaries separating regions with different brightness or color. J.Canny suggested in [Canny86] an efficient method for detecting edges. It takes grayscale image on input and returns bi-level image where non-zero pixels mark detected edges. Below the 4-stage algorithm is described.

Stage 1. Image Smoothing

The image data is smoothed by a Gaussian function of width specified by the user parameter.

Stage 2. Differentiation

The smoothed image, retrieved at Stage 1, is differentiated with respect to the directions x and y.

From the computed gradient values x and y, the magnitude and the angle of the gradient can be calculated using the hypotenuse and arctangen functions.

In the OpenCV library smoothing and differentiation are joined in Sobel operator.

Stage 3. Non-Maximum Suppression

After the gradient has been calculated at each point of the image, the edges can be located at the points of local maximum gradient magnitude. It is done via suppression of non-maximums, that is points, whose gradient magnitudes are not local maximums. However, in this case the non-maximums perpendicular to the edge direction, rather than those in the edge direction, have to be suppressed, since the edge strength is expected to continue along an extended contour.

The algorithm starts off by reducing the angle of gradient to one of the four sectors shown in Figure 1-8. The algorithm passes the 3x3 neighborhood across the magnitude array. At each point the center element of the neighborhood is compared with its two neighbors along line of the gradient given by the sector value.

If the central value is non-maximum, that is, not greater than the neighbors, it is suppressed.

Figure 3-8 Gradient Sectors

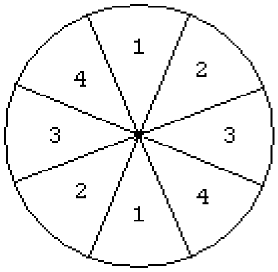

Stage 4. Edge Thresholding

The Canny operator uses the so-called “hysteresis” thresholding. Most thresholders use a single threshold limit, which means that if the edge values fluctuate above and below this value, the line appears broken. This phenomenon is commonly referred to as “streaking”. Hysteresis counters streaking by setting an upper and lower edge value limit. Considering a line segment, if a value lies above the upper threshold limit it is immediately accepted. If the value lies below the low threshold it is immediately rejected. Points which lie between the two limits are accepted if they are connected to pixels which exhibit strong response. The likelihood of streaking is reduced drastically since the line segment points must fluctuate above the upper limit and below the lower limit for streaking to occur. J. Canny recommends in [Canny86] the ratio of high to low limit to be in the range of two or three to one, based on predicted signal-to-noise ratios.

Hough Transform

The Hough Transform (HT) is a popular method of extracting geometric primitives from raster images. The simplest version of the algorithm just detects lines, but it is easily generalized to find more complex features. There are several classes of HT that differ by the image information available. If the image is arbitrary, the Standard Hough Transform (SHT, [Trucco98]) should be used.

SHT, like all HT algorithms, considers a discrete set of single primitive parameters. If lines should be detected, then the parameters are ρ and θ, such that the line equation is $\rho = x\cos(\theta) + y\sin(\theta)$. Here

ρ	is the distance from the origin to the line, and
θ	is the angle between the axis x and the perpendicular to the line vector that points from the origin to the line.

Every pixel in the image may belong to many lines described by a set of parameters. In other words, the accumulator is defined which is an integer array $A(\rho, \theta)$ containing only zeroes initially. For each non-zero pixel in the image all accumulator elements corresponding to lines that contain the pixel are incremented by 1. Then a threshold is applied to distinguish lines and noise features, that is, select all pairs (ρ, θ) for which $A(\rho, \theta)$ is greater than the threshold value. All such pairs characterize detected lines.

Multidimensional Hough Transform (MHT) is a modification of SHT. It performs precalculation of SHT on rough resolution in parameter space and detects the regions of parameter values that possibly have strong support, that is, correspond to lines in the source image. MHT should be applied to images with few lines and without noise.

[Matas98] presents advanced algorithm for detecting multiple primitives, Progressive Probabilistic Hough Transform (PPHT). The idea is to consider random pixels one by one. Every time the accumulator is changed, the highest peak is tested for threshold exceeding. If the test succeeds, points that belong to the corridor specified by the peak are removed. If the number of points exceeds the predefined value, that is, minimum line length, then the feature is considered a line, otherwise it is considered a noise. Then the process repeats from the very beginning until no pixel remains in the image. The algorithm improves the result every step, so it can be stopped any time. [Matas98] claims that PPHT is easily generalized in almost all cases where SHT could be generalized. The disadvantage of this method is that, unlike SHT, it does not process some features, for instance, crossed lines, correctly.

For more information see [Matas98] and [Trucco98].

Image Statistics

This section describes a set of functions that compute various information about images, considering their pixels as independent observations of a stochastic variable.

The computed values have statistical character and most of them depend on values of the pixels rather than on their relative positions. These statistical characteristics represent integral information about a whole image or its regions.

The functions `CountNonZero`, `SumPixels`, `Mean`, `Mean_StdDev`, `MinMaxLoc` describe the characteristics that are typical for any stochastic variable or deterministic set of numbers, such as mean value, standard deviation, min and max values.

The function `Norm` describes the function for calculating the most widely used norms for a single image or a pair of images. The latter is often used to compare images.

The functions `Moments`, `GetSpatialMoment`, `GetCentralMoment`, `GetNormalizedCentralMoment`, `GetHuMoments` describe moments functions for calculating integral geometric characteristics of a *2D* object, represented by grayscale or bi-level raster image, such as mass center, orientation, size, and rough shape description. As opposite to simple moments, that are used for characterization of any stochastic variable or other data, Hu invariants, described in the last function discussion, are unique for image processing because they are specifically designed for *2D* shape characterization. They are invariant to several common geometric transformations.

Pyramids

This section describes functions that support generation and reconstruction of Gaussian and Laplacian Pyramids.

Figure 1-9 shows the basics of creating Gaussian or Laplacian pyramids. The original image G_0 is convolved with a Gaussian, then down-sampled to get the reduced image G_1. This process can be continued as far as desired or until the image size is one pixel.

The Laplacian pyramid can be built from a Gaussian pyramid as follows: Laplacian level “k” can be built by up-sampling the lower level image G_{k+1}. Convolving the image with a Gaussian kernel “g” interpolates the pixels “missing” after up-sampling. The resulting image is subtracted from the image G_k. To rebuild the original image, the process is reversed as Figure 1-9 shows.

Figure 3-9 A Three-Level Gaussian and Laplacian Pyramid.

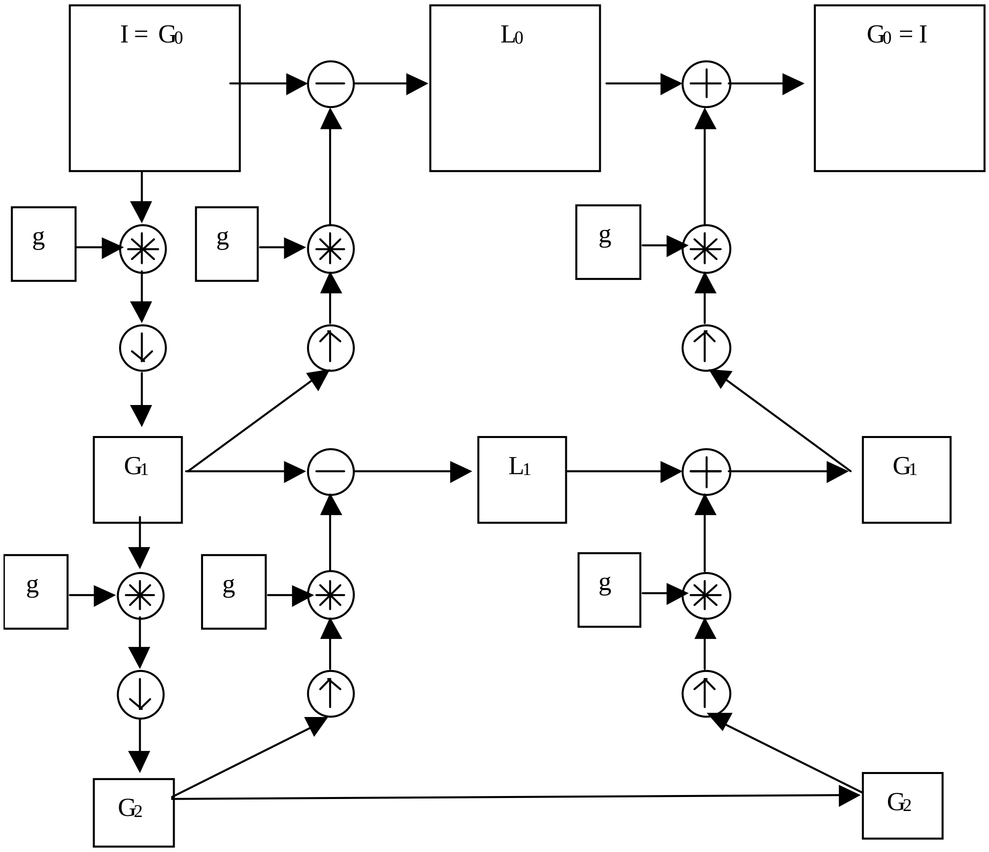

The Gaussian image pyramid on the left is used to create the Laplacian pyramid in the center, which is used to reconstruct the Gaussian pyramid and the original image on the right. In the figure, I is the original image, G is the Gaussian image, L is the Laplacian image. Subscripts denote level of the pyramid. A Gaussian kernel g is used to convolve the image before down-sampling or after up-sampling.

Image Segmentation by Pyramid

Computer vision uses pyramid based image processing techniques on a wide scale now. The pyramid provides a hierarchical smoothing, segmentation, and hierarchical computing structure that supports fast analysis and search algorithms.

P. J. Burt suggested a pyramid-linking algorithm as an effective implementation of a combined segmentation and feature computation algorithm [Burt81]. This algorithm, described also in [Jahne97], finds connected components without preliminary threshold, that is, it works on grayscale image. It is an iterative algorithm.

Burt's algorithm includes the following steps:

1. Computation of the Gaussian pyramid.
2. Segmentation by pyramid-linking.
3. Averaging of linked pixels.

Steps 2 and 3 are repeated iteratively until a stable segmentation result is reached.

After computation of the Gaussian pyramid a son-father relationship is defined between nodes (pixels) in adjacent levels. The following attributes may be defined for every node (i,j) on the level l of the pyramid:

$c[i,j,l][t]$ is the value of the local image property, e.g., intensity;

$a[i,j,l][t]$ is the area over which the property has been computed;

$p[[i,j,l][t]$ is pointer to the node's father, which is at level $l+1$;

$s[i,j,l][t]$ is the segment property, the average value for the entire segment containing the node.

The letter t stands for the iteration number $(t \geq 0)$. For $t = 0$, $c[i,j,l][0] = G^{l}_{i,j}$.

For every node (i,j) at level l there are 16 candidate son nodes at level l-1 (i',j'), where

$$i' \in \{2i-1, 2i, 2i+1, 2i+2\},\ j' \in \{2j-1, 2j, 2j+1, 2j+2\}. \quad (3.14)$$

For every node (i,j) at level l there are 4 candidate father nodes at level $l+1$ (i'',j''), (see Figure 1-10), where

$$i'' \in \{(i-1)/2, i+1)/2\},\ j'' \in \{(j-1)/2, j+1)/2\}. \quad (3.15)$$

Son-father links are established for all nodes below the top of pyramid for every iteration t. Let $d[n][t]$ be the absolute difference between the c value of the node (i,j) at level l and its nth candidate father, then

$$p[i,j,l][t] = \arg\min_{1 \le n \le 4} d[n][t] \quad (3.16)$$

Figure 3-10 Connections between Adjacent Pyramid Levels

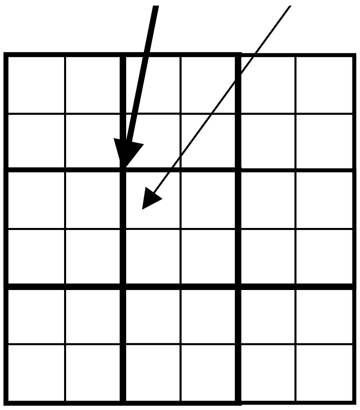

After the son-father relationship is defined, the t, c, and a values are computed from bottom to the top for the $0 \le l \le n$ as

$$a[i,j,0][t] = 1,\ c[i,j,0][t] = c[i,j,0][0],\ a[i,j,l][t] = \sum a[i',j',l-1][t],$$

where sum is calculated over all (i,j) node sons, as indicated by the links p in (3.16).

If $a[i, j, l][t] > 0$ then $c[i, j, l][t] = \sum([i', j', l' - 1][t] \cdot c[i', j', l - 1][t]) / a[i, j, l][t]$, but if $a[i, j, 0][t] = 0$, the node has no sons, $c[i, j, 0][t]$ is set to the value of one of its candidate sons selected at random. No segment values are calculated in the top down order. The value of the initial level L is an input parameter of the algorithm. At the level L the segment value of each node is set equal to its local property value:

$s[i, j, L][t] = c[i, j, L][t]$.

For lower levels $l < L$ each node value is just that of its father
$s[i, j, l][t] = c[i'', j'', l + 1][t]$.

Here node (i'', j'') is the father of (i, j), as established in Equation (3.16).

After this the current iteration t finishes and the next iteration $t + 1$ begins. Any changes in pointers in the next iteration result in changes in the values of local image properties.

The iterative process is continued until no changes occur between two successive iterations.

The choice of L only determines the maximum possible number of segments. If the number of segments less than the numbers of nodes at the level L, the values of $c[i, j, L][t]$ are clustered into a number of groups equal to the desired number of segments. The group average value is computed from the c values of its members, weighted by their areas a, and replaces the value c for each node in the group.

See Pyramid Data Types in Image Analysis Reference.

Morphology

This section describes an expanded set of morphological operators that can be used for noise filtering, merging or splitting image regions, as well as for region boundary detection.

Mathematical Morphology is a set-theory method of image analysis first developed by Matheron and Serra at the Ecole des Mines, Paris [Serra82]. The two basic morphological operations are erosion, or thinning, and dilation, or thickening. All operations involve an image A, called the *object of interest*, and a kernel element B, called the *structuring element*. The image and structuring element could be in any number of dimensions, but the most common use is with a *2D* binary image, or with a

3D grayscale image. The element B is most often a square or a circle, but could be any shape. Just like in convolution, B is a kernel or template with an anchor point. Figure 1-11 shows dilation and erosion of object A by B. The element B is rectangular with an anchor point at upper left shown as a dark square.

Figure 3-11 Dilation and Erosion of A by B

.

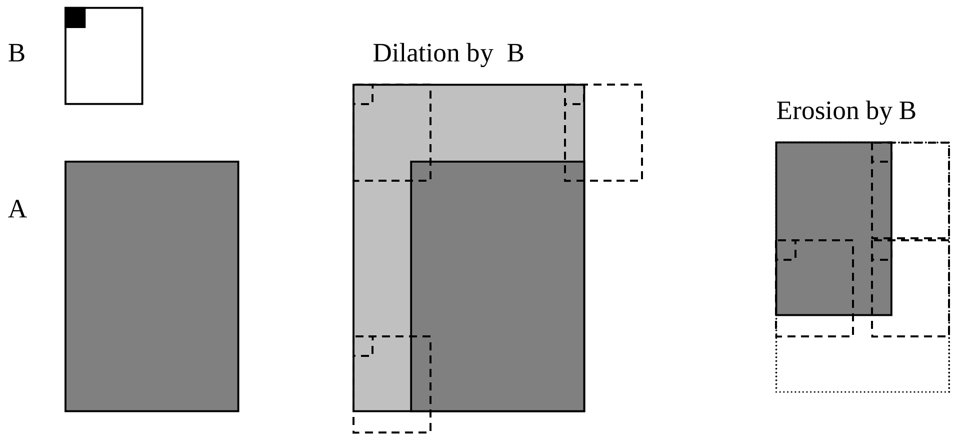

If B_t is the translation of B around the image, then dilation of object A by structuring element B is

$$A \oplus B = \{ t : B_t \cap A \neq 0 \}.$$

It means every pixel is in the set, if the intersection is not null. That is, a pixel under the anchor point of B is marked "on", if at least one pixel of B is inside of A.

$A \oplus nB$ indicates the dilation is done n times.

Erosion of object A by structuring element B is

$$A \Theta B = \{ t : B_t \subseteq A \}.$$

That is, a pixel under the anchor of B is marked "on", if B is entirely within A.

$A \Theta nB$ indicates the erosion is done n times and can be useful in finding ∂A, the boundary of A:

$$\partial A = A - (A \Theta nB).$$

Opening of A by B is

$$A \circ B = (A \Theta nB) \oplus nB. \quad (3.17)$$

Closing of A by B is

$$A \bullet B = (A \oplus nB) \Theta nB, \quad (3.18)$$

where $n > 0$.

Flat Structuring Elements for Gray Scale

Erosion and dilation can be done in *3D*, that is, with gray levels. *3D* structuring elements can be used, but the simplest and the best way is to use a flat structuring element B as shown in Figure 1-12. In the figure, B has an anchor slightly to the right of the center as shown by the dark mark on B. Figure 1-12 shows *1D* cross-section of both dilation and erosion of a gray level image A by a flat structuring element B.

Figure 3-12 Dilation and Erosion of Gray Scale Image.

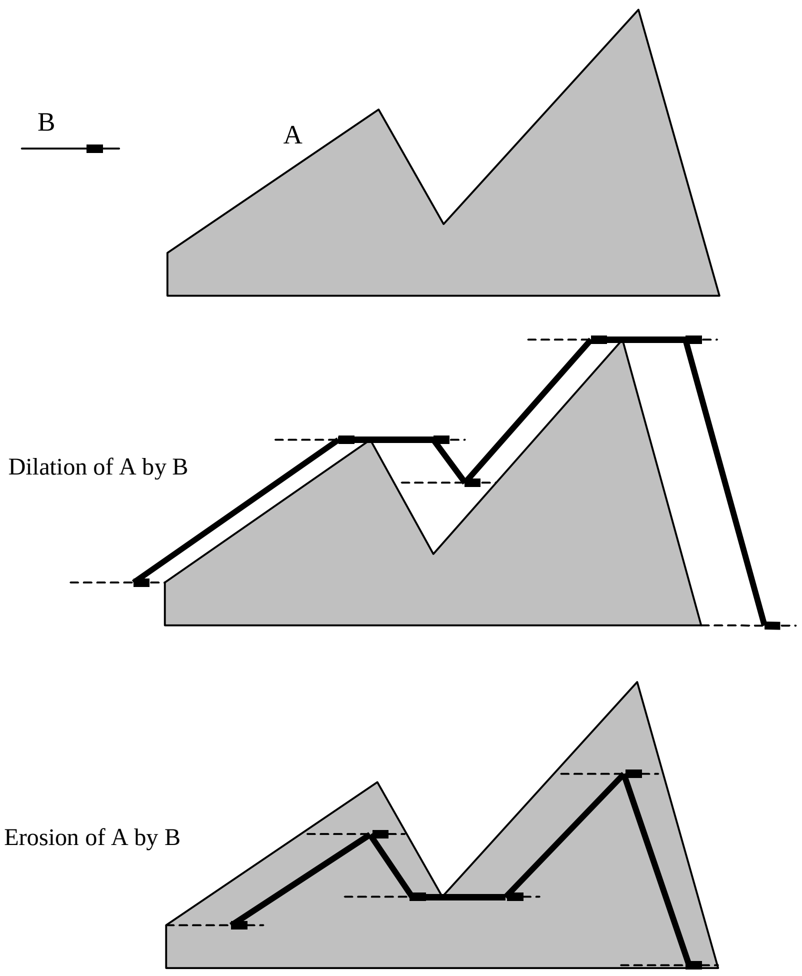

In Figure 1-12 dilation is mathematically

$\sup_{y \in B_t} A$,

and erosion is

$$\inf_{y \in B_t} A \quad .$$

Open and Close Gray Level with Flat Structuring Element

The typical position of the anchor of the structuring element B for opening and closing is in the center. Subsequent opening and closing could be done in the same manner as in the Opening (3.17) and Closing (3.18) equations above to smooth off jagged objects as opening tends to cut off peaks and closing tends to fill in valleys.

Morphological Gradient Function

A morphological gradient may be taken with the flat gray scale structuring elements as follows:

$$grad(A) = \frac{(A \oplus B_{flat}) - (A \Theta B_{flat})}{2} .$$

Top Hat and Black Hat

Top Hat (TH) is a function that isolates bumps and ridges from gray scale objects. In other words, it can detect areas that are lighter than the surrounding neighborhood of A and smaller compared to the structuring element. The function subtracts the opened version of A from the gray scale object A:

$$TH_B(A) = A - (A \circ nB_{flat}) .$$

Black Hat (TH^d) is the dual function of Top Hat in that it isolates valleys and “cracks off” ridges of a gray scale object A, that is, the function detects dark and thin areas by subtracting A from the closed image A:

$$TH_B^d(A) = (A \bullet nB_{flat}) - A .$$

Thresholding often follows both Top Hat and Black Hat operations.

Distance Transform

This section describes the distance transform used for calculating the distance to an object. The input is an image with feature and non-feature pixels. The function labels every non-feature pixel in the output image with a distance to the closest feature pixel.

Feature pixels are marked with zero. Distance transform is used for a wide variety of subjects including skeleton finding and shape analysis. The [Borgefors86] two-pass algorithm is implemented.

Thresholding

This section describes threshold functions group.

Thresholding functions are used mainly for two purposes:

— masking out some pixels that do not belong to a certain range, for example, to extract blobs of certain brightness or color from the image;

— converting grayscale image to bi-level or black-and-white image.

Usually, the resultant image is used as a mask or as a source for extracting higher-level topological information, e.g., contours (see Active Contours), skeletons (see Distance Transform), lines (see Hough Transform functions), etc.

Generally, threshold is a determined function $t(x,y)$ on the image:

$$t(x,y) = \begin{cases} A(p(x,y)), & f(x,y,p(x,y)) = true \\ B(p(x,y)), & f(x,y,p(x,y)) = false \end{cases}$$

The predicate function $f(x,y,p(x,y))$ is typically represented as $g(x,y) < p(x,y) < h(x,y)$, where g and h are some functions of pixel value and in most cases they are simply constants.

There are two basic types of thresholding operations. The first type uses a predicate function, independent from location, that is, $g(x,y)$ and $h(x,y)$ are constants over the image. However, for concrete image some optimal, in a sense, values for the constants can be calculated using image histograms (see Histogram) or other statistical criteria (see Image Statistics). The second type of the functions chooses $g(x,y)$ and $h(x,y)$ depending on the pixel neigborhood in order to extract regions of varying brightness and contrast.

The functions, described in this chapter, implement both these approaches. They support single-channel images with depth `IPL_DEPTH_8U`, `IPL_DEPTH_8S` or `IPL_DEPTH_32F` and can work in-place.

Flood Filling

This section describes the function performing flood filling of a connected domain.

Flood filling means that a group of connected pixels with close values is filled with, or is set to, a certain value. The flood filling process starts with some point, called "seed", that is specified by function caller and then it propagates until it reaches the image `ROI` boundary or cannot find any new pixels to fill due to a large difference in pixel values. For every pixel that is just filled the function analyses:

- 4 neighbors, that is, excluding the diagonal neighbors; this kind of connectivity is called 4-connectivity, or
- 8 neighbors, that is, including the diagonal neighbors; this kind of connectivity is called 8-connectivity.

The parameter *`connectivity`* of the function specifies the type of connectivity.

The function can be used for:

- segmenting a grayscale image into a set of uni-color areas,
- marking each connected component with individual color for bi-level images.

The function supports single-channel images with the depth `IPL_DEPTH_8U` or `IPL_DEPTH_32F`.

Histogram

This section describes functions that operate on multi-dimensional histograms.

Histogram is a discrete approximation of stochastic variable probability distribution. The variable can be either a scalar or a vector. Histograms are widely used in image processing and computer vision. For example, one-dimensional histograms can be used for:

- grayscale image enhancement
- determining optimal threshold levels (see Thresholding)
- selecting color objects via hue histograms back projection (see CamShift), and other operations.

Two-dimensional histograms can be used for:

- analyzing and segmenting color images, normalized to brightness (e.g. red-green or hue-saturation images),
- analyzing and segmenting motion fields (x-y or magnitude-angle histograms),
- analyzing shapes (see `CalcPGH` in Geometry Functions section of Structural Analysis Reference) or textures.

Multi-dimensional histograms can be used for:

- content based retrieval (see the function `CalcPGH`),
- bayesian-based object recognition (see [Schiele00]).

To store all the types of histograms (`1D, 2D, nD`), OpenCV introduces special structure `CvHistogram` described in Example 2-2 in Image Analysis Reference.

Any histogram can be stored either in a dense form, as a multi-dimensional array, or in a sparse form with a balanced tree used now. However, it is reasonable to store *1D* or *2D* histograms in a dense form and *3D* and higher dimensional histograms in a sparse form.

The type of histogram representation is passed into histogram creation function and then it is stored in *type* field of `CvHistogram`. The function `MakeHistHeaderForArray` can be used to process histograms allocated by the user with Histogram Functions.

Histograms and Signatures

Histograms represent a simple statistical description of an object, e.g., an image. The object characteristics are measured during iterating through that object: for example, color histograms for an image are built from pixel values in one of the color spaces. All possible values of that multi-dimensional characteristic are further quantized on each coordinate. If the quantized characteristic can take different k_1 values on the first coordinate, k_2 values on second, and k_n on the last one, the resulting histogram has the size

$$size = \prod_{i=1}^{n} k_i .$$

The histogram can be viewed as a multi-dimensional array. Each dimension corresponds to a certain object feature. An array element with coordinates [i_1, i_2 … i_n], otherwise called a histogram bin, contains a number of measurements done for the object with quantized value equal to i_1 on first coordinate, i_2 on the second coordinate, and so on. Histograms can be used to compare respective objects:

$$D_{L_1}(H, K) = \sum_i |h_i - k_i|, \text{ or}$$

$$D(H, K) = \sqrt{(\bar{h} - \bar{k})^T A(\bar{h} - \bar{k})}.$$

But these methods suffer from several disadvantages. The measure D_{L_1} sometimes gives too small difference when there is no exact correspondence between histogram bins, that is, if the bins of one histogram are slightly shifted. On the other hand, D_{L_2} gives too large difference due to cumulative property.

Another drawback of pure histograms is large space required, especially for higher-dimensional characteristics. The solution is to store only non-zero histogram bins or a few bins with the highest score. Generalization of histograms is termed *signature* and defined in the following way:

1. Characteristic values with rather fine quantization are gathered.
2. Only non-zero bins are dynamically stored.

This can be implemented using hash-tables, balanced trees, or other sparse structures. After processing, a set of clusters is obtained. Each of them is characterized by the coordinates and weight, that is, a number of measurements in the neighborhood. Removing clusters with small weight can further reduce the signature size. Although these structures cannot be compared using formulas written above, there exists a robust comparison method described in [RubnerJan98] called Earth Mover Distance.

Earth Mover Distance (EMD)

Physically, two signatures can be viewed as two systems - earth masses, spread into several localized pieces. Each piece, or cluster, has some coordinates in space and weight, that is, the earth mass it contains. The distance between two systems can be measured then as a minimal work needed to get the second configuration from the first or vice versa. To get metric, invariant to scale, the result is to be divided by the total mass of the system.

Mathematically, it can be formulated as follows.

Consider m suppliers and n consumers. Let the capacity of ith supplier be x_i and the capacity of jth consumer be y_j. Also, let the ground distance between i^{th} supplier and j^{th} consumer be c_{ij}. The following restrictions must be met:

$$x_i \geq 0, y_j \geq 0, c_{i,j} \geq 0,$$

$$\sum_i x_i \geq \sum_j y_j,$$

$$0 \leq i < m, 0 \leq j < n.$$

Then the task is to find the flow matrix $\|f_{ij}\|$, where f_{ij} is the amount of earth, transferred from i^{th} supplier to j^{th} consumer. This flow must satisfy the restrictions below:

$$f_{i,j} \geq 0,$$

$$\sum_i f_{i,j} \leq x_i,$$

$$\sum_j f_{i,j} = y$$

and minimize the overall cost:

$$min \sum_i \sum_j c_{i,j}, f_{i,j}.$$

If $\|f_{ij}\|$ is the optimal flow, then Earth Mover Distance is defined as

$$EMD(x, y) = \frac{\sum_i \sum_j c_{i,j} f_{i,j}}{\sum_i \sum_j f_{i,j}}.$$

The task of finding the optimal flow is a well known transportation problem, which can be solved, for example, using the simplex method.

Example Ground Distances

As shown in the section above, physically intuitive distance between two systems can be found if the distance between their elements can be measured. The latter distance is called ground distance and, if it is a true metric, then the resultant distance between systems is a metric too. The choice of the ground distance depends on the concrete task as well as the choice of the coordinate system for the measured characteristic. In [RubnerSept98], [RubnerOct98] three different distances are considered.

1. The first is used for human-like color discrimination between pictures. CIE Lab model represents colors in a way when a simple Euclidean distance gives true human-like discrimination between colors. So, converting image pixels into CIE Lab format, that is, representing colors as *3D* vectors (L,a,b), and quantizing them (in 25 segments on each coordinate in [RubnerSept98]), produces a color-based signature of the image. Although in experiment, made in [RubnerSept98], the maximal number of non-zero bins could be 25x25x25 = 15625, the average number of clusters was ~8.8, that is, resulting signatures were very compact.
2. The second example is more complex. Not only the color values are considered, but also the coordinates of the corresponding pixels, which makes it possible to differentiate between pictures of similar color palette but representing different color regions placements: e.g., green grass at the bottom and blue sky on top vs. green forest on top and blue lake at the bottom. *5D* space is used and metric is: $[(\Delta L)^2 + (\Delta a)^2 + (\Delta b)^2 + \lambda((\Delta x)^2 + (\Delta y)^2)]^{1/2}$, where λ regulates importance of the spatial correspondence. When $\lambda = 0$, the first metric is obtained.
3. The third example is related to texture metrics. In the example Gabor transform is used to get the *2D* vector texture descriptor (l, m), which is a log-polar characteristic of the texture. Then, no-invariance ground distance is defined as: $d((l_1, m_1), (l_2, m_2)) = |\Delta l| + \alpha|\Delta m|$, $\Delta l = min(|l_1 - l_2|, L - |l_1 - l_2|)$, $\Delta m = |m_1 - m_2|$, where α is the scale parameter of Gabor transform, L is the number of different angles used (angle resolution), and M is the number of scales used (scale resolution). To get invariance to scale and rotation, the user may calculate minimal EMD for several scales and rotations:

$(l_1, m_1), (l_2, m_2)$,

$$EMD(t_1, t_2) = \min_{\substack{0 \le l_0 < L \\ -M < m_0 < M}} EMD(t_1, t_2, l_0, m_0),$$

where d is measured as in the previous case, but Δl and Δm look slightly different:

$$\Delta l = min(|l_1 - l_2 + l_0(\mathrm{mod}L)|, L - |l_1 - l_2 + l_0(\mathrm{mod}L)|),\ \Delta m = |m_1 - m_2 + m_0|.$$

Lower Boundary for EMD

If ground distance is metric and distance between points can be calculated via the norm of their difference, and total suppliers' capacity is equal to total consumers' capacity, then it is easy to calculate lower boundary of EMD because:

$$\sum_i \sum_j c_{i,j}, f_{i,j} = \sum_i \sum_j \|p_i - q_j\| f_{i,j} = \sum_i \sum_j \|p_i - q_j\| f_{i,j}$$

$$\ge \left\| \sum_i \sum_j \|p_i - q_i\| f_{i,j} \right\| = \left\| \sum_i \left(\sum_j f_{i,j} \right) p_i - \sum_j \left(\sum_i f_{i,j} \right) q_j \right\|$$

$$= \left\| \sum_i x_i p_i - \sum_j y_j q_j \right\|$$

As it can be seen, the latter expression is the distance between the mass centers of the systems. Poor candidates can be efficiently rejected using this lower boundary for EMD distance, when searching in the large image database.

Structural Analysis 4

Contour Processing

This section describes contour processing functions.

Polygonal Approximation

As soon as all the borders have been retrieved from the image, the shape representation can be further compressed. Several algorithms are available for the purpose, including RLE coding of chain codes, higher order codes (see Figure 4-1), polygonal approximation, etc.

Figure 4-1 Higher Order Freeman Codes

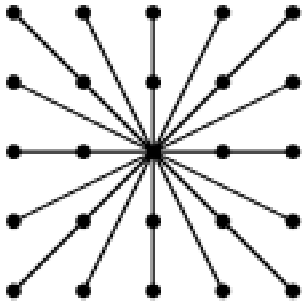

24-Point Extended Chain Code

Polygonal approximation is the best method in terms of the output data simplicity for further processing. Below follow descriptions of two polygonal approximation algorithms. The main idea behind them is to find and keep only the dominant points, that is, points where the local maximums of curvature absolute value are located on the digital curve, stored in the chain code or in another direct representation format. The first step here is the introduction of a discrete analog of curvature. In the continuous case the curvature is determined as the speed of the tangent angle changing:

$$k = \frac{x'y'' - x''y'}{(x'^2 + y'^2)^{3/2}}.$$

In the discrete case different approximations are used. The simplest one, called L1 curvature, is the difference between successive chain codes:

$$c_i^{(1)} = ((f_i - f_{i-1} + 4) mod 8) - 4. \qquad (4.1)$$

This method covers the changes from 0, that corresponds to the straight line, to 4, that corresponds to the sharpest angle, when the direction is changed to reverse.

The following algorithm is used for getting a more complex approximation. First, for the given point (x_i, y_i) the radius m_i of the neighborhood to be considered is selected. For some algorithms m_i is a method parameter and has a constant value for all points; for others it is calculated automatically for each point. The following value is calculated for all pairs (x_{i-k}, y_{i-k}) and (x_{i+k}, y_{i+k}) ($k=1...m$):

$$c_{ik} = \frac{(a_{ik} \cdot b_{ik})}{|a_{ik}||b_{ik}|} = \cos(a_{ik}, b_{ik}),$$

where $a_{ik} = (x_{i-k} - x_i, y_{i-k} - y_i)$, $b_{ik} = (x_{i+k} - x_i, y_{i-k} - y_i)$.

The next step is finding the index h_i such that $c_{im} < c_{im-1} < \ldots < c_{ih_i} \geq c_{ih_i - 1}$. The value c_{ih_i} is regarded as the curvature value of the ith point. The point value changes from –1 (straight line) to 1 (sharpest angle). This approximation is called the *k*-cosine curvature.

Rosenfeld-Johnston algorithm [Rosenfeld73] is one of the earliest algorithms for determining the dominant points on the digital curves. The algorithm requires the parameter m, the neighborhood radius that is often equal to 1/10 or 1/15 of the number of points in the input curve. Rosenfeld-Johnston algorithm is used to calculate curvature values for all points and remove points that satisfy the condition

$$\exists j, |i - j| \leq h_i/2 ; c_{ih_i} < c_{jh_j}.$$

The remaining points are treated as dominant points. Figure 4-2 shows an example of applying the algorithm.

Figure 4-2 Rosenfeld-Johnston Output for F-Letter Contour

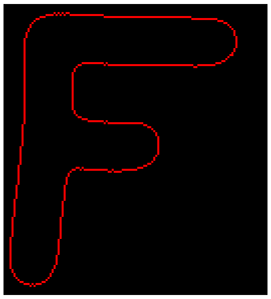

Source Image

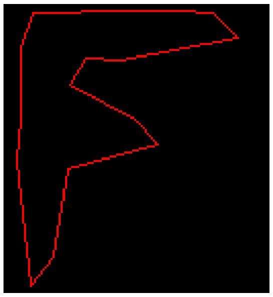

Rosenfeld-Johnston Algorithm Output

The disadvantage of the algorithm is the necessity to choose the parameter m and parameter identity for all the points, which results in either excessively rough, or excessively precise contour approximation.

The next algorithm proposed by Teh and Chin [Teh89] includes a method for the automatic selection of the parameter m for each point. The algorithm makes several passes through the curve and deletes some points at each pass. At first, all points with zero $c_i^{(1)}$ curvatures are deleted (see Equation 5.1). For other points the parameter m_i and the curvature value are determined. After that the algorithm performs a non-maxima suppression, same as in Rosenfeld-Johnston algorithm, deleting points whose curvature satisfies the previous condition where for $c_i^{(1)}$ the metric h_i is set to m_i. Finally, the algorithm replaces groups of two successive remaining points with a single point and groups of three or more successive points with a pair of the first and the last points. This algorithm does not require any parameters except for the curvature to use. Figure 4-3 shows the algorithm results.

Figure 4-3 Teh-Chin Output for F-Letter Contour

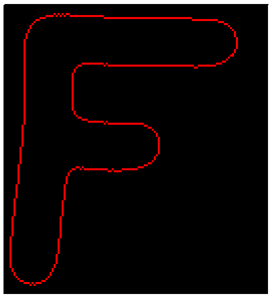

Source Picture

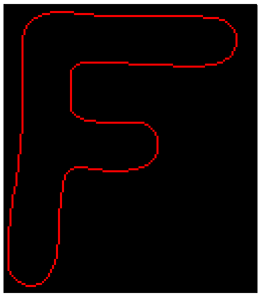

Teh-Chin Algorithm Output

Douglas-Peucker Approximation

Instead of applying a rather sophisticated Teh-Chin algorithm to the chain code, the user may try another way to get a smooth contour on a little number of vertices. The idea is to apply some very simple approximation techniques to the chain code with polylines, such as substituting ending points for horizontal, vertical, and diagonal segments, and then use the approximation algorithm on polylines. This preprocessing reduces the amount of data without any accuracy loss. Teh-Chin algorithm also involves this step, but uses removed points for calculating curvatures of the remaining points.

The algorithm to consider is a pure geometrical algorithm by Douglas-Peucker for approximating a polyline with another polyline with required accuracy:

1. Two points on the given polyline are selected, thus the polyline is approximated by the line connecting these two points. The algorithm iteratively adds new points to this initial approximation polyline until the

required accuracy is achieved. If the polyline is not closed, two ending points are selected. Otherwise, some initial algorithm should be applied to find two initial points. The more extreme the points are, the better.

2. The algorithm iterates through all polyline vertices between the two initial vertices and finds the farthest point from the line connecting two initial vertices. If this maximum distance is less than the required error, then the approximation has been found and the next segment, if any, is taken for approximation. Otherwise, the new point is added to the approximation polyline and the approximated segment is split at this point. Then the two parts are approximated in the same way, since the algorithm is recursive. For a closed polygon there are two polygonal segments to process.

Contours Moments

The moment of order *(p; q)* of an arbitrary region *R* is given by

$$\nu_{pq} = \iint_R x^p \cdot y^q dx dy . \qquad (4.2)$$

If $p = q = 0$, we obtain the area *a* of *R*. The moments are usually normalized by the area *a* of *R*. These moments are called normalized moments:

$$\alpha_{pq} = (1/a)\iint_R x^p \cdot y^q dx dy . \qquad (4.3)$$

Thus $\alpha_{00} = 1$. For $p + q \geq 2$ normalized central moments of *R* are usually the ones of interest:

$$\mu_{pq} = 1/a\iint_R (x - a_{10})^p \cdot (y - a_{01})^q dx dy \qquad (4.4)$$

It is an explicit method for calculation of moments of arbitrary closed polygons. Contrary to most implementations that obtain moments from the discrete pixel data, this approach calculates moments by using only the border of a region. Since no explicit region needs to be constructed, and because the border of a region usually consists of significantly fewer points than the entire region, the approach is very efficient. The well-known Green’s formula is used to calculate moments:

$$\iint_R (\partial Q/(\partial x - \partial P/\partial y) dx dy = \int_b (P dx + Q dy),$$

where b is the border of the region R.

It follows from the formula (4.2) that:

$$\partial Q/\partial x = x^p \cdot y^q, \partial P/\partial y = 0,$$

hence

$$P(x, y) = 0, Q(x, y) = 1/(p+1) \cdot x^{p+1} y^q.$$

Therefore, the moments from (4.2) can be calculated as follows:

$$v_{pq} = \int_b (1/(p+1) x^{p+1} \cdot y^q) dy. \tag{4.5}$$

If the border b consists of n points $p_i = (x_i, y_i)$, $0 \le i \le n$, $p_0 = p_n$, it follows that:

$$b(t) = \bigcup_{i=1}^{n} b_i(t),$$

where $b_i(t)$, $t \in [0,1]$ is defined as

$$b_i(t) = tp + (1-t) p_{i-1}.$$

Therefore, (4.5) can be calculated in the following manner:

$$v_{pq} = \sum_{i=1}^{n} \int_{b_j} (1/(p+1) x^{p+1} \cdot y^q) dy \tag{4.6}$$

After unnormalized moments have been transformed, (4.6) could be written as:

$$v_{pA} = \frac{1}{(p+q+2)(p+q+1)\binom{p+q}{p}} \times \sum_{i=1}^{n} (x_{i-1} y_i - x_i y_{i-1}) \sum_{k=0}^{p} \sum_{i=0}^{q} \binom{k+t}{t} \binom{p+q-k-t}{q-t} x_i^k x_{i-1}^{p-k} y_i^t y_{i-1}^{q-t}$$

Central unnormalized and normalized moments up to order 3 look like

$$a = 1/2 \sum_{i=1}^{''} x_{i-1}y_i - x_i y_{i-1},$$

$$a_{10} = 1/(6a) \sum_{i=1}^{''} (x_{i-1}y_i - x_i y_{i-1})(x_{i-1} + x_i),$$

$$a_{01} = 1/(6a) \sum_{i=1}^{''} (x_{i-1}y_i - x_i y_{i-1})(y_{i-1} + y_i),$$

$$a_{20} = 1/(12a) \sum_{i=1}^{''} (x_{i-1}y_i - x_i y_{i-1})(x_{i-1}^2 + x_{i-1}x_i + x_i^2),$$

$$a_{11} = 1/(24a) \sum_{i=1}^{''} (x_{i-1}y_i - x_i y_{i-1})(2x_{i-1} + x_{i-1}y_i + x_i y_{i-1} + 2x_i y_i),$$

$$a_{02} = 1/(12a) \sum_{i=1}^{''} (x_{i-1}y_i - x_i y_{i-1})(y_{i-1}^2 + y_{i-1}y_i + y_i^2),$$

$$a_{30} = 1/(20a) \sum_{i=1}^{''} (x_{i-1}y_i - x_i y_{i-1})(x_{i-1}^3 + x_{i-1}^2 x_i + x_i^2 x_{i-1} + x_i^3),$$

$$a_{21} = 1/(60a) \sum_{i=1}^{''} (x_{i-1}y_i - x_i y_{i-1})(x_{i-1}^2(3y_{i-1} + y_i) + 2x_{i-1}x_i(y_{i-1} + y_i)$$

$$+ x_i^2(y_{i-1} + 3y_i)),$$

$$a_{12} = 1/(60a) \sum_{i=1}^{''} (x_{i-1}y_i - x_i y_{i-1})(y_{i-1}^2(3x_{i-1} + x_i) + 2y_{i-1}y_i(x_{i-1} + x_i) +$$

$$y_i^2(x_{i-1} + 3x_i)),$$

$$a_{03} = 1/(20a) \sum_{i=1}^{''} (x_{i-1}y_i - x_i y_{i-1})(y_{i-1}^3 + y_{i-1}^2 y_i + y_i^2 y_{i-1} + y_i^3),$$

$$\mu_{20} = \alpha_{20} - \alpha_{10}^2,$$

$$\mu_{11} = \alpha_{11} - \alpha_{10}\alpha_{01},$$

$$\mu_{02} = \alpha_{02} - \alpha_{01}^2,$$

$$\mu_{30} = \alpha_{30} + 2\alpha_{10}^3 - 3\alpha_{10}\alpha_{20},$$

$$\mu_{21} = \alpha_{21} + 2\alpha_{10}^3\alpha_{01} - 2\alpha_{10}\alpha_{11} - \alpha_{20}\alpha_{01},$$

$$\mu_{12} = \alpha_{12} + 2\alpha_{01}^3\alpha_{10} - 2\alpha_{01}\alpha_{11} - \alpha_{02}\alpha_{10},$$

$$\mu_{03} = \alpha_{03} + 2\alpha_{01}^3 - 3\alpha_{01}\alpha_{02}.$$

Hierarchical Representation of Contours

Let T be the simple closed boundary of a shape with n points $T:\{p(1), p(2), \ldots, p(n)\}$ and n runs: $\{s(1), s(2), \ldots, s(n)\}$. Every run $s(i)$ is formed by the two points $(p(i), p(i+1))$. For every pair of the neighboring runs $s(i)$ and $s(i+1)$ a triangle is defined by the two runs and the line connecting the two far ends of the two runs (Figure 4-4).

Figure 4-4 Triangles Numbering

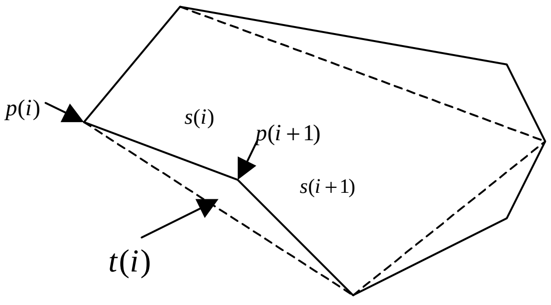

Triangles $t(i-2), t(i-1), t(i+1), t(i+2)$ are called neighboring triangles of $t(i)$ (Figure 4-5).

Figure 4-5 Location of Neighboring Triangles

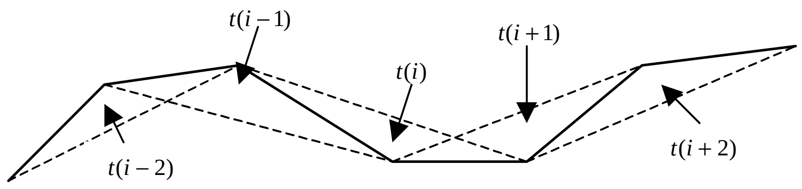

For every straight line that connects any two different vertices of a shape, the line either cuts off a region from the original shape or fills in a region of the original shape, or does both. The size of the region is called the interceptive area of that line (Figure 4-6). This line is called the base line of the triangle.

A triangle made of two boundary runs is the *locally minimum interceptive area triangle* (LMIAT) if the interceptive area of its base line is smaller than both its neighboring triangles areas.

Figure 4-6 Interceptive Area

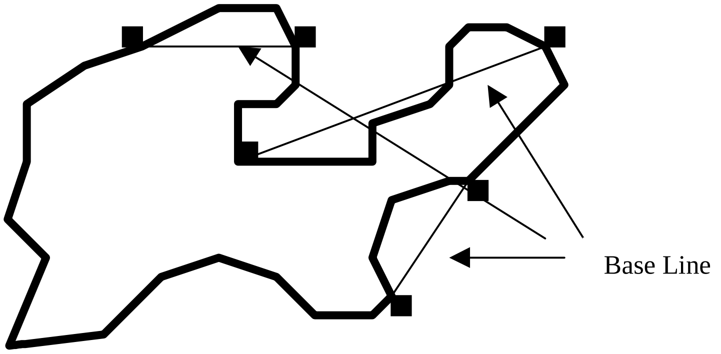

The shape-partitioning algorithm is multilevel. This procedure subsequently removes some points from the contour; the removed points become children nodes of the tree. On each iteration the procedure examines the triangles defined by all the pairs of the neighboring edges along the shape boundary and finds all LMIATs. After that all LMIATs whose areas are less than a reference value, which is the algorithm parameter, are removed. That actually means removing their middle points. If the user wants to get a precise representation, zero reference value could be passed. Other LMIATs are also removed, but the corresponding middle points are stored in the tree. After that another iteration is run. This process ends when the shape has been simplified to a quadrangle. The algorithm then determines a diagonal line that divides this quadrangle into two triangles in the most unbalanced way.

Thus the binary tree representation is constructed from the bottom to top levels. Every tree node is associated with one triangle. Except the root node, every node is connected to its parent node, and every node may have none, or single, or two child nodes. Each newly generated node becomes the parent of the nodes for which the two sides of the new node form the base line. The triangle that uses the left side of the parent triangle is the left child. The triangle that uses the right side of the parent triangle is the right child (See Figure 4-7).

Figure 4-7 Classification of Child Triangles

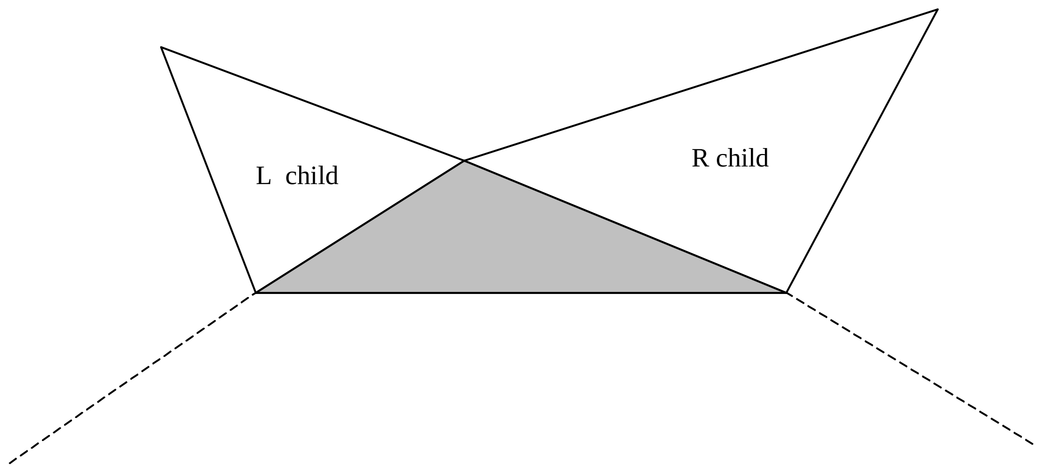

The root node is associated with the diagonal line of the quadrangle. This diagonal line divides the quadrangle into two triangles. The larger triangle is the left child and the smaller triangle is its right child.

For any tree node we record the following attributes:

- Coordinates x and y of the vertex P that do not lie on the base line of LMIAT, that is, coordinates of the middle (removed) point;
- Area of the triangle;
- Ratio of the height of the triangle h to the length of the base line a (Figure 4-8);
- Ratio of the projection of the left side of the triangle on the base line b to the length of the base line a;
- Signs "+" or "-"; the sign "+" indicates that the triangle lies outside of the new shape due to the 'cut' type merge; the sign "-" indicates that the triangle lies inside the new shape.

Figure 4-8 Triangles Properties

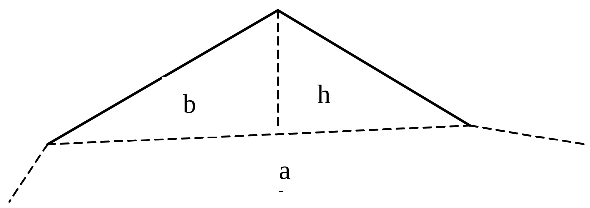

Figure 4-9 shows an example of the shape partitioning.

Figure 4-9 Shape Partitioning

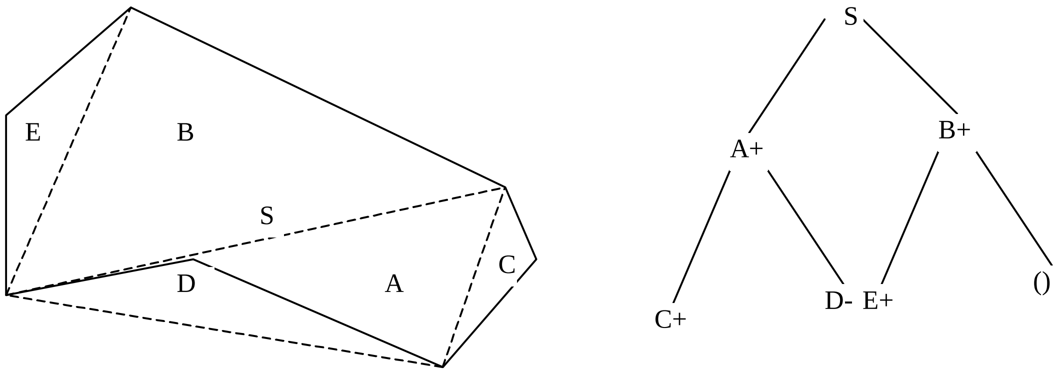

It is necessary to note that only the first attribute is sufficient for source contour reconstruction; all other attributes may be calculated from it. However, the other four attributes are very helpful for efficient contour matching.

The shape matching process that compares two shapes to determine whether they are similar or not can be effected by matching two corresponding tree representations, e.g., two trees can be compared from top to bottom, node by node, using the breadth-first traversing procedure.

Let us define the *corresponding node pair* (CNP) of two binary tree representations TA and TB. The corresponding node pair is called $[A(i), B(i)]$, if $A(i)$ and $B(i)$ are at the same level and same position in their respective trees.

The next step is defining the *node weight*. The weight of $N(i)$ denoted as $W[N(i)]$ is defined as the ratio of the size of $N(i)$ to the size of the entire shape.

Let $N(i)$ and $N(j)$ be two nodes with heights $h(i)$ and $h(j)$ and base lengths $a(i)$ and $a(j)$ respectively. The projections of their left sides on their base lines are $b(i)$ and $b(j)$ respectively. The node distance $dn[N(i), N(j)]$ between $N(i)$ and $N(j)$ is defined as:

$$dn[N(i), N(j)] = |h(i)/a(i) \cdot W[N(i)] \mp h(j)/a(j) \cdot W[N(j)]| \\ + |b(i)/a(i) \cdot W[N(i)] \mp b(j)/a(j) \cdot W[N(j)]|$$

In the above equation, the “+” signs are used when the signs of attributes in two nodes are different and the “-” signs are used when the two nodes have the same sign.

For two trees TA and TB representing two shapes SA and SB and with the corresponding node pairs $[A(1), B(1)],[A(2), B(2)],\ldots, [A(n), B(n)]$ the tree distance $dt(TA,TB)$between TA and TB is defined as:

$$dt(TA, TB) = \sum_{i=1}^{K} dn[A(i), B(i)].$$

If the two trees are different in size, the smaller tree is enlarged with trivial nodes so that the two trees can be fully compared. A trivial node is a node whose size attribute is zero. Thus, the trivial node weight is also zero. The values of other node attributes are trivial and not used in matching. The sum of the node distances of the first k CNPs of TA and TB is called the cumulative tree distance $dt(TA,TB,k)$ and is defined as:

$$dc(TA, TB, k) = \sum_{i=1}^{K} dn[A(i), B(i)].$$

Cumulative tree distance shows the dissimilarity between the approximations of the two shapes and exhibits the multiresolution nature of the tree representation in shape matching.

The shape matching algorithm is quite straightforward. For two given tree representations the two trees are traversed according to the breadth-first sequence to find CNPs of the two trees. Next `dn[A(i),B(i)]` and `dc(TA,TB,i)`are calculated for every `i`. If for some `i` `dc(TA,TB,i)`is larger than the tolerance threshold value, the matching procedure is terminated to indicate that the two shapes are dissimilar, otherwise it continues. If `dt(TA,TB)` is still less than the tolerance threshold value, then the procedure is terminated to indicate that there is a good match between `TA` and `TB`.

Geometry

This section describes functions from computational geometry field.

Ellipse Fitting

Fitting of primitive models to the image data is a basic task in pattern recognition and computer vision. A successful solution of this task results in reduction and simplification of the data for the benefit of higher level processing stages. One of the most commonly used models is the ellipse which, being a perspective projection of the circle, is of great importance for many industrial applications.

The representation of general conic by the second order polynomial is $F(\vec{a}, \vec{x}) = \vec{a}^T, \vec{x} = ax^2 + bxy + cy^2 + dx + ey + f = 0$ with the vectors denoted as $\vec{a} = [a, b, c, d, e, f]^T$ and $\vec{x} = [x^2, xy, y^2, x, y, 1]^T$.

$F(\vec{a}, \vec{x})$ is called the “algebraic distance between point (x_0, y_0) and conic $F(a, x)$“.

Minimizing the sum of squared algebraic distances $\sum_{i=1}^{''} F(\vec{x_0})^2$ may approach the fitting of conic.

In order to achieve ellipse-specific fitting polynomial coefficients must be constrained. For ellipse they must satisfy $b^2 - 4ac < 0$.

Moreover, the equality constraint $4ac - b^2 = 1$ can be imposed in order to incorporate coefficients scaling into constraint.

This constraint may be written as a matrix $\vec{a}^T C\vec{a} = 1$.

Finally, the problem could be formulated as minimizing $\|D\vec{a}\|^2$ with constraint $\vec{a}^T C\vec{a} = 1$, where D is the $n\times 6$ matrix $[\vec{x}_1, \vec{x}_2, \ldots, \vec{x}_n]^T$.

Introducing the Lagrange multiplier results in the system

$$2D^T D\vec{a} - 2\lambda C\vec{a} = 0 \quad \text{, which can be re-written as}$$
$$\vec{a}^T C\vec{a} = 1$$

$$S\vec{a} = 2\lambda C\vec{a}$$
$$\vec{a}^T C\vec{a} = 1,$$

The system solution is described in [Fitzgibbon95].

After the system is solved, ellipse center and axis can be extracted.

Line Fitting

M-estimators are used for approximating a set of points with geometrical primitives e.g., conic section, in cases when the classical least squares method fails. For example, the image of a line from the camera contains noisy data with many outliers, that is, the points that lie far from the main group, and the least squares method fails if applied.

The least squares method searches for a parameter set that minimizes the sum of squared distances:

$$m = \sum_i d_i^2,$$

where d_i is the distance from the ith point to the primitive. The distance type is specified as the function input parameter. If even a few points have a large d_i, then the perturbation in the primitive parameter values may be prohibitively big. The solution is to minimize

$$m = \sum_i \rho(d_i),$$

where $\rho(d_i)$ grows slower than d_i^2. This problem can be reduced to *weighted least squares* [Fitzgibbon95], which is solved by iterative finding of the minimum of

$$m_k = \sum_i W(d_i^{k-1}) d_i^2,$$

where k is the iteration number, d_i^{k-1} is the minimizer of the sum on the previous iteration, and $W(x) = \frac{1}{x}\frac{d\rho}{dx}$. If d_i is a linear function of parameters $p_j - d_i = \sum_j A_{ij} p_j$ then the minimization vector of the m_k is the eigenvector of $A^{T*}A$ matrix that corresponds to the smallest eigenvalue.

For more information see [Zhang96].

Convexity Defects

Let $(p_1, p_2, \ldots p_n)$ be a closed simple polygon, or contour, and $(h_1, h_2, \ldots h_m)$ a convex hull. A sequence of contour points exists normally between two consecutive convex hull vertices. This sequence forms the so-called convexity defect for which some useful characteristics can be computed. Computer Vision Library computes only one such characteristic, named “depth” (see Figure 4-10).

Figure 4-10 Convexity Defects

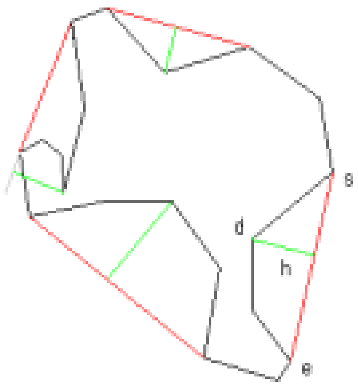

The black lines belong to the input contour. The red lines update the contour to its convex hull.

The symbols “*s*” and “*e*” signify the start and the end points of the convexity defect. The symbol “*d*” is a contour point located between “*s*” and “*e*” being the farthermost from the line that includes the segment “*se*”. The symbol “*h*” stands for the convexity defect depth, that is, the distance from “*d*” to the “*se*” line.

See `CvConvexityDefect` structure definition in Structural Analysis Reference.

Object Recognition 5

Eigen Objects

This section describes functions that operate on eigen objects.

Let us define an object $u = \{u_1, u_2 ..., u_n\}$ as a vector in the n-dimensional space. For example, u can be an image and its components u_l are the image pixel values. In this case n is equal to the number of pixels in the image. Then, consider a group of input objects $u^i = \{u^i_1, u^i_2, ..., u^i_n\}$, where $i = 1, ..., m$ and usually $m << n$. The averaged, or mean, object $\bar{u} = \{\bar{u}_1, \bar{u}_2, ..., \bar{u}_n\}$ of this group is defined as follows:

$$\bar{u}_l = \frac{1}{m} \sum_{k=1}^{m} u^k_l .$$

Covariance matrix $C = |c_{ij}|$ is a square symmetric matrix $m \times m$:

$$c_{ij} = \sum_{l=1}^{n} (u^i_l - \bar{u}_l) \cdot (u^j_l - \bar{u}_l) .$$

Eigen objects basis $e^i = \{e^i_1, e^i_2, ..., e^i_n\}$, $i = 1, ..., m_1 \le m$ of the input objects group may be calculated using the following relation:

$$e^i_l = \frac{1}{\sqrt{\lambda_i}} \sum_{k=1}^{m} v^i_k \cdot (u^k_l - \bar{u}_l) ,$$

where λ_i and $v^i = \{v^i_1, v^i_2, ..., v^i_m\}$ are eigenvalues and the corresponding eigenvectors of matrix C.

Any input object u^i as well as any other object u may be decomposed in the eigen objects m_1-D sub-space. Decomposition coefficients of the object u are:

$$w_i = \sum_{l=1}^{n} e_l^i \cdot (u_l - \bar{u}_l) .$$

Using these coefficients, we may calculate projection $\tilde{u} = \{\tilde{u}_1, \tilde{u}_2 \ldots, \tilde{u}_n\}$ of the object u to the eigen objects sub-space, or, in other words, restore the object u in that sub-space:

$$\tilde{u}_l = \sum_{k=1}^{m_1} w_k e_l^k + \bar{u}_l .$$

For examples of use of the functions and relevant data types see Image Recognition Reference Chapter.

Embedded Hidden Markov Models

This section describes functions for using Embedded Hidden Markov Models (HMM) in face recognition task. See Reference for HMM Structures.

3D Reconstruction

6

Camera Calibration

This section describes camera calibration and undistortion functions.

Camera Parameters

Camera calibration functions are used for calculating intrinsic and extrinsic camera parameters.

Camera parameters are the numbers describing a particular camera configuration.

The *intrinsic* camera parameters specify the camera characteristics proper; these parameters are:

- focal length, that is, the distance between the camera lens and the image plane,
- location of the image center in pixel coordinates,
- effective pixel size,
- radial distortion coefficient of the lens.

The *extrinsic* camera parameters describe spatial relationship between the camera and the world; they are

- rotation matrix,
- translation vector.

They specify the transformation between the camera and world reference frames.

A usual pinhole camera is used. The relationship between a *3D* point M and its image projection m is given by the formula

$$m = A[Rt]M,$$

where A is the camera intrinsic matrix:

$$A = \begin{bmatrix} f_x & 0 & c_x \\ 0 & f_y & c_y \\ 0 & 0 & 1 \end{bmatrix}$$,where

(c_x, c_y) are coordinates of the principal point;

(f_x, f_y) are the focal lengths by the axes x and y;

(R, t) are extrinsic parameters: the rotation matrix R and translation vector t that relate the world coordinate system to the camera coordinate system:

$$R = \begin{bmatrix} r_{11} & r_{12} & r_{13} \\ r_{21} & r_{22} & r_{23} \\ r_{31} & r_{32} & r_{33} \end{bmatrix}, \quad t = \begin{bmatrix} t_1 \\ t_2 \\ t_3 \end{bmatrix}.$$

Camera usually exhibits significant lens distortion, especially radial distortion. The distortion is characterized by four coefficients: k_1, k_2, p_1, p_2. The functions UnDistortOnce and UnDistortInit + UnDistort correct the image from the camera given the four coefficients:

$$\tilde{x} = x + x[k_1 r^2 + k_2 r^4] + [2p_1 xy + p_2(r^2 + 2x^2)]$$
$$\tilde{y} = y + y[k_1 r^2 + k_2 r^4] + [2p_2 xy + p_2(r^2 + 2y^2)],$$

where x, y are ideal, that is, distortion-free image physical coordinates and $\tilde{x}$, $\tilde{y}$ are real, that is, distorted image physical coordinates, while $r^2 = x^2 + y^2$.

The following algorithm, described in [Zhang99] and [Zhang00], was used for camera calibration:

1. Find homography for all points on series of images, where homography is a matrix of perspective transform between calibration pattern plane and camera view plane.
2. Initialize intrinsic parameters; distortion is set to 0.
3. Find extrinsic parameters for each image of pattern.

4. Make main optimization by minimizing error of projection points with all parameters.

Pattern

To calibrate the camera, the calibration routine is supplied with several views of a planar model object, or pattern, of known geometry. For every view the points on the model plane and their projections onto the image are passed to the calibration routine. In OpenCV a chessboard pattern is used (see Figure 6-1). To achieve more accurate calibration results, print out the pattern at high resolution on high-quality paper and put it on a hard, preferably glass, substrate.

Figure 6-1 Pattern

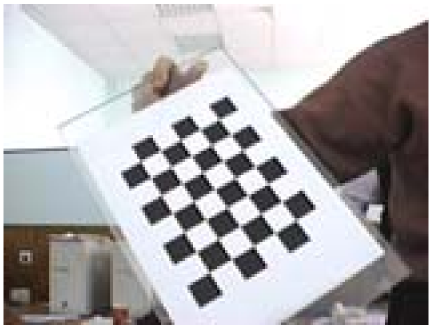

View Morphing

This section describes functions for morphing views from two cameras.

The View Morphing technique is used to get an image from a virtual camera that could be placed between two real cameras. The input for View Morphing algorithms are two images from real cameras and information about correspondence between regions in the two images. The output of the algorithms is a synthesized image - "a view from the virtual camera".

This section addresses the problem of synthesizing images of real scenes under three-dimensional transformation in viewpoint and appearance. Solving this problem enables interactive viewing of remote scenes on a computer, in which a user can move the virtual camera through the environment. A three-dimensional scene transformation can be rendered on a video display device through applying simple transformation to a set of basis images of the scene. The virtue of these transformations is that they operate directly on the image and recover only the scene information that is required to accomplish the desired effect. Consequently, the transformations are applicable in a situation when accurate three-dimensional models are difficult or impossible to obtain.

The algorithm for synthesis of a virtual camera view from a pair of images taken from real cameras is shown below.

Algorithm

1. Find fundamental matrix, for example, using correspondence points in the images.
2. Find scanlines for each image.
3. Warp the images across the scanlines.
4. Find correspondence of the warped images.
5. Morph the warped images across position of the virtual camera.
6. Unwarp the image.

7. Delete moire from the resulting image.

Figure 6-2 Original Images

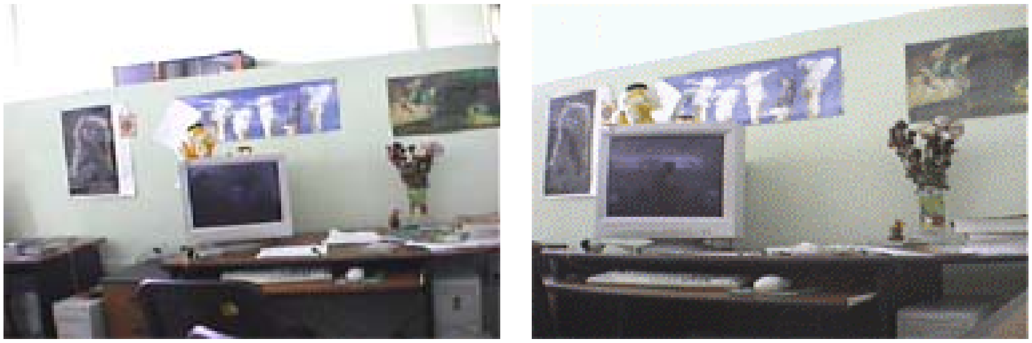

Original Image From Left Camera Original Image From Right Camera

Figure 6-3 Correspondence Points

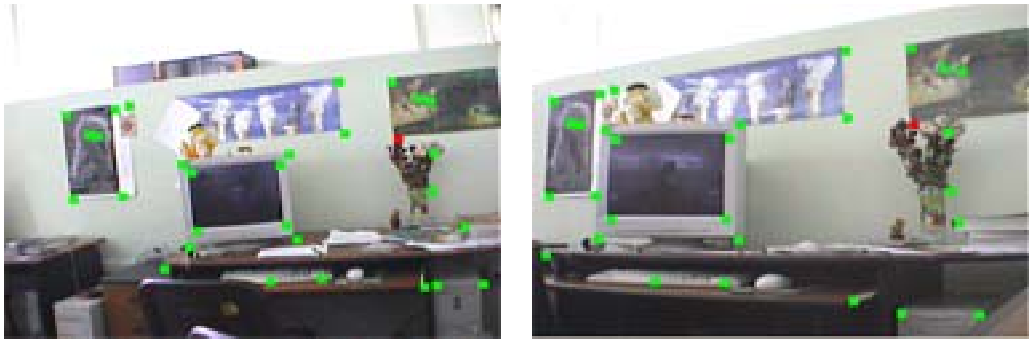

Correspondence Points on Left Image Correspondence Points on Right Image

Figure 6-4 Scan Lines

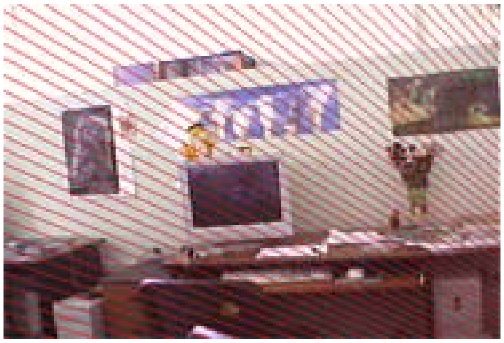

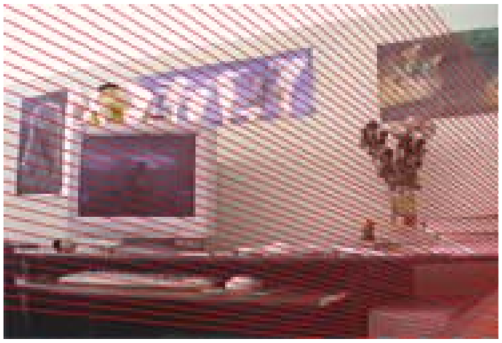

Some Scanlines on Left Iimage Some Scanlines on Right Image

Figure 6-5 Moire in Morphed Image

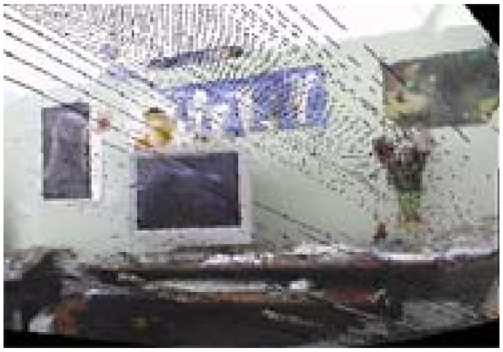

Figure 6-6 Resulting Morphed Image

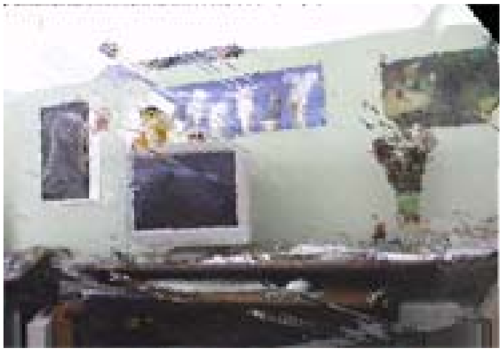

Morphed Image From Virtual Camera With Deleted Moire

Using Functions for View Morphing Algorithm

1. Find the fundamental matrix using the correspondence points in the two images of cameras by calling the function `FindFundamentalMatrix`.
2. Find the number of scanlines in the images for the given fundamental matrix by calling the function `FindFundamentalMatrix` with null pointers to the scanlines.
3. Allocate enough memory for:

— scanlines in the first image, scanlines in the second image, scanlines in the virtual image (for each *numscan*2*4*sizeof(int)*);

— lengths of scanlines in the first image, lengths of scanlines in the second image, lengths of scanlines in the virtual image (for each *numscan*2*4*sizeof(int)*);

— buffer for the prewarp first image, the second image, the virtual image (for each *width*height*2*sizeof(int)*);

— data runs for the first image and the second image (for each *width*height*4*sizeof(int)*);

— correspondence data for the first image and the second image (for each *width*height*2*sizeof(int)*);

— numbers of lines for the first and second images (for each `width*height*4*sizeof(int)`).

4. Find scanlines coordinates by calling the function `FindFundamentalMatrix`.
5. Prewarp the first and second images using scanlines data by calling the function `PreWarpImage`.
6. Find runs on the first and second images scanlines by calling the function `FindRuns`.
7. Find correspondence information by calling the function `DynamicCorrespondMulti`.
8. Find coordinates of scanlines in the virtual image for the virtual camera position `alpha` by calling the function `MakeAlphaScanlines`.
9. Morph the prewarp virtual image from the first and second images using correspondence information by calling the function `MorphEpilinesMulti`.
10. Postwarp the virtual image by calling the function `PostWarpImage`.
11. Delete moire from the resulting virtual image by calling the function `DeleteMoire`.

POSIT

This section describes functions that together perform POSIT algorithm.

The POSIT algorithm determines the six degree-of-freedom pose of a known tracked *3D* rigid object. Given the projected image coordinates of uniquely identified points on the object, the algorithm refines an initial pose estimate by iterating with a weak perspective camera model to construct new image points; the algorithm terminates when it reaches a converged image, the pose of which is the solution.

Geometric Image Formation

The link between world points and their corresponding image points is the projection from world space to image space. Figure 6-8 depicts the *perspective* (or *pinhole*) model, which is the most common projection model because of its generality and usefulness.

The points in the world are projected onto the image plane according to their distance from the center of projection. Using similar triangles, the relationship between the coordinates of an image point $p_i = (x_i, y_i)$ and its world point $P_i = (X_i, Y_i, Z_i)$ can be determined as

$$x_i = \frac{f}{Z_i}X_i,\ y_i = \frac{f}{Z_i}Y_i. \quad (6.1)$$

Figure 6-7 Perspective Geometry Projection

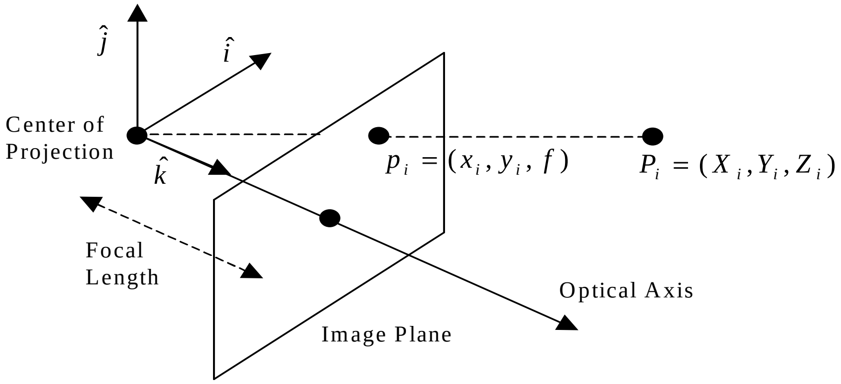

The *weak-perspective* projection model simplifies the projection equation by replacing all Z_i with a representative Z so that $s = f/Z$ is a constant scale for all points. The projection equations are then

$$x_i = sX_i,\ y_i = sY_i. \quad (6.2)$$

Because this situation can be modelled as an orthographic projection ($x_i = X_i$, $y_i = Y_i$) followed by isotropic scaling, weak-perspective projection is sometimes called *scaled orthographic projection*. Weak-perspective is a valid assumption only when the distances between any Z_i are much smaller than the distance between the Z_i

and the center of projection; in other words, the world points are clustered and far enough from the camera. Z can be set either to any Z_i or to the average computed over all Z_i.

More detailed explanations of this material can be found in [Trucco98].

Pose Approximation Method

Using weak-perspective projection, a method for determining approximate pose, termed *Pose from Orthography and Scaling* (POS) in [DeMenthon92], can be derived. First, a reference point P_0 in the world is chosen from which all other world points can be described as vectors: $\vec{P} = P_i - P_0$ (see Figure 6-9).

Figure 6-8 Scaling of Vectors in Weak-Perspective Projection

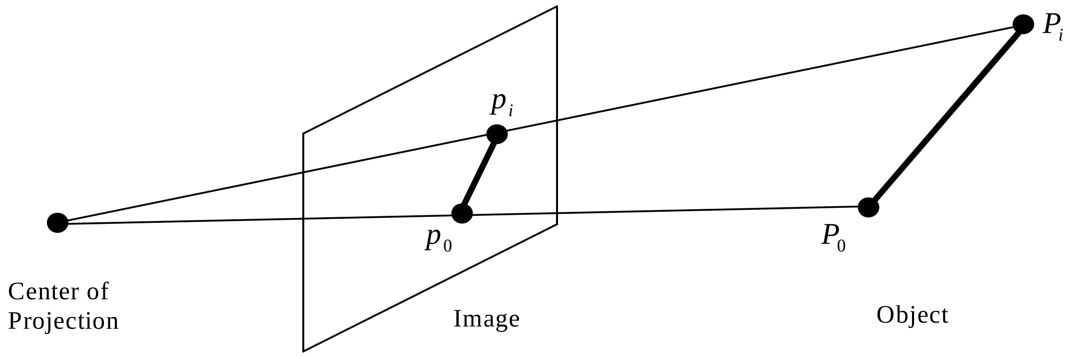

Similarly, the projection of this point, namely p_0, is a reference point for the image points: $\vec{p}_i = p_i - p_0$. As follows from the weak-perspective assumption, the x component of $\vec{p}_i$ is a scaled-down form of the x component of $\vec{P}_i$:

$$x_i - x_0 = s(X_i - X_0) = s(\vec{P}_0 \cdot \hat{i}). \quad (6.3)$$

This is also true for their y components. If I and J are defined as scaled-up versions of the unit vectors $\hat{\imath}$ and $\hat{\jmath}$ ($I = s\hat{\imath}$ and $J = s\hat{\jmath}$), then

$$x_i - x_0 = \vec{P}_i \cdot I \text{ and } y_i - y_0 = \vec{P}_i \cdot J \quad (6.4)$$

as two equations for each point for which I and J are unknown. These equations, collected over all the points, can be put into matrix form as

$$\underline{x} = MI \text{ and } \underline{y} = MJ, \quad (6.5)$$

where $\underline{x}$ and $\underline{y}$ are vectors of x and y components of $\vec{p}_i$ respectively, and M is a matrix whose rows are the $\vec{P}_i$ vectors. These two sets of equations can be further joined to construct a single set of linear equations:

$$[\underline{x}\ \ \underline{y}] = M[I\ J] \Rightarrow \underline{\vec{p}}_i C = M[I\ J], \quad (6.6)$$

where $\underline{\vec{p}}_i$ is a matrix whose rows are $\vec{p}_i$. The latter equation is an overconstrained system of linear equations that can be solved for I and J in a least-squares sense as

$$[I\ J] = M^+ \underline{\vec{p}}_i, \quad (6.7)$$

where M^+ is the pseudo-inverse of M.

Now that we have I and J, we construct the pose estimate as follows. First, $\hat{\imath}$ and $\hat{\jmath}$ are estimated as I and J normalized, that is, scaled to unit length. By construction, these are the first two rows of the rotation matrix, and their cross-product is the third row:

$$R = \begin{bmatrix} \hat{\imath}^T \\ \hat{\jmath}^T \\ (\hat{\imath} \times \hat{\jmath})^T \end{bmatrix}. \quad (6.8)$$

The average of the magnitudes of I and J is an estimate of the weak-perspective scale s. From the weak-perspective equations, the world point P_0 in camera coordinates is the image point p_0 in camera coordinates scaled by s:

$$P_0 = p_0/s = [x_0\ y_0\ f]/s, \quad (6.9)$$

which is precisely the translation vector being sought.

Algorithm

The POSIT algorithm was first presented in the paper by DeMenthon and Davis [DeMenthon92]. In this paper, the authors first describe their POS (Pose from Orthography and Scaling) algorithm. By approximating perspective projection with weak-perspective projection POS produces a pose estimate from a given image. POS can be repeatedly used by constructing a new weak perspective image from each pose estimate and feeding it into the next iteration. The calculated images are estimates of the initial perspective image with successively smaller amounts of "perspective distortion" so that the final image contains no such distortion. The authors term this iterative use of POS as POSIT (POS with ITerations).

POSIT requires three pieces of known information:

- The *object model,* consisting of *N* points, each with unique *3D* coordinates. *N* must be greater than 3, and the points must be non-degenerate (non-coplanar) to avoid algorithmic difficulties. Better results are achieved by using more points and by choosing points as far from coplanarity as possible. The object model is an *N* x 3 matrix.
- The *object image,* which is the set of *2D* points resulting from a camera projection of the model points onto an image plane; it is a function of the object current pose. The object image is an *N* x 2 matrix.
- The camera intrinsic parameters, namely, the *focal length* of the camera.

Given the object model and the object image, the algorithm proceeds as follows:

1. The object image is assumed to be a weak perspective image of the object, from which a least-squares pose approximation is calculated via the object model pseudoinverse.
2. From this approximate pose the object model is projected onto the image plane to construct a new weak perspective image.
3. From this image a new approximate pose is found using least-squares, which in turn determines another weak perspective image, and so on.

For well-behaved inputs, this procedure converges to an unchanging weak perspective image, whose corresponding pose is the final calculated object pose.

Example 6-1 POSIT Algorithm in Pseudo-Code

```
 POSIT (imagePoints, objectPoints, focalLength) {
    count = converged = 0;
    modelVectors = modelPoints – modelPoints(0);
    oldWeakImagePoints = imagePoints;
    while (!converged) {
      if (count == 0)
        imageVectors = imagePoints – imagePoints(0);
      else {
        weakImagePoints = imagePoints .*
                           ((1 + modelVectors*row3/translation(3)) * [1
1]);
        imageDifference = sum(sum(abs( round(weakImagePoints) –
                                       round(oldWeakImagePoints))));
        oldWeakImagePoints = weakImagePoints;
        imageVectors = weakImagePoints – weakImagePoints(0);
      }
      [I J] = pseudoinverse(modelVectors) * imageVectors;
      row1 = I / norm(I);
      row2 = J / norm(J);
      row3 = crossproduct(row1, row2);
      rotation = [row1; row2; row3];
      scale = (norm(I) + norm(J)) / 2;
     translation = [imagePoints(1,1); imagePoints(1,2); focalLength] /
                   scale;
      converged = (count > 0) && (diff < 1);
      count = count + 1;
    }
    return {rotation, translation};
  }
```

As the first step assumes, the object image is a weak perspective image of the object. It is a valid assumption only for an object that is far enough from the camera so that “perspective distortions” are insignificant. For such objects the correct pose is recovered immediately and convergence occurs at the second iteration. For less ideal situations, the pose is quickly recovered after several iterations. However, convergence is not guaranteed when perspective distortions are significant, for example, when an object is close to the camera with pronounced foreshortening. DeMenthon and Davis state that “convergence seems to be guaranteed if the image features are at a distance from the image center shorter than the focal length.”[DeMenthon92] Fortunately, this occurs for most realistic camera and object configurations.

Gesture Recognition

This section describes specific functions for the static gesture recognition technology.

The gesture recognition algorithm can be divided into four main components as illustrated in Figure 6-10.

The first component computes the *3D* arm pose from range image data that may be obtained from the standard stereo correspondence algorithm. The process includes *3D* line fitting, finding the arm position along the line and creating the arm mask image.

Figure 6-9 Gesture Recognition Algorithm

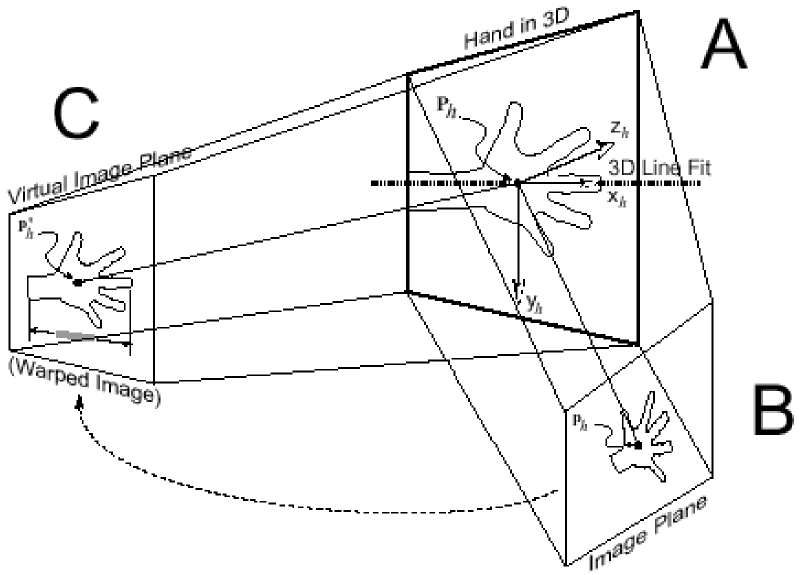

The second component produces a frontal view of the arm image and arm mask through a planar homograph transformation. The process consists of the homograph matrix calculation and warping image and image mask (See Figure 6-11).

Figure 6-10 Arm Location and Image Warping

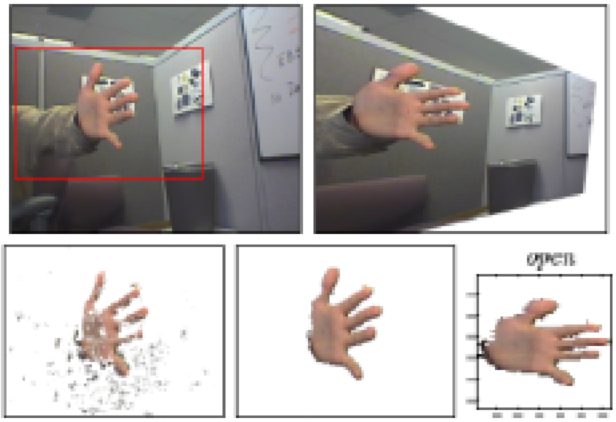

The third component segments the arm from the background based on the probability density estimate that a pixel with a given hue and saturation value belongs to the arm. For this *2D* image histogram, image mask histogram, and probability density histogram are calculated. Following that, initial estimate is iteratively refined using the maximum likelihood approach and morphology operations (See Figure 6-12)

Figure 6-11 Arm Segmentation by Probability Density Estimation

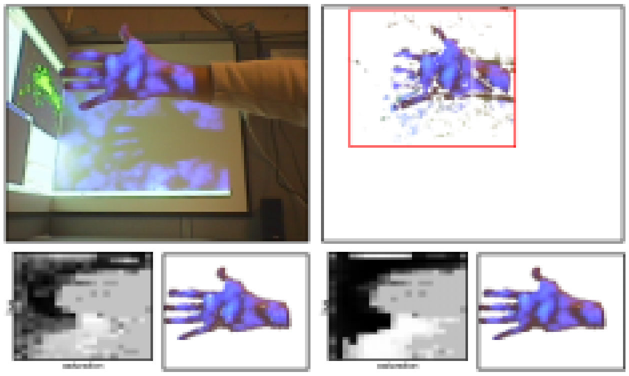

The fourth step is the recognition step when normalized central moments or seven Hu moments are calculated using the resulting image mask. These invariants are used to match masks by the Mahalanobis distance metric calculation.

The functions operate with specific data of several types. Range image data is a set of *3D* points in the world coordinate system calculated via the stereo correspondence algorithm. The second data type is a set of the original image indices of this set of *3D* points, that is, projections on the image plane. The functions of this group

- enable the user to locate the arm region in a set of *3D* points (the functions `FindHandRegion` and `FindHandRegionA`),
- create an image mask from a subset of *3D* points and associated subset indices around the arm center (the function `CreateHandMask`),
- calculate the homography matrix for the initial image transformation from the image plane to the plane defined by the frontal arm plane (the function `CalcImageHomography`),
- calculate the probability density histogram for the arm location (the function `CalcProbDensity`).

Basic Structures and Operations

7

Image Functions

This section describes basic functions for manipulating raster images.

OpenCV library represents images in the format `IplImage` that comes from Intel® Image Processing Library (IPL). IPL reference manual gives detailed information about the format, but, for completeness, it is also briefly described here.

Example 7-1 `IplImage` Structure Definition

```
    typedef struct _IplImage {
        int nSize; /* size of iplImage struct */
        int ID; /* image header version */
        int nChannels;
        int alphaChannel;
        int depth; /* pixel depth in bits */
        char colorModel[4];
        char channelSeq[4];
        int dataOrder;
        int origin;
        int align; /* 4- or 8-byte align */
        int width;
        int height;
        struct _IplROI *roi; /* pointer to ROI if any */
        struct _IplImage *maskROI; /*pointer to mask ROI if any */
        void *imageId; /* use of the application */
       struct _IplTileInfo *tileInfo; /* contains information on tiling
*/
        int imageSize; /* useful size in bytes */
        char *imageData; /* pointer to aligned image */
        int widthStep; /* size of aligned line in bytes */
        int BorderMode[4]; /* the top, bottom, left,
        and right border mode */
        int BorderConst[4]; /* constants for the top, bottom,
                                left, and right border */
        char *imageDataOrigin; /* ptr to full, nonaligned image */
    } IplImage;
```

Only a few of the most important fields of the structure are described here. The fields *width* and *height* contain image width and height in pixels, respectively. The field *depth* contains information about the type of pixel values.

All possible values of the field *depth* listed in *ipl.h* header file include:

IPL_DEPTH_8U - unsigned 8-bit integer value (unsigned *char*),

IPL_DEPTH_8S - signed 8-bit integer value (signed *char* or simply *char*),

IPL_DEPTH_16S - signed 16-bit integer value (short *int*),

IPL_DEPTH_32S - signed 32-bit integer value (*int*),

IPL_DEPTH_32F - 32-bit floating-point single-precision value (*float*).

In the above list the corresponding types in *C* are placed in parentheses. The parameter *nChannels* means the number of color planes in the image. Grayscale images contain a single channel, while color images usually include three or four channels. The parameter *origin* indicates, whether the top image row (*origin* == IPL_ORIGIN_TL) or bottom image row (*origin* == IPL_ORIGIN_BL) goes first in memory. Windows bitmaps are usually bottom-origin, while in most of other environments images are top-origin. The parameter *dataOrder* indicates, whether the color planes in the color image are interleaved (*dataOrder* == IPL_DATA_ORDER_PIXEL) or separate (*dataOrder* == IPL_DATA_ORDER_PLANE). The parameter *widthStep* contains the number of bytes between points in the same column and successive rows. The parameter *width* is not sufficient to calculate the distance, because each row may be aligned with a certain number of bytes to achieve faster processing of the image, so there can be some gaps between the end of ith row and the start of $(i+1)^{th}$ row. The parameter *imageData* contains pointer to the first row of image data. If there are several separate planes in the image (when *dataOrder* == IPL_DATA_ORDER_PLANE), they are placed consecutively as separate images with *height*nChannels* rows total.

It is possible to select some rectangular part of the image or a certain color plane in the image, or both, and process only this part. The selected rectangle is called "Region of Interest" or ROI. The structure `IplImage` contains the field *`roi`* for this purpose. If the pointer not `NULL`, it points to the structure `IplROI` that contains parameters of selected ROI, otherwise a whole image is considered selected.

Example 7-2 `IplROI` Structure Definition

```
typedef struct _IplROI {
       int  coi;     /* channel of interest or COI */
       int  xOffset;
       int  yOffset;
       int  width;
       int  height;
   } IplROI;
```

As can be seen, `IplROI` includes ROI origin and size as well as COI (“Channel of Interest”) specification. The field `coi`, equal to 0, means that all the image channels are selected, otherwise it specifies an index of the selected image plane.

Unlike IPL, OpenCV has several limitations in support of `IplImage`:

- Each function supports only a few certain depths and/or number of channels. For example, image statistics functions support only single-channel or three-channel images of the depth `IPL_DEPTH_8U`, `IPL_DEPTH_8S` or `IPL_DEPTH_32F`. The exact information about supported image formats is usually contained in the description of parameters or in the beginning of the chapter if all the functions described in the chapter are similar. It is quite different from IPL that tries to support all possible image formats in each function.
- OpenCV supports only interleaved images, not planar ones.
- The fields *`colorModel`*, *`channelSeq`*, *`BorderMode`*, and *`BorderConst`* are ignored.
- The field *`align`* is ignored and *`widthStep`* is simply used instead of recalculating it using the fields *`width`* and *`align`*.
- The fields *`maskROI`* and *`tileInfo`* must be zero.
- COI support is very limited. Now only image statistics functions accept non-zero COI values. Use the functions `CvtPixToPlane` and `CvtPlaneToPix` as a work-around.

— ROIs of all the input/output images have to match exactly one another. For example, input and output images of the function `Erode` must have ROIs with equal sizes. It is unlike IPL again, where the ROIs intersection is actually affected.

Despite all the limitations, OpenCV still supports most of the commonly used image formats that can be supported by `IplImage` and, thus, can be successfully used with IPL on common subset of possible `IplImage` formats.

The functions described in this chapter are mainly short-cuts for operations of creating, destroying, and other common operations on `IplImage`, and they are often implemented as wrappers for original IPL functions.

Dynamic Data Structures

This chapter describes several resizable data structures and basic functions that are designed to operate on these structures.

Memory Storage

Memory storages provide the space for storing all the dynamic data structures described in this chapter. A storage consists of a header and a double-linked list of memory blocks. This list is treated as a stack, that is, the storage header contains a pointer to the block that is not occupied entirely and an integer value, the number of free bytes in this block. When the free space in the block has run out, the pointer is moved to the next block, if any, otherwise, a new block is allocated and then added to the list of blocks. All the blocks are of the same size and, therefore, this technique ensures an accurate memory allocation and helps avoid memory fragmentation if the blocks are large enough (see Figure 7-1).

Figure 7-1 Memory Storage Organization

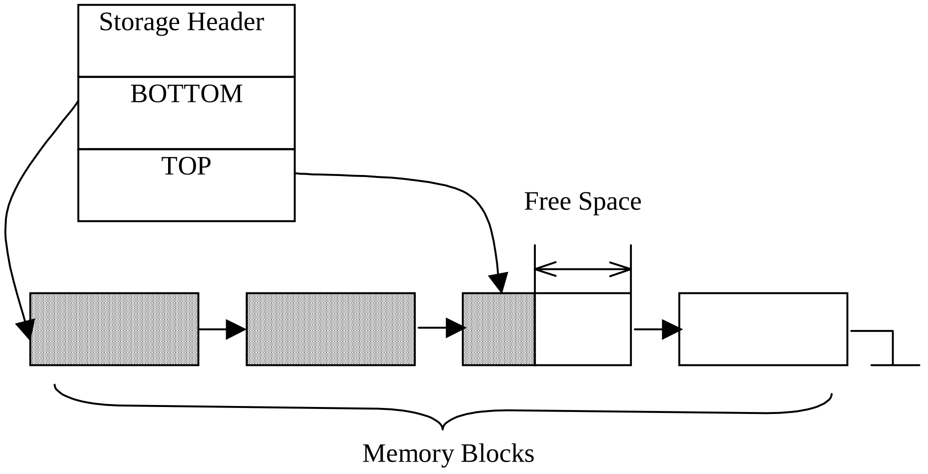

Sequences

A sequence is a resizable array of arbitrary type elements located in the memory storage. The sequence is discontinuous. Sequence data may be partitioned into several continuous blocks, called sequence blocks, that can be located in different memory blocks. Sequence blocks are connected into a circular double-linked list to store large sequences in several memory blocks or keep several small sequences in a single memory block. For example, such organization is suitable for storing contours. The sequence implementation provides fast functions for adding/removing elements to/from the head and tail of the sequence, so that the sequence implements a deque. The functions for inserting/removing elements in the middle of a sequence are also available but they are slower. The sequence is the basic type for many other dynamic data structures in the library, e.g., sets, graphs, and contours; just like all these types, the sequence never returns the occupied memory to the storage. However, the sequence keeps track of the memory released after removing elements from the

sequence; this memory is used repeatedly. To return the memory to the storage, the user may clear a whole storage, or use save/restoring position functions, or keep temporary data in child storages.

Figure 7-2 Sequence Structure

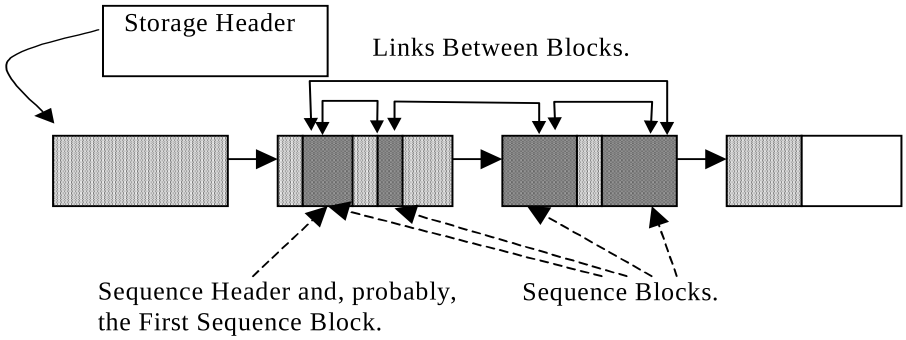

Writing and Reading Sequences

Although the functions and macros described below are irrelevant in theory because functions like `SeqPush` and `GetSeqElem` enable the user to write to sequences and read from them, the writing/reading functions and macros are very useful in practice because of their speed.

The following problem could provide an illustrative example. If the task is to create a function that forms a sequence from `N` random values, the PUSH version runs as follows:

```
CvSeq* create_seq1( CvStorage* storage, int N ) {
CvSeq* seq = cvCreateSeq( 0, sizeof(*seq), sizeof(int), storage);
for( int i = 0; i < N; i++ ) {
int a = rand();
cvSeqPush( seq, &a );
}
return seq;
```

```
}
```

The second version makes use of the fast writing scheme, that includes the following steps: initialization of the writing process (creating writer), writing, closing the writer (flush).

```
CvSeq* create_seq1( CvStorage* storage, int N ) {
CvSeqWriter writer;
cvStartWriteSeq( 0, sizeof(*seq), sizeof(int),
storage, &writer );
for( int i = 0; i < N; i++ ) {
int a = rand();
CV_WRITE_SEQ_ELEM( a, writer );
}
return cvEndWriteSeq( &writer );
}
```

If `N` = 100000 and 500 MHz Pentium® III processor is used, the first version takes 230 milliseconds and the second one takes 111 milliseconds to finish. These characteristics assume that the storage already contains a sufficient number of blocks so that no new blocks are allocated. A comparison with the simple loop that does not use sequences gives an idea as to how effective and efficient this approach is.

```
int* create_seq3( int* buffer, int N ) {
for( i = 0; i < N; i++ ) {
buffer[i] = rand();
}
return buffer;
}
```

This function takes 104 milliseconds to finish using the same machine.

Generally, the sequences do not make a great impact on the performance and the difference is very insignificant (less than 7% in the above example). However, the advantage of sequences is that the user can operate the input or output data even without knowing their amount in advance. These structures enable him/her to allocate memory iteratively. Another problem solution would be to use lists, yet the sequences are much faster and require less memory.

Sets

The set structure is mostly based on sequences but has a totally different purpose. For example, the user is unable to use sequences for location of the dynamic structure elements that have links between one another because if some elements have been removed from the middle of the sequence, other sequence elements are moved to another location and their addresses and indices change. In this case all links have to be fixed anew. Another aspect of this problem is that removing elements from the middle of the sequence is slow, with time complexity of $O(n)$, where n is the number of elements in the sequence.

The problem solution lies in making the structure sparse and unordered, that is, whenever a structure element is removed, other elements must stay where they have been, while the cell previously occupied by the element is added to the pool of three cells; when a new element is inserted into the structure, the vacant cell is used to store this new element. The set operates in this way (See Example 7-3).

The set looks like a list yet keeps no links between the structure elements. However, the user is free to make and keep such lists, if needed. The set is implemented as a sequence subclass; the set uses sequence elements as cells and organizes a list of free cells.

See Figure 7-3 for an example of a set. For simplicity, the figure does not show division of the sequence/set into memory blocks and sequence blocks.

Figure 7-3 Set Structure

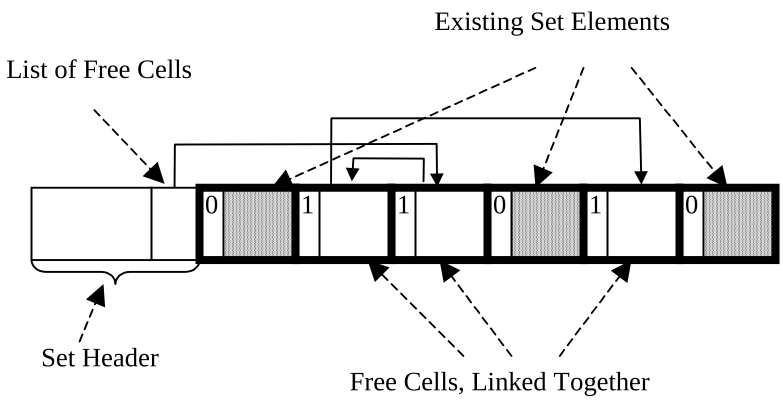

The set elements, both existing and free cells, are all sequence elements. A special bit indicates whether the set element exists or not: in the above diagram the bits marked by 1 are free cells and the ones marked by 0 are occupied cells. The macro `CV_IS_SET_ELEM_EXISTS(set_elem_ptr)` uses this special bit to return a non-zero value if the set element specified by the parameter `set_elem_ptr` belongs to the set, and 0 otherwise. Below follows the definition of the structure `CvSet`:

Example 7-3 `CvSet` Structure Definition

```
#define CV_SET_FIELDS()      \
    CV_SEQUENCE_FIELDS()     \
    CvMemBlock* free_elems;

typedef struct CvSet
{
    CV_SET_FIELDS()
}
CvSet;
```

In other words, a set is a sequence plus a list of free cells.

There are two modes of working with sets:

1. Using indices for referencing the set elements within a sequence
2. Using pointers for the same purpose.

Whereas at times the first mode is a better option, the pointer mode is faster because it does not need to find the set elements by their indices, which is done in the same way as in simple sequences. The decision on which method should be used in each particular case depends on:

- the type of operations to be performed on the set and
- the way the operations on the set should be performed.

The ways in which a new set is created and new elements are added to the existing set are the same in either mode, the only difference between the two being the way the elements are removed from the set. The user may even use both methods of access simultaneously, provided he or she has enough memory available to store both the index and the pointer to each element.

Like in sequences, the user may create a set with elements of arbitrary type and specify any size of the header subject to the following restrictions:

- size of the header may not be less than `sizeof(CvSet)`.
- size of the set elements should be divisible by 4 and not less than 8 bytes.

The reason behind the latter restriction is the internal set organization: if the set has a free cell available, the first 4-byte field of this set element is used as a pointer to the next free cell, which enables the user to keep track of all free cells. The second 4-byte field of the cell contains the cell to be returned when the cell becomes occupied.

When the user removes a set element while operating in the index mode, the index of the removed element is passed and stored in the released cell again. The bit indicating whether the element belongs to the set is the least significant bit of the first 4-byte field. This is the reason why all the elements must have their size divisible by 4. In this case they are all aligned with the 4-byte boundary, so that the least significant bits of their addresses are always 0.

In free cells the corresponding bit is set to 1 and, in order to get the real address of the next free cell, the functions mask this bit off. On the other hand, if the cell is occupied, the corresponding bit must be equal to 0, which is the second and last restriction: the

least significant bit of the first 4-byte field of the set element must be 0, otherwise the corresponding cell is considered free. If the set elements comply with this restriction, e.g., if the first field of the set element is a pointer to another set element or to some aligned structure outside the set, then the only restriction left is a non-zero number of 4- or 8-byte fields after the pointer. If the set elements do not comply with this restriction, e.g., if the user wants to store integers in the set, the user may derive his or her own structure from the structure *CvSetElem* or include it into his or her structure as the first field.

Example 7-4 `CvSetElem` Structure Definition

```
#define CV_SET_ELEM_FIELDS()    \
    int*  aligned_ptr;
typedef struct _CvSetElem
{
    CV_SET_ELEM_FIELDS()
}
CvSetElem;
```

The first field is a dummy field and is not used in the occupied cells, except the least significant bit, which is 0. With this structure the integer element could be defined as follows:

```
typedef struct _IntSetElem
{
    CV_SET_ELEM_FIELDS()
    int value;
}
IntSetElem;
```

Graphs

The structure set described above helps to build graphs because a graph consists of two sets, namely, vertices and edges, that refer to each other.

Example 7-5 `CvGraph` Structure Definition

```
#define  CV_GRAPH_FIELDS()   \
    CV_SET_FIELDS()          \
    CvSet* edges;
typedef struct _CvGraph
```

Example 7-5 `CvGraph` Structure Definition (continued)

```
{
    CV_GRAPH_FIELDS()
}
CvGraph;
```

In OOP terms, the graph structure is derived from the set of vertices and includes a set of edges. Besides, special data types exist for graph vertices and graph edges.

Example 7-6 Definitions of `CvGraphEdge` and `CvGraphVtx` Structures

```
#define CV_GRAPH_EDGE_FIELDS()    \
    struct _CvGraphEdge* next[2]; \
    struct _CvGraphVertex* vtx[2];

#define CV_GRAPH_VERTEX_FIELDS()  \
    struct _CvGraphEdge* first;

typedef struct _CvGraphEdge
{
    CV_GRAPH_EDGE_FIELDS()
}
CvGraphEdge;

typedef struct _CvGraphVertex
{
    CV_GRAPH_VERTEX_FIELDS()
}
CvGraphVtx;
```

The graph vertex has a single predefined field that assumes the value of 1 when pointing to the first edge incident to the vertex, or 0 if the vertex is isolated. The edges incident to a vertex make up the single linked non-cycle list. The edge structure is more complex: *`vtx`*`[0]` and *`vtx`*`[1]` are the starting and ending vertices of the edge, *`next`*`[0]` and *`next`*`[1]` are the next edges in the incident lists for *`vtx`*`[0]` and *`vtx`*`[1]`

respectively. In other words, each edge is included in two incident lists since any edge is incident to both the starting and the ending vertices. For example, consider the following oriented graph (see below for more information on non-oriented graphs).

Figure 7-4 Sample Graph

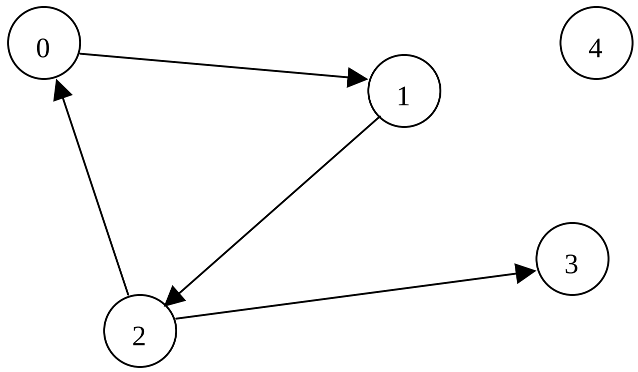

The structure can be created with the following code:

```
CvGraph* graph = cvCreateGraph( CV_SEQ_KIND_GRAPH |
CV_GRAPH_FLAG_ORIENTED,
sizeof(CvGraph),
sizeof(CvGraphVtx)+4,
sizeof(CvGraphEdge),
storage);
for( i = 0; i < 5; i++ )
{
cvGraphAddVtx( graph, 0, 0 );/* arguments like in
 cvSetAdd*/
}
cvGraphAddEdge( graph, 0, 1, 0, 0 ); /* connect vertices 0
```

```
and 1, other two arguments like in cvSetAdd */
cvGraphAddEdge( graph, 1, 2, 0, 0 );
cvGraphAddEdge( graph, 2, 0, 0, 0 );
cvGraphAddEdge( graph, 2, 3, 0, 0 );
```

The internal structure comes to be as follows:

Figure 7-5 Internal Structure for Sample Graph Shown in Figure 7-4

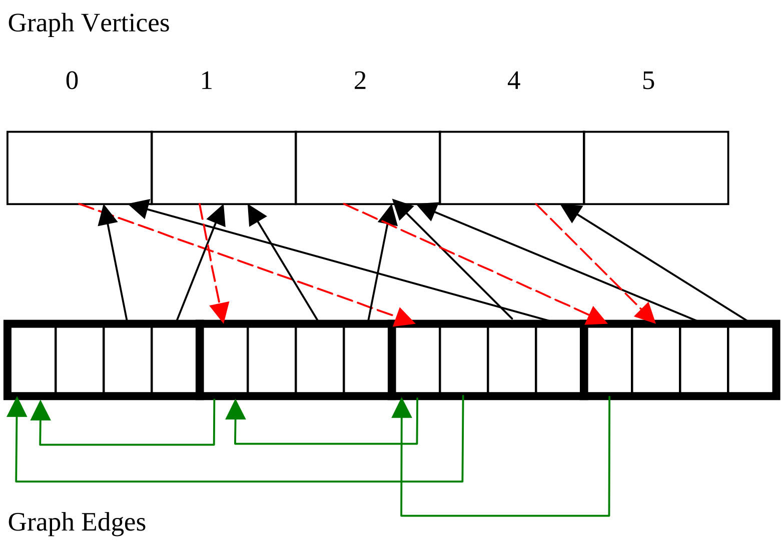

Undirected graphs can also be represented by the structure `CvGraph`. If the non-oriented edges are substituted for the oriented ones, the internal structure remains the same. However, the function used to find edges succeeds only when it finds the edge from 3 to 2, as the function looks not only for edges from 3 to 2 but also from 2 to 3, and such an edge is present as well. As follows from the code, the type of the graph is specified when the graph is created, and the user can change the behavior of the edge searching function by specifying or omitting the flag `CV_GRAPH_FLAG_ORIENTED`. Two edges connecting the same vertices in undirected graphs may never be created because the existence of the edge between two vertices is checked before a new edge is inserted

between them. However, internally the edge can be coded from the first vertex to the second or vice versa. Like in sets, the user may work with either indices or pointers. The graph implementation uses only pointers to refer to edges, but the user can choose indices or pointers for referencing vertices.

Matrix Operations

Besides `IplImage` support, OpenCV introduces special data type `CvMat`, instances of which can be stored as real or complex matrices as well as multi-channel raster data.

Example 7-7 `CvMat` Structure Definition

```
typedef struct CvMat {

int type; /* the type of matrix elements */

union
{
    int rows; /* number of rows in the matrix */
    int height; /* synonym for <rows> */
};

union
{
    int cols; /* number of columns */
    int width; /* synonym for <cols> */
};

int step; /* matrix stride */
union
{
    float* fl;
    double* db;
    uchar* ptr;
} data; /* pointer to matrix data */
};
```

The fist member of the structure *`type`* contains several bit fields:

- Bits 0..3: type of matrix elements (*`depth`*). Can be one of the following:

  `CV_8U` = 0 8-bit, unsigned (*`unsigned char`*)

  `CV_8S` = 1 8-bit, signed (*`signed char`*)

  `CV_16S` = 2 16-bit, signed (*`short`*)

`CV_32S` = 3 32-bit, signed (*`int`*)

`CV_32F` = 4 32-bit, single-precision floating point number (*`float`*)

`CV_64F` = 5 64-bit, double-precision floating point number (*`double`*)

- Bits 4..5: number of channels minus 1, that is:

0	– 1 channel
1	– 2 channels
2	– 3 channels
3	– 4 channels

- Bits 6-15: for internal use.
- Bits 16-31: always equal to 4224 heximal – this magic number is a `CvMat` signature.

The constants `CV_<`*`depth`*`>C<`*`number_of_channels`*`>` are defined to describe possible combinations of the matrix depth and number of channels, for example:

`CV_8UC1` – unsigned 8-bit single-channel data; can be used for grayscale image or binary image – mask.

`CV_8SC1` – signed 8-bit single-channel data.

…

`CV_32FC1` – single-precision real numbers, or real valued matrices.

…

`CV_64FC2` – double-precision complex numbers.

…

`CV_8UC3` – unsigned 8-bit, 3 channels; used for color images.

…

`CV_64FC4` – double-precision floating point number quadruples, e.g., quaternions.

Multiple-channel data is stored in interleaved order, that is, different channels of the same element are stored sequentially, one after another.

`CvMat` is generalization of matrices in usual sense of the word. It can store data of all most common `IplImage` formats . All the basic matrix and image operations on this type are supported. They include:

— arithmetics and logics,
— matrix multiplication,
— dot and cross product,
— perspective transform,
— Mahalonobis distance,
— SVD,
— eigen values problem solution, etc.

While some of operations operate only on arrays, that is, images or matrices, a few operations have both arrays and scalars on input/output. For example, a specific operation adds the same scalar value to all elements of the input array.

OpenCV introduces type `CvScalar` for representing arbitrary scalar value.

Example 7-8 `CvScalar` Definition

```
typedef struct CvScalar
{
        double val[4];
}
CvScalar;
```

Inline functions `cvScalar`, `cvScalarAll` and `cvRealScalar` can be used to construct the structure from scalar components.

Operations that operate on arrays and scalars have `S` suffix in their names. E.g., `cvAddS` adds a scalar to array elements.

Interchangability between IplImage and CvMat.

Most of OpenCV functions that operate on dense arrays accept pointers to both `IplImage` and `CvMat` types in any combinations. It is done via introduction of dummy type `CvArr`, which is defined as follows:

Example 7-9 `CvArr` Type Definition

```
typedef void CvArr;
```

The function analyzes the first integer field at the beginning of the passed structure and thus distinguishes between `IplImage`, the first field of which is equal to the size of `IplImage` structure, and `CvMat`, the first field of which is `0x4224xxxx`.

Drawing Primitives

This section describes simple drawing functions.

The functions described in this chapter are intended mainly to mark out recognized or tracked features in the image. With tracking or recognition pipeline implemented it is often necessary to represent results of the processing in the image. Despite the fact that most Operating Systems have advanced graphic capabilities, they often require an image, where one is going to draw, to be created by special system functions. For example, under Win32 a graphic context (DC) must be created in order to use GDI draw functions. Therefore, several simple functions for *2D* vector graphic rendering have been created. All of them are platform-independent and work with `IplImage` structure. Now supported image formats include byte-depth images with *`depth`* = `IPL_DEPTH_8U` or *`depth`* = `IPL_DEPTH_8S`. The images are either

- single channel, that is, grayscale or
- three channel, that is RGB or, more exactly, BGR as the blue channel goes first.

Several preliminary notes can be made that are relevant for each drawing function of the library:

- All of the functions take *`color`* parameter that means brightness for grayscale images and RGB color for color images. In the latter case a value, passed to the function, can be composed via `CV_RGB` macro that is defined as:

```
#define CV_RGB(r,g,b)  ((((r)&255) << 16)|(((g)&255) << 8)|((b)&255)).
```

- Any function in the group takes one or more points (`CvPoint` structure instance(s)) as input parameters. Point coordinates are counted from top-left ROI corner for top-origin images and from bottom-left ROI corner for bottom-origin images.
- All the functions are divided into two classes - with or without antialiasing. For several functions there exist antialiased versions that end with `AA` suffix. The coordinates, passed to `AA`-functions, can be specified with sub-pixel accuracy, that is, they can have several fractional bits, which number is passed via *`scale`* parameter. For example, if `cvCircleAA` function is passed `center = cvPoint(34,18)`and *`scale = 2`*, then the actual center coordinates are (34/4.,19/4.)==(16.5,4.75).

Simple (that is, non-antialiased) functions have *`thickness`* parameter that specifies thickness of lines a figure is drawn with. For some functions the parameter may take negative values. It causes the functions to draw a filled figure instead of drawing its outline. To improve code readability one may use constant `CV_FILLED = -1` as a *`thickness`* value to draw filled figures.

Utility

Utility functions are unclassified OpenCV functions described in Reference.

Library Technical Organization and System Functions 8

Error Handling

TBD

Memory Management

TBD

Interaction With Low-Level Optimized Functions

TBD

User DLL Creation

TBD

Motion Analysis and Object Tracking Reference 9

Table 9-1 Motion Analysis and Object Tracking Functions and Data Types

Group	Name	Description
	**Functions**	
Background Subtraction Functions	`Acc`	Adds a new image to the accumulating sum.
	`SquareAcc`	Calculates square of the source image and adds it to the destination image.
	`MultiplyAcc`	Calculates product of two input images and adds it to the destination image.
	`RunningAvg`	Calculates weighted sum of two images.
Motion Templates Functions	`UpdateMotionHistory`	Updates the motion history image by moving the silhouette.
	`CalcMotionGradient`	Calculates gradient orientation of the motion history image.
	`CalcGlobalOrientation`	Calculates the general motion direction in the selected region.

Table 9-1 Motion Analysis and Object Tracking Functions and Data Types (continued)

Group	Name	Description
	`SegmentMotion`	Segments the whole motion into separate moving parts.
CamShift Functions	`CamShift`	Finds an object center using the MeanShift algorithm, calculates the object size and orientation.
	`MeanShift`	Iterates to find the object center.
Active Contours Function	`SnakeImage`	Changes contour position to minimize its energy.
Optical Flow Functions	`CalcOpticalFlowHS`	Calculates optical flow for two images implementing Horn and Schunk technique.
	`CalcOpticalFlowLK`	Calculates optical flow for two images implementing Lucas and Kanade technique.
	`CalcOpticalFlowBM`	Calculates optical flow for two images implementing the Block Matching algorithm.
	`CalcOpticalFlowPyrLK`	Calculates optical flow for two images using iterative Lucas-Kanade method in pyramids.
Estimators Functions	`CreateKalman`	Allocates Kalman filter structure.
	`ReleaseKalman`	Deallocates Kalman filter structure.
	`KalmanUpdateByTime`	Estimates the subsequent stochastic model state.

Table 9-1 Motion Analysis and Object Tracking Functions and Data Types (continued)

Group	Name	Description
	KalmanUpdateByMeasurement	Adjusts the stochastic model state on the basis of the true measurements.
	CreateConDensation	Allocates a ConDensation filter structure.
	ReleaseConDensation	Deallocates a ConDensation filter structure.
	ConDensInitSampleSet	Initializes a sample set for condensation algorithm.
	ConDensUpdateByTime	Estimates the subsequent model state by its current state.
	**Data Types**	
Estimators Data Types	CvKalman	
	CvConDensation	

Background Subtraction Functions

Acc

Adds frame to accumulator.

```
void cvAcc (IplImage* img, IplImage* sum, IplImage* mask=0);
```

img Input image.

sum Accumulating image.

mask Mask image.

Discussion

The function `Acc` adds a new image *`img`* to the accumulating sum *`sum`*. If *`mask`* is not `NULL`, it specifies what accumulator pixels are affected.

SquareAcc

Calculates square of source image and adds it to destination image.

```
void cvSquareAcc(IplImage* img, IplImage* sqSum, IplImage* mask=0);
```

img	Input image.
sqSum	Accumulating image.
mask	Mask image.

Discussion

The function `SquareAcc` adds the square of the new image *`img`* to the accumulating sum *`sqSum`* of the image squares. If *`mask`* is not `NULL`, it specifies what accumulator pixels are affected.

MultiplyAcc

Calculates product of two input images and adds it to destination image.

```
void cvMultiplyAcc( IplImage* imgA, IplImage* imgB, IplImage* acc, IplImage*
mask=0);
```

imgA	First input image.
imgB	Second input image.
acc	Accumulating image.

mask Mask image.

Discussion

The function `MultiplyAcc` multiplies input *imgA* by *imgB* and adds the result to the accumulating sum *acc* of the image products. If *mask* is not `NULL`, it specifies what accumulator pixels are affected.

RunningAvg

Calculates weighted sum of two images.

```
void cvRunningAvg( IplImage* imgY, IplImage* imgU, double alpha,
IplImage* mask=0 );
```

imgY Input image.

imgU Destination image.

alpha Weight of input image.

mask Mask image.

Discussion

The function `RunningAvg` calculates weighted sum of two images. Once a statistical model is available, slow updating of the value is often required to account for slowly changing lighting, etc. This can be done by using a simple adaptive filter:

$$\mu_t = \alpha y + (1 - \alpha)\mu_{t-1},$$

where μ (*imgU*) is the updated value, $0 \le \alpha \le 1$ is an averaging constant, typically set to a small value such as 0.05, and y (*imgY*) is a new observation at time t. When the function is applied to a frame sequence, the result is called the running average of the sequence.

If *mask* is not `NULL`, it specifies what accumulator pixels are affected.

Motion Templates Functions

UpdateMotionHistory

Updates motion history image by moving silhouette.

```
void cvUpdateMotionHistory (IplImage* silhouette, IplImage* mhi, double
    timestamp, double mhiDuration);
```

silhouette	Silhouette image that has non-zero pixels where the motion occurs.
mhi	Motion history image, both an input and output parameter.
timestamp	Floating point current time in milliseconds.
mhiDuration	Maximal duration of motion track in milliseconds.

Discussion

The function `UpdateMotionHistory` updates the motion history image with a silhouette, assigning the current *timestamp* value to those `mhi` pixels that have corresponding non-zero silhouette pixels. The function also clears `mhi` pixels older than *timestamp* – *mhiDuration* if the corresponding silhouette values are 0.

CalcMotionGradient

Calculates gradient orientation of motion history image.

```
void cvCalcMotionGradient( IplImage* mhi, IplImage* mask, IplImage*
    orientation, double maxTDelta, double minTDelta, int apertureSize=3 );
```

mhi	Motion history image.
mask	Mask image; marks pixels where motion gradient data is correct. Output parameter.

orientation	Motion gradient orientation image; contains angles from 0 to ~360 degrees.
apertureSize	Size of aperture used to calculate derivatives. Value should be odd, e.g., 3, 5, etc.
maxTDelta	Upper threshold. The function considers the gradient orientation valid if the difference between the maximum and minimum `mhi` values within a pixel neighborhood is lower than this threshold.
minTDelta	Lower threshold. The function considers the gradient orientation valid if the difference between the maximum and minimum `mhi` values within a pixel neighborhood is greater than this threshold.

Discussion

The function `CalcMotionGradient` calculates the derivatives *Dx* and *Dy* for the image `mhi` and then calculates orientation of the gradient using the formula

$$\varphi = \begin{cases} 0, & x = 0, y = o \\ arc\tan(y/x) & else \end{cases}$$

Finally, the function masks off pixels with a very small (less than *minTDelta*) or very large (greater than *maxTDelta*) difference between the minimum and maximum `mhi` values in their neighborhood. The neighborhood for determining the minimum and maximum has the same size as aperture for derivative kernels - *apertureSize x apertureSize* pixels.

CalcGlobalOrientation

Calculates global motion orientation of some selected region.

```
void cvCalcGlobalOrientation( IplImage* orientation, IplImage* mask, IplImage*
    mhi, double currTimestamp, double mhiDuration );
```

orientation	Motion gradient orientation image; calculated by the function `CalcMotionGradient`.
mask	Mask image. It is a conjunction of valid gradient mask, calculated by the function `CalcMotionGradient` and mask of the region, whose direction needs to be calculated.
mhi	Motion history image.
currTimestamp	Current time in milliseconds.
mhiDuration	Maximal duration of motion track in milliseconds.

Discussion

The function `CalcGlobalOrientation` calculates the general motion direction in the selected region.

At first the function builds the orientation histogram and finds the basic orientation as a coordinate of the histogram maximum. After that the function calculates the shift relative to the basic orientation as a weighted sum of all orientation vectors: the more recent is the motion, the greater is the weight. The resultant angle is `<basic orientation> + <shift>`.

SegmentMotion

Segments whole motion into separate moving parts.

```
void cvSegmentMotion( IplImage* mhi, IplImage* segMask, CvMemStorage* storage,
    CvSeq** components, double timestamp, double segThresh );
```

mhi	Motion history image.
segMask	Image where the mask found should be stored.
Storage	Pointer to the memory storage, where the sequence of components should be saved.
components	Sequence of components found by the function.
timestamp	Floating point current time in milliseconds.

segThresh Segmentation threshold; recommended to be equal to the interval between motion history “steps” or greater.

Discussion

The function `SegmentMotion` finds all the motion segments, starting from connected components in the image `mhi` that have value of the current timestamp. Each of the resulting segments is marked with an individual value (1,2 ...).

The function stores information about each resulting motion segment in the structure `CvConnectedComp` (See Example 10-1 in Image Analysis Reference). The function returns a sequence of such structures.

CamShift Functions

CamShift

Finds object center, size, and orientation.

```
int cvCamShift( IplImage* imgProb, CvRect windowIn, CvTermCriteria criteria,
    CvConnectedComp* out, CvBox2D* box=0 );
```

imgProb *2D* object probability distribution.

windowIn Initial search window.

criteria Criteria applied to determine when the window search should be finished.

out Resultant structure that contains converged search window coordinates (*rect* field) and sum of all pixels inside the window (*area* field).

box Circumscribed box for the object. If not `NULL`, contains object size and orientation.

Discussion

The function `CamShift` finds an object center using the Mean Shift algorithm and, after that, calculates the object size and orientation. The function returns number of iterations made within the Mean Shift algorithm.

MeanShift

Iterates to find object center.

```
int cvMeanShift( IplImage* imgProb, CvRect  windowIn, CvTermCriteria
criteria, CvConnectedComp* out );
```

imgProb	*2D* object probability distribution.
windowIn	Initial search window.
criteria	Criteria applied to determine when the window search should be finished.
out	Resultant structure that contains converged search window coordinates (*rect* field) and sum of all pixels inside the window (*area* field).

Discussion

The function `MeanShift` iterates to find the object center given its *2D* color probability distribution image. The iterations are made until the search window center moves by less than the given value and/or until the function has done the maximum number of iterations. The function returns the number of iterations made.

Active Contours Function

SnakeImage

Changes contour position to minimize its energy.

```
void cvSnakeImage( IplImage* image, CvPoint* points, int length,
    float* alpha, float* beta, float* gamma, int coeffUsage, CvSize win,
    CvTermCriteria criteria, int calcGradient=1 );
```

image	Pointer to the source image.
points	Points of the contour.
length	Number of points in the contour.
alpha	Weight of continuity energy.
beta	Weight of curvature energy.
gamma	Weight of image energy.
coeffUsage	Variant of usage of the previous three parameters:<br>• `CV_VALUE` indicates that each of *alpha*, *beta*, *gamma* is a pointer to a single value to be used for all points;<br>• `CV_ARRAY` indicates that each of *alpha*, *beta*, *gamma* is a pointer to an array of coefficients different for all the points of the snake. All the arrays must have the size equal to the snake size.
win	Size of neighborhood of every point used to search the minimum; must be odd.
criteria	Termination criteria.
calcGradient	Gradient flag. If not 0, the function counts source image gradient magnitude as external energy, otherwise the image intensity is considered.

Discussion

The function `SnakeImage` uses image intensity as image energy.

The parameter *criteria.epsilon* is used to define the minimal number of points that must be moved during any iteration to keep the iteration process running.

If the number of moved points is less than *criteria.epsilon* or the function performed *criteria.maxIter* iterations, the function terminates.

Optical Flow Functions

CalcOpticalFlowHS

Calculates optical flow for two images.

```
void cvCalcOpticalFlowHS( IplImage* imgA, IplImage* imgB, int usePrevious,
    IplImage* velx, IplImage* vely, double lambda, CvTermCriteria criteria);
```

imgA	First image.
imgB	Second image.
usePrevious	Uses previous (input) velocity field.
velx	Horizontal component of the optical flow.
vely	Vertical component of the optical flow.
lambda	Lagrangian multiplier.
criteria	Criteria of termination of velocity computing.

Discussion

The function `CalcOpticalFlowHS` computes flow for every pixel, thus output images must have the same size as the input. Horn & Schunck Technique is implemented.

CalcOpticalFlowLK

Calculates optical flow for two images.

```
void cvCalcOpticalFlowLK( IplImage* imgA, IplImage* imgB, CvSize winSize,
IplImage* velx, IplImage* vely);
```

imgA	First image.
imgB	Second image.
winSize	Size of the averaging window used for grouping pixels.
velx	Horizontal component of the optical flow.
vely	Vertical component of the optical flow.

Discussion

The function `CalcOpticalFlowLK` computes flow for every pixel, thus output images must have the same size as input. Lucas & Kanade Technique is implemented.

CalcOpticalFlowBM

Calculates optical flow for two images by block matching method.

```
void cvCalcOpticalFlowBM( IplImage* imgA, IplImage* imgB, CvSize blockSize,
    CvSize shiftSize, CvSize maxRange, int usePrevious, IplImage* velx,
    IplImage* vely);
```

imgA	First image.
imgB	Second image.
blockSize	Size of basic blocks that are compared.
shiftSize	Block coordinate increments.
maxRange	Size of the scanned neighborhood in pixels around block.

usePrevious Uses previous (input) velocity field.

velx Horizontal component of the optical flow.

vely Vertical component of the optical flow.

Discussion

The function `CalcOpticalFlowBM` calculates optical flow for two images using the Block Matching algorithm. Velocity is computed for every block, but not for every pixel, so velocity image pixels correspond to input image blocks and the velocity image must have the following size:

$$velocityFrameSize.width = \left\lceil \frac{imageSize.width}{blockSize.width} \right\rceil,$$

$$velocityFrameSize.height = \left\lceil \frac{imageSize.height}{blockSize.height} \right\rceil.$$

CalcOpticalFlowPyrLK

Calculates optical flow for two images using iterative Lucas-Kanade method in pyramids.

```
void cvCalcOpticalFlowPyrLK(IplImage* imgA, IplImage* imgB, IplImage* pyrA,
    IplImage* pyrB, CvPoint2D32f* featuresA, CvPoint2D32f* featuresB, int
    count, CvSize winSize, int level, char* status, float* error,
    CvTermCriteria criteria, int flags );
```

imgA First frame, at time `t`.

imgB Second frame, at time `t+dt`.

pyrA Buffer for the pyramid for the first frame. If the pointer is not `NULL`, the buffer must have a sufficient size to store the pyramid from `level 1` to `level #<level>`; the total size of `(imgSize.width+8)*imgSize.height/3` bytes is sufficient.

pyrB Similar to `pyrA`, applies to the second frame.

featuresA Array of points for which the flow needs to be found.

featuresB Array of *2D* points containing calculated new positions of input features in the second image.

count	Number of feature points.
winSize	Size of the search window of each pyramid level.
level	Maximal pyramid level number. If `0`, pyramids are not used (single level), if `1`, two levels are used, etc.
status	Array. Every element of the array is set to `1` if the flow for the corresponding feature has been found, `0` otherwise.
error	Array of double numbers containing difference between patches around the original and moved points. Optional parameter; can be `NULL`.
criteria	Specifies when the iteration process of finding the flow for each point on each pyramid level should be stopped.
flags	Miscellaneous flags:

- `CV_LKFLOW_PYR_A_READY`, pyramid for the first frame is precalculated before the call;
- `CV_LKFLOW_PYR_B_READY`, pyramid for the second frame is precalculated before the call;
- `CV_LKFLOW_INITIAL_GUESSES`, array B contains initial coordinates of features before the function call.

Discussion

The function `CalcOpticalFlowPyrLK` calculates the optical flow between two images for the given set of points. The function finds the flow with sub-pixel accuracy.

Both parameters *pyrA* and *pyrB* comply with the following rules: if the image pointer is `0`, the function allocates the buffer internally, calculates the pyramid, and releases the buffer after processing. Otherwise, the function calculates the pyramid and stores it in the buffer unless the flag `CV_LKFLOW_PYR_A[B]_READY` is set. The image should be large enough to fit the Gaussian pyramid data. After the function call both pyramids are calculated and the ready flag for the corresponding image can be set in the next call.

Estimators Functions

CreateKalman

Allocates Kalman filter structure.

```
CvKalman* cvCreateKalman( int DynamParams, int MeasureParams );
```

DynamParams Dimension of the state vector.

MeasureParams Dimension of the measurement vector.

Discussion

The function `CreateKalman` creates `CvKalman` structure and returns pointer to the structure.

ReleaseKalman

Deallocates Kalman filter structure.

```
void cvReleaseKalman(CvKalman** Kalman);
```

Kalman Double pointer to the structure to be released.

Discussion

The function `ReleaseKalman` releases the structure `CvKalman` (see Example 9-1) and frees the memory previously allocated for the structure.

KalmanUpdateByTime

Estimates subsequent model state.

```
void cvKalmanUpdateByTime (CvKalman* Kalman);
```

Kalman Pointer to the structure to be updated.

Discussion

The function `KalmanUpdateByTime` estimates the subsequent stochastic model state by its current state.

KalmanUpdateByMeasurement

Adjusts model state.

```
void cvKalmanUpdateByMeasurement (CvKalman* Kalman,CvMat* Measurement);
```

Kalman Pointer to the structure to be updated.

Measurement Pointer to the structure `CvMat` containing the measurement vector.

Discussion

The function `KalmanUpdateByMeasurement` adjusts stochastic model state on the basis of the true measurements of the model state.

CreateConDensation

Allocates ConDensation filter structure.

```
CvConDensation* cvCreateConDensation( int DynamParams, int MeasureParams, int
    SamplesNum);
```

DynamParams Dimension of the state vector.

MeasureParams Dimension of the measurement vector.

SamplesNum Number of samples.

Discussion

The function `CreateConDensation` creates `cvConDensation` (see Example 9-2) structure and returns pointer to the structure.

ReleaseConDensation

Deallocates ConDensation filter structure.

```
void cvReleaseConDensation(CvConDensation** ConDens);
```

ConDens Pointer to the pointer to the structure to be released.

Discussion

The function `ReleaseConDensation` releases the structure `CvConDensation` (see Example 9-2) and frees all memory previously allocated for the structure.

ConDensInitSampleSet

Initializes sample set for condensation algorithm.

```
void cvConDensInitSampleSet(CvConDensation* ConDens, CvMat* lowerBound CvMat*
    upperBound);
```

ConDens Pointer to a structure to be initialized.

lowerBound Vector of the lower boundary for each dimension.

upperBound Vector of the upper boundary for each dimension.

Discussion

The function `ConDensInitSampleSet` fills the samples arrays in the structure `CvConDensation` (see Example 9-2) with values within specified ranges.

ConDensUpdateByTime

Estimates subsequent model state.

```
void cvConDensUpdateByTime(CvConDensation* ConDens);
```

ConDens Pointer to the structure to be updated.

Discussion

The function `ConDensUpdateByTime` estimates the subsequent stochastic model state from its current state.

Estimators Data Types

Example 9-1 `CvKalman`

```
typedef struct CvKalman
{
int MP;     //Dimension of measurement vector
int DP;     // Dimension of state vector
float* PosterState;   // Vector of State of the System in k-th step
float* PriorState;   // Vector of State of the System in (k-1)-th step
float* DynamMatr;           // Matrix of the linear Dynamics system
float* MeasurementMatr;     // Matrix of linear measurement
float* MNCovariance;        // Matrix of measurement noice covariance
float* PNCovariance;        // Matrix of process noice covariance
float* KalmGainMatr;        // Kalman Gain Matrix
float* PriorErrorCovariance; //Prior Error Covariance matrix
float* PosterErrorCovariance;//Poster Error Covariance matrix
float* Temp1;                // Temporary Matrixes
float* Temp2;
}CvKalman;
```

Example 9-2 `CvConDensation`

```
typedef struct
{
int MP;      //Dimension of measurement vector
int DP;      // Dimension of state vector
float* DynamMatr;        // Matrix of the linear Dynamics system
float* State;  // Vector of State
int SamplesNum;          // Number of the Samples
float** flSamples;       // array of the Sample Vectors
float** flNewSamples;    // temporary array of the Sample Vectors
float* flConfidence;     // Confidence for each Sample
float* flCumulative;     // Cumulative confidence
float* Temp;             // Temporary vector
float* RandomSample;     // RandomVector to update sample set
CvRandState* RandS;  // Array of structures to generate random vectors
}CvConDensation;
```

Image Analysis Reference 10

Table 10-1 Image Analysis Reference

Group	Name	Description
	**Functions**	
Contour Retrieving Functions	`FindContours`	Finds contours in a binary image.
	`StartFindContours`	Initializes contour scanning process.
	`FindNextContour`	Finds the next contour on the raster.
	`SubstituteContour`	Replaces the retrieved contour.
	`EndFindContours`	Finishes scanning process.
Features Functions	`Laplace`	Calculates convolution of the input image with Laplacian operator.
	`Sobel`	Calculates convolution of the input image with Sobel operator.
	`Canny`	Implements Canny algorithm for edge detection.
	`PreCornerDetect`	Calculates two constraint images for corner detection.

Table 10-1 Image Analysis Reference (continued)

Group	Name	Description
	CornerEigenValsAndVecs	Calculates eigenvalues and eigenvectors of image blocks for corner detection.
	CornerMinEigenVal	Calculates minimal eigenvalues of image blocks for corner detection.
	FindCornerSubPix	Refines corner locations.
	GoodFeaturesToTrack	Determines strong corners on the image.
	HoughLines	Finds lines in a binary image, SHT algorithm.
	HoughLinesSDiv	Finds lines in a binary image, MHT algorithm.
	HoughLinesP	Finds line segments in a binary image, PPHT algorithm.
Image Statistics Functions	CountNonZero	Counts non-zero pixels in an image.
	SumPixels	Summarizes pixel values in an image.
	Mean	Calculates mean value in an image region.
	Mean_StdDev	Calculates mean and standard deviation in an image region.
	MinMaxLoc	Finds global minimum and maximum in an image region.
	Norm	Calculates image norm, difference norm or relative difference norm.

Table 10-1 Image Analysis Reference (continued)

Group	Name	Description
	Moments	Calculates all moments up to the third order of the image plane and fills the moment state structure.
	GetSpatialMoment	Retrieves spatial moment from the moment state structure.
	GetCentralMoment	Retrieves the central moment from the moment state structure.
	GetNormalizedCentralMoment	Retrieves the normalized central moment from the moment state structure.
	GetHuMoments	Calculates seven Hu moment invariants from the moment state structure.
Pyramid Functions	PyrDown	Downsamples an image.
	PyrUp	Upsamples an image.
	PyrSegmentation	Implements image segmentation by pyramids.
Morphology Functions	CreateStructuringElementEx	Creates a structuring element.
	ReleaseStructuringElement	Deletes the structuring element.
	Erode	Erodes the image by using an arbitrary structuring element.
	Dilate	Dilates the image by using an arbitrary structuring element.
	MorphologyEx	Performs advanced morphological transformations.

Table 10-1 Image Analysis Reference (continued)

Group	Name	Description
Distance Transform Function	`DistTransform`	Calculates distance to the closest zero pixel for all non-zero pixels of the source image.
Threshold Functions	`AdaptiveThreshold`	Provides an adaptive thresholding binary image.
	`Threshold`	Thresholds the binary image.
Flood Filling Function	`FloodFill`	Makes flood filling of the image connected domain.
Histogram Functions	`CreateHist`	Creates a histogram.
	`ReleaseHist`	Releases the histogram header and the underlying data.
	`MakeHistHeaderForArray`	Initializes the histogram header.
	`QueryHistValue_1D`	Queries the value of a 1D histogram bin.
	`QueryHistValue_2D`	Queries the value of a 2D histogram bin
	`QueryHistValue_3D`	Queries the value of a 3D histogram bin
	`QueryHistValue_nD`	Queries the value of an nD histogram bin
	`GetHistValue_1D`	Returns the pointer to 1D histogram bin.
	`GetHistValue_2D`	Returns the pointer to 2D histogram bin.
	`GetHistValue_3D`	Returns the pointer to 3D histogram bin.
	`GetHistValue_nD`	Returns the pointer to nD histogram bin.

Table 10-1 Image Analysis Reference (continued)

Group	Name	Description
	GetMinMaxHistValue	Finds minimum and maximum histogram bins.
	NormalizeHist	Normalizes a histogram.
	ThreshHist	Thresholds a histogram.
	CompareHist	Compares two histograms.
	CopyHist	Makes a copy of a histogram.
	SetHistBinRanges	Sets bounds of histogram bins.
	CalcHist	Calculates a histogram of an array of single-channel images.
	CalcBackProject	Calculates back projection of a histogram.
	CalcBackProjectPatch	Calculates back projection by comparing histograms of the source image patches with the given histogram.
	CalcEMD	Computes earth mover distance and/or a lower boundary of the distance.
	CalcContrastHist	Calculates a histogram of contrast for the one-channel image.
	**Data Types**	
Pyramid Data Types	CvConnectedComp	Represents an element for each single connected components representation in memory.
Histogram Data Types	CvHistogram	Stores all the types of histograms (`1D, 2D, nD`).

Contour Retrieving Functions

FindContours

Finds contours in binary image.

```
int cvFindContours (IplImage* img, CvMemStorage* storage, CvSeq**
    firstContour, int headerSize=sizeof(CvContour),
    CvContourRetrievalMode mode=CV_RETR_LIST,CvChainApproxMethod
    method=CV_CHAIN_APPROX_SIMPLE);
```

img	Single channel image of `IPL_DEPTH_8U` type. Non-zero pixels are treated as 1-pixels. The function modifies the content of the input parameter.
storage	Contour storage location.
firstContour	Output parameter. Pointer to the first contour on the highest level.
headerSize	Size of the sequence header; must be equal to or greater than *sizeof(CvChain)* when the method `CV_CHAIN_CODE` is used, and equal to or greater than *sizeof(CvContour)* otherwise.
mode	Retrieval mode.<br>• `CV_RETR_EXTERNAL` retrieves only the extreme outer contours (list);<br>• `CV_RETR_LIST` retrieves all the contours (list);<br>• `CV_RETR_CCOMP` retrieves the two-level hierarchy (list of connected components);<br>• `CV_RETR_TREE` retrieves the complete hierarchy (tree).
method	Approximation method.<br>• `CV_CHAIN_CODE` outputs contours in the Freeman chain code.

- `CV_CHAIN_APPROX_NONE` translates all the points from the chain code into points;
- `CV_CHAIN_APPROX_SIMPLE` compresses horizontal, vertical, and diagonal segments, that is, it leaves only their ending points;
- `CV_CHAIN_APPROX_TC89_L1`, `CV_CHAIN_APPROX_TC89_KCOS` are two versions of the Teh-Chin approximation algorithm.

Discussion

The function `FindContours` retrieves contours from the binary image and returns the pointer to the first contour. Access to other contours may be gained through the *`h_next`* and *`v_next`* fields of the returned structure. The function returns total number of retrieved contours.

StartFindContours

Initializes contour scanning process.

```
CvContourScanner cvStartFindContours (IplImage* img, CvMemStorage* storage,
    int  headerSize, CvContourRetrievalMode mode, CvChainApproxMethod method);
```

`img` Single channel image of `IPL_DEPTH_8U` type. Non-zero pixels are treated as 1-pixels. The function damages the image.

`storage` Contour storage location.

`headerSize` Must be equal to or greater than *`sizeof(CvChain)`* when the method `CV_CHAIN_CODE` is used, and equal to or greater than *`sizeof(CvContour)`* otherwise.

`mode` Retrieval mode.

- `CV_RETR_EXTERNAL` retrieves only the extreme outer contours (list);

- `CV_RETR_LIST` retrieves all the contours (list);
- `CV_RETR_CCOMP` retrieves the two-level hierarchy (list of connected components);
- `CV_RETR_TREE` retrieves the complete hierarchy (tree).

method Approximation method.

- `CV_CHAIN_CODE` codes the output contours in the chain code;
- `CV_CHAIN_APPROX_NONE` translates all the points from the chain code into points;
- `CV_CHAIN_APPROX_SIMPLE` substitutes ending points for horizontal, vertical, and diagonal segments;
- `CV_CHAIN_APPROX_TC89_L1`, `CV_CHAIN_APPROX_TC89_KCOS` are two versions of the Teh-Chin approximation algorithm.

Discussion

The function `StartFindContours` initializes the contour scanner and returns the pointer to it. The structure is internal and no description is provided.

FindNextContour

Finds next contour on raster.

```
CvSeq* cvFindNextContour (CvContourScanner scanner);
```

scanner Contour scanner initialized by the function `cvStartFindContours`.

Discussion

The function `FindNextContour` returns the next contour or 0, if the image contains no other contours.

SubstituteContour

Replaces retrieved contour.

```
void cvSubstituteContour (CvContourScanner scanner, CvSeq* newContour);
```

scanner Contour scanner initialized by the function `cvStartFindContours`.

newContour Substituting contour.

Discussion

The function `SubstituteContour` replaces the retrieved contour, that was returned from the preceding call of the function `FindNextContour` and stored inside the contour scanner state, with the user-specified contour. The contour is inserted into the resulting structure, list, two-level hierarchy, or tree, depending on the retrieval mode. If the parameter *newContour* is 0, the retrieved contour is not included into the resulting structure, nor all of its children that might be added to this structure later.

EndFindContours

Finishes scanning process.

```
CvSeq* cvEndFindContours (CvContourScanner* scanner);
```

scanner Pointer to the contour scanner.

Discussion

The function `EndFindContours` finishes the scanning process and returns the pointer to the first contour on the highest level.

Features Functions

Fixed Filters Functions

For background on fundamentals of Fixed Filters Functions see Fixed Filters in Image Analysis Chapter.

Laplace

Calculates convolution of input image with Laplacian operator.

```
void cvLaplace (IplImage* src, IplImage* dst, int apertureSize=3);
```

src	Input image.
dst	Destination image.
apertureSize	Size of the Laplacian kernel.

Discussion

The function `Laplace` calculates the convolution of the input image *src* with the Laplacian kernel of a specified size *apertureSize* and stores the result in *dst*.

Sobel

Calculates convolution of input image with Sobel operator.

```
void cvSobel (IplImage* src, IplImage* dst, int dx, int dy, int
apertureSize=3);
```

src	Input image.
dst	Destination image.

dx Order of the derivative *x*.

dy Order of the derivative *y*.

apertureSize Size of the extended Sobel kernel. The special value `CV_SCHARR`, equal to `-1`, corresponds to the Scharr filter `1/16[-3,-10,-3; 0, 0, 0; 3, 10, 3]`; may be transposed.

Discussion

The function `Sobel` calculates the convolution of the input image *src* with a specified Sobel operator kernel and stores the result in *dst*.

Feature Detection Functions

For background on fundamentals of Feature Detection Functions see Feature Detection in Image Analysis Chapter.

Canny

Implements Canny algorithm for edge detection.

```
void cvCanny (IplImage* img, IplImage* edges, double lowThresh, double
    highThresh, int apertureSize=3);
```

img Input image.

edges Image to store the edges found by the function.

lowThresh Low threshold used for edge searching.

highThresh High threshold used for edge searching.

apertureSize Size of the Sobel operator to be used in the algorithm.

Discussion

The function `Canny` finds the edges on the input image *img* and puts them into the output image *edges* using the Canny algorithm described above.

PreCornerDetect

Calculates two constraint images for corner detection.

```
void cvPreCornerDetect (IplImage* img, IplImage* corners, Int apertureSize);
```

img	Input image.
corners	Image to store the results.
apertureSize	Size of the Sobel operator to be used in the algorithm.

Discussion

The function `PreCornerDetect` finds the corners on the input image *img* and stores them into the output image *corners* in accordance with Method 1for corner detection.

CornerEigenValsAndVecs

Calculates eigenvalues and eigenvectors of image blocks for corner detection.

```
void cvCornerEigenValsAndVecs (IplImage* img, IplImage* eigenvv, int
    blockSize, int apertureSize=3);
```

img	Input image.
eigenvv	Image to store the results.
blockSize	Linear size of the square block over which derivatives averaging is done.
apertureSize	Derivative operator aperture size in the case of byte source format. In the case of floating-point input format this parameter is the number of the fixed float filter used for differencing.

Discussion

For every raster pixel the function `CornerEigenValsAndVecs` takes a block of $blockSize \times blockSize$ pixels with the top-left corner, or top-bottom corner for bottom-origin images, at the pixel, computes first derivatives D_x and D_y within the block and then computes eigenvalues and eigenvectors of the matrix:

$$C = \begin{bmatrix} \sum D_x^2 & \sum D_x D_y \\ \sum D_x D_y & \sum D_y^2 \end{bmatrix}$$, where summations are performed over the block.

The format of the frame *eigenvv* is the following: for every pixel of the input image the frame contains 6 float values $(\lambda 1, \lambda 2, x1, y1, x2, y2)$.

$\lambda 1, \lambda 2$ are eigenvalues of the above matrix, not sorted by value.

$x1, y1$ are coordinates of the normalized eigenvector that corresponds to $\lambda 1$.

$x2, y2$ are coordinates of the normalized eigenvector that corresponds to $\lambda 2$.

In case of a singular matrix or if one of the eigenvalues is much less than another, all six values are set to 0. The Sobel operator with aperture width *aperureSize* is used for differentiation.

CornerMinEigenVal

Calculates minimal eigenvalues of image blocks for corner detection.

```
void cvCornerMinEigenVal (IplImage* img, IplImage* eigenvv, int blockSize, int
    apertureSize=3);
```

img	Input image.
eigenvv	Image to store the results.
blockSize	Linear size of the square block over which derivatives averaging is done.

apertureSize Derivative operator aperture size in the case of byte source format. In the case of floating-point input format this parameter is the number of the fixed float filter used for differencing.

Discussion

For every raster pixel the function `CornerMinEigenVal` takes a block of $blockSize \times blockSize$ pixels with the top-left corner, or top-bottom corner for bottom-origin images, at the pixel, computes first derivatives D_x and D_y within the block and then computes eigenvalues and eigenvectors of the matrix:

$$C = \begin{bmatrix} \sum D_x^2 & \sum D_x D_y \\ \sum D_x D_y & \sum D_y^2 \end{bmatrix}$$, where summations are made over the block.

In case of a singular matrix the minimal eigenvalue is set to 0. The Sobel operator with aperture width *aperureSize* is used for differentiation.

FindCornerSubPix

Refines corner locations.

```
void cvFindCornerSubPix (IplImage* img, CvPoint2D32f* corners, int count,
CvSize win, CvSize zeroZone, CvTermCriteria criteria);
```

img Input raster image.

corners Initial coordinates of the input corners and refined coordinates on output.

count Number of corners.

win Half sizes of the search window. For example, if `win = (5,5)`, then $5*2+1 \times 5*2+1 = 11 \times 11$ pixel window to be used.

zeroZone Half size of the dead region in the middle of the search zone to avoid possible singularities of the autocorrelation matrix. The value of `(-1,-1)`indicates that there is no such zone.

`criteria` Criteria for termination of the iterative process of corner refinement. Iterations may specify a stop when either required precision is achieved or the maximal number of iterations done.

Discussion.

The function `FindCornerSubPix` iterates to find the accurate sub-pixel location of a corner, or "radial saddle point", as shown in Figure 10-1.

Figure 10-1 Sub-Pixel Accurate Corner

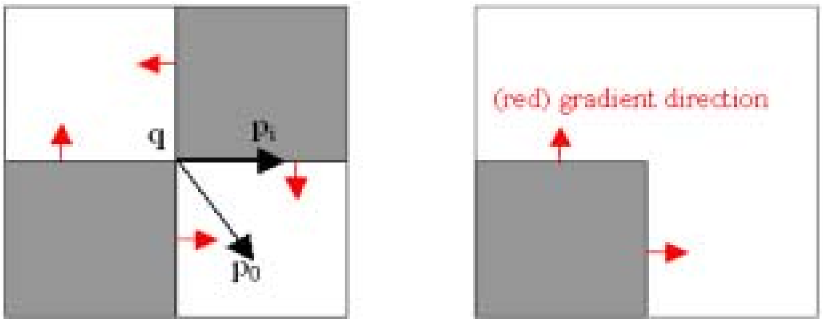

Sub-pixel accurate corner (radial saddle point) locator is based on the observation that any vector from q to p is orthogonal to the image gradient.

The core idea of this algorithm is based on the observation that every vector from the center q to a point p located within a neighborhood of q is orthogonal to the image gradient at p subject to image and measurement noise. Thus:

$$\varepsilon_i = \nabla I_{p_i}^T \cdot (q - p_i) ,$$

where ∇I_{p_i} is the image gradient at the one of the points p in a neighborhood of q. The value of q is to be found such that ε_i is minimized. A system of equations may be set up with ε_i 's set to zero:

$$\left(\sum_i \nabla I_{p_i} \cdot \nabla I_{p_i}^T\right) \bullet q - \left(\sum_i \nabla I_{p_i} \cdot \nabla I_{p_i}^T \cdot p_i\right) = 0,$$

where the gradients are summed within a neighborhood (“search window”) of q. Calling the first gradient term G and the second gradient term b gives:

$$q = G^{-1} \cdot b.$$

The algorithm sets the center of the neighborhood window at this new center q and then iterates until the center keeps within a set threshold.

GoodFeaturesToTrack

Determines strong corners on image.

```
void cvGoodFeaturesToTrack (IplImage* image, IplImage* eigImage, IplImage*
    tempImage, CvPoint2D32f* corners, int* cornerCount, double  qualityLevel,
    double minDistance);
```

image	Source image with byte, signed byte, or floating-point depth, single channel.
eigImage	Temporary image for minimal eigenvalues for pixels: floating-point, single channel.
tempImage	Another temporary image: floating-point, single channel.
corners	Output parameter. Detected corners.
cornerCount	Output parameter. Number of detected corners.
qualityLevel	Multiplier for the maxmin eigenvalue; specifies minimal accepted quality of image corners.
minDistance	Limit, specifying minimum possible distance between returned corners; Euclidian distance is used.

Discussion

The function `GoodFeaturesToTrack` finds corners with big eigenvalues in the image. The function first calculates the minimal eigenvalue for every pixel of the source image and then performs non-maxima suppression (only local maxima in 3x3 neighborhood remain). The next step is rejecting the corners with the minimal eigenvalue less than *qualityLevel*<max_of_min_eigen_vals>*. Finally, the function ensures that all the corners found are distanced enough from one another by getting two strongest features and checking that the distance between the points is satisfactory. If not, the point is rejected.

Hough Transform Functions

For background on fundamentals of Hough Transform Functions see Hough Transform in Image Analysis Chapter.

HoughLines

Finds lines in binary image, SHT algorithm.

```
void cvHoughLines (IplImage* src, double rho, double theta, int threshold,
    float* lines, int linesNumber);
```

src	Source image.
rho	Radius resolution.
theta	Angle resolution.
threshold	Threshold parameter.
lines	Pointer to the array of output lines parameters. The array should have *2*linesNumber* elements.
linesNumber	Maximum number of lines.

Discussion

The function `HoughLines` implements Standard Hough Transform (SHT) and demonstrates average performance on arbitrary images. The function returns number of detected lines. Every line is characterized by pair (ρ,θ), where ρ is distance from line to point *(0,0)* and θ is the angle between the line and horizontal axis.

HoughLinesSDiv

Finds lines in binary image, MHT algorithm.

```
int cvHoughLinesSDiv (IplImage* src, double rho, int srn, double theta,  int
    stn, int threshold, float* lines, int linesNumber);
```

src	Source image.
rho	Rough radius resolution.
srn	Radius accuracy coefficient, *rho/srn* is accurate *rho* resolution.
theta	Rough angle resolution.
stn	Angle accuracy coefficient, *theta/stn* is accurate angle resolution.
threshold	Threshold parameter.
lines	Pointer to array of the detected lines parameters. The array should have *2*linesNumber* elements.
linesNumber	Maximum number of lines.

Discussion

The function `HoughLinesSDiv` implements coarse-to-fine variant of SHT and is significantly faster than the latter on images without noise and with a small number of lines. The output of the function has the same format as the output of the function `HoughLines`.

HoughLinesP

Finds line segments in binary image, PPHT algorithm.

```
int cvHoughLinesP (IplImage* src, double rho, double theta, int threshold,
    int lineLength, int lineGap, int* lines, int linesNumber);
```

src	Source image.
rho	Rough radius resolution.
theta	Rough angle resolution.
threshold	Threshold parameter.
lineLength	Minimum accepted line length.
lineGap	Maximum length of accepted line gap.
lines	Pointer to array of the detected line segments' ending coordinates. The array should have `linesNumber*4` elements.
linesNumber	Maximum number of line segments.

Discussion

The function `HoughLinesP` implements Progressive Probabilistic Standard Hough Transform. It retrieves no more than `linesNumber` line segments; each of those must be not shorter than `lineLength` pixels. The method is significantly faster than SHT on noisy images, containing several long lines. The function returns number of detected segments. Every line segment is characterized by the coordinates of its ends (x_1, y_1, x_2, y_2).

Image Statistics Functions

CountNonZero

Counts non-zero pixels in image.

```
int cvCountNonZero (IplImage* image);
```

image	Pointer to the source image.

Discussion

The function `CountNonZero` returns the number of non-zero pixels in the whole image or selected image `ROI`.

SumPixels

Summarizes pixel values in image.

```
double cvSumPixels (IplImage* image);
```

image	Pointer to the source image.

Discussion

The function `SumPixels` returns sum of pixel values in the whole image or selected image `ROI`.

Mean

Calculates mean value in image region.

```
double cvMean( IplImage* image, IplImage* mask=0 );
```

image	Pointer to the source image.
mask	Mask image.

Discussion

The function `Mean` calculates the mean of pixel values in the whole image, selected `ROI` or, if *mask* is not `NULL`, in an image region of arbitrary shape.

Mean_StdDev

Calculates mean and standard deviation in image region.

```
void cvMean_StdDev (IplImage* image, double* mean, double* stddev,
IplImage* mask=0);
```

image	Pointer to the source image.
mean	Pointer to returned mean.
stddev	Pointer to returned standard deviation.
mask	Pointer to the single-channel mask image.

Discussion

The function `Mean_StdDev` calculates mean and standard deviation of pixel values in the whole image, selected `ROI` or, if *mask* is not `NULL`, in an image region of arbitrary shape. If the image has more than one channel, the `COI` must be selected.

MinMaxLoc

Finds global minimum and maximum in image region.

```
void cvMinMaxLoc (IplImage* image, double* minVal, double* maxVal,
    CvPoint* minLoc, CvPoint* maxLoc, IplImage* mask=0);
```

image	Pointer to the source image.
minVal	Pointer to returned minimum value.
maxVal	Pointer to returned maximum value.
minLoc	Pointer to returned minimum location.
maxLoc	Pointer to returned maximum location.
mask	Pointer to the single-channel mask image.

Discussion

The function `MinMaxLoc` finds minimum and maximum pixel values and their positions. The extremums are searched over the whole image, selected `ROI` or, if *mask* is not `NULL`, in an image region of arbitrary shape. If the image has more than one channel, the `COI` must be selected.

Norm

Calculates image norm, difference norm or relative difference norm.

```
double cvNorm (IplImage* imgA, IplImage* imgB, int normType, IplImage*
mask=0);
```

imgA	Pointer to the first source image.
imgA	Pointer to the second source image if any, `NULL` otherwise.
normType	Type of norm.

`mask` Pointer to the single-channel mask image.

Discussion

The function `Norm` calculates images norms defined below. If `imgB = NULL`, the following three norm types of image `A` are calculated:

$$NormType = CV\_C: \|A\|_C = max(|A_{ij}|),$$

$$NormType = CV\_L1: \|A\|_{L_1} = \sum_{i=1}^{N_x}\sum_{j=1}^{N_y}|A_{ij}|,$$

$$NormType = CV\_L2: \|A\|_{L_2} = \sqrt{\sum_{i=1}^{N_x}\sum_{j=1}^{N_y}A_{ij}^2}.$$

If $imgB \neq$ `NULL`, the difference or relative difference norms are calculated:

$$NormType = CV\_C: \|A-B\|_C = max(|A_i - B_i|),$$

$$NormType = CV\_L1: \|A-B\|_{L_1} = \sum_{i=1}^{N_x}\sum_{j=1}^{N_y}|A_{ij}-B_{ij}|,$$

$$NormType = CV\_L2: \|A-B\|_{L_2} = \sqrt{\sum_{i=1}^{N_x}\sum_{j=1}^{N_y}(A_{ij}-B_{ij})^2},$$

$$NormType = CV\_RELATIVEC: \|A-B\|_C / \|B\|_C = \frac{max(|A_{ij}-B_{ij}|)}{max(|B_{ij}|)},$$

$$NormType = CV\_RELATIVEL1: \|A-B\|_{L_1} / \|B\|_{L_1} = \frac{\sum_{i=1}^{N_x}\sum_{j=1}^{N_y}|A_{ij}-B_{ij}|}{\sum_{i=1}^{N_x}\sum_{j=1}^{N_y}|B_{ij}|},$$

$$NormType = \text{CV\_RELATIVEL2: } \|A-B\|_{L_2} / \|B\|_{L_2} = \frac{\sqrt{\sum_{i=1}^{N_x}\sum_{j=1}^{N_y}(A_{ij}-B_{ij})^2}}{\sqrt{\sum_{i=1}^{N_x}\sum_{j=1}^{N_y}(B_{ij})^2}}.$$

The function `Norm` returns the calculated norm.

Moments

Calculates all moments up to third order of image plane and fills moment state structure.

```
void cvMoments (IplImage* image, CvMoments* moments, int isBinary=0);
```

image	Pointer to the image or to top-left corner of its `ROI`.
moments	Pointer to returned moment state structure.
isBinary	If the flag is non-zero, all the zero pixel values are treated as zeroes, all the others are treated as ones.

Discussion

The function `Moments` calculates moments up to the third order and writes the result to the moment state structure. This structure is used then to retrieve a certain spatial, central, or normalized moment or to calculate Hu moments.

GetSpatialMoment

Retrieves spatial moment from moment state structure.

```
double cvGetSpatialMoment (CvMoments* moments, int x_order, int y_order);
```

moments	Pointer to the moment state structure.
x_order	Order x of required moment.
y_order	Order y of required moment<br>(0<= *x_order*, *y_order*; *x_order* + *y_order* <= 3).

Discussion

The function GetSpatialMoment retrieves the spatial moment, which is defined as:

$$M_{x\_order,\,y\_order} = \sum_{x,\,y} I(x,y)\,x^{x\_order}\,y^{y\_order}$$, where

$I(x,y)$ is the intensity of the pixel (x,y).

GetCentralMoment

Retrieves central moment from moment state structure.

```
double cvGetCentralMoment (CvMoments* moments, int x_order, int y_order);
```

moments	Pointer to the moment state structure.
x_order	Order x of required moment.
y_order	Order y of required moment<br>(0<= *x_order*, *y_order*; *x_order* + *y_order* <= 3).

Discussion

The function `GetCentralMoment` retrieves the central moment, which is defined as:

$$\mu_{x\_order, y\_order} = \sum_{x, y} I(x, y)(x - \bar{x})^{x\_order}(y - \bar{y})^{y\_order}$$, where

$I(x, y)$ is the intensity of pixel (x, y), $\bar{x}$ is the coordinate x of the mass center, $\bar{y}$ is the coordinate y of the mass center:

$$\bar{x} = \frac{M_{1, 0}}{M_{0, 0}}, \bar{y} = \frac{M_{0, 1}}{M_{0, 0}}.$$

GetNormalizedCentralMoment

Retrieves normalized central moment from moment state structure.

```
double cvGetNormalizedCentralMoment (CvMoments* moments, int x_order, int
    y_order);
```

moments — Pointer to the moment state structure.

x_order — Order x of required moment.

y_order — Order y of required moment

($0 <= x\_order, y\_order; x\_order + y\_order <= 3$).

Discussion

The function `GetNormalizedCentralMoment` retrieves the normalized central moment, which is defined as:

$$\eta_{x\_order, y\_order} = \frac{\mu_{x\_order, y\_order}}{M_{0, 0}^{((x\_order + y\_order)/2 + 1)}}.$$

GetHuMoments

Calculates seven moment invariants from moment state structure.

```
void cvGetHuMoments (CvMoments* moments, CvHuMoments* HuMoments);
```

`moments`	Pointer to the moment state structure.
`HuMoments`	Pointer to Hu moments structure.

Discussion

The function `GetHuMoments` calculates seven Hu invariants using the following formulas:

$$h_1 = \eta_{20} + \eta_{02},$$

$$h_2 = (\eta_{20} - \eta_{02})^2 + 4\eta_{11}^2,$$

$$h_3 = (\eta_{30} - 3\eta_{12})^2 + (3\eta_{21} - \eta_{03})^2,$$

$$h_4 = (\eta_{30} + \eta_{12})^2 + (\eta_{21} + \eta_{03})^2,$$

$$h_5 = (\eta_{30} - 3\eta_{12})(\eta_{30} + \eta_{12})[(\eta_{30} + \eta_{12})^2 - 3(\eta_{21} + \eta_{03})^2] + (3\eta_{21} - \eta_{03})(\eta_{21} + \eta_{03})[3(\eta_{30} + \eta_{12})^2 - (\eta_{21} + \eta_{03})^2]$$

$$h_6 = (\eta_{20} - \eta_{02})[(\eta_{30} + \eta_{12})^2 - (\eta_{21} + \eta_{03})^2] + 4\eta_{11}(\eta_{30} + \eta_{12})(\eta_{21} + \eta_{03}),$$

$$h_7 = (3\eta_{21} - \eta_{03})(\eta_{21} + \eta_{03})[3(\eta_{30} + \eta_{12})^2 - (\eta_{21} + \eta_{03})^2] - (\eta_{30} - 3\eta_{12})(\eta_{21} + \eta_{03})[3(\eta_{30} + \eta_{12})^2 - (\eta_{21} + \eta_{03})^2]$$

These values are proved to be invariants to the image scale, rotation, and reflection except the seventh one, whose sign is changed by reflection.

Pyramid Functions

PyrDown

Downsamples image.

```
void cvPyrDown (IplImage* src, IplImage* dst, IplFilter
    filter=IPL_GAUSSIAN_5x5);
```

src	Pointer to the source image.
dst	Pointer to the destination image.
filter	Type of the filter used for convolution; only `IPL_GAUSSIAN_5x5` is currently supported.

Discussion

The function `PyrDown` performs downsampling step of Gaussian pyramid decomposition. First it convolves source image with the specified filter and then downsamples the image by rejecting even rows and columns. So the destination image is four times smaller than the source image.

PyrUp

Upsamples image.

```
void cvPyrUp (IplImage* src, IplImage* dst, IplFilter
    filter=IPL_GAUSSIAN_5x5);
```

src	Pointer to the source image.
dst	Pointer to the destination image.
filter	Type of the filter used for convolution; only `IPL_GAUSSIAN_5x5` is currently supported.

Discussion

The function `PyrUp` performs up-sampling step of Gaussian pyramid decomposition. First it upsamples the source image by injecting even zero rows and columns and then convolves result with the specified filter multiplied by 4 for interpolation. So the destination image is four times larger than the source image.

PyrSegmentation

Implements image segmentation by pyramids.

```
void cvPyrSegmentation (IplImage* srcImage, IplImage* dstImage, CvMemStorage*
    storage, CvSeq** comp, int  level, double threshold1, double threshold2);
```

srcImage	Pointer to the input image data.
dstImage	Pointer to the output segmented data.
storage	Storage; stores the resulting sequence of connected components.
comp	Pointer to the output sequence of the segmented components.
level	Maximum level of the pyramid for the segmentation.
threshold1	Error threshold for establishing the links.
threshold2	Error threshold for the segments clustering.

Discussion

The function `PyrSegmentation` implements image segmentation by pyramids. The pyramid builds up to the level *level*. The links between any pixel *a* on level *i* and its candidate father pixel *b* on the adjacent level are established if $\rho(c(a), c(b)) < threshold1$. After the connected components are defined, they are joined into several clusters. Any two segments *A* and *B* belong to the same cluster, if $\rho(c(A), c(B)) < threshold2$. The input image has only one channel, then $\rho(c^1, c^2) = |c^1 - c^2|$. If the input image has three channels (red, green and blue), then $\rho(c^1, c^2) = 0{,}3 \cdot (c_r^1 - c_r^2) + 0{,}59 \cdot (c_g^1 - c_g^2) + 0{,}11 \cdot (c_b^1 - c_b^2)$. There may be more than one connected component per a cluster.

Input *srcImage* and output *dstImage* should have the identical IPL_DEPTH_8U depth and identical number of channels (1 or 3).

Morphology Functions

CreateStructuringElementEx

Creates structuring element.

```
IplConvKernel* cvCreateStructuringElementEx (int nCols, int nRows, int
    anchorX, int anchorY, CvElementShape shape, int* values);
```

nCols Number of columns in the structuring element.

nRows Number of rows in the structuring element.

anchorX Relative horizontal offset of the anchor point.

anchorY Relative vertical offset of the anchor point.

shape Shape of the structuring element; may have the following values:

- CV_SHAPE_RECT, a rectangular element;
- CV_SHAPE_CROSS, a cross-shaped element;
- CV_SHAPE_ELLIPSE, an elliptic element;
- CV_SHAPE_CUSTOM, a user-defined element. In this case the parameter *values* specifies the mask, that is, which neighbors of the pixel must be considered.

values Pointer to the structuring element data, a plane array, representing row-by-row scanning of the element matrix. Non-zero values indicate points that belong to the element. If the pointer is NULL, then all values are considered non-zero, that is, the element is of a rectangular shape. This parameter is considered only if the shape is CV_SHAPE_CUSTOM.

Discussion

The function `CreateStructuringElementEx` allocates and fills the structure `IplConvKernel`, which can be used as a structuring element in the morphological operations.

ReleaseStructuringElement

Deletes structuring element.

```
void cvReleaseStructuringElement (IplConvKernel** ppElement);
```

ppElement	Pointer to the deleted structuring element.

Discussion

The function `ReleaseStructuringElement` releases the structure `IplConvKernel` that is no longer needed. If `*ppElement` is `NULL`, the function has no effect. The function returns created structuring element.

Erode

Erodes image by using arbitrary structuring element.

```
void cvErode (IplImage* src, IplImage* dst, IplConvKernel* B, int iterations);
```

src	Source image.
dst	Destination image.
B	Structuring element used for erosion. If `NULL`, a 3x3 rectangular structuring element is used.
iterations	Number of times erosion is applied.

Discussion

The function `Erode` erodes the source image. The function takes the pointer to the structuring element, consisting of “zeros” and “minus ones”; the minus ones determine neighbors of each pixel from which the minimum is taken and put to the corresponding destination pixel. The function supports the in-place mode when the source and destination pointers are the same. Erosion can be applied several times (*iterations* parameter). Erosion on a color image means independent transformation of all the channels.

Dilate

Dilates image by using arbitrary structuring element.

```
void cvDilate (IplImage* pSrc, IplImage* pDst, IplConvKernel* B, int
    iterations);
```

pSrc	Source image.
pDst	Destination image.
B	Structuring element used for dilation. If `NULL`, a 3x3 rectangular structuring element is used.
iterations	Number of times dilation is applied.

Discussion

The function `Dilate` performs dilation of the source image. It takes pointer to the structuring element that consists of “zeros” and “minus ones”; the minus ones determine neighbors of each pixel from which the maximum is taken and put to the corresponding destination pixel. The function supports in-place mode. Dilation can be applied several times (*iterations* parameter). Dilation of a color image means independent transformation of all the channels.

MorphologyEx

Performs advanced morphological transformations.

```
void cvMorphologyEx (IplImage* src, IplImage* dst, IplImage* temp,
IplConvKernel* B, CvMorphOp op, int iterations);
```

src	Source image.
dst	Destination image.
temp	Temporary image, required in some cases.
B	Structuring element.
op	Type of morphological operation:<br>• `CV_MOP_OPEN`, opening;<br>• `CV_MOP_CLOSE`, closing;<br>• `CV_MOP_GRADIENT`, morphological gradient;<br>• `CV_MOP_TOPHAT`, top hat;<br>• `CV_MOP_BLACKHAT`, black hat.<br>(See Morphology for description of these operations).
iterations	Number of times erosion and dilation are applied during the complex operation.

Discussion

The function `MorphologyEx` performs advanced morphological transformations. The function uses `Erode` and `Dilate` to perform more complex operations. The parameter *temp* must be non-`NULL` and point to the image of the same size and format as *src* and *dst* when *op* is `CV_MOP_GRADIENT`, or when *op* is `CV_MOP_TOPHAT` or *op* is `CV_MOP_BLACKHAT` and *src* is equal to *dst* (in-place operation).

Distance Transform Function

DistTransform

Calculates distance to closest zero pixel for all non-zero pixels of source image.

```
void cvDistTransform (IplImage*  src, IplImage* dst, CvDisType disType,
    CvDisMaskType maskType, float* mask);
```

src	Source image.
dst	Output image with calculated distances.
disType	Type of distance; can be CV_DIST_L1, CV_DIST_L2, CV_DIST_C or CV_DIST_USER.
maskType	Size of distance transform mask; can be CV_DIST_MASK_3x3 or CV_DIST_MASK_5x5.
mask	Pointer to the user-defined mask used with the distance type CV_DIST_USER.

Discussion

The function `DistTransform` approximates the actual distance from the closest zero pixel with a sum of fixed distance values: two for 3x3 mask and three for 5x5 mask. Figure 10-2 shows the result of the distance transform of a 7x7 image with a zero central pixel.

Figure 10-2 3x3 Mask

4.5	4	3.5	3	3.5	4	4.5
4	3	2.5	2	2.5	3	4
3.5	2.5	1.5	1	1.5	2.5	3.5
3	2	1	0	1	2	3
3.5	2.5	1.5	1	1.5	2.5	3.5
4	3	2.5	2	2.5	3	4
4.5	4	3.5	3	3.5	4	4.5

This example corresponds to a 3x3 mask; in case of user-defined distance type the user sets the distance between two pixels, that share the edge, and the distance between the pixels, that share the corner only. For this case the values are 1 and 1.5 correspondingly. Figure 10-3 shows the distance transform for the same image, but for a 5x5 mask. For the 5x5 mask the user sets an additional distance that is the distance between pixels corresponding to the chess knight move. In this example the additional distance is equal to 2. For `CV_DIST_L1`, `CV_DIST_L2`, and `CV_DIST_C` the optimal precalculated distance values are used.

Figure 10-3 5x5 Mask

4.5	3.5	3	3	3	3.5	4
3.5	3	2	2	2	3	3.5
3	2	1.5	1	1.5	2	3
3	2	1	0	1	2	3
3	2	1.5	1	1.5	2	3
3.5	3	2	2	2	3	3.5
4	3.5	3	3	3	3.5	4

Threshold Functions

AdaptiveThreshold

Provides adaptive thresholding binary image.

```
void cvAdaptiveThreshold (IplImage* src, IplImage* dst, double max,
    CvAdaptiveThreshMethod method, CvThreshType type, double* parameters);
```

src Source image.

dst Destination image.

max	Max parameter used with the types `CV_THRESH_BINARY` and `CV_THRESH_BINARY_INV` only.
method	Method for the adaptive threshold definition; now `CV_STDDEF_ADAPTIVE_THRESH` only.
type	Thresholding type; must be one of

- `CV_THRESH_BINARY`, $val = (val > Thresh?\mathrm{MAX}:0)$;
- `CV_THRESH_BINARY_INV`, $val = (val > Thresh?0:\mathrm{MAX})$;
- `CV_THRESH_TOZERO`, $val = (val > Thresh?val:0)$;
- `CV_THRESH_TOZERO_INV`, $val = (val > Thresh?0:val)$.

parameters	Pointer to the list of method-specific input parameters. For the method `CV_STDDEF_ADAPTIVE_THRESH` the value *parameters*`[0]` is the size of the neighborhood: 1-`(3x3)`, 2-`(5x5)`, or 3-`(7x7)`, and *parameters*`[1]` is the value of the minimum variance.

Discussion

The function `AdaptiveThreshold` calculates the adaptive threshold for every input image pixel and segments image. The algorithm is as follows.

Let $\{f_{ij}\}, 1 \le i \le l, 1 \le j \le J$ be the input image. For every pixel i, j the mean m_{ij} and variance v_{ij} are calculated as follows:

$$m_{ij} = 1/2p \cdot \sum_{s=-p}^{p} \sum_{t=-p}^{p} f_{i+s,j+t},\ v_{ij} = 1/2p \cdot \sum_{s=-p}^{p} \sum_{t=-p}^{p} \left|f_{i+s,j+t} - m_{ij}\right|,$$

where $p \times p$ is the neighborhood.

Local threshold for pixel i, j is $t_{ij} = m_{ij} + v_{ij}$ for $v_{ij} > v_{min}$, and $t_{ij} = t_{ij-1}$ for $v_{ij} \le v_{min}$, where v_{min} is the minimum variance value. If $j = 1$, then $t_{ij} = t_{i-1,j}$, $t_{11} = t_{i_0 j_0}$, where $v_{i_0 j_0} > v_{min}$ and $v_{ij} \le v_{min}$ for $(i < i_0) \vee ((i = i_0) \wedge (j < j_0))$.

Output segmented image is calculated as in the function `Threshold`.

Threshold

Thresholds binary image.

```
void cvThreshold (IplImage* src, IplImage* dst, float thresh, float maxvalue,
CvThreshType type);
```

src	Source image.
dst	Destination image; can be the same as the parameter *src*.
thresh	Threshold parameter.
maxvalue	Maximum value; parameter, used with threshold types CV_THRESH_BINARY, CV_THRESH_BINARY_INV, and CV_THRESH_TRUNC.
type	Thresholding type; must be one of

- CV_THRESH_BINARY, *val* = (*val* > *thresh* *maxvalue*:0);
- CV_THRESH_BINARY_INV, *val* = (*val* > *thresh* 0:*maxvalue*);
- CV_THRESH_TRUNC, *val* = (*val* > *thresh*?*thresh*:*maxvalue*);
- CV_THRESH_TOZERO, *val* = (*val* > *thresh* *val*:0);
- CV_THRESH_TOZERO_INV, *val* = (*val* > *thresh* 0:*val*).

Discussion

The function `Threshold` applies fixed-level thresholding to grayscale image. The result is either a grayscale image or a bi-level image. The former variant is typically used to remove noise from the image, while the latter one is used to represent a grayscale image as composition of connected components and after that build contours on the components via the function `FindContours`. Figure 10-4 illustrates meanings of different threshold types:

Figure 10-4 Meanings of Threshold Types

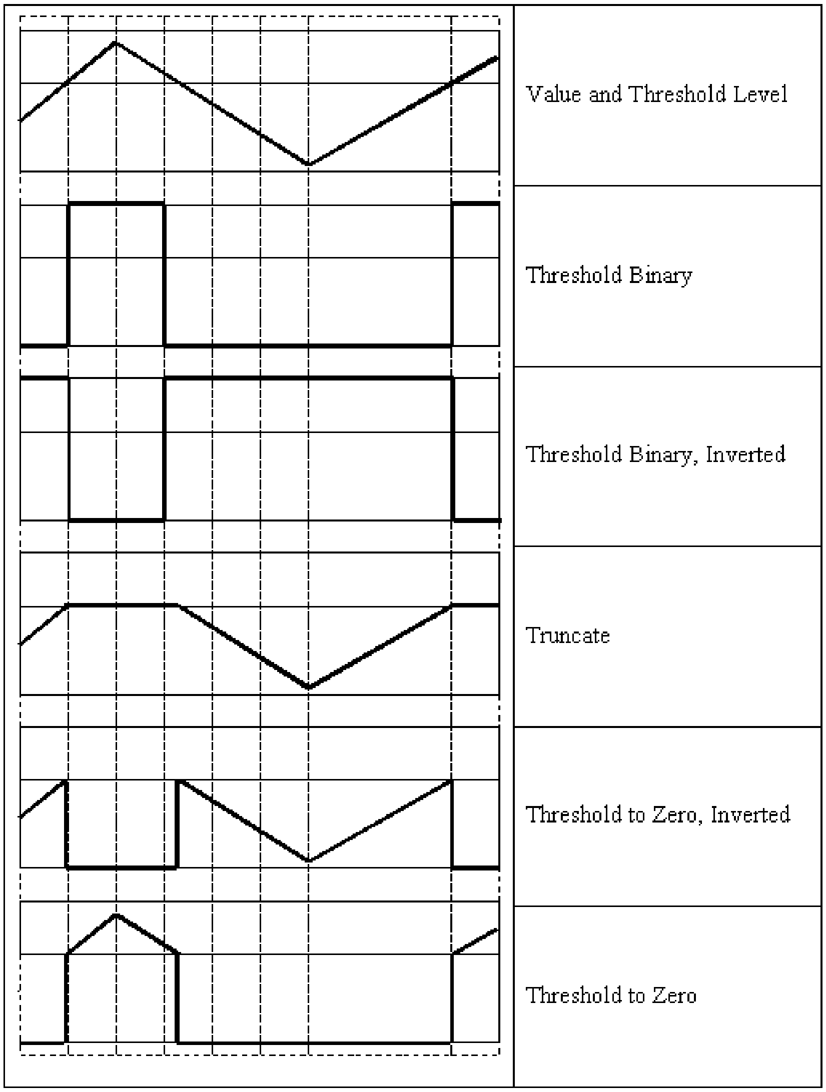

Flood Filling Function

FloodFill

Makes flood filling of image connected domain.

```
void cvFloodFill (IplImage* img, CvPoint seedPoint, double newVal, double
    loDiff, double updiff, CvConnectedComp* comp, int connectivity=4);
```

img	Input image; repainted by the function.
seedPoint	Coordinates of the seed point inside the image ROI.
newVal	New value of repainted domain pixels.
loDiff	Maximal lower difference between the values of pixel belonging to the repainted domain and one of the neighboring pixels to identify the latter as belonging to the same domain.
upDiff	Maximal upper difference between the values of pixel belonging to the repainted domain and one of the neighboring pixels to identify the latter as belonging to the same domain.
comp	Pointer to structure the function fills with the information about the repainted domain.
connectivity	Type of connectivity used within the function. If it is four, which is default value, the function tries out four neighbors of the current pixel, otherwise the function tries out all the eight neighbors.

Discussion

The function `FloodFill` fills the seed pixel neighborhoods inside which all pixel values are close to each other. The pixel is considered to belong to the repainted domain if its value *v* meets the following conditions:

$$v_0 - d_{lw} \le v \le v_0 + d_{up},$$

where v_0 is the value of at least one of the current pixel neighbors, which already belongs to the repainted domain. The function checks 4-connected neighborhoods of each pixel, that is, its side neighbors.

Histogram Functions

CreateHist

Creates histogram.

```
CvHistogram* cvCreateHist (int cDims, int* dims, CvHistType type,
float** ranges=0, int uniform=1);
```

cDims	Number of histogram dimensions.
dims	Array, elements of which are numbers of bins per each dimension.
type	Histogram representation format: `CV_HIST_ARRAY` means that histogram data is represented as an array; `CV_HIST_TREE` means that histogram data is represented as a sparse structure, that is, the balanced tree in this implementation.
ranges	*2D* array, or more exactly, an array of arrays, of bin ranges for every histogram dimension. Its meaning depends on the uniform parameter value.
uniform	Uniformity flag; if not 0, the histogram has evenly spaced bins and every element of *ranges* array is an array of two numbers: lower and upper boundaries for the corresponding histogram dimension. If the parameter is equal to 0, then i^{th} element of ranges array contains *dims[i]+1* elements: *l(0), u(0) == l(1), u(1) == l(2), ..., u(n-1)*, where *l(i)* and *u(i)* are lower and upper boundaries for the ith bin, respectively.

Discussion

The function `CreateHist` creates a histogram of the specified size and returns the pointer to the created histogram. If the array *`ranges`* is 0, the histogram bin ranges must be specified later via the function `SetHistBinRanges`.

ReleaseHist

Releases histogram header and underlying data.

```
void cvReleaseHist (CvHistogram** hist);
```

hist	Pointer to the released histogram.

Discussion

The function `ReleaseHist` releases the histogram header and underlying data. The pointer to histogram is cleared by the function. If *`*hist`* pointer is already `NULL`, the function has no effect.

MakeHistHeaderForArray

Initializes histogram header.

```
void cvMakeHistHeaderForArray (int cDims, int* dims, CvHistogram* hist,
    float* data, float** ranges=0, int uniform=1);
```

cDims	Histogram dimension number.
dims	Dimension size array.
hist	Pointer to the histogram to be created.
data	Pointer to the source data histogram.
ranges	*2D* array of bin ranges.
uniform	If not 0, the histogram has evenly spaced bins.

Discussion

The function `MakeHistHeaderForArray` initializes the histogram header and sets the data pointer to the given value *`data`*. The histogram must have the type `CV_HIST_ARRAY`. If the array *`ranges`* is 0, the histogram bin ranges must be specified later via the function `SetHistBinRanges`.

QueryHistValue_1D

Queries value of histogram bin.

```
float cvQueryHistValue_1D (CvHistogram* hist, int idx0);
```

`hist`	Pointer to the source histogram.
`idx0`	Index of the bin.

Discussion

The function `QueryHistValue_1D` returns the value of the specified bin of *1D* histogram. If the histogram representation is a sparse structure and the specified bin is not present, the function return 0.

QueryHistValue_2D

Queries value of histogram bin.

```
float cvQueryHistValue_2D (CvHistogram* hist, int idx0, int idx1);
```

`hist`	Pointer to the source histogram.
`idx0`	Index of the bin in the first dimension.
`idx1`	Index of the bin in the second dimension.

Discussion

The function `QueryHistValue_2D` returns the value of the specified bin of *2D* histogram. If the histogram representation is a sparse structure and the specified bin is not present, the function return 0.

QueryHistValue_3D

Queries value of histogram bin.

```
float cvQueryHistValue_3D (CvHistogram* hist, int idx0, int idx1, int idx2 );
```

hist	Pointer to the source histogram.
idx0	Index of the bin in the first dimension.
idx1	Index of the bin in the second dimension.
idx2	Index of the bin in the third dimension.

Discussion

The function `QueryHistValue_3D` returns the value of the specified bin of *3D* histogram. If the histogram representation is a sparse structure and the specified bin is not present, the function return 0.

QueryHistValue_nD

Queries value of histogram bin.

```
float cvQueryHistValue_nD (CvHistogram* hist, int* idx);
```

hist	Pointer to the source histogram.
idx	Array of bin indices, that is, a multi-dimensional index.

Discussion

The function `QueryHistValue_nD` returns the value of the specified bin of *nD* histogram. If the histogram representation is a sparse structure and the specified bin is not present, the function return 0. The function is the most general in the family of `QueryHistValue` functions.

GetHistValue_1D

Returns pointer to histogram bin.

```
float* cvGetHistValue_1D (CvHistogram* hist, int idx0);
```

hist	Pointer to the source histogram.
idx0	Index of the bin.

Discussion

The function `GetHistValue_1D` returns the pointer to the histogram bin, given its coordinates. If the bin is not present, it is created and initialized with 0. The function returns `NULL` pointer if the input coordinates are invalid.

GetHistValue_2D

Returns pointer to histogram bin.

```
float* cvGetHistValue_2D (CvHistogram* hist, int idx0, int idx1);
```

hist	Pointer to the source histogram.
idx0	Index of the bin in the first dimension.
idx1	Index of the bin in the second dimension.

Discussion

The function `GetHistValue_2D` returns the pointer to the histogram bin, given its coordinates. If the bin is not present, it is created and initialized with 0. The function returns `NULL` pointer if the input coordinates are invalid.

GetHistValue_3D

Returns pointer to histogram bin.

```
float* cvGetHistValue_3D (CvHistogram* hist,int idx0, int idx1, int idx2);
```

hist	Pointer to the source histogram.
idx0	Index of the bin in the first dimension.
idx1	Index of the bin in the second dimension.
idx2	Index of the bin in the third dimension.

Discussion

The function `GetHistValue_3D` returns the pointer to the histogram bin, given its coordinates. If the bin is not present, it is created and initialized with 0. The function returns `NULL` pointer if the input coordinates are invalid.

GetHistValue_nD

Returns pointer to histogram bin.

```
float* cvGetHistValue_nD (CvHistogram* hist, int* idx);
```

hist	Pointer to the source histogram.
idx	Array of bin indices, that is, a multi-dimensional index.

Discussion

The function `GetHistValue_nD` returns the pointer to the histogram bin, given its coordinates. If the bin is not present, it is created and initialized with 0. The function returns `NULL` pointer if the input coordinates are invalid.

GetMinMaxHistValue

Finds minimum and maximum histogram bins.

```
void cvGetMinMaxHistValue (CvHistogram* hist, float* minVal, float* maxVal,
    int* minIdx=0, int* maxIdx=0);
```

hist	Pointer to the histogram.
minVal	Pointer to the minimum value of the histogram; can be `NULL`.
maxVal	Pointer to the maximum value of the histogram; can be `NULL`.
minIdx	Pointer to the array of coordinates for minimum. If not `NULL`, must have *hist->c_dims* elements.
maxIdx	Pointer to the array of coordinates for maximum. If not `NULL`, must have *hist->c_dims* elements.

Discussion

The function `GetMinMaxHistValue` finds the minimum and maximum histogram bins and their positions. In case of several maximums or minimums the leftmost ones are returned.

NormalizeHist

Normalizes histogram.

```
void cvNormalizeHist (CvHistogram* hist, float factor);
```

hist	Pointer to the histogram.

`factor` Normalization factor.

Discussion

The function `NormalizeHist` normalizes the histogram, such that the sum of histogram bins becomes equal to *`factor`*.

ThreshHist

Thresholds histogram.

```
void cvThreshHist (CvHistogram* hist, float thresh);
```

`hist` Pointer to the histogram.

`thresh` Threshold level.

Discussion

The function `ThreshHist` clears histogram bins that are below the specified level.

CompareHist

Compares two histograms.

```
double cvCompareHist (CvHistogram* hist1, CvHistogram* hist2, CvCompareMethod
    method);
```

`hist1` First histogram.

`hist2` Second histogram.

`method` Comparison method; may be any of those listed below:

- `CV_COMP_CORREL`;
- `CV_COMP_CHISQR`;
- `CV_COMP_INTERSECT`.

Discussion

The function `CompareHist` compares two histograms using specified method.

CV_COMP_CORREL $result = \frac{\sum_i \hat{q}_i \hat{v}_i}{\sqrt{\sum_i \hat{q}_i^{2} * \sum_i \hat{v}_i^2}}$,

CV_COMP_CHISQR $result = \sum_i \frac{(q_i - v_i)^2}{q_i + v_i}$,

CV_COMP_INTERSECT $result = \sum_i min(q_i, v_i)$.

The function returns the comparison result.

CopyHist

Copies histogram.

```
void cvCopyHist (CvHistogram* src, CvHistogram** dst);
```

src	Source histogram.
dst	Pointer to destination histogram.

Discussion

The function `CopyHist` makes a copy of the histogram. If the second histogram pointer **dst* is null, it is allocated and the pointer is stored at **dst*. Otherwise, both histograms must have equal types and sizes, and the function simply copies the source histogram bins values to destination histogram.

SetHistBinRanges

Sets bounds of histogram bins.

```
void cvSetHistBinRanges (CvHistogram* hist, float** ranges, int uniform=1);
```

hist	Destination histogram.
ranges	*2D* array of bin ranges.
uniform	If not 0, the histogram has evenly spaced bins.

Discussion

The function `SetHistBinRanges` is a stand-alone function for setting bin ranges in the histogram. For more detailed description of the parameters *ranges* and *uniform* see `CreateHist` function, that can initialize the ranges as well. Ranges for histogram bins must be set before the histogram is calculated or backproject of the histogram is calculated.

CalcHist

Calculates histogram of image(s).

```
void cvCalcHist (IplImage** img, CvHistogram* hist, int doNotClear=0,
IplImage* mask=0);
```

img	Source images.
hist	Pointer to the histogram.
doNotClear	Clear flag.
mask	Mask; determines what pixels of the source images are considered in process of histogram calculation.

Discussion

The function `CalcHist` calculates the histogram of the array of single-channel images. If the parameter *`doNotClear`* is 0, then the histogram is cleared before calculation; otherwise the histogram is simply updated.

CalcBackProject

Calculates back project.

```
void cvCalcBackProject (IplImage** img, IplImage* dstImg, CvHistogram* hist);
```

`img`	Source images array.
`dstImg`	Destination image.
`hist`	Source histogram.

Discussion

The function `CalcBackProject` calculates the back project of the histogram. For each group of pixels taken from the same position from all input single-channel images the function puts the histogram bin value to the destination image, where the coordinates of the bin are determined by the values of pixels in this input group. In terms of statistics, the value of each output image pixel characterizes probability that the corresponding input pixel group belongs to the object whose histogram is used.

For example, to find a red object in the picture, the procedure is as follows:

1. Calculate a hue histogram for the red object assuming the image contains only this object. The histogram is likely to have a strong maximum, corresponding to red color.
2. Calculate back project using the histogram and get the picture, where bright pixels correspond to typical colors (e.g., red) in the searched object.
3. Find connected components in the resulting picture and choose the right component using some additional criteria, for example, the largest connected component.

CalcBackProjectPatch

Calculates back project patch of histogram.

```
void cvCalcBackProjectPatch (IplImage** img, IplImage* dst, CvSize patchSize,
    CvHistogram* hist, CvCompareMethod method, float normFactor);
```

img	Source images array.
dst	Destination image.
patchSize	Size of patch slid though the source image.
hist	Probabilistic model.
method	Method of comparison.
normFactor	Normalization factor.

Discussion

The function `CalcBackProjectPatch` calculates back projection by comparing histograms of the source image patches with the given histogram. Taking measurement results from some image at each location over ROI creates an array *img*. These results might be one or more of hue, *x* derivative, *y* derivative, Laplacian filter, oriented Gabor filter, etc. Each measurement output is collected into its own separate image. The *img* image array is a collection of these measurement images. A multi-dimensional histogram *hist* is constructed by sampling from the *img* image array. The final histogram is normalized. The *hist* histogram has as many dimensions as the number of elements in *img* array.

Each new image is measured and then converted into an *img* image array over a chosen ROI. Histograms are taken from this *img* image in an area covered by a “patch” with anchor at center as shown in Figure 10-5. The histogram is normalized using the parameter *norm_factor* so that it may be compared with *hist*. The calculated histogram is compared to the model histogram; *hist* uses the function `cvCompareHist` (the parameter *method*). The resulting output is placed at the location corresponding to the patch anchor in the probability image *dst*. This process is repeated as the patch is slid over the ROI. Subtracting trailing pixels covered by the patch and adding newly covered pixels to the histogram can save a lot of operations.

Figure 10-5 Back Project Calculation by Patches

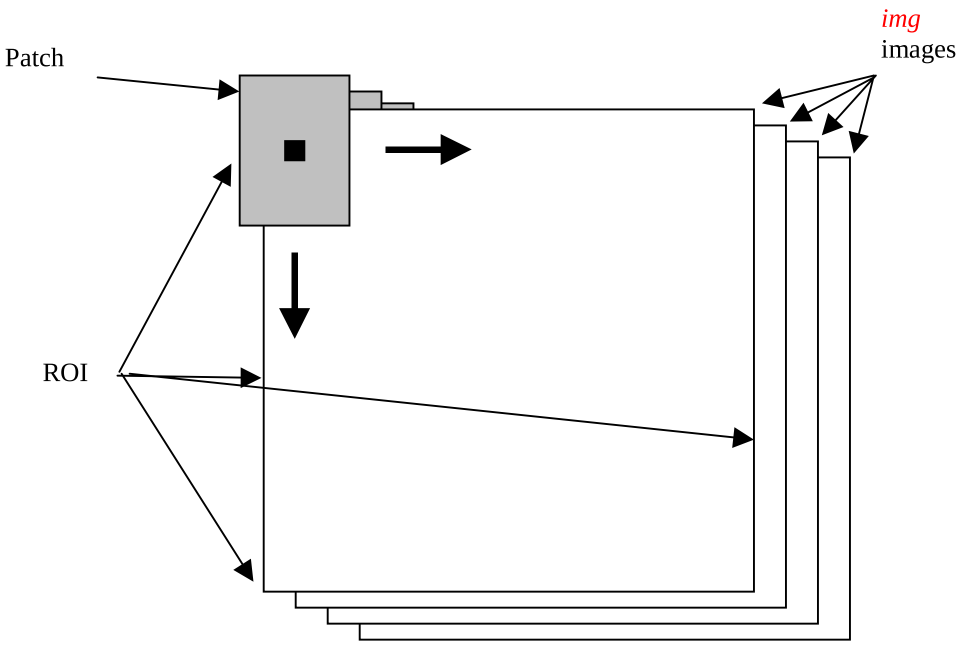

Each image of the image array `img` shown in the figure stores the corresponding element of a multi-dimensional measurement vector. Histogram measurements are drawn from measurement vectors over a patch with anchor at the center. A multi-dimensional histogram `hist` is used via the function `CompareHist` to calculate the output at the patch anchor. The patch is slid around until the values are calculated over the whole ROI.

CalcEMD

Computes earth mover distance.

```
void cvCalcEMD (float* signature1, int size1, float* signature2, int size2, int
    dims, CvDisType distType, float *distFunc (float* f1, float* f2, void*
    userParam), float* emd, float* lowerBound, void* userParam);
```

signature1	First signature, array of `size1 * (dims + 1)` elements.
size1	Number of elements in the first compared signature.
signature2	Second signature, array of `size2 * (dims + 1)` elements.
size2	Number of elements in the second compared signature.
dims	Number of dimensions in feature space.
distType	Metrics used; `CV_DIST_L1`, `CV_DIST_L2`, and `CV_DIST_C` stand for one of the standard metrics. `CV_DIST_USER` means that a user-defined function is used as the metric. The function takes two coordinate vectors and user parameter and returns the distance between two vectors.
distFunc	Pointer to the user-defined ground distance function if *distType* is `CV_DIST_USER`.
emd	Pointer to the calculated *emd* distance.
lowerBound	Pointer to the calculated lower boundary.
userParam	Pointer to optional data that is passed into the distance function.

Discussion

The function `CalcEMD` computes earth mover distance and/or a lower boundary of the distance. The lower boundary can be calculated only if *dims* > 0, and it has sense only if the metric used satisfies all metric axioms. The lower boundary is calculated very fast and can be used to determine roughly whether the two signatures are far enough so that they cannot relate to the same object. If the parameter *dims* is equal to 0, then *signature1* and *signature2* are considered simple *1D* histograms. Otherwise, both signatures must look as follows:

(*weight_i0*, *x0_i0*, *x1_i0*, . . ., *x*(*dims*-1)*_i0*,

weight_i1, *x0_i1*, *x1_i1*, . . ., *x*(*dims*-1)_i1,

…

*weight*_(*size1*-1), *x0*_(*size1*-1), *x1*_(*size1*-1, . . ., *x*(*dims*-1)_(*size1*-1)),

where *weight_ik* is the weight of *ik cluster*, while *x0_ik*,..., *x*(*dims*-1)*_ik* are coordinates of the cluster *ik*.

If the parameter *lower_bound* is equal to 0, only *emd* is calculated. If the calculated lower boundary is greater than or equal to the value stored at this pointer, then the true *emd* is not calculated, but is set to that *lower_bound*.

CalcContrastHist

Calculates histogram of contrast.

```
void cvCalcContrastHist (IplImage **src, CvHistogram* hist, int dontClear,
    IplImage* mask);
```

src	Pointer to the source images, (now only *src[0]* is used).
hist	Destination histogram.
dontClear	Clear flag.
mask	Mask image.

Discussion

The function `CalcContrastHist` calculates a histogram of contrast for the one-channel image. If *dont_clear* parameter is 0 then the histogram is cleared before calculation, otherwise it is simply updated. The algorithm works as follows. Let *S* be a set of pairs (x_1, x_2) of neighbor pixels in the image $f(x)$ and

$S(t) = \{(x_1,x_2) \in S, f(x_1) \le t < f(x_2) \vee f(x_2) \le t < f(x_1)\}$.

Let's denote

$\{G_t\}$	as the destination histogram,

E_t as the summary contrast corresponding to the threshold *t*,

N_t as the counter of the edges detected by the threshold *t*.

Then

$$N_t = \|S(t)\|, E_t = \sum_{(x_1,x_2) \in S(t)} C(x_1,x_2,t),$$

where $C(x_1,x_2,t) = min\{|f(x_1) - t|,|f(x_2) - t|\}$ and the resulting histogram is calculated as

$$G_t = \begin{cases} E_t/N_t, N_t \neq 0, \\ 0, N_t = 0. \end{cases}$$

If pointer to the mask is NULL, the histogram is calculated for the all image pixels. Otherwise only those pixels are considered that have non-zero value in the mask in the corresponding position.

Pyramid Data Types

The pyramid functions use the data structure `IplImage` for image representation and the data structure `CvSeq` for the sequence of the connected components representation. Every element of this sequence is the data structure `CvConnectedComp` for the single connected component representation in memory.

The *C* language definition for the `CvConnectedComp` structure is given below.

Example 10-1 `CvConnectedComp`

```
typedef struct CvConnectedComp
{
    double area;           /*  area of the segmented
                               component   */
    float value;           /*  gray scale value of the
                               segmented component   */
    CvRect rect;         /*  ROI of the segmented component
                         */
} CvConnectedComp;
```

Histogram Data Types

Example 10-2 `CvHistogram`

```
typedef struct CvHistogram
{
    int     header_size;  /* header's size         */
    CvHistType type;      /* type of histogram     */
    int     flags;        /* histogram’s flags     */
    int     c_dims;       /* histogram’s dimension */
    int     dims[CV_HIST_MAX_DIM];
                          /* every dimension size  */
    int     mdims[CV_HIST_MAX_DIM];
                         /* coefficients for fast
                            access to element      */
                         /* &m[a,b,c] = m + a*mdims[0] +
                            b*mdims[1] + c*mdims[2] */
    float*  thresh[CV_HIST_MAX_DIM];
                         /* bin boundaries arrays for every
                            dimension */
    float*  array; /* all the histogram data, expanded into
                      the single row */
    struct  CvNode* root; /* tree – histogram data */
    CvSet*          set;  /* pointer to memory storage
                             (for tree data) */
    int*  chdims[CV_HIST_MAX_DIM];
                          /* cache data for fast calculating */
} CvHistogram;
```

Structural Analysis Reference

11

Table 11-1 Structural Analysis Functions

Group	Name	Description
	**Functions**	
Contour Processing Functions	`ApproxChains`	Approximates Freeman chain(s) with a polygonal curve.
	`StartReadChainPoints`	Initializes the chain reader.
	`ReadChainPoint`	Returns the current chain point and moves to the next point.
	`ApproxPoly`	Approximates one or more contours with desired precision.
	`DrawContours`	Draws contour outlines in the image.
	`ContourBoundingRect`	Calculates the bounding box of the contour.
	`ContoursMoments`	Calculates unnormalized spatial and central moments of the contour up to order 3.
	`ContourArea`	Calculates the region area within the contour or contour section.

Table 11-1 Structural Analysis Functions (continued)

Group	Name	Description
	MatchContours	Calculates one of the three similarity measures between two contours.
	CreateContourTree	Creates binary tree representation for the input contour and returns the pointer to its root.
	ContourFromContourTree	Restores the contour from its binary tree representation.
	MatchContourTrees	Calculates the value of the matching measure for two contour trees.
Geometry Functions	FitEllipse	Fits an ellipse to a set of *2D* points.
	FitLine2D	Fits a *2D* line to a set of points on the plane.
	FitLine3D	Fits a *3D* line to a set of points on the plane.
	Project3D	Provides a general way of projecting a set of *3D* points to a *2D* plane.
	ConvexHull	Finds the convex hull of a set of points.
	ContourConvexHull	Finds the convex hull of a set of points returning `cvSeq.`
	ConvexHullApprox	Finds approximate convex hull of a set of points.
	ContourConvexHullApprox	Finds approximate convex hull of a set of points returning `cvSeq.`

Table 11-1 Structural Analysis Functions (continued)

Group	Name	Description
	CheckContourConvexity	Tests whether the input is a contour convex or not.
	ConvexityDefects	Finds all convexity defects of the input contour.
	MinAreaRect	Finds a circumscribed rectangle of the minimal area for a given convex contour.
	CalcPGH	Calculates a pair-wise geometrical histogram for the contour.
	MinEnclosingCircle	Finds the minimal enclosing circle for the planar point set.
	**Data Types**	
Contour Processing Data Types	CvContourTree	Represents the contour binary tree in memory.
Geometry Data Types	CvConvexityDefect	Represents the convexity defect.

Contour Processing Functions

ApproxChains

Approximates Freeman chain(s) with polygonal curve.

```
CvSeq* cvApproxChains( CvSeq* srcSeq, CvMemStorage* storage,
    CvChainApproxMethod method=CV_CHAIN_APPROX_SIMPLE,
    float parameter=0,int  minimalPerimeter=0,
    int  recursive=0);
```

srcSeq	Pointer to the chain that can refer to other chains.
storage	Storage location for the resulting polylines.
method	Approximation method (see the description of the function FindContours).
parameter	Method parameter (not used now).
minimalPerimeter	Approximates only those contours whose perimeters are not less than *minimalPerimeter*. Other chains are removed from the resulting structure.
recursive	If not 0, the function approximates all chains that access can be obtained to from *srcSeq* by *h_next* or *v_next* links. If 0, the single chain is approximated.

Discussion

This is a stand-alone approximation routine. The function `ApproxChains` works exactly in the same way as the functions `FindContours` / `FindNextContour` with the corresponding approximation flag. The function returns pointer to the first resultant contour. Other contours, if any, can be accessed via *v_next* or *h_next* fields of the returned structure.

StartReadChainPoints

Initializes chain reader.

```
void cvStartReadChainPoints( CvChain* chain, CvChainPtReader* reader );
```

chain	Pointer to chain.
reader	Chain reader state.

Discussion

The function `StartReadChainPoints` initializes a special reader (see Dynamic Data Structures for more information on sets and sequences).

ReadChainPoint

Gets next chain point.

```
CvPoint cvReadChainPoint( CvChainPtReader* reader );
```

reader Chain reader state.

Discussion

The function `ReadChainPoint` returns the current chain point and moves to the next point.

ApproxPoly

Approximates polygonal contour(s) with desired precision.

```
CvSeq* cvApproxPoly( CvSeq* srcSeq, int headerSize, CvMemStorage* storage,
    CvPolyApproxMethod method, float parameter,int recursive=0 );
```

srcSeq Pointer to the contour that can refer to other chains.

headerSize Size of the header for resulting sequences.

storage Resulting contour storage location.

method Approximation method; only `CV_POLY_APPROX_DP` is supported, that corresponds to Douglas-Peucker method.

parameter Method-specific parameter; a desired precision for `CV_POLY_APPROX_DP`.

recursive If not 0, the function approximates all contours that can be accessed from *srcSeq* by *h_next* or *v_next* links. If 0, the single contour is approximated.

Discussion

The function `ApproxPoly` approximates one or more contours and returns pointer to the first resultant contour. Other contours, if any, can be accessed via *`v_next`* or *`h_next`* fields of the returned structure.

DrawContours

Draws contours in image.

```
void cvDrawContours( IplImage *img, CvSeq* contour, int externalColor, int
holeColor, int maxLevel, int thickness=1 );
```

img	Image where the contours are to be drawn. Like in any other drawing function, every output is clipped with the ROI.
contour	Pointer to the first contour.
externalColor	Color to draw external contours with.
holeColor	Color to draw holes with.
maxLevel	Maximal level for drawn contours. If 0, only the contour is drawn. If 1, the contour and all contours after it on the same level are drawn. If 2, all contours after and all contours one level below the contours are drawn, etc.
thickness	Thickness of lines the contours are drawn with.

Discussion

The function `DrawContours` draws contour outlines in the image if the thickness is positive or zero or fills area bounded by the contours if thickness is negative, for example, if *`thickness`*`==CV_FILLED`.

ContourBoundingRect

Calculates bounding box of contour.

```
CvRect* rect cvContourBoundingRect (CvSeq* contour, int update);
```

`contour`	Pointer to the source contour.
`update`	Attribute of the bounding rectangle updating.

Discussion

The function `ContourBoundingRect` returns the bounding box parameters, that is, co-ordinates of the top-left corner, width, and height, of the source contour as Figure 11-1 shows. If the parameter `update` is not equal to 0, the parameters of the bounding box are updated.

Figure 11-1 Bounding Box Parameters

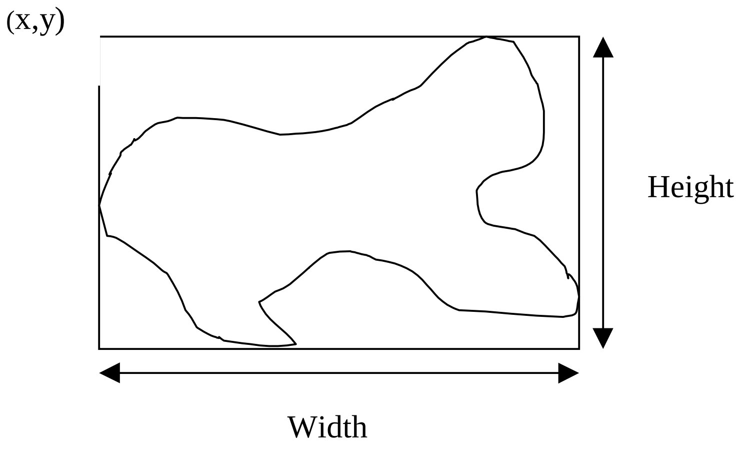

ContoursMoments

Calculates contour moments up to order 3.

```
void cvContoursMoments(CvSeq* contour, CvMoments* moments);
```

contour	Pointer to the input contour header.
moments	Pointer to the output structure of contour moments; must be allocated by the caller.

Discussion

The function `ContoursMoments` calculates unnormalized spatial and central moments of the contour up to order 3.

ContourArea

Calculates region area inside contour or contour section.

```
double cvContourSecArea(CvSeq* contour, CvSlice slice=CV_WHOLE_SEQ(seq));
```

contour	Pointer to the input contour header.
slice	Starting and ending points of the contour section of interest.

Discussion

The function `ContourSecArea` calculates the region area within the contour consisting of n points $p_i = (x_i, y_i)$, $0 \le i \le n$, $p_0 = p_n$, as a spatial moment:

$$\alpha_{00} = 1/2 \sum_{i=1}^{n} x_{i-1} y_i - x_i y_{i-1} .$$

If a part of the contour is selected and the chord, connecting ending points, intersects the contour in several places, then the sum of all subsection areas is calculated. If the input contour has points of self-intersection, the region area within the contour may be calculated incorrectly.

MatchContours

Matches two contours.

```
double cvMatchContours (CvSeq *contour1, CvSeq* contour2,int method, long
    parameter=0 );
```

contour1	Pointer to the first input contour header.
contour2	Pointer to the second input contour header.
parameter	Method-specific parameter, currently ignored.
method	Method for the similarity measure calculation; must be any of

- CV_CONTOURS_MATCH_I1;
- CV_CONTOURS_MATCH_I2;
- CV_CONTOURS_MATCH_I3.

Discussion

The function `MatchContours` calculates one of the three similarity measures between two contours.

Let two closed contours A and B have n and m points respectively:

$A = \{(x_i, y_i), 1 \le i \le n\}$ $B = \{(u_i, v_i), 1 \le i \le m\}$. Normalized central moments of a contour may be denoted as $\eta_{pq}, 0 \le p + q \le 3$. M. Hu has shown that a set of the next seven features derived from the second and third moments of contours is an invariant to translation, rotation, and scale change [Hu62].

$$h_1 = \eta_{20} + \eta_{02} ,$$

$$h_2 = (\eta_{20} - \eta_{02})^2 + 4\eta_{11}^2 ,$$

$$h_3 = (\eta_{30} - 3\eta_{12})^2 + (3\eta_{21} - \eta_{03})^2 ,$$

$$h_4 = (\eta_{30} + \eta_{12})^2 + (\eta_{21} + \eta_{03})^2 ,$$

$$h_5 = (\eta_{30} - 3\eta_{12})(\eta_{30} + \eta_{12})[(\eta_{30} + \eta_{12})^2 - 3(\eta_{21} + \eta_{03})^2] + (3\eta_{21} - \eta_{03})(\eta_{21} + \eta_{03})[3(\eta_{30} + \eta_{12})^2 - (\eta_{21} + \eta_{03})^2],$$

$$h_6 = (\eta_{20} - \eta_{02})[(\eta_{30} + \eta_{12})^2 - (\eta_{21} + \eta_{03})^2] + 4\eta_{11}(\eta_{30} + \eta_{12})(\eta_{21} + \eta_{03}) ,$$

$$h_7 = (3\eta_{21} - \eta_{03})(\eta_{30} + \eta_{12})[(\eta_{30} + \eta_{12})^2 - 3(\eta_{21} + \eta_{03})^2] + -(\eta_{30} - 3\eta_{12})(\eta_{21} + \eta_{03})\left[3(\eta_{30} + \eta_{12})^2 - (\eta_{21} + \eta_{03})^2\right].$$

From these seven invariant features the three similarity measures I_1, I_2, and I_3 may be calculated:

$$I_1(A, B) = \sum_{i=1}^{7} \left|-1/m_i^A + 1/m_i^B\right| ,$$

$$I_2(A, B) = \sum_{i=1}^{7} \left|-m_i^A + m_i^B\right| ,$$

$$I_3(A, B) = \max_i \left|(m_i^A - m_i^B)/m_i^A\right| ,$$

where $m_i^A = \mathrm{sgn}(h_i^A)\log_{10}\left|h_i^A\right|$, $m_i^B = \mathrm{sgn}(h_i^B)\log_{10}\left|h_i^B\right|$.

CreateContourTree

Creates binary tree representation for input contour.

```
CvContourTree* cvCreateContourTree(CvSeq *contour, CvMemStorage* storage,
    double threshold);
```

contour	Pointer to the input contour header.
storage	Pointer to the storage block.
threshold	Value of the threshold.

Discussion

The function `CreateContourTree` creates binary tree representation for the input contour *`contour`* and returns the pointer to its root. If the parameter *`threshold`* is less than or equal to 0, the function creates full binary tree representation. If the threshold is more than 0, the function creates representation with the precision *`threshold`*: if the vertices with the interceptive area of its base line are less than *`threshold`*, the tree should not be built any further. The function returns created tree.

ContourFromContourTree

Restores contour from binary tree representation.

```
CvSeq* cvContourFromContourTree (CvContourTree *tree, CvMemStorage* storage,
    CvTermCriteria criteria);
```

`tree`	Pointer to the input tree.
`storage`	Pointer to the storage block.
`criteria`	Criteria for the definition of the threshold value for contour reconstruction (level of precision).

Discussion

The function `ContourFromContourTree` restores the contour from its binary tree representation. The parameter *`criterion`* defines the threshold, that is, level of precision for the contour restoring. If *`criterion.type`* `= CV_TERMCRIT_ITER`, the function restores *`criterion. maxIter`* tree levels. If *`criterion.type`* `= CV_TERMCRIT_EPS`, the function restores the contour as long as *`tri_area`* > *`criterion. epsilon *contour_area`*, where *`contour_area`* is the magnitude of the contour area and *`tri_area`* is the magnitude of the current triangle area. If *`criterion.type`* `= CV_TERMCRIT_EPS + CV_TERMCRIT_ITER`, the function restores the contour as long as one of these conditions is true. The function returns reconstructed contour.

MatchContourTrees

Compares two binary tree representations.

```
double cvMatchContourTrees (CvContourTree *tree1, CvContourTree *tree2,
    CvTreeMatchMethod method, double threshold);
```

tree1	Pointer to the first input tree.
tree2	Pointer to the second input tree.
method	Method for calculation of the similarity measure; now must be only `CV_CONTOUR_TREES_MATCH_I1`.
threshold	Value of the compared threshold.

Discussion

The function `MatchContourTrees` calculates the value of the matching measure for two contour trees. The similarity measure is calculated level by level from the binary tree roots. If the total calculating value of the similarity for levels from 0 to the specified one is more than the parameter *threshold*, the function stops calculations and value of the total similarity measure is returned as *result*. If the total calculating value of the similarity for levels from 0 to the specified one is less than or equal to *threshold*, the function continues calculation on the next tree level and returns the value of the total similarity measure for the binary trees.

Geometry Functions

FitEllipse

Fits ellipse to set of 2D points.

```
void cvFitEllipse (CvPoint32f* points, int n, CvBox2D* box);
```

points	Pointer to the set of *2D* points.

n	Number of points; must be more than or equal to 6.
box	Pointer to the structure for representation of the output ellipse.

Discussion

The function `FitEllipse` fills the output structure in the following way:

box→center	Point of the center of the ellipse;
box→size	Sizes of two ellipse axes;
box→angle	Angle between the horizontal axis and the ellipse axis with the length of *box->size.width*.

The output ellipse has the property of *box→size.width* > *box→size.height*.

FitLine2D

Fits 2D line to set of points on the plane.

```
void cvFitLine2D ( CvPoint2D32f* points, int count, CvDisType disType, void*
    param, float reps, float aeps, float* line);
```

points	Array of *2D* points.
count	Number of points.
disType	Type of the distance used to fit the data to a line.
param	Pointer to a user-defined function that calculates the weights for the type `CV_DIST_USER`, or the pointer to a float user-defined metric parameter *c* for the Fair and Welsch distance types.
reps, *aeps*	Used for iteration stop criteria. If zero, the default value of 0.01 is used.
line	Pointer to the array of four floats. When the function exits, the first two elements contain the direction vector of the line normalized to 1, the other two contain coordinates of a point that belongs to the line.

Discussion

The function `FitLine2D` fits a *2D* line to a set of points on the plane. Possible distance type values are listed below.

CV_DIST_L2	Standard least squares $\rho(x) = x^2$.
CV_DIST_L1	
CV_DIST_L12	
CV_DIST_FAIR	c =1.3998.
CV_DIST_WELSCH	$\rho(x) = \frac{c^2}{2}\left[1 - \exp\left(-\left(\frac{x}{c}\right)^2\right)\right]$, c = 2.9846.
CV_DIST_USER	Uses a user-defined function to calculate the weight. The parameter *param* should point to the function.

The line equation is $[\vec{V} \times (\vec{r} - \vec{r}_0)] = 0$, where $\vec{V} = (line[0], line[1], line[2])$, $|\vec{V}| = 1$ and $\vec{r}_0 = (line[3], line[4], line[5])$.

In this algorithm $\vec{r}_0$ is the mean of the input vectors with weights, that is,

$$\vec{r}_0 = \frac{\sum_i W(d(\vec{r}_i))\vec{r}_i}{\sum_i W(d(\vec{r}_i))}.$$

The parameters *reps* and *aeps* are iteration thresholds. If the distance of the type `CV_DIST_C` between two values of $\vec{r}_0$ calculated from two iterations is less than the value of the parameter *reps* and the angle in radians between two vectors $\vec{V}$ is less than the parameter *aeps*, then the iteration is stopped.

The specification for the user-defined weight function is

```
void userWeight ( float* dist, int count, float* w );
```

dist	Pointer to the array of distance values.
count	Number of elements.
w	Pointer to the output array of weights.

The function should fill the weights array with values of weights calculated from the distance values $w[i] = f(d[i])$. The function $f(x) = \frac{1}{x}\frac{d\rho}{dx}$ has to be monotone decreasing.

FitLine3D

Fits 3D line to set of points in 3D space.

```
void cvFitLine3D ( CvPoint3D32f*  points, int count, CvDisType disType, void*
    param, float reps, float aeps, float* line);
```

points	Array of *3D* points.
count	Number of points.
disType	Type of the distance used to fit the data to a line.
param	Pointer to a user-defined function that calculates the weights for the type `CV_DIST_USER` or the pointer to a float user-defined metric parameter *c* for the Fair and Welsch distance types.
reps, aeps	Used for iteration stop criteria. If zero, the default value of 0.01 is used.
line	Pointer to the array of 6 floats. When the function exits, the first three elements contain the direction vector of the line normalized to 1, the other three contain coordinates of a point that belongs to the line.

Discussion

The function `FitLine3D` fits a *3D* line to a set of points on the plane. Possible distance type values are listed below.

`CV_DIST_L2`	Standard least squares $\rho(x) = x^2$.
`CV_DIST_L1`	
`CV_DIST_L12`	
`CV_DIST_FAIR`	c =1.3998.
`CV_DIST_WELSCH`	$\rho(x) = \frac{c^2}{2}\left[1 - \exp\left(-\left(\frac{x}{c}\right)^2\right)\right]$, c = 2.9846.
`CV_DIST_USER`	Uses a user-defined function to calculate the weight. The parameter *param* should point to the function.

The line equation is $[\vec{V} \times (\vec{r} - \vec{r_0})] = 0$, where $\vec{V} = (line[0], line[1], line[2])$, $\|\vec{V}\| = 1$ and $= (line[3], line[4], line[$[illegible].

In this algorithm $\vec{r_0}$ is the mean of the input vectors with weights, that is,

$$\vec{r_0} = \frac{\sum_i W(d(\vec{r_i}))\vec{r_i}}{\sum_i W(d(\vec{r_i}))}.$$

The parameters *reps* and *aeps* are iteration thresholds. If the distance between two values of $\vec{r_0}$ calculated from two iterations is less than the value of the parameter *reps*, (the distance type `CV_DIST_C` is used in this case) and the angle in radians between two vectors $\vec{V}$ is less than the parameter *aeps*, then the iteration is stopped.

The specification for the user-defined weight function is

```
void userWeight ( float* dist, int count, float* w );
```

dist	Pointer to the array of distance values.
count	Number of elements.
w	Pointer to the output array of weights.

The function should fill the weights array with values of weights calculated from distance values $w[i] = f(d[i])$. The function $f(x) = \frac{1}{x}\frac{d\rho}{dx}$ has to be monotone decreasing.

Project3D

Projects array of 3D points to coordinate axis.

```
void cvProject3D ( CvPoint3D32f*  points3D, int count, CvPoint2D32f* points2D,
    int xindx, int yindx);
```

points3D	Source array of *3D* points.
count	Number of points.
points2D	Target array of *2D* points.

`xindx` Index of the *3D* coordinate from 0 to 2 that is to be used as `x`-coordinate.

`yindx` Index of the *3D* coordinate from 0 to 2 that is to be used as `y`-coordinate.

Discussion

The function `Project3D` used with the function `PerspectiveTransform` is intended to provide a general way of projecting a set of *3D* points to a *2D* plane. The function copies two of the three coordinates specified by the parameters `xindx` and `yindx` of each *3D* point to a *2D* points array.

ConvexHull

Finds convex hull of points set.

```
void cvConvexHull( CvPoint* points, int numPoints, CvRect* boundRect, int
    orientation, int* hull, int* hullsize );
```

`points`	Pointer to the set of *2D* points.
`numPoints`	Number of points.
`boundRect`	Pointer to the bounding rectangle of points set; not used.
`orientation`	Output order of the convex hull vertices `CV_CLOCKWISE` or `CV_COUNTER_CLOCKWISE`.
`hull`	Indices of convex hull vertices in the input array.
`hullsize`	Number of vertices in convex hull; output parameter.

Discussion

The function `ConvexHull` takes an array of points and puts out indices of points that are convex hull vertices. The function uses Quicksort algorithm for points sorting.

The standard, that is, bottom-left `XY` coordinate system, is used to define the order in which the vertices appear in the output array.

ContourConvexHull

Finds convex hull of points set.

```
CvSeq* cvContourConvexHull( CvSeq* contour, int orientation,
    CvMemStorage*  storage );
```

`contour` Sequence of *2D* points.

`orientation` Output order of the convex hull vertices `CV_CLOCKWISE` or `CV_COUNTER_CLOCKWISE`.

`storage` Memory storage where the convex hull must be allocated.

Discussion

The function `ContourConvexHull` takes an array of points and puts out indices of points that are convex hull vertices. The function uses Quicksort algorithm for points sorting.

The standard, that is, bottom-left *XY* coordinate system, defines the order in which the vertices appear in the output array.

The function returns `CvSeq` that is filled with pointers to those points of the source contour that belong to the convex hull.

ConvexHullApprox

Finds approximate convex hull of points set.

```
void cvConvexHullApprox( CvPoint* points, int numPoints, CvRect* boundRect,
    int bandWidth,int orientation, int* hull, int* hullsize );
```

`points` Pointer to the set of *2D* points.

`numPoints` Number of points.

`boundRect` Pointer to the bounding rectangle of points set; not used.

`bandWidth` Width of band used by the algorithm.

orientation	Output order of the convex hull vertices `CV_CLOCKWISE` or `CV_COUNTER_CLOCKWISE`.
hull	Indices of convex hull vertices in the input array.
hullsize	Number of vertices in the convex hull; output parameter.

Discussion

The function `ConvexHullApprox` finds approximate convex hull of points set. The following algorithm is used:

1. Divide the plane into vertical bands of specified width, starting from the extreme left point of the input set.
2. Find points with maximal and minimal vertical coordinates within each band.
3. Exclude all the other points.
4. Find the exact convex hull of all the remaining points (see Figure 11-2).

Figure 11-2 Finding Approximate Convex Hull

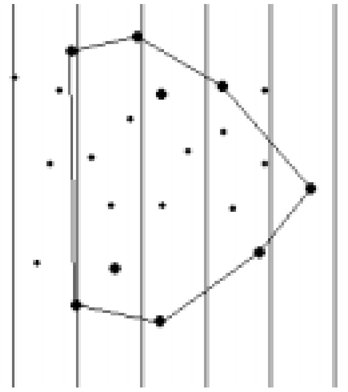

The algorithm can be used to find the exact convex hull; the value of the parameter *bandwidth* must then be equal to 1.

ContourConvexHullApprox

Finds approximate convex hull of points set.

```
CvSeq* cvContourConvexHullApprox( CvSeq* contour, int bandwidth, int
orientation, CvMemStorage*  storage );
```

contour	Sequence of *2D* points.
bandwidth	Bandwidth used by the algorithm.
orientation	Output order of the convex hull vertices `CV_CLOCKWISE` or `CV_COUNTER_CLOCKWISE`.
storage	Memory storage where the convex hull must be allocated.

Discussion

The function `ContourConvexHullApprox` finds approximate convex hull of points set. The following algorithm is used:

1. Divide the plane into vertical bands of specified width, starting from the extreme left point of the input set.
2. Find points with maximal and minimal vertical coordinates within each band.
3. Exclude all the other points.
4. Find the exact convex hull of all the remaining points (see Figure 11-2)

In case of points with integer coordinates, the algorithm can be used to find the exact convex hull; the value of the parameter *bandwidth* must then be equal to 1.

The function `ContourConvexHullApprox` returns `CvSeq` that is filled with pointers to those points of the source contour that belong to the approximate convex hull.

CheckContourConvexity

Tests contour convex.

```
int cvCheckContourConvexity( CvSeq* contour );
```

contour Tested contour.

Discussion

The function `CheckContourConvexity` tests whether the input is a contour convex or not. The function returns 1 if the contour is convex, 0 otherwise.

ConvexityDefects

Finds defects of convexity of contour.

```
CvSeq* cvConvexityDefects( CvSeq* contour, CvSeq* convexhull, CvMemStorage*
    storage );
```

contour Input contour, represented by a sequence of *CvPoint* structures.

convexhull Exact convex hull of the input contour; must be computed by the function `cvContourConvexHull`.

storage Memory storage where the sequence of convexity defects must be allocated.

Discussion

The function `ConvexityDefects` finds all convexity defects of the input contour and returns a sequence of the `CvConvexityDefect` structures.

MinAreaRect

Finds circumscribed rectangle of minimal area for given convex contour.

```
void cvMinAreaRect ( CvPoint* points, int n, int left, int bottom, int right,
    int top, CvPoint2D32f* anchor, CvPoint2D32f* vect1, CvPoint2D32f* vect2 );
```

points	Sequence of convex polygon points.
n	Number of input points.
left	Index of the extreme left point.
bottom	Index of the extreme bottom point.
right	Index of the extreme right point.
top	Index of the extreme top point.
anchor	Pointer to one of the output rectangle corners.
vect1	Pointer to the vector that represents one side of the output rectangle.
vect2	Pointer to the vector that represents another side of the output rectangle.

Discussion

The function `MinAreaRect` returns a circumscribed rectangle of the minimal area. The output parameters of this function are the corner of the rectangle and two incident edges of the rectangle (see Figure 11-3).

Figure 11-3 Minimal Area Bounding Rectangle

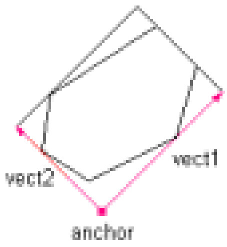

CalcPGH

Calculates pair-wise geometrical histogram for contour.

```
void cvCalcPGH( CvSeq* contour, CvHistogram* hist );
```

contour	Input contour.
hist	Calculated histogram; must be two-dimensional.

Discussion

The function `CalcPGH` calculates a pair-wise geometrical histogram for the contour. The algorithm considers every pair of the contour edges. The angle between the edges and the minimum/maximum distances are determined for every pair. To do this each of the edges in turn is taken as the base, while the function loops through all the other edges. When the base edge and any other edge are considered, the minimum and

maximum distances from the points on the non-base edge and line of the base edge are selected. The angle between the edges defines the row of the histogram in which all the bins that correspond to the distance between the calculated minimum and maximum distances are incremented. The histogram can be used for contour matching.

MinEnclosingCircle

Finds minimal enclosing circle for 2D-point set.

```
void cvFindMinEnclosingCircle (CvSeq* seq, CvPoint2D32f* center, float*
    radius);
```

seq	Sequence that contains the input point set. Only points with integer coordinates (`CvPoint`) are supported.
center	Output parameter. The center of the enclosing circle.
radius	Output parameter. The radius of the enclosing circle.

Discussion

The function `FindMinEnclosingCircle` finds the minimal enclosing circle for the planar point set. *Enclosing* means that all the points from the set are either inside or on the boundary of the circle. *Minimal* means that there is no enclosing circle of a smaller radius.

Contour Processing Data Types

The OpenCV Library functions use special data structures to represent the contours and contour binary tree in memory, namely the structures `CvSeq` and `CvContourTree`. Below follows the definition of the structure `CvContourTree` in the C language.

Example 11-1 `CvContourTree`

```
typedef struct CvContourTree
{ CV_SEQUENCE_FIELDS()
  CvPoint p1;            /*the start point of the binary tree
                    root*/
  CvPoint p2;            /*the end point of the binary tree
```

Example 11-1 CvContourTree (continued)

```
                           root*/
} CvContourTree;
```

Geometry Data Types

Example 11-2 CvConvexityDefect

```
typedef struct
{
    CvPoint* start;       //start point of defect
    CvPoint* end;         //end point of defect
    CvPoint* depth_point; //fathermost point
    float    depth;       //depth of defect
} CvConvexityDefect;
```

Object Recognition Reference

12

Table 12-1 Image Recognition Functions and Data Types

Group	Function Name	Description
	**Functions**	
Eigen Objects Functions	`CalcCovarMatrixEx`	Calculates a covariance matrix of the input objects group using previously calculated averaged object.
	`CalcEigenObjects`	Calculates orthonormal eigen basis and the averaged object for a group of the input objects.
	`CalcDecompCoeff`	Calculates one decomposition coefficient of the input object using the previously calculated eigen object and the averaged object.
	`EigenDecomposite`	Calculates all decomposition coefficients for the input object.
	`EigenProjection`	Calculates an object projection to the eigen sub-space.

Table 12-1 Image Recognition Functions and Data Types (continued)

Group	Function Name	Description
Embedded Hidden Markov Models Functions	Create2DHMM	Creates a *2D* embedded HMM.
	Release2DHMM	Frees all the memory used by HMM.
	CreateObsInfo	Creates new structures to store image observation vectors.
	ReleaseObsInfo	Frees all memory used by observations and clears pointer to the structure `CvImgObsInfo`.
	ImgToObs_DCT	Extracts observation vectors from the image.
	UniformImgSegm	Performs uniform segmentation of image observations by HMM states.
	InitMixSegm	Segments all observations within every internal state of HMM by state mixture components.
	EstimateHMMStateParams	Estimates all parameters of every HMM state.
	EstimateTransProb	Computes transition probability matrices for embedded HMM.
	EstimateObsProb	Computes probability of every observation of several images.
	EViterbi	Executes Viterbi algorithm for embedded HMM.

Table 12-1 Image Recognition Functions and Data Types (continued)

Group	Function Name	Description
	`MixSegmL2`	Segments observations from all training images by mixture components of newly Viterbi algorithm-assigned states.
	**Data Types**	
Use of Eigen Object Functions	Use of Function `cvCalcEigenObjects` in Direct Access Mode	Shows the use of the function when the size of free RAM is sufficient for all input and eigen objects allocation.
	User Data Structure, I/O Callback Functions, and Use of Function `cvCalcEigenObjects` in Callback Mode	Shows the use of the function when all objects and/or eigen objects cannot be allocated in free RAM.
HMM Structures	Embedded HMM Structure	Represents *1D* HMM and *2D* embedded HMM models.
	Image Observation Structure	Represents image observations.

Eigen Objects Functions

CalcCovarMatrixEx

Calculates covariance matrix for group of input objects.

```
void cvCalcCovarMatrixEx( int nObjects, void* input, int ioFlags, int
    ioBufSize, uchar* buffer, void* userData, IplImage* avg, float*
    covarMatrix );
```

nObjects	Number of source objects.
input	Pointer either to the array of *IplImage* input objects or to the read callback function according to the value of the parameter *ioFlags*.
ioFlags	Input/output flags.
ioBufSize	Input/output buffer size.
buffer	Pointer to the input/output buffer.
userData	Pointer to the structure that contains all necessary data for the callback functions.
avg	Averaged object.
covarMatrix	Covariance matrix. An output parameter; must be allocated before the call.

Discussion

The function `CalcCovarMatrixEx` calculates a covariance matrix of the input objects group using previously calculated averaged object. Depending on *ioFlags* parameter it may be used either in direct access or callback mode. If *ioFlags* is not `CV_EIGOBJ_NO_CALLBACK`, buffer must be allocated before calling the function.

CalcEigenObjects

Calculates orthonormal eigen basis and averaged object for group of input objects.

```
void cvCalcEigenObjects (int nObjects, void* input, void* output, int ioFlags,
    int ioBufSize, void* userData, CvTermCriteria* calcLimit, IplImage* avg,
    float* eigVals);
```

nObjects	Number of source objects.
input	Pointer either to the array of *IplImage* input objects or to the read callback function according to the value of the parameter *ioFlags*.
output	Pointer either to the array of eigen objects or to the write callback function according to the value of the parameter *ioFlags*.

ioFlags	Input/output flags.
ioBufSize	Input/output buffer size in bytes. The size is zero, if unknown.
userData	Pointer to the structure that contains all necessary data for the callback functions.
calcLimit	Criteria that determine when to stop calculation of eigen objects.
avg	Averaged object.
eigVals	Pointer to the eigenvalues array in the descending order; may be `NULL`.

Discussion

The function `CalcEigenObjects` calculates orthonormal eigen basis and the averaged object for a group of the input objects. Depending on *ioFlags* parameter it may be used either in direct access or callback mode. Depending on the parameter *calcLimit*, calculations are finished either after first *calcLimit.maxIters* dominating eigen objects are retrieved or if the ratio of the current eigenvalue to the largest eigenvalue comes down to *calcLimit.epsilon* threshold. The value *calcLimit->type* must be `CV_TERMCRIT_NUMB`, `CV_TERMCRIT_EPS`, or `CV_TERMCRIT_NUMB | CV_TERMCRIT_EPS`. The function returns the real values *calcLimit->maxIter* and *calcLimit->epsilon*.

The function also calculates the averaged object, which must be created previously. Calculated eigen objects are arranged according to the corresponding eigenvalues in the descending order.

The parameter *eigVals* may be equal to `NULL`, if eigenvalues are not needed.

The function `CalcEigenObjects` uses the function`CalcCovarMatrixEx`.

CalcDecompCoeff

Calculates decomposition coefficient of input object.

```
double cvCalcDecompCoeff( IplImage* obj, IplImage* eigObj, IplImage* avg );
```

obj	Input object.
eigObj	Eigen object.
avg	Averaged object.

Discussion

The function `CalcDecompCoeff` calculates one decomposition coefficient of the input object using the previously calculated eigen object and the averaged object.

EigenDecomposite

Calculates all decomposition coefficients for input object.

```
void cvEigenDecomposite( IplImage* obj, int nEigObjs, void* eigInput, int
    ioFlags, void* userData, IplImage* avg, float* coeffs );
```

obj	Input object.
nEigObjs	Number of eigen objects.
eigInput	Pointer either to the array of *IplImage* input objects or to the read callback function according to the value of the parameter *ioFlags*.
ioFlags	Input/output flags.
userData	Pointer to the structure that contains all necessary data for the callback functions.
avg	Averaged object.
coeffs	Calculated coefficients; an output parameter.

Discussion

The function `EigenDecomposite` calculates all decomposition coefficients for the input object using the previously calculated eigen objects basis and the averaged object. Depending on *ioFlags* parameter it may be used either in direct access or callback mode.

EigenProjection

Calculates object projection to the eigen sub-space.

```
void cvEigenProjection ( int nEigObjs, void* eigInput, int ioFlags, void*
    userData, float* coeffs, IplImage* avg, IplImage* proj );
```

nEigObjs	Number of eigen objects.
eigInput	Pointer either to the array of *IplImage* input objects or to the read callback function according to the value of the parameter *ioFlags*.
ioFlags	Input/output flags.
userData	Pointer to the structure that contains all necessary data for the callback functions.
coeffs	Previously calculated decomposition coefficients.
avg	Averaged object.
proj	Decomposed object projection to the eigen sub-space.

Discussion

The function `EigenProjection` calculates an object projection to the eigen sub-space or, in other words, restores an object using previously calculated eigen objects basis, averaged object, and decomposition coefficients of the restored object. Depending on *ioFlags* parameter it may be used either in direct access or callback mode.

Use of Eigen Object Functions

The functions of the eigen objects group have been developed to be used for any number of objects, even if their total size exceeds free RAM size. So the functions may be used in two main modes.

Direct access mode is the best choice if the size of free RAM is sufficient for all input and eigen objects allocation. This mode is set if the parameter *ioFlags* is equal to `CV_EIGOBJ_NO_CALLBACK`. In this case *input* and *output* parameters are pointers to

arrays of input/output objects of `IplImage*` type. The parameters *`ioBufSize`* and *`userData`* are not used. An example of the function `CalcEigenObjects` used in direct access mode is given below.

Example 12-1 Use of Function `cvCalcEigenObjects` in Direct Access Mode

```
IplImage** objects;
IplImage** eigenObjects;
IplImage*  avg;
float*     eigVals;
CvSize     size = cvSize( nx, ny );
. . . . . . . . . . . . . . . . . . . . . . . . . . . . . . . . . . . .
if( !( eigVals = (float*) cvAlloc( nObjects*sizeof(float) ) ) )
    __ERROR_EXIT__;
if( !( avg = cvCreateImage( size, IPL_DEPTH_32F, 1 ) ) )
    __ERROR_EXIT__;
for( i=0; i< nObjects; i++ )
{
    objects[i]      = cvCreateImage( size, IPL_DEPTH_8U,  1 );
    eigenObjects[i] = cvCreateImage( size, IPL_DEPTH_32F, 1 );
    if( !( objects[i] & eigenObjects[i] ) )
        __ERROR_EXIT__;
}
. . . . . . . . . . . . . . . . . . . . . . . . . . . . . . . . . . . .
cvCalcEigenObjects ( nObjects,
                     (void*)objects,
                     (void*)eigenObjects,
              CV_EIGOBJ_NO_CALLBACK,
                     0,
                     NULL,
                     calcLimit,
                     avg,
                     eigVals );
```

The *callback mode* is the right choice in case when the number and the size of objects are large, which happens when all objects and/or eigen objects cannot be allocated in free RAM. In this case input/output information may be read/written and developed by portions. Such regime is called callback mode and is set by the parameter *`ioFlags`*. Three kinds of the callback mode may be set:

`IoFlag` = `CV_EIGOBJ_INPUT_CALLBACK`, only input objects are read by portions;

`IoFlag` = `CV_EIGOBJ_OUTPUT_CALLBACK`, only eigen objects are calculated and written by portions;

IoFlag = CV_EIGOBJ_BOTH_CALLBACK, or *IoFlag* = CV_EIGOBJ_INPUT_CALLBACK | CV_EIGOBJ_OUTPUT_CALLBACK, both processes take place. If one of the above modes is realized, the parameters *input* and *output*, both or either of them, are pointers to read/write callback functions. These functions must be written by the user; their prototypes are the same:

```
CvStatus callback_read ( int ind, void* buffer, void* userData);
CvStatus callback_write( int ind, void* buffer, void* userData);
```

ind Index of the read or written object.

buffer Pointer to the start memory address where the object will be allocated.

userData Pointer to the structure that contains all necessary data for the callback functions.

The user must define the user data structure which may carry all information necessary to read/write procedure, such as the start address or file name of the first object on the HDD or any other device, row length and full object length, etc.

If *ioFlag* is not equal to CV_EIGOBJ_NO_CALLBACK, the function CalcEigenObjects allocates a buffer in RAM for objects/eigen objects portion storage. The size of the buffer may be defined either by the user or automatically. If the parameter *ioBufSize* is equal to 0, or too large, the function will define the buffer size. The read data must be located in the buffer compactly, that is, row after row, without alignment and gaps.

An example of the user data structure, i/o callback functions, and the use of the function CalcEigenObjects in the callback mode is shown below.

Example 12-2 User Data Structure, I/O Callback Functions, and Use of Function cvCalcEigenObjects in Callback Mode

```
// User data structure
typedef struct _UserData
{
    int      objLength; /* Obj. length (in elements, not in bytes !) */
    int      step;      /* Obj. step   (in elements, not in bytes !) */
    CvSize  size;      /* ROI or full size */
    CvPoint roiIndent;
    char*     read_name;
    char*     write_name;
} UserData;
//----------------------------------------------------------------------
--
```

Example 12-2 User Data Structure, I/O Callback Functions, and Use of Function `cvCalcEigenObjects` in Callback Mode (continued)

```
// Read callback function
CvStatus callback_read_8u ( int ind, void* buffer, void* userData)
{
    int i, j, k = 0, m;
    UserData* data = (UserData*)userData;
    uchar* buff  = (uchar*)buf;
    char name[32];
    FILE *f;

    if( ind<0 ) return CV_StsBadArg;
    if( buf==NULL || userData==NULL ) CV_StsNullPtr;

    for(i=0; i<28; i++)
    {
        name[i] = data->read_name[i];
        if(name[i]=='.' || name[i]==' '))break;
    }
    name[i  ] = 48 + ind/100;
    name[i+1] = 48 + (ind%100)/10;
    name[i+2] = 48 + ind%10;
    if((f=fopen(name, "r"))==NULL) return CV_BadCallBack;
    m = data->roiIndent.y*step + data->roiIndent.x;

    for( i=0; i<data->size.height; i++, m+=data->step )
    {
        fseek(f, m , SEEK_SET);
        for( j=0; j<data->size.width; j++, k++ )
            fread(buff+k, 1, 1, f);
    }

    fclose(f);
    return CV_StsOk;
}
//-------------------------------------------------------------------
// Write callback function
cvStatus callback_write_32f ( int ind, void* buffer, void* userData)
{
    int i, j, k = 0, m;
    UserData* data = (UserData*)userData;
    float* buff  = (float*)buf;
    char name[32];
    FILE *f;

    if( ind<0 ) return CV_StsBadArg;
    if( buf==NULL || userData==NULL ) CV_StsNullPtr;

    for(i=0; i<28; i++)
    {
```

Example 12-2 User Data Structure, I/O Callback Functions, and Use of Function `cvCalcEigenObjects` in Callback Mode (continued)

```
            name[i] = data->read_name[i];
            if(name[i]=='.' || name[i]==' '))break;
        }
        if((f=fopen(name, "w"))==NULL) return CV_BadCallBack;
        m = 4 * (ind*data->objLength + data->roiIndent.y*step
                                      + data->roiIndent.x);

        for( i=0; i<data->size.height; i++, m+=4*data->step )
        {
            fseek(f, m , SEEK_SET);
            for( j=0; j<data->size.width; j++, k++ )
                fwrite(buff+k, 4, 1, f);
        }

        fclose(f);
        return CV_StsOk;
    }
    //---------------------------------------------------------------------
    --
    // fragments of the main function
    {
    . . . . . . . . . . . . . . . . . . . . . . . . . . . . . . . . . . . .
        int bufSize = 32*1024*1024;  //32 MB RAM for i/o buffer
        float* avg;
       cv UserData data;
        cvStatus r;
        cvStatus (*read_callback)( int ind, void* buf, void* userData)=
                     read_callback_8u;
        cvStatus (*write_callback)( int ind, void* buf, void* userData)=
                     write_callback_32f;
        cvInput* u_r = (cvInput*)&read_callback;
        cvInput* u_w = (cvInput*)&write_callback;
        void* read_    = (u_r)->data;
        void* write_   = (u_w)->data;
    . . . . . . . . . . . . . . . . . . . . . . . . . . . . . . . . . . . .
        data->read_name  = "input";
        data->write_name = "eigens";
        avg = (float*)cvAlloc(sizeof(float) * obj_width * obj_height );

        cvCalcEigenObjects( obj_number,
                            read_,
                            write_,
                            CV_EIGOBJ_BOTH_CALLBACK,
                            bufSize,
                            (void*)&data,
                            &limit,
                            avg,
```

Example 12-2 User Data Structure, I/O Callback Functions, and Use of Function `cvCalcEigenObjects` in Callback Mode (continued)

```
                              eigVal );
      . . . . . . . . . . . . . . . . . . . . . . . . . . . . . . . . . . . .
      }
```

Embedded Hidden Markov Models Functions

Create2DHMM

Creates 2D embedded HMM.

```
CvEHMM* cvCreate2DHMM( int* stateNumber, int* numMix, int obsSize );
```

stateNumber	Array, the first element of the which specifies the number of superstates in the HMM. All subsequent elements specify the number of states in every embedded HMM, corresponding to each superstate. So, the length of the array is *stateNumber*[0]+1.
numMix	Array with numbers of Gaussian mixture components per each internal state. The number of elements in the array is equal to number of internal states in the HMM, that is, superstates are not counted here.
obsSize	Size of observation vectors to be used with created HMM.

Discussion

The function `Create2DHMM` returns the created structure of the type `CvEHMM` with specified parameters.

Release2DHMM

Releases 2D embedded HMM.

```
void cvRelease2DHMM(CvEHMM** hmm);
```

hmm Address of pointer to HMM to be released.

Discussion

The function `Release2DHMM` frees all the memory used by HMM and clears the pointer to HMM.

CreateObsInfo

Creates structure to store image observation vectors.

```
CvImgObsInfo* cvCreateObsInfo( CvSize numObs, int obsSize );
```

numObs Numbers of observations in the horizontal and vertical directions. For the given image and scheme of extracting observations the parameter can be computed via the macro `CV_COUNT_OBS( roi, dctSize, delta, numObs )`, where *roi*, *dctSize*, *delta*, *numObs* are the pointers to structures of the type `CvSize`. The pointer *roi* means size of *roi* of image observed, *numObs* is the output parameter of the macro.

obsSize Size of observation vectors to be stored in the structure.

Discussion

The function `CreateObsInfo` creates new structures to store image observation vectors. For definitions of the parameters *roi*, *dctSize*, and *delta* see the specification of the function `ImgToObs_DCT`.

ReleaseObsInfo

Releases observation vectors structure.

```
void cvReleaseObsInfo( CvImgObsInfo** obsInfo );
```

obsInfo Address of the pointer to the structure `CvImgObsInfo`.

Discussion

The function `ReleaseObsInfo` frees all memory used by observations and clears pointer to the structure `CvImgObsInfo`.

ImgToObs_DCT

Extracts observation vectors from image.

```
void cvImgToObs_DCT( IplImage* image, float* obs, CvSize dctSize, CvSize
    obsSize, CvSize delta );
```

image Input image.

obs Pointer to consequently stored observation vectors.

dctSize Size of image blocks for which DCT (Discrete Cosine Transform) coefficients are to be computed.

obsSize Number of the lowest DCT coefficients in the horizontal and vertical directions to be put into the observation vector.

delta Shift in pixels between two consecutive image blocks in the horizontal and vertical directions.

Discussion

The function `ImgToObs_DCT` extracts observation vectors, that is, DCT coefficients, from the image. The user must pass *`obsInfo.obs`* as the parameter *`obs`* to use this function with other HMM functions and use the structure `obsInfo` of the `CvImgObsInfo` type.

Example 12-3 Calculating Observations for HMM

```
CvImgObsInfo* obs_info;
...
cvImgToObs_DCT( image,obs_info->obs, //!!!
dctSize, obsSize, delta );
```

UniformImgSegm

Performs uniform segmentation of image observations by HMM states.

```
void cvUniformImgSegm( CvImgObsInfo* obsInfo, CvEHMM* hmm);
```

`obsInfo`	Observations structure.
`hmm`	HMM structure.

Discussion

The function `UniformImgSegm` segments image observations by HMM states uniformly (see Figure 12-1 for *2D* embedded HMM with 5 superstates and 3, 6, 6, 6, 3 internal states of every corresponding superstate).

Figure 12-1 Initial Segmentation for *2D* Embedded HMM

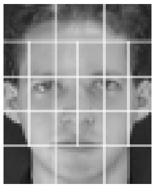

InitMixSegm

Segments all observations within every internal state of HMM by state mixture components.

```
void cvInitMixSegm( CvImgObsInfo** obsInfoArray, int numImg, CvEHMM* hmm);
```

obsInfoArray	Array of pointers to the observation structures.
numImg	Length of above array.
hmm	HMM.

Discussion

The function `InitMixSegm` takes a group of observations from several training images already segmented by states and splits a set of observation vectors within every internal HMM state into as many clusters as the number of mixture components in the state.

EstimateHMMStateParams

Estimates all parameters of every HMM state.

```
void cvEstimateHMMStateParams(CvImgObsInfo** obsInfoArray, int numImg,
    CvEHMM* hmm);
```

obsInfoArray	Array of pointers to the observation structures.
numImg	Length of the array.
hmm	HMM.

Discussion

The function `EstimateHMMStateParams` computes all inner parameters of every HMM state, including Gaussian means, variances, etc.

EstimateTransProb

Computes transition probability matrices for embedded HMM.

```
void cvEstimateTransProb( CvImgObsInfo** obsInfoArray, int numImg, CvEHMM*
    hmm);
```

obsInfoArray	Array of pointers to the observation structures.
numImg	Length of the above array.
hmm	HMM.

Discussion

The function `EstimateTransProb` uses current segmentation of image observations to compute transition probability matrices for all embedded and external HMMs.

EstimateObsProb

Computes probability of every observation of several images.

```
void cvEstimateObsProb( CvImgObsInfo* obsInfo, CvEHMM* hmm);
```

obsInfo	Observation structure.
hmm	HMM structure.

Discussion

The function `EstimateObsProb` computes Gaussian probabilities of each observation to occur in each of the internal HMM states.

EViterbi

Executes Viterbi algorithm for embedded HMM.

```
Float cvEViterbi( CvImgObsInfo* obsInfo, CvEHMM* hmm);
```

obsInfo	Observation structure.
hmm	HMM structure.

Discussion

The function `EViterbi` executes Viterbi algorithm for embedded HMM. Viterbi algorithm evaluates the likelihood of the best match between the given image observations and the given HMM and performs segmentation of image observations by HMM states. The segmentation is done on the basis of the match found.

MixSegmL2

Segments observations from all training images by mixture components of newly assigned states.

```
void cvMixSegmL2( CvImgObsInfo** obsInfoArray, int numImg, CvEHMM* hmm);
```

obsInfoArray	Array of pointers to the observation structures.
numImg	Length of the array.
hmm	HMM.

Discussion

The function `MixSegmL2` segments observations from all training images by mixture components of newly Viterbi algorithm-assigned states. The function uses Euclidean distance to group vectors around the existing mixtures centers.

HMM Structures

In order to support embedded models the user must define structures to represent *1D* HMM and *2D* embedded HMM model.

Example 12-4 Embedded HMM Structure

```
typedef struct _CvEHMM
{
    int level;
    int num_states;
   float*  transP;
   float** obsProb;
   union
   {
       CvEHMMState* state;
       struct _CvEHMM* ehmm;
   } u;
}CvEHMM;
```

Below is the description of the `CvEHMM` fields:

`level` Level of embedded HMM. If `level==0`, HMM is most external. In 2D HMM there are two types of HMM: 1 external and several embedded. External HMM has `level==1`, embedded HMMs have `level==0`.

`num_states` Number of states in *1D* HMM.

`transP` State-to-state transition probability, square matrix ($num\_state \times num\_state$).

`obsProb` Observation probability matrix.

`state` Array of HMM states. For the last-level HMM, that is, an HMM without embedded HMMs, HMM states are real.

`ehmm` Array of embedded HMMs. If HMM is not last-level, then HMM states are not real and they are HMMs.

For representation of observations the following structure is defined:

Example 12-5 Image Observation Structure

```
typedef struct CvImgObsInfo
{
    int obs_x;
    int obs_y;
    int obs_size;
    float** obs;
    int* state;
    int* mix;
}CvImgObsInfo;
```

This structure is used for storing observation vectors extracted from *2D* image.

`obs_x` Number of observations in the horizontal direction.

`obs_y` Number of observations in the vertical direction.

`obs_size` Length of every observation vector.

`obs` Pointer to observation vectors stored consequently. Number of vectors is `obs_x*obs_y`.

`state` Array of indices of states, assigned to every observation vector.

`mix` Index of mixture component, corresponding to the observation vector within an assigned state.

3D Reconstruction Reference

13

Table 13-1 *3D* Reconstruction Functions

Group	Function Name	Description
Camera Calibration Functions	`CalibrateCamera`	Calibrates the camera with single precision.
	`CalibrateCamera_64d`	Calibrates camera with double precision.
	`FindExtrinsicCameraParams`	Finds the extrinsic camera parameters for the pattern.
	`FindExtrinsicCameraParams_64d`	Finds extrinsic camera parameters for the pattern with double precision.
	`Rodrigues`	Converts the rotation matrix to the rotation vector and vice versa with single precision.
	`Rodrigues_64d`	Converts the rotation matrix to the rotation vector or vice versa with double precision.
	`UnDistortOnce`	Corrects camera lens distortion in the case of a single image.
	`UnDistortInit`	Calculates arrays of distorted points indices and interpolation coefficients.

Table 13-1 3D Reconstruction Functions (continued)

Group	Function Name	Description
	UnDistort	Corrects camera lens distortion using previously calculated arrays of distorted points indices and undistortion coefficients.
	FindChessBoardCornerGuesses	Finds approximate positions of internal corners of the chessboard.
View Morphing Functions	FindFundamentalMatrix	Calculates the fundamental matrix from several pairs of correspondent points in images from two cameras.
	MakeScanlines	Calculates scanlines coordinates for two cameras by fundamental matrix.
	PreWarpImage	Rectifies the image so that the scanlines in the rectified image are horizontal.
	FindRuns	Retrieves scanlines from the rectified image and breaks each scanline down into several runs.
	DynamicCorrespondMulti	Finds correspondence between two sets of runs of two warped images.
	MakeAlphaScanlines	Finds coordinates of scanlines for the virtual camera with the given camera position.

Table 13-1 *3D* Reconstruction Functions (continued)

Group	Function Name	Description
	MorphEpilinesMulti	Morphs two pre-warped images using information about stereo correspondence.
	PostWarpImage	Warps the rectified morphed image back.
	DeleteMoire	Deletes moire from the given image.
POSIT Functions	CreatePOSITObject	Allocates memory for the object structure and computes the object inverse matrix.
	POSIT	Implements POSIT algorithm.
	ReleasePOSITObject	Deallocates the 3D object structure.
Gesture Recognition Functions	FindHandRegion	Finds an arm region in the 3D range image data.
	FindHandRegionA	Finds an arm region in the 3D range image data and defines the arm orientation.
	CreateHandMask	Creates an arm mask on the image plane.
	CalcImageHomography	Calculates the homograph matrix for the initial image transformation.
	CalcProbDensity	Calculates the arm mask probability density from the two *2D* histograms.
	MaxRect	Calculates the maximum rectangle for two input rectangles.

Camera Calibration Functions

CalibrateCamera

Calibrates camera with single precision.

```
void cvCalibrateCamera( int numImages, int* numPoints, CvSize imageSize,
    CvPoint2D32f* imagePoints32f, CvPoint3D32f* objectPoints32f, CvVect32f
    distortion32f, CvMatr32f cameraMatrix32f, CvVect32f transVects32f,
    CvMatr32f rotMatrs32f, int useIntrinsicGuess);
```

numImages	Number of the images.
numPoints	Array of the number of points in each image.
imageSize	Size of the image.
imagePoints32f	Pointer to the images.
objectPoints32f	Pointer to the pattern.
distortion32f	Array of four distortion coefficients found.
cameraMatrix32f	Camera matrix found.
transVects32f	Array of translate vectors for each pattern position in the image.
rotMatrs32f	Array of the rotation matrix for each pattern position in the image.
useIntrinsicGuess	Intrinsic guess. If equal to 1, intrinsic guess is needed.

Discussion

The function `CalibrateCamera` calculates the camera parameters using information points on the pattern object and pattern object images.

CalibrateCamera_64d

Calibrates camera with double precision.

```
void cvCalibrateCamera_64d( int numImages, int* numPoints, CvSize imageSize,
    CvPoint2D64d* imagePoints, CvPoint3D64d* objectPoints, CvVect64d
    distortion, CvMatr64d cameraMatrix, CvVect64d transVects, CvMatr64d
    rotMatrs, int useIntrinsicGuess);
```

numImages	Number of the images.
numPoints	Array of the number of points in each image.
imageSize	Size of the image.
imagePoints	Pointer to the images.
objectPoints	Pointer to the pattern.
distortion	Distortion coefficients found.
cameraMatrix	Camera matrix found.
transVects	Array of the translate vectors for each pattern position on the image.
rotMatrs	Array of the rotation matrix for each pattern position on the image.
useIntrinsicGuess	Intrinsic guess. If equal to 1, intrinsic guess is needed.

Discussion

The function `CalibrateCamera_64d` is basically the same as the function `CalibrateCamera`, but uses double precision.

FindExtrinsicCameraParams

Finds extrinsic camera parameters for pattern.

```
void cvFindExtrinsicCameraParams( int numPoints, CvSize imageSize,
    CvPoint2D32f* imagePoints32f, CvPoint3D32f* objectPoints32f, CvVect32f
    focalLength32f, CvPoint2D32f  principalPoint32f, CvVect32f distortion32f,
    CvVect32f rotVect32f, CvVect32f transVect32f);
```

numPoints	Number of the points.
ImageSize	Size of the image.
imagePoints32f	Pointer to the image.
objectPoints32f	Pointer to the pattern.
focalLength32f	Focal length.
principalPoint32f	Principal point.
distortion32f	Distortion.
rotVect32f	Rotation vector.
transVect32f	Translate vector.

Discussion

The function `FindExtrinsicCameraParams` finds the extrinsic parameters for the pattern.

FindExtrinsicCameraParams_64d

Finds extrinsic camera parameters for pattern with double precision.

```
void cvFindExtrinsicCameraParams_64d( int numPoints, CvSize imageSize,
    CvPoint2D64d* imagePoints,  CvPoint3D64d* objectPoints, CvVect64d
    focalLength, CvPoint2D64d principalPoint, CvVect64d distortion, CvVect64d
    rotVect, CvVect64d transVect);
```

numPoints	Number of the points.
ImageSize	Size of the image.
imagePoints	Pointer to the image.
objectPoints	Pointer to the pattern.
focalLength	Focal length.
principalPoint	Principal point.
distortion	Distortion.
rotVect	Rotation vector.
transVect	Translate vector.

Discussion

The function `FindExtrinsicCameraParams_64d` finds the extrinsic parameters for the pattern with double precision.

Rodrigues

Converts rotation matrix to rotation vector and vice versa with single precision.

```
void cvRodrigues( CvMatr32f rotMatr32f, CvVect32f rotVect32f, CvMatr32f
Jacobian32f, CvRodriguesType convType);
```

rotMatr32f	Rotation matrix.
rotVect32f	Rotation vector.
Jacobian32f	Jacobian matrix 3 x 9.
convType	Type of conversion; must be `CV_RODRIGUES_M2V` for converting the matrix to the vector or `CV_RODRIGUES_V2M` for converting the vector to the matrix.

Discussion

The function `Rodrigues` converts the rotation matrix to the rotation vector or vice versa.

Rodrigues_64d

Converts rotation matrix to rotation vector and vice versa with double precision.

```
void cvRodrigues_64d( CvMatr64d  rotMatr, CvVect64d rotVect, CvMatr64d
Jacobian, CvRodriguesType convType);
```

rotMatr	Rotation matrix.
rotVect	Rotation vector.
Jacobian	Jacobian matrix 3 x 9.
convType	Type of conversion; must be `CV_RODRIGUES_M2V` for converting the matrix to the vector or `CV_RODRIGUES_V2M` for converting the vector to the matrix.

Discussion

The function `Rodrigues_64d` converts the rotation matrix to the rotation vector or vice versa with double precision.

UnDistortOnce

Corrects camera lens distortion.

```
void cvUnDistortOnce ( IplImage* srcImage, IplImage* dstImage, float*
intrMatrix, float* distCoeffs, int interpolate=1 );
```

`srcImage`	Source (distorted) image.
`dstImage`	Destination (corrected) image.
`intrMatrix`	Matrix of the camera intrinsic parameters.
`distCoeffs`	Vector of the four distortion coefficients k_1, k_2, p_1 and p_2 .
`interpolate`	Interpolation toggle (optional).

Discussion

The function `UnDistortOnce` corrects camera lens distortion in case of a single image. Matrix of the camera intrinsic parameters and distortion coefficients k_1, k_2, p_1, and p_2 must be preliminarily calculated by the function `CalibrateCamera`.

If *interpolate* = 0, inter-pixel interpolation is disabled; otherwise, default bilinear interpolation is used.

UnDistortInit

Calculates arrays of distorted points indices and interpolation coefficients.

```
void cvUnDistortInit ( IplImage* srcImage, float* IntrMatrix, float*
distCoeffs, int* data, int interpolate=1 );
```

`srcImage`	Source (distorted) image.
`intrMatrix`	Matrix of the camera intrinsic parameters.
`distCoeffs`	Vector of the 4 distortion coefficients k_1, k_2, p_1 and p_2 .

`data`	Distortion data array.
`interpolate`	Interpolation toggle (optional).

Discussion

The function `UnDistortInit` calculates arrays of distorted points indices and interpolation coefficients using known matrix of the camera intrinsic parameters and distortion coefficients. It must be used before calling the function `UnDistort`.

Matrix of the camera intrinsic parameters and distortion coefficients k_1, k_2, p_1, and p_2 must be preliminarily calculated by the function `CalibrateCamera`.

The *data* array must be allocated in the main function before use of the function `UnDistortInit`. If *interpolate* = 0, its length must be *size.width*size.height* elements; otherwise *3*size.width*size.height* elements.

If *interpolate* = 0, inter-pixel interpolation is disabled; otherwise default bilinear interpolation is used.

UnDistort

Corrects camera lens distortion.

```
void cvUnDistort ( IplImage* srcImage, IplImage* dstImage, int* data, int
interpolate=1 );
```

`srcImage`	Source (distorted) image.
`dstImage`	Destination (corrected) image.
`data`	Distortion data array.
`interpolate`	Interpolation toggle (optional).

Discussion

The function `UnDistort` corrects camera lens distortion using previously calculated arrays of distorted points indices and undistortion coefficients. It is used if a sequence of frames must be corrected.

Preliminarily, the function `UnDistortInit` calculates the array *data*.

If *interpolate* = 0, then inter-pixel interpolation is disabled; otherwise bilinear interpolation is used. In the latter case the function acts slower, but quality of the corrected image increases.

FindChessBoardCornerGuesses

Finds approximate positions of internal corners of the chessboard.

```
int cvFindChessBoardCornerGuesses (IplImage* img, IplImage* thresh, CvSize
    etalonSize, CvPoint2D32f* corners, int* cornerCount);
```

img	Source chessboard view; must have the depth of `IPL_DEPTH_8U`.
thresh	Temporary image of the same size and format as the source image.
etalonSize	Number of inner corners per chessboard row and column. The width (the number of columns) must be less or equal to the height (the number of rows). For chessboard see Figure 6-1.
corners	Pointer to the corner array found.
cornerCount	Signed value whose absolute value is the number of corners found. A positive number means that a whole chessboard has been found and a negative number means that not all the corners have been found.

Discussion

The function `FindChessBoardCornerGuesses` attempts to determine whether the input image is a view of the chessboard pattern and locate internal chessboard corners. The function returns non-zero value if all the corners have been found and they have been placed in a certain order (row by row, left to right in every row), otherwise, if the function fails to find all the corners or reorder them, the function returns 0. For example, a simple chessboard has 8x8 squares and 7x7 internal corners, that is, points, where the squares are tangent. The word “approximate” in the above description

means that the corner coordinates found may differ from the actual coordinates by a couple of pixels. To get more precise coordinates, the user may use the function FindCornerSubPix.

View Morphing Functions

FindFundamentalMatrix

Calculates fundamental matrix from several pairs of correspondent points in images from two cameras.

```
void cvFindFundamentalMatrix (int* points1, int* points2, int numpoints, int
method, CvMatrix3* matrix);
```

points1	Pointer to the array of correspondence points in the first image.
points2	Pointer to the array of correspondence points in the second image.
numpoints	Number of the point pairs.
method	Method for finding the fundamental matrix; currently not used, must be zero.
matrix	Resulting fundamental matrix.

Discussion

The function `FindFundamentalMatrix` finds the fundamental matrix for two cameras from several pairs of correspondent points in images from the cameras. If the number of pairs is less than 8 or the points lie very close to each other or on the same planar surface, the matrix is calculated incorrectly.

MakeScanlines

Calculates scanlines coordinates for two cameras by fundamental matrix.

```
void cvMakeScanlines (CvMatrix3* matrix, CvSize imgSize, int* scanlines1, int*
scanlines2, int* lens1, int* lens2, int* numlines);
```

matrix	Fundamental matrix.
imgSize	Size of the image.
scanlines1	Pointer to the array of calculated scanlines of the first image.
scanlines2	Pointer to the array of calculated scanlines of the second image.
lens1	Pointer to the array of calculated lengths (in pixels) of the first image scanlines.
lens2	Pointer to the array of calculated lengths (in pixels) of the second image scanlines.
numlines	Pointer to the variable that stores the number of scanlines.

Discussion

The function `MakeScanlines` finds coordinates of scanlines for two images.

This function returns the number of scanlines. The function does nothing except calculating the number of scanlines if the pointers *scanlines1* or *scanlines2* are equal to zero.

PreWarpImage

Rectifies image.

```
void cvPreWarpImage (int numLines, IplImage* img, uchar* dst, int*  dstNums,
    int* scanlines);
```

numLines	Number of scanlines for the image.

img	Image to prewarp.
dst	Data to store for the prewarp image.
dstNums	Pointer to the array of lengths of scanlines.
scanlines	Pointer to the array of coordinates of scanlines.

Discussion

The function `PreWarpImage` rectifies the image so that the scanlines in the rectified image are horizontal. The output buffer of size `max(width,height)*numscanlines*3` must be allocated before calling the function.

FindRuns

Retrieves scanlines from rectified image and breaks them down into runs.

```
void cvFindRuns (int numLines, uchar* prewarp_1, uchar* prewarp_2, int*
    lineLens_1, int* lineLens_2, int* runs_1, int* runs_2, int* numRuns_1,
    int* numRuns_2);
```

numLines	Number of the scanlines.
prewarp_1	Prewarp data of the first image.
prewarp_2	Prewarp data of the second image.
lineLens_1	Array of lengths of scanlines in the first image.
lineLens_2	Array of lengths of scanlines in the second image.
runs_1	Array of runs in each scanline in the first image.
runs_2	Array of runs in each scanline in the second image.
numRuns_1	Array of numbers of runs in each scanline in the first image.
numRuns_2	Array of numbers of runs in each scanline in the second image.

Discussion

The function `FindRuns` retrieves scanlines from the rectified image and breaks each scanline down into several runs, that is, series of pixels of almost the same brightness.

DynamicCorrespondMulti

Finds correspondence between two sets of runs of two warped images.

```
void cvDynamicCorrespondMulti (int  lines, int* first, int* firstRuns, int*
    second, int* secondRuns, int* firstCorr, int* secondCorr);
```

lines	Number of scanlines.
first	Array of runs of the first image.
firstRuns	Array of numbers of runs in each scanline of the first image.
second	Array of runs of the second image.
secondRuns	Array of numbers of runs in each scanline of the second image.
firstCorr	Pointer to the array of correspondence information found for the first runs.
secondCorr	Pointer to the array of correspondence information found for the second runs.

Discussion

The function `DynamicCorrespondMulti` finds correspondence between two sets of runs of two images. Memory must be allocated before calling this function. Memory size for one array of correspondence information is `max(width,height)*numscanlines*3*sizeof(int)`.

MakeAlphaScanlines

Calculates coordinates of scanlines of image from virtual camera.

```
void cvMakeAlphaScanlines (int* scanlines_1, int* scanlines_2, int*
    scanlinesA, int* lens, int numlines, float alpha);
```

scanlines_1	Pointer to the array of the first scanlines.
scanlines_2	Pointer to the array of the second scanlines.
scanlinesA	Pointer to the array of the scanlines found in the virtual image.
lens	Pointer to the array of lengths of the scanlines found in the virtual image.
numlines	Number of scanlines.
alpha	Position of virtual camera (`0.0 - 1.0`).

Discussion

The function `MakeAlphaScanlines` finds coordinates of scanlines for the virtual camera with the given camera position.

Memory must be allocated before calling this function. Memory size for the array of correspondence runs is *numscanlines*2*4*sizeof(int)*). Memory size for the array of the scanline lengths is *numscanlines*2*4*sizeof(int)*.

MorphEpilinesMulti

Morphs two pre-warped images using information about stereo correspondence.

```
void cvMorphEpilinesMulti (int lines, uchar* firstPix, int* firstNum, uchar*
    secondPix, int* secondNum, uchar* dstPix, int* dstNum, float alpha, int*
    first, int* firstRuns, int* second, int* secondRuns, int* firstCorr, int*
    secondCorr);
```

lines	Number of scanlines in the prewarp image.
firstPix	Pointer to the first prewarp image.
firstNum	Pointer to the array of numbers of points in each scanline in the first image.
secondPix	Pointer to the second prewarp image.
secondNum	Pointer to the array of numbers of points in each scanline in the second image.
dstPix	Pointer to the resulting morphed warped image.
dstNum	Pointer to the array of numbers of points in each line.
alpha	Virtual camera position `(0.0 - 1.0)`.
first	First sequence of runs.
firstRuns	Pointer to the number of runs in each scanline in the first image.
second	Second sequence of runs.
secondRuns	Pointer to the number of runs in each scanline in the second image.
firstCorr	Pointer to the array of correspondence information found for the first runs.
secondCorr	Pointer to the array of correspondence information found for the second runs.

Discussion

The function `MorphEpilinesMulti` morphs two pre-warped images using information about correspondence between the scanlines of two images.

PostWarpImage

Warps rectified morphed image back.

```
void cvPostWarpImage (int numLines, uchar* src, int* srcNums, IplImage* img,
    int* scanlines);
```

numLines	Number of the scanlines.

src	Pointer to the prewarp image virtual image.
srcNums	Number of the scanlines in the image.
img	Resulting unwarp image.
scanlines	Pointer to the array of scanlines data.

Discussion

The function `PostWarpImage` warps the resultant image from the virtual camera by storing its rows across the scanlines whose coordinates are calculated by `MakeAlphaScanlines` function.

DeleteMoire

Deletes moire in given image.

```
void cvDeleteMoire (IplImage* img);
```

img	Image.

Discussion

The function `DeleteMoire` deletes moire from the given image. The post-warped image may have black (un-covered) points because of possible holes between neighboring scanlines. The function deletes moire (black pixels) from the image by substituting neighboring pixels for black pixels. If all the scanlines are horizontal, the function may be omitted.

POSIT Functions

CreatePOSITObject

Initializes structure containing object information.

```
CvPOSITObject* cvCreatePOSITObject (CvPoint3D32f* points, int numPoints);
```

points	Pointer to the points of the *3D* object model.
numPoints	Number of object points.

Discussion

The function `CreatePOSITObject` allocates memory for the object structure and computes the object inverse matrix.

The preprocessed object data is stored in the structure `CvPOSITObject`, internal for OpenCV, which means that the user cannot directly access the structure data. The user may only create this structure and pass its pointer to the function.

Object is defined as a set of points given in a coordinate system. The function `POSIT` computes a vector that begins at a camera-related coordinate system center and ends at the `points[0]` of the object.

Once the work with a given object is finished, the function `ReleasePOSITObject` must be called to free memory.

POSIT

Implements POSIT algorithm.

```
void cvPOSIT (CvPoint2D32f* imagePoints, CvPOSITObject* pObject, double
    focalLength, CvTermCriteria criteria, CvMatrix3* rotation, CvPoint3D32f*
    translation);
```

imagePoints	Pointer to the object points projections on the *2D* image plane.
pObject	Pointer to the object structure.
focalLength	Focal length of the camera used.
criteria	Termination criteria of the iterative POSIT algorithm.
rotation	Matrix of rotations.
translation	Translation vector.

Discussion

The function `POSIT` implements POSIT algorithm. Image coordinates are given in a camera-related coordinate system. The focal length may be retrieved using camera calibration functions. At every iteration of the algorithm new perspective projection of estimated pose is computed.

Difference norm between two projections is the maximal distance between corresponding points. The parameter *`criteria.epsilon`* serves to stop the algorithm if the difference is small.

ReleasePOSITObject

Deallocates 3D object structure.

```
void cvReleasePOSITObject ( CvPOSITObject** ppObject );
```

ppObject	Address of the pointer to the object structure.

Discussion

The function `ReleasePOSITObject` releases memory previously allocated by the function `CreatePOSITObject`.

Gesture Recognition Functions

FindHandRegion

Finds arm region in 3D range image data.

```
void cvFindHandRegion (CvPoint3D32f* points, int count, CvSeq* indexs, float*
     line, CvSize2D32f size, int flag,  CvPoint3D32f* center, CvMemStorage*
     storage, CvSeq** numbers);
```

points	Pointer to the input *3D* point data.
count	Numbers of the input points.
indexs	Sequence of the input points indices in the initial image.
line	Pointer to the input points approximation line.
size	Size of the initial image.
flag	Flag of the arm orientation.
center	Pointer to the output arm center.
storage	Pointer to the memory storage.
numbers	Pointer to the output sequence of the points indices.

Discussion

The function `FindHandRegion` finds the arm region in *3D* range image data. The coordinates of the points must be defined in the world coordinates system. Each input point has user-defined transform indices *indexs* in the initial image. The function finds the arm region along the approximation line from the left, if `flag = 0`, or from the right, if `flag = 1`, in the points maximum accumulation by the points projection histogram calculation. Also the function calculates the center of the arm region and the indices of the points that lie near the arm center. The function `FindHandRegion` assumes that the arm length is equal to about 0.25m in the world coordinate system.

FindHandRegionA

Finds arm region in 3D range image data and defines arm orientation.

```
void cvFindHandRegionA (CvPoint3D32f* points, int count, CvSeq* indexs, float*
    line, CvSize2D32f size, int jCenter, CvPoint3D32f* center, CvMemStorage*
    storage, CvSeq** numbers);
```

points	Pointer to the input *3D* point data.
count	Number of the input points.
indexs	Sequence of the input points indices in the initial image.
line	Pointer to the input points approximation line.
size	Size of the initial image.
jCenter	Input *j*-index of the initial image center.
center	Pointer to the output arm center.
storage	Pointer to the memory storage.
numbers	Pointer to the output sequence of the points indices.

Discussion

The function `FindHandRegionA` finds the arm region in the *3D* range image data and defines the arm orientation (left or right). The coordinates of the points must be defined in the world coordinates system. The input parameter *jCenter* is the index *j* of the initial image center in pixels (*width*/2). Each input point has user-defined transform indices on the initial image (*indexs*). The function finds the arm region along approximation line from the left or from the right in the points maximum accumulation by the points projection histogram calculation. Also the function calculates the center of the arm region and the indices of points that lie near the arm center. The function `FindHandRegionA` assumes that the arm length is equal to about 0.25m in the world coordinate system.

CreateHandMask

Creates arm mask on image plane.

```
void cvCreateHandMask(CvSeq* numbers, IplImage *imgMask, CvRect *roi);
```

numbers Sequence of the input points indices in the initial image.

imgMask Pointer to the output image mask.

roi Pointer to the output arm ROI.

Discussion

The function `CreateHandMask` creates an arm mask on the image plane. The pixels of the resulting mask associated with the set of the initial image indices *indexs* associated with hand region have the maximum unsigned char value (255). All remaining pixels have the minimum unsigned char value (0). The output image mask *imgMask* has to have the `IPL_DEPTH_8U` type and the number of channels is 1.

CalcImageHomography

Calculates homography matrix.

```
void cvCalcImageHomography(float* line, CvPoint3D32f* center, float
    intrinsic[3][3], float homography[3][3]);
```

line Pointer to the input *3D* line.

center Pointer to the input arm center.

intrinsic Matrix of the intrinsic camera parameters.

homography Output homography matrix.

Discussion

The function `CalcImageHomography` calculates the homograph matrix for the initial image transformation from image plane to the plane, defined by *3D* arm line (See Figure 6-10 in Programmer Guide *3D* Reconstruction Chapter). If $n_1=(nx,ny)$ and $n_2=(nx,nz)$ are coordinates of the normals of the *3D* line projection of planes XY and XZ, then the resulting image homography matrix is calculated as $H = A \cdot (R_h + (I_{3\times3} - R_h) \cdot \bar{x}_h \cdot [0, 0, 1]) \cdot A^{-1}$, where R_h is the 3x3 matrix $R_h = R_1 \cdot R_2$, and $R_1 = [n_1 \times u_z, n_1, u_z], R_2 = [u_y \times n_2, u_y, n_2], u_z = [0, 0, 1]^T, u_y = [0, 1, 0]^T, \bar{x}_h = \frac{T_h}{T_z} = \left[\frac{T_x}{T_z}, \frac{T_y}{T_z}, 1\right]^T$,

where (T_x, T_y, T_z) is the arm center coordinates in the world coordinate system, and A is the intrinsic camera parameters matrix

$$A = \begin{bmatrix} f_x & 0 & c_x \\ 0 & f_y & c_y \\ 0 & 0 & 1 \end{bmatrix}.$$

The diagonal entries f_x and f_y are the camera focal length in units of horizontal and vertical pixels and the two remaining entries c_x, c_y are the principal point image coordinates.

CalcProbDensity

Calculates arm mask probability density on image plane.

```
void cvCalcProbDensity (CvHistogram* hist, CvHistogram* histMask, CvHistogram*
    histDens);
```

hist	Input image histogram.
histMask	Input image mask histogram.
histDens	Resulting probability density histogram.

Discussion

The function `CalcProbDensity` calculates the arm mask probability density from the two *2D* histograms. The input histograms have to be calculated in two channels on the initial image. If $\{h_{ij}\}$ and $\{hm_{ij}\}$, $1 \le i \le B_i$, $1 \le j \le B_j$ are input histogram and mask histogram respectively, then the resulting probability density histogram p_{ij} is calculated as

$$p_{ij} = \begin{cases} \frac{m_{ij}}{h_{ij}} \cdot 255, if \quad h_{ij} \neq 0, \\ 0, if \quad h_{ij} = 0, \\ 255, if \quad m_{ij} > h_{ij} \end{cases}$$

So the values of the p_{ij} are between 0 and 255.

MaxRect

Calculates the maximum rectangle.

```
void cvMaxRect (CvRect* rect1, CvRect* rect2, CvRect* maxRect);
```

rect1	First input rectangle.
rect2	Second input rectangle.
maxRect	Resulting maximum rectangle.

Discussion

The function `MaxRect` calculates the maximum rectangle for two input rectangles (Figure 13-1).

Figure 13-1 Maximum Rectangle for Two Input Rectangles

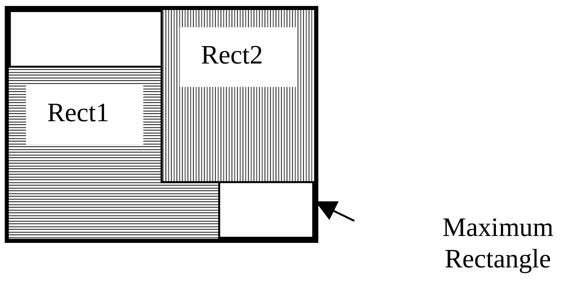

Basic Structures and Operations Reference 14

Table 14-1 Basic Structures and Operations Functions, Macros, and Data Types

Name	Description
	**Functions**
**Image Functions**	
`CreateImageHeader`	Allocates, initializes, and returns structure `IplImage`.
`CreateImage`	Creates the header and allocates data.
`ReleaseImageHeader`	Releases the header.
`ReleaseImage`	Releases the header and the image data.
`CreateImageData`	Allocates the image data.
`ReleaseImageData`	Releases the image data.
`SetImageData`	Sets the pointer to *`data`* and *`step`* parameters to given values.
`SetImageCOI`	Sets the channel of interest to a given value.
`SetImageROI`	Sets the image ROI to a given rectangle.
`GetImageRawData`	Fills output variables with the image parameters.
`InitImageHeader`	Initializes the image header structure without memory allocation.
`CopyImage`	Copies the entire image to another without considering ROI.
**Dynamic Data Structures Functions**	
`CreateMemStorage`	Creates a memory storage and returns the pointer to it.
`CreateChildMemStorage`	Creates a child memory storage.

Table 14-1 Basic Structures and Operations Functions, Macros, and Data Types (continued)

Name	Description
ReleaseMemStorage	De-allocates all storage memory blocks or returns them to the parent, if any.
ClearMemStorage	Clears the memory storage.
SaveMemStoragePos	Saves the current position of the storage top.
RestoreMemStoragePos	Restores the position of the storage top.
CreateSeq	Creates a sequence and returns the pointer to it.
SetSeqBlockSize	Sets up the sequence block size.
SeqPush	Adds an element to the end of the sequence.
SeqPop	Removes an element from the sequence.
SeqPushFront	Adds an element to the beginning of the sequence.
SeqPopFront	Removes an element from the beginning of the sequence.
SeqPushMulti	Adds several elements to the end of the sequence.
SeqPopMulti	Removes several elements from the end of the sequence.
SeqInsert	Inserts an element in the middle of the sequence.
SeqRemove	Removes elements with the given index from the sequence.
ClearSeq	Empties the sequence.
GetSeqElem	Finds the element with the given index in the sequence and returns the pointer to it.
SeqElemIdx	Returns index of concrete sequence element.
CvtSeqToArray	Copies the sequence to a continuous block of memory.
MakeSeqHeaderForArray	Builds a sequence from an array.
StartAppendToSeq	Initializes the writer to write to the sequence.
StartWriteSeq	Is the exact sum of the functions CreateSeq and StartAppendToSeq.
EndWriteSeq	Finishes the process of writing.
FlushSeqWriter	Updates sequence headers using the writer state.
GetSeqReaderPos	Returns the index of the element in which the reader is currently located.
SetSeqReaderPos	Moves the read position to the absolute or relative position.

Table 14-1 Basic Structures and Operations Functions, Macros, and Data Types (continued)

Name	Description
CreateSet	Creates an empty set with a specified header size.
SetAdd	Adds an element to the set.
SetRemove	Removes an element from the set.
GetSetElem	Finds a set element by index.
ClearSet	Empties the set.
CreateGraph	Creates an empty graph.
GraphAddVtx	Adds a vertex to the graph.
GraphRemoveVtx	Removes a vertex from the graph.
GraphRemoveVtxByPtr	Removes a vertex from the graph together with all the edges incident to it.
GraphAddEdge	Adds an edge to the graph.
GraphAddEdgeByPtr	Adds an edge to the graph given the starting and the ending vertices.
GraphRemoveEdge	Removes an edge from the graph.
GraphRemoveEdgeByPtr	Removes an edge from the graph that connects given vertices.
FindGraphEdge	Finds the graph edge that connects given vertices.
FindGraphEdgeByPtr	Finds the graph edge that connects given vertices.
GraphVtxDegree	Finds an edge in the graph.
GraphVtxDegreeByPtr	Counts the edges incident to the graph vertex, both incoming and outcoming, and returns the result.
ClearGraph	Removes all the vertices and edges from the graph.
GetGraphVtx	Finds the graph vertex by index.
GraphVtxIdx	Returns the index of the graph vertex.
GraphEdgeIdx	Returns the index of the graph edge.
**Matrix Operations Functions**	
CreateMat	Creates a new matrix.
CreateMatHeader	Creates a new matrix header.
ReleaseMat	Deallocates the matrix.

Table 14-1 Basic Structures and Operations Functions, Macros, and Data Types (continued)

Name	Description
ReleaseMatHeader	Deallocates the matrix header.
InitMatHeader	Initializes a matrix header.
CloneMat	Creates a copy of the matrix.
SetData	Attaches data to the matrix header.
GetMat	Initializes a matrix header for an arbitrary array.
GetAt	Returns value of the specified array element.
SetAt	Changes value of the specified array element.
GetAtPtr	Returns pointer of the specified array element.
GetSubArr	Returns a rectangular sub-array of the given array.
GetRow	Returns an array row.
GetCol	Returns an array column.
GetDiag	Returns an array diagonal.
GetRawData	Returns low level information on the array.
GetSize	Returns width and height of the array.
CreateData	Allocates memory for the array data.
AllocArray	Allocates memory for the array data.
ReleaseData	Frees memory allocated for the array data.
FreeArray	Frees memory allocated for the array data.
Copy	Copies one array to another.
Set	Sets every element of array to given value.
Add	Computes sum of two arrays.
AddS	Computes sum of array and scalar.
Sub	Computes difference of two arrays.
SubS	Computes difference of array and scalar.
SubRS	Computes difference of scalar and array.
Mul	Calculates per-element product of two arrays.
And	Calculates logical conjunction of two arrays.
AndS	Calculates logical conjunction of an array and a scalar.

Table 14-1 Basic Structures and Operations Functions, Macros, and Data Types (continued)

Name	Description
`Or`	Calculates logical disjunction of two arrays.
`OrS`	Calculates logical disjunction of an array and a scalar.
`Xor`	Calculates logical “exclusive or” operation on two arrays.
`XorS`	Calculates logical “exclusive or” operation on an array and a scalar.
`DotProduct`	Calculates dot product of two arrays in Euclidian metrics.
`CrossProduct`	Calculates the cross product of two 3D vectors.
`ScaleAdd`	Calculates sum of a scaled array and another array.
`MatMulAdd`	Calculates a shifted matrix product.
`MatMulAddS`	Performs matrix transform on every element of an array.
`MulTransposed`	Calculates product of an array and the transposed array.
`Invert`	Inverts an array.
`Trace`	Returns the trace of an array.
`Det`	Returns the determinant of an array.
`Invert`	Inverts an array.
`Mahalonobis`	Calculates the weighted distance between two vectors.
`Transpose`	Transposes an array
`Flip`	Reflects an array around horizontal or vertical axis, or both.
`Reshape`	Changes dimensions and/or number of channels in a matrix.
`SetZero`	Sets the array to zero.
`SetIdentity`	Sets the array to identity.
`SVD`	Performs singular value decomposition of a matrix.
`PseudoInv`	Finds pseudo inverse of a matrix.
`EigenVV`	Computes eigenvalues and eigenvectors of a symmetric array.
`PerspectiveTransform`	Implements general transform of a *3D* vector array.
**Drawing Primitives Functions**	
`Line`	Draws a simple or thick line segment.

Table 14-1 Basic Structures and Operations Functions, Macros, and Data Types (continued)

Name	Description
LineAA	Draws an antialiased line segment.
Rectangle	Draws a simple, thick or filled rectangle.
Circle	Draws a simple, thick or filled circle.
Ellipse	Draws a simple or thick elliptic arc or fills an ellipse sector.
EllipseAA	Draws an antialiased elliptic arc.
FillPoly	Fills an area bounded by several polygonal contours.
FillConvexPoly	Fills convex polygon interior.
PolyLine	Draws a set of simple or thick polylines.
PolyLineAA	Draws a set of antialiased polylines.
InitFont	Initializes the font structure.
PutText	Draws a text string.
GetTextSize	Retrieves width and height of the text string.
**Utility Functions**	
AbsDiff	Calculates absolute difference between two images.
AbsDiffS	Calculates absolute difference between an image and a scalar.
MatchTemplate	Fills a specific image for a given image and template.
CvtPixToPlane	Divides a color image into separate planes.
CvtPlaneToPix	Composes a color image from separate planes.
ConvertScale	Converts one image to another with linear transformation.
LUT	Performs look-up table transformation on an image.
InitLineIterator	Initializes the line iterator and returns the number of pixels between two end points.
SampleLine	Reads a raster line to buffer.
GetRectSubPix	Retrieves a raster rectangle from the image with sub-pixel accuracy.
bFastArctan	Calculates fast arctangent approximation for arrays of abscissas and ordinates.
Sqrt	Calculates square root of a single argument.

Table 14-1 Basic Structures and Operations Functions, Macros, and Data Types (continued)

Name	Description
bSqrt	Calculates the square root of an array of floats.
InvSqrt	Calculates the inverse square root of a single float.
bInvSqrt	Calculates the inverse square root of an array of floats.
bReciprocal	Calculates the inverse of an array of floats.
bCartToPolar	Calculates the magnitude and the angle for an array of abscissas and ordinates.
bFastExp	Calculates fast exponent approximation for each element of the input array of floats.
bFastLog	Calculates fast logarithm approximation for each element of the input array.
RandInit	Initializes state of the random number generator.
bRand	Fills the array with random numbers and updates generator state.
Rand	Fills the array with uniformly distributed random numbers.
FillImage	Fills the image with a constant value.
RandSetRange	Changes the range of generated random numbers without reinitializing RNG state.
KMeans	Splits a set of vectors into a given number of clusters.
	**Data Types**
**Memory Storage**	
CvMemStorage Structure Definition	
CvMemBlock Structure Definition	
CvMemStoragePos Structure Definition	
**Sequence Data**	
CvSequence Structure Definition	Simplifies the extension of the structure CvSeq with additional parameters.
Standard Types of Sequence Elements	Provides definitions of standard sequence elements.
Standard Kinds of Sequences	Specifies the kind of the sequence.

Table 14-1 Basic Structures and Operations Functions, Macros, and Data Types (continued)

Name	Description
`CvSeqBlock` Structure Definition	Defines the building block of sequences.
**Set Data Structures**	
`CvSet` Structure Definition	
`CvSetElem` Structure Definition	
**Graphs Data Structures**	
`CvGraph` Structure Definition	
Definitions of `CvGraphEdge` and `CvGraphVtx` Structures	
**Matrix Operations**	
`CvMat` Structure Definition	Stores real single-precision or double-precision arrays.
`CvMatArray` Structure Definition	Stores arrays of matrices to reduce time call overhead.
**Pixel Access**	
`CvPixelPosition` Structures Definition	
	**Pixel Access Macros**
CV_INIT_PIXEL_POS	Initializes one of `CvPixelPosition` structures.
CV_MOVE_TO	Moves to a specified absolute position.
CV_MOVE	Moves by one pixel relative to the current position.
CV_MOVE_WRAP	Moves by one pixel relative to the current position and wraps when the position reaches the image boundary.
CV_MOVE_PARAM	Moves by one pixel in a specified direction.
CV_MOVE_PARAM_WRAP	Moves by one pixel in a specified direction with wrapping.

Image Functions Reference

CreateImageHeader

Allocates, initializes, and returns structure IplImage.

```
IplImage* cvCreateImageHeader (CvSize size, int depth, int channels);
```

size	Image width and height.
depth	Image depth.
channels	Number of channels.

Discussion

The function `CreateImageHeader` allocates, initializes, and returns the structure `IplImage`. This call is a shortened form of

```
iplCreateImageHeader( channels, 0, depth,
  channels == 1 ? "GRAY" : "RGB",
  channels == 1 ? "GRAY" : channels == 3 ? "BGR" : "BGRA",
  IPL_DATA_ORDER_PIXEL, IPL_ORIGIN_TL, 4,
  size.width, size.height,
  0,0,0,0);
```

CreateImage

Creates header and allocates data.

```
IplImage* cvCreateImage (CvSize size, int depth, int channels);
```

size	Image width and height.
depth	Image depth.

channels Number of channels.

Discussion

The function `CreateImage` creates the header and allocates data. This call is a shortened form of

```
header = cvCreateImageHeader(size,depth,channels);
cvCreateImageData(header);
```

ReleaseImageHeader

Releases header.

```
void cvReleaseImageHeader (IplImage** image);
```

image Double pointer to the deallocated header.

Discussion

The function `ReleaseImageHeader` releases the header. This call is a shortened form of

```
if( image )
{
    iplDeallocate( *image,
                   IPL_IMAGE_HEADER | IPL_IMAGE_ROI );
    *image = 0;
}
```

ReleaseImage

Releases header and image data.

```
void cvReleaseImage (IplImage** image)
```

image Double pointer to the header of the deallocated image.

Discussion

The function `ReleaseImage` releases the header and the image data. This call is a shortened form of

```
if( image )
{
    iplDeallocate( *image, IPL_IMAGE_ALL );
    *image = 0;
}
```

CreateImageData

Allocates image data.

```
void cvCreateImageData (IplImage* image);
```

image Image header.

Discussion

The function `CreateImageData` allocates the image data. This call is a shortened form of

```
if( image->depth == IPL_DEPTH_32F )
{
    iplAllocateImageFP( image, 0, 0 );
}
else
{
    iplAllocateImage( image, 0, 0 );
}
```

ReleaseImageData

Releases image data.

```
void cvReleaseImageData (IplImage* image);
```

image Image header.

Discussion

The function `ReleaseImageData` releases the image data. This call is a shortened form of

```
iplDeallocate( image, IPL_IMAGE_DATA );
```

SetImageData

Sets pointer to data and step parameters to given values.

```
void cvSetImageData (IplImage* image, void* data, int step);
```

image Image header.

data User data.

step Distance between the raster lines in bytes.

Discussion

The function `SetImageData` sets the pointer to *data* and *step* parameters to given values.

SetImageCOI

Sets channel of interest to given value.

```
void cvSetImageCOI (IplImage* image, int coi);
```

image	Image header.
coi	Channel of interest.

Discussion

The function `SetImageCOI` sets the channel of interest to a given value. If ROI is `NULL` and `coi != 0`, ROI is allocated.

SetImageROI

Sets image ROI to given rectangle.

```
void cvSetImageROI (IplImage* image, CvRect rect);
```

image	Image header.
rect	ROI rectangle.

Discussion

The function `SetImageROI` sets the image ROI to a given rectangle. If ROI is `NULL` and the value of the parameter `rect` is not equal to the whole image, ROI is allocated.

GetImageRawData

Fills output variables with image parameters.

```
void cvGetImageRawData (const IplImage* image, uchar** data, int* step,
    CvSize* roiSize);
```

image	Image header.
data	Pointer to the top-left corner of ROI.
step	Full width of the raster line, equals to `image->widthStep`.
roiSize	ROI width and height.

Discussion

The function `GetImageRawData` fills output variables with the image parameters. All output parameters are optional and could be set to `NULL`.

InitImageHeader

Initializes image header structure without memory allocation.

```
void cvInitImageHeader (IplImage* image, CvSize size, int depth, int channels,
    int origin, int align, int clear);
```

image	Image header.
size	Image width and height.
depth	Image depth.
channels	Number of channels.
origin	`IPL_ORIGIN_TL` or `IPL_ORIGIN_BL`.
align	Alignment for the raster lines.

clear	If the parameter value equals 1, the header is cleared before initialization.

Discussion

The function `InitImageHeader` initializes the image header structure without memory allocation.

CopyImage

Copies entire image to another without considering ROI.

```
void cvCopyImage (IplImage* src, IplImage* dst);
```

src	Source image.
dst	Destination image.

Discussion

The function `CopyImage` copies the entire image to another without considering ROI. If the destination image is smaller, the destination image data is reallocated.

Pixel Access Macros

This section describes macros that are useful for fast and flexible access to image pixels. The basic ideas behind these macros are as follows:

1. Some structures of `CvPixelAccess` type are introduced. These structures contain all information about ROI and its current position. The only difference across all these structures is the data type, not the number of channels.
2. There exist fast versions for moving in a specific direction, e.g., `CV_MOVE_LEFT`, wrap and non-wrap versions. More complicated and slower macros are used for moving in an arbitrary direction that is passed as a parameter.

3. Most of the macros require the parameter *cs* that specifies the number of the image channels to enable the compiler to remove superfluous multiplications in case the image has a single channel, and substitute faster machine instructions for them in case of three and four channels.

Example 14-1 `CvPixelPosition` Structures Definition

```
typedef struct _CvPixelPosition8u
{
    unsigned char*   currline;
                  /* pointer to the start of the current
                     pixel line */
    unsigned char*   topline;
                  /* pointer to the start of the top pixel
                     line */
    unsigned char*   bottomline;
                  /* pointer to the start of the first
                     line which is below the image */
    int    x;   /* current x coordinate ( in pixels ) */
    int    width; /* width of the image ( in pixels )*/
    int    height; /* height of the image ( in pixels )*/
    int     step;  /* distance between lines ( in
                      elements of single plane ) */
    int     step_arr[3]; /* array: ( 0, -step, step ).
                            It is used for vertical
                            moving */
} CvPixelPosition8u;

/*this structure differs from the above only in data type*/
typedef struct _CvPixelPosition8s
{
    char*   currline;
    char*   topline;
    char*   bottomline;
    int     x;
    int     width;
    int     height;
    int     step;
    int     step_arr[3];
} CvPixelPosition8s;

/* this structure differs from the CvPixelPosition8u only in data type
*/
typedef struct _CvPixelPosition32f
{
    float*  currline;
    float*  topline;
    float*  bottomline;
    int     x;
```

Example 14-1 CvPixelPosition Structures Definition (continued)

```
    int     width;
    int     height;
    int     step;
    int     step_arr[3];
} CvPixelPosition32f;
```

CV_INIT_PIXEL_POS

Initializes one of CvPixelPosition structures.

```
#define CV_INIT_PIXEL_POS( pos, origin, step, roi, x, y, orientation )
```

pos	Initialization of structure.
origin	Pointer to the left-top corner of ROI.
step	Width of the whole image in bytes.
roi	Width and height of ROI.
x, y	Initial position.
orientation	Image orientation; could be either<br>`CV_ORIGIN_TL` - top/left orientation, or<br>`CV_ORIGIN_BL` - bottom/left orientation.

CV_MOVE_TO

Moves to specified absolute position.

```
#define CV_MOVE_TO( pos, x, y, cs )
```

pos	Position structure.
x, y	Coordinates of the new position.
cs	Number of the image channels.

CV_MOVE

Moves by one pixel relative to current position.

```
#define CV_MOVE_LEFT( pos, cs )
#define CV_MOVE_RIGHT( pos, cs )
#define CV_MOVE_UP( pos, cs )
#define CV_MOVE_DOWN( pos, cs )
#define CV_MOVE_LU( pos, cs )
#define CV_MOVE_RU( pos, cs )
#define CV_MOVE_LD( pos, cs )
#define CV_MOVE_RD( pos, cs )
```

`pos`	Position structure.
`cs`	Number of the image channels.

CV_MOVE_WRAP

Moves by one pixel relative to current position and wraps when position reaches image boundary.

```
#define CV_MOVE_LEFT_WRAP( pos, cs )
#define CV_MOVE_RIGHT_WRAP( pos, cs )
#define CV_MOVE_UP_WRAP( pos, cs )
#define CV_MOVE_DOWN_WRAP( pos, cs )
#define CV_MOVE_LU_WRAP( pos, cs )
#define CV_MOVE_RU_WRAP( pos, cs )
#define CV_MOVE_LD_WRAP( pos, cs )
#define CV_MOVE_RD_WRAP( pos, cs )
```

`pos`	Position structure.

cs Number of the image channels.

CV_MOVE_PARAM

Moves by one pixel in specified direction.

```
#define CV_MOVE_PARAM( pos, shift, cs )
```

pos Position structure.

cs Number of the image channels.

shift Direction; could be any of the following:

CV_SHIFT_NONE,
CV_SHIFT_LEFT,
CV_SHIFT_RIGHT,
CV_SHIFT_UP,
CV_SHIFT_DOWN,
CV_SHIFT_UL,
CV_SHIFT_UR,
CV_SHIFT_DL.

CV_MOVE_PARAM_WRAP

Moves by one pixel in specified direction with wrapping.

```
#define CV_MOVE_PARAM_WRAP( pos, shift, cs )
```

pos Position structure.

cs Number of the image channels.

shift Direction; could be any of the following:

CV_SHIFT_NONE,

CV_SHIFT_LEFT,

CV_SHIFT_RIGHT,

CV_SHIFT_UP,

CV_SHIFT_DOWN,

CV_SHIFT_UL,

CV_SHIFT_UR,

CV_SHIFT_DL.

Dynamic Data Structures Reference

Memory Storage Reference

Example 14-2 CvMemStorage Structure Definition

```
typedef struct CvMemStorage
{
    CvMemBlock* bottom;/* first allocated block */
    CvMemBlock* top; /*current memory block - top of the stack */
    struct  CvMemStorage* parent; /* borrows new blocks from */
    int     block_size;  /* block size */
    int     free_space;  /* free space in the current block */
} CvMemStorage;
```

Example 14-3 CvMemBlock Structure Definition

```
typedef struct CvMemBlock
{
    struct CvMemBlock*  prev;
    struct CvMemBlock*  next;
} CvMemBlock;
```

Actual data of the memory blocks follows the header, that is, the ith byte of the memory block can be retrieved with the expression $((char*)(mem\_block\_ptr + 1))[i]$. However, the occasions on which the need for direct access to the memory blocks arises are quite rare. The structure described below stores the position of the stack top that can be saved/restored:

Example 14-4 CvMemStoragePos Structure Definition

```
typedef struct CvMemStoragePos
{
    CvMemBlock* top;
    int  free_space;
}
CvMemStoragePos;
```

CreateMemStorage

Creates memory storage.

```
CvMemStorage* cvCreateMemStorage (int blockSize=0);
```

blockSize Size of the memory blocks in the storage; bytes.

Discussion

The function `CreateMemStorage` creates a memory storage and returns the pointer to it. Initially the storage is empty. All fields of the header are set to 0. The parameter *blockSize* must be positive or zero; if the parameter equals 0, the block size is set to the default value, currently 64K.

CreateChildMemStorage

Creates child memory storage.

```
CvMemStorage* cvCreateChildMemStorage (CvMemStorage* parent);
```

parent Parent memory storage.

Discussion

The function `CreateChildMemStorage` creates a child memory storage similar to the simple memory storage except for the differences in the memory allocation/de-allocation mechanism. When a child storage needs a new block to add to the block list, it tries to get this block from the parent. The first unoccupied parent block available is taken and excluded from the parent block list. If no blocks are available, the parent either allocates a block or borrows one from its own parent, if any. In other words, the chain, or a more complex structure, of memory storages where every storage is a child/parent of another is possible. When a child storage is released or even cleared, it returns all blocks to the parent. Note again, that in other aspects, the child storage is the same as the simple storage.

ReleaseMemStorage

Releases memory storage.

```
void cvReleaseMemStorage (CvMemStorage** storage);
```

storage Pointer to the released storage.

Discussion

The function `ReleaseMemStorage` de-allocates all storage memory blocks or returns them to the parent, if any. Then it de-allocates the storage header and clears the pointer to the storage. All children of the storage must be released before the parent is released.

ClearMemStorage

Clears memory storage.

```
void cvClearMemStorage (CvMemStorage* storage);
```

storage Memory storage.

Discussion

The function `ClearMemStorage` resets the top (free space boundary) of the storage to the very beginning. This function does not de-allocate any memory. If the storage has a parent, the function returns all blocks to the parent.

SaveMemStoragePos

Saves memory storage position.

```
void cvSaveMemStoragePos (CvMemStorage* storage, CvMemStoragePos* pos);
```

storage	Memory storage.
pos	Currently retrieved position of the in-memory storage top.

Discussion

The function `SaveMemStoragePos` saves the current position of the storage top to the parameter *pos*. The function `RestoreMemStoragePos` can further retrieve this position.

RestoreMemStoragePos

Restores memory storage position.

```
void cvRestoreMemStoragePos (CvMemStorage* storage, CvMemStoragePos* pos);
```

storage	Memory storage.
pos	New storage top position.

Discussion

The function `RestoreMemStoragePos` restores the position of the storage top from the parameter *pos*. This function and the function `ClearMemStorage` are the only methods to release memory occupied in memory blocks.

In other words, the occupied space and free space in the storage are continuous. If the user needs to process data and put the result to the storage, there arises a need for the storage space to be allocated for temporary results. In this case the user may simply write all the temporary data to that single storage. However, as a result garbage appears in the middle of the occupied part. See Figure 14-1.

Figure 14-1 Storage Allocation for Temporary Results

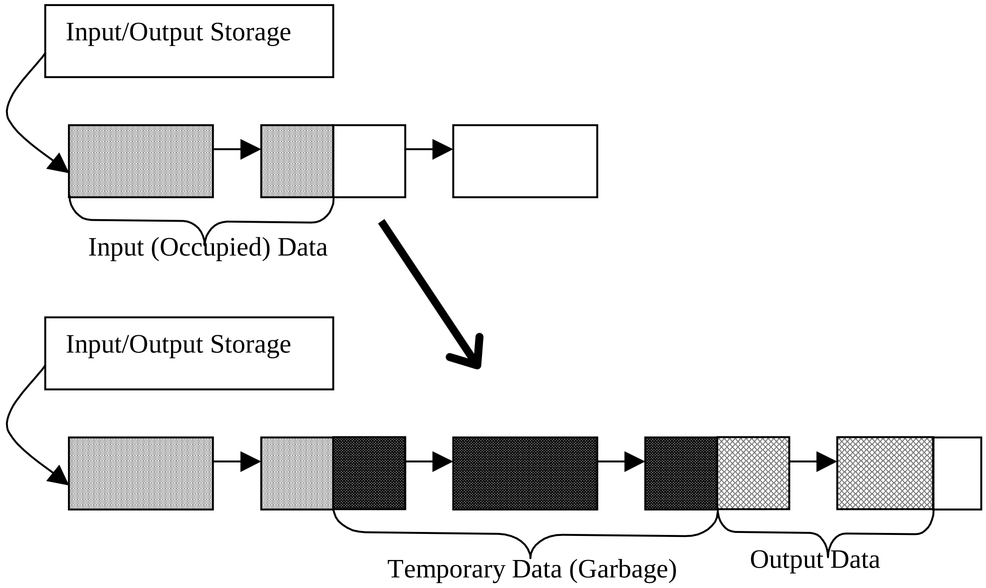

Saving/Restoring does not work in this case. Creating a child memory storage, however, can resolve this problem. The algorithm writes to both storages simultaneously, and, once done, releases the temporary storage. See Figure 14-2.

Figure 14-2 Release of Temporary Storage

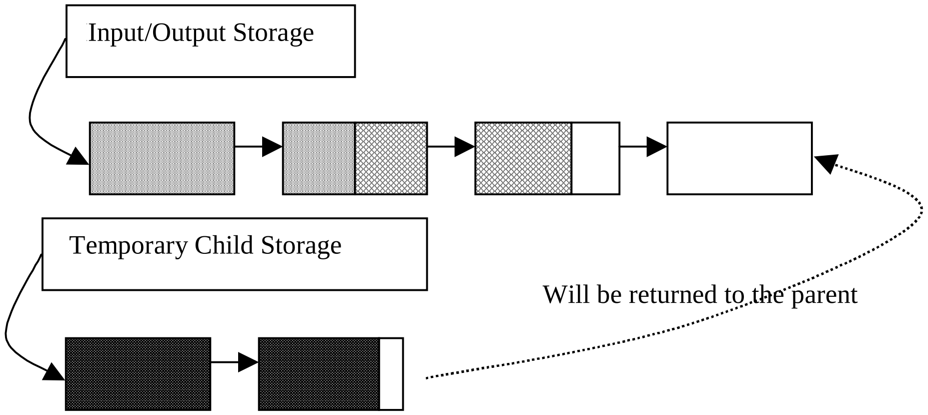

Sequence Reference

Example 14-5 `CvSequence` Structure Definition

```
#define CV_SEQUENCE_FIELDS()                                    \
    int       header_size;   /* size of sequence header */      \
    struct    CvSeq* h_prev; /* previous sequence */            \
    struct    CvSeq* h_next; /* next sequence */                \
    struct    CvSeq* v_prev; /* 2nd previous sequence */        \
    struct    CvSeq* v_next; /* 2nd next sequence */            \
    int       flags;         /* micsellaneous flags */          \
    int       total;         /* total number of elements */     \
    int       elem_size;/* size of sequence element in bytes */ \
    char*     block_max;/* maximal bound of the last block */   \
    char*     ptr;      /* current write pointer */             \
    int       delta_elems; /* how many elements allocated when the seq
grows */                                                \
    CvMemStorage* storage;  /* where the seq is stored */       \
    CvSeqBlock* free_blocks;  /* free blocks list */            \
    CvSeqBlock* first; /* pointer to the first sequence block */

typedef struct CvSeq
{
    CV_SEQUENCE_FIELDS()
} CvSeq;
```

Such an unusual definition simplifies the extension of the structure *CvSeq* with additional parameters. To extend *CvSeq* the user may define a new structure and put user-defined fields after all *CvSeq* fields that are included via the macro `CV_SEQUENCE_FIELDS()`. The field *header_size* contains the actual size of the sequence header and must be more than or equal to *sizeof(CvSeq)*. The fields *h_prev*, *h_next*, *v_prev*, *v_next* can be used to create hierarchical structures from separate sequences. The fields *h_prev* and *h_next* point to the previous and the next sequences on the same hierarchical level while the fields *v_prev* and *v_next* point to the previous and the next sequence in the vertical direction, that is, parent and its first child. But these are just names and the pointers can be used in a different way. The field *first* points to the first sequence block, whose structure is described below. The field flags contain miscellaneous information on the type of the sequence and should be discussed in greater detail. By convention, the lowest `CV_SEQ_ELTYPE_BITS` bits contain the ID of the element type. The current version has `CV_SEQ_ELTYPE_BITS` equal to 5, that is, it supports up to 32 non-overlapping element types now. The file `CVTypes.h` declares the predefined types.

Example 14-6 Standard Types of Sequence Elements

```
#define CV_SEQ_ELTYPE_POINT          1 /* (x,y) */
#define CV_SEQ_ELTYPE_CODE           2 /* freeman code: 0..7 */
#define CV_SEQ_ELTYPE_PPOINT         3 /* &(x,y) */
#define CV_SEQ_ELTYPE_INDEX          4 /* #(x,y) */
#define CV_SEQ_ELTYPE_GRAPH_EDGE     5 /* &next_o,&next_d,&vtx_o,
&vtx_d */
#define CV_SEQ_ELTYPE_GRAPH_VERTEX   6 /* first_edge, &(x,y) */
#define CV_SEQ_ELTYPE_TRIAN_ATR      7 /* vertex of the binary tree
*/
#define CV_SEQ_ELTYPE_CONNECTED_COMP 8 /* connected component  */
#define CV_SEQ_ELTYPE_POINT3D        9  /* (x,y,z)  */
```

The next `CV_SEQ_KIND_BITS` bits, also 5 in number, specify the kind of the sequence. Again, predefined kinds of sequences are declared in the file `CVTypes.h`.

Example 14-7 Standard Kinds of Sequences

```
#define CV_SEQ_KIND_SET       (0 << CV_SEQ_ELTYPE_BITS)
#define CV_SEQ_KIND_CURVE     (1 << CV_SEQ_ELTYPE_BITS)
#define CV_SEQ_KIND_BIN_TREE  (2 << CV_SEQ_ELTYPE_BITS)
#define CV_SEQ_KIND_GRAPH     (3 << CV_SEQ_ELTYPE_BITS)
```

The remaining bits are used to identify different features specific to certain sequence kinds and element types. For example, curves made of points (`CV_SEQ_KIND_CURVE|CV_SEQ_ELTYPE_POINT`), together with the flag `CV_SEQ_FLAG_CLOSED` belong to the type `CV_SEQ_POLYGON` or, if other flags are used, its subtype. Many contour processing functions check the type of the input sequence and report an error if they do not support this type. The file `CVTypes.h` stores the complete list of all supported predefined sequence types and helper macros designed to get the sequence type of other properties.

Below follows the definition of the building block of sequences.

Example 14-8 `CvSeqBlock` Structure Definition

```
typedef struct CvSeqBlock
{
    struct CvSeqBlock*  prev; /* previous sequence block */
    struct CvSeqBlock*  next; /* next sequence block */
    int    start_index; /* index of the first element in the block +
sequence->first->start_index */
    int    count; /* number of elements in the block */
    char*  data;  /* pointer to the first element of the block */
} CvSeqBlock;
```

Sequence blocks make up a circular double-linked list, so the pointers *`prev`* and *`next`* are never `NULL` and point to the previous and the next sequence blocks within the sequence. It means that *`next`* of the last block is the first block and *`prev`* of the first block is the last block. The fields *`start_index`* and *`count`* help to track the block location within the sequence. For example, if the sequence consists of 10 elements and splits into three blocks of 3, 5, and 2 elements, and the first block has the parameter *`start_index = 2`*, then pairs <*`start_index`*, *`count`*> for the sequence blocks are <*`2,3`*>, <*`5,5`*>, and <*`10,2`*> correspondingly. The parameter *`start_index`* of the first block is usually *`0`* unless some elements have been inserted at the beginning of the sequence.

CreateSeq

Creates sequence.

```
CvSeq* cvCreateSeq (int seqFlags, int headerSize, int elemSize, CvMemStorage*
    storage);
```

seqFlags	Flags of the created sequence. If the sequence is not passed to any function working with a specific type of sequences, the sequence value may be equal to 0, otherwise the appropriate type must be selected from the list of predefined sequence types.
headerSize	Size of the sequence header; must be more than or equal to *sizeof(CvSeq)*. If a specific type or its extension is indicated, this type must fit the base type header.
elemSize	Size of the sequence elements in bytes. The size must be consistent with the sequence type. For example, for a sequence of points to be created, the element type `CV_SEQ_ELTYPE_POINT` should be specified and the parameter *elemSize* must be equal to *sizeof(CvPoint)*.
storage	Sequence location.

Discussion

The function `CreateSeq` creates a sequence and returns the pointer to it. The function allocates the sequence header in the storage block as one continuous chunk and fills the parameter *elemSize*, flags *headerSize*, and *storage* with passed values, sets the parameter *deltaElems* (see the function `SetSeqBlockSize`) to the default value, and clears other fields, including the space behind *sizeof(CvSeq)*.

NOTE. *All headers in the memory storage, including sequence headers and sequence block headers, are aligned with the 4-byte boundary.*

SetSeqBlockSize

Sets up sequence block size.

```
void cvSetSeqBlockSize (CvSeq* seq, int blockSize);
```

`seq` Sequence.

`blockSize` Desirable block size.

Discussion

The function `SetSeqBlockSize` affects the memory allocation granularity. When the free space in the internal sequence buffers has run out, the function allocates `blockSize` bytes in the storage. If this block immediately follows the one previously allocated, the two blocks are concatenated, otherwise, a new sequence block is created. Therefore, the bigger the parameter, the lower the sequence fragmentation probability, but the more space in the storage is wasted. When the sequence is created, the parameter `blockSize` is set to the default value ~1K. The function can be called any time after the sequence is created and affects future allocations. The final block size can be different from the one desired, e.g., if it is larger than the storage block size, or smaller than the sequence header size plus the sequence element size.

The next four functions `SeqPush`, `SeqPop`, `SeqPushFront`, `SeqPopFront` add or remove elements to/from one of the sequence ends. Their time complexity is `O(1)`, that is, all these operations do not shift existing sequence elements.

SeqPush

Adds element to sequence end.

```
void cvSeqPush (CvSeq* seq, void* element);
```

`seq` Sequence.

`element` Added element.

Discussion

The function `SeqPush` adds an element to the end of the sequence. Although this function can be used to create a sequence element by element, there is a faster method (refer to Writing and Reading Sequences).

SeqPop

Removes element from sequence end.

```
void cvSeqPop (CvSeq* seq, void* element);
```

seq	Sequence.
element	Optional parameter. If the pointer is not zero, the function copies the removed element to this location.

Discussion

The function `SeqPop` removes an element from the sequence. The function reports an error if the sequence is already empty.

SeqPushFront

Adds element to sequence beginning.

```
void cvSeqPushFront (CvSeq* seq, void* element);
```

seq	Sequence.
element	Added element.

Discussion

The function `SeqPushFront` adds an element to the beginning of the sequence.

SeqPopFront

Removes element from sequence beginning.

```
void cvSeqPopFront (CvSeq* seq, void* element);
```

seq	Sequence.
element	Optional parameter. If the pointer is not zero, the function copies the removed element to this location.

Discussion

The function `SeqPopFront` removes an element from the beginning of the sequence. The function reports an error if the sequence is already empty.

Next two functions `SeqPushMulti`, `SeqPopMulti` are batch versions of the PUSH/POP operations.

SeqPushMulti

Pushes several elements to sequence end.

```
void cvSeqPushMulti (CvSeq* seq, void* elements, int count);
```

seq	Sequence.
elements	Added elements.
count	Number of elements to push.

Discussion

The function `SeqPushMulti` adds several elements to the end of the sequence. The elements are added to the sequence in the same order as they are arranged in the input array but they can fall into different sequence blocks.

SeqPopMulti

Removes several elements from sequence end.

```
void cvSeqPopMulti (CvSeq* seq, void* elements, int count);
```

seq	Sequence.
elements	Removed elements.
count	Number of elements to pop.

Discussion

The function `SeqPopMulti` removes several elements from the end of the sequence. If the number of the elements to be removed exceeds the total number of elements in the sequence, the function removes as many elements as possible.

SeqInsert

Inserts element in sequence middle.

```
void cvSeqInsert (CvSeq* seq, int beforeIndex, void* element);
```

seq	Sequence.
beforeIndex	Index before which the element is inserted. Inserting before 0 is equal to *cvSeqPushFront* and inserting before *seq->total* is equal to *cvSeqPush*. The index values in these two examples are boundaries for allowed parameter values.
element	Inserted element.

Discussion

The function `SeqInsert` shifts the sequence elements from the inserted position to the nearest end of the sequence before it copies an element there, therefore, the algorithm time complexity is `O(n/2)`.

SeqRemove

Removes element from sequence middle.

```
void cvSeqRemove (CvSeq* seq, int index);
```

seq Sequence.

index Index of removed element.

Discussion

The function `SeqRemove` removes elements with the given index. If the index is negative or greater than the total number of elements less 1, the function reports an error. An attempt to remove an element from an empty sequence is a specific case of this situation. The function removes an element by shifting the sequence elements from the nearest end of the sequence *index*.

ClearSeq

Clears sequence.

```
void cvClearSeq (CvSeq* seq);
```

seq Sequence.

Discussion

The function `ClearSeq` empties the sequence. The function does not return the memory to the storage, but this memory is used again when new elements are added to the sequence. This function time complexity is `O(1)`.

GetSeqElem

Returns n-th element of sequence.

```
char* cvGetSeqElem (CvSeq* seq, int index, CvSeqBlock** block=0);
```

seq	Sequence.
index	Index of element.
block	Optional argument. If the pointer is not `NULL`, the address of the sequence block that contains the element is stored in this location.

Discussion

The function `GetSeqElem` finds the element with the given index in the sequence and returns the pointer to it. In addition, the function can return the pointer to the sequence block that contains the element. If the element is not found, the function returns 0. The function supports negative indices, where -1 stands for the last sequence element, -2 stands for the one before last, etc. If the sequence is most likely to consist of a single sequence block or the desired element is likely to be located in the first block, then the macro `CV_GET_SEQ_ELEM` (`elemType`, `seq`, `index`) should be used, where the parameter *`elemType`* is the type of sequence elements (*`CvPoint`* for example), the parameter *`seq`* is a sequence, and the parameter *`index`* is the index of the desired element. The macro checks first whether the desired element belongs to the first block of the sequence and, if so, returns the element, otherwise the macro calls the main function `GetSeqElem`. Negative indices always cause the *`cvGetSeqElem`* call.

SeqElemIdx

Returns index of concrete sequence element.

```
int cvSeqElemIdx (CvSeq* seq, void* element, CvSeqBlock** block=0);
```

seq	Sequence.
element	Pointer to the element within the sequence.

block Optional argument. If the pointer is not `NULL`, the address of the sequence block that contains the element is stored in this location.

Discussion

The function `SeqElemIdx` returns the index of a sequence element or a negative number if the element is not found.

CvtSeqToArray

Copies sequence to one continuous block of memory.

```
void* cvCvtSeqToArray (CvSeq* seq, void* array, CvSlice
slice=CV_WHOLE_SEQ(seq));
```

seq Sequence.

array Pointer to the destination array that must fit all the sequence elements.

slice Start and end indices within the sequence so that the corresponding subsequence is copied.

Discussion

The function `CvtSeqToArray` copies the entire sequence or subsequence to the specified buffer and returns the pointer to the buffer.

MakeSeqHeaderForArray

Constructs sequence from array.

```
void cvMakeSeqHeaderForArray (int seqType, int headerSize, int elemSize, void*
    array, int total, CvSeq* sequence, CvSeqBlock* block);
```

seqType	Type of the created sequence.
headerSize	Size of the header of the sequence. Parameter sequence must point to the structure of that size or greater size.
elemSize	Size of the sequence element.
array	Pointer to the array that makes up the sequence.
total	Total number of elements in the sequence. The number of array elements must be equal to the value of this parameter.
sequence	Pointer to the local variable that is used as the sequence header.
block	Pointer to the local variable that is the header of the single sequence block.

Discussion

The function `MakeSeqHeaderForArray`, the exact opposite of the function `CvtSeqToArray`, builds a sequence from an array. The sequence always consists of a single sequence block, and the total number of elements may not be greater than the value of the parameter *`total`*, though the user may remove elements from the sequence, then add other elements to it with the above restriction.

Writing and Reading Sequences Reference

StartAppendToSeq

Initializes process of writing to sequence.

```
void cvStartAppendToSeq (CvSeq* seq, CvSeqWriter* writer);
```

seq	Pointer to the sequence.
writer	Pointer to the working structure that contains the current status of the writing process.

Discussion

The function `StartAppendToSeq` initializes the writer to write to the sequence. Written elements are added to the end of the sequence. Note that during the writing process other operations on the sequence may yield incorrect result or even corrupt the sequence (see Discussion of the function `FlushSeqWriter`).

StartWriteSeq

Creates new sequence and initializes writer for it.

```
void cvStartWriteSeq (int seqFlags, int headerSize, int elemSize,
    CvMemStorage* storage, CvSeqWriter* writer);
```

seqFlags	Flags of the created sequence. If the sequence is not passed to any function working with a specific type of sequences, the sequence value may be equal to 0, otherwise the appropriate type must be selected from the list of predefined sequence types.
headerSize	Size of the sequence header. The parameter value may not be less than *sizeof(CvSeq)*. If a certain type or extension is specified, it must fit the base type header.
elemSize	Size of the sequence elements in bytes; must be consistent with the sequence type. For example, if the sequence of points is created (element type `CV_SEQ_ELTYPE_POINT`), then the parameter *elemSize* must be equal to *sizeof(CvPoint)*.
storage	Sequence location.
writer	Pointer to the writer status.

Discussion

The function `StartWriteSeq` is the exact sum of the functions `CreateSeq` and `StartAppendToSeq`.

EndWriteSeq

Finishes process of writing.

```
CvSeq* cvEndWriteSeq (CvSeqWriter* writer);
```

writer Pointer to the writer status.

Discussion

The function `EndWriteSeq` finishes the writing process and returns the pointer to the resulting sequence. The function also truncates the last sequence block to return the whole of unfilled space to the memory storage. After that the user may read freely from the sequence and modify it.

FlushSeqWriter

Updates sequence headers using writer state.

```
void cvFlushSeqWriter (CvSeqWriter* writer);
```

writer Pointer to the writer status.

Discussion

The function `FlushSeqWriter` is intended to enable the user to read sequence elements, whenever required, during the writing process, e.g., in order to check specific conditions. The function updates the sequence headers to make reading from the sequence possible. The writer is not closed, however, so that the writing process can be continued any time. Frequent flushes are not recommended, the function `SeqPush` is preferred.

StartReadSeq

Initializes process of sequential reading from sequence.

```
void cvStartReadSeq( CvSeq* seq, CvSeqReader* reader, int reverse=0 );
```

seq Sequence.

reader Pointer to the reader status.

reverse Whenever the parameter value equals 0, the reading process is going in the forward direction, that is, from the beginning to the end, otherwise the reading process direction is reverse, from the end to the beginning.

Discussion

The function `StartReadSeq` initializes the reader structure. After that all the sequence elements from the first down to the last one can be read by subsequent calls of the macro `CV_READ_SEQ_ELEM` (`elem`, `reader`) that is similar to `CV_WRITE_SEQ_ELEM`. The function puts the reading pointer to the last sequence element if the parameter *reverse* does not equal zero. After that the macro `CV_REV_READ_SEQ_ELEM` (`elem`, `reader`) can be used to get sequence elements from the last to the first. Both macros put the sequence element to *elem* and move the reading pointer forward (`CV_READ_SEQ_ELEM`) or backward (`CV_REV_READ_SEQ_ELEM`). A circular structure of sequence blocks is used for the reading process, that is, after the last element has been read by the macro `CV_READ_SEQ_ELEM`, the first element is read when the macro is called again. The same applies to `CV_REV_READ_SEQ_ELEM`. Neither function ends reading since the reading process does not modify the sequence, nor requires any temporary buffers. The reader field *ptr* points to the current element of the sequence that is to be read first.

GetSeqReaderPos

Returns index of element to read position.

```
int cvGetSeqReaderPos (CvSeqReader* reader);
```

reader Pointer to the reader status.

Discussion

The function `GetSeqReaderPos` returns the index of the element in which the reader is currently located.

SetSeqReaderPos

Moves read position to specified index.

```
void cvSetSeqReaderPos (CvSeqReader* reader, int index, int is_relative=0);
```

reader Pointer to the reader status.

index Position where the reader must be moved.

is_relative If the parameter value is not equal to zero, the index means an offset relative to the current position.

Discussion

The function `SetSeqReaderPos` moves the read position to the absolute or relative position. This function allows for cycle character of the sequence.

Sets Reference

CreateSet

Creates empty set.

```
CvSet* cvCreateSet (int setFlags, int headerSize, int elemSize, CvMemStorage*
    storage);
```

setFlags	Type of the created set.
headerSize	Set header size; may not be less than *sizeof(CvSeq)*.
elemSize	Set element size; may not be less than 8 bytes, must be divisible by 4.
storage	Future set location.

Discussion

The function `CreateSet` creates an empty set with a specified header size and returns the pointer to the set. The function simply redirects the call to the function `CreateSeq`.

SetAdd

Adds element to set.

```
int cvSetAdd (CvSet* set, CvSet* elem, CvSet** insertedElem=0);
```

set	Set.
elem	Optional input argument, inserted element. If not `NULL`, the function copies the data to the allocated cell omitting the first 4-byte field.
insertedElem	Optional output argument; points to the allocated cell.

Discussion

The function `SetAdd` allocates a new cell, optionally copies input element data to it, and returns the pointer and the index to the cell. The index value is taken from the second 4-byte field of the cell. In case the cell was previously deleted and a wrong index was specified, the function returns this wrong index. However, if the user works in the pointer mode, no problem occurs and the pointer stored at the parameter *`insertedElem`* may be used to get access to the added set element.

SetRemove

Removes element from set.

```
void cvSetRemove (CvSet* set, int index);
```

`set`	Set.
`index`	Index of the removed element.

Discussion

The function `SetRemove` removes an element with a specified index from the set. The function is typically used when set elements are accessed by their indices. If pointers are used, the macro `CV_REMOVE_SET_ELEM(set, index, elem)`, where `elem` is a pointer to the removed element and `index` is any non-negative value, may be used to remove the element. Alternative way to remove an element by its pointer is to calculate index of the element via the function `SeqElemIdx` after which the function `SetRemove` may be called, but this method is much slower than the macro.

GetSetElem

Finds set element by index.

```
CvSetElem* cvGetSetElem (CvSet* set, int index);
```

`set` Set.

`index` Index of the set element within a sequence.

Discussion

The function `GetSetElem` finds a set element by index. The function returns the pointer to it or 0 if the index is invalid or the corresponding cell is free. The function supports negative indices through calling the function `GetSeqElem`.

NOTE. *The user can check whether the element belongs to the set with the help of the macro* `CV_IS_SET_ELEM_EXISTS(elem)` *once the pointer is set to a set element.*

ClearSet

Clears set.

```
void cvClearSet (CvSet* set);
```

`set` Cleared set.

Discussion

The function `ClearSet` empties the set by calling the function `ClearSeq` and setting the pointer to the list of free cells. The function takes *O(1)* time.

Sets Data Structures

Example 14-9 CvSet Structure Definition

```
#define CV_SET_FIELDS()      \
    CV_SEQUENCE_FIELDS()     \
    CvMemBlock* free_elems;

typedef struct CvSet
{
    CV_SET_FIELDS()
}
CvSet;
```

Example 14-10 CvSetElem Structure Definition

```
#define CV_SET_ELEM_FIELDS()    \
    int*  aligned_ptr;
typedef struct _CvSetElem
{
    CV_SET_ELEM_FIELDS()
}
CvSetElem;
```

The first field is a dummy field and is not used in the occupied cells, except the least significant bit, which is 0. With this structure the integer element could be defined as follows:

```
typedef struct _IntSetElem
{
    CV_SET_ELEM_FIELDS()
    int value;
}
IntSetElem;
```

Graphs Reference

CreateGraph

Creates empty graph.

```
CvGraph* cvCreateGraph (int graphFlags, int headerSize, int vertexSize, int
    edgeSize, CvStorage* storage);
```

graphFlags Type of the created graph. The kind of the sequence must be graph (CV_SEQ_KIND_GRAPH) and flag CV_GRAPH_FLAG_ORIENTED allows the oriented graph to be created. User may choose other flags, as well as types of graph vertices and edges.

headerSize Graph header size; may not be less than *sizeof(CvGraph)*.

vertexSize Graph vertex size; must be greater than *sizeof(CvGraphVertex)* and meet all restrictions on the set element.

edgeSize Graph edge size; may not be less than *sizeof(CvGraphEdge)* and must be divisible by 4.

storage Future location of the graph.

Discussion

The function CreateGraph creates an empty graph, that is, two empty sets, a set of vertices and a set of edges, and returns it.

GraphAddVtx

Adds vertex to graph.

```
int cvGraphAddVtx (CvGraph* graph, CvGraphVtx* vtx, CvGraphVtx**
    insertedVtx=0);
```

graph	Graph.
vtx	Optional input argument. Similar to the parameter *elem* of the function SetAdd, the parameter *vtx* could be used to initialize new vertices with concrete values. If *vtx* is not NULL, the function copies it to a new vertex, except the first 4-byte field.
insertedVtx	Optional output argument. If not NULL, the address of the new vertex is written there.

Discussion

The function GraphAddVtx adds a vertex to the graph and returns the vertex index.

GraphRemoveVtx

Removes vertex from graph.

```
void cvGraphRemoveAddVtx (CvGraph* graph, int vtxIdx);
```

graph	Graph.
vtxIdx	Index of the removed vertex.

Discussion

The function GraphRemoveAddVtx removes a vertex from the graph together with all the edges incident to it. The function reports an error, if input vertices do not belong to the graph, that makes it safer than GraphRemoveVtxByPtr, but less efficient.

GraphRemoveVtxByPtr

Removes vertex from graph.

```
void cvGraphRemoveVtxByPtr (CvGraph* graph, CvGraphVtx* vtx);
```

graph	Graph.

vtx Pointer to the removed vertex.

Discussion

The function `GraphRemoveVtxByPtr` removes a vertex from the graph together with all the edges incident to it. The function is more efficient than `GraphRemoveVtx` but less safe, because it does not check whether the input vertices belong to the graph.

GraphAddEdge

Adds edge to graph.

```
int cvGraphAddEdge (CvGraph* graph, int startIdx, int endIdx, CvGraphEdge*
    edge, CvGraphEdge** insertedEdge=0);
```

graph	Graph.
startIdx	Index of the starting vertex of the edge.
endIdx	Index of the ending vertex of the edge.
edge	Optional input parameter, initialization data for the edge. If not `NULL`, the parameter is copied starting from the 5th 4-byte field.
insertedEdge	Optional output parameter to contain the address of the inserted edge within the edge set.

Discussion

The function `GraphAddEdge` adds an edge to the graph given the starting and the ending vertices. The function returns the index of the inserted edge, which is the value of the second 4-byte field of the free cell.

The function reports an error if

- the edge that connects the vertices already exists; in this case graph orientation is taken into account;
- a pointer is `NULL` or indices are invalid;
- some of vertices do not exist, that is, not checked when the pointers are passed to vertices; or

- the starting vertex is equal to the ending vertex, that is, it is impossible to create loops from a single vertex.

The function reports an error, if input vertices do not belong to the graph, that makes it safer than GraphAddEdgeByPtr, but less efficient.

GraphAddEdgeByPtr

Adds edge to graph.

```
int cvGraphAddEdgeByPtr (CvGraph* graph, CvGraphVtx* startVtx, CvGraphVtx*
    endVtx, CvGraphEdge* edge, CvGraphEdge** insertedEdge=0);
```

graph	Graph.
startVtx	Pointer to the starting vertex of the edge.
endVtx	Pointer to the ending vertex of the edge.
edge	Optional input parameter, initialization data for the edge. If not `NULL`, the parameter is copied starting from the 5th 4-byte field.
insertedEdge	Optional output parameter to contain the address of the inserted edge within the edge set.

Discussion

The function `GraphAddEdgeByPtr` adds an edge to the graph given the starting and the ending vertices. The function returns the index of the inserted edge, which is the value of the second 4-byte field of the free cell.

The function reports an error if

- the edge that connects the vertices already exists; in this case graph orientation is taken into account;
- a pointer is `NULL` or indices are invalid;
- some of vertices do not exist, that is, not checked when the pointers are passed to vertices; or

- the starting vertex is equal to the ending vertex, that is, it is impossible to create loops from a single vertex.

The function is more efficient than `GraphAddEdge` but less safe, because it does not check whether the input vertices belong to the graph.

GraphRemoveEdge

Removes edge from graph.

```
void cvGraphRemoveEdge (CvGraph* graph, int startIdx, int endIdx);
```

graph	Graph.
startIdx	Index of the starting vertex of the edge.
endIdx	Index of the ending vertex of the edge.

Discussion

The function `GraphRemoveEdge` removes an edge from the graph that connects given vertices. If the graph is oriented, the vertices must be passed in the appropriate order. The function reports an error if any of the vertices or edges between them do not exist.

The function reports an error, if input vertices do not belong to the graph, that makes it safer than `GraphRemoveEdgeByPtr`, but less efficient.

GraphRemoveEdgeByPtr

Removes edge from graph.

```
void cvGraphRemoveEdgeByPtr (CvGraph* graph, CvGraphVtx* startVtx, CvGraphVtx*
    endVtx);
```

graph	Graph.
startVtx	Pointer to the starting vertex of the edge.

`endVtx` Pointer to the ending vertex of the edge.

Discussion

The function `GraphRemoveEdgeByPtr` removes an edge from the graph that connects given vertices. If the graph is oriented, the vertices must be passed in the appropriate order. The function reports an error if any of the vertices or edges between them do not exist.

The function is more efficient than `GraphRemoveEdge` but less safe, because it does not check whether the input vertices belong to the graph.

FindGraphEdge

Finds edge in graph.

```
CvGraphEdge* cvFindGraphEdge (CvGraph* graph, int startIdx, int endIdx);
```

`graph` Graph.

`startIdx` Index of the starting vertex of the edge.

`endIdx` Index of the ending vertex of the edge.

Discussion

The function `FindGraphEdge` finds the graph edge that connects given vertices. If the graph is oriented, the vertices must be passed in the appropriate order. Function returns `NULL` if any of the vertices or edges between them do not exist.

The function reports an error, if input vertices do not belong to the graph, that makes it safer than `FindGraphEdgeByPtr`, but less efficient.

FindGraphEdgeByPtr

Finds edge in graph.

```
CvGraphEdge* cvFindGraphEdgeByPtr (CvGraph* graph, CvGraphVtx* startVtx,
    CvGraphVtx* endVtx);
```

graph	Graph.
startVtx	Pointer to the starting vertex of the edge.
endVtx	Pointer to the ending vertex of the edge.

Discussion

The function `FindGraphEdgeByPtr` finds the graph edge that connects given vertices. If the graph is oriented, the vertices must be passed in the appropriate order. Function returns `NULL` if any of the vertices or edges between them do not exist.

The function is more efficient than `FindGraphEdge` but less safe, because it does not check whether the input vertices belong to the graph.

GraphVtxDegree

Finds edge in graph.

```
int  cvGraphVtxDegree (CvGraph* graph, int vtxIdx);
```

graph	Graph.
vtx	Pointer to the graph vertex.

Discussion

The function `GraphVtxDegree` counts the edges incident to the graph vertex, both incoming and outcoming, and returns the result. To count the edges, the following code is used:

```
CvGraphEdge* edge = vertex->first; int count = 0;
```

```
while( edge ) {
edge = CV_NEXT_GRAPH_EDGE( edge, vertex );
count++;
}.
```

The macro `CV_NEXT_GRAPH_EDGE( edge, vertex )` returns the next edge after the edge incident to the vertex.

The function reports an error, if input vertices do not belong to the graph, that makes it safer than `GraphVtxDegreeByPtr`, but less efficient.

GraphVtxDegreeByPtr

Finds edge in graph.

```
int cvGraphVtxDegreeByPtr (CvGraph* graph, CvGraphVtx* vtx);
```

graph	Graph.
vtx	Pointer to the graph vertex.

Discussion

The function `GraphVtxDegreeByPtr` counts the edges incident to the graph vertex, both incoming and outcoming, and returns the result. To count the edges, the following code is used:

```
CvGraphEdge* edge = vertex->first; int count = 0;
while( edge ) {
edge = CV_NEXT_GRAPH_EDGE( edge, vertex );
count++;
}.
```

The macro `CV_NEXT_GRAPH_EDGE( edge, vertex )` returns the next edge after the edge incident to the vertex.

The function is more efficient than `GraphVtxDegree` but less safe, because it does not check whether the input vertices belong to the graph.

ClearGraph

Clears graph.

```
void  cvClearGraph (CvGraph* graph);
```

graph Graph.

Discussion

The function `ClearGraph` removes all the vertices and edges from the graph. Similar to the function `ClearSet`, this function takes *O(1)* time.

GetGraphVtx

Finds graph vertex by index.

```
CvGraphVtx* cvGetGraphVtx (CvGraph* graph, int vtxIdx);
```

graph Graph.

vtxIdx Index of the vertex.

Discussion

The function `GetGraphVtx` finds the graph vertex by index and returns the pointer to it or, if not found, to a free cell at this index. Negative indices are supported.

GraphVtxIdx

Returns index of graph vertex.

```
int cvGraphVtxIdx (CvGraph* graph, CvGraphVtx* vtx);
```

graph Graph.

vtx Pointer to the graph vertex.

Discussion

The function `GraphVtxIdx` returns the index of the graph vertex by setting pointers to it.

GraphEdgeIdx

Returns index of graph edge.

```
int cvGraphEdgeIdx (CvGraph* graph, CvGraphEdge* edge);
```

graph Graph.

edge Pointer to the graph edge.

Discussion

The function `GraphEdgeIdx` returns the index of the graph edge by setting pointers to it.

Graphs Data Structures

.

Example 14-11 `CvGraph` Structure Definition

```
#define  CV_GRAPH_FIELDS()   \
    CV_SET_FIELDS()          \
    CvSet* edges;
typedef struct _CvGraph
{
    CV_GRAPH_FIELDS()
}
CvGraph;
```

In OOP terms, the graph structure is derived from the set of vertices and includes a set of edges. Besides, special data types exist for graph vertices and graph edges.

Example 14-12 Definitions of `CvGraphEdge` and `CvGraphVtx` Structures

```
#define CV_GRAPH_EDGE_FIELDS()     \
    struct _CvGraphEdge* next[2]; \
    struct _CvGraphVertex* vtx[2];

#define CV_GRAPH_VERTEX_FIELDS()  \
    struct _CvGraphEdge* first;

typedef struct _CvGraphEdge
{
    CV_GRAPH_EDGE_FIELDS()
}
CvGraphEdge;

typedef struct _CvGraphVertex
{
    CV_GRAPH_VERTEX_FIELDS()
}
CvGraphVtx;
```

Matrix Operations Reference

Example 14-13 `CvMat` Structure Definition

```
typedef struct CvMat {

int type; /* the type of matrix elements */

union
{
    int rows; /* number of rows in the matrix */
    int height; /* synonym for <rows> */
};

union
{
    int cols; /* number of columns */
    int width; /* synonym for <cols> */
};

int step; /* matrix stride */
union
{
    float* fl;
    double* db;
    uchar* ptr;
} data; /* pointer to matrix data */
};
```

Example 14-14 `CvMatArray` Structure Definition

```
typedef struct CvMatArray
{
    int rows;  //number of rows
    int cols;  //number pf cols
    int type;  // type of matrices
    int step;  // not used
    int count; // number of matrices in aary
    union
    {
        float* fl;
        float* db;
    }data; // pointer to matrix array data
}CvMatArray
```

CreateMat

Creates new matrix.

```
CvMat* cvCreateMat (int rows, int cols, int type);
```

rows	Number of rows in the matrix.
cols	Number of columns in the matrix.
type	Type of the new matrix – depth and number of channels; may be specified in form `CV_<bit depth>(S|U)C<number of channels>`, e.g., `CV_8UC1` means an 8-bit unsigned single-channel matrix, `CV_32SC2` means a 32-bit signed matrix with two channels. See `CvMat Structure Definition` and description in the Guide.

Discussion

The function `CreateMat` allocates header for the new matrix and underlying data, and returns a pointer to the created matrix. It is a short form for:

```
CvMat* mat = cvCreateMatrixHeader( rows, cols, type );
cvCreateData( mat );
```

Matrices are stored row by row. All the rows are aligned by 4 bytes.

To get different alignment, use `InitMatHeader` to reinitialize header, created by `CreateMatHeader`, and then call `CreateData` separately.

CreateMatHeader

Creates new matrix header.

```
CvMat* cvCreateMatHeader (int rows, int cols, int type);
```

rows	Number of rows in the matrix.
cols	Number of columns in the matrix.

type Type of the new matrix – depth and number of channels; may be specified in form `CV_<bit depth>(S|U)C<number of channels>`, e.g., `CV_8UC1` means an 8-bit unsigned single-channel matrix, `CV_32SC2` means a 32-bit signed matrix with two channels. See `CvMat Structure Definition` and description in the Guide.

Discussion

The function `CreateMatHeader` allocates new matrix header and returns pointer to it. The matrix data can further be allocated using `CreateData` or set explicitly to user-allocated data via `SetData`. See also description of `CreateMat`.

ReleaseMat

Deallocates matrix.

```
void cvReleaseMat (CvMat** mat);
```

mat Double pointer to the matrix.

Discussion

The function `ReleaseMat` releases memory occupied by the matrix header and underlying data. If `*mat` is null pointer, the function has no effect. The pointer `*mat` is cleared upon the function exit.

It is the short form for:

```
if( *mat )
    cvReleaseData( *mat );
cvReleaseMatHeader( mat );
```

ReleaseMatHeader

Deallocates matrix header.

```
void cvReleaseMatHeader (CvMat** mat);
```

mat	Double pointer to the matrix header.

Discussion

The function `ReleaseMatHeader` releases memory occupied by the matrix header. If `*mat` is null pointer, the function has no effect. The pointer `*mat` is cleared upon the function exit.

Unlike `ReleaseMat`, the function `ReleaseMatHeader` does not deallocate the matrix data, so the user should do it on his/her own.

InitMatHeader

Initializes matrix header.

```
void cvInitMatHeader (CvMat* mat, int rows, int cols, int type, void* data = 0,
    int step = CV_AUTOSTEP);
```

mat	Pointer to the matrix header to be initialized.
rows	Number of rows in the matrix.
cols	Number of columns in the matrix.
type	Type of the new matrix – depth and number of channels; may be specified in form `CV_<bit depth>(S|U)C<number of channels>`, e.g., `CV_8UC1` means an 8-bit unsigned single-channel matrix, `CV_32SC2` means a 32-bit signed matrix with two channels. See `CvMat Structure Definition` and description in the Guide.
data	Optional data pointer assigned to the matrix header.

step Full row width in bytes of the data assigned. By default, the minimal possible step is used, i.e., no gaps assumed between subsequent rows of the matrix.

Discussion

The function `InitMatHeader` initializes already allocated `CvMat` structure. It can be used to process raw data with OpenCV matrix functions.

For example, the following code computes matrix product of two matrices, stored as ordinary arrays.

Example 14-15 Calculating Product of Two Matrices

```
    double  a[] = {  1, 2, 3, 4
                     5, 6, 7, 8,
                     9, 10, 11, 12 };
    double  b[] = {  1, 5, 9,
                     2, 6, 10,
                     3, 7, 11,
                     4, 8, 12 };
    double  c[9];

    CvMat Ma, Mb, Mc;
    cvInitMatHeader( &Ma, 3, 4, CV_64FC1, a );
cvInitMatHeader( &Mb, 4, 3, CV_64FC1, b );
cvInitMatHeader( &Mc, 3, 3, CV_64FC1, c );
cvMatMulAdd( &Ma, &Mb, 0, &Mc ); // c array now contains product of a(3x4) and
b(4x3) matrices
```

CloneMat

Creates matrix copy.

```
CvMat* cvCloneMat (CvMat* mat);
```

mat Input matrix.

Discussion

The function `CloneMat` creates a copy of input matrix and returns the pointer to it. If the input matrix pointer is null, the resultant matrix also has a null data pointer.

SetData

Attaches data to matrix header.

```
void cvSetData (CvArr* mat, void* data, int step);
```

mat	Pointer to the matrix header.
data	Data pointer assigned to the matrix header.
step	Full row width in bytes of the data assigned.

Discussion

The function `SetData` attaches user-allocated data to the matrix header. It is a faster and shorter equivalent for `InitMatHeader` (*mat*, *mat* $\rightarrow$ *rows*, *mat* $\rightarrow$ *cols*, *mat* $\rightarrow$ *type*, *data*, *step*) that is useful in situation when multiple matrices of the same size and type are processed, e.g., video frames and their blocks, feature points, etc.

The *data* pointer can be null and such a function call is useful in preventing outside data from being deallocated occasionally by `ReleaseMat`.

GetMat

Initializes matrix header for arbitrary array.

```
CvMat* cvGetMat (const CvArr* arr, CvMat* mat, int* coi = 0);
```

arr	Input array.
mat	Pointer to `CvMat` structure used a temporary buffer.
coi	Optional output parameter for storing COI.

Discussion

The function `GetMat` creates a matrix header for an input array that can be matrix – `CvMat`, or image – `IplImage`. In the case of matrix the function simply returns the input pointer. In the case of `IplImage` it initializes *`mat`* structure with parameters of the current image ROI and returns pointer to this temporary structure. Because COI is not supported by `CvMat`, it is returned separately.

The function provides an easy way to handle both types of array - `IplImage` and `CvMat` -, using the same code. Reverse transform from `CvMat` to `IplImage` can be done using *`cvGetImage`* function.

Input array must have underlying data allocated or attached, otherwise the function fails.

If the input array is `IplImage` with planar data layout and COI set, the function returns pointer to the selected plane and COI = 0. It enables per-plane processing of multi-channel images with planar data layout using OpenCV functions.

GetAt

Returns array element.

```
CvScalar cvGetAt (const CvArr* arr, int row, int col = 0);
```

`arr`	Array.
`row`	Zero-based index of the row containing the requested element.
`col`	Zero-based index of the column containing the requested element; equal to 0 by default to simplify access to *1D* arrays.

Discussion

The function `GetAt` returns value of the specified array element. In the case of `IplImage`, the whole element is returned regardless of COI settings.

The function is not the fastest way to retrieve array elements. The function *`cvmGet`* is the fastest variant for single-channel floating-point arrays.

If the array has a different format, it is still more efficient to avoid `GetAt` and use `GetAtPtr` instead.

Finally, if the fast sequential access to array elements is needed, `GetRawData` is still a better option than any of the above methods.

SetAt

Sets array element to given value.

```
void cvSetAt (CvArr* arr, CvScalar value, int row, int col = 0);
```

arr	Array.
value	New element value.
row	Zero-based index of the row containing the requested element.
col	Zero-based index of the column containing the requested element; equal to 0 by default to simplify access to *1D* arrays.

Discussion

The function `SetAt` changes value of the specified array element. In the case of `IplImage`, the whole element is changed regardless of COI settings.

The function is not the fastest way to change array elements. The function *cvmSet* is the fastest variant for single-channel floating-point arrays.

If the array has a different format, it is still more efficient to avoid `SetAt` and use `GetAtPtr` instead.

Finally, if the fast sequential access to array elements is needed, `GetRawData` is still a better option than any of the above methods.

GetAtPtr

Returns pointer to array element.

```
uchar* cvGetAtPtr (const CvArr* arr, int row, int col = 0);
```

arr	Array.
row	Zero-based index of the row containing the requested element.
col	Zero-based index of the column containing the requested element; equal to 0 by default to simplify access to *1D* arrays.

Discussion

The function `GetAtPtr` returns pointer of the specified array element. In the case of `IplImage`, pointer to the first channel value of the element is returned regardless of COI settings.

The function is more efficient than `GetAt` and `SetAt`, but for faster sequential access to array elements `GetRawData` is still a better option.

GetSubArr

Returns rectangular sub-array of given array.

```
CvMat* cvGetSubArr (const CvArr* arr, CvMat* subarr, CvRect rect);
```

arr	Input array.
subarr	Pointer to the resulting sub-array header.
rect	Zero-based coordinates of top-left corner of the sub-array and its linear sizes.

Discussion

The function `GetSubArr` returns header, corresponding to a specified rectangle of the input array. In other words, it allows the user to treat a rectangular part of input array as a stand-alone array. ROI is taken into account by the function so the sub-array of ROI is really extracted.

GetRow

Returns array row.

```
CvMat* cvGetRow (const CvArr* arr, CvMat* subarr, int row);
```

arr	Input array.
subarr	Pointer to the resulting sub-array header.
row	Zero-based index of the selected row.

Discussion

The function `GetRow` returns the header, corresponding to a specified row of the input array. The function is a short form for:

```
cvGetSubArr (arr, subarr, cvRect (0, row, arr →cols, 1));
```

GetCol

Returns array column.

```
CvMat* cvGetCol (const CvArr* arr, CvMat* subarr, int col);
```

arr	Input array.
subarr	Pointer to the resulting sub-array header.
col	Zero-based index of the selected column.

Discussion

The function `GetCol` returns the header, corresponding to a specified column of the input array. The function is a short form for:

```
cvGetSubArr (arr, subarr, cvRect (col, 0, 1, arr →rows));
```

GetDiag

Returns array diagonal.

```
CvMat* cvGetDiag (const CvArr* arr, CvMat* subarr, int diag);
```

arr	Input array.
subarr	Pointer to the resulting sub-array header.
diag	Diagonal number; 0 corresponds to the main diagonal, 1 corresponds to the diagonal above the main diagonal, -1 corresponds to the diagonal below the main diagonal, etc.

Discussion

The function `GetDiag` returns the header, corresponding to a specified diagonal of the input array.

GetRawData

Returns low level information on array.

```
void  cvRawData (const CvArr* arr, uchar** data, int* step, CvSize* roiSize );
```

arr	Input array.
data	Pointer to the retrieved array data pointer.
step	Pointer to the retrieved array step.
roiSize	Pointer to the retrieved array size, or selected ROI size.

Discussion

The function `GetRawData` returns array data pointer, step, or full row width in bytes. and linear size. All the output parameters are optional, that is, the correspondent pointers may be null. The function provides the fastest sequential access to array elements if the format of elements is known.

For example, the following code finds absolute value of every element of a single-channel floating-point array:

Example 14-16 Using `GetRawData` for Image Pixels Access.

```
float* data;
int step;
CvSize size;
int x, y;

cvGetRawData( Array, (uchar**)&data, &step, &size );
step /= sizeof(data[0]);

for( y = 0; y < size.height; y++, data += step )
    for( x = 0; x < size.width; x++ )
        data[x] = (float)fabs(data[x]);
```

If array is `IplImage` with ROI set, parameters of ROI are returned.

GetSize

Returns width and height of array.

```
CvSize cvGetSize (const CvArr* arr);
```

arr	Array.

Discussion

The function `GetSize` returns width, or the number of columns, and height, or the number of rows, of the array.

If array is `IplImage` with ROI set, size ROI is returned.

CreateData

Allocates memory for array data.

```
void cvCreateData (CvArr* mat);
```

`mat` Pointer to the array for which memory must be allocated.

Discussion

The function `CreateData` allocates memory for the array data.

AllocArray

Allocates memory for matrix array data.

```
void cvmAllocArray (CvMatArray* matArr);
```

`matArr` Pointer to the matrix array for which memory must be allocated.

Discussion

The function `AllocArray` allocates memory for the matrix array data.

Structure `CvMatArray` is obsolete. Use multi-channel matrices `CvMat` and functions `MatMulAddS` and `PerspectiveTransform` to operate on a group of small vectors.

ReleaseData

Frees memory allocated for array data.

```
void cvReleaseData (CvArr* mat);
```

`mat` Pointer to the array.

Discussion

The function `ReleaseData` releases the memory allocated by the function `CreateData`.

FreeArray

Frees memory allocated for matrix array data.

```
void cvmFreeArray (CvMatArr* matArr);
```

matArr	Pointer to the matrix array.

Discussion

The function `FreeArray` releases the memory allocated by the function `AllocArray`.

Structure `CvMatArray` is obsolete. Use multi-channel matrices `CvMat` and functions `MatMulAddS` and `PerspectiveTransform` to operate on a group of small vectors.

Copy

Copies one array to another.

```
void cvCopy (const CvArr* A, CvArr* B, const CvArr* mask=0);
```

A	Pointer to the source array.
B	Pointer to the destination array.
mask	Operation mask, 8-bit single channel array; specifies elements of destination array to be changed.

Discussion

The function `Copy` copies selected pixels from input array to output array. If any of the passed arrays is of `IplImage` type, then its ROI and COI fields are used. Both arrays should be of the same type and their sizes, or their ROIs sizes, must be the same.

$B_{ij} = A_{ij}$, if $mask_{ij} \neq 0$.

All array parameters should have the same size or selected ROI sizes and all of them, except *mask*, must be of the same type.

Set

Sets every element of array to given value.

```
void  cvSet (CvArr* A, CvScalar S, const CvArr* mask=0 );
```

A	Pointer to the destination array.
S	Fill value.
mask	Operation mask, 8-bit single channel array; specifies elements of destination array to be changed.

Discussion

The function `Set` copies scalar *S* to every selected element of the destination array. If array *A* is of `IplImage` type, then is ROI used, but COI should not be set.

$A_{ij} = S$, if $mask_{ij} \neq 0$.

Add

Computes sum of two arrays.

```
void cvAdd (const CvArr* A, const CvArr* B, CvArr* C, const CvArr* mask=0);
```

A	Pointer to the first source array.

B	Pointer to the second source array.
C	Pointer to the destination array.
mask	Operation mask, 8-bit single channel array; specifies elements of destination array to be changed.

Discussion

The function `Add` adds array *B* to array *A* and stores the result in *C*.

$C_{ij} = A_{ij} + B_{ij}$, if $mask_{ij} \neq 0$.

All array parameters should have the same size or selected ROI sizes and all of them, except *mask*, must be of the same type.

AddS

Computes sum of array and scalar.

```
void cvAddS (const CvArr* A, CvScalar S, CvArr* C, const CvArr* mask=0);
```

A	Pointer to the source array.
S	Added scalar.
C	Pointer to the destination array.
mask	Operation mask, 8-bit single channel array; specifies elements of destination array to be changed.

Discussion

The function `AddS` adds scalar *S* to every element in the source array *A* and stores the result in *C*.

$C_{ij} = A_{ij} + S$, if $mask_{ij} \neq 0$.

All array parameters should have the same size or selected ROI sizes and all of them, except *mask*, must be of the same type.

Sub

Computes difference of two arrays.

```
void cvSub (const CvArr* A, const CvArr* B, CvArr* C, const CvArr* mask=0);
```

A	Pointer to the first source array.
B	Pointer to the second source array.
C	Pointer to the destination array.
mask	Operation mask, 8-bit single channel array; specifies elements of destination array to be changed.

Discussion

The function `Sub` subtracts array *B* from array *A* and stores the result in *C*.

$C_{ij} = A_{ij} - B_{ij}$, if $mask_{ij} \neq 0$.

All array parameters should have the same size or selected ROI sizes and all of them, except *mask*, must be of the same type.

SubS

Computes difference of array and scalar.

```
void cvSubS (const CvArr* A, CvScalar S, CvArr* C, const CvArr* mask=0);
```

A	Pointer to the first source array.
S	Subtracted scalar.
C	Pointer to the destination array.
mask	Operation mask, 8-bit single channel array; specifies elements of destination array to be changed.

Discussion

The function `SubS` subtracts scalar *S* from every element in the source array *A* and stores the result in *C*.

$C_{ij} = A_{ij} - S$, if $mask_{ij} \neq 0$.

All array parameters should be of the same type and size or have the same ROI size.

SubRS

Computes difference of scalar and array.

```
void cvSubRS (const CvArr* A, CvScalar S, CvArr* C, const CvArr* mask=0);
```

A	Pointer to the first source array.
S	Scalar to subtract from.
C	Pointer to the destination array.
mask	Operation mask, 8-bit single channel array; specifies elements of destination array to be changed.

Discussion

The function `SubRS` subtracts every element of source array *A* from scalar *S* and stores the result in *C*.

$C_{ij} = S - A_{ij}$, if $mask_{ij} \neq 0$.

All array parameters should have the same size or selected ROI sizes and all of them, except *mask*, must be of the same type.

Mul

Calculates per-element product of two arrays.

```
void  cvMul (const CvArr* A, const CvArr* B, CvArr* C);
```

A	Pointer to the first source array.
B	Pointer to the second source array.
C	Pointer to the destination array.

Discussion

The function `Mul` calculates per-element product of arrays *A* and *B* and stores the result in *C*.

$C_{ij} = A_{ij} \cdot B_{ij}$.

All array parameters should be of the same size or selected ROI sizes and of the same type.

And

Calculates logical conjunction of two arrays.

```
void cvAnd (const CvArr* A, const CvArr* B, CvArr* C, const CvArr* mask=0);
```

A	Pointer to the first source array.
B	Pointer to the second source array.
C	Pointer to the destination array.
mask	Operation mask, 8-bit single channel array; specifies elements of destination array to be changed.

Discussion

The function `And` calculates per-element logical conjunction of arrays *A* and *B* and stores the result in *C*.

$C_{ij} = A_{ij}$ and B_{ij} , if $mask_{ij} \neq 0$.

Table 14-2 shows the way to compute the result from input bits.

Table 14-2 Result Computation for `cvAnd`

k-th bit of A_{ij}	*k*-th bit of B_{ij}	*k*-th bit of C_{ij}
0	0	0
0	1	0
1	0	0
1	1	1

In the case of floating-point images their bit representations are used for the operation.

All array parameters should have the same size or selected ROI sizes and all of them, except *mask*, must be of the same type.

AndS

Calculates logical conjunction of array and scalar.

```
void  cvAndS (const CvArr* A, CvScalar S, CvArr* C, const CvArr* mask = 0);
```

A	Pointer to the source array.
S	Scalar to use in the operation.
C	Pointer to the destination array.
mask	Operation mask, 8-bit single channel array; specifies elements of destination array to be changed.

Discussion

The function AndS calculates per-element logical conjunction of array *A* and scalar *S* and stores the result in *C*.

Before the operation is implemented the scalar is converted to the same type as arrays.

$C_{ij} = A_{ij}$ and S , if $mask_{ij} \neq 0$.

Table 14-3 shows the way to compute the result from input bits.

Table 14-3 Result Computation for cvAndS

k-th bit of A_{ij}	k-th bit of S	k-th bit of C_{ij}
0	0	0
0	1	0
1	0	0
1	1	1

In the case of floating-point images their bit representations are used for the operation.

All array parameters should have the same size or selected ROI sizes and all of them, except *mask*, must be of the same type.

Or

Calculates logical disjunction of two arrays.

```
void  cvOr (const CvArr* A, const CvArr* B, CvArr* C, const CvArr* mask = 0);
```

A	Pointer to the first source array.
B	Pointer to the second source array.
C	Pointer to the destination array.
mask	Operation mask, 8-bit single channel array; specifies elements of destination array to be changed.

Discussion

The function `Or` calculates per-element logical disjunction of arrays *A* and *B* and stores the result in *C*.

$C_{ij} = A_{ij}$ or B_{ij}, if $mask_{ij} \neq 0$.

Table 14-4 shows the way to compute the result from input bits.

Table 14-4 Result Computation for `Or`

k-th bit of A_{ij}	*k*-th bit of B_{ij}	*k*-th bit of C_{ij}
0	0	0
0	1	1
1	0	1
1	1	1

In the case of floating-point images their bit representations are used for the operation.

All array parameters should have the same size or selected ROI sizes and all of them, except *mask*, must be of the same type.

OrS

Calculates logical disjunction of array and scalar.

```
void  cvAndS (const CvArr* A, CvScalar S, CvArr* C, const CvArr* mask = 0);
```

A Pointer to the source array.

S Scalar to use in the operation.

C Pointer to the destination array.

mask Operation mask, 8-bit single channel array; specifies elements of destination array to be changed.

Discussion

The function `OrS` calculates per-element logical disjunction of array A and scalar S and stores the result in C.

$C_{ij} = A_{ij}$ or S, if $mask_{ij} \neq 0$.

Table 14-5 shows the way to compute the result from input bits.

Table 14-5 Result Computation for `OrS`

k-th bit of A_{ij}	k-th bit of S	k-th bit of C_{ij}
0	0	0
0	1	1
1	0	1
1	1	1

In the case of floating-point images their bit representations are used for the operation.

All array parameters should have the same size or selected ROI sizes and all of them, except *mask*, must be of the same type.

Xor

Calculates logical "exclusive or" operation on two arrays.

```
void  cvXor (const CvArr* A, const CvArr* B, CvArr* C, const CvArr* mask = 0);
```

A Pointer to the first source array.

B Pointer to the second source array.

C Pointer to the destination array.

mask Operation mask, 8-bit single channel array; specifies elements of destination array to be changed.

Discussion

The function `Xor` calculates per-element logical “exclusive or” operation on arrays *A* and *B* and stores the result in *C*.

$C_{ij} = A_{ij} \text{ xor } B_{ij}$, if $mask_{ij} \neq 0$.

Table 14-6 shows the way to compute the result from input bits.

Table 14-6 Result Computation for `Xor`

k-th bit of A_{ij}	k-th bit of B_{ij}	k-th bit of C_{ij}
0	0	0
0	1	1
1	0	1
1	1	0

In the case of floating-point images their bit representations are used for the operation.

All array parameters should have the same size or selected ROI sizes and all of them, except *mask*, must be of the same type.

XorS

Calculates logical “exclusive or” operation on array and scalar.

```
void  cvAndS (const CvArr* A, CvScalar S, CvArr* C, const CvArr* mask = 0);
```

A Pointer to the source array.

S Scalar to use in the operation.

C Pointer to the destination array.

mask Operation mask, 8-bit single channel array; specifies elements of destination array to be changed.

Discussion

The function `XorS` calculates per-element logical “exclusive or” operation array *A* and scalar *S* and stores the result in *C*.

$C_{ij} = A_{ij} \text{ xor } S$, if $mask_{ij} \neq 0$.

Table 14-7 shows the way to compute the result from input bits.

Table 14-7 Result Computation for `XorS`

k-th bit of A_{ij}	*k*-th bit of *S*	*k*-th bit of C_{ij}
0	0	0
0	1	1
1	0	1
1	1	0

In the case of floating-point images their bit representations are used for the operation.

All array parameters should have the same size or selected ROI sizes and all of them, except *mask*, must be of the same type.

DotProduct

Calculates dot product of two arrays in Euclidian metrics.

```
double cvDotProduct (const CvArr* A, cjnst CvArr* B);
```

A Pointer to the first source array.

B Pointer to the second source array.

Discussion

The function `DotProduct` calculates and returns the Euclidean dot product of two arrays.

$$DP = A \cdot B = \sum_{i,j} A_{ij} B_{ij} .$$

CrossProduct

Calculates cross product of two 3D vectors.

```
void cvCrossProduct (const CvArr* A, const CvArr* B, CvArr* C);
```

A	Pointer to the first source vector.
B	Pointer to the second source vector.
C	Pointer to the destination vector.

Discussion

The function `CrossProduct` calculates the cross product of two *3D* vectors:

$$C = A \times B, \quad (C_1 = A_2B_3 - A_3B_2, C_2 = A_3B_1 - A_1B_3, C_3 = A_1B_2 - A_2B_1) .$$

ScaleAdd

Calculates sum of scaled array and another array.

```
void  cvScaleAdd (const CvArr* A, CvScalar S, const CvArr* B, CvArr* C);
```

A	Pointer to the first source array.
S	Scale factor for the first array.
B	Pointer to the second source array.
C	Pointer to the destination array

Discussion

The function `ScaleAdd` calculates sum of scaled array *A* and array *B* and stores the result in *C*.

$$C_{ij} = A_{ij} \cdot S + B_{ij} .$$

All array parameters should be of the same size or selected ROI sizes and of the same type.

The function name `MulAddS` may be used as a synonym of `ScaleAdd`.

MatMulAdd

Calculates shifted matrix product.

```
void  cvMatMulAdd (const CvArr* A, const CvArr* B, const CvArr* C, CvArr* D);
```

A	Pointer to the first source array.
B	Pointer to the second source array.
C	Pointer to the third source array (shift).
D	Pointer to the destination array.

Discussion

The function `MatMulAdd` calculates matrix product of arrays *A* and *B*, adds array *C* to the product and stores the final result in *D*.

$$D = A \cdot B + C,\ D_{ij} = \sum_k A_{ik} \cdot B_{kj} + C_{ij} .$$

All parameters should be of the same type – single-precision or double-precision floating point real or complex numbers (`32fC1`, `64fC1`, `32fC2` or `64fC2`). Dimensions of *A*, *B*, *C* and, *D* must co-agree: if matrix *A* has *m* rows and *k* columns and matrix *B* has *k* rows and *n* columns, then matrix *C*, if present, must have *m* rows and *n* columns and matrix *D* must have *m* rows and *n* columns too.

MatMulAddS

Performs matrix transform on every element of array.

```
void  cvMatMulAddS (const CvArr* A, CvArr* C, const CvArr* M, const CvArr* V =
   0);
```

`A`	Pointer to the first source array.
`C`	Pointer to the destination array.
`M`	Transformation matrix.
`V`	Optional shift.

Discussion

The function `MatMulAddS` performs matrix transform on every element of array `A` and stores the result in `C`.

The function considers every element of `N`-channel array `A` as a vector of `N` components.

$$C_{ij} = M \cdot A_{ij} + V,\ C_{ij}(cn) = \sum_k M_{cn,k} \cdot A_{ij}(k) + V_{cn}$$, if `M` is `(N x N)`

or

$$[C_{ij}\ 1] = [M; 0 \ldots 0\ 1] \cdot [A_{ij}\ 1],\ C_{ij}(cn) = \sum M_{cn,k} \cdot A_{ij}(k) + M_{cn,N-1}$$, if `M` is `(N x N+1)`.

In the second variant the shift vector is stored in the right column of the matrix `M`.

Both source and destination arrays should be of the same size or selected ROI size and of the same type. `M` and `V` should be real single-precision or double-precision matrices.

The function can be used for geometrical transforms of point sets and linear color transformations.

MulTransposed

Calculates product of array and transposed array.

```
void cvMulTransposed (const CvArr* A, CvArr* C, int order);
```

A	Pointer to the source array.
C	Pointer to the destination array.
order	Order of multipliers.

Discussion

The function `MulTransposed` calculates the product of *A* and its transposition.

The function evaluates $B = A^T A$ if *order* is non-zero, $B = AA^T$ otherwise.

Invert

Inverts array.

```
void cvInvert (const CvArr* A, CvArr* B);
```

A	Pointer to the source array.
B	Pointer to the destination array.

Discussion

The function `Invert` inverts *A* and stores the result in *B*.

$B = A^{-1}, AB = BA = I$.

The function name `Inv` can be used as a synonym for `Invert`.

Trace

Returns trace of array.

```
CvScalar cvTrace (const CvArr* A);
```

`A` Pointer to the source array.

Discussion

The function `Trace` returns the sum of diagonal elements of the array `A`.

$$trA = \sum_i A_{ii} .$$

Det

Returns determinant of array.

```
CvScalar cvDet (const CvArr* A);
```

`A` Pointer to the source array.

Discussion

The function `Det` returns the determinant of the array `A`.

Mahalonobis

Calculates Mahalonobis distance between vectors.

```
double cvMahalonobis  (const CvArr* A, const CvArr* B, CvArr* T);
```

`A` Pointer to the first source vector.

B	Pointer to the second source vector.
T	Pointer to the inverse covariance array.

Discussion

The function `Mahalonobis` calculates the weighted distance between two vectors and returns it:

$$dist = \sqrt{\sum_{i,j} T_{ij}(A_i - B_i)(A_j - B_j)}.$$

Transpose

Transposes array.

```
void cvTranspose (const CvArr* A, CvArr* B);
```

A	Pointer to the source array.
B	Pointer to the destination array.

Discussion

The function `Transpose` transposes *A* and stores result in *B*.

$$B = A^T, B_{ij} = A_{ji}.$$

The function name `T` can be used as a synonym of `Transpose`.

Flip

Reflects array around horizontal or vertical axis or both.

```
void cvFlip (const CvArr* A, CvArr* B, int flipMode);
```

A	Pointer to the source array.
B	Pointer to the destination array.
flipMode	Flip mode; specifies an axis to reflect the array around.

Discussion

The function `Flip` flips array *A* horizontally, vertically or in both directions and stores the result in *C*. Both arrays must be of the same size or selected ROI size and of the same type.

Let array *A* have *M* rows and *N* columns, then array *C* is calculated as follows:

$C_{M-i-1,j} = A_{ij}$, if *flipMode* = 0,

$C_{i,N-j-1} = A_{ij}$, if *flipMode* > 0,

$C_{M-i-1,N-j-1} = A_{ij}$, if *flipMode* < 0.

Reshape

Changes dimensions and/or number of channels in matrix.

```
CvMat* cvReshape (const CvArr* A, CvMat* header, int newNumChannels, int
    newRows = 0);
```

A	Source matrix.
header	Destination matrix header; the data must not be allocated because data pointer is taken from the source matrix and the previous pointer is lost.
newNumChannels	New number of channels.
newRows	New number of rows; the default value is 0 and it means that the number of rows is not changed.

Discussion

The function `Reshape` initializes destination header with the parameters of the source matrix but with a different number of channels and/or a different number of rows. The new number of columns is calculated from these new parameters. The following examples illustrate use of the function:

1. Suppose, *A* is a 3x3 floating-point matrix and is treated as a *1D* vector of 9 elements. It is done via:

```
CvMat vec;
cvReshape( A, &vec, 1, 1 ); // leave a single-channel and change number
of rows to 1.
```

2. Suppose, *A* is a *YUV* video frame with interleaved channels and decimated *U* and *V* planes: `Y0 U0 Y1 V0 Y2 U1 Y3 V1 …,` treated as a 4-channel image where each element (quadruple) represents two pixels in the original image. The respective code is as follows:

```
CvMat c1img;
cvReshape( A, &c1img, 4, 0 ); // make the image 4-channel and leave the
                 number of rows unchanged.
```

After that call the function `CvtPixToPlane` may be used to extract *U*, *V* and two halves of *Y* planes.

The number of rows can be changed only if the matrix is continuous, i.e., no gaps exist between subsequent rows. Also, if the number of channels changes, a new number of columns should be a multiple of the new number of channels.

SetZero

Sets array to zero.

```
void cvSetZero (CvArr* A);
```

A Pointer to the array to be set to zero.

Discussion

The function `SetZero` sets the array to zero.

$A = 0, A_{ij} = 0$.

The function name `Zero` can be used as a synonym for `SetZero`.

SetIdentity

Sets array to identity.

```
void cvSetIdentity (CvArr* A);
```

A	Pointer to the array to be set to identity.

Discussion

The function `SetIdentity` sets the array to identity.

$$A = I, A_{ij} = \delta_{ij} = \begin{cases} 1, & i = j \\ 0, & i \neq j \end{cases} .$$

SVD

Performs singular value decomposition of matrix.

```
void cvSVD (CvArr* A, CvArr* W, CvArr* U = 0, CvArr* V = 0, int flags = 0);
```

A	Source matrix.
W	Resulting singular value matrix or vector.
U	Optional left orthogonal matrix.
V	Optional right orthogonal matrix.
flags	Operation flags; can be combination of the following:

- `CV_SVD_MODIFY_A` enables modification of matrix *A* during the operation. It makes the processing faster.

- `CV_SVD_U_T` means that the matrix *U* is transposed on exit.
- `CV_SVD_V_T` means that the matrix *V* is transposed on exit.

Discussion

The function `SVD` decomposes matrix *A* into a product of a diagonal matrix and two orthogonal matrices:

$A = U^T W V$, where

A	is an arbitrary *M* x *N* matrix,
U	is an orthogonal *M x M* matrix,
V	is an orthogonal *N x N* matrix,
W	is a diagonal *M x N* matrix with non-negative diagonal elements or just a vector of *min(M,N)* elements storing diagonal elements.

The function `SVD` is numerically robust and its typical applications include:

- accurate eigenvalue problem solution when matrix *A* is symmetric and positively defined, e.g., it is a covariation matrix
- accurate solution of poor-conditioned linear systems
- least-squares solution of overdetermined linear systems
- accurate calculation of different matrix characteristics such as rank, condition number, determinant, `L2`-norm. This does not require calculation of *U* and *V* matrices.

See also `PseudoInv` function.

PseudoInv

Finds pseudo inverse of matrix.

```
void cvPseudoInv (CvArr* A, CvArr* B, int flags = 0);
```

A	Source matrix.
W	Resultant pseudo inverse matrix.
flags	Operation flags - 0 or `CV_SVD_MODIFY_A`, which means that the function can modify matrix *A* during processing.

Discussion

The function `PseudoInv` finds pseudo inverse of matrix *A* using the function `SVD`:

$B = V^T \tilde{W} U$, where U, V and W from the formula below are components of singular value decomposition of matrix A, and $\tilde{W}$ is calculated as follows:

$$\|\tilde{W}\|_{i,j} = \begin{cases} \dfrac{1}{\|W\|_{i,j}}, & \|W\|_{i,j} \neq 0 \\ 0, & \text{else} \end{cases}$$

EigenVV

Computes eigenvalues and eigenvectors of symmetric array.

```
void cvEigenVV (CvArr* A, CvArr* evects, CvArr* evals, Double eps);
```

A	Pointer to the source array.
evects	Pointer to the array where eigenvectors must be stored.
evals	Pointer to the array where eigenvalues must be stored.
eps	Accuracy of diagonalization.

Discussion

The function `EigenVV` computes the eigenvalues and eigenvectors of the array *A* and stores them in the parameters *evals* and *evects* correspondingly. Jacobi method is used. Eigenvectors are stored in successive rows of array eigenvectors. The resultant eigenvalues are in descending order.

NOTE. *The function EigenVV destroys the source array A. Therefore, if the source array is needed after eigenvalues have been calculated, clone it before running the function EigenVV.*

PerspectiveTransform

Implements general transform of 3D vector array.

```
void cvPerspectiveTransform (const CvArr* A, CvArr* B, const CvArr* M);
```

A	Pointer to the source three-channel floating-point array, 32f or 64f.
B	Pointer to the destination three-channel floating-point array, 32f or 64f.
M	4x4 transformation array.

Discussion

The function `PerspectiveTransform` maps every element of array *A* - *3D* vector $(x, y, z)^T$ to $(x'/w, y'/w, z'/w)^T$, where

$$(x', y', z', w')^T = M \times (x, y, z, 1)^T \text{ and } w = \begin{cases} w', & w' \neq 0 \\ 1, & w' = 0 \end{cases}.$$

Drawing Primitives Reference

Line

Draws simple or thick line segment.

```
void cvLine (IplImage* img, CvPoint pt1, CvPoint pt2, int color, int
    thickness=1);
```

img	Image.
pt1	First point of the line segment.
pt2	Second point of the line segment.
color	Line color (RGB) or brightness (grayscale image).
thickness	Line thickness.

Discussion

The function `Line` draws the line segment between *pt1* and *pt2* points in the image. The line is clipped by the image or ROI rectangle. The Bresenham algorithm is used for simple line segments. Thick lines are drawn with rounding endings. To specify the line color, the user may use the macro `CV_RGB` (*r*, *g*, *b*) that makes a 32-bit color value from the color components.

LineAA

Draws antialiased line segment.

```
void cvLineAA (IplImage* img, CvPoint pt1, CvPoint pt2, int color, int
    scale=0);
```

img	Image.
pt1	First point of the line segment.

pt2	Second point of the line segment.
color	Line color (RGB) or brightness (grayscale image).
scale	Number of fractional bits in the end point coordinates.

Discussion

The function `LineAA` draws the line segment between *pt1* and *pt2* points in the image. The line is clipped by the image or ROI rectangle. Drawing algorithm includes some sort of Gaussian filtering to get a smooth picture. To specify the line color, the user may use the macro `CV_RGB (r, g, b)` that makes a 32-bit color value from the color components.

Rectangle

Draws simple, thick or filled rectangle.

```
void cvRectangle (IplImage* img, CvPoint pt1, CvPoint pt2, int color, int
    thickness);
```

img	Image.
pt1	One of the rectangle vertices.
pt2	Opposite rectangle vertex.
color	Line color (RGB) or brightness (grayscale image).
thickness	Thickness of lines that make up the rectangle.

Discussion

The function `Rectangle` draws a rectangle with two opposite corners *pt1* and *pt2*. If the parameter *thickness* is positive or zero, the outline of the rectangle is drawn with that thickness, otherwise a filled rectangle is drawn.

Circle

Draws simple, thick or filled circle.

```
void cvCircle (IplImage* img, CvPoint center, int radius, int color,
    int thickness=1);
```

img	Image where the line is drawn.
center	Center of the circle.
radius	Radius of the circle.
color	Circle color (RGB) or brightness (grayscale image).
thickness	Thickness of the circle outline if positive, otherwise indicates that a filled circle is to be drawn.

Discussion

The function `Circle` draws a simple or filled circle with given center and radius. The circle is clipped by ROI rectangle. The Bresenham algorithm is used both for simple and filled circles. To specify the circle color, the user may use the macro `CV_RGB` (*r*, *g*, *b*) that makes a 32-bit color value from the color components.

Ellipse

Draws simple or thick elliptic arc or fills ellipse sector.

```
void cvEllipse (IplImage* img, CvPoint center, CvSize axes, double angle,
    double startAngle, double endAngle, int color, int thickness=1);
```

img	Image.
center	Center of the ellipse.
axes	Length of the ellipse axes.
angle	Rotation angle.

startAngle	Starting angle of the elliptic arc.
endAngle	Ending angle of the elliptic arc.
color	Ellipse color (RGB) or brightness (grayscale image).
thickness	Thickness of the ellipse arc.

Discussion

The function `Ellipse` draws a simple or thick elliptic arc or fills an ellipse sector. The arc is clipped by ROI rectangle. The generalized Bresenham algorithm for conic section is used for simple elliptic arcs here, and piecewise-linear approximation is used for antialiased arcs and thick arcs. All the angles are given in degrees. Figure 14-3 shows the meaning of the parameters.

Figure 14-3 Parameters of Elliptic Arc

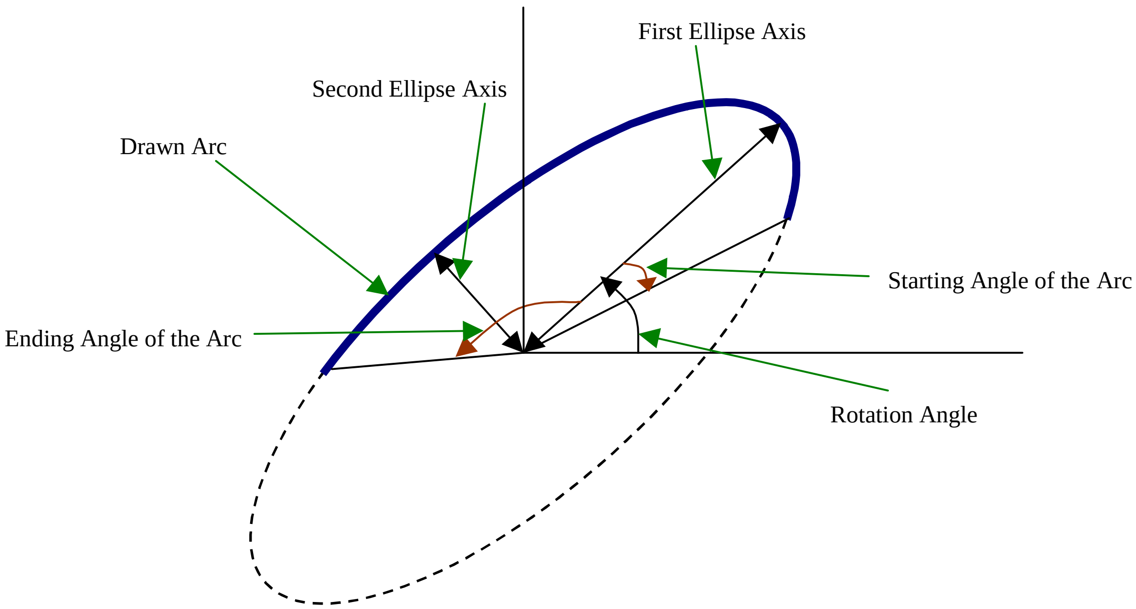

EllipseAA

Draws antialiased elliptic arc.

```
void cvEllipseAA (IplImage* img, CvPoint center, CvSize axes, double angle,
    double startAngle, double endAngle, int color, int scale=0);
```

img	Image.
center	Center of the ellipse.
axes	Length of the ellipse axes.
angle	Rotation angle.
startAngle	Starting angle of the elliptic arc.
endAngle	Ending angle of the elliptic arc.
color	Ellipse color (RGB) or brightness (grayscale image).
scale	Specifies the number of fractional bits in the center coordinates and axes sizes.

Discussion

The function `EllipseAA` draws an antialiased elliptic arc. The arc is clipped by ROI rectangle. The generalized Bresenham algorithm for conic section is used for simple elliptic arcs here, and piecewise-linear approximation is used for antialiased arcs and thick arcs. All the angles are in degrees. Figure 14-3 shows the meaning of the parameters.

FillPoly

Fills polygons interior.

```
void cvFillPoly (IplImage* img, CvPoint** pts, int* npts, int contours,
    int color);
```

img	Image.

pts	Array of pointers to polygons.
npts	Array of polygon vertex counters.
contours	Number of contours that bind the filled region.
color	Polygon color (RGB) or brightness (grayscale image).

Discussion

The function `FillPoly` fills an area bounded by several polygonal contours. The function fills complex areas, for example, areas with holes, contour self-intersection, etc.

FillConvexPoly

Fills convex polygon.

```
void cvFillConvexPoly (IplImage* img, CvPoint* pts, int npts, int color);
```

img	Image.
pts	Array of pointers to a single polygon.
npts	Polygon vertex counter.
color	Polygon color (RGB) or brightness (grayscale image).

Discussion

The function `FillConvexPoly` fills convex polygon interior. This function is much faster than the function `FillPoly` and fills not only the convex polygon but any monotonic polygon, that is, a polygon whose contour intersects every horizontal line (scan line) twice at the most.

PolyLine

Draws simple or thick polygons.

```
void cvPolyLine (IplImage* img, CvPoint** pts, int* npts, int contours, int
    isClosed, int color, int thickness=1);
```

img	Image.
pts	Array of pointers to polylines.
npts	Array of polyline vertex counters.
contours	Number of polyline contours.
isClosed	Indicates whether the polylines must be drawn closed. If closed, the function draws the line from the last vertex of every contour to the first vertex.
color	Polygon color (RGB) or brightness (grayscale image).
thickness	Thickness of the polyline edges.

Discussion

The function `PolyLine` draws a set of simple or thick polylines.

PolyLineAA

Draws antialiased polygons.

```
void cvPolyLineAA (IplImage* img, CvPoint** pts, int* npts, int contours, int
    isClosed, int color, int scale=0);
```

img	Image.
pts	Array of pointers to polylines.
npts	Array of polyline vertex counters.
contours	Number of polyline contours.

isClosed	Indicates whether the polylines must be drawn closed. If closed, the function draws the line from the last vertex of every contour to the first vertex.
color	Polygon color (RGB) or brightness (grayscale image).
scale	Specifies number of fractional bits in the coordinates of polyline vertices.

Discussion

The function `PolyLineAA` draws a set of antialiased polylines.

InitFont

Initializes font structure.

```
void cvInitFont (CvFont* font, CvFontFace fontFace, float hscale, float
    vscale, float italicScale, int thickness);
```

font	Pointer to the resultant font structure.
fontFace	Font name identifier. Only the font `CV_FONT_VECTOR0` is currently supported.
hscale	Horizontal scale. If equal to `1.0f`, the characters have the original width depending on the font type. If equal to `0.5f`, the characters are of half the original width.
vscale	Vertical scale. If equal to `1.0f`, the characters have the original height depending on the font type. If equal to `0.5f`, the characters are of half the original height.
italicScale	Approximate tangent of the character slope relative to the vertical line. Zero value means a non-italic font, `1.0f` means ~`45×` slope, etc.
thickness	Thickness of lines composing letters outlines. The function `cvLine` is used for drawing letters.

Discussion

The function `InitFont` initializes the font structure that can be passed further into text drawing functions. Although only one font is supported, it is possible to get different font flavors by varying the scale parameters, slope, and thickness.

PutText

Draws text string.

```
void cvPutText (IplImage* img, const char* text, CvPoint org, CvFont* font, int
    color);
```

img	Input image.
text	String to print.
org	Coordinates of the bottom-left corner of the first letter.
font	Pointer to the font structure.
color	Text color (RGB) or brightness (grayscale image).

Discussion

The function `PutText` renders the text in the image with the specified font and color. The printed text is clipped by ROI rectangle. Symbols that do not belong to the specified font are replaced with the rectangle symbol.

GetTextSize

Retrieves width and height of text string.

```
void cvGetTextSize (CvFont* font, const char* textString, CvSize* textSize,
    int* ymin);
```

font	Pointer to the font structure.

textString	Input string.
textSize	Resultant size of the text string. Height of the text does not include the height of character parts that are below the baseline.
ymin	Lowest *y* coordinate of the text relative to the baseline. Negative, if the text includes such characters as *g*, *j*, *p*, *q*, *y*, etc., and zero otherwise.

Discussion

The function `GetTextSize` calculates the binding rectangle for the given text string when a specified font is used.

Utility Reference

AbsDiff

Calculates absolute difference between two images.

```
void cvAbsDiff (IplImage* srcA, IplImage* srcB, IplImage* dst);
```

srcA	First compared image.
srcB	Second compared image.
dst	Destination image.

Discussion

The function `AbsDiff` calculates absolute difference between two images.

$dst(x, y) = abs(srcA(x, y) - srcB(x, y))$.

AbsDiffS

Calculates absolute difference between image and scalar.

```
void cvAbsDiffS (IplImage* srcA, IplImage* dst, double value);
```

`srcA`	Compared image.
`dst`	Destination image.
`value`	Value to compare.

Discussion

The function `AbsDiffS` calculates absolute difference between an image and a scalar.

$dst(x,y) = abs(srcA(x,y) - value)$.

MatchTemplate

Fills characteristic image for given image and template.

```
void cvMatchTemplate (IplImage* img, IplImage* templ, IplImage* result,
    CvTemplMatchMethod method);
```

`img`	Image where the search is running.
`templ`	Searched template; must be not greater than the source image. The parameters `img` and `templ` must be single-channel images and have the same depth (`IPL_DEPTH_8U`, `IPL_DEPTH_8S`, or `IPL_DEPTH_32F`).
`result`	Output characteristic image. It has to be a single-channel image with depth equal to `IPL_DEPTH_32F`. If the parameter `img` has the size of $W \times H$ and the template has the size $w \times h$, the resulting image must have the size or selected ROI $W - w + 1 \times H - h + 1$.

`method` Specifies the way the template must be compared with image regions.

Discussion

The function `MatchTemplate` implements a set of methods for finding the image regions that are similar to the given template.

Given a source image with $W \times H$ pixels and a template with $w \times h$ pixels, the resulting image has $W - w + 1 \times H - h + 1$ pixels, and the pixel value in each location (x,y) characterizes the similarity between the template and the image rectangle with the top-left corner at (x,y) and the right-bottom corner at (`x + w - 1`, `y + h - 1`). Similarity can be calculated in several ways:

Squared difference (`method == CV_TM_SQDIFF`)

$$S(x,y) = \sum_{y'=0}^{h-1}\sum_{x'=0}^{w-1}[T(x',y') - I(x+x',y+y')]^2,$$

where $I(x,y)$ is the value of the image pixel in the location (x,y), while $T(x,y)$ is the value of the template pixel in the location (x,y).

Normalized squared difference (`method == CV_TM_SQDIFF_NORMED`)

$$S(x,y) = \frac{\sum_{y'=0}^{h-1}\sum_{x'=0}^{w-1}[T(x',y') - I(x+x',y+y')]^2}{\sqrt{\sum_{y'=0}^{h-1}\sum_{x'=0}^{w-1}T(x',y')^2\sum_{y'=0}^{h-1}\sum_{x'=0}^{w-1}I(x+x',y+y')^2}}.$$

Cross correlation (`method == CV_TM_CCORR`):

$$C(x,y) = \sum_{y'=0}^{h-1}\sum_{x'=0}^{w-1}T(x',y')I(x+x',y+y').$$

Cross correlation, normalized (`method == CV_TM_CCORR_NORMED`):

$$\tilde{C}(x, y) = \frac{\sum_{y'=0}^{h-1} \sum_{x'=0}^{w-1} T(x', y') I(x + x', y + y')}{\sqrt{\sum_{y'=0}^{h-1} \sum_{x'=0}^{w-1} T(x', y')^2 \sum_{y'=0}^{h-1} \sum_{x'=0}^{w-1} I(x + x', y + y')^2}} .$$

Correlation coefficient (`method == CV_TM_CCOEFF`):

$$R(x, y) = \sum_{y'=0}^{h-1} \sum_{x'=0}^{w-1} \tilde{T}(x', y') \tilde{I}(x + x', y + y') ,$$

where $\tilde{T}(x', y') = T(x', y') - \bar{T}$, $\tilde{I}'(x + x', y + y') = I(x + x', y + y') - \bar{I}(x, y)$, and where $\bar{T}$ stands for the average value of pixels in the template raster and $\bar{I}(x, y)$ stands for the average value of the pixels in the current window of the image.

Correlation coefficient, normalized (`method == CV_TM_CCOEFF_NORMED`):

$$\tilde{R}(x, y) = \frac{\sum_{y'=0}^{h-1} \sum_{x'=0}^{w-1} \tilde{T}(x', y') \tilde{I}(x + x', y + y')}{\sqrt{\sum_{y'=0}^{h-1} \sum_{x'=0}^{w-1} \tilde{T}(x', y')^2 \sum_{y'=0}^{h-1} \sum_{x'=0}^{w-1} \tilde{I}(x + x', y + y')^2}} .$$

After the function `MatchTemplate` returns the resultant image, probable positions of the template in the image could be located as the local or global maximums of the resultant image brightness.

CvtPixToPlane

Divides pixel image into separate planes.

```
void cvCvtPixToPlane (IplImage* src, IplImage* dst0, IplImage* dst1, IplImage*
    dst2, IplImage* dst3);
```

src	Source image.
dst0...dst3	Destination planes.

Discussion

The function `CvtPixToPlane` divides a color image into separate planes. Two modes are available for the operation. Under the first mode the parameters *dst0*, *dst1*, and *dst2* are non-zero, while *dst3* must be zero for the three-channel source image. For the four-channel source image all the destination image pointers are non-zero. In this case the function splits the three/four channel image into separate planes and writes them to destination images. Under the second mode only one of the destination images is not `NULL;` in this case, the corresponding plane is extracted from the image and placed into destination image.

CvtPlaneToPix

Composes color image from separate planes.

```
void cvCvtPlaneToPix (IplImage* src0, IplImage* src1, IplImage* src2,
    IplImage* src3, IplImage* dst);
```

src0...src3	Source planes.
dst	Destination image.

Discussion

The function `CvtPlaneToPix` composes a color image from separate planes. If the *dst* has three channels, then *src0*, *src1*, and *src2* must be non-zero, otherwise *dst* must have four channels and all the source images must be non-zero.

ConvertScale

Converts one image to another with linear transformation.

```
void cvConvertScale (IplImage* src, IplImage* dst, double scale, double
    shift);
```

src Source image.

dst Destination image.

scale Scale factor.

shift Value added to the scaled source image pixels.

Discussion

The function `ConvertScale` applies linear transform to all pixels in the source image and puts the result into the destination image with appropriate type conversion. The following conversions are supported:

IPL_DEPTH_8U ↔ IPL_DEPTH_32F,

IPL_DEPTH_8U ↔ IPL_DEPTH_16S,

IPL_DEPTH_8S ↔ IPL_DEPTH_32F,

IPL_DEPTH_8S ↔ IPL_DEPTH_16S,

IPL_DEPTH_16S ↔ IPL_DEPTH_32F,

IPL_DEPTH_32S ↔ IPL_DEPTH_32F.

Applying the following formula converts integer types to float:

*dst(x,y) = (float)(src(x,y)*scale + shift)*,

while the following formula does the other conversions:

*dst(x,y) = saturate(round(src(x,y)*scale + shift))*,

where *round* function converts the floating-point number to the nearest integer number and *saturate* function performs as follows:

- Destination depth is `IPL_DEPTH_8U`: *saturate(x) = x < 0 ? 0 : x > 255 ? 255 : x*
- Destination depth is `IPL_DEPTH_8S`: *saturate(x) = x < -128 ? –128 : x > 127 ? 127 : x*
- Destination depth is `IPL_DEPTH_16S`: *saturate(x) = x < -32768 ? –32768 : x > 32767 ? 32767 : x*
- Destination depth is `IPL_DEPTH_32S`: *saturate(x) = x*.

LUT

Performs look-up table transformation on image.

```
CvMat* cvLUT (const CvArr* A, CvArr* B, const CvArr* lut);
```

A	Source image of 8-bit elements.
B	Destination array of arbitrary depth and of the same number of channels as the source array has.
lut	Look-up table of 256 elements; should be of the same depth as the destination array.

Discussion

The function `LUT` fills the destination array with values of look-up table entries. Indices of the entries are taken from the source array. That is, the function processes each pixel as follows:

$B_{ij} = lut[A_{ij} + \Delta]$, where Δ is equal to 0 for `8u` source image and to 128 for `8s` source image.

InitLineIterator

Initializes line iterator.

```
int cvInitLineIterator (IplImage* img, CvPoint pt1, CvPoint pt2,
    CvLineIterator* lineIterator);
```

img	Image.
pt1	Starting the line point.
pt2	Ending the line point.
lineIterator	Pointer to the line iterator state structure.

Discussion

The function `InitLineIterator` initializes the line iterator and returns the number of pixels between two end points. Both points must be inside the image. After the iterator has been initialized, all the points on the raster line that connects the two ending points may be retrieved by successive calls of `CV_NEXT_LINE_POINT` point. The points on the line are calculated one by one using the 8-point connected Bresenham algorithm. See Example 14-17 for the method of drawing the line in the RGB image with the image pixels that belong to the line mixed with the given color using the XOR operation.

Example 14-17 Drawing Line Using XOR Operation

```
void put_xor_line( IplImage* img, CvPoint pt1, CvPoint pt2, int r, int
g, int b ) {
CvLineIterator iterator;
int count = cvInitLineIterator( img, pt1, pt2, &iterator);
for( int i = 0; i < count; i++ ){
iterator.ptr[0] ^= (uchar)b;
iterator.ptr[1] ^= (uchar)g;
iterator.ptr[2] ^= (uchar)r;
CV_NEXT_LINE_POINT(iterator);
}
}
```

SampleLine

Reads raster line to buffer.

```
int cvSampleLine (IplImage* img, CvPoint pt1, CvPoint pt2, void* buffer);
```

img	Image.
pt1	Starting the line point.
pt2	Ending the line point.
buffer	Buffer to store the line points; must have enough size to store `MAX(|pt2.x - pt1.x| + 1,|pt2.y - pt1.y|+1)` points.

Discussion

The function `SampleLine` implements a particular case of application of line iterators. The function reads all the image points lying on the line between *pt1* and *pt2*, including the ending points, and stores them into the buffer.

GetRectSubPix

Retrieves raster rectangle from image with sub-pixel accuracy.

```
void cvGetRectSubPix (IplImage* src, IplImage* rect, CvPoint2D32f center);
```

src	Source image.
rect	Extracted rectangle; must have odd width and height.
center	Floating point coordinates of the rectangle center. The center must be inside the image.

Discussion

The function `GetRectSubPix` extracts pixels from *src*, if the pixel coordinates meet the following conditions:

```
center.x –(widthrect-1)/2 <= x <= center.x + (widthrect-1)/2;
center.y -(heightrect-1)/2 <= y <= center.y +(heightrect-1)/2.
```

Since the center coordinates are not integer, bilinear interpolation is applied to get the values of pixels in non-integer locations. Although the rectangle center must be inside the image, the whole rectangle may be partially occluded. In this case, the pixel values are spread from the boundaries outside the image to approximate values of occluded pixels.

bFastArctan

Calculates fast arctangent approximation for arrays of abscissas and ordinates.

```
void cvbFastAcrtan (const float* y, const float* x, float* angle, int len);
```

y	Array of ordinates.
x	Array of abscissas.
angle	Calculated angles of points (*x*[*i*],*y*[*i*]).
len	Number of elements in the arrays.

Discussion

The function `bFastAcrtan` calculates an approximate arctangent value, the angle of the point (*x*,*y*). The angle is in the range from 0° to 360°. Accuracy is about 0.1°. For point (0,0) the resultant angle is 0.

Sqrt

Calculates square root of single float.

```
float cvSqrt (float x);
```

x	Scalar argument.

Discussion

The function `Sqrt` calculates square root of a single argument. The argument should be non-negative, otherwise the result is unpredictable. The relative error is less than `9e-6`.

bSqrt

Calculates square root of array of floats.

```
void cvbSqrt (const float* x, float* y, int len);
```

x	Array of arguments.
y	Resultant array.
len	Number of elements in the arrays.

Discussion

The function `cvbSqrt` calculates the square root of an array of floats. The arguments should be non-negative, otherwise the results are unpredictable. The relative error is less than `3e-7`.

InvSqrt

Calculates inverse square root of single float.

```
float cvInvSqrt (float x);
```

x	Scalar argument.

Discussion

The function `InvSqrt` calculates the inverse square root of a single float. The argument should be positive, otherwise the result is unpredictable. The relative error is less than `9e-6`.

bInvSqrt

Calculates inverse square root of array of floats.

```
void cvbInvSqrt (const float* x, float* y, int len);
```

x	Array of arguments.
y	Resultant array.
len	Number of elements in the arrays.

Discussion

The function `bInvSqrt` calculates the inverse square root of an array of floats. The arguments should be positive, otherwise the results are unpredictable. The relative error is less than `3e-7`.

bReciprocal

Calculates inverse of array of floats.

```
void cvbReciprocal (const float* x, float* y, int len);
```

x	Array of arguments.
y	Resultant array.
len	Number of elements in the arrays.

Discussion

The function `bReciprocal` calculates the inverse (`1/x`) of arguments. The arguments should be non-zero. The function gives a very precise result with the relative error less than `1e-7`.

bCartToPolar

Calculates magnitude and angle for array of abscissas and ordinates.

```
void cvbCartToPolar (const float* y, const float* x, float* mag, float* angle,
    int len);
```

y	Array of ordinates.
x	Array of abscissas.
mag	Calculated magnitudes of points (x[i],y[i]).
angle	Calculated angles of points (x[i],y[i]).
len	Number of elements in the arrays.

Discussion

The function bCartToPolar calculates the magnitude $\sqrt{x[i]^2 + y[i]^2}$ and the angle $\arctan(y[i]/x[i])$ of each point (x[i],y[i]). The angle is measured in degrees and varies from 0° to 360°. The function is a combination of the functions bFastArctan and bSqrt, so the accuracy is the same as in these functions. If pointers to the angle array or the magnitude array are NULL, the corresponding part is not calculated.

bFastExp

Calculates fast exponent approximation for array of floats.

```
void cvbFastExp (const float* x, double* exp_x, int len);
```

x	Array of arguments.
exp_x	Array of results.
len	Number of elements in the arrays.

Discussion

The function `bFastExp` calculates fast exponent approximation for each element of the input array. The maximal relative error is about `7e-6`.

bFastLog

Calculates fast approximation of natural logarithm for array of doubles.

```
void cvbFastLog (const double* x, float* log_x, int len);
```

x	Array of arguments.
log_x	Array of results.
len	Number of elements in the arrays.

Discussion

The function `bFastLog` calculates fast logarithm approximation for each element of the input array. Maximal relative error is about `7e-6`.

RandInit

Initializes state of random number generator.

```
void cvRandInit (CvRandState* state, float lower, float upper, int seed);
```

state	Pointer to the initialized random number generator state.
lower	Lower boundary of uniform distribution.
upper	Upper boundary of uniform distribution.
seed	Initial 32-bit value to start a random sequence.

Discussion

The function `RandInit` initializes the *`state`* structure that is used for generating uniformly distributed numbers in the range `[`*`lower`*`, `*`upper`*`)`. A multiply-with-carry generator is used.

bRand

Fills array with random numbers.

```
void cvbRand (CvRandState* state, float* x, int len);
```

`state` Random number generator state.

`x` Destination array.

`len` Number of elements in the array.

Discussion

The function `bRand` fills the array with random numbers and updates generator state.

Rand

Fills array with uniformly distributed random numbers.

```
void cvRand (CvRandState* state, CvArr* arr);
```

`state` RNG state initialized by the function `RandInit` and, optionally, by `RandSetRange`.

`arr` Destination array.

Discussion

The function `Rand` fills the destination array with uniformly distributed random numbers and updates RNG state.

FillImage

Fills image with constant value.

```
void cvFillImage (IplImage* img, double val);
```

img	Filled image.
val	Value to fill the image.

Discussion

The function `FillImage` is equivalent to either `iplSetFP` or `iplSet`, depending on the pixel type, that is, floating-point or integer.

RandSetRange

Sets range of generated random numbers without reinitializing RNG state.

```
void cvRandSetRange (CvRandState* state, double lower, double upper);
```

state	State of random number generator (RNG).
lower	New lower bound of generated numbers.
upper	New upper bound of generated numbers.

Discussion

The function `RandSetRange` changes the range of generated random numbers without reinitializing RNG state. For the current implementation of RNG the function is equivalent to the following code:

```
unsigned seed = state.seed;
unsigned carry = state.carry;
cvRandInit( &state, lower, upper, 0 );
state.seed = seed;
state.carry = carry;
```

However, the function is preferable because of compatibility with the next versions of the library.

KMeans

Splits set of vectors into given number of clusters.

```
void cvKMeans (int numClusters, CvVect32f* samples, int numSamples, int
    vecSize, CvTermCriteria termcrit, int* cluster);
```

numClusters	Number of required clusters.
samples	Pointer to the array of input vectors.
numSamples	Number of input vectors.
vecSize	Size of every input vector.
termcrit	Criteria of iterative algorithm termination.
cluster	Characteristic array of cluster numbers, corresponding to each input vector.

Discussion

The function `KMeans` iteratively adjusts mean vectors of every cluster. Termination criteria must be used to stop the execution of the algorithm. At every iteration the convergence value is computed as follows:

$$\sum_{i=1}^{K} \| old\_mean_i - new\_mean_i \|^2 .$$

The function terminates if $E < Termcrit.epsilon$.

System Functions

15

This chapter describes system library functions.

Table 15-1 System Library Functions

Name	Description
`LoadPrimitives`	Loads versions of functions that are optimized for a specific platform.
`GetLibraryInfo`	Retrieves information about the library.

LoadPrimitives

Loads optimized versions of functions for specific platform.

```
int cvLoadPrimitives (char* dllName, char* processorType);
```

`dllName`	Name of dynamically linked library without postfix that contains the optimized versions of functions
`processorType`	Postfix that specifies the platform type:<br>“*`W7`*” for Pentium® 4 processor, “*`A6`*” for Intel® Pentium® II processor, “*`M6`*” for Intel® Pentium® II processor, `NULL` for auto detection of the platform type.

Discussion

The function `LoadPrimitives` loads the versions of functions that are optimized for a specific platform. The function is automatically called before the first call to the library function, if not called earlier.

GetLibraryInfo

Gets the library information string.

```
void cvGetLibraryInfo (char** version, int* loaded, char** dllName);
```

version	Pointer to the string that will receive the build date information; can be `NULL`.
loaded	Postfix that specifies the platform type:
	"*W7*" for Pentium® 4 processor, "*A6*" for Intel® Pentium® III processor, "*M6*" for Intel® Pentium® II processor, `NULL` for auto detection of the platform type.
dllName	Pointer to the full name of dynamically linked library without path, could be `NULL`.

Discussion

The function `GetLibraryInfo` retrieves information about the library: the build date, the flag that indicates whether optimized *DLLs* have been loaded or not, and their names, if loaded.

Bibliography 16

This bibliography provides a list of publications that might be useful to the Intel® Computer Vision Library users. This list is not complete; it serves only as a starting point.

[Borgefors86] Gunilla Borgefors. *Distance Transformations in Digital Images.* Computer Vision, Graphics and Image Processing 34, 344-371 (1986).

[Bradski00] G. Bradski and J. Davis. *Motion Segmentation and Pose Recognition with Motion History Gradients*. IEEE WACV'00, 2000.

[Burt81] P. J. Burt, T. H. Hong, A. Rosenfeld. *Segmentation and Estimation of Image Region Properties Through Cooperative Hierarchical Computation.* IEEE Tran. On SMC, Vol. 11, N.12, 1981, pp. 802-809.

[Canny86] J. Canny. *A Computational Approach to Edge Detection*, IEEE Trans. on Pattern Analysis and Machine Intelligence, 8(6), pp. 679-698 (1986).

[Davis97] J. Davis and Bobick. *The Representation and Recognition of Action Using Temporal Templates*. MIT Media Lab Technical Report 402, 1997.

[DeMenthon92] Daniel F. DeMenthon and Larry S. Davis. *Model-Based Object Pose in 25 Lines of Code.* In Proceedings of ECCV '92, pp. 335-343, 1992.

[Fitzgibbon95] Andrew W. Fitzgibbon, R.B.Fisher. *A Buyer's Guide to Conic Fitting*. Proc.5th British Machine Vision Conference, Birmingham, pp. 513-522, 1995.

[Horn81] Berthold K.P. Horn and Brian G. Schunck. *Determining Optical Flow.* Artificial Intelligence, 17, pp. 185-203, 1981.

[Hu62] M. Hu. *Visual Pattern Recognition by Moment Invariants*, IRE Transactions on Information Theory, 8:2, pp. 179-187, 1962.

[Jahne97] B. Jahne. *Digital Image Processing*. Springer, New York, 1997.

[Kass88] M. Kass, A. Witkin, and D. Terzopoulos. *Snakes: Active Contour Models*, International Journal of Computer Vision, pp. 321-331, 1988.

[Matas98] J.Matas, C.Galambos, J.Kittler. *Progressive Probabilistic Hough Transform*. British Machine Vision Conference, 1998.

[Rosenfeld73] A. Rosenfeld and E. Johnston. *Angle Detection on Digital Curves*. IEEE Trans. Computers, 22:875-878, 1973.

[RubnerJan98] Y. Rubner. C. Tomasi, L.J. Guibas. *Metrics for Distributions with Applications to Image Databases*. Proceedings of the 1998 IEEE International Conference on Computer Vision, Bombay, India, January 1998, pp. 59-66.

[RubnerSept98] Y. Rubner. C. Tomasi, L.J. Guibas. *The Earth Mover's Distance as a Metric for Image Retrieval*. Technical Report STAN-CS-TN-98-86, Department of Computer Science, Stanford University, September 1998.

[RubnerOct98] Y. Rubner. C. Tomasi. *Texture Metrics*. Proceeding of the IEEE International Conference on Systems, Man, and Cybernetics, San-Diego, CA, October 1998, pp. 4601-4607.

http://robotics.stanford.edu/~rubner/publications.html

[Serra82] J. Serra. *Image Analysis and Mathematical Morphology*. Academic Press, 1982.

[Schiele00] Bernt Schiele and James L. Crowley. *Recognition without Correspondence Using Multidimensional Receptive Field Histograms*. In International Journal of Computer Vision 36 (1), pp. 31-50, January 2000.

[Suzuki85] S. Suzuki, K. Abe. *Topological Structural Analysis of Digital Binary Images by Border Following*. CVGIP, v.30, n.1. 1985, pp. 32-46.

[Teh89] C.H. Teh, R.T. Chin. *On the Detection of Dominant Points on Digital Curves*. - IEEE Tr. PAMI, 1989, v.11, No.8, p. 859-872.

[Trucco98] Emanuele Trucco, Alessandro Verri. *Introductory Techniques for 3-D Computer Vision*. Prentice Hall, Inc., 1998.

[Welsh95] Greg Welsh, Gary Bishop. An Introduction To the Kalman Filter. Technical Report TR95-041, University of North Carolina at Chapel Hill, 1995.

[Williams92] D. J. Williams and M. Shah. *A Fast Algorithm for Active Contours and Curvature Estimation*. CVGIP: Image Understanding, Vol. 55, No. 1, pp. 14-26, Jan., 1992. http://www.cs.ucf.edu/~vision/papers/shah/92/WIS92A.pdf.

[Yuille89] A.Y.Yuille, D.S.Cohen, and P.W.Hallinan. *Feature Extraction from Faces Using Deformable Templates in CVPR*, pp. 104-109, 1989.

[Zhang96] Z. Zhang. *Parameter Estimation Techniques: A Tutorial with Application to Conic Fitting*, Image and Vision Computing Journal, 1996.

[Zhang99] Z. Zhang. *Flexible Camera Calibration By Viewing a Plane From Unknown Orientations*. International Conference on Computer Vision (ICCV'99), Corfu, Greece, pages 666-673, September 1999.

[Zhang00] Z. Zhang. *A Flexible New Technique for Camera Calibration*. IEEE Transactions on Pattern Analysis and Machine Intelligence, 22(11):1330-1334, 2000.

Supported Image Attributes and Operation Modes

A

The table below specifies what combinations of input/output parameters are accepted by different OpenCV functions. Currently, the table describes only array-processing functions, that is, functions, taking on input, output or both the structures `IplImage` and `CvMat`. Functions, working with complex data structures, e.g., contour processing, computational geometry, etc. are not included yet.

Format is coded in form *`depth`* , where *`depth`* is coded as `number of bits{u|s|f}`, `u` stands for "integer Unsigned", `s` stands for "integer Signed" and `f` stands for "Floating point".

For example, `8u` means 8-bit unsigned image or array, `32f` means floating-point image or array. `8u-64f` is a short form of `8u, 8s, 16s, 32s, 32f, 64f.`

If a function has several input/output arrays, they all must have the same type unless opposite is explicitly stated.

Word same in Output Format column means that the output array must have the same format with input array[s]. Word inplace in Output Format column means that the function changes content of one of the input arrays and thus produces the output. Word n/a means that the function output is not an image and format information is not applicable.

Mask parameter, if present, must have format `8u` or `8s`.

The following table includes only the functions that have raster images or matrices on input and/or on output.

Table A-1 Image Atributes and Operation Modes for Array-Processing Functions

Function	Input Format	Number of Channels	Output Format
AbsDiff	8u - 64f	1 - 4	same
AbsDiffS	8u - 64f	1 - 4	same
Acc	*src* = 8u, 8s, 32f	1, 3	inplace
	acc = 32f (same channels number as *src*)	1 - 3	
AdaptiveThreshold	8u, 8s, 32f	1	same
Add	8u, 16s, 32s, 32f, 64f	1 - 4	same
AddS	8u, 16s, 32s, 32f, 64f	1 - 4	same
And	8u - 64f	1 - 4	same
AndS	8u - 64f	1 - 4	same
bCartToPolar	32f	1	32f
bFastArctan	32f	1	32f
bFastExp	32f	1	64f
bFastLog	64f	1	64f
bInvSqrt	32f	1	32f
bRand	none	1	32f
bReciprocal	32f	1	32f
bSqrt	32f	1	32f
CalcAffineFlowPyrLK	*img* = 8u	1	32f
CalcBackProject	*histogram, img* = 8u, 8s, 32f	1	same as *img*
CalcEigenObjects	*img* = 8u	1	*eig* = 32f
CalcGlobalOrientation	*mhi* = 32f, *orient* = 32f, *mask* = 8u	1	32f

Table A-1 Image Atributes and Operation Modes for Array-Processing Functions (continued)

Function	Input Format	Number of Channels	Output Format
CalcHist	*img* = 8u, 8s, 32f	1	*histogram*
CalcMotionGradient	*mhi* = 32f	1	*orient* = 32f, *mask*
CalcOpticalFlowBM	8u	1	32f
CalcOpticalFlowHS	8u	1	32f
CalcOpticalFlowLK	8u	1	32f
CalcOpticalFlowPyrLK	*img* = 8u	1	32f
CamShift	8u, 8s, 32f	1	n/a
Canny	8u	1	8u
Circle	8u - 64f	1 - 4	inplace
CircleAA	8u	1, 3	inplace
Cmp	8u - 64f	1 - 4	8u
CmpS	8u - 64f	1 - 4	8u
ConvertScale	8u - 64f	1 - 4	8u - 64f, the same channels number
Copy	8u - 64f	1 - 4	same
CornerEigenValsAndVecs	8u, 8s, 32f	1	32f
CornerMinEigenVal	8u, 8s, 32f	1	32f
CountNonZero	8u - 64f	1 - 4	64f
CrossProduct	32f, 64f	1, 3	same (array size=3)
CvtPixToPlane	8u - 64f	input - 2, 3 or 4, output - 1	8u - 64f
CvtPlaneToPix	8u - 64f	input - 1, output - 2,3 or 4	8u - 64f
Det	32f, 64f	1	CvScalar
Dilate	8u, 32f	1, 3, 4	same

Table A-1 Image Atributes and Operation Modes for Array-Processing Functions (continued)

Function	Input Format	Number of Channels	Output Format
DistTransform	8u, 8s	1	32f
	8u - 64f	1 - 4	double
DrawContours	*contour*, *img* = 8u - 64f	1 - 4	inplace
EigenVV	32f, 64f	1	same
Ellipse	8u - 64f	1 - 4	inplace
EllipseAA	8u	1, 3	inplace
Erode	8u, 32f	1, 3, 4	same
FillConvexPoly	8u - 64f	1 - 4	inplace
FillPoly	8u - 64f	1 - 4	inplace
FindChessBoardCornerGuesses	8u	1	n/a
FindContours	*img* = 8u, 8s	1	contour
FindCornerSubPix	*img* = 8u, 8s, 32f	1	n/a
Flip	8u - 64f	1 - 4	same
FloodFill	8u, 32f	1	inplace
GetRectSubPix	8u, 8s, 32f, 64f	1	same or 32f or 64f for 8u & 8s
GoodFeaturesToTrack	*img* = 8u, 8s, 32f, *eig* = 32f, *temp* = 32f	1	n/a
HoughLines	*img* = 8u	1	n/a
HoughLinesP	*img* = 8u	1	n/a
HoughLinesSDiv	*img* = 8u	1	n/a
ImgToObs_DCT	*img* = 8u	1	n/a
Invert	32f, 64f	1	same
Laplace	8u, 8s, 32f	1	16s, 32f
Line	8u - 64f	1 - 4	inplace
LineAA	8u	1, 3	inplace

Table A-1 Image Atributes and Operation Modes for Array-Processing Functions (continued)

Function	Input Format	Number of Channels	Output Format
LUT	8u - 8s	1 - 4	8u - 64f
MatchTemplate	8u, 8s, 32f	1	32f
MatMulAdd	32f, 64f	1, 2	same
MatMulAddS	8u, 32s, 32f, 64f	2, 3, 4	same
MatMulAddEx	32f, 64f	1	same
Mean	8u - 64f	1 - 4	64f
Mean_StdDev	8u - 64f	1 - 4	64f
MeanShift	8u, 8s, 32f	1	n/a
MinMaxLoc	8u - 64f (*coi*!=0)	1 - 4	CvPoint, 64f
Moments	8u - 64f (*coi*!=0)	1 - 4	CvMoments
MorphologyEx	8u, 32f	1, 3, 4	same
Mul	8u, 16s, 32s, 32f, 64f	1 - 4	same
MulAddS (See Mul)	32f, 64f	1, 2	same
MultiplyAcc	*src* = 8u, 8s, 32f	1, 3	inplace
	acc = 32f (same channels number as *src*)	1 - 3	
MulTransposed	32f, 64f	1	same
Norm	8u - 64f (*coi*!=0, if *mask*!=0)	1 - 4	64f
Or	8u - 64f	1 - 4	same
OrS	8u - 64f	1 - 4	same
PerspectiveTransform	32f, 64f	3	same
PolyLine	8u - 64f	1 - 4	inplace

Table A-1 Image Atributes and Operation Modes for Array-Processing Functions (continued)

Function	Input Format	Number of Channels	Output Format
PolyLineAA	8u	1, 3	inplace
PreCornerDetect	8u, 8s, 32f	1	32f
PseudoInv	32f, 64f	1	same
PutText	8u - 64f	1 - 4	inplace
PyrDown	8u, 8s, 32f	1, 3	same
PyrSegmentation	8u	1, 3	same
PyrUp	8u, 8s, 32f	1, 3	same
Rand	-	1 - 4	8u - 64f
RandNext	none	1	32u
Rectangle	8u - 64f	1 - 4	inplace
Reshape	8u - 32f	1 - 4	same depth
RunningAvg	*src* = 8u, 8s, 32f	1, 3	inplace
	acc = 32f (same channels number as *src*)	1 - 3	
SampleLine	8u - 64f	1 - 4	inplace
ScaleAdd	32f, 64f	1, 2	same
SegmentMotion	32f	1	32f
Set	8u - 64f	1 - 4	inplace
SetIdentity	8u - 64f	1 - 4	inplace
SetZero	8u - 64f	1 - 4	inplace
SnakeImage	*img* = 8u, 8s, 32f	1	n/a
Sobel	8u, 8s, 32f	1	16s, 32f
SquareAcc	*src* = 8u, 8s, 32f	1, 3	inplace
	acc = 32f (same channels number as *src*)	1 - 3	

Table A-1 Image Atributes and Operation Modes for Array-Processing Functions (continued)

Function	Input Format	Number of Channels	Output Format
StartFindContours	*img* = 8u, 8s	1	contour
Sub	8u, 16s, 32s, 32f, 64f	1 - 4	same
SubRS	8u, 16s, 32s, 32f, 64f	1 - 4	same
SubS	8u, 16s, 32s, 32f, 64f	1 - 4	same
SubRS	8u, 16s, 32s, 32f, 64f	1 - 4	same
Sum	8u - 64f	1 - 4	64f
SVD	32f, 64f	1	same
Threshold	8u, 8s, 32f	1	same
Trace	8u - 64f	1 - 4	CvScalar
Invert	8u - 64f	1 - 4	same
UnDistort	8u	1, 3	same
UnDistortOnce	8u	1, 3	same
UpdateMotionHistory	*mhi* = 32f, *silh* = 8u, 8s	1	*mhi* = 32f
Xor	8u - 64f	1 - 4	same
XorS	8u - 64f	1 - 4	same

Glossary

arithmetic operation	An operation that adds, subtracts, multiplies, or squares the image pixel values.
background	A set of motionless image pixels, that is, pixels that do not belong to any object moving in front of the camera. This definition can vary if considered in other techniques of object extraction. For example, if a depth map of the scene is obtained, background can be defined as parts of scene that are located far enough from the camera.
blob	A region, either a positive or negative, that results from applying the Laplacian to an image. *See* Laplacian pyramid.
Burt's algorithm	An iterative pyramid-linking algorithm implementing a combined segmentation and feature computation. The algorithm finds connected components without a preliminary threshold, that is, it works on a grayscale image.
CamShift	Continuously Adaptive Mean-SHIFT algorithm. It is a modification of MeanShift algorithm that can track an object varying in size, e.g., because distance between the object and the camera varies.
channel of interest	channel in the image to process.
COI	*See* channel of interest.
connected component	A number of pixels sharing a side (or, in some cases, a corner as well).
corner	An area where level curves multiplied by the gradient magnitude assume a local maximum.

down-sampling	Down-sampling conceptually decreases image size by integer through replacing a pixel block with a single pixel. For instance, down-sampling by factor of 2 replaces a 2 X 2 block with a single pixel. In image processing convolution of the original image with blurring or Gaussian kernel precedes down-sampling.
earth mover distance	minimal work needed to translate one point mass configuration to another, normalized by the total configuration mass. The EMD is a optimal solution of transportation problem.
edge	A point at which the gradient assumes a local maximum along the gradient direction.
EMD	*See* earth mover distance.
flood filling	Flood filling means that a group of connected pixels with close values is filled with, or is set to, a certain value. The flood filling process starts with some point, called "seed", that is specified by function caller and then it propagates until it reaches the image ROI boundary or cannot find any new pixels to fill due to a large difference in pixel values.
Gaussian pyramid	A set of images derived from each other with combination of convolution with Gaussian kernel and down-sampling. *See* down-sampling and up-sampling.
histogram	A discrete approximation of stochastic variable probability distribution. The variable can be both a scalar value and a vector. Histograms represent a simple statistical description of an object, e.g., an image. The object characteristics are measured during iterations through that object
image features	*See* edge, ridge, and blob.
Laplacian pyramid	A set of images, which can be obtained by subtracting upsampled images from the original Gaussian Pyramid, that is, $L_i = G_i$ – up-sample (G_{i+1}) or $L_i = G_i$ – up-sample

	(down-sample (G_i)), where L_i are images from Laplacian Pyramid and G_i are images from Gaussian Pyramid. *See* also down-sampling and up-sampling.
locally minimum interceptive area triangle	A triangle made of two boundary runs in hierarchical representation of contours, if the interceptive area of its base line is smaller than both its neighboring triangles areas.
LMIAT	*See* locally minimum interceptive area triangle.
mathematical morphology	A set-theoretic method of image analysis first developed by Matheron and Serra. The two basic morphological operations are erosion (thinning) and dilation (thickening). All operations involve an image A (object of interest) and a kernel element B (structuring element).
memory storage	Storage that provides the space for storing dynamic data structures. A storage consists of a header and a double-linked list of memory blocks treated as a stack, that is, the storage header contains a pointer to the block not occupied entirely and an integer value, the number of free bytes in this block.
minimal enclosing circle	A circle in a planar point set whose points are entirely located either inside or on the boundary of the circle. *Minimal* means that there is no enclosing circle of a smaller radius.
MHI	*See* motion history image.
motion history image	Motion history image (MHI) represents how the motion took place. Each MHI pixel has a value of timestamp corresponding to the latest motion in that pixel. Very early motions, which occured in the past beyond a certain time threshold set from the current moment, are cleared out. As the person or object moves, copying the most recent foreground silhouette as the highest values in the motion history image creates a layered history of the resulting motion.
optical flow	An apparent motion of image brightness.

pixel value	An integer or float point value that defines brightness of the image point corresponding to this pixel. For instance, in the case of `8u` format images, the pixel value is an integer number from 0 to 255.
region of interest	A part of the image or a certain color plane in the image, or both.
ridge	Sort of a skeletonized high contrast object within an image. Ridges are found at points where the gradient is non-zero (or the gradient is above a small threshold).
ROI	*See* region of interest.
sequence	A resizable array of arbitrary type elements located in the memory storage. The sequence is discontinuous. Sequence data may be divided into several continuous blocks, called sequence blocks, that can be located in different memory blocks.
signature	Generalization of histograms under which characteristic values with rather fine quantization are gathered and only non-zero bins are dynamically stored.
snake	An energy-minimizing parametric closed curve guided by external forces.
template matching	Marking the image regions coinciding with the given template according to a certain rule (minimum squared difference or maximum correlation between the region and template).
tolerance interval	Lower and upper levels of pixel values corresponding to certain conditions. *See* pixel value.
up-sampling	Up-sampling conceptually increases image size through replacing a single pixel with a pixel block. For instance, up-sampling by factor of 2 replaces a single pixel with a 2 X 2 block. In image processing convolution of the original image with Gaussian kernel, multiplied by the squared up-sampling factor, follows up-sampling.

Index

A

about this manual, 1-4

about this software, 1-1

Active Contours
- energy function, 2-15
  - contour continuity, 2-16
  - contour continuity energy, 2-16
  - contour curvature energy, 2-16
  - external energy, 2-15
  - internal energy, 2-15
  - snake corners, 2-17
- full snake energy, 2-16

Active Contours Function, 9-11
- SnakeImage, 9-11

audience for this manual, 1-8

B

Backgroud Subtraction Functions, 9-3

Background subtraction
- background, 2-1
- background model, 2-1

Background Subtraction Functions
- Acc, 9-3
- MultiplyAcc, 9-4
- RunningAvg, 9-5
- SquareAcc, 9-4

bi-level image, 3-11, 3-15, 3-24

binary tree representation, 4-10

black-and-white image, 3-24

blob, 3-24

Block Matching, 2-20

Burt's algorithm, 3-17

C

Camera Calibration, 6-1
- homography, 6-2
- lens distortion, 6-4
- pattern, 6-3

Camera Calibration Functions
- CalibrateCamera, 13-4
- CalibrateCamera_64d, 13-5
- FindChessBoardCornerGuesses, 13-11
- FindExtrinsicCameraParams, 13-6
- FindExtrinsicCameraParams_64d, 13-7
- Rodrigues, 13-7
- Rodrigues_64d, 13-8
- UnDistort, 13-10
- UnDistortInit, 13-9
- UnDistortOnce, 13-9

camera parameters, 6-1
- extrinsic, 6-1
  - rotation matrix, 6-1, 6-2
  - translation vector, 6-1, 6-2
- intrinsic, 6-1
  - effective pixel size, 6-1
  - focal length, 6-1
  - location of the image center, 6-1
  - radial distortion coefficient, 6-1

camera undistortion functions, 6-5

CamShift algorithm, 2-9, 2-10, 2-12
- calculation of 2D orientation, 2-14
- discrete distributions, 2-11

- dynamically changing distributions, 2-11
- mass center calculation, 2-11
- probability distribution image, 2-10
- search window, 2-11
- zeroth moment, 2-11

CamShift Functions, 9-9
- CamShift, 9-9
- MeanShift, 9-10

centroid, 2-11

channel of interest, 7-3

child node, 4-10

CNP, See corresponding node pair

codes
- chain codes, 4-1
- higher order codes, 4-1

COI, See channel of interest

conic fitting, 4-14

Contour Processing, 4-1
- contours moments, 4-5
- Douglas-Peucker approximation, 4-4
- hierarchical representation of contours, 4-8
- locally minimum interceptive area triangle, 4-9
- polygonal approximation, 4-1

Contour Processing Functions
- ApproxChains, 11-3
- ApproxPoly, 11-5
- ContourArea, 11-8
- ContourBoundingRect, 11-7
- ContourFromContourTree, 11-11
- ContoursMoments, 11-8
- CreateContourTree, 11-10
- DrawContours, 11-6
- EndFindContours, 10-9
- FindContours, 10-6
- FindNextContour, 10-8
- MatchContours, 11-9
- MatchContourTrees, 11-12
- ReadChainPoint, 11-5
- StartFindContours, 10-7
- StartReadChainPoints, 11-4
- SubstituteContour, 10-9

Contour Retrieving
- 1-component
  - border, 3-3
  - border point, 3-3
  - hole, 3-3
  - hole border, 3-3
  - outer border, 3-3
- 4-connected pixels, 3-1
- 8-connected pixels, 3-1
- algorithm, 3-4
- border following procedure, 3-5
- chain code See Freeman method
- contour See 1-component border
- Freeman method, 3-3 See also chain code
- hierarchical connected components, 3-2
- polygonal representation, 3-4

contours moments, 4-5

conventions
- font, 1-9
- naming, 1-9

convergence, 6-15

convexity defects, 4-16

corresponding node pair, 4-13

covariance matrix, 5-1

D

Data Types supported, 1-3

decomposition coefficients, 5-2

deque, 7-5

Distance Transform Function
- DistTransform, 10-34

Douglas-Peucker approximation, 4-4

Drawing Primitives Functions
- Circle, 14-96
- Ellipse, 14-96
- EllipseAA, 14-98
- FillConvexPoly, 14-99
- FillPoly, 14-98
- GetTextSize, 14-102
- InitFont, 14-101
- Line, 14-94
- LineAA, 14-94

PolyLine, 14-100
PolyLineAA, 14-100
PutText, 14-102
Rectangle, 14-95

Dynamic Data Structures
Graphs
ClearGraph, 14-54
CreateGraph, 14-46
FindGraphEdge, 14-51
FindGraphEdgeByPtr, 14-52
GetGraphVtx, 14-54
GraphAddEdge, 14-48
GraphAddEdgeByPtr, 14-49
GraphAddVtx, 14-46
GraphEdgeIdx, 14-55
GraphRemoveEdge, 14-50
GraphRemoveEdgeByPtr, 14-50
GraphRemoveVtx, 14-47
GraphRemoveVtxByPtr, 14-47
GraphVtxDegree, 14-52
GraphVtxDegreeByPtr, 14-53
GraphVtxIdx, 14-54
Memory Functions
ClearMemStorage, 14-23
CreateChildMemStorage, 14-22
CreateMemStorage, 14-22
ReleaseMemStorage, 14-23
RestoreMemStoragePos, 14-24
Sequence Reference
cvSeqBlock Structure Definition, 14-28
cvSequence Structure Definition, 14-26
Standard Kinds of Sequences, 14-27
Standard Types of Sequence Elements, 14-27
Sequences
ClearSeq, 14-34
CreateSeq, 14-29
CvtSeqToArray, 14-36
GetSeqElem, 14-35
MakeSeqHeaderForArray, 14-36
SeqElemIdx, 14-35
SeqInsert, 14-33
SeqPop, 14-31
SeqPopFront, 14-32
SeqPopMulti, 14-33
SeqPush, 14-30
SeqPushFront, 14-31
SeqPushMulti, 14-32
SeqRemove, 14-34
SetSeqBlockSize, 14-30
Sets
ClearSet, 14-44
CreateSet, 14-42
GetSetElem, 14-43
SetAdd, 14-42
SetRemove, 14-43
Writing and Reading Sequences
EndWriteSeq, 14-39
FlushSeqWriter, 14-39
GetSeqReaderPos, 14-41
SetSeqReaderPos, 14-41
StartAppendToSeq, 14-37
StartReadSeq, 14-40
StartWriteSeq, 14-38

Dynamic Data Structures Reference
Memory Storage
cvMemBlock Structure Definition, 14-21
cvMemStorage Structure Definition, 14-21
cvMemStoragePos Structure Definition, 14-21

E

Earth mover distance, 3-27

Eigen Objects, 5-1

Eigen Objects Functions
CalcCovarMatrixEx, 12-3
CalcDecompCoeff, 12-5
CalcEigenObjects, 12-4
EigenDecomposite, 12-6
EigenProjection, 12-7

eigenvectors, 5-1

ellipse fitting, 4-14

Embedded Hidden Markov Models, 5-2

Embedded Hidden Markov Models Functions
Create2DHMM, 12-12
CreateObsInfo, 12-13
EstimateHMMStateParams, 12-17

EstimateObsProb, 12-18
EstimateTransProb, 12-17
EViterbi, 12-18
ImgToObs_DCT, 12-14
InitMixSegm, 12-16
MixSegmL2, 12-19
Release2DHMM, 12-13
ReleaseObsInfo, 12-14
UniformImgSegm, 12-15
EMD, See Earth mover distance
error handling, 1-3
Estimators
ConDensation algorithm, 2-23
discrete Kalman estimator, 2-22
Kalman filter, 2-22
measurement update, 2-21
equations, 2-23
state estimation programs, 2-20
system model, 2-21
system parameters, 2-21
system state, 2-20
time update, 2-21
equations, 2-23
Estimators Functions, 9-16
ConDensInitSampleSet, 9-18
ConDensUpdatebyTime, 9-19
CreateConDensation, 9-17
CreateKalman, 9-16
KalmanUpdateByMeasurement, 9-17
KalmanUpdateByTime, 9-17
ReleaseConDensation, 9-18
ReleaseKalman, 9-16

F

Features, 3-5
Canny edge detection, 3-11
differentiation, 3-12
edge thresholding, 3-13
hysteresis thresholding, 3-13
image smoothing, 3-12
non-maximum suppression, 3-12
streaking, 3-13
corner detection, 3-11
feature detection, 3-10
Fixed Filters, 3-5
convolution primitives, 3-6
first Sobel derivative operators, 3-6
second Sobel derivative operators, 3-7
third Sobel derivative operators, 3-9
Hough transform, 3-14
multidimentsional Hough Transform, 3-14 See also standard Hough transform, 3-14
Optimal Filter Kernels with Floating Point Coefficients
first derivative filters, 3-9
optimal filter kernels with floating point coefficients, 3-9
Laplacian approximation, 3-10
second derivative filters, 3-10
progressive probabilistic Hough Transform, 3-14 See also Hough transform, 3-14
standard Hough Transform, 3-14 See also Hough transform, 3-14
Features Functions
Feature Detection Functions
Canny, 10-11
CornerEigenValsandVecs, 10-12
CornerMinEigenVal, 10-13
FindCornerSubPix, 10-14
GoodFeaturesToTrack, 10-16
PreCornerDetect, 10-12
Fixed Filters Functions
Laplace, 10-10
Sobel, 10-10
Hough Transform Functions
HoughLines, 10-17
HoughLinesP, 10-19
HoughLinesSDiv, 10-18
Flood Filling
4-connectivity, 3-25
8-connectivity, 3-25
definition, 3-25
seed, 3-25
Flood Filling Function
FloodFill, 10-40

flush, 7-7
focal length, 6-2
font conventions, 1-9
function descriptions, 1-8

G

Gabor transform, 3-29
Gaussian window, 2-19
GDI draw functions, 7-18
geometric image formation, 6-10
Geometry
- convexity defects, 4-16
- ellipse fitting, 4-14
- fitting of conic, 4-14
- line fitting, 4-15
- weighted least squares, 4-16

Geometry Data Types, 11-25
- cvConvexityDefect Structure Definition, 11-25

Geometry Functions
- CalcPGH, 11-23
- CheckContourConvexity, 11-21
- ContourConvexHull, 11-18
- ContourConvexHullApprox, 11-20
- ConvexHull, 11-17
- ConvexHullApprox, 11-18
- ConvexityDefects, 11-21
- FitEllipse, 11-12
- FitLine2D, 11-13
- FitLine3D, 11-15
- MinAreaRect, 11-22
- MinEnclosingCircle, 11-24
- Project3D, 11-16

Gesture Recognition
- algorithm, 6-16
- homography matrix, 6-18
- image mask, 6-17
- probability density, 6-17

Gesture Recognition Functions
- CalcImageHomography, 13-23
- CalcProbDensity, 13-24
- CreateHandMask, 13-23
- FindHandRegion, 13-21
- FindHandRegionA, 13-22
- MaxRect, 13-25

graph
- non-oriented, 7-13
- oriented, 7-13

graphs, 7-11
grayscale image, 3-11, 3-15, 3-20, 3-24, 7-2, 7-18
Green’s formula, 4-5

H

hardware and software requirements, 1-3
header, 7-4, 7-10
hierarchical representation of contours, 4-8
Histogram
- analyzing shapes, 3-26
- bayesian-based object recognition, 3-26
- content based retrieval, 3-26
- definition, 3-25
- histogram back-projection, 2-10
- signature, 3-27

Histogram Data Types, 10-57
Histogram Functions
- CalcBackProject, 10-51
- CalcBackProjectPatch, 10-52
- CalcContrastHist, 10-55
- CalcEMD, 10-54
- CalcHist, 10-50
- CompareHist, 10-48
- CopyHist, 10-49
- CreateHist, 10-41
- GetHistValue_1D, 10-45
- GetHistValue_2D, 10-45
- GetHistValue_3D, 10-46
- GetHistValue_nD, 10-46
- GetMinMaxHistValue, 10-47
- MakeHistHeaderForArray, 10-42
- NormalizeHist, 10-47
- QueryHistValue_1D, 10-43
- QueryHistValue_2D, 10-43
- QueryHistValue_3D, 10-44

QueryHistValue_nD, 10-44
ReleaseHist, 10-42
SetHistThresh, 10-50
ThreshHist, 10-48
HMM, See Embedded Hidden Markov Models
homography, 6-2
homography matrix, 6-2, 6-18
Horn & Schunck Technique, 2-19
Lagrangian multiplier, 2-19
HT, See Hough Transform in Features
Hu invariants, 3-15
Hu moments, 6-18

I

Image Functions, 7-1
Image Functions Reference
CopyImage, 14-15
CreateImage, 14-9
CreateImageData, 14-11
CreateImageHeader, 14-9
GetImageRawData, 14-14
InitImageHeader, 14-14
ReleaseImage, 14-10
ReleaseImageData, 14-12
ReleaseImageHeader, 14-10
SetImageCOI, 14-13
SetImageData, 14-12
SetImageROI, 14-13
Image Statistics Functions
CountNonZero, 10-20
GetCentralMoment, 10-25
GetHuMoments, 10-27
GetNormalizedCentralMoment, 10-26
GetSpatialMoment, 10-25
Mean, 10-21
Mean_StdDev, 10-21
MinMaxLoc, 10-22
Moments, 10-24
Norm, 10-22
SumPixels, 10-20
Intel® Image Processing Library, 1-1, 7-1
IPL, See Intel® Image Processing Library

L

Lagrange multiplier, 4-15
least squares method, 4-15
lens distortion, 6-2
distortion coefficients
radial, 6-4
tangenial, 6-4
line fitting, 4-15
LMIAT, See locally minimum interceptive area triangle
Lucas & Kanade Technique, 2-19

M

Mahalanobis distance, 6-18
manual organization, 1-4
mathematical morphology, 3-19
Matrix Operations, 7-15
Matrix Operations Data Types
cvMatArray Structure Definition, 14-57
Matrix Operations Functions
Add, 14-71
AddS, 14-72
AllocArray, 14-69
And, 14-75
AndS, 14-76
CloneMat, 14-61
Copy, 14-70, 14-89
CreateData, 14-69
CreateMat, 14-58
CreateMatHeader, 14-58
CrossProduct, 14-82
Det, 14-86
DotProduct, 14-75
Flip, 14-87
FreeArray, 14-70
GetAt, 14-63
GetAtPtr, 14-65
GetCol, 14-66
GetDiag, 14-67

GetMat, 14-62
GetRawData, 14-67
GetRow, 14-66
GetSize, 14-68
GetSubArr, 14-65
InitMatHeader, 14-60
Invert, 14-85
Mahalonobis, 14-86
MatMulAdd, 14-75
MatMulAddS, 14-84
Mul, 14-75
MulAddS, 14-83
MulTransposed, 14-75
Or, 14-77
OrS, 14-78
PerspectiveTransform, 14-93
PseudoInv, 14-91
ReleaseData, 14-69
ReleaseMat, 14-59
ReleaseMatHeader, 14-60
Reshape, 14-88
ScaleAdd, 14-75, 14-82
SetAt, 14-64
SetData, 14-62
SetIdentity, 14-90
SetZero, 14-89
Sub, 14-73
SubRS, 14-74
SubS, 14-73
SVD, 14-88, 14-90, 14-91
Trace, 14-86
Transpose, 14-85, 14-87
Xor, 14-79
XorS, 14-80

mean location, 2-11

Mean Shift algorithm, 2-9

memory block, 7-4

memory storage, 7-4

M-estimators, 4-15

MHT See multidimesional Hough transform in Features

model plane, 6-3

moire, 6-8

Morphology
angle resolution, 3-29
black hat, 3-23
CIE Lab model, 3-29
closing equation, 3-21
dilation, 3-19
dilation formula, 3-20
dilation formula in 3D, 3-22
dilation in 3D, 3-21
Earth mover distance, 3-27
erision in 3D, 3-21
erosion, 3-19
erosion formula, 3-20
erosion formula in 3D, 3-23
flow matrix, 3-28
ground distance, 3-29
lower boundary of EMD, 3-30
morphological gradient function, 3-23
object of interest, 3-19
opening equation, 3-21
optimal flow, 3-28
scale resolution, 3-29
structuring element, 3-19
thickening, See dilation
thinning, See erosion
top hat, 3-23

Morphology Functions
CreateStructuringElementEx, 10-30
Dilate, 10-32
Erode, 10-31
MorphologyEx, 10-33
ReleaseStructuringElement, 10-31

Motion History Image, 2-3

motion representation, 2-2
motion gradient image, 2-3
regional orientation, 2-6

motion segmentation, 2-7
downward stepping floodfill, 2-7

Motion Templates
motion template images, 2-2
normal optical flow method, 2-2

Motion Templates Functions, 9-6
CalcGlobalOrientation, 9-7

CalcMotionGradient, 9-6
SegmentMotion, 9-8
UpdateMotionHistory, 9-6

N

node
child, 4-10
parent, 4-10
root, 4-10
trivial, 4-13
node distance, 4-13
node weight, 4-13
non-coplanar points, See also non-degenerate points, 6-14
non-degenerate points, See also non-coplanar points, 6-14
non-maxima suppression, 4-3
notational conventions, 1-8

O

object model pseudoinverse, 6-14
online version, 1-8
optical flow, 2-18
Optical Flow Functions, 9-12
CalcOpticalFlowBM, 9-13
CalcOpticalFlowHS, 9-12
CalcOpticalFlowLK, 9-13
CalcOpticalFlowPyrLK, 9-14

P

parent node, 4-10
perspective distortion, 6-14
perspective model, 6-10
pinhole model, See perspective model
Pixel Access Macros, 14-15
Pixel Access Macros Reference
CV_INIT_PIXEL_POS, 14-17
CV_MOVE, 14-18
CV_MOVE_PARAM, 14-19
CV_MOVE_PARAM_WRAP, 14-19
CV_MOVE_TO, 14-17
CV_MOVE_WRAP, 14-18
Pixel Access Macros Structures
cvPixelPosition Structures, 14-16
platforms supported, 1-4
polygonal approximation, 4-1
k-cosine curvature, 4-2
L1 curvature, 4-2
Rosenfeld-Johnston algorithm, 4-2
Teh and Chin algorithm, 4-3
POS, See pose from orthography ans scaling
pose, 6-10
pose approximation method, 6-12
pose from orthography and scaling, 6-12
POSIT
algorithm, 6-14
focal length, 6-14
geometric image formation, 6-10
object image, 6-14
object model, 6-14
pose approximation method, 6-12
pose from orthography and scaling, 6-12
POSIT algorithm, 6-10
POSIT Functions
CreatePOSITObject, 13-19
POSIT, 13-19
ReleasePOSITObject, 13-20
PPHT See progressive probabilistic Hough transform in Features
prefix, in function names, 1-9
PUSH version, 7-6
Pyramid, 10-56
Pyramid Data Types
cvConnectedComp Structure Definition, 10-56
Pyramid Functions
PyrDown, 10-28
PyrSegmentation, 10-29
PyrUp, 10-28
Pyramids

down-sampling, 3-15
Gaussian, 3-15
image segmentation, 3-17
hierarchical computing structure, 3-17
hierarchical smoothing, 3-17
segmentation, 3-17
Laplacian, 3-15
son-father relationship, 3-17
up-sampling, 3-16

R

radial distortion, 6-2
radial distortion coefficients, 6-4
region of interest, 7-3
related publications, 1-8
RLE coding, 4-1
ROI, See region of interest
root node, 4-10
Rosenfeld-Johnston algorithm, 4-2
rotation matrix, 6-6
rotation vector, 6-6

S

scalar factor, 6-3
scaled orthographic projection, See also weak-perspective projection model, 6-11
scanlines, 6-6
Sequence Reference, 14-26
sequences, 7-5
sets, 7-8
shape partitioning, 4-12
SHT, See standard Hough transform in Features
stochastic variable, 3-15
synthesized image, 6-6
System Functions
GetLibraryInfo, 15-2
LoadPrimitives, 15-1

T

tangential distortion coefficients, 6-4
Teh and Chin algorithm, 4-3
three sigmas rule, 2-1
Threshold Functions
AdaptiveThreshold, 10-36
Threshold, 10-38
trivial node, 4-13

U

Use of Eigen Object Functions, 12-7
Use of Eigen Objects Functions
cvCalcEigenObjects in Callback Mode, 12-9
cvCalcEigenObjects in Direct Access Mode, 12-8
Utility Functions
AbsDiff, 14-103
AbsDiffS, 14-104
bCartToPolar, 14-115
bFastArctan, 14-112
bFastExp, 14-115
bFastLog, 14-116
bInvSqrt, 14-114
bRand, 14-117
bReciprocal, 14-114
bSqrt, 14-113
ConvertScale, 14-108
CvtPixToPlane, 14-107
CvtPlaneToPix, 14-107
FillImage, 14-118
GetRectSubPix, 14-111
InitLineIterator, 14-110
InvSqrt, 14-113
KMeans, 14-119
LUT, 14-109
MatchTemplate, 14-104
Rand, 14-117
RandInit, 14-116
RandSetRange, 14-118
SampleLine, 14-111
Sqrt, 14-112

V

vectoring algorithms, 3-1
View Morphing, 6-6
  moire, 6-8
  scanlines, 6-6
  warped image, 6-6
view morphing algorithm, 6-6
View Morphing Functions
  DeleteMoire, 13-18
  DynamicCorrespondMulti, 13-15
  FindFundamentalMatrix, 13-12
  FindRuns, 13-14
  MakeAlphaScanlines, 13-16
  MakeScanlines, 13-13
  MorphEpilinesMulti, 13-16
  PostWarpImage, 13-17
  PreWarpImage, 13-13

W

warped image, 6-6
weak-perspective projection, 6-12
weak-perspective projection model, 6-11
weighted least squares, 4-16
world coordinate system, 6-2
