# Supplementary material for: Rapid whole brain imaging of neural activity in freely behaving larval zebrafish (Danio rerio)
Source: eLife. 2017 Sep 20;6:e28158. doi: 10.7554/eLife.28158 (PMC5644961; doi:10.7554/eLife.28158)
Supplement: Source code 3. [file elife-28158-code3.zip › RT analysis code/OpenCV1.1pre.zip!/OpenCV/docs/papers/algo_tracking.pdf]

Pyramidal Implementation of the Lucas Kanade Feature Tracker Description of the algorithm

Jean-Yves Bouguet

Intel Corporation
Microprocessor Research Labs
jean-yves.bouguet@intel.com

1 Problem Statement

Let I and J be two 2D grayscaled images. The two quantities $I(\mathbf{x}) = I(x, y)$ and $J(\mathbf{x}) = J(x, y)$ are then the grayscale value of the two images are the location $\mathbf{x} = [x \;\; y]^T$, where x and y are the two pixel coordinates of a generic image point $\mathbf{x}$. The image I will sometimes be referenced as the first image, and the image J as the second image. For practical issues, the images I and J are discret function (or arrays), and the upper left corner pixel coordinate vector is $[0 \;\; 0]^T$. Let n_x and n_y be the width and height of the two images. Then the lower right pixel coordinate vector is $[n_x - 1 \;\; n_y - 1]^T$.
Consider an image point $\mathbf{u} = [u_x \;\; u_y]^T$ on the first image I. The goal of feature tracking is to find the location $\mathbf{v} = \mathbf{u} + \mathbf{d} = [u_x + d_x \;\; u_y + d_y]^T$ on the second image J such as $I(\mathbf{u})$ and $J(\mathbf{v})$ are "similar". The vector $\mathbf{d} = [d_x \;\; d_y]^T$ is the image velocity at $\mathbf{x}$, also known as the optical flow at $\mathbf{x}$. Because of the *aperture problem*, it is essential to define the notion of similarity in a 2D neighborhood sense. Let ω_x and ω_y two integers. We define the image velocity $\mathbf{d}$ as being the vector that minimizes the residual function ϵ defined as follows:

$$\epsilon(\mathbf{d}) = \epsilon(d_x, d_y) = \sum_{x=u_x-\omega_x}^{u_x+\omega_x} \sum_{y=u_y-\omega_y}^{u_y+\omega_y} \left(I(x, y) - J(x + d_x, y + d_y) \right)^2 . \tag{1}$$

Observe that following that defintion, the similarity function is measured on a image neighborhood of size $(2\omega_x + 1) \times (2\omega_y + 1)$. This neighborhood will be also called integration window. Typical values for ω_x and ω_y are 2,3,4,5,6,7 pixels.

2 Description of the tracking algorithm

The two key components to any feature tracker are accuracy and robustness. The accuracy component relates to the local sub-pixel accuracy attached to tracking. Intuitively, a small integration window would be preferable in order not to "smooth out" the details contained in the images (i.e. small values of ω_x and ω_y). That is especially required at occluding areas in the images where two patchs potentially move with very different velocities.

The robustness component relates to sensitivity of tracking with respect to changes of lighting, size of image motion,... In particular, in oder to handle large motions, it is intuively preferable to pick a large integration window. Indeed, considering only equation 1, it is preferable to have $d_x \leq \omega_x$ and $d_y \leq \omega_y$ (unless some prior matching information is available). There is therefore a natural tradeoff between local accuracy and robustness when choosing the integration window size. In provide to provide a solution to that problem, we propose a pyramidal implementation of the classical Lucas-Kanade algorithm. An iterative implementation of the Lucas-Kanade optical flow computation provides sufficient local tracking accuracy.

2.1 Image pyramid representation

Let us define the pyramid representsation of a generic image I of size $n_x \times n_y$. Let $I^0 = I$ be the "zeroth" level image. This image is essentially the highest resolution image (the raw image). The image width and height at that level are defined as $n_x^0 = n_x$ and $n_y^0 = n_y$. The pyramid representation is then built in a recursive fashion: compute I^1 from I^0, then compute I^2 from I^1, and so on... Let $L = 1, 2, \ldots$ be a generic pyramidal level, and let I^{L-1} be the image at level $L - 1$. Denote n_x^{L-1} and n_y^{L-1} the width and height of I^{L-1}. The image I^{L-1} is then defined as follows:

$$\begin{aligned} I^L(x, y) \;\; = \;\; & \frac{1}{4} I^{L-1}(2x, 2y) + \\ & \frac{1}{8} \left(I^{L-1}(2x-1, 2y) + I^{L-1}(2x+1, 2y) + I^{L-1}(2x, 2y-1) + I^{L-1}(2x, 2y+1) \right) + \\ & \frac{1}{16} \left(I^{L-1}(2x-1, 2y-1) + I^{L-1}(2x+1, 2y+1) + I^{L-1}(2x-1, 2y+1) + I^{L-1}(2x+1, 2y+1) \right) . \end{aligned} \tag{2}$$

For simplicity in the notation, let us define dummy image values one pixel around the image I^{L-1} (for $0 \leq x \leq n_x^{L-1}-1$ and $0 \leq y \leq n_y^{L-1}-1$):

$$
\begin{aligned}
I^{L-1}(-1,y) &\doteq I^{L-1}(0,y),\\
I^{L-1}(x,-1) &\doteq I^{L-1}(x,0),\\
I^{L-1}(n_x^{L-1},y) &\doteq I^{L-1}(n_x^{L-1}-1,y),\\
I^{L-1}(x,n_y^{L-1}) &\doteq I^{L-1}(x,n_y^{L-1}-1),\\
I^{L-1}(n_x^{L-1},n_y^{L-1}) &\doteq I^{L-1}(n_x^{L-1}-1,n_y^{L-1}-1).
\end{aligned}
$$

Then, equation 2 is only defined for values of x and y such that $0 \leq 2x \leq n_x^{L-1}-1$ and $0 \leq 2y \leq n_y^{L-1}-1$. Therefore, the width n_x^L and height n_y^L of I^L are the largest integers that satisfy the two conditions:

$$n_x^L \leq \frac{n_x^{L-1}+1}{2}, \tag{3}$$

$$n_y^L \leq \frac{n_y^{L-1}+1}{2}. \tag{4}$$

Equations (2), (3) and (4) are used to construct recursively the pyramidal representations of the two images I and J: $\{I^L\}_{L=0,\dots,L_m}$ and $\{J^L\}_{L=0,\dots,L_m}$. The value L_m is the height of the pyramid (picked heuristically). Practical values of L_m are 2,3,4. For typical image sizes, it makes no sense to go above a level 4. For example, for an image I of size 640×480, the images I^1, I^2, I^3 and I^4 are of respective sizes 320×240, 160×120, 80×60 and 40×30. Going beyond level 4 does not make much sense in most cases. The central motivation behind pyramidal representation is to be able to handle large pixel motions (larger than the integration window sizes ω_x and ω_y). Therefore the pyramid height (L_m) should also be picked appropriately according to the maximum expected optical flow in the image. The next section describing the tracking operation in detail we let us understand that concept better. Final observation: equation 2 suggests that the lowpass filter $[1/4\ \ 1/2\ \ 1/4] \times [1/4\ \ 1/2\ \ 1/4]^T$ is used for image anti-aliasing before image subsampling. In practice however (in the C code) a larger anti-aliasing filter kernel is used for pyramid construction $[1/16\ \ 1/4\ \ 3/8\ \ 1/4\ \ 1/16] \times [1/16\ \ 1/4\ \ 3/8\ \ 1/4\ \ 1/16]^T$. The formalism remains the same.

2.2 Pyramidal Feature Tracking

Recall the goal of feature tracking: for a given point $\mathbf{u}$ in image I, find its corresponding location $\mathbf{v} = \mathbf{u} + \mathbf{d}$ in image J, or alternatively find its pixel displacement vector $\mathbf{d}$ (see equation 1).

For $L = 0, \dots, L_m$, define $\mathbf{u}^L = [u_x^L\ \ u_y^L]$, the corresponding coordinates of the point $\mathbf{u}$ on the pyramidal images I^L. Following our definition of the pyramid representation equations (2), (3) and (4), the vectors $\mathbf{u}^L$ are computed as follows:

$$\mathbf{u}^L = \frac{\mathbf{u}}{2^L}. \tag{5}$$

The division operation in equation 5 is applied to both coordinates independently (so will be the multiplication operations appearing in subsequent equations). Observe that in particular, $\mathbf{u}^0 = \mathbf{u}$.

The overall pyramidal tracking algorithm proceeds as follows: first, the optical flow is comptuted at the deepest pyramid level L_m. Then, the result of the that computation is propagated to the upper level $L_m - 1$ in a form of an initial guess for the pixel displacement (at level $L_m - 1$). Given that initial guess, the refined optical flow is computed at level $L_m - 1$, and the result is propagated to level $L_m - 2$ and so on up to the level 0 (the original image).

Let us now describe the recursive operation between two generics levels $L+1$ and L in more mathematical details. Assume that an initial guess for optical flow at level L, $\mathbf{g}^L = [g_x^L\ \ g_y^L]^T$ is available from the computations done from level L_m to level $L+1$. Then, in order to compute the optical flow at level L, it is necessary to find the residual pixel displacement vector $\mathbf{d}^L = [d_x^L\ \ d_y^L]^T$ that minimizes the new image matching error function ϵ^L:

$$\epsilon^L(\mathbf{d}^L) = \epsilon^L(d_x^L, d_y^L) = \sum_{x=u_x^L-\omega_x}^{u_x^L+\omega_x} \sum_{y=u_y^L-\omega_y}^{u_y^L+\omega_y} \left(I^L(x,y) - J^L(x+g_x^L+d_x^L, y+g_y^L+d_y^L)\right)^2. \tag{6}$$

Observe that the window of integration is of constant size $(2\omega_x + 1) \times (2\omega_y + 1)$ for all values of L. Notice that the initial guess flow vector $\mathbf{g}^L$ is used to pre-translate the image patch in the second image J. That way, the residual flow vector $\mathbf{d}^L = [d_x^L\ \ d_y^L]^T$ is small and therefore easy to compute through a standard Lucas Kanade step.

The details of computation of the residual optical flow $\mathbf{d}^L$ will be described in the next section 2.3. For now, let us assume that this vector is computed (to close the main loop of the algorithm). Then, the result of this computation is propagated to the next level $L-1$ by passing the new initial guess $\mathbf{g}^{L-1}$ of expression:

$$\mathbf{g}^{L-1} = 2\,(\mathbf{g^L} + \mathbf{d}^L). \tag{7}$$

The next level optical flow residual vector $\mathbf{d}^{L-1}$ is then computed through the same procedure. This vector, computed by optical flow computation (decribed in Section 2.3), minimizes the functional $\epsilon^{L-1}(\mathbf{d}^{L-1})$ (equation 6). This procedure goes on until the finest image resolution is reached ($L = 0$). The algorithm is initialized by setting the initial guess for level L_m to zero (no initial guess is available at the deepest level of the pyramid):

$$\mathbf{g}^{L_m} = [0 \;\; 0]^T. \tag{8}$$

The final optical flow solution $\mathbf{d}$ (refer to equation 1) is then available after the finest optical flow computation:

$$\mathbf{d} = \mathbf{g^0} + \mathbf{d}^0. \tag{9}$$

Observe that this solution may be expressed in the following extended form:

$$\mathbf{d} = \sum_{L=0}^{L_m} 2^L\, \mathbf{d}^L. \tag{10}$$

The clear advantage of a pyramidal implementation is that each residual optical flow vector $\mathbf{d}^L$ can be kept very small while computing a large overall pixel displacement vector $\mathbf{d}$. Assuming that each elementary optical flow computation step can handle pixel motions up to $d_{\max}$, then the overall pixel motion that the pyramidal implementation can handle becomes $d_{\max\text{ final}} = (2^{L_m+1} - 1)\, d_{\max}$. For example, for a pyramid depth of $L_m = 3$, this means a maximum pixel displacement gain of 15! This enables large pixel motions, while keeping the size of the integration window relatively small.

2.3 Iterative Optical Flow Computation (Iterative Lucas-Kanade)

Let us now describe the core optical flow computation. At every level L in the pyramid, the goal is finding the vector $\mathbf{d}^L$ that minimizes the matching function ϵ^L defined in equation 6. Since the same type of operation is performed for all levels L, let us now drop the superscripts L and define the new images A and B as follows:

$\forall (x,y) \in [p_x - \omega_x - 1, p_x + \omega_x + 1] \times [p_y - \omega_y - 1, p_y + \omega_y + 1]$,

$$A(x,y) \doteq I^L(x,y), \tag{11}$$

$\forall (x,y) \in [p_x - \omega_x, p_x + \omega_x] \times [p_y - \omega_y, p_y + \omega_y]$,

$$B(x,y) \doteq J^L(x + g_x^L, y + g_y^L). \tag{12}$$

Observe that the domains of definition of $A(x,y)$ and $B(x,y)$ are slightly different. Indeed, $A(x,y)$ is defined over a window of size $(2\omega_x+3)\times(2\omega_y+3)$ instead of $(2\omega_x+1)\times(2\omega_y+1)$. This difference will become clear when computing spatial derivatives of $A(x,y)$ using the central difference operator (eqs. 19 and 20). For clarity purposes, let us change the name of the displacement vector $\overline{\nu} = [\nu_x \;\; \nu_y]^T = \mathbf{d}^L$, as well as the image position vector $\mathbf{p} = [p_x \;\; p_y]^T = \mathbf{u}^L$. Following that new notation, the goal is to find the displacement vector $\overline{\nu} = [\nu_x \;\; \nu_y]^T$ that minimizes the matching function:

$$\varepsilon(\overline{\nu}) = \varepsilon(\nu_x, \nu_y) = \sum_{x=p_x-\omega_x}^{p_x+\omega_x} \sum_{y=p_y-\omega_y}^{p_y+\omega_y} \left(A(x,y) - B(x+\nu_x, y+\nu_y)\right)^2. \tag{13}$$

A standard iterative Lucas-Kanade may be applied for that task. At the optimimum, the first derivative of ε with respect to $\overline{\nu}$ is zero:

$$\left.\frac{\partial \varepsilon(\overline{\nu})}{\partial \overline{\nu}}\right|_{\overline{\nu}=\overline{\nu}_{\text{opt}}} = [0 \;\; 0]. \tag{14}$$

After expansion of the derivative, we obtain:

$$\frac{\partial \varepsilon(\overline{\nu})}{\partial \overline{\nu}} = -2 \sum_{x=p_x-\omega_x}^{p_x+\omega_x} \sum_{y=p_y-\omega_y}^{p_y+\omega_y} \left(A(x,y) - B(x+\nu_x, y+\nu_y)\right) . \begin{bmatrix} \frac{\partial B}{\partial x} & \frac{\partial B}{\partial y} \end{bmatrix}. \tag{15}$$

Let us now substitute $B(x+\nu_x, y+\nu_y)$ by its first order Taylor expansion about the point $\overline{\nu} = [0\ \ 0]^T$ (this has a good chance to be a valid approximation since we are expecting a small displacement vector, thanks to the pyramidal scheme):

$$\frac{\partial \varepsilon(\overline{\nu})}{\partial \overline{\nu}} \approx -2 \sum_{x=p_x-\omega_x}^{p_x+\omega_x} \sum_{y=p_y-\omega_y}^{p_y+\omega_y} \left(A(x,y) - B(x,y) - \begin{bmatrix} \frac{\partial B}{\partial x} & \frac{\partial B}{\partial y} \end{bmatrix} \overline{\nu} \right) \cdot \begin{bmatrix} \frac{\partial B}{\partial x} & \frac{\partial B}{\partial y} \end{bmatrix}. \tag{16}$$

Observe that the quatity $A(x,y) - B(x,y)$ can be interpreted as the temporal image derivative at the point $[x\ \ y]^T$:

$\forall (x,y) \in [p_x - \omega_x, p_x + \omega_x] \times [p_y - \omega_y, p_y + \omega_y]$,

$$\delta I(x,y) \doteq A(x,y) - B(x,y). \tag{17}$$

The matrix $\begin{bmatrix} \frac{\partial B}{\partial x} & \frac{\partial B}{\partial y} \end{bmatrix}$ is merely the image gradient vector. Let's make a slight change of notation:

$$\nabla I = \begin{bmatrix} I_x \\ I_y \end{bmatrix} \doteq \begin{bmatrix} \frac{\partial B}{\partial x} & \frac{\partial B}{\partial y} \end{bmatrix}^T. \tag{18}$$

Observe that the image derivatives I_x and I_y may be computed directly from the first image $A(x,y)$ in the $(2\omega_x + 1) \times (2\omega_y + 1)$ neighborhood of the point $\mathbf{p}$ independently from the second image $B(x,y)$ (the importance of this observation will become apparent later on when describing the iterative version of the flow computation). If a central difference operator is used for derivative, the two derivative images have the following expression:

$\forall (x,y) \in [p_x - \omega_x, p_x + \omega_x] \times [p_y - \omega_y, p_y + \omega_y]$,

$$I_x(x,y) = \frac{\partial A(x,y)}{\partial x} = \frac{A(x+1,y) - A(x-1,y)}{2}, \tag{19}$$

$$I_y(x,y) = \frac{\partial A(x,y)}{\partial y} = \frac{A(x,y+1) - A(x,y-1)}{2}. \tag{20}$$

In practice, the Sharr operator is used for computing image derivatives (very similar to the central difference operator). Following this new notation, equation 16 may be written:

$$\frac{1}{2}\frac{\partial \varepsilon(\overline{\nu})}{\partial \overline{\nu}} \approx \sum_{x=p_x-\omega_x}^{p_x+\omega_x} \sum_{y=p_y-\omega_y}^{p_y+\omega_y} \left(\nabla I^T \overline{\nu} - \delta I \right) \nabla I^T, \tag{21}$$

$$\frac{1}{2}\left[\frac{\partial \varepsilon(\overline{\nu})}{\partial \overline{\nu}}\right]^T \approx \sum_{x=p_x-\omega_x}^{p_x+\omega_x} \sum_{y=p_y-\omega_y}^{p_y+\omega_y} \left(\begin{bmatrix} I_x^2 & I_x I_y \\ I_x I_y & I_y^2 \end{bmatrix} \overline{\nu} - \begin{bmatrix} \delta I\, I_x \\ \delta I\, I_y \end{bmatrix} \right). \tag{22}$$

Denote

$$G \doteq \sum_{x=p_x-\omega_x}^{p_x+\omega_x} \sum_{y=p_y-\omega_y}^{p_y+\omega_y} \begin{bmatrix} I_x^2 & I_x I_y \\ I_x I_y & I_y^2 \end{bmatrix} \quad \text{and} \quad \overline{b} \doteq \sum_{x=p_x-\omega_x}^{p_x+\omega_x} \sum_{y=p_y-\omega_y}^{p_y+\omega_y} \begin{bmatrix} \delta I\, I_x \\ \delta I\, I_y \end{bmatrix}. \tag{23}$$

Then, equation 22 may be written:

$$\frac{1}{2}\left[\frac{\partial \varepsilon(\overline{\nu})}{\partial \overline{\nu}}\right]^T \approx G\,\overline{\nu} - \overline{b}. \tag{24}$$

Therefore, following equation 14, the optimimum optical flow vector is

$$\overline{\nu}_{\text{opt}} = G^{-1}\,\overline{b}. \tag{25}$$

This expression is valid only if the matrix G is invertible. That is equivalent to saying that the image $A(x,y)$ contains gradient information in both x and y directions in the neighborhood of the point $\mathbf{p}$.

This is the standard Lucas-Kanade optical flow equation, which is valid only if the pixel displacement is small (in order ot validate the first order Taylor expansion). In practice, to get an accurate solution, is is necessary to iterate multiple times on this scheme (in a Newton-Raphson fashion).

Now that we have introduced the mathematical background, let us give the details of the iterative version of the algorithm. Recall the goal: find the vector $\overline{\nu}$ that minimizes the error functional $\varepsilon(\overline{\nu})$ introduced in equation 13.

Let k be the iterative index, initialized to 1 at the very first iteration. Let us describe the algorithm recursively: at a generic iteration $k \geq 1$, assume that the previous computations from iterations $1, 2, \ldots, k-1$ provide an initial guess $\overline{\nu}^{k-1} = [\nu_x^{k-1} \;\; \nu_x^{k-1}]^T$ for the pixel displacement $\overline{\nu}$. Let B_k be the new translated image according to that initial guess $\overline{\nu}^{k-1}$:

$\forall (x, y) \in [p_x - \omega_x, p_x + \omega_x] \times [p_y - \omega_y, p_y + \omega_y]$,

$$B_k(x, y) = B(x + \nu_x^{k-1}, y + \nu_y^{k-1}). \tag{26}$$

The goal is then to compute the residual pixel motion vector $\overline{\eta}^k = [\eta_x^k \;\; \eta_y^k]$ that minimizes the error function

$$\varepsilon^k(\overline{\eta}^k) = \varepsilon(\eta_x^k, \eta_y^k) = \sum_{x=p_x-\omega_x}^{p_x+\omega_x} \sum_{y=p_y-\omega_y}^{p_y+\omega_y} \left(A(x, y) - B_k(x + \eta_x^k, y + \eta_y^k)\right)^2. \tag{27}$$

The solution of this minimization may be computed through a one step Lucas-Kanade optical flow computation (equation 25)

$$\overline{\eta}^k = G^{-1}\, \overline{b}_k, \tag{28}$$

where the 2×1 vector $\overline{b}_k$ is defined as follows (also called image mismatch vector):

$$\overline{b}_k = \sum_{x=p_x-\omega_x}^{p_x+\omega_x} \sum_{y=p_y-\omega_y}^{p_y+\omega_y} \begin{bmatrix} \delta I_k(x, y)\, I_x(x, y) \\ \delta I_k(x, y)\, I_y(x, y) \end{bmatrix}, \tag{29}$$

where the kth image difference δI_k are defined as follows:

$\forall (x, y) \in [p_x - \omega_x, p_x + \omega_x] \times [p_y - \omega_y, p_y + \omega_y]$,

$$\delta I_k(x, y) = A(x, y) - B_k(x, y). \tag{30}$$

Observe that the spatial derivatives I_x and I_y (at all points in the neighborhood of $\overline{p}$) are computed only once at the beginning of the iterations following equations 19 and 20. Therefore the 2×2 matrix G also remains constant throughout the iteration loop (expression given in equation 23). That constitutes a clear computational advantage. The only quantity that needs to be recomputed at each step k is the vector $\overline{b}_k$ that really captures the amount of residual difference between the image patches after translation by the vector $\overline{\nu}^{k-1}$. Once the residual optical flow $\overline{\eta}^k$ is computed through equation 28, a new pixel displacement guess $\overline{\nu}^k$ is computed for the next iteration step $k+1$:

$$\overline{\nu}^k = \overline{\nu}^{k-1} + \overline{\eta}^k. \tag{31}$$

The iterative scheme goes on until the computed pixel residual $\overline{\eta}^k$ is smaller than a threshold (for example 0.03 pixel), or a maximum number of iteration (for example 20) is reached. On average, 5 iterations are enough to reach convergence. At the first iteration ($k = 1$) the initial guess is initialized to zero:

$$\overline{\nu}^0 = [0 \;\; 0]^T. \tag{32}$$

Assuming that K iterations were necessary to reach convergence, the final solution for the optical flow vector $\overline{\nu} = \mathbf{d}^L$ is:

$$\overline{\nu} = \mathbf{d}^L = \overline{\nu}^K = \sum_{k=1}^{K} \overline{\eta}^k. \tag{33}$$

This vector minimizes the error functional described in equation 13 (or equation 6). This ends the description of the iterative Lucas-Kanade optical flow computation. The vector $\mathbf{d}^L$ is fed to equation 7 and this overall procedure is repeated at all subsequent levels $L-1, L-2, \ldots, 0$ (see section 2.2).

2.4 Summary of the pyramidal tracking algorithm

Let is now summarize the entire tracking algorithm in a form of a pseudo-code. Find the details of the equations in the main body of the text (especially for the domains of definition).

Goal: Let $\mathbf{u}$ be a point on image I. Find its corresponding location $\mathbf{v}$ on image J

Build pyramid representations of I and J: $\{I^L\}_{L=0,\ldots,L_m}$ and $\{J^L\}_{L=0,\ldots,L_m}$ (eqs. 2,3,4)

Initialization of pyramidal guess: $\mathbf{g}^{L_m} = [g_x^{L_m} \;\; g_x^{L_m}]^T = [0 \;\; 0]^T$ (eq. 8)

for $L = L_m$ **down to** 0 **with step of** -1

Location of point $\mathbf{u}$ on image I^L: $\mathbf{u}^L = [p_x \;\; p_y]^T = \mathbf{u}/2^L$ (eq. 5)

Derivative of I^L with respect to x: $I_x(x,y) = \dfrac{I^L(x+1,y) - I^L(x-1,y)}{2}$ (eqs. 19,11)

Derivative of I^L with respect to y: $I_y(x,y) = \dfrac{I^L(x,y+1) - I^L(x,y-1)}{2}$ (eqs. 20,11)

Spatial gradient matrix: $G = \displaystyle\sum_{x=p_x-\omega_x}^{p_x+\omega_x} \sum_{y=p_y-\omega_y}^{p_y+\omega_y} \begin{bmatrix} I_x^2(x,y) & I_x(x,y)\, I_y(x,y) \\ I_x(x,y)\, I_y(x,y) & I_y^2(x,y) \end{bmatrix}$ (eq. 23)

Initialization of iterative L-K: $\overline{\nu}^0 = [0 \;\; 0]^T$ (eq. 32)

for $k = 1$ **to** K **with step of** 1 (or until $\|\overline{\eta}^k\| <$ accuracy threshold)

Image difference: $\delta I_k(x,y) = I^L(x,y) - J^L(x + g_x^L + \nu_x^{k-1}, y + g_y^L + \nu_y^{k-1})$ (eqs. 30,26,12)

Image mismatch vector: $\overline{b}_k = \displaystyle\sum_{x=p_x-\omega_x}^{p_x+\omega_x} \sum_{y=p_y-\omega_y}^{p_y+\omega_y} \begin{bmatrix} \delta I_k(x,y)\, I_x(x,y) \\ \delta I_k(x,y)\, I_y(x,y) \end{bmatrix}$ (eq. 29)

Optical flow (Lucas-Kanade): $\overline{\eta}^k = G^{-1}\, \overline{b}_k$ (eq. 28)

Guess for next iteration: $\overline{\nu}^k = \overline{\nu}^{k-1} + \overline{\eta}^k$ (eq. 31)

end of for-loop on k

Final optical flow at level L: $\mathbf{d}^L = \overline{\nu}^K$ (eq. 33)

Guess for next level $L-1$: $\mathbf{g}^{L-1} = [g_x^{L-1} \;\; g_y^{L-1}]^T = 2\,(\mathbf{g^L} + \mathbf{d}^L)$ (eq. 7)

end of for-loop on L

Final optical flow vector: $\mathbf{d} = \mathbf{g}^0 + \mathbf{d}^0$ (eq. 9)

Location of point on J: $\mathbf{v} = \mathbf{u} + \mathbf{d}$

Solution: The corresponding point is at location $\mathbf{v}$ on image J

2.5 Subpixel Computation

It is absolutely essential to keep all computation at a subpixel accuracy level. It is therefore necessary to be able to compute image brightness values at locations between integer pixels (refer for example to equations 11,12 and 26). In order to compute image brightness at subpixel locations we propose to use bilinear interpolation.

Let L be a generic pyramid level. Assume that we need the image value $I^L(x, y)$ where x and y are not integers. Let x_o and y_o be the integer parts of x and y (larger integers that are smallers than x and y). Let α_x and α_y be the two reminder values (between 0 and 1) such that:

$$x = x_o + \alpha_x, \tag{34}$$

$$y = y_o + \alpha_y. \tag{35}$$

Then $I^L(x, y)$ may be computed by bilinear interpolation from the original image brightness values:

$$\begin{aligned} I^L(x,y) = \; & (1-\alpha_x)\,(1-\alpha_y)\,I^L(x_o,y_o) + \alpha_x\,(1-\alpha_y)\,I^L(x_o+1,y_o) + \\ & (1-\alpha_x)\,\alpha_y\,I^L(x_o,y_o+1) + \alpha_x\,\alpha_y\,I^L(x_o+1,y_o+1). \end{aligned}$$

Let us make a few observations that are associated to subpixel computation (important implementation issues). Refer to the summary of the algorithm given in section 2.4. When computing the two image derivatives $I_x(x, y)$ and $I_y(x, y)$ in the neigborhood $(x, y) \in [p_x - \omega_x, p_x + \omega_x] \times [p_y - \omega_y, p_y + \omega_y]$, it is necessary to have the brightness values of $I^L(x, y)$ in the neighborhood $(x, y) \in [p_x - \omega_x - 1, p_x + \omega_x + 1] \times [p_y - \omega_y - 1, p_y + \omega_y + 1]$ (see equations 19, 20). Of course, the coordinates of the central point $\mathbf{p} = [p_x \;\; p_y]^T$ are not garanteed to be integers. Call p_{x_o} and p_{y_o} the integer parts of p_x and p_y. Then we may write:

$$p_x = p_{x_o} + p_{x_\alpha}, \tag{36}$$

$$p_y = p_{y_o} + p_{y_\alpha}, \tag{37}$$

where p_{x_α} and p_{y_α} are the associated reminder values between 0 and 1. Therefore, in order to compute the image patch $I^L(x, y)$ in the neighborhood $(x, y) \in [p_x - \omega_x - 1, p_x + \omega_x + 1] \times [p_y - \omega_y - 1, p_y + \omega_y + 1]$ through bilinear interpolation, it is necessary to use the set of original brightness values $I^L(x, y)$ in the integer grid patch $(x, y) \in [p_{x_o} - \omega_x - 1, p_{x_o} + \omega_x + 2] \times [p_{y_o} - \omega_y - 1, p_{y_o} + \omega_y + 2]$ (recall that ω_x and ω_y are integers).

A similar situation occurs when computing the image difference $\delta I_k(x, y)$ in the neigborhood $(x, y) \in [p_x - \omega_x, p_x + \omega_x] \times [p_y - \omega_y, p_y + \omega_y]$ (refer to section 2.4). Indeed, in order to compute $\delta I_k(x, y)$, it is required to have the values $J^L(x + g^L_x + \nu^{k-1}_x, y + g^L_y + \nu^{k-1}_y)$ for all $(x, y) \in [p_x - \omega_x, p_x + \omega_x] \times [p_y - \omega_y, p_y + \omega_y]$, or, in other words, the values of $J^L(\alpha, \beta)$ for all $(\alpha, \beta) \in [p_x + g^L_x + \nu^{k-1}_x - \omega_x, p_x + g^L_x + \nu^{k-1}_x + \omega_x] \times [p_y + g^L_y + \nu^{k-1}_y - \omega_y, p_y + g^L_y + \nu^{k-1}_y + \omega_y]$. Of course, $p_x + g^L_x + \nu^{k-1}_x$ and $p_y + g^L_y + \nu^{k-1}_y$ are not necessarily integers. Call q_{x_o} and q_{y_o} the integer parts of $p_x + g^L_x + \nu^{k-1}_x$ and $p_y + g^L_y + \nu^{k-1}_y$:

$$p_x + g^L_x + \nu^{k-1}_x = q_{x_o} + q_{x_\alpha}, \tag{38}$$

$$p_y + g^L_y + \nu^{k-1}_y = q_{y_o} + q_{y_\alpha}, \tag{39}$$

where q_{x_α} and q_{y_α} are the associated reminder values between 0 and 1. Then, in order to compute the image patch $J^L(\alpha, \beta)$ in the neighborhood $(\alpha, \beta) \in [p_x+g^L_x+\nu^{k-1}_x-\omega_x, p_x+g^L_x+\nu^{k-1}_x+\omega_x]\times[p_y+g^L_y+\nu^{k-1}_y-\omega_y, p_y+g^L_y+\nu^{k-1}_y+\omega_y]$, it is necessary to use the set of original brightness values $J^L(\alpha, \beta)$ in the integer grid patch $(\alpha, \beta) \in [q_{x_o} - \omega_x, q_{x_o} + \omega_x + 1] \times [q_{y_o} - \omega_y, q_{y_o} + \omega_y + 1]$.

2.6 Tracking features close to the boundary of the images

It is very useful to observe that it is possible to process points that are close enough to the image boundary to have a portion of their integration window outside the image. This observation becomes very significant as the the number of pyramid levels L_m is large. Indeed, if we always enforce the complete $(2\omega_x + 1) \times (2\omega_y + 1)$ window to be within the image in order to be tracked, then there is "forbidden band" of width ω_x (and ω_y) all around the images I^L. If L_m is the height of the pyramid, this means an effective forbidden band of width $2^{L_m}\,\omega_x$ (and $2^{L_m}\,\omega_y$) around the original image I. For small values of ω_x, ω_y and L_m, this might not constitute a significant limitation, howerver, this may become very troublesome for large integration window sizes, and more importantly, for large values of L_m. Some numbers: $\omega_x = \omega_y = 5$ pixels, and $L_m = 3$ leads to a forbidden band of 40 pixels around the image!

In order to prevent this from happenning, we propose to keep tracking points whose integration windows partially fall outside the image (at any pyramid level). In this case, the tracking procedure reamins the same, expect that the summations appearing in all expressions (see pseudo-code of section 2.4) should be done only over the valid portion of the image neigborhood, i.e. the portion of the neighborhood that have valid entries for $I_x(x, y)$, $I_y(x, y)$ and $\delta I_k(x, y)$

(see section 2.4). Observe that doing so, the valid summation regions may vary while going through the Lucas-Kanade iteration loop (loop over the index k in the pseudo-code - Sec. 2.4). Indeed, from iteration to iteration, the valid entry of the the image difference $\delta I_k(x,y)$ may vary as the translation vector $[g_x^L + \nu_x^{k-1} \quad g_y^L + \nu_y^{k-1}]^T$ vary. In addition, observe that when computing the mismatch vector $\overline{b}_k$ and the gradient matrix G, the summations regions must be identical (for the mathematics to remain valid). Therefore, in that case, the G matrix must be recomputed within the loop at each iteration. The differential patches $I_x(x,y)$ and $I_y(x,y)$ may however be computed once before the iteration loop.

Of course, if the point $\mathbf{p} = [p_x \quad p_y]^T$ (the center of the neighborhood) falls outside of the image I^L, or if its corresponding tracked point $[p_x + g_x^L + \nu_x^{k-1} \quad p_y + g_y^L + \nu_y^{k-1}]$ falls outside of the image J^L, it is reasonable to declare the point "lost", and not pursue tracking for it.

2.7 Declaring a feature "lost"

There are two cases that should give rise to a "lost" feature. The first case is very intuitive: the point falls outside of the image. We have discussed this issue in section 2.6. The other case of loss is when the image patch around the tracked point varies too much between image I and image J (the point disappears due to occlusion). Observe that this condition is a lot more challenging to quantify precisely. For example, a feature point may be declared "lost" if its final cost function $\epsilon(\mathbf{d})$ is larger than a threshold (see equation 1). A problem comes in when having decide about a threshold. It is particularly sensitive when tracking a point over many images in a complete sequence. Indeed, if tracking is done based on consecutive pairs of images, the tracked image patch (used as reference) is implicitely initialized at every track. Consequently, the point may drift throughout the extended sequence even if the image difference between two consecutive images is very small. This drift problem is a very classical issue when dealing with long sequences. One approach is to track feature points through a sequence while keeping a fixed reference for the appearance of the feature patch (use the first image the feature appeared). Following this technique, the quantity $\epsilon(\mathbf{d})$ has a lot more meaning. While adopting this approach however another problem rises: a feature may be declared "lost" too quickly. One direction that we envision to answer that problem is to use affine image matching for deciding for lost track (see Shi and Tomasi in [1]).

The best technique known so far is to combine a traditional tracking approach presented so far (based on image pairs) to compute matching, with an affine image matching (using a unique reference for feature patch) to decide for false track. More information regarding this technique is presented by Shi and Tomasi in [1]. We believe that eventually, this approach should be implemented for rejection. It is worth observing that for 2D tracking itself, the standard tracking scheme presented in this report performs a lot better (more accurate) that affine tracking alone. The reason is that affine tracking requires to estimate a very large number of parameters: 6 instead of 2 for the standard scheme. Many people have made that observation (see for example `http://www.stanford.edu/ ssorkin/cs223b/final.html`). Therefore, affine tracking should only be considered for building a reliable rejection scheme on top of the main 2D tracking engine.

3 Feature Selection

So far, we have described the tracking procedure that takes care of following a point $\mathbf{u}$ on an image I to another location $\mathbf{v}$ on another image J. However, we have not described means to select the point $\mathbf{u}$ on I in the first place. This step is called feature selection. It is very intuitive to approach the problem of feature selection once the mathematical ground for tracking is led out. Indeed, the central step of tracking is the computation of the optical flow vector $\overline{\eta}^k$ (see pseudo-code of algorithm in section 2.4). At that step, the G matrix is required to be invertible, or in other words, the minimum eigenvalue of G must be large enough (larger than a threshold). This characterizes pixels that are "easy to track".

Therefore, the process of selection goes as follows:

1. Compute the G matrix and its minimum eigenvalue λ_m at every pixel in the image I.
2. Call $\lambda_{\max}$ the maximum value of λ_m over the whole image.
3. Retain the image pixels that have a λ_m value larger than a percentage of $\lambda_{\max}$. This percentage can be 10% or 5%.
4. From those pixels, retain the local max. pixels (a pixel is kept if its λ_m value is larger than that of any other pixel in its 3×3 neighborhood).
5. Keep the subset of those pixels so that the minimum distance between any pair of pixels is larger than a given threshold distance (e.g. 10 or 5 pixels).

After that process, the remaining pixels are typically "good to track". They are the selected feature points that are fed to the tracker.

The last step of the algorithm consisting of enforcing a minimum pairwise distance between pixels may be omited if a very large number of points can be handled by the tracking engine. It all depends on the computational performances of the feature tracker.

Finally, it is important to observe that it is not necessary to take a very large integration window for feature selection (in order to compute the G matrix). In fact, a 3×3 window is sufficient $\omega_x = \omega_x = 1$ for selection, and should be used. For tracking, this window size (3×3) is typically too small (see section 1).

References

[1] Jianbo Shi and Carlo Tomasi, "Good features to track", *Proc. IEEE Comput. Soc. Conf. Comput. Vision and Pattern Recogn.*, pages 593–600, 1994.
