# Supplementary material for: Rapid whole brain imaging of neural activity in freely behaving larval zebrafish (Danio rerio)
Source: eLife. 2017 Sep 20;6:e28158. doi: 10.7554/eLife.28158 (PMC5644961; doi:10.7554/eLife.28158)
Supplement: Source code 3. [file elife-28158-code3.zip › RT analysis code/OpenCV1.1pre.zip!/OpenCV/docs/papers/camshift.pdf]

Computer Vision Face Tracking For Use in a Perceptual User Interface

Gary R. Bradski, Microcomputer Research Lab, Santa Clara, CA, Intel Corporation

Index words: computer vision, face tracking, mean shift algorithm, perceptual user interface, 3D graphics interface

Abstract

As a first step towards a perceptual user interface, a computer vision color tracking algorithm is developed and applied towards tracking human faces. Computer vision algorithms that are intended to form part of a perceptual user interface must be fast and efficient. They must be able to track in real time yet not absorb a major share of computational resources: other tasks must be able to run while the visual interface is being used. The new algorithm developed here is based on a robust non-parametric technique for climbing density gradients to find the mode (peak) of probability distributions called the mean shift algorithm. In our case, we want to find the mode of a color distribution within a video scene. Therefore, the mean shift algorithm is modified to deal with dynamically changing color probability distributions derived from video frame sequences. The modified algorithm is called the Continuously Adaptive Mean Shift (CAMSHIFT) algorithm. CAMSHIFT's tracking accuracy is compared against a Polhemus tracker. Tolerance to noise, distractors and performance is studied.

CAMSHIFT is then used as a computer interface for controlling commercial computer games and for exploring immersive 3D graphic worlds.

Introduction

This paper is part of a program to develop a Perceptual User Interface for computers. Perceptual interfaces are ones in which the computer is given the ability to sense and produce analogs of the human senses, such as allowing computers to perceive and produce localized sound and speech, giving computers a sense of touch and force feedback, and in our case, giving computers an ability to see. The work described in this paper is part of a larger effort aimed at giving computers the ability to segment, track, and understand the pose, gestures, and emotional expressions of humans and the tools they might be using in front of a computer or settop box. In this paper we describe the development of the first core module in this effort: a 4-degree of freedom color object tracker and its application to flesh-tone-based face tracking.

Computer vision face tracking is an active and developing field, yet the face trackers that have been developed are not sufficient for our needs. Elaborate methods such as tracking contours with snakes [[10][12][13]], using Eigenspace matching techniques [14], maintaining large sets of statistical hypotheses [15], or convolving images with feature detectors [16] are far too computationally expensive. We want a tracker that will track a given face in the presence of noise, other faces, and hand movements. Moreover, it must run fast and efficiently so that objects may be tracked in real time (30 frames per second) while consuming as few system resources as possible. In other words, this tracker should be able to serve as *part* of a user interface that is in turn *part* of the computational tasks that a computer might routinely be expected to carry out. This tracker also needs to run on inexpensive consumer cameras and not require calibrated lenses.

In order, therefore, to find a fast, simple algorithm for basic tracking, we have focused on color-based tracking [[7][8][9][10][11]], yet even these simpler algorithms are too computationally complex (and therefore slower at any given CPU speed) due to their use of color correlation, blob and region growing, Kalman filter smoothing and prediction, and contour considerations. The complexity of the these algorithms derives from their attempts to deal with irregular object motion due to perspective (near objects to the camera seem to move faster than distal objects); image noise; distractors, such as other faces in the scene; facial occlusion by hands or other objects; and lighting variations. We want a fast, computationally efficient algorithm that handles these problems in the course of its operation, i.e., an algorithm that mitigates the above problems "for free."

To develop such an algorithm, we drew on ideas from robust statistics and probability distributions. Robust statistics are those that tend to ignore outliers in the data (points far away from the region of interest). Thus, robust

algorithms help compensate for noise and distractors in the vision data. We therefore chose to use a robust non-parametric technique for climbing density gradients to find the mode of probability distributions called the mean shift algorithm [2]. (The mean shift algorithm was never intended to be used as a tracking algorithm, but it is quite effective in this role.)

The mean shift algorithm operates on probability distributions. To track colored objects in video frame sequences, the color image data has to be represented as a probability distribution [1]; we use color histograms to accomplish this. Color distributions derived from video image sequences change over time, so the mean shift algorithm has to be modified to adapt dynamically to the probability distribution it is tracking. The new algorithm that meets all these requirements is called CAMSHIFT.

For face tracking, CAMSHIFT tracks the X, Y, and Area of the flesh color probability distribution representing a face. Area is proportional to Z, the distance from the camera. Head roll is also tracked as a further degree of freedom. We then use the X, Y, Z, and Roll derived from CAMSHIFT face tracking as a perceptual user interface for controlling commercial computer games and for exploring 3D graphic virtual worlds.

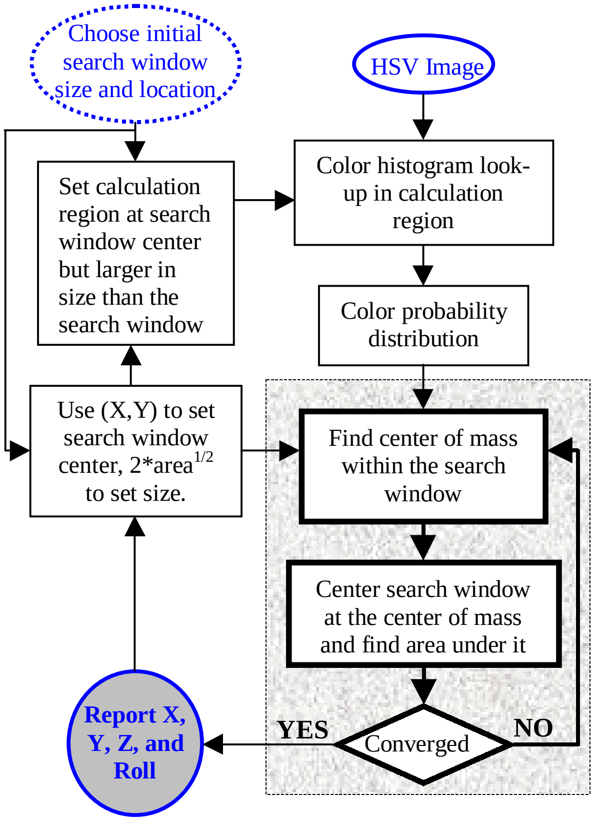

Figure 1: Block diagram of color object tracking

Figure 1 summarizes the algorithm described below. For each video frame, the raw image is converted to a color probability distribution image via a color histogram model of the color being tracked (flesh for face tracking). The center and size of the color object are found via the CAMSHIFT algorithm operating on the color probability image (the gray box is the mean shift algorithm). The current size and location of the tracked object are reported and used to set the size and location of the search window in the next video image. The process is then repeated for continuous tracking.

Video Demonstrations

The following three videos demonstrate CAMSHIFT in action.

1. FaceTrack_Fast.avi
2. FaceTrack_Distractors.avi
3. FaceTrack_HandOcclusion.avi

The first video shows CAMSHIFT tracking rapid face movements. The second video shows CAMSHIFT tracking a face with other faces moving in the scene. The third video shows CAMSHIFT tracking a face through hand occlusions. These videos are available from this paper on the Web in the *Intel Technology Journal Q2'98* under the site http://developer.intel.com/technology/itj.

Color Probability Distributions

In order to use CAMSHIFT to track colored objects in a video scene, a probability distribution image of the desired color (flesh color in the case of face tracking) in the video scene must be created. In order to do this, we first create a model of the desired hue using a color histogram. We use the Hue Saturation Value (HSV) color system [5][6] that corresponds to projecting standard Red, Green, Blue (RGB) color space along its principle diagonal from white to black (see arrow in Figure 2). This results in the hexcone in Figure 3. Descending the V axis in Figure 3 gives us smaller hexcones corresponding to smaller (darker) RGB subcubes in Figure 2.

HSV space separates out hue (color) from saturation (how concentrated the color is) and from brightness. We create our color models by taking 1D histograms from the H (hue) channel in HSV space.

For face tracking via a flesh color model, flesh areas from the user are sampled by prompting users to center their face in an onscreen box, or by using motion cues to find flesh areas from which to sample colors. The hues derived from flesh pixels in the image are sampled from the H channel and binned into an 1D histogram. When sampling is complete, the histogram is saved for future use. More robust histograms may be made by sampling flesh hues

from multiple people. Even simple flesh histograms tend to work well with a wide variety of people without having to be updated. A common misconception is that different color models are needed for different races of people, for example, for blacks and whites. This is not true. Except for albinos, humans are all the same color (hue). Dark-skinned people simply have greater flesh color saturation than light-skinned people, and this is separated out in the HSV color system and ignored in our flesh-tracking color model.

During operation, the stored flesh color histogram is used as a model, or lookup table, to convert incoming video pixels to a corresponding probability of flesh image as can be seen in the right-hand image of Figure 6. This is done for each video frame. Using this method, probabilities range in discrete steps from zero (probability 0.0) to the maximum probability pixel value (probability 1.0). For 8-bit hues, this range is between 0 and 255. We then track using CAMSHIFT on this probability of flesh image.

When using real cameras with discrete pixel values, a problem can occur when using HSV space as can be seen in Figure 3. When brightness is low (V near 0), saturation is also low (S near 0). Hue then becomes quite noisy, since in such a small hexcone, the small number of discrete hue pixels cannot adequately represent slight changes in RGB. This then leads to wild swings in hue values. To overcome this problem, we simply ignore hue pixels that have very low corresponding brightness values. This means that for very dim scenes, the camera must auto-adjust or be adjusted for more brightness or else it simply cannot track. With sunlight, bright white colors can take on a flesh hue so we also use an upper threshold to ignore flesh hue pixels with corresponding high brightness. At very low saturation, hue is not defined so we also ignore hue pixels that have very low corresponding saturation (see Implementation Details section below).

Originally, we used a 2D color histogram built from normalized red green (r,g) space (r = R/(R+G+B), g = G/(R+G+B)). However, we found that such color models are much more sensitive to lighting changes since saturation (which is influenced by lighting) is not separated out of that model.

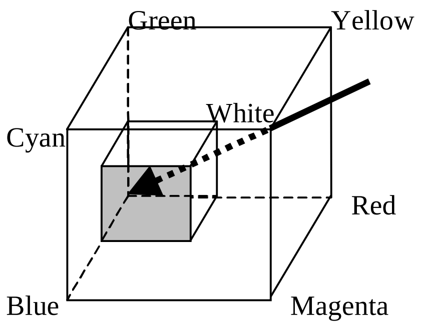

Figure 2: RGB color cube

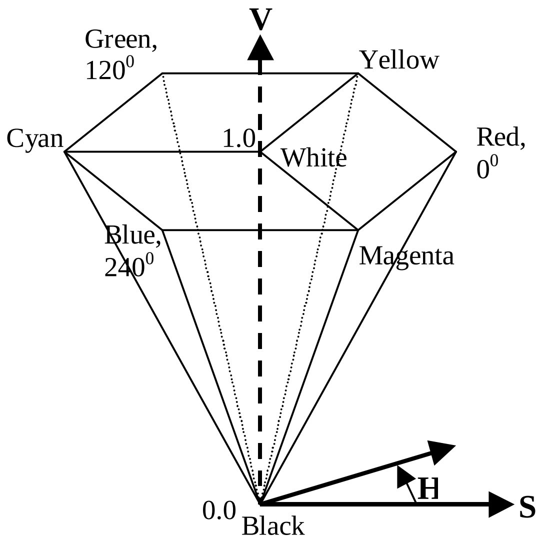

Figure 3: HSV color system

CAMSHIFT Derivation

The closest existing algorithm to CAMSHIFT is known as the mean shift algorithm [2][18]. The mean shift algorithm is a non-parametric technique that climbs the gradient of a probability distribution to find the nearest dominant mode (peak).

How to Calculate the Mean Shift Algorithm

1. Choose a search window size.
2. Choose the initial location of the search window.
3. Compute the mean location in the search window.
4. Center the search window at the mean location computed in Step 3.
5. Repeat Steps 3 and 4 until convergence (or until the mean location moves less than a preset threshold).

Proof of Convergence [18]

Assuming a Euclidean distribution space containing distribution f, the proof is as follows reflecting the steps above:

1. A window *W* is chosen at size *s*.

2. The initial search window is centered at data point p_k
3. Compute the mean position within the search window

$$\hat{\bar{p}}_k(W) = \frac{1}{|W|} \sum_{j \in W} p_j.$$

The mean shift climbs the gradient of *f(p)*

$$\hat{\bar{p}}_k(W) - p_k \approx \frac{f'(p_k)}{f(p_k)}.$$

4. Center the window at point

$$\hat{\bar{p}}_k(W).$$

5. Repeat Steps 3 and 4 until convergence.

Near the mode $f'(p) \cong 0$, so the mean shift algorithm converges there.

For discrete 2D image probability distributions, the mean location (the centroid) within the search window (Steps 3 and 4 above) is found as follows:

Find the zeroth moment

$$M_{00} = \sum_x \sum_y I(x,y).$$

Find the first moment for *x* and *y*

$$M_{10} = \sum_x \sum_y xI(x,y); \; M_{01} = \sum_x \sum_y yI(x,y).$$

Then the mean search window location (the centroid) is

$$x_c = \frac{M_{10}}{M_{00}}; \quad y_c = \frac{M_{01}}{M_{00}};$$

where *I(x,y)* is the pixel (probability) value at position *(x,y)* in the image, and *x* and *y* range over the search window.

Unlike the Mean Shift algorithm, which is designed for static distributions, CAMSHIFT is designed for dynamically changing distributions. These occur when objects in video sequences are being tracked and the object moves so that the size and location of the probability distribution changes in time. The CAMSHIFT algorithm adjusts the search window size in the course of its operation. Initial window size can be set at any reasonable value. For discrete distributions (digital data), the minimum window size is three as explained in the Implementation Details section. Instead of a set or externally adapted window size, CAMSHIFT relies on the zeroth moment information, extracted as part of the internal workings of the algorithm, to continuously adapt its window size within or over each video frame. One can think of the zeroth moment as the distribution "area" found under the search window. Thus, window radius, or height and width, is set to a function of the the zeroth moment found during search. The CAMSHIFT algorithm is then calculated using any initial non-zero window size (greater or equal to three if the distribution is discrete).

How to Calculate the Continuously Adaptive Mean Shift Algorithm

1. Choose the initial location of the search window.
2. Mean Shift as above (one or many iterations); store the zeroth moment.
3. Set the search window size equal to a function of the zeroth moment found in Step 2.
4. Repeat Steps 2 and 3 until convergence (mean location moves less than a preset threshold).

In Figure 4 below, CAMSHIFT is shown beginning the search process at the top left step by step down the left then right columns until convergence at bottom right. In this figure, the red graph is a 1D cross-section of an actual sub-sampled flesh color probability distribution of an image of a face and a nearby hand. In this figure, yellow is the CAMSHIFT search window, and purple is the mean shift point. The ordinate is the distribution value, and the abscissa is the horizontal spatial position within the original image. The window is initialized at size three and converges to cover the tracked face but not the hand in six iterations. In this sub-sampled image, the maximum distribution pixel value is 206 so we set the width of the search window to be *2*M$_0$/206* (see discussion of window size in the Implementation Details section below). In this process, CAMSHIFT exhibits typical behavior: it finds the center of the nearest connected distribution region (the face), but ignores nearby distractors (the hand).

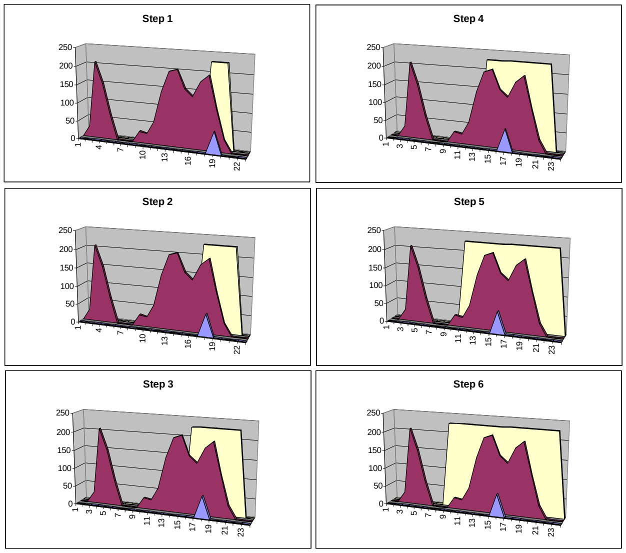

Figure 4: CAMSHIFT in operation down the left then right columns

Figure 4 shows CAMSHIFT at startup. Figure 5 below shows frame to frame tracking. In this figure, the red color probability distribution has shifted left and changed form. At the left in Figure 5, the search window starts at its previous location from the bottom right in Figure 4. In one iteration it converges to the new face center.

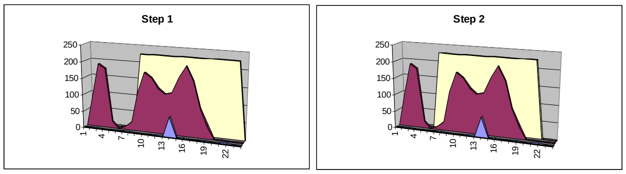

Figure 5: Example of CAMSHIFT tracking starting from the converged search location in Figure 4 bottom right

Mean Shift Alone Does Not Work

The mean shift algorithm alone would fail as a tracker. A window size that works at one distribution scale is not suitable for another scale as the color object moves towards and away from the camera. Small fixed-sized windows may get lost entirely for large object translation in the scene. Large fixed-sized windows may include distractors (other people or hands) and too much noise.

CAMSHIFT for Video Sequences

When tracking a colored object, CAMSHIFT operates on a color probability distribution image derived from color histograms. CAMSHIFT calculates the centroid of the 2D color probability distribution within its 2D window of calculation, re-centers the window, then calculates the area for the next window size. Thus, we needn't calculate the color probability distribution over the whole image, but can instead restrict the calculation of the distribution to a smaller image region surrounding the current CAMSHIFT window. This tends to result in large computational savings when flesh color does not dominate the image. We refer to this feedback of calculation region size as the Coupled CAMSHIFT algorithm.

How to Calculate the Coupled CAMSHIFT Algorithm

1. First, set the calculation region of the probability distribution to the whole image.
2. Choose the initial location of the 2D mean shift search window.
3. Calculate the color probability distribution in the 2D region centered at the search window location in an area slightly larger than the mean shift window size.
4. Mean shift to convergence or for a set number of iterations. Store the zeroth moment (area or size) and mean location.
5. For the next video frame, center the search window at the mean location stored in Step 4 and set the window size to a function of the zeroth moment found there. Go to Step 3.

For each frame, the mean shift algorithm will tend to converge to the mode of the distribution. Therefore, CAMSHIFT for video will tend to track the center (mode) of color objects moving in a video scene. Figure 6 shows CAMSHIFT locked onto the mode of a flesh color probability distribution (mode center and area are marked on the original video image). In this figure, CAMSHIFT marks the face centroid with a cross and displays its search window with a box.

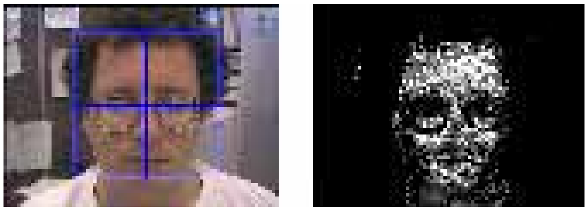

Figure 6: A video image and its flesh probability image

Calculation of Head Roll

The 2D orientation of the probability distribution is also easy to obtain by using the second moments during the course of CAMSHIFT's operation where (x,y) range over the search window, and I(x,y) is the pixel (probability) value at (x,y):

Second moments are

$$M_{20} = \sum_x \sum_y x^2 I(x,y); \quad M_{20} = \sum_x \sum_y x^2 I(x,y).$$

Then the object orientation (major axis) is

$$\theta = \frac{\arctan\left(\dfrac{2\left(\dfrac{M_{11}}{M_{00}} - x_c y_c \right)}{\left(\dfrac{M_{20}}{M_{00}} - x_c^2 \right) - \left(\dfrac{M_{02}}{M_{00}} - y_c^2 \right)} \right)}{2}$$

The first two Eigenvalues (major length and width) of the probability distribution "blob" found by CAMSHIFT may be calculated in closed form as follows [4]. Let

$$a = \frac{M_{20}}{M_{00}} - x_c^2,$$

$$b = 2\left(\frac{M_{11}}{M_{00}} - x_c y_c \right),$$

and

$$c = \frac{M_{02}}{M_{00}} - y_c^2,$$

Then length l and width w from the distribution centroid are

$$l = \sqrt{\frac{(a+c) + \sqrt{b^2 + (a-c)^2}}{2}},$$

$$w = \sqrt{\frac{(a+c) - \sqrt{b^2 + (a-c)^2}}{2}}.$$

When used in face tracking, the above equations give us head roll, length, and width as marked in Figure 7.

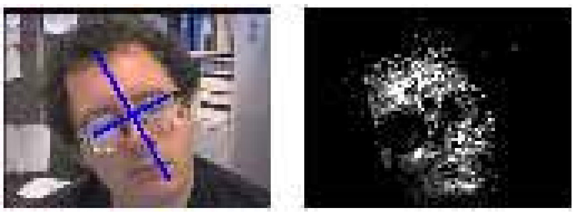

Figure 7: Orientation of the flesh probability distribution marked on the source video image

CAMSHIFT thus gives us a computationally efficient, simple to implement algorithm that tracks four degrees of freedom (see Figure 8).

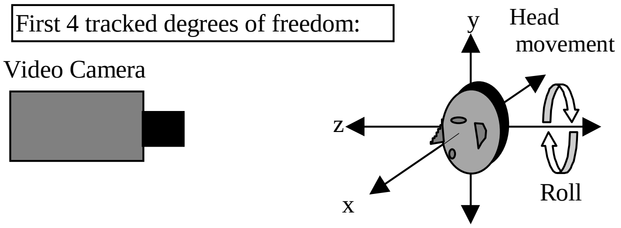

Figure 8: First four head tracked degrees of freedom: X, Y, Z location, and head roll

How CAMSHIFT Deals with Image Problems

When tracking color objects, CAMSHIFT deals with the image problems mentioned previously of irregular object motion due to perspective, image noise, distractors, and facial occlusion as described below.

CAMSHIFT continuously re-scales itself in a way that naturally fits the structure of the data. A colored object's potential velocity and acceleration scale with its distance to the camera, which in turn, scales the size of its color distribution in the image plane. Thus, when objects are close, they can move rapidly in the image plane, but their probability distribution also occupies a large area. In this situation, CAMSHIFT's window size is also large and so can catch large movements. When objects are distant, the color distribution is small so CAMSHIFT's window size is small, but distal objects are slower to traverse the video scene. This natural adaptation to distribution scale and translation allows us to do without predictive filters or variables–a further computational saving–and serves as an in-built antidote to the problem of erratic object motion.

CAMSHIFT's windowed distribution gradient climbing causes it to ignore distribution outliers. Therefore, CAMSHIFT produces very little jitter in noise and, as a result, tracking variables do not have to be smoothed or filtered. This gives us robust noise tolerance.

CAMSHIFT's robust ability to ignore outliers also allows it to be robust against distractors. Once CAMSHIFT is locked onto the mode of a color distribution, it will tend to ignore other nearby but non-connected color distributions. Thus, when CAMSHIFT is tracking a face, the presence of other faces or hand movements in the scene will not cause CAMSHIFT to loose the original face unless the other faces or hand movements substantially occlude the original face.

CAMSHIFT's provable convergence to the mode of probability distributions helps it ignore partial occlusions of the colored object. CAMSHIFT will tend to stick to the mode of the color distribution that remains.

Moreover, when CAMSHIFT's window size is set somewhat greater than the root of the distribution area under its window, CAMSHIFT tends to grow to encompass the connected area of the distribution that is being tracked (see Figure 4). This is just what is desired for tracking whole objects such as faces, hands, and colored tools. This property enables CAMSHIFT to not get stuck tracking, for example, the nose of a face, but instead to track the whole face.

Implementation Details

Initial Window Size and Placement

In practice, we work with digital video images so our distributions are discrete. Since CAMSHIFT is an algorithm that climbs the gradient of a distribution, the minimum search window size must be greater than one in order to detect a gradient. Also, in order to center the window, it should be of odd size. Thus for discrete distributions, the minimum window size is set at three. For this reason too, as CAMSHIFT adapts its search window size, the size of the search window is rounded up to the current or next greatest odd number. In practice, at start up, we calculate the color probability of the whole scene and use the zeroth moment to set the window size (see subsection below) and the centroid to set the window center.

Setting Adaptive Window Size Function

Deciding what function of the zeroth moment to set the search window size to in Step 3 of the CAMSHIFT algorithm depends on an understanding of the distribution that one wants to track and the goal that one wants to achieve. The first consideration is to translate the zeroth moment information into units that make sense for setting window size. Thus, in Figure 4, the maximum distribution value per discrete cell is 206, so we divide the zeroth moment by 206 to convert the calculated area under the search window to units of number of cells. Our goal is then to track the whole color object so we need an expansive window. Thus, we further multiply the result by two so that the window grows to encompass the connected distribution area. We then round to the next greatest odd search window size so that the window has a center.

For 2D color probability distributions where the maximum pixel value is 255, we set window size *s* to

$$s = 2 * \sqrt{\frac{M_{00}}{256}}.$$

We divide by 256 for the same reason stated above, but to convert the resulting 2D region to a 1D length, we need to take the square root. In practice, for tracking faces, we set window width to *s* and window length to *1.2s* since faces are somewhat elliptical.

Comments on Software Calibration

Much of CAMSHIFT’s robustness to noise, transient occlusions, and distractors depends on the search window matching the size of the object being tracked—it is better to err on the side of the search window being a little too small. The search window size depends on the function of the zeroth moment M_{00} chosen above. To indirectly control the search window size, we adjust the color histogram up or down by a constant, truncating at zero or saturating at the maximum pixel value. This adjustment affects the pixel values in the color probability distribution image which affects M_{00} and hence window size. For 8-bit hue, we adjust the histogram down by 20 to 80 (out of a maximum of 255), which tends to shrink the CAMSHIFT window to just within the object being tracked and also reduces image noise.

HSV brightness and saturation thresholds are employed since hue is not well defined for very low or high brightness or low saturation. Low and high thresholds are set off 10% of the maximum pixel value.

Comments on Hardware Calibration

To use CAMSHIFT as a video color object tracker, the camera’s field of view (zoom) must be set so that it covers the space that one intends to track in. Turn off automatic white balance if possible to avoid sudden color shifts. Try to set (or auto-adjust) AGC, shutter speed, iris or CCD integration time so that image brightness is neither too dim nor saturating. The camera need not be in focus to track colors. CAMSHIFT will work well with cheap cameras and does not need calibrated lenses.

CAMSHIFT’S Use as a Perceptual Interface

Treatment of CAMSHIFT Tracking Variables for Use in a Perceptual User Interface

Figure 8 above shows the variables X, Y, Z, and Roll returned by the CAMSHIFT face tracker. For game and graphics control, X, Y, Z, and Roll often require a “neutral” position; that is, a position relative to which further face movement is measured. For example, if the captured video image has dimensions (Y, X) of 120x160, a typical neutral position might be Y=60, X=80. Then if X < 80, the user has moved 80-X left; if X > 80, the user has moved X-80 right and so on for each variable.

Piecewise Linear Transformation of Control Variables

To obtain differential control at various positions including a jitter-damping neutral movement region, each variable’s relative movement (above or below the neutral position) is scaled in “N” different ranges.

In the X variable example above, if X is in range #1, X would be scaled by “X scale 1”; if X is in range #2, X would be scaled by “X scale 2” and so on.

The formula for mapping captured video head position P to a screen movement factor F is

$F = \min(b_1,P)s_1 + [\min(b_2-b_1, P-b_1)]^+s_2 + \ldots + [\min(b_{(i+1)}-b_i, P-b_i)]^+s_{(i+1)} + \ldots + [P-b_{(N-1)}]^+s_{N},$ **(control equation 1)**

where $[\#]^+$ equals “#” if # > 0, and zero otherwise; min(A, B) returns the minimum of A or B; b_1-b_N represents the bounds of the ranges, s_1-s_N are the corresponding scale factors for each range, and P is the absolute value of the difference of the variable’s location from neutral.

This allows for stability close to the neutral position, with growing acceleration of movement as the distance away from neutral increases, in a piecewise linear manner as shown in Figure 9.

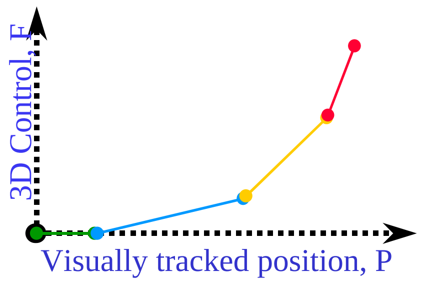

Figure 9: Piecewise linear transformation of CAMSHIFT position P interface control variable F

Frame Rate Adjustment

If the graphic's rendering rate R can be determined, the final screen movement S is

$S = F/R$ **(control equation 2)**

Computer graphics and game movement commands S can be issued on each rendered graphic's frame. Thus, it is best for the movement amount S to be sensitive to frame rate. In computer-rendered graphic or game scenes, simple views (for example, looking up at blue sky) are rendered much faster than complex views (for example, texture mapped city skylines). The final rate of movement should not depend on the complexity of the 3D view if one wants to achieve satisfying motion when immersed in a 3D scene.

Y Variable Special Case for Seated User

If a user sits facing the camera (neutral X,Y) and then leans left or right (X movement) by pivoting on his/her chair, Y will decrease as shown in Figure 10 below.

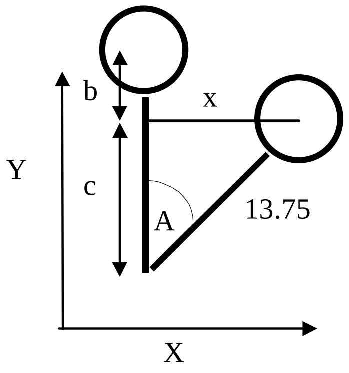

Figure 10: Lean changes Y and head roll

In order to overcome this, we use an empirical observation that the 2nd Eigenvalue (face half width) of the local face flesh color distribution length is proportional to face size (see Figure 7), which is, on average, often proportional to body size. Empirically, the ratio of the 2nd Eigenvector to torso length from face centroid is

1 to 13.75 (2 inches to 27.5 inches). **(control equation 3)**

Given lean distance x (in 2nd Eigenvector units), and seated size of 13.75, as in Figure 10 so that sin(A) = x/13.75. Then,

$A = \sin^{-1}(x/13.75)$, **(control equation 4)**

so $c = 13.75\cos(A)$, and

$b = 13.75(1 - \cos(A))$ **(control equation 5)**

in units of 2nd Eigenvectors. This is the Y distance to correct for (add back) when leaning.

Roll Considerations

As can be seen from Figure 10, for seated users lean also induces a change in head roll by the angle A. Thus, for control that relies on head roll, this lean-induced roll should be corrected for. Correction can be accomplished in two ways:

- Make the first range boundary b1 in control equation 1 large enough to contain the changes in face orientation that result from leaning. Then use a scale value s1 = 0 so that leaning causes no roll.
- Subtract the measured roll from the lean-induced roll, A, calculated in control Equation 4 above.

Another possible problem can result when the user looks down too much as shown in Figure 11. In this case, the user is looking down at the keyboard. Looking down too much causes the forehead to dominate the view which in turn causes the face flesh color "blob" to look like it is oriented horizontally.

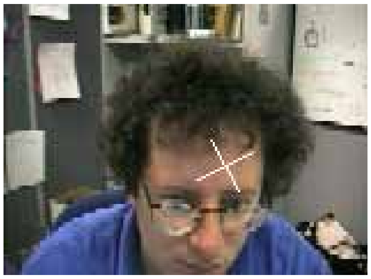

Figure 11: Extreme down head pitch causes a corrupted head roll value

To correct for such problems, we define a new variable, Q called "Roll Quality." Q is the ratio of the first two Eigenvalues, length l and width w, of the distribution color "blob" in the CAMSHIFT search window:

$Q = l/w$. **(control equation 6)**

For problem views of the face such as in Figure 11, we observe that Roll Quality is nearly 1.0. So, for face

tracking, roll should be ignored (treated as vertical) for quality measures less than 1.25. Roll should also be ignored for very high quality scores greater than 2.0 since such distributions are un-facelike and likely to have resulted from noise or occlusions.

CAMSHIFT's Actual Use as an Interface

CAMSHIFT is being used as a face tracker to control games and 3D graphics. By inserting face control variables into the mouse queue, we can control unmodified commercial games such as Quake 2 shown in Figure 12. We used left and right head movements to slide a user left and right in the game, back and forth head movements to move the user backwards and forwards, up or down movements to let the user shoot (as if ducking or getting jolted by the gun), and roll left or right to turn the user left or right in the game. This methodology has been used extensively in a series of demos with over 30 different users.

Head tracking via CAMSHIFT has also been used to experiment with immersive 3D graphics control in which natural head movements are translated to moving the corresponding 3D graphics camera viewpoint. This has been extensively tested using a 3D graphics model of the Forbidden City in China as well as in exploring a 3D graphics model of the big island of Hawaii as shown in Figure 13. Most users find it an enjoyable experience in which they naturally pick up how to control the graphics viewpoint movement.

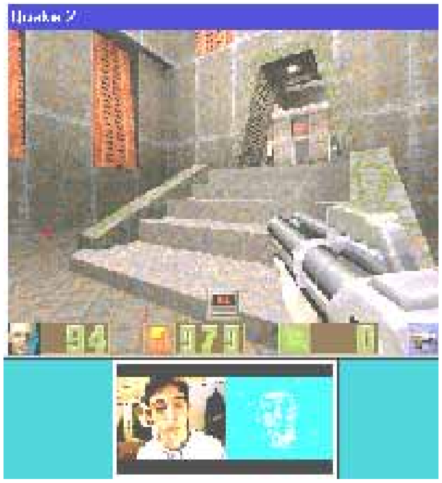

Figure 12: CAMSHIFT-based face tracker used to play Quake 2 hands free by inserting control variables into the mouse queue

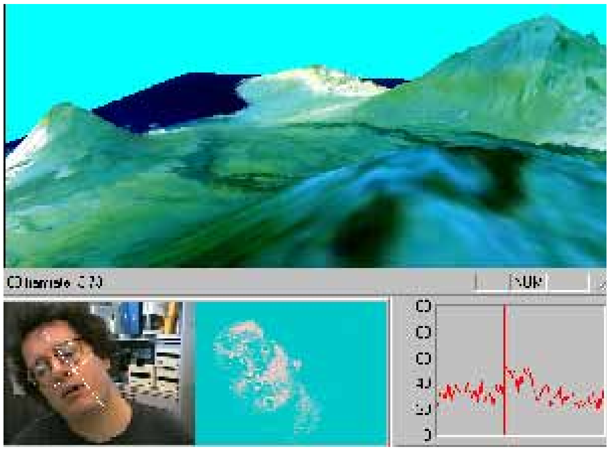

Figure 13: CAMSHIFT-based face tracker used to "fly" over a 3D graphic's model of Hawaii

CAMSHIFT Analysis

Comparison to Polhemus

In order to assess the tracking accuracy of CAMSHIFT, we compared its accuracy against a Polhemus tracker. Polhemus is a magnetic sensor connected to a system that measures six degrees of spatial freedom and thus can be used for object tracking when tethered to an object. The observed accuracy of Polhemus is +/- 1.5cm in spatial location and about 2.5^{o} in orientation within 30 inches of the Polhemus antenna. We compared Polhemus tracking to CAMSHIFT color object tracking using a 320x240 image size (see Figure 14a-d). The coordinate systems of Polhemus and the camera were carefully aligned prior to testing. The object tracked was pulled on a cart in a set trajectory away from the Polhemus origin. The comparison between CAMSHIFT and Polhemus in each of X, Y, Z, and Roll yielded the results shown Table 1.

Tracking Variable	X	Y	Z	Roll
Standard Deviation of Difference	0.27cm	0.58cm	3.4cm	2.4^{o}

Table 1: Standard deviation of Polhemus vs. CAMSHIFT tracking differences

Z exhibited the worst difference because CAMSHIFT determines Z by measuring color area, which is inherently noisy. X, Y, and Roll are well within Polhemus's observed tracking error and therefore indistinguishable. Z is about 2cm off. Except for Z, these results are as good or better than much more elaborate vision tracking systems [17], although CAMSHIFT does not yet track pitch and yaw.

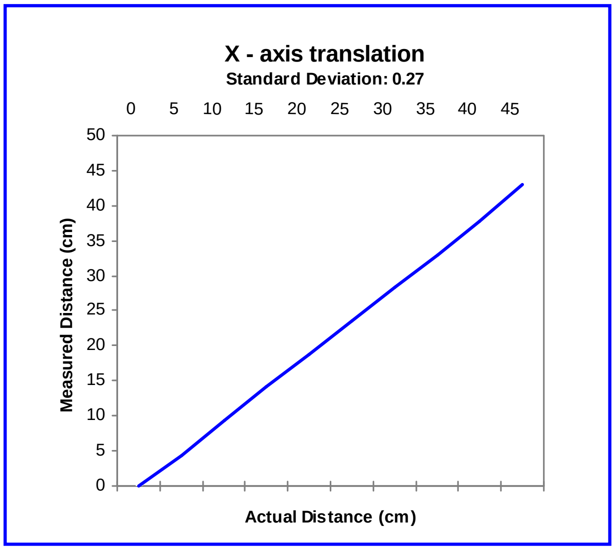

Figure 14a: Comparision of X tracking accuracy

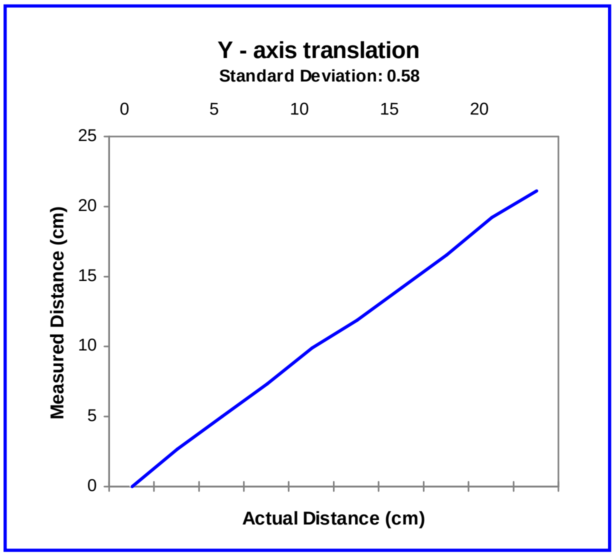

Figure 14b: Comparision of Y tracking accuracy

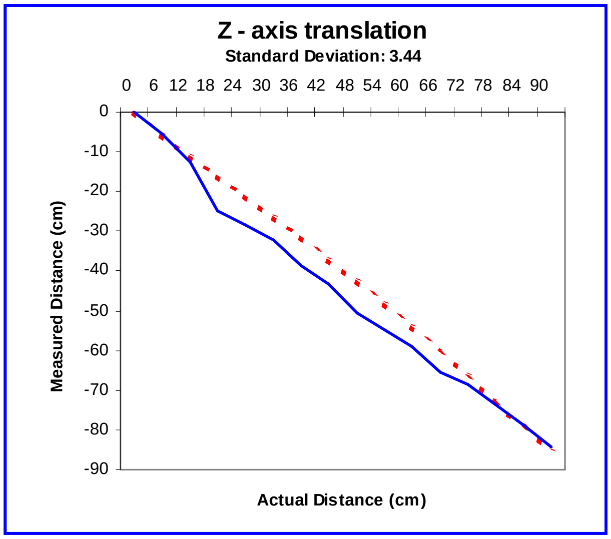

Figure 14c: Comparision of Z tracking accuracy

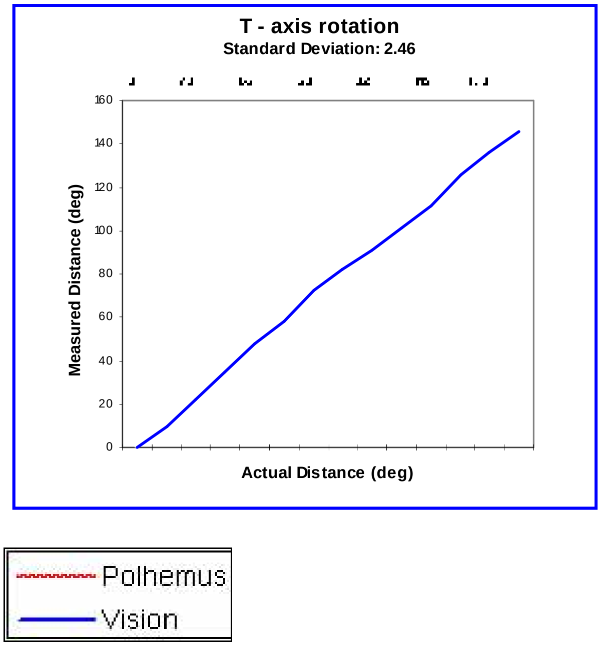

Figure 14d: Accuracy comparison of Polhemus and CAMSHIFT tracking for roll.

Tracking in Noise

CAMSHIFT's robust ability to find and track the mode of a dynamically changing probability distribution also gives it good tracking behavior in noise. We videotaped a head movement sequence and then played it back adding 0, 10, 30, and 50% uniform noise. Figure 15 shows 50% noise added to the raw image on the left, and the resulting color probability distribution on the right. Note that the use of a color model greatly cuts down the random noise since color noise has a low probability of being flesh color.

Nevertheless, the flesh color model is highly degraded and there are many spurious flesh pixels in the color probability distribution image. But CAMSHIFT is still able to track X, Y, and Roll quite well in up to 30% white noise as shown in Figure 16a-d. Z is more of a problem because CAMSHIFT measures Z by tracking distribution area under its search window, and one can see in Figure 15 that area is highly effected by noise. Y shows an upward shift simply because the narrower chin region exhibits more degradation in noise than the wider forehead. Roll tracks well until noise is such that the length and width of the face color distribution are obscured. Thus, CAMSHIFT handles noise well without the need for extra filtering or adaptive smoothing.

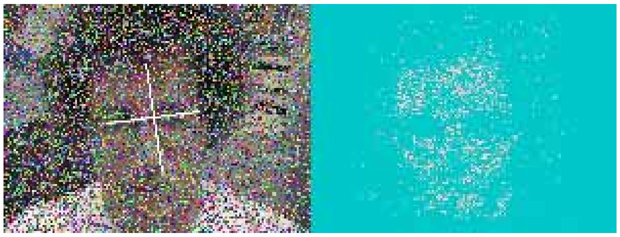

Figure 15: Tracking in 50% uniform noise

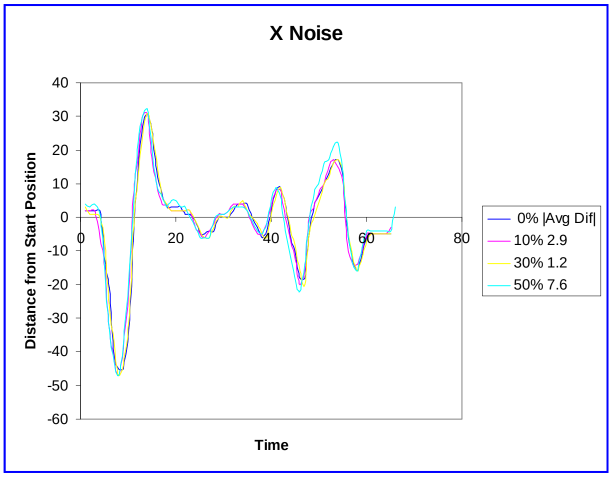

Figure 16a: X accuracy in 0-50% uniform noise

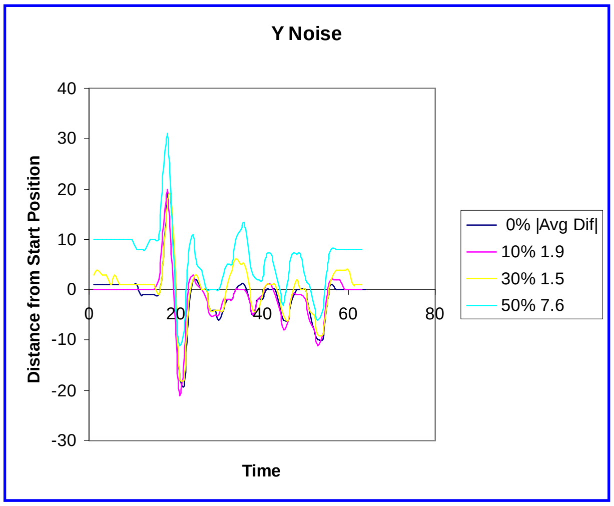

Figure 16b: Y accuracy in 0-50% uniform noise

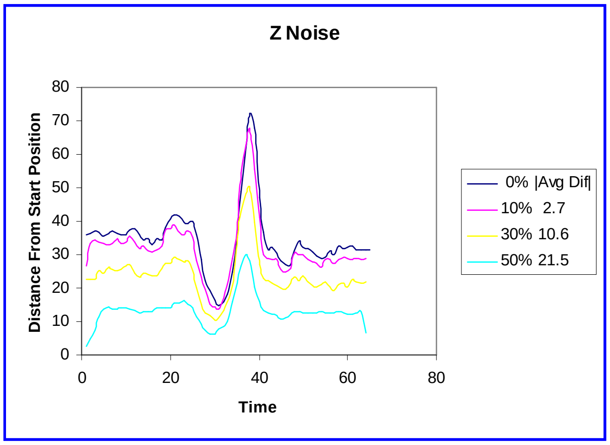

Figure 16c: Z accuracy in 0-50% uniform noise

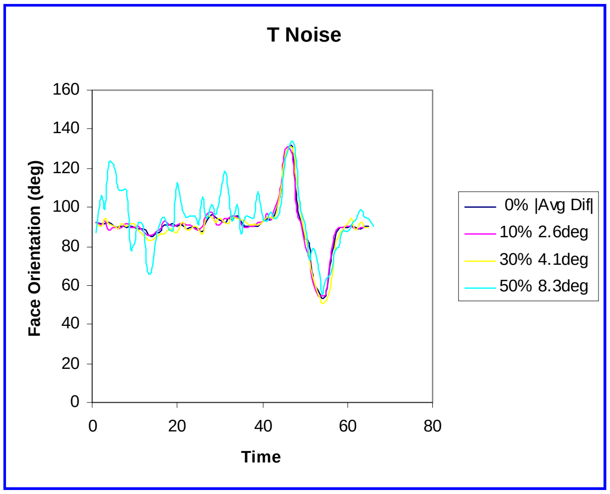

Figure 16d: Roll accuracy in 0-50% uniform noise

Tracking in the Presence of Distractions

CAMSHIFT's search window converges to span the nearest dominant connected probability distribution. If we adjust the nature of the probability distribution by properly setting the HSV brightness and saturation threshold (see Implementation Details section above), the search window will tend to stay just within the object being tracked as shown in the marked image at top left in Figure 17. In such cases, CAMSHIFT is robust against distracting (nearby) distributions and transient occlusions. This robustness occurs for distractors because the search window rarely contains the distractor as shown sequentially down the left, then right, columns of Figure 17.

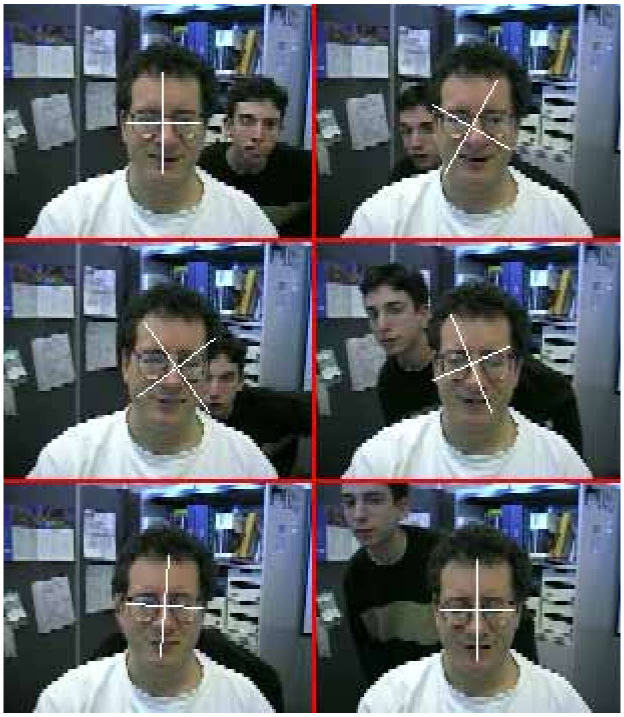

Figure 17: Tracking a face with background distractor faces (sequence: down left then right columns)

Table 2 shows the results collected from 44 sample point on five tracking runs with active background face distraction such as that shown in Figure 17. Since the distracting face rarely intersects much of CAMSHIFT's search window, the X, Y, and Z tracking variables are perturbed very little. Roll is more strongly affected since even a small intersection of a distractor in CAMSHIFT's search window can change the effective orientation of the flesh pixels as measured by CAMSHIFT.

Tracked Variable	Average Std. Deviation	Maximum Std. Deviation
X (pixels)	0.42	2.00
Y(pixels)	0.53	1.79
Z(pixels)	0.54	1.50
Roll (degrees)	5.18	46.80

Table 2: Perturbation of CAMSHIFT tracking variables by face distractors

CAMSHIFT tends to be robust against transient occlusion because the search window will tend to first absorb the occlusion and then stick with the dominant distribution mode when the occlusion passes. Figure 18 demonstrates robustness to hand occlusion in sequential steps down the left, then right columns.

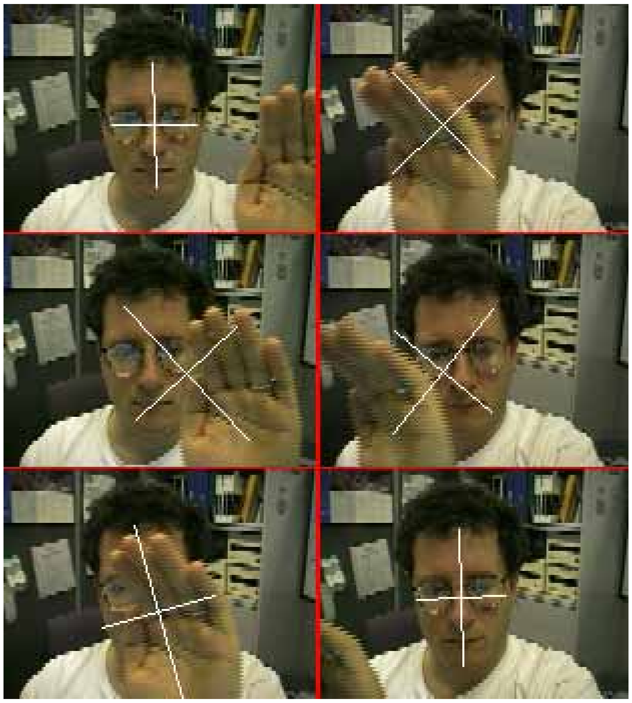

Figure 18: Tracking a face in the presence of passing hand occlusions (sequence: down left then right columns)

Table 3 shows the results collected from 43 sample points on five tracking runs with active transient hand occlusion of the face. Average perturbation is less than three pixels for X, Y, and Z. Roll is more strongly effected due to the arbitrary orientation of the hand as it passes through the search window.

Tracked Variable	Average Std. Deviation	Maximum Std. Deviation
X (pixels)	2.35	7.17
Y(pixels)	2.81	6.29
Z(pixels)	2.10	4.65
Roll (degrees)	14.64	34.40

Table 3: Perturbation of CAMSHIFT tracking variables by passing hand occlusion

We see from the above table that CAMSHIFT gives us wide tolerance for distraction and occlusion "for free" due to the statistically robust workings of the algorithm.

Performance

The order of complexity of CAMSHIFT is O(αN^2) where α is some constant, and the image is taken to be NxN. α is most influenced by the moment calculations and the average number of mean shift iterations until convergence. The biggest computational savings come through scaling the region of calculation to an area around the search window size as previously discussed.

CAMSHIFT was run on a 300 MHz Pentium® II processor, using an image size of 160x120 at 30 frames per second (see Figure 19). CAMSHIFT's performance scales with tracked object size. Figure 19 shows the CPU load from the entire computer vision thread including image acquisition, conversion to color probability distribution, and CAMSHIFT tracking. In Figure 19 when the tracked face is far from the camera, the CAMSHIFT thread consumes only 10% of the CPU cycles. When the face fills the frame, CAMSHIFT consumes 55% of the CPU.

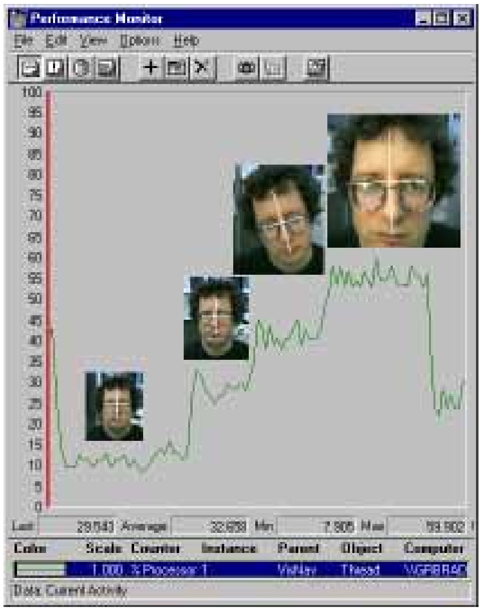

Figure 19: Performance scales inversely with tracked object size (ordinate is percent of CPU used)

Figure 20 traces computer vision thread's performance in an actual control task of "flying" over a 3D model of Hawaii using head movements. In this case, the average CPU usage was 29%. VTUNE™ analysis showed that the actual CAMSHIFT operation (excluding image capture, color conversion or image copying) consumed under 12% of the CPU. CAMSHIFT relies on Intel's MMX™ technology optimized Image Processing Library available on the Web [3] to do RGB-to-HSV image conversion and image allocation. MMX technology optimized image moments calculation has recently been added to the Image Processing Library, but not in time for publication. The use of such optimized moment calculations will boost performance noticeably since this forms part of the inner mean shift calculation loop. Even without this improvement, CAMSHIFT's current average actual use efficiency of 29% allows it to be used as a visual user interface.

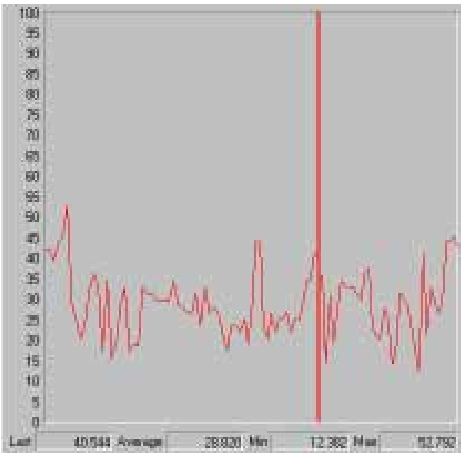

Figure 20: In actual use, CAMSHIFT consumed an average of 29% of one 300 MHz Pentium® II CPU when used to control a 3D graphic’s Hawaii fly through

Discussion

This paper discussed a core tracking module that is part of a larger effort to allow computers to track and understand human motion, pose, and tool use. As such, the module was designed to be simple and computationally efficient. Yet, this core module must still handle the basic computer-vision problems outlined in this paper. We’ve seen that CAMSHIFT handles these problems as follows:

- **Irregular object motion**: CAMSHIFT scales its search window to object size thus naturally handling perspective-induced motion irregularities.
- **Image noise**: The color model eliminates much of the noise, and CAMSHIFT tends to ignore the remaining outliers.
- **Distractors:** CAMSHIFT ignores objects outside its search window so objects such as nearby faces and hands do not affect CAMSHIFT’s tracking.
- **Occlusion:** As long as occlusion isn’t 100%, CAMSHIFT will still tend to follow what is left of the objects’ probability distribution.
- **Lighting variation:** Using only hue from the HSV color space and ignoring pixels with high or low brightness gives CAMSHIFT wide lighting tolerance.

CAMSHIFT’s simplicity does cause limitations however. Since CAMSHIFT derives Z from object area estimates, Z is subject to noise and spurious values. The effects of noise are evident in Figure 16c. That CAMSHIFT can get spurious area values is evident in Figure 11.

Since CAMSHIFT relies on color distributions alone, errors in color (colored lighting, dim illumination, too much illumination) will cause errors in tracking. More sophisticated trackers use multiple modes such as feature tracking and motion analysis to compensate for this, but more complexity would undermine the original design criterion for CAMSHIFT.

CAMSHIFT also only detects four (X, Y, Z, and Roll) of the six modes of freedom (above plus pitch and yaw). Unfortunately, of the six degrees of head movement possible, Roll is the least useful control variable since it is the least “natural” head movement and is therefore fatiguing for the user to use constantly.

Conclusion

CAMSHIFT is a simple, computationally efficient face and colored object tracker. While acknowledging the limitation imposed by its simplicity, we can still see that CAMSHIFT tracks virtually as well as more expensive tethered trackers (Polhemus) or much more sophisticated, computationally expensive vision systems [17], and it tracks well in noisy environments. Thus, as we have shown, even though CAMSHIFT was conceived as a simple part of a larger tracking system, it has many uses right now in game and 3D graphics’ control.

Adding perceptual interfaces can make computers more natural to use, more fun for games and graphics, and a better medium of communication. These new features consume more MIPs and so will take advantage of more MIPs available with future Intel® CPUs.

In this project, we designed a highly efficient face tracking algorithm rather than a more complex, higher MIPs usage algorithm. This was done because we want to be able to demonstrate compelling applications and interfaces on today’s systems in order to prepare the way for the future use of computer vision on PCs. CAMSHIFT is usable as a visual interface now, yet designed to be part of a more robust, larger tracking system in the future. CAMSHIFT will be incorporated into larger, more complex, higher MIPs-demanding modules that provide more robust tracking, posture understanding, gesture and face recognition, and object understanding. In this way, the functionality of the computer vision interface will increase with increasing Intel CPU speeds. A user will thus be able to upgrade their computer vision interface by upgrading to higher speed Intel CPUs in the future.

Acknowledgments

Many thanks to Mark Holler for countless discussions and feedback leading to the development of this work. Special thanks to Ryan Boller for rapid implementation of the CAMSHIFT game control demo and for major help in implementing the testing routines for CAMSHIFT.

References

[1] D. Comaniciu and P. Meer, "Robust Analysis of Feature Spaces: Color Image Segmentation," CVPR'97, pp. 750-755.

[2] K. Fukunaga, "Introduction to Statistical Pattern Recognition," Academic Press, Boston, 1990.

[3] MMX^{TM} technology optimized libraries in image, signal processing, pattern recognition and matrix math can be downloaded from http://developer.intel.com/design/perftool/perflibst/index.htm)

[4] W.T. Freeman, K. Tanaka, J.Ohta, and K. Kyuma, "Computer Vision for Computer Games," Int. Conf. On Automatic Face and Gesture Recognition, pp. 100-105, 1996.

[5] A.R. Smith, "Color Gamut Transform Pairs," SIGGRAPH 78, pp. 12-19, 1978.

[6] J.D. Foley, A. van Dam, S. K. Feiner and J.F. Hughes, "Computer graphics principles and practice," Addison-Wesley, pp. 590-591.

[7] P. Fieguth and D. Terzopoulos, "Color-based tracking of heads and other mobile objects at video frame rates," In Proc. Of IEEE CVPR, pp. 21-27, 1997.

[8] C. Wren, A. Azarbayejani, T. Darrell, A.Pentland, "Pfinder: Real-Time Tracking of the Human Body," SPIE Vol. 2615, 1995.

[9] M. Hunke and A. Waibel, "Face locating and tracking for human-computer interaction," Proc. Of the 28th Asilomar Conf. On Signals, Sys. and Comp., pp. 1277-1281, 1994.

[10] K. Sobottka and I. Pitas, "Segmentation and tracking of faces in color images," Proc. Of the Second Intl. Conf. On Auto. Face and Gesture Recognition, pp. 236-241, 1996.

[11] M. Swain and D. Ballard, "Color indexing," Intl. J. of Computer Vision, 7(1) pp. 11-32, 1991.

[12] M. Kass, A. Witkin D.Terzopoulos, "Snakes: Active contour Models," Int. J. o f Computer Vision (1) #4, pp. 321-331, 1988.

[13] C. Vieren, F. Cabestaing, J. Postaire, "Catching moving objects with snakes for motion tracking," Pattern Recognition Letters (16) #7, pp. 679-685, 1995.

[14] A. Pentland, B. Moghaddam, T. Starner, "View-based and Modular Eigenspaces for face recognition," CVPR'94, pp. 84-91, 1994.

[15] M. Isard, A. Blake, "Contour tracking by stochastic propagation of conditional density," Proc. 4th European Conf. On Computer Vision, Cambridge, UK, April 1996.

[16] T. Maurer, and C. von der Malsburg, "Tracking and learning graphs and pose on image sequence of faces," Proc. Of the Second Intl. Conf. On Auto. Face and Gesture Recognition, pp. 176-181, 1996.

[17] A. Azabayejani, T. Starner, B. Horowitz, and A. Pentland, "Visually Controlled Graphics," IEEE Tran. Pat. Anal. and Mach. Intel. pp. 602-605, Vol. 15, No. 6, June 1993.

[18] Y. Cheng, "Mean shift, mode seeking, and clustering," IEEE Trans. Pattern Anal. Machine Intell., 17:790-799, 1995.

Author's Biography

Dr. Gary R. Bradski received a Ph.D. in pattern recognition and computer vision from Boston University. He works in computer vision research in the Microcomputer Research Labs at Intel's Mission College campus. His interests include segmenting and tracking people in visual scenes; perceptual computer interfaces; applying visual 3D structure to 3D graphics; pattern recognition; biological perception and self-organization. His e-mail is gary.bradski@intel.com.
