# Supplementary material for: Rapid whole brain imaging of neural activity in freely behaving larval zebrafish (Danio rerio)
Source: eLife. 2017 Sep 20;6:e28158. doi: 10.7554/eLife.28158 (PMC5644961; doi:10.7554/eLife.28158)
Supplement: Source code 3. [file elife-28158-code3.zip › RT analysis code/OpenCV1.1pre.zip!/OpenCV/docs/ref/opencvref_cv.htm]

OpenCV: Image Processing and Computer Vision Reference Manual

# CV Reference Manual

---

- Image Processing
  - Gradients, Edges, Corners and Features- Sampling, Interpolation and Geometrical Transforms- Morphological Operations- Filters and Color Conversion- Pyramids and the Applications- Image Segmentation, Connected Components and Contour Retrieval- Image and Contour Moments- Special Image Transforms- Histograms- Matching
  - Structural Analysis
    - Contour Processing- Computational Geometry- Planar Subdivisions
    - Motion Analysis and Object Tracking
      - Accumulation of Background Statistics- Motion Templates- Object Tracking- Optical Flow- Feature Matching- Estimators
      - Pattern Recognition
        - Object Detection
        - Camera Calibration and 3D Reconstruction
          - Single and Stereo Camera Calibration- Pose Estimation- Epipolar Geometry, Stereo Correspondence
          - Alphabetical List of Functions- Bibliography

---

# Image Processing

Note:  
The chapter describes functions for image processing and analysis.
Most of the functions work with 2d arrays of pixels. We refer the arrays
as "images" however they do not necessarily have to be IplImage’s, they may
be CvMat’s or CvMatND’s as well.

---

## Gradients, Edges, Corners and Features

---

### Sobel

Calculates first, second, third or mixed image derivatives using extended Sobel operator

```
void cvSobel( const CvArr* src, CvArr* dst, int xorder, int yorder, int aperture_size=3 );
```

src: Source image. dst: Destination image. xorder: Order of the derivative x . yorder: Order of the derivative y . aperture\_size: Size of the extended Sobel kernel, must be 1, 3, 5 or 7. In all cases except 1, aperture\_size ×aperture\_size separable kernel will be used to calculate the derivative. For `aperture_size`=1 3x1 or 1x3 kernel is used (Gaussian smoothing is not done). There is also special value `CV_SCHARR` (=-1) that corresponds to 3x3 Scharr filter that may give more accurate results than 3x3 Sobel. Scharr aperture is: ``` | -3 0 3| |-10 0 10| | -3 0 3| ``` for x-derivative or transposed for y-derivative.

The function `cvSobel` calculates the image derivative by convolving the image
with the appropriate kernel:

```
dst(x,y) = dxorder+yordersrc/dxxorder•dyyorder |(x,y)
```

The Sobel operators combine Gaussian smoothing and differentiation so the result is more or less
robust to the noise. Most often, the function is called with (xorder=1, yorder=0, aperture\_size=3) or
(xorder=0, yorder=1, aperture\_size=3) to calculate first x- or y- image derivative.
The first case corresponds to

```
  |-1  0  1|
  |-2  0  2|
  |-1  0  1|
```

kernel and the second one corresponds to

```
  |-1 -2 -1|
  | 0  0  0|
  | 1  2  1|
or
  | 1  2  1|
  | 0  0  0|
  |-1 -2 -1|
```

kernel, depending on the image origin (`origin` field of `IplImage` structure).
No scaling is done, so the destination image usually has larger by absolute value numbers than
the source image. To avoid overflow, the function requires 16-bit destination image if
the source image is 8-bit. The result can be converted back to 8-bit using cvConvertScale or
cvConvertScaleAbs functions. Besides 8-bit images the function
can process 32-bit floating-point images.
Both source and destination must be single-channel images of equal size or ROI size.


---

### Laplace

Calculates Laplacian of the image

```
void cvLaplace( const CvArr* src, CvArr* dst, int aperture_size=3 );
```

src: Source image. dst: Destination image. aperture\_size: Aperture size (it has the same meaning as in cvSobel).

The function `cvLaplace` calculates Laplacian of the source image by summing
second x- and y- derivatives calculated using Sobel operator:

```
dst(x,y) = d2src/dx2 + d2src/dy2
```

Specifying `aperture_size`=1 gives the fastest variant that is equal to
convolving the image with the following kernel:

```
|0  1  0|
|1 -4  1|
|0  1  0|
```

Similar to cvSobel function, no scaling is done and the same combinations of input and
output formats are supported.

---

### Canny

Implements Canny algorithm for edge detection

```
void cvCanny( const CvArr* image, CvArr* edges, double threshold1,
              double threshold2, int aperture_size=3 );
```

image: Input image. edges: Image to store the edges found by the function. threshold1: The first threshold. threshold2: The second threshold. aperture\_size: Aperture parameter for Sobel operator (see cvSobel).

The function `cvCanny` finds the edges on the input image `image` and marks them in the
output image `edges` using the Canny algorithm. The smallest of `threshold1` and
`threshold2` is used for edge linking, the largest - to find initial segments of strong edges.

---

### PreCornerDetect

Calculates feature map for corner detection

```
void cvPreCornerDetect( const CvArr* image, CvArr* corners, int aperture_size=3 );
```

image: Input image. corners: Image to store the corner candidates. aperture\_size: Aperture parameter for Sobel operator (see cvSobel).

The function `cvPreCornerDetect` calculates the function
D_x_^2^D_yy_+D_y_^2^D_xx_ - 2D_x_D_y_D_xy_
where D_?_ denotes one of the first image derivatives and D_??_ denotes a second image
derivative. The corners can be found as local maximums of the function:

```
// assume that the image is floating-point
IplImage* corners = cvCloneImage(image);
IplImage* dilated_corners = cvCloneImage(image);
IplImage* corner_mask = cvCreateImage( cvGetSize(image), 8, 1 );
cvPreCornerDetect( image, corners, 3 );
cvDilate( corners, dilated_corners, 0, 1 );
cvSubS( corners, dilated_corners, corners );
cvCmpS( corners, 0, corner_mask, CV_CMP_GE );
cvReleaseImage( &corners );
cvReleaseImage( &dilated_corners );
```

---

### CornerEigenValsAndVecs

Calculates eigenvalues and eigenvectors of image blocks for corner detection

```
void cvCornerEigenValsAndVecs( const CvArr* image, CvArr* eigenvv,
                               int block_size, int aperture_size=3 );
```

image: Input image. eigenvv: Image to store the results. It must be 6 times wider than the input image. block\_size: Neighborhood size (see discussion). aperture\_size: Aperture parameter for Sobel operator (see cvSobel).

For every pixel The function `cvCornerEigenValsAndVecs` considers
`block_size` × `block_size` neighborhood S(p). It calculates
covariation matrix of derivatives over the neighborhood as:

```
    | sumS(p)(dI/dx)2   sumS(p)(dI/dx•dI/dy)|
M = |                                 |
    | sumS(p)(dI/dx•dI/dy)  sumS(p)(dI/dy)2 |
```

After that it finds eigenvectors and eigenvalues of the matrix and stores
them into destination image in form
(λ_1_, λ_2_, x_1_, y_1_, x_2_, y_2_),
where  
λ_1_, λ_2_ - eigenvalues of `M`; not sorted  
(x_1_, y_1_) - eigenvector corresponding to λ_1_  
(x_2_, y_2_) - eigenvector corresponding to λ_2_

---

### CornerMinEigenVal

Calculates minimal eigenvalue of gradient matrices for corner detection

```
void cvCornerMinEigenVal( const CvArr* image, CvArr* eigenval, int block_size, int aperture_size=3 );
```

image: Input image. eigenval: Image to store the minimal eigenvalues. Should have the same size as `image` block\_size: Neighborhood size (see discussion of cvCornerEigenValsAndVecs). aperture\_size: Aperture parameter for Sobel operator (see cvSobel). format. In the case of floating-point input format this parameter is the number of the fixed float filter used for differencing.

The function `cvCornerMinEigenVal` is similar to cvCornerEigenValsAndVecs but
it calculates and stores only the minimal eigenvalue of derivative covariation matrix for every pixel,
i.e. min(λ_1_, λ_2_) in terms of the previous function.

---

### CornerHarris

Harris edge detector

```
void cvCornerHarris( const CvArr* image, CvArr* harris_dst,
                     int block_size, int aperture_size=3, double k=0.04 );
```

image: Input image. harris\_dst: Image to store the Harris detector responses. Should have the same size as `image` block\_size: Neighborhood size (see discussion of cvCornerEigenValsAndVecs). aperture\_size: Aperture parameter for Sobel operator (see cvSobel). format. In the case of floating-point input format this parameter is the number of the fixed float filter used for differencing. k: Harris detector free parameter. See the formula below.

The function `cvCornerHarris` finds feature points (corners) in the image
using Harris' method. Similarly to
cvCornerMinEigenVal and
cvCornerEigenValsAndVecs,
for each pixel it calculates 2x2 gradient covariation matrix
`M` over `block_size×block_size` neighborhood.
Then, it stores

```
det(M) - k*trace(M)2
```

to the corresponding pixel of the destination image.
The corners can be found as local maxima in the destination image.

---

### FindCornerSubPix

Refines corner locations

```
void cvFindCornerSubPix( const CvArr* image, CvPoint2D32f* corners,
                         int count, CvSize win, CvSize zero_zone,
                         CvTermCriteria criteria );
```

image: Input image. corners: Initial coordinates of the input corners and refined coordinates on output. count: Number of corners. win: Half sizes of the search window. For example, if `win`=(5,5) then 5\*2+1 × 5\*2+1 = 11 × 11 search window is used. zero\_zone: Half size of the dead region in the middle of the search zone over which the summation in formulae below is not done. It is used sometimes to avoid possible singularities of the autocorrelation matrix. The value of (-1,-1) indicates that there is no such size. criteria: Criteria for termination of the iterative process of corner refinement. That is, the process of corner position refinement stops either after certain number of iteration or when a required accuracy is achieved. The `criteria` may specify either of or both the maximum number of iteration and the required accuracy.

The function `cvFindCornerSubPix` iterates to find the sub-pixel accurate location
of corners, or radial saddle points, as shown in on the picture below.

Sub-pixel accurate corner locator is based on the observation that every vector
from the center `q` to a point `p` located within a neighborhood of `q` is orthogonal
to the image gradient at `p` subject to image and measurement noise. Consider the expression:

```
εi=DIpiT•(q-pi)
```

where `DIpi` is the image gradient
at the one of the points `pi` in a neighborhood of `q`.
The value of `q` is to be found such that `εi` is minimized.
A system of equations may be set up with `εi`' set to zero:

```
sumi(DIpi•DIpiT)•q - sumi(DIpi•DIpiT•pi) = 0
```

where the gradients are summed within a neighborhood ("search window") of `q`.
Calling the first gradient term `G` and the second gradient term `b` gives:

```
q=G-1•b
```

The algorithm sets the center of the neighborhood window at this new center `q`
and then iterates until the center keeps within a set threshold.

---

### GoodFeaturesToTrack

Determines strong corners on image

```
void cvGoodFeaturesToTrack( const CvArr* image, CvArr* eig_image, CvArr* temp_image,
                            CvPoint2D32f* corners, int* corner_count,
                            double quality_level, double min_distance,
                            const CvArr* mask=NULL, int block_size=3,
                            int use_harris=0, double k=0.04 );
```

image: The source 8-bit or floating-point 32-bit, single-channel image. eig\_image: Temporary floating-point 32-bit image of the same size as `image`. temp\_image: Another temporary image of the same size and same format as `eig_image`. corners: Output parameter. Detected corners. corner\_count: Output parameter. Number of detected corners. quality\_level: Multiplier for the maxmin eigenvalue; specifies minimal accepted quality of image corners. min\_distance: Limit, specifying minimum possible distance between returned corners; Euclidean distance is used. mask: Region of interest. The function selects points either in the specified region or in the whole image if the mask is NULL. block\_size: Size of the averaging block, passed to underlying cvCornerMinEigenVal or cvCornerHarris used by the function. use\_harris: If nonzero, Harris operator (cvCornerHarris) is used instead of default cvCornerMinEigenVal. k: Free parameter of Harris detector; used only if `use_harris≠0`

The function `cvGoodFeaturesToTrack` finds corners with big eigenvalues in the
image. The function first calculates the minimal eigenvalue for every source image pixel
using cvCornerMinEigenVal function and stores them in `eig_image`.
Then it performs non-maxima suppression (only local maxima in 3x3 neighborhood remain).
The next step is rejecting the corners with the
minimal eigenvalue less than `quality_level`•max(`eig_image`(x,y)). Finally,
the function ensures that all the corners found are distanced enough one from
another by considering the corners (the most strongest corners are considered first)
and checking that the distance between the newly considered feature and the features considered earlier
is larger than `min_distance`. So, the function removes the features than are too close
to the stronger features.

---

### ExtractSURF

Extracts Speeded Up Robust Features from image

```
void cvExtractSURF( const CvArr* image, const CvArr* mask,
                    CvSeq** keypoints, CvSeq** descriptors,
                    CvMemStorage* storage, CvSURFParams params );
```

image: The input 8-bit grayscale image. mask: The optional input 8-bit mask. The features are only found in the areas that contain more than 50% of non-zero mask pixels. keypoints: The output parameter; double pointer to the sequence of keypoints. This will be the sequence of `CvSURFPoint` structures: ``` typedef struct CvSURFPoint { CvPoint2D32f pt; // position of the feature within the image int laplacian; // -1, 0 or +1. sign of the laplacian at the point. // can be used to speedup feature comparison // (normally features with laplacians of different signs can not match) int size; // size of the feature float dir; // orientation of the feature: 0..360 degrees float hessian; // value of the hessian (can be used to approximately estimate the feature strengths; // see also params.hessianThreshold) } CvSURFPoint; ``` descriptors: The optional output parameter; double pointer to the sequence of descriptors; Depending on the `params.extended` value, each element of the sequence will be either 64-element or 128-element floating-point (CV\_32F) vector. If the parameter is NULL, the descriptors are not computed. storage: Memory storage where keypoints and descriptors will be stored. params: Various algorithm parameters put to the structure `CvSURFParams`: ``` typedef struct CvSURFParams { int extended; // 0 means basic descriptors (64 elements each), // 1 means extended descriptors (128 elements each) double hessianThreshold; // only features with keypoint.hessian larger than that are extracted. // good default value is ~300-500 (can depend on the average // local contrast and sharpness of the image). // user can further filter out some features based on their hessian values // and other characteristics int nOctaves; // the number of octaves to be used for extraction. // With each next octave the feature size is doubled (3 by default) int nOctaveLayers; // The number of layers within each octave (4 by default) } CvSURFParams; CvSURFParams cvSURFParams(double hessianThreshold, int extended=0); // returns default parameters ```

The function `cvExtractSURF` finds robust features in the image, as described in
[Bay06]. For each feature it returns its location, size, orientation
and optionally the descriptor, basic or extended. The function can be used for object tracking
and localization, image stitching etc. See find\_obj.cpp demo in OpenCV samples directory.

---

## Sampling, Interpolation and Geometrical Transforms

---

### SampleLine

Reads raster line to buffer

```
int cvSampleLine( const CvArr* image, CvPoint pt1, CvPoint pt2,
                  void* buffer, int connectivity=8 );
```

image: Image to sample the line from. pt1: Starting the line point. pt2: Ending the line point. buffer: Buffer to store the line points; must have enough size to store max( |`pt2.x`-`pt1.x`|+1, |`pt2.y`-`pt1.y`|+1 ) points in case of 8-connected line and |`pt2.x`-`pt1.x`|+|`pt2.y`-`pt1.y`|+1 in case of 4-connected line. connectivity: The line connectivity, 4 or 8.

The function `cvSampleLine` implements a particular case of application of line
iterators. The function reads all the image points lying on the line between `pt1`
and `pt2`, including the ending points, and stores them into the buffer.

---

### GetRectSubPix

Retrieves pixel rectangle from image with sub-pixel accuracy

```
void cvGetRectSubPix( const CvArr* src, CvArr* dst, CvPoint2D32f center );
```

src: Source image. dst: Extracted rectangle. center: Floating point coordinates of the extracted rectangle center within the source image. The center must be inside the image.

The function `cvGetRectSubPix` extracts pixels from `src`:

```
dst(x, y) = src(x + center.x - (width(dst)-1)*0.5, y + center.y - (height(dst)-1)*0.5)
```

where the values of pixels at non-integer coordinates are
retrieved using bilinear interpolation. Every channel of multiple-channel images is processed
independently.
Whereas the rectangle center must be inside the image, the whole rectangle may be partially occluded.
In this case, the replication border mode is used to get pixel values beyond the image boundaries.

---

### GetQuadrangleSubPix

Retrieves pixel quadrangle from image with sub-pixel accuracy

```
void cvGetQuadrangleSubPix( const CvArr* src, CvArr* dst, const CvMat* map_matrix );
```

src: Source image. dst: Extracted quadrangle. map\_matrix: The transformation 2 × 3 matrix [`A`|`b`] (see the discussion).

The function `cvGetQuadrangleSubPix` extracts pixels from `src` at sub-pixel accuracy
and stores them to `dst` as follows:

```
dst(x, y)= src( A11x'+A12y'+b1, A21x'+A22y'+b2),

where A and b are taken from map_matrix
             | A11 A12  b1 |
map_matrix = |            |
             | A21 A22  b2 |,

x'=x-(width(dst)-1)*0.5, y'=y-(height(dst)-1)*0.5
```

where the values of pixels at non-integer coordinates A•(x,y)^T^+b are
retrieved using bilinear interpolation. When the function needs pixels outside of the image, it uses replication
border mode to reconstruct the values. Every channel of multiple-channel images is processed
independently.

---

### Resize

Resizes image

```
void cvResize( const CvArr* src, CvArr* dst, int interpolation=CV_INTER_LINEAR );
```

src: Source image. dst: Destination image. interpolation: Interpolation method: - CV\_INTER\_NN - nearest-neighbor interpolation,- CV\_INTER\_LINEAR - bilinear interpolation (used by default)- CV\_INTER\_AREA - resampling using pixel area relation. It is the preferred method for image decimation that gives moire-free results. In case of zooming it is similar to `CV_INTER_NN` method.- CV\_INTER\_CUBIC - bicubic interpolation.

The function `cvResize` resizes image `src` (or its ROI)
so that it fits exactly to `dst` (or its ROI).

---

### WarpAffine

Applies affine transformation to the image

```
void cvWarpAffine( const CvArr* src, CvArr* dst, const CvMat* map_matrix,
                   int flags=CV_INTER_LINEAR+CV_WARP_FILL_OUTLIERS,
                   CvScalar fillval=cvScalarAll(0) );
```

src: Source image. dst: Destination image. map\_matrix: 2×3 transformation matrix. flags: A combination of interpolation method and the following optional flags: - CV\_WARP\_FILL\_OUTLIERS - fill all the destination image pixels. If some of them correspond to outliers in the source image, they are set to `fillval`.- CV\_WARP\_INVERSE\_MAP - indicates that `matrix` is inverse transform from destination image to source and, thus, can be used directly for pixel interpolation. Otherwise, the function finds the inverse transform from `map_matrix`. fillval: A value used to fill outliers.

The function `cvWarpAffine` transforms source image using the specified
matrix:

```
dst(x’,y’)<-src(x,y)
(x’,y’)T=map_matrix•(x,y,1)T+b if CV_WARP_INVERSE_MAP is not set,
(x, y)T=map_matrix•(x’,y',1)T+b otherwise
```

The function is similar to cvGetQuadrangleSubPix but they are
not exactly the same. cvWarpAffine requires input and output
image have the same data type, has larger overhead (so it is not quite suitable for small images)
and can leave part of destination image unchanged. While cvGetQuadrangleSubPix
may extract quadrangles from 8-bit images into floating-point buffer, has smaller overhead and
always changes the whole destination image content.

To transform a sparse set of points, use cvTransform
function from cxcore.

---

### GetAffineTransform

Calculates affine transform from 3 corresponding points

```
CvMat* cvGetAffineTransform( const CvPoint2D32f* src, const CvPoint2D32f* dst, 
                             CvMat* map_matrix );
```

src: Coordinates of 3 triangle vertices in the source image. dst: Coordinates of the 3 corresponding triangle vertices in the destination image. map\_matrix: Pointer to the destination 2×3 matrix.

The function `cvGetAffineTransform` calculates the
matrix of an affine transform such that:

```
(x'i,y'i)T=map_matrix•(xi,yi,1)T
```

where `dst(i)=(x'i,y'i), src(i)=(xi,yi), i=0..2`.

---

### 2DRotationMatrix

Calculates affine matrix of 2d rotation

```
CvMat* cv2DRotationMatrix( CvPoint2D32f center, double angle,
                           double scale, CvMat* map_matrix );
```

center: Center of the rotation in the source image. angle: The rotation angle in degrees. Positive values mean counter-clockwise rotation (the coordinate origin is assumed at top-left corner). scale: Isotropic scale factor. map\_matrix: Pointer to the destination 2×3 matrix.

The function `cv2DRotationMatrix` calculates matrix:

```
[  α  β  |  (1-α)*center.x - β*center.y ]
[ -β  α  |  β*center.x + (1-α)*center.y ]

where α=scale*cos(angle), β=scale*sin(angle)
```

The transformation maps the rotation center to itself. If this is not the purpose,
the shift should be adjusted.

---

### WarpPerspective

Applies perspective transformation to the image

```
void cvWarpPerspective( const CvArr* src, CvArr* dst, const CvMat* map_matrix,
                        int flags=CV_INTER_LINEAR+CV_WARP_FILL_OUTLIERS,
                        CvScalar fillval=cvScalarAll(0) );
```

src: Source image. dst: Destination image. map\_matrix: 3×3 transformation matrix. flags: A combination of interpolation method and the following optional flags: - CV\_WARP\_FILL\_OUTLIERS - fill all the destination image pixels. If some of them correspond to outliers in the source image, they are set to `fillval`.- CV\_WARP\_INVERSE\_MAP - indicates that `matrix` is inverse transform from destination image to source and, thus, can be used directly for pixel interpolation. Otherwise, the function finds the inverse transform from `map_matrix`. fillval: A value used to fill outliers.

The function `cvWarpPerspective` transforms source image using
the specified matrix:

```
dst(x’,y’)<-src(x,y)
(t•x’,t•y’,t)T=map_matrix•(x,y,1)T+b if CV_WARP_INVERSE_MAP is not set,
(t•x, t•y, t)T=map_matrix•(x’,y',1)T+b otherwise
```

For a sparse set of points
use cvPerspectiveTransform function from cxcore.

---

### GetPerspectiveTransform

Calculates perspective transform from 4 corresponding points

```
CvMat* cvGetPerspectiveTransform( const CvPoint2D32f* src, const CvPoint2D32f* dst,
                                  CvMat* map_matrix );

#define cvWarpPerspectiveQMatrix cvGetPerspectiveTransform
```

src: Coordinates of 4 quadrangle vertices in the source image. dst: Coordinates of the 4 corresponding quadrangle vertices in the destination image. map\_matrix: Pointer to the destination 3×3 matrix.

The function `cvGetPerspectiveTransform` calculates
matrix of perspective transform such that:

```
(ti•x'i,ti•y'i,ti)T=map_matrix•(xi,yi,1)T
```

where `dst(i)=(x'i,y'i), src(i)=(xi,yi), i=0..3`.

---

### Remap

Applies generic geometrical transformation to the image

```
void cvRemap( const CvArr* src, CvArr* dst,
              const CvArr* mapx, const CvArr* mapy,
              int flags=CV_INTER_LINEAR+CV_WARP_FILL_OUTLIERS,
              CvScalar fillval=cvScalarAll(0) );
```

src: Source image. dst: Destination image. mapx: The map of x-coordinates (32FC1 image). mapy: The map of y-coordinates (32FC1 image). flags: A combination of interpolation method and the following optional flag(s): - CV\_WARP\_FILL\_OUTLIERS - fill all the destination image pixels. If some of them correspond to outliers in the source image, they are set to `fillval`. fillval: A value used to fill outliers.

The function `cvRemap` transforms source image using
the specified map:

```
dst(x,y)<-src(mapx(x,y),mapy(x,y))
```

Similar to other geometrical transformations, some interpolation method (specified by user)
is used to extract pixels with non-integer coordinates.

---

### LogPolar

Remaps image to log-polar space

```
void cvLogPolar( const CvArr* src, CvArr* dst,
                 CvPoint2D32f center, double M,
                 int flags=CV_INTER_LINEAR+CV_WARP_FILL_OUTLIERS );
```

src: Source image. dst: Destination image. center: The transformation center, where the output precision is maximal. M: Magnitude scale parameter. See below. flags: A combination of interpolation method and the following optional flags: - CV\_WARP\_FILL\_OUTLIERS - fill all the destination image pixels. If some of them correspond to outliers in the source image, they are set to zeros.- CV\_WARP\_INVERSE\_MAP - indicates that `matrix` is inverse transform from destination image to source and, thus, can be used directly for pixel interpolation. Otherwise, the function finds the inverse transform from `map_matrix`. fillval: A value used to fill outliers.

The function `cvLogPolar` transforms source image using
the following transformation:

```
Forward transformation (CV_WARP_INVERSE_MAP is not set):
dst(phi,rho)<-src(x,y)

Inverse transformation (CV_WARP_INVERSE_MAP is set):
dst(x,y)<-src(phi,rho),

where rho=M*log(sqrt(x2+y2))
      phi=atan(y/x)
```

The function emulates the human "foveal" vision and can be used for fast scale and rotation-invariant
template matching, for object tracking etc.

#### Example. Log-polar transformation.

```
#include <cv.h>
#include <highgui.h>

int main(int argc, char** argv)
{
    IplImage* src;

    if( argc == 2 && (src=cvLoadImage(argv[1],1) != 0 )
    {
        IplImage* dst = cvCreateImage( cvSize(256,256), 8, 3 );
        IplImage* src2 = cvCreateImage( cvGetSize(src), 8, 3 );
        cvLogPolar( src, dst, cvPoint2D32f(src->width/2,src->height/2), 40, CV_INTER_LINEAR+CV_WARP_FILL_OUTLIERS );
        cvLogPolar( dst, src2, cvPoint2D32f(src->width/2,src->height/2), 40, CV_INTER_LINEAR+CV_WARP_FILL_OUTLIERS+CV_WARP_INVERSE_MAP );
        cvNamedWindow( "log-polar", 1 );
        cvShowImage( "log-polar", dst );
        cvNamedWindow( "inverse log-polar", 1 );
        cvShowImage( "inverse log-polar", src2 );
        cvWaitKey();
    }
    return 0;
}
```

And this is what the program displays when opencv/samples/c/fruits.jpg is passed to it

---

## Morphological Operations

---

### CreateStructuringElementEx

Creates structuring element

```
IplConvKernel* cvCreateStructuringElementEx( int cols, int rows, int anchor_x, int anchor_y,
                                             int shape, int* values=NULL );
```

cols: Number of columns in the structuring element. rows: Number of rows in the structuring element. anchor\_x: Relative horizontal offset of the anchor point. anchor\_y: Relative vertical offset of the anchor point. shape: Shape of the structuring element; may have the following values: - `CV_SHAPE_RECT`, a rectangular element;- `CV_SHAPE_CROSS`, a cross-shaped element;- `CV_SHAPE_ELLIPSE`, an elliptic element;- `CV_SHAPE_CUSTOM`, a user-defined element. In this case the parameter `values` specifies the mask, that is, which neighbors of the pixel must be considered. values: Pointer to the structuring element data, a plane array, representing row-by-row scanning of the element matrix. Non-zero values indicate points that belong to the element. If the pointer is `NULL`, then all values are considered non-zero, that is, the element is of a rectangular shape. This parameter is considered only if the shape is `CV_SHAPE_CUSTOM` .

The function cv CreateStructuringElementEx allocates and fills the structure
 `IplConvKernel`, which can be used as a structuring element in the morphological
operations.

---

### ReleaseStructuringElement

Deletes structuring element

```
void cvReleaseStructuringElement( IplConvKernel** element );
```

element: Pointer to the deleted structuring element.

The function `cvReleaseStructuringElement` releases the structure `IplConvKernel`
that is no longer needed. If `*element` is `NULL`, the function has no effect.

---

### Erode

Erodes image by using arbitrary structuring element

```
void cvErode( const CvArr* src, CvArr* dst, IplConvKernel* element=NULL, int iterations=1 );
```

src: Source image. dst: Destination image. element: Structuring element used for erosion. If it is `NULL`, a 3×3 rectangular structuring element is used. iterations: Number of times erosion is applied.

The function `cvErode` erodes the source image using the specified structuring element
that determines the shape of a pixel neighborhood over which the minimum is taken:

```
dst=erode(src,element):  dst(x,y)=min((x',y') in element))src(x+x',y+y')
```

The function supports the in-place mode. Erosion can be applied several (`iterations`)
times. In case of color image each channel is processed independently.

---

### Dilate

Dilates image by using arbitrary structuring element

```
void cvDilate( const CvArr* src, CvArr* dst, IplConvKernel* element=NULL, int iterations=1 );
```

src: Source image. dst: Destination image. element: Structuring element used for erosion. If it is `NULL`, a 3×3 rectangular structuring element is used. iterations: Number of times erosion is applied.

The function `cvDilate` dilates the source image using the specified structuring element
that determines the shape of a pixel neighborhood over which the maximum is taken:

```
dst=dilate(src,element):  dst(x,y)=max((x',y') in element))src(x+x',y+y')
```

The function supports the in-place mode. Dilation can be applied several (`iterations`)
times. In case of color image each channel is processed independently.

---

### MorphologyEx

Performs advanced morphological transformations

```
void cvMorphologyEx( const CvArr* src, CvArr* dst, CvArr* temp,
                     IplConvKernel* element, int operation, int iterations=1 );
```

src: Source image. dst: Destination image. temp: Temporary image, required in some cases. element: Structuring element. operation: Type of morphological operation, one of: `CV_MOP_OPEN` - opening `CV_MOP_CLOSE` - closing `CV_MOP_GRADIENT` - morphological gradient `CV_MOP_TOPHAT` - "top hat" `CV_MOP_BLACKHAT` - "black hat" iterations: Number of times erosion and dilation are applied.

The function `cvMorphologyEx` can perform advanced morphological
transformations using erosion and dilation as basic operations.

```
Opening:
dst=open(src,element)=dilate(erode(src,element),element)

Closing:
dst=close(src,element)=erode(dilate(src,element),element)

Morphological gradient:
dst=morph_grad(src,element)=dilate(src,element)-erode(src,element)

"Top hat":
dst=tophat(src,element)=src-open(src,element)

"Black hat":
dst=blackhat(src,element)=close(src,element)-src
```

The temporary image `temp` is required for morphological gradient and, in case of in-place
operation, for "top hat" and "black hat".

---

## Filters and Color Conversion

---

### Smooth

Smoothes the image in one of several ways

```
void cvSmooth( const CvArr* src, CvArr* dst,
               int smoothtype=CV_GAUSSIAN,
               int size1=3, int size2=0, double sigma1=0, double sigma2=0 );
```

src: The source image. dst: The destination image. smoothtype: Type of the smoothing operation: - CV\_BLUR\_NO\_SCALE (simple blur with no scaling) - for each pixel the result is a sum of pixels values in `size1`×`size2` neighborhood of the pixel. If the neighborhood size varies from pixel to pixel, compute the sums using integral image (cvIntegral).- CV\_BLUR (simple blur) - for each pixel the result is the average value (brightness/color) of `size1`×`size2` neighborhood of the pixel.- CV\_GAUSSIAN (Gaussian blur) - the image is smoothed using the Gaussian kernel of aperture size `size1`×`size2`. `sigma1` and `sigma2` may optionally be used to specify shape of the kernel.- CV\_MEDIAN (median blur) - the image is smoothed using medial filter of size `size1`×`size1` (i.e. only square aperture can be used). That is, for each pixel the result is the median computed over `size1`×`size1` neighborhood.- CV\_BILATERAL (bilateral filter) - the image is smoothed using a bilateral 3x3 filter with color sigma=`sigma1` and spatial sigma=`sigma2`. If `size1!=0`, then a circular kernel with diameter `size1` is used; otherwise the diameter of the kernel is computed from `sigma2`. Information about bilateral filtering can be found at http://www.dai.ed.ac.uk/CVonline/LOCAL\_COPIES/MANDUCHI1/Bilateral\_Filtering.html size1: The first parameter of smoothing operation. It should be odd (1, 3, 5, ...), so that a pixel neighborhood used for smoothing operation is symmetrical relative to the pixel. size2: The second parameter of smoothing operation. In case of simple scaled/non-scaled and Gaussian blur if `size2` is zero, it is set to `size1`. When not 0, it should be odd too. sigma1: In case of Gaussian kernel this parameter may specify Gaussian sigma (standard deviation). If it is zero, it is calculated from the kernel size: ``` sigma = (n/2 - 1)*0.3 + 0.8, where n=param1 for horizontal kernel, n=param2 for vertical kernel. ``` With the standard sigma for small kernels (3×3 to 7×7) the performance is better. If `param3` is not zero, while `param1` and `param2` are zeros, the kernel size is calculated from the sigma (to provide accurate enough operation). In case of Bilateral filter the parameter specifies color sigma; the larger the value, the stronger the pasterization effect of the filter is. sigma2: In case of non-square Gaussian kernel the parameter may be used to specify a different (from `param3`) sigma in the vertical direction. In case of Bilateral filter the parameter specifies spatial sigma; the larger the value, the stronger the blurring effect of the filter. Note that with large `sigma2` the processing speed decreases substantionally, so it is recommended to limit the kernel size using the parameter `size1`.

The function `cvSmooth` smoothes image using one of the pre-defined methods. Every of the methods
has some features and restrictions listed below:

- Blur with no scaling works with single-channel images only and supports accumulation of
  8u to 16s format (similar to cvSobel and cvLaplace)
  and accumulation of 32f to 32f format.
- Simple blur and Gaussian blur support 1- or 3-channel, 8-bit, 16-bit and 32-bit floating-point images.
  These two methods can process images in-place.
- Median filter works with 1- or 3-channel 8-bit images and can not process images in-place.
- Bilateral filter works with 1- or 3-channel, 8-bit or 32-bit floating-point images and can not process images in-place.

---

### Filter2D

Applies linear filter to image

```
void cvFilter2D( const CvArr* src, CvArr* dst,
                 const CvMat* kernel,
                 CvPoint anchor=cvPoint(-1,-1));
```

src: The source image. dst: The destination image. kernel: The filter mask, single-channel 2d floating point matrix of coefficients. In case of multi-channel images every channel is processed independently using the same kernel. To process different channels differently, split the image using cvSplit and filter the channels one by one. anchor: The anchor of the kernel. It is relative position of the filtered pixel inside its neighborhood covered by the kernel mask. The anchor should be inside the kernel. The special default value (-1,-1) means that the anchor is at the kernel center.

The function `cvFilter2D` applies the specified linear filter to the image.
In-place operation is supported. When the aperture is partially outside the image,
the function interpolates outlier pixel values from the nearest pixels at the
image boundary. If ROI is set in the input
image, cvFilter2D treats it, similarly to many other OpenCV functions, as if
it were an isolated image, i.e. pixels inside the image but outside of the ROI
are ignored. If it is undesirable, consider new C++ filtering classes declared
in cv.hpp.

---

### CopyMakeBorder

Copies image and makes border around it

```
void cvCopyMakeBorder( const CvArr* src, CvArr* dst, CvPoint offset,
                       int bordertype, CvScalar value=cvScalarAll(0) );
```

src: The source image. dst: The destination image. offset: Coordinates of the top-left corner (or bottom-left in case of images with bottom-left origin) of the destination image rectangle where the source image (or its ROI) is copied. Size of the rectangle matches the source image size/ROI size. bordertype: Type of the border to create around the copied source image rectangle: `IPL_BORDER_CONSTANT` - border is filled with the fixed value, passed as last parameter of the function. `IPL_BORDER_REPLICATE` - the pixels from the top and bottom rows, the left-most and right-most columns are replicated to fill the border. (The other two border types from IPL, `IPL_BORDER_REFLECT` and `IPL_BORDER_WRAP`, are currently unsupported). value: Value of the border pixels if `bordertype=IPL_BORDER_CONSTANT`.

The function `cvCopyMakeBorder` copies the source 2D array into interior of destination array
and makes a border of the specified type around the copied area.
The function is useful when one needs to emulate border type that is different from the one embedded into a specific
algorithm implementation. For example, morphological functions, as well as most of other filtering functions in OpenCV,
internally use replication border type, while the user may need zero border or a border, filled with 1's or 255's.

---

### Integral

Calculates integral images

```
void cvIntegral( const CvArr* image, CvArr* sum, CvArr* sqsum=NULL, CvArr* tilted_sum=NULL );
```

image: The source image, `W`×`H`, 8-bit or floating-point (32f or 64f) image. sum: The integral image, `W+1`×`H+1`, 32-bit integer or double precision floating-point (64f). sqsum: The optional integral image for squared pixel values, `W+1`×`H+1`, double precision floating-point (64f). tilted\_sum: The optional integral for the image rotated by 45 degrees, `W+1`×`H+1`, of the same data type as `sum`.

The function `cvIntegral` calculates one or more integral images for the source image as following:

```
sum(X,Y)=sumx<X,y<Yimage(x,y)

sqsum(X,Y)=sumx<X,y<Yimage(x,y)2

tilted_sum(X,Y)=sumy<Y,abs(x-X)<yimage(x,y)
```

Using these integral images, one may calculate sum, mean, standard deviation over
arbitrary up-right or rotated rectangular region of the image in a constant time, for example:

```
sumx1<=x<x2,y1<=y<y2image(x,y)=sum(x2,y2)-sum(x1,y2)-sum(x2,y1)+sum(x1,x1)
```

Using integral images it is possible to do variable-size image blurring,
block correlation etc.
In case of multi-channel input images the integral images must have
the same number of channels, and every channel is processed independently.

---

### CvtColor

Converts image from one color space to another

```
void cvCvtColor( const CvArr* src, CvArr* dst, int code );
```

src: The source 8-bit (8u), 16-bit (16u) or single-precision floating-point (32f) image. dst: The destination image of the same data type as the source one. The number of channels may be different. code: Color conversion operation that can be specified using CV\_<src\_color\_space>2<dst\_color\_space> constants (see below).

The function `cvCvtColor` converts input image from one color space to another.
The function ignores `colorModel` and `channelSeq` fields of `IplImage` header,
so the source image color space should be specified correctly (including order of the channels in case
of RGB space, e.g. BGR means 24-bit format with B_0_ G_0_ R_0_ B_1_ G_1_ R_1_ ... layout,
whereas RGB means 24-bit format with R_0_ G_0_ B_0_ R_1_ G_1_ B_1_ ... layout).

The conventional range for R,G,B channel values is:

- 0..255 for 8-bit images- 0..65535 for 16-bit images and- 0..1 for floating-point images.

Of course, in case of linear transformations the range can be arbitrary,
but in order to get correct results in case of non-linear transformations,
the input image should be scaled if necessary.

The function can do the following transformations:

- Transformations within RGB space like adding/removing alpha channel, reversing the channel order,
  conversion to/from 16-bit RGB color (R5:G6:B5 or R5:G5:B5) color, as well as conversion to/from grayscale using:

  ```
  RGB[A]->Gray: Y<-0.299*R + 0.587*G + 0.114*B
  Gray->RGB[A]: R<-Y G<-Y B<-Y A<-0
  ```

  - RGB<=>CIE XYZ.Rec 709 with D65 white point (`CV_BGR2XYZ, CV_RGB2XYZ, CV_XYZ2BGR, CV_XYZ2RGB`):

    ```
    |X|    |0.412453  0.357580  0.180423| |R|
    |Y| <- |0.212671  0.715160  0.072169|*|G|
    |Z|    |0.019334  0.119193  0.950227| |B|

    |R|    | 3.240479  -1.53715  -0.498535| |X|
    |G| <- |-0.969256   1.875991  0.041556|*|Y|
    |B|    | 0.055648  -0.204043  1.057311| |Z|

    X, Y and Z cover the whole value range (in case of floating-point images Z may exceed 1).
    ```

    - RGB<=>YCrCb JPEG (a.k.a. YCC) (`CV_BGR2YCrCb, CV_RGB2YCrCb, CV_YCrCb2BGR, CV_YCrCb2RGB`)

      ```
      Y <- 0.299*R + 0.587*G + 0.114*B
      Cr <- (R-Y)*0.713 + delta
      Cb <- (B-Y)*0.564 + delta

      R <- Y + 1.403*(Cr - delta)
      G <- Y - 0.344*(Cr - delta) - 0.714*(Cb - delta)
      B <- Y + 1.773*(Cb - delta),

                    { 128 for 8-bit images,
      where delta = { 32768 for 16-bit images
                    { 0.5 for floating-point images

      Y, Cr and Cb cover the whole value range.
      ```

      - RGB<=>HSV (`CV_BGR2HSV, CV_RGB2HSV, CV_HSV2BGR, CV_HSV2RGB`)

        ```
        // In case of 8-bit and 16-bit images
        // R, G and B are converted to floating-point format and scaled to fit 0..1 range

        V <- max(R,G,B)
        S <- (V-min(R,G,B))/V   if V≠0, 0 otherwise

                 (G - B)*60/S,  if V=R
        H <- 180+(B - R)*60/S,  if V=G
             240+(R - G)*60/S,  if V=B

        if H<0 then H<-H+360

        On output 0≤V≤1, 0≤S≤1, 0≤H≤360.
        The values are then converted to the destination data type:
            8-bit images:
                V <- V*255, S <- S*255, H <- H/2 (to fit to 0..255)
            16-bit images (currently not supported):
                V <- V*65535, S <- S*65535, H <- H
            32-bit images:
                H, S, V are left as is
        ```

        - RGB<=>HLS (`CV_BGR2HLS, CV_RGB2HLS, CV_HLS2BGR, CV_HLS2RGB`)

          ```
          // In case of 8-bit and 16-bit images
          // R, G and B are converted to floating-point format and scaled to fit 0..1 range

          Vmax <- max(R,G,B)
          Vmin <- min(R,G,B)

          L <- (Vmax + Vmin)/2

          S <- (Vmax - Vmin)/(Vmax + Vmin)  if L < 0.5
               (Vmax - Vmin)/(2 - (Vmax + Vmin))  if L ≥ 0.5

                   (G - B)*60/S,  if Vmax=R
          H <- 180+(B - R)*60/S,  if Vmax=G
               240+(R - G)*60/S,  if Vmax=B

          if H<0 then H<-H+360

          On output 0≤L≤1, 0≤S≤1, 0≤H≤360.
          The values are then converted to the destination data type:
              8-bit images:
                  L <- L*255, S <- S*255, H <- H/2
              16-bit images (currently not supported):
                  L <- L*65535, S <- S*65535, H <- H
              32-bit images:
                  H, L, S are left as is
          ```

          - RGB<=>CIE L\*a\*b\* (`CV_BGR2Lab, CV_RGB2Lab, CV_Lab2BGR, CV_Lab2RGB`)

            ```
            // In case of 8-bit and 16-bit images
            // R, G and B are converted to floating-point format and scaled to fit 0..1 range

            // convert R,G,B to CIE XYZ
            |X|    |0.412453  0.357580  0.180423| |R|
            |Y| <- |0.212671  0.715160  0.072169|*|G|
            |Z|    |0.019334  0.119193  0.950227| |B|

            X <- X/Xn, where Xn = 0.950456
            Z <- Z/Zn, where Zn = 1.088754

            L <- 116*Y1/3      for Y>0.008856
            L <- 903.3*Y      for Y<=0.008856

            a <- 500*(f(X)-f(Y)) + delta
            b <- 200*(f(Y)-f(Z)) + delta
            where f(t)=t1/3              for t>0.008856
                  f(t)=7.787*t+16/116   for t<=0.008856


            where delta = 128 for 8-bit images,
                          0 for floating-point images

            On output 0≤L≤100, -127≤a≤127, -127≤b≤127
            The values are then converted to the destination data type:
                8-bit images:
                    L <- L*255/100, a <- a + 128, b <- b + 128
                16-bit images are currently not supported
                32-bit images:
                    L, a, b are left as is
            ```

            - RGB<=>CIE L\*u\*v\* (`CV_BGR2Luv, CV_RGB2Luv, CV_Luv2BGR, CV_Luv2RGB`)

              ```
              // In case of 8-bit and 16-bit images
              // R, G and B are converted to floating-point format and scaled to fit 0..1 range

              // convert R,G,B to CIE XYZ
              |X|    |0.412453  0.357580  0.180423| |R|
              |Y| <- |0.212671  0.715160  0.072169|*|G|
              |Z|    |0.019334  0.119193  0.950227| |B|

              L <- 116*Y1/3-16   for Y>0.008856
              L <- 903.3*Y      for Y<=0.008856

              u' <- 4*X/(X + 15*Y + 3*Z)
              v' <- 9*Y/(X + 15*Y + 3*Z)

              u <- 13*L*(u' - un), where un=0.19793943
              v <- 13*L*(v' - vn), where vn=0.46831096

              On output 0≤L≤100, -134≤u≤220, -140≤v≤122
              The values are then converted to the destination data type:
                  8-bit images:
                      L <- L*255/100, u <- (u + 134)*255/354, v <- (v + 140)*255/256
                  16-bit images are currently not supported
                  32-bit images:
                      L, u, v are left as is
              ```

              The above formulae for converting RGB to/from various color spaces have been taken
              from multiple sources on Web, primarily from Color Space Conversions (**[Ford98]**)
              document at Charles Poynton site.- Bayer=>RGB (`CV_BayerBG2BGR, CV_BayerGB2BGR, CV_BayerRG2BGR, CV_BayerGR2BGR,  
                CV_BayerBG2RGB, CV_BayerGB2RGB, CV_BayerRG2RGB, CV_BayerGR2RGB`)

                Bayer pattern is widely used in CCD and CMOS cameras. It allows to get color picture
                out of a single plane where R,G and B pixels (sensors of a particular component) are interleaved like
                this:

                |  |  |  |  |  |
                | --- | --- | --- | --- | --- |
                | R | G | R | G | R |
                | G | B | G | B | G |
                | R | G | R | G | R |
                | G | B | G | B | G |
                | R | G | R | G | R |
                | G | B | G | B | G |

                The output RGB components of a pixel are interpolated from 1, 2 or 4 neighbors of the pixel
                having the same color. There are several modifications of the above pattern that can be achieved
                by shifting the pattern one pixel left and/or one pixel up.
                The two letters C_1_ and C_2_ in the conversion constants CV\_BayerC_1_C_2_2{BGR|RGB}
                indicate the particular pattern type -
                these are components from the second row, second and third columns, respectively.
                For example, the above pattern has very popular "BG" type.

---

### Threshold

Applies fixed-level threshold to array elements

```
double cvThreshold( const CvArr* src, CvArr* dst, double threshold,
                    double max_value, int threshold_type );
```

src: Source array (single-channel, 8-bit of 32-bit floating point). dst: Destination array; must be either the same type as `src` or 8-bit. threshold: Threshold value. max\_value: Maximum value to use with `CV_THRESH_BINARY` and `CV_THRESH_BINARY_INV` thresholding types. threshold\_type: Thresholding type (see the discussion)

The function `cvThreshold` applies fixed-level thresholding to single-channel array.
The function is typically used to get bi-level (binary) image out of grayscale image
(cvCmpS
could be also used for this purpose) or for removing a noise, i.e. filtering out pixels with too small or too large values.
There are several types of thresholding the function supports that are determined by `threshold_type`:

```
threshold_type=CV_THRESH_BINARY:
dst(x,y) = max_value, if src(x,y)>threshold
           0, otherwise

threshold_type=CV_THRESH_BINARY_INV:
dst(x,y) = 0, if src(x,y)>threshold
           max_value, otherwise

threshold_type=CV_THRESH_TRUNC:
dst(x,y) = threshold, if src(x,y)>threshold
           src(x,y), otherwise

threshold_type=CV_THRESH_TOZERO:
dst(x,y) = src(x,y), if src(x,y)>threshold
           0, otherwise

threshold_type=CV_THRESH_TOZERO_INV:
dst(x,y) = 0, if src(x,y)>threshold
           src(x,y), otherwise
```

And this is the visual description of thresholding types:

Also, the special value `CV_THRESH_OTSU` may be combined with
one of the above values. In this case the function determines the optimal threshold
value using Otsu algorithm and uses it instead of the specified `thresh`.
The function returns the computed threshold value.
Currently, Otsu method is implemented only for 8-bit images.

---

### AdaptiveThreshold

Applies adaptive threshold to array

```
void cvAdaptiveThreshold( const CvArr* src, CvArr* dst, double max_value,
                          int adaptive_method=CV_ADAPTIVE_THRESH_MEAN_C,
                          int threshold_type=CV_THRESH_BINARY,
                          int block_size=3, double param1=5 );
```

src: Source image. dst: Destination image. max\_value: Maximum value that is used with `CV_THRESH_BINARY` and `CV_THRESH_BINARY_INV`. adaptive\_method: Adaptive thresholding algorithm to use: `CV_ADAPTIVE_THRESH_MEAN_C` or `CV_ADAPTIVE_THRESH_GAUSSIAN_C` (see the discussion). threshold\_type: Thresholding type; must be one of - `CV_THRESH_BINARY,`- `CV_THRESH_BINARY_INV` block\_size: The size of a pixel neighborhood that is used to calculate a threshold value for the pixel: 3, 5, 7, ... param1: The method-dependent parameter. For the methods `CV_ADAPTIVE_THRESH_MEAN_C` and `CV_ADAPTIVE_THRESH_GAUSSIAN_C` it is a constant subtracted from mean or weighted mean (see the discussion), though it may be negative.

The function `cvAdaptiveThreshold` transforms grayscale image to binary image according to
the formulae:

```
threshold_type=CV_THRESH_BINARY:
dst(x,y) = max_value, if src(x,y)>T(x,y)
           0, otherwise

threshold_type=CV_THRESH_BINARY_INV:
dst(x,y) = 0, if src(x,y)>T(x,y)
           max_value, otherwise
```

where T_I_ is a threshold calculated individually for each pixel.

For the method `CV_ADAPTIVE_THRESH_MEAN_C` it is a mean of `block_size` × `block_size`
pixel neighborhood, subtracted by `param1`.

For the method `CV_ADAPTIVE_THRESH_GAUSSIAN_C` it is a weighted sum (Gaussian) of
`block_size` × `block_size` pixel neighborhood, subtracted by `param1`.

---

## Pyramids and the Applications

---

### PyrDown

Downsamples image

```
void cvPyrDown( const CvArr* src, CvArr* dst, int filter=CV_GAUSSIAN_5x5 );
```

src: The source image. dst: The destination image, should have 2x smaller width and height than the source. filter: Type of the filter used for convolution; only `CV_GAUSSIAN_5x5` is currently supported.

The function `cvPyrDown` performs downsampling step of Gaussian pyramid
decomposition. First it convolves source image with the specified filter and
then downsamples the image by rejecting even rows and columns.

---

### PyrUp

Upsamples image

```
void cvPyrUp( const CvArr* src, CvArr* dst, int filter=CV_GAUSSIAN_5x5 );
```

src: The source image. dst: The destination image, should have 2x smaller width and height than the source. filter: Type of the filter used for convolution; only `CV_GAUSSIAN_5x5` is currently supported.

The function `cvPyrUp` performs up-sampling step of Gaussian pyramid decomposition.
First it upsamples the source image by injecting even zero rows and columns and
then convolves result with the specified filter multiplied by 4 for
interpolation. So the destination image is four times larger than the source
image.

---

## Image Segmentation, Connected Components and Contour Retrieval

---

### CvConnectedComp

Connected component

```
    typedef struct CvConnectedComp
    {
        double area; /* area of the segmented component */
        float value; /* gray scale value of the segmented component */
        CvRect rect; /* ROI of the segmented component */
    } CvConnectedComp;
```

---

### FloodFill

Fills a connected component with given color

```
void cvFloodFill( CvArr* image, CvPoint seed_point, CvScalar new_val,
                  CvScalar lo_diff=cvScalarAll(0), CvScalar up_diff=cvScalarAll(0),
                  CvConnectedComp* comp=NULL, int flags=4, CvArr* mask=NULL );
#define CV_FLOODFILL_FIXED_RANGE (1 << 16)
#define CV_FLOODFILL_MASK_ONLY   (1 << 17)
```

image: Input 1- or 3-channel, 8-bit or floating-point image. It is modified by the function unless CV\_FLOODFILL\_MASK\_ONLY flag is set (see below). seed\_point: The starting point. new\_val: New value of repainted domain pixels. lo\_diff: Maximal lower brightness/color difference between the currently observed pixel and one of its neighbor belong to the component or seed pixel to add the pixel to component. In case of 8-bit color images it is packed value. up\_diff: Maximal upper brightness/color difference between the currently observed pixel and one of its neighbor belong to the component or seed pixel to add the pixel to component. In case of 8-bit color images it is packed value. comp: Pointer to structure the function fills with the information about the repainted domain. flags: The operation flags. Lower bits contain connectivity value, 4 (by default) or 8, used within the function. Connectivity determines which neighbors of a pixel are considered. Upper bits can be 0 or combination of the following flags: - CV\_FLOODFILL\_FIXED\_RANGE - if set the difference between the current pixel and seed pixel is considered, otherwise difference between neighbor pixels is considered (the range is floating).- CV\_FLOODFILL\_MASK\_ONLY - if set, the function does not fill the image (`new_val` is ignored), but the fills mask (that must be non-NULL in this case). mask: Operation mask, should be singe-channel 8-bit image, 2 pixels wider and 2 pixels taller than `image`. If not NULL, the function uses and updates the mask, so user takes responsibility of initializing `mask` content. Floodfilling can't go across non-zero pixels in the mask, for example, an edge detector output can be used as a mask to stop filling at edges. Or it is possible to use the same mask in multiple calls to the function to make sure the filled area do not overlap. *Note*: because mask is larger than the filled image, pixel in `mask` that corresponds to `(x,y)` pixel in `image` will have coordinates `(x+1,y+1)`.

The function `cvFloodFill` fills a connected component starting from the seed point
with the specified color. The connectivity is determined by the closeness of pixel values.
The pixel at `(x, y)` is considered to belong to the repainted domain if:

```
src(x',y')-lo_diff<=src(x,y)<=src(x',y')+up_diff,     grayscale image, floating range
src(seed.x,seed.y)-lo<=src(x,y)<=src(seed.x,seed.y)+up_diff, grayscale image, fixed range

src(x',y')r-lo_diffr<=src(x,y)r<=src(x',y')r+up_diffr and
src(x',y')g-lo_diffg<=src(x,y)g<=src(x',y')g+up_diffg and
src(x',y')b-lo_diffb<=src(x,y)b<=src(x',y')b+up_diffb, color image, floating range

src(seed.x,seed.y)r-lo_diffr<=src(x,y)r<=src(seed.x,seed.y)r+up_diffr and
src(seed.x,seed.y)g-lo_diffg<=src(x,y)g<=src(seed.x,seed.y)g+up_diffg and
src(seed.x,seed.y)b-lo_diffb<=src(x,y)b<=src(seed.x,seed.y)b+up_diffb, color image, fixed range
```

where `src(x',y')` is value of one of pixel neighbors.
That is, to be added to the connected component, a pixel’s color/brightness should be close enough to:

- color/brightness of one of its neighbors that are already referred to the connected
  component in case of floating range- color/brightness of the seed point in case of fixed range.

---

### FindContours

Finds contours in binary image

```
int cvFindContours( CvArr* image, CvMemStorage* storage, CvSeq** first_contour,
                    int header_size=sizeof(CvContour), int mode=CV_RETR_LIST,
                    int method=CV_CHAIN_APPROX_SIMPLE, CvPoint offset=cvPoint(0,0) );
```

image: The source 8-bit single channel image. Non-zero pixels are treated as 1’s, zero pixels remain 0’s - that is image treated as `binary`. To get such a binary image from grayscale, one may use cvThreshold, cvAdaptiveThreshold or cvCanny. The function modifies the source image content. storage: Container of the retrieved contours. first\_contour: Output parameter, will contain the pointer to the first outer contour. header\_size: Size of the sequence header, >=sizeof(CvChain) if `method`=CV\_CHAIN\_CODE, and >=sizeof(CvContour) otherwise. mode: Retrieval mode. - `CV_RETR_EXTERNAL` - retrieve only the extreme outer contours- `CV_RETR_LIST` - retrieve all the contours and puts them in the list- `CV_RETR_CCOMP` - retrieve all the contours and organizes them into two-level hierarchy: top level are external boundaries of the components, second level are boundaries of the holes- `CV_RETR_TREE` - retrieve all the contours and reconstructs the full hierarchy of nested contours method: Approximation method (for all the modes, except `CV_RETR_RUNS`, which uses built-in approximation). - `CV_CHAIN_CODE` - output contours in the Freeman chain code. All other methods output polygons (sequences of vertices).- `CV_CHAIN_APPROX_NONE` - translate all the points from the chain code into points;- `CV_CHAIN_APPROX_SIMPLE` - compress horizontal, vertical, and diagonal segments, that is, the function leaves only their ending points;- `CV_CHAIN_APPROX_TC89_L1, CV_CHAIN_APPROX_TC89_KCOS` - apply one of the flavors of Teh-Chin chain approximation algorithm.- `CV_LINK_RUNS` - use completely different contour retrieval algorithm via linking of horizontal segments of 1’s. Only `CV_RETR_LIST` retrieval mode can be used with this method. offset: Offset, by which every contour point is shifted. This is useful if the contours are extracted from the image ROI and then they should be analyzed in the whole image context.

The function `cvFindContours` retrieves contours from the binary image and returns
the number of retrieved contours. The pointer `first_contour` is filled by the function.
It will contain pointer to the first most outer contour or NULL if no contours is detected (if the image is completely black).
Other contours may be reached from `first_contour` using `h_next` and `v_next` links.
The sample in cvDrawContours discussion shows how to use contours for connected component
detection. Contours can be also used for shape analysis and object recognition - see `squares.c`
in OpenCV sample directory.

---

### StartFindContours

Initializes contour scanning process

```
CvContourScanner cvStartFindContours( CvArr* image, CvMemStorage* storage,
                                      int header_size=sizeof(CvContour),
                                      int mode=CV_RETR_LIST,
                                      int method=CV_CHAIN_APPROX_SIMPLE,
                                      CvPoint offset=cvPoint(0,0) );
```

image: The source 8-bit single channel binary image. storage: Container of the retrieved contours. header\_size: Size of the sequence header, >=sizeof(CvChain) if `method`=CV\_CHAIN\_CODE, and >=sizeof(CvContour) otherwise. mode: Retrieval mode; see cvFindContours. method: Approximation method. It has the same meaning as in cvFindContours, but CV\_LINK\_RUNS can not be used here. offset: ROI offset; see cvFindContours.

The function `cvStartFindContours` initializes and returns pointer to the contour
scanner. The scanner is used further in cvFindNextContour to retrieve the rest of contours.

---

### FindNextContour

Finds next contour in the image

```
CvSeq* cvFindNextContour( CvContourScanner scanner );
```

scanner: Contour scanner initialized by The function `cvStartFindContours` .

The function `cvFindNextContour` locates and retrieves the next contour in the image and
returns pointer to it. The function returns NULL, if there is no more contours.

---

### SubstituteContour

Replaces retrieved contour

```
void cvSubstituteContour( CvContourScanner scanner, CvSeq* new_contour );
```

scanner: Contour scanner initialized by the function cvStartFindContours . new\_contour: Substituting contour.

The function `cvSubstituteContour` replaces the retrieved contour, that was returned
from the preceding call of The function `cvFindNextContour` and stored inside
the contour scanner state, with the user-specified contour. The contour is
inserted into the resulting structure, list, two-level hierarchy, or tree,
depending on the retrieval mode. If the parameter `new_contour`=NULL, the retrieved
contour is not included into the resulting structure, nor all of its children
that might be added to this structure later.

---

### EndFindContours

Finishes scanning process

```
CvSeq* cvEndFindContours( CvContourScanner* scanner );
```

scanner: Pointer to the contour scanner.

The function `cvEndFindContours` finishes the scanning process and returns the
pointer to the first contour on the highest level.

---

### PyrSegmentation

Does image segmentation by pyramids

```
void cvPyrSegmentation( IplImage* src, IplImage* dst,
                        CvMemStorage* storage, CvSeq** comp,
                        int level, double threshold1, double threshold2 );
```

src: The source image. dst: The destination image. storage: Storage; stores the resulting sequence of connected components. comp: Pointer to the output sequence of the segmented components. level: Maximum level of the pyramid for the segmentation. threshold1: Error threshold for establishing the links. threshold2: Error threshold for the segments clustering.

The function `cvPyrSegmentation` implements image segmentation by pyramids. The
pyramid builds up to the level `level`. The links between any pixel `a` on level `i`
and its candidate father pixel `b` on the adjacent level are established if

`p(c(a),c(b))<threshold1`.
After the connected components are defined, they are joined into several
clusters. Any two segments A and B belong to the same cluster, if

`p(c(A),c(B))<threshold2`. The input
image has only one channel, then

`p(c¹,c²)=|c¹-c²|`. If the input image has three channels (red,
green and blue), then

`p(c¹,c²)=0,3·(c¹r-c²r)+0,59·(c¹g-c²g)+0,11·(c¹b-c²b)`  .
There may be more than one connected component per a cluster.

The images `src` and `dst` should be 8-bit single-channel or 3-channel images
or equal size

---

### PyrMeanShiftFiltering

Does MeanShift image segmentation

```
void cvPyrMeanShiftFiltering( const CvArr* src, CvArr* dst,
     double sp, double sr, int max_level=1,
     CvTermCriteria termcrit=cvTermCriteria(CV_TERMCRIT_ITER+CV_TERMCRIT_EPS,5,1));
```

src: The source 8-bit 3-channel image. dst: The destination image of the same format and the same size as the source. sp: The spatial window radius. sr: The color window radius. max\_level: Maximum level of the pyramid for the segmentation. termcrit: Termination criteria: when to stop meanshift iterations.

The function `cvPyrMeanShiftFiltering` implements the filtering stage of meanshift
segmentation, that is, the output of the function is the filtered "posterized" image with
color gradients and fine-grain texture flattened. At every pixel `(X,Y)` of the
input image (or down-sized input image, see below) the function executes meanshift iterations,
that is, the pixel `(X,Y)` neighborhood in the joint space-color
hyper-space is considered:

```
{(x,y): X-sp≤x≤X+sp && Y-sp≤y≤Y+sp && ||(R,G,B)-(r,g,b)|| ≤ sr},
```

where `(R,G,B)` and `(r,g,b)` are the vectors of color components
at `(X,Y)` and `(x,y)`, respectively (though, the algorithm does not depend
on the color space used, so any 3-component color space can be used instead).
Over the neighborhood the average spatial value `(X',Y')` and average color vector
`(R',G',B')` are found and they act as the neighborhood center on the next iteration:

```
(X,Y)~(X',Y'), (R,G,B)~(R',G',B').
```

After the iterations over, the color components of the initial pixel
(that is, the pixel from where the iterations started)
are set to the final value (average color at the last iteration):

```
I(X,Y) <- (R*,G*,B*).
```

Then `max_level`>0, the Gaussian pyramid of `max_level`+1 levels is built,
and the above procedure is run on the smallest layer. After that, the results are propagated to the
larger layer and the iterations are run again only on those pixels where the layer colors
differ much (>`sr`) from the lower-resolution layer, that is,
the boundaries of the color regions are clarified. Note, that the results
will be actually different from the ones obtained by running the meanshift procedure
on the whole original image (i.e. when `max_level`==0).

---

### Watershed

Does watershed segmentation

```
void cvWatershed( const CvArr* image, CvArr* markers );
```

image: The input 8-bit 3-channel image. markers: The input/output 32-bit single-channel image (map) of markers.

The function `cvWatershed` implements one of the variants of watershed,
non-parametric marker-based segmentation algorithm, described in [Meyer92]
Before passing the image to the function, user has to outline roughly the desired regions
in the image `markers` with positive (>0) indices, i.e. every region is represented as one or more
connected components with the pixel values 1, 2, 3 etc. Those components will be "seeds" of the
future image regions.
All the other pixels in `markers`, which relation to the outlined regions is not known
and should be defined by the algorithm, should be set to 0's. On the output of the function,
each pixel in `markers` is set to one of values of the "seed" components, or to -1
at boundaries between the regions.

Note, that it is not necessary that every
two neighbor connected components
are separated by a watershed boundary (-1's pixels), for example, in case when such tangent components
exist in the initial marker image. Visual demonstration and usage example of the function can be
found in OpenCV samples directory; see `watershed.cpp` demo.

---

## Image and Contour moments

---

### Moments

Calculates all moments up to third order of a polygon or rasterized shape

```
void cvMoments( const CvArr* arr, CvMoments* moments, int binary=0 );
```

arr: Image (1-channel or 3-channel with COI set) or polygon (CvSeq of points or a vector of points). moments: Pointer to returned moment state structure. binary: (For images only) If the flag is non-zero, all the zero pixel values are treated as zeroes, all the others are treated as 1’s.

The function `cvMoments` calculates spatial and central moments up to the third order and
writes them to `moments`. The moments may be used then to calculate gravity center of the shape,
its area, main axises and various shape characteristics including 7 Hu invariants.

---

### GetSpatialMoment

Retrieves spatial moment from moment state structure

```
double cvGetSpatialMoment( CvMoments* moments, int x_order, int y_order );
```

moments: The moment state, calculated by cvMoments. x\_order: x order of the retrieved moment, `x_order` >= 0. y\_order: y order of the retrieved moment, `y_order` >= 0 and `x_order` + `y_order` <= 3.

The function `cvGetSpatialMoment` retrieves the spatial moment, which in case of
image moments is defined as:

```
Mx_order,y_order=sumx,y(I(x,y)•xx_order•yy_order)
```

where `I(x,y)` is the intensity of the pixel `(x, y)`.

---

### GetCentralMoment

Retrieves central moment from moment state structure

```
double cvGetCentralMoment( CvMoments* moments, int x_order, int y_order );
```

moments: Pointer to the moment state structure. x\_order: x order of the retrieved moment, `x_order` >= 0. y\_order: y order of the retrieved moment, `y_order` >= 0 and `x_order` + `y_order` <= 3.

The function `cvGetCentralMoment` retrieves the central moment, which in case of
image moments is defined as:

```
μx_order,y_order=sumx,y(I(x,y)•(x-xc)x_order•(y-yc)y_order),
```

where `xc=M10/M00, yc=M01/M00` - coordinates of the gravity center

---

### GetNormalizedCentralMoment

Retrieves normalized central moment from moment state structure

```
double cvGetNormalizedCentralMoment( CvMoments* moments, int x_order, int y_order );
```

moments: Pointer to the moment state structure. x\_order: x order of the retrieved moment, `x_order` >= 0. y\_order: y order of the retrieved moment, `y_order` >= 0 and `x_order` + `y_order` <= 3.

The function `cvGetNormalizedCentralMoment`
retrieves the normalized central moment:

```
ηx_order,y_order= μx_order,y_order/M00((y_order+x_order)/2+1)
```

---

### GetHuMoments

Calculates seven Hu invariants

```
void cvGetHuMoments( CvMoments* moments, CvHuMoments* hu_moments );
```

moments: Pointer to the moment state structure. hu\_moments: Pointer to Hu moments structure.

The function `cvGetHuMoments` calculates seven Hu invariants that are defined as:

```
 h1=η20+η02

 h2=(η20-η02)²+4η11²

 h3=(η30-3η12)²+ (3η21-η03)²

 h4=(η30+η12)²+ (η21+η03)²

 h5=(η30-3η12)(η30+η12)[(η30+η12)²-3(η21+η03)²]+(3η21-η03)(η21+η03)[3(η30+η12)²-(η21+η03)²]

 h6=(η20-η02)[(η30+η12)²- (η21+η03)²]+4η11(η30+η12)(η21+η03)

 h7=(3η21-η03)(η21+η03)[3(η30+η12)²-(η21+η03)²]-(η30-3η12)(η21+η03)[3(η30+η12)²-(η21+η03)²]
```

where `ηi,j` are normalized central moments of
2-nd and 3-rd orders.
The computed values are proved to be invariant to the image scaling, rotation, and
reflection except the seventh one, whose sign is changed by reflection.

---

## Special Image Transforms

---

### HoughLines2

Finds lines in binary image using Hough transform

```
CvSeq* cvHoughLines2( CvArr* image, void* line_storage, int method,
                      double rho, double theta, int threshold,
                      double param1=0, double param2=0 );
```

image: The input 8-bit single-channel binary image. In case of probabilistic method the image is modified by the function. line\_storage: The storage for the lines detected. It can be a memory storage (in this case a sequence of lines is created in the storage and returned by the function) or single row/single column matrix (CvMat\*) of a particular type (see below) to which the lines' parameters are written. The matrix header is modified by the function so its `cols` or `rows` will contain a number of lines detected. If `line_storage` is a matrix and the actual number of lines exceeds the matrix size, the maximum possible number of lines is returned (in case of standard hough transform the lines are sorted by the accumulator value). method: The Hough transform variant, one of: - `CV_HOUGH_STANDARD` - classical or standard Hough transform. Every line is represented by two floating-point numbers (ρ, θ), where ρ is a distance between (0,0) point and the line, and θ is the angle between x-axis and the normal to the line. Thus, the matrix must be (the created sequence will be) of CV\_32FC2 type.- `CV_HOUGH_PROBABILISTIC` - probabilistic Hough transform (more efficient in case if picture contains a few long linear segments). It returns line segments rather than the whole lines. Every segment is represented by starting and ending points, and the matrix must be (the created sequence will be) of CV\_32SC4 type.- `CV_HOUGH_MULTI_SCALE` - multi-scale variant of classical Hough transform. The lines are encoded the same way as in CV\_HOUGH\_STANDARD. rho: Distance resolution in pixel-related units. theta: Angle resolution measured in radians. threshold: Threshold parameter. A line is returned by the function if the corresponding accumulator value is greater than `threshold`. param1: The first method-dependent parameter: - For classical Hough transform it is not used (0).- For probabilistic Hough transform it is the minimum line length.- For multi-scale Hough transform it is divisor for distance resolution `rho`. (The coarse distance resolution will be `rho` and the accurate resolution will be (`rho` / `param1`)). param2: The second method-dependent parameter: - For classical Hough transform it is not used (0).- For probabilistic Hough transform it is the maximum gap between line segments lying on the same line to treat them as the single line segment (i.e. to join them).- For multi-scale Hough transform it is divisor for angle resolution `theta`. (The coarse angle resolution will be `theta` and the accurate resolution will be (`theta` / `param2`)).

The function `cvHoughLines2` implements a few variants of Hough transform for line detection.

#### Example. Detecting lines with Hough transform.

```
/* This is a stand-alone program. Pass an image name as a first parameter of the program.
   Switch between standard and probabilistic Hough transform by changing "#if 1" to "#if 0" and back */
#include <cv.h>
#include <highgui.h>
#include <math.h>

int main(int argc, char** argv)
{
    IplImage* src;
    if( argc == 2 && (src=cvLoadImage(argv[1], 0))!= 0)
    {
        IplImage* dst = cvCreateImage( cvGetSize(src), 8, 1 );
        IplImage* color_dst = cvCreateImage( cvGetSize(src), 8, 3 );
        CvMemStorage* storage = cvCreateMemStorage(0);
        CvSeq* lines = 0;
        int i;
        cvCanny( src, dst, 50, 200, 3 );
        cvCvtColor( dst, color_dst, CV_GRAY2BGR );
#if 1
        lines = cvHoughLines2( dst, storage, CV_HOUGH_STANDARD, 1, CV_PI/180, 100, 0, 0 );

        for( i = 0; i < MIN(lines->total,100); i++ )
        {
            float* line = (float*)cvGetSeqElem(lines,i);
            float rho = line[0];
            float theta = line[1];
            CvPoint pt1, pt2;
            double a = cos(theta), b = sin(theta);
            double x0 = a*rho, y0 = b*rho;
            pt1.x = cvRound(x0 + 1000*(-b));
            pt1.y = cvRound(y0 + 1000*(a));
            pt2.x = cvRound(x0 - 1000*(-b));
            pt2.y = cvRound(y0 - 1000*(a));
            cvLine( color_dst, pt1, pt2, CV_RGB(255,0,0), 3, 8 );
        }
#else
        lines = cvHoughLines2( dst, storage, CV_HOUGH_PROBABILISTIC, 1, CV_PI/180, 50, 50, 10 );
        for( i = 0; i < lines->total; i++ )
        {
            CvPoint* line = (CvPoint*)cvGetSeqElem(lines,i);
            cvLine( color_dst, line[0], line[1], CV_RGB(255,0,0), 3, 8 );
        }
#endif
        cvNamedWindow( "Source", 1 );
        cvShowImage( "Source", src );

        cvNamedWindow( "Hough", 1 );
        cvShowImage( "Hough", color_dst );

        cvWaitKey(0);
    }
}
```

This is the sample picture the function parameters have been tuned for:

And this is the output of the above program in case of probabilistic Hough transform ("#if 0" case):

---

### HoughCircles

Finds circles in grayscale image using Hough transform

```
CvSeq* cvHoughCircles( CvArr* image, void* circle_storage,
                       int method, double dp, double min_dist,
                       double param1=100, double param2=100,
                       int min_radius=0, int max_radius=0 );
```

image: The input 8-bit single-channel grayscale image. circle\_storage: The storage for the circles detected. It can be a memory storage (in this case a sequence of circles is created in the storage and returned by the function) or single row/single column matrix (CvMat\*) of type CV\_32FC3, to which the circles' parameters are written. The matrix header is modified by the function so its `cols` or `rows` will contain a number of lines detected. If `circle_storage` is a matrix and the actual number of lines exceeds the matrix size, the maximum possible number of circles is returned. Every circle is encoded as 3 floating-point numbers: center coordinates (x,y) and the radius. method: Currently, the only implemented method is `CV_HOUGH_GRADIENT`, which is basically 21HT, described in **[Yuen03]**. dp: Resolution of the accumulator used to detect centers of the circles. For example, if it is 1, the accumulator will have the same resolution as the input image, if it is 2 - accumulator will have twice smaller width and height, etc. min\_dist: Minimum distance between centers of the detected circles. If the parameter is too small, multiple neighbor circles may be falsely detected in addition to a true one. If it is too large, some circles may be missed. param1: The first method-specific parameter. In case of `CV_HOUGH_GRADIENT` it is the higher threshold of the two passed to Canny edge detector (the lower one will be twice smaller). param2: The second method-specific parameter. In case of `CV_HOUGH_GRADIENT` it is accumulator threshold at the center detection stage. The smaller it is, the more false circles may be detected. Circles, corresponding to the larger accumulator values, will be returned first. min\_radius: Minimal radius of the circles to search for. max\_radius: Maximal radius of the circles to search for. By default the maximal radius is set to `max(image_width, image_height)`.

The function `cvHoughCircles` finds circles in grayscale image using some modification of Hough transform.

#### Example. Detecting circles with Hough transform.

```
#include <cv.h>
#include <highgui.h>
#include <math.h>

int main(int argc, char** argv)
{
    IplImage* img;
    if( argc == 2 && (img=cvLoadImage(argv[1], 1))!= 0)
    {
        IplImage* gray = cvCreateImage( cvGetSize(img), 8, 1 );
        CvMemStorage* storage = cvCreateMemStorage(0);
        cvCvtColor( img, gray, CV_BGR2GRAY );
        cvSmooth( gray, gray, CV_GAUSSIAN, 9, 9 ); // smooth it, otherwise a lot of false circles may be detected
        CvSeq* circles = cvHoughCircles( gray, storage, CV_HOUGH_GRADIENT, 2, gray->height/4, 200, 100 );
        int i;
        for( i = 0; i < circles->total; i++ )
        {
             float* p = (float*)cvGetSeqElem( circles, i );
             cvCircle( img, cvPoint(cvRound(p[0]),cvRound(p[1])), 3, CV_RGB(0,255,0), -1, 8, 0 );
             cvCircle( img, cvPoint(cvRound(p[0]),cvRound(p[1])), cvRound(p[2]), CV_RGB(255,0,0), 3, 8, 0 );
        }
        cvNamedWindow( "circles", 1 );
        cvShowImage( "circles", img );
    }
    return 0;
}
```

---

### DistTransform

Calculates distance to closest zero pixel for all non-zero pixels of source
image

```
void cvDistTransform( const CvArr* src, CvArr* dst, int distance_type=CV_DIST_L2,
                      int mask_size=3, const float* mask=NULL, CvArr* labels=NULL );
```

src: Source 8-bit single-channel (binary) image. dst: Output image with calculated distances. In most cases it should be 32-bit floating-point, single-channel array of the same size as the input image. When `distance_type`==`CV_DIST_L1`, 8-bit, single-channel destination array may be also used (in-place operation is also supported in this case). distance\_type: Type of distance; can be `CV_DIST_L1, CV_DIST_L2, CV_DIST_C` or `CV_DIST_USER`. mask\_size: Size of distance transform mask; can be 3, 5 or 0. In case of `CV_DIST_L1` or `CV_DIST_C` the parameter is forced to 3, because 3×3 mask gives the same result as 5×5 yet it is faster. When `mask_size`==0, a different non-approximate algorithm is used to calculate distances. mask: User-defined mask in case of user-defined distance, it consists of 2 numbers (horizontal/vertical shift cost, diagonal shift cost) in case of 3×3 mask and 3 numbers (horizontal/vertical shift cost, diagonal shift cost, knight’s move cost) in case of 5×5 mask. labels: The optional output 2d array of labels of integer type and the same size as `src` and `dst`, can now be used only with `mask_size`==3 or 5.

The function `cvDistTransform` calculates the approximated or exact distance from every binary image pixel
to the nearest zero pixel. When `mask_size`==0, the function uses the accurate algorithm
[Felzenszwalb04]. When `mask_size`==3 or 5, the function
uses the approximate algorithm [Borgefors86].

Here is how the approximate
algorithm works. For zero pixels the function sets the zero distance. For others it finds
the shortest path to a zero pixel, consisting of basic shifts: horizontal, vertical, diagonal or knight’s move (the
latest is available for 5×5 mask). The overall distance is calculated as a sum of these basic distances.
Because the distance function should be symmetric, all the horizontal and vertical shifts must have
the same cost (that is denoted as `a`), all the diagonal shifts must have the same cost
(denoted `b`), and all knight’s moves must have the same cost (denoted `c`).
For `CV_DIST_C` and `CV_DIST_L1` types the distance is calculated precisely,
whereas for `CV_DIST_L2` (Euclidean distance) the distance can be calculated only with
some relative error (5×5 mask gives more accurate results), OpenCV uses the values suggested in
[Borgefors86]:

```
CV_DIST_C (3×3):
a=1, b=1

CV_DIST_L1 (3×3):
a=1, b=2

CV_DIST_L2 (3×3):
a=0.955, b=1.3693

CV_DIST_L2 (5×5):
a=1, b=1.4, c=2.1969
```

And below are samples of distance field (black (0) pixel is in the middle of white square)
in case of user-defined distance:

#### User-defined 3×3 mask (a=1, b=1.5)

|  |  |  |  |  |  |  |
| --- | --- | --- | --- | --- | --- | --- |
| 4.5 | 4 | 3.5 | 3 | 3.5 | 4 | 4.5 |
| 4 | 3 | 2.5 | 2 | 2.5 | 3 | 4 |
| 3.5 | 2.5 | 1.5 | 1 | 1.5 | 2.5 | 3.5 |
| 3 | 2 | 1 | 0 | 1 | 2 | 3 |
| 3.5 | 2.5 | 1.5 | 1 | 1.5 | 2.5 | 3.5 |
| 4 | 3 | 2.5 | 2 | 2.5 | 3 | 4 |
| 4.5 | 4 | 3.5 | 3 | 3.5 | 4 | 4.5 |

#### User-defined 5×5 mask (a=1, b=1.5, c=2)

|  |  |  |  |  |  |  |
| --- | --- | --- | --- | --- | --- | --- |
| 4.5 | 3.5 | 3 | 3 | 3 | 3.5 | 4.5 |
| 3.5 | 3 | 2 | 2 | 2 | 3 | 3.5 |
| 3 | 2 | 1.5 | 1 | 1.5 | 2 | 3 |
| 3 | 2 | 1 | 0 | 1 | 2 | 3 |
| 3 | 2 | 1.5 | 1 | 1.5 | 2 | 3 |
| 3.5 | 3 | 2 | 2 | 2 | 3 | 3.5 |
| 4 | 3.5 | 3 | 3 | 3 | 3.5 | 4 |

Typically, for fast coarse distance estimation CV\_DIST\_L2, 3×3 mask is used,
and for more accurate distance estimation CV\_DIST\_L2, 5×5 mask is used.

When the output parameter `labels` is not `NULL`, for every non-zero pixel
the function also finds the nearest connected component consisting of zero pixels. The connected components
themselves are found as contours in the beginning of the function.

In this mode the processing time is still O(N), where N is the number of pixels.
Thus, the function provides a very fast way to compute approximate Voronoi diagram for the binary image.

---

### Inpaint

Inpaints the selected region in the image

```
void cvInpaint( const CvArr* src, const CvArr* mask, CvArr* dst,
                int flags, double inpaintRadius );
```

src: The input 8-bit 1-channel or 3-channel image. mask: The inpainting mask, 8-bit 1-channel image. Non-zero pixels indicate the area that needs to be inpainted. dst: The output image of the same format and the same size as input. flags: The inpainting method, one of the following: `CV_INPAINT_NS` - Navier-Stokes based method. `CV_INPAINT_TELEA` - The method by Alexandru Telea [Telea04] inpaintRadius: The radius of circular neighborhood of each point inpainted that is considered by the algorithm.

The function `cvInpaint` reconstructs the selected image area from the pixel near the
area boundary. The function may be used to remove dust and scratches from a scanned photo, or
to remove undesirable objects from still images or video.

---

## Histograms

---

### CvHistogram

Multi-dimensional histogram

```
typedef struct CvHistogram
{
    int     type;
    CvArr*  bins;
    float   thresh[CV_MAX_DIM][2]; /* for uniform histograms */
    float** thresh2; /* for non-uniform histograms */
    CvMatND mat; /* embedded matrix header for array histograms */
}
CvHistogram;
```

---

### CreateHist

Creates histogram

```
CvHistogram* cvCreateHist( int dims, int* sizes, int type,
                           float** ranges=NULL, int uniform=1 );
```

dims: Number of histogram dimensions. sizes: Array of histogram dimension sizes. type: Histogram representation format: `CV_HIST_ARRAY` means that histogram data is represented as an multi-dimensional dense array CvMatND; `CV_HIST_SPARSE` means that histogram data is represented as a multi-dimensional sparse array CvSparseMat. ranges: Array of ranges for histogram bins. Its meaning depends on the `uniform` parameter value. The ranges are used for when histogram is calculated or back-projected to determine, which histogram bin corresponds to which value/tuple of values from the input image[s]. uniform: Uniformity flag; if not 0, the histogram has evenly spaced bins and for every `0<=i<cDims` `ranges[i]` is array of two numbers: lower and upper boundaries for the i-th histogram dimension. The whole range [lower,upper] is split then into `dims[i]` equal parts to determine `i-th` input tuple value ranges for every histogram bin. And if `uniform=0`, then `i-th` element of `ranges` array contains `dims[i]+1` elements: `lower0, upper0, lower1, upper1 == lower2, ..., upperdims[i]-1`, where `lowerj` and `upperj` are lower and upper boundaries of `i-th` input tuple value for `j-th` bin, respectively. In either case, the input values that are beyond the specified range for a histogram bin, are not counted by cvCalcHist and filled with 0 by cvCalcBackProject.

The function `cvCreateHist` creates a histogram of the specified size and returns
the pointer to the created histogram. If the array `ranges` is 0, the histogram
bin ranges must be specified later via The function `cvSetHistBinRanges`, though
cvCalcHist and cvCalcBackProject may process 8-bit images without setting
bin ranges, they assume equally spaced in 0..255 bins.

---

### SetHistBinRanges

Sets bounds of histogram bins

```
void cvSetHistBinRanges( CvHistogram* hist, float** ranges, int uniform=1 );
```

hist: Histogram. ranges: Array of bin ranges arrays, see cvCreateHist. uniform: Uniformity flag, see cvCreateHist.

The function `cvSetHistBinRanges` is a stand-alone function for setting bin ranges
in the histogram. For more detailed description of the parameters `ranges` and
`uniform` see cvCalcHist function,
that can initialize the ranges as well.
Ranges for histogram bins must be set before the histogram is calculated or
back projection of the histogram is calculated.

---

### ReleaseHist

Releases histogram

```
void cvReleaseHist( CvHistogram** hist );
```

hist: Double pointer to the released histogram.

The function `cvReleaseHist` releases the histogram (header and the data).
The pointer to histogram is cleared by the function. If `*hist` pointer is already
`NULL`, the function does nothing.

---

### ClearHist

Clears histogram

```
void cvClearHist( CvHistogram* hist );
```

hist: Histogram.

The function `cvClearHist` sets all histogram bins to 0 in case of dense histogram and
removes all histogram bins in case of sparse array.

---

### MakeHistHeaderForArray

Makes a histogram out of array

```
CvHistogram*  cvMakeHistHeaderForArray( int dims, int* sizes, CvHistogram* hist,
                                        float* data, float** ranges=NULL, int uniform=1 );
```

dims: Number of histogram dimensions. sizes: Array of histogram dimension sizes. hist: The histogram header initialized by the function. data: Array that will be used to store histogram bins. ranges: Histogram bin ranges, see cvCreateHist. uniform: Uniformity flag, see cvCreateHist.

The function `cvMakeHistHeaderForArray` initializes the histogram, which header and
bins are allocated by user. No cvReleaseHist need to be called afterwards.
Only dense histograms can be initialized this way. The function returns `hist`.

---

### QueryHistValue\_\*D

Queries value of histogram bin

```
#define cvQueryHistValue_1D( hist, idx0 ) \
    cvGetReal1D( (hist)->bins, (idx0) )
#define cvQueryHistValue_2D( hist, idx0, idx1 ) \
    cvGetReal2D( (hist)->bins, (idx0), (idx1) )
#define cvQueryHistValue_3D( hist, idx0, idx1, idx2 ) \
    cvGetReal3D( (hist)->bins, (idx0), (idx1), (idx2) )
#define cvQueryHistValue_nD( hist, idx ) \
    cvGetRealND( (hist)->bins, (idx) )
```

hist: Histogram. idx0, idx1, idx2, idx3: Indices of the bin. idx: Array of indices

The macros cvQueryHistValue\_\*D return the value of the specified bin of 1D, 2D, 3D or
N-D histogram. In case of sparse histogram the function returns 0, if the bin is not present in the
histogram, and no new bin is created.

---

### GetHistValue\_\*D

Returns pointer to histogram bin

```
#define cvGetHistValue_1D( hist, idx0 ) \
    ((float*)(cvPtr1D( (hist)->bins, (idx0), 0 ))
#define cvGetHistValue_2D( hist, idx0, idx1 ) \
    ((float*)(cvPtr2D( (hist)->bins, (idx0), (idx1), 0 ))
#define cvGetHistValue_3D( hist, idx0, idx1, idx2 ) \
    ((float*)(cvPtr3D( (hist)->bins, (idx0), (idx1), (idx2), 0 ))
#define cvGetHistValue_nD( hist, idx ) \
    ((float*)(cvPtrND( (hist)->bins, (idx), 0 ))
```

hist: Histogram. idx0, idx1, idx2, idx3: Indices of the bin. idx: Array of indices

The macros cvGetHistValue\_\*D return pointer to the specified bin of 1D, 2D, 3D or
N-D histogram. In case of sparse histogram the function creates a new bin and sets it to 0,
unless it exists already.

---

### GetMinMaxHistValue

Finds minimum and maximum histogram bins

```
void cvGetMinMaxHistValue( const CvHistogram* hist,
                           float* min_value, float* max_value,
                           int* min_idx=NULL, int* max_idx=NULL );
```

hist: Histogram. min\_value: Pointer to the minimum value of the histogram max\_value: Pointer to the maximum value of the histogram min\_idx: Pointer to the array of coordinates for minimum max\_idx: Pointer to the array of coordinates for maximum

The function `cvGetMinMaxHistValue` finds the minimum and maximum histogram bins and
their positions. Any of output arguments is optional.
Among several extremums with the same value the ones with minimum index (in lexicographical order)
In case of several maximums or minimums the earliest in lexicographical order
extrema locations are returned.

---

### NormalizeHist

Normalizes histogram

```
void cvNormalizeHist( CvHistogram* hist, double factor );
```

hist: Pointer to the histogram. factor: Normalization factor.

The function `cvNormalizeHist` normalizes the histogram bins by scaling them,
such that the sum of the bins becomes equal to `factor`.

---

### ThreshHist

Thresholds histogram

```
void cvThreshHist( CvHistogram* hist, double threshold );
```

hist: Pointer to the histogram. threshold: Threshold level.

The function `cvThreshHist` clears histogram bins
that are below the specified threshold.

---

### CompareHist

Compares two dense histograms

```
double cvCompareHist( const CvHistogram* hist1, const CvHistogram* hist2, int method );
```

hist1: The first dense histogram. hist2: The second dense histogram. method: Comparison method, one of: - CV\_COMP\_CORREL- CV\_COMP\_CHISQR- CV\_COMP\_INTERSECT- CV\_COMP\_BHATTACHARYYA

The function `cvCompareHist` compares two dense histograms using
the specified method as following
(`H1` denotes the first histogram, `H2` - the second):

```
Correlation (method=CV_COMP_CORREL):
d(H1,H2)=sumI(H'1(I)•H'2(I))/sqrt(sumI[H'1(I)2]•sumI[H'2(I)2])
where
H'k(I)=Hk(I)-1/N•sumJHk(J) (N=number of histogram bins)

Chi-Square (method=CV_COMP_CHISQR):
d(H1,H2)=sumI[(H1(I)-H2(I))/(H1(I)+H2(I))]

Intersection (method=CV_COMP_INTERSECT):
d(H1,H2)=sumImin(H1(I),H2(I))

Bhattacharyya distance (method=CV_COMP_BHATTACHARYYA):
d(H1,H2)=sqrt(1-sumI(sqrt(H1(I)•H2(I))))
```

The function returns `d(H1,H2)` value.

Note: the method `CV_COMP_BHATTACHARYYA` only works with normalized histograms.

To compare sparse histogram or more general sparse configurations of weighted points,
consider using cvCalcEMD2 function.

---

### CopyHist

Copies histogram

```
void cvCopyHist( const CvHistogram* src, CvHistogram** dst );
```

src: Source histogram. dst: Pointer to destination histogram.

The function `cvCopyHist` makes a copy of the histogram. If the second histogram
pointer `*dst` is NULL, a new histogram of the same size as `src` is created.
Otherwise, both histograms must have equal types and sizes.
Then the function copies the source histogram bins values to destination histogram and
sets the same bin values ranges as in `src`.

---

### CalcHist

Calculates histogram of image(s)

```
void cvCalcHist( IplImage** image, CvHistogram* hist,
                 int accumulate=0, const CvArr* mask=NULL );
```

image: Source images (though, you may pass CvMat\*\* as well), all are of the same size and type hist: Pointer to the histogram. accumulate: Accumulation flag. If it is set, the histogram is not cleared in the beginning. This feature allows user to compute a single histogram from several images, or to update the histogram online. mask: The operation mask, determines what pixels of the source images are counted.

The function `cvCalcHist` calculates the histogram of one or more single-channel images.
The elements of a tuple that is used to increment a histogram bin are taken at the same
location from the corresponding input images.

#### Sample. Calculating and displaying 2D Hue-Saturation histogram of a color image

```
#include <cv.h>
#include <highgui.h>

int main( int argc, char** argv )
{
    IplImage* src;
    if( argc == 2 && (src=cvLoadImage(argv[1], 1))!= 0)
    {
        IplImage* h_plane = cvCreateImage( cvGetSize(src), 8, 1 );
        IplImage* s_plane = cvCreateImage( cvGetSize(src), 8, 1 );
        IplImage* v_plane = cvCreateImage( cvGetSize(src), 8, 1 );
        IplImage* planes[] = { h_plane, s_plane };
        IplImage* hsv = cvCreateImage( cvGetSize(src), 8, 3 );
        int h_bins = 30, s_bins = 32;
        int hist_size[] = {h_bins, s_bins};
        float h_ranges[] = { 0, 180 }; /* hue varies from 0 (~0°red) to 180 (~360°red again) */
        float s_ranges[] = { 0, 255 }; /* saturation varies from 0 (black-gray-white) to 255 (pure spectrum color) */
        float* ranges[] = { h_ranges, s_ranges };
        int scale = 10;
        IplImage* hist_img = cvCreateImage( cvSize(h_bins*scale,s_bins*scale), 8, 3 );
        CvHistogram* hist;
        float max_value = 0;
        int h, s;

        cvCvtColor( src, hsv, CV_BGR2HSV );
        cvCvtPixToPlane( hsv, h_plane, s_plane, v_plane, 0 );
        hist = cvCreateHist( 2, hist_size, CV_HIST_ARRAY, ranges, 1 );
        cvCalcHist( planes, hist, 0, 0 );
        cvGetMinMaxHistValue( hist, 0, &max_value, 0, 0 );
        cvZero( hist_img );

        for( h = 0; h < h_bins; h++ )
        {
            for( s = 0; s < s_bins; s++ )
            {
                float bin_val = cvQueryHistValue_2D( hist, h, s );
                int intensity = cvRound(bin_val*255/max_value);
                cvRectangle( hist_img, cvPoint( h*scale, s*scale ),
                             cvPoint( (h+1)*scale - 1, (s+1)*scale - 1),
                             CV_RGB(intensity,intensity,intensity), /* draw a grayscale histogram.
                                                                       if you have idea how to do it
                                                                       nicer let us know */
                             CV_FILLED );
            }
        }

        cvNamedWindow( "Source", 1 );
        cvShowImage( "Source", src );

        cvNamedWindow( "H-S Histogram", 1 );
        cvShowImage( "H-S Histogram", hist_img );

        cvWaitKey(0);
    }
}
```

---

### CalcBackProject

Calculates back projection

```
void cvCalcBackProject( IplImage** image, CvArr* back_project, const CvHistogram* hist );
```

image: Source images (though you may pass CvMat\*\* as well), all are of the same size and type back\_project: Destination back projection image of the same type as the source images. hist: Histogram.

The function `cvCalcBackProject` calculates the back project of the histogram. For
each tuple of pixels at the same position of all input single-channel
images the function puts the value of the histogram bin, corresponding to the tuple,
to the destination image. In terms of statistics, the value of each output image pixel
is probability of the observed tuple given the distribution (histogram).
For example, to find a red object in the picture, one may do the following:

1. Calculate a hue histogram for the red object assuming the image contains only
   this object. The histogram is likely to have a strong maximum, corresponding
   to red color.- Calculate back projection of a hue plane of input image where the object is searched,
     using the histogram. Threshold the image.- Find connected components in the resulting picture and choose the right
       component using some additional criteria, for example, the largest connected
       component.

That is the approximate algorithm of CamShift color object tracker, except for the 3rd step,
instead of which CAMSHIFT algorithm is used to locate the object on the back projection given
the previous object position.

---

### CalcBackProjectPatch

Locates a template within image by histogram comparison

```
void cvCalcBackProjectPatch( IplImage** images, CvArr* dst,
                             CvSize patch_size, CvHistogram* hist,
                             int method, float factor );
```

images: Source images (though, you may pass CvMat\*\* as well), all of the same size dst: Destination image. patch\_size: Size of patch slid though the source images. hist: Histogram method: Comparison method, passed to cvCompareHist (see description of that function). factor: Normalization factor for histograms, will affect normalization scale of destination image, pass 1. if unsure.

The function `cvCalcBackProjectPatch` compares histogram, computed over
each possible rectangular patch of the specified size in the input `images`,
and stores the results to the output map `dst`.

In pseudo-code the operation may be written as:

```
for (x,y) in images (until (x+patch_size.width-1,y+patch_size.height-1) is inside the images) do
    compute histogram over the ROI (x,y,x+patch_size.width,y+patch_size.height) in images
       (see cvCalcHist)
    normalize the histogram using the factor
       (see cvNormalizeHist)
    compare the normalized histogram with input histogram hist using the specified method
       (see cvCompareHist)
    store the result to dst(x,y)
end for
```

See also a similar function cvMatchTemplate.

#### Back Project Calculation by Patches

---

### CalcProbDensity

Divides one histogram by another

```
void  cvCalcProbDensity( const CvHistogram* hist1, const CvHistogram* hist2,
                         CvHistogram* dst_hist, double scale=255 );
```

hist1: first histogram (the divisor). hist2: second histogram. dst\_hist: destination histogram. scale: scale factor for the destination histogram.

The function `cvCalcProbDensity` calculates the object probability density from
the two histograms as:

```
dist_hist(I)=0      if hist1(I)==0
             scale  if hist1(I)!=0 && hist2(I)>hist1(I)
             hist2(I)*scale/hist1(I) if hist1(I)!=0 && hist2(I)<=hist1(I)
```

So the destination histogram bins are within less than scale.

---

### EqualizeHist

Equalizes histogram of grayscale image

```
void  cvEqualizeHist( const CvArr* src, CvArr* dst );
```

src: The input 8-bit single-channel image. dst: The output image of the same size and the same data type as `src`.

The function `cvEqualizeHist` equalizes histogram of the input image
using the following algorithm:

```
1. calculate histogram H for src.
2. normalize histogram, so that the sum of histogram bins is 255.
3. compute integral of the histogram:
   H’(i) = sum0≤j≤iH(j)
4. transform the image using H’ as a look-up table: dst(x,y)=H’(src(x,y))
```

The algorithm normalizes brightness and increases contrast of the image.

---

## Matching

---

### MatchTemplate

Compares template against overlapped image regions

```
void cvMatchTemplate( const CvArr* image, const CvArr* templ,
                      CvArr* result, int method );
```

image: Image where the search is running. It should be 8-bit or 32-bit floating-point. templ: Searched template; must be not greater than the source image and the same data type as the image. result: A map of comparison results; single-channel 32-bit floating-point. If `image` is `W`×`H` and `templ` is `w`×`h` then `result` must be `W-w+1`×`H-h+1`. method: Specifies the way the template must be compared with image regions (see below).

The function `cvMatchTemplate` is similar to cvCalcBackProjectPatch.
It slides through `image`, compares overlapped patches of size `w`×`h`
with `templ` using the specified method and stores the comparison results
to `result`. Here are the formulae for the different comparison methods one may use
(`I` denotes image, `T` - template, `R` - result.
The summation is done over template and/or the image patch: `x'=0..w-1, y'=0..h-1`):

```
method=CV_TM_SQDIFF:
R(x,y)=sumx',y'[T(x',y')-I(x+x',y+y')]2

method=CV_TM_SQDIFF_NORMED:
R(x,y)=sumx',y'[T(x',y')-I(x+x',y+y')]2/sqrt[sumx',y'T(x',y')2•sumx',y'I(x+x',y+y')2]

method=CV_TM_CCORR:
R(x,y)=sumx',y'[T(x',y')•I(x+x',y+y')]

method=CV_TM_CCORR_NORMED:
R(x,y)=sumx',y'[T(x',y')•I(x+x',y+y')]/sqrt[sumx',y'T(x',y')2•sumx',y'I(x+x',y+y')2]

method=CV_TM_CCOEFF:
R(x,y)=sumx',y'[T'(x',y')•I'(x+x',y+y')],

where T'(x',y')=T(x',y') - 1/(w•h)•sumx",y"T(x",y")
      I'(x+x',y+y')=I(x+x',y+y') - 1/(w•h)•sumx",y"I(x+x",y+y")

method=CV_TM_CCOEFF_NORMED:
R(x,y)=sumx',y'[T'(x',y')•I'(x+x',y+y')]/sqrt[sumx',y'T'(x',y')2•sumx',y'I'(x+x',y+y')2]
```

After the function finishes comparison, the best matches can be found as global minimums (CV\_TM\_SQDIFF\*)
or maximums (CV\_TM\_CCORR\* and CV\_TM\_CCOEFF\*) using cvMinMaxLoc function.
In case of color image and template summation in both numerator and each sum in denominator is done
over all the channels (and separate mean values are used for each channel).

---

### MatchShapes

Compares two shapes

```
double cvMatchShapes( const void* object1, const void* object2,
                      int method, double parameter=0 );
```

object1: First contour or grayscale image object2: Second contour or grayscale image method: Comparison method, one of CV\_CONTOUR\_MATCH\_I1, CV\_CONTOURS\_MATCH\_I2 or CV\_CONTOURS\_MATCH\_I3. parameter: Method-specific parameter (is not used now).

The function `cvMatchShapes` compares two shapes. The 3 implemented methods all
use Hu moments (see cvGetHuMoments)
(`A` ~ `object1`, `B` - `object2`):

```
method=CV_CONTOUR_MATCH_I1:
I1(A,B)=sumi=1..7abs(1/mAi - 1/mBi)

method=CV_CONTOUR_MATCH_I2:
I2(A,B)=sumi=1..7abs(mAi - mBi)

method=CV_CONTOUR_MATCH_I3:
I3(A,B)=sumi=1..7abs(mAi - mBi)/abs(mAi)

where
mAi=sign(hAi)•log(hAi),
mBi=sign(hBi)•log(hBi),
hAi, hBi - Hu moments of A and B, respectively.
```

---

### CalcEMD2

Computes "minimal work" distance between two weighted point configurations

```
float cvCalcEMD2( const CvArr* signature1, const CvArr* signature2, int distance_type,
                  CvDistanceFunction distance_func=NULL, const CvArr* cost_matrix=NULL,
                  CvArr* flow=NULL, float* lower_bound=NULL, void* userdata=NULL );
typedef float (*CvDistanceFunction)(const float* f1, const float* f2, void* userdata);
```

signature1: First signature, `size1`×`dims+1` floating-point matrix. Each row stores the point weight followed by the point coordinates. The matrix is allowed to have a single column (weights only) if the user-defined cost matrix is used. signature2: Second signature of the same format as `signature1`, though the number of rows may be different. The total weights may be different, in this case an extra "dummy" point is added to either `signature1` or `signature2`. distance\_type: Metrics used; `CV_DIST_L1, CV_DIST_L2`, and `CV_DIST_C` stand for one of the standard metrics; `CV_DIST_USER` means that a user-defined function `distance_func` or pre-calculated `cost_matrix` is used. distance\_func: The user-defined distance function. It takes coordinates of two points and returns the distance between the points. cost\_matrix: The user-defined `size1`×`size2` cost matrix. At least one of `cost_matrix` and `distance_func` must be NULL. Also, if a cost matrix is used, lower boundary (see below) can not be calculated, because it needs a metric function. flow: The resultant `size1`×`size2` flow matrix: `flowij` is a flow from i-th point of `signature1` to j-th point of `signature2` lower\_bound: Optional input/output parameter: lower boundary of distance between the two signatures that is a distance between mass centers. The lower boundary may not be calculated if the user-defined cost matrix is used, the total weights of point configurations are not equal, or there is the signatures consist of weights only (i.e. the signature matrices have a single column). User *must* initialize `*lower_bound`. If the calculated distance between mass centers is greater or equal to `*lower_bound` (it means that the signatures are far enough) the function does not calculate EMD. In any case `*lower_bound` is set to the calculated distance between mass centers on return. Thus, if user wants to calculate both distance between mass centers and EMD, `*lower_bound` should be set to 0. userdata: Pointer to optional data that is passed into the user-defined distance function.

The function `cvCalcEMD2` computes earth mover distance and/or a lower boundary of
the distance between the two weighted point configurations.
One of the application described in [RubnerSept98] is multi-dimensional
histogram comparison for image retrieval.
EMD is a transportation problem that is solved using some modification of simplex algorithm,
thus the complexity is exponential in the worst case, though, it is much faster in average.
In case of a real metric the lower boundary can be calculated even faster (using linear-time algorithm)
and it can be used to determine roughly whether the two
signatures are far enough so that they cannot relate to the same object.

---

# Structural Analysis

---

## Contour Processing Functions

---

### ApproxChains

Approximates Freeman chain(s) with polygonal curve

```
CvSeq* cvApproxChains( CvSeq* src_seq, CvMemStorage* storage,
                       int method=CV_CHAIN_APPROX_SIMPLE,
                       double parameter=0, int minimal_perimeter=0, int recursive=0 );
```

src\_seq: Pointer to the chain that can refer to other chains. storage: Storage location for the resulting polylines. method: Approximation method (see the description of the function cvFindContours). parameter: Method parameter (not used now). minimal\_perimeter: Approximates only those contours whose perimeters are not less than `minimal_perimeter`. Other chains are removed from the resulting structure. recursive: If not 0, the function approximates all chains that access can be obtained to from `src_seq` by `h_next` or `v_next links`. If 0, the single chain is approximated.

This is a stand-alone approximation routine. The function `cvApproxChains` works
exactly in the same way as cvFindContours with the corresponding approximation flag.
The function returns pointer to the first resultant contour.
Other approximated contours, if any, can be accessed via `v_next` or
`h_next` fields of the returned structure.

---

### StartReadChainPoints

Initializes chain reader

```
void cvStartReadChainPoints( CvChain* chain, CvChainPtReader* reader );
```

chain Pointer to chain.
reader Chain reader state.

The function `cvStartReadChainPoints` initializes a special reader
(see Dynamic Data Structures
for more information on sets and sequences).

---

### ReadChainPoint

Gets next chain point

```
CvPoint cvReadChainPoint( CvChainPtReader* reader );
```

reader: Chain reader state.

The function `cvReadChainPoint` returns the current chain point and updates the reader position.

---

### ApproxPoly

Approximates polygonal curve(s) with desired precision

```
CvSeq* cvApproxPoly( const void* src_seq, int header_size, CvMemStorage* storage,
                     int method, double parameter, int parameter2=0 );
```

src\_seq: Sequence of array of points. header\_size: Header size of approximated curve[s]. storage: Container for approximated contours. If it is NULL, the input sequences' storage is used. method: Approximation method; only `CV_POLY_APPROX_DP` is supported, that corresponds to Douglas-Peucker algorithm. parameter: Method-specific parameter; in case of `CV_POLY_APPROX_DP` it is a desired approximation accuracy. parameter2: If case if `src_seq` is sequence it means whether the single sequence should be approximated or all sequences on the same level or below `src_seq` (see cvFindContours for description of hierarchical contour structures). And if `src_seq` is array (CvMat\*) of points, the parameter specifies whether the curve is closed (`parameter2`!=0) or not (`parameter2`=0).

The function `cvApproxPoly` approximates one or more curves and returns the approximation
result[s]. In case of multiple curves approximation the resultant tree will have the same structure as
the input one (1:1 correspondence).

---

### BoundingRect

Calculates up-right bounding rectangle of point set

```
CvRect cvBoundingRect( CvArr* points, int update=0 );
```

points: Either a 2D point set, represented as a sequence (`CvSeq*`, `CvContour*`) or vector (`CvMat*`) of points, or 8-bit single-channel mask image (`CvMat*`, `IplImage*`), in which non-zero pixels are considered. update: The update flag. Here is list of possible combination of the flag values and type of `contour`: - `points` is CvContour\*, `update`=0: the bounding rectangle is not calculated, but it is read from `rect` field of the contour header.- `points` is CvContour\*, `update`=1: the bounding rectangle is calculated and written to `rect` field of the contour header. For example, this mode is used by cvFindContours.- `points` is CvSeq\* or CvMat\*: `update` is ignored, the bounding rectangle is calculated and returned.

The function `cvBoundingRect` returns the up-right bounding rectangle for 2d point set.

---

### ContourArea

Calculates area of the whole contour or contour section

```
double cvContourArea( const CvArr* contour, CvSlice slice=CV_WHOLE_SEQ );
```

contour: Contour (sequence or array of vertices). slice: Starting and ending points of the contour section of interest, by default area of the whole contour is calculated.

The function `cvContourArea` calculates area of the whole contour or contour section. In the latter
case the total area bounded by the contour arc and the chord connecting the 2 selected points is calculated as
shown on the picture below:

**NOTE:** Orientation of the contour affects the area sign, thus the function may return
`negative` result. Use `fabs()` function from C runtime to get the absolute value of
area.

---

### ArcLength

Calculates contour perimeter or curve length

```
double cvArcLength( const void* curve, CvSlice slice=CV_WHOLE_SEQ, int is_closed=-1 );
```

curve: Sequence or array of the curve points. slice: Starting and ending points of the curve, by default the whole curve length is calculated. is\_closed: Indicates whether the curve is closed or not. There are 3 cases: - is\_closed=0 - the curve is assumed to be unclosed.- is\_closed>0 - the curve is assumed to be closed.- is\_closed<0 - if curve is sequence, the flag CV\_SEQ\_FLAG\_CLOSED of ((CvSeq\*)curve)->flags is checked to determine if the curve is closed or not, otherwise (curve is represented by array (CvMat\*) of points) it is assumed to be unclosed.

The function `cvArcLength` calculates length or curve as sum of lengths of segments
between subsequent points

---

### CreateContourTree

Creates hierarchical representation of contour

```
CvContourTree* cvCreateContourTree( const CvSeq* contour, CvMemStorage* storage, double threshold );
```

contour: Input contour. storage: Container for output tree. threshold: Approximation accuracy.

The function `cvCreateContourTree` creates binary tree representation for the input
`contour` and returns the pointer to its root. If the parameter `threshold`
is less than or equal to 0, the function creates full binary tree
representation. If the threshold is greater than 0, the function creates
representation with the precision `threshold`: if the vertices with the
interceptive area of its base line are less than `threshold`, the tree should not
be built any further. The function returns the created tree.

---

### ContourFromContourTree

Restores contour from tree

```
CvSeq* cvContourFromContourTree( const CvContourTree* tree, CvMemStorage* storage,
                                 CvTermCriteria criteria );
```

tree: Contour tree. storage: Container for the reconstructed contour. criteria: Criteria, where to stop reconstruction.

The function `cvContourFromContourTree` restores the contour from its binary tree
representation. The parameter `criteria` determines the accuracy and/or the
number of tree levels used for reconstruction, so it is possible to build approximated contour.
The function returns reconstructed contour.

---

### MatchContourTrees

Compares two contours using their tree representations

```
double cvMatchContourTrees( const CvContourTree* tree1, const CvContourTree* tree2,
                            int method, double threshold );
```

tree1: First contour tree. tree2: Second contour tree. method: Similarity measure, only `CV_CONTOUR_TREES_MATCH_I1` is supported. threshold: Similarity threshold.

The function `cvMatchContourTrees` calculates the value of the matching measure for
two contour trees. The similarity measure is calculated level by level from the
binary tree roots. If at the certain level difference between contours becomes less than `threshold`,
the reconstruction process is interrupted and the current difference is returned.

---

## Computational Geometry

---

### MaxRect

Finds bounding rectangle for two given rectangles

```
CvRect cvMaxRect( const CvRect* rect1, const CvRect* rect2 );
```

rect1: First rectangle rect2: Second rectangle

The function `cvMaxRect` finds minimum area rectangle that contains both input rectangles inside:

---

### CvBox2D

Rotated 2D box

```
typedef struct CvBox2D
{
    CvPoint2D32f center;  /* center of the box */
    CvSize2D32f  size;    /* box width and length */
    float angle;          /* angle between the horizontal axis
                             and the first side (i.e. length) in degrees */
}
CvBox2D;
```

---

### PointSeqFromMat

Initializes point sequence header from a point vector

```
CvSeq* cvPointSeqFromMat( int seq_kind, const CvArr* mat,
                          CvContour* contour_header,
                          CvSeqBlock* block );
```

seq\_kind: Type of the point sequence: point set (0), a curve (`CV_SEQ_KIND_CURVE`), closed curve (`CV_SEQ_KIND_CURVE+CV_SEQ_FLAG_CLOSED`) etc. mat: Input matrix. It should be continuous 1-dimensional vector of points, that is, it should have type `CV_32SC2` or `CV_32FC2`. contour\_header: Contour header, initialized by the function. block: Sequence block header, initialized by the function.

The function `cvPointSeqFromMat` initializes sequence header to create a "virtual" sequence which
elements reside in the specified matrix. No data is copied. The initialized sequence header may be passed to
any function that takes a point sequence on input. No extra elements could be added to the sequence,
but some may be removed. The function is a specialized variant of
cvMakeSeqHeaderForArray and uses the latter internally.
It returns pointer to the initialized contour header. Note that the bounding rectangle (field `rect` of
`CvContour` structure is not initialized by the function. If you need one, use
cvBoundingRect.

Here is the simple usage example.

```
CvContour header;
CvSeqBlock block;
CvMat* vector = cvCreateMat( 1, 3, CV_32SC2 );

CV_MAT_ELEM( *vector, CvPoint, 0, 0 ) = cvPoint(100,100);
CV_MAT_ELEM( *vector, CvPoint, 0, 1 ) = cvPoint(100,200);
CV_MAT_ELEM( *vector, CvPoint, 0, 2 ) = cvPoint(200,100);

IplImage* img = cvCreateImage( cvSize(300,300), 8, 3 );
cvZero(img);

cvDrawContours( img, cvPointSeqFromMat(CV_SEQ_KIND_CURVE+CV_SEQ_FLAG_CLOSED,
   vector, &header, &block), CV_RGB(255,0,0), CV_RGB(255,0,0), 0, 3, 8, cvPoint(0,0));
```

---

### BoxPoints

Finds box vertices

```
void cvBoxPoints( CvBox2D box, CvPoint2D32f pt[4] );
```

box: Box pt: Array of vertices

The function `cvBoxPoints` calculates vertices of the input 2d box.
Here is the function code:

```
void cvBoxPoints( CvBox2D box, CvPoint2D32f pt[4] )
{
    double angle = box.angle*CV_PI/180.
    float a = (float)cos(angle)*0.5f;
    float b = (float)sin(angle)*0.5f;

    pt[0].x = box.center.x - a*box.size.height - b*box.size.width;
    pt[0].y = box.center.y + b*box.size.height - a*box.size.width;
    pt[1].x = box.center.x + a*box.size.height - b*box.size.width;
    pt[1].y = box.center.y - b*box.size.height - a*box.size.width;
    pt[2].x = 2*box.center.x - pt[0].x;
    pt[2].y = 2*box.center.y - pt[0].y;
    pt[3].x = 2*box.center.x - pt[1].x;
    pt[3].y = 2*box.center.y - pt[1].y;
}
```

---

### FitEllipse

Fits ellipse to set of 2D points

```
CvBox2D cvFitEllipse2( const CvArr* points );
```

points: Sequence or array of points.

The function `cvFitEllipse` calculates ellipse that fits best (in least-squares sense)
to a set of 2D points. The meaning of the returned structure fields is similar to those
in cvEllipse except that `size` stores the full lengths of the ellipse axises,
not half-lengths

---

### FitLine

Fits line to 2D or 3D point set

```
void  cvFitLine( const CvArr* points, int dist_type, double param,
                 double reps, double aeps, float* line );
```

points: Sequence or array of 2D or 3D points with 32-bit integer or floating-point coordinates. dist\_type: The distance used for fitting (see the discussion). param: Numerical parameter (`C`) for some types of distances, if 0 then some optimal value is chosen. reps, aeps: Sufficient accuracy for radius (distance between the coordinate origin and the line) and angle, respectively, 0.01 would be a good defaults for both. line: The output line parameters. In case of 2d fitting it is array of 4 floats `(vx, vy, x0, y0)` where `(vx, vy)` is a normalized vector collinear to the line and `(x0, y0)` is some point on the line. In case of 3D fitting it is array of 6 floats `(vx, vy, vz, x0, y0, z0)` where `(vx, vy, vz)` is a normalized vector collinear to the line and `(x0, y0, z0)` is some point on the line.

The function `cvFitLine` fits line to 2D or 3D point set by minimizing sum_i_ρ(r_i_),
where r_i_ is distance between i-th point and the line and ρ(r) is a distance function, one of:

```
dist_type=CV_DIST_L2 (L2):
ρ(r)=r2/2 (the simplest and the fastest least-squares method)

dist_type=CV_DIST_L1 (L1):
ρ(r)=r

dist_type=CV_DIST_L12 (L1-L2):
ρ(r)=2•[sqrt(1+r2/2) - 1]

dist_type=CV_DIST_FAIR (Fair):
ρ(r)=C2•[r/C - log(1 + r/C)],  C=1.3998

dist_type=CV_DIST_WELSCH (Welsch):
ρ(r)=C2/2•[1 - exp(-(r/C)2)],  C=2.9846

dist_type=CV_DIST_HUBER (Huber):
ρ(r)= r2/2,   if r < C
      C•(r-C/2),   otherwise;   C=1.345
```

---

### ConvexHull2

Finds convex hull of point set

```
CvSeq* cvConvexHull2( const CvArr* input, void* hull_storage=NULL,
                      int orientation=CV_CLOCKWISE, int return_points=0 );
```

points: Sequence or array of 2D points with 32-bit integer or floating-point coordinates. hull\_storage: The destination array (CvMat\*) or memory storage (CvMemStorage\*) that will store the convex hull. If it is array, it should be 1d and have the same number of elements as the input array/sequence. On output the header is modified: the number of columns/rows is truncated down to the hull size. orientation: Desired orientation of convex hull: `CV_CLOCKWISE` or `CV_COUNTER_CLOCKWISE`. return\_points: If non-zero, the points themselves will be stored in the hull instead of indices if `hull_storage` is array, or pointers if `hull_storage` is memory storage.

The function `cvConvexHull2` finds convex hull of 2D point set using Sklansky’s algorithm.
If `hull_storage` is memory storage, the function creates a sequence containing the hull points or
pointers to them, depending on `return_points` value and returns the sequence on output.

#### Example. Building convex hull for a sequence or array of points

```
#include "cv.h"
#include "highgui.h"
#include <stdlib.h>

#define ARRAY  0 /* switch between array/sequence method by replacing 0<=>1 */

void main( int argc, char** argv )
{
    IplImage* img = cvCreateImage( cvSize( 500, 500 ), 8, 3 );
    cvNamedWindow( "hull", 1 );

#if !ARRAY
        CvMemStorage* storage = cvCreateMemStorage();
#endif

    for(;;)
    {
        int i, count = rand()%100 + 1, hullcount;
        CvPoint pt0;
#if !ARRAY
        CvSeq* ptseq = cvCreateSeq( CV_SEQ_KIND_GENERIC|CV_32SC2, sizeof(CvContour),
                                     sizeof(CvPoint), storage );
        CvSeq* hull;

        for( i = 0; i < count; i++ )
        {
            pt0.x = rand() % (img->width/2) + img->width/4;
            pt0.y = rand() % (img->height/2) + img->height/4;
            cvSeqPush( ptseq, &pt0 );
        }
        hull = cvConvexHull2( ptseq, 0, CV_CLOCKWISE, 0 );
        hullcount = hull->total;
#else
        CvPoint* points = (CvPoint*)malloc( count * sizeof(points[0]));
        int* hull = (int*)malloc( count * sizeof(hull[0]));
        CvMat point_mat = cvMat( 1, count, CV_32SC2, points );
        CvMat hull_mat = cvMat( 1, count, CV_32SC1, hull );

        for( i = 0; i < count; i++ )
        {
            pt0.x = rand() % (img->width/2) + img->width/4;
            pt0.y = rand() % (img->height/2) + img->height/4;
            points[i] = pt0;
        }
        cvConvexHull2( &point_mat, &hull_mat, CV_CLOCKWISE, 0 );
        hullcount = hull_mat.cols;
#endif
        cvZero( img );
        for( i = 0; i < count; i++ )
        {
#if !ARRAY
            pt0 = *CV_GET_SEQ_ELEM( CvPoint, ptseq, i );
#else
            pt0 = points[i];
#endif
            cvCircle( img, pt0, 2, CV_RGB( 255, 0, 0 ), CV_FILLED );
        }

#if !ARRAY
        pt0 = **CV_GET_SEQ_ELEM( CvPoint*, hull, hullcount - 1 );
#else
        pt0 = points[hull[hullcount-1]];
#endif

        for( i = 0; i < hullcount; i++ )
        {
#if !ARRAY
            CvPoint pt = **CV_GET_SEQ_ELEM( CvPoint*, hull, i );
#else
            CvPoint pt = points[hull[i]];
#endif
            cvLine( img, pt0, pt, CV_RGB( 0, 255, 0 ));
            pt0 = pt;
        }

        cvShowImage( "hull", img );

        int key = cvWaitKey(0);
        if( key == 27 ) // 'ESC'
            break;

#if !ARRAY
        cvClearMemStorage( storage );
#else
        free( points );
        free( hull );
#endif
    }
}
```

---

### CheckContourConvexity

Tests contour convex

```
int cvCheckContourConvexity( const CvArr* contour );
```

contour: Tested contour (sequence or array of points).

The function `cvCheckContourConvexity` tests whether the input contour is convex or not.
The contour must be simple, i.e. without self-intersections.

---

### CvConvexityDefect

Structure describing a single contour convexity detect

```
typedef struct CvConvexityDefect
{
    CvPoint* start; /* point of the contour where the defect begins */
    CvPoint* end; /* point of the contour where the defect ends */
    CvPoint* depth_point; /* the farthest from the convex hull point within the defect */
    float depth; /* distance between the farthest point and the convex hull */
} CvConvexityDefect;
```

#### Picture. Convexity defects of hand contour.

---

### ConvexityDefects

Finds convexity defects of contour

```
CvSeq* cvConvexityDefects( const CvArr* contour, const CvArr* convexhull,
                           CvMemStorage* storage=NULL );
```

contour: Input contour. convexhull: Convex hull obtained using cvConvexHull2 that should contain pointers or indices to the contour points, not the hull points themselves, i.e. `return_points` parameter in cvConvexHull2 should be 0. storage: Container for output sequence of convexity defects. If it is NULL, contour or hull (in that order) storage is used.

The function `cvConvexityDefects` finds all convexity defects of the input contour
and returns a sequence of the CvConvexityDefect structures.

---

### PointPolygonTest

Point in contour test

```
double cvPointPolygonTest( const CvArr* contour,
                           CvPoint2D32f pt, int measure_dist );
```

contour: Input contour. pt: The point tested against the contour. measure\_dist: If it is non-zero, the function estimates distance from the point to the nearest contour edge.

The function `cvPointPolygonTest` determines whether the point is inside contour, outside, or lies
on an edge (or coincides with a vertex). It returns positive, negative or zero value, correspondingly.
When `measure_dist=0`, the return value is +1, -1 and 0, respectively.
When `measure_dist≠0`, it is a signed distance between the point and the nearest contour edge.

Here is the sample output of the function, where each image pixel is tested against the contour.

---

### MinAreaRect2

Finds circumscribed rectangle of minimal area for given 2D point set

```
CvBox2D  cvMinAreaRect2( const CvArr* points, CvMemStorage* storage=NULL );
```

points: Sequence or array of points. storage: Optional temporary memory storage.

The function `cvMinAreaRect2` finds a circumscribed rectangle of the minimal area for 2D point set
by building convex hull for the set and applying rotating calipers technique to the hull.

#### Picture. Minimal-area bounding rectangle for contour

---

### MinEnclosingCircle

Finds circumscribed circle of minimal area for given 2D point set

```
int cvMinEnclosingCircle( const CvArr* points, CvPoint2D32f* center, float* radius );
```

points: Sequence or array of 2D points. center: Output parameter. The center of the enclosing circle. radius: Output parameter. The radius of the enclosing circle.

The function `cvMinEnclosingCircle` finds the minimal circumscribed circle for
2D point set using iterative algorithm. It returns nonzero if the resultant circle contains all the
input points and zero otherwise (i.e. algorithm failed).

---

### CalcPGH

Calculates pair-wise geometrical histogram for contour

```
void cvCalcPGH( const CvSeq* contour, CvHistogram* hist );
```

contour: Input contour. Currently, only integer point coordinates are allowed. hist: Calculated histogram; must be two-dimensional.

The function `cvCalcPGH` calculates 2D pair-wise geometrical histogram (PGH), described in
[Iivarinen97], for the contour.
The algorithm considers every pair of the contour edges. The angle
between the edges and the minimum/maximum distances are determined for every
pair. To do this each of the edges in turn is taken as the base, while the
function loops through all the other edges. When the base edge and any other
edge are considered, the minimum and maximum distances from the points on the
non-base edge and line of the base edge are selected. The angle between the
edges defines the row of the histogram in which all the bins that correspond to
the distance between the calculated minimum and maximum distances are
incremented (that is, the histogram is transposed relatively to [Iivarninen97] definition).
The histogram can be used for contour matching.

---

## Planar Subdivisions

---

### CvSubdiv2D

Planar subdivision

```
#define CV_SUBDIV2D_FIELDS()    \
    CV_GRAPH_FIELDS()           \
    int  quad_edges;            \
    int  is_geometry_valid;     \
    CvSubdiv2DEdge recent_edge; \
    CvPoint2D32f  topleft;      \
    CvPoint2D32f  bottomright;

typedef struct CvSubdiv2D
{
    CV_SUBDIV2D_FIELDS()
}
CvSubdiv2D;
```

Planar subdivision is a subdivision of a plane into a set of non-overlapped regions (facets) that
cover the whole plane. The above structure describes a subdivision built on 2d point set, where
the points are linked together and form a planar graph, which, together with a few edges connecting
exterior subdivision points (namely, convex hull points) with infinity, subdivides a plane into facets
by its edges.

For every subdivision there exists dual subdivision there facets and points (subdivision vertices)
swap their roles, that is, a facet is treated as a vertex (called virtual point below) of dual subdivision
and the original subdivision vertices become facets. On the picture below original subdivision is marked with solid lines
and dual subdivision with dot lines

OpenCV subdivides plane into triangles using Delaunay’s algorithm.
Subdivision is built iteratively starting from a dummy triangle that includes
all the subdivision points for sure.
In this case the dual subdivision is Voronoi diagram of input 2d point set.
The subdivisions can be used for 3d piece-wise transformation of a plane, morphing, fast location of
points on the plane, building special graphs (such as NNG,RNG) etc.

---

### CvQuadEdge2D

Quad-edge of planar subdivision

```
/* one of edges within quad-edge, lower 2 bits is index (0..3)
   and upper bits are quad-edge pointer */
typedef long CvSubdiv2DEdge;

/* quad-edge structure fields */
#define CV_QUADEDGE2D_FIELDS()     \
    int flags;                     \
    struct CvSubdiv2DPoint* pt[4]; \
    CvSubdiv2DEdge  next[4];

typedef struct CvQuadEdge2D
{
    CV_QUADEDGE2D_FIELDS()
}
CvQuadEdge2D;
```

Quad-edge is a basic element of subdivision, it contains four edges (e, eRot (in red) and reversed e & eRot (in green)):

---

### CvSubdiv2DPoint

Point of original or dual subdivision

```
#define CV_SUBDIV2D_POINT_FIELDS()\
    int            flags;      \
    CvSubdiv2DEdge first;      \
    CvPoint2D32f   pt;

#define CV_SUBDIV2D_VIRTUAL_POINT_FLAG (1 << 30)

typedef struct CvSubdiv2DPoint
{
    CV_SUBDIV2D_POINT_FIELDS()
}
CvSubdiv2DPoint;
```

---

### Subdiv2DGetEdge

Returns one of edges related to given

```
CvSubdiv2DEdge  cvSubdiv2DGetEdge( CvSubdiv2DEdge edge, CvNextEdgeType type );
#define cvSubdiv2DNextEdge( edge ) cvSubdiv2DGetEdge( edge, CV_NEXT_AROUND_ORG )
```

edge: Subdivision edge (not a quad-edge) type: Specifies, which of related edges to return, one of: - CV\_NEXT\_AROUND\_ORG - next around the edge origin (`eOnext` on the picture above if `e` is the input edge)- CV\_NEXT\_AROUND\_DST - next around the edge vertex (`eDnext`)- CV\_PREV\_AROUND\_ORG - previous around the edge origin (reversed `eRnext`)- CV\_PREV\_AROUND\_DST - previous around the edge destination (reversed `eLnext`)- CV\_NEXT\_AROUND\_LEFT - next around the left facet (`eLnext`)- CV\_NEXT\_AROUND\_RIGHT - next around the right facet (`eRnext`)- CV\_PREV\_AROUND\_LEFT - previous around the left facet (reversed `eOnext`)- CV\_PREV\_AROUND\_RIGHT - previous around the right facet (reversed `eDnext`)

The function `cvSubdiv2DGetEdge` returns one the edges related to the input edge.

---

### Subdiv2DRotateEdge

Returns another edge of the same quad-edge

```
CvSubdiv2DEdge  cvSubdiv2DRotateEdge( CvSubdiv2DEdge edge, int rotate );
```

edge: Subdivision edge (not a quad-edge) type: Specifies, which of edges of the same quad-edge as the input one to return, one of: - 0 - the input edge (`e` on the picture above if `e` is the input edge)- 1 - the rotated edge (`eRot`)- 2 - the reversed edge (reversed `e` (in green))- 3 - the reversed rotated edge (reversed `eRot` (in green))

The function `cvSubdiv2DRotateEdge` returns one the edges of the same quad-edge as the input edge.

---

### Subdiv2DEdgeOrg

Returns edge origin

```
CvSubdiv2DPoint* cvSubdiv2DEdgeOrg( CvSubdiv2DEdge edge );
```

edge: Subdivision edge (not a quad-edge)

The function `cvSubdiv2DEdgeOrg` returns the edge origin. The returned pointer may be NULL if
the edge is from dual subdivision and the virtual point coordinates are not calculated yet.
The virtual points can be calculated using function cvCalcSubdivVoronoi2D.

---

### Subdiv2DEdgeDst

Returns edge destination

```
CvSubdiv2DPoint* cvSubdiv2DEdgeDst( CvSubdiv2DEdge edge );
```

edge: Subdivision edge (not a quad-edge)

The function `cvSubdiv2DEdgeDst` returns the edge destination. The returned pointer may be NULL if
the edge is from dual subdivision and the virtual point coordinates are not calculated yet.
The virtual points can be calculated using function cvCalcSubdivVoronoi2D.

---

### CreateSubdivDelaunay2D

Creates empty Delaunay triangulation

```
CvSubdiv2D* cvCreateSubdivDelaunay2D( CvRect rect, CvMemStorage* storage );
```

rect: Rectangle that includes all the 2d points that are to be added to subdivision. storage: Container for subdivision.

The function `cvCreateSubdivDelaunay2D` creates an empty Delaunay subdivision,
where 2d points can be added further using function cvSubdivDelaunay2DInsert.
All the points to be added must be within the specified rectangle, otherwise a runtime error will be
raised.

---

### SubdivDelaunay2DInsert

Inserts a single point to Delaunay triangulation

```
CvSubdiv2DPoint*  cvSubdivDelaunay2DInsert( CvSubdiv2D* subdiv, CvPoint2D32f pt);
```

subdiv: Delaunay subdivision created by function cvCreateSubdivDelaunay2D. pt: Inserted point.

The function `cvSubdivDelaunay2DInsert` inserts a single point to subdivision and
modifies the subdivision topology appropriately.
If a points with same coordinates exists already, no new points is added.
The function returns pointer to the allocated point.
No virtual points coordinates is calculated at this stage.

---

### Subdiv2DLocate

Inserts a single point to Delaunay triangulation

```
CvSubdiv2DPointLocation  cvSubdiv2DLocate( CvSubdiv2D* subdiv, CvPoint2D32f pt,
                                           CvSubdiv2DEdge* edge,
                                           CvSubdiv2DPoint** vertex=NULL );
```

subdiv: Delaunay or another subdivision. pt: The point to locate. edge: The output edge the point falls onto or right to. vertex: Optional output vertex double pointer the input point coincides with.

The function `cvSubdiv2DLocate` locates input point within subdivision.
There are 5 cases:

- point falls into some facet. The function returns CV\_PTLOC\_INSIDE and `*edge` will contain one of edges of the facet.- point falls onto the edge. The function returns CV\_PTLOC\_ON\_EDGE and `*edge` will contain this edge.- point coincides with one of subdivision vertices. The function returns CV\_PTLOC\_VERTEX and `*vertex` will contain pointer to the vertex.- point is outside the subdivision reference rectangle. The function returns CV\_PTLOC\_OUTSIDE\_RECT and no pointers is filled.- one of input arguments is invalid. Runtime error is raised or, if silent or "parent" error processing mode
          is selected, CV\_PTLOC\_ERROR is returned.

---

### FindNearestPoint2D

Finds the closest subdivision vertex to given point

```
CvSubdiv2DPoint* cvFindNearestPoint2D( CvSubdiv2D* subdiv, CvPoint2D32f pt );
```

subdiv: Delaunay or another subdivision. pt: Input point.

The function `cvFindNearestPoint2D` is another function that locates input point within subdivision.
It finds subdivision vertex that is the closest to the input point. It is not necessarily one of
vertices of the facet containing the input point, though the facet (located using cvSubdiv2DLocate)
is used as a starting point. The function returns pointer to the found subdivision vertex


---

### CalcSubdivVoronoi2D

Calculates coordinates of Voronoi diagram cells

```
void cvCalcSubdivVoronoi2D( CvSubdiv2D* subdiv );
```

subdiv: Delaunay subdivision, where all the points are added already.

The function `cvCalcSubdivVoronoi2D` calculates coordinates of virtual points.
All virtual points corresponding to some vertex of original subdivision form (when connected together)
a boundary of Voronoi cell of that point.

---

### ClearSubdivVoronoi2D

Removes all virtual points

```
void cvClearSubdivVoronoi2D( CvSubdiv2D* subdiv );
```

subdiv: Delaunay subdivision.

The function `cvClearSubdivVoronoi2D` removes all virtual points.
It is called internally in cvCalcSubdivVoronoi2D if the subdivision was modified
after previous call to the function.

---

There are a few other lower-level functions that work with planar subdivisions, see cv.h
and the sources. Demo script delaunay.c that builds Delaunay triangulation and Voronoi diagram of
random 2d point set can be found at opencv/samples/c.

---

# Motion Analysis and Object Tracking Reference

---

## Accumulation of Background Statistics

---

### Acc

Adds frame to accumulator

```
void cvAcc( const CvArr* image, CvArr* sum, const CvArr* mask=NULL );
```

image: Input image, 1- or 3-channel, 8-bit or 32-bit floating point. (each channel of multi-channel image is processed independently). sum: Accumulator of the same number of channels as input image, 32-bit floating-point. mask: Optional operation mask.

The function `cvAcc` adds the whole image `image` or its selected region to accumulator `sum`:

```
sum(x,y)=sum(x,y)+image(x,y) if mask(x,y)!=0
```

---

### SquareAcc

Adds the square of source image to accumulator

```
void cvSquareAcc( const CvArr* image, CvArr* sqsum, const CvArr* mask=NULL );
```

image: Input image, 1- or 3-channel, 8-bit or 32-bit floating point (each channel of multi-channel image is processed independently). sqsum: Accumulator of the same number of channels as input image, 32-bit or 64-bit floating-point. mask: Optional operation mask.

The function `cvSquareAcc` adds the input image `image` or its selected region,
raised to power 2, to the accumulator `sqsum`:

```
sqsum(x,y)=sqsum(x,y)+image(x,y)2 if mask(x,y)!=0
```

---

### MultiplyAcc

Adds product of two input images to accumulator

```
void cvMultiplyAcc( const CvArr* image1, const CvArr* image2, CvArr* acc, const CvArr* mask=NULL );
```

image1: First input image, 1- or 3-channel, 8-bit or 32-bit floating point (each channel of multi-channel image is processed independently). image2: Second input image, the same format as the first one. acc: Accumulator of the same number of channels as input images, 32-bit or 64-bit floating-point. mask: Optional operation mask.

The function `cvMultiplyAcc` adds product of 2 images
or their selected regions to accumulator `acc`:

```
acc(x,y)=acc(x,y) + image1(x,y)•image2(x,y) if mask(x,y)!=0
```

---

### RunningAvg

Updates running average

```
void cvRunningAvg( const CvArr* image, CvArr* acc, double alpha, const CvArr* mask=NULL );
```

image: Input image, 1- or 3-channel, 8-bit or 32-bit floating point (each channel of multi-channel image is processed independently). acc: Accumulator of the same number of channels as input image, 32-bit or 64-bit floating-point. alpha: Weight of input image. mask: Optional operation mask.

The function `cvRunningAvg` calculates weighted sum of input image `image` and
the accumulator `acc` so that `acc` becomes a running average of frame sequence:

```
acc(x,y)=(1-α)•acc(x,y) + α•image(x,y) if mask(x,y)!=0
```

where α (alpha) regulates update speed (how fast accumulator forgets about previous frames).

---

## Motion Templates

---

### UpdateMotionHistory

Updates motion history image by moving silhouette

```
void cvUpdateMotionHistory( const CvArr* silhouette, CvArr* mhi,
                            double timestamp, double duration );
```

silhouette: Silhouette mask that has non-zero pixels where the motion occurs. mhi: Motion history image, that is updated by the function (single-channel, 32-bit floating-point) timestamp: Current time in milliseconds or other units. duration: Maximal duration of motion track in the same units as `timestamp`.

The function `cvUpdateMotionHistory` updates the motion history image as following:

```
mhi(x,y)=timestamp  if silhouette(x,y)!=0
         0          if silhouette(x,y)=0 and mhi(x,y)<timestamp-duration
         mhi(x,y)   otherwise
```

That is, MHI pixels where motion occurs are set to the current timestamp, while the pixels
where motion happened far ago are cleared.

---

### CalcMotionGradient

Calculates gradient orientation of motion history image

```
void cvCalcMotionGradient( const CvArr* mhi, CvArr* mask, CvArr* orientation,
                           double delta1, double delta2, int aperture_size=3 );
```

mhi: Motion history image. mask: Mask image; marks pixels where motion gradient data is correct. Output parameter. orientation: Motion gradient orientation image; contains angles from 0 to ~360°. delta1, delta2: The function finds minimum (m(x,y)) and maximum (M(x,y)) mhi values over each pixel (x,y) neighborhood and assumes the gradient is valid only if ``` min(delta1,delta2) <= M(x,y)-m(x,y) <= max(delta1,delta2). ``` aperture\_size: Aperture size of derivative operators used by the function: CV\_SCHARR, 1, 3, 5 or 7 (see cvSobel).

The function `cvCalcMotionGradient` calculates the derivatives `Dx` and `Dy` of
`mhi` and then calculates gradient orientation as:

```
orientation(x,y)=arctan(Dy(x,y)/Dx(x,y))
```

where both `Dx(x,y)`' and `Dy(x,y)`' signs are taken into account
(as in cvCartToPolar function).
After that `mask` is filled to indicate
where the orientation is valid (see `delta1` and `delta2` description).

---

### CalcGlobalOrientation

Calculates global motion orientation of some selected region

```
double cvCalcGlobalOrientation( const CvArr* orientation, const CvArr* mask, const CvArr* mhi,
                                double timestamp, double duration );
```

orientation: Motion gradient orientation image; calculated by the function cvCalcMotionGradient. mask: Mask image. It may be a conjunction of valid gradient mask, obtained with cvCalcMotionGradient and mask of the region, whose direction needs to be calculated. mhi: Motion history image. timestamp: Current time in milliseconds or other units, it is better to store time passed to cvUpdateMotionHistory before and reuse it here, because running cvUpdateMotionHistory and cvCalcMotionGradient on large images may take some time. duration: Maximal duration of motion track in milliseconds, the same as in cvUpdateMotionHistory.

The function `cvCalcGlobalOrientation`
calculates the general motion direction in
the selected region and returns the angle between 0° and 360°.
At first the function builds the orientation histogram and finds the basic
orientation as a coordinate of the histogram maximum. After that the function
calculates the shift relative to the basic orientation as a weighted sum of all
orientation vectors: the more recent is the motion, the greater is the weight.
The resultant angle is a circular sum of the basic orientation and the shift.

---

### SegmentMotion

Segments whole motion into separate moving parts

```
CvSeq* cvSegmentMotion( const CvArr* mhi, CvArr* seg_mask, CvMemStorage* storage,
                        double timestamp, double seg_thresh );
```

mhi: Motion history image. seg\_mask: Image where the mask found should be stored, single-channel, 32-bit floating-point. storage: Memory storage that will contain a sequence of motion connected components. timestamp: Current time in milliseconds or other units. seg\_thresh: Segmentation threshold; recommended to be equal to the interval between motion history "steps" or greater.

The function `cvSegmentMotion` finds all the motion segments and marks them in `seg_mask`
with individual values each (1,2,...). It also returns a sequence of CvConnectedComp structures,
one per each motion components. After than the motion direction for every component can be calculated
with cvCalcGlobalOrientation using extracted mask of the particular component
(using cvCmp)

---

## Object Tracking

---

### MeanShift

Finds object center on back projection

```
int cvMeanShift( const CvArr* prob_image, CvRect window,
                 CvTermCriteria criteria, CvConnectedComp* comp );
```

prob\_image: Back projection of object histogram (see cvCalcBackProject). window: Initial search window. criteria: Criteria applied to determine when the window search should be finished. comp: Resultant structure that contains converged search window coordinates (`comp->rect` field) and sum of all pixels inside the window (`comp->area` field).

The function `cvMeanShift` iterates to find the object center given its back projection and
initial position of search window. The iterations are made until the search window
center moves by less than the given value and/or until the function has done the
maximum number of iterations. The function returns the number of iterations
made.

---

### CamShift

Finds object center, size, and orientation

```
int cvCamShift( const CvArr* prob_image, CvRect window, CvTermCriteria criteria,
                CvConnectedComp* comp, CvBox2D* box=NULL );
```

prob\_image: Back projection of object histogram (see cvCalcBackProject). window: Initial search window. criteria: Criteria applied to determine when the window search should be finished. comp: Resultant structure that contains converged search window coordinates (`comp->rect` field) and sum of all pixels inside the window (`comp->area` field). box: Circumscribed box for the object. If not `NULL`, contains object size and orientation.

The function `cvCamShift` implements CAMSHIFT object tracking
algorithm ([Bradski98]).
First, it finds an object center using cvMeanShift and,
after that, calculates the object size and orientation. The function returns
number of iterations made within cvMeanShift.

CvCamShiftTracker class declared in cv.hpp implements color object tracker that uses
the function.

---

### SnakeImage

Changes contour position to minimize its energy

```
void cvSnakeImage( const IplImage* image, CvPoint* points, int length,
                   float* alpha, float* beta, float* gamma, int coeff_usage,
                   CvSize win, CvTermCriteria criteria, int calc_gradient=1 );
```

image: The source image or external energy field. points: Contour points (snake). length: Number of points in the contour. alpha: Weight[s] of continuity energy, single float or array of `length` floats, one per each contour point. beta: Weight[s] of curvature energy, similar to `alpha`. gamma: Weight[s] of image energy, similar to `alpha`. coeff\_usage: Variant of usage of the previous three parameters: - `CV_VALUE` indicates that each of `alpha, beta, gamma` is a pointer to a single value to be used for all points;- `CV_ARRAY` indicates that each of `alpha, beta, gamma` is a pointer to an array of coefficients different for all the points of the snake. All the arrays must have the size equal to the contour size. win: Size of neighborhood of every point used to search the minimum, both `win.width` and `win.height` must be odd. criteria: Termination criteria. calc\_gradient: Gradient flag. If not 0, the function calculates gradient magnitude for every image pixel and considers it as the energy field, otherwise the input image itself is considered.

The function `cvSnakeImage` updates snake in order to minimize its total energy that is a sum
of internal energy that depends on contour shape (the smoother contour is, the smaller internal energy is)
and external energy that depends on the energy field and reaches minimum at the local energy extremums
that correspond to the image edges in case of image gradient.

The parameter `criteria.epsilon` is used to define the minimal number of points
that must be moved during any iteration to keep the iteration process running.

If at some iteration the number of moved points is less than `criteria.epsilon` or the function
performed `criteria.max_iter` iterations, the function terminates.

---

## Optical Flow

---

### CalcOpticalFlowHS

Calculates optical flow for two images

```
void cvCalcOpticalFlowHS( const CvArr* prev, const CvArr* curr, int use_previous,
                          CvArr* velx, CvArr* vely, double lambda,
                          CvTermCriteria criteria );
```

prev: First image, 8-bit, single-channel. curr: Second image, 8-bit, single-channel. use\_previous: Uses previous (input) velocity field. velx: Horizontal component of the optical flow of the same size as input images, 32-bit floating-point, single-channel. vely: Vertical component of the optical flow of the same size as input images, 32-bit floating-point, single-channel. lambda: Lagrangian multiplier. criteria: Criteria of termination of velocity computing.

The function `cvCalcOpticalFlowHS` computes flow for every pixel of the first input image using
Horn & Schunck algorithm [Horn81].

---

### CalcOpticalFlowLK

Calculates optical flow for two images

```
void cvCalcOpticalFlowLK( const CvArr* prev, const CvArr* curr, CvSize win_size,
                          CvArr* velx, CvArr* vely );
```

prev: First image, 8-bit, single-channel. curr: Second image, 8-bit, single-channel. win\_size: Size of the averaging window used for grouping pixels. velx: Horizontal component of the optical flow of the same size as input images, 32-bit floating-point, single-channel. vely: Vertical component of the optical flow of the same size as input images, 32-bit floating-point, single-channel.

The function `cvCalcOpticalFlowLK` computes flow for every pixel of the first input image using
Lucas & Kanade algorithm [Lucas81].

---

### CalcOpticalFlowBM

Calculates optical flow for two images by block matching method

```
void cvCalcOpticalFlowBM( const CvArr* prev, const CvArr* curr, CvSize block_size,
                          CvSize shift_size, CvSize max_range, int use_previous,
                          CvArr* velx, CvArr* vely );
```

prev: First image, 8-bit, single-channel. curr: Second image, 8-bit, single-channel. block\_size: Size of basic blocks that are compared. shift\_size: Block coordinate increments. max\_range: Size of the scanned neighborhood in pixels around block. use\_previous: Uses previous (input) velocity field. velx: Horizontal component of the optical flow of floor((prev->width - block\_size.width)/shiftSize.width) × floor((prev->height - block\_size.height)/shiftSize.height) size, 32-bit floating-point, single-channel. vely: Vertical component of the optical flow of the same size `velx`, 32-bit floating-point, single-channel.

The function `cvCalcOpticalFlowBM` calculates optical flow for
overlapped blocks `block_size.width×block_size.height` pixels each,
thus the velocity fields are smaller than the original images. For every block in `prev`
the functions tries to find a similar block in `curr` in some neighborhood of the original
block or shifted by (velx(x0,y0),vely(x0,y0)) block as has been calculated
by previous function call (if `use_previous=1`)

---

### CalcOpticalFlowPyrLK

Calculates optical flow for a sparse feature set using iterative Lucas-Kanade method in
pyramids

```
void cvCalcOpticalFlowPyrLK( const CvArr* prev, const CvArr* curr, CvArr* prev_pyr, CvArr* curr_pyr,
                             const CvPoint2D32f* prev_features, CvPoint2D32f* curr_features,
                             int count, CvSize win_size, int level, char* status,
                             float* track_error, CvTermCriteria criteria, int flags );
```

prev: First frame, at time `t`. curr: Second frame, at time `t + dt` . prev\_pyr: Buffer for the pyramid for the first frame. If the pointer is not `NULL` , the buffer must have a sufficient size to store the pyramid from level `1` to level #`level` ; the total size of `(image_width+8)*image_height/3` bytes is sufficient. curr\_pyr: Similar to `prev_pyr`, used for the second frame. prev\_features: Array of points for which the flow needs to be found. curr\_features: Array of 2D points containing calculated new positions of input features in the second image. count: Number of feature points. win\_size: Size of the search window of each pyramid level. level: Maximal pyramid level number. If `0` , pyramids are not used (single level), if `1` , two levels are used, etc. status: Array. Every element of the array is set to `1` if the flow for the corresponding feature has been found, `0` otherwise. track\_error: Array of double numbers containing difference between patches around the original and moved points. Optional parameter; can be `NULL` . criteria: Specifies when the iteration process of finding the flow for each point on each pyramid level should be stopped. flags: Miscellaneous flags: - `CV_LKFLOW_PYR_A_READY` , pyramid for the first frame is pre-calculated before the call;- `CV_LKFLOW_PYR_B_READY` , pyramid for the second frame is pre-calculated before the call;- `CV_LKFLOW_INITIAL_GUESSES` , array B contains initial coordinates of features before the function call.

The function `cvCalcOpticalFlowPyrLK` implements
sparse iterative version of Lucas-Kanade optical flow in pyramids ([Bouguet00]).
It calculates coordinates of the feature points on the current video frame given
their coordinates on the previous frame. The function finds the coordinates with sub-pixel accuracy.

Both parameters `prev_pyr` and `curr_pyr` comply with the following rules: if the image
pointer is 0, the function allocates the buffer internally, calculates the
pyramid, and releases the buffer after processing. Otherwise, the function
calculates the pyramid and stores it in the buffer unless the flag
`CV_LKFLOW_PYR_A[B]_READY` is set. The image should be large enough to fit the
Gaussian pyramid data. After the function call both pyramids are calculated and
the readiness flag for the corresponding image can be set in the next call (i.e., typically,
for all the image pairs except the very first one `CV_LKFLOW_PYR_A_READY` is set).

---

## Feature Matching

---

### CreateFeatureTree

Constructs a tree of feature vectors

```
CvFeatureTree* cvCreateFeatureTree(CvMat* desc);
```

desc: n x d matrix of n d-dimensional feature vectors (CV\_32FC1 or CV\_64FC1).

The function `cvCreateFeatureTree` constructs a balanced kd-tree index of
the given feature vectors. The lifetime of the desc matrix must exceed that
of the returned tree. I.e., no copy is made of the vectors.

---

### ReleaseFeatureTree

Destroys a tree of feature vectors

```
void cvReleaseFeatureTree(CvFeatureTree* tr);
```

tr: pointer to tree being destroyed.

The function `cvReleaseFeatureTree` deallocates the given kd-tree.

---

### FindFeatures

Finds approximate k nearest neighbors of given vectors using best-bin-first search

```
void cvFindFeatures(CvFeatureTree* tr, CvMat* desc,
		    CvMat* results, CvMat* dist, int k=2, int emax=20);
```

tr: pointer to kd-tree index of reference vectors. desc: m x d matrix of (row-)vectors to find the nearest neighbors of. results: m x k set of row indices of matching vectors (referring to matrix passed to cvCreateFeatureTree). Contains -1 in some columns if fewer than k neighbors found. dist: m x k matrix of distances to k nearest neighbors. k: The number of neighbors to find. emax: The maximum number of leaves to visit.

The function `cvFindFeatures` finds (with high probability) the `k` nearest
neighbors in `tr` for each of the given (row-)vectors in `desc`, using
best-bin-first searching ([Beis97]).
The complexity of the entire operation is at most `O(m*emax*log2(n))`,
where `n` is the number of vectors in the tree.

---

### FindFeaturesBoxed

Orthogonal range search

```
int cvFindFeaturesBoxed(CvFeatureTree* tr,
			CvMat* bounds_min, CvMat* bounds_max,
			CvMat* results);
```

tr: pointer to kd-tree index of reference vectors. bounds\_min: 1 x d or d x 1 vector (CV\_32FC1 or CV\_64FC1) giving minimum value for each dimension. bounds\_max: 1 x d or d x 1 vector (CV\_32FC1 or CV\_64FC1) giving maximum value for each dimension. results: 1 x m or m x 1 vector (CV\_32SC1) to contain output row indices (referring to matrix passed to cvCreateFeatureTree).

The function `cvFindFeaturesBoxed` performs orthogonal range seaching on the
given kd-tree. That is, it returns the set of vectors `v` in `tr` that satisfy
`bounds_min[i] <= v[i] <= bounds_max[i], 0 <= i < d`, where `d` is the dimension
of vectors in the tree.
The function returns the number of such vectors found.

---

## Estimators

---

### CvKalman

Kalman filter state

```
typedef struct CvKalman
{
    int MP;                     /* number of measurement vector dimensions */
    int DP;                     /* number of state vector dimensions */
    int CP;                     /* number of control vector dimensions */

    /* backward compatibility fields */
#if 1
    float* PosterState;         /* =state_pre->data.fl */
    float* PriorState;          /* =state_post->data.fl */
    float* DynamMatr;           /* =transition_matrix->data.fl */
    float* MeasurementMatr;     /* =measurement_matrix->data.fl */
    float* MNCovariance;        /* =measurement_noise_cov->data.fl */
    float* PNCovariance;        /* =process_noise_cov->data.fl */
    float* KalmGainMatr;        /* =gain->data.fl */
    float* PriorErrorCovariance;/* =error_cov_pre->data.fl */
    float* PosterErrorCovariance;/* =error_cov_post->data.fl */
    float* Temp1;               /* temp1->data.fl */
    float* Temp2;               /* temp2->data.fl */
#endif

    CvMat* state_pre;           /* predicted state (x'(k)):
                                    x(k)=A*x(k-1)+B*u(k) */
    CvMat* state_post;          /* corrected state (x(k)):
                                    x(k)=x'(k)+K(k)*(z(k)-H*x'(k)) */
    CvMat* transition_matrix;   /* state transition matrix (A) */
    CvMat* control_matrix;      /* control matrix (B)
                                   (it is not used if there is no control)*/
    CvMat* measurement_matrix;  /* measurement matrix (H) */
    CvMat* process_noise_cov;   /* process noise covariance matrix (Q) */
    CvMat* measurement_noise_cov; /* measurement noise covariance matrix (R) */
    CvMat* error_cov_pre;       /* priori error estimate covariance matrix (P'(k)):
                                    P'(k)=A*P(k-1)*At + Q)*/
    CvMat* gain;                /* Kalman gain matrix (K(k)):
                                    K(k)=P'(k)*Ht*inv(H*P'(k)*Ht+R)*/
    CvMat* error_cov_post;      /* posteriori error estimate covariance matrix (P(k)):
                                    P(k)=(I-K(k)*H)*P'(k) */
    CvMat* temp1;               /* temporary matrices */
    CvMat* temp2;
    CvMat* temp3;
    CvMat* temp4;
    CvMat* temp5;
}
CvKalman;
```

The structure CvKalman is used to keep Kalman filter state. It is created
by cvCreateKalman function, updated by cvKalmanPredict and
cvKalmanCorrect functions and released by cvReleaseKalman functions.
Normally, the structure is used for standard Kalman filter (notation and the formulae below are borrowed
from the excellent Kalman tutorial [Welch95]):

```
xk=A•xk-1+B•uk+wk
zk=H•xk+vk,
```

where:

```
xk (xk-1) - state of the system at the moment k (k-1)
zk - measurement of the system state at the moment k
uk - external control applied at the moment k

wk and vk are normally-distributed process and measurement noise, respectively:
p(w) ~ N(0,Q)
p(v) ~ N(0,R),

that is,
Q - process noise covariance matrix, constant or variable,
R - measurement noise covariance matrix, constant or variable
```

In case of standard Kalman filter, all the matrices: A, B, H, Q and R are initialized once after
CvKalman structure is allocated via cvCreateKalman.
However, the same structure and the same functions may be used to simulate extended Kalman filter by
linearizing extended Kalman filter equation in the current system state neighborhood,
in this case A, B, H (and, probably, Q and R) should be updated on every step.

---

### CreateKalman

Allocates Kalman filter structure

```
CvKalman* cvCreateKalman( int dynam_params, int measure_params, int control_params=0 );
```

dynam\_params: dimensionality of the state vector measure\_params: dimensionality of the measurement vector control\_params: dimensionality of the control vector

The function `cvCreateKalman` allocates CvKalman and all its matrices
and initializes them somehow.

---

### ReleaseKalman

Deallocates Kalman filter structure

```
void cvReleaseKalman( CvKalman** kalman );
```

kalman: double pointer to the Kalman filter structure.

The function `cvReleaseKalman` releases the structure CvKalman
and all underlying matrices.

---

### KalmanPredict

Estimates subsequent model state

```
const CvMat* cvKalmanPredict( CvKalman* kalman, const CvMat* control=NULL );
#define cvKalmanUpdateByTime cvKalmanPredict
```

kalman: Kalman filter state. control: Control vector (u_k_), should be NULL iff there is no external control (`control_params`=0).

The function `cvKalmanPredict` estimates the subsequent stochastic model state
by its current state and stores it at `kalman->state_pre`:

```
    x'k=A•xk+B•uk
    P'k=A•Pk-1*AT + Q,
where
x'k is predicted state (kalman->state_pre),
xk-1 is corrected state on the previous step (kalman->state_post)
                (should be initialized somehow in the beginning, zero vector by default),
uk is external control (control parameter),
P'k is priori error covariance matrix (kalman->error_cov_pre)
Pk-1 is posteriori error covariance matrix on the previous step (kalman->error_cov_post)
                (should be initialized somehow in the beginning, identity matrix by default),
```

The function returns the estimated state.

---

### KalmanCorrect

Adjusts model state

```
const CvMat* cvKalmanCorrect( CvKalman* kalman, const CvMat* measurement );
#define cvKalmanUpdateByMeasurement cvKalmanCorrect
```

kalman: Pointer to the structure to be updated. measurement: Pointer to the structure CvMat containing the measurement vector.

The function `cvKalmanCorrect` adjusts stochastic model state on the
basis of the given measurement of the model state:

```
Kk=P'k•HT•(H•P'k•HT+R)-1
xk=x'k+Kk•(zk-H•x'k)
Pk=(I-Kk•H)•P'k
where
zk - given measurement (mesurement parameter)
Kk - Kalman "gain" matrix.
```

The function stores adjusted state at `kalman->state_post` and returns it on output.

#### Example. Using Kalman filter to track a rotating point

```
#include "cv.h"
#include "highgui.h"
#include <math.h>

int main(int argc, char** argv)
{
    /* A matrix data */
    const float A[] = { 1, 1, 0, 1 };

    IplImage* img = cvCreateImage( cvSize(500,500), 8, 3 );
    CvKalman* kalman = cvCreateKalman( 2, 1, 0 );
    /* state is (phi, delta_phi) - angle and angle increment */
    CvMat* state = cvCreateMat( 2, 1, CV_32FC1 );
    CvMat* process_noise = cvCreateMat( 2, 1, CV_32FC1 );
    /* only phi (angle) is measured */
    CvMat* measurement = cvCreateMat( 1, 1, CV_32FC1 );
    CvRandState rng;
    int code = -1;

    cvRandInit( &rng, 0, 1, -1, CV_RAND_UNI );

    cvZero( measurement );
    cvNamedWindow( "Kalman", 1 );

    for(;;)
    {
        cvRandSetRange( &rng, 0, 0.1, 0 );
        rng.disttype = CV_RAND_NORMAL;

        cvRand( &rng, state );

        memcpy( kalman->transition_matrix->data.fl, A, sizeof(A));
        cvSetIdentity( kalman->measurement_matrix, cvRealScalar(1) );
        cvSetIdentity( kalman->process_noise_cov, cvRealScalar(1e-5) );
        cvSetIdentity( kalman->measurement_noise_cov, cvRealScalar(1e-1) );
        cvSetIdentity( kalman->error_cov_post, cvRealScalar(1));
        /* choose random initial state */
        cvRand( &rng, kalman->state_post );

        rng.disttype = CV_RAND_NORMAL;

        for(;;)
        {
            #define calc_point(angle)                                      \
                cvPoint( cvRound(img->width/2 + img->width/3*cos(angle)),  \
                         cvRound(img->height/2 - img->width/3*sin(angle)))

            float state_angle = state->data.fl[0];
            CvPoint state_pt = calc_point(state_angle);

            /* predict point position */
            const CvMat* prediction = cvKalmanPredict( kalman, 0 );
            float predict_angle = prediction->data.fl[0];
            CvPoint predict_pt = calc_point(predict_angle);
            float measurement_angle;
            CvPoint measurement_pt;

            cvRandSetRange( &rng, 0, sqrt(kalman->measurement_noise_cov->data.fl[0]), 0 );
            cvRand( &rng, measurement );

            /* generate measurement */
            cvMatMulAdd( kalman->measurement_matrix, state, measurement, measurement );

            measurement_angle = measurement->data.fl[0];
            measurement_pt = calc_point(measurement_angle);

            /* plot points */
            #define draw_cross( center, color, d )                                 \
                cvLine( img, cvPoint( center.x - d, center.y - d ),                \
                             cvPoint( center.x + d, center.y + d ), color, 1, 0 ); \
                cvLine( img, cvPoint( center.x + d, center.y - d ),                \
                             cvPoint( center.x - d, center.y + d ), color, 1, 0 )

            cvZero( img );
            draw_cross( state_pt, CV_RGB(255,255,255), 3 );
            draw_cross( measurement_pt, CV_RGB(255,0,0), 3 );
            draw_cross( predict_pt, CV_RGB(0,255,0), 3 );
            cvLine( img, state_pt, predict_pt, CV_RGB(255,255,0), 3, 0 );

            /* adjust Kalman filter state */
            cvKalmanCorrect( kalman, measurement );

            cvRandSetRange( &rng, 0, sqrt(kalman->process_noise_cov->data.fl[0]), 0 );
            cvRand( &rng, process_noise );
            cvMatMulAdd( kalman->transition_matrix, state, process_noise, state );

            cvShowImage( "Kalman", img );
            code = cvWaitKey( 100 );

            if( code > 0 ) /* break current simulation by pressing a key */
                break;
        }
        if( code == 27 ) /* exit by ESCAPE */
            break;
    }

    return 0;
}
```

---

### CvConDensation

ConDenstation state

```
    typedef struct CvConDensation
    {
        int MP;     //Dimension of measurement vector
        int DP;     // Dimension of state vector
        float* DynamMatr;       // Matrix of the linear Dynamics system
        float* State;           // Vector of State
        int SamplesNum;         // Number of the Samples
        float** flSamples;      // array of the Sample Vectors
        float** flNewSamples;   // temporary array of the Sample Vectors
        float* flConfidence;    // Confidence for each Sample
        float* flCumulative;    // Cumulative confidence
        float* Temp;            // Temporary vector
        float* RandomSample;    // RandomVector to update sample set
        CvRandState* RandS;     // Array of structures to generate random vectors
    } CvConDensation;
```

The structure CvConDensation stores CONditional DENSity propagATION tracker state.
The information about the algorithm can be found at
http://www.dai.ed.ac.uk/CVonline/LOCAL\_COPIES/ISARD1/condensation.html

---

### CreateConDensation

Allocates ConDensation filter structure

```
CvConDensation* cvCreateConDensation( int dynam_params, int measure_params, int sample_count );
```

dynam\_params: Dimension of the state vector. measure\_params: Dimension of the measurement vector. sample\_count: Number of samples.

The function `cvCreateConDensation` creates CvConDensation
structure and returns pointer to the structure.

---

### ReleaseConDensation

Deallocates ConDensation filter structure

```
void cvReleaseConDensation( CvConDensation** condens );
```

condens: Pointer to the pointer to the structure to be released.

The function `cvReleaseConDensation` releases the structure CvConDensation (see
cvConDensation) and frees all memory previously allocated for the structure.

---

### ConDensInitSampleSet

Initializes sample set for ConDensation algorithm

```
void cvConDensInitSampleSet( CvConDensation* condens, CvMat* lower_bound, CvMat* upper_bound );
```

condens: Pointer to a structure to be initialized. lower\_bound: Vector of the lower boundary for each dimension. upper\_bound: Vector of the upper boundary for each dimension.

The function `cvConDensInitSampleSet` fills the samples arrays in the structure
CvConDensation with values within specified ranges.

---

### ConDensUpdateByTime

Estimates subsequent model state

```
void cvConDensUpdateByTime( CvConDensation* condens );
```

condens: Pointer to the structure to be updated.

The function `cvConDensUpdateByTime`
estimates the subsequent stochastic model state from its current state.

---

# Pattern Recognition

---

## Object Detection

The object detector described below has been initially proposed by Paul Viola
[Viola01] and improved by Rainer Lienhart
[Lienhart02].
First, a classifier (namely a `cascade of boosted classifiers working
with haar-like features`) is trained with a few hundreds of sample
views of a particular object (i.e., a face or a car), called positive
examples, that are scaled to the same size (say, 20x20), and negative examples
- arbitrary images of the same size.

After a classifier is trained, it can be applied to a region of interest (of
the same size as used during the training) in an input image. The
classifier outputs a "1" if the region is likely to show the object
(i.e., face/car), and "0" otherwise. To search for the object in the
whole image one can move the search window across the image and check
every location using the classifier. The classifier is designed so that it can
be easily "resized" in order to be able to find the objects of interest
at different sizes, which is more efficient than resizing the image itself. So,
to find an object of an unknown size in the image the scan procedure should be
done several times at different scales.

The word "cascade" in the classifier name means that the resultant classifier
consists of several simpler classifiers (`stages`) that are applied
subsequently to a region of interest until at some stage the candidate
is rejected or all the stages are passed. The word
"boosted" means that the classifiers at every stage of the cascade are complex
themselves and they are built out of basic classifiers using one of four
different `boosting` techniques (weighted voting). Currently
Discrete Adaboost, Real Adaboost, Gentle Adaboost and Logitboost are supported.
The basic classifiers are decision-tree classifiers with at least
2 leaves. Haar-like features are the input to the basic classifiers, and
are calculated as described below. The current algorithm uses the following
Haar-like features:

The feature used in a particular classifier is specified by its shape (1a,
2b etc.), position within the region of interest and the scale (this scale is
not the same as the scale used at the detection stage, though these two scales
are multiplied). For example, in case of the third line feature (2c) the
response is calculated as the difference between the sum of image pixels
under the rectangle covering the whole feature (including the two white
stripes and the black stripe in the middle) and the sum of the image
pixels under the black stripe multiplied by 3 in order to compensate for
the differences in the size of areas. The sums of pixel values over a
rectangular regions are calculated rapidly using integral images
(see below and 
cvIntegral description).

To see the object detector at work, have a look at HaarFaceDetect demo.

The following reference is for the detection part only. There is a
separate application called `haartraining` that can train a
cascade of boosted classifiers from a set of samples.
See `opencv/apps/haartraining` for details.

---

### CvHaarFeature, CvHaarClassifier, CvHaarStageClassifier, CvHaarClassifierCascade

Boosted Haar classifier structures

```
#define CV_HAAR_FEATURE_MAX  3

/* a haar feature consists of 2-3 rectangles with appropriate weights */
typedef struct CvHaarFeature
{
    int  tilted;  /* 0 means up-right feature, 1 means 45--rotated feature */
    
    /* 2-3 rectangles with weights of opposite signs and
       with absolute values inversely proportional to the areas of the rectangles.
       if rect[2].weight !=0, then
       the feature consists of 3 rectangles, otherwise it consists of 2 */
    struct
    {
        CvRect r;
        float weight;
    } rect[CV_HAAR_FEATURE_MAX];
}
CvHaarFeature;

/* a single tree classifier (stump in the simplest case) that returns the response for the feature
   at the particular image location (i.e. pixel sum over sub-rectangles of the window) and gives out
   a value depending on the response */
typedef struct CvHaarClassifier
{
    int count;  /* number of nodes in the decision tree */

    /* these are "parallel" arrays. Every index i
       corresponds to a node of the decision tree (root has 0-th index).

       left[i] - index of the left child (or negated index if the left child is a leaf)
       right[i] - index of the right child (or negated index if the right child is a leaf)
       threshold[i] - branch threshold. if feature response is <= threshold, left branch
                      is chosen, otherwise right branch is chosen.
       alpha[i] - output value corresponding to the leaf. */
    CvHaarFeature* haar_feature;
    float* threshold;
    int* left;
    int* right;
    float* alpha;
}
CvHaarClassifier;

/* a boosted battery of classifiers(=stage classifier):
   the stage classifier returns 1
   if the sum of the classifiers' responses
   is greater than threshold and 0 otherwise */
typedef struct CvHaarStageClassifier
{
    int  count;  /* number of classifiers in the battery */
    float threshold; /* threshold for the boosted classifier */
    CvHaarClassifier* classifier; /* array of classifiers */

    /* these fields are used for organizing trees of stage classifiers,
       rather than just straight cascades */
    int next;
    int child;
    int parent;
}
CvHaarStageClassifier;

typedef struct CvHidHaarClassifierCascade CvHidHaarClassifierCascade;

/* cascade or tree of stage classifiers */
typedef struct CvHaarClassifierCascade
{
    int  flags; /* signature */
    int  count; /* number of stages */
    CvSize orig_window_size; /* original object size (the cascade is trained for) */

    /* these two parameters are set by cvSetImagesForHaarClassifierCascade */
    CvSize real_window_size; /* current object size */
    double scale; /* current scale */
    CvHaarStageClassifier* stage_classifier; /* array of stage classifiers */
    CvHidHaarClassifierCascade* hid_cascade; /* hidden optimized representation of the cascade,
                                                created by cvSetImagesForHaarClassifierCascade */
}
CvHaarClassifierCascade;
```

All the structures are used for representing a cascaded of boosted Haar
classifiers. The cascade has the following hierarchical structure:

```
    Cascade:
        Stage1:
            Classifier11:
                Feature11
            Classifier12:
                Feature12
            ...
        Stage2:
            Classifier21:
                Feature21
            ...
        ...
```

The whole hierarchy can be constructed manually or loaded from a file
using functions cvLoadHaarClassifierCascade
or cvLoad.

---

### cvLoadHaarClassifierCascade

Loads a trained cascade classifier from file
or the classifier database embedded in OpenCV

```
CvHaarClassifierCascade* cvLoadHaarClassifierCascade(
                         const char* directory,
                         CvSize orig_window_size );
```

directory: Name of directory containing the description of a trained cascade classifier. orig\_window\_size: Original size of objects the cascade has been trained on. Note that it is not stored in the cascade and therefore must be specified separately.

The function `cvLoadHaarClassifierCascade`
loads a trained cascade of haar classifiers from a file or the classifier
database embedded in OpenCV. The base can be trained using `haartraining`
application (see opencv/apps/haartraining for details).

*The function is obsolete*. Nowadays object detection classifiers are stored in
XML or YAML files, rather than in directories. To load cascade from a
file, use cvLoad function.

---

### cvReleaseHaarClassifierCascade

Releases haar classifier cascade

```
void cvReleaseHaarClassifierCascade( CvHaarClassifierCascade** cascade );
```

cascade: Double pointer to the released cascade. The pointer is cleared by the function.

The function `cvReleaseHaarClassifierCascade`
deallocates the cascade that has been created manually or loaded using
cvLoadHaarClassifierCascade or
cvLoad.

---

### cvHaarDetectObjects

Detects objects in the image

```
typedef struct CvAvgComp
{
    CvRect rect; /* bounding rectangle for the object (average rectangle of a group) */
    int neighbors; /* number of neighbor rectangles in the group */
}
CvAvgComp;

CvSeq* cvHaarDetectObjects( const CvArr* image, CvHaarClassifierCascade* cascade,
                            CvMemStorage* storage, double scale_factor=1.1,
                            int min_neighbors=3, int flags=0,
                            CvSize min_size=cvSize(0,0) );
```

image: Image to detect objects in. cascade: Haar classifier cascade in internal representation. storage: Memory storage to store the resultant sequence of the object candidate rectangles. scale\_factor: The factor by which the search window is scaled between the subsequent scans, for example, 1.1 means increasing window by 10%. min\_neighbors: Minimum number (minus 1) of neighbor rectangles that makes up an object. All the groups of a smaller number of rectangles than `min_neighbors`-1 are rejected. If `min_neighbors` is 0, the function does not any grouping at all and returns all the detected candidate rectangles, which may be useful if the user wants to apply a customized grouping procedure. flags: Mode of operation. It can be a combination of zero or more of the following values: `CV_HAAR_SCALE_IMAGE` - for each scale factor used the function will downscale the image rather than "zoom" the feature coordinates in the classifier cascade. Currently, the option can only be used alone, i.e. the flag can not be set together with the others. `CV_HAAR_DO_CANNY_PRUNING` - If it is set, the function uses Canny edge detector to reject some image regions that contain too few or too much edges and thus can not contain the searched object. The particular threshold values are tuned for face detection and in this case the pruning speeds up the processing. `CV_HAAR_FIND_BIGGEST_OBJECT` - If it is set, the function finds the largest object (if any) in the image. That is, the output sequence will contain one (or zero) element(s). `CV_HAAR_DO_ROUGH_SEARCH` - It should be used only when `CV_HAAR_FIND_BIGGEST_OBJECT` is set and `min_neighbors` > 0. If the flag is set, the function does not look for candidates of a smaller size as soon as it has found the object (with enough neighbor candidates) at the current scale. Typically, when `min_neighbors` is fixed, the mode yields less accurate (a bit larger) object rectangle than the regular single-object mode (`flags`=`CV_HAAR_FIND_BIGGEST_OBJECT`), but it is much faster, up to an order of magnitude. A greater value of `min_neighbors` may be specified to improve the accuracy. Note, that in single-object mode `CV_HAAR_DO_CANNY_PRUNING` does not improve performance much and can even slow down the processing. min\_size: Minimum window size. By default, it is set to the size of samples the classifier has been trained on (~20×20 for face detection).

The function `cvHaarDetectObjects` finds
rectangular regions in the given image that are likely to contain objects
the cascade has been trained for and returns those regions as
a sequence of rectangles. The function scans the image several
times at different scales (see 
cvSetImagesForHaarClassifierCascade). Each time it considers
overlapping regions in the image and applies the classifiers to the regions
using cvRunHaarClassifierCascade.
It may also apply some heuristics to reduce number of analyzed regions, such as
Canny pruning. After it has proceeded and collected the candidate rectangles
(regions that passed the classifier cascade), it groups them and returns a
sequence of average rectangles for each large enough group. The default
parameters (`scale_factor`=1.1, `min_neighbors`=3, `flags`=0)
are tuned for accurate yet slow object detection. For a faster operation on
real video images the more preferable settings are: `scale_factor`=1.2, `min_neighbors`=2,
`flags`=CV\_HAAR\_DO\_CANNY\_PRUNING, `min_size`=<minimum possible face size>
(for example, ~1/4 to 1/16 of the image area in case of video conferencing).

#### Example. Using cascade of Haar classifiers to find objects (e.g. faces).

```
#include "cv.h"
#include "highgui.h"

CvHaarClassifierCascade* load_object_detector( const char* cascade_path )
{
    return (CvHaarClassifierCascade*)cvLoad( cascade_path );
}

void detect_and_draw_objects( IplImage* image,
                              CvHaarClassifierCascade* cascade,
                              int do_pyramids )
{
    IplImage* small_image = image;
    CvMemStorage* storage = cvCreateMemStorage(0);
    CvSeq* faces;
    int i, scale = 1;

    /* if the flag is specified, down-scale the input image to get a
       performance boost w/o loosing quality (perhaps) */
    if( do_pyramids )
    {
        small_image = cvCreateImage( cvSize(image->width/2,image->height/2), IPL_DEPTH_8U, 3 );
        cvPyrDown( image, small_image, CV_GAUSSIAN_5x5 );
        scale = 2;
    }

    /* use the fastest variant */
    faces = cvHaarDetectObjects( small_image, cascade, storage, 1.2, 2, CV_HAAR_DO_CANNY_PRUNING );

    /* draw all the rectangles */
    for( i = 0; i < faces->total; i++ )
    {
        /* extract the rectangles only */
        CvRect face_rect = *(CvRect*)cvGetSeqElem( faces, i, 0 );
        cvRectangle( image, cvPoint(face_rect.x*scale,face_rect.y*scale),
                     cvPoint((face_rect.x+face_rect.width)*scale,
                             (face_rect.y+face_rect.height)*scale),
                     CV_RGB(255,0,0), 3 );
    }

    if( small_image != image )
        cvReleaseImage( &small_image );
    cvReleaseMemStorage( &storage );
}

/* takes image filename and cascade path from the command line */
int main( int argc, char** argv )
{
    IplImage* image;
    if( argc==3 && (image = cvLoadImage( argv[1], 1 )) != 0 )
    {
        CvHaarClassifierCascade* cascade = load_object_detector(argv[2]);
        detect_and_draw_objects( image, cascade, 1 );
        cvNamedWindow( "test", 0 );
        cvShowImage( "test", image );
        cvWaitKey(0);
        cvReleaseHaarClassifierCascade( &cascade );
        cvReleaseImage( &image );
    }

    return 0;
}
```

---

### cvSetImagesForHaarClassifierCascade

Assigns images to the hidden cascade

```
void cvSetImagesForHaarClassifierCascade( CvHaarClassifierCascade* cascade,
                                          const CvArr* sum, const CvArr* sqsum,
                                          const CvArr* tilted_sum, double scale );
```

cascade: Hidden Haar classifier cascade, created by cvCreateHidHaarClassifierCascade. sum: Integral (sum) single-channel image of 32-bit integer format. This image as well as the two subsequent images are used for fast feature evaluation and brightness/contrast normalization. They all can be retrieved from input 8-bit or floating point single-channel image using The function `cvIntegral`. sqsum: Square sum single-channel image of 64-bit floating-point format. tilted\_sum: Tilted sum single-channel image of 32-bit integer format. scale: Window scale for the cascade. If `scale`=1, original window size is used (objects of that size are searched) - the same size as specified in cvLoadHaarClassifierCascade (24x24 in case of "<default\_face\_cascade>"), if `scale`=2, a two times larger window is used (48x48 in case of default face cascade). While this will speed-up search about four times, faces smaller than 48x48 cannot be detected.

The function `cvSetImagesForHaarClassifierCascade`
assigns images and/or window scale to the hidden classifier cascade.
If image pointers are NULL, the previously set images are used further
(i.e. NULLs mean "do not change images"). Scale parameter has no such a "protection" value, but
the previous value can be retrieved by 
cvGetHaarClassifierCascadeScale function and reused again. The function
is used to prepare cascade for detecting object of the particular size in the
particular image. The function is called internally by 
cvHaarDetectObjects, but it can be called by user if there is a need in
using lower-level function cvRunHaarClassifierCascade.

---

### cvRunHaarClassifierCascade

Runs cascade of boosted classifier at given image location

```
int cvRunHaarClassifierCascade( CvHaarClassifierCascade* cascade,
                                CvPoint pt, int start_stage=0 );
```

cascade: Haar classifier cascade. pt: Top-left corner of the analyzed region. Size of the region is a original window size scaled by the currently set scale. The current window size may be retrieved using cvGetHaarClassifierCascadeWindowSize function. start\_stage: Initial zero-based index of the cascade stage to start from. The function assumes that all the previous stages are passed. This feature is used internally by cvHaarDetectObjects for better processor cache utilization.

The function `cvRunHaarHaarClassifierCascade`
runs Haar classifier cascade at a single image location. Before using this
function the integral images and the appropriate scale (=> window size)
should be set using cvSetImagesForHaarClassifierCascade.
The function returns positive value if the analyzed rectangle passed all the classifier
stages (it is a candidate) and zero or negative value otherwise.

---

# Camera Calibration and 3D Reconstruction

---

## Pinhole Camera Model, Distortion

The functions in this section use so-called pinhole camera model. That is,
a scene view is formed by projecting 3D points into the image plane using perspective transformation.

```
s*m' = A*[R|t]*M', or

 [u]   [fx 0 cx] [r11 r12 r13 t1] [X]
s[v] = [0 fy cy]*[r21 r22 r23 t2]*[Y]
 [1]   [0  0  1] [r31 r32 r33 t2] [Z]
                                 [1]
```

Where `(X, Y, Z)` are coordinates of a 3D point in the world coordinate space,
`(u, v)` are coordinates of point projection in pixels.
`A` is called a camera matrix, or matrix of intrinsic parameters.
`(cx, cy)` is a principal point (that is usually at the image center),
and `fx, fy` are focal lengths expressed in pixel-related units.
Thus, if an image from camera is up-sampled/down-sampled by some factor,
all these parameters (`fx, fy, cx` and `cy`) should be scaled
(multiplied/divided, respectively) by the same factor.
The matrix of intrinsic parameters does not depend on the scene viewed
and, once estimated, can be re-used (as long as the focal length is fixed (in case of zoom lens)).
The joint rotation-translation matrix `[R|t]` is called a matrix of extrinsic parameters.
It is used to describe the camera motion around a static scene, or vice versa,
rigid motion of an object in front of still camera. That is, `[R|t]` translates coordinates
of a point `(X, Y, Z)` to some coordinate system, fixed with respect to the camera.
The transformation above is equivalent to the following (when z≠0):

```
[x]     [X]
[y] = R*[Y] + t
[z]     [Z]

x' = x/z
y' = y/z

u = fx*x' + cx
v = fy*y' + cy
```

Real lens usually have some distortion, which is mainly a radial distortion
and slight tangential distortion. So, the above model is extended as:

```
[x]     [X]
[y] = R*[Y] + t
[z]     [Z]

x' = x/z
y' = y/z

x" = x'*(1 + k1r2 + k2r4 + k3r6) + 2*p1x'*y' + p2(r2+2*x'2)
y" = y'*(1 + k1r2 + k2r4 + k3r6) + p1(r2+2*y'2) + 2*p2*x'*y'
where r2 = x'2+y'2

u = fx*x" + cx
v = fy*y" + cy
```

k_1_, k_2_, k_3_ are radial distortion coefficients,
p_1_, p_2_ are tangential distortion coefficients.
Higher-order coefficients are not considered in OpenCV.
The distortion coefficients also do not depend on the scene viewed,
thus they are intrinsic camera parameters.
**And they remain the same regardless of the captured image resolution.**
That is, if, for example, a camera has been calibrated on images of 320x240 resolution, absolutely the same distortion
coefficients can be used for images of 640x480 resolution from the same camera (while fx, fy, cx and cy need to be scaled appropriately).

Another note.Many of calibration related functions take the vector of distortion coefficients. It can be 4x1, 1x4, 5x1 or 1x5
floating-point vector (CvMat\*). The ordering of the distortion coefficients is the following:

```
(k1, k2, p1, p2[, k3]).
```

That is, the first 2 radial distortion coefficients are followed by 2 tangential distortion coefficients and then, optionally,
by the third radial distortion coefficients. Such ordering is used to keep backward compatibility with previous versions of OpenCV.

The functions below use the above model to

- Project 3D points to the image plane given intrinsic and extrinsic parameters- Compute extrinsic parameters given intrinsic parameters, a few 3D points and their projections.- Estimate intrinsic and extrinsic camera parameters from several views of a known calibration pattern
      (i.e. every view is described by several 3D-2D point correspondences).

The functionality described in this section is largely based on the camera calibration toolbox
[Bouguet04]


---

## Single and Stereo Camera Calibration

---

### ProjectPoints2

Projects 3D points to image plane

```
void cvProjectPoints2( const CvMat* object_points, const CvMat* rotation_vector,
                       const CvMat* translation_vector, const CvMat* intrinsic_matrix,
                       const CvMat* distortion_coeffs, CvMat* image_points,
                       CvMat* dpdrot=NULL, CvMat* dpdt=NULL, CvMat* dpdf=NULL,
                       CvMat* dpdc=NULL, CvMat* dpddist=NULL, double aspect_ratio=0 );
```

object\_points: The array of object points, 3xN or Nx3, where N is the number of points in the view. rotation\_vector: The rotation vector, 1x3 or 3x1. translation\_vector: The translation vector, 1x3 or 3x1. intrinsic\_matrix: The camera matrix (A) [fx 0 cx; 0 fy cy; 0 0 1]. distortion\_coeffs: The vector of distortion coefficients, 4x1, 1x4, 5x1 or 1x5. If the vector is NULL, the function assumes that all the distortion coefficients are 0's. image\_points: The output array of image points, 2xN or Nx2, where N is the total number of points in the view. dpdrot: Optional Nx3 matrix of derivatives of image points with respect to components of the rotation vector. dpdt: Optional Nx3 matrix of derivatives of image points w.r.t. components of the translation vector. dpdf: Optional Nx2 matrix of derivatives of image points w.r.t. fx and fy. dpdc: Optional Nx2 matrix of derivatives of image points w.r.t. cx and cy. dpddist: Optional Nx4 matrix of derivatives of image points w.r.t. distortion coefficients. aspect\_ratio: Optional aspect ratio parameter used to correct the output `dpdf`. (When cvCalibrateCamera2 or cvStereoCalibrate are called with the flag `CV_CALIB_FIX_ASPECT_RATIO`, only `fy` is estimated as independent parameter, and `fx` is computed as `fy*apect_ratio`; this affects `dpdf` too). If the parameter is 0, it means that the aspect ratio is not fixed.

The function `cvProjectPoints2` computes projections of 3D points to the image plane given
intrinsic and extrinsic camera parameters. Optionally, the function computes Jacobians - matrices of
partial derivatives of image points as functions of all the input parameters w.r.t. the particular parameters,
intrinsic and/or extrinsic. The Jacobians are used during the global optimization in
cvCalibrateCamera2 and
cvFindExtrinsicCameraParams2.
The function itself is also used to compute reprojection error for with current
intrinsic and extrinsic parameters.

Note, that with intrinsic and/or extrinsic parameters set to special values,
the function can be used to compute just extrinsic transformation or just intrinsic
transformation (i.e. distortion of a sparse set of points).

---

### FindHomography

Finds perspective transformation between two planes

```
void cvFindHomography( const CvMat* src_points, const CvMat* dst_points,
                       CvMat* homography, int method=0,
                       double ransacReprojThreshold=0, CvMat* mask=NULL );
```

src\_points: Point coordinates in the original plane, 2xN, Nx2, 3xN or Nx3 array (the latter two are for representation in homogeneous coordinates), where N is the number of points. dst\_points: Point coordinates in the destination plane, 2xN, Nx2, 3xN or Nx3 array (the latter two are for representation in homogeneous coordinates) homography: Output 3x3 homography matrix. method: The method used to computed homography matrix. One of: `0` - regular method using all the point pairs `CV_RANSAC` - RANSAC-based robust method `CV_LMEDS` - Least-Median robust method ransacReprojThreshold: The maximum allowed reprojection error to treat a point pair as an inlier. The parameter is only used in RANSAC-based homography estimation. E.g. if `dst_points` coordinates are measured in pixels with pixel-accurate precision, it makes sense to set this parameter somewhere in the range ~1..3. mask: The optional output mask set by a robust method (`CV_RANSAC` or `CV_LMEDS`).

The function `cvFindHomography` finds perspective transformation `H=||hij||` between the source
and the destination planes:

```
  [x'i]   [xi]
si[y'i]~H*[yi]
  [1  ]  [ 1]
```

So that the reprojection error is minimized:

```
sum_i((x'i-(h11*xi + h12*yi + h13)/(h31*xi + h32*yi + h33))2+
      (y'i-(h21*xi + h22*yi + h23)/(h31*xi + h32*yi + h33))2) -> min
```

If the parameter `method` is set to the default value 0, the function uses all the point pairs and estimates
the best suitable homography matrix. However, if there can not all the points pairs
(`src_points`_i_, `dst_points`_i_) fit the rigid perspective transformation
(i.e. there can be outliers), it is still possible to estimate the correct transformation using one of the robust methods available.
Both methods, `CV_RANSAC` and `CV_LMEDS`, try many different random subsets of the corresponding point pairs
(of 5 pairs each), estimate homography matrix using this subset using simple least-square algorithm and then compute
quality/goodness of the computed homography (which is the number of inliers for RANSAC or the median reprojection error for LMeDs).
The best subset is then used to produce the initial estimate of the homography matrix and the mask of inliers/outliers.

Regardless of the method, robust or not, the computed homography matrix is refined further (using inliers only in case of a robust method)
with Levenberg-Marquardt method in order to reduce the reprojection error even more.

The method `CV_RANSAC` can handle practically any ratio of outliers, but it needs the threshold to distinguish inliers from outliers.
The method `CV_LMEDS` does not need any threshold, but it works correctly only when there are more than 50% of inliers.
Finally, if you are sure in the computed features and there can be only some small noise, but no outliers,
the default method could be the best choice.

The function is used to find initial intrinsic and extrinsic matrices.
Homography matrix is determined up to a scale, thus it is normalized to make h33=1.

---

### CalibrateCamera2

Finds intrinsic and extrinsic camera parameters using calibration pattern

```
void cvCalibrateCamera2( const CvMat* object_points, const CvMat* image_points,
                         const CvMat* point_counts, CvSize image_size,
                         CvMat* intrinsic_matrix, CvMat* distortion_coeffs,
                         CvMat* rotation_vectors=NULL, CvMat* translation_vectors=NULL,
                         int flags=0 );
```

object\_points: The joint matrix of object points, 3xN or Nx3, where N is the total number of points in all views. image\_points: The joint matrix of corresponding image points, 2xN or Nx2, where N is the total number of points in all views. point\_counts: Vector containing numbers of points in each particular view, 1xM or Mx1, where M is the number of a scene views. image\_size: Size of the image, used only to initialize intrinsic camera matrix. intrinsic\_matrix: The output camera matrix (A) [fx 0 cx; 0 fy cy; 0 0 1]. If `CV_CALIB_USE_INTRINSIC_GUESS` and/or `CV_CALIB_FIX_ASPECT_RATIO` are specified, some or all of `fx, fy, cx, cy` must be initialized. distortion\_coeffs: The output vector of distortion coefficients, 4x1, 1x4, 5x1 or 1x5. rotation\_vectors: The output 3xM or Mx3 array of rotation vectors (compact representation of rotation matrices, see cvRodrigues2). translation\_vectors: The output 3xM or Mx3 array of translation vectors. flags: Different flags, may be 0 or combination of the following values: `CV_CALIB_USE_INTRINSIC_GUESS` - `intrinsic_matrix` contains valid initial values of `fx, fy, cx, cy` that are optimized further. Otherwise, `(cx, cy)` is initially set to the image center (`image_size` is used here), and focal distances are computed in some least-squares fashion. Note, that if intrinsic parameters are known, there is no need to use this function. Use cvFindExtrinsicCameraParams2 instead. `CV_CALIB_FIX_PRINCIPAL_POINT` - The principal point is not changed during the global optimization, it stays at the center and at the other location specified (when `CV_CALIB_FIX_FOCAL_LENGTH` - Both fx and fy are fixed. `CV_CALIB_USE_INTRINSIC_GUESS` is set as well). `CV_CALIB_FIX_ASPECT_RATIO` - The optimization procedure consider only one of `fx` and `fy` as independent variable and keeps the aspect ratio `fx/fy` the same as it was set initially in `intrinsic_matrix`. In this case the actual initial values of `(fx, fy)` are either taken from the matrix (when `CV_CALIB_USE_INTRINSIC_GUESS` is set) or estimated somehow (in the latter case `fx, fy` may be set to arbitrary values, only their ratio is used). `CV_CALIB_ZERO_TANGENT_DIST` - Tangential distortion coefficients are set to zeros and do not change during the optimization. `CV_CALIB_FIX_K1` - The 0-th distortion coefficient (k1) is fixed (to 0 or to the initial passed value if `CV_CALIB_USE_INTRINSIC_GUESS` is passed) `CV_CALIB_FIX_K2` - The 1-st distortion coefficient (k2) is fixed (see above) `CV_CALIB_FIX_K3` - The 4-th distortion coefficient (k3) is fixed (see above)

The function `cvCalibrateCamera2` estimates intrinsic camera parameters and, optionally, the extrinsic parameters
for each view of the calibration pattern.
The coordinates of 3D object points and their correspondent 2D projections in each view
must be specified. That may be achieved by using an object with known geometry and easily detectable
feature points. Such an object is called a calibration rig or calibration pattern, and OpenCV has built-in
support for a chess board as a calibration rig
(see cvFindChessboardCorners).
Currently, initialization of intrinsic parameters (when `CV_CALIB_USE_INTRINSIC_GUESS`
is not set) is only implemented for planar calibration rigs (z-coordinates of object points
must be all 0's or all 1's). 3D rigs can still be used as long as the initial `intrinsic_matrix`
is provided. After the initial values of intrinsic and extrinsic parameters are obtained by the function, they are
optimized further to minimize the total reprojection error - the sum of squared differences between the
actual coordinates of image points and the ones computed using
cvProjectPoints2 with current intrinsic and extrinsic parameters.

---

### CalibrationMatrixValues

Finds intrinsic and extrinsic camera parameters using calibration pattern

```
void cvCalibrationMatrixValues( const CvMat *calibMatr,
                                int imgWidth, int imgHeight,
                                double apertureWidth=0, double apertureHeight=0,
                                double *fovx=NULL, double *fovy=NULL,
                                double *focalLength=NULL,
                                CvPoint2D64f *principalPoint=NULL,
                                double *pixelAspectRatio=NULL );
```

calibMatr: The matrix of intrinsic parameters, e.g. computed by cvCalibrateCamera2 imgWidth: Image width in pixels imgHeight: Image height in pixels apertureWidth: Aperture width in realworld units (optional input parameter) apertureHeight: Aperture width in realworld units (optional input parameter) fovx: Field of view angle in x direction in degrees (optional output parameter) fovx: Field of view angle in y direction in degrees (optional output parameter) focalLength: Focal length in realworld units (optional output parameter) principalPoint: The principal point in realworld units (optional output parameter) pixelAspectRatio: The pixel aspect ratio ~ fy/fx (optional output parameter)

The function `cvCalibrationMatrixValues` computes various useful camera (sensor/lens)
characteristics using the computed camera calibration matrix, image frame resolution in pixels
and the physical aperture size.

---

### FindExtrinsicCameraParams2

Finds extrinsic camera parameters for particular view

```
void cvFindExtrinsicCameraParams2( const CvMat* object_points,
                                   const CvMat* image_points,
                                   const CvMat* intrinsic_matrix,
                                   const CvMat* distortion_coeffs,
                                   CvMat* rotation_vector,
                                   CvMat* translation_vector );
```

object\_points: The array of object points, 3xN or Nx3, where N is the number of points in the view. image\_points: The array of corresponding image points, 2xN or Nx2, where N is the number of points in the view. intrinsic\_matrix: The camera matrix (A) [fx 0 cx; 0 fy cy; 0 0 1].: distortion\_coeffs: The vector of distortion coefficients, 4x1, 1x4, 5x1 or 1x5. If it is NULL, the function assumes that all the distortion coefficients are 0's. rotation\_vector: The output 3x1 or 1x3 rotation vector (compact representation of a rotation matrix, see cvRodrigues2). translation\_vector: The output 3x1 or 1x3 translation vector.

The function `cvFindExtrinsicCameraParams2` estimates the object pose
using the intrinsic camera parameters and a few (>=4) 2D<->3D point correspondences.

---

### StereoCalibrate

Calibrates stereo camera

```
void cvStereoCalibrate( const CvMat* object_points, const CvMat* image_points1,
                        const CvMat* image_points2, const CvMat* point_counts,
                        CvMat* camera_matrix1, CvMat* dist_coeffs1,
                        CvMat* camera_matrix2, CvMat* dist_coeffs2,
                        CvSize image_size, CvMat* R, CvMat* T,
                        CvMat* E=0, CvMat* F=0,
                        CvTermCriteria term_crit=cvTermCriteria(
                               CV_TERMCRIT_ITER+CV_TERMCRIT_EPS,30,1e-6),
                        int flags=CV_CALIB_FIX_INTRINSIC );
```

object\_points: The joint matrix of object points, 3xN or Nx3, where N is the total number of points in all views. image\_points1: The joint matrix of corresponding image points in the views from the 1st camera, 2xN or Nx2, where N is the total number of points in all views. image\_points2: The joint matrix of corresponding image points in the views from the 2nd camera, 2xN or Nx2, where N is the total number of points in all views. point\_counts: Vector containing numbers of points in each view, 1xM or Mx1, where M is the number of views. camera\_matrix1, camera\_matrix2: The input/output camera matrices [fx_k_ 0 cx_k_; 0 fy_k_ cy_k_; 0 0 1]. If `CV_CALIB_USE_INTRINSIC_GUESS` or `CV_CALIB_FIX_ASPECT_RATIO` are specified, some or all of the elements of the matrices must be initialized. dist\_coeffs1, dist\_coeffs2: The input/output vectors of distortion coefficients for each camera, 4x1, 1x4, 5x1 or 1x5. image\_size: Size of the image, used only to initialize intrinsic camera matrix. R: The rotation matrix between the 1st and the 2nd cameras' coordinate systems T: The translation vector between the cameras' coordinate systems. E: The optional output essential matrix F: The optional output fundamental matrix term\_crit: Termination criteria for the iterative optimiziation algorithm. flags: Different flags, may be 0 or combination of the following values: `CV_CALIB_FIX_INTRINSIC` - If it is set, `camera_matrix1,2`, as well as `dist_coeffs1,2` are fixed, so that only extrinsic parameters are optimized. `CV_CALIB_USE_INTRINSIC_GUESS` - The flag allows the function to optimize some or all of the intrinsic parameters, depending on the other flags, but the initial values are provided by the user `CV_CALIB_FIX_PRINCIPAL_POINT` - The principal points are fixed during the optimization. `CV_CALIB_FIX_FOCAL_LENGTH` - fx_k_ and fy_k_ are fixed `CV_CALIB_FIX_ASPECT_RATIO` - fy_k_ is optimized, but the ratio fx_k_/fy_k_ is fixed. `CV_CALIB_SAME_FOCAL_LENGTH` - Enforces fx_0_=fx_1_ and fy_0_=fy_1_. `CV_CALIB_ZERO_TANGENT_DIST` - Tangential distortion coefficients for each camera are set to zeros and fixed there. `CV_CALIB_FIX_K1` - The 0-th distortion coefficients (k1) are fixed `CV_CALIB_FIX_K2` - The 1-st distortion coefficients (k2) are fixed `CV_CALIB_FIX_K3` - The 4-th distortion coefficients (k3) are fixed

The function `cvStereoCalibrate` estimates transformation between the 2 cameras making a stereo pair. If we have
a stereo camera, where the relative position and orientatation of the 2 cameras is fixed, and if we computed poses of an object relative to
the fist camera and to the second camera, (R_1_, T_1_) and (R_2_, T_2_), respectively (that can be done with
cvFindExtrinsicCameraParams2), obviously, those poses will relate to each other, i.e. given (R_1_, T_1_)
it should be possible to compute (R_2_, T_2_) - we only need to know the position and orientation of the 2nd camera
relative to the 1st camera. That's what the described function does. It computes (R, T) such that:

```
R2=R*R1
T2=R*T1 + T,
```

Optionally, it computes the essential matrix E:

```
    [0 -T2 T1]
E = [T2 0 -T0]*R,
    [-T1 T0 0]
```

where T_i_ are components of the translation vector T:
T=[T_0_, T_1_, T_2_]^T^.
And also the function can compute the fundamental matrix F:

```
F = inv(camera_matrix2)T*E*inv(camera_matrix1),
```

Besides the stereo-related information, the function can also perform full calibration of each of the 2 cameras. However,
because of the high dimensionality of the parameter space and noise in the input data the function can diverge from the correct solution.
Thus, if intrinsic parameters can be estimated with high accuracy for each of the cameras individually
(e.g. using cvCalibrateCamera2), it is recommended to do so and then pass
`CV_CALIB_FIX_INTRINSIC` flag to the function along with the computed intrinsic parameters. Otherwise, if all the parameters
are estimated at once, it makes sense to restrict some parameters, e.g. pass `CV_CALIB_SAME_FOCAL_LENGTH` and
`CV_CALIB_ZERO_TANGENT_DIST` flags, which are usually reasonable assumptions.

---

### StereoRectify

Computes rectification transform for stereo camera

```
void cvStereoRectify( const CvMat* camera_matrix1, const CvMat* camera_matrix2,
                      const CvMat* dist_coeffs1, const CvMat* dist_coeffs2,
                      CvSize image_size, const CvMat* R, const CvMat* T,
                      CvMat* R1, CvMat* R2, CvMat* P1, CvMat* P2,
                      CvMat* Q=0, int flags=CV_CALIB_ZERO_DISPARITY );
```

camera\_matrix1, camera\_matrix2: The camera matrices [fx_k_ 0 cx_k_; 0 fy_k_ cy_k_; 0 0 1]. dist\_coeffs1, dist\_coeffs2: The vectors of distortion coefficients for each camera, 4x1, 1x4, 5x1 or 1x5. image\_size: Size of the image used for stereo calibration. R: The rotation matrix between the 1st and the 2nd cameras' coordinate systems T: The translation vector between the cameras' coordinate systems. R1, R2: 3x3 Rectification transforms (rotation matrices) for the first and the second cameras, respectively P1, P2: 3x4 Projection matrices in the new (rectified) coordinate systems Q: The optional output disparity-to-depth mapping matrix, 4x4, see cvReprojectImageTo3D. flags: The operation flags; may be 0 or `CV_CALIB_ZERO_DISPARITY`. If the flag is set, the function makes the principal points of each camera have the same pixel coordinates in the rectified views. And if the flag is not set, the function can shift one of the image in horizontal or vertical direction (depending on the orientation of epipolar lines) in order to maximise the useful image area.

The function `cvStereoRectify` computes the rotation matrices for each camera that (virtually) make both
camera image planes the same plane. Consequently, that makes all the epipolar lines parallel and thus simplifies
the dense stereo correspondence problem. On input the function takes the matrices computed by cvStereoCalibrate
and on output it gives 2 rotation matrices and also 2 projection matrices in the new coordinates.
The function is normally called after cvStereoCalibrate that computes
both camera matrices, the distortion coefficients, `R` and `T`.
The 2 cases are distinguished by the function:

1. horizontal stereo, when 1st and 2nd camera views are shifted relative to each other mainly along the x axis (with possible small vertical shift).
   Then in the rectified images the corresponding epipolar lines in left and right cameras will be horizontal and have the same y-coordinate.
   P1 and P2 will look as:

   ```
        [f 0 cx1 0]
     P1=[0 f cy  0]
        [0 0  1  0]

        [f 0 cx2 Tx*f]
     P2=[0 f cy   0 ],
        [0 0  1   0 ]
   ```

   where Tx is horizontal shift between the cameras and cx1=cx2 if `CV_CALIB_ZERO_DISPARITY` is set.- vertical stereo, when 1st and 2nd camera views are shifted relative to each other mainly in vertical direction
     (and probably a bit in the horizontal direction too). Then the epipolar lines in the rectified images will be vertical and have the same x coordinate.
     P2 and P2 will look as:

     ```
          [f 0 cx  0]
       P1=[0 f cy1 0]
          [0 0  1  0]
       
          [f 0 cx   0  ]
       P2=[0 f cy2 Ty*f],
          [0 0  1   0  ]
     ```

     where Ty is vertical shift between the cameras and cy1=cy2 if `CV_CALIB_ZERO_DISPARITY` is set.

As you can see, the first 3 columns of P1 and P2 will effectively be the new "rectified" camera matrices.

---

### StereoRectifyUncalibrated

Computes rectification transform for uncalibrated stereo camera

```
void cvStereoRectifyUncalibrated( const CvMat* points1, const CvMat* points2,
                                  const CvMat* F, CvSize image_size,
                                  CvMat* H1, CvMat* H2,
                                  double threshold=5 );
```

points1, points2: The 2 arrays of corresponding 2D points. F: Fundamental matrix. It can be computed using the same set of point pairs `points1` and `points2` using cvFindFundamentalMat image\_size: Size of the image. H1, H2: The rectification homography matrices for the first and for the second images. threshold: Optional threshold used to filter out the outliers. If the parameter is greater than zero, then all the point pairs that do not comply the epipolar geometry well enough (that is, the points for which `fabs(points2[i]T*F*points1[i])>threshold`) are rejected prior to computing the homographies.

The function `cvStereoRectifyUncalibrated` computes the rectification transformations
without knowing intrinsic parameters of the cameras and their relative position in space,
hence the suffix "Uncalibrated". Another related difference from cvStereoRectify
is that the function outputs not the rectification transformations in the object (3D) space, but
the planar perspective transformations, encoded by the homography matrices `H1` and `H2`.
The function implements the following algorithm [Hartley99].

Note that while the algorithm does not need to know the intrinsic parameters of the cameras,
it heavily depends on the epipolar geometry. Therefore, if the camera lenses have significant
distortion, it would better be corrected before computing the fundamental matrix
and calling this function.
For example, distortion coefficients can be estimated for each head of stereo camera separately by using
cvCalibrateCamera2 and then the images can be corrected using
cvUndistort2.

---

### Rodrigues2

Converts rotation matrix to rotation vector or vice versa

```
int  cvRodrigues2( const CvMat* src, CvMat* dst, CvMat* jacobian=0 );
```

src: The input rotation vector (3x1 or 1x3) or rotation matrix (3x3). dst: The output rotation matrix (3x3) or rotation vector (3x1 or 1x3), respectively. jacobian: Optional output Jacobian matrix, 3x9 or 9x3 - partial derivatives of the output array components w.r.t the input array components.

The function `cvRodrigues2` converts a rotation vector to rotation matrix or
vice versa. Rotation vector is a compact representation of rotation matrix.
Direction of the rotation vector is the rotation axis and the length of the vector is the rotation
angle around the axis.
The rotation matrix `R`, corresponding to the rotation vector `r`,
is computed as following:

```
theta <- norm(r)
r <- r/theta
                                                   [0 -rz ry]
R = cos(theta)*I + (1-cos(theta))*rrT + sin(theta)*[rz 0 -rx]
                                                   [ry rx 0]
```

Inverse transformation can also be done easily as

```
[0 -rz ry]
sin(theta)*[rz 0 -rx] = (R - RT)/2
[ry rx 0]
```

Rotation vector is a convenient representation of a rotation matrix as a matrix with only 3 degrees of freedom.
The representation is used in the global optimization procedures inside
cvFindExtrinsicCameraParams2 and
cvCalibrateCamera2.

---

### Undistort2

Transforms image to compensate lens distortion

```
void cvUndistort2( const CvArr* src, CvArr* dst,
                   const CvMat* intrinsic_matrix,
                   const CvMat* distortion_coeffs );
```

src: The input (distorted) image. dst: The output (corrected) image. intrinsic\_matrix: The camera matrix (A) [fx 0 cx; 0 fy cy; 0 0 1].: distortion\_coeffs: The vector of distortion coefficients, 4x1, 1x4, 5x1 or 1x5.

The function `cvUndistort2` transforms the image to compensate radial and tangential lens distortion.
The camera matrix and distortion parameters can be determined using
cvCalibrateCamera2.
For every pixel in the output image the function computes coordinates of the corresponding location in the input
image using the formulae in the section beginning. Then, the pixel value is computed using bilinear interpolation.
If the resolution of images is different from what was used at the calibration stage,
`fx, fy, cx` and `cy` need to be adjusted appropriately, while
the distortion coefficients remain the same.

In the undistorted image the principal point will be at the image center.

---

### InitUndistortMap

Computes undistortion map

```
void cvInitUndistortMap( const CvMat* camera_matrix,
                         const CvMat* distortion_coeffs,
                         CvArr* mapx, CvArr* mapy );
```

camera\_matrix: The camera matrix (A) [fx 0 cx; 0 fy cy; 0 0 1].: distortion\_coeffs: The vector of distortion coefficients, 4x1, 1x4, 5x1 or 1x5. mapx: The output array of x-coordinates of the map. mapy: The output array of y-coordinates of the map.

The function `cvInitUndistortMap` pre-computes the undistortion map
- coordinates of the corresponding pixel in the distorted image for every pixel in the corrected image.
Then, the map (together with input and output images) can be passed
to cvRemap function.

In the undistorted image the principal point
will be at the image center.

---

### InitUndistortRectifyMap

Computes undistortion+rectification transformation map a head of stereo camera

```
void cvInitUndistortRectifyMap( const CvMat* camera_matrix,
                                const CvMat* dist_coeffs,
                                const CvMat* R,
                                const CvMat* new_camera_matrix,
                                CvArr* mapx, CvArr* mapy );
```

camera\_matrix: The camera matrix A=[fx 0 cx; 0 fy cy; 0 0 1]. distortion\_coeffs: The vector of distortion coefficients, 4x1, 1x4, 5x1 or 1x5. R: The rectification transformation in object space (3x3 matrix). `R1` or `R2`, computed by cvStereoRectify can be passed here. If the parameter is NULL, the identity matrix is used. new\_camera\_matrix: The new camera matrix A'=[fx' 0 cx'; 0 fy' cy'; 0 0 1]. mapx: The output array of x-coordinates of the map. mapy: The output array of y-coordinates of the map.

The function `cvInitUndistortRectifyMap` is an extended version of
cvInitUndistortMap. That is, in addition to the correction of lens distortion,
the function can also apply arbitrary perspective transformation R and finally it
can scale and shift the image according to the new camera matrix. That is, in pseudo code the
transformation can be represented as:

```
// (u,v) is the input point,
// camera_matrix=[fx 0 cx; 0 fy cy; 0 0 1]
// new_camera_matrix=[fx' 0 cx'; 0 fy' cy'; 0 0 1]
x = (u - cx')/fx'
y = (v - cy')/fy'
[X,Y,W]T = R-1*[x y 1]T
x' = X/W, y' = Y/W
x" = x'*(1 + k1r2 + k2r4 + k3r6) + 2*p1x'*y' + p2(r2+2*x'2)
y" = y'*(1 + k1r2 + k2r4 + k3r6) + p1(r2+2*y'2) + 2*p2*x'*y'
mapx(u,v) = x"*fx + cx
mapy(u,v) = y"*fy + cy
```

Note that the code above does the reverse transformation from the target image (i.e. the ideal one,
after undistortion and rectification) to the original "raw" image straight from the camera. That's for bilinear
interpolation purposes and in order to fill the whole destination image w/o gaps using
cvRemap.

Normally, this function is called [twice, once for each head of stereo camera] after cvStereoRectify.
But it is also possible to compute the rectification transformations directly from the fundamental matrix, e.g.
by using cvStereoRectifyUncalibrated. Such functions
work with pixels and produce homographies as rectification transformations, not rotation matrices
`R` in 3D space. In this case, the `R` can be computed from the homography matrix
`H` as

```
R = inv(camera_matrix)*H*camera_matrix
```

---

### UndistortPoints

Computes the ideal point coordinates from the observed point coordinates

```
void cvUndistortPoints( const CvMat* src, CvMat* dst,
                        const CvMat* camera_matrix,
                        const CvMat* dist_coeffs,
                        const CvMat* R=NULL,
                        const CvMat* P=NULL);
```

src: The observed point coordinates. dst The ideal point coordinates, after undistortion and reverse perspective transformation. camera\_matrix: The camera matrix A=[fx 0 cx; 0 fy cy; 0 0 1]. distortion\_coeffs: The vector of distortion coefficients, 4x1, 1x4, 5x1 or 1x5. R: The rectification transformation in object space (3x3 matrix). `R1` or `R2`, computed by cvStereoRectify can be passed here. If the parameter is NULL, the identity matrix is used. P: The new camera matrix (3x3) or the new projection matrix (3x4). `P1` or `P2`, computed by cvStereoRectify can be passed here. If the parameter is NULL, the identity matrix is used.

The function `cvUndistortPoints` is similar to
cvInitUndistortRectifyMap and is opposite to it at the same time.
The functions are similar in that they both are used to correct lens distortion and to perform
the optional perspective (rectification) transformation. They are opposite because the
function cvInitUndistortRectifyMap does actually perform
the reverse transformation in order to initialize the maps properly, while this function
does the forward transformation. That is, in pseudo-code it can be expressed as:

```
// (u,v) is the input point, (u', v') is the output point
// camera_matrix=[fx 0 cx; 0 fy cy; 0 0 1]
// P=[fx' 0 cx' tx; 0 fy' cy' ty; 0 0 1 tz]
x" = (u - cx)/fx
y" = (v - cy)/fy
(x',y') = undistort(x",y",dist_coeffs)
[X,Y,W]T = R*[x' y' 1]T
x = X/W, y = Y/W
u' = x*fx' + cx'
v' = y*fy' + cy',
```

where undistort() is approximate iterative algorithm that estimates the normalized original point coordinates
out of the normalized distorted point coordinates
("normalized" means that the coordinates do not depend on the camera matrix).

The function can be used as for stereo cameras, as well as for individual cameras when R=NULL.

---

### FindChessboardCorners

Finds positions of internal corners of the chessboard

```
int cvFindChessboardCorners( const void* image, CvSize pattern_size,
                             CvPoint2D32f* corners, int* corner_count=NULL,
                             int flags=CV_CALIB_CB_ADAPTIVE_THRESH );
```

image: Source chessboard view; it must be 8-bit grayscale or color image. pattern\_size: The number of inner corners per chessboard row and column. corners: The output array of corners detected. corner\_count: The output corner counter. If it is not NULL, the function stores there the number of corners found. flags: Various operation flags, can be 0 or a combination of the following values: `CV_CALIB_CB_ADAPTIVE_THRESH` - use adaptive thresholding to convert the image to black-n-white, rather than a fixed threshold level (computed from the average image brightness). `CV_CALIB_CB_NORMALIZE_IMAGE` - normalize the image using cvNormalizeHist before applying fixed or adaptive thresholding. `CV_CALIB_CB_FILTER_QUADS` - use additional criteria (like contour area, perimeter, square-like shape) to filter out false quads that are extracted at the contour retrieval stage.

The function `cvFindChessboardCorners` attempts to determine whether the input
image is a view of the chessboard pattern and locate internal chessboard
corners. The function returns non-zero value if all the corners have been found
and they have been placed in a certain order (row by row, left to right in every
row), otherwise, if the function fails to find all the corners or reorder them,
it returns 0. For example, a regular chessboard has 8 x 8 squares and 7
x 7 internal corners, that is, points, where the black squares touch each other.
The coordinates detected are approximate, and to determine their position more accurately,
the user may use the function cvFindCornerSubPix.

---

### DrawChessBoardCorners

Renders the detected chessboard corners

```
void cvDrawChessboardCorners( CvArr* image, CvSize pattern_size,
                              CvPoint2D32f* corners, int count,
                              int pattern_was_found );
```

image: The destination image; it must be 8-bit color image. pattern\_size: The number of inner corners per chessboard row and column. corners: The array of corners detected. count: The number of corners. pattern\_was\_found: Indicates whether the complete board was found (≠0) or not (=0). One may just pass the return value cvFindChessboardCorners here.

The function `cvDrawChessboardCorners` draws the individual chessboard corners detected (as red circles)
in case if the board was not found (`pattern_was_found`=0) or the colored corners connected with lines
when the board was found (`pattern_was_found`≠0).

---

## Pose Estimation

---

### CreatePOSITObject

Initializes structure containing object information

```
CvPOSITObject* cvCreatePOSITObject( CvPoint3D32f* points, int point_count );
```

points: Pointer to the points of the 3D object model. point\_count: Number of object points.

The function `cvCreatePOSITObject` allocates memory for the object structure and
computes the object inverse matrix.

The pre-processed object data is stored in the structure CvPOSITObject, internal
for OpenCV, which means that the user cannot directly access the structure data.
The user may only create this structure and pass its pointer to the function.

Object is defined as a set of points given in a coordinate system. The function
cvPOSIT computes a vector that begins at a camera-related coordinate system center
and ends at the `points[0]` of the object.

Once the work with a given object is finished, the function
cvReleasePOSITObject
must be called to free memory.

---

### POSIT

Implements POSIT algorithm

```
void cvPOSIT( CvPOSITObject* posit_object, CvPoint2D32f* image_points, double focal_length,
              CvTermCriteria criteria, CvMatr32f rotation_matrix, CvVect32f translation_vector );
```

posit\_object: Pointer to the object structure. image\_points: Pointer to the object points projections on the 2D image plane. focal\_length: Focal length of the camera used. criteria: Termination criteria of the iterative POSIT algorithm. rotation\_matrix: Matrix of rotations. translation\_vector: Translation vector.

The function `cvPOSIT` implements POSIT algorithm. Image coordinates are given in a
camera-related coordinate system. The focal length may be retrieved using camera
calibration functions. At every iteration of the algorithm new perspective
projection of estimated pose is computed.

Difference norm between two projections is the maximal distance between
corresponding points. The parameter `criteria.epsilon` serves to stop the
algorithm if the difference is small.

---

### ReleasePOSITObject

Deallocates 3D object structure

```
void cvReleasePOSITObject( CvPOSITObject** posit_object );
```

posit\_object: Double pointer to `CvPOSIT` structure.

The function `cvReleasePOSITObject` releases memory previously allocated by the
function cvCreatePOSITObject.

---

### CalcImageHomography

Calculates homography matrix for oblong planar object (e.g. arm)

```
void cvCalcImageHomography( float* line, CvPoint3D32f* center,
                            float* intrinsic, float* homography );
```

line: the main object axis direction (vector (dx,dy,dz)). center: object center ((cx,cy,cz)). intrinsic: intrinsic camera parameters (3x3 matrix). homography: output homography matrix (3x3).

The function `cvCalcImageHomography` calculates the homography matrix for the initial
image transformation from image plane to the plane, defined by 3D oblong object line (See
Figure 6-10 in OpenCV Guide 3D Reconstruction Chapter).


---

## Epipolar Geometry, Stereo Correspondence

---

### FindFundamentalMat

Calculates fundamental matrix from corresponding points in two images

```
int cvFindFundamentalMat( const CvMat* points1,
                          const CvMat* points2,
                          CvMat* fundamental_matrix,
                          int    method=CV_FM_RANSAC,
                          double param1=3.,
                          double param2=0.99,
                          CvMat* status=NULL);
```

points1: Array of the first image points of `2xN, Nx2, 3xN` or `Nx3` size (where `N` is number of points). Multi-channel `1xN` or `Nx1` array is also acceptable. The point coordinates should be floating-point (single or double precision) points2: Array of the second image points of the same size and format as `points1` fundamental\_matrix: The output fundamental matrix or matrices. The size should be 3x3 or 9x3 (7-point method may return up to 3 matrices). method: Method for computing the fundamental matrix: CV\_FM\_7POINT - for 7-point algorithm. N == 7: CV\_FM\_8POINT - for 8-point algorithm. N >= 8: CV\_FM\_RANSAC - for RANSAC algorithm. N > 8: CV\_FM\_LMEDS - for LMedS algorithm. N > 8 param1: The parameter is used for RANSAC method only. It is the maximum distance from point to epipolar line in pixels, beyond which the point is considered an outlier and is not used for computing the final fundamental matrix. Usually it is set somewhere from 1 to 3. param2: The parameter is used for RANSAC or LMedS methods only. It denotes the desirable level of confidence of the fundamental matrix estimate. status: The optional output array of N elements, every element of which is set to 0 for outliers and to 1 for the "inliers", i.e. points that comply well with the estimated epipolar geometry. The array is computed only in RANSAC and LMedS methods. For other methods it is set to all 1’s.

The epipolar geometry is described by the following equation:

```
p2T*F*p1=0,
```

where `F` is fundamental matrix, `p1` and `p2` are corresponding
points in the first and the second images, respectively.

The function `cvFindFundamentalMat` calculates fundamental matrix using one of four
methods listed above and returns the number of fundamental matrices found (1 or 3) and 0,
if no matrix is found.

The calculated fundamental matrix may be passed further to `cvComputeCorrespondEpilines`
that finds epipolar lines corresponding to the specified points.

#### Example. Estimation of fundamental matrix using RANSAC algorithm

```
int point_count = 100;
CvMat* points1;
CvMat* points2;
CvMat* status;
CvMat* fundamental_matrix;

points1 = cvCreateMat(1,point_count,CV_32FC2);
points2 = cvCreateMat(1,point_count,CV_32FC2);
status = cvCreateMat(1,point_count,CV_8UC1);

/* Fill the points here ... */
for( i = 0; i < point_count; i++ )
{
    points1->data.db[i*2] = <x1,i>;
    points1->data.db[i*2+1] = <y1,i>;
    points2->data.db[i*2] = <x2,i>;
    points2->data.db[i*2+1] = <y2,i>;
}

fundamental_matrix = cvCreateMat(3,3,CV_32FC1);
int fm_count = cvFindFundamentalMat( points1,points2,fundamental_matrix,
                                     CV_FM_RANSAC,3,0.99,status );
```

---

### ComputeCorrespondEpilines

For points in one image of stereo pair computes the corresponding epilines in the other image

```
void cvComputeCorrespondEpilines( const CvMat* points,
                                  int which_image,
                                  const CvMat* fundamental_matrix,
                                  CvMat* correspondent_lines);
```

points: The input points. `2xN, Nx2, 3xN` or `Nx3` array (where `N` number of points). Multi-channel `1xN` or `Nx1` array is also acceptable. which\_image: Index of the image (1 or 2) that contains the `points` fundamental\_matrix: Fundamental matrix correspondent\_lines: Computed epilines, `3xN` or `Nx3` array

For every point in one of the two images of stereo-pair the function
`cvComputeCorrespondEpilines` finds equation of a line that contains
the corresponding point (i.e. projection of the same 3D point) in the other image.
Each line is encoded by a vector of 3 elements `l=[a,b,c]T`, so that:

```
lT*[x, y, 1]T=0, or
a*x + b*y + c = 0
```

From the fundamental matrix definition (see cvFindFundamentalMatrix
discussion), line `l2` for a point `p1`
in the first image (`which_image`=1) can be computed as:

```
l2=F*p1
```

and the line `l1` for a point `p2`
in the second image (`which_image`=1) can be computed as:

```
l1=FT*p2
```

Line coefficients are defined up to a scale.
They are normalized (`a2+b2=1`)
are stored into `correspondent_lines`.

---

### ConvertPointsHomogeneous

Convert points to/from homogeneous coordinates

```
void cvConvertPointsHomogeneous( const CvMat* src, CvMat* dst );
```

src: The input point array, `2xN, Nx2, 3xN, Nx3, 4xN or Nx4` (where `N` is the number of points). Multi-channel `1xN` or `Nx1` array is also acceptable. dst: The output point array, must contain the same number of points as the input; The dimensionality must be the same, 1 less or 1 more than the input, and also within 2..4.

The function `cvConvertPointsHomogeneous` converts 2D or 3D points
from/to homogeneous coordinates, or simply copies or transposes the array.
In case if the input array dimensionality is larger than the output,
each point coordinates are divided by the last coordinate:

```
(x,y[,z],w) -> (x',y'[,z']):

x' = x/w
y' = y/w
z' = z/w (if output is 3D)
```

If the output array dimensionality is larger, an extra 1 is appended to each point.

```
(x,y[,z]) -> (x,y[,z],1)
```

Otherwise, the input array is simply copied (with optional transposition) to the output.
**Note** that, because the function accepts a large variety of array layouts, it
may report an error when input/output array dimensionality is ambiguous.
It is always safe to use the function with number of points `N`>=5, or
to use multi-channel `Nx1` or `1xN` arrays.

---

### CvStereoBMState

The structure for block matching stereo correspondence algorithm

```
typedef struct CvStereoBMState
{
    //pre filters (normalize input images):
    int       preFilterType; // 0 for now
    int       preFilterSize; // ~5x5..21x21
    int       preFilterCap;  // up to ~31
    //correspondence using Sum of Absolute Difference (SAD):
    int       SADWindowSize; // Could be 5x5..21x21
    int       minDisparity;  // minimum disparity (=0)
    int       numberOfDisparities; // maximum disparity - minimum disparity
    //post filters (knock out bad matches):
    int       textureThreshold; // areas with no texture are ignored
    float     uniquenessRatio;// filter out pixels if there are other close matches
                              // with different disparity
    int       speckleWindowSize;// Disparity variation window (not used)
    int       speckleRange; // Acceptable range of variation in window (not used)
    // internal buffers, do not modify (!)
    CvMat* preFilteredImg0;
    CvMat* preFilteredImg1;
    CvMat* slidingSumBuf;
}
CvStereoBMState;
```

The block matching stereo correspondence algorithm, by Kurt Konolige, is very fast
one-pass stereo matching algorithm that uses sliding sums of absolute differences
between pixels in the left image and the pixels in the right image,
shifted by some varying amount of pixels (from `minDisparity` to
`minDisparity+numberOfDisparities`). On a pair of images WxH the algorithm
computes disparity in `O(W*H*numberOfDisparities)` time. In order to improve
quality and reability of the disparity map, the algorithm includes pre-filtering and
post-filtering procedures.

Note that the algorithm searches for the corresponding blocks in x direction only.
It means that the supplied stereo pair should be rectified. Vertical stereo layout is not directly
supported, but in such a case the images could be transposed by user.

---

### CreateStereoBMState

Creates block matching stereo correspondence structure

```
#define CV_STEREO_BM_BASIC 0
#define CV_STEREO_BM_FISH_EYE 1
#define CV_STEREO_BM_NARROW 2

CvStereoBMState* cvCreateStereoBMState( int preset=CV_STEREO_BM_BASIC,
                                        int numberOfDisparities=0 );
```

preset: ID of one of the pre-defined parameter sets. Any of the parameters can be overridden after creating the structure. numberOfDisparities: The number of disparities. If the parameter is 0, it is taken from the preset, otherwise the supplied value overrides the one from preset.

The function cvCreateStereoBMState creates the stereo correspondence structure and initializes it.
It is possible to override any of the parameters at any time between the calls to
cvFindStereoCorrespondenceBM.

---

### ReleaseStereoBMState

Releases block matching stereo correspondence structure

```
void cvReleaseStereoBMState( CvStereoBMState** state );
```

state: Double pointer to the released structure

The function cvReleaseStereoBMState releases the stereo correspondence structure and
all the associated internal buffers.

---

### FindStereoCorrespondenceBM

Computes the disparity map using block matching algorithm

```
void cvFindStereoCorrespondenceBM( const CvArr* left, const CvArr* right,
                                   CvArr* disparity, CvStereoBMState* state );
```

left: The left single-channel, 8-bit image. right: The right image of the same size and the same type. disparity: The output single-channel 16-bit signed disparity map of the same size as input images. Its elements will be the computed disparities, multiplied by 16 and rounded to integer's. state: Stereo correspondence structure.

The function cvFindStereoCorrespondenceBM computes disparity map for
the input rectified stereo pair.

---

### CvStereoGCState

The structure for graph cuts-based stereo correspondence algorithm

```
typedef struct CvStereoGCState
{
    int Ithreshold; // threshold for piece-wise linear data cost function (5 by default)
    int interactionRadius; // radius for smoothness cost function (1 by default; means Potts model)
    float K, lambda, lambda1, lambda2; // parameters for the cost function
                                       // (usually computed adaptively from the input data)
    int occlusionCost; // 10000 by default
    int minDisparity; // 0 by default; see CvStereoBMState
    int numberOfDisparities; // defined by user; see CvStereoBMState
    int maxIters; // number of iterations; defined by user.

    // internal buffers
    CvMat* left;
    CvMat* right;
    CvMat* dispLeft;
    CvMat* dispRight;
    CvMat* ptrLeft;
    CvMat* ptrRight;
    CvMat* vtxBuf;
    CvMat* edgeBuf;
}
CvStereoGCState;
```

The graph cuts stereo correspondence algorithm,
described in [Kolmogorov03] (as **KZ1**), is non-realtime
stereo correpsondence algorithm that usually gives very accurate depth map with well-defined
object boundaries. The algorithm represents stereo problem as a sequence of binary
optimization problems, each of those is solved using maximum graph flow algorithm.
The state structure above should not be allocated and initialized manually; instead, use
cvCreateStereoGCState and then override
necessary parameters if needed.

---

### CreateStereoGCState

Creates the state of graph cut-based stereo correspondence algorithm

```
CvStereoGCState* cvCreateStereoGCState( int numberOfDisparities,
                                        int maxIters );
```

numberOfDisparities: The number of disparities. The disparity search range will be `state->minDisparity ≤ disparity < state->minDisparity + state->numberOfDisparities` maxIters: Maximum number of iterations. On each iteration all possible (or reasonable) alpha-expansions are tried. The algorithm may terminate earlier if it could not find an alpha-expansion that decreases the overall cost function value. See [Kolmogorov03] for details.

The function cvCreateStereoGCState creates the stereo correspondence structure and initializes it.
It is possible to override any of the parameters at any time between the calls to
cvFindStereoCorrespondenceGC.

---

### ReleaseStereoGCState

Releases the state structure of the graph cut-based stereo correspondence algorithm

```
void cvReleaseStereoGCState( CvStereoGCState** state );
```

state: Double pointer to the released structure

The function cvReleaseStereoGCState releases the stereo correspondence structure and
all the associated internal buffers.

---

### FindStereoCorrespondenceGC

Computes the disparity map using graph cut-based algorithm

```
void cvFindStereoCorrespondenceGC( const CvArr* left, const CvArr* right,
                                   CvArr* dispLeft, CvArr* dispRight,
                                   CvStereoGCState* state,
                                   int useDisparityGuess CV_DEFAULT(0) );
```

left: The left single-channel, 8-bit image. right: The right image of the same size and the same type. dispLeft: The optional output single-channel 16-bit signed left disparity map of the same size as input images. dispRight: The optional output single-channel 16-bit signed right disparity map of the same size as input images. state: Stereo correspondence structure. useDisparityGuess: If the parameter is not zero, the algorithm will start with pre-defined disparity maps. Both dispLeft and dispRight should be valid disparity maps. Otherwise, the function starts with blank disparity maps (all pixels are marked as occlusions).

The function cvFindStereoCorrespondenceGC computes disparity maps for
the input rectified stereo pair. Note that the left disparity image will contain values in the following range:

```
-state->numberOfDisparities-state->minDisparity < dispLeft(x,y) ≤ -state->minDisparity,
or
dispLeft(x,y) == CV_STEREO_GC_OCCLUSION,
```

where as for the right disparity image the following will be true:

```
state->minDisparity ≤ dispRight(x,y) < state->minDisparity+state->numberOfDisparities,
or
dispRight(x,y) == CV_STEREO_GC_OCCLUSION,
```

that is, the range for the left disparity image will be inversed, and the pixels for
which no good match has been found, will be marked as occlusions.

Here is how the function can be called:

```
// image_left and image_right are the input 8-bit single-channel images
// from the left and the right cameras, respectively
CvSize size = cvGetSize(image_left);
CvMat* disparity_left = cvCreateMat( size.height, size.width, CV_16S );
CvMat* disparity_right = cvCreateMat( size.height, size.width, CV_16S );
CvStereoGCState* state = cvCreateStereoGCState( 16, 2 );
cvFindStereoCorrespondenceGC( image_left, image_right,
    disparity_left, disparity_right, state, 0 );
cvReleaseStereoGCState( &state );
// now process the computed disparity images as you want ...
```

and this is the output left disparity image computed from the well-known
Tsukuba stereo pair and multiplied by -16 (because the values in the left
disparity images are usually negative):

```
CvMat* disparity_left_visual = cvCreateMat( size.height, size.width, CV_8U );
cvConvertScale( disparity_left, disparity_left_visual, -16 );
cvSave( "disparity.png", disparity_left_visual );
```

---

### ReprojectImageTo3D

Reprojects disparity image to 3D space

```
void cvReprojectImageTo3D( const CvArr* disparity,
                           CvArr* _3dImage, const CvMat* Q );
```

disparity: Disparity map. \_3dImage: 3-channel, 16-bit integer or 32-bit floating-point image - the output map of 3D points. Q: The reprojection 4x4 matrix.

The function cvReprojectImageTo3D transforms 1-channel disparity map to 3-channel image, a 3D surface.
That is, for each pixel `(x,y)` and the corresponding disparity
`d=disparity(x,y)` it computes:

```
[X Y Z W]T = Q*[x y d 1]T
_3dImage(x,y) = (X/W, Y/W, Z/W)
```

The matrix Q can be arbitrary, e.g. the one, computed by
cvStereoRectify.
To reproject a sparse set of points {(x,y,d),...} to 3D space, use
cvPerspectiveTransform.

---

# Alphabetical List of Functions

---

### 2

|  |  |  |
| --- | --- | --- |
| 2DRotationMatrix |  |  |

---

### A

|  |  |  |
| --- | --- | --- |
| Acc | ApproxChains | ArcLength |
| AdaptiveThreshold | ApproxPoly |  |

---

### B

|  |  |  |
| --- | --- | --- |
| BoundingRect | BoxPoints |  |

---

### C

|  |  |  |
| --- | --- | --- |
| CalcBackProject | CalibrationMatrixValues | CopyHist |
| CalcBackProjectPatch | CamShift | CopyMakeBorder |
| CalcEMD2 | Canny | CornerEigenValsAndVecs |
| CalcGlobalOrientation | CheckContourConvexity | CornerHarris |
| CalcHist | ClearHist | CornerMinEigenVal |
| CalcImageHomography | ClearSubdivVoronoi2D | CreateConDensation |
| CalcMotionGradient | CompareHist | CreateContourTree |
| CalcOpticalFlowBM | ComputeCorrespondEpilines | CreateFeatureTree |
| CalcOpticalFlowHS | ConDensInitSampleSet | CreateHist |
| CalcOpticalFlowLK | ConDensUpdateByTime | CreateKalman |
| CalcOpticalFlowPyrLK | ContourArea | CreatePOSITObject |
| CalcPGH | ContourFromContourTree | CreateStateBM |
| CalcProbDensity | ConvertPointsHomogeneous | CreateStructuringElementEx |
| CalcSubdivVoronoi2D | ConvexHull2 | CreateSubdivDelaunay2D |
| CalibrateCamera2 | ConvexityDefects | CvtColor |

---

### D

|  |  |  |
| --- | --- | --- |
| Dilate | DistTransform | DrawChessBoardCorners |

---

### E

|  |  |  |
| --- | --- | --- |
| EndFindContours | EqualizeHist | Erode |

---

### F

|  |  |  |
| --- | --- | --- |
| Filter2D | FindFeatures | FindNextContour |
| FindChessboardCorners | FindFeaturesBoxed | FindStereoCorrespondenceBM |
| FindContours | FindFundamentalMat | FitEllipse |
| FindCornerSubPix | FindHomography | FitLine2D |
| FindExtrinsicCameraParams2 | FindNearestPoint2D | FloodFill |

---

### G

|  |  |  |
| --- | --- | --- |
| GetAffineTransform | GetMinMaxHistValue | GetRectSubPix |
| GetCentralMoment | GetNormalizedCentralMoment | GetSpatialMoment |
| GetHistValue\_\*D | GetPerspectiveTransform | GoodFeaturesToTrack |
| GetHuMoments | GetQuadrangleSubPix |  |

---

### H

|  |  |  |
| --- | --- | --- |
| HaarDetectObjects | HoughCircles | HoughLines2 |

---

### I

|  |  |  |
| --- | --- | --- |
| InitUndistortMap | Inpaint |  |
| InitUndistortRectifyMap | Integral |  |

---

### K

|  |  |  |
| --- | --- | --- |
| KalmanCorrect | KalmanPredict |  |

---

### L

|  |  |  |
| --- | --- | --- |
| Laplace | LoadHaarClassifierCascade | LogPolar |

---

### M

|  |  |  |
| --- | --- | --- |
| MakeHistHeaderForArray | MaxRect | Moments |
| MatchContourTrees | MeanShift | MorphologyEx |
| MatchShapes | MinAreaRect2 | MultiplyAcc |
| MatchTemplate | MinEnclosingCircle |  |

---

### N

|  |  |  |
| --- | --- | --- |
| NormalizeHist |  |  |

---

### P

|  |  |  |
| --- | --- | --- |
| POSIT | PreCornerDetect | PyrMeanShiftFiltering |
| PointPolygonTest | ProjectPoints2 | PyrSegmentation |
| PointSeqFromMat | PyrDown | PyrUp |

---

### Q

|  |  |  |
| --- | --- | --- |
| QueryHistValue\_\*D |  |  |

---

### R

|  |  |  |
| --- | --- | --- |
| ReadChainPoint | ReleaseKalman | ReprojectImageTo3D |
| ReleaseConDensation | ReleasePOSITObject | Resize |
| ReleaseFeatureTree | ReleaseStateBM | Rodrigues2 |
| ReleaseHaarClassifierCascade | ReleaseStructuringElement | RunHaarClassifierCascade |
| ReleaseHist | Remap | RunningAvg |

---

### S

|  |  |  |
| --- | --- | --- |
| SampleLine | SquareAcc | Subdiv2DEdgeOrg |
| SegmentMotion | StartFindContours | Subdiv2DGetEdge |
| SetHistBinRanges | StartReadChainPoints | Subdiv2DLocate |
| SetImagesForHaarClassifierCascade | StereoCalibrate | Subdiv2DRotateEdge |
| Smooth | StereoRectify | SubdivDelaunay2DInsert |
| SnakeImage | StereoRectifyUncalibrated | SubstituteContour |
| Sobel | Subdiv2DEdgeDst |  |

---

### T

|  |  |  |
| --- | --- | --- |
| ThreshHist | Threshold |  |

---

### U

|  |  |  |
| --- | --- | --- |
| Undistort2 | UndistortPoints | UpdateMotionHistory |

---

### W

|  |  |  |
| --- | --- | --- |
| WarpAffine | WarpPerspective | Watershed |

---

# Bibliography

This bibliography provides a list of publications that were might be useful to
the OpenCV users. This list is not complete; it serves only
as a starting point.

1. **[Bay06]**
   Herbert Bay, Tinne Tuytelaars and Luc Van Gool
   "SURF: Speeded Up Robust Features", Proceedings of the 9th European Conference
   on Computer Vision, Springer LNCS volume 3951, part 1, pp 404--417, 2006.
   - **[Beis97]**
     J.S. Beis and D.G. Lowe, "Shape indexing using approximate nearest-neighbor search in highdimensional spaces".
     In Proc. IEEE Conf. Comp. Vision Patt. Recog., pages 1000--1006, 1997.
     - **[Borgefors86]**
       Gunilla Borgefors, "Distance Transformations in Digital Images". Computer Vision, Graphics and Image Processing 34, 344-371 (1986).
       - **[Bouguet00]**
         Jean-Yves Bouguet. Pyramidal Implementation of the Lucas Kanade Feature Tracker.  
         The paper is included into OpenCV distribution (algo\_tracking.pdf)- **[Bouguet04]**
           Jean-Yves Bouguet. The Camera Calibration Toolbox for Matlab.
           http://www.vision.caltech.edu/bouguetj/calib\_doc/- **[Bradski98]**
             G.R. Bradski. Computer vision face tracking as a component of a perceptual
             user interface. In Workshop on Applications of Computer Vision, pages 214�219,
             Princeton, NJ, Oct. 1998.  
             Updated version can be found at
             http://www.intel.com/technology/itj/q21998/articles/art\_2.htm.  
             Also, it is included into OpenCV distribution (camshift.pdf)- **[Bradski00]** G. Bradski and J. Davis. Motion Segmentation and Pose Recognition
               with Motion History Gradients. IEEE WACV'00, 2000.- **[Burt81]** P. J. Burt, T. H. Hong, A. Rosenfeld. Segmentation and Estimation of
                 Image Region Properties Through Cooperative Hierarchical Computation. IEEE Tran.
                 On SMC, Vol. 11, N.12, 1981, pp. 802-809.- **[Canny86]** J. Canny. A Computational Approach to Edge Detection, IEEE Trans. on
                   Pattern Analysis and Machine Intelligence, 8(6), pp. 679-698 (1986).- **[Davis97]** J. Davis and Bobick. The Representation and Recognition of Action
                     Using Temporal Templates. MIT Media Lab Technical Report 402, 1997.- **[DeMenthon92]** Daniel F. DeMenthon and Larry S. Davis. Model-Based Object Pose in
                       25 Lines of Code. In Proceedings of ECCV '92, pp. 335-343, 1992.- **[Felzenszwalb04]**
                         Pedro F. Felzenszwalb and Daniel P. Huttenlocher.
                         Distance Transforms of Sampled Functions.
                         Cornell Computing and Information Science TR2004-1963.- **[Fitzgibbon95]** Andrew W. Fitzgibbon, R.B.Fisher. A Buyer’s Guide to Conic
                           Fitting. Proc.5th British Machine Vision Conference, Birmingham, pp. 513-522,
                           1995.- **[Ford98]** Adrian Ford, Alan Roberts. Colour Space Conversions.
                             http://www.poynton.com/PDFs/coloureq.pdf- **[Hartley99]** Richard I. Hartley.
                               Theory and practice of projective rectification. Int J Computer Vision 35(2): 1�16- **[Horn81]**
                                 Berthold K.P. Horn and Brian G. Schunck. Determining Optical Flow.
                                 Artificial Intelligence, 17, pp. 185-203, 1981.
                                 - **[Hu62]** M. Hu. Visual Pattern Recognition by Moment Invariants, IRE Transactions
                                   on Information Theory, 8:2, pp. 179-187, 1962.- **[Iivarinen97]**
                                     Jukka Iivarinen, Markus Peura, Jaakko Srel, and Ari Visa.
                                     Comparison of Combined Shape Descriptors for Irregular Objects, 8th British Machine Vision Conference, BMVC'97.  

                                     http://www.cis.hut.fi/research/IA/paper/publications/bmvc97/bmvc97.html- **[Jahne97]** B. Jahne. Digital Image Processing. Springer, New York, 1997.- **[Lucas81]**
                                         Lucas, B., and Kanade, T. An Iterative Image
                                         Registration Technique with an Application to Stereo
                                         Vision, Proc. of 7th International Joint Conference on
                                         Artificial Intelligence (IJCAI), pp. 674-679.
                               - **[Kass88]** M. Kass, A. Witkin, and D. Terzopoulos. Snakes: Active Contour Models,
                                 International Journal of Computer Vision, pp. 321-331, 1988.- **[Kolmogorov03]**V. Kolmogorov. Graph Based Algorithms for Scene Reconstruction
                                   from Two or More Views. PhD thesis, Cornell University, September 2003.- **[Lienhart02]**
                                     Rainer Lienhart and Jochen Maydt.
                                     An Extended Set of Haar-like Features for Rapid Object Detection.
                                     IEEE ICIP 2002, Vol. 1, pp. 900-903, Sep. 2002.  
                                     This paper, as well as the extended technical report, can be retrieved at
                                     http://www.lienhart.de/Publications/publications.html- **[Matas98]** J.Matas, C.Galambos, J.Kittler. Progressive Probabilistic Hough
                                       Transform. British Machine Vision Conference, 1998.- **[Meyer92]**
                                         Meyer, F. (1992). Color image segmentation. In Proceedings of the International Conference on Image Processing and its Applications, pages 303--306.- **[Rosenfeld73]** A. Rosenfeld and E. Johnston. Angle Detection on Digital Curves.
                                           IEEE Trans. Computers, 22:875-878, 1973.- **[RubnerJan98]** Y. Rubner. C. Tomasi, L.J. Guibas. Metrics for Distributions with
                                             Applications to Image Databases. Proceedings of the 1998 IEEE International
                                             Conference on Computer Vision, Bombay, India, January 1998, pp. 59-66.- **[RubnerSept98]**
                                               Y. Rubner. C. Tomasi, L.J. Guibas. The Earth Mover’s Distance as a
                                               Metric for Image Retrieval. Technical Report STAN-CS-TN-98-86,
                                               Department of Computer Science, Stanford University, September
                                               1998.
                                               - **[RubnerOct98]** Y. Rubner. C. Tomasi. Texture Metrics. Proceeding of the IEEE
                                                 International Conference on Systems, Man, and Cybernetics, San-Diego, CA,
                                                 October 1998, pp. 4601-4607.
                                                 http://robotics.stanford.edu/~rubner/publications.html- **[Serra82]** J. Serra. Image Analysis and Mathematical Morphology. Academic Press,
                                                   1982.- **[Schiele00]** Bernt Schiele and James L. Crowley. Recognition without
                                                     Correspondence Using Multidimensional Receptive Field Histograms. In
                                                     International Journal of Computer Vision 36 (1), pp. 31-50, January 2000.- **[Suzuki85]** S. Suzuki, K. Abe. Topological Structural Analysis of Digital Binary
                                                       Images by Border Following. CVGIP, v.30, n.1. 1985, pp. 32-46.- **[Teh89]** C.H. Teh, R.T. Chin. On the Detection of Dominant Points on Digital
                                                         Curves. - IEEE Tr. PAMI, 1989, v.11, No.8, p. 859-872.- **[Telea04]**
                                                           A. Telea, "An image inpainting technique based on the fast marching method,"
                                                           J. Graphics Tools, vol.9, no.1, pp.25�36, 2004.  
                                                           - **[Trucco98]** Emanuele Trucco, Alessandro Verri. Introductory Techniques for 3-D
                                                             Computer Vision. Prentice Hall, Inc., 1998.- **[Viola01]**
                                                               Paul Viola and Michael J. Jones.
                                                               Rapid Object Detection using a Boosted Cascade of Simple Features. IEEE CVPR, 2001.  
                                                               The paper is available online at
                                                               http://www.ai.mit.edu/people/viola/- **[Welch95]**
                                                                 Greg Welch, Gary Bishop. An Introduction To the Kalman Filter.
                                                                 Technical Report TR95-041, University of North Carolina at Chapel Hill, 1995.  
                                                                 Online version is available at 
                                                                 http://www.cs.unc.edu/~welch/kalman/kalmanIntro.html- **[Williams92]** D. J. Williams and M. Shah. A Fast Algorithm for Active Contours
                                                                   and Curvature Estimation. CVGIP: Image Understanding, Vol. 55, No. 1, pp. 14-26,
                                                                   Jan., 1992. http://www.cs.ucf.edu/~vision/papers/shah/92/WIS92A.pdf.- **[Yuen03]**
                                                                     H.K. Yuen, J. Princen, J. Illingworth and J. Kittler.
                                                                     Comparative study of Hough Transform methods for circle finding.  

                                                                     http://www.sciencedirect.com/science/article/B6V09-48TCV4N-5Y/2/91f551d124777f7a4cf7b18325235673- **[Yuille89]** A.Y.Yuille, D.S.Cohen, and P.W.Hallinan. Feature Extraction from
                                                                       Faces Using Deformable Templates in CVPR, pp. 104-109, 1989.- **[Zhang96]** Z. Zhang. Parameter Estimation Techniques: A Tutorial with Application
                                                                         to Conic Fitting, Image and Vision Computing Journal, 1996.- **[Zhang99]** Z. Zhang. Flexible Camera Calibration By Viewing a Plane From Unknown
                                                                           Orientations. International Conference on Computer Vision (ICCV'99), Corfu,
                                                                           Greece, pages 666-673, September 1999.- **[Zhang00]** Z. Zhang. A Flexible New Technique for Camera Calibration. IEEE
                                                                             Transactions on Pattern Analysis and Machine Intelligence, 22(11):1330-1334,
                                                                             2000.
