# Supplementary material for: Rapid whole brain imaging of neural activity in freely behaving larval zebrafish (Danio rerio)
Source: eLife. 2017 Sep 20;6:e28158. doi: 10.7554/eLife.28158 (PMC5644961; doi:10.7554/eLife.28158)
Supplement: Source code 3. [file elife-28158-code3.zip › RT analysis code/OpenCV1.1pre.zip!/OpenCV/docs/ref/opencvref_cvaux.htm]

OpenCV: Experimental and Obsolete Functionality

# Experimental and Obsolete Functionality Reference

The functionality resides in cvaux library. To use it in your application, place
#include "cvaux.h" in your source files and:

- In case of Win32 link the app against cvaux.lib that is import library for cvaux.dll- In case of Linux use -lcvaux compiler option

---

- Stereo Correspondence Functions
  - FindStereoCorrespondence
  - View Morphing Functions
    - MakeScanlines- PreWarpImage- FindRuns- DynamicCorrespondMulti- MakeAlphaScanlines- MorphEpilinesMulti- PostWarpImage- DeleteMoire
    - 3D Tracking Functions
      - 3dTrackerCalibrateCameras- 3dTrackerLocateObjects
      - Eigen Objects (PCA) Functions
        - CalcCovarMatrixEx- CalcEigenObjects- CalcDecompCoeff- EigenDecomposite- EigenProjection
        - Embedded Hidden Markov Models Functions
          - HMM- ImgObsInfo- Create2DHMM- Release2DHMM- CreateObsInfo- ReleaseObsInfo- ImgToObs\_DCT- UniformImgSegm- InitMixSegm- EstimateHMMStateParams- EstimateTransProb- EstimateObsProb- EViterbi- MixSegmL2

---

## Stereo Correspondence Functions

---

### FindStereoCorrespondence

Calculates disparity for stereo-pair

```
cvFindStereoCorrespondence(
                   const  CvArr* leftImage, const  CvArr* rightImage,
                   int     mode, CvArr*  depthImage,
                   int     maxDisparity,
                   double  param1, double  param2, double  param3,
                   double  param4, double  param5  );
```

leftImage: Left image of stereo pair, rectified grayscale 8-bit image rightImage: Right image of stereo pair, rectified grayscale 8-bit image mode: Algorithm used to find a disparity (now only CV\_DISPARITY\_BIRCHFIELD is supported) depthImage: Destination depth image, grayscale 8-bit image that codes the scaled disparity, so that the zero disparity (corresponding to the points that are very far from the cameras) maps to 0, maximum disparity maps to 255. maxDisparity: Maximum possible disparity. The closer the objects to the cameras, the larger value should be specified here. Too big values slow down the process significantly. param1, param2, param3, param4, param5: - parameters of algorithm. For example, param1 is the constant occlusion penalty, param2 is the constant match reward, param3 defines a highly reliable region (set of contiguous pixels whose reliability is at least param3), param4 defines a moderately reliable region, param5 defines a slightly reliable region. If some parameter is omitted default value is used. In Birchfield's algorithm param1 = 25, param2 = 5, param3 = 12, param4 = 15, param5 = 25 (These values have been taken from "Depth Discontinuities by Pixel-to-Pixel Stereo" Stanford University Technical Report STAN-CS-TR-96-1573, July 1996.)

The function `cvFindStereoCorrespondence` calculates disparity map
for two rectified grayscale images.

Example. Calculating disparity for pair of 8-bit color images

```
/*---------------------------------------------------------------------------------*/
IplImage* srcLeft = cvLoadImage("left.jpg",1);
IplImage* srcRight = cvLoadImage("right.jpg",1);
IplImage* leftImage = cvCreateImage(cvGetSize(srcLeft), IPL_DEPTH_8U, 1);
IplImage* rightImage = cvCreateImage(cvGetSize(srcRight), IPL_DEPTH_8U, 1);
IplImage* depthImage = cvCreateImage(cvGetSize(srcRight), IPL_DEPTH_8U, 1);

cvCvtColor(srcLeft, leftImage, CV_BGR2GRAY);
cvCvtColor(srcRight, rightImage, CV_BGR2GRAY);

cvFindStereoCorrespondence( leftImage, rightImage, CV_DISPARITY_BIRCHFIELD, depthImage, 50, 15, 3, 6, 8, 15 );
/*---------------------------------------------------------------------------------*/
```

And here is the example stereo pair that can be used to test the example

---

## View Morphing Functions

---

### MakeScanlines

Calculates scanlines coordinates for two cameras by fundamental matrix

```
void cvMakeScanlines( const CvMatrix3* matrix, CvSize img_size, int* scanlines1,
                      int* scanlines2, int* lengths1, int* lengths2, int* line_count );
```

matrix: Fundamental matrix. imgSize: Size of the image. scanlines1: Pointer to the array of calculated scanlines of the first image. scanlines2: Pointer to the array of calculated scanlines of the second image. lengths1: Pointer to the array of calculated lengths (in pixels) of the first image scanlines. lengths2: Pointer to the array of calculated lengths (in pixels) of the second image scanlines. line\_count: Pointer to the variable that stores the number of scanlines.

The function `cvMakeScanlines` finds coordinates of scanlines for two images.

This function returns the number of scanlines. The function does nothing except
calculating the number of scanlines if the pointers `scanlines1` or `scanlines2` are
equal to zero.

---

### PreWarpImage

Rectifies image

```
void cvPreWarpImage( int line_count, IplImage* img, uchar* dst,
                     int* dst_nums, int* scanlines );
```

line\_count: Number of scanlines for the image. img: Image to prewarp. dst: Data to store for the prewarp image. dst\_nums: Pointer to the array of lengths of scanlines. scanlines: Pointer to the array of coordinates of scanlines.

The function `cvPreWarpImage` rectifies the image so that the scanlines in the
rectified image are horizontal. The output buffer of size
`max(width,height)*line_count*3` must be allocated before calling the function.

---

### FindRuns

Retrieves scanlines from rectified image and breaks them down into runs

```
void cvFindRuns( int line_count, uchar* prewarp1, uchar* prewarp2,
                 int* line_lengths1, int* line_lengths2,
                 int* runs1, int* runs2,
                 int* num_runs1, int* num_runs2 );
```

line\_count: Number of the scanlines. prewarp1: Prewarp data of the first image. prewarp2: Prewarp data of the second image. line\_lengths1: Array of lengths of scanlines in the first image. line\_lengths2: Array of lengths of scanlines in the second image. runs1: Array of runs in each scanline in the first image. runs2: Array of runs in each scanline in the second image. num\_runs1: Array of numbers of runs in each scanline in the first image. num\_runs2: Array of numbers of runs in each scanline in the second image.

The function `cvFindRuns` retrieves scanlines from the rectified image and breaks
each scanline down into several runs, that is, series of pixels of almost the
same brightness.

---

### DynamicCorrespondMulti

Finds correspondence between two sets of runs of two warped images

```
void cvDynamicCorrespondMulti( int line_count, int* first, int* first_runs,
                               int* second, int* second_runs,
                               int* first_corr, int* second_corr );
```

line\_count: Number of scanlines. first: Array of runs of the first image. first\_runs: Array of numbers of runs in each scanline of the first image. second: Array of runs of the second image. second\_runs: Array of numbers of runs in each scanline of the second image. first\_corr: Pointer to the array of correspondence information found for the first runs. second\_corr: Pointer to the array of correspondence information found for the second runs.

The function `cvDynamicCorrespondMulti` finds correspondence between two sets of
runs of two images. Memory must be allocated before calling this function.
Memory size for one array of correspondence information is

`max( width,height )* numscanlines*3*sizeof ( int )` .

---

### MakeAlphaScanlines

Calculates coordinates of scanlines of image from virtual camera

```
void cvMakeAlphaScanlines( int* scanlines1, int* scanlines2,
                           int* scanlinesA, int* lengths,
                           int line_count, float alpha );
```

scanlines1: Pointer to the array of the first scanlines. scanlines2: Pointer to the array of the second scanlines. scanlinesA: Pointer to the array of the scanlines found in the virtual image. lengths: Pointer to the array of lengths of the scanlines found in the virtual image. line\_count: Number of scanlines. alpha: Position of virtual camera `(0.0 - 1.0)` .

The function `cvMakeAlphaScanlines` finds coordinates of scanlines for the virtual
camera with the given camera position.

Memory must be allocated before calling this function. Memory size for the array
of correspondence runs is `numscanlines*2*4*sizeof(int)` . Memory size for the
array of the scanline lengths is `numscanlines*2*4*sizeof(int)` .

---

### MorphEpilinesMulti

Morphs two pre-warped images using information about stereo correspondence

```
void cvMorphEpilinesMulti( int line_count, uchar* first_pix, int* first_num,
                           uchar* second_pix, int* second_num,
                           uchar* dst_pix, int* dst_num,
                           float alpha, int* first, int* first_runs,
                           int* second, int* second_runs,
                           int* first_corr, int* second_corr );
```

line\_count: Number of scanlines in the prewarp image. first\_pix: Pointer to the first prewarp image. first\_num: Pointer to the array of numbers of points in each scanline in the first image. second\_pix: Pointer to the second prewarp image. second\_num: Pointer to the array of numbers of points in each scanline in the second image. dst\_pix: Pointer to the resulting morphed warped image. dst\_num: Pointer to the array of numbers of points in each line. alpha: Virtual camera position `(0.0 - 1.0)` . first: First sequence of runs. first\_runs: Pointer to the number of runs in each scanline in the first image. second: Second sequence of runs. second\_runs: Pointer to the number of runs in each scanline in the second image. first\_corr: Pointer to the array of correspondence information found for the first runs. second\_corr: Pointer to the array of correspondence information found for the second runs.

The function `cvMorphEpilinesMulti` morphs two pre-warped images using information
about correspondence between the scanlines of two images.

---

### PostWarpImage

Warps rectified morphed image back

```
void cvPostWarpImage( int line_count, uchar* src, int* src_nums,
                      IplImage* img, int* scanlines );
```

line\_count: Number of the scanlines. src: Pointer to the prewarp image virtual image. src\_nums: Number of the scanlines in the image. img: Resulting unwarp image. scanlines: Pointer to the array of scanlines data.

The function `cvPostWarpImage` warps the resultant image from the virtual camera by
storing its rows across the scanlines whose coordinates are calculated by
cvMakeAlphaScanlines.

---

### DeleteMoire

Deletes moire in given image

```
void cvDeleteMoire( IplImage* img );
```

img: Image.

The function `cvDeleteMoire` deletes moire from the given image. The post-warped
image may have black (un-covered) points because of possible holes between
neighboring scanlines. The function deletes moire (black pixels) from the image
by substituting neighboring pixels for black pixels. If all the scanlines are
horizontal, the function may be omitted.

---

## 3D Tracking Functions

The section discusses functions for tracking objects in 3d space using a stereo camera.
Besides C API, there is DirectShow 3dTracker filter
and the wrapper application 3dTracker.
Here you may find a description how to test the filter on sample data.

---

### 3dTrackerCalibrateCameras

Simultaneously determines position and orientation of multiple cameras

```
CvBool cv3dTrackerCalibrateCameras(int num_cameras,
           const Cv3dTrackerCameraIntrinsics camera_intrinsics[],
           CvSize checkerboard_size,
           IplImage *samples[],
           Cv3dTrackerCameraInfo camera_info[]);
```

num\_cameras: the number of cameras to calibrate. This is the size of each of the three array parameters. camera\_intrinsics: camera intrinsics for each camera, such as determined by CalibFilter. checkerboard\_size: the width and height (in number of squares) of the checkerboard. samples: images from each camera, with a view of the checkerboard. camera\_info: filled in with the results of the camera calibration. This is passed into 3dTrackerLocateObjects to do tracking.

The function `cv3dTrackerCalibrateCameras`
searches for a checkerboard of the specified size in each
of the images. For each image in which it finds the checkerboard, it fills
in the corresponding slot in `camera_info` with the position
and orientation of the camera
relative to the checkerboard and sets the `valid` flag.
If it finds the checkerboard in all the images, it returns true;
otherwise it returns false.

This function does not change the members of the `camera_info` array
that correspond to images in which the checkerboard was not found.
This allows you to calibrate each camera independently, instead of
simultaneously.
To accomplish this, do the following:

1. clear all the `valid` flags before calling this function the first time;
2. call this function with each set of images;
3. check all the `valid` flags after each call.
   When all the `valid` flags are set, calibration is complete.

Note that this method works well only if the checkerboard is rigidly mounted;
if it is handheld, all the cameras should be calibrated simultaneously
to get an accurate result.
To ensure that all cameras are calibrated simultaneously,
ignore the `valid` flags and
use the return value to decide when calibration is complete.

---

### 3dTrackerLocateObjects

Determines 3d location of tracked objects

```
int  cv3dTrackerLocateObjects(int num_cameras,
         int num_objects,
         const Cv3dTrackerCameraInfo camera_info[],
         const Cv3dTracker2dTrackedObject tracking_info[],
         Cv3dTrackerTrackedObject tracked_objects[]);
```

num\_cameras: the number of cameras. num\_objects: the maximum number of objects found by any camera. (Also the maximum number of objects returned in `tracked_objects`.) camera\_info: camera position and location information for each camera, as determined by 3dTrackerCalibrateCameras. tracking\_info: the 2d position of each object as seen by each camera. Although this is specified as a one-dimensional array, it is actually a two-dimensional array: `const Cv3dTracker2dTrackedObject tracking_info[num_cameras][num_objects]`. The `id` field of any unused slots must be -1. Ids need not be ordered or consecutive. tracked\_objects: filled in with the results.

The function `cv3dTrackerLocateObjects`
determines the 3d position of tracked objects
based on the 2d tracking information from multiple cameras and
the camera position and orientation information computed by
3dTrackerCalibrateCameras.
It locates any objects with the same `id` that are tracked by more
than one camera.
It fills in the `tracked_objects` array and
returns the number of objects located. The `id` fields of
any unused slots in `tracked_objects` are set to -1.

---

## Eigen Objects (PCA) Functions

The functions described in this section do PCA analysis and compression for
a set of 8-bit images that may not fit into memory all together.
If your data fits into memory and the vectors are not 8-bit (or you want a simpler
interface), use
cvCalcCovarMatrix,
cvSVD and
cvGEMM
to do PCA

---

### CalcCovarMatrixEx

Calculates covariance matrix for group of input objects

```
void cvCalcCovarMatrixEx( int object_count, void* input, int io_flags,
                          int iobuf_size, uchar* buffer, void* userdata,
                          IplImage* avg, float* covar_matrix );
```

object\_count: Number of source objects. input: Pointer either to the array of `IplImage` input objects or to the read callback function according to the value of the parameter `ioFlags`. io\_flags: Input/output flags. iobuf\_size: Input/output buffer size. buffer: Pointer to the input/output buffer. userdata: Pointer to the structure that contains all necessary data for the callback: functions. avg: Averaged object. covar\_matrix: Covariance matrix. An output parameter; must be allocated before the call.

The function `cvCalcCovarMatrixEx` calculates a covariance matrix of the input
objects group using previously calculated averaged object. Depending on `ioFlags`
parameter it may be used either in direct access or callback mode. If `ioFlags` is
not `CV_EIGOBJ_NO_CALLBACK`, buffer must be allocated before calling the
function.

---

### CalcEigenObjects

Calculates orthonormal eigen basis and averaged object for group of input
objects

```
void cvCalcEigenObjects( int nObjects, void* input, void* output, int ioFlags,
                         int ioBufSize, void* userData, CvTermCriteria* calcLimit,
                         IplImage* avg, float* eigVals );
```

nObjects: Number of source objects. input: Pointer either to the array of `IplImage` input objects or to the read callback function according to the value of the parameter `ioFlags`. output: Pointer either to the array of eigen objects or to the write callback function according to the value of the parameter ioFlags . ioFlags: Input/output flags. ioBufSize: Input/output buffer size in bytes. The size is zero, if unknown. userData: Pointer to the structure that contains all necessary data for the callback functions. calcLimit: Criteria that determine when to stop calculation of eigen objects. avg: Averaged object. eigVals: Pointer to the eigenvalues array in the descending order; may be `NULL` .

The function `cvCalcEigenObjects` calculates orthonormal eigen basis and the
averaged object for a group of the input objects. Depending on `ioFlags` parameter
it may be used either in direct access or callback mode. Depending on the
parameter `calcLimit`, calculations are finished either after first
`calcLimit.max_iter` dominating eigen objects are retrieved or if the ratio of the
current eigenvalue to the largest eigenvalue comes down to `calcLimit.epsilon`
threshold. The value `calcLimit -> type` must be `CV_TERMCRIT_NUMB,
CV_TERMCRIT_EPS`, or `CV_TERMCRIT_NUMB | CV_TERMCRIT_EPS` . The function returns
the real values `calcLimit->max_iter` and `calcLimit->epsilon` .

The function also calculates the averaged object, which must be created
previously. Calculated eigen objects are arranged according to the corresponding
eigenvalues in the descending order.

The parameter `eigVals` may be equal to `NULL`, if eigenvalues are not needed.

The function `cvCalcEigenObjects` uses the function
cvCalcCovarMatrixEx.

---

### CalcDecompCoeff

Calculates decomposition coefficient of input object

```
double cvCalcDecompCoeff( IplImage* obj, IplImage* eigObj, IplImage* avg );
```

obj: Input object. eigObj: Eigen object. avg: Averaged object.

The function `cvCalcDecompCoeff` calculates one decomposition coefficient of the
input object using the previously calculated eigen object and the averaged
object.

---

### EigenDecomposite

Calculates all decomposition coefficients for input object

```
void cvEigenDecomposite( IplImage* obj, int eigenvec_count, void* eigInput,
                         int ioFlags, void* userData, IplImage* avg, float* coeffs );
```

obj: Input object. eigenvec\_count: Number of eigen objects. eigInput: Pointer either to the array of `IplImage` input objects or to the read callback function according to the value of the parameter `ioFlags`. ioFlags: Input/output flags. userData: Pointer to the structure that contains all necessary data for the callback functions. avg: Averaged object. coeffs: Calculated coefficients; an output parameter.

The function `cvEigenDecomposite` calculates all decomposition coefficients for the
input object using the previously calculated eigen objects basis and the
averaged object. Depending on `ioFlags` parameter it may be used either in direct
access or callback mode.

---

### EigenProjection

Calculates object projection to the eigen sub-space

```
void cvEigenProjection( void* input_vecs, int eigenvec_count, int io_flags, void* userdata,
                        float* coeffs, IplImage* avg, IplImage* proj );
```

input\_vec: Pointer to either an array of `IplImage` input objects or to a callback function, depending on `io_flags`. eigenvec\_count: Number of eigenvectors. io\_flags: Input/output flags; see cvCalcEigenObjects. userdata: Pointer to the structure that contains all necessary data for the callback functions. coeffs: Previously calculated decomposition coefficients. avg: Average vector, calculated by cvCalcEigenObjects. proj: Projection to the eigen sub-space.

The function `cvEigenProjection` calculates an object projection to the eigen
sub-space or, in other words, restores an object using previously calculated
eigen objects basis, averaged object, and decomposition coefficients of the
restored object. Depending on `io_flags` parameter it may be used either in direct
access or callback mode.

---

## Embedded Hidden Markov Models Functions

In order to support embedded models the user must define structures to represent
1D HMM and 2D embedded HMM model.

---

### CvHMM

Embedded HMM Structure

```
    typedef struct _CvEHMM 
    {
        int level; 
        int num_states; 
        float* transP; 
        float** obsProb; 
        union 
        { 
            CvEHMMState* state;
            struct _CvEHMM* ehmm; 
        } u;
    } CvEHMM;
```

level: Level of embedded HMM. If `level ==0`, HMM is most external. In 2D HMM there are two types of HMM: 1 external and several embedded. External HMM has `level ==1`, embedded HMMs have `level ==0` . num\_states: Number of states in 1D HMM. transP: State-to-state transition probability, square matrix `(num_state×num_state )`. obsProb: Observation probability matrix. state: Array of HMM states. For the last-level HMM, that is, an HMM without embedded HMMs, HMM states are real. ehmm: Array of embedded HMMs. If HMM is not last-level, then HMM states are not real and they are HMMs.

For representation of observations the following structure is defined:

---

### CvImgObsInfo

Image Observation Structure

```
    typedef struct CvImgObsInfo
    {
        int obs_x;
        int obs_y;
        int obs_size;
        float** obs;
        int* state;
        int* mix;
    } CvImgObsInfo;
```

obs\_x: Number of observations in the horizontal direction. obs\_y: Number of observations in the vertical direction. obs\_size: Length of every observation vector. obs: Pointer to observation vectors stored consequently. Number of vectors is `obs_x*obs_y` . state: Array of indices of states, assigned to every observation vector. mix: Index of mixture component, corresponding to the observation vector within an assigned state.

---

### Create2DHMM

Creates 2D embedded HMM

```
CvEHMM* cvCreate2DHMM( int* stateNumber, int* numMix, int obsSize );
```

stateNumber: Array, the first element of the which specifies the number of superstates in the HMM. All subsequent elements specify the number of states in every embedded HMM, corresponding to each superstate. So, the length of the array is `stateNumber [0]+1` . numMix: Array with numbers of Gaussian mixture components per each internal state. The number of elements in the array is equal to number of internal states in the HMM, that is, superstates are not counted here. obsSize: Size of observation vectors to be used with created HMM.

The function `cvCreate2DHMM` returns the created structure of the type CvEHMM with
specified parameters.

---

### Release2DHMM

Releases 2D embedded HMM

```
void cvRelease2DHMM(CvEHMM** hmm );
```

hmm: Address of pointer to HMM to be released.

The function `cvRelease2DHMM` frees all the memory used by HMM and clears the
pointer to HMM.

---

### CreateObsInfo

Creates structure to store image observation vectors

```
CvImgObsInfo* cvCreateObsInfo( CvSize numObs, int obsSize );
```

numObs: Numbers of observations in the horizontal and vertical directions. For the given image and scheme of extracting observations the parameter can be computed via the macro `CV_COUNT_OBS( roi, dctSize, delta, numObs )`, where `roi, dctSize, delta, numObs` are the pointers to structures of the type CvSize . The pointer `roi` means size of `roi` of image observed, `numObs` is the output parameter of the macro. obsSize: Size of observation vectors to be stored in the structure.

The function `cvCreateObsInfo` creates new structures to store image observation
vectors. For definitions of the parameters `roi, dctSize`, and `delta` see the
specification of The function `cvImgToObs_DCT`.

---

### ReleaseObsInfo

Releases observation vectors structure

```
void cvReleaseObsInfo( CvImgObsInfo** obsInfo );
```

obsInfo: Address of the pointer to the structure CvImgObsInfo .

The function `cvReleaseObsInfo` frees all memory used by observations and clears
pointer to the structure CvImgObsInfo .

---

### ImgToObs\_DCT

Extracts observation vectors from image

```
void cvImgToObs_DCT( IplImage* image, float* obs, CvSize dctSize,
                     CvSize obsSize, CvSize delta );
```

image: Input image. obs: Pointer to consequently stored observation vectors. dctSize: Size of image blocks for which DCT (Discrete Cosine Transform) coefficients are to be computed. obsSize: Number of the lowest DCT coefficients in the horizontal and vertical directions to be put into the observation vector. delta: Shift in pixels between two consecutive image blocks in the horizontal and vertical directions.

The function `cvImgToObs_DCT` extracts observation vectors, that is, DCT
coefficients, from the image. The user must pass `obsInfo.obs` as the parameter
`obs` to use this function with other HMM functions and use the structure `obsInfo`
of the CvImgObsInfo type.

`Calculating Observations for HMM`

```
    CvImgObsInfo* obs_info;

        ...

        cvImgToObs_DCT( image,obs_info->obs, //!!!

        dctSize, obsSize, delta );
```

---

### UniformImgSegm

Performs uniform segmentation of image observations by HMM states

```
void cvUniformImgSegm( CvImgObsInfo* obsInfo, CvEHMM* hmm );
```

obsInfo: Observations structure. hmm: HMM structure.

The function `cvUniformImgSegm` segments image observations by HMM states uniformly
(see Initial Segmentation for 2D Embedded HMM for 2D embedded HMM with 5
superstates and 3, 6, 6, 6, 3 internal states of every corresponding
superstate).

Initial Segmentation for 2D Embedded HMM

---

### InitMixSegm

Segments all observations within every internal state of HMM by state mixture
components

```
void cvInitMixSegm( CvImgObsInfo** obsInfoArray, int numImg, CvEHMM* hmm );
```

obsInfoArray: Array of pointers to the observation structures. numImg: Length of above array. hmm: HMM.

The function `cvInitMixSegm` takes a group of observations from several training
images already segmented by states and splits a set of observation vectors
within every internal HMM state into as many clusters as the number of mixture
components in the state.

---

### EstimateHMMStateParams

Estimates all parameters of every HMM state

```
void cvEstimateHMMStateParams( CvImgObsInfo** obsInfoArray, int numImg, CvEHMM* hmm );
```

obsInfoArray: Array of pointers to the observation structures. numImg: Length of the array. hmm: HMM.

The function `cvEstimateHMMStateParams` computes all inner parameters of every HMM
state, including Gaussian means, variances, etc.

---

### EstimateTransProb

Computes transition probability matrices for embedded HMM

```
void cvEstimateTransProb( CvImgObsInfo** obsInfoArray, int numImg, CvEHMM* hmm );
```

obsInfoArray: Array of pointers to the observation structures. numImg: Length of the above array. hmm: HMM.

The function `cvEstimateTransProb` uses current segmentation of image observations
to compute transition probability matrices for all embedded and external HMMs.

---

### EstimateObsProb

Computes probability of every observation of several images

```
void cvEstimateObsProb( CvImgObsInfo* obsInfo, CvEHMM* hmm );
```

obsInfo: Observation structure. hmm: HMM structure.

The function `cvEstimateObsProb` computes Gaussian probabilities of each observation
to occur in each of the internal HMM states.

---

### EViterbi

Executes Viterbi algorithm for embedded HMM

```
float cvEViterbi( CvImgObsInfo* obsInfo, CvEHMM* hmm );
```

obsInfo: Observation structure. hmm: HMM structure.

The function `cvEViterbi` executes Viterbi algorithm for embedded HMM. Viterbi
algorithm evaluates the likelihood of the best match between the given image
observations and the given HMM and performs segmentation of image observations
by HMM states. The segmentation is done on the basis of the match found.

---

### MixSegmL2

Segments observations from all training images by mixture components of newly
assigned states

```
void cvMixSegmL2( CvImgObsInfo** obsInfoArray, int numImg, CvEHMM* hmm );
```

obsInfoArray: Array of pointers to the observation structures. numImg: Length of the array. hmm: HMM.

The function `cvMixSegmL2` segments observations from all training images by mixture
components of newly Viterbi algorithm-assigned states. The function uses
Euclidean distance to group vectors around the existing mixtures centers.
