# Supplementary material for: Rapid whole brain imaging of neural activity in freely behaving larval zebrafish (Danio rerio)
Source: eLife. 2017 Sep 20;6:e28158. doi: 10.7554/eLife.28158 (PMC5644961; doi:10.7554/eLife.28158)
Supplement: Source code 3. [file elife-28158-code3.zip › RT analysis code/OpenCV1.1pre.zip!/OpenCV/docs/ref/opencvref_cxcore.htm]

CXCORE Reference Manual

# CXCORE Reference Manual

---

- Basic Structures- Operations on Arrays
    - Initialization- Accessing Elements and sub-Arrays- Copying and Filling- Transforms and Permutations- Arithmetic, Logic and Comparison- Statistics- Linear Algebra- Math Functions- Random Number Generation- Discrete Transforms
    - Dynamic Structures
      - Memory Storages- Sequences- Sets- Graphs- Trees
      - Drawing Functions
        - Curves and Shapes- Text- Point Sets and Contours
        - Data Persistence and RTTI
          - File Storage- Writing Data- Reading Data- RTTI and Generic Functions
          - Miscellaneous Functions- Error Handling and System Functions
              - Error Handling- System Functions
              - Alphabetical List of Functions- List of Examples


---

# Basic Structures

---

### CvPoint

2D point with integer coordinates

```
    typedef struct CvPoint
    {
        int x; /* x-coordinate, usually zero-based */
        int y; /* y-coordinate, usually zero-based */
    }
    CvPoint;

    /* the constructor function */
    inline CvPoint cvPoint( int x, int y );

    /* conversion from CvPoint2D32f */
    inline CvPoint cvPointFrom32f( CvPoint2D32f point );
```

---

### CvPoint2D32f

2D point with floating-point coordinates

```
    typedef struct CvPoint2D32f
    {
        float x; /* x-coordinate, usually zero-based */
        float y; /* y-coordinate, usually zero-based */
    }
    CvPoint2D32f;

    /* the constructor function */
    inline CvPoint2D32f cvPoint2D32f( double x, double y );

    /* conversion from CvPoint */
    inline CvPoint2D32f cvPointTo32f( CvPoint point );
```

---

### CvPoint3D32f

3D point with floating-point coordinates

```
    typedef struct CvPoint3D32f
    {
        float x; /* x-coordinate, usually zero-based */
        float y; /* y-coordinate, usually zero-based */
        float z; /* z-coordinate, usually zero-based */
    }
    CvPoint3D32f;

    /* the constructor function */
    inline CvPoint3D32f cvPoint3D32f( double x, double y, double z );
```

---

### CvPoint2D64f

2D point with double precision floating-point coordinates

```
    typedef struct CvPoint2D64f
    {
        double x; /* x-coordinate, usually zero-based */
        double y; /* y-coordinate, usually zero-based */
    }
    CvPoint2D64f;

    /* the constructor function */
    inline CvPoint2D64f cvPoint2D64f( double x, double y );

    /* conversion from CvPoint */
    inline CvPoint2D64f cvPointTo64f( CvPoint point );
```

---

### CvPoint3D64f

3D point with double precision floating-point coordinates

```
    typedef struct CvPoint3D64f
    {
        double x; /* x-coordinate, usually zero-based */
        double y; /* y-coordinate, usually zero-based */
        double z; /* z-coordinate, usually zero-based */
    }
    CvPoint3D64f;

    /* the constructor function */
    inline CvPoint3D64f cvPoint3D64f( double x, double y, double z );
```

---

### CvSize

pixel-accurate size of a rectangle

```
    typedef struct CvSize
    {
        int width; /* width of the rectangle */
        int height; /* height of the rectangle */
    }
    CvSize;

    /* the constructor function */
    inline CvSize cvSize( int width, int height );
```

---

### CvSize2D32f

sub-pixel accurate size of a rectangle

```
    typedef struct CvSize2D32f
    {
        float width; /* width of the box */
        float height; /* height of the box */
    }
    CvSize2D32f;

    /* the constructor function */
    inline CvSize2D32f cvSize2D32f( double width, double height );
```

---

### CvRect

offset and size of a rectangle

```
    typedef struct CvRect
    {
        int x; /* x-coordinate of the left-most rectangle corner[s] */
        int y; /* y-coordinate of the top-most or bottom-most
                  rectangle corner[s] */
        int width; /* width of the rectangle */
        int height; /* height of the rectangle */
    }
    CvRect;

    /* the constructor function */
    inline CvRect cvRect( int x, int y, int width, int height );
```

---

### CvScalar

A container for 1-,2-,3- or 4-tuples of numbers

```
    typedef struct CvScalar
    {
        double val[4];
    }
    CvScalar;

    /* the constructor function: initializes val[0] with val0, val[1] with val1 etc. */
    inline CvScalar cvScalar( double val0, double val1=0,
                              double val2=0, double val3=0 );
    /* the constructor function: initializes val[0]...val[3] with val0123 */
    inline CvScalar cvScalarAll( double val0123 );

    /* the constructor function: initializes val[0] with val0, val[1]...val[3] with zeros */
    inline CvScalar cvRealScalar( double val0 );
```

---

### CvTermCriteria

Termination criteria for iterative algorithms

```
#define CV_TERMCRIT_ITER    1
#define CV_TERMCRIT_NUMBER  CV_TERMCRIT_ITER
#define CV_TERMCRIT_EPS     2

typedef struct CvTermCriteria
{
    int    type;  /* a combination of CV_TERMCRIT_ITER and CV_TERMCRIT_EPS */
    int    max_iter; /* maximum number of iterations */
    double epsilon; /* accuracy to achieve */
}
CvTermCriteria;

/* the constructor function */
inline  CvTermCriteria  cvTermCriteria( int type, int max_iter, double epsilon );

/* check termination criteria and transform it so that type=CV_TERMCRIT_ITER+CV_TERMCRIT_EPS,
   and both max_iter and epsilon are valid */
CvTermCriteria cvCheckTermCriteria( CvTermCriteria criteria,
                                    double default_eps,
                                    int default_max_iters );
```


---

### CvMat

Multi-channel matrix

```
    typedef struct CvMat
    {
        int type; /* CvMat signature (CV_MAT_MAGIC_VAL), element type and flags */
        int step; /* full row length in bytes */

        int* refcount; /* underlying data reference counter */

        union
        {
            uchar* ptr;
            short* s;
            int* i;
            float* fl;
            double* db;
        } data; /* data pointers */

    #ifdef __cplusplus
        union
        {
            int rows;
            int height;
        };

        union
        {
            int cols;
            int width;
        };
    #else
        int rows; /* number of rows */
        int cols; /* number of columns */
    #endif

    } CvMat;
```

---

### CvMatND

Multi-dimensional dense multi-channel array

```
    typedef struct CvMatND
    {
        int type; /* CvMatND signature (CV_MATND_MAGIC_VAL), element type and flags */
        int dims; /* number of array dimensions */

        int* refcount; /* underlying data reference counter */

        union
        {
            uchar* ptr;
            short* s;
            int* i;
            float* fl;
            double* db;
        } data; /* data pointers */

        /* pairs (number of elements, distance between elements in bytes) for
           every dimension */
        struct
        {
            int size;
            int step;
        }
        dim[CV_MAX_DIM];

    } CvMatND;
```

---

### CvSparseMat

Multi-dimensional sparse multi-channel array

```
    typedef struct CvSparseMat
    {
        int type; /* CvSparseMat signature (CV_SPARSE_MAT_MAGIC_VAL), element type and flags */
        int dims; /* number of dimensions */
        int* refcount; /* reference counter - not used */
        struct CvSet* heap; /* a pool of hashtable nodes */
        void** hashtable; /* hashtable: each entry has a list of nodes
                             having the same "hashvalue modulo hashsize" */
        int hashsize; /* size of hashtable */
        int total; /* total number of sparse array nodes */
        int valoffset; /* value offset in bytes for the array nodes */
        int idxoffset; /* index offset in bytes for the array nodes */
        int size[CV_MAX_DIM]; /* arr of dimension sizes */

    } CvSparseMat;
```

---

### IplImage

IPL image header

```
    typedef struct _IplImage
    {
        int  nSize;         /* sizeof(IplImage) */
        int  ID;            /* version (=0)*/
        int  nChannels;     /* Most of OpenCV functions support 1,2,3 or 4 channels */
        int  alphaChannel;  /* ignored by OpenCV */
        int  depth;         /* pixel depth in bits: IPL_DEPTH_8U, IPL_DEPTH_8S, IPL_DEPTH_16U,
                               IPL_DEPTH_16S, IPL_DEPTH_32S, IPL_DEPTH_32F and IPL_DEPTH_64F are supported */
        char colorModel[4]; /* ignored by OpenCV */
        char channelSeq[4]; /* ditto */
        int  dataOrder;     /* 0 - interleaved color channels, 1 - separate color channels.
                               cvCreateImage can only create interleaved images */
        int  origin;        /* 0 - top-left origin,
                               1 - bottom-left origin (Windows bitmaps style) */
        int  align;         /* Alignment of image rows (4 or 8).
                               OpenCV ignores it and uses widthStep instead */
        int  width;         /* image width in pixels */
        int  height;        /* image height in pixels */
        struct _IplROI *roi;/* image ROI. when it is not NULL, this specifies image region to process */
        struct _IplImage *maskROI; /* must be NULL in OpenCV */
        void  *imageId;     /* ditto */
        struct _IplTileInfo *tileInfo; /* ditto */
        int  imageSize;     /* image data size in bytes
                               (=image->height*image->widthStep
                               in case of interleaved data)*/
        char *imageData;  /* pointer to aligned image data */
        int  widthStep;   /* size of aligned image row in bytes */
        int  BorderMode[4]; /* border completion mode, ignored by OpenCV */
        int  BorderConst[4]; /* ditto */
        char *imageDataOrigin; /* pointer to a very origin of image data
                                  (not necessarily aligned) -
                                  it is needed for correct image deallocation */
    }
    IplImage;
```

The structure `IplImage` came from *Intel Image Processing Library* where
the format is native. OpenCV supports only a subset of possible `IplImage` formats:

- `alphaChannel` is ignored by OpenCV.- `colorModel` and `channelSeq` are ignored by OpenCV. The single OpenCV function
    cvCvtColor working with color spaces takes the source and destination color spaces
    as a parameter.- `dataOrder` must be IPL\_DATA\_ORDER\_PIXEL (the color channels are interleaved), however
      selected channels of planar images can be processed as well if COI is set.- `align` is ignored by OpenCV, while `widthStep` is used to access to subsequent image rows.- `maskROI` is not supported. The function that can work with mask take it as a
          separate parameter. Also the mask in OpenCV is 8-bit, whereas in IPL it is 1-bit.- `tileInfo` is not supported.- `BorderMode` and `BorderConst` are not supported. Every OpenCV function
              working with a pixel neighborhood uses a single hard-coded border mode (most often, replication).

Besides the above restrictions, OpenCV handles ROI differently. It requires that the sizes or ROI sizes of
all source and destination images match exactly (according to the operation, e.g. for cvPyrDown
destination width(height) must be equal to source width(height) divided by 2 ±1),
whereas IPL processes the intersection area - that is, the sizes or ROI sizes of all images may
vary independently.

---

### CvArr

Arbitrary array

```
    typedef void CvArr;
```

The metatype `CvArr*` is used *only* as a function parameter to
specify that the function accepts arrays of more than a
single type, for example IplImage\*, CvMat\* or even CvSeq\*. The particular array
type is determined at runtime by analysing the first 4 bytes of the header.


---

# Operations on Arrays

---

## Initialization

---

### CreateImage

Creates header and allocates data

```
IplImage* cvCreateImage( CvSize size, int depth, int channels );
```

size: Image width and height. depth: Bit depth of image elements. Can be one of: IPL\_DEPTH\_8U - unsigned 8-bit integers IPL\_DEPTH\_8S - signed 8-bit integers IPL\_DEPTH\_16U - unsigned 16-bit integers IPL\_DEPTH\_16S - signed 16-bit integers IPL\_DEPTH\_32S - signed 32-bit integers IPL\_DEPTH\_32F - single precision floating-point numbers IPL\_DEPTH\_64F - double precision floating-point numbers channels: Number of channels per element(pixel). Can be 1, 2, 3 or 4. The channels are interleaved, for example the usual data layout of a color image is: b0 g0 r0 b1 g1 r1 ... Although in general IPL image format can store non-interleaved images as well and some of OpenCV can process it, this function can create interleaved images only.

The function `cvCreateImage` creates the header and allocates data. This call is a
shortened form of

```
    header = cvCreateImageHeader(size,depth,channels);
    cvCreateData(header);
```

---

### CreateImageHeader

Allocates, initializes, and returns structure IplImage

```
IplImage* cvCreateImageHeader( CvSize size, int depth, int channels );
```

size: Image width and height. depth: Image depth (see CreateImage). channels: Number of channels (see CreateImage).

The function `cvCreateImageHeader` allocates, initializes, and returns the structure
`IplImage`. This call is an analogue of

```
  iplCreateImageHeader( channels, 0, depth,
                        channels == 1 ? "GRAY" : "RGB",
                        channels == 1 ? "GRAY" : channels == 3 ? "BGR" :
                        channels == 4 ? "BGRA" : "",
                        IPL_DATA_ORDER_PIXEL, IPL_ORIGIN_TL, 4,
                        size.width, size.height,
                        0,0,0,0);
```

though it does not use IPL functions by default
(see also `CV_TURN_ON_IPL_COMPATIBILITY` macro)

---

### ReleaseImageHeader

Releases header

```
void cvReleaseImageHeader( IplImage** image );
```

image: Double pointer to the deallocated header.

The function `cvReleaseImageHeader` releases the header.
This call is an analogue of

```
    if( image )
    {
        iplDeallocate( *image, IPL_IMAGE_HEADER | IPL_IMAGE_ROI );
        *image = 0;
    }
```

though it does not use IPL functions by default
(see also `CV_TURN_ON_IPL_COMPATIBILITY`)

---

### ReleaseImage

Releases header and image data

```
void cvReleaseImage( IplImage** image );
```

image: Double pointer to the header of the deallocated image.

The function `cvReleaseImage` releases the header and the image data. This call is a
shortened form of

```
    if( *image )
    {
        cvReleaseData( *image );
        cvReleaseImageHeader( image );
    }
```

---

### InitImageHeader

Initializes allocated by user image header

```
IplImage* cvInitImageHeader( IplImage* image, CvSize size, int depth,
                             int channels, int origin=0, int align=4 );
```

image: Image header to initialise. size: Image width and height. depth: Image depth (see CreateImage). channels: Number of channels (see CreateImage). origin: `IPL_ORIGIN_TL` or `IPL_ORIGIN_BL`. align: Alignment for image rows, typically 4 or 8 bytes.

The function `cvInitImageHeader` initializes the image header structure,
pointer to which is passed by the user, and returns the pointer.

---

### CloneImage

Makes a full copy of image

```
IplImage* cvCloneImage( const IplImage* image );
```

image: Original image.

The function `cvCloneImage` makes a full copy of the image including
header, ROI and data

---

### SetImageCOI

Sets channel of interest to given value

```
void cvSetImageCOI( IplImage* image, int coi );
```

image: Image header. coi: Channel of interest.

The function `cvSetImageCOI` sets the channel of interest
to a given value. Value 0 means that all channels are selected,
1 means that the first channel is selected etc.
If ROI is `NULL` and `coi != 0`, ROI is allocated.
Note that most of OpenCV functions do not support COI, so
to process separate image/matrix channel one may copy (via cvCopy or
cvSplit) the channel to separate image/matrix,
process it and copy the result back (via cvCopy or cvCvtPlaneToPix)
if need.

---

### GetImageCOI

Returns index of channel of interest

```
int cvGetImageCOI( const IplImage* image );
```

image: Image header.

The function `cvGetImageCOI` returns channel of interest of
the image (it returns 0 if all the channels are selected).

---

### SetImageROI

Sets image ROI to given rectangle

```
void cvSetImageROI( IplImage* image, CvRect rect );
```

image: Image header. rect: ROI rectangle.

The function `cvSetImageROI` sets the image ROI to a given rectangle. If ROI is `NULL`
and the value of the parameter `rect` is not equal to the whole image, ROI is
allocated. Unlike COI, most of OpenCV functions do support ROI and treat
it in a way as it would be a separate image (for example, all the pixel
coordinates are counted from top-left or bottom-left
(depending on the image origin) corner of ROI)

---

### ResetImageROI

Releases image ROI

```
void cvResetImageROI( IplImage* image );
```

image: Image header.

The function `cvResetImageROI` releases image ROI. After that the whole image
is considered selected. The similar result can be achieved by

```
cvSetImageROI( image, cvRect( 0, 0, image->width, image->height ));
cvSetImageCOI( image, 0 );
```

But the latter variant does not deallocate `image->roi`.

---

### GetImageROI

Returns image ROI coordinates

```
CvRect cvGetImageROI( const IplImage* image );
```

image: Image header.

The function `cvGetImageROI` returns image ROI coordinates.
The rectangle cvRect(0,0,image->width,image->height) is returned if
there is no ROI

---

### CreateMat

Creates new matrix

```
CvMat* cvCreateMat( int rows, int cols, int type );
```

rows: Number of rows in the matrix. cols: Number of columns in the matrix. type: Type of the matrix elements. Usually it is specified in form `CV_<bit_depth>(S|U|F)C<number_of_channels>`, for example: `CV_8UC1` means an 8-bit unsigned single-channel matrix, `CV_32SC2` means a 32-bit signed matrix with two channels.

The function `cvCreateMat` allocates header for the new matrix and underlying data,
and returns a pointer to the created matrix. It is a short form for:

```
    CvMat* mat = cvCreateMatHeader( rows, cols, type );
    cvCreateData( mat );
```

Matrices are stored row by row. All the rows are aligned by 4 bytes.

---

### CreateMatHeader

Creates new matrix header

```
CvMat* cvCreateMatHeader( int rows, int cols, int type );
```

rows: Number of rows in the matrix. cols: Number of columns in the matrix. type: Type of the matrix elements (see cvCreateMat).

The function `cvCreateMatHeader` allocates new matrix header and returns pointer to
it. The matrix data can further be allocated using cvCreateData or set
explicitly to user-allocated data via cvSetData.

---

### ReleaseMat

Deallocates matrix

```
void cvReleaseMat( CvMat** mat );
```

mat: Double pointer to the matrix.

The function `cvReleaseMat` decrements the matrix data reference counter and
releases matrix header:

```
    if( *mat )
        cvDecRefData( *mat );
    cvFree( (void**)mat );
```

---

### InitMatHeader

Initializes matrix header

```
CvMat* cvInitMatHeader( CvMat* mat, int rows, int cols, int type,
                        void* data=NULL, int step=CV_AUTOSTEP );
```

mat: Pointer to the matrix header to be initialised. rows: Number of rows in the matrix. cols: Number of columns in the matrix. type: Type of the matrix elements. data: Optional data pointer assigned to the matrix header. step: Full row width in bytes of the data assigned. By default, the minimal possible step is used, i.e., no gaps is assumed between subsequent rows of the matrix.

The function `cvInitMatHeader` initializes already allocated CvMat structure. It can
be used to process raw data with OpenCV matrix functions.

For example, the following code computes matrix product of two matrices, stored
as ordinary arrays.

Calculating Product of Two Matrices

```
   double a[] = { 1, 2, 3, 4
                  5, 6, 7, 8,
                  9, 10, 11, 12 };

   double b[] = { 1, 5, 9,
                  2, 6, 10,
                  3, 7, 11,
                  4, 8, 12 };

   double c[9];
   CvMat Ma, Mb, Mc ;

   cvInitMatHeader( &Ma, 3, 4, CV_64FC1, a );
   cvInitMatHeader( &Mb, 4, 3, CV_64FC1, b );
   cvInitMatHeader( &Mc, 3, 3, CV_64FC1, c );

   cvMatMulAdd( &Ma, &Mb, 0, &Mc );
   // c array now contains product of a(3x4) and b(4x3) matrices
```

---

### Mat

Initializes matrix header (light-weight variant)

```
CvMat cvMat( int rows, int cols, int type, void* data=NULL );
```

rows: Number of rows in the matrix. cols: Number of columns in the matrix. type: Type of the matrix elements (see CreateMat). data: Optional data pointer assigned to the matrix header.

The function `cvMat` is a fast inline substitution for cvInitMatHeader.
Namely, it is equivalent to:

```
     CvMat mat;
     cvInitMatHeader( &mat, rows, cols, type, data, CV_AUTOSTEP );
```

---

### CloneMat

Creates matrix copy

```
CvMat* cvCloneMat( const CvMat* mat );
```

mat: Input matrix.

The function `cvCloneMat` creates a copy of input matrix and returns the pointer to
it.

---

### CreateMatND

Creates multi-dimensional dense array

```
CvMatND* cvCreateMatND( int dims, const int* sizes, int type );
```

dims: Number of array dimensions. It must not exceed CV\_MAX\_DIM (=32 by default, though it may be changed at build time) sizes: Array of dimension sizes. type: Type of array elements. The same as for CvMat

The function `cvCreateMatND` allocates header for multi-dimensional dense array
and the underlying data, and returns pointer to the created array. It is a short form for:

```
    CvMatND* mat = cvCreateMatNDHeader( dims, sizes, type );
    cvCreateData( mat );
```

Array data is stored row by row. All the rows are aligned by 4 bytes.

---

### CreateMatNDHeader

Creates new matrix header

```
CvMatND* cvCreateMatNDHeader( int dims, const int* sizes, int type );
```

dims: Number of array dimensions. sizes: Array of dimension sizes. type: Type of array elements. The same as for CvMat

The function `cvCreateMatND` allocates header for multi-dimensional dense array.
The array data can further be allocated using cvCreateData or set
explicitly to user-allocated data via cvSetData.

---

### ReleaseMatND

Deallocates multi-dimensional array

```
void cvReleaseMatND( CvMatND** mat );
```

mat: Double pointer to the array.

The function `cvReleaseMatND` decrements the array data reference counter and
releases the array header:

```
    if( *mat )
        cvDecRefData( *mat );
    cvFree( (void**)mat );
```

---

### InitMatNDHeader

Initializes multi-dimensional array header

```
CvMatND* cvInitMatNDHeader( CvMatND* mat, int dims, const int* sizes, int type, void* data=NULL );
```

mat: Pointer to the array header to be initialized. dims: Number of array dimensions. sizes: Array of dimension sizes. type: Type of array elements. The same as for CvMat data: Optional data pointer assigned to the matrix header.

The function `cvInitMatNDHeader` initializes CvMatND structure allocated by
the user.

---

### CloneMatND

Creates full copy of multi-dimensional array

```
CvMatND* cvCloneMatND( const CvMatND* mat );
```

mat: Input array.

The function `cvCloneMatND` creates a copy of input array and returns pointer to
it.

---

### DecRefData

Decrements array data reference counter

```
void cvDecRefData( CvArr* arr );
```

arr: array header.

The function `cvDecRefData` decrements CvMat or CvMatND data reference counter if the
reference counter pointer is not NULL and deallocates the data if the counter reaches zero.
In the current implementation the reference counter is not NULL only if the data was allocated using
cvCreateData function, in other cases such as:  
external data was assigned to the header using cvSetData  
the matrix header presents a part of a larger matrix or image  
the matrix header was converted from image or n-dimensional matrix header  
  
the reference counter is set to NULL and thus it is not decremented.
Whenever the data is deallocated or not,
the data pointer and reference counter pointers are cleared by the function.

---

### IncRefData

Increments array data reference counter

```
int cvIncRefData( CvArr* arr );
```

arr: array header.

The function `cvIncRefData` increments CvMat or CvMatND data reference counter and
returns the new counter value if the reference counter pointer is not NULL, otherwise it returns zero.

---

### CreateData

Allocates array data

```
void cvCreateData( CvArr* arr );
```

arr: Array header.

The function `cvCreateData` allocates image, matrix or multi-dimensional array data.
Note that in case of matrix types OpenCV allocation functions are used and
in case of IplImage they are used too unless `CV_TURN_ON_IPL_COMPATIBILITY` was called.
In the latter case IPL functions are used to allocate the data

---

### ReleaseData

Releases array data

```
void cvReleaseData( CvArr* arr );
```

arr: Array header

The function `cvReleaseData` releases the array data.
In case of CvMat or CvMatND it simply calls cvDecRefData(), that is
the function can not deallocate external data. See also the note to cvCreateData.

---

### SetData

Assigns user data to the array header

```
void cvSetData( CvArr* arr, void* data, int step );
```

arr: Array header. data: User data. step: Full row length in bytes.

The function `cvSetData` assigns user data to the array header.
Header should be initialized before using cvCreate\*Header,
cvInit\*Header or cvMat (in case of matrix) function.

---

### GetRawData

Retrieves low-level information about the array

```
void cvGetRawData( const CvArr* arr, uchar** data,
                   int* step=NULL, CvSize* roi_size=NULL );
```

arr: Array header. data: Output pointer to the whole image origin or ROI origin if ROI is set. step: Output full row length in bytes. roi\_size: Output ROI size.

The function `cvGetRawData` fills output variables with low-level
information about the array data.
All output parameters are optional, so some of the pointers
may be set to `NULL`.
If the array is `IplImage` with ROI set,
parameters of ROI are returned.

The following example shows how to get access to array elements using
this function.

Using GetRawData to calculate absolute value of elements of
a single-channel floating-point array.

```
    float* data;
    int step;

    CvSize size;
    int x, y;

    cvGetRawData( array, (uchar**)&data, &step, &size );
    step /= sizeof(data[0]);

    for( y = 0; y < size.height; y++, data += step )
        for( x = 0; x < size.width; x++ )
            data[x] = (float)fabs(data[x]);
```

---

### GetMat

Returns matrix header for arbitrary array

```
CvMat* cvGetMat( const CvArr* arr, CvMat* header, int* coi=NULL, int allowND=0 );
```

arr: Input array. header: Pointer to CvMat structure used as a temporary buffer. coi: Optional output parameter for storing COI. allowND: If non-zero, the function accepts multi-dimensional dense arrays (CvMatND\*) and returns 2D (if CvMatND has two dimensions) or 1D matrix (when CvMatND has 1 dimension or more than 2 dimensions). The array must be continuous.

The function `cvGetMat` returns matrix header for the input array that can be
matrix - CvMat, image - `IplImage` or multi-dimensional dense array - CvMatND\*
(latter case is allowed only if `allowND != 0`) .
In the case of matrix the function simply returns the input pointer.
In the case of `IplImage*` or CvMatND\* it initializes `header` structure
with parameters of the current image ROI and returns pointer to this temporary
structure. Because COI is not supported by CvMat, it is returned separately.

The function provides an easy way to handle both types of array - `IplImage` and
CvMat -, using the same code. Reverse transform from CvMat to `IplImage` can be
done using cvGetImage function.

Input array must have underlying data allocated or attached, otherwise the
function fails.

If the input array is `IplImage` with planar data layout and COI set, the function
returns pointer to the selected plane and COI = 0. It enables per-plane
processing of multi-channel images with planar data layout using OpenCV
functions.

---

### GetImage

Returns image header for arbitrary array

```
IplImage* cvGetImage( const CvArr* arr, IplImage* image_header );
```

arr: Input array. image\_header: Pointer to `IplImage` structure used as a temporary buffer.

The function `cvGetImage` returns image header for the input array that can be
matrix - CvMat\*, or image - `IplImage*`. In the case of image the function simply
returns the input pointer. In the case of CvMat\* it initializes `image_header` structure
with parameters of the input matrix. Note that if we transform `IplImage` to CvMat and then transform
CvMat back to IplImage, we can get different headers if the ROI is set, and thus some IPL functions
that calculate image stride from its width and align may fail on the resultant image.

---

### CreateSparseMat

Creates sparse array

```
CvSparseMat* cvCreateSparseMat( int dims, const int* sizes, int type );
```

dims: Number of array dimensions. As opposite to the dense matrix, the number of dimensions is practically unlimited (up to 2^16^). sizes: Array of dimension sizes. type: Type of array elements. The same as for CvMat

The function `cvCreateSparseMat` allocates multi-dimensional sparse array.
Initially the array contains no elements, that is cvGet\*D or
cvGetReal\*D return zero for every index

---

### ReleaseSparseMat

Deallocates sparse array

```
void cvReleaseSparseMat( CvSparseMat** mat );
```

mat: Double pointer to the array.

The function `cvReleaseSparseMat` releases the sparse array and clears the array pointer upon exit

---

### CloneSparseMat

Creates full copy of sparse array

```
CvSparseMat* cvCloneSparseMat( const CvSparseMat* mat );
```

mat: Input array.

The function `cvCloneSparseMat` creates a copy of the input array and returns pointer to the copy.

---

## Accessing Elements and sub-Arrays

---

### GetSubRect

Returns matrix header corresponding to the rectangular sub-array of input image or matrix

```
CvMat* cvGetSubRect( const CvArr* arr, CvMat* submat, CvRect rect );
```

arr: Input array. submat: Pointer to the resultant sub-array header. rect: Zero-based coordinates of the rectangle of interest.

The function `cvGetSubRect` returns header, corresponding
to a specified rectangle of the input array.
In other words, it allows the user to treat a rectangular part
of input array as a stand-alone array. ROI is taken into account by the function
so the sub-array of ROI is actually extracted.

---

### GetRow, GetRows

Returns array row or row span

```
CvMat* cvGetRow( const CvArr* arr, CvMat* submat, int row );
CvMat* cvGetRows( const CvArr* arr, CvMat* submat, int start_row, int end_row, int delta_row=1 );
```

arr: Input array. submat: Pointer to the resulting sub-array header. row: Zero-based index of the selected row. start\_row: Zero-based index of the starting row (inclusive) of the span. end\_row: Zero-based index of the ending row (exclusive) of the span. delta\_row: Index step in the row span. That is, the function extracts every `delta_row`-th row from `start_row` and up to (but not including) `end_row`.

The functions `GetRow` and `GetRows` return the header, corresponding to a specified
row/row span of the input array. Note that `GetRow` is a shortcut for cvGetRows:

```
cvGetRow( arr, submat, row ) ~ cvGetRows( arr, submat, row, row + 1, 1 );
```

---

### GetCol, GetCols

Returns array column or column span

```
CvMat* cvGetCol( const CvArr* arr, CvMat* submat, int col );
CvMat* cvGetCols( const CvArr* arr, CvMat* submat, int start_col, int end_col );
```

arr: Input array. submat: Pointer to the resulting sub-array header. col: Zero-based index of the selected column. start\_col: Zero-based index of the starting column (inclusive) of the span. end\_col: Zero-based index of the ending column (exclusive) of the span.

The functions `GetCol` and `GetCols` return the header, corresponding to a specified
column/column span of the input array. Note that `GetCol` is a shortcut for cvGetCols:

```
cvGetCol( arr, submat, col ); // ~ cvGetCols( arr, submat, col, col + 1 );
```

---

### GetDiag

Returns one of array diagonals

```
CvMat* cvGetDiag( const CvArr* arr, CvMat* submat, int diag=0 );
```

arr: Input array. submat: Pointer to the resulting sub-array header. diag: Array diagonal. Zero corresponds to the main diagonal, -1 corresponds to the diagonal above the main etc., 1 corresponds to the diagonal below the main etc.

The function `cvGetDiag` returns the header, corresponding to a specified
diagonal of the input array.

---

### GetSize

Returns size of matrix or image ROI

```
CvSize cvGetSize( const CvArr* arr );
```

arr: array header.

The function `cvGetSize` returns number of rows (CvSize::height) and number of columns
(CvSize::width) of the input matrix or image. In case of image the size of ROI is returned.

---

### InitSparseMatIterator

Initializes sparse array elements iterator

```
CvSparseNode* cvInitSparseMatIterator( const CvSparseMat* mat,
                                       CvSparseMatIterator* mat_iterator );
```

mat: Input array. mat\_iterator: Initialized iterator.

The function `cvInitSparseMatIterator` initializes iterator of sparse array elements and
returns pointer to the first element, or NULL if the array is empty.

---

### GetNextSparseNode

Initializes sparse array elements iterator

```
CvSparseNode* cvGetNextSparseNode( CvSparseMatIterator* mat_iterator );
```

mat\_iterator: Sparse array iterator.

The function `cvGetNextSparseNode` moves iterator to the next sparse matrix element and returns
pointer to it. In the current version there is no any particular order of the elements, because they
are stored in hash table. The sample below demonstrates how to iterate through the sparse matrix:

Using cvInitSparseMatIterator and cvGetNextSparseNode to calculate sum of
floating-point sparse array.

```
    double sum;
    int i, dims = cvGetDims( array );
    CvSparseMatIterator mat_iterator;
    CvSparseNode* node = cvInitSparseMatIterator( array, &mat_iterator );

    for( ; node != 0; node = cvGetNextSparseNode( &mat_iterator ))
    {
        const int* idx = CV_NODE_IDX( array, node ); /* get pointer to the element indices */
        float val = *(float*)CV_NODE_VAL( array, node ); /* get value of the element
                                                          (assume that the type is CV_32FC1) */
        printf( "(" );
        for( i = 0; i < dims; i++ )
            printf( "%4d%s", idx[i], i < dims - 1 "," : "): " );
        printf( "%g\n", val );

        sum += val;
    }

    printf( "\nTotal sum = %g\n", sum );
```

---

### GetElemType

Returns type of array elements

```
int cvGetElemType( const CvArr* arr );
```

arr: Input array.

The functions `GetElemType` returns type of the array elements as it is described in
cvCreateMat discussion:

```
CV_8UC1 ... CV_64FC4
```

---

### GetDims, GetDimSize

Return number of array dimensions and their sizes or the size of particular dimension

```
int cvGetDims( const CvArr* arr, int* sizes=NULL );
int cvGetDimSize( const CvArr* arr, int index );
```

arr: Input array. sizes: Optional output vector of the array dimension sizes. For 2d arrays the number of rows (height) goes first, number of columns (width) next. index: Zero-based dimension index (for matrices 0 means number of rows, 1 means number of columns; for images 0 means height, 1 means width).

The function `cvGetDims` returns number of array dimensions and their sizes.
In case of `IplImage` or CvMat it always returns 2 regardless of number of image/matrix rows.
The function `cvGetDimSize` returns the particular dimension size (number of elements per that dimension).
For example, the following code calculates total number of array elements in two ways:

```
// via cvGetDims()
int sizes[CV_MAX_DIM];
int i, total = 1;
int dims = cvGetDims( arr, size );
for( i = 0; i < dims; i++ )
    total *= sizes[i];

// via cvGetDims() and cvGetDimSize()
int i, total = 1;
int dims = cvGetDims( arr );
for( i = 0; i < dims; i++ )
    total *= cvGetDimsSize( arr, i );
```

---

### Ptr\*D

Return pointer to the particular array element

```
uchar* cvPtr1D( const CvArr* arr, int idx0, int* type=NULL );
uchar* cvPtr2D( const CvArr* arr, int idx0, int idx1, int* type=NULL );
uchar* cvPtr3D( const CvArr* arr, int idx0, int idx1, int idx2, int* type=NULL );
uchar* cvPtrND( const CvArr* arr, const int* idx, int* type=NULL, int create_node=1, unsigned* precalc_hashval=NULL );
```

arr: Input array. idx0: The first zero-based component of the element index idx1: The second zero-based component of the element index idx2: The third zero-based component of the element index idx: Array of the element indices type: Optional output parameter: type of matrix elements create\_node: Optional input parameter for sparse matrices. Non-zero value of the parameter means that the requested element is created if it does not exist already. precalc\_hashval: Optional input parameter for sparse matrices. If the pointer is not NULL, the function does not recalculate the node hash value, but takes it from the specified location. It is useful for speeding up pair-wise operations (TODO: provide an example)

The functions `>cvPtr*D` return pointer to the particular array element.
Number of array dimension should match to the number of indices passed to the function except
for `cvPtr1D` function that can be used for sequential access to 1D, 2D or nD dense arrays.

The functions can be used for sparse arrays as well - if the requested node does not exist they
create it and set it to zero.

All these as well as other functions accessing array elements
(cvGet\*D, cvGetReal\*D,
cvSet\*D, cvSetReal\*D)
raise an error in case if the element index is out of range.

---

### Get\*D

Return the particular array element

```
CvScalar cvGet1D( const CvArr* arr, int idx0 );
CvScalar cvGet2D( const CvArr* arr, int idx0, int idx1 );
CvScalar cvGet3D( const CvArr* arr, int idx0, int idx1, int idx2 );
CvScalar cvGetND( const CvArr* arr, const int* idx );
```

arr: Input array. idx0: The first zero-based component of the element index idx1: The second zero-based component of the element index idx2: The third zero-based component of the element index idx: Array of the element indices

The functions `cvGet*D` return the particular array element. In case of sparse array
the functions return 0 if the requested node does not exist (no new node is created
by the functions)

---

### GetReal\*D

Return the particular element of single-channel array

```
double cvGetReal1D( const CvArr* arr, int idx0 );
double cvGetReal2D( const CvArr* arr, int idx0, int idx1 );
double cvGetReal3D( const CvArr* arr, int idx0, int idx1, int idx2 );
double cvGetRealND( const CvArr* arr, const int* idx );
```

arr: Input array. Must have a single channel. idx0: The first zero-based component of the element index idx1: The second zero-based component of the element index idx2: The third zero-based component of the element index idx: Array of the element indices

The functions `cvGetReal*D` return the particular element of single-channel array.
If the array has multiple channels, runtime error is raised. Note that cvGet\*D function
can be used safely for both single-channel and multiple-channel arrays though they are
a bit slower.

In case of sparse array
the functions return 0 if the requested node does not exist (no new node is created
by the functions)

---

### mGet

Return the particular element of single-channel floating-point matrix

```
double cvmGet( const CvMat* mat, int row, int col );
```

mat: Input matrix. row: The zero-based index of row. col: The zero-based index of column.

The function `cvmGet` is a fast replacement for cvGetReal2D in case of
single-channel floating-point matrices. It is faster because it is inline,
it does less checks for array type and array element type and it
checks for the row and column ranges only in debug mode.

---

### Set\*D

Change the particular array element

```
void cvSet1D( CvArr* arr, int idx0, CvScalar value );
void cvSet2D( CvArr* arr, int idx0, int idx1, CvScalar value );
void cvSet3D( CvArr* arr, int idx0, int idx1, int idx2, CvScalar value );
void cvSetND( CvArr* arr, const int* idx, CvScalar value );
```

arr: Input array. idx0: The first zero-based component of the element index idx1: The second zero-based component of the element index idx2: The third zero-based component of the element index idx: Array of the element indices value: The assigned value

The functions `cvSet*D` assign the new value to the particular element of array.
In case of sparse array the functions create the node if it does not exist yet

---

### SetReal\*D

Change the particular array element

```
void cvSetReal1D( CvArr* arr, int idx0, double value );
void cvSetReal2D( CvArr* arr, int idx0, int idx1, double value );
void cvSetReal3D( CvArr* arr, int idx0, int idx1, int idx2, double value );
void cvSetRealND( CvArr* arr, const int* idx, double value );
```

arr: Input array. idx0: The first zero-based component of the element index idx1: The second zero-based component of the element index idx2: The third zero-based component of the element index idx: Array of the element indices value: The assigned value

The functions `cvSetReal*D` assign the new value to the particular element of single-channel array.
If the array has multiple channels, runtime error is raised. Note that cvSet\*D function
can be used safely for both single-channel and multiple-channel arrays though they are
a bit slower.

In case of sparse array the functions create the node if it does not exist yet

---

### mSet

Return the particular element of single-channel floating-point matrix

```
void cvmSet( CvMat* mat, int row, int col, double value );
```

mat: The matrix. row: The zero-based index of row. col: The zero-based index of column. value: The new value of the matrix element

The function `cvmSet` is a fast replacement for cvSetReal2D in case of
single-channel floating-point matrices. It is faster because it is inline,
it does less checks for array type and array element type and it
checks for the row and column ranges only in debug mode.

---

### ClearND

Clears the particular array element

```
void cvClearND( CvArr* arr, const int* idx );
```

arr: Input array. idx: Array of the element indices

The function cvClearND clears (sets to zero)
the particular element of dense array or deletes the element of sparse array.
If the element does not exists, the function does nothing.

---

## Copying and Filling

---

### Copy

Copies one array to another

```
void cvCopy( const CvArr* src, CvArr* dst, const CvArr* mask=NULL );
```

src: The source array. dst: The destination array. mask: Operation mask, 8-bit single channel array; specifies elements of destination array to be changed.

The function `cvCopy` copies selected elements from input array to output array:

dst(I)=src(I) if mask(I)!=0.

If any of the passed arrays is of `IplImage` type, then its ROI and COI fields are
used. Both arrays must have the same type, the same number of dimensions and the same size.
The function can also copy sparse arrays (mask is not supported in this case).

---

### Set

Sets every element of array to given value

```
void cvSet( CvArr* arr, CvScalar value, const CvArr* mask=NULL );
```

arr: The destination array. value: Fill value. mask: Operation mask, 8-bit single channel array; specifies elements of destination array to be changed.

The function `cvSet` copies scalar `value` to every selected element of the destination
array:

```
arr(I)=value if mask(I)!=0
```

If array `arr` is of `IplImage` type, then is ROI used, but COI must not be
set.

---

### SetZero

Clears the array

```
void cvSetZero( CvArr* arr );
#define cvZero cvSetZero
```

arr: array to be cleared.

The function `cvSetZero` clears the array. In case of dense arrays
(CvMat, CvMatND or IplImage) cvZero(array) is equivalent to cvSet(array,cvScalarAll(0),0),
in case of sparse arrays all the elements are removed.

---

### SetIdentity

Initializes scaled identity matrix

```
void cvSetIdentity( CvArr* mat, CvScalar value=cvRealScalar(1) );
```

arr: The matrix to initialize (not necessarily square). value: The value to assign to the diagonal elements.

The function `cvSetIdentity` initializes scaled identity matrix:

```
arr(i,j)=value if i=j,
       0 otherwise
```

---

### Range

Fills matrix with given range of numbers

```
void cvRange( CvArr* mat, double start, double end );
```

mat: The matrix to initialize. It should be single-channel 32-bit, integer or floating-point. start: The lower inclusive boundary of the range. end: The upper exclusive boundary of the range.

The function `cvRange` initializes the matrix as following:

```
arr(i,j)=(end-start)*(i*cols(arr)+j)/(cols(arr)*rows(arr))
```

For example, the following code will initialize 1D vector with subsequent integer numbers.

```
CvMat* A = cvCreateMat( 1, 10, CV_32S );
cvRange( A, 0, A->cols ); // A will be initialized as [0,1,2,3,4,5,6,7,8,9]
```


---

## Transforms and Permutations

---

### Reshape

Changes shape of matrix/image without copying data

```
CvMat* cvReshape( const CvArr* arr, CvMat* header, int new_cn, int new_rows=0 );
```

arr: Input array. header: Output header to be filled. new\_cn: New number of channels. `new_cn = 0` means that number of channels remains unchanged. new\_rows: New number of rows. `new_rows = 0` means that number of rows remains unchanged unless it needs to be changed according to `new_cn` value. destination array to be changed.

The function `cvReshape` initializes CvMat header so that it points to the same data as
the original array but has different shape - different number of channels,
different number of rows or both.

For example, the following code creates one image buffer and
two image headers, first is for 320x240x3 image and the second is for 960x240x1 image:

```
IplImage* color_img = cvCreateImage( cvSize(320,240), IPL_DEPTH_8U, 3 );
CvMat gray_mat_hdr;
IplImage gray_img_hdr, *gray_img;
cvReshape( color_img, &gray_mat_hdr, 1 );
gray_img = cvGetImage( &gray_mat_hdr, &gray_img_hdr );
```

And the next example converts 3x3 matrix to a single 1x9 vector

```
CvMat* mat = cvCreateMat( 3, 3, CV_32F );
CvMat row_header, *row;
row = cvReshape( mat, &row_header, 0, 1 );
```

---

### ReshapeMatND

Changes shape of multi-dimensional array w/o copying data

```
CvArr* cvReshapeMatND( const CvArr* arr,
                       int sizeof_header, CvArr* header,
                       int new_cn, int new_dims, int* new_sizes );

#define cvReshapeND( arr, header, new_cn, new_dims, new_sizes )   \
      cvReshapeMatND( (arr), sizeof(*(header)), (header),         \
                      (new_cn), (new_dims), (new_sizes))
```

arr: Input array. sizeof\_header: Size of output header to distinguish between IplImage, CvMat and CvMatND output headers. header: Output header to be filled. new\_cn: New number of channels. `new_cn = 0` means that number of channels remains unchanged. new\_dims: New number of dimensions. `new_dims = 0` means that number of dimensions remains the same. new\_sizes: Array of new dimension sizes. Only `new_dims-1` values are used, because the total number of elements must remain the same. Thus, if `new_dims = 1`, `new_sizes` array is not used

The function `cvReshapeMatND` is an advanced version of cvReshape that can work
with multi-dimensional arrays as well (though, it can work with ordinary images and matrices)
and change the number of dimensions. Below are the two samples
from the cvReshape description rewritten using cvReshapeMatND:

```
IplImage* color_img = cvCreateImage( cvSize(320,240), IPL_DEPTH_8U, 3 );
IplImage gray_img_hdr, *gray_img;
gray_img = (IplImage*)cvReshapeND( color_img, &gray_img_hdr, 1, 0, 0 );

...

/* second example is modified to convert 2x2x2 array to 8x1 vector */
int size[] = { 2, 2, 2 };
CvMatND* mat = cvCreateMatND( 3, size, CV_32F );
CvMat row_header, *row;
row = cvReshapeND( mat, &row_header, 0, 1, 0 );
```

---

### Repeat

Fill destination array with tiled source array

```
void cvRepeat( const CvArr* src, CvArr* dst );
```

src: Source array, image or matrix. dst: Destination array, image or matrix.

The function `cvRepeat` fills the destination array with source array tiled:

```
dst(i,j)=src(i mod rows(src), j mod cols(src))
```

So the destination array may be as larger as well as smaller than
the source array.

---

### Flip

Flip a 2D array around vertical, horizontal or both axises

```
void  cvFlip( const CvArr* src, CvArr* dst=NULL, int flip_mode=0);
#define cvMirror cvFlip
```

src: Source array. dst: Destination array. If `dst = NULL` the flipping is done in-place. flip\_mode: Specifies how to flip the array. flip\_mode = 0 means flipping around x-axis, flip\_mode > 0 (e.g. 1) means flipping around y-axis and flip\_mode < 0 (e.g. -1) means flipping around both axises. See also the discussion below for the formulas

The function `cvFlip` flips the array in one of different 3 ways
(row and column indices are 0-based):

```
dst(i,j)=src(rows(src)-i-1,j) if flip_mode = 0
```

```
dst(i,j)=src(i,cols(src1)-j-1) if flip_mode > 0
```

```
dst(i,j)=src(rows(src)-i-1,cols(src)-j-1) if flip_mode < 0
```

The example scenario of the function use are:

- vertical flipping of the image (flip\_mode > 0) to switch between top-left
  and bottom-left image origin, which is typical operation in video processing under Win32 systems.
- horizontal flipping of the image with subsequent horizontal shift and absolute difference calculation
  to check for a vertical-axis symmetry (flip\_mode > 0)
- simultaneous horizontal and vertical flipping of the image with subsequent shift and
  absolute difference calculation to check for a central symmetry (flip\_mode < 0)
- reversing the order of 1d point arrays(flip\_mode > 0)

---

### Split

Divides multi-channel array into several single-channel arrays or extracts
a single channel from the array

```
void cvSplit( const CvArr* src, CvArr* dst0, CvArr* dst1,
              CvArr* dst2, CvArr* dst3 );
#define cvCvtPixToPlane cvSplit
```

src: Source array. dst0...dst3: Destination channels.

The function `cvSplit` divides a multi-channel array into separate single-channel arrays.
Two modes are available for the operation. If the source array has N channels then if
the first N destination channels are not NULL, all they are extracted from the source array,
otherwise if only a single destination channel of the first N is not NULL, this particular channel
is extracted, otherwise an error is raised. Rest of destination channels (beyond the first N)
must always be NULL.
For IplImage cvCopy with COI set can be also used to extract a single channel from the image.

---

### Merge

Composes multi-channel array from several single-channel arrays or inserts a
single channel into the array

```
void cvMerge( const CvArr* src0, const CvArr* src1,
              const CvArr* src2, const CvArr* src3, CvArr* dst );
#define cvCvtPlaneToPix cvMerge
```

src0... src3: Input channels. dst: Destination array.

The function `cvMerge` is the opposite to the previous.
If the destination array has N channels then if
the first N input channels are not NULL, all they are copied to the destination array,
otherwise if only a single source channel of the first N is not NULL, this particular channel is copied
into the destination array, otherwise an error is raised. Rest of source channels (beyond the first N)
must always be NULL.
For IplImage cvCopy with COI set can be also used to insert a single channel into the image.

---

### MixChannels

Copies several channels from input arrays to
certain channels of output arrays

```
void cvMixChannels( const CvArr** src, int src_count,
                    CvArr** dst, int dst_count,
                    const int* from_to, int pair_count );
```

src: The array of input arrays. src\_count: The number of input arrays. dst: The array of output arrays. dst\_count: The number of output arrays. from\_to: The array of pairs of indices of the planes copied. `from_to[k*2]` is the 0-based index of the input plane, and `from_to[k*2+1]` is the index of the output plane, where the continuous numbering of the planes over all the input and over all the output arrays is used. When `from_to[k*2]` is negative, the corresponding output plane is filled with 0's. pair\_count: The number of pairs in `from_to`, or the number of the planes copied.

The function `cvMixChannels` is a generalized form of cvSplit and
cvMerge and some forms of cvCvtColor.
It can be used to change the order of the planes, add/remove alpha
channel, extract or insert a single plane or multiple planes etc.
Below is the example, how to split 4-channel RGBA image into 3-channel BGR (i.e. with R&B swapped) and
separate alpha channel images:

```
    CvMat* rgba = cvCreateMat( 100, 100, CV_8UC4 );
    CvMat* bgr = cvCreateMat( rgba->rows, rgba->cols, CV_8UC3 );
    CvMat* alpha = cvCreateMat( rgba->rows, rgba->cols, CV_8UC1 );
    CvArr* out[] = { bgr, alpha };
    int from_to[] = { 0, 2, 1, 1, 2, 0, 3, 3 };
    cvSet( rgba, cvScalar(1,2,3,4) );
    cvMixChannels( (const CvArr**)&rgba, 1, out, 2, from_to, 4 );
```

---

### RandShuffle

Randomly shuffles the array elements

```
void cvRandShuffle( CvArr* mat, CvRNG* rng, double iter_factor=1. );
```

mat: The input/output matrix. It is shuffled in-place. rng: The Random Number Generator used to shuffle the elements. When the pointer is NULL, a temporary RNG will be created and used. iter\_factor: The relative parameter that characterizes intensity of the shuffling performed. See the description below.

The function `cvRandShuffle` shuffles the matrix by swapping randomly chosen pairs
of the matrix elements on each iteration (where each element may contain several components in case of multi-channel arrays).
The number of iterations (i.e. pairs swapped) is `round(iter_factor*rows(mat)*cols(mat))`, so
`iter_factor=0` means that no shuffling is done,
`iter_factor=1` means that the function swaps
`rows(mat)*cols(mat)` random pairs etc.

---

## Arithmetic, Logic and Comparison

---

### LUT

Performs look-up table transform of array

```
void cvLUT( const CvArr* src, CvArr* dst, const CvArr* lut );
```

src: Source array of 8-bit elements. dst: Destination array of arbitrary depth and of the same number of channels as the source array. lut: Look-up table of 256 elements; should have the same depth as the destination array. In case of multi-channel source and destination arrays, the table should either have a single-channel (in this case the same table is used for all channels), or the same number of channels as the source/destination array.

The function `cvLUT` fills the destination array with values
from the look-up table. Indices of the entries are taken from the source array. That is, the
function processes each element of `src` as following:

```
dst(I)=lut[src(I)+DELTA]
```

where `DELTA=0` if `src` has depth `CV_8U`, and
`DELTA=128` if `src` has depth `CV_8S`.

---

### ConvertScale

Converts one array to another with optional linear transformation

```
void cvConvertScale( const CvArr* src, CvArr* dst, double scale=1, double shift=0 );

#define cvCvtScale cvConvertScale
#define cvScale  cvConvertScale
#define cvConvert( src, dst )  cvConvertScale( (src), (dst), 1, 0 )
```

src: Source array. dst: Destination array. scale: Scale factor. shift: Value added to the scaled source array elements.

The function `cvConvertScale` has several different purposes and thus has several synonyms.
It copies one array to another with optional scaling, which is performed first, and/or optional type conversion, performed after:

```
dst(I)=src(I)*scale + (shift,shift,...)
```

All the channels of multi-channel arrays are processed independently.

The type conversion is done with rounding and saturation, that is if a result of scaling + conversion can not
be represented exactly by a value of destination array element type, it is set to the nearest
representable value on the real axis.

In case of `scale=1, shift=0` no pre-scaling is done. This is a specially optimized case and it
has the appropriate cvConvert synonym. If source and destination array types have
equal types, this is also a special case that can be used to scale and shift a matrix or an image and
that fits to cvScale synonym.

---

### ConvertScaleAbs

Converts input array elements to 8-bit unsigned integer another with optional linear transformation

```
void cvConvertScaleAbs( const CvArr* src, CvArr* dst, double scale=1, double shift=0 );
#define cvCvtScaleAbs cvConvertScaleAbs
```

src: Source array. dst: Destination array (should have 8u depth). scale: ScaleAbs factor. shift: Value added to the scaled source array elements.

The function `cvConvertScaleAbs` is similar to the previous one, but it stores absolute values
of the conversion results:

```
dst(I)=abs(src(I)*scale + (shift,shift,...))
```

The function supports only destination arrays of 8u (8-bit unsigned integers) type, for
other types the function can be emulated by combination of cvConvertScale and cvAbs
functions.

---

### Add

Computes per-element sum of two arrays

```
void cvAdd( const CvArr* src1, const CvArr* src2, CvArr* dst, const CvArr* mask=NULL );
```

src1: The first source array. src2: The second source array. dst: The destination array. mask: Operation mask, 8-bit single channel array; specifies elements of destination array to be changed.

The function `cvAdd` adds one array to another one:

```
dst(I)=src1(I)+src2(I) if mask(I)!=0
```

All the arrays must have the same type, except the mask, and the same size (or ROI size)

---

### AddS

Computes sum of array and scalar

```
void cvAddS( const CvArr* src, CvScalar value, CvArr* dst, const CvArr* mask=NULL );
```

src: The source array. value: Added scalar. dst: The destination array. mask: Operation mask, 8-bit single channel array; specifies elements of destination array to be changed.

The function `cvAddS` adds scalar `value` to every element in the source array `src1` and
stores the result in `dst`

```
dst(I)=src(I)+value if mask(I)!=0
```

All the arrays must have the same type, except the mask, and the same size (or ROI size)

---

### AddWeighted

Computes weighted sum of two arrays

```
void  cvAddWeighted( const CvArr* src1, double alpha,
                     const CvArr* src2, double beta,
                     double gamma, CvArr* dst );
```

src1: The first source array. alpha: Weight of the first array elements. src2: The second source array. beta: Weight of the second array elements. dst: The destination array. gamma: Scalar, added to each sum.

The function `cvAddWeighted` calculated weighted sum
of two arrays as following:

```
dst(I)=src1(I)*alpha+src2(I)*beta+gamma
```

All the arrays must have the same type and the same size (or ROI size)

---

### Sub

Computes per-element difference between two arrays

```
void cvSub( const CvArr* src1, const CvArr* src2, CvArr* dst, const CvArr* mask=NULL );
```

src1: The first source array. src2: The second source array. dst: The destination array. mask: Operation mask, 8-bit single channel array; specifies elements of destination array to be changed.

The function `cvSub` subtracts one array from another one:

```
dst(I)=src1(I)-src2(I) if mask(I)!=0
```

All the arrays must have the same type, except the mask, and the same size (or ROI size)

---

### SubS

Computes difference between array and scalar

```
void cvSubS( const CvArr* src, CvScalar value, CvArr* dst, const CvArr* mask=NULL );
```

src: The source array. value: Subtracted scalar. dst: The destination array. mask: Operation mask, 8-bit single channel array; specifies elements of destination array to be changed.

The function `cvSubS` subtracts a scalar from every element of the source array:

```
dst(I)=src(I)-value if mask(I)!=0
```

All the arrays must have the same type, except the mask, and the same size (or ROI size)

---

### SubRS

Computes difference between scalar and array

```
void cvSubRS( const CvArr* src, CvScalar value, CvArr* dst, const CvArr* mask=NULL );
```

src: The first source array. value: Scalar to subtract from. dst: The destination array. mask: Operation mask, 8-bit single channel array; specifies elements of destination array to be changed.

The function `cvSubRS` subtracts every element of source array from a scalar:

```
dst(I)=value-src(I) if mask(I)!=0
```

All the arrays must have the same type, except the mask, and the same size (or ROI size)

---

### Mul

Calculates per-element product of two arrays

```
void cvMul( const CvArr* src1, const CvArr* src2, CvArr* dst, double scale=1 );
```

src1: The first source array. src2: The second source array. dst: The destination array. scale: Optional scale factor

The function `cvMul` calculates per-element product of two arrays:

```
dst(I)=scale•src1(I)•src2(I)
```

All the arrays must have the same type, and the same size (or ROI size)

---

### Div

Performs per-element division of two arrays

```
void cvDiv( const CvArr* src1, const CvArr* src2, CvArr* dst, double scale=1 );
```

src1: The first source array. If the pointer is NULL, the array is assumed to be all 1’s. src2: The second source array. dst: The destination array. scale: Optional scale factor

The function `cvDiv` divides one array by another:

```
dst(I)=scale•src1(I)/src2(I), if src1!=NULL
dst(I)=scale/src2(I),      if src1=NULL
```

All the arrays must have the same type, and the same size (or ROI size)

---

### And

Calculates per-element bit-wise conjunction of two arrays

```
void cvAnd( const CvArr* src1, const CvArr* src2, CvArr* dst, const CvArr* mask=NULL );
```

src1: The first source array. src2: The second source array. dst: The destination array. mask: Operation mask, 8-bit single channel array; specifies elements of destination array to be changed.

The function `cvAnd` calculates per-element bit-wise logical conjunction of two arrays:

```
dst(I)=src1(I)&src2(I) if mask(I)!=0
```

In the case of floating-point arrays their bit representations are used for the
operation. All the arrays must have the same type, except the mask, and the same size

---

### AndS

Calculates per-element bit-wise conjunction of array and scalar

```
void cvAndS( const CvArr* src, CvScalar value, CvArr* dst, const CvArr* mask=NULL );
```

src: The source array. value: Scalar to use in the operation. dst: The destination array. mask: Operation mask, 8-bit single channel array; specifies elements of destination array to be changed.

The function AndS calculates per-element bit-wise conjunction of array and scalar:

```
dst(I)=src(I)&value if mask(I)!=0
```

Prior to the actual operation the scalar is converted to the same type as
the arrays. In the case of floating-point arrays their bit representations are used for the
operation. All the arrays must have the same type, except the mask, and the same size

The following sample demonstrates how to calculate absolute value of floating-point array elements
by clearing the most-significant bit:

```
float a[] = { -1, 2, -3, 4, -5, 6, -7, 8, -9 };
CvMat A = cvMat( 3, 3, CV_32F, &a );
int i, abs_mask = 0x7fffffff;
cvAndS( &A, cvRealScalar(*(float*)&abs_mask), &A, 0 );
for( i = 0; i < 9; i++ )
    printf("%.1f ", a[i] );
```

The code should print:

```
1.0 2.0 3.0 4.0 5.0 6.0 7.0 8.0 9.0
```

---

### Or

Calculates per-element bit-wise disjunction of two arrays

```
void cvOr( const CvArr* src1, const CvArr* src2, CvArr* dst, const CvArr* mask=NULL );
```

src1: The first source array. src2: The second source array. dst: The destination array. mask: Operation mask, 8-bit single channel array; specifies elements of destination array to be changed.

The function `cvOr` calculates per-element bit-wise disjunction of two arrays:

```
dst(I)=src1(I)|src2(I)
```

In the case of floating-point arrays their bit representations are used for the
operation. All the arrays must have the same type, except the mask, and the same size

---

### OrS

Calculates per-element bit-wise disjunction of array and scalar

```
void cvOrS( const CvArr* src, CvScalar value, CvArr* dst, const CvArr* mask=NULL );
```

src1: The source array. value: Scalar to use in the operation. dst: The destination array. mask: Operation mask, 8-bit single channel array; specifies elements of destination array to be changed.

The function OrS calculates per-element bit-wise disjunction of array and scalar:

```
dst(I)=src(I)|value if mask(I)!=0
```

Prior to the actual operation the scalar is converted to the same type as
the arrays. In the case of floating-point arrays their bit representations are used for the
operation. All the arrays must have the same type, except the mask, and the same size

---

### Xor

Performs per-element bit-wise "exclusive or" operation on two arrays

```
void cvXor( const CvArr* src1, const CvArr* src2, CvArr* dst, const CvArr* mask=NULL );
```

src1: The first source array. src2: The second source array. dst: The destination array. mask: Operation mask, 8-bit single channel array; specifies elements of destination array to be changed.

The function `cvXor` calculates per-element bit-wise logical conjunction of two arrays:

```
dst(I)=src1(I)^src2(I) if mask(I)!=0
```

In the case of floating-point arrays their bit representations are used for the
operation. All the arrays must have the same type, except the mask, and the same size

---

### XorS

Performs per-element bit-wise "exclusive or" operation on array and scalar

```
void cvXorS( const CvArr* src, CvScalar value, CvArr* dst, const CvArr* mask=NULL );
```

src: The source array. value: Scalar to use in the operation. dst: The destination array. mask: Operation mask, 8-bit single channel array; specifies elements of destination array to be changed.

The function XorS calculates per-element bit-wise conjunction of array and scalar:

```
dst(I)=src(I)^value if mask(I)!=0
```

Prior to the actual operation the scalar is converted to the same type as
the arrays. In the case of floating-point arrays their bit representations are used for the
operation. All the arrays must have the same type, except the mask, and the same size

The following sample demonstrates how to conjugate complex vector
by switching the most-significant bit of imaging part:

```
float a[] = { 1, 0, 0, 1, -1, 0, 0, -1 }; /* 1, j, -1, -j */
CvMat A = cvMat( 4, 1, CV_32FC2, &a );
int i, neg_mask = 0x80000000;
cvXorS( &A, cvScalar( 0, *(float*)&neg_mask, 0, 0 ), &A, 0 );
for( i = 0; i < 4; i++ )
    printf("(%.1f, %.1f) ", a[i*2], a[i*2+1] );
```

The code should print:

```
(1.0,0.0) (0.0,-1.0) (-1.0,0.0) (0.0,1.0)
```

---

### Not

Performs per-element bit-wise inversion of array elements

```
void cvNot( const CvArr* src, CvArr* dst );
```

src1: The source array. dst: The destination array.

The function Not inverses every bit of every array element:

```
dst(I)=~src(I)
```

---

### Cmp

Performs per-element comparison of two arrays

```
void cvCmp( const CvArr* src1, const CvArr* src2, CvArr* dst, int cmp_op );
```

src1: The first source array. src2: The second source array. Both source array must have a single channel. dst: The destination array, must have 8u or 8s type. cmp\_op: The flag specifying the relation between the elements to be checked: CV\_CMP\_EQ - src1(I) "equal to" src2(I) CV\_CMP\_GT - src1(I) "greater than" src2(I) CV\_CMP\_GE - src1(I) "greater or equal" src2(I) CV\_CMP\_LT - src1(I) "less than" src2(I) CV\_CMP\_LE - src1(I) "less or equal" src2(I) CV\_CMP\_NE - src1(I) "not equal to" src2(I)

The function `cvCmp` compares the corresponding elements of two arrays and
fills the destination mask array:

```
dst(I)=src1(I) op src2(I),
```

where `op` is '=', '>', '>=', '<', '<=' or '!='.

`dst(I)` is set to 0xff (all '1'-bits) if the particular relation between the elements
is true and 0 otherwise.
All the arrays must have the same type, except the destination, and the same size (or ROI size)

---

### CmpS

Performs per-element comparison of array and scalar

```
void cvCmpS( const CvArr* src, double value, CvArr* dst, int cmp_op );
```

src: The source array, must have a single channel. value: The scalar value to compare each array element with. dst: The destination array, must have 8u or 8s type. cmp\_op: The flag specifying the relation between the elements to be checked: CV\_CMP\_EQ - src1(I) "equal to" value CV\_CMP\_GT - src1(I) "greater than" value CV\_CMP\_GE - src1(I) "greater or equal" value CV\_CMP\_LT - src1(I) "less than" value CV\_CMP\_LE - src1(I) "less or equal" value CV\_CMP\_NE - src1(I) "not equal" value

The function `cvCmpS` compares the corresponding elements of array and scalar
and fills the destination mask array:

```
dst(I)=src(I) op scalar,
```

where `op` is '=', '>', '>=', '<', '<=' or '!='.

`dst(I)` is set to 0xff (all '1'-bits) if the particular relation between the elements
is true and 0 otherwise.
All the arrays must have the same size (or ROI size)

---

### InRange

Checks that array elements lie between elements of two other arrays

```
void cvInRange( const CvArr* src, const CvArr* lower, const CvArr* upper, CvArr* dst );
```

src: The first source array. lower: The inclusive lower boundary array. upper: The exclusive upper boundary array. dst: The destination array, must have 8u or 8s type.

The function `cvInRange` does the range check for every element of the input array:

```
dst(I)=lower(I)0 <= src(I)0 < upper(I)0
```

for single-channel arrays,

```
dst(I)=lower(I)0 <= src(I)0 < upper(I)0 &&
     lower(I)1 <= src(I)1 < upper(I)1
```

for two-channel arrays etc.

`dst(I)` is set to 0xff (all '1'-bits) if `src(I)` is within the range and 0 otherwise.
All the arrays must have the same type, except the destination, and the same size (or ROI size)

---

### InRangeS

Checks that array elements lie between two scalars

```
void cvInRangeS( const CvArr* src, CvScalar lower, CvScalar upper, CvArr* dst );
```

src: The first source array. lower: The inclusive lower boundary. upper: The exclusive upper boundary. dst: The destination array, must have 8u or 8s type.

The function `cvInRangeS` does the range check for every element of the input array:

```
dst(I)=lower0 <= src(I)0 < upper0
```

for a single-channel array,

```
dst(I)=lower0 <= src(I)0 < upper0 &&
     lower1 <= src(I)1 < upper1
```

for a two-channel array etc.

`dst(I)` is set to 0xff (all '1'-bits) if `src(I)` is within the range and 0 otherwise.
All the arrays must have the same size (or ROI size)

---

### Max

Finds per-element maximum of two arrays

```
void cvMax( const CvArr* src1, const CvArr* src2, CvArr* dst );
```

src1: The first source array. src2: The second source array. dst: The destination array.

The function `cvMax` calculates per-element maximum of two arrays:

```
dst(I)=max(src1(I), src2(I))
```

All the arrays must have a single channel, the same data type
and the same size (or ROI size).

---

### MaxS

Finds per-element maximum of array and scalar

```
void cvMaxS( const CvArr* src, double value, CvArr* dst );
```

src: The first source array. value: The scalar value. dst: The destination array.

The function `cvMaxS` calculates per-element maximum of array and scalar:

```
dst(I)=max(src(I), value)
```

All the arrays must have a single channel, the same data type
and the same size (or ROI size).

---

### Min

Finds per-element minimum of two arrays

```
void cvMin( const CvArr* src1, const CvArr* src2, CvArr* dst );
```

src1: The first source array. src2: The second source array. dst: The destination array.

The function `cvMin` calculates per-element minimum of two arrays:

```
dst(I)=min(src1(I),src2(I))
```

All the arrays must have a single channel, the same data type
and the same size (or ROI size).

---

### MinS

Finds per-element minimum of array and scalar

```
void cvMinS( const CvArr* src, double value, CvArr* dst );
```

src: The first source array. value: The scalar value. dst: The destination array.

The function `cvMinS` calculates minimum of array and scalar:

```
dst(I)=min(src(I), value)
```

All the arrays must have a single channel, the same data type
and the same size (or ROI size).

---

### AbsDiff

Calculates absolute difference between two arrays

```
void cvAbsDiff( const CvArr* src1, const CvArr* src2, CvArr* dst );
```

src1: The first source array. src2: The second source array. dst: The destination array.

The function `cvAbsDiff` calculates absolute difference between two arrays.

```
dst(I)c = abs(src1(I)c - src2(I)c).
```

All the arrays must have the same data type and the same size (or ROI size).

---

### AbsDiffS

Calculates absolute difference between array and scalar

```
void cvAbsDiffS( const CvArr* src, CvArr* dst, CvScalar value );
#define cvAbs(src, dst) cvAbsDiffS(src, dst, cvScalarAll(0))
```

src: The source array. dst: The destination array. value: The scalar.

The function `cvAbsDiffS` calculates absolute difference between array and scalar.

```
dst(I)c = abs(src(I)c - valuec).
```

All the arrays must have the same data type and the same size (or ROI size).

---

## Statistics

---

### CountNonZero

Counts non-zero array elements

```
int cvCountNonZero( const CvArr* arr );
```

arr: The array, must be single-channel array or multi-channel image with COI set.

The function `cvCountNonZero` returns the number of non-zero elements in src1:

```
result = sumI arr(I)!=0
```

In case of `IplImage` both ROI and COI are supported.

---

### Sum

Summarizes array elements

```
CvScalar cvSum( const CvArr* arr );
```

arr: The array.

The function `cvSum` calculates sum `S` of array elements, independently for each channel:

```
Sc = sumI arr(I)c
```

If the array is `IplImage` and COI is set, the function processes the selected channel only
and stores the sum to the first scalar component (S_0_).

---

### Avg

Calculates average (mean) of array elements

```
CvScalar cvAvg( const CvArr* arr, const CvArr* mask=NULL );
```

arr: The array. mask: The optional operation mask.

The function `cvAvg` calculates the average value `M` of array elements, independently for each channel:

```
N = sumI mask(I)!=0

Mc = 1/N • sumI,mask(I)!=0 arr(I)c
```

If the array is `IplImage` and COI is set, the function processes the selected channel only
and stores the average to the first scalar component (S_0_).

---

### AvgSdv

Calculates average (mean) of array elements

```
void cvAvgSdv( const CvArr* arr, CvScalar* mean, CvScalar* std_dev, const CvArr* mask=NULL );
```

arr: The array. mean: Pointer to the mean value, may be NULL if it is not needed. std\_dev: Pointer to the standard deviation. mask: The optional operation mask.

The function `cvAvgSdv` calculates the average value and
standard deviation of array elements, independently for each channel:

```
N = sumI mask(I)!=0

meanc = 1/N • sumI,mask(I)!=0 arr(I)c

std_devc = sqrt(1/N • sumI,mask(I)!=0 (arr(I)c - Mc)2)
```

If the array is `IplImage` and COI is set, the function processes the selected channel only
and stores the average and standard deviation to the first components of output scalars (M_0_
and S_0_).

---

### MinMaxLoc

Finds global minimum and maximum in array or subarray

```
void cvMinMaxLoc( const CvArr* arr, double* min_val, double* max_val,
                  CvPoint* min_loc=NULL, CvPoint* max_loc=NULL, const CvArr* mask=NULL );
```

arr: The source array, single-channel or multi-channel with COI set. min\_val: Pointer to returned minimum value. max\_val: Pointer to returned maximum value. min\_loc: Pointer to returned minimum location. max\_loc: Pointer to returned maximum location. mask: The optional mask that is used to select a subarray.

The function `MinMaxLoc` finds minimum and maximum element values and their
positions. The extremums are searched over the whole array, selected `ROI` (in case
of `IplImage`) or, if `mask` is not `NULL`, in the specified array region.
If the array has more than one channel, it must be `IplImage` with `COI` set.
In case if multi-dimensional arrays `min_loc->x` and `max_loc->x` will contain raw (linear)
positions of the extremums.

---

### Norm

Calculates absolute array norm, absolute difference norm or relative difference norm

```
double cvNorm( const CvArr* arr1, const CvArr* arr2=NULL, int norm_type=CV_L2, const CvArr* mask=NULL );
```

arr1: The first source image. arr2: The second source image. If it is NULL, the absolute norm of `arr1` is calculated, otherwise absolute or relative norm of `arr1`-`arr2` is calculated. normType: Type of norm, see the discussion. mask: The optional operation mask.

The function `cvNorm` calculates the absolute norm of `arr1` if `arr2` is NULL:

```
norm = ||arr1||C = maxI abs(arr1(I)),  if normType = CV_C

norm = ||arr1||L1 = sumI abs(arr1(I)),  if normType = CV_L1

norm = ||arr1||L2 = sqrt( sumI arr1(I)2),  if normType = CV_L2
```

And the function calculates absolute or relative difference norm if `arr2` is not NULL:

```
norm = ||arr1-arr2||C = maxI abs(arr1(I)-arr2(I)),  if normType = CV_C

norm = ||arr1-arr2||L1 = sumI abs(arr1(I)-arr2(I)),  if normType = CV_L1

norm = ||arr1-arr2||L2 = sqrt( sumI (arr1(I)-arr2(I))2 ),  if normType = CV_L2

or

norm = ||arr1-arr2||C/||arr2||C, if normType = CV_RELATIVE_C

norm = ||arr1-arr2||L1/||arr2||L1, if normType = CV_RELATIVE_L1

norm = ||arr1-arr2||L2/||arr2||L2, if normType = CV_RELATIVE_L2
```

The function `Norm` returns the calculated norm.
The multiple-channel array are treated as single-channel, that is, the results for all channels
are combined.

---

### Reduce

Reduces matrix to a vector

```
void cvReduce( const CvArr* src, CvArr* dst, int op=CV_REDUCE_SUM );
```

src: The input matrix. dst: The output single-row/single-column vector that accumulates somehow all the matrix rows/columns. dim: The dimension index along which the matrix is reduce. 0 means that the matrix is reduced to a single row, 1 means that the matrix is reduced to a single column. -1 means that the dimension is chosen automatically by analysing the `dst` size. op: The reduction operation. It can take of the following values: `CV_REDUCE_SUM` - the output is the sum of all the matrix rows/columns. `CV_REDUCE_AVG` - the output is the mean vector of all the matrix rows/columns. `CV_REDUCE_MAX` - the output is the maximum (column/row-wise) of all the matrix rows/columns. `CV_REDUCE_MIN` - the output is the minimum (column/row-wise) of all the matrix rows/columns.

The function `cvReduce` reduces matrix to a vector by treating the matrix rows/columns as a set
of 1D vectors and performing the specified operation on the vectors until a single row/column is obtained.
For example, the function can be used to compute horizontal and vertical projections of an raster image.
In case of `CV_REDUCE_SUM` and `CV_REDUCE_AVG` the output may have a larger
element bit-depth to preserve accuracy. And multi-channel arrays are also supported in these two reduction modes.


---

## Linear Algebra

---

### DotProduct

Calculates dot product of two arrays in Euclidean metrics

```
double cvDotProduct( const CvArr* src1, const CvArr* src2 );
```

src1: The first source array. src2: The second source array.

The function `cvDotProduct` calculates and returns the Euclidean dot product of two
arrays.

```
src1•src2 = sumI(src1(I)*src2(I))
```

In case of multiple channel arrays the results for all channels are
accumulated. In particular, `cvDotProduct(a,a)`,
where `a` is a complex vector, will return `||a||2`.
The function can process multi-dimensional arrays, row by row, layer by layer and so on.

---

### Normalize

Normalizes array to a certain norm or value range

```
void cvNormalize( const CvArr* src, CvArr* dst,
                  double a=1, double b=0, int norm_type=CV_L2,
                  const CvArr* mask=NULL );
```

src: The input array. dst: The output array; in-place operation is supported. a: The minimum/maximum value of the output array or the norm of output array. b: The maximum/minimum value of the output array. norm\_type: The normalization type. It can take one of the following values: `CV_C` - the C-norm (maximum of absolute values) of the array is normalized. `CV_L1` - the L_1_-norm (sum of absolute values) of the array is normalized. `CV_L2` - the (Euclidean) L_2_-norm of the array is normalized. `CV_MINMAX` - the array values are scaled and shifted to the specified range. mask: The operation mask. Makes the function consider and normalize only certain array elements.

The function `cvNormalize` normalizes the input array so that it's norm or value
range takes the certain value(s).

When `norm_type==CV_MINMAX`:

```
    dst(i,j)=(src(i,j)-min(src))*(b'-a')/(max(src)-min(src)) + a',  if mask(i,j)!=0
    dst(i,j)=src(i,j)  otherwise
```

where `b'=MAX(a,b)`, `a'=MIN(a,b)`;  
`min(src)` and `max(src)` are the global minimum and maximum, respectively,
of the input array, computed over the whole array or the specified subset of it.

When `norm_type!=CV_MINMAX`:

```
    dst(i,j)=src(i,j)*a/cvNorm(src,0,norm_type,mask), if mask(i,j)!=0
    dst(i,j)=src(i,j)  otherwise
```

Here is the short example:

```
float v[3] = { 1, 2, 3 };
CvMat V = cvMat( 1, 3, CV_32F, v );

// make vector v unit-length;
// equivalent to
//   for(int i=0;i<3;i++) v[i]/=sqrt(v[0]*v[0]+v[1]*v[1]+v[2]*v[2]);
cvNormalize( &V, &V );
```

---

### CrossProduct

Calculates cross product of two 3D vectors

```
void cvCrossProduct( const CvArr* src1, const CvArr* src2, CvArr* dst );
```

src1: The first source vector. src2: The second source vector. dst: The destination vector.

The function `cvCrossProduct` calculates the cross product of two 3D vectors:

```
dst = src1 × src2, (dst1 = src12src23 - src13src22 , dst2 = src13src21 - src11src23 , dst3 = src11src22 - src12src21).
```

---

### ScaleAdd

Calculates sum of scaled array and another array

```
void cvScaleAdd( const CvArr* src1, CvScalar scale, const CvArr* src2, CvArr* dst );
#define cvMulAddS cvScaleAdd
```

src1: The first source array. scale: Scale factor for the first array. src2: The second source array. dst: The destination array

The function `cvScaleAdd` calculates sum of scaled array and another array:

```
dst(I)=src1(I)*scale + src2(I)
```

All array parameters should have the same type and the same size.

---

### GEMM

Performs generalized matrix multiplication

```
void  cvGEMM( const CvArr* src1, const CvArr* src2, double alpha,
              const CvArr* src3, double beta, CvArr* dst, int tABC=0 );
#define cvMatMulAdd( src1, src2, src3, dst ) cvGEMM( src1, src2, 1, src3, 1, dst, 0 )
#define cvMatMul( src1, src2, dst ) cvMatMulAdd( src1, src2, 0, dst )
```

src1: The first source array. src2: The second source array. src3: The third source array (shift). Can be NULL, if there is no shift. dst: The destination array. tABC: The operation flags that can be 0 or combination of the following values: CV\_GEMM\_A\_T - transpose src1 CV\_GEMM\_B\_T - transpose src2 CV\_GEMM\_C\_T - transpose src3 for example, CV\_GEMM\_A\_T+CV\_GEMM\_C\_T corresponds to ``` alpha*src1T*src2 + beta*srcT ```

The function `cvGEMM` performs generalized matrix multiplication:

```
dst = alpha*op(src1)*op(src2) + beta*op(src3), where op(X) is X or XT
```

All the matrices should have the same data type and the coordinated sizes.
Real or complex floating-point matrices are supported

---

### Transform

Performs matrix transform of every array element

```
void cvTransform( const CvArr* src, CvArr* dst, const CvMat* transmat, const CvMat* shiftvec=NULL );
```

src: The first source array. dst: The destination array. transmat: Transformation matrix. shiftvec: Optional shift vector.

The function `cvTransform` performs matrix transformation of every element of array `src`
and stores the results in `dst`:

```
dst(I)=transmat*src(I) + shiftvec   or   dst(I)k=sumj(transmat(k,j)*src(I)j) + shiftvec(k)
```

That is every element of `N`-channel array `src` is considered as `N`-element vector, which is
transformed using matrix `M`×`N` matrix `transmat` and shift vector `shiftvec`
into an element of `M`-channel array `dst`.
There is an option to embed `shiftvec` into `transmat`. In this case `transmat` should be
`M`×`N+1` matrix and the right-most column is treated as the shift vector.

Both source and destination arrays should have the same depth and the same size or selected ROI
size. `transmat` and `shiftvec` should be real floating-point matrices.

The function may be used for geometrical transformation of `N`D point set,
arbitrary linear color space transformation, shuffling the channels etc.

---

### PerspectiveTransform

Performs perspective matrix transform of vector array

```
void cvPerspectiveTransform( const CvArr* src, CvArr* dst, const CvMat* mat );
```

src: The source three-channel floating-point array. dst: The destination three-channel floating-point array. mat: 3×3 or 4×4 transformation matrix.

The function `cvPerspectiveTransform` transforms every element of `src`
(by treating it as 2D or 3D vector) in the following way:

```
(x, y, z) -> (x’/w, y’/w, z’/w) or
(x, y) -> (x’/w, y’/w),

where
(x’, y’, z’, w’) = mat4x4*(x, y, z, 1) or
(x’, y’, w’) = mat3x3*(x, y, 1)

and w = w’   if w’!=0,
        inf  otherwise
```

---

### MulTransposed

Calculates product of array and transposed array

```
void cvMulTransposed( const CvArr* src, CvArr* dst, int order, const CvArr* delta=NULL );
```

src: The source matrix. dst: The destination matrix. order: Order of multipliers. delta: An optional array, subtracted from `src` before multiplication.

The function `cvMulTransposed` calculates the product of src and its transposition.

The function evaluates

```
dst=(src-delta)*(src-delta)T
```

if order=0, and

```
dst=(src-delta)T*(src-delta)
```

otherwise.

---

### Trace

Returns trace of matrix

```
CvScalar cvTrace( const CvArr* mat );
```

mat: The source matrix.

The function `cvTrace` returns sum of diagonal elements of the matrix `src1`.

```
tr(src1)=sumimat(i,i)
```

---

### Transpose

Transposes matrix

```
void cvTranspose( const CvArr* src, CvArr* dst );
#define cvT cvTranspose
```

src: The source matrix. dst: The destination matrix.

The function `cvTranspose` transposes matrix `src1`:

```
dst(i,j)=src(j,i)
```

Note that no complex conjugation is done in case of complex matrix. Conjugation
should be done separately: look at the sample code in cvXorS for example

---

### Det

Returns determinant of matrix

```
double cvDet( const CvArr* mat );
```

mat: The source matrix.

The function `cvDet` returns determinant of the square matrix `mat`.
The direct method is used for small matrices and Gaussian elimination is used for larger matrices.
For symmetric positive-determined matrices it is also possible to run SVD
with `U=V=NULL` and then calculate determinant as a product of the diagonal elements of `W`

---

### Invert

Finds inverse or pseudo-inverse of matrix

```
double cvInvert( const CvArr* src, CvArr* dst, int method=CV_LU );
#define cvInv cvInvert
```

src: The source matrix. dst: The destination matrix. method: Inversion method: CV\_LU - Gaussian elimination with optimal pivot element chose CV\_SVD - Singular value decomposition (SVD) method CV\_SVD\_SYM - SVD method for a symmetric positively-defined matrix

The function `cvInvert` inverts matrix `src1` and stores the result in `src2`

In case of `LU` method the function returns `src1` determinant (src1 must be square).
If it is 0, the matrix is not inverted and `src2` is filled with zeros.

In case of `SVD` methods the function returns the inverted condition number of `src1`
(ratio of the smallest singular value to the largest singular value) and 0 if `src1` is all zeros.
The SVD methods calculate a pseudo-inverse matrix if `src1` is singular

---

### Solve

Solves linear system or least-squares problem

```
int cvSolve( const CvArr* A, const CvArr* B, CvArr* X, int method=CV_LU );
```

A: The source matrix. B: The right-hand part of the linear system. X: The output solution. method: The solution (matrix inversion) method: CV\_LU - Gaussian elimination with optimal pivot element chose CV\_SVD - Singular value decomposition (SVD) method CV\_SVD\_SYM - SVD method for a symmetric positively-defined matrix.

The function `cvSolve` solves linear system or least-squares problem (the latter
is possible with SVD methods):

```
dst = arg minX||A*X-B||
```

If `CV_LU` method is used, the function returns 1 if `src1` is non-singular and
0 otherwise, in the latter case `dst` is not valid

---

### SVD

Performs singular value decomposition of real floating-point matrix

```
void cvSVD( CvArr* A, CvArr* W, CvArr* U=NULL, CvArr* V=NULL, int flags=0 );
```

A: Source `M`×`N` matrix. W: Resulting singular value matrix (`M`×`N` or `N`×`N`) or vector (`N`×`1`). U: Optional left orthogonal matrix (`M`×`M` or `M`×`N`). If CV\_SVD\_U\_T is specified, the number of rows and columns in the sentence above should be swapped. V: Optional right orthogonal matrix (`N`×`N`) flags: Operation flags; can be 0 or combination of the following values: - `CV_SVD_MODIFY_A` enables modification of matrix `src1` during the operation. It speeds up the processing.- `CV_SVD_U_T` means that the transposed matrix `U` is returned. Specifying the flag speeds up the processing.- `CV_SVD_V_T` means that the transposed matrix `V` is returned. Specifying the flag speeds up the processing.

The function `cvSVD` decomposes matrix `A` into a product of a diagonal matrix and two
orthogonal matrices:

```
A=U*W*VT
```

Where `W` is diagonal matrix of singular values that can be coded as a 1D
vector of singular values and `U` and `V`. All the singular values are non-negative and sorted (together with `U` and
and `V` columns) in descending order.

SVD algorithm is numerically robust and its typical applications include:

- accurate eigenvalue problem solution when matrix `A` is square, symmetric and positively
  defined matrix, for example, when it is a covariation matrix.
  `W` in this case will be a vector of eigenvalues, and `U`=`V` is matrix of
  eigenvectors (thus, only one of `U` or `V` needs to be calculated if
  the eigenvectors are required)
- accurate solution of poor-conditioned linear systems
- least-squares solution of overdetermined linear systems. This and previous is done by cvSolve
  function with `CV_SVD` method
- accurate calculation of different matrix characteristics such as rank
  (number of non-zero singular values),
  condition number (ratio of the largest singular value to the smallest one),
  determinant (absolute value of determinant is equal to the product of singular values).
  All the things listed in this item do not require calculation of `U`
  and `V` matrices.

---

### SVBkSb

Performs singular value back substitution

```
void  cvSVBkSb( const CvArr* W, const CvArr* U, const CvArr* V,
                const CvArr* B, CvArr* X, int flags );
```

W: Matrix or vector of singular values. U: Left orthogonal matrix (transposed, perhaps) V: Right orthogonal matrix (transposed, perhaps) B: The matrix to multiply the pseudo-inverse of the original matrix `A` by. This is the optional parameter. If it is omitted then it is assumed to be an identity matrix of an appropriate size (So `X` will be the reconstructed pseudo-inverse of `A`). X: The destination matrix: result of back substitution. flags: Operation flags, should match exactly to the `flags` passed to cvSVD.

The function `cvSVBkSb` calculates back substitution for decomposed matrix `A` (see
cvSVD description) and matrix `B`:

```
X=V*W-1*UT*B
```

Where

```
W-1(i,i)=1/W(i,i) if W(i,i) > epsilon•sumiW(i,i),
                    0        otherwise
```

And `epsilon` is a small number that depends on the matrix data type.

This function together with cvSVD is used inside cvInvert and cvSolve,
and the possible reason to use these (svd & bksb) "low-level" function is to avoid
temporary matrices allocation inside the high-level counterparts (inv & solve).

---

### EigenVV

Computes eigenvalues and eigenvectors of symmetric matrix

```
void cvEigenVV( CvArr* mat, CvArr* evects, CvArr* evals, double eps=0 );
```

mat: The input symmetric square matrix. It is modified during the processing. evects: The output matrix of eigenvectors, stored as a subsequent rows. evals: The output vector of eigenvalues, stored in the descending order (order of eigenvalues and eigenvectors is synchronized, of course). eps: Accuracy of diagonalization (typically, DBL\_EPSILON=≈10^-15^ is enough).

The function `cvEigenVV` computes the eigenvalues and eigenvectors of the matrix `A`:

```
mat*evects(i,:)' = evals(i)*evects(i,:)' (in MATLAB notation)
```

The contents of matrix `A` is destroyed by the function.

Currently the function is slower than cvSVD yet less accurate,
so if `A` is known to be positively-defined (for example, it is a covariation matrix),
it is recommended to use cvSVD to find eigenvalues and eigenvectors of `A`,
especially if eigenvectors are not required. That is, instead of

```
cvEigenVV(mat, eigenvals, eigenvects);
```

call

```
cvSVD(mat, eigenvals, eigenvects, 0, CV_SVD_U_T + CV_SVD_MODIFY_A);
```

---

### CalcCovarMatrix

Calculates covariation matrix of the set of vectors

```
void cvCalcCovarMatrix( const CvArr** vects, int count, CvArr* cov_mat, CvArr* avg, int flags );
```

vects: The input vectors. They all must have the same type and the same size. The vectors do not have to be 1D, they can be 2D (e.g. images) etc. count: The number of input vectors. cov\_mat: The output covariation matrix that should be floating-point and square. avg: The input or output (depending on the flags) array - the mean (average) vector of the input vectors. flags: The operation flags, a combination of the following values: `CV_COVAR_SCRAMBLED` - the output covariation matrix is calculated as: `scale*[vects[0]-avg,vects[1]-avg,...]T*[vects[0]-avg,vects[1]-avg,...]`, that is, the covariation matrix is `count`×`count`. Such an unusual covariation matrix is used for fast PCA of a set of very large vectors (see, for example, Eigen Faces technique for face recognition). Eigenvalues of this "scrambled" matrix will match to the eigenvalues of the true covariation matrix and the "true" eigenvectors can be easily calculated from the eigenvectors of the "scrambled" covariation matrix. `CV_COVAR_NORMAL` - the output covariation matrix is calculated as: `scale*[vects[0]-avg,vects[1]-avg,...]*[vects[0]-avg,vects[1]-avg,...]T`, that is, `cov_mat` will be a usual covariation matrix with the same linear size as the total number of elements in every input vector. One and only one of `CV_COVAR_SCRAMBLED` and `CV_COVAR_NORMAL` must be specified `CV_COVAR_USE_AVG` - if the flag is specified, the function does not calculate `avg` from the input vectors, but, instead, uses the passed `avg` vector. This is useful if `avg` has been already calculated somehow, or if the covariation matrix is calculated by parts - in this case, `avg` is not a mean vector of the input sub-set of vectors, but rather the mean vector of the whole set. `CV_COVAR_SCALE` - if the flag is specified, the covariation matrix is scaled by the number of input vectors. `CV_COVAR_ROWS` - Means that all the input vectors are stored as rows of a single matrix, `vects[0]`. `count` is ignored in this case, and `avg` should be a single-row vector of an appropriate size. `CV_COVAR_COLS` - Means that all the input vectors are stored as columns of a single matrix, `vects[0]`. `count` is ignored in this case, and `avg` should be a single-column vector of an appropriate size.

The function `cvCalcCovarMatrix` calculates the covariation matrix and, optionally,
mean vector of the set of input vectors. The function can be used for PCA, for comparing vectors using Mahalanobis distance etc.

---

### Mahalonobis

Calculates Mahalonobis distance between two vectors

```
double cvMahalanobis( const CvArr* vec1, const CvArr* vec2, CvArr* mat );
```

vec1: The first 1D source vector. vec2: The second 1D source vector. mat: The inverse covariation matrix.

The function `cvMahalonobis` calculates the weighted distance between two vectors
and returns it:

```
d(vec1,vec2)=sqrt( sumi,j {mat(i,j)*(vec1(i)-vec2(i))*(vec1(j)-vec2(j))} )
```

The covariation matrix may be calculated using cvCalcCovarMatrix function and further
inverted using cvInvert function (CV\_SVD method is the preferred one, because the matrix
might be singular).

---

### CalcPCA

Performs Principal Component Analysis of a vector set

```
void cvCalcPCA( const CvArr* data, CvArr* avg,
                CvArr* eigenvalues, CvArr* eigenvectors, int flags );
```

data: The input data; each vector is either a single row (`CV_PCA_DATA_AS_ROW`) or a single column (`CV_PCA_DATA_AS_COL`). avg: The mean (average) vector, computed inside the function or provided by user. eigenvalues: The output eigenvalues of covariation matrix. eigenvectors: The output eigenvectors of covariation matrix (i.e. principal components); one vector per row. flags: The operation flags, a combination of the following values: `CV_PCA_DATA_AS_ROW` - the vectors are stored as rows (i.e. all the components of a certain vector are stored continuously) `CV_PCA_DATA_AS_COL` - the vectors are stored as columns (i.e. values of a certain vector component are stored continuously) (the above two flags are mutually exclusive) `CV_PCA_USE_AVG` - use pre-computed average vector

The function `cvCalcPCA` performs PCA analysis of the vector set.
First, it uses cvCalcCovarMatrix to compute covariation matrix
and then it finds its eigenvalues and eigenvectors. The output number of eigenvalues/eigenvectors
should be less than or equal to `MIN(rows(data),cols(data))`.

---

### ProjectPCA

Projects vectors to the specified subspace

```
void cvProjectPCA( const CvArr* data, const CvArr* avg,
                   const CvArr* eigenvectors, CvArr* result )
```

data: The input data; each vector is either a single row or a single column. avg: The mean (average) vector. If it is a single-row vector, it means that the input vectors are stored as rows of `data`; otherwise, it should be a single-column vector, then the vectors are stored as columns of `data`. eigenvectors: The eigenvectors (principal components); one vector per row. result: The output matrix of decomposition coefficients. The number of rows must be the same as the number of vectors, the number of columns must be less than or equal to the number of rows in `eigenvectors`. That it is less, the input vectors are projected into subspace of the first `cols(result)` principal components.

The function `cvProjectPCA` projects input vectors to the subspace represented by the
orthonormal basis (`eigenvectors`). Before computing the dot products, `avg`
vector is subtracted from the input vectors:

```
result(i,:)=(data(i,:)-avg)*eigenvectors' // for CV_PCA_DATA_AS_ROW layout.
```

---

### BackProjectPCA

Reconstructs the original vectors from the projection coefficients

```
void cvBackProjectPCA( const CvArr* proj, const CvArr* avg,
                       const CvArr* eigenvects, CvArr* result );
```

proj: The input data; in the same format as `result` in cvProjectPCA. avg: The mean (average) vector. If it is a single-row vector, it means that the output vectors are stored as rows of `result`; otherwise, it should be a single-column vector, then the vectors are stored as columns of `result`. eigenvectors: The eigenvectors (principal components); one vector per row. result: The output matrix of reconstructed vectors.

The function `cvBackProjectPCA` reconstructs the vectors from the projection coefficients:

```
result(i,:)=proj(i,:)*eigenvectors + avg // for CV_PCA_DATA_AS_ROW layout.
```

---

## Math Functions

---

### Round, Floor, Ceil

Converts floating-point number to integer

```
int cvRound( double value );
int cvFloor( double value );
int cvCeil( double value );
```

value: The input floating-point value

The functions `cvRound, cvFloor` and `cvCeil` convert input floating-point number to
integer using one of the rounding modes. `cvRound` returns the nearest integer value to
the argument.
`cvFloor` returns the maximum integer value that is not larger than the argument.
`cvCeil` returns the minimum integer value that is not smaller than the argument.
On some architectures the functions work *much* faster than the standard cast operations in C.
If absolute value of the argument is greater than 2^31^, the result is not determined.
Special values (±Inf, NaN) are not handled.

---

### Sqrt

Calculates square root

```
float cvSqrt( float value );
```

value: The input floating-point value

The function `cvSqrt` calculates square root of the argument.
If the argument is negative, the result is not determined.

---

### InvSqrt

Calculates inverse square root

```
float cvInvSqrt( float value );
```

value: The input floating-point value

The function `cvInvSqrt` calculates inverse square root of the argument, and
normally it is faster than `1./sqrt(value)`. If the argument is zero or negative, the result is
not determined. Special values (±Inf, NaN) are not handled.

---

### Cbrt

Calculates cubic root

```
float cvCbrt( float value );
```

value: The input floating-point value

The function `cvCbrt` calculates cubic root of the argument, and
normally it is faster than `pow(value,1./3)`. Besides, negative arguments are handled properly.
Special values (±Inf, NaN) are not handled.

---

### FastArctan

Calculates angle of 2D vector

```
float cvFastArctan( float y, float x );
```

x: x-coordinate of 2D vector y: y-coordinate of 2D vector

The function `cvFastArctan` calculates full-range angle of input 2D vector.
The angle is measured in degrees and varies from 0° to 360°. The accuracy is ~0.1°

---

### IsNaN

Determines if the argument is Not A Number

```
int cvIsNaN( double value );
```

value: The input floating-point value

The function `cvIsNaN` returns 1 if the argument is Not A Number (as defined
by IEEE754 standard), 0 otherwise.

---

### IsInf

Determines if the argument is Infinity

```
int cvIsInf( double value );
```

value: The input floating-point value

The function `cvIsInf` returns 1 if the argument is ±Infinity (as defined
by IEEE754 standard), 0 otherwise.

---

### CartToPolar

Calculates magnitude and/or angle of 2d vectors

```
void cvCartToPolar( const CvArr* x, const CvArr* y, CvArr* magnitude,
                    CvArr* angle=NULL, int angle_in_degrees=0 );
```

x: The array of x-coordinates y: The array of y-coordinates magnitude: The destination array of magnitudes, may be set to NULL if it is not needed angle: The destination array of angles, may be set to NULL if it is not needed. The angles are measured in radians (0..2π) or in degrees (0..360°). angle\_in\_degrees: The flag indicating whether the angles are measured in radians, which is default mode, or in degrees.

The function `cvCartToPolar` calculates either magnitude, angle,
or both of every 2d vector `(x(I),y(I))`:

```
magnitude(I)=sqrt( x(I)2+y(I)2 ),
angle(I)=atan( y(I)/x(I) )
```

The angles are calculated with ≈0.1° accuracy. For (0,0) point the angle
is set to 0.

---

### PolarToCart

Calculates Cartesian coordinates of 2d vectors represented in polar form

```
void cvPolarToCart( const CvArr* magnitude, const CvArr* angle,
                    CvArr* x, CvArr* y, int angle_in_degrees=0 );
```

magnitude: The array of magnitudes. If it is NULL, the magnitudes are assumed all 1’s. angle: The array of angles, whether in radians or degrees. x: The destination array of x-coordinates, may be set to NULL if it is not needed. y: The destination array of y-coordinates, may be set to NULL if it is not needed. angle\_in\_degrees: The flag indicating whether the angles are measured in radians, which is default mode, or in degrees.

The function `cvPolarToCart` calculates either x-coordinate, y-coordinate
or both of every vector `magnitude(I)*exp(angle(I)*j), j=sqrt(-1)`:

```
x(I)=magnitude(I)*cos(angle(I)),
y(I)=magnitude(I)*sin(angle(I))
```

---

### Pow

Raises every array element to power

```
void cvPow( const CvArr* src, CvArr* dst, double power );
```

src: The source array. dst: The destination array, should be the same type as the source. power: The exponent of power.

The function `cvPow` raises every element of input array to `p`:

```
dst(I)=src(I)p, if p is integer
dst(I)=abs(src(I))p, otherwise
```

That is, for non-integer power exponent the absolute values of input
array elements are used. However, it is possible to get true values
for negative values using some extra operations, as the following sample,
computing cube root of array elements, shows:

```
CvSize size = cvGetSize(src);
CvMat* mask = cvCreateMat( size.height, size.width, CV_8UC1 );
cvCmpS( src, 0, mask, CV_CMP_LT ); /* find negative elements */
cvPow( src, dst, 1./3 );
cvSubRS( dst, cvScalarAll(0), dst, mask ); /* negate the results of negative inputs */
cvReleaseMat( &mask );
```

For some values of `power`, such as integer values, 0.5 and -0.5,
specialized faster algorithms are used.

---

### Exp

Calculates exponent of every array element

```
void cvExp( const CvArr* src, CvArr* dst );
```

src: The source array. dst: The destination array, it should have `double` type or the same type as the source.

The function `cvExp` calculates exponent of every element of input array:

```
dst(I)=exp(src(I))
```

Maximum relative error is ≈7e-6. Currently,
the function converts denormalized values to zeros on output.

---

### Log

Calculates natural logarithm of every array element absolute value

```
void cvLog( const CvArr* src, CvArr* dst );
```

src: The source array. dst: The destination array, it should have `double` type or the same type as the source.

The function `cvLog` calculates natural logarithm
of absolute value of every element of input array:

```
dst(I)=log(abs(src(I))), src(I)!=0
dst(I)=C,  src(I)=0
```

Where `C` is large negative number (≈-700 in the current implementation)

---

### SolveCubic

Finds real roots of a cubic equation

```
int cvSolveCubic( const CvMat* coeffs, CvMat* roots );
```

coeffs: The equation coefficients, array of 3 or 4 elements. roots: The output array of real roots. Should have 3 elements.

The function `cvSolveCubic` finds real roots of a cubic equation:

```
coeffs[0]*x3 + coeffs[1]*x2 + coeffs[2]*x + coeffs[3] = 0
(if coeffs is 4-element vector)

or

x3 + coeffs[0]*x2 + coeffs[1]*x + coeffs[2] = 0
(if coeffs is 3-element vector)
```

The function returns the number of real roots found. The roots are stored
to `root` array, which is padded with zeros if there is only one root.

---

### SolvePoly

Finds real and complex roots of a polynomial equation with
real coefficients

```
void cvSolvePoly(const CvMat* coeffs, CvMat *roots,
                 int maxiter = 10, int fig = 10);
```

coeffs: The (degree + 1)-length array of equation coefficients (CV\_32FC1 or CV\_64FC1). roots: The degree-length output array of real or complex roots (CV\_32FC2 or CV\_64FC2). maxiter: The maximum number of iterations. fig: The required figures of precision required.

The function `cvSolvePoly` finds all real and complex roots of any
degree polynomial with real coefficients.

---

## Random Number Generation

---

### RNG

Initializes random number generator state

```
CvRNG cvRNG( int64 seed=-1 );
```

seed: 64-bit value used to initiate a random sequence.

The function `cvRNG` initializes random number generator
and returns the state. Pointer to the state can be then passed to cvRandInt,
cvRandReal and cvRandArr functions.
In the current implementation a multiply-with-carry generator is used.

---

### RandArr

Fills array with random numbers and updates the RNG state

```
void cvRandArr( CvRNG* rng, CvArr* arr, int dist_type, CvScalar param1, CvScalar param2 );
```

rng: RNG state initialized by cvRNG. arr: The destination array. dist\_type: Distribution type: CV\_RAND\_UNI - uniform distribution CV\_RAND\_NORMAL - normal or Gaussian distribution param1: The first parameter of distribution. In case of uniform distribution it is the inclusive lower boundary of random numbers range. In case of normal distribution it is the mean value of random numbers. param2: The second parameter of distribution. In case of uniform distribution it is the exclusive upper boundary of random numbers range. In case of normal distribution it is the standard deviation of random numbers.

The function `cvRandArr` fills the destination array with
uniformly or normally distributed random numbers. In the sample below
the function is used to add a few normally distributed
floating-point numbers to random locations within a 2d array

```
/* let noisy_screen be the floating-point 2d array that is to be "crapped" */
CvRNG rng_state = cvRNG(0xffffffff);
int i, pointCount = 1000;
/* allocate the array of coordinates of points */
CvMat* locations = cvCreateMat( pointCount, 1, CV_32SC2 );
/* arr of random point values */
CvMat* values = cvCreateMat( pointCount, 1, CV_32FC1 );
CvSize size = cvGetSize( noisy_screen );

cvRandInit( &rng_state,
            0, 1, /* use dummy parameters now and adjust them further */
            0xffffffff /* just use a fixed seed here */,
            CV_RAND_UNI /* specify uniform type */ );

/* initialize the locations using a uniform distribution */
cvRandArr( &rng_state, locations, CV_RAND_UNI, cvScalar(0,0,0,0), cvScalar(size.width,size.height,0,0) );

/* generate normally distributed random values */
cvRandArr( &rng_state, values, CV_RAND_NORMAL,
           cvRealScalar(100), // average intensity
           cvRealScalar(30) // deviation of the intensity
           );

/* set the points */
for( i = 0; i < pointCount; i++ )
{
    CvPoint pt = *(CvPoint*)cvPtr1D( locations, i, 0 );
    float value = *(float*)cvPtr1D( values, i, 0 );
    *((float*)cvPtr2D( noisy_screen, pt.y, pt.x, 0 )) += value;
}

/* not to forget to release the temporary arrays */
cvReleaseMat( &locations );
cvReleaseMat( &values );

/* RNG state does not need to be deallocated */
```

---

### RandInt

Returns 32-bit unsigned integer and updates RNG

```
unsigned cvRandInt( CvRNG* rng );
```

rng: RNG state initialized by `RandInit` and, optionally, customized by `RandSetRange` (though, the latter function does not affect on the discussed function outcome).

The function `cvRandInt` returns uniformly-distributed random
32-bit unsigned integer and updates RNG state. It is similar to rand() function from C runtime library,
but it always generates 32-bit number whereas rand() returns a number in between 0
and `RAND_MAX` which is 2\*\*16 or 2\*\*32, depending on the platform.

The function is useful for generating scalar random numbers, such as points, patch sizes,
table indices etc, where integer numbers of a certain range can be generated using
modulo operation and floating-point numbers can be generated by scaling to
0..1 of any other specific range. Here is the example from the previous function discussion
rewritten using cvRandInt:

```
/* the input and the task is the same as in the previous sample. */
CvRNG rng_state = cvRNG(0xffffffff);
int i, pointCount = 1000;
/* ... - no arrays are allocated here */
CvSize size = cvGetSize( noisy_screen );
/* make a buffer for normally distributed numbers to reduce call overhead */
#define bufferSize 16
float normalValueBuffer[bufferSize];
CvMat normalValueMat = cvMat( bufferSize, 1, CV_32F, normalValueBuffer );
int valuesLeft = 0;

for( i = 0; i < pointCount; i++ )
{
    CvPoint pt;
    /* generate random point */
    pt.x = cvRandInt( &rng_state ) % size.width;
    pt.y = cvRandInt( &rng_state ) % size.height;

    if( valuesLeft <= 0 )
    {
        /* fulfill the buffer with normally distributed numbers if the buffer is empty */
        cvRandArr( &rng_state, &normalValueMat, CV_RAND_NORMAL, cvRealScalar(100), cvRealScalar(30) );
        valuesLeft = bufferSize;
    }
    *((float*)cvPtr2D( noisy_screen, pt.y, pt.x, 0 ) = normalValueBuffer[--valuesLeft];
}

/* there is no need to deallocate normalValueMat because we have
both the matrix header and the data on stack. It is a common and efficient
practice of working with small, fixed-size matrices */
```

---

### RandReal

Returns floating-point random number and updates RNG

```
double cvRandReal( CvRNG* rng );
```

rng: RNG state initialized by cvRNG.

The function `cvRandReal` returns uniformly-distributed random
floating-point number from 0..1 range (1 is not included).

---

## Discrete Transforms

---

### DFT

Performs forward or inverse Discrete Fourier transform of 1D or 2D floating-point array

```
#define CV_DXT_FORWARD  0
#define CV_DXT_INVERSE  1
#define CV_DXT_SCALE    2
#define CV_DXT_ROWS     4
#define CV_DXT_INV_SCALE (CV_DXT_SCALE|CV_DXT_INVERSE)
#define CV_DXT_INVERSE_SCALE CV_DXT_INV_SCALE

void cvDFT( const CvArr* src, CvArr* dst, int flags, int nonzero_rows=0 );
```

src: Source array, real or complex. dst: Destination array of the same size and same type as the source. flags: Transformation flags, a combination of the following values: `CV_DXT_FORWARD` - do forward 1D or 2D transform. The result is not scaled. `CV_DXT_INVERSE` - do inverse 1D or 2D transform. The result is not scaled. `CV_DXT_FORWARD` and `CV_DXT_INVERSE` are mutually exclusive, of course. `CV_DXT_SCALE` - scale the result: divide it by the number of array elements. Usually, it is combined with `CV_DXT_INVERSE`, and one may use a shortcut `CV_DXT_INV_SCALE`. `CV_DXT_ROWS` - do forward or inverse transform of every individual row of the input matrix. This flag allows user to transform multiple vectors simultaneously and can be used to decrease the overhead (which is sometimes several times larger than the processing itself), to do 3D and higher-dimensional transforms etc. nonzero\_rows: Number of nonzero rows to in the source array (in case of forward 2d transform), or a number of rows of interest in the destination array (in case of inverse 2d transform). If the value is negative, zero, or greater than the total number of rows, it is ignored. The parameter can be used to speed up 2d convolution/correlation when computing them via DFT. See the sample below.

The function `cvDFT` performs forward or inverse transform of
1D or 2D floating-point array:

```
Forward Fourier transform of 1D vector of N elements:
y = F(N)•x, where F(N)jk=exp(-i•2Pi•j•k/N), i=sqrt(-1)

Inverse Fourier transform of 1D vector of N elements:
x'= (F(N))-1•y = conj(F(N))•y
x = (1/N)•x

Forward Fourier transform of 2D vector of M×N elements:
Y = F(M)•X•F(N)

Inverse Fourier transform of 2D vector of M×N elements:
X'= conj(F(M))•Y•conj(F(N))
X = (1/(M•N))•X'
```

In case of real (single-channel) data, the packed format, borrowed from IPL, is used to
to represent a result of forward Fourier transform or input for inverse Fourier transform:

```
Re Y0,0      Re Y0,1    Im Y0,1    Re Y0,2     Im Y0,2  ...  Re Y0,N/2-1   Im Y0,N/2-1  Re Y0,N/2
Re Y1,0      Re Y1,1    Im Y1,1    Re Y1,2     Im Y1,2  ...  Re Y1,N/2-1   Im Y1,N/2-1  Re Y1,N/2
Im Y1,0      Re Y2,1    Im Y2,1    Re Y2,2     Im Y2,2  ...  Re Y2,N/2-1   Im Y2,N/2-1  Im Y2,N/2
............................................................................................
Re YM/2-1,0   Re YM-3,1   Im YM-3,1  Re YM-3,2   Im YM-3,2 ...  Re YM-3,N/2-1  Im YM-3,N/2-1 Re YM-3,N/2
Im YM/2-1,0   Re YM-2,1   Im YM-2,1  Re YM-2,2   Im YM-2,2 ...  Re YM-2,N/2-1  Im YM-2,N/2-1 Im YM-2,N/2
Re YM/2,0    Re YM-1,1   Im YM-1,1  Re YM-1,2   Im YM-1,2  ... Re YM-1,N/2-1  Im YM-1,N/2-1 Im YM-1,N/2
```

Note: the last column is present if `N` is even, the last row is present if `M` is even.

In case of 1D real transform the result looks like the first row of the above matrix

Computing 2D Convolution using DFT

```
   CvMat* A = cvCreateMat( M1, N1, CV_32F );
   CvMat* B = cvCreateMat( M2, N2, A->type );

   // it is also possible to have only abs(M2-M1)+1×abs(N2-N1)+1
   // part of the full convolution result
   CvMat* conv = cvCreateMat( A->rows + B->rows - 1, A->cols + B->cols - 1, A->type );

   // initialize A and B
   ...

   int dft_M = cvGetOptimalDFTSize( A->rows + B->rows - 1 );
   int dft_N = cvGetOptimalDFTSize( A->cols + B->cols - 1 );

   CvMat* dft_A = cvCreateMat( dft_M, dft_N, A->type );
   CvMat* dft_B = cvCreateMat( dft_M, dft_N, B->type );
   CvMat tmp;

   // copy A to dft_A and pad dft_A with zeros
   cvGetSubRect( dft_A, &tmp, cvRect(0,0,A->cols,A->rows));
   cvCopy( A, &tmp );
   cvGetSubRect( dft_A, &tmp, cvRect(A->cols,0,dft_A->cols - A->cols,A->rows));
   cvZero( &tmp );
   // no need to pad bottom part of dft_A with zeros because of
   // use nonzero_rows parameter in cvDFT() call below

   cvDFT( dft_A, dft_A, CV_DXT_FORWARD, A->rows );

   // repeat the same with the second array
   cvGetSubRect( dft_B, &tmp, cvRect(0,0,B->cols,B->rows));
   cvCopy( B, &tmp );
   cvGetSubRect( dft_B, &tmp, cvRect(B->cols,0,dft_B->cols - B->cols,B->rows));
   cvZero( &tmp );
   // no need to pad bottom part of dft_B with zeros because of
   // use nonzero_rows parameter in cvDFT() call below

   cvDFT( dft_B, dft_B, CV_DXT_FORWARD, B->rows );

   cvMulSpectrums( dft_A, dft_B, dft_A, 0 /* or CV_DXT_MUL_CONJ to get correlation
                                             rather than convolution */ );

   cvDFT( dft_A, dft_A, CV_DXT_INV_SCALE, conv->rows ); // calculate only the top part
   cvGetSubRect( dft_A, &tmp, cvRect(0,0,conv->cols,conv->rows) );

   cvCopy( &tmp, conv );
```

---

### GetOptimalDFTSize

Returns optimal DFT size for given vector size

```
int cvGetOptimalDFTSize( int size0 );
```

size0: Vector size.

The function `cvGetOptimalDFTSize` returns the minimum
number `N` that is greater to equal to `size0`, such that DFT of a vector
of size `N` can be computed fast. In the current implementation
`N=2p×3q×5r` for some `p, q, r`.

The function returns a negative number if `size0` is too large (very close to `INT_MAX`)

---

### MulSpectrums

Performs per-element multiplication of two Fourier spectrums

```
void cvMulSpectrums( const CvArr* src1, const CvArr* src2, CvArr* dst, int flags );
```

src1: The first source array. src2: The second source array. dst: The destination array of the same type and the same size of the sources. flags: A combination of the following values: `CV_DXT_ROWS` - treat each row of the arrays as a separate spectrum (see cvDFT parameters description). `CV_DXT_MUL_CONJ` - conjugate the second source array before the multiplication.

The function `cvMulSpectrums` performs per-element multiplication
of the two CCS-packed or complex matrices that are results of real or complex Fourier transform.

The function, together with cvDFT, may be used to calculate convolution
of two arrays fast.

---

### DCT

Performs forward or inverse Discrete Cosine transform of 1D or 2D floating-point array

```
#define CV_DXT_FORWARD  0
#define CV_DXT_INVERSE  1
#define CV_DXT_ROWS     4

void cvDCT( const CvArr* src, CvArr* dst, int flags );
```

src: Source array, real 1D or 2D array. dst: Destination array of the same size and same type as the source. flags: Transformation flags, a combination of the following values: `CV_DXT_FORWARD` - do forward 1D or 2D transform. `CV_DXT_INVERSE` - do inverse 1D or 2D transform. `CV_DXT_ROWS` - do forward or inverse transform of every individual row of the input matrix. This flag allows user to transform multiple vectors simultaneously and can be used to decrease the overhead (which is sometimes several times larger than the processing itself), to do 3D and higher-dimensional transforms etc.

The function `cvDCT` performs forward or inverse transform of
1D or 2D floating-point array:

```
Forward Cosine transform of 1D vector of N elements:
y = C(N)•x, where C(N)jk=sqrt((j==0?1:2)/N)•cos(Pi•(2k+1)•j/N)

Inverse Cosine transform of 1D vector of N elements:
x = (C(N))-1•y = (C(N))T•y

Forward Cosine transform of 2D vector of M×N elements:
Y = (C(M))•X•(C(N))T

Inverse Cosine transform of 2D vector of M×N elements:
X = (C(M))T•Y•C(N)
```

---

# Dynamic Structures

---

## Memory Storages

---

### CvMemStorage

Growing memory storage

```
typedef struct CvMemStorage
{
    struct CvMemBlock* bottom;/* first allocated block */
    struct CvMemBlock* top; /* the current memory block - top of the stack */
    struct CvMemStorage* parent; /* borrows new blocks from */
    int block_size; /* block size */
    int free_space; /* free space in the top block (in bytes) */
} CvMemStorage;
```

Memory storage is a low-level structure used to store dynamically growing data structures such as
sequences, contours, graphs, subdivisions etc. It is organized as a list of memory blocks of
equal size - `bottom` field is the beginning of the list of blocks
and `top` is the currently used block, but not necessarily the last block of the list. All blocks
between `bottom` and `top`, not including the latter, are considered fully occupied;
and all blocks between `top` and the last block, not including `top`,
are considered free and `top` block itself is partly occupied - `free_space`
contains the number of free bytes left in the end of `top`.

New memory buffer that may be allocated explicitly by cvMemStorageAlloc function
or implicitly by
higher-level functions, such as cvSeqPush, cvGraphAddEdge etc., `always`
starts in the end of the current block if it fits there. After allocation `free_space`
is decremented by the size of the allocated buffer plus some padding to keep the proper alignment.
When the allocated buffer does not fit into the available part of `top`, the next storage
block from the list is taken as `top` and `free_space` is reset to the
whole block size prior to the allocation.

If there is no more free blocks, a new block is allocated (or borrowed from parent, see
cvCreateChildMemStorage) and added to the end of list. Thus, the storage behaves as a stack
with `bottom` indicating bottom of the stack and the pair (`top`, `free_space`)
indicating top of the stack. The stack top may be saved via cvSaveMemStoragePos,
restored via cvRestoreMemStoragePos or reset via cvClearStorage.

---

### CvMemBlock

Memory storage block

```
typedef struct CvMemBlock
{
    struct CvMemBlock* prev;
    struct CvMemBlock* next;
} CvMemBlock;
```

The structure CvMemBlock represents a single block of memory storage.
Actual data of the memory blocks follows the header, that is, the `i-th` byte of
the memory block can be retrieved with the expression `((char*)(mem_block_ptr+1))[i]`.
However, normally there is no need to access the storage structure fields directly.

---

### CvMemStoragePos

Memory storage position

```
typedef struct CvMemStoragePos
{
    CvMemBlock* top;
    int free_space;
} CvMemStoragePos;
```

The structure described below stores the position of the stack top that can be
saved via cvSaveMemStoragePos and restored via cvRestoreMemStoragePos.

---

### CreateMemStorage

Creates memory storage

```
CvMemStorage* cvCreateMemStorage( int block_size=0 );
```

block\_size: Size of the storage blocks in bytes. If it is 0, the block size is set to default value - currently it is ≈64K.

The function `cvCreateMemStorage` creates a memory storage and returns pointer
to it. Initially the storage is empty. All fields of the header, except the `block_size`,
are set to 0.

---

### CreateChildMemStorage

Creates child memory storage

```
CvMemStorage* cvCreateChildMemStorage( CvMemStorage* parent );
```

parent: Parent memory storage.

The function `cvCreateChildMemStorage` creates a child memory storage that is similar
to simple memory storage except for the differences in the memory
allocation/deallocation mechanism. When a child storage needs a new block to
add to the block list, it tries to get this block from the parent. The first
unoccupied parent block available is taken and excluded from the parent block
list. If no blocks are available, the parent either allocates a block or borrows
one from its own parent, if any. In other words, the chain, or a more complex
structure, of memory storages where every storage is a child/parent of another
is possible. When a child storage is released or even cleared, it returns all
blocks to the parent. In other aspects, the child storage is the same as the simple storage.

The children storages are useful in the following situation.
Imagine that user needs to process dynamical data resided in some storage and
put the result back to the same storage. With the simplest approach, when temporary
data is resided in the same storage as the input and output data, the storage
will look as following after processing:

Dynamic data processing without using child storage

That is, garbage appears in the middle of the storage.
However, if one creates a child memory storage in the beginning of the processing,
writes temporary data there and releases the child storage in the end, no garbage will
appear in the source/destination storage:

Dynamic data processing using a child storage

---

### ReleaseMemStorage

Releases memory storage

```
void cvReleaseMemStorage( CvMemStorage** storage );
```

storage: Pointer to the released storage.

The function `cvReleaseMemStorage` deallocates all storage memory blocks or returns
them to the parent, if any. Then it deallocates the storage header and clears
the pointer to the storage. All children of the storage must be released before
the parent is released.

---

### ClearMemStorage

Clears memory storage

```
void cvClearMemStorage( CvMemStorage* storage );
```

storage: Memory storage.

The function `cvClearMemStorage` resets the top (free space boundary) of the storage
to the very beginning. This function does not deallocate any memory. If the
storage has a parent, the function returns all blocks to the parent.

---

### MemStorageAlloc

Allocates memory buffer in the storage

```
void* cvMemStorageAlloc( CvMemStorage* storage, size_t size );
```

storage: Memory storage. size: Buffer size.

The function `cvMemStorageAlloc` allocates memory buffer in the storage. The buffer size
must not exceed the storage block size, otherwise runtime error is raised. The buffer address is
aligned by `CV_STRUCT_ALIGN` (=`sizeof(double)` for the moment) bytes.

---

### MemStorageAllocString

Allocates text string in the storage

```
typedef struct CvString
{
    int len;
    char* ptr;
}
CvString;

CvString cvMemStorageAllocString( CvMemStorage* storage, const char* ptr, int len=-1 );
```

storage: Memory storage. ptr: The string. len: Length of the string (not counting the ending '\0'). If the parameter is negative, the function computes the length.

The function `cvMemStorageAllocString` creates copy of the
string in the memory storage. It returns the structure that contains user-passed or computed length
of the string and pointer to the copied string.

---

### SaveMemStoragePos

Saves memory storage position

```
void cvSaveMemStoragePos( const CvMemStorage* storage, CvMemStoragePos* pos );
```

storage: Memory storage. pos: The output position of the storage top.

The function `cvSaveMemStoragePos` saves the current position of the storage top to
the parameter `pos`. The function `cvRestoreMemStoragePos` can further retrieve
this position.

---

### RestoreMemStoragePos

Restores memory storage position

```
void cvRestoreMemStoragePos( CvMemStorage* storage, CvMemStoragePos* pos );
```

storage: Memory storage. pos: New storage top position.

The function `cvRestoreMemStoragePos` restores the position of the storage top from
the parameter `pos`. This function and The function `cvClearMemStorage` are the
only methods to release memory occupied in memory blocks.
Note again that there is no way to free memory in the middle of the occupied part of the storage.

---

## Sequences

---

### CvSeq

Growable sequence of elements

```
#define CV_SEQUENCE_FIELDS() \
    int flags; /* miscellaneous flags */ \
    int header_size; /* size of sequence header */ \
    struct CvSeq* h_prev; /* previous sequence */ \
    struct CvSeq* h_next; /* next sequence */ \
    struct CvSeq* v_prev; /* 2nd previous sequence */ \
    struct CvSeq* v_next; /* 2nd next sequence */ \
    int total; /* total number of elements */ \
    int elem_size;/* size of sequence element in bytes */ \
    char* block_max;/* maximal bound of the last block */ \
    char* ptr; /* current write pointer */ \
    int delta_elems; /* how many elements allocated when the sequence grows (sequence granularity) */ \
    CvMemStorage* storage; /* where the seq is stored */ \
    CvSeqBlock* free_blocks; /* free blocks list */ \
    CvSeqBlock* first; /* pointer to the first sequence block */


typedef struct CvSeq
{
    CV_SEQUENCE_FIELDS()
} CvSeq;
```

The structure CvSeq is a base for all of OpenCV dynamic data structures.

Such an unusual definition via a helper macro simplifies the extension of the structure
CvSeq with additional parameters.
To extend CvSeq the user may define a new structure and
put user-defined fields after all CvSeq fields that are included via the macro
`CV_SEQUENCE_FIELDS()`.

There are two types of sequences - dense and sparse. Base type for dense sequences is CvSeq
and such sequences are used to represent growable 1d arrays - vectors, stacks, queues, deques.
They have no gaps in the middle - if an element is removed from the middle or inserted into the middle
of the sequence the elements from the closer end are shifted.
Sparse sequences have CvSet base
class and they are discussed later in more details. They are sequences of nodes each of those may be
either occupied or free as indicated by the node flag. Such sequences are used for unordered data
structures such as sets of elements, graphs, hash tables etc.

The field `header_size` contains the actual size of the
sequence header and should be greater or equal to `sizeof(CvSeq)`.

The fields
`h_prev, h_next, v_prev, v_next` can be used to create hierarchical structures
from separate sequences. The fields `h_prev` and `h_next` point to the previous and
the next sequences on the same hierarchical level while the fields `v_prev` and
`v_next` point to the previous and the next sequence in the vertical direction,
that is, parent and its first child. But these are just names and the pointers
can be used in a different way.

The field `first` points to the first sequence
block, whose structure is described below.

The field `total` contains the actual number of dense sequence elements and
number of allocated nodes in sparse sequence.

The field `flags`contain the particular dynamic type
signature (`CV_SEQ_MAGIC_VAL` for dense sequences and `CV_SET_MAGIC_VAL` for sparse sequences)
in the highest 16 bits and miscellaneous information about the sequence.
The lowest `CV_SEQ_ELTYPE_BITS` bits contain the ID of the
element type. Most of sequence processing functions do not use element type
but element size stored in `elem_size`.
If sequence contains the numeric data of one of CvMat type
then the element type matches to the corresponding CvMat element type, e.g.
CV\_32SC2 may be used for sequence of 2D points, CV\_32FC1 for sequences of floating-point values etc.
`CV_SEQ_ELTYPE(seq_header_ptr)` macro retrieves the type of sequence elements.
Processing function that work with numerical sequences check that `elem_size` is equal
to the calculated from the type element size.
Besides CvMat compatible types, there are few extra element types defined
in cvtypes.h header:

Standard Types of Sequence Elements

```
    #define CV_SEQ_ELTYPE_POINT          CV_32SC2  /* (x,y) */
    #define CV_SEQ_ELTYPE_CODE           CV_8UC1   /* freeman code: 0..7 */
    #define CV_SEQ_ELTYPE_GENERIC        0 /* unspecified type of sequence elements */
    #define CV_SEQ_ELTYPE_PTR            CV_USRTYPE1 /* =6 */
    #define CV_SEQ_ELTYPE_PPOINT         CV_SEQ_ELTYPE_PTR  /* &elem: pointer to element of other sequence */
    #define CV_SEQ_ELTYPE_INDEX          CV_32SC1  /* #elem: index of element of some other sequence */
    #define CV_SEQ_ELTYPE_GRAPH_EDGE     CV_SEQ_ELTYPE_GENERIC  /* &next_o, &next_d, &vtx_o, &vtx_d */
    #define CV_SEQ_ELTYPE_GRAPH_VERTEX   CV_SEQ_ELTYPE_GENERIC  /* first_edge, &(x,y) */
    #define CV_SEQ_ELTYPE_TRIAN_ATR      CV_SEQ_ELTYPE_GENERIC  /* vertex of the binary tree   */
    #define CV_SEQ_ELTYPE_CONNECTED_COMP CV_SEQ_ELTYPE_GENERIC  /* connected component  */
    #define CV_SEQ_ELTYPE_POINT3D        CV_32FC3  /* (x,y,z)  */
```

The next `CV_SEQ_KIND_BITS` bits specify the kind of the sequence:

Standard Kinds of Sequences

```
    /* generic (unspecified) kind of sequence */
    #define CV_SEQ_KIND_GENERIC     (0 << CV_SEQ_ELTYPE_BITS)

    /* dense sequence subtypes */
    #define CV_SEQ_KIND_CURVE       (1 << CV_SEQ_ELTYPE_BITS)
    #define CV_SEQ_KIND_BIN_TREE    (2 << CV_SEQ_ELTYPE_BITS)

    /* sparse sequence (or set) subtypes */
    #define CV_SEQ_KIND_GRAPH       (3 << CV_SEQ_ELTYPE_BITS)
    #define CV_SEQ_KIND_SUBDIV2D    (4 << CV_SEQ_ELTYPE_BITS)
```

The remaining bits are used to identify different features specific to certain
sequence kinds and element types. For example, curves made of points (
`CV_SEQ_KIND_CURVE|CV_SEQ_ELTYPE_POINT` ), together with the flag
`CV_SEQ_FLAG_CLOSED` belong to the type `CV_SEQ_POLYGON` or, if other flags are
used, to its subtype. Many contour processing functions check the type of the input
sequence and report an error if they do not support this type. The file
cvtypes.h stores the complete list of all supported predefined sequence types
and helper macros designed to get the sequence type of other properties.
Below follows the definition of the building block of sequences.

---

### CvSeqBlock

Continuous sequence block

```
typedef struct CvSeqBlock
{
    struct CvSeqBlock* prev; /* previous sequence block */
    struct CvSeqBlock* next; /* next sequence block */
    int start_index; /* index of the first element in the block +
    sequence->first->start_index */
    int count; /* number of elements in the block */
    char* data; /* pointer to the first element of the block */
} CvSeqBlock;
```

Sequence blocks make up a circular double-linked list, so the pointers `prev` and
`next` are never `NULL` and point to the previous and the next sequence blocks
within the sequence. It means that `next` of the last block is the first block and
`prev` of the first block is the last block. The fields `start_index` and `count` help
to track the block location within the sequence. For example, if the sequence
consists of 10 elements and splits into three blocks of 3, 5, and 2 elements,
and the first block has the parameter `start_index = 2`, then pairs `(start_index, count)`
for the sequence blocks are `(2,3), (5, 5)`, and `(10, 2)`
correspondingly. The parameter `start_index` of the first block is usually `0`
unless some elements have been inserted at the beginning of the sequence.

---

### CvSlice

A sequence slice

```
typedef struct CvSlice
{
    int start_index;
    int end_index;
} CvSlice;

inline CvSlice cvSlice( int start, int end );
#define CV_WHOLE_SEQ_END_INDEX 0x3fffffff
#define CV_WHOLE_SEQ  cvSlice(0, CV_WHOLE_SEQ_END_INDEX)

/* calculates the sequence slice length */
int cvSliceLength( CvSlice slice, const CvSeq* seq );
```

Some of functions that operate on sequences take `CvSlice slice` parameter that is
often set to the whole sequence (CV\_WHOLE\_SEQ) by default. Either of the `start_index`
and `end_index` may be negative or exceed the sequence length, `start_index`
is inclusive, `end_index` is exclusive boundary. If they are equal, the slice is considered
empty (i.e. contains no elements). Because sequences are treated as circular structures,
the slice may select a few elements in the end of a sequence followed by a few elements in the beginning
of the sequence, for example, `cvSlice(-2, 3)` in case of 10-element sequence will select 5-element slice,
containing pre-last (8th), last (9th), the very first (0th), second (1th) and third (2nd) elements.
The functions normalize the slice argument in the following way: first, cvSliceLength
is called to determine the length of the slice, then, `start_index` of the slice is
normalized similarly to the argument of cvGetSeqElem
(i.e. negative indices are allowed).
The actual slice to process starts at the normalized `start_index`
and lasts cvSliceLength elements
(again, assuming the sequence is a circular structure).

If a function does not take slice argument, but you want to process only a part of the sequence,
the sub-sequence may be extracted using cvSeqSlice function,
or stored as into a continuous buffer with cvCvtSeqToArray
(optionally, followed by cvMakeSeqHeaderForArray.

---

### CreateSeq

Creates sequence

```
CvSeq* cvCreateSeq( int seq_flags, int header_size,
                    int elem_size, CvMemStorage* storage );
```

seq\_flags: Flags of the created sequence. If the sequence is not passed to any function working with a specific type of sequences, the sequence value may be set to 0, otherwise the appropriate type must be selected from the list of predefined sequence types. header\_size: Size of the sequence header; must be greater or equal to `sizeof(CvSeq)`. If a specific type or its extension is indicated, this type must fit the base type header. elem\_size: Size of the sequence elements in bytes. The size must be consistent with the sequence type. For example, for a sequence of points to be created, the element type `CV_SEQ_ELTYPE_POINT` should be specified and the parameter `elem_size` must be equal to `sizeof(CvPoint)`. storage: Sequence location.

The function `cvCreateSeq` creates a sequence and returns the pointer to it. The
function allocates the sequence header in the storage block as one continuous
chunk and sets the structure fields `flags`, `elem_size`, `header_size` and `storage` to
passed values, sets `delta_elems` to the default value (that may be reassigned using
cvSetSeqBlockSize function), and clears other header fields,
including the space after the first `sizeof(CvSeq)` bytes.

---

### SetSeqBlockSize

Sets up sequence block size

```
void cvSetSeqBlockSize( CvSeq* seq, int delta_elems );
```

seq: Sequence. delta\_elems: Desirable sequence block size in elements.

The function `cvSetSeqBlockSize` affects memory allocation granularity.
When the free space in the sequence buffers has run out, the function allocates the space
for `delta_elems` sequence elements. If this block immediately follows the one
previously allocated, the two blocks are concatenated, otherwise, a new sequence
block is created. Therefore, the bigger the parameter is, the lower the possible sequence
fragmentation, but the more space in the storage is wasted. When the
sequence is created, the parameter `delta_elems` is set to the default value ≈1K.
The function can be called any time after the sequence is created and affects
future allocations. The function can modify the passed value of the parameter to
meet the memory storage constraints.

---

### SeqPush

Adds element to sequence end

```
schar* cvSeqPush( CvSeq* seq, void* element=NULL );
```

seq: Sequence. element: Added element.

The function `cvSeqPush` adds an element to the end of sequence and returns pointer
to the allocated element. If the input `element` is NULL,
the function simply allocates a space for one more element.

The following code demonstrates how to create a new sequence using this function:

```
CvMemStorage* storage = cvCreateMemStorage(0);
CvSeq* seq = cvCreateSeq( CV_32SC1, /* sequence of integer elements */
                          sizeof(CvSeq), /* header size - no extra fields */
                          sizeof(int), /* element size */
                          storage /* the container storage */ );
int i;
for( i = 0; i < 100; i++ )
{
    int* added = (int*)cvSeqPush( seq, &i );
    printf( "%d is added\n", *added );
}

...
/* release memory storage in the end */
cvReleaseMemStorage( &storage );
```

The function `cvSeqPush` has O(1) complexity, but there is a faster method for
writing large sequences (see cvStartWriteSeq and related functions).

---

### SeqPop

Removes element from sequence end

```
void cvSeqPop( CvSeq* seq, void* element=NULL );
```

seq: Sequence. element: Optional parameter. If the pointer is not zero, the function copies the removed element to this location.

The function `cvSeqPop` removes an element from the sequence. The function reports
an error if the sequence is already empty. The function has O(1) complexity.

---

### SeqPushFront

Adds element to sequence beginning

```
schar* cvSeqPushFront( CvSeq* seq, void* element=NULL );
```

seq: Sequence. element: Added element.

The function `cvSeqPushFront` is similar to cvSeqPush but it adds the new element
to the beginning of the sequence. The function has O(1) complexity.

---

### SeqPopFront

Removes element from sequence beginning

```
void cvSeqPopFront( CvSeq* seq, void* element=NULL );
```

seq: Sequence. element: Optional parameter. If the pointer is not zero, the function copies the removed element to this location.

The function `cvSeqPopFront` removes an element from the beginning of the sequence.
The function reports an error if the sequence is already empty. The function has O(1) complexity.

---

### SeqPushMulti

Pushes several elements to the either end of sequence

```
void cvSeqPushMulti( CvSeq* seq, void* elements, int count, int in_front=0 );
```

seq: Sequence. elements: Added elements. count: Number of elements to push. in\_front: The flags specifying the modified sequence end: CV\_BACK (=0) - the elements are added to the end of sequence CV\_FRONT(!=0) - the elements are added to the beginning of sequence

The function `cvSeqPushMulti` adds several elements to either end of the sequence.
The elements are added to the sequence in the same order as they are arranged in the
input array but they can fall into different sequence blocks.

---

### SeqPopMulti

Removes several elements from the either end of sequence

```
void cvSeqPopMulti( CvSeq* seq, void* elements, int count, int in_front=0 );
```

seq: Sequence. elements: Removed elements. count: Number of elements to pop. in\_front: The flags specifying the modified sequence end: CV\_BACK (=0) - the elements are removed from the end of sequence CV\_FRONT(!=0) - the elements are removed from the beginning of sequence

The function `cvSeqPopMulti` removes several elements from either end of the sequence.
If the number of the elements to be removed exceeds the total number of elements
in the sequence, the function removes as many elements as possible.

---

### SeqInsert

Inserts element in sequence middle

```
schar* cvSeqInsert( CvSeq* seq, int before_index, void* element=NULL );
```

seq: Sequence. before\_index: Index before which the element is inserted. Inserting before 0 (the minimal allowed value of the parameter) is equal to cvSeqPushFront and inserting before `seq->total` (the maximal allowed value of the parameter) is equal to cvSeqPush. element: Inserted element.

The function `cvSeqInsert` shifts the sequence elements from the inserted position
to the nearest end of the sequence and copies the `element` content there if
the pointer is not NULL. The function returns pointer to the inserted element.

---

### SeqRemove

Removes element from sequence middle

```
void cvSeqRemove( CvSeq* seq, int index );
```

seq: Sequence. index: Index of removed element.

The function `cvSeqRemove` removes elements with the given index. If the index is
out of range the function reports an error.
An attempt to remove an element from an empty sequence is a
partial case of this situation. The function removes an element by shifting the
sequence elements between the nearest end of the sequence and the `index`-th position, not
counting the latter.

---

### ClearSeq

Clears sequence

```
void cvClearSeq( CvSeq* seq );
```

seq: Sequence.

The function `cvClearSeq` removes all elements from the sequence. The function does not return the
memory to the storage, but this memory is reused later when new elements are added
to the sequence. This function time complexity is `O(1)`.

---

### GetSeqElem

Returns pointer to sequence element by its index

```
char* cvGetSeqElem( const CvSeq* seq, int index );
#define CV_GET_SEQ_ELEM( TYPE, seq, index )  (TYPE*)cvGetSeqElem( (CvSeq*)(seq), (index) )
```

seq: Sequence. index: Index of element.

The function `cvGetSeqElem` finds the element with the given index in the sequence
and returns the pointer to it. If the element is not found,
the function returns 0. The function supports negative indices, where -1 stands
for the last sequence element, -2 stands for the one before last, etc. If the
sequence is most likely to consist of a single sequence block or the desired
element is likely to be located in the first block, then the macro
`CV_GET_SEQ_ELEM( elemType, seq, index )` should be used, where the parameter
`elemType` is the type of sequence elements ( CvPoint for example), the parameter
`seq` is a sequence, and the parameter `index` is the index of the desired element.
The macro checks first whether the desired element belongs to the first block of
the sequence and returns it if it does, otherwise the macro calls the main
function `GetSeqElem`. Negative indices always cause the cvGetSeqElem call.
The function has O(1) time complexity assuming that number of blocks is much smaller than the
number of elements.

---

### SeqElemIdx

Returns index of concrete sequence element

```
int cvSeqElemIdx( const CvSeq* seq, const void* element, CvSeqBlock** block=NULL );
```

seq: Sequence. element: Pointer to the element within the sequence. block: Optional argument. If the pointer is not `NULL`, the address of the sequence block that contains the element is stored in this location.

The function `cvSeqElemIdx` returns the index of a sequence element or a negative
number if the element is not found.

---

### CvtSeqToArray

Copies sequence to one continuous block of memory

```
void* cvCvtSeqToArray( const CvSeq* seq, void* elements, CvSlice slice=CV_WHOLE_SEQ );
```

seq: Sequence. elements: Pointer to the destination array that must be large enough. It should be a pointer to data, not a matrix header. slice: The sequence part to copy to the array.

The function `cvCvtSeqToArray` copies the entire sequence or subsequence to the
specified buffer and returns the pointer to the buffer.

---

### MakeSeqHeaderForArray

Constructs sequence from array

```
CvSeq* cvMakeSeqHeaderForArray( int seq_type, int header_size, int elem_size,
                                void* elements, int total,
                                CvSeq* seq, CvSeqBlock* block );
```

seq\_type: Type of the created sequence. header\_size: Size of the header of the sequence. Parameter sequence must point to the structure of that size or greater size. elem\_size: Size of the sequence element. elements: Elements that will form a sequence. total: Total number of elements in the sequence. The number of array elements must be equal to the value of this parameter. seq: Pointer to the local variable that is used as the sequence header. block: Pointer to the local variable that is the header of the single sequence block.

The function `cvMakeSeqHeaderForArray` initializes sequence header for array.
The sequence header as well as the sequence block are allocated by the user (for example, on stack).
No data is copied by the function. The resultant sequence will consists of a single block and have
NULL storage pointer, thus, it is possible to read its elements, but the attempts to
add elements to the sequence will raise an error in most cases.

---

### SeqSlice

Makes separate header for the sequence slice

```
CvSeq* cvSeqSlice( const CvSeq* seq, CvSlice slice,
                   CvMemStorage* storage=NULL, int copy_data=0 );
```

seq: Sequence. slice: The part of the sequence to extract. storage: The destination storage to keep the new sequence header and the copied data if any. If it is NULL, the function uses the storage containing the input sequence. copy\_data: The flag that indicates whether to copy the elements of the extracted slice (`copy_data`!=0) or not (`copy_data`=0)

The function `cvSeqSlice` creates a sequence
that represents the specified slice of the input sequence. The new sequence either shares the elements
with the original sequence or has own copy of the elements.
So if one needs to process a part of sequence but the processing function does not have a slice parameter,
the required sub-sequence may be extracted using this function.

---

### CloneSeq

Creates a copy of sequence

```
CvSeq* cvCloneSeq( const CvSeq* seq, CvMemStorage* storage=NULL );
```

seq: Sequence. storage: The destination storage to keep the new sequence header and the copied data if any. If it is NULL, the function uses the storage containing the input sequence.

The function `cvCloneSeq` makes a complete copy of the input sequence and returns it.
The call `cvCloneSeq( seq, storage )` is equivalent to
`cvSeqSlice( seq, CV_WHOLE_SEQ, storage, 1 )`

---

### SeqRemoveSlice

Removes sequence slice

```
void cvSeqRemoveSlice( CvSeq* seq, CvSlice slice );
```

seq: Sequence. slice: The part of the sequence to remove.

The function `cvSeqRemoveSlice` removes slice from the sequence.

---

### SeqInsertSlice

Inserts array in the middle of sequence

```
void cvSeqInsertSlice( CvSeq* seq, int before_index, const CvArr* from_arr );
```

seq: Sequence. slice: The part of the sequence to remove. from\_arr: The array to take elements from.

The function `cvSeqInsertSlice` inserts all `from_arr`
array elements at the specified position of the sequence. The array `from_arr`
can be a matrix or another sequence.

---

### SeqInvert

Reverses the order of sequence elements

```
void cvSeqInvert( CvSeq* seq );
```

seq: Sequence.

The function `cvSeqInvert` reverses the sequence in-place - makes the first element go last,
the last element go first etc.

---

### SeqSort

Sorts sequence element using the specified comparison function

```
/* a < b ? -1 : a > b ? 1 : 0 */
typedef int (CV_CDECL* CvCmpFunc)(const void* a, const void* b, void* userdata);

void cvSeqSort( CvSeq* seq, CvCmpFunc func, void* userdata=NULL );
```

seq: The sequence to sort func: The comparison function that returns negative, zero or positive value depending on the elements relation (see the above declaration and the example below) - similar function is used by `qsort` from C runtime except that in the latter `userdata` is not used userdata: The user parameter passed to the comparison function; helps to avoid global variables in some cases.

The function `cvSeqSort` sorts the sequence in-place using the specified criteria.
Below is the example of the function use:

```
/* Sort 2d points in top-to-bottom left-to-right order */
static int cmp_func( const void* _a, const void* _b, void* userdata )
{
    CvPoint* a = (CvPoint*)_a;
    CvPoint* b = (CvPoint*)_b;
    int y_diff = a->y - b->y;
    int x_diff = a->x - b->x;
    return y_diff ? y_diff : x_diff;
}

...

CvMemStorage* storage = cvCreateMemStorage(0);
CvSeq* seq = cvCreateSeq( CV_32SC2, sizeof(CvSeq), sizeof(CvPoint), storage );
int i;

for( i = 0; i < 10; i++ )
{
    CvPoint pt;
    pt.x = rand() % 1000;
    pt.y = rand() % 1000;
    cvSeqPush( seq, &pt );
}

cvSeqSort( seq, cmp_func, 0 /* userdata is not used here */ );

/* print out the sorted sequence */
for( i = 0; i < seq->total; i++ )
{
    CvPoint* pt = (CvPoint*)cvSeqElem( seq, i );
    printf( "(%d,%d)\n", pt->x, pt->y );
}

cvReleaseMemStorage( &storage );
```

---

### SeqSearch

Searches element in sequence

```
/* a < b ? -1 : a > b ? 1 : 0 */
typedef int (CV_CDECL* CvCmpFunc)(const void* a, const void* b, void* userdata);

char* cvSeqSearch( CvSeq* seq, const void* elem, CvCmpFunc func,
                   int is_sorted, int* elem_idx, void* userdata=NULL );
```

seq: The sequence elem: The element to look for func: The comparison function that returns negative, zero or positive value depending on the elements relation (see also cvSeqSort). is\_sorted: Whether the sequence is sorted or not. elem\_idx: Output parameter; index of the found element. userdata: The user parameter passed to the comparison function; helps to avoid global variables in some cases.

The function `cvSeqSearch` searches the element in the sequence.
If the sequence is sorted, binary O(log(N)) search is used, otherwise, a simple linear search is used.
If the element is not found, the function returns NULL pointer and the index is set to the number of
sequence elements if the linear search is used, and to the smallest index `i, seq(i)>elem`.

---

### StartAppendToSeq

Initializes process of writing data to sequence

```
void cvStartAppendToSeq( CvSeq* seq, CvSeqWriter* writer );
```

seq: Pointer to the sequence. writer: Writer state; initialized by the function.

The function `cvStartAppendToSeq` initializes the process of writing data to the sequence.
Written elements are added to the end of the sequence by `CV_WRITE_SEQ_ELEM( written_elem, writer )` macro.
Note that during the writing process other operations on the sequence may yield incorrect result or
even corrupt the sequence (see description of cvFlushSeqWriter that helps to avoid
some of these problems).

---

### StartWriteSeq

Creates new sequence and initializes writer for it

```
void cvStartWriteSeq( int seq_flags, int header_size, int elem_size,
                      CvMemStorage* storage, CvSeqWriter* writer );
```

seq\_flags: Flags of the created sequence. If the sequence is not passed to any function working with a specific type of sequences, the sequence value may be equal to 0, otherwise the appropriate type must be selected from the list of predefined sequence types. header\_size: Size of the sequence header. The parameter value may not be less than `sizeof(CvSeq)`. If a certain type or extension is specified, it must fit the base type header. elem\_size: Size of the sequence elements in bytes; must be consistent with the sequence type. For example, if the sequence of points is created (element type `CV_SEQ_ELTYPE_POINT` ), then the parameter elem\_size must be equal to `sizeof(CvPoint)`. storage: Sequence location. writer: Writer state; initialized by the function.

The function `cvStartWriteSeq` is a composition of cvCreateSeq and cvStartAppendToSeq.
The pointer to the created sequence is stored at `writer->seq` and is also returned
by cvEndWriteSeq function that should be called in the end.

---

### EndWriteSeq

Finishes process of writing sequence

```
CvSeq* cvEndWriteSeq( CvSeqWriter* writer );
```

writer: Writer state

The function `cvEndWriteSeq` finishes the writing process and returns the pointer to
the written sequence. The function also truncates the last incomplete sequence block to
return the remaining part of the block to the memory storage. After that the sequence
can be read and modified safely.

---

### FlushSeqWriter

Updates sequence headers from the writer state

```
void cvFlushSeqWriter( CvSeqWriter* writer );
```

writer: Writer state

The function `cvFlushSeqWriter` is intended to enable the user to read sequence
elements, whenever required, during the writing process, e.g., in order to check
specific conditions. The function updates the sequence headers to make reading
from the sequence possible. The writer is not closed, however, so that the
writing process can be continued any time. If some algorithm requires often flushes,
consider using cvSeqPush instead.

---

### StartReadSeq

Initializes process of sequential reading from sequence

```
void cvStartReadSeq( const CvSeq* seq, CvSeqReader* reader, int reverse=0 );
```

seq: Sequence. reader: Reader state; initialized by the function. reverse: Determines the direction of the sequence traversal. If `reverse` is 0, the reader is positioned at the first sequence element, otherwise it is positioned at the last element.

The function `cvStartReadSeq` initializes the reader state. After that all the
sequence elements from the first down to the last one can be read by subsequent
calls of the macro `CV_READ_SEQ_ELEM( read_elem, reader )` in case of forward reading
and by using `CV_REV_READ_SEQ_ELEM( read_elem, reader )` in case of reversed reading.
Both macros put the sequence element to `read_elem` and move the
reading pointer toward the next element.
A circular structure of sequence blocks is used for the reading process, that
is, after the last element has been read by the macro `CV_READ_SEQ_ELEM`, the
first element is read when the macro is called again. The same applies to
`CV_REV_READ_SEQ_ELEM` . There is no function to finish the reading process,
since it neither changes the sequence nor creates any temporary buffers. The reader
field `ptr` points to the current element of the sequence that is to be read
next. The code below demonstrates how to use sequence writer and reader.

```
CvMemStorage* storage = cvCreateMemStorage(0);
CvSeq* seq = cvCreateSeq( CV_32SC1, sizeof(CvSeq), sizeof(int), storage );
CvSeqWriter writer;
CvSeqReader reader;
int i;

cvStartAppendToSeq( seq, &writer );
for( i = 0; i < 10; i++ )
{
    int val = rand()%100;
    CV_WRITE_SEQ_ELEM( val, writer );
    printf("%d is written\n", val );
}
cvEndWriteSeq( &writer );

cvStartReadSeq( seq, &reader, 0 );
for( i = 0; i < seq->total; i++ )
{
    int val;
#if 1
    CV_READ_SEQ_ELEM( val, reader );
    printf("%d is read\n", val );
#else /* alternative way, that is preferable if sequence elements are large,
         or their size/type is unknown at compile time */
    printf("%d is read\n", *(int*)reader.ptr );
    CV_NEXT_SEQ_ELEM( seq->elem_size, reader );
#endif
}
...

cvReleaseStorage( &storage );
```

---

### GetSeqReaderPos

Returns the current reader position

```
int cvGetSeqReaderPos( CvSeqReader* reader );
```

reader: Reader state.

The function `cvGetSeqReaderPos` returns the current reader position
(within 0 ... `reader->seq->total` - 1).

---

### SetSeqReaderPos

Moves the reader to specified position

```
void cvSetSeqReaderPos( CvSeqReader* reader, int index, int is_relative=0 );
```

reader: Reader state. index: The destination position. If the positioning mode is used (see the next parameter) the actual position will be `index` mod `reader->seq->total`. is\_relative: If it is not zero, then `index` is a relative to the current position.

The function `cvSetSeqReaderPos` moves the read position to the absolute position or
relative to the current position.

---

## Sets

---

### CvSet

Collection of nodes

```
    typedef struct CvSetElem
    {
        int flags; /* it is negative if the node is free and zero or positive otherwise */
        struct CvSetElem* next_free; /* if the node is free, the field is a
                                        pointer to next free node */
    }
    CvSetElem;

    #define CV_SET_FIELDS()    \
        CV_SEQUENCE_FIELDS()   /* inherits from CvSeq */ \
        struct CvSetElem* free_elems; /* list of free nodes */

    typedef struct CvSet
    {
        CV_SET_FIELDS()
    } CvSet;
```

The structure CvSet is a base for OpenCV sparse data structures.

As follows from the above declaration CvSet inherits from CvSeq
and it adds `free_elems` field it to, which is a list of free nodes.
Every set node, whether free or not, is the element of the underlying sequence.
While there is no restrictions on elements of dense sequences, the set (and derived structures)
elements must start with integer field and be able to fit CvSetElem structure, because
these two fields (integer followed by the pointer) are required for organization of node set with
the list of free nodes. If a node is free, `flags` field is negative (the most-significant
bit, or MSB, of the field is set), and `next_free`
points to the next free node (the first free node is referenced by `free_elems` field of
CvSet). And if a node is occupied, `flags` field is positive and contains the node index
that may be retrieved using (set\_elem->flags & CV\_SET\_ELEM\_IDX\_MASK) expression,
the rest of the node content is determined by the user. In particular, the occupied nodes
are not linked as the free nodes are, so the second field can be used for such a link as well as
for some different purpose. The macro `CV_IS_SET_ELEM(set_elem_ptr)`
can be used to determined whether the specified node is occupied or not.

Initially the set and the list are empty. When a new node is requested from the set,
it is taken from the list of free nodes, which is updated then. If the list appears to be empty,
a new sequence block is allocated and all the nodes within the block are joined in the list of free
nodes. Thus, `total` field of the set is the total number of nodes both occupied and free.
When an occupied node is released, it is added to the list of free nodes. The node released last
will be occupied first.

In OpenCV CvSet is used for representing graphs (CvGraph),
sparse multi-dimensional arrays (CvSparseMat), planar subdivisions (CvSubdiv2D) etc.

---

### CreateSet

Creates empty set

```
CvSet* cvCreateSet( int set_flags, int header_size,
                    int elem_size, CvMemStorage* storage );
```

set\_flags: Type of the created set. header\_size: Set header size; may not be less than `sizeof(CvSet)`. elem\_size: Set element size; may not be less than CvSetElem. storage: Container for the set.

The function `cvCreateSet` creates an empty set with a specified header size and element size, and
returns the pointer to the set. The function is just a thin layer on top of cvCreateSeq.

---

### SetAdd

Occupies a node in the set

```
int cvSetAdd( CvSet* set_header, CvSetElem* elem=NULL, CvSetElem** inserted_elem=NULL );
```

set\_header: Set. elem: Optional input argument, inserted element. If not NULL, the function copies the data to the allocated node (The MSB of the first integer field is cleared after copying). inserted\_elem: Optional output argument; the pointer to the allocated cell.

The function `cvSetAdd` allocates a new node, optionally copies input element data
to it, and returns the pointer and the index to the node. The index value is
taken from the lower bits of `flags` field of the node. The function has O(1) complexity,
however there exists a faster function for allocating set nodes (see cvSetNew).

---

### SetRemove

Removes element from set

```
void cvSetRemove( CvSet* set_header, int index );
```

set\_header: Set. index: Index of the removed element.

The function `cvSetRemove` removes an element with a specified index from the set.
If the node at the specified location is not occupied the function does nothing.
The function has O(1) complexity, however, cvSetRemoveByPtr provides yet
faster way to remove a set element if it is located already.

---

### SetNew

Adds element to set (fast variant)

```
CvSetElem* cvSetNew( CvSet* set_header );
```

set\_header: Set.

The function `cvSetNew` is inline light-weight variant of cvSetAdd.
It occupies a new node and returns pointer to it rather than index.

---

### SetRemoveByPtr

Removes set element given its pointer

```
void cvSetRemoveByPtr( CvSet* set_header, void* elem );
```

set\_header: Set. elem: Removed element.

The function `cvSetRemoveByPtr` is inline light-weight variant of cvSetRemove
that takes element pointer.
The function does not check whether the node is occupied or not - the user should take care of it.

---

### GetSetElem

Finds set element by its index

```
CvSetElem* cvGetSetElem( const CvSet* set_header, int index );
```

set\_header: Set. index: Index of the set element within a sequence.

The function `cvGetSetElem` finds a set element by index. The function returns the
pointer to it or 0 if the index is invalid or the corresponding node is free.
The function supports negative indices as it uses cvGetSeqElem to locate the node.

---

### ClearSet

Clears set

```
void cvClearSet( CvSet* set_header );
```

set\_header: Cleared set.

The function `cvClearSet` removes all elements from set. It has O(1) time complexity.

---

## Graphs

---

### CvGraph

Oriented or undirected weighted graph

```
    #define CV_GRAPH_VERTEX_FIELDS()    \
        int flags; /* vertex flags */   \
        struct CvGraphEdge* first; /* the first incident edge */

    typedef struct CvGraphVtx
    {
        CV_GRAPH_VERTEX_FIELDS()
    }
    CvGraphVtx;

    #define CV_GRAPH_EDGE_FIELDS()      \
        int flags; /* edge flags */     \
        float weight; /* edge weight */ \
        struct CvGraphEdge* next[2]; /* the next edges in the incidence lists for staring (0) */ \
                                     /* and ending (1) vertices */ \
        struct CvGraphVtx* vtx[2]; /* the starting (0) and ending (1) vertices */

    typedef struct CvGraphEdge
    {
        CV_GRAPH_EDGE_FIELDS()
    }
    CvGraphEdge;

    #define  CV_GRAPH_FIELDS()                  \
        CV_SET_FIELDS() /* set of vertices */   \
        CvSet* edges;   /* set of edges */

    typedef struct CvGraph
    {
        CV_GRAPH_FIELDS()
    }
    CvGraph;
```

The structure CvGraph is a base for graphs used in OpenCV.

Graph structure inherits from CvSet - this part describes common graph properties and
the graph vertices, and contains another set as a member - this part describes the graph edges.

The vertex, edge and the graph header structures are declared using the same technique as other
extendible OpenCV structures - via macros, that simplifies extension and customization of the structures.
While the vertex and edge structures do not inherit from CvSetElem explicitly, they satisfy
both conditions on the set elements - have an integer field in the beginning and fit CvSetElem structure.
The `flags` fields are used as for indicating occupied vertices and edges as well as
for other purposes, for example, for graph traversal (see cvCreateGraphScanner et al.), so
it is better not to use them directly.

The graph is represented as a set of edges each of whose has the list of incident edges. The incidence
lists for different vertices are interleaved to avoid information duplication as much as possible.

The graph may be oriented or undirected. In the latter case there is no distinction between edge
connecting vertex A with vertex B and the edge connecting vertex B with vertex A - only one of them
can exist in the graph at the same moment and it represents both <A, B> and <B, A> edges..

---

### CreateGraph

Creates empty graph

```
CvGraph* cvCreateGraph( int graph_flags, int header_size, int vtx_size,
                        int edge_size, CvMemStorage* storage );
```

graph\_flags: Type of the created graph. Usually, it is either `CV_SEQ_KIND_GRAPH` for generic undirected graphs and `CV_SEQ_KIND_GRAPH | CV_GRAPH_FLAG_ORIENTED` for generic oriented graphs. header\_size: Graph header size; may not be less than `sizeof(CvGraph).` vtx\_size: Graph vertex size; the custom vertex structure must start with CvGraphVtx (use `CV_GRAPH_VERTEX_FIELDS()`) edge\_size: Graph edge size; the custom edge structure must start with CvGraphEdge (use `CV_GRAPH_EDGE_FIELDS()`) storage: The graph container.

The function `cvCreateGraph` creates an empty graph and returns pointer to it.

---

### GraphAddVtx

Adds vertex to graph

```
int cvGraphAddVtx( CvGraph* graph, const CvGraphVtx* vtx=NULL,
                   CvGraphVtx** inserted_vtx=NULL );
```

graph: Graph. vtx: Optional input argument used to initialize the added vertex (only user-defined fields beyond `sizeof(CvGraphVtx)` are copied). inserted\_vertex: Optional output argument. If not `NULL`, the address of the new vertex is written there.

The function `cvGraphAddVtx` adds a vertex to the graph and returns the vertex
index.

---

### GraphRemoveVtx

Removes vertex from graph

```
int cvGraphRemoveVtx( CvGraph* graph, int index );
```

graph: Graph. vtx\_idx: Index of the removed vertex.

The function `cvGraphRemoveAddVtx` removes a vertex from the graph together with all
the edges incident to it. The function reports an error, if the input vertex does
not belong to the graph. The return value is number of edges deleted,
or -1 if the vertex does not belong to the graph.

---

### GraphRemoveVtxByPtr

Removes vertex from graph

```
int cvGraphRemoveVtxByPtr( CvGraph* graph, CvGraphVtx* vtx );
```

graph: Graph. vtx: Pointer to the removed vertex.

The function `cvGraphRemoveVtxByPtr` removes a vertex from the graph together with
all the edges incident to it. The function reports an error, if the vertex does not belong to the graph.
The return value is number of edges deleted, or -1 if the vertex does not belong to the graph.

---

### GetGraphVtx

Finds graph vertex by index

```
CvGraphVtx* cvGetGraphVtx( CvGraph* graph, int vtx_idx );
```

graph: Graph. vtx\_idx: Index of the vertex.

The function `cvGetGraphVtx` finds the graph vertex by index and returns the pointer
to it or NULL if the vertex does not belong to the graph.

---

### GraphVtxIdx

Returns index of graph vertex

```
int cvGraphVtxIdx( CvGraph* graph, CvGraphVtx* vtx );
```

graph: Graph. vtx: Pointer to the graph vertex.

The function `cvGraphVtxIdx` returns index of the graph vertex.

---

### GraphAddEdge

Adds edge to graph

```
int cvGraphAddEdge( CvGraph* graph, int start_idx, int end_idx,
                    const CvGraphEdge* edge=NULL, CvGraphEdge** inserted_edge=NULL );
```

graph: Graph. start\_idx: Index of the starting vertex of the edge. end\_idx: Index of the ending vertex of the edge. For undirected graph the order of the vertex parameters does not matter. edge: Optional input parameter, initialization data for the edge. inserted\_edge: Optional output parameter to contain the address of the inserted edge.

The function `cvGraphAddEdge` connects two specified vertices.
The function returns 1 if the edge has been added successfully, 0 if the edge connecting
the two vertices exists already and -1 if either of the vertices was not found, the starting and
the ending vertex are the same or there is some other critical situation. In the latter case
(i.e. when the result is negative) the function also reports an error by default.

---

### GraphAddEdgeByPtr

Adds edge to graph

```
int cvGraphAddEdgeByPtr( CvGraph* graph, CvGraphVtx* start_vtx, CvGraphVtx* end_vtx,
                         const CvGraphEdge* edge=NULL, CvGraphEdge** inserted_edge=NULL );
```

graph: Graph. start\_vtx: Pointer to the starting vertex of the edge. end\_vtx: Pointer to the ending vertex of the edge. For undirected graph the order of the vertex parameters does not matter. edge: Optional input parameter, initialization data for the edge. inserted\_edge: Optional output parameter to contain the address of the inserted edge within the edge set.

The function `cvGraphAddEdge` connects two specified vertices.
The function returns 1 if the edge has been added successfully, 0 if the edge connecting
the two vertices exists already and -1 if either of the vertices was not found, the starting and
the ending vertex are the same or there is some other critical situation. In the latter case
(i.e. when the result is negative) the function also reports an error by default.

---

### GraphRemoveEdge

Removes edge from graph

```
void cvGraphRemoveEdge( CvGraph* graph, int start_idx, int end_idx );
```

graph: Graph. start\_idx: Index of the starting vertex of the edge. end\_idx: Index of the ending vertex of the edge. For undirected graph the order of the vertex parameters does not matter.

The function `cvGraphRemoveEdge` removes the edge connecting two specified vertices.
If the vertices are not connected [in that order], the function does nothing.

---

### GraphRemoveEdgeByPtr

Removes edge from graph

```
void cvGraphRemoveEdgeByPtr( CvGraph* graph, CvGraphVtx* start_vtx, CvGraphVtx* end_vtx );
```

graph: Graph. start\_vtx: Pointer to the starting vertex of the edge. end\_vtx: Pointer to the ending vertex of the edge. For undirected graph the order of the vertex parameters does not matter.

The function `cvGraphRemoveEdgeByPtr` removes the edge connecting two specified vertices.
If the vertices are not connected [in that order], the function does nothing.

---

### FindGraphEdge

Finds edge in graph

```
CvGraphEdge* cvFindGraphEdge( const CvGraph* graph, int start_idx, int end_idx );
#define cvGraphFindEdge cvFindGraphEdge
```

graph: Graph. start\_idx: Index of the starting vertex of the edge. end\_idx: Index of the ending vertex of the edge. For undirected graph the order of the vertex parameters does not matter.

The function `cvFindGraphEdge` finds the graph edge connecting two specified vertices
and returns pointer to it or NULL if the edge does not exists.

---

### FindGraphEdgeByPtr

Finds edge in graph

```
CvGraphEdge* cvFindGraphEdgeByPtr( const CvGraph* graph, const CvGraphVtx* start_vtx,
                                   const CvGraphVtx* end_vtx );
#define cvGraphFindEdgeByPtr cvFindGraphEdgeByPtr
```

graph: Graph. start\_vtx: Pointer to the starting vertex of the edge. end\_vtx: Pointer to the ending vertex of the edge. For undirected graph the order of the vertex parameters does not matter.

The function `cvFindGraphEdge` finds the graph edge connecting two specified vertices
and returns pointer to it or NULL if the edge does not exists.

---

### GraphEdgeIdx

Returns index of graph edge

```
int cvGraphEdgeIdx( CvGraph* graph, CvGraphEdge* edge );
```

graph: Graph. edge: Pointer to the graph edge.

The function `cvGraphEdgeIdx` returns index of the graph edge.

---

### GraphVtxDegree

Counts edges incident to the vertex

```
int cvGraphVtxDegree( const CvGraph* graph, int vtx_idx );
```

graph: Graph. vtx: Index of the graph vertex.

The function `cvGraphVtxDegree` returns the number of edges
incident to the specified vertex, both incoming and outgoing.
To count the edges, the following code is used:

```
    CvGraphEdge* edge = vertex->first; int count = 0;
    while( edge )
    {
        edge = CV_NEXT_GRAPH_EDGE( edge, vertex );
        count++;
    }
```

The macro `CV_NEXT_GRAPH_EDGE( edge, vertex )` returns the edge incident to `vertex`
that follows after `edge`.

---

### GraphVtxDegreeByPtr

Finds edge in graph

```
int cvGraphVtxDegreeByPtr( const CvGraph* graph, const CvGraphVtx* vtx );
```

graph: Graph. vtx: Pointer to the graph vertex.

The function `cvGraphVtxDegree` returns the number of edges
incident to the specified vertex, both incoming and outgoing.

---

### ClearGraph

Clears graph

```
void cvClearGraph( CvGraph* graph );
```

graph: Graph.

The function `cvClearGraph` removes all vertices and edges from the graph.
The function has O(1) time complexity.

---

### CloneGraph

Clone graph

```
CvGraph* cvCloneGraph( const CvGraph* graph, CvMemStorage* storage );
```

graph: The graph to copy. storage: Container for the copy.

The function `cvCloneGraph` creates full copy of the graph. If the graph vertices
or edges have pointers to some external data, it still be shared between the copies.
The vertex and edge indices in the new graph may be different from the original, because
the function defragments the vertex and edge sets.

---

### CvGraphScanner

Graph traversal state

```
    typedef struct CvGraphScanner
    {
        CvGraphVtx* vtx;       /* current graph vertex (or current edge origin) */
        CvGraphVtx* dst;       /* current graph edge destination vertex */
        CvGraphEdge* edge;     /* current edge */

        CvGraph* graph;        /* the graph */
        CvSeq*   stack;        /* the graph vertex stack */
        int      index;        /* the lower bound of certainly visited vertices */
        int      mask;         /* event mask */
    }
    CvGraphScanner;
```

The structure CvGraphScanner is used for depth-first graph traversal.
See discussion of the functions below.

---

### CreateGraphScanner

Creates structure for depth-first graph traversal

```
CvGraphScanner*  cvCreateGraphScanner( CvGraph* graph, CvGraphVtx* vtx=NULL,
                                       int mask=CV_GRAPH_ALL_ITEMS );
```

graph: Graph. vtx: Initial vertex to start from. If NULL, the traversal starts from the first vertex (a vertex with the minimal index in the sequence of vertices). mask: Event mask indicating which events are interesting to the user (where cvNextGraphItem function returns control to the user) It can be `CV_GRAPH_ALL_ITEMS` (all events are interesting) or combination of the following flags: - CV\_GRAPH\_VERTEX - stop at the graph vertices visited for the first time - CV\_GRAPH\_TREE\_EDGE - stop at tree edges (`tree edge` is the edge connecting the last visited vertex and the vertex to be visited next) - CV\_GRAPH\_BACK\_EDGE - stop at back edges (`back edge` is an edge connecting the last visited vertex with some of its ancestors in the search tree) - CV\_GRAPH\_FORWARD\_EDGE - stop at forward edges (`forward edge` is an edge connecting the last visited vertex with some of its descendants in the search tree). The `forward edges` are only possible during oriented graph traversal) - CV\_GRAPH\_CROSS\_EDGE - stop at cross edges (`cross edge` is an edge connecting different search trees or branches of the same tree. The `cross edges` are only possible during oriented graphs traversal) - CV\_GRAPH\_ANY\_EDGE - stop and any edge (`tree, back, forward` and `cross edges`) - CV\_GRAPH\_NEW\_TREE - stop in the beginning of every new search tree. When the traversal procedure visits all vertices and edges reachable from the initial vertex (the visited vertices together with tree edges make up a tree), it searches for some unvisited vertex in the graph and resumes the traversal process from that vertex. Before starting a new tree (including the very first tree when `cvNextGraphItem` is called for the first time) it generates `CV_GRAPH_NEW_TREE` event. For undirected graphs each search tree corresponds to a connected component of the graph. - CV\_GRAPH\_BACKTRACKING - stop at every already visited vertex during backtracking - returning to already visited vertexes of the traversal tree.

The function `cvCreateGraphScanner` creates structure for
depth-first graph traversal/search.
The initialized structure is used in cvNextGraphItem function
- the incremental traversal procedure.

---

### NextGraphItem

Makes one or more steps of the graph traversal procedure

```
int cvNextGraphItem( CvGraphScanner* scanner );
```

scanner: Graph traversal state. It is updated by the function.

The function `cvNextGraphItem` traverses through the graph until an event interesting to the user
(that is, an event, specified in the `mask` in cvCreateGraphScanner call)
is met or the traversal is over. In the first case it returns one of the events,
listed in the description of `mask` parameter above and with the next call
it resumes the traversal. In the latter case it returns CV\_GRAPH\_OVER (-1).
When the event is `CV_GRAPH_VERTEX`, or `CV_GRAPH_BACKTRACKING` or `CV_GRAPH_NEW_TREE`,
the currently observed vertex is stored in `scanner->vtx`. And if the event is edge-related,
the edge itself is stored at `scanner->edge`,
the previously visited vertex - at `scanner->vtx` and the other ending vertex of the edge -
at `scanner->dst`.

---

### ReleaseGraphScanner

Finishes graph traversal procedure

```
void cvReleaseGraphScanner( CvGraphScanner** scanner );
```

scanner: Double pointer to graph traverser.

The function `cvGraphScanner` finishes graph traversal procedure
and releases the traverser state.

---

## Trees

---

### CV\_TREE\_NODE\_FIELDS

Helper macro for a tree node type declaration

```
#define CV_TREE_NODE_FIELDS(node_type)                          \
    int       flags;         /* miscellaneous flags */          \
    int       header_size;   /* size of sequence header */      \
    struct    node_type* h_prev; /* previous sequence */        \
    struct    node_type* h_next; /* next sequence */            \
    struct    node_type* v_prev; /* 2nd previous sequence */    \
    struct    node_type* v_next; /* 2nd next sequence */
```

The macro `CV_TREE_NODE_FIELDS()` is used to declare structures
that can be organized into hierarchical structures (trees), such as CvSeq -
the basic type for all dynamical structures.
The trees made of nodes declared using this macro can be processed using
the functions described below in this section.

---

### CvTreeNodeIterator

Opens existing or creates new file storage

```
typedef struct CvTreeNodeIterator
{
    const void* node;
    int level;
    int max_level;
}
CvTreeNodeIterator;
```

The structure CvTreeNodeIterator is used to traverse trees.
The tree node declaration should start with `CV_TREE_NODE_FIELDS(...)` macro.

---

### InitTreeNodeIterator

Initializes tree node iterator

```
void cvInitTreeNodeIterator( CvTreeNodeIterator* tree_iterator,
                             const void* first, int max_level );
```

tree\_iterator: Tree iterator initialized by the function. first: The initial node to start traversing from. max\_level: The maximal level of the tree (`first` node assumed to be at the first level) to traverse up to. For example, 1 means that only nodes at the same level as `first` should be visited, 2 means that the nodes on the same level as `first` and their direct children should be visited etc.

The function `cvInitTreeNodeIterator` initializes tree iterator.
The tree is traversed in depth-first order.

---

### NextTreeNode

Returns the currently observed node and moves iterator toward the next node

```
void* cvNextTreeNode( CvTreeNodeIterator* tree_iterator );
```

tree\_iterator: Tree iterator initialized by the function.

The function `cvNextTreeNode` returns the currently observed node and then
updates the iterator - moves it toward the next node. In other words, the function behavior
is similar to \*p++ expression on usual C pointer or C++ collection iterator.
The function returns NULL if there is no more nodes.

---

### PrevTreeNode

Returns the currently observed node and moves iterator toward the previous node

```
void* cvPrevTreeNode( CvTreeNodeIterator* tree_iterator );
```

tree\_iterator: Tree iterator initialized by the function.

The function `cvPrevTreeNode` returns the currently observed node and then
updates the iterator - moves it toward the previous node. In other words, the function behavior
is similar to \*p-- expression on usual C pointer or C++ collection iterator.
The function returns NULL if there is no more nodes.

---

### TreeToNodeSeq

Gathers all node pointers to the single sequence

```
CvSeq* cvTreeToNodeSeq( const void* first, int header_size, CvMemStorage* storage );
```

first: The initial tree node. header\_size: Header size of the created sequence (sizeof(CvSeq) is the most used value). storage: Container for the sequence.

The function `cvTreeToNodeSeq` puts pointers of all nodes reachable from `first`
to the single sequence. The pointers are written subsequently in the depth-first order.

---

### InsertNodeIntoTree

Adds new node to the tree

```
void cvInsertNodeIntoTree( void* node, void* parent, void* frame );
```

node: The inserted node. parent: The parent node that is already in the tree. frame: The top level node. If `parent` and `frame` are the same, `v_prev` field of `node` is set to NULL rather than `parent`.

The function `cvInsertNodeIntoTree` adds another node into tree. The function does not
allocate any memory, it can only modify links of the tree nodes.

---

### RemoveNodeFromTree

Removes node from tree

```
void cvRemoveNodeFromTree( void* node, void* frame );
```

node: The removed node. frame: The top level node. If `node->v_prev = NULL` and `node->h_prev` is NULL (i.e. if `node` is the first child of `frame`), `frame->v_next` is set to `node->h_next` (i.e. the first child or frame is changed).

The function `cvRemoveNodeFromTree` removes node from tree. The function does not
deallocate any memory, it can only modify links of the tree nodes.

---

# Drawing Functions

Drawing functions work with matrices/images or arbitrary depth.
Antialiasing is implemented only for 8-bit images.
All the functions include parameter color that means rgb value (that may be
constructed with `CV_RGB` macro or `cvScalar` function)
for color images and brightness for grayscale images.

If a drawn figure is partially or completely outside the image, it is clipped.
For color images the order channel is: Blue Green Red ...
If one needs a different channel order, it is possible to construct color
via `cvScalar` with the particular channel order, or
convert the image before and/or after drawing in it with
cvCvtColor or
cvTransform.

---

## Curves and Shapes

---

### CV\_RGB

Constructs a color value

```
#define CV_RGB( r, g, b )  cvScalar( (b), (g), (r) )
```

---

### Line

Draws a line segment connecting two points

```
void cvLine( CvArr* img, CvPoint pt1, CvPoint pt2, CvScalar color,
             int thickness=1, int line_type=8, int shift=0 );
```

img: The image. pt1: First point of the line segment. pt2: Second point of the line segment. color: Line color. thickness: Line thickness. line\_type: Type of the line: `8` (or `0`) - 8-connected line. `4` - 4-connected line. `CV_AA` - antialiased line. shift: Number of fractional bits in the point coordinates.

The function `cvLine` draws the line segment between `pt1` and `pt2` points
in the image. The line is clipped by the image or ROI rectangle. For non-antialiased lines
with integer coordinates the 8-connected or 4-connected Bresenham algorithm is used.
Thick lines are drawn with rounding endings. Antialiased lines are drawn using Gaussian filtering.
To specify the line color, the user may use the macro `CV_RGB( r, g, b )`.

---

### Rectangle

Draws simple, thick or filled rectangle

```
void cvRectangle( CvArr* img, CvPoint pt1, CvPoint pt2, CvScalar color,
                  int thickness=1, int line_type=8, int shift=0 );
```

img: Image. pt1: One of the rectangle vertices. pt2: Opposite rectangle vertex. color: Line color (RGB) or brightness (grayscale image). thickness: Thickness of lines that make up the rectangle. Negative values, e.g. CV\_FILLED, make the function to draw a filled rectangle. line\_type: Type of the line, see cvLine description. shift: Number of fractional bits in the point coordinates.

The function `cvRectangle` draws a rectangle with
two opposite corners `pt1` and `pt2`.

---

### Circle

Draws a circle

```
void cvCircle( CvArr* img, CvPoint center, int radius, CvScalar color,
               int thickness=1, int line_type=8, int shift=0 );
```

img: Image where the circle is drawn. center: Center of the circle. radius: Radius of the circle. color: Circle color. thickness: Thickness of the circle outline if positive, otherwise indicates that a filled circle has to be drawn. line\_type: Type of the circle boundary, see cvLine description. shift: Number of fractional bits in the center coordinates and radius value.

The function `cvCircle` draws a simple or filled circle with given center and
radius. The circle is clipped by ROI rectangle. To specify the circle color, the user may
use the macro `CV_RGB ( r, g, b )`.

---

### Ellipse

Draws simple or thick elliptic arc or fills ellipse sector

```
void cvEllipse( CvArr* img, CvPoint center, CvSize axes, double angle,
                double start_angle, double end_angle, CvScalar color,
                int thickness=1, int line_type=8, int shift=0 );
```

img: Image. center: Center of the ellipse. axes: Length of the ellipse axes. angle: Rotation angle. start\_angle: Starting angle of the elliptic arc. end\_angle: Ending angle of the elliptic arc. color: Ellipse color. thickness: Thickness of the ellipse arc. line\_type: Type of the ellipse boundary, see cvLine description. shift: Number of fractional bits in the center coordinates and axes' values.

The function `cvEllipse` draws a simple or thick elliptic arc or fills an ellipse
sector. The arc is clipped by ROI rectangle. A piecewise-linear
approximation is used for antialiased arcs and thick arcs. All the angles are
given in degrees. The picture below explains the meaning of the parameters.

Parameters of Elliptic Arc

---

### EllipseBox

Draws simple or thick elliptic arc or fills ellipse sector

```
void cvEllipseBox( CvArr* img, CvBox2D box, CvScalar color,
                   int thickness=1, int line_type=8, int shift=0 );
```

img: Image. box: The enclosing box of the ellipse drawn thickness: Thickness of the ellipse boundary. line\_type: Type of the ellipse boundary, see cvLine description. shift: Number of fractional bits in the box vertex coordinates.

The function `cvEllipseBox` draws a simple or thick ellipse outline,
or fills an ellipse. The functions provides a convenient way to draw an ellipse
approximating some shape; that is what cvCamShift
and cvFitEllipse do.
The ellipse drawn is clipped by ROI rectangle. A piecewise-linear
approximation is used for antialiased arcs and thick arcs.

---

### FillPoly

Fills polygons interior

```
void cvFillPoly( CvArr* img, CvPoint** pts, int* npts, int contours,
                 CvScalar color, int line_type=8, int shift=0 );
```

img: Image. pts: Array of pointers to polygons. npts: Array of polygon vertex counters. contours: Number of contours that bind the filled region. color: Polygon color. line\_type: Type of the polygon boundaries, see cvLine description. shift: Number of fractional bits in the vertex coordinates.

The function `cvFillPoly` fills an area bounded by several polygonal contours.
The function fills complex areas, for example, areas with holes, contour self-intersection, etc.

---

### FillConvexPoly

Fills convex polygon

```
void cvFillConvexPoly( CvArr* img, CvPoint* pts, int npts,
                       CvScalar color, int line_type=8, int shift=0 );
```

img: Image. pts: Array of pointers to a single polygon. npts: Polygon vertex counter. color: Polygon color. line\_type: Type of the polygon boundaries, see cvLine description. shift: Number of fractional bits in the vertex coordinates.

The function `cvFillConvexPoly` fills convex polygon interior.
This function is much faster than The function `cvFillPoly` and can fill
not only the convex polygons but any monotonic polygon, i.e. a polygon whose contour intersects every
horizontal line (scan line) twice at the most.

---

### PolyLine

Draws simple or thick polygons

```
void cvPolyLine( CvArr* img, CvPoint** pts, int* npts, int contours, int is_closed,
                 CvScalar color, int thickness=1, int line_type=8, int shift=0 );
```

img: Image. pts: Array of pointers to polylines. npts: Array of polyline vertex counters. contours: Number of polyline contours. is\_closed: Indicates whether the polylines must be drawn closed. If closed, the function draws the line from the last vertex of every contour to the first vertex. color: Polyline color. thickness: Thickness of the polyline edges. line\_type: Type of the line segments, see cvLine description. shift: Number of fractional bits in the vertex coordinates.

The function `cvPolyLine` draws a single or multiple polygonal curves.

---

## Text

---

### InitFont

Initializes font structure

```
void cvInitFont( CvFont* font, int font_face, double hscale,
                 double vscale, double shear=0,
                 int thickness=1, int line_type=8 );
```

font: Pointer to the font structure initialized by the function. font\_face: Font name identifier. Only a subset of Hershey fonts (http://sources.isc.org/utils/misc/hershey-font.txt) are supported now: `CV_FONT_HERSHEY_SIMPLEX` - normal size sans-serif font `CV_FONT_HERSHEY_PLAIN` - small size sans-serif font `CV_FONT_HERSHEY_DUPLEX` - normal size sans-serif font (more complex than `CV_FONT_HERSHEY_SIMPLEX`) `CV_FONT_HERSHEY_COMPLEX` - normal size serif font `CV_FONT_HERSHEY_TRIPLEX` - normal size serif font (more complex than `CV_FONT_HERSHEY_COMPLEX`) `CV_FONT_HERSHEY_COMPLEX_SMALL` - smaller version of `CV_FONT_HERSHEY_COMPLEX` `CV_FONT_HERSHEY_SCRIPT_SIMPLEX` - hand-writing style font `CV_FONT_HERSHEY_SCRIPT_COMPLEX` - more complex variant of `CV_FONT_HERSHEY_SCRIPT_SIMPLEX` The parameter can be composed from one of the values above and optional `CV_FONT_ITALIC` flag, that means italic or oblique font. hscale: Horizontal scale. If equal to `1.0f`, the characters have the original width depending on the font type. If equal to `0.5f`, the characters are of half the original width. vscale: Vertical scale. If equal to `1.0f`, the characters have the original height depending on the font type. If equal to `0.5f`, the characters are of half the original height. shear: Approximate tangent of the character slope relative to the vertical line. Zero value means a non-italic font, `1.0f` means `≈45°` slope, etc. thickness Thickness of lines composing letters outlines. The function `cvLine` is used for drawing letters. thickness: Thickness of the text strokes. line\_type: Type of the strokes, see cvLine description.

The function `cvInitFont` initializes the font structure that can be passed to
text rendering functions.

---

### PutText

Draws text string

```
void cvPutText( CvArr* img, const char* text, CvPoint org, const CvFont* font, CvScalar color );
```

img: Input image. text: String to print. org: Coordinates of the bottom-left corner of the first letter. font: Pointer to the font structure. color: Text color.

The function `cvPutText` renders the text in the image with the specified font and
color. The printed text is clipped by ROI rectangle. Symbols that do not belong
to the specified font are replaced with the rectangle symbol.

---

### GetTextSize

Retrieves width and height of text string

```
void cvGetTextSize( const char* text_string, const CvFont* font, CvSize* text_size, int* baseline );
```

font: Pointer to the font structure. text\_string: Input string. text\_size: Resultant size of the text string. Height of the text does not include the height of character parts that are below the baseline. baseline: y-coordinate of the baseline relatively to the bottom-most text point.

The function `cvGetTextSize` calculates the binding rectangle for the given text
string when a specified font is used.

---

## Point Sets and Contours

---

### DrawContours

Draws contour outlines or interiors in the image

```
void cvDrawContours( CvArr *img, CvSeq* contour,
                     CvScalar external_color, CvScalar hole_color,
                     int max_level, int thickness=1,
                     int line_type=8, CvPoint offset=cvPoint(0,0) );
```

img: Image where the contours are to be drawn. Like in any other drawing function, the contours are clipped with the ROI. contour: Pointer to the first contour. external\_color: Color of the external contours. hole\_color: Color of internal contours (holes). max\_level: Maximal level for drawn contours. If 0, only `contour` is drawn. If 1, the contour and all contours after it on the same level are drawn. If 2, all contours after and all contours one level below the contours are drawn, etc. If the value is negative, the function does not draw the contours following after `contour` but draws child contours of `contour` up to abs(`max_level`)-1 level. thickness: Thickness of lines the contours are drawn with. If it is negative (e.g. =CV\_FILLED), the contour interiors are drawn. line\_type: Type of the contour segments, see cvLine description. offset: Shift all the point coordinates by the specified value. It is useful in case if the contours retrieved in some image ROI and then the ROI offset needs to be taken into account during the rendering.

The function `cvDrawContours` draws contour outlines in the image if `thickness`>=0
or fills area bounded by the contours if `thickness`<0.

#### Example. Connected component detection via contour functions

```
#include "cv.h"
#include "highgui.h"

int main( int argc, char** argv )
{
    IplImage* src;
    // the first command line parameter must be file name of binary (black-n-white) image
    if( argc == 2 && (src=cvLoadImage(argv[1], 0))!= 0)
    {
        IplImage* dst = cvCreateImage( cvGetSize(src), 8, 3 );
        CvMemStorage* storage = cvCreateMemStorage(0);
        CvSeq* contour = 0;

        cvThreshold( src, src, 1, 255, CV_THRESH_BINARY );
        cvNamedWindow( "Source", 1 );
        cvShowImage( "Source", src );

        cvFindContours( src, storage, &contour, sizeof(CvContour), CV_RETR_CCOMP, CV_CHAIN_APPROX_SIMPLE );
        cvZero( dst );

        for( ; contour != 0; contour = contour->h_next )
        {
            CvScalar color = CV_RGB( rand()&255, rand()&255, rand()&255 );
            /* replace CV_FILLED with 1 to see the outlines */
            cvDrawContours( dst, contour, color, color, -1, CV_FILLED, 8 );
        }

        cvNamedWindow( "Components", 1 );
        cvShowImage( "Components", dst );
        cvWaitKey(0);
    }
}
```

Replace CV\_FILLED with 1 in the sample below to see the contour outlines

---

### InitLineIterator

Initializes line iterator

```
int cvInitLineIterator( const CvArr* image, CvPoint pt1, CvPoint pt2,
                        CvLineIterator* line_iterator, int connectivity=8,
                        int left_to_right=0 );
```

image: Image to sample the line from. pt1: First ending point of the line segment. pt2: Second ending point of the line segment. line\_iterator: Pointer to the line iterator state structure. connectivity: The scanned line connectivity, 4 or 8. left\_to\_right: The flag, indicating whether the line should be always scanned from the left-most point to the right-most out of `pt1` and `pt2` (`left_to_right≠0`), or it is scanned in the specified order, from `pt1` to `pt2` (`left_to_right=0`).

The function `cvInitLineIterator` initializes the line iterator and returns the
number of pixels between two end points. Both points must be inside the image.
After the iterator has been initialized, all the points on the raster line that
connects the two ending points may be retrieved by successive calls of
`CV_NEXT_LINE_POINT` point. The points on the line are calculated one by one using
4-connected or 8-connected Bresenham algorithm.

#### Example. Using line iterator to calculate sum of pixel values along the color line

```
    CvScalar sum_line_pixels( IplImage* image, CvPoint pt1, CvPoint pt2 )
    {
        CvLineIterator iterator;
        int blue_sum = 0, green_sum = 0, red_sum = 0;
        int count = cvInitLineIterator( image, pt1, pt2, &iterator, 8, 0 );

        for( int i = 0; i < count; i++ ){
            blue_sum += iterator.ptr[0];
            green_sum += iterator.ptr[1];
            red_sum += iterator.ptr[2];
            CV_NEXT_LINE_POINT(iterator);

            /* print the pixel coordinates: demonstrates how to calculate the coordinates */
            {
            int offset, x, y;
            /* assume that ROI is not set, otherwise need to take it into account. */
            offset = iterator.ptr - (uchar*)(image->imageData);
            y = offset/image->widthStep;
            x = (offset - y*image->widthStep)/(3*sizeof(uchar) /* size of pixel */);
            printf("(%d,%d)\n", x, y );
            }
        }
        return cvScalar( blue_sum, green_sum, red_sum );
    }
```

---

### ClipLine

Clips the line against the image rectangle

```
int cvClipLine( CvSize img_size, CvPoint* pt1, CvPoint* pt2 );
```

img\_size: Size of the image. pt1: First ending point of the line segment. It is modified by the function. pt2: Second ending point of the line segment. It is modified by the function.

The function `cvClipLine` calculates a part of the line segment which is entirely in the image.
It returns 0 if the line segment is completely outside the image and 1 otherwise.

---

### Ellipse2Poly

Approximates elliptic arc with polyline

```
int cvEllipse2Poly( CvPoint center, CvSize axes,
                    int angle, int arc_start,
                    int arc_end, CvPoint* pts, int delta );
```

center: Center of the arc. axes: Half-sizes of the arc. See cvEllipse. angle: Rotation angle of the ellipse in degrees. See cvEllipse. start\_angle: Starting angle of the elliptic arc. end\_angle: Ending angle of the elliptic arc. pts: The array of points, filled by the function. delta: Angle between the subsequent polyline vertices, approximation accuracy. So, the total number of output points will ceil((end\_angle - start\_angle)/delta) + 1 at max.

The function `cvEllipse2Poly` computes vertices of the polyline that approximates the specified elliptic arc.
It is used by cvEllipse.

---

### ClipLine

CVAPI(int) cvEllipse2Poly( CvPoint center, CvSize axes,
int angle, int arc\_start, int arc\_end, CvPoint \* pts, int delta );

---

# Data Persistence and RTTI

---

## File Storage

---

### CvFileStorage

File Storage

```
    typedef struct CvFileStorage
    {
        ...       // hidden fields
    } CvFileStorage;
```

The structure CvFileStorage is "black box" representation of
file storage that is associated with a file on disk. Several functions that are described below
take `CvFileStorage` on input and allow user to save or to load hierarchical collections
that consist of scalar values, standard CXCORE objects (such as matrices, sequences, graphs) and
user-defined objects.

CXCORE can read and write data in XML (http://www.w3c.org/XML)
or YAML (http://www.yaml.org) formats. Below is the example of
3×3 floating-point identity matrix `A`, stored in XML and YAML files using CXCORE functions:

**XML:**: ``` <?xml version="1.0"> <opencv_storage> <A type_id="opencv-matrix"> <rows>3</rows> <cols>3</cols> <dt>f</dt> <data>1. 0. 0. 0. 1. 0. 0. 0. 1.</data> </A> </opencv_storage> ``` **YAML:**: ``` %YAML:1.0 A: !!opencv-matrix rows: 3 cols: 3 dt: f data: [ 1., 0., 0., 0., 1., 0., 0., 0., 1.] ```

As it can be seen from the examples, XML uses nested tags to represent hierarchy,
while YAML uses indentation for that purpose (similarly to Python programming language).

The same CXCORE functions can read and write data in both formats, the particular format is determined
by the extension of the opened file, .xml for XML files and .yml or .yaml for YAML.

---

### CvFileNode

File Storage Node

```
/* file node type */
#define CV_NODE_NONE        0
#define CV_NODE_INT         1
#define CV_NODE_INTEGER     CV_NODE_INT
#define CV_NODE_REAL        2
#define CV_NODE_FLOAT       CV_NODE_REAL
#define CV_NODE_STR         3
#define CV_NODE_STRING      CV_NODE_STR
#define CV_NODE_REF         4 /* not used */
#define CV_NODE_SEQ         5
#define CV_NODE_MAP         6
#define CV_NODE_TYPE_MASK   7

/* optional flags */
#define CV_NODE_USER        16
#define CV_NODE_EMPTY       32
#define CV_NODE_NAMED       64

#define CV_NODE_TYPE(tag)  ((tag) & CV_NODE_TYPE_MASK)

#define CV_NODE_IS_INT(tag)        (CV_NODE_TYPE(tag) == CV_NODE_INT)
#define CV_NODE_IS_REAL(tag)       (CV_NODE_TYPE(tag) == CV_NODE_REAL)
#define CV_NODE_IS_STRING(tag)     (CV_NODE_TYPE(tag) == CV_NODE_STRING)
#define CV_NODE_IS_SEQ(tag)        (CV_NODE_TYPE(tag) == CV_NODE_SEQ)
#define CV_NODE_IS_MAP(tag)        (CV_NODE_TYPE(tag) == CV_NODE_MAP)
#define CV_NODE_IS_COLLECTION(tag) (CV_NODE_TYPE(tag) >= CV_NODE_SEQ)
#define CV_NODE_IS_FLOW(tag)       (((tag) & CV_NODE_FLOW) != 0)
#define CV_NODE_IS_EMPTY(tag)      (((tag) & CV_NODE_EMPTY) != 0)
#define CV_NODE_IS_USER(tag)       (((tag) & CV_NODE_USER) != 0)
#define CV_NODE_HAS_NAME(tag)      (((tag) & CV_NODE_NAMED) != 0)

#define CV_NODE_SEQ_SIMPLE 256
#define CV_NODE_SEQ_IS_SIMPLE(seq) (((seq)->flags & CV_NODE_SEQ_SIMPLE) != 0)

typedef struct CvString
{
    int len;
    char* ptr;
}
CvString;

/* all the keys (names) of elements in the read file storage
   are stored in the hash to speed up the lookup operations */
typedef struct CvStringHashNode
{
    unsigned hashval;
    CvString str;
    struct CvStringHashNode* next;
}
CvStringHashNode;

/* basic element of the file storage - scalar or collection */
typedef struct CvFileNode
{
    int tag;
    struct CvTypeInfo* info; /* type information
            (only for user-defined object, for others it is 0) */
    union
    {
        double f; /* scalar floating-point number */
        int i;    /* scalar integer number */
        CvString str; /* text string */
        CvSeq* seq; /* sequence (ordered collection of file nodes) */
        struct CvMap* map; /* map (collection of named file nodes) */
    } data;
}
CvFileNode;
```

The structure is used only for retrieving data from file storage (i.e. for loading data from file).
When data is written to file, it is done sequentially, with minimal buffering. No data is stored in the
file storage.

In opposite, when data is read from file, the whole file is parsed and represented in memory
as a tree. Every node of the tree is represented by CvFileNode. Type of the file node
`N` can be retrieved as `CV_NODE_TYPE(N->tag)`. Some file nodes (leaves) are scalars:
text strings, integer or floating-point numbers. Other file nodes are collections of file nodes, which can
be scalars or collections in their turn. There are two types of collections: sequences and maps
(we use YAML notation, however, the same is true for XML streams). Sequences (do not mix them with
CvSeq) are ordered collections of unnamed file nodes,
maps are unordered collections of named file nodes. Thus, elements of sequences are
accessed by index (cvGetSeqElem),
while elements of maps are accessed by name
(cvGetFileNodeByName).
The table below describes the different types of a file node:

|  |  |  |
| --- | --- | --- |
| Type | CV\_NODE\_TYPE(node->tag) | Value |
| Integer | CV\_NODE\_INT | node->data.i |
| Floating-point | CV\_NODE\_REAL | node->data.f |
| Text string | CV\_NODE\_STR | node->data.str.ptr |
| Sequence | CV\_NODE\_SEQ | node->data.seq |
| Map | CV\_NODE\_MAP | node->data.map\* |

\*: There is no need to access `map` field directly (BTW, `CvMap` is a hidden structure). The elements of the map can be retrieved with cvGetFileNodeByName function that takes pointer to the "map" file node.

A user (custom) object is instance of either one of standard CXCORE types, such as CvMat, CvSeq etc.,
or any type registered with cvRegisterTypeInfo. Such an object is initially represented in file as a map
(as shown in XML and YAML sample files above), after file storage has been opened and parsed.
Then the object can be decoded (converted to the native representation) by request -
when user calls cvRead or
cvReadByName function.

---

### CvAttrList

List of attributes

```
typedef struct CvAttrList
{
    const char** attr; /* NULL-terminated array of (attribute_name,attribute_value) pairs */
    struct CvAttrList* next; /* pointer to next chunk of the attributes list */
}
CvAttrList;

/* initializes CvAttrList structure */
inline CvAttrList cvAttrList( const char** attr=NULL, CvAttrList* next=NULL );

/* returns attribute value or 0 (NULL) if there is no such attribute */
const char* cvAttrValue( const CvAttrList* attr, const char* attr_name );
```

In the current implementation attributes are used to pass extra parameters when writing user objects
(see cvWrite).
XML attributes inside tags are not supported, besides the object type specification
(`type_id` attribute).

---

### OpenFileStorage

Opens file storage for reading or writing data

```
CvFileStorage* cvOpenFileStorage( const char* filename, CvMemStorage* memstorage, int flags );
```

filename: Name of the file associated with the storage. memstorage: Memory storage used for temporary data and for storing dynamic structures, such as CvSeq or CvGraph. If it is NULL, a temporary memory storage is created and used. flags: Can be one of the following: `CV_STORAGE_READ` - the storage is open for reading `CV_STORAGE_WRITE` - the storage is open for writing

The function `cvOpenFileStorage` opens file storage for
reading or writing data. In the latter case a new file is created or existing file is
rewritten. Type of the read of written file is determined by the filename extension: `.xml`
for *XML*, and `.yml` or `.yaml` for *YAML*.
The function returns pointer to CvFileStorage structure.

---

### ReleaseFileStorage

Releases file storage

```
void  cvReleaseFileStorage( CvFileStorage** fs );
```

fs: Double pointer to the released file storage.

The function `cvReleaseFileStorage` closes the file
associated with the storage and releases all the temporary structures.
It must be called after all I/O operations with the storage are finished.

---

## Writing Data

---

### StartWriteStruct

Starts writing a new structure

```
void  cvStartWriteStruct( CvFileStorage* fs, const char* name,
                          int struct_flags, const char* type_name=NULL,
                          CvAttrList attributes=cvAttrList());
```

fs: File storage. name: Name of the written structure. The structure can be accessed by this name when the storage is read. struct\_flags: A combination one of the following values: `CV_NODE_SEQ` - the written structure is a sequence (see discussion of CvFileStorage), that is, its elements do not have a name. `CV_NODE_MAP` - the written structure is a map (see discussion of CvFileStorage), that is, all its elements have names. *One and only one of the two above flags must be specified* `CV_NODE_FLOW` - the optional flag that has sense only for YAML streams. It means that the structure is written as a flow (not as a block), which is more compact. It is recommended to use this flag for structures or arrays whose elements are all scalars. type\_name: Optional parameter - the object type name. In case of XML it is written as `type_id` attribute of the structure opening tag. In case of YAML it is written after a colon following the structure name (see the example in CvFileStorage description). Mainly it comes with user objects. When the storage is read, the encoded type name is used to determine the object type (see CvTypeInfo and cvFindTypeInfo). attributes: This parameter is not used in the current implementation.

The function `cvStartWriteStruct` starts writing
a compound structure (collection) that can be a sequence or a map. After all the structure
fields, which can be scalars or structures, are written,
cvEndWriteStruct should be called.
The function can be used to group some objects or to implement *write* function for a
some user object (see CvTypeInfo).

---

### EndWriteStruct

Ends writing a structure

```
void  cvEndWriteStruct( CvFileStorage* fs );
```

fs: File storage.

The function `cvEndWriteStruct` finishes the
currently written structure.

---

### WriteInt

Writes an integer value

```
void  cvWriteInt( CvFileStorage* fs, const char* name, int value );
```

fs: File storage. name: Name of the written value. Should be NULL if and only if the parent structure is a sequence. value: The written value.

The function `cvWriteInt` writes a single integer value
(with or without a name) to the file storage.

---

### WriteReal

Writes a floating-point value

```
void  cvWriteReal( CvFileStorage* fs, const char* name, double value );
```

fs: File storage. name: Name of the written value. Should be NULL if and only if the parent structure is a sequence. value: The written value.

The function `cvWriteReal` writes a single
floating-point value (with or without a name) to the file storage. The special
values are encoded: NaN (Not A Number) as .NaN, ±Infinity as +.Inf (-.Inf).

The following example shows how to use the low-level writing functions to
store custom structures, such as termination criteria, without registering
a new type.

```
void write_termcriteria( CvFileStorage* fs, const char* struct_name,
                         CvTermCriteria* termcrit )
{
    cvStartWriteStruct( fs, struct_name, CV_NODE_MAP, NULL, cvAttrList(0,0));
    cvWriteComment( fs, "termination criteria", 1 ); // just a description
    if( termcrit->type & CV_TERMCRIT_ITER )
        cvWriteInt( fs, "max_iterations", termcrit->max_iter );
    if( termcrit->type & CV_TERMCRIT_EPS )
        cvWriteReal( fs, "accuracy", termcrit->epsilon );
    cvEndWriteStruct( fs );
}
```

---

### WriteString

Writes a text string

```
void  cvWriteString( CvFileStorage* fs, const char* name,
                     const char* str, int quote=0 );
```

fs: File storage. name: Name of the written string. Should be NULL if and only if the parent structure is a sequence. str: The written text string. quote: If non-zero, the written string is put in quotes, regardless of whether they are required or not. Otherwise, if the flag is zero, quotes are used only when they are required (e.g. when the string starts with a digit or contains spaces).

The function `cvWriteString` writes a text string
to the file storage.

---

### WriteComment

Writes comment

```
void  cvWriteComment( CvFileStorage* fs, const char* comment, int eol_comment );
```

fs: File storage. comment: The written comment, single-line or multi-line. eol\_comment: If non-zero, the function tries to put the comment in the end of current line. If the flag is zero, if the comment is multi-line, or if it does not fit in the end of the current line, the comment starts from a new line.

The function `cvWriteComment` writes a comment into the
file storage. The comments are skipped when the storage is read, so they may be
used only for debugging or descriptive purposes.

---

### StartNextStream

Starts the next stream

```
void  cvStartNextStream( CvFileStorage* fs );
```

fs: File storage.

The function `cvStartNextStream` starts the next
stream in the file storage. Both YAML and XML supports multiple "streams". This
is useful for concatenating files or for resuming the writing process.

---

### Write

Writes user object

```
void  cvWrite( CvFileStorage* fs, const char* name,
               const void* ptr, CvAttrList attributes=cvAttrList() );
```

fs: File storage. name: Name, of the written object. Should be NULL if and only if the parent structure is a sequence. ptr: Pointer to the object. attributes: The attributes of the object. They are specific for each particular type (see the discussion).

The function `cvWrite` writes the object to file storage.
First, the appropriate type info is found using cvTypeOf.
Then, `write` method of the type info is called.

Attributes are used to customize the writing procedure.
The standard types support the following attributes
(all the `*dt` attributes have the same format as in
cvWriteRawData):

CvSeq: - `header_dt` - description of user fields of the sequence header that follow CvSeq, or CvChain (if the sequence is Freeman chain) or CvContour (if the sequence is a contour or point sequence)- `dt` - description of the sequence elements.- `recursive` - if the attribute is present and is not equal to "0" or "false", the whole tree of sequences (contours) is stored. `CvGraph`: - `header_dt` - description of user fields of the graph header that follow CvGraph;- `vertex_dt` - description of user fields of graph vertices- `edge_dt` - description of user fields of graph edges (note, that edge weight is always written, so there is no need to specify it explicitly)

Below is the code that creates the YAML file shown in `CvFileStorage`
description:

```
#include "cxcore.h"

int main( int argc, char** argv )
{
    CvMat* mat = cvCreateMat( 3, 3, CV_32F );
    CvFileStorage* fs = cvOpenFileStorage( "example.yml", 0, CV_STORAGE_WRITE );

    cvSetIdentity( mat );
    cvWrite( fs, "A", mat, cvAttrList(0,0) );

    cvReleaseFileStorage( &fs );
    cvReleaseMat( &mat );
    return 0;
}
```

---

### WriteRawData

Writes multiple numbers

```
void  cvWriteRawData( CvFileStorage* fs, const void* src,
                      int len, const char* dt );
```

fs: File storage. src: Pointer to the written array len: Number of the array elements to write. dt: Specification of each array element that has the following format: `([count]{'u'|'c'|'w'|'s'|'i'|'f'|'d'})...`, where the characters correspond to fundamental C types: - 'u' - 8-bit unsigned number- 'c' - 8-bit signed number- 'w' - 16-bit unsigned number- 's' - 16-bit signed number- 'i' - 32-bit signed number- 'f' - single precision floating-point number- 'd' - double precision floating-point number- 'r' - pointer. 32 lower bits of it are written as a signed integer. The type can be used to store structures with links between the elements. `count` is the optional counter of values of the certain type. For example, `dt='2if'` means that each array element is a structure of 2 integers, followed by a single-precision floating-point number. The equivalent notations of the above specification are `'iif'`, `'2i1f'` etc. Other examples: `dt='u'` means that the array consists of bytes, `dt='2d'` - the array consists of pairs of double’s.

The function `cvWriteRawData` writes array,
which elements consist of a single of multiple numbers. The function call can
be replaced with a loop containing a few cvWriteInt
and cvWriteReal calls,
but a single call is more efficient. Note, that because none of the elements
have a name, they should be written to a sequence rather than a map.

---

### WriteFileNode

Writes file node to another file storage

```
void cvWriteFileNode( CvFileStorage* fs, const char* new_node_name,
                      const CvFileNode* node, int embed );
```

fs: Destination file storage. new\_file\_node: New name of the file node in the destination file storage. To keep the existing name, use `cvGetFileNodeName(node)`. node: The written node embed: If the written node is a collection and this parameter is not zero, no extra level of hierarchy is created. Instead, all the elements of `node` are written into the currently written structure. Of course, map elements may be written only to map, and sequence elements may be written only to sequence.

The function `cvWriteFileNode`
writes a copy of file node to file storage. The possible application of the function are:
merging several file storages into one. Conversion between XML and YAML formats etc.

---

## Reading Data

Data are retrieved from file storage in 2 steps:
first, the file node containing the requested data is found;
then, data is extracted from the node manually or using custom `read` method.

---

### GetRootFileNode

Retrieves one of top-level nodes of the file storage

```
CvFileNode* cvGetRootFileNode( const CvFileStorage* fs, int stream_index=0 );
```

fs: File storage. stream\_index: Zero-based index of the stream. See cvStartNextStream. In most cases, there is only one stream in the file, however there can be several.

The function `cvGetRootFileNode` returns
one of top-level file nodes. The top-level nodes do not have a name, they correspond to
the streams, that are stored one after another in the file storage.
If the index is out of range, the function returns NULL pointer, so all the top-level
nodes may be iterated by subsequent calls to the function with `stream_index=0,1,...`,
until NULL pointer is returned. This function may be used as a base for recursive traversal of the
file storage.

---

### GetFileNodeByName

Finds node in the map or file storage

```
CvFileNode* cvGetFileNodeByName( const CvFileStorage* fs,
                                 const CvFileNode* map,
                                 const char* name );
```

fs: File storage. map: The parent map. If it is NULL, the function searches in all the top-level nodes (streams), starting from the first one. name: The file node name.

The function `cvGetFileNodeByName` finds
a file node by `name`. The node is searched either in `map`
or, if the pointer is NULL, among the top-level file nodes of the storage.
Using this function for maps and cvGetSeqElem
(or sequence reader) for sequences, it is possible to navigate through the file storage.
To speed up multiple queries for a certain key (e.g. in case of array of structures)
one may use a pair of cvGetHashedKey and
cvGetFileNode.

---

### GetHashedKey

Returns a unique pointer for given name

```
CvStringHashNode* cvGetHashedKey( CvFileStorage* fs, const char* name,
                                  int len=-1, int create_missing=0 );
```

fs: File storage. name: Literal node name. len: Length of the name (if it is known a priori), or -1 if it needs to be calculated. create\_missing: Flag that specifies, whether an absent key should be added into the hash table, or not.

The function `cvGetHashedKey` returns
the unique pointer for each particular file node name. This pointer can be
then passed to cvGetFileNode function that
is faster than cvGetFileNodeByName
because it compares text strings by comparing pointers rather than the
strings' content.

Consider the following example: an array of points
is encoded as a sequence of 2-entry maps, e.g.:

```
%YAML:1.0
points:
  - { x: 10, y: 10 }
  - { x: 20, y: 20 }
  - { x: 30, y: 30 }
  # ...
```

Then, it is possible to get hashed "x" and "y" pointers to speed up
decoding of the points.

#### Example. Reading an array of structures from file storage

```
#include "cxcore.h"

int main( int argc, char** argv )
{
    CvFileStorage* fs = cvOpenFileStorage( "points.yml", 0, CV_STORAGE_READ );
    CvStringHashNode* x_key = cvGetHashedNode( fs, "x", -1, 1 );
    CvStringHashNode* y_key = cvGetHashedNode( fs, "y", -1, 1 );
    CvFileNode* points = cvGetFileNodeByName( fs, 0, "points" );

    if( CV_NODE_IS_SEQ(points->tag) )
    {
        CvSeq* seq = points->data.seq;
        int i, total = seq->total;
        CvSeqReader reader;
        cvStartReadSeq( seq, &reader, 0 );
        for( i = 0; i < total; i++ )
        {
            CvFileNode* pt = (CvFileNode*)reader.ptr;
#if 1 /* faster variant */
            CvFileNode* xnode = cvGetFileNode( fs, pt, x_key, 0 );
            CvFileNode* ynode = cvGetFileNode( fs, pt, y_key, 0 );
            assert( xnode && CV_NODE_IS_INT(xnode->tag) &&
                    ynode && CV_NODE_IS_INT(ynode->tag));
            int x = xnode->data.i; // or x = cvReadInt( xnode, 0 );
            int y = ynode->data.i; // or y = cvReadInt( ynode, 0 );
#elif 1 /* slower variant; does not use x_key & y_key */
            CvFileNode* xnode = cvGetFileNodeByName( fs, pt, "x" );
            CvFileNode* ynode = cvGetFileNodeByName( fs, pt, "y" );
            assert( xnode && CV_NODE_IS_INT(xnode->tag) &&
                    ynode && CV_NODE_IS_INT(ynode->tag));
            int x = xnode->data.i; // or x = cvReadInt( xnode, 0 );
            int y = ynode->data.i; // or y = cvReadInt( ynode, 0 );
#else /* the slowest yet the easiest to use variant */
            int x = cvReadIntByName( fs, pt, "x", 0 /* default value */ );
            int y = cvReadIntByName( fs, pt, "y", 0 /* default value */ );
#endif
            CV_NEXT_SEQ_ELEM( seq->elem_size, reader );
            printf("%d: (%d, %d)\n", i, x, y );
        }
    }
    cvReleaseFileStorage( &fs );
    return 0;
}
```

Please note that, whatever method of accessing map you are using,
it is still *much* slower than using plain sequences, for example,
in the above sample, it is more efficient to encode the points as pairs of
integers in the single numeric sequence.

---

### GetFileNode

Finds node in the map or file storage

```
CvFileNode* cvGetFileNode( CvFileStorage* fs, CvFileNode* map,
                           const CvStringHashNode* key, int create_missing=0 );
```

fs: File storage. map: The parent map. If it is NULL, the function searches a top-level node. If both `map` and `key` are NULLs, the function returns the root file node - a map that contains top-level nodes. key: Unique pointer to the node name, retrieved with cvGetHashedKey. create\_missing: Flag that specifies, whether an absent node should be added to the map, or not.

The function `cvGetFileNode` finds a file node. It is
a faster version cvGetFileNodeByName
(see cvGetHashedKey discussion). Also,
the function can insert a new node, if it is not in the map yet (which is used
by parsing functions).

---

### GetFileNodeName

Returns name of file node

```
const char* cvGetFileNodeName( const CvFileNode* node );
```

node: File node

The function `cvGetFileNodeName` returns name of the file node
or NULL, if the file node does not have a name, or if `node` is `NULL`.

---

### ReadInt

Retrieves integer value from file node

```
int cvReadInt( const CvFileNode* node, int default_value=0 );
```

node: File node. default\_value: The value that is returned if `node` is NULL.

The function `cvReadInt` returns integer that is
represented by the file node. If the file node is NULL, `default_value`
is returned (thus, it is convenient to call the function right after
cvGetFileNode without checking for NULL pointer),
otherwise if the file node has type `CV_NODE_INT`,
then `node->data.i` is returned, otherwise if the file node has
type `CV_NODE_REAL`, then `node->data.f`
is converted to integer and returned, otherwise the result is not determined.

---

### ReadIntByName

Finds file node and returns its value

```
int cvReadIntByName( const CvFileStorage* fs, const CvFileNode* map,
                     const char* name, int default_value=0 );
```

fs: File storage. map: The parent map. If it is NULL, the function searches a top-level node. name: The node name. default\_value: The value that is returned if the file node is not found.

The function `cvReadIntByName` is a simple
superposition of cvGetFileNodeByName and
cvReadInt.

---

### ReadReal

Retrieves floating-point value from file node

```
double cvReadReal( const CvFileNode* node, double default_value=0. );
```

node: File node. default\_value: The value that is returned if `node` is NULL.

The function `cvReadReal` returns floating-point value that is
represented by the file node. If the file node is NULL, `default_value`
is returned (thus, it is convenient to call the function right after
cvGetFileNode without checking for NULL pointer),
otherwise if the file node has type `CV_NODE_REAL`,
then `node->data.f` is returned, otherwise if the file node has
type `CV_NODE_INT`, then `node->data.f`
is converted to floating-point and returned, otherwise the result is not determined.

---

### ReadRealByName

Finds file node and returns its value

```
double  cvReadRealByName( const CvFileStorage* fs, const CvFileNode* map,
                          const char* name, double default_value=0. );
```

fs: File storage. map: The parent map. If it is NULL, the function searches a top-level node. name: The node name. default\_value: The value that is returned if the file node is not found.

The function `cvReadRealByName` is a simple
superposition of cvGetFileNodeByName and
cvReadReal.

---

### ReadString

Retrieves text string from file node

```
const char* cvReadString( const CvFileNode* node, const char* default_value=NULL );
```

node: File node. default\_value: The value that is returned if `node` is NULL.

The function `cvReadString` returns text string that is
represented by the file node. If the file node is NULL, `default_value`
is returned (thus, it is convenient to call the function right after
cvGetFileNode without checking for NULL pointer),
otherwise if the file node has type `CV_NODE_STR`,
then `node->data.str.ptr` is returned, otherwise the result is not determined.

---

### ReadStringByName

Finds file node and returns its value

```
const char* cvReadStringByName( const CvFileStorage* fs, const CvFileNode* map,
                                const char* name, const char* default_value=NULL );
```

fs: File storage. map: The parent map. If it is NULL, the function searches a top-level node. name: The node name. default\_value: The value that is returned if the file node is not found.

The function `cvReadStringByName` is a simple
superposition of cvGetFileNodeByName and
cvReadString.

---

### Read

Decodes object and returns pointer to it

```
void* cvRead( CvFileStorage* fs, CvFileNode* node,
              CvAttrList* attributes=NULL );
```

fs: File storage. node: The root object node. attributes: Unused parameter.

The function `cvRead` decodes user object (creates object
in a native representation from the file storage subtree) and returns it.
The object to be decoded must be an instance of registered type that supports `read`
method (see CvTypeInfo). Type of the object is determined by
the type name that is encoded in the file. If the object is dynamic structure, it is
created either in memory storage, passed to cvOpenFileStorage
or, if NULL pointer was passed, in temporary memory storage, which is release when
cvReleaseFileStorage is called.
Otherwise, if the object is not a dynamic structure, it is created in heap and should be
released with a specialized function or using generic cvRelease.

---

### ReadByName

Finds object and decodes it

```
void* cvReadByName( CvFileStorage* fs, const CvFileNode* map,
                    const char* name, CvAttrList* attributes=NULL );
```

fs: File storage. map: The parent map. If it is NULL, the function searches a top-level node. name: The node name. attributes: Unused parameter.

The function `cvReadByName` is a simple
superposition of cvGetFileNodeByName and
cvRead.

---

### ReadRawData

Reads multiple numbers

```
void cvReadRawData( const CvFileStorage* fs, const CvFileNode* src,
                    void* dst, const char* dt );
```

fs: File storage. src: The file node (a sequence) to read numbers from. dst: Pointer to the destination array. dt: Specification of each array element. It has the same format as in cvWriteRawData.

The function `cvReadRawData` reads elements
from a file node that represents a sequence of scalars

---

### StartReadRawData

Initializes file node sequence reader

```
void cvStartReadRawData( const CvFileStorage* fs, const CvFileNode* src,
                         CvSeqReader* reader );
```

fs: File storage. src: The file node (a sequence) to read numbers from. reader: Pointer to the sequence reader.

The function `cvStartReadRawData` initializes
sequence reader to read data from file node. The initialized reader can be then
passed to cvReadRawDataSlice.

---

### ReadRawDataSlice

Initializes file node sequence reader

```
void cvReadRawDataSlice( const CvFileStorage* fs, CvSeqReader* reader,
                         int count, void* dst, const char* dt );
```

fs: File storage. reader: The sequence reader. Initialize it with cvStartReadRawData. count: The number of elements to read. dst: Pointer to the destination array. dt: Specification of each array element. It has the same format as in cvWriteRawData.

The function `cvReadRawDataSlice` reads one
or more elements from the file node, representing a sequence, to user-specified
array. The total number of read sequence elements is a product of `total`
and the number of components in each array element.
For example, if `dt='2if'`, the function will read `total*3` sequence elements.
As with any sequence, some parts of the file node sequence may be skipped or read repeatedly
by repositioning the reader using cvSetSeqReaderPos.

---

## RTTI and Generic Functions

---

### CvTypeInfo

Type information

```
typedef int (CV_CDECL *CvIsInstanceFunc)( const void* struct_ptr );
typedef void (CV_CDECL *CvReleaseFunc)( void** struct_dblptr );
typedef void* (CV_CDECL *CvReadFunc)( CvFileStorage* storage, CvFileNode* node );
typedef void (CV_CDECL *CvWriteFunc)( CvFileStorage* storage,
                                      const char* name,
                                      const void* struct_ptr,
                                      CvAttrList attributes );
typedef void* (CV_CDECL *CvCloneFunc)( const void* struct_ptr );

typedef struct CvTypeInfo
{
    int flags; /* not used */
    int header_size; /* sizeof(CvTypeInfo) */
    struct CvTypeInfo* prev; /* previous registered type in the list */
    struct CvTypeInfo* next; /* next registered type in the list */
    const char* type_name; /* type name, written to file storage */

    /* methods */
    CvIsInstanceFunc is_instance; /* checks if the passed object belongs to the type */
    CvReleaseFunc release; /* releases object (memory etc.) */
    CvReadFunc read; /* reads object from file storage */
    CvWriteFunc write; /* writes object to file storage */
    CvCloneFunc clone; /* creates a copy of the object */
}
CvTypeInfo;
```

The structure CvTypeInfo contains information about
one of standard or user-defined types. Instances of the type may or may not contain
pointer to the corresponding CvTypeInfo structure.
In any case there is a way to find type info structure for given object - using
cvTypeOf function. Alternatively, type info can be
found by the type name using cvFindType, which is
used when object is read from file storage. User can register a new type with
cvRegisterType that adds the type information structure
into the beginning of the type list - thus, it is possible to create specialized types
from generic standard types and override the basic methods.

---

### RegisterType

Registers new type

```
void cvRegisterType( const CvTypeInfo* info );
```

info: Type info structure.

The function `cvRegisterType` registers a new type,
which is described by `info`. The function creates a copy of the structure,
so user should delete it after calling the function.

---

### UnregisterType

Unregisters the type

```
void cvUnregisterType( const char* type_name );
```

type\_name: Name of the unregistered type.

The function `cvUnregisterType` unregisters the type
with the specified name. If the name is unknown, it is possible to locate the type info
by an instance of the type using cvTypeOf or by iterating
the type list, starting from cvFirstType, and then
call `cvUnregisterType(info->type_name)`.

---

### FirstType

Returns the beginning of type list

```
CvTypeInfo* cvFirstType( void );
```

The function `cvFirstType` returns the first type of the list
of registered types. Navigation through the list can be done via `prev` and `next`
fields of CvTypeInfo structure.

---

### FindType

Finds type by its name

```
CvTypeInfo* cvFindType( const char* type_name );
```

type\_name: Type name.

The function `cvFindType` finds a registered type by its name.
It returns NULL, if there is no type with the specified name.

---

### TypeOf

Returns type of the object

```
CvTypeInfo* cvTypeOf( const void* struct_ptr );
```

struct\_ptr: The object pointer.

The function `cvTypeOf` finds the type of given object. It iterates through the list
of registered types and calls `is_instance` function/method of every type info structure with
the object until one of them return non-zero or until the whole list has been traversed. In the latter
case the function returns NULL.

---

### Release

Releases the object

```
void cvRelease( void** struct_ptr );
```

struct\_ptr: Double pointer to the object.

The function `cvRelease` finds the type of given object and calls
`release` with the double pointer.

---

### Clone

Makes a clone of the object

```
void* cvClone( const void* struct_ptr );
```

struct\_ptr: The object to clone.

The function `cvClone` finds the type of given object and calls
`clone` with the passed object.

---

### Save

Saves object to file

```
void cvSave( const char* filename, const void* struct_ptr,
             const char* name=NULL,
             const char* comment=NULL,
             CvAttrList attributes=cvAttrList());
```

filename: File name. struct\_ptr: Object to save. name: Optional object name. If it is NULL, the name will be formed from `filename`. comment: Optional comment to put in the beginning of the file. attributes: Optional attributes passed to cvWrite.

The function `cvSave` saves object to file. It provides a simple interface
to cvWrite.

---

### Load

Loads object from file

```
void* cvLoad( const char* filename, CvMemStorage* memstorage=NULL,
              const char* name=NULL, const char** real_name=NULL );
```

filename: File name. memstorage: Memory storage for dynamic structures, such as CvSeq or CvGraph. It is not used for matrices or images. name: Optional object name. If it is NULL, the first top-level object in the storage will be loaded. real\_name: Optional output parameter that will contain name of the loaded object (useful if `name=NULL`).

The function `cvLoad` loads object from file.
It provides a simple interface to cvRead.
After object is loaded,
the file storage is closed and all the temporary buffers are deleted. Thus, to load a dynamic structure,
such as sequence, contour or graph, one should pass a valid destination
memory storage to the function.

---

# Miscellaneous Functions

---

### CheckArr

Checks every element of input array for invalid values

```
int  cvCheckArr( const CvArr* arr, int flags=0,
                 double min_val=0, double max_val=0);
#define cvCheckArray cvCheckArr
```

arr: The array to check. flags: The operation flags, 0 or combination of: `CV_CHECK_RANGE` - if set, the function checks that every value of array is within [minVal,maxVal) range, otherwise it just checks that every element is neither NaN nor ±Infinity. `CV_CHECK_QUIET` - if set, the function does not raises an error if an element is invalid or out of range min\_val: The inclusive lower boundary of valid values range. It is used only if `CV_CHECK_RANGE` is set. max\_val: The exclusive upper boundary of valid values range. It is used only if `CV_CHECK_RANGE` is set.

The function `cvCheckArr` checks that every array element
is neither NaN nor ±Infinity.
If `CV_CHECK_RANGE` is set, it also checks that every element is
greater than or equal to `minVal` and less than `maxVal`.
The function returns nonzero if the check succeeded, i.e. all elements
are valid and within the range, and zero otherwise.
In the latter case if `CV_CHECK_QUIET` flag is not set, the function
raises runtime error.

---

### KMeans2

Splits set of vectors by given number of clusters

```
void cvKMeans2( const CvArr* samples, int cluster_count,
                CvArr* labels, CvTermCriteria termcrit );
```

samples: Floating-point matrix of input samples, one row per sample. cluster\_count: Number of clusters to split the set by. labels: Output integer vector storing cluster indices for every sample. termcrit: Specifies maximum number of iterations and/or accuracy (distance the centers move by between the subsequent iterations).

The function `cvKMeans2` implements k-means algorithm that finds centers of `cluster_count` clusters
and groups the input samples around the clusters. On output `labels(i)` contains a cluster index
for sample stored in the i-th row of `samples` matrix.

#### Example. Clustering random samples of k-variate Gaussian distribution

```
#include "cxcore.h"
#include "highgui.h"

void main( int argc, char** argv )
{
    #define MAX_CLUSTERS 5
    CvScalar color_tab[MAX_CLUSTERS];
    IplImage* img = cvCreateImage( cvSize( 500, 500 ), 8, 3 );
    CvRNG rng = cvRNG(0xffffffff);

    color_tab[0] = CV_RGB(255,0,0);
    color_tab[1] = CV_RGB(0,255,0);
    color_tab[2] = CV_RGB(100,100,255);
    color_tab[3] = CV_RGB(255,0,255);
    color_tab[4] = CV_RGB(255,255,0);

    cvNamedWindow( "clusters", 1 );

    for(;;)
    {
        int k, cluster_count = cvRandInt(&rng)%MAX_CLUSTERS + 1;
        int i, sample_count = cvRandInt(&rng)%1000 + 1;
        CvMat* points = cvCreateMat( sample_count, 1, CV_32FC2 );
        CvMat* clusters = cvCreateMat( sample_count, 1, CV_32SC1 );

        /* generate random sample from multivariate Gaussian distribution */
        for( k = 0; k < cluster_count; k++ )
        {
            CvPoint center;
            CvMat point_chunk;
            center.x = cvRandInt(&rng)%img->width;
            center.y = cvRandInt(&rng)%img->height;
            cvGetRows( points, &point_chunk, k*sample_count/cluster_count,
                       k == cluster_count - 1 ? sample_count : (k+1)*sample_count/cluster_count );
            cvRandArr( &rng, &point_chunk, CV_RAND_NORMAL,
                       cvScalar(center.x,center.y,0,0),
                       cvScalar(img->width/6, img->height/6,0,0) );
        }

        /* shuffle samples */
        for( i = 0; i < sample_count/2; i++ )
        {
            CvPoint2D32f* pt1 = (CvPoint2D32f*)points->data.fl + cvRandInt(&rng)%sample_count;
            CvPoint2D32f* pt2 = (CvPoint2D32f*)points->data.fl + cvRandInt(&rng)%sample_count;
            CvPoint2D32f temp;
            CV_SWAP( *pt1, *pt2, temp );
        }

        cvKMeans2( points, cluster_count, clusters,
                   cvTermCriteria( CV_TERMCRIT_EPS+CV_TERMCRIT_ITER, 10, 1.0 ));

        cvZero( img );

        for( i = 0; i < sample_count; i++ )
        {
            CvPoint2D32f pt = ((CvPoint2D32f*)points->data.fl)[i];
            int cluster_idx = clusters->data.i[i];
            cvCircle( img, cvPointFrom32f(pt), 2, color_tab[cluster_idx], CV_FILLED );
        }

        cvReleaseMat( &points );
        cvReleaseMat( &clusters );

        cvShowImage( "clusters", img );

        int key = cvWaitKey(0);
        if( key == 27 ) // 'ESC'
            break;
    }
}
```

---

### SeqPartition

Splits sequence into equivalence classes

```
typedef int (CV_CDECL* CvCmpFunc)(const void* a, const void* b, void* userdata);
int cvSeqPartition( const CvSeq* seq, CvMemStorage* storage, CvSeq** labels,
                    CvCmpFunc is_equal, void* userdata );
```

seq: The sequence to partition. storage: The storage to store the sequence of equivalence classes. If it is NULL, the function uses `seq->storage` for output labels. labels: Output parameter. Double pointer to the sequence of 0-based labels of input sequence elements. is\_equal: The relation function that should return non-zero if the two particular sequence elements are from the same class, and zero otherwise. The partitioning algorithm uses transitive closure of the relation function as equivalence criteria. userdata: Pointer that is transparently passed to the `is_equal` function.

The function `cvSeqPartition` implements quadratic algorithm for splitting
a set into one or more classes of equivalence. The function returns the number of equivalence classes.

#### Example. Partitioning 2d point set.

```
#include "cxcore.h"
#include "highgui.h"
#include <stdio.h>

CvSeq* point_seq = 0;
IplImage* canvas = 0;
CvScalar* colors = 0;
int pos = 10;

int is_equal( const void* _a, const void* _b, void* userdata )
{
    CvPoint a = *(const CvPoint*)_a;
    CvPoint b = *(const CvPoint*)_b;
    double threshold = *(double*)userdata;
    return (double)(a.x - b.x)*(a.x - b.x) + (double)(a.y - b.y)*(a.y - b.y) <= threshold;
}

void on_track( int pos )
{
    CvSeq* labels = 0;
    double threshold = pos*pos;
    int i, class_count = cvSeqPartition( point_seq, 0, &labels, is_equal, &threshold );
    printf("%4d classes\n", class_count );
    cvZero( canvas );

    for( i = 0; i < labels->total; i++ )
    {
        CvPoint pt = *(CvPoint*)cvGetSeqElem( point_seq, i, 0 );
        CvScalar color = colors[*(int*)cvGetSeqElem( labels, i, 0 )];
        cvCircle( canvas, pt, 1, color, -1 );
    }

    cvShowImage( "points", canvas );
}

int main( int argc, char** argv )
{
    CvMemStorage* storage = cvCreateMemStorage(0);
    point_seq = cvCreateSeq( CV_32SC2, sizeof(CvSeq), sizeof(CvPoint), storage );
    CvRNG rng = cvRNG(0xffffffff);

    int width = 500, height = 500;
    int i, count = 1000;
    canvas = cvCreateImage( cvSize(width,height), 8, 3 );

    colors = (CvScalar*)cvAlloc( count*sizeof(colors[0]) );
    for( i = 0; i < count; i++ )
    {
        CvPoint pt;
        int icolor;
        pt.x = cvRandInt( &rng ) % width;
        pt.y = cvRandInt( &rng ) % height;
        cvSeqPush( point_seq, &pt );
        icolor = cvRandInt( &rng ) | 0x00404040;
        colors[i] = CV_RGB(icolor & 255, (icolor >> 8)&255, (icolor >> 16)&255);
    }

    cvNamedWindow( "points", 1 );
    cvCreateTrackbar( "threshold", "points", &pos, 50, on_track );
    on_track(pos);
    cvWaitKey(0);
    return 0;
}
```

---

# Error Handling and System Functions

---

## Error Handling

Error handling in OpenCV is similar to IPL (Image Processing Library).
In case of error functions do not return the error code. Instead, they raise
an error using CV\_ERROR macro that calls
cvError that, in its turn, sets the error status
with cvSetErrStatus and
calls a standard or user-defined error handler (that can display a message box,
write to log etc., see cvRedirectError,
cvNulDevReport, cvStdErrReport, cvGuiBoxReport).
There is global variable, one per each program thread, that contains current
error status (an integer value). The status can be retrieved with
cvGetErrStatus function.

There are three modes of error handling (see cvSetErrMode
and cvGetErrMode):

Leaf: The program is terminated after error handler is called. *This is the default value*. It is useful for debugging, as the error is signalled immediately after it occurs. However, for production systems other two methods may be preferable as they provide more control. Parent: The program is not terminated, but the error handler is called. The stack is unwinded (it is done w/o using C++ exception mechanism). User may check error code after calling Cxcore function with cvGetErrStatus and react. Silent: Similar to *Parent* mode, but no error handler is called.

Actually, the semantics of *Leaf* and *Parent* modes is implemented by
error handlers and the above description is true for cvNulDevReport, cvStdErrReport.
cvGuiBoxReport behaves slightly differently,
and some custom error handler may implement quite different semantics.

---

### ERROR Handling Macros

Macros for raising an error, checking for errors etc.

```
/* special macros for enclosing processing statements within a function and separating
   them from prologue (resource initialization) and epilogue (guaranteed resource release) */
#define __BEGIN__       {
#define __END__         goto exit; exit: ; }
/* proceeds to "resource release" stage */
#define EXIT            goto exit

/* Declares locally the function name for CV_ERROR() use */
#define CV_FUNCNAME( Name )  \
    static char cvFuncName[] = Name

/* Raises an error within the current context */
#define CV_ERROR( Code, Msg )                                       \
{                                                                   \
     cvError( (Code), cvFuncName, Msg, __FILE__, __LINE__ );        \
     EXIT;                                                          \
}

/* Checks status after calling CXCORE function */
#define CV_CHECK()                                                  \
{                                                                   \
    if( cvGetErrStatus() < 0 )                                   \
        CV_ERROR( CV_StsBackTrace, "Inner function failed." );      \
}

/* Provides shorthand for CXCORE function call and CV_CHECK() */
#define CV_CALL( Statement )                                        \
{                                                                   \
    Statement;                                                      \
    CV_CHECK();                                                     \
}

/* Checks some condition in both debug and release configurations */
#define CV_ASSERT( Condition )                                          \
{                                                                       \
    if( !(Condition) )                                                  \
        CV_ERROR( CV_StsInternal, "Assertion: " #Condition " failed" ); \
}

/* these macros are similar to their CV_... counterparts, but they
   do not need exit label nor cvFuncName to be defined */
#define OPENCV_ERROR(status,func_name,err_msg) ...
#define OPENCV_ERRCHK(func_name,err_msg) ...
#define OPENCV_ASSERT(condition,func_name,err_msg) ...
#define OPENCV_CALL(statement) ...
```

Instead of a discussion, here are the documented example
of typical CXCORE function and the example of the function use.

#### Use of Error Handling Macros

```
#include "cxcore.h"
#include <stdio.h>

void cvResizeDCT( CvMat* input_array, CvMat* output_array )
{
    CvMat* temp_array = 0; // declare pointer that should be released anyway.

    CV_FUNCNAME( "cvResizeDCT" ); // declare cvFuncName

    __BEGIN__; // start processing. There may be some declarations just after this macro,
               // but they couldn't be accessed from the epilogue.

    if( !CV_IS_MAT(input_array) || !CV_IS_MAT(output_array) )
        // use CV_ERROR() to raise an error
        CV_ERROR( CV_StsBadArg, "input_array or output_array are not valid matrices" );

    // some restrictions that are going to be removed later, may be checked with CV_ASSERT()
    CV_ASSERT( input_array->rows == 1 && output_array->rows == 1 );

    // use CV_CALL for safe function call
    CV_CALL( temp_array = cvCreateMat( input_array->rows, MAX(input_array->cols,output_array->cols),
                                       input_array->type ));

    if( output_array->cols > input_array->cols )
        CV_CALL( cvZero( temp_array ));

    temp_array->cols = input_array->cols;
    CV_CALL( cvDCT( input_array, temp_array, CV_DXT_FORWARD ));
    temp_array->cols = output_array->cols;
    CV_CALL( cvDCT( temp_array, output_array, CV_DXT_INVERSE ));
    CV_CALL( cvScale( output_array, output_array, 1./sqrt((double)input_array->cols*output_array->cols), 0 ));

    __END__; // finish processing. Epilogue follows after the macro.

    // release temp_array. If temp_array has not been allocated before an error occurred, cvReleaseMat
    // takes care of it and does nothing in this case.
    cvReleaseMat( &temp_array );
}


int main( int argc, char** argv )
{
    CvMat* src = cvCreateMat( 1, 512, CV_32F );
#if 1 /* no errors */
    CvMat* dst = cvCreateMat( 1, 256, CV_32F );
#else
    CvMat* dst = 0; /* test error processing mechanism */
#endif
    cvSet( src, cvRealScalar(1.), 0 );
#if 0 /* change 0 to 1 to suppress error handler invocation */
    cvSetErrMode( CV_ErrModeSilent );
#endif
    cvResizeDCT( src, dst ); // if some error occurs, the message box will pop up, or a message will be
                             // written to log, or some user-defined processing will be done
    if( cvGetErrStatus() < 0 )
        printf("Some error occurred" );
    else
        printf("Everything is OK" );
    return 0;
}
```

---

### GetErrStatus

Returns the current error status

```
int cvGetErrStatus( void );
```

The function `cvGetErrStatus` returns the current error status -
the value set with the last cvSetErrStatus call. Note, that
in *Leaf* mode the program terminates immediately after error occurred, so to
always get control after the function call, one should call cvSetErrMode
and set *Parent* or *Silent* error mode.

---

### SetErrStatus

Sets the error status

```
void cvSetErrStatus( int status );
```

status: The error status.

The function `cvSetErrStatus` sets the error status to
the specified value. Mostly, the function is used to reset the error status (set to it `CV_StsOk`)
to recover after error. In other cases it is more natural to call cvError or
CV\_ERROR.

---

### GetErrMode

Returns the current error mode

```
int cvGetErrMode( void );
```

The function `cvGetErrMode` returns the current error mode -
the value set with the last cvSetErrMode call.

---

### SetErrMode

Sets the error mode

```
#define CV_ErrModeLeaf    0
#define CV_ErrModeParent  1
#define CV_ErrModeSilent  2
int cvSetErrMode( int mode );
```

mode: The error mode.

The function `cvSetErrMode` sets the specified error mode.
For description of different error modes see the beginning of the section.

---

### Error

Raises an error

```
int cvError( int status, const char* func_name,
             const char* err_msg, const char* file_name, int line );
```

status: The error status. func\_name: Name of the function where the error occurred. err\_msg: Additional information/diagnostics about the error. file\_name: Name of the file where the error occurred. line: Line number, where the error occurred.

The function `cvError` sets the error status
to the specified value (via cvSetErrStatus)
and, if the error mode is not *Silent*, calls the error handler.

---

### ErrorStr

Returns textual description of error status code

```
const char* cvErrorStr( int status );
```

status: The error status.

The function `cvErrorStr` returns the textual description
for the specified error status code. In case of unknown status the function returns NULL pointer.

---

### RedirectError

Sets a new error handler

```
typedef int (CV_CDECL *CvErrorCallback)( int status, const char* func_name,
                    const char* err_msg, const char* file_name, int line );

CvErrorCallback cvRedirectError( CvErrorCallback error_handler,
                                 void* userdata=NULL, void** prev_userdata=NULL );
```

error\_handler: The new error\_handler. userdata: Arbitrary pointer that is transparently passed to the error handler. prev\_userdata: Pointer to the previously assigned user data pointer.

The function `cvRedirectError` sets a new error handler that
can be one of standard handlers or a custom handler that has
the certain interface. The handler takes the same parameters as cvError
function. If the handler returns non-zero value, the program is terminated, otherwise, it continues.
The error handler may check the current error mode with cvGetErrMode
to make a decision.

---

### cvNulDevReport cvStdErrReport cvGuiBoxReport

Provide standard error handling

```
int cvNulDevReport( int status, const char* func_name,
                    const char* err_msg, const char* file_name,
                    int line, void* userdata );

int cvStdErrReport( int status, const char* func_name,
                    const char* err_msg, const char* file_name,
                    int line, void* userdata );

int cvGuiBoxReport( int status, const char* func_name,
                    const char* err_msg, const char* file_name,
                    int line, void* userdata );
```

status: The error status. func\_name: Name of the function where the error occurred. err\_msg: Additional information/diagnostics about the error. file\_name: Name of the file where the error occurred. line: Line number, where the error occurred. userdata: Pointer to the user data. Ignored by the standard handlers.

The functions `cvNullDevReport, cvStdErrReport` and `cvGuiBoxReport`
provide standard error handling. `cvGuiBoxReport` is the default
error handler on Win32 systems, `cvStdErrReport` - on other systems.
`cvGuiBoxReport` pops up message box with the error description and
suggest a few options. Below is the sample message box that may be received with the
sample code above, if one introduce an error as described
in the sample

#### Error Message Box

If the error handler is set `cvStdErrReport`, the above message will
be printed to standard error output and program will be terminated or continued, depending on the
current error mode.

#### Error Message printed to Standard Error Output (in *Leaf* mode)

```
OpenCV ERROR: Bad argument (input_array or output_array are not valid matrices)
        in function cvResizeDCT, D:\User\VP\Projects\avl_proba\a.cpp(75)
Terminating the application...
```

---

## System and Utility Functions

---

### Alloc

Allocates memory buffer

```
void* cvAlloc( size_t size );
```

size: Buffer size in bytes.

The function `cvAlloc` allocates `size` bytes and
returns pointer to the allocated buffer. In case of error
the function reports an error and returns NULL pointer.
By default cvAlloc calls icvAlloc which itself calls malloc,
however it is possible to assign user-defined memory allocation/deallocation
functions using cvSetMemoryManager function.

---

### Free

Deallocates memory buffer

```
void cvFree( T** ptr );
```

buffer: Double pointer to released buffer.

The function `cvFree` deallocates memory buffer allocated by cvAlloc.
It clears the pointer to buffer upon exit, that is why the double pointer is
used. If \*buffer is already NULL, the function does nothing

---

### GetTickCount

Returns number of tics

```
int64 cvGetTickCount( void );
```

The function `cvGetTickCount` returns number of tics
starting from some platform-dependent event (number of CPU ticks from the startup, number of milliseconds
from 1970th year etc.). The function is useful for accurate measurement of
a function/user-code execution time. To convert the number of tics to time units, use
cvGetTickFrequency.

---

### GetTickFrequency

Returns number of tics per microsecond

```
double cvGetTickFrequency( void );
```

The function `cvGetTickFrequency` returns number of tics
per microsecond. Thus, the quotient of cvGetTickCount() and
cvGetTickFrequency() will give a number of microseconds
starting from the platform-dependent event.

---

### RegisterModule

Registers another module

```
typedef struct CvPluginFuncInfo
{
    void** func_addr;
    void* default_func_addr;
    const char* func_names;
    int search_modules;
    int loaded_from;
}
CvPluginFuncInfo;

typedef struct CvModuleInfo
{
    struct CvModuleInfo* next;
    const char* name;
    const char* version;
    CvPluginFuncInfo* func_tab;
}
CvModuleInfo;

int cvRegisterModule( const CvModuleInfo* module_info );
```

module\_info: Information about the module.

The function `cvRegisterModule` adds module to the list of registered
modules. After the module is registered, information about it can be retrieved
using cvGetModuleInfo function. Also, the registered module
makes full use of optimized plugins (IPP, MKL, ...), supported by CXCORE.
CXCORE itself, CV (computer vision), CVAUX (auxiliary computer vision) and HIGHGUI
(visualization & image/video acquisition) are examples of modules. Registration is usually done
then the shared library is loaded. See cxcore/src/cxswitcher.cpp and cv/src/cvswitcher.cpp
for details, how registration is done and
look at cxcore/src/cxswitcher.cpp, cxcore/src/\_cxipp.h on how IPP and MKL are connected to the modules.

---

### GetModuleInfo

Retrieves information about the registered module(s) and plugins

```
void  cvGetModuleInfo( const char* module_name,
                       const char** version,
                       const char** loaded_addon_plugins );
```

module\_name: Name of the module of interest, or NULL, which means all the modules. version: The output parameter. Information about the module(s), including version. loaded\_addon\_plugins: The list of names and versions of the optimized plugins that CXCORE was able to find and load.

The function `cvGetModuleInfo` returns information about one of
or all of the registered modules. The returned information is stored inside the libraries, so
user should not deallocate or modify the returned text strings.

---

### UseOptimized

Switches between optimized/non-optimized modes

```
int cvUseOptimized( int on_off );
```

on\_off: Use optimized (<>0) or not (0).

The function `cvUseOptimized` switches between the mode, where
only pure C implementations from cxcore, OpenCV etc. are used, and the mode, where
IPP and MKL functions are used if available. When `cvUseOptimized(0)` is called,
all the optimized libraries are unloaded. The function may be useful for debugging, IPP&MKL upgrade
on the fly, online speed comparisons etc. It returns the number of optimized functions loaded.
Note that by default the optimized plugins are loaded, so it is not necessary to
call `cvUseOptimized(1)` in the beginning of the program (actually, it will only
increase the startup time)

---

### SetMemoryManager

Assigns custom/default memory managing functions

```
typedef void* (CV_CDECL *CvAllocFunc)(size_t size, void* userdata);
typedef int (CV_CDECL *CvFreeFunc)(void* pptr, void* userdata);

void cvSetMemoryManager( CvAllocFunc alloc_func=NULL,
                         CvFreeFunc free_func=NULL,
                         void* userdata=NULL );
```

alloc\_func: Allocation function; the interface is similar to `malloc`, except that `userdata` may be used to determine the context. free\_func: Deallocation function; the interface is similar to `free`. userdata: User data that is transparently passed to the custom functions.

The function `cvSetMemoryManager`
sets user-defined memory management functions (replacements for malloc and free) that
will be called by cvAlloc, cvFree and higher-level functions (e.g. cvCreateImage).
Note, that the function should be called when there is data allocated using cvAlloc.
Also, to avoid infinite recursive calls, it is not allowed to call cvAlloc
and cvFree from the custom allocation/deallocation functions.

If `alloc_func` and `free_func` pointers are `NULL`,
the default memory managing functions are restored.

---

### SetIPLAllocators

Switches to IPL functions for image allocation/deallocation

```
typedef IplImage* (CV_STDCALL* Cv_iplCreateImageHeader)
                            (int,int,int,char*,char*,int,int,int,int,int,
                            IplROI*,IplImage*,void*,IplTileInfo*);
typedef void (CV_STDCALL* Cv_iplAllocateImageData)(IplImage*,int,int);
typedef void (CV_STDCALL* Cv_iplDeallocate)(IplImage*,int);
typedef IplROI* (CV_STDCALL* Cv_iplCreateROI)(int,int,int,int,int);
typedef IplImage* (CV_STDCALL* Cv_iplCloneImage)(const IplImage*);

void cvSetIPLAllocators( Cv_iplCreateImageHeader create_header,
                         Cv_iplAllocateImageData allocate_data,
                         Cv_iplDeallocate deallocate,
                         Cv_iplCreateROI create_roi,
                         Cv_iplCloneImage clone_image );

#define CV_TURN_ON_IPL_COMPATIBILITY()                                  \
    cvSetIPLAllocators( iplCreateImageHeader, iplAllocateImage,         \
                        iplDeallocate, iplCreateROI, iplCloneImage )
```

create\_header: Pointer to iplCreateImageHeader. allocate\_data: Pointer to iplAllocateImage. deallocate: Pointer to iplDeallocate. create\_roi: Pointer to iplCreateROI. clone\_image: Pointer to iplCloneImage.

The function `cvSetIPLAllocators`
makes CXCORE to use IPL functions for image allocation/deallocation operations.
For convenience, there is the wrapping macro `CV_TURN_ON_IPL_COMPATIBILITY`.
The function is useful for applications where IPL and CXCORE/OpenCV are used together and still
there are calls to `iplCreateImageHeader` etc. The function is not necessary if
IPL is called only for data processing and all the allocation/deallocation is done by CXCORE,
or if all the allocation/deallocation is done by IPL and some of OpenCV functions are used to
process the data.

---

### GetNumThreads

Returns the current number of threads used

```
int cvGetNumThreads(void);
```

The function `cvGetNumThreads` return the current number of threads
that are used by parallelized (via OpenMP) OpenCV functions.

---

### SetNumThreads

Sets the number of threads

```
void cvSetNumThreads( int threads=0 );
```

threads: The number of threads.

The function `cvSetNumThreads` sets the number of threads
that are used by parallelized OpenCV functions. When the argument
is zero or negative, and at the beginning of the program,
the number of threads is set to the number of processors in the system,
as returned by the function `omp_get_num_procs()` from OpenMP runtime.

---

### GetThreadNum

Returns index of the current thread

```
int cvGetThreadNum( void );
```

The function `cvGetThreadNum` returns the index, from 0 to
cvGetNumThreads()-1, of the thread that called the function.
It is a wrapper for the function `omp_get_thread_num()` from OpenMP runtime.
The retrieved index may be used to access local-thread data inside the parallelized code fragments.

---

# Alphabetical List of Functions

---

### A

|  |  |  |
| --- | --- | --- |
| AbsDiff | AddWeighted | Avg |
| AbsDiffS | Alloc | AvgSdv |
| Add | And |  |
| AddS | AndS |  |

---

### B

|  |  |  |
| --- | --- | --- |
| BackProjectPCA |  |  |

---

### C

|  |  |  |
| --- | --- | --- |
| CalcCovarMatrix | CloneGraph | CreateGraph |
| CalcPCA | CloneImage | CreateGraphScanner |
| CartToPolar | CloneMat | CreateImage |
| Cbrt | CloneMatND | CreateImageHeader |
| CheckArr | CloneSeq | CreateMat |
| Circle | CloneSparseMat | CreateMatHeader |
| ClearGraph | Cmp | CreateMatND |
| ClearMemStorage | CmpS | CreateMatNDHeader |
| ClearND | ConvertScale | CreateMemStorage |
| ClearSeq | ConvertScaleAbs | CreateSeq |
| ClearSet | Copy | CreateSet |
| ClipLine | CountNonZero | CreateSparseMat |
| ClipLine | CreateChildMemStorage | CrossProduct |
| Clone | CreateData | CvtSeqToArray |

---

### D

|  |  |  |
| --- | --- | --- |
| DCT | Det | DrawContours |
| DFT | Div |  |
| DecRefData | DotProduct |  |

---

### E

|  |  |  |
| --- | --- | --- |
| EigenVV | EllipseBox | Error |
| Ellipse | EndWriteSeq | ErrorStr |
| Ellipse2Poly | EndWriteStruct | Exp |

---

### F

|  |  |  |
| --- | --- | --- |
| FastArctan | FindGraphEdgeByPtr | FlushSeqWriter |
| FillConvexPoly | FindType | Free |
| FillPoly | FirstType |  |
| FindGraphEdge | Flip |  |

---

### G

|  |  |  |
| --- | --- | --- |
| GEMM | GetMat | GetTickCount |
| Get\*D | GetModuleInfo | GetTickFrequency |
| GetCol | GetNextSparseNode | GraphAddEdge |
| GetDiag | GetNumThreads | GraphAddEdgeByPtr |
| GetDims | GetOptimalDFTSize | GraphAddVtx |
| GetElemType | GetRawData | GraphEdgeIdx |
| GetErrMode | GetReal\*D | GraphRemoveEdge |
| GetErrStatus | GetRootFileNode | GraphRemoveEdgeByPtr |
| GetFileNode | GetRow | GraphRemoveVtx |
| GetFileNodeByName | GetSeqElem | GraphRemoveVtxByPtr |
| GetFileNodeName | GetSeqReaderPos | GraphVtxDegree |
| GetGraphVtx | GetSetElem | GraphVtxDegreeByPtr |
| GetHashedKey | GetSize | GraphVtxIdx |
| GetImage | GetSubRect | GuiBoxReport |
| GetImageCOI | GetTextSize | Get |
| GetImageROI | GetThreadNum |  |

---

### I

|  |  |  |
| --- | --- | --- |
| InRange | InitLineIterator | InsertNodeIntoTree |
| InRangeS | InitMatHeader | InvSqrt |
| IncRefData | InitMatNDHeader | Invert |
| InitFont | InitSparseMatIterator | IsInf |
| InitImageHeader | InitTreeNodeIterator | IsNaN |

---

### K

|  |  |  |
| --- | --- | --- |
| KMeans2 |  |  |

---

### L

|  |  |  |
| --- | --- | --- |
| LUT | Load |  |
| Line | Log |  |

---

### M

|  |  |  |
| --- | --- | --- |
| Mahalonobis | MemStorageAlloc | MinS |
| MakeSeqHeaderForArray | MemStorageAllocString | MixChannels |
| Mat | Merge | Mul |
| Max | Min | MulSpectrums |
| MaxS | MinMaxLoc | MulTransposed |

---

### N

|  |  |  |
| --- | --- | --- |
| NextGraphItem | Norm | Not |
| NextTreeNode | Normalize | NulDevReport |

---

### O

|  |  |  |
| --- | --- | --- |
| OpenFileStorage | Or | OrS |

---

### P

|  |  |  |
| --- | --- | --- |
| PerspectiveTransform | Pow | Ptr\*D |
| PolarToCart | PrevTreeNode | PutText |
| PolyLine | ProjectPCA |  |

---

### R

|  |  |  |
| --- | --- | --- |
| RNG | ReadRealByName | ReleaseImageHeader |
| RandArr | ReadString | ReleaseMat |
| RandInt | ReadStringByName | ReleaseMatND |
| RandReal | Rectangle | ReleaseMemStorage |
| RandShuffle | RedirectError | ReleaseSparseMat |
| Range | Reduce | RemoveNodeFromTree |
| Read | RegisterModule | Repeat |
| ReadByName | RegisterType | ResetImageROI |
| ReadInt | Release | Reshape |
| ReadIntByName | ReleaseData | ReshapeMatND |
| ReadRawData | ReleaseFileStorage | RestoreMemStoragePos |
| ReadRawDataSlice | ReleaseGraphScanner | Round |
| ReadReal | ReleaseImage |  |

---

### S

|  |  |  |  |
| --- | --- | --- | --- |
| SVBkSb | SeqSlice | SetSeqReaderPos |  |
| SVD | SeqSort | SetZero |  |
| Save | Set | Solve |  |
| SaveMemStoragePos | Set\*D | SolveCubic |  |
| ScaleAdd | SetAdd | SolvePoly |  |
| SeqElemIdx | SetData | Sqrt |  |
| SeqInsert | SetErrMode | StartAppendToSeq |  |
| SeqInsertSlice | SetErrStatus | StartNextStream |  |
| SeqInvert | SetIPLAllocators | StartReadRawData |  |
| SeqPartition | SetIdentity | StartReadSeq |  |
| SeqPop | SetImageCOI | StartWriteSeq |  |
| SeqPopFront | SetImageROI | StartWriteStruct |  |
| SeqPopMulti | SetMemoryManager | StdErrReport |  |
| SeqPush | SetNew | Sub |  |
| SeqPushFront | SetNumThreads | SubRS |  |
| SeqPushMulti | SetReal\*D | SubS |  |
| SeqRemove | SetRemove | Sum |  |
| SeqRemoveSlice | SetRemoveByPtr | Set |  |
| SeqSearch | SetSeqBlockSize | Split |  |

---

### T

|  |  |  |
| --- | --- | --- |
| Trace | Transpose | TypeOf |
| Transform | TreeToNodeSeq |  |

---

### U

|  |  |  |
| --- | --- | --- |
| UnregisterType | UseOptimized |  |

---

### W

|  |  |  |
| --- | --- | --- |
| Write | WriteInt | WriteString |
| WriteComment | WriteRawData |  |
| WriteFileNode | WriteReal |  |

---

### X

|  |  |  |
| --- | --- | --- |
| Xor | XorS |  |

---

# List of Examples
