# Supplementary material for: Rapid whole brain imaging of neural activity in freely behaving larval zebrafish (Danio rerio)
Source: eLife. 2017 Sep 20;6:e28158. doi: 10.7554/eLife.28158 (PMC5644961; doi:10.7554/eLife.28158)
Supplement: Source code 3. [file elife-28158-code3.zip › RT analysis code/OpenCV1.1pre.zip!/OpenCV/docs/ref/opencvref_highgui.htm]

OpenCV: HighGUI Reference Manual

# HighGUI Reference Manual

---

- Simple GUI- Loading and Saving Images- Video I/O- Utility and System Functions- Alphabetical List of Functions

---

## HighGUI overview

TODO

---

## Simple GUI

---

### cvNamedWindow

Creates window

```
int cvNamedWindow( const char* name, int flags=CV_WINDOW_AUTOSIZE );
```

name: Name of the window which is used as window identifier and appears in the window caption. flags: Flags of the window. Currently the only supported flag is `CV_WINDOW_AUTOSIZE`. If it is set, window size is automatically adjusted to fit the displayed image (see cvShowImage), while user can not change the window size manually.

The function `cvNamedWindow`
creates a window which can be used as a placeholder for images and trackbars.
Created windows are referred by their names.

If the window with such a name already exists, the function does nothing.

---

### cvDestroyWindow

Destroys a window

```
void cvDestroyWindow( const char* name );
```

name: Name of the window to be destroyed.

The function `cvDestroyWindow`
destroys the window with a given name.

---

### cvDestroyAllWindows

Destroys all the HighGUI windows

```
void cvDestroyAllWindows(void);
```

The function `cvDestroyAllWindows`
destroys all the opened HighGUI windows.

---

### cvResizeWindow

Sets window size

```
void cvResizeWindow( const char* name, int width, int height );
```

name: Name of the window to be resized. width: New width height: New height

The function `cvResizeWindow` changes size of the window.

---

### cvMoveWindow

Sets window position

```
void cvMoveWindow( const char* name, int x, int y );
```

name: Name of the window to be resized. x: New x coordinate of top-left corner y: New y coordinate of top-left corner

The function `cvMoveWindow` changes position of the window.

---

### cvGetWindowHandle

Gets window handle by name

```
void* cvGetWindowHandle( const char* name );
```

name: Name of the window.

The function `cvGetWindowHandle`
returns native window handle (HWND in case of Win32 and GtkWidget in case of GTK+).

---

### cvGetWindowName

Gets window name by handle

```
const char* cvGetWindowName( void* window_handle );
```

window\_handle: Handle of the window.

The function `cvGetWindowName`
returns the name of window given its native handle
(HWND in case of Win32 and GtkWidget in case of GTK+).

---

### cvShowImage

Shows the image in the specified window

```
void cvShowImage( const char* name, const CvArr* image );
```

name: Name of the window. image: Image to be shown.

The function `cvShowImage`
shows the image in the specified window. If the window was created with `CV_WINDOW_AUTOSIZE`
flag then the image is shown with its original size, otherwise
the image is scaled to fit the window.

---

### cvCreateTrackbar

Creates the trackbar and attaches it to the specified window

```
CV_EXTERN_C_FUNCPTR( void (*CvTrackbarCallback)(int pos) );

int cvCreateTrackbar( const char* trackbar_name, const char* window_name,
                      int* value, int count, CvTrackbarCallback on_change );
```

trackbar\_name: Name of created trackbar. window\_name: Name of the window which will e used as a parent for created trackbar. value: Pointer to the integer variable, which value will reflect the position of the slider. Upon the creation the slider position is defined by this variable. count: Maximal position of the slider. Minimal position is always 0. on\_change: Pointer to the function to be called every time the slider changes the position. This function should be prototyped as `void Foo(int);`Can be NULL if callback is not required.

The function `cvCreateTrackbar`
creates the trackbar (a.k.a. slider or range control) with the specified name and range,
assigns the variable to be synchronized with trackbar position and specifies callback function
to be called on trackbar position change. The created trackbar is displayed on top
of given window.

---

### cvGetTrackbarPos

Retrieves trackbar position

```
int cvGetTrackbarPos( const char* trackbar_name, const char* window_name );
```

trackbar\_name: Name of trackbar. window\_name: Name of the window which is the parent of trackbar.

The function `cvGetTrackbarPos`
returns the current position of the specified trackbar.

---

### cvSetTrackbarPos

Sets trackbar position

```
void cvSetTrackbarPos( const char* trackbar_name, const char* window_name, int pos );
```

trackbar\_name: Name of trackbar. window\_name: Name of the window which is the parent of trackbar. pos: New position.

The function `cvSetTrackbarPos`
sets the position of the specified trackbar.

---

### cvSetMouseCallback

Assigns callback for mouse events

```
#define CV_EVENT_MOUSEMOVE      0
#define CV_EVENT_LBUTTONDOWN    1
#define CV_EVENT_RBUTTONDOWN    2
#define CV_EVENT_MBUTTONDOWN    3
#define CV_EVENT_LBUTTONUP      4
#define CV_EVENT_RBUTTONUP      5
#define CV_EVENT_MBUTTONUP      6
#define CV_EVENT_LBUTTONDBLCLK  7
#define CV_EVENT_RBUTTONDBLCLK  8
#define CV_EVENT_MBUTTONDBLCLK  9

#define CV_EVENT_FLAG_LBUTTON   1
#define CV_EVENT_FLAG_RBUTTON   2
#define CV_EVENT_FLAG_MBUTTON   4
#define CV_EVENT_FLAG_CTRLKEY   8
#define CV_EVENT_FLAG_SHIFTKEY  16
#define CV_EVENT_FLAG_ALTKEY    32

CV_EXTERN_C_FUNCPTR( void (*CvMouseCallback )(int event, int x, int y, int flags, void* param) );

void cvSetMouseCallback( const char* window_name, CvMouseCallback on_mouse, void* param=NULL );
```

window\_name: Name of the window. on\_mouse: Pointer to the function to be called every time mouse event occurs in the specified window. This function should be prototyped as ``` void Foo(int event, int x, int y, int flags, void* param); ``` where `event` is one of `CV_EVENT_*`, `x` and `y` are coordinates of mouse pointer in image coordinates (not window coordinates), `flags` is a combination of `CV_EVENT_FLAG`, and `param` is a user-defined parameter passed to the `cvSetMouseCallback` function call. param: User-defined parameter to be passed to the callback function.

The function `cvSetMouseCallback`
sets the callback function for mouse events occurring within the specified
window. To see how it works, look at 
opencv/samples/c/ffilldemo.c demo

---

### cvWaitKey

Waits for a pressed key

```
int cvWaitKey( int delay=0 );
```

delay: Delay in milliseconds.

The function `cvWaitKey` waits for key event infinitely (delay<=0) or for "delay" milliseconds.
Returns the code of the pressed key or -1 if no key were pressed until the specified timeout has elapsed.

**Note**: This function is the only method in HighGUI to fetch and handle events so
it needs to be called periodically for normal event processing, unless HighGUI is used
within some environment that takes care of event processing.

---

## Loading and Saving Images

---

### cvLoadImage

Loads an image from file

```
/* 8 bit, color or gray - deprecated, use CV_LOAD_IMAGE_ANYCOLOR */
#define CV_LOAD_IMAGE_UNCHANGED  -1
/* 8 bit, gray */
#define CV_LOAD_IMAGE_GRAYSCALE   0
/* 8 bit unless combined with CV_LOAD_IMAGE_ANYDEPTH, color */
#define CV_LOAD_IMAGE_COLOR       1
/* any depth, if specified on its own gray */
#define CV_LOAD_IMAGE_ANYDEPTH    2
/* by itself equivalent to CV_LOAD_IMAGE_UNCHANGED
   but can be modified with CV_LOAD_IMAGE_ANYDEPTH */
#define CV_LOAD_IMAGE_ANYCOLOR    4

IplImage* cvLoadImage( const char* filename, int flags=CV_LOAD_IMAGE_COLOR );
```

filename: Name of file to be loaded. flags: Specifies colorness and depth of the loaded image: The colorness specifies whether the loaded image is to be converted to 3 channels (CV\_LOAD\_IMAGE\_COLOR), 1 channel (CV\_LOAD\_IMAGE\_GRAYSCALE), or left as it was in the input file (CV\_LOAD\_IMAGE\_ANYCOLOR). Depth specifies whether the loaded image is to be converted to 8 bits per pixel per color channel as was customary in previous versions of OpenCV or left as they were in the input file. If CV\_LOAD\_IMAGE\_ANYDEPTH is passed the pixel format can be 8 bit unsigned, 16 bit unsigned, 32 bit signed or 32 bit floating point. If conflicting flags are passed the flag with the smaller numerical value wins. That is if CV\_LOAD\_IMAGE\_COLOR | CV\_LOAD\_IMAGE\_ANYCOLOR is passed the image is loaded with 3 channels. CV\_LOAD\_IMAGE\_ANYCOLOR is equivalent to specifying CV\_LOAD\_IMAGE\_UNCHANGED. However, CV\_LOAD\_IMAGE\_ANYCOLOR has the advantage that it can be combined with CV\_LOAD\_IMAGE\_ANYDEPTH. So CV\_LOAD\_IMAGE\_UNCHANGED should not be used any longer. If you want to load the image as truthfully as possible pass CV\_LOAD\_IMAGE\_ANYDEPTH | CV\_LOAD\_IMAGE\_ANYCOLOR.

The function `cvLoadImage` loads an image
from the specified file and returns the pointer to the loaded image.
Currently the following file formats are supported:

- Windows bitmaps - BMP, DIB;- JPEG files - JPEG, JPG, JPE;- Portable Network Graphics - PNG;- Portable image format - PBM, PGM, PPM, PXM, PNM;- Sun rasters - SR, RAS;- TIFF files - TIFF, TIF;- OpenEXR HDR images - EXR;- JPEG 2000 images - jp2.

---

### cvSaveImage

Saves an image to the file

```
int cvSaveImage( const char* filename, const CvArr* image );
```

filename: Name of the file. image: Image to be saved.

The function `cvSaveImage`
saves the image to the specified file.
The image format is chosen depending on the `filename` extension, see
cvLoadImage.
Only 8-bit single-channel or 3-channel
(with 'BGR' channel order) images can be saved using this function.
If the format, depth or channel order is different, use `cvCvtScale`
and `cvCvtColor` to convert it before saving, or use
universal `cvSave` to save the image to XML or YAML format.

---

## Video I/O functions

---

### CvCapture

Video capturing structure

```
typedef struct CvCapture CvCapture;
```

The structure CvCapture
does not have public interface and is used only as a parameter for video
capturing functions.

---

### cvCreateFileCapture

Initializes capturing video from file

```
CvCapture* cvCreateFileCapture( const char* filename );
```

filename: Name of the video file.

The function `cvCreateFileCapture`
allocates and initialized the CvCapture structure for reading the video stream
from the specified file.

After the allocated structure is not used any more it should be released by
cvReleaseCapture function.

---

### cvCreateCameraCapture

Initializes capturing video from camera

```
CvCapture* cvCreateCameraCapture( int index );
```

index: Index of the camera to be used. If there is only one camera or it does not matter what camera to use -1 may be passed.

The function `cvCreateCameraCapture`
allocates and initialized the CvCapture structure for reading a video stream
from the camera. Currently two camera interfaces can be used on Windows: Video for
Windows (VFW) and Matrox Imaging Library (MIL); and two on Linux:
V4L and FireWire (IEEE1394).

To release the structure, use cvReleaseCapture.

---

### cvReleaseCapture

Releases the CvCapture structure

```
void cvReleaseCapture( CvCapture** capture );
```

capture: pointer to video capturing structure.

The function `cvReleaseCapture`
releases the CvCapture structure allocated by cvCreateFileCapture
or cvCreateCameraCapture.

---

### cvGrabFrame

Grabs frame from camera or file

```
int cvGrabFrame( CvCapture* capture );
```

capture: video capturing structure.

The function `cvGrabFrame`
grabs the frame from camera or file. The grabbed frame is stored internally.
The purpose of this function is to grab frame *fast* that is important
for synchronization in case of reading from several cameras simultaneously. The
grabbed frames are not exposed because they may be stored in compressed format
(as defined by camera/driver). To retrieve the grabbed frame,
cvRetrieveFrame should be used.

---

### cvRetrieveFrame

Gets the image grabbed with cvGrabFrame

```
IplImage* cvRetrieveFrame( CvCapture* capture );
```

capture: video capturing structure.

The function `cvRetrieveFrame`
returns the pointer to the image grabbed with cvGrabFrame
function. The returned image should not be released or modified by user.

---

### cvQueryFrame

Grabs and returns a frame from camera or file

```
IplImage* cvQueryFrame( CvCapture* capture );
```

capture: video capturing structure.

The function `cvQueryFrame`
grabs a frame from camera or video file, decompresses and returns it.
This function is just a combination of cvGrabFrame and
cvRetrieveFrame in one call. The
returned image should not be released or modified by user.

---

### cvGetCaptureProperty

Gets video capturing properties

```
double cvGetCaptureProperty( CvCapture* capture, int property_id );
```

capture: video capturing structure. property\_id: property identifier. Can be one of the following: `CV_CAP_PROP_POS_MSEC` - film current position in milliseconds or video capture timestamp `CV_CAP_PROP_POS_FRAMES` - 0-based index of the frame to be decoded/captured next `CV_CAP_PROP_POS_AVI_RATIO` - relative position of video file (0 - start of the film, 1 - end of the film) `CV_CAP_PROP_FRAME_WIDTH` - width of frames in the video stream `CV_CAP_PROP_FRAME_HEIGHT` - height of frames in the video stream `CV_CAP_PROP_FPS` - frame rate `CV_CAP_PROP_FOURCC` - 4-character code of codec. `CV_CAP_PROP_FRAME_COUNT` - number of frames in video file.

The function `cvGetCaptureProperty`
retrieves the specified property of camera or video file.

---

### cvSetCaptureProperty

Sets video capturing properties

```
int cvSetCaptureProperty( CvCapture* capture, int property_id, double value );
```

capture: video capturing structure. property\_id: property identifier. Can be one of the following: `CV_CAP_PROP_POS_MSEC` - position in milliseconds from the file beginning `CV_CAP_PROP_POS_FRAMES` - position in frames (only for video files) `CV_CAP_PROP_POS_AVI_RATIO` - position in relative units (0 - start of the file, 1 - end of the file) `CV_CAP_PROP_FRAME_WIDTH` - width of frames in the video stream (only for cameras) `CV_CAP_PROP_FRAME_HEIGHT` - height of frames in the video stream (only for cameras) `CV_CAP_PROP_FPS` - frame rate (only for cameras) `CV_CAP_PROP_FOURCC` - 4-character code of codec (only for cameras). value: value of the property.

The function `cvSetCaptureProperty`
sets the specified property of video capturing. Currently the function supports only
video files: `CV_CAP_PROP_POS_MSEC, CV_CAP_PROP_POS_FRAMES, CV_CAP_PROP_POS_AVI_RATIO`

---

### cvCreateVideoWriter

Creates video file writer

```
typedef struct CvVideoWriter CvVideoWriter;
CvVideoWriter* cvCreateVideoWriter( const char* filename, int fourcc, double fps, CvSize frame_size, int is_color=1 );
```

filename: Name of the output video file. fourcc: 4-character code of codec used to compress the frames. For example, `CV_FOURCC('P','I','M','1')` is MPEG-1 codec, `CV_FOURCC('M','J','P','G')` is motion-jpeg codec etc. There are two special values that can be passed, that behave as follows: `CV_FOURCC_PROMPT` - Opens a dialog box where the user can choose the compression method and parameters (WIN32 only). `CV_FOURCC_DEFAULT` - Use the default compression method for the given file extension (Linux only). fps: Framerate of the created video stream. frame\_size: Size of video frames. is\_color: If it is not zero, the encoder will expect and encode color frames, otherwise it will work with grayscale frames (the flag is currently supported on Windows only).

The function `cvCreateVideoWriter`
creates video writer structure.

---

### cvReleaseVideoWriter

Releases video file writer

```
void cvReleaseVideoWriter( CvVideoWriter** writer );
```

writer: pointer to video file writer structure.

The function `cvReleaseVideoWriter`
finishes writing to video file and releases the structure.

---

### cvWriteFrame

Writes a frame to video file

```
int cvWriteFrame( CvVideoWriter* writer, const IplImage* image );
```

writer: video writer structure. image : the written frame

The function `cvWriteFrame`
writes/appends one frame to video file.

---

## Utility and System Functions

---

### cvInitSystem

Initializes HighGUI

```
int cvInitSystem( int argc, char** argv );
```

argc: Number of command line arguments. argv: Array of command line arguments

The function `cvInitSystem`
initializes HighGUI. If it wasn't called explicitly by the user before the first window
is created, it is called implicitly then with `argc`=0, `argv`=NULL.
Under Win32 there is no need to call it explicitly. Under X Window the arguments may
be used to customize a look of HighGUI windows and controls.

---

### cvConvertImage

Converts one image to another with optional vertical flip

```
void cvConvertImage( const CvArr* src, CvArr* dst, int flags=0 );
```

src: Source image. dst: Destination image. Must be single-channel or 3-channel 8-bit image. flags : The operation flags: `CV_CVTIMG_FLIP` - flip the image vertically `CV_CVTIMG_SWAP_RB` - swap red and blue channels. In OpenCV color images have `BGR` channel order, however on some systems the order needs to be reversed before displaying the image (cvShowImage does this automatically).

The function `cvConvertImage`
converts one image to another and flips the result vertically if required.
The function is used by cvShowImage.

---

# Alphabetical List of Functions

---

### C

|  |  |  |
| --- | --- | --- |
| ConvertImage | CreateFileCapture | CreateVideoWriter |
| CreateCameraCapture | CreateTrackbar |  |

---

### D

|  |  |  |
| --- | --- | --- |
| DestroyAllWindows | DestroyWindow |  |

---

### G

|  |  |  |
| --- | --- | --- |
| GetCaptureProperty | GetWindowHandle | GrabFrame |
| GetTrackbarPos | GetWindowName |  |

---

### I

|  |  |  |
| --- | --- | --- |
| InitSystem |  |  |

---

### L

|  |  |  |
| --- | --- | --- |
| LoadImage |  |  |

---

### M

|  |  |  |
| --- | --- | --- |
| MoveWindow |  |  |

---

### N

|  |  |  |
| --- | --- | --- |
| NamedWindow |  |  |

---

### Q

|  |  |  |
| --- | --- | --- |
| QueryFrame |  |  |

---

### R

|  |  |  |
| --- | --- | --- |
| ReleaseCapture | ResizeWindow |  |
| ReleaseVideoWriter | RetrieveFrame |  |

---

### S

|  |  |  |
| --- | --- | --- |
| SaveImage | SetMouseCallback | ShowImage |
| SetCaptureProperty | SetTrackbarPos |  |

---

### W

|  |  |  |
| --- | --- | --- |
| WaitKey | WriteFrame |  |
