# Supplementary material for: Rapid whole brain imaging of neural activity in freely behaving larval zebrafish (Danio rerio)
Source: eLife. 2017 Sep 20;6:e28158. doi: 10.7554/eLife.28158 (PMC5644961; doi:10.7554/eLife.28158)
Supplement: Source code 3. [file elife-28158-code3.zip › RT analysis code/OpenCV1.1pre.zip!/OpenCV/docs/ref/opencvref_ml.htm]

Machine Learning Reference

# Machine Learning Reference

---

- Introduction. Common classes and functions- Normal Bayes Classifier- K Nearest Neighbors- Support Vector Machine- Decision Trees- Boosting- Random Trees- Expectation-Maximization- Neural Networks

---

## Introduction. Common classes and functions

The Machine Learning Library (MLL) is a set of classes and functions for statistical classification,
regression and clustering of data.

Most of the classification and regression algorithms are implemented as C++ classes.
As the algorithms have different set of features (like ability to handle missing measurements,
or categorical input variables etc.), there is a little common ground between the classes.
This common ground is defined by the class `CvStatModel` that all
the other ML classes are derived from.

---

### CvStatModel

Base class for statistical models in ML

```
class CvStatModel
{
public:
    /* CvStatModel(); */
    /* CvStatModel( const CvMat* train_data ... ); */

    virtual ~CvStatModel();

    virtual void clear()=0;

    /* virtual bool train( const CvMat* train_data, [int tflag,] ..., const CvMat* responses, ...,
    [const CvMat* var_idx,] ..., [const CvMat* sample_idx,] ...
    [const CvMat* var_type,] ..., [const CvMat* missing_mask,] <misc_training_alg_params> ... )=0;
     */

    /* virtual float predict( const CvMat* sample ... ) const=0; */

    virtual void save( const char* filename, const char* name=0 )=0;
    virtual void load( const char* filename, const char* name=0 )=0;

    virtual void write( CvFileStorage* storage, const char* name )=0;
    virtual void read( CvFileStorage* storage, CvFileNode* node )=0;
};
```

In this declaration some methods are commented off.
Actually, these are methods for which there is no unified API
(with the exception of the default constructor), however, there are many similarities
in the syntax and semantics that are briefly described below in this section,
as if they are a part of the base class.

---

### CvStatModel::CvStatModel

Default constructor

```
CvStatModel::CvStatModel();
```

Each statistical model class in ML has default constructor without parameters.
This constructor is useful for 2-stage model construction, when the default constructor
is followed by train() or
load().

---

### CvStatModel::CvStatModel(...)

Training constructor

```
CvStatModel::CvStatModel( const CvMat* train_data ... ); */
```

Most ML classes provide single-step construct+train constructor.
This constructor is equivalent to the default constructor, followed by the
train() method with the parameters that
passed to the constructor.

---

### CvStatModel::~CvStatModel

Virtual destructor

```
CvStatModel::~CvStatModel();
```

The destructor of the base class is declared as virtual,
so it is safe to write the following code:

```
CvStatModel* model;
if( use_svm )
    model = new CvSVM(... /* SVM params */);
else
    model = new CvDTree(... /* Decision tree params */);
...
delete model;
```

Normally, the destructor of each derived class does nothing, but calls
the overridden method clear() that deallocates all the memory.

---

### CvStatModel::clear

Deallocates memory and resets the model state

```
void CvStatModel::clear();
```

The method `clear` does the same job as the destructor, i.e. it deallocates all the memory
occupied by the class members. But the object itself is not destructed, and it can be reused further.
This method is called from the destructor, from the `train` methods of the derived classes,
from the methods load(),
read() etc., or even explicitly by user.

---

### CvStatModel::save

Saves the model to file

```
void CvStatModel::save( const char* filename, const char* name=0 );
```

The method `save` stores the complete model state to the specified XML or YAML file
with the specified name or default name (that depends on the particular class).
Data persistence
functionality from cxcore is used.

---

### CvStatModel::load

Loads the model from file

```
void CvStatModel::load( const char* filename, const char* name=0 );
```

The method `load` loads the complete model state with the specified
name
(or default model-dependent name) from the specified XML or YAML file. The
previous model state is cleared by clear().

Note that the method is virtual, therefore any model can be loaded using this virtual method.
However, unlike the C types of OpenCV that can be loaded using generic
cvLoad(), in this case the model type
must be known anyway, because an empty model, an instance of the appropriate class,
must be constructed beforehand.
This limitation will be removed in the later ML versions.

---

### CvStatModel::write

Writes the model to file storage

```
void CvStatModel::write( CvFileStorage* storage, const char* name );
```

The method `write` stores the complete model state
to the file storage
with the specified name or default name (that depends on the particular class).
The method is called by save().

---

### CvStatModel::read

Reads the model from file storage

```
void CvStatMode::read( CvFileStorage* storage, CvFileNode* node );
```

The method `read` restores the complete model state from the specified node
of the file storage. The node must be located by user, for example, using
the function cvGetFileNodeByName().
The method is called by load().

The previous model state is cleared by clear().

---

### CvStatModel::train

Trains the model

```
bool CvStatMode::train( const CvMat* train_data, [int tflag,] ..., const CvMat* responses, ...,
    [const CvMat* var_idx,] ..., [const CvMat* sample_idx,] ...
    [const CvMat* var_type,] ..., [const CvMat* missing_mask,] <misc_training_alg_params> ... );
```

The method trains the statistical model using a set of input feature vectors and the corresponding
output values (responses). Both input and output vectors/values are passed as matrices.
By default the input feature vectors are stored as `train_data` rows,
i.e. all the components (features) of a training vector are stored
continuously. However, some algorithms can handle the transposed representation,
when all values of each particular feature (component/input variable)
over the whole input set are stored continuously. If both layouts are supported, the method
includes `tflag` parameter that specifies the orientation:  
`tflag=CV_ROW_SAMPLE` means that the feature vectors are stored as rows,  
`tflag=CV_COL_SAMPLE` means that the feature vectors are stored as columns.  
The `train_data` must have
`32fC1` (32-bit floating-point, single-channel) format.
Responses are usually stored in the 1d
vector (a row or a column) of `32sC1` (only in the classification problem) or
`32fC1` format, one value per an input vector (although some algorithms, like various flavors of neural nets,
take vector responses).

For classification problems the responses are discrete class labels,
for regression problems - the responses are values of the function to be approximated.
Some algorithms can deal only with classification problems, some - only with regression problems,
and some can deal with both problems. In the latter case the type of output variable is either passed as separate parameter,
or as a last element of `var_type` vector:  
`CV_VAR_CATEGORICAL` means that the output values are discrete class labels,  
`CV_VAR_ORDERED(=CV_VAR_NUMERICAL)` means that the output values are ordered, i.e.
2 different values can be compared as numbers, and this is a regression problem  
The types of input variables can be also specified using `var_type`.
Most algorithms can handle only ordered input variables.

Many models in the ML may be trained on a selected feature subset,
and/or on a selected sample subset of the training set. To make it easier for user,
the method `train` usually includes `var_idx`
and `sample_idx` parameters. The former identifies variables (features) of interest,
and the latter identifies samples of interest. Both vectors are either integer (`32sC1`)
vectors, i.e. lists of 0-based indices, or 8-bit (`8uC1`) masks of active variables/samples.
User may pass `NULL` pointers instead of either of the argument, meaning that
all the variables/samples are used for training.

Additionally some algorithms can handle missing measurements,
that is when certain features of
certain training samples have unknown values (for example, they forgot to
measure a temperature
of patient A on Monday). The parameter `missing_mask`, 8-bit matrix of the same size as
`train_data`, is used to mark the missed values (non-zero elements of the mask).

Usually, the previous model state is cleared by
clear() before running the training procedure.
However, some algorithms may optionally update the model state with the new
training data, instead of resetting it.


---

### CvStatModel::predict

Predicts the response for sample

```
float CvStatMode::predict( const CvMat* sample[, <prediction_params>] ) const;
```

The method is used to predict the response for a new sample. In case of
classification the method returns the class label, in case of regression - the
output function value. The input sample must have as many components as the
`train_data` passed to train
contains. If the `var_idx` parameter is passed to train,
it is remembered and then is used to extract only the necessary components from the input sample
in the method `predict`.

The suffix "const" means that prediction does not affect the internal model state, so
the method can be safely called from within different threads.


---

## Normal Bayes Classifier

This is a simple classification model assuming that feature vectors from each class
are normally distributed (though, not necessarily independently distributed),
so the whole data distribution function is assumed to be a Gaussian mixture, one component per a class.
Using the training data the algorithm estimates mean vectors and covariation matrices for every class,
and then it uses them for prediction.

**[Fukunaga90] K. Fukunaga.
Introduction to Statistical Pattern Recognition. second ed., New York: Academic Press, 1990.**

---

### CvNormalBayesClassifier

Bayes classifier for normally distributed data

```
class CvNormalBayesClassifier : public CvStatModel
{
public:
    CvNormalBayesClassifier();
    virtual ~CvNormalBayesClassifier();

    CvNormalBayesClassifier( const CvMat* _train_data, const CvMat* _responses,
        const CvMat* _var_idx=0, const CvMat* _sample_idx=0 );

    virtual bool train( const CvMat* _train_data, const CvMat* _responses,
        const CvMat* _var_idx = 0, const CvMat* _sample_idx=0, bool update=false );

    virtual float predict( const CvMat* _samples, CvMat* results=0 ) const;
    virtual void clear();

    virtual void save( const char* filename, const char* name=0 );
    virtual void load( const char* filename, const char* name=0 );

    virtual void write( CvFileStorage* storage, const char* name );
    virtual void read( CvFileStorage* storage, CvFileNode* node );
protected:
    ...
};
```

---

### CvNormalBayesClassifier::train

Trains the model

```
bool CvNormalBayesClassifier::train( const CvMat* _train_data, const CvMat* _responses,
               const CvMat* _var_idx = 0, const CvMat* _sample_idx=0, bool update=false );
```

The method trains the Normal Bayes classifier. It follows the conventions of
generic train "method" with the following limitations:
only CV\_ROW\_SAMPLE data layout is supported; the input variables are all ordered;
the output variable is categorical (i.e. elements of `_responses`
must be integer numbers, though the vector may have `32fC1` type),
missing measurements are not supported.

In addition, there is `update` flag that identifies, whether the model should
be trained from scratch (`update=false`) or should be updated using new training data
(`update=true`).

---

### CvNormalBayesClassifier::predict

Predicts the response for sample(s)

```
float CvNormalBayesClassifier::predict( const CvMat* samples, CvMat* results=0 ) const;
```

The method `predict` estimates the most probable classes for the input vectors.
The input vectors (one or more) are stored as rows of the matrix `samples`.
In case of multiple input vectors, there should be output vector `results`.
The predicted class for a single input vector is returned by the method.


---

## K Nearest Neighbors

The algorithm caches all the training samples, and it predicts the response for
a new sample by analyzing a certain number (*K*) of the nearest neighbors
of the sample (using voting, calculating weighted sum etc.) The method is
sometimes referred to as "learning by example", i.e. for prediction it looks for
the feature vector
with a known response that is closest to the given vector.

---

### CvKNearest

K Nearest Neighbors model

```
class CvKNearest : public CvStatModel
{
public:

    CvKNearest();
    virtual ~CvKNearest();

    CvKNearest( const CvMat* _train_data, const CvMat* _responses,
                const CvMat* _sample_idx=0, bool _is_regression=false, int max_k=32 );

    virtual bool train( const CvMat* _train_data, const CvMat* _responses,
                        const CvMat* _sample_idx=0, bool is_regression=false,
                        int _max_k=32, bool _update_base=false );

    virtual float find_nearest( const CvMat* _samples, int k, CvMat* results,
        const float** neighbors=0, CvMat* neighbor_responses=0, CvMat* dist=0 ) const;

    virtual void clear();
    int get_max_k() const;
    int get_var_count() const;
    int get_sample_count() const;
    bool is_regression() const;

protected:
    ...
};
```

---

### CvKNearest::train

Trains the model

```
bool CvKNearest::train( const CvMat* _train_data, const CvMat* _responses,
                        const CvMat* _sample_idx=0, bool is_regression=false,
                        int _max_k=32, bool _update_base=false );
```

The method trains the K-Nearest model. It follows the conventions of
generic train "method" with the following limitations:
only CV\_ROW\_SAMPLE data layout is supported, the input variables are all ordered,
the output variables can be either categorical (`is_regression=false`)
or ordered (`is_regression=true`), variable subsets (`var_idx`) and
missing measurements are not supported.

The parameter `_max_k` specifies the number of maximum neighbors that may be
passed to the method find\_nearest.

The parameter `_update_base` specifies, whether the model is trained from scratch (`_update_base=false`), or
it is updated using the new training data
(`_update_base=true`). In the latter case the parameter `_max_k`
must not be larger than the original value.

---

### CvKNearest::find\_nearest

Finds the neighbors for the input vectors

```
float CvKNearest::find_nearest( const CvMat* _samples, int k, CvMat* results=0,
        const float** neighbors=0, CvMat* neighbor_responses=0, CvMat* dist=0 ) const;
```

For each input vector (which are rows of the matrix `_samples`)
the method finds `k≤get_max_k()` nearest neighbor. In case of regression,
the predicted result will be a mean value of the particular vector's neighbor responses.
In case of classification the class is determined by voting.

For custom classification/regression prediction, the method can optionally return
pointers to the neighbor vectors themselves (`neighbors`, array of
`k*_samples->rows` pointers), their corresponding output values
(`neighbor_responses`, a vector of `k*_samples->rows` elements)
and the distances from the input vectors to the neighbors
(`dist`, also a vector of `k*_samples->rows` elements).

For each input vector the neighbors are sorted by their distances to the vector.

If only a single input vector is passed, all output matrices are optional and the
predicted value is returned by the method.

---

### Example. Classification of 2D samples from a Gaussian mixture with k-nearest classifier

```
#include "ml.h"
#include "highgui.h"

int main( int argc, char** argv )
{
    const int K = 10;
    int i, j, k, accuracy;
    float response;
    int train_sample_count = 100;
    CvRNG rng_state = cvRNG(-1);
    CvMat* trainData = cvCreateMat( train_sample_count, 2, CV_32FC1 );
    CvMat* trainClasses = cvCreateMat( train_sample_count, 1, CV_32FC1 );
    IplImage* img = cvCreateImage( cvSize( 500, 500 ), 8, 3 );
    float _sample[2];
    CvMat sample = cvMat( 1, 2, CV_32FC1, _sample );
    cvZero( img );

    CvMat trainData1, trainData2, trainClasses1, trainClasses2;

    // form the training samples
    cvGetRows( trainData, &trainData1, 0, train_sample_count/2 );
    cvRandArr( &rng_state, &trainData1, CV_RAND_NORMAL, cvScalar(200,200), cvScalar(50,50) );

    cvGetRows( trainData, &trainData2, train_sample_count/2, train_sample_count );
    cvRandArr( &rng_state, &trainData2, CV_RAND_NORMAL, cvScalar(300,300), cvScalar(50,50) );

    cvGetRows( trainClasses, &trainClasses1, 0, train_sample_count/2 );
    cvSet( &trainClasses1, cvScalar(1) );

    cvGetRows( trainClasses, &trainClasses2, train_sample_count/2, train_sample_count );
    cvSet( &trainClasses2, cvScalar(2) );

    // learn classifier
    CvKNearest knn( trainData, trainClasses, 0, false, K );
    CvMat* nearests = cvCreateMat( 1, K, CV_32FC1);

    for( i = 0; i < img->height; i++ )
    {
        for( j = 0; j < img->width; j++ )
        {
            sample.data.fl[0] = (float)j;
            sample.data.fl[1] = (float)i;

            // estimates the response and get the neighbors' labels
            response = knn.find_nearest(&sample,K,0,0,nearests,0);

            // compute the number of neighbors representing the majority
            for( k = 0, accuracy = 0; k < K; k++ )
            {
                if( nearests->data.fl[k] == response)
                    accuracy++;
            }
            // highlight the pixel depending on the accuracy (or confidence)
            cvSet2D( img, i, j, response == 1 ?
                (accuracy > 5 ? CV_RGB(180,0,0) : CV_RGB(180,120,0)) :
                (accuracy > 5 ? CV_RGB(0,180,0) : CV_RGB(120,120,0)) );
        }
    }

    // display the original training samples
    for( i = 0; i < train_sample_count/2; i++ )
    {
        CvPoint pt;
        pt.x = cvRound(trainData1.data.fl[i*2]);
        pt.y = cvRound(trainData1.data.fl[i*2+1]);
        cvCircle( img, pt, 2, CV_RGB(255,0,0), CV_FILLED );
        pt.x = cvRound(trainData2.data.fl[i*2]);
        pt.y = cvRound(trainData2.data.fl[i*2+1]);
        cvCircle( img, pt, 2, CV_RGB(0,255,0), CV_FILLED );
    }

    cvNamedWindow( "classifier result", 1 );
    cvShowImage( "classifier result", img );
    cvWaitKey(0);

    cvReleaseMat( &trainClasses );
    cvReleaseMat( &trainData );
    return 0;
}
```


---

## Support Vector Machines

Originally, support vector machines (SVM) was a technique for building an optimal (in some sense)
binary (2-class) classifier. Then the technique has been extended to regression and clustering problems.
SVM is a partial case of kernel-based methods,
it maps feature vectors into higher-dimensional space using some kernel function, and then it builds
an optimal linear discriminating function in this space (or an optimal hyper-plane that fits into
the training data, ...). In case of SVM the kernel is not defined explicitly. Instead, a distance
between any 2 points in the hyper-space needs to be defined.

The solution is optimal in a sense that the margin between the separating hyper-plane and the
nearest feature vectors from the both classes (in case of 2-class classifier) is maximal. The
feature vectors that are the closest to the hyper-plane are called "support vectors", meaning that
the position of other vectors does not affect the hyper-plane (the decision function).

There are a lot of good references on SVM. Here are only a few ones to start with.

**[Burges98] C. Burges. "A tutorial on support vector machines for pattern recognition", Knowledge Discovery and Data
Mining 2(2), 1998.**  
(available online at http://citeseer.ist.psu.edu/burges98tutorial.html).  
**LIBSVM - A Library for Support Vector Machines. By Chih-Chung Chang and Chih-Jen Lin**  
(http://www.csie.ntu.edu.tw/~cjlin/libsvm/)

---

### CvSVM

Support Vector Machines

```
class CvSVM : public CvStatModel
{
public:
    // SVM type
    enum { C_SVC=100, NU_SVC=101, ONE_CLASS=102, EPS_SVR=103, NU_SVR=104 };

    // SVM kernel type
    enum { LINEAR=0, POLY=1, RBF=2, SIGMOID=3 };

    // SVM params type
    enum { C=0, GAMMA=1, P=2, NU=3, COEF=4, DEGREE=5 };

    CvSVM();
    virtual ~CvSVM();

    CvSVM( const CvMat* _train_data, const CvMat* _responses,
           const CvMat* _var_idx=0, const CvMat* _sample_idx=0,
           CvSVMParams _params=CvSVMParams() );

    virtual bool train( const CvMat* _train_data, const CvMat* _responses,
                        const CvMat* _var_idx=0, const CvMat* _sample_idx=0,
                        CvSVMParams _params=CvSVMParams() );

    virtual bool train_auto( const CvMat* _train_data, const CvMat* _responses,
        const CvMat* _var_idx, const CvMat* _sample_idx, CvSVMParams _params,
        int k_fold = 10,
        CvParamGrid C_grid      = get_default_grid(CvSVM::C),
        CvParamGrid gamma_grid  = get_default_grid(CvSVM::GAMMA),
        CvParamGrid p_grid      = get_default_grid(CvSVM::P),
        CvParamGrid nu_grid     = get_default_grid(CvSVM::NU),
        CvParamGrid coef_grid   = get_default_grid(CvSVM::COEF),
        CvParamGrid degree_grid = get_default_grid(CvSVM::DEGREE) );

    virtual float predict( const CvMat* _sample ) const;
    virtual int get_support_vector_count() const;
    virtual const float* get_support_vector(int i) const;
    virtual CvSVMParams get_params() const { return params; };
    virtual void clear();

    static CvParamGrid get_default_grid( int param_id );

    virtual void save( const char* filename, const char* name=0 );
    virtual void load( const char* filename, const char* name=0 );

    virtual void write( CvFileStorage* storage, const char* name );
    virtual void read( CvFileStorage* storage, CvFileNode* node );
    int get_var_count() const { return var_idx ? var_idx->cols : var_all; }

protected:
    ...
};
```

---

### CvSVMParams

SVM training parameters

```
struct CvSVMParams
{
    CvSVMParams();
    CvSVMParams( int _svm_type, int _kernel_type,
                 double _degree, double _gamma, double _coef0,
                 double _C, double _nu, double _p,
                 CvMat* _class_weights, CvTermCriteria _term_crit );

    int         svm_type;
    int         kernel_type;
    double      degree; // for poly
    double      gamma;  // for poly/rbf/sigmoid
    double      coef0;  // for poly/sigmoid

    double      C;  // for CV_SVM_C_SVC, CV_SVM_EPS_SVR and CV_SVM_NU_SVR
    double      nu; // for CV_SVM_NU_SVC, CV_SVM_ONE_CLASS, and CV_SVM_NU_SVR
    double      p; // for CV_SVM_EPS_SVR
    CvMat*      class_weights; // for CV_SVM_C_SVC
    CvTermCriteria term_crit; // termination criteria
};
```

svm\_type: Type of SVM, one of the following types: CvSVM::C\_SVC - n-class classification (n>=2), allows imperfect separation of classes with penalty multiplier `C` for outliers. CvSVM::NU\_SVC - n-class classification with possible imperfect separation. Parameter `nu` (in the range 0..1, the larger the value, the smoother the decision boundary) is used instead of `C`. CvSVM::ONE\_CLASS - one-class SVM. All the training data are from the same class, SVM builds a boundary that separates the class from the rest of the feature space. CvSVM::EPS\_SVR - regression. The distance between feature vectors from the training set and the fitting hyper-plane must be less than `p`. For outliers the penalty multiplier `C` is used. CvSVM::NU\_SVR - regression; `nu` is used instead of `p`. kernel\_type: The kernel type, one of the following types: CvSVM::LINEAR - no mapping is done, linear discrimination (or regression) is done in the original feature space. It is the fastest option. *d(x,y) = x•y == (x,y)* CvSVM::POLY - polynomial kernel: *d(x,y) = (gamma\*(x•y)+coef0)^degree^* CvSVM::RBF - radial-basis-function kernel; a good choice in most cases: *d(x,y) = exp(-gamma\*|x-y|^2^)* CvSVM::SIGMOID - sigmoid function is used as a kernel: *d(x,y) = tanh(gamma\*(x•y)+coef0)* degree, gamma, coef0: Parameters of the kernel, see the formulas above. C, nu, p: Parameters in the generalized SVM optimization problem. class\_weights: Optional weights, assigned to particular classes. They are multiplied by `C` and thus affect the misclassification penalty for different classes. The larger weight, the larger penalty on misclassification of data from the corresponding class. term\_crit: Termination procedure for iterative SVM training procedure (which solves a partial case of constrained quadratic optimization problem)

The structure must be initialized and passed to the training method of CvSVM


---

### CvSVM::train

Trains SVM

```
bool CvSVM::train( const CvMat* _train_data, const CvMat* _responses,
                   const CvMat* _var_idx=0, const CvMat* _sample_idx=0,
                   CvSVMParams _params=CvSVMParams() );
```

The method trains the SVM model. It follows the conventions of
generic train "method" with the following limitations:
only CV\_ROW\_SAMPLE data layout is supported, the input variables are all ordered,
the output variables can be either categorical (`_params.svm_type=CvSVM::C_SVC` or
`_params.svm_type=CvSVM::NU_SVC`), or ordered
(`_params.svm_type=CvSVM::EPS_SVR` or
`_params.svm_type=CvSVM::NU_SVR`), or not required at all
(`_params.svm_type=CvSVM::ONE_CLASS`),
missing measurements are not supported.

All the other parameters are gathered in CvSVMParams
structure.

---

### CvSVM::train\_auto

Trains SVM with optimal parameters

```
train_auto( const CvMat* _train_data, const CvMat* _responses,
        const CvMat* _var_idx, const CvMat* _sample_idx,
        CvSVMParams params, int k_fold = 10,
        CvParamGrid C_grid      = get_default_grid(CvSVM::C),
        CvParamGrid gamma_grid  = get_default_grid(CvSVM::GAMMA),
        CvParamGrid p_grid      = get_default_grid(CvSVM::P),
        CvParamGrid nu_grid     = get_default_grid(CvSVM::NU),
        CvParamGrid coef_grid   = get_default_grid(CvSVM::COEF),
        CvParamGrid degree_grid = get_default_grid(CvSVM::DEGREE) );
```

k\_fold: Cross-validation parameter. The training set is divided into *k\_fold* subsets, one subset being used to train the model, the others forming the test set. So, the SVM algorithm is executed *k\_fold* times.

The method trains the SVM model automatically by choosing
the optimal parameters *C*, *gamma*,
*p*, *nu*, *coef0*, *degree* from
CvSVMParams. By the optimality one mean that
the cross-validation estimate of the test set error is minimal.
The parameters are iterated by logarithmic grid, for example, the parameter
*gamma* takes the values in the set
{*gamma\_grid*.min\_val, *gamma\_grid*.min\_val\**gamma\_grid*.step,
*gamma\_grid*.min\_val\**gamma\_grid*.step^2^,...,
*gamma\_grid*.min\_val\**gamma\_grid*.step^*n*^},
where *n* is the maximal index such, that
*gamma\_grid*.min\_val\**gamma\_grid*.step^*n*^ <
*gamma\_grid*.max\_val. So, *step* must always be greater than 1.

If there is no need in optimization in some parameter, the according grid step should be
set to any value less or equal to 1. For example, to avoid optimization in
*gamma* one should set *gamma\_grid*.step = 0,
*gamma\_grid*.min\_val, *gamma\_grid*.max\_val being arbitrary numbers.
In this case, the value *params*.gamma will be taken for *gamma*.

And, finally, if the optimization in some parameter is required,
but there is no idea of the corresponding grid, one may call the function
CvSVM::get\_default\_grid.
In order to generate a grid, say,
for *gamma*, call `CvSVM::get_default_grid(CvSVM::GAMMA)`.

This function works for the case of classification
(`params.svm_type=CvSVM::C_SVC` or
`params.svm_type=CvSVM::NU_SVC`) as well as for the regression
(`params.svm_type=CvSVM::EPS_SVR`  or
`params.svm_type=CvSVM::NU_SVR`). If
`params.svm_type=CvSVM::ONE_CLASS`, no
optimization is made and usual SVM with
specified in `params` parameters is executed.

---

### CvSVM::get\_default\_grid

Generates a grid for SVM parameters

```
CvParamGrid CvSVM::get_default_grid( int param_id );
```

param\_id: Must be one of the following: `CvSVM::C, CvSVM::GAMMA, CvSVM::P, CvSVM::NU, CvSVM::COEF, CvSVM::DEGREE`. The grid will be generated for the parameter with this ID.

The function generates a grid for the specified parameter of the SVM algorithm.
The grid may be passed to the function
CvSVM::train\_auto

---

### CvSVM::get\_params

Returns current SVM parameters

```
CvSVMParams CvSVM::get_params() const;
```

This function may be used to get the optimal parameters that were obtained
while automatic training CvSVM::train\_auto.

---

### CvSVM::get\_support\_vector\*

Retrieves the number of support vectors and the particular vector

```
int CvSVM::get_support_vector_count() const;
const float* CvSVM::get_support_vector(int i) const;
```

The methods can be used to retrieve the set of support vectors.


---

## Decision Trees

The ML classes discussed in this section implement
Classification And Regression Tree algorithms, which is described in
[Breiman84].

The class CvDTree represents a single decision tree that
may be used alone, or as a base class in tree ensembles
(see Boosting and Random Trees).

Decision tree is a binary tree (i.e. tree where each non-leaf node has exactly 2 child nodes).
It can be used either for classification, when
each tree leaf is marked with some class label (multiple leafs may have the same label),
or for regression, when each tree leaf is also assigned a constant
(so the approximation function is piecewise constant).

### Predicting with Decision Trees

To reach a leaf node, and thus
to obtain a response for the input feature vector, the prediction procedure starts
with the root node. From each non-leaf node the procedure goes to the left (i.e. selects the
left child node as the next observed node), or to the right based on the value of
a certain variable, which index is stored in the observed node. The variable can be either
ordered or categorical. In the first case, the variable value is compared with the certain threshold
(which is also stored in the node); if the value is less than the threshold, the
procedure goes to the left,
otherwise, to the right (for example, if the weight is less than 1 kilogram, the
procedure goes to the left,
else to the right). And in the second case the discrete variable value is tested, whether it
belongs to a certain subset of values (also stored in the node)
from a limited set of values the variable could take;
if yes, the procedure goes to the left, else - to the right (for example,
if the color is green or red, go to the left, else to the right).
That is, in each node, a pair of entities (<variable\_index>, <decision\_rule (threshold/subset)>)
is used.
This pair is called split (split on the variable #<variable\_index>).
Once a leaf node is reached, the value assigned to this node is used as the output of prediction procedure.

Sometimes, certain features of the input vector are missed (for example, in the darkness
it is difficult to determine the object color), and the prediction procedure
may get stuck in the certain node (in the mentioned example if the node is split by color).
To avoid such situations, decision trees use so-called
surrogate splits. That is, in addition to the best "primary" split, every tree node may also
be split on one or more other variables with nearly the same results.

### Training Decision Trees

The tree is built recursively, starting from the root node. The whole training data (feature
vectors and the responses) are used to split the root node. In each node the optimum
decision rule (i.e. the best "primary" split) is found based on some criteria (in ML *gini* "purity" criteria is used
for classification, and sum of squared errors is used for regression). Then, if necessary,
the surrogate
splits are found that resemble at the most the results of the primary split on
the training data; all data are divided using the primary and the surrogate splits
(just like it is done in the prediction procedure)
between the left and the right child node. Then the procedure recursively splits both left and right
nodes etc. At each node the recursive procedure may stop (i.e. stop splitting the node further)
in one of the following cases:  

- depth of the tree branch being constructed has reached the specified maximum value.- number of training samples in the node is less than the specified threshold, i.e.
    it is not statistically representative set to split the node further.- all the samples in the node belong to the same class (or, in case of regression,
      the variation is too small).- the best split found does not give any noticeable improvement comparing to just a random
        choice.

When the tree is built, it may be pruned using cross-validation procedure, if need.
That is, some branches of the tree that may lead to the model overfitting are cut off.
Normally, this procedure is only applied to standalone decision trees, while tree ensembles
usually build small enough trees and use their own protection schemes against overfitting.

### Variable importance

Besides the obvious use of decision trees - prediction, the tree can be also
used
for various data analysis.
One of the key properties of the constructed decision tree algorithms is that it is possible
to compute importance (relative decisive power) of each variable. For example, in a spam
filter that uses a set of words occurred in the message as a feature vector, the variable importance
rating can be used to determine the most "spam-indicating" words and thus help to keep the dictionary
size reasonable.

Importance of each variable is computed over all the splits on this variable in the tree, primary
and surrogate ones. Thus, to compute variable importance correctly, the surrogate splits must be enabled
in the training parameters, even if there is no missing data.

**[Breiman84]
Breiman, L., Friedman, J. Olshen, R. and Stone, C. (1984), "Classification and Regression Trees", Wadsworth.**

---

### CvDTreeSplit

Decision tree node split

```
struct CvDTreeSplit
{
    int var_idx;
    int inversed;
    float quality;
    CvDTreeSplit* next;
    union
    {
        int subset[2];
        struct
        {
            float c;
            int split_point;
        }
        ord;
    };
};
```

var\_idx: Index of the variable used in the split inversed: When it equals to 1, the inverse split rule is used (i.e. left and right branches are exchanged in the expressions below) quality: The split quality, a positive number. It is used to choose the best primary split, then to choose and sort the surrogate splits. After the tree is constructed, it is also used to compute variable importance. next: Pointer to the next split in the node split list. subset: Bit array indicating the value subset in case of split on a categorical variable. The rule is: `if var_value in subset then next_node<-left else next_node<-right` c: The threshold value in case of split on an ordered variable. The rule is: `if var_value < c then next_node<-left else next_node<-right` split\_point: Used internally by the training algorithm.

---

### CvDTreeNode

Decision tree node

```
struct CvDTreeNode
{
    int class_idx;
    int Tn;
    double value;

    CvDTreeNode* parent;
    CvDTreeNode* left;
    CvDTreeNode* right;

    CvDTreeSplit* split;

    int sample_count;
    int depth;
    ...
};
```

value: The value assigned to the tree node. It is either a class label, or the estimated function value. class\_idx: The assigned to the node normalized class index (to 0..class\_count-1 range), it is used internally in classification trees and tree ensembles. Tn: The tree index in a ordered sequence of trees. The indices are used during and after the pruning procedure. The root node has the maximum value `Tn` of the whole tree, child nodes have `Tn` less than or equal to the parent's `Tn`, and the nodes with `Tn≤CvDTree::pruned_tree_idx` are not taken into consideration at the prediction stage (the corresponding branches are considered as cut-off), even if they have not been physically deleted from the tree at the pruning stage. parent, left, right: Pointers to the parent node, left and right child nodes. split: Pointer to the first (primary) split. sample\_count: The number of samples that fall into the node at the training stage. It is used to resolve the difficult cases - when the variable for the primary split is missing, and all the variables for other surrogate splits are missing too, the sample is directed to the left if `left->sample_count>right->sample_count` and to the right otherwise. depth: The node depth, the root node depth is 0, the child nodes depth is the parent's depth + 1.

Other numerous fields of `CvDTreeNode` are used internally at the training stage.

---

### CvDTreeParams

Decision tree training parameters

```
struct CvDTreeParams
{
    int max_categories;
    int max_depth;
    int min_sample_count;
    int cv_folds;
    bool use_surrogates;
    bool use_1se_rule;
    bool truncate_pruned_tree;
    float regression_accuracy;
    const float* priors;

    CvDTreeParams() : max_categories(10), max_depth(INT_MAX), min_sample_count(10),
        cv_folds(10), use_surrogates(true), use_1se_rule(true),
        truncate_pruned_tree(true), regression_accuracy(0.01f), priors(0)
    {}

    CvDTreeParams( int _max_depth, int _min_sample_count,
                   float _regression_accuracy, bool _use_surrogates,
                   int _max_categories, int _cv_folds,
                   bool _use_1se_rule, bool _truncate_pruned_tree,
                   const float* _priors );
};
```

max\_depth: This parameter specifies the maximum possible depth of the tree. That is the training algorithms attempts to split a node while its depth is less than `max_depth`. The actual depth may be smaller if the other termination criteria are met (see the outline of the training procedure in the beginning of the section), and/or if the tree is pruned. min\_sample\_count: A node is not split if the number of samples directed to the node is less than the parameter value. regression\_accuracy: Another stop criteria - only for regression trees. As soon as the estimated node value differs from the node training samples responses by less than the parameter value, the node is not split further. use\_surrogates: If `true`, surrogate splits are built. Surrogate splits are needed to handle missing measurements and for variable importance estimation. max\_categories: If a discrete variable, on which the training procedure tries to make a split, takes more than `max_categories` values, the precise best subset estimation may take a very long time (as the algorithm is exponential). Instead, many decision trees engines (including ML) try to find sub-optimal split in this case by clustering all the samples into `max_categories` clusters (i.e. some categories are merged together). Note that this technique is used only in `N(>2)`-class classification problems. In case of regression and 2-class classification the optimal split can be found efficiently without employing clustering, thus the parameter is not used in these cases. cv\_folds: If this parameter is >1, the tree is pruned using `cv_folds`-fold cross validation. use\_1se\_rule: If `true`, the tree is truncated a bit more by the pruning procedure. That leads to compact, and more resistant to the training data noise, but a bit less accurate decision tree. truncate\_pruned\_tree: If `true`, the cut off nodes (with `Tn`≤`CvDTree::pruned_tree_idx`) are physically removed from the tree. Otherwise they are kept, and by decreasing `CvDTree::pruned_tree_idx` (e.g. setting it to -1) it is still possible to get the results from the original un-pruned (or pruned less aggressively) tree. priors: The array of a priori class probabilities, sorted by the class label value. The parameter can be used to tune the decision tree preferences toward a certain class. For example, if users want to detect some rare anomaly occurrence, the training base will likely contain much more normal cases than anomalies, so a very good classification performance will be achieved just by considering every case as normal. To avoid this, the priors can be specified, where the anomaly probability is artificially increased (up to 0.5 or even greater), so the weight of the misclassified anomalies becomes much bigger, and the tree is adjusted properly. A note about memory management: the field `priors` is a pointer to the array of floats. The array should be allocated by user, and released just after the `CvDTreeParams` structure is passed to CvDTreeTrainData or CvDTree constructors/methods (as the methods make a copy of the array).

The structure contains all the decision tree training parameters.
There is a default constructor that initializes all the parameters with the default values
tuned for standalone classification tree. Any of the parameters can be
overridden then,
or the structure may be fully initialized using the advanced variant of the constructor.

---

### CvDTreeTrainData

Decision tree training data and shared data for tree ensembles

```
struct CvDTreeTrainData
{
    CvDTreeTrainData();
    CvDTreeTrainData( const CvMat* _train_data, int _tflag,
                      const CvMat* _responses, const CvMat* _var_idx=0,
                      const CvMat* _sample_idx=0, const CvMat* _var_type=0,
                      const CvMat* _missing_mask=0,
                      const CvDTreeParams& _params=CvDTreeParams(),
                      bool _shared=false, bool _add_labels=false );
    virtual ~CvDTreeTrainData();

    virtual void set_data( const CvMat* _train_data, int _tflag,
                          const CvMat* _responses, const CvMat* _var_idx=0,
                          const CvMat* _sample_idx=0, const CvMat* _var_type=0,
                          const CvMat* _missing_mask=0,
                          const CvDTreeParams& _params=CvDTreeParams(),
                          bool _shared=false, bool _add_labels=false,
                          bool _update_data=false );

    virtual void get_vectors( const CvMat* _subsample_idx,
         float* values, uchar* missing, float* responses, bool get_class_idx=false );

    virtual CvDTreeNode* subsample_data( const CvMat* _subsample_idx );

    virtual void write_params( CvFileStorage* fs );
    virtual void read_params( CvFileStorage* fs, CvFileNode* node );

    // release all the data
    virtual void clear();

    int get_num_classes() const;
    int get_var_type(int vi) const;
    int get_work_var_count() const;

    virtual int* get_class_labels( CvDTreeNode* n );
    virtual float* get_ord_responses( CvDTreeNode* n );
    virtual int* get_labels( CvDTreeNode* n );
    virtual int* get_cat_var_data( CvDTreeNode* n, int vi );
    virtual CvPair32s32f* get_ord_var_data( CvDTreeNode* n, int vi );
    virtual int get_child_buf_idx( CvDTreeNode* n );

    ////////////////////////////////////

    virtual bool set_params( const CvDTreeParams& params );
    virtual CvDTreeNode* new_node( CvDTreeNode* parent, int count,
                                   int storage_idx, int offset );

    virtual CvDTreeSplit* new_split_ord( int vi, float cmp_val,
                int split_point, int inversed, float quality );
    virtual CvDTreeSplit* new_split_cat( int vi, float quality );
    virtual void free_node_data( CvDTreeNode* node );
    virtual void free_train_data();
    virtual void free_node( CvDTreeNode* node );

    int sample_count, var_all, var_count, max_c_count;
    int ord_var_count, cat_var_count;
    bool have_labels, have_priors;
    bool is_classifier;

    int buf_count, buf_size;
    bool shared;

    CvMat* cat_count;
    CvMat* cat_ofs;
    CvMat* cat_map;

    CvMat* counts;
    CvMat* buf;
    CvMat* direction;
    CvMat* split_buf;

    CvMat* var_idx;
    CvMat* var_type; // i-th element =
                     //   k<0  - ordered
                     //   k>=0 - categorical, see k-th element of cat_* arrays
    CvMat* priors;

    CvDTreeParams params;

    CvMemStorage* tree_storage;
    CvMemStorage* temp_storage;

    CvDTreeNode* data_root;

    CvSet* node_heap;
    CvSet* split_heap;
    CvSet* cv_heap;
    CvSet* nv_heap;

    CvRNG rng;
};
```

This structure is mostly used internally for storing both standalone trees and tree ensembles
efficiently. Basically, it contains 3 types of information:

1. The training parameters, CvDTreeParams instance.- The training data, preprocessed in order to find the best splits more efficiently.
     For tree ensembles this preprocessed data is reused by all the trees.
     Additionally, the training data characteristics that are shared by
     all trees in the ensemble are stored here: variable types,
     the number of classes, class label compression map etc.- Buffers, memory storages for tree nodes, splits and other elements of the trees constructed.

There are 2 ways of using this structure.
In simple cases (e.g. standalone tree,
or ready-to-use "black box" tree ensemble from ML, like Random Trees
or Boosting) there is no need to care or even to know about the structure -
just construct the needed statistical model, train it and use it. The `CvDTreeTrainData`
structure will be constructed and used internally. However, for custom tree algorithms,
or another sophisticated cases, the structure may be constructed and used explicitly.
The scheme is the following:

1. The structure is initialized using the default constructor, followed by
   `set_data` (or it is built using the full form of constructor).
   The parameter `_shared` must be set to `true`.- One or more trees are trained using this data, see the special form of the method
     CvDTree::train.- Finally, the structure can be released only after all the trees using it are released.

---

### CvDTree

Decision tree

```
class CvDTree : public CvStatModel
{
public:
    CvDTree();
    virtual ~CvDTree();

    virtual bool train( const CvMat* _train_data, int _tflag,
                        const CvMat* _responses, const CvMat* _var_idx=0,
                        const CvMat* _sample_idx=0, const CvMat* _var_type=0,
                        const CvMat* _missing_mask=0,
                        CvDTreeParams params=CvDTreeParams() );

    virtual bool train( CvDTreeTrainData* _train_data, const CvMat* _subsample_idx );

    virtual CvDTreeNode* predict( const CvMat* _sample, const CvMat* _missing_data_mask=0,
                                  bool raw_mode=false ) const;
    virtual const CvMat* get_var_importance();
    virtual void clear();

    virtual void read( CvFileStorage* fs, CvFileNode* node );
    virtual void write( CvFileStorage* fs, const char* name );

    // special read & write methods for trees in the tree ensembles
    virtual void read( CvFileStorage* fs, CvFileNode* node,
                       CvDTreeTrainData* data );
    virtual void write( CvFileStorage* fs );

    const CvDTreeNode* get_root() const;
    int get_pruned_tree_idx() const;
    CvDTreeTrainData* get_data();

protected:

    virtual bool do_train( const CvMat* _subsample_idx );

    virtual void try_split_node( CvDTreeNode* n );
    virtual void split_node_data( CvDTreeNode* n );
    virtual CvDTreeSplit* find_best_split( CvDTreeNode* n );
    virtual CvDTreeSplit* find_split_ord_class( CvDTreeNode* n, int vi );
    virtual CvDTreeSplit* find_split_cat_class( CvDTreeNode* n, int vi );
    virtual CvDTreeSplit* find_split_ord_reg( CvDTreeNode* n, int vi );
    virtual CvDTreeSplit* find_split_cat_reg( CvDTreeNode* n, int vi );
    virtual CvDTreeSplit* find_surrogate_split_ord( CvDTreeNode* n, int vi );
    virtual CvDTreeSplit* find_surrogate_split_cat( CvDTreeNode* n, int vi );
    virtual double calc_node_dir( CvDTreeNode* node );
    virtual void complete_node_dir( CvDTreeNode* node );
    virtual void cluster_categories( const int* vectors, int vector_count,
        int var_count, int* sums, int k, int* cluster_labels );

    virtual void calc_node_value( CvDTreeNode* node );

    virtual void prune_cv();
    virtual double update_tree_rnc( int T, int fold );
    virtual int cut_tree( int T, int fold, double min_alpha );
    virtual void free_prune_data(bool cut_tree);
    virtual void free_tree();

    virtual void write_node( CvFileStorage* fs, CvDTreeNode* node );
    virtual void write_split( CvFileStorage* fs, CvDTreeSplit* split );
    virtual CvDTreeNode* read_node( CvFileStorage* fs, CvFileNode* node, CvDTreeNode* parent );
    virtual CvDTreeSplit* read_split( CvFileStorage* fs, CvFileNode* node );
    virtual void write_tree_nodes( CvFileStorage* fs );
    virtual void read_tree_nodes( CvFileStorage* fs, CvFileNode* node );

    CvDTreeNode* root;

    int pruned_tree_idx;
    CvMat* var_importance;

    CvDTreeTrainData* data;
};
```

---

### CvDTree::train

Trains decision tree

```
bool CvDTree::train( const CvMat* _train_data, int _tflag,
                     const CvMat* _responses, const CvMat* _var_idx=0,
                     const CvMat* _sample_idx=0, const CvMat* _var_type=0,
                     const CvMat* _missing_mask=0,
                     CvDTreeParams params=CvDTreeParams() );

bool CvDTree::train( CvDTreeTrainData* _train_data, const CvMat* _subsample_idx );
```

There are 2 `train` methods in `CvDTree`.

The first method follows the generic CvStatModel::train
conventions,  it is the most complete form of it. Both data layouts
(`_tflag=CV_ROW_SAMPLE` and `_tflag=CV_COL_SAMPLE`) are supported,
as well as sample and variable subsets, missing measurements, arbitrary combinations
of input and output variable types etc. The last parameter contains all
the necessary training parameters, see CvDTreeParams description.

The second method `train` is mostly used for building tree ensembles.
It takes the pre-constructed CvDTreeTrainData instance and
the optional subset of training set. The indices in `_subsample_idx` are counted
relatively to the `_sample_idx`, passed to `CvDTreeTrainData` constructor.
For example, if `_sample_idx=[1, 5, 7, 100]`, then
`_subsample_idx=[0,3]` means that the samples `[1, 100]` of the original
training set are used.

---

### CvDTree::predict

Returns the leaf node of decision tree corresponding to the input vector

```
CvDTreeNode* CvDTree::predict( const CvMat* _sample, const CvMat* _missing_data_mask=0,
                               bool raw_mode=false ) const;
```

The method takes the feature vector and the optional missing measurement mask on input,
traverses the decision tree and returns the reached leaf node on output.
The prediction result, either the class label or the estimated function value, may
be retrieved as `value` field of the CvDTreeNode
structure, for example: dtree->predict(sample,mask)->value

The last parameter is normally set to `false` that implies a regular input.
If it is `true`, the method assumes that all the values of the discrete input variables
have been already normalized to `0..<num_of_categoriesi>-1` ranges.
(as the decision tree uses such normalized representation internally). It is useful for faster
prediction with tree ensembles. For ordered input variables the flag is not used.

---

### Example. Building Tree for Classifying Mushrooms

See mushroom.cpp sample that
demonstrates how to build and use the decision tree.


---

## Boosting

A common machine learning task is supervised learning of the following kind:
Predict the output y for an unseen input sample x given a training set
consisting of input and its desired output. In other words, the goal is to learn
the functional relationship F: y = F(x)
between input x and output y.
Predicting qualitative output is called classification, while predicting
quantitative output is called regression.

Boosting is a powerful learning concept, which provide a solution
to supervised classification learning task.
It combines the performance of many "weak" classifiers
to produce a powerful 'committee' [HTF01].
A weak classifier is only required to be better
than chance, and thus can be very simple and computationally inexpensive.
Many of them smartly combined, however, result in a strong classifier,
which often outperforms most 'monolithic' strong classifiers such as SVMs and Neural Networks.

Decision trees are the most popular weak classifiers used in boosting schemes.
Often the simplest decision trees with only a single split node per tree (called stumps)
are sufficient.

Learning of boosted model is based on N training examples
{(x_i_,y_i_)}1N
with x_i_ ∈ R^K^ and
y_i_ ∈ {−1, +1}.
x_i_ is a K-component vector.
Each component encodes a feature relevant for the learning task at hand.
The desired two-class output is encoded as −1 and +1.

Different variants of boosting are known such as Discrete Adaboost, Real AdaBoost, LogitBoost,
and Gentle AdaBoost [FHT98].
All of them are very similar in their overall structure.
Therefore, we will look only at the standard two-class Discrete AdaBoost algorithm
as shown in the box below.
Each sample is initially assigned the same weight (step 2).
Next a weak classifier f_m_(x)
is trained on the weighted training data (step 3a).
Its weighted training error and scaling factor c_m_ is computed (step 3b).
The weights are increased for training samples, which have been misclassified (step 3c).
All weights are then normalized, and the process of finding the next week classifier continues
for another M-1 times. The final classifier F(x) is the sign
of the weighted sum over the individual weak classifiers (step 4).

1. Given N examples
   {(x_i_,y_i_)}1N
   with x_i_ ∈ R^K^,
   y_i_ ∈ {−1, +1}.
2. Start with weights w_i_ = 1/N,
   i = 1,…,N.
3. Repeat for m = 1,2,…,M:
   1. Fit the classifier f_m_(x) ∈ {−1,1},
      using weights w_i_ on the training data.
   2. Compute err_m_ = E_w_
      [1_(y ≠ fm(x))_],
      c_m_ = log((1 − err_m_)/err_m_).
   3. Set w_i_ ← w_i_
      exp[c_m_
      1_(yi ≠ fm(xi))_],
      i = 1,2,…,N,
      and renormalize so that ∑ i w_i_ = 1.
4. Output the classifier sign[∑ m = 1M
   c_m_ f_m_(x)].

Two-class Discrete AdaBoost Algorithm: Training (steps 1 to 3)
and Evaluation (step 4)

**NOTE.** As well as the classical boosting methods, the current implementation
supports 2-class classifiers only. For M>2 classes there is *AdaBoost.MH* algorithm,
described in [FHT98], that reduces the problem to the 2-class problem,
yet with much larger training set.

In order to reduce computation time for boosted models without substantial loosing
of the accuracy, the influence trimming technique may be employed.
As the training algorithm proceeds and the number of trees in the ensemble is increased,
a larger number of the training samples are classified correctly
and with increasing confidence, thereby those samples receive
smaller weights on the subsequent iterations. Examples with very low relative weight have small impact on training of
the weak classifier. Thus such examples may be excluded during the weak classifier training
without having much effect on the induced classifier. This process is controlled
via the weight\_trim\_rate parameter.
Only examples with the summary fraction weight\_trim\_rate
of the total weight mass are used in the weak classifier training.
Note that the weights for *all* training examples are recomputed at each
training iteration. Examples deleted at a particular iteration may
be used again for learning some of the weak classifiers further
[FHT98].

 **[HTF01] Hastie, T., Tibshirani, R., Friedman, J. H.
The Elements of Statistical Learning:
Data Mining, Inference, and Prediction. Springer Series in Statistics. 2001.

[FHT98] Friedman, J. H., Hastie, T. and Tibshirani, R. Additive Logistic
Regression: a Statistical View of Boosting. Technical Report, Dept. of Statistics, Stanford
University, 1998.** 


---

### CvBoostParams

Boosting training parameters

```
struct CvBoostParams : public CvDTreeParams
{
    int boost_type;
    int weak_count;
    int split_criteria;
    double weight_trim_rate;

    CvBoostParams();
    CvBoostParams( int boost_type, int weak_count, double weight_trim_rate,
                   int max_depth, bool use_surrogates, const float* priors );
};
```

boost\_type: Boosting type, one of the following: `CvBoost::DISCRETE` - Discrete AdaBoost `CvBoost::REAL` - Real AdaBoost `CvBoost::LOGIT` - LogitBoost `CvBoost::GENTLE` - Gentle AdaBoost Gentle AdaBoost and Real AdaBoost are often the preferable choices. weak\_count: The number of weak classifiers to build. split\_criteria: Splitting criteria, used to choose optimal splits during a weak tree construction: `CvBoost::DEFAULT` - Use the default criteria for the particular boosting method, see below. `CvBoost::GINI` - Use Gini index. This is default option for Real AdaBoost; may be also used for Discrete AdaBoost. `CvBoost::MISCLASS` - Use misclassification rate. This is default option for Discrete AdaBoost; may be also used for Real AdaBoost. `CvBoost::SQERR` - Use least squares criteria. This is default and the only option for LogitBoost and Gentle AdaBoost. weight\_trim\_rate: The weight trimming ratio, within 0..1. See the discussion of it above. If the parameter is ≤0 or >1, the trimming is not used, all the samples are used at each iteration. The default value is 0.95.

The structure is derived from `CvDTreeParams`,
but not all of the decision tree parameters are supported. In particular, cross-validation
is not supported.

---

### CvBoostTree

Weak tree classifier

```
class CvBoostTree: public CvDTree
{
public:
    CvBoostTree();
    virtual ~CvBoostTree();

    virtual bool train( CvDTreeTrainData* _train_data,
                        const CvMat* subsample_idx, CvBoost* ensemble );
    virtual void scale( double s );
    virtual void read( CvFileStorage* fs, CvFileNode* node,
                       CvBoost* ensemble, CvDTreeTrainData* _data );
    virtual void clear();

protected:
    ...
    CvBoost* ensemble;
};
```

The weak classifier, a component of boosted tree classifier
CvBoost, is a derivative of CvDTree.
Normally, there is no need to use the weak classifiers directly,
however they can be accessed as elements of sequence `CvBoost::weak`,
retrieved by CvBoost::get\_weak\_predictors.

Note, that in case of LogitBoost and Gentle AdaBoost each weak predictor is a regression
tree, rather than a classification tree. Even in case of Discrete AdaBoost and Real AdaBoost
the `CvBoostTree::predict` return value (`CvDTreeNode::value`) is not
the output class label; a negative value "votes" for class #0, a positive - for class #1.
And the votes are weighted. The weight of each individual tree may be increased or decreased using
method `CvBoostTree::scale`.

---

### CvBoost

Boosted tree classifier

```
class CvBoost : public CvStatModel
{
public:
    // Boosting type
    enum { DISCRETE=0, REAL=1, LOGIT=2, GENTLE=3 };

    // Splitting criteria
    enum { DEFAULT=0, GINI=1, MISCLASS=3, SQERR=4 };

    CvBoost();
    virtual ~CvBoost();

    CvBoost( const CvMat* _train_data, int _tflag,
             const CvMat* _responses, const CvMat* _var_idx=0,
             const CvMat* _sample_idx=0, const CvMat* _var_type=0,
             const CvMat* _missing_mask=0,
             CvBoostParams params=CvBoostParams() );

    virtual bool train( const CvMat* _train_data, int _tflag,
             const CvMat* _responses, const CvMat* _var_idx=0,
             const CvMat* _sample_idx=0, const CvMat* _var_type=0,
             const CvMat* _missing_mask=0,
             CvBoostParams params=CvBoostParams(),
             bool update=false );

    virtual float predict( const CvMat* _sample, const CvMat* _missing=0,
                           CvMat* weak_responses=0, CvSlice slice=CV_WHOLE_SEQ,
                           bool raw_mode=false ) const;

    virtual void prune( CvSlice slice );

    virtual void clear();

    virtual void write( CvFileStorage* storage, const char* name );
    virtual void read( CvFileStorage* storage, CvFileNode* node );

    CvSeq* get_weak_predictors();
    const CvBoostParams& get_params() const;
    ...

protected:
    virtual bool set_params( const CvBoostParams& _params );
    virtual void update_weights( CvBoostTree* tree );
    virtual void trim_weights();
    virtual void write_params( CvFileStorage* fs );
    virtual void read_params( CvFileStorage* fs, CvFileNode* node );

    CvDTreeTrainData* data;
    CvBoostParams params;
    CvSeq* weak;
    ...
};
```

---

### CvBoost::train

Trains boosted tree classifier

```
bool CvBoost::train( const CvMat* _train_data, int _tflag,
             const CvMat* _responses, const CvMat* _var_idx=0,
             const CvMat* _sample_idx=0, const CvMat* _var_type=0,
             const CvMat* _missing_mask=0,
             CvBoostParams params=CvBoostParams(),
             bool update=false );
```

The train method follows the common template, the last parameter `update`
specifies whether the classifier needs to be updated
(i.e. the new weak tree classifiers added to the existing ensemble), or the classifier
needs to be rebuilt from scratch.
The responses must be categorical, i.e. boosted trees can not be built for regression,
and there should be 2 classes.

---

### CvBoost::predict

Predicts response for the input sample

```
float CvBoost::predict( const CvMat* sample, const CvMat* missing=0,
                        CvMat* weak_responses=0, CvSlice slice=CV_WHOLE_SEQ,
                        bool raw_mode=false ) const;
```

sample: The input sample. missing: The optional mask of missing measurements. To handle missing measurements, the weak classifiers must include surrogate splits (see `CvDTreeParams::use_surrogates`). weak\_responses: The optional output parameter, a floating-point vector, of responses from each individual weak classifier. The number of elements in the vector must be equal to the `slice` length. slice: The continuous subset of the sequence of weak classifiers to be used for prediction. By default, all the weak classifiers are used. raw\_mode: It has the same meaning as in `CvDTree::predict`. Normally, it should be set to false.

The method `CvBoost::predict` runs the sample through the trees
in the ensemble and returns the output class label based on the weighted voting.

---

### CvBoost::prune

Removes specified weak classifiers

```
void CvBoost::prune( CvSlice slice );
```

The method removes the specified weak classifiers from the sequence. Note that
this method should not be confused with the pruning of individual decision trees, which is currently
not supported.

---

### CvBoost::get\_weak\_predictors

Returns the sequence of weak tree classifiers

```
CvSeq* CvBoost::get_weak_predictors();
```

The method returns the sequence of weak classifiers.
Each element of the sequence is a pointer to `CvBoostTree` class
(or, probably, to some of its derivatives).


---

## Random Trees

Random trees have been introduced by Leo Breiman and
Adele Cutler: 
http://www.stat.berkeley.edu/users/breiman/RandomForests/.
The algorithm can deal with both classification and regression problems.
Random trees is a collection (ensemble) of tree
predictors that is called **forest** further in this section
(the term has been also introduced by L. Breiman).
The classification works as following:
the random trees classifier takes the input feature vector, classifies
it with every tree in the forest, and outputs the class label that
has got the majority of "votes". In case of regression the classifier response
is the average of responses over all the trees in the forest.

All the trees are trained with the same parameters, but on the different training sets, which
are generated from the original training set using bootstrap procedure:
for each training set we randomly select the same number of vectors
as in the original set (`=N`). The vectors are chosen with replacement.
That is, some vectors will occur more than once and some will be absent.
At each node of each tree trained not all the variables are used to find the best split,
rather than a random subset of them. The each node a new subset is generated, however its
size is fixed for all the nodes and all the trees. It is a training parameter,
set to `sqrt(<number_of_variables>)` by default.
None of the tree built is pruned.

In random trees there is no need in any accuracy estimation procedures, such
as cross-validation or bootstrap, or a separate test set to get an estimate of the
training error. The error is estimated internally during the training.
When the training set for the current tree is drawn by sampling with replacement,
some vectors are left out (so-called *oob (out-of-bag) data*).
The size of oob data is about `N/3`. The classification error
is estimated by using this oob-data as following:

- Get a prediction for each vector, which is oob relatively to the i-th tree,
  using the very i-th tree.- After all the trees have been trained, for each vector that has ever been oob,
    find the class-"winner" for it (i.e. the class that has got the majority of votes
    in the trees, where the vector was oob) and compare it to the ground-truth response.- Then the classification error estimate is computed as ratio of
      number of misclassified oob vectors to all the vectors in the original data.
      In the case of regression the oob-error is computed as
      the squared error for oob vectors difference divided by the total number of vectors.

**References:  

1. Machine Learning, Wald I, July 2002
2. Looking Inside the Black Box, Wald II, July 2002
3. Software for the Masses, Wald III, July 2002
4. And other articles from the web-site
   http://www.stat.berkeley.edu/users/breiman/RandomForests/cc\_home.htm.**

---

### CvRTParams

Training Parameters of Random Trees

```
struct CvRTParams : public CvDTreeParams
{
    bool calc_var_importance;
    int nactive_vars;
    CvTermCriteria term_crit;

    CvRTParams() : CvDTreeParams( 5, 10, 0, false, 10, 0, false, false, 0 ),
        calc_var_importance(false), nactive_vars(0)
    {
        term_crit = cvTermCriteria( CV_TERMCRIT_ITER+CV_TERMCRIT_EPS, 50, 0.1 );
    }

    CvRTParams( int _max_depth, int _min_sample_count,
                float _regression_accuracy, bool _use_surrogates,
                int _max_categories, const float* _priors,
                bool _calc_var_importance,
                int _nactive_vars, int max_tree_count,
                float forest_accuracy, int termcrit_type );
};
```

calc\_var\_importance: If it is set, then variable importance is computed by the training procedure. To retrieve the computed variable importance array, call the method `CvRTrees::get_var_importance()`. nactive\_vars: The number of variables that are randomly selected at each tree node and that are used to find the best split(s). term\_crit: Termination criteria for growing the forest: `term_crit.max_iter` is the maximum number of trees in the forest (see also `max_tree_count` parameter of the constructor, by default it is set to 50) `term_crit.epsilon` is the sufficient accuracy (OOB error).

The set of training parameters for the forest is the superset of the training parameters for
a single tree. However, Random trees do not need all the functionality/features of decision trees,
most noticeably, the trees are not pruned, so the cross-validation parameters are not used.

---

### CvRTrees

Random Trees

```
class CvRTrees : public CvStatModel
{
public:
    CvRTrees();
    virtual ~CvRTrees();
    virtual bool train( const CvMat* _train_data, int _tflag,
                        const CvMat* _responses, const CvMat* _var_idx=0,
                        const CvMat* _sample_idx=0, const CvMat* _var_type=0,
                        const CvMat* _missing_mask=0,
                        CvRTParams params=CvRTParams() );
    virtual float predict( const CvMat* sample, const CvMat* missing = 0 ) const;
    virtual void clear();

    virtual const CvMat* get_var_importance();
    virtual float get_proximity( const CvMat* sample_1, const CvMat* sample_2 ) const;

    virtual void read( CvFileStorage* fs, CvFileNode* node );
    virtual void write( CvFileStorage* fs, const char* name );

    CvMat* get_active_var_mask();
    CvRNG* get_rng();

    int get_tree_count() const;
    CvForestTree* get_tree(int i) const;

protected:

    bool grow_forest( const CvTermCriteria term_crit );

    // array of the trees of the forest
    CvForestTree** trees;
    CvDTreeTrainData* data;
    int ntrees;
    int nclasses;
    ...
};
```

---

### CvRTrees::train

Trains Random Trees model

```
bool CvRTrees::train( const CvMat* train_data, int tflag,
                    const CvMat* responses, const CvMat* comp_idx=0,
                    const CvMat* sample_idx=0, const CvMat* var_type=0,
                    const CvMat* missing_mask=0,
                    CvRTParams params=CvRTParams() );
```

The method `CvRTrees::train` is very similar to the first form
of CvDTree::train() and follows
the generic method CvStatModel::train conventions.
All the specific to the algorithm training parameters are passed as
CvRTParams instance.
The estimate of the training error (oob-error)
is stored in the protected class member `oob_error`.

---

### CvRTrees::predict

Predicts the output for the input sample

```
double CvRTrees::predict( const CvMat* sample, const CvMat* missing=0 ) const;
```

The input parameters of the prediction method are the same as in
CvDTree::predict, but the return value type is different.
This method returns the cumulative result from all the trees in the forest
(the class that receives the majority of voices, or the mean of the regression function estimates).

---

### CvRTrees::get\_var\_importance

Retrieves the variable importance array

```
const CvMat* CvRTrees::get_var_importance() const;
```

The method returns the variable importance vector, computed at the training stage
when `CvRTParams::calc_var_importance`
is set. If the training flag is not set, then the `NULL` pointer is returned.
This is unlike decision trees, where variable importance can be computed anytime
after the training.

---

### CvRTrees::get\_proximity

Retrieves proximity measure between two training samples

```
float CvRTrees::get_proximity( const CvMat* sample_1, const CvMat* sample_2 ) const;
```

The method returns proximity measure between any two samples
(the ratio of the those trees in the ensemble, in which the samples fall into
the same leaf node, to the total number of the trees).

---

#### Example. Prediction of mushroom goodness using random trees classifier

```
#include <float.h>
#include <stdio.h>
#include <ctype.h>
#include "ml.h"

int main( void )
{
    CvStatModel*    cls = NULL;
    CvFileStorage*  storage = cvOpenFileStorage( "Mushroom.xml", NULL,CV_STORAGE_READ );
    CvMat*          data = (CvMat*)cvReadByName(storage, NULL, "sample", 0 );
    CvMat           train_data, test_data;
    CvMat           response;
    CvMat*          missed = NULL;
    CvMat*          comp_idx = NULL;
    CvMat*          sample_idx = NULL;
    CvMat*          type_mask = NULL;
    int             resp_col = 0;
    int             i,j;
    CvRTreesParams  params;
    CvTreeClassifierTrainParams cart_params;
    const int       ntrain_samples = 1000;
    const int       ntest_samples  = 1000;
    const int       nvars = 23;

    if(data == NULL || data->cols != nvars)
    {
        puts("Error in source data");
        return -1;
    }

    cvGetSubRect( data, &train_data, cvRect(0, 0, nvars, ntrain_samples) );
    cvGetSubRect( data, &test_data, cvRect(0, ntrain_samples, nvars,
        ntrain_samples + ntest_samples) );

    resp_col = 0;
    cvGetCol( &train_data, &response, resp_col);

    /* create missed variable matrix */
    missed = cvCreateMat(train_data.rows, train_data.cols, CV_8UC1);
    for( i = 0; i < train_data.rows; i++ )
        for( j = 0; j < train_data.cols; j++ )
            CV_MAT_ELEM(*missed,uchar,i,j) = (uchar)(CV_MAT_ELEM(train_data,float,i,j) < 0);

    /* create comp_idx vector */
    comp_idx = cvCreateMat(1, train_data.cols-1, CV_32SC1);
    for( i = 0; i < train_data.cols; i++ )
    {
        if(i<resp_col)CV_MAT_ELEM(*comp_idx,int,0,i) = i;
        if(i>resp_col)CV_MAT_ELEM(*comp_idx,int,0,i-1) = i;
    }

    /* create sample_idx vector */
    sample_idx = cvCreateMat(1, train_data.rows, CV_32SC1);
    for( j = i = 0; i < train_data.rows; i++ )
    {
        if(CV_MAT_ELEM(response,float,i,0) < 0) continue;
        CV_MAT_ELEM(*sample_idx,int,0,j) = i;
        j++;
    }
    sample_idx->cols = j;

    /* create type mask */
    type_mask = cvCreateMat(1, train_data.cols+1, CV_8UC1);
    cvSet( type_mask, cvRealScalar(CV_VAR_CATEGORICAL), 0);

    // initialize training parameters
    cvSetDefaultParamTreeClassifier((CvStatModelParams*)&cart_params);
    cart_params.wrong_feature_as_unknown = 1;
    params.tree_params = &cart_params;
    params.term_crit.max_iter = 50;
    params.term_crit.epsilon = 0.1;
    params.term_crit.type = CV_TERMCRIT_ITER|CV_TERMCRIT_EPS;

    puts("Random forest results");
    cls = cvCreateRTreesClassifier( &train_data, CV_ROW_SAMPLE, &response,
        (CvStatModelParams*)& params, comp_idx, sample_idx, type_mask, missed );
    if( cls )
    {
        CvMat sample = cvMat( 1, nvars, CV_32FC1, test_data.data.fl );
        CvMat test_resp;
        int wrong = 0, total = 0;
        cvGetCol( &test_data, &test_resp, resp_col);
        for( i = 0; i < ntest_samples; i++, sample.data.fl += nvars )
        {
            if( CV_MAT_ELEM(test_resp,float,i,0) >= 0 )
            {
                float resp = cls->predict( cls, &sample, NULL );
                wrong += (fabs(resp-response.data.fl[i]) > 1e-3 ) ? 1 : 0;
                total++;
            }
        }
        printf( "Test set error = %.2f\n", wrong*100.f/(float)total );
    }
    else
       puts("Error forest creation");


    cvReleaseMat(&missed);
    cvReleaseMat(&sample_idx);
    cvReleaseMat(∁_idx);
    cvReleaseMat(&type_mask);
    cvReleaseMat(&data);
    cvReleaseStatModel(&cls);
    cvReleaseFileStorage(&storage);
    return 0;
}
```


---

## Expectation-Maximization

The EM (Expectation-Maximization) algorithm estimates the parameters of the
multivariate probability density function in a form of the Gaussian mixture
distribution with a specified number of mixtures.

Consider the set of the feature vectors {x_1_, x_2_,..., x_N_}:
`N` vectors from `d`-dimensional Euclidean space drawn from a Gaussian mixture:

where `m` is the number of mixtures, p_k_
is the normal distribution density with the mean `ak`
and covariance matrix `Sk`, `πk` is the weight of k-th mixture.
Given the number of mixtures `m` and the samples `{xi, i=1..N}`
the algorithm finds the maximum-likelihood estimates (MLE)
of the all the mixture parameters, i.e. `ak`, `Sk` and
`πk`:

EM algorithm is an iterative procedure. Each iteration of it includes two steps.
At the first step (Expectation-step, or E-step), we find a probability `pi,k`
(denoted `αi,k` in the formula below)
of sample `#i` to belong to mixture `#k`
using the currently available mixture parameter estimates:

At the second step (Maximization-step, or M-step) the mixture parameter estimates
are refined using the computed probabilities:

Alternatively, the algorithm may start with M-step when initial values for `pi,k`
can be provided. Another alternative, when `pi,k` are unknown,
is to use a simpler clustering algorithm to pre-cluster the input samples and thus obtain
initial `pi,k`. Often (and in ML)
k-means algorithm is used for that purpose.

One of the main that EM algorithm should deal with is the large number of parameters to estimate.
The majority of the parameters sits in covariation matrices, which are `d×d` elements each
(where `d` is the feature space dimensionality). However, in many practical problems
the covariation matrices are close to diagonal, or even to `μk*I`,
where `I` is identity matrix and `μk` is mixture-dependent "scale" parameter.
So a robust computation scheme could be to start with the harder constraints on
the covariation matrices and then use the estimated parameters as an input for
a less constrained optimization problem (often a diagonal covariation matrix is already a good enough
approximation).

**References:**  

1. **[Bilmes98] J. A. Bilmes. A Gentle Tutorial of the EM Algorithm and its Application to Parameter
   Estimation for Gaussian Mixture and Hidden Markov Models.
   Technical Report TR-97-021,
   International Computer Science Institute and Computer Science Division,
   University of California at Berkeley, April 1998.**

---

### CvEMParams

Parameters of EM algorithm

```
struct CvEMParams
{
    CvEMParams() : nclusters(10), cov_mat_type(CvEM::COV_MAT_DIAGONAL),
        start_step(CvEM::START_AUTO_STEP), probs(0), weights(0), means(0), covs(0)
    {
        term_crit=cvTermCriteria( CV_TERMCRIT_ITER+CV_TERMCRIT_EPS, 100, FLT_EPSILON );
    }

    CvEMParams( int _nclusters, int _cov_mat_type=1/*CvEM::COV_MAT_DIAGONAL*/,
                int _start_step=0/*CvEM::START_AUTO_STEP*/,
                CvTermCriteria _term_crit=cvTermCriteria(CV_TERMCRIT_ITER+CV_TERMCRIT_EPS, 100, FLT_EPSILON),
                CvMat* _probs=0, CvMat* _weights=0, CvMat* _means=0, CvMat** _covs=0 ) :
                nclusters(_nclusters), cov_mat_type(_cov_mat_type), start_step(_start_step),
                probs(_probs), weights(_weights), means(_means), covs(_covs), term_crit(_term_crit)
    {}

    int nclusters;
    int cov_mat_type;
    int start_step;
    const CvMat* probs;
    const CvMat* weights;
    const CvMat* means;
    const CvMat** covs;
    CvTermCriteria term_crit;
};
```

nclusters: The number of mixtures. Some of EM implementation could determine the optimal number of mixtures within a specified value range, but that is not the case in ML yet. cov\_mat\_type: The type of the mixture covariation matrices; should be one of the following: `CvEM::COV_MAT_GENERIC` - a covariation matrix of each mixture may be arbitrary symmetrical positively defined matrix, so the number of free parameters in each matrix is about `d`^2^/2. It is not recommended to use this option, unless there is pretty accurate initial estimation of the parameters and/or a huge number of training samples. `CvEM::COV_MAT_DIAGONAL` - a covariation matrix of each mixture may be arbitrary diagonal matrix with positive diagonal elements, that is, non-diagonal elements are forced to be 0's, so the number of free parameters is `d` for each matrix. This is most commonly used option yielding good estimation results. `CvEM::COV_MAT_SPHERICAL` - a covariation matrix of each mixture is a scaled identity matrix, `μk*I`, so the only parameter to be estimated is `μk`. The option may be used in special cases, when the constraint is relevant, or as a first step in the optimization (e.g. in case when the data is preprocessed with PCA). The results of such preliminary estimation may be passed again to the optimization procedure, this time with `cov_mat_type=CvEM::COV_MAT_DIAGONAL`. start\_step: The initial step the algorithm starts from; should be one of the following: `CvEM::START_E_STEP` - the algorithm starts with E-step. At least, the initial values of mean vectors, `CvEMParams::means` must be passed. Optionally, the user may also provide initial values for weights (`CvEMParams::weights`) and/or covariation matrices (`CvEMParams::covs`). `CvEM::START_M_STEP` - the algorithm starts with M-step. The initial probabilities `pi,k` must be provided. `CvEM::START_AUTO_STEP` - No values are required from the user, k-means algorithm is used to estimate initial mixtures parameters. term\_crit: Termination criteria of the procedure. EM algorithm stops either after a certain number of iterations (`term_crit.num_iter`), or when the parameters change too little (no more than `term_crit.epsilon`) from iteration to iteration. probs: Initial probabilities `pi,k`; are used (and must be not NULL) only when `start_step=CvEM::START_M_STEP`. weights: Initial mixture weights `πk`; are used (if not NULL) only when `start_step=CvEM::START_E_STEP`. covs: Initial mixture covariation matrices `Sk`; are used (if not NULL) only when `start_step=CvEM::START_E_STEP`. means: Initial mixture means `ak`; are used (and must be not NULL) only when `start_step=CvEM::START_E_STEP`.

The structure has 2 constructors, the default one represents a rough rule-of-thumb,
with another one it is possible to override a variety of parameters,
from a single number of mixtures (the only essential problem-dependent parameter),
to the initial values for the mixture parameters.

---

### CvEM

EM model

```
class CV_EXPORTS CvEM : public CvStatModel
{
public:
    // Type of covariation matrices
    enum { COV_MAT_SPHERICAL=0, COV_MAT_DIAGONAL=1, COV_MAT_GENERIC=2 };

    // The initial step
    enum { START_E_STEP=1, START_M_STEP=2, START_AUTO_STEP=0 };

    CvEM();
    CvEM( const CvMat* samples, const CvMat* sample_idx=0,
          CvEMParams params=CvEMParams(), CvMat* labels=0 );
    virtual ~CvEM();

    virtual bool train( const CvMat* samples, const CvMat* sample_idx=0,
                        CvEMParams params=CvEMParams(), CvMat* labels=0 );

    virtual float predict( const CvMat* sample, CvMat* probs ) const;
    virtual void clear();

    int get_nclusters() const { return params.nclusters; }
    const CvMat* get_means() const { return means; }
    const CvMat** get_covs() const { return covs; }
    const CvMat* get_weights() const { return weights; }
    const CvMat* get_probs() const { return probs; }

protected:

    virtual void set_params( const CvEMParams& params,
                             const CvVectors& train_data );
    virtual void init_em( const CvVectors& train_data );
    virtual double run_em( const CvVectors& train_data );
    virtual void init_auto( const CvVectors& samples );
    virtual void kmeans( const CvVectors& train_data, int nclusters,
                         CvMat* labels, CvTermCriteria criteria,
                         const CvMat* means );
    CvEMParams params;
    double log_likelihood;

    CvMat* means;
    CvMat** covs;
    CvMat* weights;
    CvMat* probs;

    CvMat* log_weight_div_det;
    CvMat* inv_eigen_values;
    CvMat** cov_rotate_mats;
};
```

---

### CvEM::train

Estimates Gaussian mixture parameters from the sample set

```
void CvEM::train( const CvMat* samples, const CvMat*  sample_idx=0,
                  CvEMParams params=CvEMParams(), CvMat* labels=0 );
```

Unlike many of ML models, EM is an unsupervised learning algorithm and it does not
take responses (class labels or the function values) on input. Instead, it computes
MLE of Gaussian mixture parameters from the input sample set,
stores all the parameters inside the structure: `pi,k` in `probs`,
`ak` in `means` `Sk` in `covs[k]`,
`πk` in `weights` and
optionally computes the output "class label" for each sample:
`labelsi=arg maxk(pi,k), i=1..N`
(i.e. indices of the most-probable mixture for each sample).

The trained model can be used further for prediction, just like any other classifier.
The model trained is similar to the normal Bayes classifier.

#### Example. Clustering random samples of multi-Gaussian distribution using EM

```
#include "ml.h"
#include "highgui.h"

int main( int argc, char** argv )
{
    const int N = 4;
    const int N1 = (int)sqrt((double)N);
    const CvScalar colors[] = {{{0,0,255}},{{0,255,0}},{{0,255,255}},{{255,255,0}}};
    int i, j;
    int nsamples = 100;
    CvRNG rng_state = cvRNG(-1);
    CvMat* samples = cvCreateMat( nsamples, 2, CV_32FC1 );
    CvMat* labels = cvCreateMat( nsamples, 1, CV_32SC1 );
    IplImage* img = cvCreateImage( cvSize( 500, 500 ), 8, 3 );
    float _sample[2];
    CvMat sample = cvMat( 1, 2, CV_32FC1, _sample );
    CvEM em_model;
    CvEMParams params;
    CvMat samples_part;

    cvReshape( samples, samples, 2, 0 );
    for( i = 0; i < N; i++ )
    {
        CvScalar mean, sigma;

        // form the training samples
        cvGetRows( samples, &samples_part, i*nsamples/N, (i+1)*nsamples/N );
        mean = cvScalar(((i%N1)+1.)*img->width/(N1+1), ((i/N1)+1.)*img->height/(N1+1));
        sigma = cvScalar(30,30);
        cvRandArr( &rng_state, &samples_part, CV_RAND_NORMAL, mean, sigma );
    }
    cvReshape( samples, samples, 1, 0 );

    // initialize model's parameters
    params.covs      = NULL;
    params.means     = NULL;
    params.weights   = NULL;
    params.probs     = NULL;
    params.nclusters = N;
    params.cov_mat_type       = CvEM::COV_MAT_SPHERICAL;
    params.start_step         = CvEM::START_AUTO_STEP;
    params.term_crit.max_iter = 10;
    params.term_crit.epsilon  = 0.1;
    params.term_crit.type     = CV_TERMCRIT_ITER|CV_TERMCRIT_EPS;

    // cluster the data
    em_model.train( samples, 0, params, labels );

#if 0
    // the piece of code shows how to repeatedly optimize the model
    // with less-constrained parameters (COV_MAT_DIAGONAL instead of COV_MAT_SPHERICAL)
    // when the output of the first stage is used as input for the second.
    CvEM em_model2;
    params.cov_mat_type = CvEM::COV_MAT_DIAGONAL;
    params.start_step = CvEM::START_E_STEP;
    params.means = em_model.get_means();
    params.covs = (const CvMat**)em_model.get_covs();
    params.weights = em_model.get_weights();

    em_model2.train( samples, 0, params, labels );
    // to use em_model2, replace em_model.predict() with em_model2.predict() below
#endif
    // classify every image pixel
    cvZero( img );
    for( i = 0; i < img->height; i++ )
    {
        for( j = 0; j < img->width; j++ )
        {
            CvPoint pt = cvPoint(j, i);
            sample.data.fl[0] = (float)j;
            sample.data.fl[1] = (float)i;
            int response = cvRound(em_model.predict( &sample, NULL ));
            CvScalar c = colors[response];

            cvCircle( img, pt, 1, cvScalar(c.val[0]*0.75,c.val[1]*0.75,c.val[2]*0.75), CV_FILLED );
        }
    }

    //draw the clustered samples
    for( i = 0; i < nsamples; i++ )
    {
        CvPoint pt;
        pt.x = cvRound(samples->data.fl[i*2]);
        pt.y = cvRound(samples->data.fl[i*2+1]);
        cvCircle( img, pt, 1, colors[labels->data.i[i]], CV_FILLED );
    }

    cvNamedWindow( "EM-clustering result", 1 );
    cvShowImage( "EM-clustering result", img );
    cvWaitKey(0);

    cvReleaseMat( &samples );
    cvReleaseMat( &labels );
    return 0;
}
```


---

## Neural Networks

ML implements feed-forward artificial neural networks, more particularly, multi-layer
perceptrons (MLP), the most commonly used type of neural networks.
MLP consists of the input layer, output layer and one or more
hidden layers. Each layer of MLP includes one or more neurons that are
directionally linked with the neurons from the previous and the next layer.
Here is an example of 3-layer perceptron with 3 inputs, 2 outputs and the hidden layer
including 5 neurons:

All the neurons in MLP are similar. Each of them has several input links (i.e.
it takes the output values from several neurons in the previous layer on input) and
several output links (i.e. it passes the response to several neurons in the next layer).
The values retrieved from the previous layer are summed with certain weights, individual for each
neuron, plus the bias term, and the sum is transformed using the activation function `f` that may be also
different for different neurons. Here is the picture:

In other words, given the outputs `{xj}` of the layer `n`,
the outputs `{yi}` of the layer `n+1` are computed as:

```
    ui=sumj(w(n+1)i,j*xj) + w(n+1)i,bias
    yi=f(ui)
```

Different activation functions may be used, the ML implements 3 standard ones:

- Identity function (`CvANN_MLP::IDENTITY`): `f(x)=x`- Symmetrical sigmoid (`CvANN_MLP::SIGMOID_SYM`):
    `f(x)=β*(1-e-αx)/(1+e-αx)`,
    the default choice for MLP; the standard sigmoid with β=1, α=1 is shown below:- Gaussian function (`CvANN_MLP::GAUSSIAN`):
      `f(x)=βe-αx*x`,
      not completely supported by the moment.

In ML all the neurons have the same activation functions, with the same free parameters
(α, β) that are specified by user and are not altered by the training algorithms.

So the whole trained network works as following. It takes the feature vector on input, the vector size
is equal to the size of the input layer, when the values are passed as input to the first hidden layer,
the outputs of the hidden layer are computed using the weights and the activation functions and passed
further downstream, until we compute the output layer.

So, in order to compute the network one need to know all the weights `w(n+1)i,j`.
The weights are computed by the training algorithm. The algorithm takes a training set: multiple
input vectors with the corresponding output vectors, and iteratively adjusts the weights to try to make
the network give the desired response on the provided input vectors.

The larger the network size (the number of hidden layers and their sizes), the more is the potential
network flexibility, and the error on the training set could be made arbitrarily small. But at the same
time the learned network will also "learn" the noise present in the training set, so the error
on the test set usually starts increasing after the network size reaches some limit.
Besides, the larger networks are train much longer than the smaller ones, so it is reasonable to preprocess
the data (using PCA or similar technique) and
train a smaller network on only the essential features.

Another feature of the MLP's is their inability to handle categorical data as is, however
there is a workaround. If a certain
feature in the input or output (i.e. in case of `n`-class classifier for `n>2`)
layer is categorical and can take `M` (>2) different values,
it makes sense to represent it as binary tuple of `M` elements, where `i`-th
element is `1` if and only if the feature is equal to the `i`-th value out of
`M` possible. It will increase the size of the input/output layer, but will speedup the
training algorithm convergence and at the same time enable "fuzzy" values of such variables, i.e.
a tuple of probabilities instead of a fixed value.

ML implements 2 algorithms for training MLP's. The first is the classical random sequential
back-propagation algorithm and the second (default one) is
batch RPROP algorithm

**References:  

1. http://en.wikipedia.org/wiki/Backpropagation.
   Wikipedia article about the back-propagation algorithm.- Y. LeCun, L. Bottou, G.B. Orr and K.-R. Muller, "Efficient backprop", in Neural Networks---Tricks of the Trade, Springer Lecture Notes in Computer Sciences 1524, pp.5-50, 1998.- M. Riedmiller and H. Braun, "A Direct Adaptive Method for Faster Backpropagation Learning: The RPROP Algorithm", Proc. ICNN, San Francisco (1993).**

---

### CvANN\_MLP\_TrainParams

Parameters of MLP training algorithm

```
struct CvANN_MLP_TrainParams
{
    CvANN_MLP_TrainParams();
    CvANN_MLP_TrainParams( CvTermCriteria term_crit, int train_method,
                           double param1, double param2=0 );
    ~CvANN_MLP_TrainParams();

    enum { BACKPROP=0, RPROP=1 };

    CvTermCriteria term_crit;
    int train_method;

    // backpropagation parameters
    double bp_dw_scale, bp_moment_scale;

    // rprop parameters
    double rp_dw0, rp_dw_plus, rp_dw_minus, rp_dw_min, rp_dw_max;
};
```

term\_crit: The termination criteria for the training algorithm. It identifies how many iterations is done by the algorithm (for sequential backpropagation algorithm the number is multiplied by the size of the training set) and how much the weights could change between the iterations to make the algorithm continue. train\_method: The training algorithm to use; can be one of `CvANN_MLP_TrainParams::BACKPROP` (sequential backpropagation algorithm) or `CvANN_MLP_TrainParams::RPROP` (RPROP algorithm, default value). bp\_dw\_scale: (Backpropagation only): The coefficient to multiply the computed weight gradient by. The recommended value is about `0.1`. The parameter can be set via `param1` of the constructor. bp\_moment\_scale: (Backpropagation only): The coefficient to multiply the difference between weights on the 2 previous iterations. This parameter provides some inertia to smooth the random fluctuations of the weights. It can vary from `0` (the feature is disabled) to `1` and beyond. The value `0.1` or so is good enough. The parameter can be set via `param2` of the constructor. rp\_dw0: (RPROP only): Initial magnitude of the weight delta. The default value is `0.1`. This parameter can be set via `param1` of the constructor. rp\_dw\_plus: (RPROP only): The increase factor for the weight delta. It must be >1, default value is `1.2` that should work well in most cases, according to the algorithm's author. The parameter can only be changed explicitly by modifying the structure member. rp\_dw\_minus: (RPROP only): The decrease factor for the weight delta. It must be <1, default value is `0.5` that should work well in most cases, according to the algorithm's author. The parameter can only be changed explicitly by modifying the structure member. rp\_dw\_min: (RPROP only): The minimum value of the weight delta. It must be >0, the default value is `FLT_EPSILON`. The parameter can be set via `param2` of the constructor. rp\_dw\_max: (RPROP only): The maximum value of the weight delta. It must be >1, the default value is `50`. The parameter can only be changed explicitly by modifying the structure member.

The structure has default constructor that initializes parameters for `RPROP` algorithm.
There is also more advanced constructor to customize the parameters and/or choose backpropagation algorithm.
Finally, the individual parameters can be adjusted after the structure is created.

---

### CvANN\_MLP

MLP model

```
class CvANN_MLP : public CvStatModel
{
public:
    CvANN_MLP();
    CvANN_MLP( const CvMat* _layer_sizes,
               int _activ_func=SIGMOID_SYM,
               double _f_param1=0, double _f_param2=0 );

    virtual ~CvANN_MLP();

    virtual void create( const CvMat* _layer_sizes,
                         int _activ_func=SIGMOID_SYM,
                         double _f_param1=0, double _f_param2=0 );

    virtual int train( const CvMat* _inputs, const CvMat* _outputs,
                       const CvMat* _sample_weights, const CvMat* _sample_idx=0,
                       CvANN_MLP_TrainParams _params = CvANN_MLP_TrainParams(),
                       int flags=0 );
    virtual float predict( const CvMat* _inputs,
                           CvMat* _outputs ) const;

    virtual void clear();

    // possible activation functions
    enum { IDENTITY = 0, SIGMOID_SYM = 1, GAUSSIAN = 2 };

    // available training flags
    enum { UPDATE_WEIGHTS = 1, NO_INPUT_SCALE = 2, NO_OUTPUT_SCALE = 4 };

    virtual void read( CvFileStorage* fs, CvFileNode* node );
    virtual void write( CvFileStorage* storage, const char* name );

    int get_layer_count() { return layer_sizes ? layer_sizes->cols : 0; }
    const CvMat* get_layer_sizes() { return layer_sizes; }

protected:

    virtual bool prepare_to_train( const CvMat* _inputs, const CvMat* _outputs,
            const CvMat* _sample_weights, const CvMat* _sample_idx,
            CvANN_MLP_TrainParams _params,
            CvVectors* _ivecs, CvVectors* _ovecs, double** _sw, int _flags );

    // sequential random backpropagation
    virtual int train_backprop( CvVectors _ivecs, CvVectors _ovecs, const double* _sw );

    // RPROP algorithm
    virtual int train_rprop( CvVectors _ivecs, CvVectors _ovecs, const double* _sw );

    virtual void calc_activ_func( CvMat* xf, const double* bias ) const;
    virtual void calc_activ_func_deriv( CvMat* xf, CvMat* deriv, const double* bias ) const;
    virtual void set_activ_func( int _activ_func=SIGMOID_SYM,
                                 double _f_param1=0, double _f_param2=0 );
    virtual void init_weights();
    virtual void scale_input( const CvMat* _src, CvMat* _dst ) const;
    virtual void scale_output( const CvMat* _src, CvMat* _dst ) const;
    virtual void calc_input_scale( const CvVectors* vecs, int flags );
    virtual void calc_output_scale( const CvVectors* vecs, int flags );

    virtual void write_params( CvFileStorage* fs );
    virtual void read_params( CvFileStorage* fs, CvFileNode* node );

    CvMat* layer_sizes;
    CvMat* wbuf;
    CvMat* sample_weights;
    double** weights;
    double f_param1, f_param2;
    double min_val, max_val, min_val1, max_val1;
    int activ_func;
    int max_count, max_buf_sz;
    CvANN_MLP_TrainParams params;
    CvRNG rng;
};
```

Unlike many other models in ML that are constructed and trained at once,
in the MLP model these steps are separated. First, a network with the specified topology
is created using the non-default constructor or the method create.
All the weights are set to zeros.
Then the network is trained using the set of input and output vectors.
The training procedure can be repeated more than once, i.e. the weights can be adjusted
based on the new training data.

---

### CvANN\_MLP::create

Constructs the MLP with the specified topology

```
void CvANN_MLP::create( const CvMat* _layer_sizes,
                        int _activ_func=SIGMOID_SYM,
                        double _f_param1=0, double _f_param2=0 );
```

\_layer\_sizes: The integer vector specifies the number of neurons in each layer including the input and output layers. \_activ\_func: Specifies the activation function for each neuron; one of `CvANN_MLP::IDENTITY`, `CvANN_MLP::SIGMOID_SYM` and `CvANN_MLP::GAUSSIAN`. \_f\_param1, \_f\_param2: Free parameters of the activation function, α and β, respectively. See the formulas in the introduction section.

The method creates MLP network with the specified topology and assigns the same activation
function to all the neurons.

---

### CvANN\_MLP::train

Trains/updates MLP

```
int CvANN_MLP::train( const CvMat* _inputs, const CvMat* _outputs,
                      const CvMat* _sample_weights, const CvMat* _sample_idx=0,
                      CvANN_MLP_TrainParams _params = CvANN_MLP_TrainParams(),
                      int flags=0 );
```

\_inputs: A floating-point matrix of input vectors, one vector per row. \_outputs: A floating-point matrix of the corresponding output vectors, one vector per row. \_sample\_weights: (RPROP only) The optional floating-point vector of weights for each sample. Some samples may be more important than others for training, e.g. user may want to gain the weight of certain classes to find the right balance between hit-rate and false-alarm rate etc. \_sample\_idx: The optional integer vector indicating the samples (i.e. rows of `_inputs` and `_outputs`) that are taken into account. \_params: The training params. See CvANN\_MLP\_TrainParams description. \_flags: The various parameters to control the training algorithm. May be a combination of the following: `UPDATE_WEIGHTS = 1` - algorithm updates the network weights, rather than computes them from scratch (in the latter case the weights are initialized using *Nguyen-Widrow* algorithm). `NO_INPUT_SCALE` - algorithm does not normalize the input vectors. If this flag is not set, the training algorithm normalizes each input feature independently, shifting its mean value to 0 and making the standard deviation `=1`. If the network is assumed to be updated frequently, the new training data could be much different from original one. In this case user should take care of proper normalization. `NO_OUTPUT_SCALE` - algorithm does not normalize the output vectors. If the flag is not set, the training algorithm normalizes each output features independently, by transforming it to the certain range depending on the activation function used.

This method applies the specified training algorithm to compute/adjust the network weights.
It returns the number of done iterations.
