# Supplementary material for: Rapid whole brain imaging of neural activity in freely behaving larval zebrafish (Danio rerio)
Source: eLife. 2017 Sep 20;6:e28158. doi: 10.7554/eLife.28158 (PMC5644961; doi:10.7554/eLife.28158)
Supplement: Source code 3. [file elife-28158-code3.zip › RT analysis code/OpenCV1.1pre.zip!/OpenCV/docs/vidsurv/Blob_Tracking_Modules.doc]

# Blob Tracking Modules

## Table of contents

Blob Tracking Modules	1

Table of contents	1

General Description	1

Data structures description	2

CvBlob	2

CvBlobList	2

Modules descriptions	2

CvFGDetector	2

CvBlobDetector	3

CvBlobTracker	4

CvBlobTrackerList	4

CvBlobTrackerOne	5

CvBlobTrackGen	5

CvBlobTrackPostProc	6

CvBlobTrackPostProcList	6

CvBlobTrackPostProcOne	7

## General Description

The blob tracking system includes 5 modules as depicted on diagram.

This pipeline is called “Blob Tracking Auto” module and implemented as individual module in BlobTrackingAuto.cpp and BlobTrackingAuto.h files.

“*FG/BG Detection*” module performs foreground/background segmentation for each pixel.

“*Blob Entering Detection*” module uses the result (FG/BG mask) of “FG/BG Detection” module to detect new blob object entered to a scene on each frame.

“*Blob Tracking*” module initialized by “Blob Entering Detection” results and tracks each new entered blob.

“*Trajectory Generation*” module performs a saving function. It collects all blobs positions and save each whole blob trajectory to hard disk when it finished (for example tracking is lost).

“*Trajectory PostProcessing*” module performs a blob trajectory smoothing function. This module is optional and can not be included in specific pipeline.

The blob tracking system includes 4 modules as depicted on diagram.

## Data structures description

### CvBlob

It is a simple structure describing a blob position, size and ID.

```
typedef struct CvBlob
{
    float   x,y; /* blob position   */
    float   w,h; /* blob sizes      */
    int     ID;  /* blbo ID         */
}CvBlob;
```

### CvBlobList

This is a class to collect set of blobs. This set can be trajectories or (for example) list of blob on current frame. So this list is the indexed array and each element of this array can be addressed by key.

```
class CvBlobSeq
{
public:
    CvBlobSeq(int BlobSize = sizeof(CvBlob));
    virtual ~CvBlobSeq();
    virtual CvBlob* GetBlob(int BlobIndex);
    virtual CvBlob* GetBlobByID(int BlobID);
    virtual void DelBlob(int BlobIndex);
    virtual void DelBlobByID(int BlobID);
    virtual void Clear();
    virtual void AddBlob(CvBlob* pB);
    virtual int GetBlobNum();
};
```

Now this class is implemented using CvSeq from OpenCV. Initially this class was implemented to simplify blob list creation and manipulation.

## Modules descriptions

### CvFGDetector

This is virtual class, describing interface of “FG/BG Detection” module.

The interface is defined in videomining\Surveillance\src\BG-FG-Estimation\PM\BG_FG_Estimation.h file. If someone wants to create own module, he must create a class inherited from CvFGDetector and implement all virtual methods declared in CvFGDetector.

*Input Data:*

Image of current frame.

*Output Data:*

FG/BG mask of current frame.

```
class CvFGDetector
{
public:
    virtual IplImage* GetMask() = 0;
    /* process current image */
    virtual void    Process(IplImage* pImg) = 0;
    /* release foreground detector */
    virtual void    Release() = 0;
};
```

This module classify pixels on current frame to FG or BG pixels. As result the FG mask is returned by GetMask method. All methods must be redefined in child class. Also some creation function must be written.

### CvBlobDetector

This is virtual class, describing interface of “Blob Entering Detection” module.

The interface is defined in EnteringBlobDetection.h file. If someone wants to create own module, he must create a class inherited from CvBlobDetector and implement all virtual methods declared in CvBlobDetector.

*Input Data:*

FG/BG mask of current frame;

List of existed Blobs.

*Output Data:*

List of newly detected blobs.

```
class CvBlobDetector
{
public:
    /* try to detect new blob entrance based on foreground mask */
    /* pFGMask - image of foreground mask */
    /* pNewBlobList - pointer to sequence to save new detected blobs  */
    /* pOldBlobList - pointer to blob list which already exist on image */
    virtual int DetectNewBlob(IplImage* pFGMask, CvBlobSeq* pNewBlobList, CvBlobSeq* pOldBlobList) = 0;
	/* return number of detected blobs */
    /* release blob detector */
    virtual void Release()=0;
};
```

This module detects new blob using FG mask. As result the newly detected blobs saved to sequence of blob pNewBlobList. Also this module may use list of existing blobs pOldBlobList to recognize actually new blob. All methods must be redefined in child class. Also some creation function must be written.

### CvBlobTracker

This is virtual class, describing interface of “Blob Tracking” module.

The interface is defined in BlobTracking.h file.

If someone wants to create own module, he must create are class inherited from CvBlobTracker and implement all virtual methods declared in CvBlobTracker.

*Input Data:*

BGR Image of current frame;

FG/BG mask of current frame;

*Output Data:*

Blobs (Id, pos, size) on current frame.

```
class CvBlobTracker
{
public:
    /* Add new blob to track it and assign to this blob personal ID */
    /* pBlob - pinter to structure with blob parameters (ID is ignored)*/
    /* pImg - current image */
    /* pImgFG - current foreground mask */
    /* return pointer to new added blob */
    virtual CvBlob* AddBlob(CvBlob* pBlob, IplImage* pImg, IplImage* pImgFG = NULL ) = 0;
    /* return number of currently tracked blobs */
    virtual int     GetBlobNum() = 0;
    /* return pointer to specified by index blob */
    virtual CvBlob* GetBlob(int BlobIndex) = 0;
    /* delete blob by its index */
    virtual void    DelBlob(int BlobIndex) = 0;
    /* process current image and track all existed blobs */
    virtual void    Process(IplImage* pImg, IplImage* pImgFG = NULL) = 0;
    /* release blob tracker */
    virtual void    Release() = 0;

    /* return pinter to blob by its unique ID */
    virtual int     GetBlobIndexByID(int BlobID);
    /* return pinter to blob by its unique ID */
    virtual CvBlob* GetBlobByID(int BlobID);
    /* delete blob by its ID */
    virtual void    DelBlobByID(int BlobID);
    /* Set new parameters for specified (by index) blob */
    virtual void    SetBlob(int BlobIndex, CvBlob* pBlob);
    /* Set new parameters for specified (by ID) blob */
    virtual void    SetBlobByID(int BlobID, CvBlob* pBlob);
};
```

This module must contain list of blobs. This list of blob updated after Process() is called. User can update blob parameters manually by SetBlob and SetBlobByID methods.

Methods AddBlob, GetBlobNum, DelBlob, Process, Release must be redefined in child class. Other methods may be redefined but it is not necessary. Also some creation function must be written.

#### CvBlobTrackerList

One implementation of “Blob Tracking” module is CvBlobTrackerList class. It is some “shell” module. The purpose of this module is simplifying creation of “Blob Tracking” module. The creation function cvCreateBlobTrackerList of this module has one parameter – pointer to a more simple blob tracker for one blob - CvBlobTrackerOne.

CvBlobTracker* cvCreateBlobTrackerList(CvBlobTrackerOne* (*create)());

So, the CvBlobTrackerList and CvBlobTrackerOne modules can be depicted as

#### CvBlobTrackerOne

This is virtual interface for “Blob Tracking One” module for one trajectory. This interface is used for example by CvBlobTrackerList module

```
class CvBlobTrackerOne
{
public:
    virtual void Init(CvBlob* pBlobInit, IplImage* pImg, IplImage* pImgFG = NULL) = 0;
    virtual CvBlob* Process(CvBlob* pBlobPrev, IplImage* pImg, IplImage* pImgFG = NULL) = 0;
    virtual void Release() =  0;
};
```

This class produces the tracking for one trajectory. One each frame the Process method is called to estimate new position and size of blob. As result the tracked position and size are returned by this method.

### CvBlobTrackGen

This is virtual class describing interface for “Trajectory Generator” module. The purpose of this module is to save whole trajectory to specified file. Also this module can calculate some features (using original image and FG mask) for each blob and saves it too.

The interface is defined in BlobTrackGen.h file.

If someone wants to create own module, he must create are class inherited from CvBlobTrackGen and implement all virtual methods declared in CvBlobTrackGen.

*Input Data:*

Blobs on current frame

*Output Data:*

Saved trajectory list

```
class CvBlobTrackGen
{
public:
    virtual void    SetFileName(char* pFileName) = 0;
    virtual void    AddBlob(CvBlob* pBlob) = 0;
    virtual void    Process(IplImage* pImg = NULL, IplImage* pFG = NULL) = 0;
    virtual void    Release() = 0;
};
```

All methods must be redefined in child class. Also some creation function must be written.

### CvBlobTrackPostProc

This is virtual class describing interface for “Trajectory Post Processing” module. The purpose of this module is to produce some filtering operation on blob trajectory. For example this module can be Klaman filter or another smoothing filter.

The interface is defined in BlobTrackPostProc.h file.

If someone wants to create own module, he must create are class inherited from CvBlobTrackPostProc and implement all virtual methods declared in CvBlobTrackPostProc.

*Input Data:*

Blobs on current frame

*Output Data:*

Blobs on current frame

```
class CvBlobTrackPostProc
{
public:
    virtual void    AddBlob(CvBlob* pBlob) = 0;
    virtual void    Process() = 0;
    virtual int     GetBlobNum() = 0;
    virtual CvBlob* GetBlob(int index) = 0;
    virtual void    Release() = 0;

    /* additional functionality */
    virtual CvBlob* GetBlobByID(int BlobID);
};
```

Methods Process, GetBlobNum, GetBlob and Release must be redefined in child class. Also some creation function must be written.

#### CvBlobTrackPostProcList

One implementation of “Trajectory Post Processing” module is CvBlobTrackPostProcList class. It is some “shell” module. The purpose of this module is simplifying creation of “Trajectory Post Processing” module. The creation function cvCreateBlobTrackPostProcList of this module has one parameter – pointer to a more simple one post processing module CvBlobTrackPostProcOne.

CvBlobTrackPostProc* cvCreateBlobTrackPostProcList(CvBlobTrackPostProcOne* (*create)());

So, the CvBlobTrackPostProcList and CvBlobTrackPostProcOne modules can be depicted as

#### CvBlobTrackPostProcOne

This is cvirtual interface for “Trajectory Post Processing” module for one trajectory. This interface is used for example CvBlobTrackPostProcList module

```
class CvBlobTrackPostProcOne
{
public:
    virtual CvBlob* Process(CvBlob* pBlob) = 0;
    virtual void    Release() = 0;
};
```

This class produces the post processing for one trajectory. One each frame the Process method is called to process new position and size of blob. As result the filtered position and size are returned by this method.
