# Supplementary material for: Rapid whole brain imaging of neural activity in freely behaving larval zebrafish (Danio rerio)
Source: eLife. 2017 Sep 20;6:e28158. doi: 10.7554/eLife.28158 (PMC5644961; doi:10.7554/eLife.28158)
Supplement: Source code 3. [file elife-28158-code3.zip › RT analysis code/OpenCV1.1pre.zip!/OpenCV/docs/vidsurv/Blob_Tracking_Tests.doc]

# Blob Tracking Tests

## General Description

The blob tracking system includes 5 modules as depicted on diagram.

“*FG/BG Detection*” module performs foreground/background segmentation for each pixel.

“*Blob Entering Detection*” module uses the result (FG/BG mask) of “FG/BG Detection” module to detect new blob object entered to a scene on each frame.

“*Blob Tracking*” module initialized by “Blob Entering Detection” results and tracks each new entered blob.

“*Trajectory Generation*” module performs a saving function. It collects all blobs positions and save each whole blob trajectory to hard disk when it finished (for example tracking is lost).

“*Trajectory PostProcessing*” module performs a blob trajectory smoothing function. This module is optional and can not be included in specific pipeline.

## Test system description

The test is based on synthetic video composed from different backgrounds and several video sprites. It allows calculating a ground through data. These data include a blob positions and sizes on each frame of whole video sequence and true FG/BG mask.

In the test system two real modules are exist. These modules output real data. These are real “FG/BG detection” module and real “Blob Entering Detection” module. So, using these modules a different pipeline configuration can be tested.

There are 3 main pipeline configurations:

1. The test of one “Blob Tracking” module;
2. Test of pair “Blob Entering Detection” and “Blob Tracking” modules;
3. Test of whole pipeline included “FG/BG Detection”, “Blob Entering Detection” and “Blob Tracking” modules.

### “Blob Tracking” Module Test

This is test of the one “Blob Tracking” module. This test is test of whole pipeline but real “FG/BG Detection” and real “Blob Enering Detection” module is used. So, the following pipeline is tested

The input data for this pipeline are image of current frame, indexes of frame on which new blob is entrance, position and size of new entered blob and real FG mask.

Example command line:

*BlobTracking fg=real bd=real btpp=none res=BlobTrackerRes.csv testseq.yml bg1 fg1*

All “Blob Tracking” modules will be tested on bg1 background and fg1 foreground videos. Results will be saved to BlobTrackerRes.csv file.

### “Blob Entering Detection” and “Blob Tracking” test.

This is test of the whole pipeline but with using real “FG/BG Detection” module. So, the following pipeline is tested.

The input data for this pipeline are image of current frame and real FG mask.

Example command line:

*BlobTracking fg=Real btpp=none res=BlobTrackerRes.csv testseq.yml bg1 fg1*

All “Blob Tracking” and “Blob Entering Detection” modules will be tested on bg1 background and fg1 foreground videos. Results will be saved to BlobTrackerRes.csv file.

### “FG/BG Detection”, “Blob Entering Detection” and “Blob Tracking” test

This is test of the whole pipeline:

The input data for this pipeline is image of current frame.

Example command line:

*BlobTracking btpp=none res=BlobTrackerRes.csv testseq.yml bg1 fg1*

All “FG/BG Detection”, “Blob Tracking” and “Blob Entering Detection” modules will be tested on bg1 background and fg1 foreground videos. Results will be saved to BlobTrackerRes.csv file.

### Test running

To run test the following command line is used:

```
BlobTracking.exe [fg=<fg_name>] [bd=<bd_name>]
                 [bt=<bt_name>] [btpp=<btpp_name>]
                 [bt_corr=<bt_corr_way>]
                 [trackgen=<btgen_name>]
                 [track=<track_file_name>]
                 [scale=<scale val>] [noise=<noise_name>] [IVar=<IVar_name>]
                 [res=<res_file_name>] [FGTrainFrames=<FGTrainFrames>]
                 <yml_file video1 video2 video3>|<avi_file>
  <bt_corr_way> is way of blob position corrrection for "Blob Tracking" module:
```

- None,
- PostProcRes

<noise_name> =

- No,
- Gauss_10,
- Gauss_20,
- Salt&Pepper_0.01,
- Salt&Pepper_0.05

<IVar_name> =

- No,
- I+=0.1,
- I+=1

```
  <scale val> is scale ratio for the source video
  <FGTrainFrames> is number of frames for FG training
  <track_file_name> is file name for save tracked trajectories
  <yml_file video1 video2 video3> are yml file name and videos used to create
    synthetic film to test blobtracking system
  <avi_file> is file name of avi to process by BlobTrackerAuto

Modules:
<fg_name> =
```

- Real
- FG_0,
- FG_1,

<bd_name> is "Blob Entrance Detection" module name and can be:

- Real - True blob entrance (ground truth data)
- BD_Simple - Detect new blob by uniform moving of connected components of FG mask

<bt_name> is "Blob Tracking" module name and can be:

- CC - Simple connected component tracking
- MS1 - Mean shift algorithm without scale adaptation
- MS2 - Mean shift algorithm with scale adaptation

<btpp_name> is "Blob Trajectory Post Processing" module name and can be:

- None - No post processing filter
- Kalman - Kalman filtering of blob position and size

<btgen_name> is "Blob Trajectory Generation" module name and can be:

- RawTracks - Generate raw track record (x,y,sx,sy),()... in each line

### Test method description

The results of each pipeline are blob trajectories represented as blob position, size and ID on each frame. The test system collects these data to “tracked trajectory” list. Also the test system collects ground truth data to “real trajectory” list. So, on each frame there are “tracked trajectory” and “real trajectory” lists.

On each frame the each record of “real trajectory” list is updated as follows:

- If “real trajectory” is not assigned to a “tracked trajectory” (blob ID of the real trajectory is not filled), then the test system tries to find the nearest blob, which is currently tracked and not assigned to any “real trajectory” yet. If such blob exists and the distance is less than the size of the blob, then this blob is assigned to the current “real trajectory”.
- If “real trajectory” is already assigned to some “tracked trajectory”, then this blob is checked whether it is still tracked, and “real trajectory” data is updated (summary error, tracked frame number and so on).

So when all the frames were processed, we have a “real trajectory” list. For each “real trajectory” we have the following information:

- a flag: was this track detected or not
- frame indices: when this track was detected and lost by system
- average error of tracked coordinates on each frame.

Based on this data the final characteristics are calculated and saved to CSV file.

Each row in output CSV file is results of one video sequence testing. Each row has several columns with parameters and result of test. These columns are following:

Test parameters:

- **VIDEO_CFG** – name of yml config file;
- **VIDEO_BG** – name (ID) of background video (which are declared in yml config file);
- **VIDEO_FG** – name (ID) of foreground video (which are declared in yml config file too);
- **SIZE** – image size of resulting test video sequence;
- **METHOD_FG** – type fo “FG/BG detector”
  - FG_0 - FG/GB estimation with ACM MM method
  - FG_1 - FG/BG estimation with Mixture of Gaussian ()
  - Real_FG – real FG mask (ground truth data)
- **METHOD_BD** – type of blob entrance detector
  - BD_Simple – simple blob detector based on FG mask connected component analysis.
- **METHOD_BT** - type of blob tracking method
  - MS1 - Mean Shift tracker (fixed scale)
  - MS2 - Mean Shift tracker (with scale search)
  - CC – simple tracker based on FG mask connected component analysis.
- **METHOD_BTPP** - type of Post processing module
  - None – Non post processing
  - Kalman – Kalman filtering of position and size.
- **Frames** – number of frames in the test sequence.

system performance characteristics:

- **TimeAll ms/frame** – Average time of one frame processing by BlobTrackerAuto module.
- **Time_FG ms/frame** - Average time of one frame processing by “FG/BG detector” module.
- **Time_BD ms/frame** - Average time of one frame processing by “BlobDetector” module.
- **Time_BT ms/frame** - Average time of one frame processing by “BlobTracker” module.
- **Time_BT ms/blob** – Average time of one blob tacking on one frame by “BlobTracker” module.
- **ObjectNum** – Number of real objects appeared in test sequence.
- **True Detected Object** – Number of real objects that were correctly detected.
- **False Detected Object** – Number of detected objects that never appeared in test video sequence.
- **Missed Object** - Number of real objects that were not detected.
- **Aver Late** - Average delay (in frames) between the moment when an object arrives in test video sequence and when it is detected by system. The averaging is done over all real trajectorys, which were detected. **Aver Late =** $\frac{\sum_{k}^{TDN} (DF_{k}-AF_{k})}{TDN}$. Where *TDN* is number of real object, which were detected (**True Detected Object**), *DF_k_* is frame on which real object was detected, *AF_k_* is frame on which real object was appeared in test sequence,
- **Max Late** - Maximal delay (in frames) between the moments when an object arrives in test video sequence and when it is detected. The maximum is calculated over all real trajectories, which were detected. Max Late = $\max_{k} (DF_{k}-AF_{k})$
- **Aver Pos Error** - average error of the tracked object coordinates. Averaging is done over all frames and all the objects. This error is calculated as $\frac{\sum_{i}^{N} \sum_{j}^{M_{i}} d_{i,j}}{\sum_{i}^{N} M_{i}}$. Where *N* is number of frames, *M_i_* is number of real object, which are detected on *i* frame, *d_i,j_* is distance on *i* frame (in pixels) between real object *j* and detected object.
- **Tracked trajectory in %** - Ratio (in %) of the real blob trajectory, which was tracked by the system. This value is calculated as $\frac{\sum_{k}^{TN} l_{k}}{\sum_{k}^{TN} L_{k}}$. Where *TN* is number of real objects and its trajectories, *L_k_* is number of frames on which object *k* was really appeared (real trajectory length), *l_k_* is number of frames on which object *k* was tracked and distance from tracked blob to real position is less than real size of object.

### Test files description

Folder: videomining\Surveillance\src\ObjectTracking\Blobs

*TestBlobTrackingAuto.cpp* – main file of test system.

*BlobTrackingAuto.h*, *BlobTrackingAuto.cpp*,– declaration and definition of “Blob Tracking Auto” module.

### Add new module to the test system

To add new module to test system one must implement it as class inherited form corresponded virtual class. After this *.cpp file (or files) with the implemented class code must be inserted to BlobTracking project. Also this file(s) must contain a creation function of new class. After this the description of new module must be added in structure array placed in begin of TestBlobTrackingAuto.cpp file. For example list of “Blob Tracking” modules in TestBlobTrackingAuto.cpp locks like:

```
/* list of BLOB TRACKING modules */
DefModule_BlobTracker BlobTracker_Modules[] =
{
    {CreateBTMS1,"MS1","Mean shift algorithm without scale adaptation"},
    {CreateBTMS2,"MS2","Mean shift algorithm with scale adaptation"},
    {cvCreateBlobTrackerCC,"CC","Simple connected component tracking"},
    {NULL,NULL,NULL}
};
/* list of Blob Tracker modules */
```

Here CreateBTMS1, CreateBTMS2 and cvCreateBlobTrackerCC are functions, which creates new module and returns pointer to virtual class CvBlobTracker. All class creation function declared as CvBlobTracker* cvCreateBlobTRackerModule();
