# Supplementary material for: Rapid whole brain imaging of neural activity in freely behaving larval zebrafish (Danio rerio)
Source: eLife. 2017 Sep 20;6:e28158. doi: 10.7554/eLife.28158 (PMC5644961; doi:10.7554/eLife.28158)
Supplement: Source code 3. [file elife-28158-code3.zip › RT analysis code/OpenCV1.1pre.zip!/OpenCV/docs/vidsurv/TestSeq.doc]

## Test sequence generator

## (videomining\Surveillance\src\TestSeq\testseq.h)

This module is used to generate different test video sequence with ground truth.

Interface of this module is following:

```
/* pConfigfile - name of file (yml or xml) with description of test sequence */
/* videos - array of names of test videos described in "pConfigfile" file */
/* numvideos - size of "videos" array */
CvTestSeq* cvCreateTestSeq(char* pConfigfile, char** videos, int numvideo, float Scale = 1);
void cvReleaseTestSeq(CvTestSeq** ppTestSeq);

/* generete next frame from test video seq and return pointer to it */
IplImage* cvTestSeqQueryFrame(CvTestSeq* pTestSeq);
/* return pointer to current foreground mask */
IplImage* cvTestSeqGetFGMask(CvTestSeq* pTestSeq);
/* return pointer to current image */
IplImage* cvTestSeqGetImage(CvTestSeq* pTestSeq);
/* return frame size of result test video */
CvSize cvTestSeqGetImageSize(CvTestSeq* pTestSeq);

/* return number of existed objects
 this is general number of any objects.
for example number of trajectories may be equal or less than returned value*/
int cvTestSeqGetObjectNum(CvTestSeq* pTestSeq);

/* return 0 if there is not position for current defined on current frame */
/* return 1 if there is object position and pPos was filled */
int cvTestSeqGetObjectPos(CvTestSeq* pTestSeq, int ObjIndex, CvPoint2D32f* pPos);
```

The description of test video is placed in yml file.

This file consists of some “structures”. Each structure has name and, after a colon, the content. Each structure consists of one or several “records”. Each record is a set of pairs: “field_name:field_value” …

For example:

One record structure

```
Structure_name:
  Field1: value1
  Field2: value2
```

Multiple record structure

```
Structure_name:
  -
    Field1: value1
    Field2: value2
  -
    Field1: value1
    Field2: value2
….
```

There are several fields and values that may be used:

- **File: file_name –** Define source AVI or BMP file.
- **Video: structure_name –** Instead of a file, use earlier defined structure **structure_name**. Specal cases of **structure_name**.
  - **noise_gaussian** or **noise_normal** – record is based on video that is Gaussian noise with deviation = 1 and mean = 0. (deviation can be modified by NoiseAmp in Trans).
  - **noise_uniform** – record is based on video that is normal noise with deviation = 1 and mean = 0. (deviation can be modified by NoiseAmp in Trans)
  - **noise_speckle** - – record is based on video that is speckle noise with deviation = 1 and mean = 0. (deviation can be modified by NoiseAmp in Trans)
  - **Noise_salt_and_pepper** – record is nased on video that is salt&pepper noise, the density of noise is 1. (density can be modified by NoiseAmp in Trans)
- **FrameBegin: start_frame** - Define a frame, starting from which the record is embedded into the test sequence.
- **FrameNum:** **duration** - Define the number of frames during which the record is embedded into the test sequence.
- **Pos:** **val** - Define position of the object.
  - **Val = [x,y,x,y,…] –** sequence of coordinates on each frame
  - **Val = auto** – automatic calculate object position by foreground mask.
- **Shift: val –** Define shift of image and object
  - **Val = [x,y,x,y,…] –** shifts on each frame
  - **Val = auto** – shift calculated automatically on each frame by foreground mask. So after such shift gravity center of foreground mask placed to (0,0)
- **BG:**
  - **0 –** record is foreground (default value)
  - **1–** record is background
- **Trans:** list_of_transformation: offset, scale, intensity, contrast, noise …. Trans can be as sequence of key transformation as one transformation for whole video. Each transformation record can include.
  - **s:** sclale coefficient
  - **sx:** scale by x
  - **sy:** scale by y
  - **dx:** shift by x
  - **dy:** shift by y
  - **angle:** rotate by angle in degree.
  - **I:** change intensity Image = Image + I
  - **C:** change contrast Image=C*Image
  - **NoiseAmp:** change noise amplitude.

To describe format of this file the several example are written below.

Example1:

```
bg1:
  BG: 1
  File: "video.avi"
```

Such yml file describes one background video from AVI file “video.avi”. No object is present.

Example2:

bg1: {BG: 1, File: "image.bmp", FrameNum: 120}

Such yml file describes one background video created from BMP file “image.bmp”. Length of such video is 120 frames. No object is present.

Example3:

```
bg1: {BG: 1, File: "image.bmp", FrameNum: 120}
video:
  -
    Video: bg1
  -
    File: “sprite.bmp”
    FrameNum: 120
    Trans: [{dx:0,dx:1}]
```

Such yml file describes background video “bg1” created from BMP file “image.bmp” and synthetic video “video” that is moving image “sprite.bmp” from left to right during 120 frames on image “image.bmp”. No any object is present. The file “sprite.bmp” must be image of some object on black background.

Example4:

```
bg1: {BG: 1, File: "image.bmp", FrameNum: 120}
video:
  -
    Video: bg1
  -
    File: “sprite.bmp”
    Pos: [0.5,0.5]
    FrameNum: 120
    Trans: [{dx:0},{dx:1}]
```

Such yml file describes background video “bg1” created from BMP file “image.bmp” and synthetic video “video” that is moving image “sprite.bmp” from left to right during 120 frames on image “image.bmp”. One object is present. Its coordinate lineary changes from (0.5,0.5) to (1.5,0.5) during 120 frames.

Example5:

```
bg1: {BG: 1, File: "video.avi", FrameNum: 120}
video:
  -
    Video: bg1
  -
    File: “sprite.bmp”
    Pos: auto
    FrameNum: 120
    Trans: [{dx:0},{dx:1}]
```

Such yml file describes background video “bg1” created from first 120 frames captured from AVI file “video.avi” and synthetic video “video” that is moving image “sprite.bmp” from left to right during 120 frames on “bg1” video. One object is present. Its coordinate lineary changes from (x,y) to (x+1,y) during 120 frames. Where (x,y) is gravity center calculated for mask created from “sprite.bmp” image

Example5:

```
bg1: {BG: 1, File: "video.avi", FrameNum: 120}
video:
  -
    Video: bg1
  -
    File: “sprite.bmp”
    Pos: auto
    Shift: auto
    FrameNum: 120
    Trans: [{dx:0,dy:0.5},{dx:1,dy:0.5}]
```

Such yml file describes background video “bg1” and synthetic video “video” that is moving image “sprite.bmp” from left to right during 120 frames on “bg1” video. One object is present. Its coordinate linearly changes from (0,0.5) to (1,0.5) during 120 frames.
