# Supplementary material for: Rapid whole brain imaging of neural activity in freely behaving larval zebrafish (Danio rerio)
Source: eLife. 2017 Sep 20;6:e28158. doi: 10.7554/eLife.28158 (PMC5644961; doi:10.7554/eLife.28158)
Supplement: Source code 3. [file elife-28158-code3.zip › RT analysis code/RT analysis instruction.doc]

Instruction on using the RT analysis code

1. Install git for windows here: <https://git-scm.com/downloads>

2. Install MinGW (Minimalist GNU for Windows) development environment
1) Go to <http://www.mingw.org/wiki/Getting_Started>. Follow the instruction and download mingw-get-setup.exe
2) Install **mingw32-base**, **mingw32-gcc-g++**, and msys-base.
3) Go to Control Panel\System and Security\System, click advanced system settings and click environmental variables. Add **C:\mingw\bin** to the system path.
4) Go to **C:\MinGW\msys\1.0**, and double click msys. This is the compiling environment for C and C++. A detailed account of how to compile and build a C/C++ project can be found <https://www3.ntu.edu.sg/home/ehchua/programming/cpp/gcc_make.html>

3. OpenCV_1.1 pre1a may be difficult to find in the internet (because the version is too old), so we provide a compressed file that we’ve done (named OpenCV1.1a.zip). You should decompress it into **C:\Program Files (x86)\OpenCV**, and make sure **that C:\Program Files (x86)\OpenCV\bin** is on the system path.

4. Run the low mag RT code
1) Use MinGW to compile the code. In the MinGW compiling environment, go to the folder where the makefile is located. Run **make all** to compile the whole project.
2) Run cmd.exe , then run the command “CD Folder” to go to the low resolution code folder. Access the bin dictionary, using command like this:

**Low_mag_acquisition.exe –i [absolute video path]**

For instance,

**Low_mag_acquisition.exe –i C: \low_sample.avi**

5. The High mag RT code is similar to the low mag RT code. Use command

**Fish_Eye_Tracking.exe –i C:\high_sample.avi**

Note that the code has only been tested in Windows 7 (64bit). It may not work in other systems. When running the code on your personal computer, you might get different fps as what is reported on the paper. You can switch the Display mode from raw image mode to analyzed mode on the trackbar (0, 1 or 2). The full software is developed based on the same framework as the Colbert system (https://github.com/samuellab/mindcontrol). But a large portion of the code has been rewritten so that it works for larval zebrafish.
